# Age‐Dependent KLK8 Upregulation Contributes to Elevated Susceptibility to Ventilator‐Induced Lung Injury in the Elderly Mice

**DOI:** 10.1111/acel.70304

**Published:** 2025-11-27

**Authors:** Di Liu, Tian‐Tian Lin, Hui Zhang, Ying Zhao, Chu‐Fan Xu, Yu‐Jian Liu, Lai Jiang, Xiao‐Yan Zhu

**Affiliations:** ^1^ Department of Anesthesiology and Surgical Intensive Care Unit, Xinhua Hospital Shanghai Jiaotong University School of Medicine Shanghai China; ^2^ Department of Physiology Naval Medical University Shanghai China; ^3^ School of Kinesiology, the Key Laboratory of Exercise and Health Sciences of Ministry of Education Shanghai University of Sport Shanghai China

**Keywords:** cell senescence, poly(ADP‐ribose) polymerase, pulmonary vascular endothelial cell, tissue kallikrein‐related peptidase 8, ventilator‐induced lung injury

## Abstract

There is a growing contradiction between the rising demand for mechanical ventilation among the elderly and their heightened sensitivity to ventilator‐induced lung injury (VILI). This discrepancy compels us to explore therapeutic targets for VILI in elderly patients. Our research revealed that aging increases the sensitivity of pulmonary endothelial cells to low‐magnitude mechanical stretch. By analyzing transcriptome sequencing data from lung tissues of humans and mice at different ages, as well as published transcriptome sequencing data from senescent endothelial cells, we identified tissue kallikrein‐related peptidase 8 (KLK8) as an age‐dependent upregulated gene in lung tissues. Using KLK8 knockout mice, intra‐pulmonary KLK8‐overexpressing mice, and mouse lung vascular endothelial cells (MLVECs) with KLK8 overexpression or knockdown, we demonstrated that age‐dependent KLK8 upregulation contributes to pulmonary endothelial senescence and increased susceptibility of aged mice to VILI. Mechanistically, KLK8 promotes pulmonary endothelial senescence by inactivating the fibronectin/focal adhesion kinase (FAK) pathway. Through transcriptional profiling, we identified the poly(ADP‐ribose) polymerase 1/2 (PARP1/2) inhibitor olaparib as a potential agent that rescues KLK8‐induced pulmonary endothelial cell senescence and alleviates VILI in aged mice. Our findings underscore the critical role of KLK8 in pulmonary endothelial senescence and provide preclinical evidence for PARP1/2 inhibitors as a therapeutic target for VILI in elderly individuals.

## Graphical abstract

Our research firstly indicated that age‐dependent upregulation of tissue kallikrein‐related peptidase 8 (KLK8) induces endothelial senescence through modulating the fibronectin‐FAK‐Akt‐PARP1/2 axis, thereby increasing the vulnerability of aged mice to low‐tidal‐volume ventilator‐induced lung injury (VILI). Notably, we identified the PARP1/2 inhibitor olaparib as a potential therapeutic agent for alleviating VILI in the elderly.

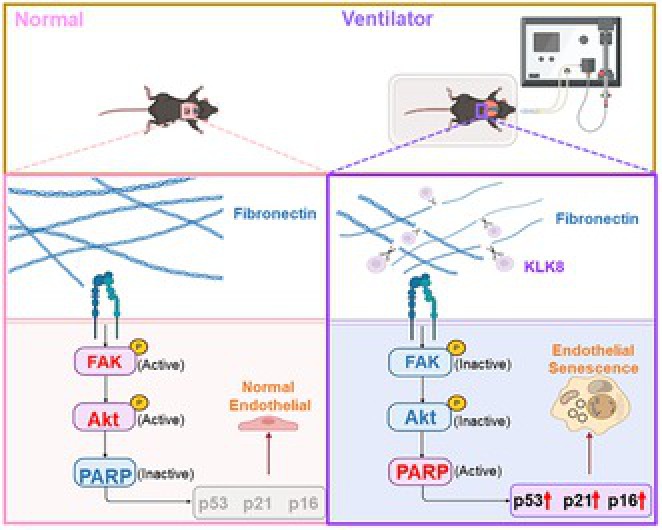

## 1 Introduction

Mechanical ventilation is a critical intervention for respiratory failure, providing essential respiratory support and prolonging life. However, lung overdistension caused by mechanical ventilation can lead to ventilator‐induced lung injury (VILI), characterized by alveolar damage, increased pulmonary vascular permeability, and elevated alveolar exudate (Gattinoni et al. 2024; Marini and Gattinoni 2024; Songsangvorn et al. 2024). Consequently, in recent years, the clinical strategy of “low‐tidal‐volume (LTV) protective lung ventilation (VT: 6–8 mL/kg)” has been widely adopted, significantly reducing VILI by lowering tidal volume (Asehnoune et al. 2023; Al‐Khalisy et al. 2024). With the aging population and the global pandemic of coronavirus disease (COVID‐19), the proportion of elderly patients requiring mechanical ventilation support has rapidly increased (Herbert et al. 2016; Bouza et al. 2021; Valentine et al. 2023; Huang 2024). Elderly patients exhibit higher mortality rates after mechanical ventilation and are more susceptible to VILI (Herbert et al. 2016; Bouza et al. 2021; Lim et al. 2021; Smolin et al. 2022; Huang 2024; Liu et al. 2024). The high demand for mechanical ventilation and the increased vulnerability of elderly patients to VILI present a significant challenge, necessitating the exploration of effective treatment strategies for VILI in this population.

The injurious effect of mechanical stretch on the pulmonary endothelium has been implicated in the development of VILI, characterized by pulmonary inflammation and significantly elevated endothelial permeability (Kikuchi et al. 2024). Previous studies have confirmed that mechanical ventilation causes endothelial cell injury and hyperpermeability both in vitro and in vivo (Xu et al. 2019; Wang et al. 2021). Emerging literature demonstrates that senescent endothelial cells exhibit impaired proliferative capacity and increased expression of senescence‐associated secretory phenotype (SASP) factors, leading to tissue regeneration disorders, widespread vascular thrombosis, and pathogenic microenvironment formation in various lung injuries, such as those seen in COVID‐19 and lung fibrosis (Blandinières et al. 2019; D'Agnillo et al. 2021; Caporarello et al. 2022; Huang et al. 2023; Cheng et al. 2025). However, the role of endothelial senescence in VILI and its underlying mechanisms remains understudied, which may provide valuable insights into therapeutic strategies for VILI in elderly patients.

In this study, using cellular and animal models, we confirmed for the first time that aging exacerbates mechanical stretch‐induced pulmonary endothelial injury. To identify age‐related genes in lung tissues, we collected lung samples from 18 patients spanning a wide age range for RNA sequencing (RNA‐seq). By integrating our RNA‐seq dataset with published RNA‐seq and single‐nucleus RNA‐seq datasets of aging lungs and senescent endothelial cells, along with validation experiments, we identified tissue kallikrein‐related peptidase 8 (KLK8), a secreted serine protease, as an age‐dependent upregulated gene in lung tissues. Intrapulmonary KLK8 overexpression alone induced pulmonary endothelial cell senescence and increased susceptibility to VILI, while KLK8 deficiency mitigated the elevated susceptibility to VILI in elderly mice. Mechanistically, we found that age‐dependent KLK8 upregulation promoted pulmonary endothelial senescence via the fibronectin/focal adhesion kinase (FAK) signaling pathway. To identify compounds capable of reversing genetic alterations in aging lung tissues and senescent endothelial cells, we used the age‐related genes in lung tissues and the differentially expressed genes (DEGs) of senescent endothelial cells to query the Connectivity Map (CMap) platform. We identified poly(ADP‐ribose) polymerase 1/2 (PARP1/2) inhibitor as a potential candidate that mitigated pulmonary endothelial cell senescence and improved susceptibility to VILI in both intrapulmonary KLK8‐overexpressing mice and aged mice.

## 2 Results

### 2.1 Aging Enhances the Sensitivity of Pulmonary Endothelial Cells to Low‐Magnitude Mechanical Stretch

To examine whether senescent endothelial cells exhibit increased sensitivity to mechanical stretch, we established an in vitro model of senescent mouse lung vascular endothelial cells (MLVECs) using H_2_O_2_ treatment. H_2_O_2_‐induced senescence was characterized by increased expression of p53 and p21 proteins (Figure S1A), as well as a higher number of senescence‐associated β‐galactosidase (SA‐β‐gal) positive MLVECs (Figure S1B). It is well established that the endothelial cell‐specific adherens junction molecule vascular endothelial cadherin (VE‐cadherin) plays a crucial role in maintaining endothelial barrier integrity (Serafin et al. 2024). Additionally, vascular cell adhesion molecule 1 (VCAM‐1), which is abundantly expressed on activated endothelial cells, serves as a marker of endothelial dysfunction (Pickett et al. 2023). As shown in Figure S2A, H_2_O_2_‐induced senescent endothelial cells exhibited significant damage, evidenced by decreased VE‐cadherin expression and increased VCAM‐1 expression. Endothelial barrier function was assessed by measuring macromolecular permeability using FITC‐labeled avidin in MLVEC monolayers. H_2_O_2_‐induced senescent MLVECs showed marked accumulation of FITC‐labeled avidin, indicating increased endothelial monolayer permeability (Figure S2B,C). Subsequently, H_2_O_2_‐induced senescent MLVECs were subjected to physiological, low‐magnitude (7%) cyclic stretch (Felder et al. 2019) for 4 h. Although low‐magnitude cyclic stretch alone did not induce endothelial cell damage, it significantly exacerbated H_2_O_2_‐induced endothelial cell damage and hyperpermeability, as indicated by further decreased VE‐cadherin expression, increased VCAM‐1 expression (Figure S2A), and enhanced permeability of the MLVEC monolayer to FITC‐labeled avidin (Figure S2B,C). Furthermore, ZO‐1/DAPI immunofluorescence staining was performed to evaluate the effects of H_2_O_2_ and physiological cyclic stretch on endothelial cell junctional integrity. Our results showed that H_2_O_2_ disrupted the membrane continuity of ZO‐1 between neighboring MLVECs, and this disruption was further exacerbated by physiological cyclic stretch (Figure S3A). These results suggest that endothelial cell senescence increases the sensitivity of pulmonary endothelial cells to low‐magnitude mechanical stretch.

To examine the impact of natural aging on the sensitivity of pulmonary endothelial cells to LTV mechanical ventilation, aged mice (18 months) and young mice (2 months) were subjected to 8 mL/kg mechanical ventilation for 4 h. Compared to young mice, neither LTV ventilation nor natural aging alone had a significant effect on pulmonary protein levels of VE‐cadherin and VCAM‐1 or the morphological structure of lung tissues (Figure S2D–F). These findings indicate that neither LTV ventilation (Wienhold et al. 2018; Koh et al. 2021) nor natural aging (Cui et al. 2022) alone is sufficient to cause lung injuries, which is consistent with previous studies. Notably, LTV ventilation resulted in profound decreases in VE‐cadherin and increases in VCAM‐1 in the lung tissues of aged mice (Figure S2D). Histopathological analysis of hematoxylin and eosin (H&E)‐stained lung tissue sections revealed that LTV‐ventilated aged mice exhibited significant lung injuries, evidenced by diffuse interstitial edema, alveolar thickening, narrowing of alveolar air spaces, and recruitment of leukocytes, with an increased lung injury score compared to LTV‐ventilated young mice (Figure S2E,F).

Combining the results from the H_2_O_2_‐induced senescent MLVECs and the LTV‐ventilated aged mice, these findings suggest that aging enhances the sensitivity of pulmonary endothelial cells to mechanical stretch.

### 2.2 Age‐Dependent KLK8 Upregulation Contributes to Pulmonary Endothelial Senescence

**FIGURE 1:**
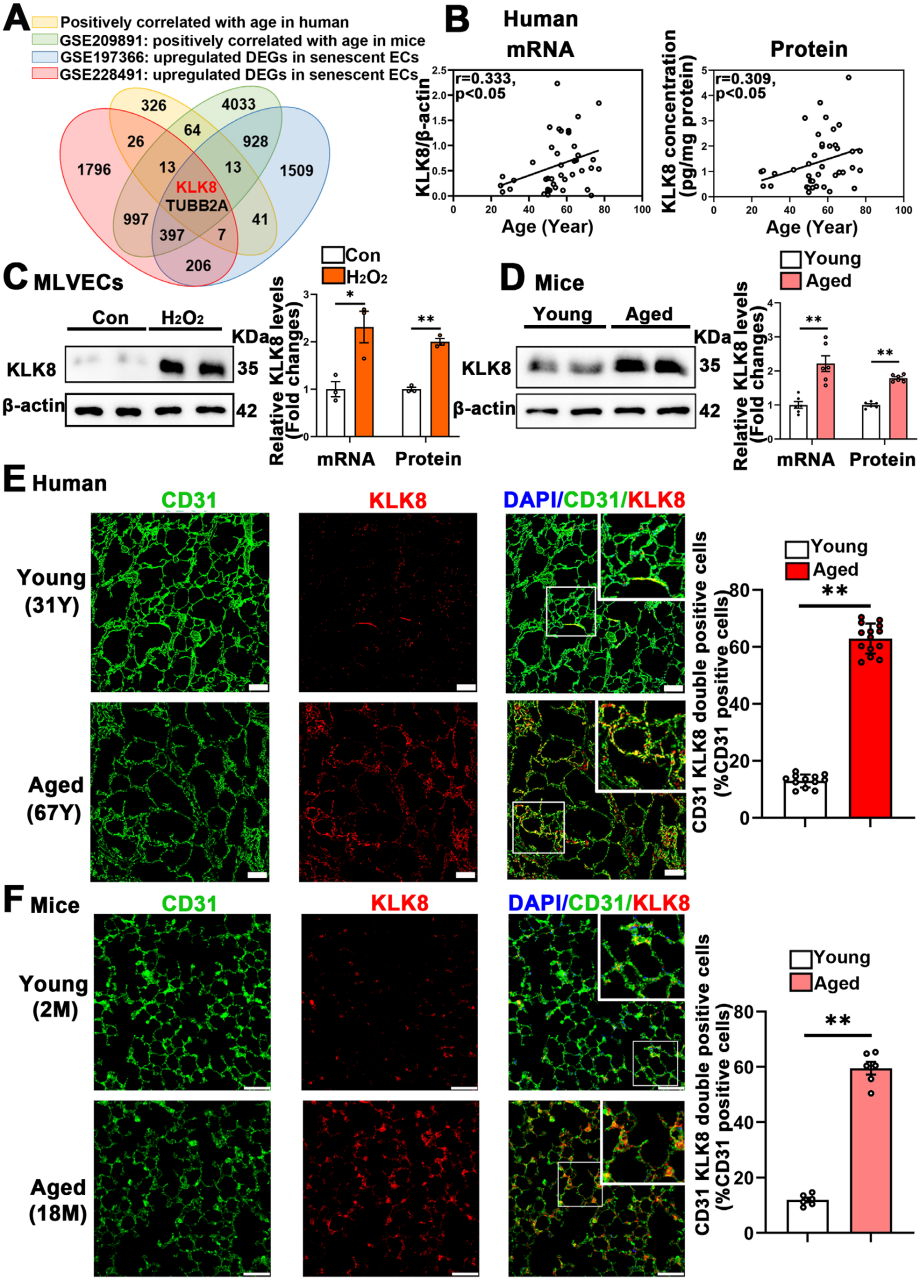
KLK8 exhibits age‐dependent upregulation. (A) Venn Diagram of genes positively correlated with age in human and mice, and upregulated differentially expressed genes (DEGs) in senescent endothelial cells (ECs). Transcriptomic dataset of lung samples from 18 patients within a wide age range and a published transcriptomic dataset of lung tissues from mice at different ages (GSE209891) were analyzed to obtain genes positively correlated with age. Single‐nucleus RNA‐seq of lung tissues from progeria mice (GSE228491) and a transcriptome dataset of ECs from aged mice (GSE197366) were analyzed to obtain upregulated DEGs in senescent ECs. (B) Correlation of the lung KLK8 mRNA (left) and protein (right) expression with age of 41 patients by qRT‐PCR (Pearson correlation test, *p* = 0.0334) and ELISA (Pearson correlation test, *p* = 0.0496), respectively. (C) The levels of KLK8 mRNA and protein were measured in the primary cultured MLVECs treated with H_2_O_2_ (250 μM) for 72 h by qRT‐PCR and western blotting, respectively. Representative protein bands were presented on the left of the histograms (*n* = 3). (D) The levels of KLK8 mRNA and protein were measured in the young (2 months) and aged (18 months) mice by qRT‐PCR and western blotting, respectively. Representative protein bands were presented on the left of the histograms (*n* = 6). (E) Immunofluorescent staining showed KLK8 (red) expression in the lung sections of young (19–40 years old) and aged patients (67–78 years old). Endothelial cells were immunostained with anti‐CD31 (green). Nuclei were counterstained with DAPI (blue). Scale bar = 200 μm. The percentage of CD31^+^KLK8^+^ cell numbers in total CD31^+^ cells were presented in the right panels (*n* = 12 for young human and *n* = 14 for aged human). (F) Immunofluorescent staining showed KLK8 (red) expression in the lung sections of young and aged mice lungs. Endothelial cells were immunostained with anti‐CD31 (green). Nuclei were counterstained with DAPI (blue). Scale bar = 50 μm. The percentage of CD31^+^KLK8^+^ cell numbers in total CD31^+^ cells were presented in the right panels (*n* = 6). Data were presented as means ± SEM. *p < 0.05, **p < 0.01.

To identify age‐related genes in lung tissues, we first collected lung tissues from 18 patients within a wide age range (25–77 years old) who met our enrollment criteria for transcriptome sequencing (Tables S1 and S2). This analysis yielded 492 genes positively and 719 genes negatively correlated with age (|correlation| ≥ 0.5, *p* < 0.05) (Figure 1A; Figure S4A,B). Additionally, we analyzed a published transcriptomic dataset of lung tissues from mice at different ages (3, 6, 12, 24 months, GSE209891), identifying 6447 genes positively and 7048 genes negatively correlated with age (|correlation| ≥ 0.5, *p* < 0.05) (Figure 1A; Figure S4C,D). By intersecting the age‐correlated genes in human and mouse lungs, we obtained 92 positively and 150 negatively correlated genes (Figure 1A; Figure S4E). To investigate senescence‐associated genes in endothelial cells, we analyzed two published datasets: single‐nucleus RNA‐seq of lung tissues from progeria mice (GSE228491) and a transcriptome dataset of endothelial cells from aged mice (80 weeks old, GSE197366). We identified DEGs between control and senescent endothelial cells (|FC| ≥ 1.5, *p* < 0.05) (Tables S3 and S4). Intersecting upregulated and downregulated DEGs from these datasets resulted in 612 upregulated and 1168 downregulated DEGs in senescent endothelial cells (Figure 1A; Figure S4E). Those age‐correlated genes were then intersected with the DEGs in senescent endothelial cells, yielding a total of 21 intersected genes (Figure 1A; Figure S4E).

Notably, among these genes, KLK8 was recently reported to activate the p53 signaling pathway in cardiovascular cells, thus attracting our attention (Du et al. 2021; Sun et al. 2022). To validate the bioinformatics analysis results, we recruited an additional 23 patients (total of 41 patients, aged 25–77 years) for PCR and ELISA analyses. Both KLK8 mRNA and protein levels in human lung tissues were positively correlated with age (*p* < 0.05, correlation coefficients *r* = 0.333 and *r* = 0.309 for mRNA and protein, respectively, Figure 1B). Higher mRNA and protein levels of KLK8 were also observed in H_2_O_2_‐induced senescent MLVECs (Figure 1C) and lung tissues of aged mice (18 months) compared to control cells and young mice (2 months), respectively (Figure 1D). The expression of KLK8 in pulmonary endothelial cells was subsequently evaluated through double immunofluorescence staining, utilizing antibodies specific for KLK8 and the vascular endothelial cell marker CD31 and VE‐cadherin. As shown in Figure 1E,F, Figure S5A,B, the percentages of KLK8^+^/CD31^+^ cells in total CD31^+^ cells and the percentages of KLK8^+^/VE‐cadherin^+^ cells in total VE‐cadherin^+^ cells in lung tissue sections were significantly elevated in both aged patients (≥ 67 years old, *n* = 14) and mice (18 months, *n* = 6) compared to young patients (≤ 40 years old, *n* = 12) and mice (2 months, *n* = 6).

**FIGURE 2:**
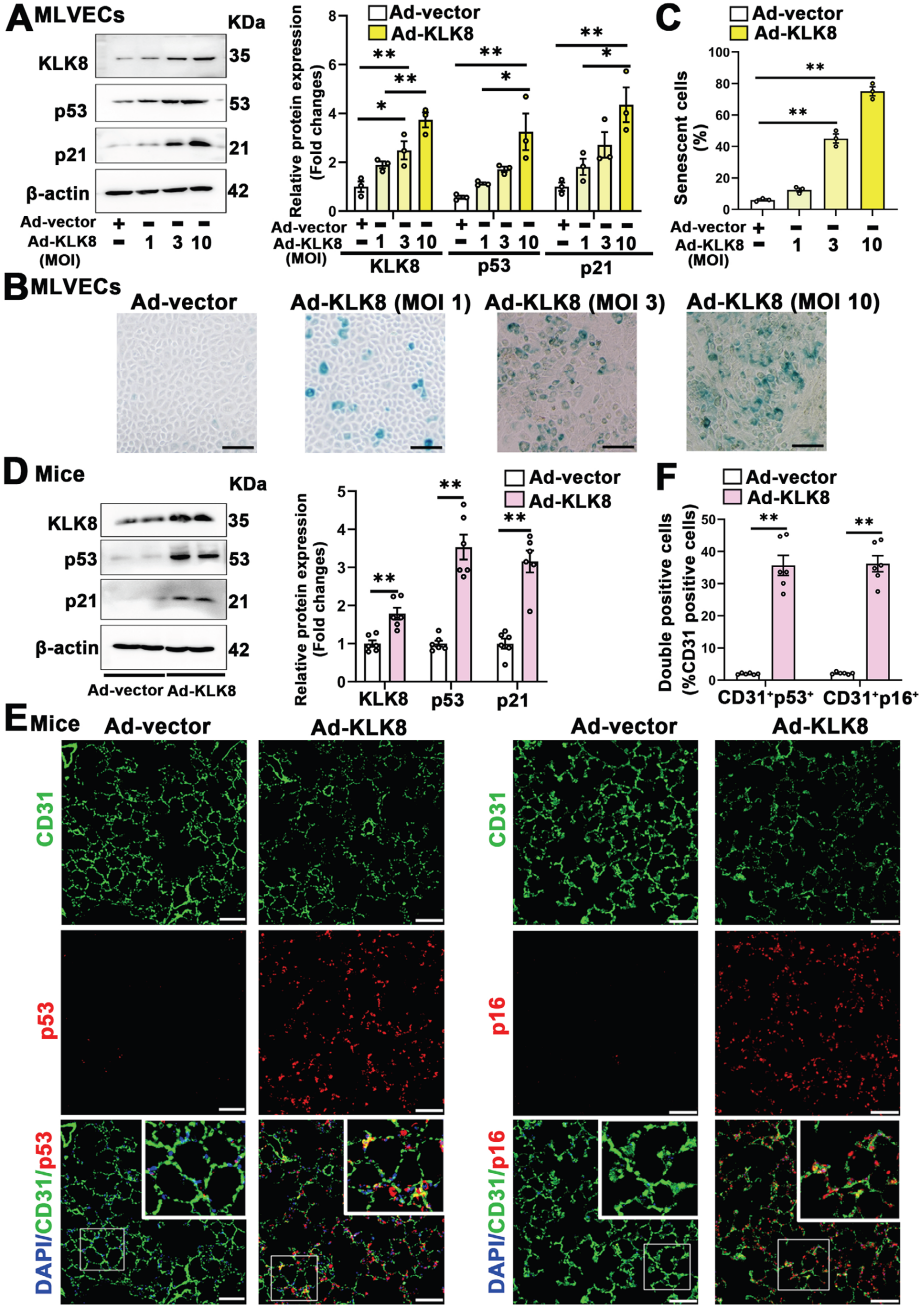
KLK8 overexpression alone is sufficient to induce endothelial cell senescence. (A–C) Primary cultured MLVECs were transfected with KLK8 adenovirus (Ad‐KLK8) at MOI 1–10 or control adenovirus (Ad‐vector) at MOI 10 for 72 h (*n* = 3). (A) Western blot showing KLK8, p53 and p21 protein levels in MLVECs. Corresponding histograms were shown on the right of representative protein bands. (B) Representative SA‐β‐gal staining of MLVECs. Scale bar, 100 μm. (C) Quantification of SA‐β‐gal positive MLVECs. (D–F) Young mice (2 months) were instilled intratracheally with Ad‐KLK8 or Ad‐vector. Seventy hours later, lung tissues were harvested for Western Blot and immunofluorescence analysis (*n* = 6). (D) Western blot showing KLK8, p53 and p21 protein levels in lung tissues. Corresponding histograms were shown on the right of representative protein bands. (E) Representative senescent markers staining showing p53 and p16 (red) expression in lung sections. Endothelial cells were immunostained with anti‐CD31 (green). Nuclei were counterstained with DAPI (blue). Scale bar, 50 μm. (F) The percentage of CD31^+^p53^+^ and CD31^+^p16^+^ cell numbers in total CD31^+^ cells. Data were presented as means ± SEM. *p < 0.05, **p < 0.01.

We next investigated whether age‐related KLK8 upregulation was involved in pulmonary endothelial senescence. KLK8 was overexpressed in primary cultured MLVECs using KLK8 adenovirus (Ad‐KLK8). Increasing concentrations of Ad‐KLK8 dose‐dependently increased KLK8 expression (Figure 2A). Ad‐KLK8 also induced endothelial senescence in a dose‐dependent manner, as evidenced by increased protein levels of the senescence markers p53 and p21 (Figure 2A), as well as elevated SA‐β‐Gal activity (Figure 2B,C). It is well established that senescent cells secrete a variety of factors collectively referred to as the SASP, which includes cytokines, chemokines, matrix remodeling proteases, and growth factors. Our results showed that Ad‐KLK8 treatment significantly increased the transcript levels of profibrotic and proinflammatory SASP factors, such as transforming growth factor‐β (TGF‐β), interleukin‐1α (IL‐1α), monocyte chemoattractant protein 1 (MCP1), tumor necrosis factor‐α (TNF‐α), interleukin‐6 (IL‐6), and C‐X‐C motif ligand 1 (CXCL1) in MLVECs (Figure S6A).

We subsequently investigated the in vivo effects of KLK8 overexpression by administering intratracheal transfection of Ad‐KLK8 in young mice. As expected, intratracheal transfection of Ad‐KLK8 significantly increased pulmonary KLK8 expression (Figure 2D). Double immunofluorescence staining showed that intra‐pulmonary Ad‐KLK8 transfected mice exhibited significantly higher percentages of p53^+^/CD31^+^, p21^+^/CD31^+^, and p16^+^/CD31^+^ vascular endothelial cells in total CD31^+^ endothelial cells and higher percentages of p53^+^/ VE‐cadherin^+^, p21^+^/ VE‐cadherin ^+^, and p16^+^/ VE‐cadherin^+^ vascular endothelial cells in total VE‐cadherin^+^ endothelial cells compared to control adenovirus (Ad‐vector) transfected mice (Figure 2E,F; Figure S7A–D). Collectively, these in vitro and in vivo findings suggest that KLK8 overexpression alone is sufficient to induce endothelial cell senescence.

Since H_2_O_2_ treatment significantly induced KLK8 expression in pulmonary vascular endothelial cells (Figure 1C), we examined whether upregulated KLK8 contributed to H_2_O_2_‐induced endothelial senescence. As shown in Figure S8A, KLK8 small interfering RNA (siRNA) reduced KLK8 expression by approximately 80% in MLVECs. KLK8 siRNA significantly attenuated H_2_O_2_‐induced endothelial senescence, as evidenced by decreased p53 and p21 protein levels (Figure S8A) and reduced SA‐β‐Gal activity (Figure S8B,C). In addition, we found that KLK8 siRNA significantly attenuated the H_2_O_2_‐induced profibrotic and proinflammatory secretome in senescent endothelial cells, as indicated by reduced transcript levels of profibrotic and proinflammatory SASP factors (Figure S8D). These results indicate that H_2_O_2_ may induce endothelial senescence through a KLK8‐dependent pathway in MLVECs.

**FIGURE 3:**
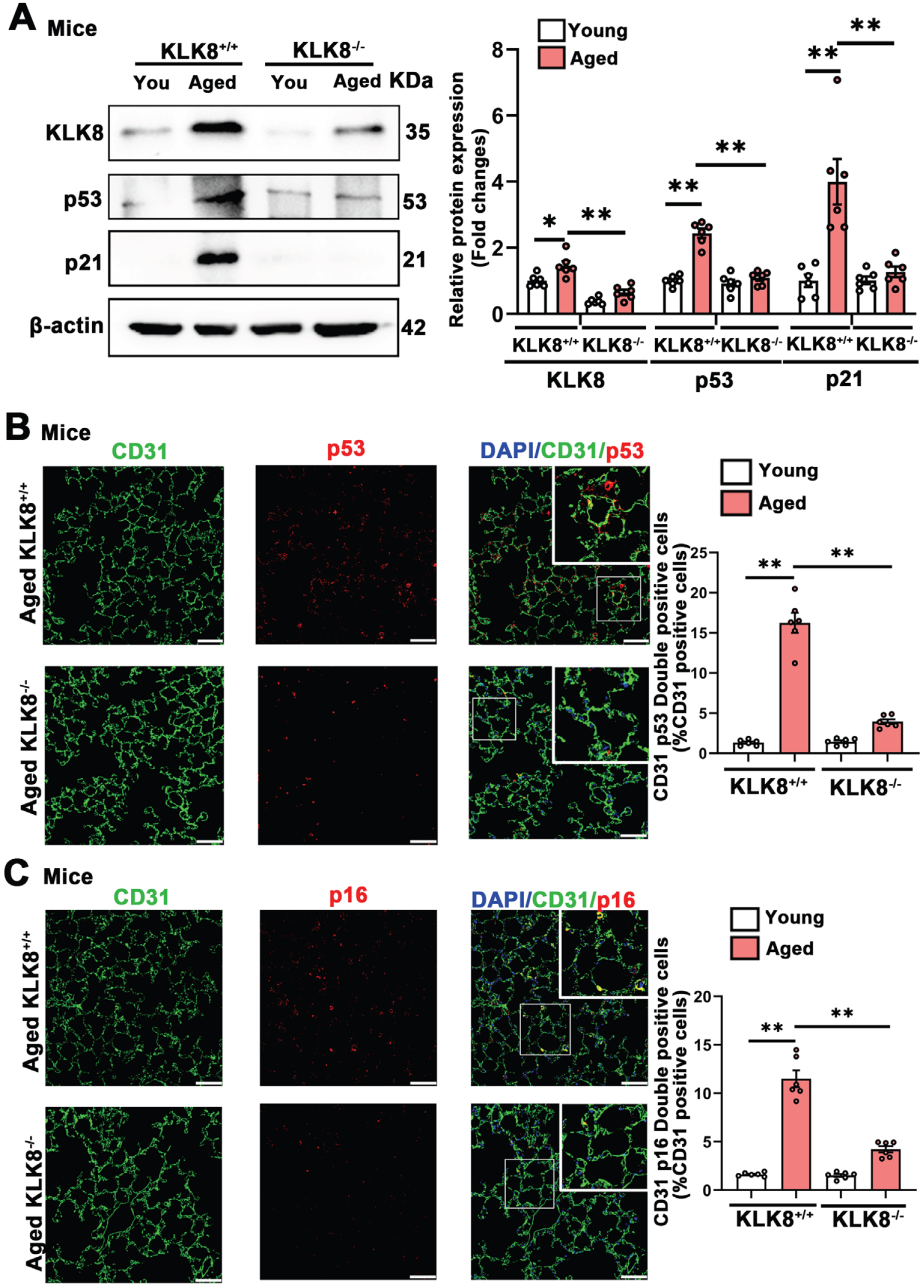
KLK8 deficiency relieves pulmonary endothelial cells senescence. (A) Western blot showing KLK8, p53 and p21 protein levels in young (2 months) and aged (18 months) KLK8^+/+^ and KLK8^−/−^ mice. Corresponding histograms were shown on the right of representative protein bands. (B, C) Representative senescent markers staining showing p53 and p16 (red) expression in lung sections of young and aged KLK8^+/+^ and KLK8^−/−^ mice. Endothelial cells were immunostained with anti‐CD31 (green). Nuclei were counterstained with DAPI (blue). Scale bar, 50 μm. The percentage of CD31^+^p53^+^ and CD31^+^p16^+^ cell numbers in total CD31^+^ cells were shown on the right. Data were presented as means ± SEM (*n* = 6). *p < 0.05, **p < 0.01. “You” represents “Young”.

To investigate whether KLK8 deficiency affected pulmonary endothelial senescence in vivo, we generated global deletion of KLK8 by crossing KLK8f/f mice with EIIa cre(+) mice. As expected, the upregulation of pulmonary KLK8 expression in aged mice was blunted in KLK8‐deficient (KLK8^−/−^) mice (Figure 3A). Aged mice exhibited increased pulmonary p53 and p21 expressions, which were significantly alleviated in KLK8‐deficient mice (Figure 3A). Notably, double immunofluorescence staining showed that KLK8 deficiency led to significant decreases in the percentages of p53^+^/CD31^+^, p21^+^/CD31^+^, and p16^+^/CD31^+^ cells in total CD31^+^ endothelial cells and p53^+^/ VE‐cadherin^+^, p21^+^/VE‐cadherin^+^, and p16^+^/VE‐cadherin^+^ cells in total VE‐cadherin^+^ endothelial cells in aged mice (Figure 3B,C; Figures S9A–C and S10A–C).

Taken together, these findings suggest that age‐dependent KLK8 upregulation contributes to pulmonary endothelial senescence.

### 2.3 KLK8 Upregulation Contributes to Elevated Susceptibility to Low‐Magnitude Mechanical Stretch in the Elderly

**FIGURE 4:**
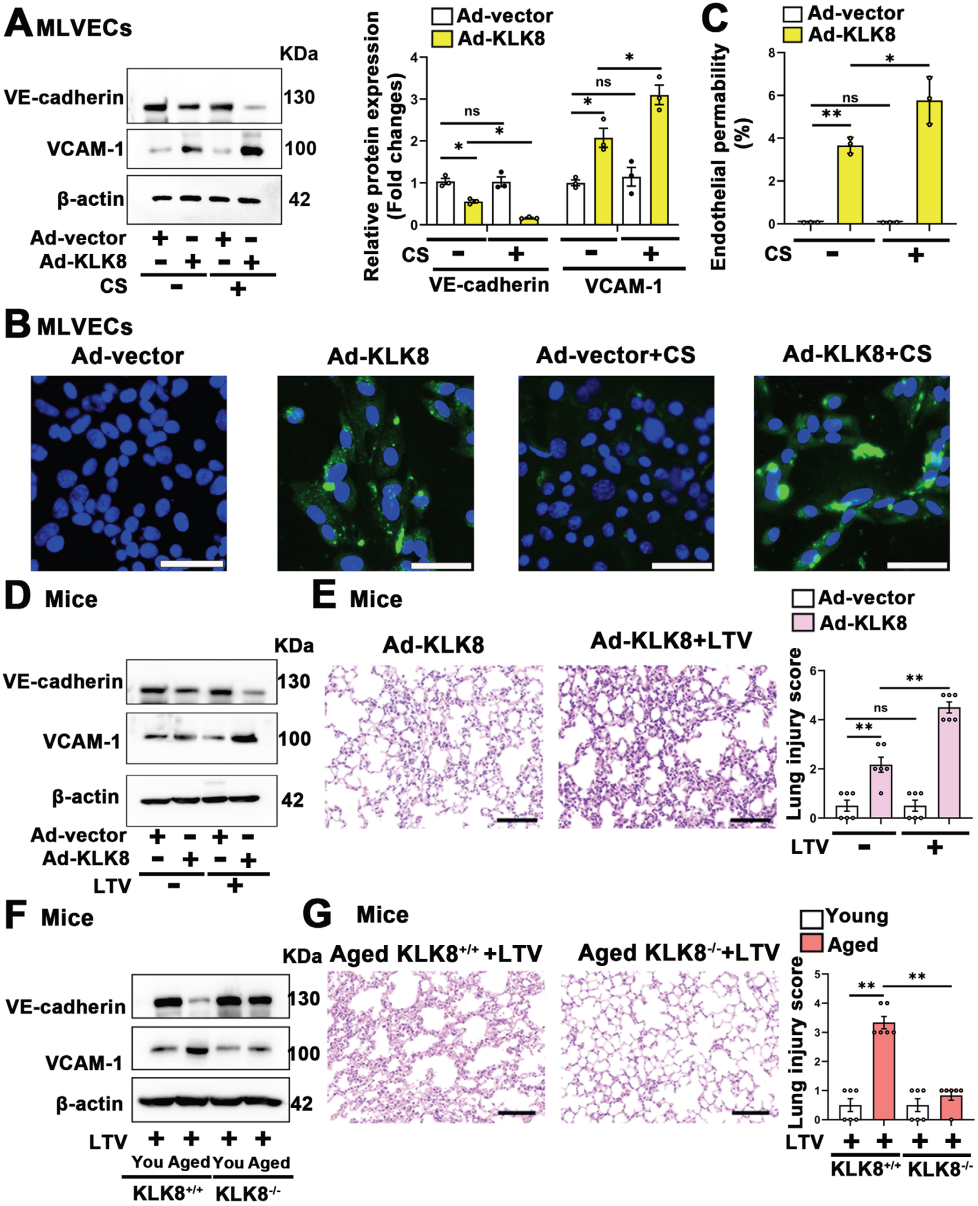
KLK8 upregulation contributes to elevated susceptibility to low‐magnitude mechanical stretch in the elderly. (A–C) Primary cultured MLVECs were transfected with KLK8 adenovirus (Ad‐KLK8) at MOI 1–10 or control adenovirus (Ad‐vector) at MOI 10. Seventy‐two hours later, cells were given 7% cyclic stretch (CS) for 4 h (*n* = 3). (A) Western blot showing VCAM‐1 and VE‐cadherin protein levels. Corresponding histograms were shown on the right of representative protein bands. (B) Primary cultured MLVECs were seeded on Collagen I coated Bioflex culture plates and the FITC fluorescence was detected as described in Materials and Methods. FITC fluorescence signal was visualized by fluorescence microscopy. (C) Quantification of FITC fluorescence signal by using Image J. Original magnification, × 200. Scale bar = 200 μm. (D, E) Young mice (2 months) were instilled intratracheally with Ad‐KLK8 or Ad‐vector. Seventy‐two hours later, they were subjected to low‐tidal‐volume (LTV) mechanical ventilation (8 mL/kg) for 4 h (*n* = 6). (D) Western blot showing VE‐cadherin and VCAM‐1 protein levels in lung tissues. (E) The left lower lung was used for histological evaluation by H&E staining. Original magnification, × 200. Scale bar = 100 μm. The severity of lung injury was scored by two pathologists blinded to group allocation. (F, G) Young (2 months) and aged (18 months) KLK8^+/+^ and KLK8^−/−^ mice were subjected to LTV mechanical ventilation (8 mL/kg) for 4 h (*n* = 6). (F) Western blot showing VE‐cadherin and VCAM‐1 protein levels in lung tissues. (G) The left lower lung was used for histological evaluation by H&E staining. Original magnification, × 200. Scale bar = 100 μm. The severity of lung injury was scored by two pathologists blinded to group allocation. Data were presented as means ± SEM. *p < 0.05, **p < 0.01. ns, not significant. “You” represents “Young.”

To investigate the role of KLK8 upregulation in the elevated susceptibility to mechanical stretch, we first examined the effect of Ad‐KLK8‐mediated KLK8 overexpression on endothelial cell sensitivity to mechanical stretch. In vitro studies were conducted by subjecting Ad‐KLK8 or Ad‐vector treated MLVECs to 7% cyclic stretch for 4 h. Ad‐KLK8‐treated MLVECs exhibited significant damage, evidenced by decreased VE‐cadherin expression, increased VCAM‐1 expression (Figure 4A), enhanced permeability of the MLVEC monolayer to FITC‐labeled avidin (Figure 4B,C), and disrupted membrane continuity of ZO‐1 between adjacent MLVECs (Figure S11A). Although 7% cyclic stretch alone did not induce endothelial cell damage, it significantly exacerbated KLK8 overexpression‐induced endothelial cell damage and hyperpermeability, as indicated by further decreased VE‐cadherin expression, increased VCAM‐1 expression (Figure 4A), enhanced endothelial monolayer permeability to FITC‐labeled avidin (Figure 4B,C), and impaired ZO‐1 membrane continuity between neighboring MLVECs (Figure S11A). These results suggest that KLK8 upregulation increases the sensitivity of pulmonary endothelial cells to low‐magnitude mechanical stretch.

We subsequently investigated the effects of intra‐pulmonary KLK8 overexpression on the sensitivity of pulmonary endothelial cells to LTV mechanical ventilation in young mice. It was found that intra‐pulmonary administration of Ad‐KLK8 led to decreased VE‐cadherin expression, increased VCAM‐1 expression (Figure 4D; Figure S12A), and higher lung injury scores, which were further aggravated by LTV mechanical ventilation (Figure 4E). Combining these in vitro and in vivo results, these findings suggest that KLK8 upregulation enhances the sensitivity of pulmonary endothelial cells to mechanical stretch.

H_2_O_2_‐induced senescent endothelial cells exhibited increased sensitivity to 7% cyclic stretch. We found that KLK8 siRNA‐mediated KLK8 knockdown significantly attenuated H_2_O_2_‐induced endothelial cell injury when exposed to 4 h of 7% cyclic stretch, as evidenced by increased VE‐cadherin expression and decreased VCAM‐1 expression (Figure S12B) and endothelial monolayer permeability (Figure S12C,D). We next explored the effect of KLK8 deficiency on the sensitivity of pulmonary endothelial cells to LTV mechanical ventilation in aged mice. LTV ventilation led to decreased VE‐cadherin expression, increased VCAM‐1 expression (Figure 4F; Figure S12E), and higher lung injury scores in aged mice, which were significantly improved by KLK8 deficiency (Figure 4G).

Combining the results from the KLK8‐overexpressing MLVECs, H_2_O_2_‐induced senescent MLVECs, intra‐pulmonary KLK8‐overexpressing mice, and KLK8‐deficient aged mice, these findings suggest that KLK8 upregulation contributes to elevated susceptibility to mechanical stretch in the elderly.

### 2.4 KLK8 Promotes Pulmonary Endothelial Senescence via Inactivation of the Fibronectin/FAK Signaling Pathway

**FIGURE 5:**
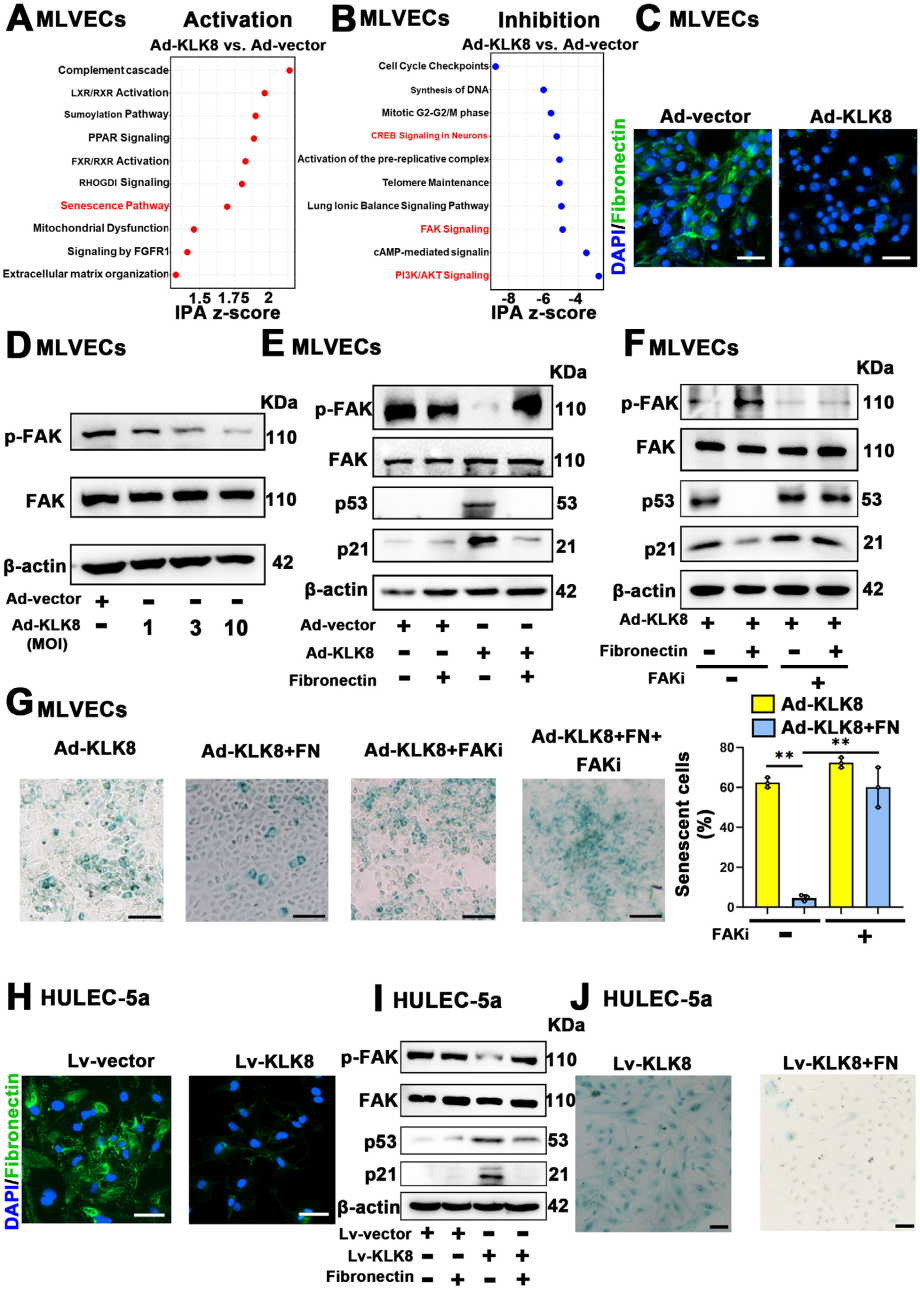
KLK8 promotes pulmonary endothelial senescence via inactivation of the fibronectin/FAK signaling pathway. (A, B) MLVECs were transfected with KLK8 adenovirus (Ad‐KLK8) or control adenovirus (Ad‐vector) at MOI 3. Forty‐eight hours later, total RNA of MLVECs was extracted for RNA‐seq analysis. (A) Ingenuity Pathway Analysis (IPA) activation *Z*‐scores for selected pathways in Ad‐KLK8 compared to Ad‐vector treated MLVECs. (B) Ingenuity Pathway Analysis (IPA) inhibition *Z*‐scores for selected pathways in Ad‐KLK8 compared to Ad‐vector MLVECs. (C, D) MLVECs were treated with Ad‐KLK8 (MOI 10) or Ad‐vector (MOI 10) for 72 h. (C) Representative fibronectin (FN) staining showing fibronectin (green) expression in MLVECs. Nuclei were counterstained with DAPI (blue). Scale bar, 200 μm. (D) Western blot showing p‐FAK/FAK protein levels in MLVECs. (E) MLVECs were seeded on plates coated with 5 μg/cm^2^ fibronectin. Twenty‐four hours later, MLVECs were treated with Ad‐KLK8 (MOI 10) or Ad‐vector (MOI 10) for 72 h. Western blot showing p‐FAK/FAK, p53 and p21 protein levels in MLVECs. (F, G) MLVECs were seeded on plates coated with 5 μg/cm^2^ fibronectin. Twenty‐four hours later, MLVECs were treated with Ad‐KLK8 (MOI 10) with or without FAK inhibitor (FAKi) Defactinib (5 μM) for 72 h. (F) Western blot showing p‐FAK/FAK, p53 and p21 protein levels in MLVECs. (G) Representative SA‐β‐gal staining. Scale bar, 100 μm. Quantification of SA‐β‐gal positive MLVECs was shown on the right. (H) Human pulmonary microvascular endothelial cells (HULEC‐5a) were treated with KLK8 lentivirus (Lv‐KLK8) or control lentivirus (Lv‐vector) at MOI 10. Representative fibronectin staining showing fibronectin expression in HULEC‐5a. Nuclei were counterstained with DAPI (blue). Scale bar, 50 μm. (I, J) HULEC‐5a were seeded on plates coated with 5 μg/cm^2^ fibronectin. Twenty‐four hours later, HULEC‐5a were treated with Lv‐KLK8 (MOI 10) for 72 h. (I) Western blot showing p‐FAK/FAK, p53 and p21 protein levels in HULEC‐5a. (J) Representative SA‐β‐gal staining. Scale bar, 100 μm. Data were presented as means ± SEM (*n* = 3). **p < 0.01.

To gain insights into the mechanisms responsible for KLK8‐induced endothelial senescence, we examined DEGs in Ad‐KLK8‐treated MLVECs using RNA‐Seq (Table S5). Among the DEGs (|log2FC| > 1, *p* < 0.05) identified between Ad‐vector and Ad‐KLK8‐treated MLVECs, 2158 (69%) were downregulated in KLK8‐overexpressing endothelial cells (Table S5). Pathway enrichment analysis using Ingenuity Pathway Analysis (IPA) revealed that signaling pathways associated with cell senescence, sumoylation, and mitochondrial dysfunction were significantly upregulated, while those related to cell cycle, synthesis of DNA and telomere maintenance were significantly downregulated in Ad‐KLK8‐treated MLVECs (Figure 5A,B). Notably, Ad‐KLK8 treatment profoundly downregulated FAK and cAMP response element binding protein (CREB) signaling pathways, both of which have been previously reported to inhibit endothelial cell senescence (Lee et al. 2015; Tien et al. 2023; Chu et al. 2024).

As a secreted serine protease, KLK8 is known to cleave extracellular matrix (ECM) proteins, including fibronectin (Rajapakse et al. 2005). Previous studies have demonstrated that fibronectin can improve endothelial function by activating the FAK signaling pathway (Jin et al. 2011; Benwell et al. 2021) and can also activate the CREB signaling pathway in astrocytes (Xia and Zhu 2014). Therefore, we hypothesized that KLK8 might induce endothelial senescence by promoting fibronectin degradation, thereby inactivating FAK and CREB signaling pathways. As shown in Figure 5C, Figure S13A, Ad‐vector‐treated MLVECs exhibited an obvious fibronectin network, which was disrupted in KLK8‐overexpressing MLVECs. Increasing concentrations of Ad‐KLK8 dose‐dependently decreased the phosphorylation of both FAK and CREB (Figure 5D; Figure S13B,C). To investigate whether exogenous supplementation of fibronectin could modulate KLK8 overexpression‐induced changes in endothelial cells, MLVECs were seeded on plates coated with 5 μg/cm^2^ fibronectin. Fibronectin supplementation significantly increased FAK phosphorylation but did not affect CREB phosphorylation in KLK8‐overexpressing endothelial cells (Figure 5E; Figure S13D,E). Moreover, fibronectin supplementation markedly alleviated KLK8 overexpression‐induced cell senescence, as evidenced by decreased p53/p21 expression (Figure 5E; Figure S13D) and reduced SA‐β‐Gal‐positive senescent MLVECs (Figure 5G). The FAK inhibitor defactinib, but not the CREB inhibitor KG‐501, significantly abolished the anti‐senescence effect of fibronectin on KLK8‐overexpressing endothelial cells (Figure 5F,G; Figure S13F–H).

We attempted to establish stable KLK8 overexpression in the human lung microvascular endothelial cell line HULEC‐5a using lentivirus‐mediated KLK8 overexpression (Lv‐KLK8). However, due to significant endothelial senescence induced by KLK8, HULEC‐5a cells treated with Lv‐KLK8 were unable to survive puromycin selection, primarily manifesting as arrested cell proliferation and an inability to re‐adhere after trypsin digestion. Therefore, we performed transient transfection using Lv‐KLK8 and found that KLK8‐overexpressing HULEC‐5a cells exhibited disrupted fibronectin networks (Figure 5H; Figure S14A), decreased FAK phosphorylation (Figure 5I; Figure S14C), and increased KLK8/p53/p21 expression (Figure 5I; Figure S14B,C) and SA‐β‐Gal‐positive senescent cells (Figure 5J; Figure S14D). Exogenous fibronectin supplementation similarly mitigated Lv‐KLK8‐induced FAK dephosphorylation and cell senescence in HULEC‐5a cells, mirroring the results observed in Ad‐KLK8‐treated MLVECs (Figure 5I,J; Figure S14C,D).

Collectively, these findings suggest that KLK8 promotes pulmonary endothelial senescence via inactivation of the fibronectin/FAK signaling pathway.

### 2.5 KLK8 Might Activate PARP1/2 via Inactivation of the Fibronectin/FAK/Akt Signaling Pathway, Thus Promoting Pulmonary Endothelial Senescence

**FIGURE 6:**
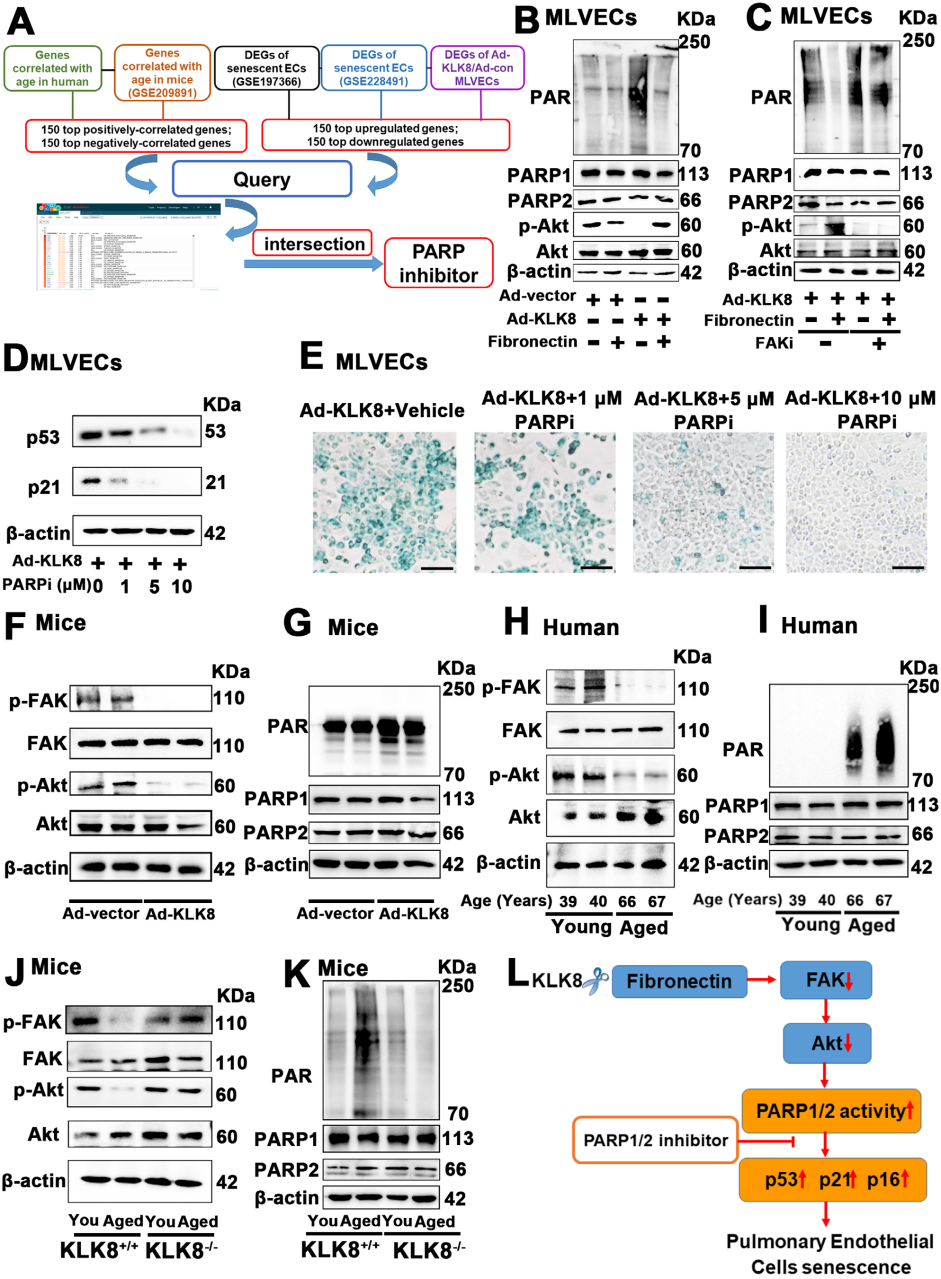
KLK8 activates PARP1/2 via inactivation of the fibronectin/FAK/Akt signaling pathway, thus promoting pulmonary endothelial senescence. (A) Flowchart of CMAP platform analysis. Top 150 genes positively and negatively correlated with age in human and mouse lungs, and differentially expressed genes (DEGs) in senescent endothelial cells were queried in the Connectivity Map (CMap) platform to identify candidate drugs. (B) MLVECs were seeded on plates coated with 5 μg/cm^2^ fibronectin. Twenty‐four hours later, MLVECs were treated with Ad‐KLK8 (MOI 10) or Ad‐vector (MOI 10) for 72 h. Western blot showing PARylation levels and p‐Akt/Akt protein levels in MLVECs (*n* = 3). (C) MLVECs were seeded on plates coated with 5 μg/cm^2^ fibronectin. Twenty‐four hours later, MLVECs were treated with Ad‐KLK8 (MOI 10) with or without the FAK inhibitor (FAKi) Defactinib (5 μM) for 72 h. Western blot showing PARylation levels and p‐Akt/Akt protein levels in MLVECs (*n* = 3). (D, E) MLVECs were treated with vehicle or olaparib (1, 5, 10 μM). Twenty‐four hours later, MLVECs were treated with Ad‐KLK8 at an MOI of 10 for 72 h (*n* = 3). (D) Western blot showing p53 and p21 protein levels in MLVECs. E, Representative SA‐β‐gal staining. Scale bar, 100 μm. (F, G) Young mice (2 months) were instilled intratracheally with Ad‐KLK8 or Ad‐vector. Seventy hours later, lung tissues were harvested for analysis (*n* = 6). (F) Western blot showing p‐FAK/FAK and p‐Akt/Akt protein levels in lung tissues. (G) Western blot showing PARylation levels in lung tissues. (H) Western blot showing p‐FAK/FAK and p‐Akt/Akt protein levels in lung tissues of young (13–40 years old) and aged (66–76 years old) humans (*n* = 6). (I) Western blot showing PARylation levels of young and aged humans (*n* = 6). (J) Western blot showing p‐FAK/FAK and p‐Akt/Akt protein levels in lung tissues of young (2 months) and aged (18 months) KLK8^+/+^ and KLK8^−/−^ mice (*n* = 6). (K) Western blot showing PARylation levels of young and aged KLK8^+/+^ and KLK8^−/−^ mice (*n* = 6). (L) Schematic diagram of the mechanism underlying KLK8‐induced pulmonary endothelial cell senescence. KLK8 cleaves extracellular matrix (ECM) protein fibronectin, leading to the inhibition of the fibronectin‐FAK‐Akt signaling pathway and subsequent PARP1/2 activation, contributing to pulmonary endothelial cell senescence. Data were presented as means ± SEM. “You” represents “Young.”

As shown in Figure 1A, Figure S4A–E, by analyzing transcriptomic sequencing data from human and mouse lung tissues across different ages, our study identified age‐related gene profiles. Additionally, DEGs were identified in KLK8‐induced senescent MLVECs, senescent endothelial cells from progeria mice, and naturally aged mice (Tables S3–S5). These alterations in gene expression detailed the genetic signature of aging lung tissues and endothelial cells. To identify compounds that could reverse these genetic alterations, we used the top 150 gene profiles with opposite alteration directions as signatures (Figure 6A; Table S6) to query the CMap platform. Special attention was given to drug candidates with negative connectivity scores, indicating their potential to revert the aging or senescence‐induced gene expression patterns. Intriguingly, the PARP1/2 inhibitor emerged as one of the top 40 drug candidates in CMap results derived from age‐related gene profiles in human lung tissues, DEGs in KLK8‐induced senescent MLVECs, and senescent endothelial cells from progeria mice or naturally aged mice (Figure 6A; Table S7). Additionally, the PARP1/2 inhibitor was also identified as a candidate drug in CMap results obtained from age‐related gene profiles in mouse lung tissues (Figure 6A, Table S7).

Among the 17 members of the human PARP family, PARP1 and PARP2 are the only known members involved in DNA repair (Petropoulos et al. 2024), with PARP1 being particularly implicated in aging‐related disorders, such as aging‐associated vascular barrier leakage (Pacher et al. 2004; Radovits et al. 2007; Zhang et al. 2015; Tarantini et al. 2019; Zhan et al. 2023). PARP1 activity has been reported to be induced by inhibition of the Akt signaling pathway (Zhang et al. 2022b). Notably, previous studies have shown that fibronectin can maintain endothelial homeostasis by activating the FAK/Akt signaling pathway (Jin et al. 2011). Therefore, we hypothesized that KLK8 might elevate PARP1/2 activity through inactivation of the fibronectin/FAK signaling pathway. As PARP1/2 activation creates poly(ADP‐ribose) (PAR) using nicotinamide adenine dinucleotide (NAD^+^) as a substrate (Kelleher et al. 2021; Szántó et al. 2024), and the total protein expression levels of PARP1 and PARP2 remained largely unchanged, PARP1/2 activity was assessed quantitatively by measuring PARylation levels using western blot (Zha et al. 2018). As shown in Figure 6B, Figure S15A, KLK8‐overexpressing MLVECs exhibited elevated PARP1/2 activity and reduced Akt phosphorylation, which could be reversed by fibronectin supplementation. Both the inhibitory effect of fibronectin on PARP1/2 activity and its stimulatory effect on Akt phosphorylation in KLK8‐overexpressing endothelial cells were blocked by the FAK inhibitor defactinib (Figure 6C; Figure S15B). Intriguingly, increasing concentrations of the PARP1/2 inhibitor olaparib (1–10 μM) dose‐dependently alleviated KLK8‐induced endothelial cell senescence, as evidenced by decreased p53/p21 expression (Figure 6D; Figure S15C) and reduced SA‐β‐Gal‐positive cells (Figure 6E; Figure S15D).

Consistent with in vitro findings, lung tissues from intra‐pulmonary KLK8‐overexpressing mice, aged mice, and aged patients exhibited decreased FAK and Akt phosphorylation and increased PARP1/2 activity (Figure 6F–K; Figure S15E–J). KLK8 deficiency significantly enhanced FAK and Akt phosphorylation and reduced PARP1/2 activity in lung tissues of aged mice (Figure 6J,K; Figure S15I–J). Collectively, these findings demonstrate that KLK8 cleaves the ECM protein fibronectin, thereby inhibiting the FAK‐Akt signaling pathway and subsequently activating PARP1/2, which contributes to pulmonary endothelial cell senescence (Figure 6L).

**FIGURE 7:**
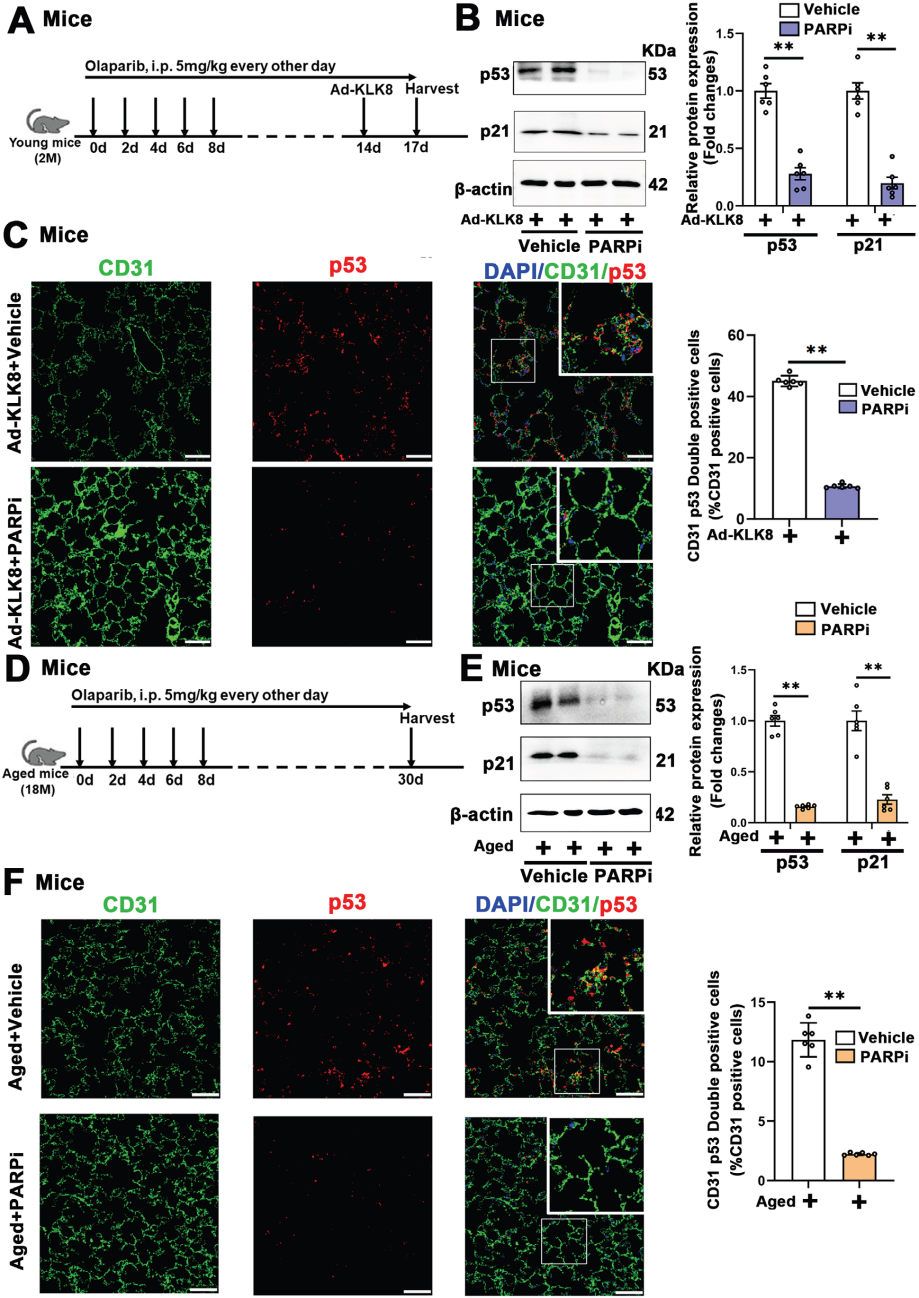
The PARP1/2 inhibitor alleviates pulmonary endothelial senescence in both intra‐pulmonary KLK8‐overexpressing mice and aged mice. (A–C) Young mice (2 months) were intraperitoneally injected with vehicle or the PARP1/2 inhibitor (PARPi) olaparib (5 mg/kg) in saline containing 0.1% DMSO every other day. Fourteen days later, they were intratracheally transfected with Ad‐KLK8 (1 × 10^8^ pfu). Seventy‐two hours after Ad‐KLK8 transfection, lung tissues were harvested for analysis. (A) Timeline of the experimental procedures. (B) Western blot showing p53 and p21 protein levels in lung tissues. Corresponding histograms were shown on the right of representative protein bands. (C) Representative senescent markers staining showing p53 (red) expression in lung sections. Endothelial cells were immunostained with anti‐CD31 (green). Nuclei were counterstained with DAPI (blue). Scale bar, 50 μm. The percentage of CD31^+^p53^+^ cell numbers in total CD31^+^ cells was shown on the right. (D–F) Aged mice were intraperitoneally injected with vehicle or olaparib (5 mg/kg) in saline containing 0.1% DMSO every other day. Thirty days later, lung tissues were harvested for western blot analysis and immunofluorescence. (D) Timeline of the experimental procedures. (E) Western blot showing p53 and p21 protein levels in lung tissues. Corresponding histograms were shown on the right of representative protein bands. (F) Representative senescent markers staining showing p53 (red) expression in lung sections. Endothelial cells were immunostained with anti‐CD31 (green). Nuclei were counterstained with DAPI (blue). Scale bar, 50 μm. The percentage of CD31^+^p53^+^ cell numbers in total CD31^+^ cells was shown on the right. Data were presented as means ± SEM (*n* = 6). **p < 0.01.

To evaluate the anti‐senescence effects of PARP1/2 inhibitors in vivo, intra‐pulmonary KLK8‐overexpressing mice and aged mice were intraperitoneally injected with olaparib at doses of 5 mg/kg for 14 and 30 days, respectively (Figure 7A,D). Olaparib treatment not only reduced PARP1/2 activity (Figure S16A,B) but also decreased p53 and p21 expressions in lung tissues of both intra‐pulmonary KLK8‐overexpressing mice (Figure 7B). and aged mice (Figure 7E). Notably, double immunofluorescence staining showed that olaparib treatment led to significant decreases in the percentages of p53^+^/CD31^+^, p21^+^/CD31^+^, and p16^+^/CD31^+^ cells among total CD31^+^ endothelial cells and percentages of p53^+^/ VE‐cadherin^+^, p21^+^/ VE‐cadherin^+^, and p16^+^/ VE‐cadherin^+^ cells among total VE‐cadherin^+^ endothelial cells in lung sections of intra‐pulmonary KLK8‐overexpressing mice (Figure 7C; Figures S17A,B and S18A–C) and aged mice (Figure 7F; Figures S19A,B and S20A–C).

Collectively, these findings suggest that KLK8 may activate PARP1/2 via inactivation of the fibronectin/FAK/Akt signaling pathway, thereby promoting pulmonary endothelial senescence. PARP1/2 inhibitors exhibit therapeutic potential to alleviate pulmonary endothelial senescence in both intra‐pulmonary KLK8‐overexpressing mice and aged mice.

### 2.6 The PARP1/2 Inhibitor Mitigates the Elevated Susceptibility to Low‐Magnitude Mechanical Stretch in Both Intra‐Pulmonary KLK8‐Overepxressing Mice and Aged Mice

**FIGURE 8:**
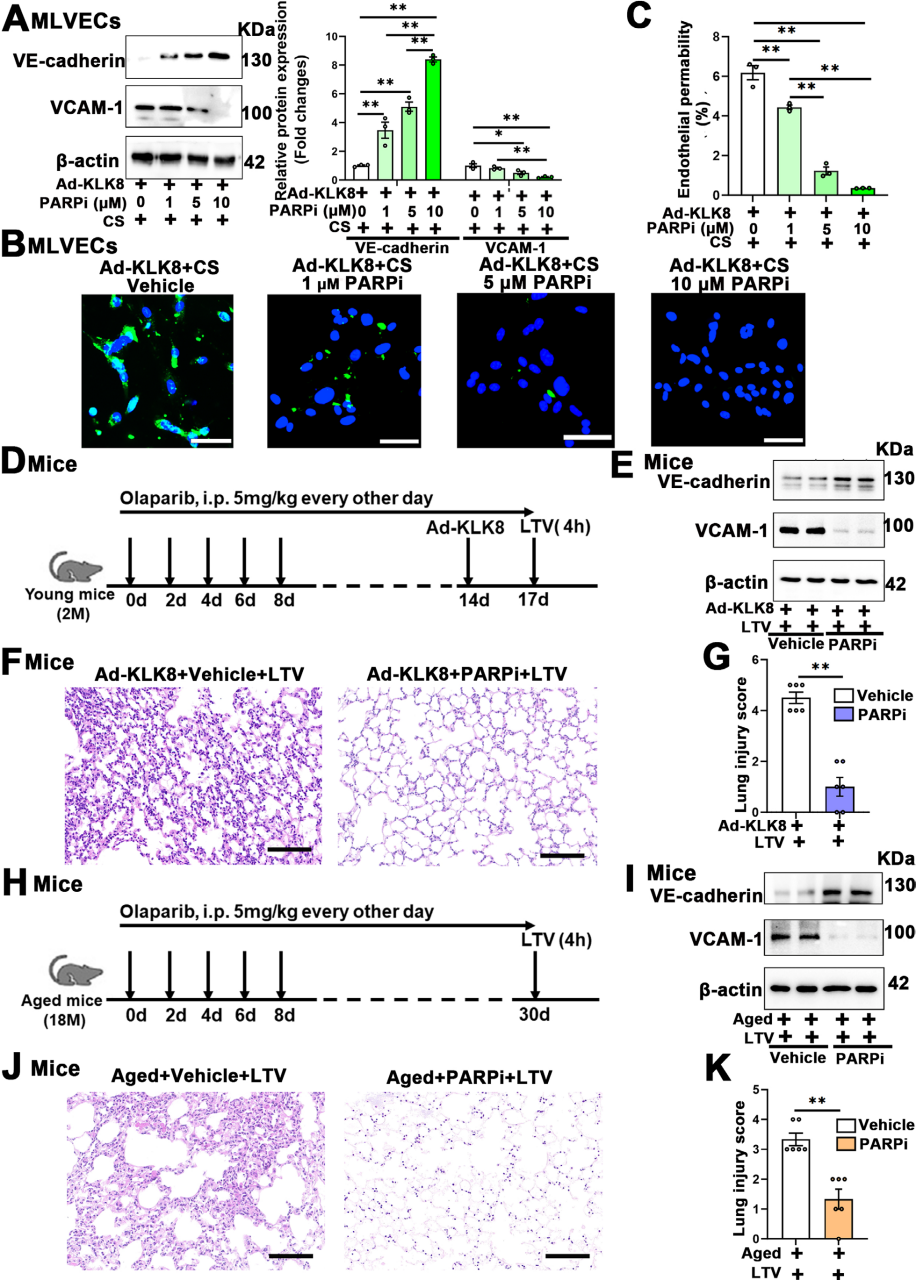
The PARP1/2 inhibitor mitigates the elevated susceptibility to low‐magnitude mechanical stretch in KLK8‐overexpressing MLVECs, both intra‐pulmonary KLK8‐overexpressing mice and aged mice. (A–C) MLVECs were treated with vehicle or olaparib (1, 5, 10 μM). Twenty‐four hours later, MLVECs were treated with Ad‐KLK8 at an MOI of 10. Seventy‐two hours later, MLVECs were subjected to physiological, low‐magnitude (7%) cyclic stretch (CS) for 4 h (*n* = 3). (A) Western blot showing VE‐cadherin and VCAM‐1 protein levels in MLVECs. Corresponding histograms were shown on the right of representative protein bands. (B), MLVECs were seeded on Collagen I coated Bioflex culture plates and the FITC fluorescence was detected as described in Materials and Methods. FITC fluorescence signal was visualized by fluorescence microscopy. (C) Quantification of FITC fluorescence signal by using ImageJ. Original magnification, × 200. Scale bar = 200 μm. (D–G) Young mice were intraperitoneally injected with vehicle or the PARP1/2 inhibitor olaparib (5 mg/kg) in saline containing 0.1% DMSO every other day. Fourteen days later, they were intratracheally transfected with Ad‐KLK8 (1 × 10^8^ pfu). Seventy‐two hours later, mice with intra‐pulmonary KLK8 overexpression that were treated with either vehicle or olaparib were subjected to low‐tidal‐volume (LTV) mechanical ventilation (8 mL/kg) for 4 h. Lung tissues were harvested for analysis (*n* = 6). (D) Timeline of the experimental procedures. (E) Western blot showing VE‐cadherin and VCAM‐1 protein levels in mice. (F) The left lower lung was used for histological evaluation by H&E staining. Original magnification, × 200. Scale bar = 100 μm. (G) The severity of lung injury was scored by two pathologists blinded to group allocation. (H–K) Aged mice were intraperitoneally injected with vehicle or olaparib (5 mg/kg) in saline containing 0.1% DMSO every other day. Thirty days later, aged mice treated with either vehicle or olaparib were subjected to LTV mechanical ventilation (8 mL/kg) for 4 h (*n* = 6). (H) Timeline of the experimental procedures. (I) Western blot showing VE‐cadherin and VCAM‐1 protein levels in lung tissues. (J) The left lower lung was used for histological evaluation by H&E staining. Original magnification, × 200. Scale bar = 100 μm. (K) The severity of lung injury was scored by two pathologists blinded to group allocation. Data were presented as means ± SEM. **p < 0.01.

As shown in Figure 4A–C, KLK8‐overexpressing endothelial cells exhibited increased sensitivity to low‐magnitude 7% cyclic stretch. We found that increasing concentrations of the PARP1/2 inhibitor olaparib (1–10 μM) dose‐dependently attenuated Ad‐KLK8‐induced endothelial cell injury when exposed to 4 h of 7% cyclic stretch, as evidenced by increased VE‐cadherin expression and decreased VCAM‐1 expression (Figure 8A) and endothelial monolayer permeability (Figure 8B,C).

In in vivo studies, we observed that LTV ventilation resulted in decreased VE‐cadherin expression, increased VCAM‐1 expression, and higher lung injury scores in both intra‐pulmonary KLK8‐overexpressing mice (Figure 4D,E; Figure S12A), and aged mice (Figure S2D–F). These effects were significantly improved by intraperitoneal injection of the PARP1/2 inhibitor olaparib (Figure 8D–K; Figure S21A,B).

These findings provide evidence indicating that the inhibition of PARP1/2 can mitigate the elevated susceptibility to low‐magnitude mechanical stretch in both intra‐pulmonary KLK8‐overexpressing mice and aged mice.

## 3 Discussion

Cellular senescence is a hallmark of lung aging, characterized by cell cycle arrest, distinct morphological changes, transcriptional alterations, and a unique secretory phenotype that modulates the local tissue microenvironment through autocrine and paracrine signaling (Xie et al. 2023). Senescent endothelial cells accumulate in the lungs with age, contributing significantly to the decline in lung function, lung remodeling, reduced proliferative capacity, and increased sensitivity to various lung diseases such as pulmonary fibrosis (Blandinières et al. 2019; Caporarello et al. 2022; Cheng et al. 2025), acute respiratory distress syndrome (ARDS) (D'Agnillo et al. 2021; Huang et al. 2023), and pulmonary arterial hypertension (Pei et al. 2025). However, the mechanisms underlying pulmonary endothelial senescence and how senescence increases susceptibility to lung injuries remain largely unclear. Through multiple RNA‐seq studies in humans and mice, as well as validation experiments, we found that the secreted serine protease KLK8 may inhibit the FAK/Akt signaling pathway and promote PARP1/2 activity by cleaving fibronectin, an ECM component. Its expression increases during the aging process, thereby promoting endothelial senescence. Our data also demonstrated enhanced KLK8 expression and PARP1/2 activity in lung tissues from patients over 65 years old compared to those aged less than 45 years. Consistent with these findings, recent studies have reported that KLK8 can cleave VE‐cadherin, leading to the upregulation of p53 and downregulation of forkhead box M1 (FOXM1), two key transcription factors that regulate cell proliferation and senescence in endothelial cells (Du et al. 2021; Zhao et al. 2025). These findings, together with our observations, suggest that KLK8 may regulate endothelial cell senescence by cleaving various membrane and ECM target proteins. These functions are further enhanced with age and conserved between mice and humans.

Increasing evidence from animal experiments has confirmed that aging increases susceptibility to VILI (Setzer et al. 2013; Herbert et al. 2016; Hay et al. 2024; Manji et al. 2025). Clinically, older patients exhibit higher mortality rates following even LTV protective lung ventilation compared to younger patients (Herbert et al. 2016; Bouza et al. 2021; Lim et al. 2021; Smolin et al. 2022; Liu et al. 2024; Valentine et al. 2023). The increased severity and mortality of VILI, combined with the greater need for mechanical ventilation among the elderly, underscores the importance of investigating changes within the aging lung. A key finding of our study is that age‐dependent upregulation of KLK8 and the consequent activation of PARP1/2 promote pulmonary endothelial cell senescence and heighten susceptibility to VILI. This represents the first report linking pulmonary endothelial cell senescence to increased vulnerability to VILI in aging. Notably, prior research has identified functional alterations in macrophages and type II alveolar epithelial cells in aged lungs as important contributors to increased VILI susceptibility (Valentine et al. 2018, 2023; Hay et al. 2024; Manji et al. 2025). Our double immunofluorescence staining further shows elevated KLK8 expression in non‐endothelial regions of both human and murine aging lung tissues. Given that our study used global KLK8 knockout mice and adenovirus‐mediated lung‐wide KLK8 overexpression without endothelial‐specific models, we cannot rule out contributions from non‐endothelial lung cells to the increased VILI susceptibility observed in aging. Future studies should employ endothelial cell‐specific KLK8 knockout models or targeted intrapulmonary endothelial overexpression strategies to definitively determine the role of endothelial KLK8 upregulation in age‐associated lung injury vulnerability.

FAK is a cytoplasmic tyrosine kinase uniquely positioned at the convergence point of integrin and tyrosine kinase signal transduction pathways, transmitting signals from the ECM to intracellular signaling molecules (Chen et al. 2023). Previous studies have demonstrated its essential role in maintaining vascular homeostasis (Tian et al. 2012; Visavadiya et al. 2016; Benwell et al. 2021; Akhter et al. 2024), suppressing endothelial apoptosis (Tian et al. 2012), and promoting angiogenesis (Jin et al. 2011; Tian et al. 2012, 2023). Notably, fibronectin has been shown to improve endothelial functions by activating the FAK signaling pathway (Jin et al. 2011; Benwell et al. 2021). As a secreted serine protease, KLK8 has been reported to remodel the extracellular microenvironment through the degradation of fibronectin (Sher et al. 2006). Our immunofluorescence results revealed that KLK8 disrupted the fibronectin network in the ECM of cultured endothelial cells, leading to FAK/Akt inactivation and increased PARP1/2 activity. These effects were reversed by exogenous supplementation of fibronectin. Furthermore, FAK inhibitors blocked the ability of fibronectin to inhibit KLK8‐induced PARP1/2 activation and endothelial senescence. Collectively, these findings suggest that upregulation of KLK8 promotes PARP1/2 activation and endothelial cell senescence by directly mediating the proteolytic cleavage of fibronectin and subsequent inactivation of the FAK/Akt signaling pathway.

PARP1/2 is known to transfer ADP‐ribose moieties from NAD+ to various acceptor proteins, thereby forming PAR and modulating essential cellular processes (Kelleher et al. 2021; Szántó et al. 2024). Excessive activation of PARP1 plays a critical role in the pathogenesis of endothelial senescence and dysfunction (Pacher et al. 2004; Radovits et al. 2007; Zhang et al. 2015; Tarantini et al. 2019; Zhan et al. 2023). Inhibition of PARP1 has been shown to protect against age‐related endothelial dysfunction in both cerebromicrovascular and cardiovascular systems (Pacher et al. 2004; Radovits et al. 2007; Zhang et al. 2015; Tarantini et al. 2019; Zhan et al. 2023). However, the mechanisms underlying age‐related PARP1/2 activation remain largely unknown. Zhan et al. (Zhan et al. 2023) recently reported that cerebral vascular endothelial cells in aging mice exhibit downregulation of connexin 43 (CX43), which interacts with and negatively regulates PARP1. Their findings establish the involvement of the endothelial CX43/PARP1 pathway in cerebromicrovascular aging and aging‐associated blood–brain barrier leakage. The present study demonstrated increased KLK8 expression and PARP1/2 activity, along with decreased FAK and Akt phosphorylation, in aged mice and human lung tissues. Age‐related changes in PARP1/2 activity and FAK/Akt phosphorylation in mouse lung tissues were significantly reversed by KLK8 deficiency. In vitro studies indicated that KLK8 promotes PARP1/2 activation and cell senescence in pulmonary microvascular endothelial cells by inactivating the fibronectin/FAK/Akt signaling pathway. Our findings not only reveal a novel mechanism involved in aging‐related PARP1/2 activation but also identify a potential therapeutic strategy targeting the KLK8/PARP signaling pathway to combat aging‐associated pulmonary endothelial dysfunction with lung‐protective implications.

Previous clinical and laboratory evidence has demonstrated that aging increases susceptibility to VILI. Until recently, many studies have utilized unphysiologically high tidal volumes (> 20 mL/kg) to establish rodent models of VILI in aged animals (Setzer et al. 2013; Herbert et al. 2016; Manji et al. 2025). Our data revealed that young and aged mice exhibited different basal levels of KLK8 expression and PARP1/2 activity, which might contribute to the heightened susceptibility of aged mice to even LTV (8 mL/kg) mechanical ventilation. Additionally, we observed improved resolution of VILI in aged mice with either KLK8 deficiency or PARP1/2 inhibition. Based on our findings, we propose that the Food and Drug Administration (FDA)‐approved drug olaparib may have potential therapeutic value in preventing VILI in older patients. Indeed, PARP1/2 inhibitors such as olaparib have been clinically used for treating solid tumors, including ovarian, breast, and prostate cancers (Bastos et al. 2024; Luo et al. 2024). Furthermore, clinical studies have confirmed that olaparib is an effective and safe PARP1/2 inhibitor (Drew et al. 2024; Montégut et al. 2024). However, caution is warranted when considering the use of olaparib to mitigate VILI susceptibility in elderly populations due to potential side effects, such as hematological toxicity (Liu et al. 2023; Wu et al. 2023).

There are two potential limitations to this study. First, we did not employ KLK8‐targeted therapies, such as KLK8 inhibitors, to investigate their effects on improving VILI in aged mice. This was due to the lack of clinical trial data ensuring the safety of KLK8 inhibitors, despite recent rapid progress in identifying these inhibitors (Masurier et al. 2018; Daneva et al. 2024). We opted for the PARP1/2 inhibitor olaparib for rescue studies, given its established clinical application in cancer therapy. Future research will focus on conducting preclinical experiments with novel KLK8 inhibitors to provide evidence of their safety and efficacy for potential clinical applications in preventing VILI in elderly individuals. Second, cellular senescence can be triggered by multiple intrinsic and extrinsic stressors, including but not limited to replicative stress, epigenetic changes, genomic instability, mitochondrial dysfunction, reactive metabolites, and oxidative stress (Zhang et al. 2022a; Wu et al. 2024). Although H_2_O_2_ remains the most widely used agent for inducing oxidative stress‐associated premature senescence, it cannot fully recapitulate the mechanisms underlying age‐related or KLK8‐induced endothelial senescence. Therefore, the potential involvement of KLK8 in other in vitro models of endothelial senescence induced by alternative stressors deserves further exploration.

In summary, our study elucidates a novel mechanism by which KLK8 induces endothelial senescence through modulation of the fibronectin‐FAK‐Akt‐PARP axis, thereby enhancing the susceptibility of senescent pulmonary endothelial cells to physiological low‐magnitude cyclic stretch and increasing the vulnerability of aged mice to LTV mechanical ventilation. Moreover, we propose the FDA‐approved PARP1/2 inhibitor olaparib as a potential therapeutic agent for alleviating the susceptibility of elderly individuals to VILI.

## 4 Materials and Methods

### 4.1 Lung Tissues of Patients With Intraoperative Mechanical Ventilation Support

The study was approved by the Ethics Committee of Xinhua Hospital, Shanghai Jiaotong University School of Medicine (XHEC‐C‐2023‐053‐1), and informed consent was obtained from all participants. This research project is registered in ClinicalTrials.gov (NCT06367946; https://clinicaltrials.gov). Details were described in the Supporting Information.

### 4.2 Animals

All laboratory mice used in this study were housed in a pathogen‐free facility at the Animal Research Center of Naval Medical University. The animal studies were conducted in accordance with the Guide for the Care and Use of Laboratory Animals published by the NIH (NIH publication No. 85‐23, revised 1996) and were approved by the Ethics Committee of Xinhua Hospital affiliated with Shanghai Jiaotong University School of Medicine. Details were described in the Supporting Information.

### 4.3 Murine Model of LTV Mechanical Ventilation

Mechanical ventilation was performed at a LTV of 8 mL/kg for 4 h, as previously described (Xu et al. 2019; Koh et al. 2021). Details were described in the Supporting Information.

### 4.4 Lung Histopathological Examination

The lung tissues were fixed in 4% paraformaldehyde and processed for hematoxylin and eosin (H&E) staining as previously described (Wang et al. 2021; Zhang et al. 2024). Details were shown in Supporting Information.

### 4.5 Experimental Groups and Drug Treatment

Details for the experimental groups and drug treatment were shown in Supporting Information.

### 4.6 Cell Culture and Infection of Adenovirus or Lentivirus

MLVECs were isolated from male ICR mice (3–4 weeks old) using a modified method as previously described (Wang et al. 2021; Zhang et al. 2024). The Ad‐KLK8 was designed and synthesized by Shanghai GeneChem Co. Ltd. (Shanghai, China) (Zhao et al. 2025).

The human lung microvascular endothelial cell line HULEC‐5a was purchased from Gineo Biotechnology (Guangzhou, China). The Lv‐KLK8 was designed and synthesized by Shanghai GeneChem Co. Ltd. (Shanghai, China) (Hua et al. 2021). Details for cell culture and infection were shown in Supporting Information.

### 4.7 RNA‐Seq and Bioinformatic Analysis

Total RNA was extracted from 18 human lung samples using the Trizol reagent (10296010, Invitrogen, CA, USA) according to the manufacturer's protocol. RNA‐seq analysis was performed by OE Biotech (Shanghai, China).

MLVECs for RNA‐seq experiments were seeded into 6‐well plates and infected with Ad‐KLK8 or Ad‐Vector at a multiplicity of infection (MOI) of 3 in serum‐free medium for 48 h. Total RNA was extracted from MLVECs using Trizol reagent. RNA‐seq analysis was performed by OE Biotech. RNA‐seq data of MLVECs have been deposited in the National Center for Biotechnology Information (NCBI) Gene Expression Omnibus (GEO) database under accession number GSE216969 (Zhao et al. 2025). Details are shown in Supporting Information.

### 4.8 The Analysis of Publicly Available Gene Expression Data

The bulk RNA‐seq dataset GSE209891 (Kawaguchi et al. 2023), the bulk RNA‐seq dataset GSE197366 (Gimbel et al. 2022) and the single‐nucleus RNA‐seq dataset GSE228491 (Ramadhiani et al. 2023) were obtained and downloaded from the Gene Expression Omnibus (GEO, http://www.ncbi.nlm.nih.gov/geo/) for analysis. Details were shown in Supporting Information.

### 4.9 Fibronectin Supplementation in MLVECs and HULEC‐5a

For fibronectin supplementation rescue experiments, MLVECs and HULEC‐5a cells were seeded onto plates precoated with 5 μg/cm^2^ fibronectin (F2006, Sigma‐Aldrich, MO, USA) (Schwefel et al. 2020; Camillo et al. 2021). Cells seeded on uncoated plates served as controls.

### 4.10 Cyclic Stretch

MLVECs were exposed to low‐magnitude (7% linear elongation, sinusoidal wave, 30 cycles/min) for 4 h using the Flexcell FX‐5000 Tension System, as previously described (Felder et al. 2019). Details were shown in Supporting Information.

### 4.11 Measurement of MLVECs Permeability

Endothelial permeability was assessed by express permeability testing assay (XperT), using a previously published technique (Wang et al. 2021). Details were shown in Supporting Information.

### 4.12 SA‐β‐Gal Staining

SA‐β‐gal staining was processed as previously published technique (Wang et al. 2024). Details were shown in Supporting Information.

### 4.13 Transfection of siRNA

Mouse KLK8 siRNA was synthesized by Genepharma Corp. (Shanghai, China). Transfection of siRNA in MLVECs was performed by using the XfectTM RNA transfection reagent (631450, Takara, Osaka, Japan) according to the manufacturer's instructions (Zhao et al. 2025). The target sequences for mouse KLK8 siRNA are shown in Supporting Information.

### 4.14 Western Blot

Details were shown in Supporting Information.

### 4.15 Quantitative Real‐Time Polymerase Chain Reaction (PCR) Analysis

Total RNA was extracted from lung tissues and MLVECs using Trizol Reagent. PrimeScript RT reagent Kit (RR047A, Takara, Osaka, Japan) with gDNA Eraser was used to reverse RNA into cDNA, followed by relative quantitative PCR using ChamQ Universal SYBR qPCR Master Mix (Q711, Vazyme, Nanjing, China). RT‐PCR was detected on Real‐Time Quantitative PCR instrument (Biorad). Primer information was shown in Table S8.

### 4.16 ELISA Determination of KLK8 in Human Lungs

The levels of KLK8 in human lungs were detected via enzyme‐linked immunosorbent assay (FS11820, Westtang, Shanghai, China) according to the manufacturer's instructions.

### 4.17 Detection of PARP1/2 Activity

PARP1/2 activation leads to the synthesis of PAR using NAD+ as a substrate (Kelleher et al. 2021; Szántó et al. 2024). To assess PARP1/2 activity, we quantified PARylation levels using western blot analysis (Zha et al. 2018).

### 4.18 Immunofluorescence Analysis

For immunofluorescence staining, MLVECs or HULEC‐5a cells were cultured on glass coverslips in 24‐well plates and transfected with Ad‐KLK8/Ad‐vector or Lv‐KLK8/Lv‐vector, respectively.

For immunofluorescence staining on paraffin sections, 4 μm‐thick lung tissue sections were rehydrated and antigen retrieval was performed by microwaving in citric acid buffer. Sections were incubated with 10% BSA (ST023, Beyotime, Shanghai, China) for 1 h to block non‐specific binding. Details were shown in Supporting Information.

### 4.19 CMap L1000 Query

At present, the CMap web portal (https://clue.io) can be used to predict the mechanisms of action of novel drugs and perform in silico screening for drug repurposing. In this study, we analyzed five datasets. To identify compounds that could reverse the genetic alterations, we used the top 150 gene profiles with opposite alteration directions as signatures (Table S6) to query the CMap platform. Special attention was given to drug candidates with negative connectivity scores, indicating their potential to revert aging or senescence‐induced gene expression patterns. Details are shown in Supporting Information.

### 4.20 Cell Treatments

H_2_O_2_ (323381, Sigma‐Aldrich, MO, USA) was used to induce MLVEC senescence at the dose of 250 μM. The PARP1/2 inhibitor olaparib (HY‐10162, MCE, Shanghai, China) was used at a concentration of 1, 5, and 10 μM to inhibit MLVEC senescence. Details were shown in Supporting Information.

### 4.21 Statistical Analysis

Data are expressed as mean ± SEM. Statistical analyses were done with Graphpad Prism 10.0 (GraphPad Software Inc., CA, USA). Details are shown in Supporting Information.

## Author Contributions

Xiao‐Yan Zhu and Lai Jiang conceptualized the study. Xiao‐Yan Zhu and Lai Jiang designed the experiments. Di Liu provided methodology, data curation and formal analysis. Tian‐Tian Lin provided additional methodological guidance and technical support. Hui Zhang, Ying Zhao, Chu‐Fan Xu, and Yu‐Jian Liu supervised the study. Di Liu wrote the manuscript. All authors reviewed and approved the manuscript.

## Funding

This work was supported by the National Natural Science Foundation of China, 82272229, 32171124, 32471185, 32271180, 82472187. Shanghai Healthcare System Key Supporting Discipline Construction Project, 2023ZDFC0202. Construction Project of the “Discipline Peak‐Climbing Plan” of Xinhua Hospital Affiliated to Shanghai Jiao Tong University School of Medicine, XKPF2024C103.

## Ethics Statement

Human study was approved by the Ethics Committee of Xinhua Hospital, Shanghai Jiaotong University School of Medicine (XHEC‐C‐2023‐053‐1) and all participants provided written informed consent.

## Conflicts of Interest

The authors declare no conflicts of interest.

## Supporting information


**Appendix S1:** acel70304‐sup‐0001‐AppendixS1.pdf.

## Data Availability

All data generated or analyzed during this study are included in this paper and its Supporting Information files. RNA‐seq data of MLVECs have been deposited in the National Center for Biotechnology Information (NCBI) Gene Expression Omnibus (GEO) database under accession number GSE216969.
