# Supplementary material for: Age‐Dependent KLK8 Upregulation Contributes to Elevated Susceptibility to Ventilator‐Induced Lung Injury in the Elderly Mice
Source: Aging Cell. 2025 Nov 27;25(1):e70304. doi: 10.1111/acel.70304 (PMC12741043; doi:10.1111/acel.70304)
Supplement: Supplementary file 1 — Appendix S1: acel70304‐sup‐0001‐AppendixS1.pdf. [file ACEL-25-e70304-s001.pdf]

Age-dependent KLK8 upregulation contributes to elevated susceptibility to ventilator-induced lung injury in the elderly mice

Di Liu, PhD[1,2#], Tian-Tian Lin[1#], Hui Zhang[1], Ying Zhao[3], Chu-Fan Xu[1], Yu-Jian Liu[3], Lai Jiang, PhD[1,*] and Xiao-Yan Zhu, PhD[2,*]

1. Department of Anesthesiology and Surgical Intensive Care Unit, Xinhua Hospital, Shanghai Jiaotong University School of Medicine, Shanghai, 200092, China.
2. Department of Physiology, Naval Medical University, Shanghai, 200433, China.
3. School of Kinesiology, The Key Laboratory of Exercise and Health Sciences of Ministry of Education, Shanghai University of Sport, Shanghai, 200438, China.

# Di Liu and Tian-Tian Lin contributed equally to this work.

*Corresponding authors: Prof. Xiao-Yan Zhu, Department of Physiology, Naval Medical University, 800 Xiangyin Road, Shanghai, China. E-mail: xiaoyanzhu@smmu.edu.cn. Dr. Lai Jiang, Department of Anesthesiology and Surgical Intensive Care Unit, Xinhua Hospital, Shanghai Jiaotong University School of Medicine, Shanghai, China; E-mail: jianglai@xinhuamed.com.cn;

Supplementary Materials and Methods

Lung Tissues of Patients with Intraoperative Mechanical Ventilation Support

The study was approved by the Ethics Committee of Xinhua Hospital, Shanghai Jiaotong University School of Medicine (XHEC-C-2023-053-1), and informed consent was obtained from all participants. This research project is registered in ClinicalTrials.gov (NCT06367946; https://clinicaltrials.gov). Adult patients scheduled for elective pulmonary lobectomy under general anesthesia with mechanical ventilation were included if they were classified as American Society of Anesthesiologists (ASA) Physical Status I to III. Exclusion criteria comprised a history of any lung disease or distant metastases, recent exposure to anesthetics or mechanical ventilation, and participation in another clinical trial. All patients underwent general anesthesia with double-lumen endotracheal intubation and received a standardized mechanical ventilation protocol: tidal volume of 6-8 mL/kg, positive end-expiratory pressure (PEEP) of 5 cm H_2O, inspired oxygen concentration of 40%, respiratory rate of 10-15 breaths per minute, and an inspiratory-to-expiratory ratio of 1:2. The ventilation protocol could be adjusted at any time based on patient safety concerns or at the surgeon's discretion; however, patients whose protocols were altered were excluded from the study. Investigators recorded the duration from the initiation of mechanical ventilation to the start of one-lung ventilation. Following lung resection surgery, non-tumor tissues from the excised specimens were dissected into small pieces, washed with saline to remove residual blood, and processed accordingly. Some samples were transferred to clean tubes, immediately frozen in liquid nitrogen, and stored at −80 °C

for ELISA and Western Blot analysis. Other samples were fixed in 4% paraformaldehyde (BL539A, Biosharp, Hefei, China) for immunofluorescence experiments.

Animals

All laboratory mice used in this study were housed in a pathogen-free facility at the Animal Research Center of Naval Medical University. The animal studies were conducted in accordance with the Guide for the Care and Use of Laboratory Animals published by the NIH (NIH publication No. 85-23, revised 1996) and were approved by the Ethics Committee of Xinhua Hospital affiliated to Shanghai Jiaotong University School of Medicine. The tissue kallikrein-related peptidase 8 (KLK8)-flox mouse line was generated at Shanghai Biomodel Organism Science & Technology Development Co., Ltd. (Shanghai, China) using a LoxP targeting system with two LoxP elements flanking exons 1-3 of the KLK8 gene. Specifically, the two LoxP elements were inserted into the KLK8 gene via homologous recombination in embryonic stem cells. To generate global KLK8 knockout mice, KLK8-flox mice were crossed with EIIa-Cre transgenic mice (The Jackson Laboratory). Deletion of the KLK8 gene was confirmed by PCR analysis of genomic DNA using the following primers as previously described (Xu et al. 2023): forward (5’-GGACGTTGGAGTCACAGC-3’) and reverse (5’-CCCAGGAGCAGAAGAGTG-3’). KLK8flox/flox; EIIa-Cre(+) mice (KLK8$^{-/-}$), along with age-matched KLK8flox/flox; EIIa-Cre(-) littermates as controls, were utilized to investigate the implications of KLK8 deficiency.

Animal experiments were randomized using a random number table. During the measurement and analysis phases of all *in vivo* studies, both genotype and treatment were blinded to ensure unbiased results. To eliminate potential sex-related variability, only male mice were included in the experiments.

Murine model of low-tidal-volume (LTV) mechanical ventilation

The mice were anesthetized via intraperitoneal injection with a combination of 70 mg/kg ketamine and 10 mg/kg xylazine, followed by tracheotomy using a 20 G intravenous catheter. They were then connected to a ventilator (Inspira, Harvard Apparatus Ltd., Boston, MA, USA). Mechanical ventilation was performed at a LTV of 8 mL/kg for 4 hours, as previously described (Xu et al. 2019; Koh et al. 2021). Control mice underwent identical surgical procedures but were allowed to breathe spontaneously. At the end of the experiment, the mice were euthanized by exsanguination under anesthesia, and lung tissue samples were collected for analysis.

Lung Histopathological Examination

The lung tissues were fixed in 4% paraformaldehyde and processed for hematoxylin and eosin (H&E) staining. The lung injury score was assessed by two blinded pathologists with expertise in lung pathology, using a previously reported grading system (Wang et al. 2021; Zhang et al. 2024): 0 = normal tissue; 1 = tiny inflammatory change; 2 = mild to moderate inflammatory changes without marked damage in the lung architecture; 3 = moderate inflammatory injury with thickening of the alveolar septa; 4 = moderate to severe inflammatory injury with formation of

nodules or areas of pneumonitis; and 5 = severe inflammatory injury with total obliteration of the field. The mean score for each section was calculated.

Experimental groups and drug treatment

For the first part of the study, experiments were designed to investigate the impact of KLK8 overexpression on endothelial senescence and ventilator-induced lung injury (VILI). Young mice (2 months old) were randomly assigned to four groups: (a) Ad-vector group: Mice were intratracheally injected with Ad-vector. Seventy-two hours later, they underwent the same surgical procedures but were allowed to breathe spontaneously for 4 hours. (b) Ad-KLK8 group: Mice were intratracheally injected with Ad-KLK8. Seventy-two hours later, they underwent the same surgical procedures but were allowed to breathe spontaneously for 4 hours. (c) Ad-vector + LTV group: Mice were intratracheally injected with Ad-vector. Seventy-two hours later, mice were subjected to LTV mechanical ventilation (8 ml/kg) for 4 hours. (d) Ad-KLK8 + LTV group: Mice were intratracheally injected with Ad-KLK8. Seventy-two hours later, mice were subjected to LTV mechanical ventilation (8 ml/kg) for 4 hours. For intra-pulmonary adenovirus transfection, mice were intratracheally instilled with 1 × 10^8 plaque-forming units (pfu) of Ad-KLK8 or Ad-vector in 30 μL of enhanced infection solution. The dose of adenovirus was determined based on previous reports and our preliminary experiments (Zhao et al. 2025). Lung tissues were harvested for Western Blot analysis, immunofluorescence, and H&E staining.

For the second part of the study, the experiments were designed to investigate the

effects of KLK8 deficiency on endothelial senescence and VILI. Mice were randomly assigned to four groups: (a) Young $KLK8^{+/+}$ group (2 months old). (b) Aged $KLK8^{+/+}$ group (18 months old). (c) Young $KLK8^{-/-}$ group (2 months old). (d) Aged $KLK8^{-/-}$ group (18 months old). Lung tissues were harvested for Western Blot analysis and immunofluorescence to assess pulmonary endothelial cell senescence. To examine the impact of KLK8 deficiency on VILI, young and aged $KLK8^{+/+}$ or $KLK8^{-/-}$ mice were subjected to LTV mechanical ventilation (8 ml/kg) for 4 hours. Lung tissues were harvested for Western Blot analysis and H&E staining.

For the third part of the study, we aimed to investigate the effects of systemic administration of the poly(ADP-ribose) polymerase 1/2 (PARP1/2) inhibitor olaparib on endothelial senescence and VILI in mice with intra-pulmonary KLK8 overexpression. Olaparib (5 mg/kg**,** HY-10162, MCE, Shanghai, China) was dissolved in saline containing 0.1% DMSO. Young mice (2 months old) were randomly allocated to the following groups: (a) Ad-KLK8 + vehicle group: Mice were intraperitoneally injected with vehicle (saline with 0.1% DMSO) every other day for 14 days. Fourteen days later, they were intratracheally transfected with Ad-KLK8 (1 × 10^8 pfu). (b) Ad-KLK8 + olaparib group: Mice were intraperitoneally injected with olaparib (5 mg/kg) in saline containing 0.1% DMSO every other day for 14 days. Fourteen days later, they were intratracheally transfected with Ad-KLK8 (1 × 10^8 pfu). Seventy-two hours after Ad-KLK8 transfection, lung tissues were harvested for Western Blot analysis and immunofluorescence to assess pulmonary endothelial cell senescence. To examine the impact of systemic administration of olaparib on VILI, mice with intra-pulmonary

KLK8 overexpression that were treated with either vehicle or olaparib were subjected to LTV mechanical ventilation (8 mL/kg) for 4 hours. Lung tissues were harvested for Western Blot analysis and H&E staining. The selection of the olaparib dosage was based on previous study (Zhan et al. 2023) and our preliminary experiments.

For the fourth part of the study, we aimed to investigate the effects of systemic administration of the PARP1/2 inhibitor olaparib on endothelial senescence and VILI in naturally aged mice. Aged mice (18 months old) were randomly allocated to the following groups: (a) Aged + vehicle group: Aged mice were intraperitoneally injected with vehicle every other day. (b) Aged + olaparib group: Aged mice were intraperitoneally injected with olaparib (5 mg/kg) every other day. Thirty days later, lung tissues were harvested for Western Blot analysis and immunofluorescence to assess pulmonary endothelial cell senescence. To examine the impact of systemic administration of olaparib on VILI, aged mice treated with either vehicle or olaparib were subjected to LTV mechanical ventilation (8 mL/kg) for 4 hours. Lung tissues were harvested for Western Blot analysis and H&E staining.

Cell culture and infection of adenovirus or lentivirus

Mouse lung vascular endothelial cells (MLVECs) were isolated from male ICR mice (3-4 weeks old) using a modified method as previously described (Wang et al. 2021; Zhang et al. 2024). Briefly, the right ventricle of anesthetized mice was perfused with Dulbecco's Modified Eagle Medium (DMEM, C11995500BT, Gibco, CA, USA) to remove residual blood from the lungs. Peripheral subpleural pulmonary tissue was

dissected into small pieces and cultured in DMEM supplemented with 20% fetal bovine serum (FBS, 10099-141, Gibco, CA, USA) under a 5% CO_2 atmosphere at 37°C for 60 hours. The diced tissue was then removed, and the adherent cells were maintained in DMEM with 10% FBS. MLVECs that had been passaged three or four times were used for experiments. The KLK8 adenovirus (Ad-KLK8) was designed and synthesized by Shanghai GeneChem Co., Ltd. (Shanghai, China) (Zhao et al. 2025). Transient transfection of Ad-KLK8 into MLVECs was performed according to the manufacturer's protocol in DMEM medium supplemented with 10% FBS.

The human lung microvascular endothelial cell line HULEC-5a was purchased from Gineo Biotechnology (Guangzhou, China) and cultured in DMEM supplemented with 10% FBS, 1% penicillin/streptomycin (15140-122, Gibco, CA, USA), and maintained at 37°C in a humidified atmosphere of 95% air and 5% CO2. The KLK8 lentivirus (Lv-KLK8) was designed and synthesized by Shanghai GeneChem Co., Ltd. (Shanghai, China) (Hua et al. 2021). To construct stable KLK8-overexpressing cell lines, Lv-KLK8 was transfected into HULEC-5a cells according to the manufacturer’s instructions. Briefly, HULEC-5a cells were seeded in 6-well plates at a density of 1 × 10^5 cells/well one day before infection. The next day, lentivirus was added to the culture medium. Twenty-four hours post-infection, the viral medium was replaced with fresh culture medium. Seventy-two hours after transfection, puromycin (2 μg/ml; ST551, Beyotime, Shanghai, China) was added to select for antibiotic-resistant cells. Transient transfection of Lv-KLK8 into HULEC-5a cells was performed according to the manufacturer's protocol in DMEM medium supplemented with 10% FBS.

RNA sequencing (RNA-seq) and bioinformatic analysis

Total RNA was extracted from 18 human lung samples using the Trizol reagent (10296010, Invitrogen, CA, USA) according to the manufacturer's protocol. RNA-seq analysis was performed by OE Biotech (Shanghai, China). The constructed library was quality-controlled using an Agilent 2100 Bioanalyzer, and sequencing was conducted on the Illumina NovaSeq 6000 platform. Gene expression levels were quantified in fragments per kilobase of exon model per million mapped reads (FPKM). For the batch assessment of correlations between gene expression and age in the RNA-seq dataset of human lung tissues, Pearson correlation analysis was performed using the rcorr function from the R package Hmisc (v5.2-3), and the results were visualized using the pheatmap package (v1.0.13) in RStudio (version 4.0.3) (Ortega et al. 2024).

MLVECs for RNA-seq experiments were seeded into 6-well plates and infected with Ad-KLK8 or Ad-Vector at a multiplicity of infection (MOI) of 3 in serum-free medium for 48 hours. After the treatment, MLVECs were collected for RNA-seq analysis. Total RNA was extracted from MLVECs using Trizol reagent. RNA-seq analysis was performed by OE Biotech. The constructed library was quality-controlled using an Agilent 2100 Bioanalyzer, and sequencing was conducted on the Illumina HiSeq™ 2500 platform. Gene expression levels were quantified in FPKM. Genes with a p-value < 0.05 and $|\log2FC| \geq 1$ were considered significantly differentially expressed genes (DEGs). Ingenuity Pathway Analysis (IPA, QIAGEN, Redwood City, CA) was used to perform "Core Analysis" to identify significant pathways associated with the DEGs. RNA-seq data of MLVECs have been deposited in the National Center for

Biotechnology Information (NCBI) Gene Expression Omnibus (GEO) database under accession number GSE216969 (Zhao et al. 2025).

The Analysis of Publicly Available Gene Expression Data

The bulk RNA-seq dataset GSE209891 (Kawaguchi et al. 2023) was obtained and downloaded from the Gene Expression Omnibus (GEO, http://www.ncbi.nlm.nih.gov/geo/). This dataset includes lung samples from male C57Bl/6J mice at 3, 6, 12, and 24 months of age. Gene expression levels were extracted separately. For the batch assessment of correlations between gene expression and age in the RNA-seq dataset of mice lung tissues, Pearson correlation analysis was performed using the rcorr function from the R package Hmisc (v5.2-3), and the results were visualized using the pheatmap package (v1.0.13) in RStudio (version 4.0.3) (Ortega et al. 2024). The bulk RNA-seq dataset GSE197366 (Gimbel et al. 2022) was retrieved from GEO datasets, containing endothelial cells from male C57BL/6 mice at 8 weeks and 80 weeks of age. The file " Old_d-flow_vs._Young_d-flow_Comparison_All_No_cutt_off.xlsx," which contains DEGs, was downloaded from GEO.

The single-nucleus RNA-seq dataset GSE228491 (Ramadhiani et al. 2023) was obtained from GEO datasets, containing nuclei from two replicates of progeria mice and wild-type (WT) mouse lungs. The dataset was analyzed using the Seurat package (v5.1.0). Firstly, quality control was performed using the PercentageFeatureSet function, retaining cells with a unique gene count greater than 500 and mitochondrial

gene content less than 20%. Subsequently, data normalization was conducted using the NormalizeData function. The Harmony method was employed for batch correction and sample merging, while Uniform Manifold Approximation and Projection (UMAP) was used for nonlinear dimensionality reduction. Major cell types were annotated at a resolution of 0.2, including endothelial cells ($Pecam1^{+}Ptprc^{-}$), epithelial cells ($Epcam^{+}Cdh1^{+}$), mesenchymal cells ($Ptprc^{-}Epcam^{-}Pecam1^{-}$), and immune cells ($Ptprc^{+}$). DEGs were identified using the FindMarkers function, filtering out genes expressed in fewer than 10% of cells.

Cyclic stretch

For cyclic stretch, MLVECs were seeded onto collagen I-coated Bioflex® six-well culture plates at density 5×10^{5} cells per well and cultured for 48 h to reach confluence. MLVECs were then exposed to low-magnitude (7% linear elongation, sinusoidal wave, 30 cycles/min) for 4 hours using the Flexcell® FX-5000 Tension System, as previously described (Felder et al. 2019). The cells that did not receive cyclic stretch were placed in the same incubator next to cyclic stretched cells. The cells and culture supernatants were collected after mechanical stretch for analyses.

Measurement of MLVECs permeability

Endothelial permeability was assessed by express permeability testing assay (XperT), using a previously published technique (Wang et al. 2021). The assay is based on high-affinity binding of avidin-conjugated, FITC-labeled tracer to the biotinylated extracellular matrix (ECM) proteins immobilized on the bottom of culture dishes

covered with MLVEC monolayers. Permeability assays were performed in a 25 mm BioFlex loading station. The BioFlex plates were coated with biotinylated gelatin (G2500, Sigma-Aldrich, MO, USA), and MLVECs seeded at a density of 5×10^5 cells per well and grown for 48 h to reach confluence. After the cyclic stretch, cells were fixed with 3.7% formaldehyde, and FITC-avidin (25 μg/ml, D111086-0001, Sangon Biotech, Shanghai, China) was added to the cultured medium for 3 min, then mounted with 4',6-diamidino-2-phenylindole (DAPI, C1006, Beyotime, Shanghai, China). After washing, the elastic bottoms of the BioFlex plates containing the MLVECs were excised with a scalpel and transferred to a microslide, and FITC-avidin fluorescence was measured with fluorescence microscopy (BX53; Olympus, Japan).

Senescence-associated β-galactosidase (SA-β-gal) staining

SA-β-gal staining kit (G1073-100T) was purchased from Servicebio (Wuhan, China). Cells were fixed at room temperature for 15 min by adding 1 mL of fixation buffer. The staining mixture was added and incubated overnight at 37 ◦C. The next day, the cells were washed with PBS (C0221A, Beyotime, Shanghai, China), and the staining results were observed under a microscope. SA-β-Gal-positive cells (bluish-green color) were counted in three random microscopic fields and expressed as % of total cells (Wang et al. 2024).

Transfection of Small interfering RNA (siRNA)

Mouse KLK8 siRNA was synthesized by Genepharma Corp. (Shanghai, China). The target sequences for mouse KLK8 siRNA are as follows: 5’-GGCCAGAAGUGCAUCAUAUTT-3’. Negative control siRNA was scrambled

sequence without any specific target: 5’-UUCUCCGAACGUGUCACGUTT -3’. Transfection of siRNA in MLVECs was performed by using the XfectTM RNA transfection reagent (631450, Takara, Osaka, Japan) according to the manufacturer’s instructions (Zhao et al. 2025). The above sequences were validated with no off-target effects.

Western Blot

Lung tissue, MLVECs and HULEC-5a cells were lysed using chilled RIPA lysis buffer (P0013B, Beyotime, Shanghai, China) containing protease and phosphatase inhibitor cocktail (P1045, Beyotime, Shanghai, China) in accordance with the manufacturer's instructions. Protein concentration was determined by BCA Protein Assay Kit (P0010, Beyotime, Shanghai, China). Equal amounts of protein were separated by 10% SDS-PAGE and transferred to PVDF membrane (IPVH00010, Merck, Barmstedt, Germany). The membranes were blocked with 5% nonfat milk and then incubated with primary antibodies. Primary antibodies included: anti-KLK8 (1:1000, 14232-1-AP, Proteintech, Wuhan, China), anti-p53 (1:1000, 10442-1-AP, Proteintech, Wuhan, China), anti-p21 (1:1000, 28248-1-AP, Proteintech, Wuhan, China), anti-VCAM-1 (1:1000, GB113376, Servicebio, Wuhan, China), anti-VE-cadherin (1:1000, ab205336, Abcam, Cambridge, UK), anti-phospho-FAK (Tyr397) (1:1000, 3283S, Cell Signaling Technology, USA), anti-FAK (1:1000; 3285T, Cell Signaling Technology, MA, USA), anti-phospho-Akt (Ser473) (1:1000, 9271S, Cell Signaling Technology, MA, USA), anti-Akt (1:1000, 60203-2-Ig, Proteintech, Wuhan, China), anti-PARP1 (1:1000, 13371-1-AP, Proteintech, Wuhan, China), anti-PARP2 (1:1000, 55149-1-AP, Proteintech, Wuhan, China), anti-αSMA (1:1000; 67735-1-Ig, Proteintech, Wuhan, China), anti-Collagen I (1:1000; GB11022, Servicebio, Wuhan, China), anti-PAR (1:500, sc-56198, santa cruz, TX, USA), anti-phospho-CREB (Ser133) (1:1000, 28792-1-AP, Proteintech, Wuhan, China), anti CREB1 (1:1000, 12208-1-AP, Proteintech, Wuhan, China), and anti-β-actin (1:3000; A1978, Sigma-Aldrich, MO, USA). HRP conjugated anti-rabbit (SA00001-2) and anti-mouse (SA00001-1) secondary antibodies were purchased from Proteintech (Wuhan, China).

Immunofluorescence Analysis

For immunofluorescence staining, MLVECs or HULEC-5a cells were cultured on glass coverslips in 24-well plates and transfected with Ad-KLK8/Ad-vector or Lv-KLK8/Lv-vector, respectively. After 72 hours of transfection, the cells on coverslips were washed twice with PBS and fixed with 4% paraformaldehyde for 30 minutes at room temperature. Following three washes with PBS (5 minutes each), cells were blocked with blocking solution (5% BSA in TBS containing 0.5% Triton X-100, ST023, Beyotime, Shanghai, China) for 2 hours. The cells were then incubated with the primary antibody against fibronectin (1:100, 66042-1-lg, Proteintech, Wuhan, China) and ZO-1 (1:100, 21773-1-AP, Proteintech, Wuhan, China) followed by coraLite488-conjugated anti-mouse (1:100, SA00013-1, Proteintech, Wuhan, China) and coraLite488-conjugated anti- rabbit (1:100, SA00013-2, Proteintech, Wuhan, China). Nuclei were counterstained with DAPI, and images were captured using fluorescence microscopy (BX53; Olympus, Japan) and analyzed using lmage J software.

For immunofluorescence staining on paraffin sections, 4 μm-thick lung tissue sections were rehydrated and antigen retrieval was performed by microwaving in citric acid buffer. Sections were incubated with 10% BSA for 1 hour to block non-specific binding. After incubation with 10% BSA for 1 h, the sections were incubated with primary antibodies included: anti-KLK8 (1:100; 14232-1-AP, Proteintech, Wuhan, China), anti-p53 (1:100, 10442-1-AP, Proteintech, Wuhan, China), anti-p21 (1:100; GB11153, Servicebio, Wuhan, China), anti-p16 (1:100; ab51243, Abcam, Cambridge, UK), anti-CD31 (1:100, GB120005, Servicebio, Wuhan, China) and anti-VE-cadherin

(1:100, ab205336, Abcam, Cambridge, UK) at 4℃ overnight. After washes, sections were incubatedwith secondary antibodies conjugated with CY5-conjugated anti-mouse (1:100, GB27301, Servicebio, Wuhan, China), CY5-conjugated anti-rabbit (1:100, GB27303, Servicebio, Wuhan, China), CY3-conjugated anti-mouse (1:100, GB21301, Servicebio, Wuhan, China) and CY3-conjugated anti-rabbit (1:100, GB21303, Servicebio, Wuhan, China) secondary antibody at 37°℃ for 1 h in the dark, Finally, nuclei were counterstained with DAPl. The fluorescent images were captured by Pannoramic MIDI II digital scanner (3D HISTECH, Budapest, Hungary), adjusted by CaseViewer (3D HISTECH, Budapest, Hungary), and analyzed using lmage J software. The investigator performing immunofluorescence analysis was blinded to group allocation. For quantification, five high-power fields were analyzed in lung tissue sections taken from each mouse.

Connectivity Map (CMap) L1000 query

At present, the CMap web portal (https://clue.io) contains 6,100 gene expression profiles from 7,056 microarray datasets, covering a total of 1,309 Food and Drug Administration (FDA)-approved small molecule drugs. This resource can be used to predict the mechanisms of action of novel drugs and perform in silico screening for drug repurposing. The tool calculates enrichment scores, which represent the degree to which a drug "flips" the disease signature, ranging from −1 to 1. A positive connectivity score indicates that a specific drug induces the expression pattern associated with the disease, while a negative connectivity score suggests that the drug is more likely to

reverse the disease-related gene expression patterns. In this study, we analyzed five datasets: 1. Gene lists positively and negatively correlated with age, derived from transcriptome sequencing results of 18 human lung tissues (aged 25-77 years). 2. Gene lists positively and negatively correlated with age, derived from transcriptome sequencing results of mouse lung tissues (GSE209891). 3. Upregulated and downregulated DEGs in MLVECs treated with Ad-KLK8 and Ad-vector. 4. Upregulated and downregulated DEGs in senescent endothelial cells from progeria mouse lungs (GSE228491). 5. Upregulated and downregulated DEGs in senescent endothelial cells from naturally aged mouse lungs (GSE197366). To identify compounds that could reverse the genetic alterations, we used the top 150 gene profiles with opposite alteration directions as signatures (Table S6) to query the CMap platform. Special attention was given to drug candidates with negative connectivity scores, indicating their potential to revert aging or senescence-induced gene expression patterns.

Cell treatments

To investigate that aging enhances the sensitivity of pulmonary endothelial cells to low-magnitude mechanical stretch, MLVECs were pretreated with vehicle or H_2O_2 at the indicated concentrations (250 μM) (323381, Sigma-Aldrich, MO, USA). Seventy-two hours later, cells were either harvested for analysis or subjected to 7% cyclic stretch for an additional 4 hours.

To investigate that KLK8 siRNA-mediated KLK8 knockdown significantly

attenuated H_2O_2-induced endothelial cell injury when exposed to 4 hours of 7% cyclic stretch, MLVECs were transfected with control siRNA or KLK8 siRNA. Twenty-four hours later, culture medium were changed with normal medium. Subsequently, MLVECs were pretreated with vehicle or H_2O_2 at the indicated concentrations (250 μM). Seventy-two hours later, cells were either harvested for analysis or subjected to 7% cyclic stretch for an additional 4 hours.

To investigate the effect of the PARP1/2 inhibitor olaparib on Ad-KLK8-induced endothelial cell senescence and the increased susceptibility of KLK8-overexpressing endothelial cells to 7% cyclic stretch, MLVECs were pretreated with vehicle or olaparib at the indicated concentrations (1, 5, 10 μM) for 24 hours. Subsequently, cells were infected with Ad-KLK8 at an MOI of 10. Seventy-two hours later, cells were either harvested for analysis or subjected to 7% cyclic stretch for an additional 4 hours.

Statistical analysis

Data are expressed as mean ± SEM. Statistical analyses were done with Graphpad Prism 10.0 (GraphPad Software Inc, CA, USA). Normal distribution was assessed using the Shapiro-Wilk test. Comparisons between two groups were determined by unpaired, two-tailed Student's t-test. Correlation analysis of genes with age in the RNA-seq datasets from lung tissues of 18 human at different ages (25-77 years old) and a published transcriptomic dataset of lung tissues from mice at different ages (3, 6, 12, 24 months, GSE209891), and correlation analysis of the lung KLK8 mRNA and protein expression with age of 41 patients by qRT-PCR and ELISA were performed using

Pearson correlation, since these data were continuous variables, showing linear relationship and normal distribution. Differences among multiple groups were assessed by One-way or two-way analysis of variance (ANOVA) with Bonferroni's post hoc tests. *p* value threshold of <0.05 was considered statistically significant.

References

Felder, M., B. Trueeb, A. O. Stucki, et al. 2019. "Impaired Wound Healing of Alveolar Lung Epithelial Cells in a Breathing Lung-On-A-Chip." *Frontiers in Bioengineering and Biotechnology* 7: 3. https://doi.org/10.3389/fbioe.2019.00003.

Gimbel, A. T., S. Koziarek, K. Theodorou, et al. 2022. "Aging-regulated TUG1 is dispensable for endothelial cell function." *PloS One* 17, no. 9: e0265160. https://doi.org/10.1371/journal.pone.0265160.

Hua, Q., T. Li, Y. Liu, X. Shen, X. Zhu, and P. Xu. 2021. "Upregulation of KLK8 Predicts Poor Prognosis in Pancreatic Cancer." *Frontiers in Oncology* 11: 624837. https://doi.org/10.3389/fonc.2021.624837.

Kawaguchi, K., A. Asai, R. Mikawa, N. Ogiso, and M. Sugimoto. 2023. "Age-related changes in lung function in National Center for Geriatrics and Gerontology Aging Farm C57BL/6N mice." *Experimental Animals* 72, no. 2: 173–182. https://doi.org/10.1538/expanim.22-0109.

Koh, M. W., R. F. Baldi, S. Soni, et al. 2021. "Secreted Extracellular Cyclophilin A Is a Novel Mediator of Ventilator-induced Lung Injury." *American Jjournal of Respiratory and Critical Care Medicine* 204, no. 4: 421–430. https://doi.org/10.1164/rccm.202009-3545OC.

Ortega, F., T. Hill, A. Van Deynze, A. Garcia-Llanos, and S. Walker. 2024. "Identification of QTLs involved in destemming and fruit quality for mechanical harvesting of New Mexico pod-type green chile." *Frontiers in plant science*15: 1357986. https://doi.org/10.3389/fpls.2024.1357986.

Ramadhiani, R., K. Ikeda, K. Miyagawa, et al. 2023. "Endothelial cell senescence exacerbates pulmonary hypertension by inducing juxtacrine Notch signaling in smooth muscle cells." *iScience* 26, no. 5: 106662. https://doi.org/10.1016/j.isci.2023.106662.

Wang, X. L., Y. T. Xu, S. L. Zhang, X. Y. Zhu, H. X. Zhang, and Y. J. Liu. 2024. "Hydrogen sulfide inhibits alveolar type II cell senescence and limits pulmonary fibrosis via promoting MDM2-mediated p53 degradation." *Acta Physiologica* 240, no. 1: e14059. https://doi.org/10.1111/apha.14059.

Wang, Y., Y. J. Liu, D. F. Xu, et al. 2021. "DRD1 downregulation contributes to mechanical stretch-induced lung endothelial barrier dysfunction." *Theranostics* 11, no.

6: 2505–2521. https://doi.org/10.7150/thno.46192.

Xu, C. F., Y. J. Liu, Y. Wang, et al. 2019. "Downregulation of R-Spondin1 Contributes to Mechanical Stretch-Induced Lung Injury." *Critical Care Medicine* 47, no. 7: e587–e596. https://doi.org/10.1097/CCM.0000000000003767.

Xu, D. H., J. K. Du, S. Y. Liu, et al. 2023. "Upregulation of KLK8 contributes to CUMS-induced hippocampal neuronal apoptosis by cleaving NCAM1." *Cell Death & Disease* 14, no. 4: 278. https://doi.org/10.1038/s41419-023-05800-5.

Zhan, R., X. Meng, D. Tian, et al. 2023. "ARDS$^+$ rescues aging-induced blood-brain barrier damage via the CX43-PARP1 axis." *Neuron* 111, no. 22: 3634–3649.e7. https://doi.org/10.1016/j.neuron.2023.08.010.

Zhang, H., D. Liu, Q. F. Xu, et al. 2024. "Endothelial RSPO3 mediates pulmonary endothelial regeneration by LGR4-dependent activation of β-catenin and ILK signaling pathways after inflammatory vascular injury." *International Journal of Biological Macromolecules* 269, no. Pt 2: 131805. https://doi.org/10.1016/j.ijbiomac.2024.131805.

Zhao, Y., H. Ji, F. Han, et al. 2025. "Inhibition of KLK8 promotes pulmonary endothelial repair by restoring the VE-cadherin/Akt/FOXM1 pathway." *Journal of Pharmaceutical Analysis* 15, no, 4: 101153. https://doi.org/10.1016/j.jpha.2024.101153.

Supplementary figures and figure legends

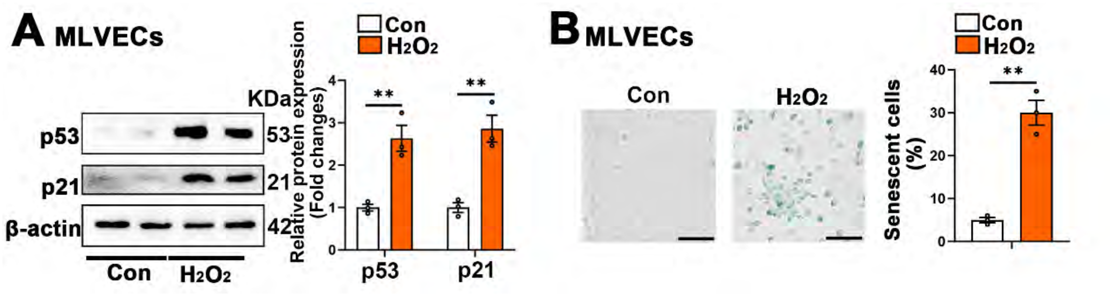

Supplementary Fig. 1 H_2O_2 induces endothelial senescence. (Related to Figure 1). Primary cultured mice MLVECs were treated with H_2O_2 (250 μM) for 72 hours. **A**, Western blot showing p53 and p21 protein levels in MLVECs. Corresponding histograms were shown on the right of representative protein bands. **B**, Representative SA-β-gal staining of MLVECs. Scale bar, 100 μm. Quantification of SA-β-gal positive MLVECs was shown on the right. Data were presented as means ± SEM (n = 3). ** $p < 0.01$.

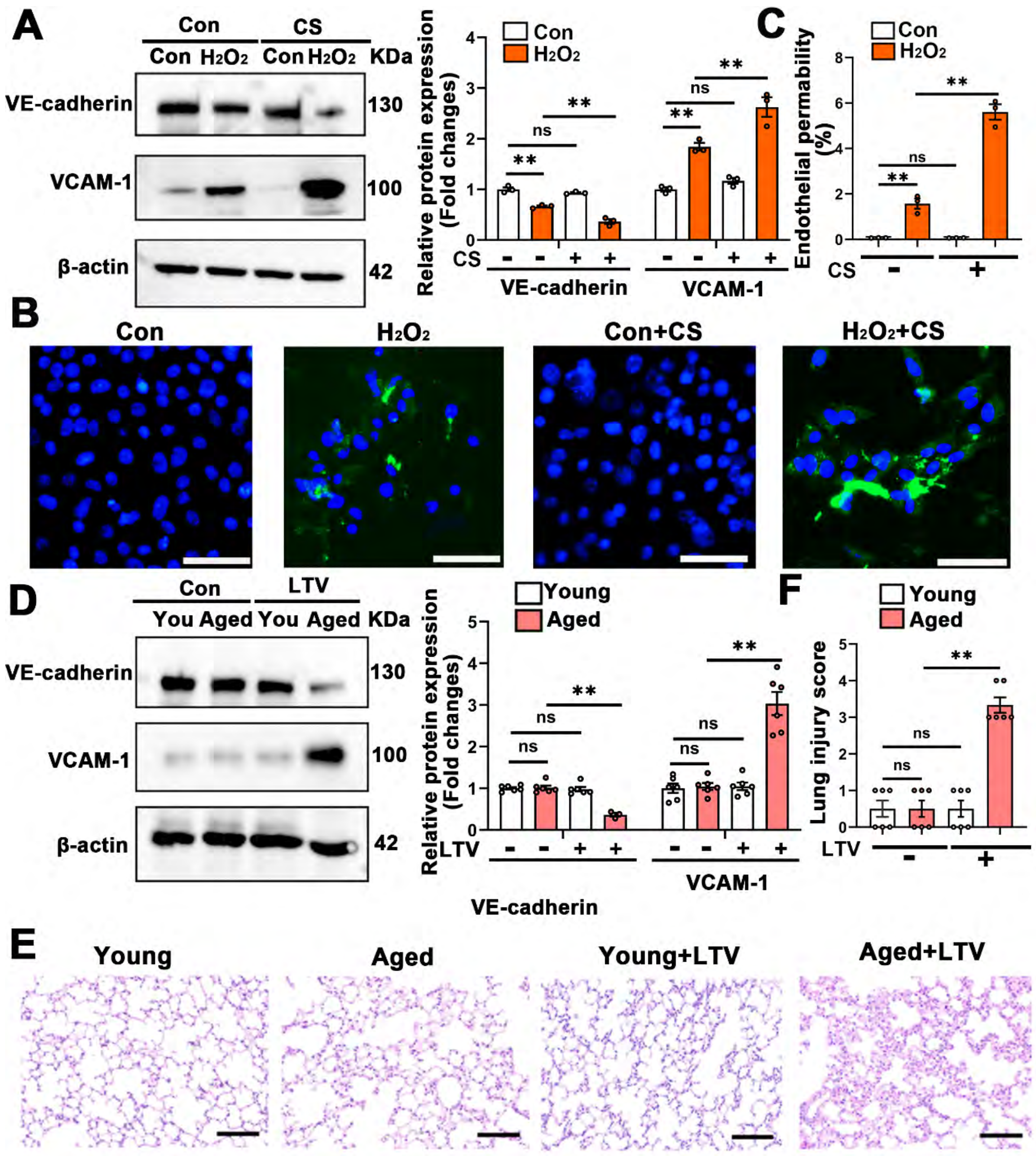

Supplementary Fig. 2 Aging enhances the sensitivity of pulmonary endothelial cells to low-magnitude mechanical stretch. A-C, Primary cultured mice MLVECs were treated with H_2O_2 (250 μM). Seventy-two hours later, MLVECs were subjected to physiological, low-magnitude (7%) cyclic stretch (CS) for 4 hours (n = 3). **A**, Western blot showing VE-cadherin and VCAM-1 protein levels in H_2O_2-induced MLVECs subjected to 4-hour 7% cyclic stretch. Corresponding histograms were shown on the right of representative protein bands. **B**, Primary cultured MLVECs were seeded on

Collagen I coated Bioflex® culture plates and the FITC fluorescence was detected as described in Materials and Methods. FITC fluorescence signal was visualized by fluorescence microscopy. **C**, Quantification of FITC fluorescence signal by using Image J. Original magnification, × 200. Scale bar = 200 μm. **D-F**, Young (2 months) and aged (18 months) mice were subjected to low-tidal-volume (LTV) mechanical ventilation (8 mL/kg) for 4 hours (n = 6). **D**, Western blot showing VCAM-1 and VE-cadherin protein levels in lung tissues. **E**, The left lower lung was used for histological evaluation by H&E staining. Original magnification, × 200. Scale bar= 100 μm. **F**, The severity of lung injury was scored by two pathologists blinded to group allocation. Data were presented as means ± SEM. ** $p < 0.01$. ns: not significant. “You” represents “Young”.

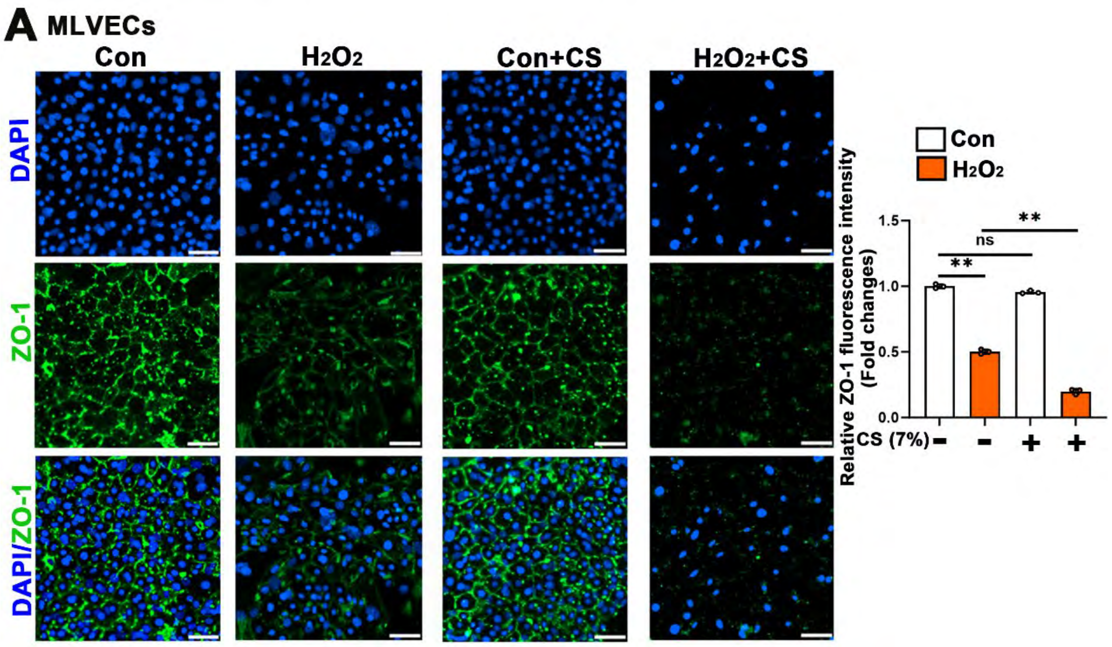

Supplementary Fig. 3 Aging enhances the sensitivity of pulmonary endothelial cells to low-magnitude mechanical stretch. Primary cultured mice MLVECs were treated with H_2O_2 (250 μM). Seventy-two hours later, H_2O_2-induced senescent MLVECs were subjected to physiological, low-magnitude (7%) cyclic stretch (CS) for 4 hours**. A**, Immunofluorescence staining showing ZO-1 distribution on MLVECs cell membrane. MLVECs were immunostained with anti-ZO-1 (green). Nuclei were counterstained with DAPI (blue). Quantification of ZO-1 fluorescence signal by using Image J was presented in the right panels. Scale bar = 50 μm. Data were presented as means ± SEM (n = 3). ** $p < 0.01$. ns: not significant.

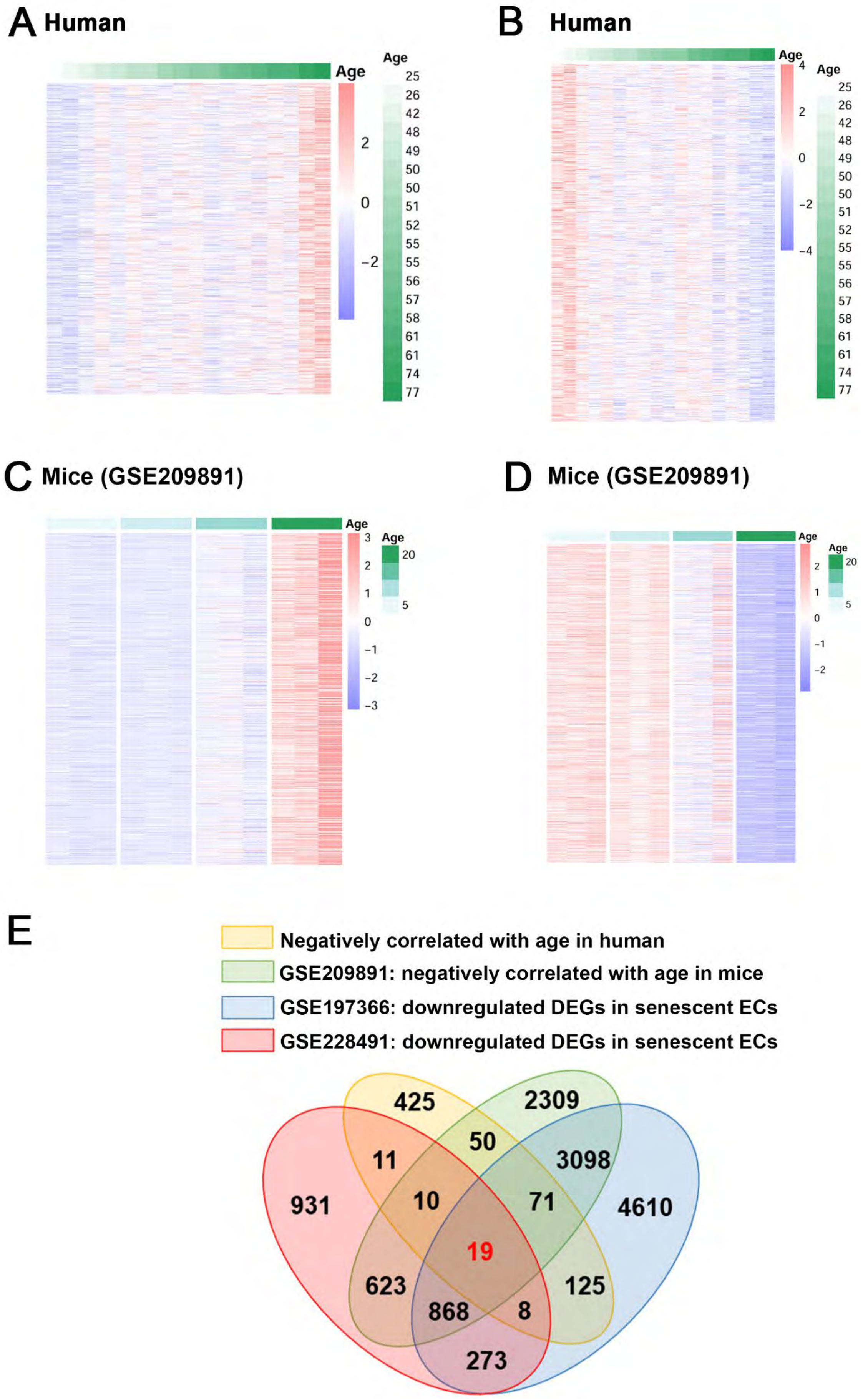

A Human
Age
B Human
Age
C Mice (GSE209891)
Age
D Mice (GSE209891)
Age
E
Negatively correlated with age in human
GSE209891: negatively correlated with age in mice
GSE197366: downregulated DEGs in senescent ECs
GSE228491: downregulated DEGs in senescent ECs
425
2309
50
11
3098
931
10
71
4610
19
623
125
868
8
273

Supplementary Fig. 4 RNA sequencing (RNA-seq) data and single-cell RNA-seq data of human and mouse lungs and senescent endothelial cells (ECs) are analyzed to obtain age-correlated genes and differentially expressed genes (DEGs). A, Heatmap of genes positively correlated with age in 18 humans within a wide age range (25-77 years old) (Correlation≥0.5, p<0.05). **B**, Heatmap of genes negatively correlated with age in 18 humans within a wide age range (25-77 years old) (Correlation≤-0.5, p<0.05). **C**, Heatmap of genes positively correlated to age in mice at different ages (3, 6, 12, 24 months, GSE209891) (Correlation≥0.5, p<0.05). **D**, Heatmap of genes negatively correlated to age in mice at different ages (3, 6, 12, 24 months, GSE209891) (Correlation≤-0.5, p<0.05). **E**, Venn Diagram of genes negatively correlated with age in human and mice, and downregulated DEGs in senescent ECs. Correlation analyses of genes with age were performed using Pearson correlation test.

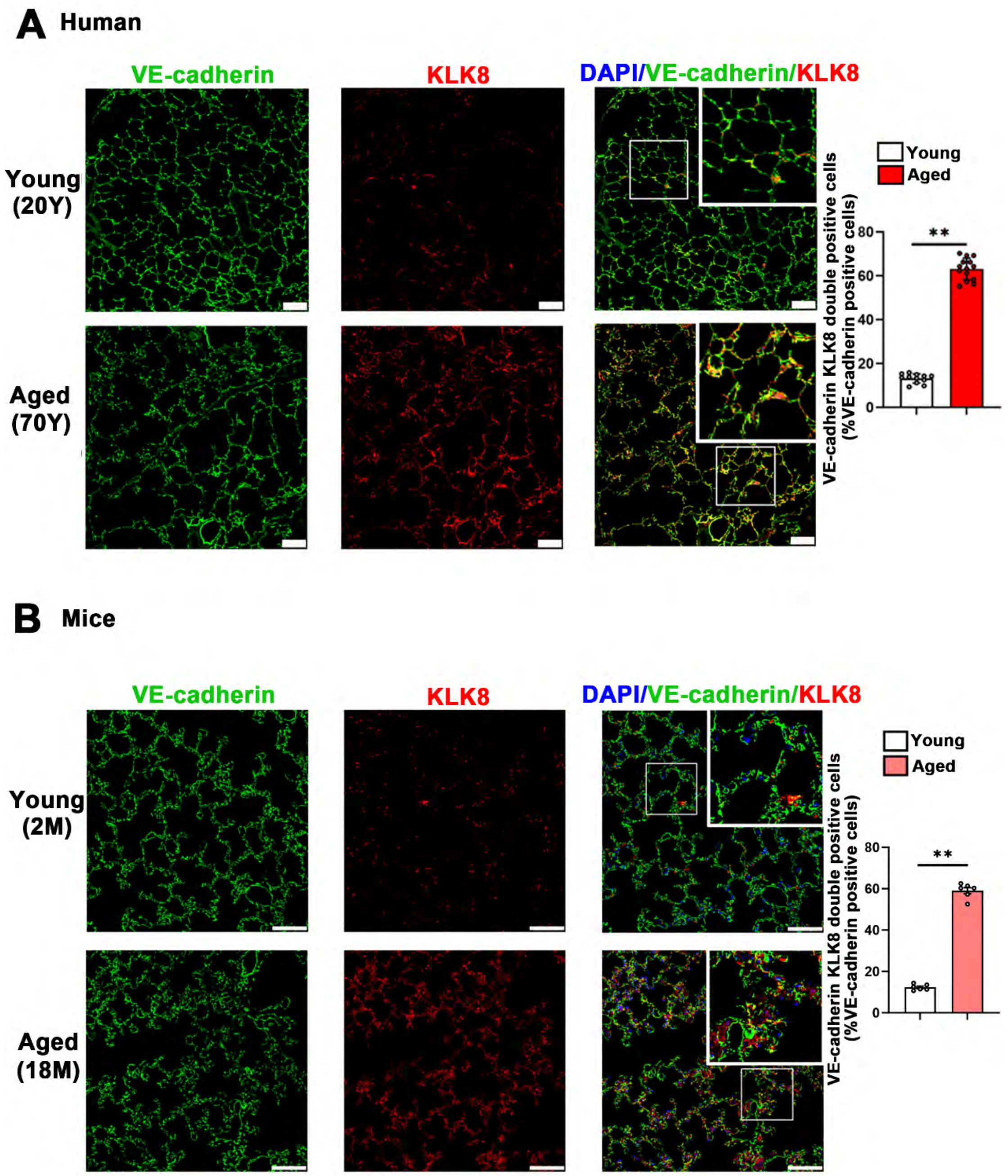

Supplementary Fig. 5 KLK8 exhibits age-dependent upregulation. A, Immunofluorescent staining showed KLK8 (red) expression in the lung sections of young (19-40 years old) and aged patients (67-78 years old). Endothelial cells were immunostained with anti-VE-cadherin (green). Nuclei were counterstained with DAPI (blue). Scale bar = 200 µm. The percentage of VE-cadherin^{+}KLK8^{+} cell numbers in total VE-cadherin^{+} cells was presented in the right panels (n = 12 for young human and

n = 14 for aged human). **B**, Immunofluorescent staining showed KLK8 (red) expression in the lung sections of young and aged mice lungs. Endothelial cells were immunostained with anti-VE-cadherin (green). Nuclei were counterstained with DAPI (blue). Scale bar = 50 μm. The percentage of VE-cadherin$^+$KLK8$^+$ cell numbers in total VE-cadherin$^+$ cells were presented in the right panels (n = 6). Data were presented as means ± SEM. ** $p < 0.01$.

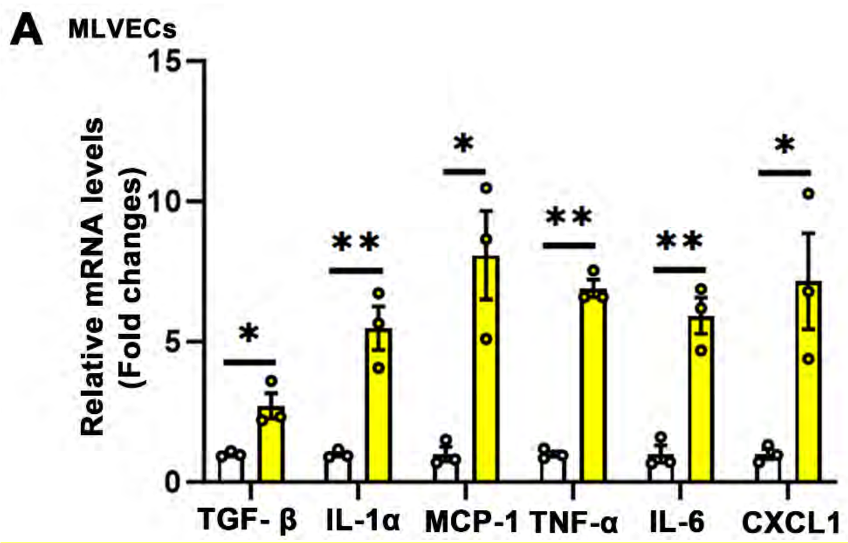

Supplementary Fig. 6 KLK8 overexpression significantly increased the transcript levels of profibrotic and proinflammatory senescence-associated secretory phenotype (SASP) factors. A, Primary cultured MLVECs were transfected with KLK8 adenovirus (Ad-KLK8) or control adenovirus (Ad-vector) at MOI 10 for 72 hours. The transcript levels of profibrotic and proinflammatory SASP factors, such as transforming growth factor-β (TGF-β), interleukin-1α (IL-1α), monocyte chemoattractant protein 1 (MCP1), tumor necrosis factor-α (TNF-α), interleukin-6 (IL-6), and C-X-C motif ligand 1 (CXCL1) in MLVECs were detected by qRT-PCR. Data were presented as means ± SEM (n = 3). * $p < 0.05$, ** $p < 0.01$.

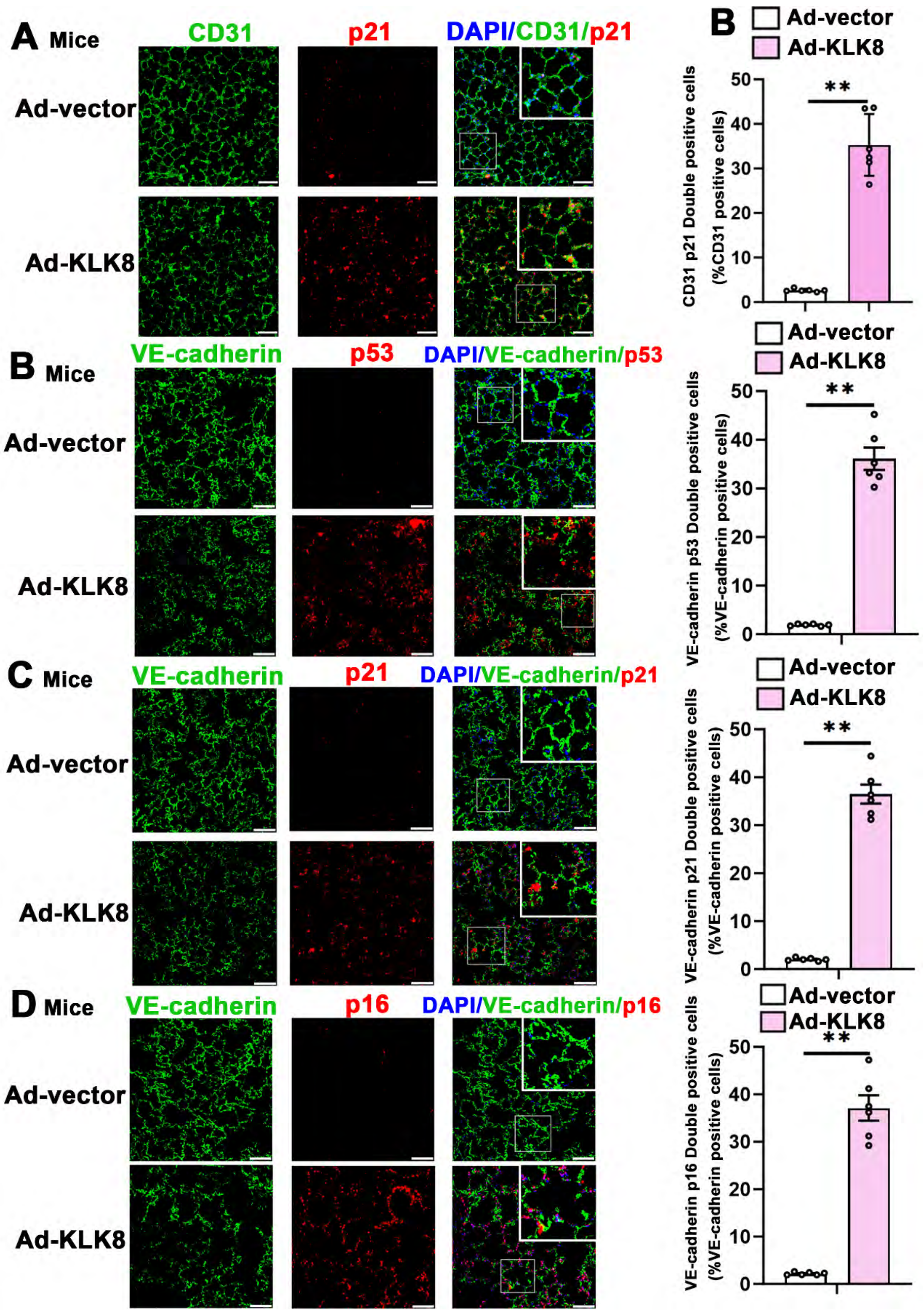

Supplementary Fig. 7 KLK8 overexpression alone is sufficient to induce endothelial cell senescence. Young mice (2 months) were instilled intratracheally with Ad-KLK8 or Ad-vector. Seventy hours later, lung tissues were harvested for

immunofluorescence analysis. **A**, Representative senescent markers staining showing p21 (red) expression in lung sections. Endothelial cells were immunostained with anti-CD31 (green). Nuclei were counterstained with DAPI (blue). Scale bar, 50 μm. The percentage of $CD31^{+}p21^{+}$ cell numbers in total $CD31^{+}$ cells were on the right. **B-D**, Representative senescent markers staining showing p53, p21 and p16 (red) expression in lung sections. Endothelial cells were immunostained with anti-VE-cadherin (green). Nuclei were counterstained with DAPI (blue). Scale bar, 50 μm. The percentage of VE-$cadherin^{+}p53^{+}$, VE-$cadherin^{+}p21^{+}$ and VE-$cadherin^{+}p16^{+}$ cell numbers in total VE-$cadherin^{+}$ cells were presented in the right panels. Data were presented as means ± SEM (n = 6). ** $p < 0.01$.

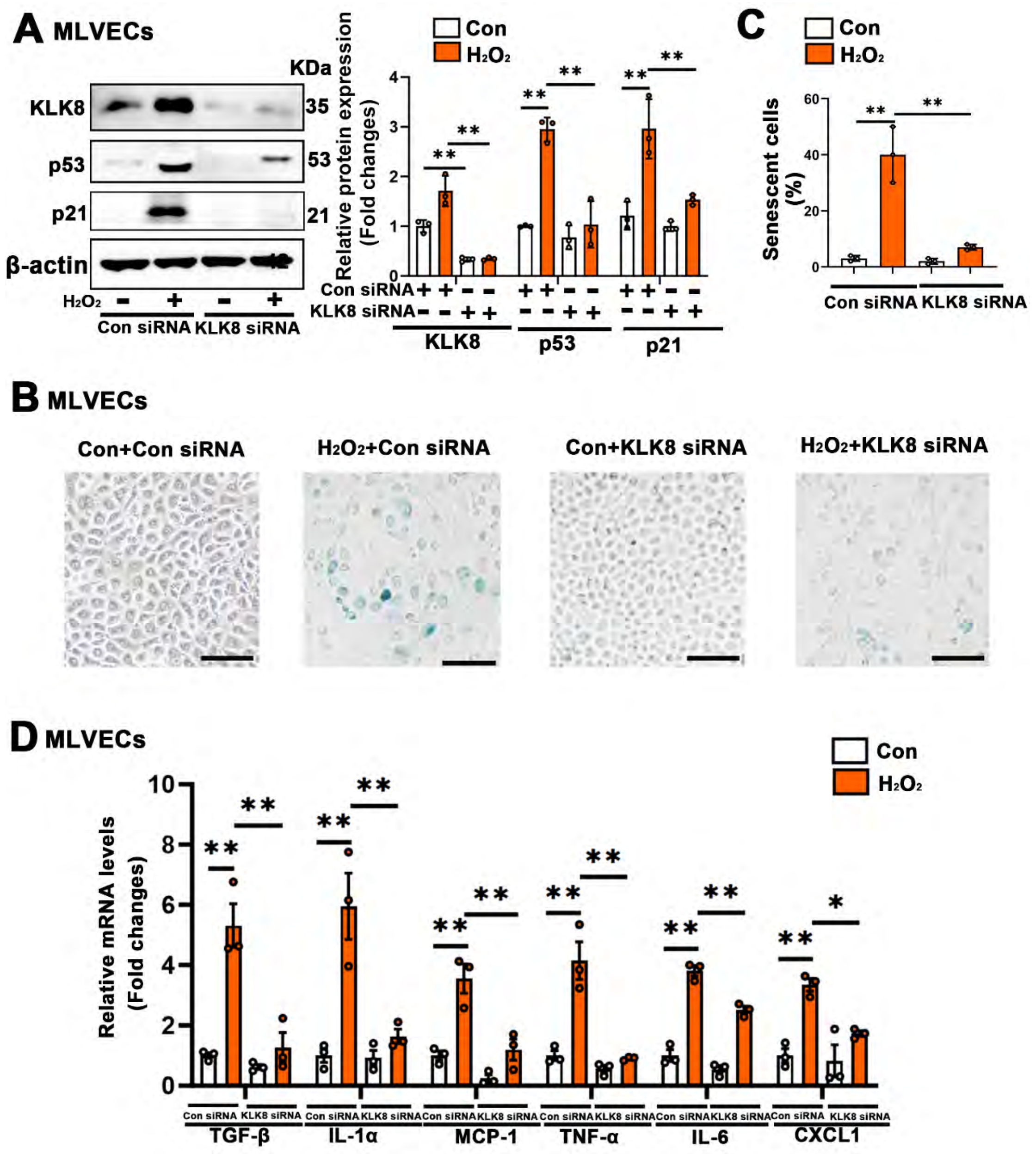

Supplementary Fig. 8 KLK8 knockdown relieves pulmonary endothelial cells senescence. Primary cultured MLVECs were transfected with control or KLK8 siRNA. Twenty-four hours later, culture medium were changed with normal medium. Cells were treated H_2O_2 (250 uM) or saline for 72 hours. **A**, Western blot showing KLK8, p53 and p21 protein levels. Corresponding histograms were shown on the right of representative protein bands. **B**, Representative SA-β-gal staining. Scale bar, 100 μm.

C, Quantification of SA-β-gal positive MLVECs. **D**, The transcript levels of profibrotic and proinflammatory SASP factors, such as TGF-β, IL-1α, MCP1, TNF-α, IL-6, and CXCL1 in MLVECs were detected by qRT-PCR. Data were presented as means ± SEM (n = 3). ** $p < 0.01$.

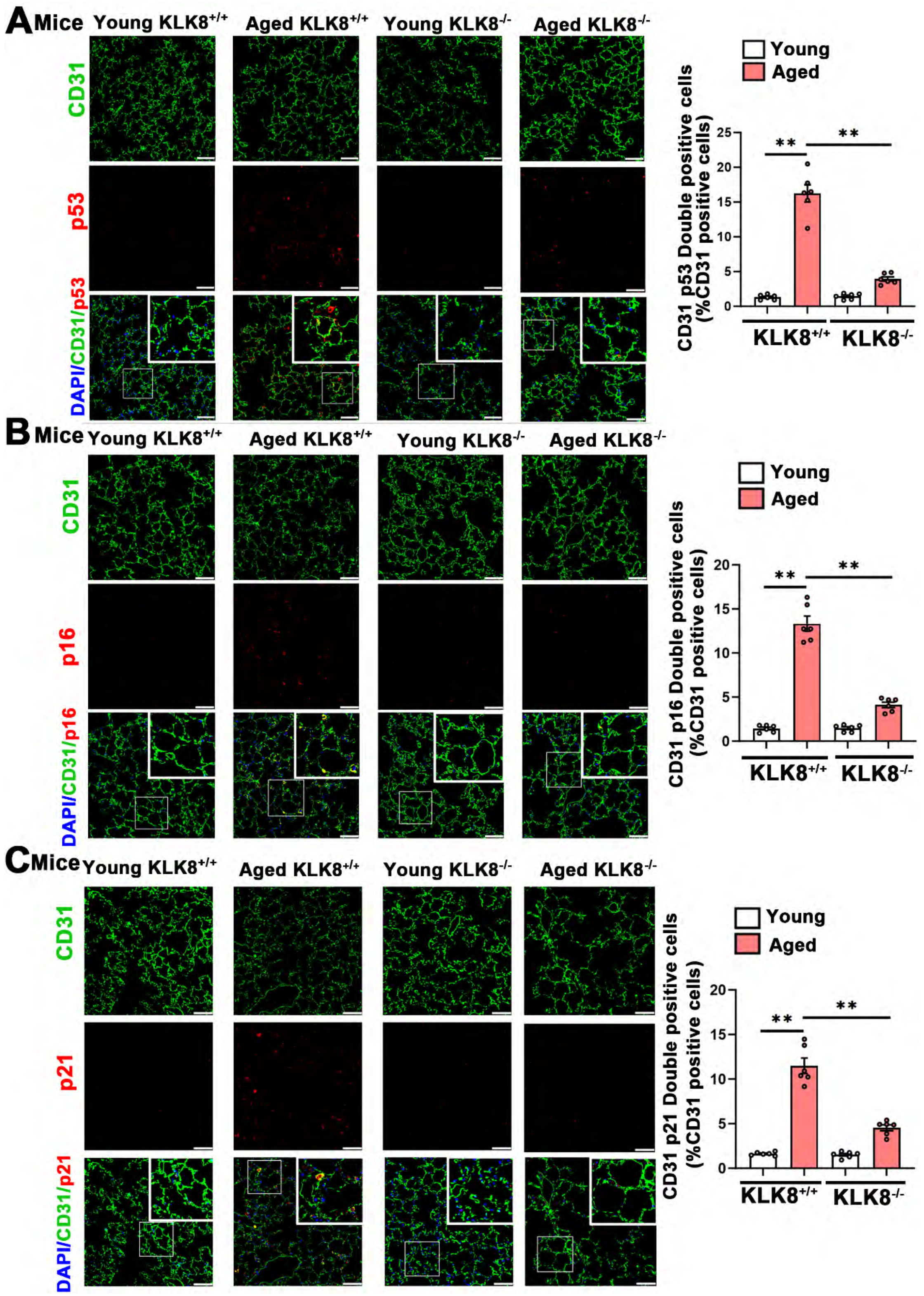

Supplementary Fig. 9 KLK8 deficiency relieves pulmonary endothelial cells senescence. A-C, Representative senescent markers staining showing p53, p21 and p16 (red) expression in lung sections of young (2 months) and aged (18 months) KLK8$^{+/+}$

and KLK8$^{-/-}$ mice. Endothelial cells were immunostained with anti-CD31 (green). Nuclei were counterstained with DAPI (blue). Scale bar, 50 μm. The percentage of CD31^{+}p53^{+}, CD31^{+}p21^{+} and CD31^{+}p16^{+} cell numbers in total CD31^{+} cells were presented in the right panels. Data were presented as means ± SEM (n = 6**)**. ** $p < 0.01$.

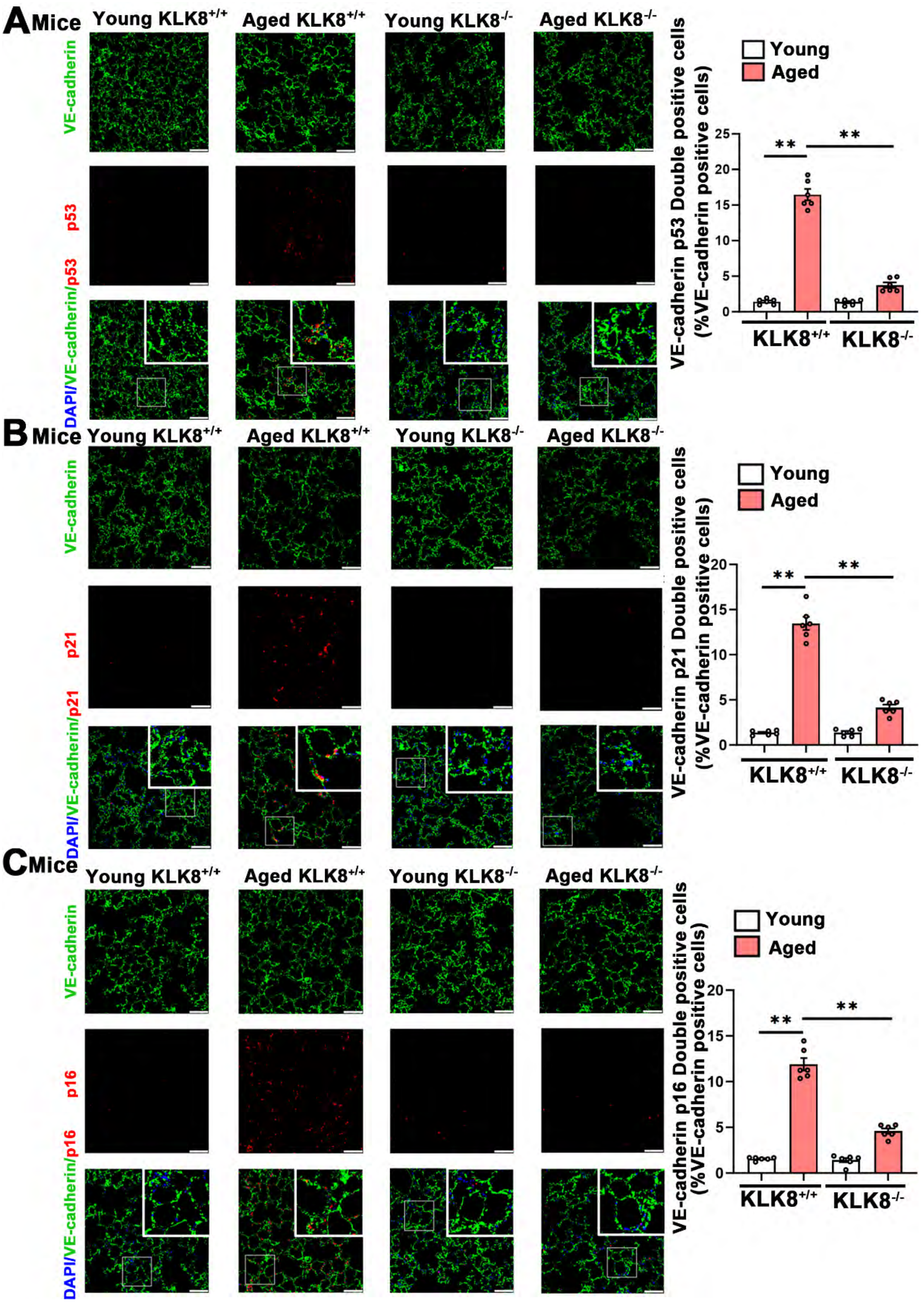

Supplementary Fig. 10 KLK8 deficiency relieves pulmonary endothelial cells senescence. A-C, Representative senescent markers staining showing p53, p21 and p16 (red) expression in lung sections of young (2 months) and aged (18 months) KLK8$^{+/+}$

and KLK8$^{-/-}$ mice. Endothelial cells were immunostained with anti-VE-cadherin (green). Nuclei were counterstained with DAPI (blue). Scale bar, 50 µm. The percentage of VE-cadherin^{+}p53^{+}, VE-cadherin^{+}p21^{+} and VE-cadherin^{+}p16^{+} cell numbers in total VE-cadherin^{+} cells were presented in the right panels. Data were presented as means ± SEM (n = 6). ** $p < 0.01$.

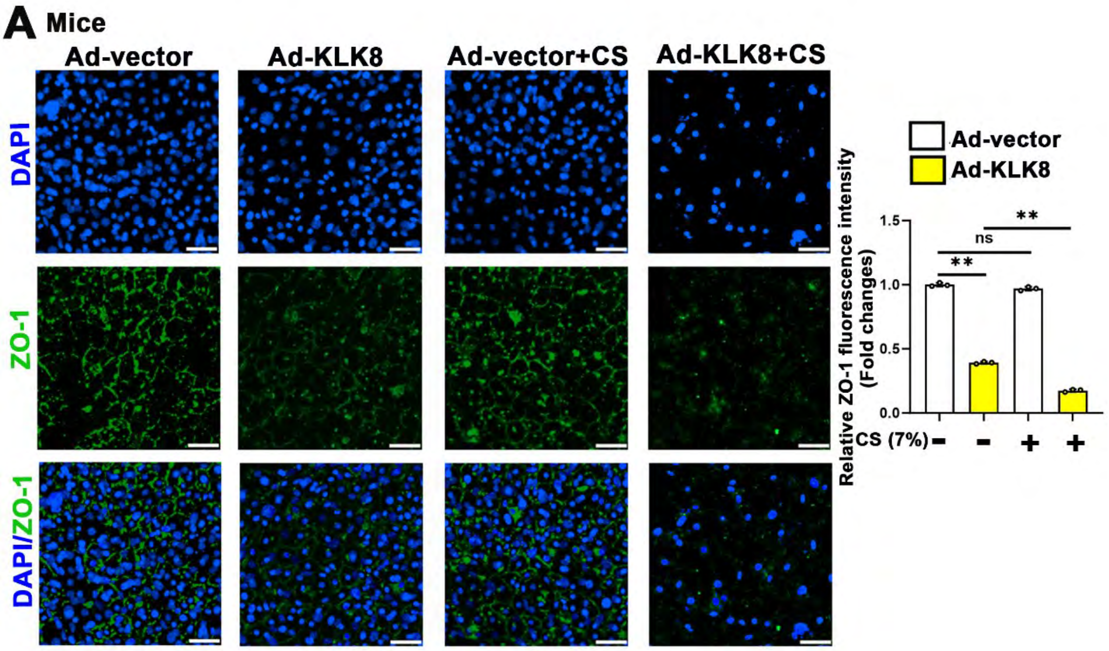

Supplementary Fig. 11 KLK8 upregulation enhances the sensitivity of pulmonary endothelial cells to low-magnitude mechanical stretch. Primary cultured MLVECs were transfected with KLK8 adenovirus (Ad-KLK8) or control adenovirus (Ad-vector) at MOI 10. Seventy-two hours later, cells were given 7% cyclic stretch (CS) for 4 hours (n = 3). **A**, Immunofluorescence staining showing ZO-1 distribution on MLVECs cell membrane. MLVECs were immunostained with anti-ZO-1 (green). Nuclei were counterstained with DAPI (blue). Quantification of ZO-1 fluorescence signal by using Image J was presented on the right panel. Scale bar = 50 μm. Data were presented as means ± SEM (n = 3). ** $p < 0.01$. ns: not significant.

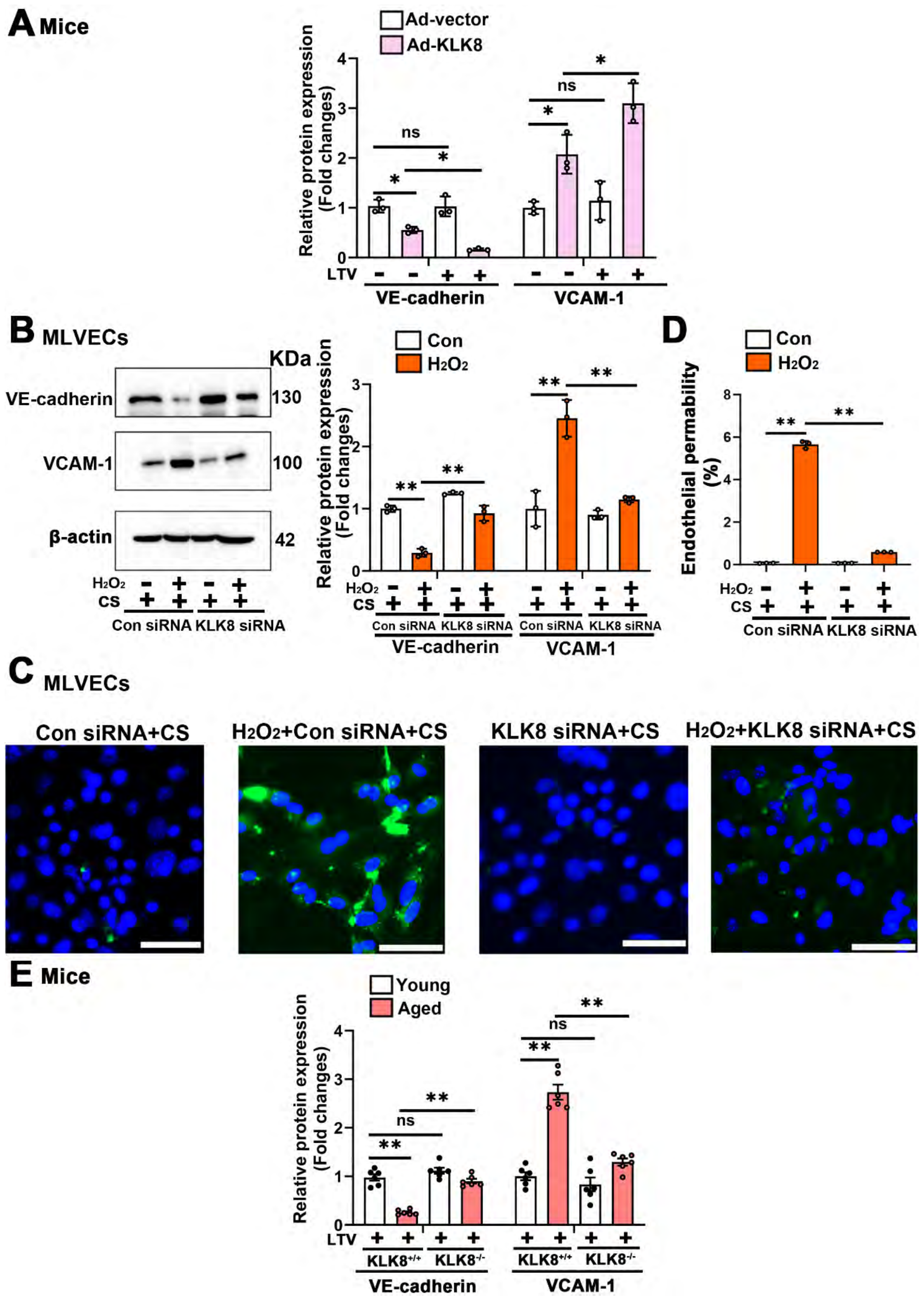

Supplementary Fig. 12 KLK8 upregulation contributes to elevated susceptibility to low-magnitude mechanical stretch in the elderly. A, Young mice (2 months) were

instilled intratracheally with Ad-KLK8 or Ad-vector. Seventy-two hours later, then subjected to LTV mechanical ventilation (8 mL/kg) for 4 hours. Relative densitometry of the VE-cadherin and VCAM-1 protein band in mice were shown in bar graphs (n = 6). **B-D,** Primary cultured MLVECs were transfected with control or KLK8 siRNA. Twenty-four hours later, culture medium were changed with normal medium. Cells were treated H_2O_2 (250 uM) or saline. Seventy-two hours later, MLVECs were subjected to physiological, low-magnitude (7%) cyclic stretch (CS) for 4 hours (n = 3). **B**, Western blot showing VE-cadherin and VCAM-1 protein levels. Corresponding histograms were shown on the right of representative protein bands. **C**, Primary cultured MLVECs were seeded on Collagen I coated Bioflex® culture plates and the FITC fluorescence was detected as described in Materials and Methods. FITC fluorescence signal was visualized by fluorescence microscopy. **D**, Quantification of FITC fluorescence signal by using Image J. Original magnification, × 200. Scale bar = 200 μm. **E**, Young (2 months) and aged (18 months) $KLK8^{+/+}$ and $KLK8^{-/-}$ mice were subjected to LTV mechanical ventilation (8 ml/kg) for 4 hours. Relative densitometry of the VE-cadherin and VCAM-1 protein band in mice were shown in bar graphs (n = 6). Data were presented as means ± SEM. * $p<0.05$, ** $p<0.01$.

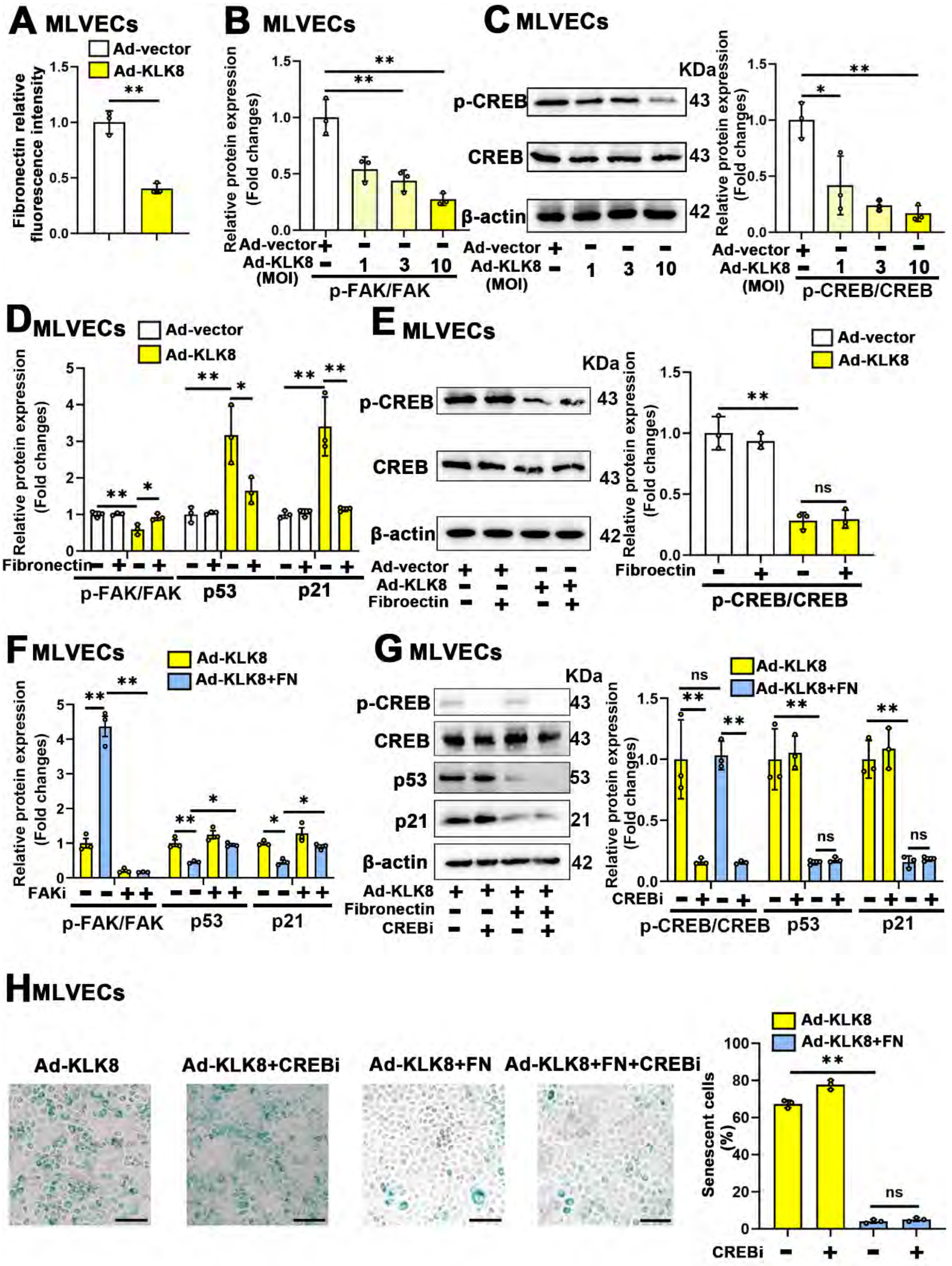

Supplementary Fig. 13 KLK8 promotes mice pulmonary endothelial senescence via inactivation of the fibronectin/FAK signaling pathway. A, Primary cultured MLVECs were transfected with KLK8 adenovirus (Ad-KLK8) at MOI 10 or control adenovirus (Ad-vector) at MOI 10. **A,** Relative fluorescence intensity of the fibronectin

(FN) in MLVECs were shown in bar graphs. **B-C,** Primary cultured MLVECs were transfected with Ad-KLK8 at MOI 1-10 or Ad-vector at MOI 10 for 72 hours. **B,** Relative densitometry of the p-FAK/FAK protein band in MLVECs were shown in bar graphs**. C,** Western blot showing p-CREB/CREB protein levels in MLVECs. Corresponding histograms were shown on the right of representative protein bands. **D-E,** MLVECs were seeded on plates coated with 5 μg/cm² fibronectin. Twenty-four hours later, MLVECs were treated with Ad-KLK8 (MOI 10) or Ad-vector (MOI 10) for 72 hours. **D**, Relative densitometry of the p-FAK/FAK, p53 and p21 protein band in MLVECs were shown in bar graphs**. E,** Western blot showing p-CREB/CREB protein levels in MLVECs. Corresponding histograms were shown on the right of representative protein bands. **F**, MLVECs were seeded on plates coated with 5 μg/cm² fibronectin. Twenty-four hours later, MLVECs were treated with Ad-KLK8 (MOI 10) in the presence or absence of FAK inhibitor (FAKi) Defactinib (5 μM) for 72 hours. Relative densitometry of the p-FAK/FAK, p53 and p21protein band in MLVECs were shown in bar graphs**. G-H,** MLVECs were seeded on plates coated with 5 μg/cm² fibronectin. Twenty-four hours later, MLVECs were treated with Ad-KLK8 (MOI 10) in the presence or absence of CREB inhibitor (CREBi) KG-501 (30 μM) for 72 hours. **G**, Western blot showing p-CREB/CREB, p53 and p21 protein levels in MLVECs. Corresponding histograms were shown on the right of representative protein bands. **H**, Representative SA-β-gal staining. Scale bar, 100 μm. Quantification of SA-β-gal positive MLVECs was shown on the right. Data were presented as means ± SEM (n = 3). * $p<0.05$, ** $p < 0.01$. ns=significant.

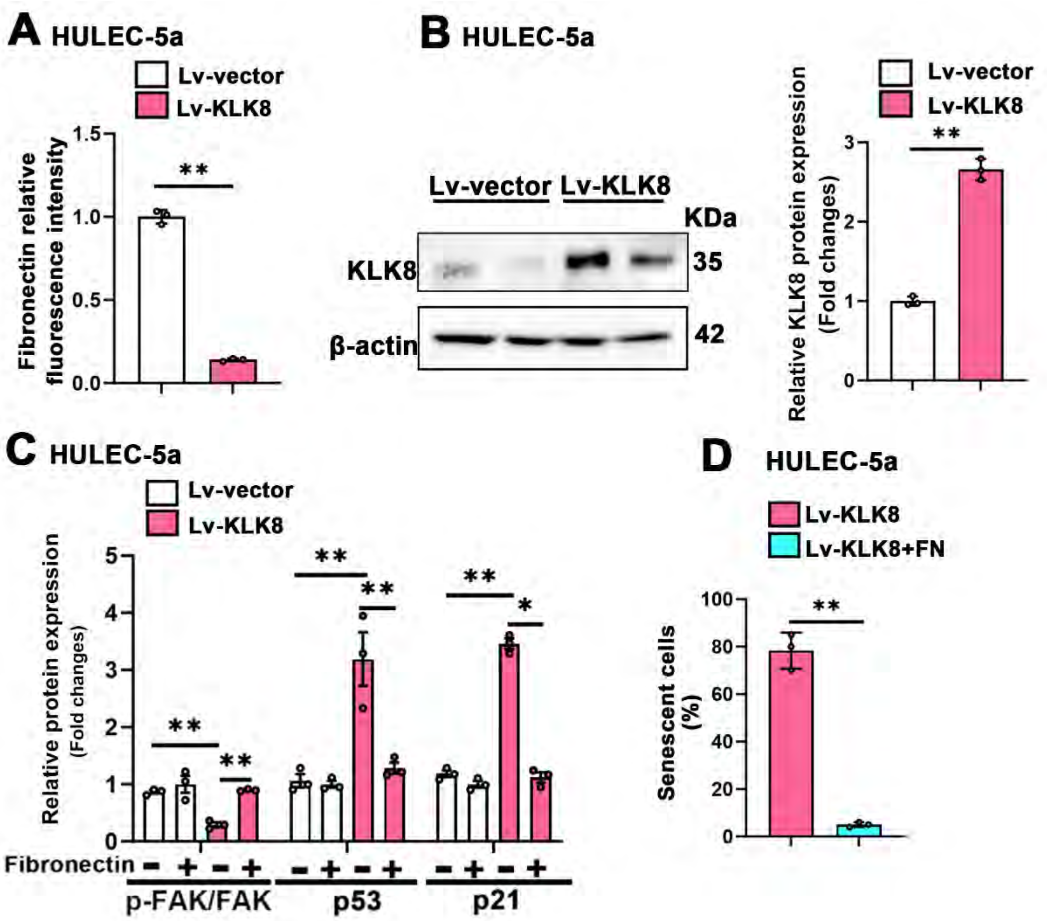

Supplementary Fig. 14 KLK8 promotes human pulmonary endothelial senescence via inactivation of the fibronectin/FAK signaling pathway. A-B, Human pulmonary microvascular endothelial cells (HULEC-5a) were treated with control or KLK8 lentivirus (Lv-vector or Lv-KLK8) at MOI 10 for 72 hours. **A**, Relative fluorescence intensity of the fibronectin (FN) in HULEC-5a. **B**, Western blot showing KLK8 protein levels in HULEC-5a. Corresponding histograms were shown on the right of representative protein bands. **C-D**, HULEC-5a were seeded on plates coated with 5 μg/cm² fibronectin. Twenty-four hours later, HULEC-5a were treated with Lv-KLK8 (MOI 10) for 72 hours. **C,** Relative densitometry of the p-FAK/FAK, p53 and p21 protein band in MLVECs were shown in bar graphs**. D,** Quantification of SA-β-gal

positive HULEC-5a. Data were presented as means ± SEM (n = 3). * $p<0.05$, ** $p<0.01$.

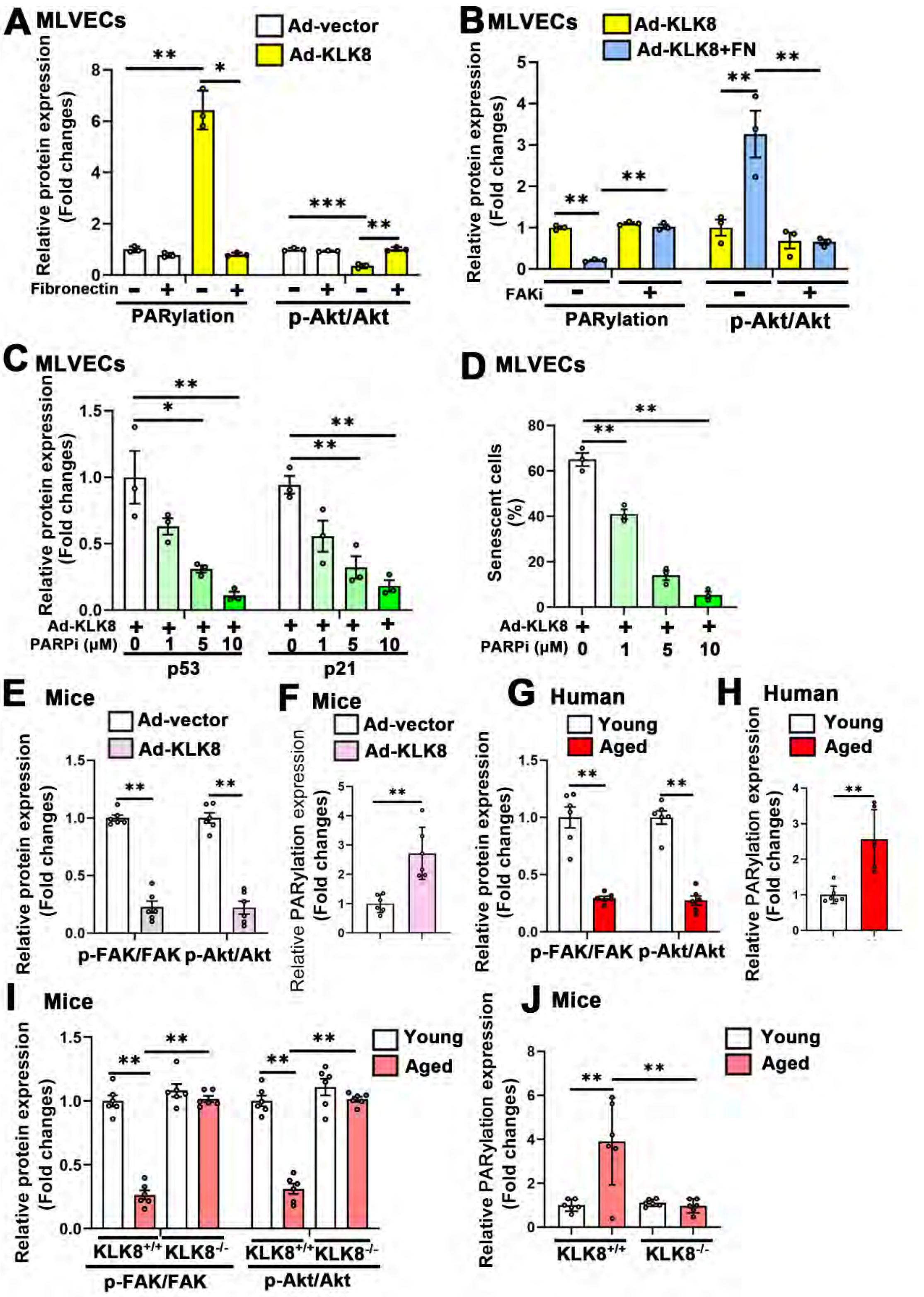

Supplementary Fig. 15 KLK8 activates PARP1/2 via inactivation of the fibronectin/FAK/Akt signaling pathway, thus promoting pulmonary endothelial senescence. A, MLVECs were seeded on plates coated with 5 μg/cm² fibronectin.

Twenty-four hours later, MLVECs were treated with Ad-KLK8 (MOI 10) or Ad-vector (MOI 10) for 72 hours. Relative densitometry of the PARylation and p-Akt/Akt protein band in MLVECs were shown in bar graphs (n = 3)**.** **B**, MLVECs were seeded on plates coated with 5 μg/cm^2 fibronectin. Twenty-four hours later, MLVECs were treated with Ad-KLK8 (MOI 10) in the presence or absence of FAK inhibitor (FAKi) Defactinib (5 μM) for 72 hours. Relative densitometry of the PARylation and p-Akt/Akt protein band in MLVECs were shown in bar graphs (n = 3)**.** **C-D**, MLVECs were treated with vehicle or olaparib (1, 5, 10 μM). Twenty-four hours later, MLVECs were treated with Ad-KLK8 at MOI of 10 for 72 hours (n = 3). **C**, Relative densitometry of the p53 and p21 protein band in MLVECs were shown in bar graphs**.** **D**, Quantification of SA-β-gal positive MLVECs. **E-F**, Young mice (2 months) were instilled intratracheally with Ad-KLK8 or Ad-vector. Seventy hours later, lung tissues were harvested for analysis (n = 6). **E**, Relative densitometry of the p-FAK/FAK and p-Akt/Akt protein band in lung tissues were shown in bar graphs**. F,** Relative densitometry of the PARylation protein band in lung tissues were shown in bar graphs**. G,** Relative densitometry of the p-FAK/FAK and p-Akt/Akt protein band in lung tissues of young (13-40 years old) and aged (66-76 years old) human (n = 6) were shown in bar graphs**. H,** Relative densitometry of the PARylation protein band in lung tissues of young and aged human (n = 6) were shown in bar graphs. **I**, Relative densitometry of the p-FAK/FAK and p-Akt/Akt protein band in in lung tissues of young (2 months) and aged (18 months) $KLK8^{+/+}$ and $KLK8^{-/-}$ mice (n = 6) were shown in bar graphs. **J**, Relative densitometry of the PARylation protein band in lung tissues of young (2 months) and aged (18 months)

KLK8$^{+/+}$ and KLK8$^{-/-}$ mice (n = 6) were shown in bar graphs. Data were presented as means ± SEM. * p<0.05, ** p<0.01.

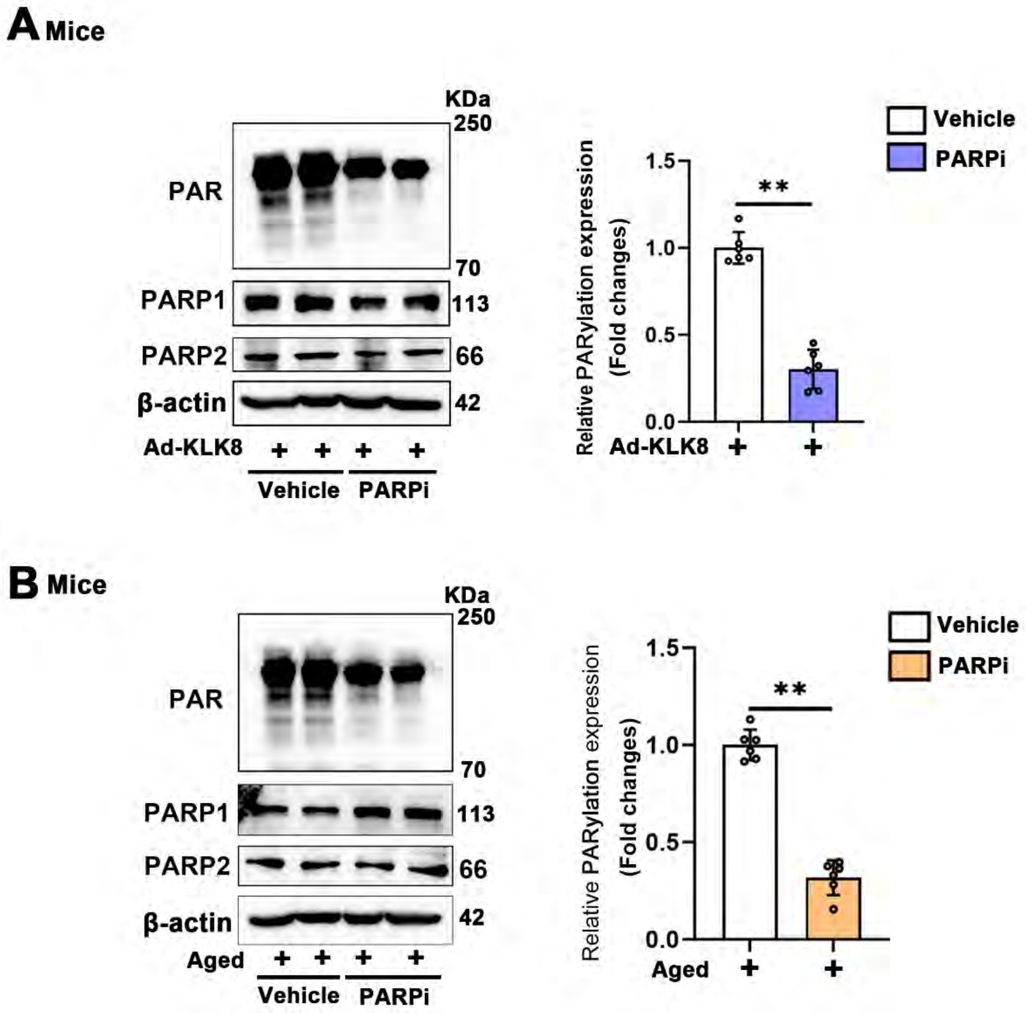

Supplementary Fig. 16 The PARP1/2 inhibitor olaparib inhibits PARP1/2 activity in both intra-pulmonary KLK8-overexpressing mice and aged mice. A, Young mice (2 months) were intraperitoneally injected with vehicle or the PARP1/2 inhibitor (PARPi) olaparib (5 mg/kg) in saline containing 0.1% DMSO every other day. Fourteen days later, they were intratracheally transfected with Ad-KLK8 (1 × 10^8 pfu). Seventy-two hours after Ad-KLK8 transfection, lung tissues were harvested for Western Blot analysis. Western blot showing PARylation protein levels of lungs from Ad-KLK8 treated mice with PARP1/2 inhibitor or saline. Corresponding histograms were shown on the right of representative protein bands. **B**, Aged mice (18 months) were

intraperitoneally injected with vehicle or olaparib (5 mg/kg) in saline containing 0.1% DMSO every other day. Thirty days later, lung tissues were harvested for Western Blot analysis. Western blot showing PARylation protein levels of lungs from Ad-KLK8 treated mice with PARP1/2 inhibitor or saline. Corresponding histograms were shown on the right of representative protein bands. Data were presented as means ± SEM (n = 6). ** $p<0.01$.

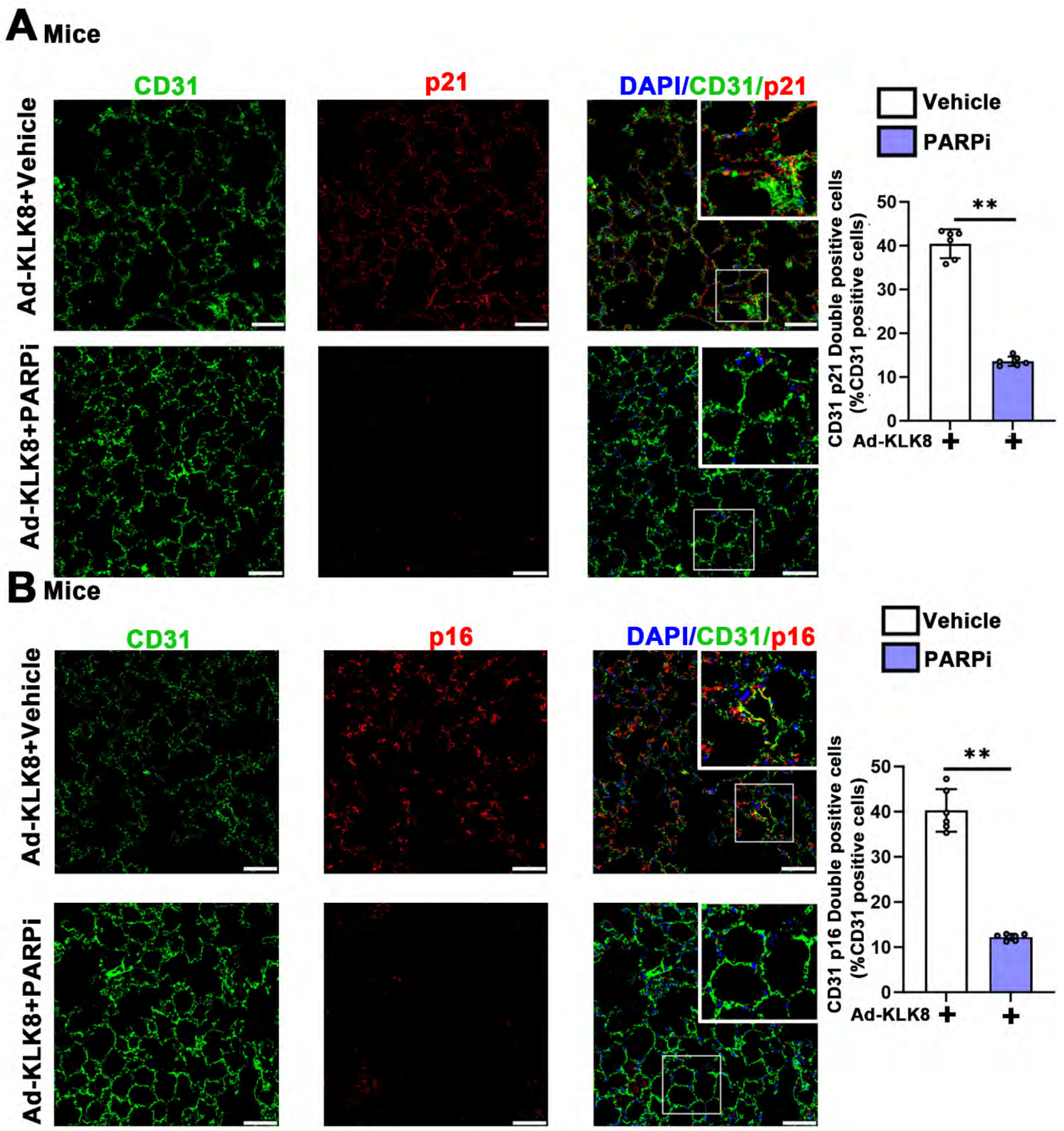

Supplementary Fig. 17 The PARP1/2 inhibitor inhibits PARP1/2 activity in intra-pulmonary KLK8-overexpressing mice. Young mice (2 months) were intraperitoneally injected with vehicle or the PARP1/2 inhibitor (PARPi) olaparib (5 mg/kg) in saline containing 0.1% DMSO every other day. Fourteen days later, they were intratracheally transfected with Ad-KLK8 (1 × 10^8 pfu). Seventy-two hours after Ad-KLK8 transfection, lung tissues were harvested for immunofluorescence. **A**, Representative senescent markers staining showing p21 (red) expression in lung

sections. Endothelial cells were immunostained with anti-CD31 (green). Nuclei were counterstained with DAPI (blue). Scale bar, 50 μm. The percentage of $CD31^{+}p21^{+}$ cell numbers in total $CD31^{+}$ cells was presented in the right panels. **B**, Representative senescent markers staining showing p16 (red) expression in lung sections. Endothelial cells were immunostained with anti-CD31 (green). Nuclei were counterstained with DAPI (blue). Scale bar, 50 μm. The percentage of $CD31^{+}p16^{+}$ cell numbers in total $CD31^{+}$ cells was presented in the right panels. Data were presented as means ± SEM (n = 6). ** $p<0.01$.

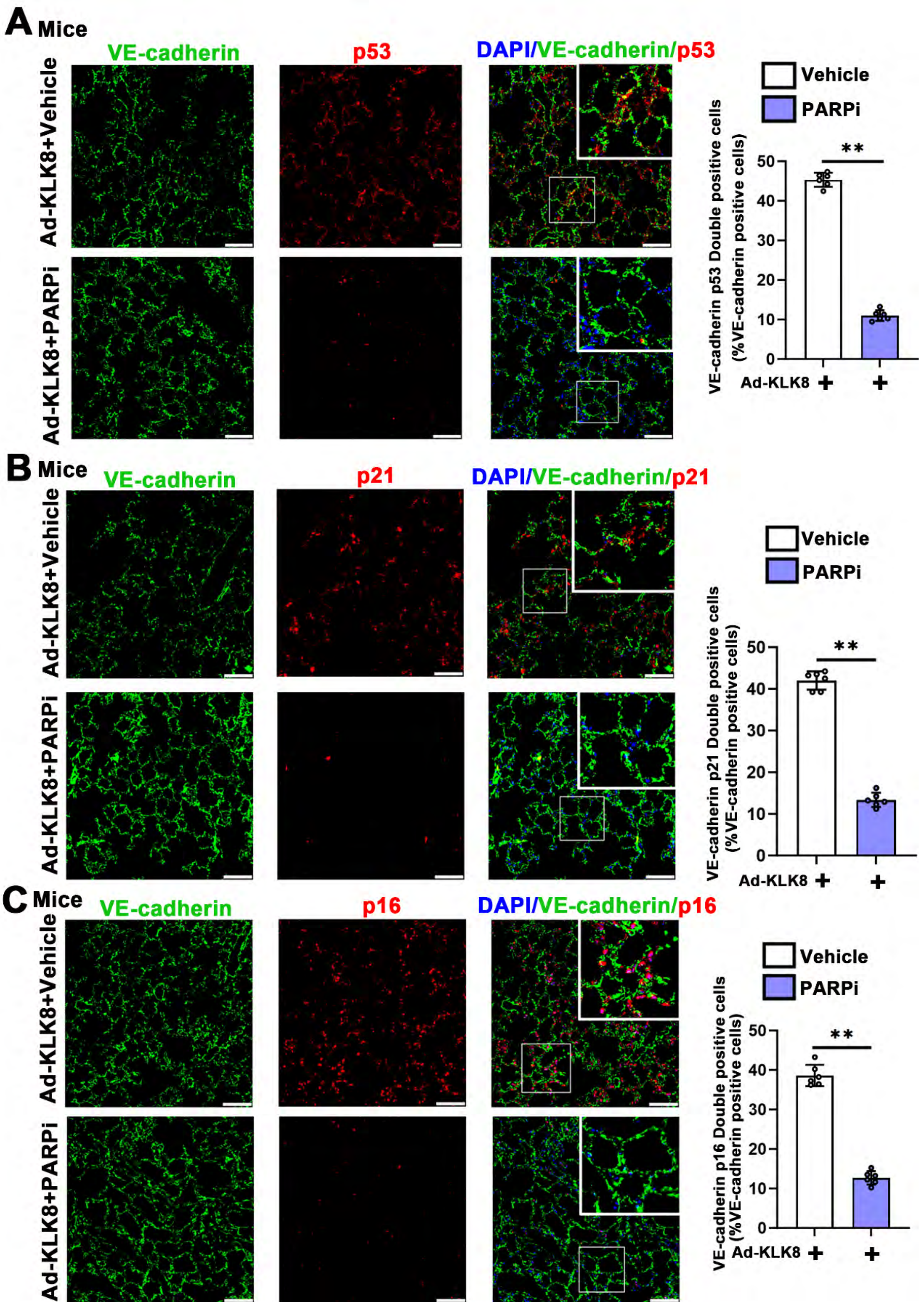

Supplementary Fig. 18 The PARP1/2 inhibitor inhibits PARP1/2 activity in intra-pulmonary KLK8-overexpressing mice. Young mice (2 months) were intraperitoneally injected with vehicle or the PARP1/2 inhibitor (PARPi) olaparib (5

mg/kg) in saline containing 0.1% DMSO every other day. Fourteen days later, they were intratracheally transfected with Ad-KLK8 (1 × 10^8 pfu). Seventy-two hours after Ad-KLK8 transfection, lung tissues were harvested for immunofluorescence. **A-C**, Representative senescent markers staining showing p53, p21 and p16 (red) expression in lung sections. Endothelial cells were immunostained with anti-VE-cadherin (green). Nuclei were counterstained with DAPI (blue). Scale bar, 50 μm. The percentage of VE-cadherin$^+$p53$^+$, VE-cadherin$^+$p21$^+$ and VE-cadherin$^+$p16$^+$ cell numbers in total VE-cadherin$^+$ cells were presented in the right panels. Data were presented as means ± SEM (n = 6). ** *p*<0.01.

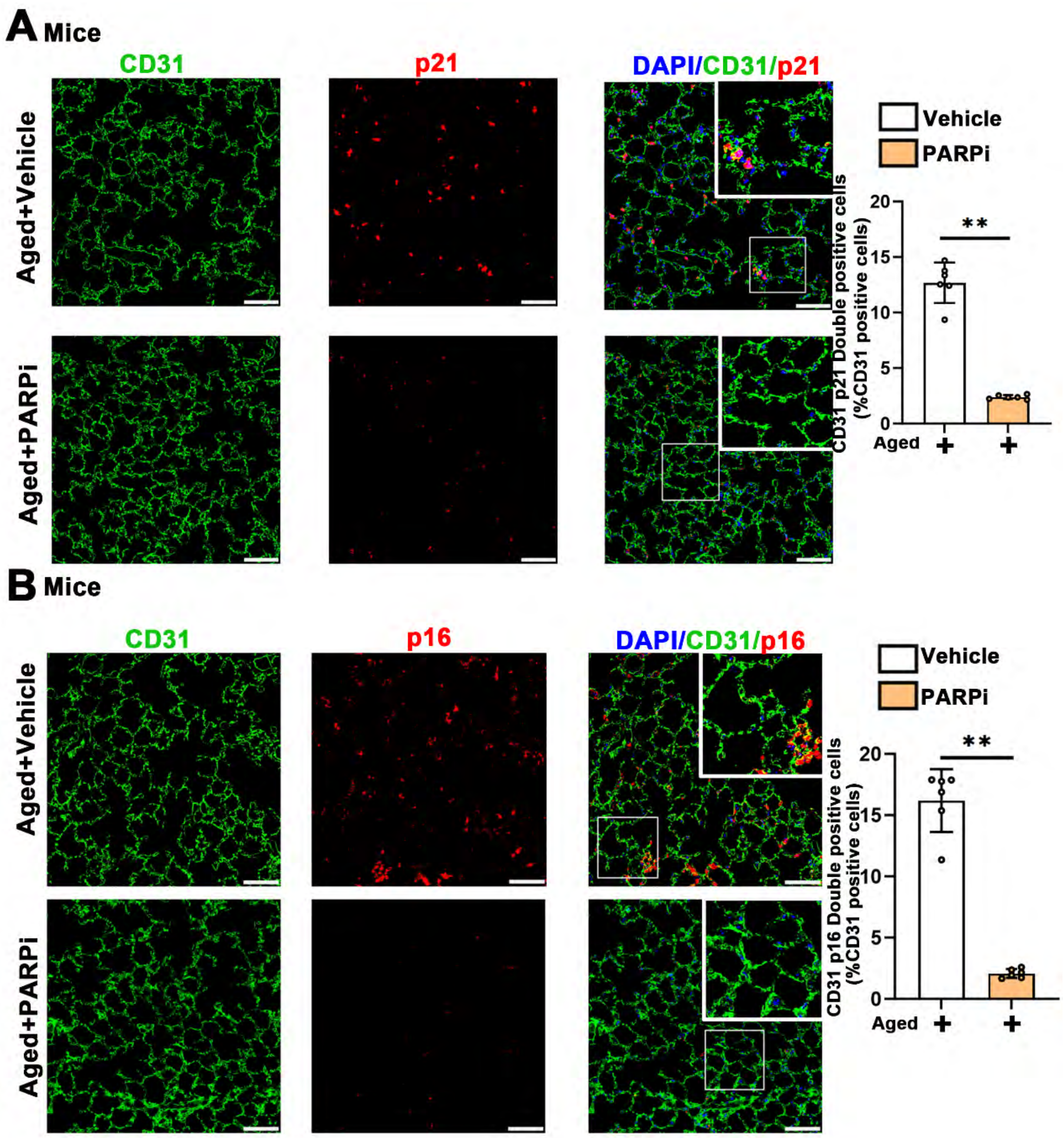

Supplementary Fig. 19 The PARP1/2 inhibitor alleviates pulmonary endothelial senescence in naturally aged mice. Aged mice were intraperitoneally injected with vehicle or the PARP1/2 inhibitor (PARPi) olaparib (5 mg/kg) in saline containing 0.1% DMSO every other day. Thirty days later, lung tissues were harvested for immunofluorescence. **A**, Representative senescent markers staining showing p21 (red) expression in lung sections. Endothelial cells were immunostained with anti-CD31 (green). Nuclei were counterstained with DAPI (blue). Scale bar, 50 μm. The

percentage of $CD31^+p21^+$ cell numbers in total $CD31^+$ cells was presented in the right panels. **B**, Representative senescent markers staining showing p16 (red) expression in lung sections. Endothelial cells were immunostained with anti-CD31 (green). Nuclei were counterstained with DAPI (blue). Scale bar, 50 μm. The percentage of $CD31^+p16^+$ cell numbers in total $CD31^+$ cells was presented in the right panels. Data were presented as means ± SEM (n = 6). ** $p<0.01$.

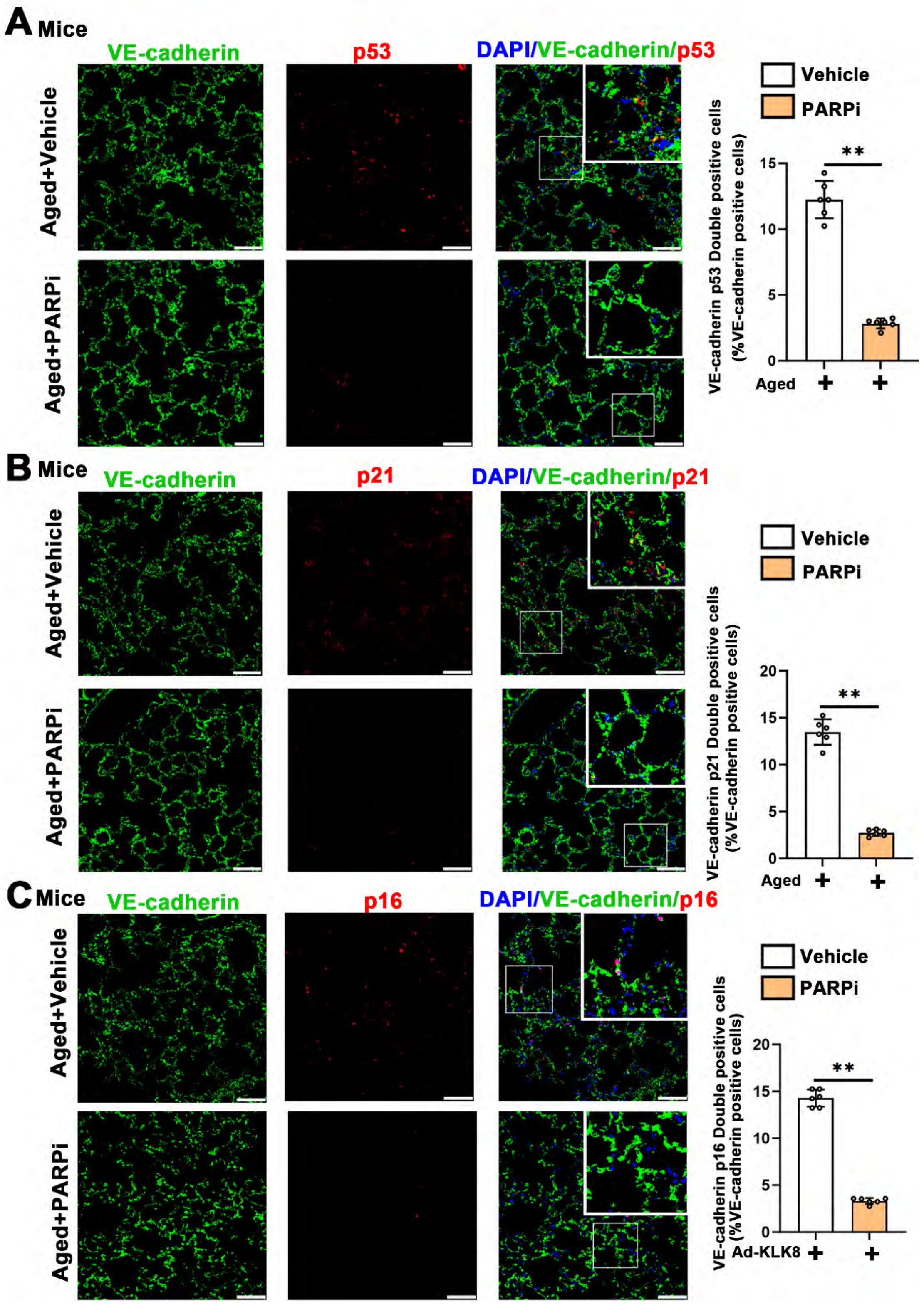

Supplementary Fig. 20 The PARP1/2 inhibitor alleviates pulmonary endothelial senescence in naturally aged mice. Aged mice were intraperitoneally injected with vehicle or the PARP1/2 inhibitor (PARPi) olaparib (5 mg/kg) in saline containing 0.1%

DMSO every other day. Thirty days later, lung tissues were harvested for immunofluorescence. **A-C**, Representative senescent markers staining showing p53, p21 and p16 (red) expression in lung sections. Endothelial cells were immunostained with anti-VE-cadherin (green). Nuclei were counterstained with DAPI (blue). Scale bar, 50 μm. The percentage of VE-cadherin$^+$p53$^+$, VE-cadherin$^+$p21$^+$ and VE-cadherin$^+$p16$^+$ cell numbers in total VE-cadherin$^+$ cells were presented in the right panels. Data were presented as means ± SEM (n = 6). ** p<0.01.

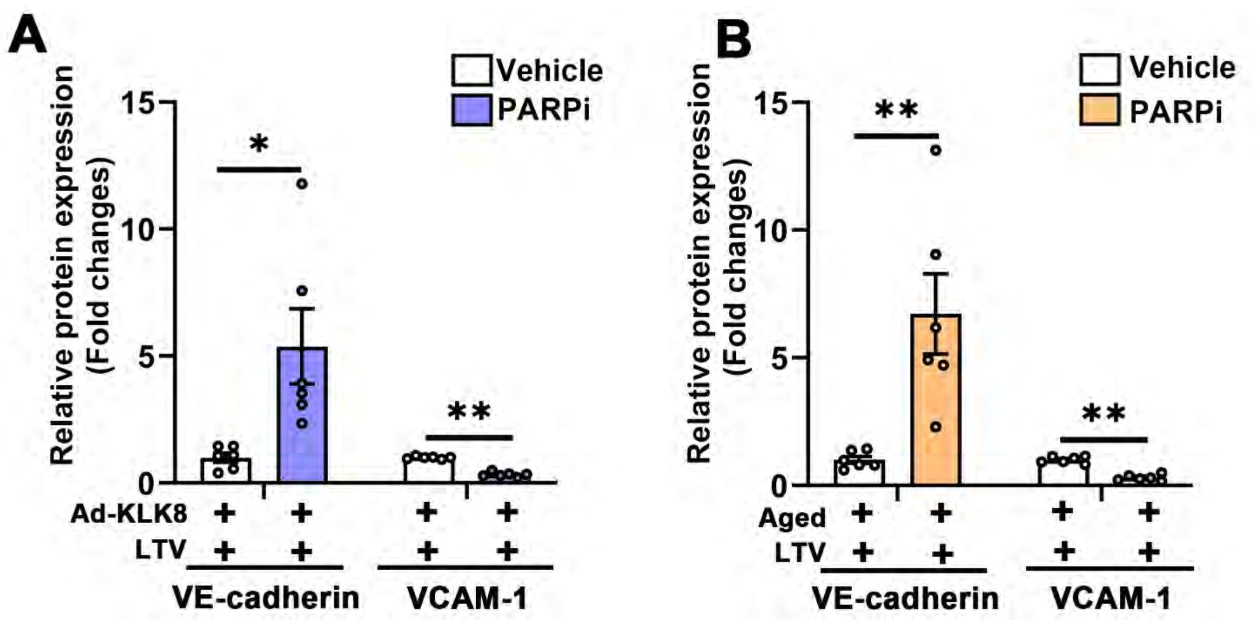

Supplementary Fig. 21 The PARP1/2 inhibitor mitigates the elevated susceptibility to low-magnitude mechanical stretch in both intra-pulmonary KLK8-overepxressing mice and aged mice. A, Mice were intraperitoneally injected with vehicle or the PARP1/2 inhibitor (PARPi) olaparib (5 mg/kg) in saline containing 0.1% DMSO every other day. Fourteen days later, they were intratracheally transfected with Ad-KLK8 (1 × 10^8 pfu). Seventy-two hours later, mice with intra-pulmonary KLK8 overexpression that were treated with either vehicle or olaparib were subjected to LTV mechanical ventilation (8 mL/kg) for 4 hours. Lung tissues were harvested for Western Blot analysis. Relative densitometry of the VE-cadherin and VCAM-1 protein band in lung tissues were shown in bar graphs. **B**, Aged mice were intraperitoneally injected with vehicle or olaparib (5 mg/kg) in saline containing 0.1% DMSO every other day. Thirty days later, aged mice treated with either vehicle or olaparib were subjected to LTV mechanical ventilation (8 mL/kg) for 4 hours. Relative densitometry of the VE-cadherin and VCAM-1 protein band in lung tissues were shown in bar graphs. Data

were presented as means ± SEM (n = 6). * $p<0.05$, ** $p<0.01$.

Supplementary Tables

Supplementary Table. S1 Characteristics of the Patients with Intraoperative Mechanical Ventilation Support

Characteristics	n=79
Age (years)	53.81±1.97
Sex, Female (%)	55.7%
BMI (kg/m^2)	23.15±0.40
Asthma, positives (%)	0
Chronic bronchitis, positives (%)	0
COPD, positives (%)	0
Pulmonary fibrosis, positives (%)	0
Acute pneumonia (Recent one month) , positives (%)	0

Supplementary Table. S8 Primer sequences used in the real-time quantitative PCR

Gene Names	Forward (5'-3')	Reverse (3'-5')
β-actin (Mouse)	CCACTGCCGCATCCTCTTCC	CTCGTTGCCAATAGTGATGACCTG
KLK8 (Mouse)	GCTCTGGTGAGCCCTGCC	CAGCTCCGGAAACACCTCCT
β-actin (Human)	TGTGTTGGCGTACAGGTCTTTG	GGGAAATCGTGCGTGACATTAAG
KLK8 (Human)	CTTCTTCAACTGCGTGACCA	AGTGCACCATCACACACCAG

Supplementary Table S2. Characteristics of the Patients with Intraoperative Mechanical Ventilation Support

Number	Analysis	Age (Years)	Gender	Height (m)	Weight (kg)	BMI (kg/m^2)	ASA Grade	asthma (Y/N)	chronic bronchitis (Y/N)	COPD (Y/N)	pulmonary fibrosis (Y/N)	acute pneumonia (Recent one month) (Y/N)
1	RNA-seq, PCR, ELISA	25	F	1.63	54	20.32444	2	N	N	N	N	N
2	RNA-seq, PCR, ELISA	26	M	1.73	66	22.05219	2	N	N	N	N	N
3	RNA-seq, PCR, ELISA	42	M	1.7	45	15.57093	2	N	N	N	N	N
4	RNA-seq, PCR, ELISA	48	F	1.55	50	20.81165	2	N	N	N	N	N
5	RNA-seq, PCR, ELISA	49	F	1.57	55	22.31328	2	N	N	N	N	N
6	RNA-seq, PCR, ELISA	50	M	1.69	70	24.50895	2	N	N	N	N	N
7	RNA-seq, PCR, ELISA	50	F	1.55	60	24.97399	2	N	N	N	N	N
8	RNA-seq, PCR, ELISA	51	F	1.6	62	24.21875	2	N	N	N	N	N
9	RNA-seq, PCR, ELISA	52	F	1.57	55	22.31328	2	N	N	N	N	N
10	RNA-seq, PCR, ELISA	55	F	1.58	52	20.83	2	N	N	N	N	N
11	RNA-seq, PCR, ELISA	55	F	1.6	48.5	18.94531	3	N	N	N	N	N
12	RNA-seq, PCR, ELISA	56	F	1.6	45.6	17.8125	3	N	N	N	N	N
13	RNA-seq, PCR, ELISA	57	M	1.84	72	21.26654	2	N	N	N	N	N
14	RNA-seq, PCR, ELISA	58	M	1.65	72	26.44628	2	N	N	N	N	N
15	RNA-seq, PCR, ELISA	61	F	1.63	67	25.21736	3	N	N	N	N	N
16	RNA-seq, PCR, ELISA	61	F	1.57	55	22.31328	2	N	N	N	N	N
17	RNA-seq, PCR, ELISA	74	F	1.67	68	24.38237	3	N	N	N	N	N
18	RNA-seq, PCR, ELISA	77	M	1.62	73	27.81588	2	N	N	N	N	N
19	PCR, ELISA	26	M	1.72	70	23.66144	2	N	N	N	N	N
20	PCR, ELISA	30	M	1.83	82	24.48565	2	N	N	N	N	N
21	PCR, ELISA	32	F	1.56	55	22.60026	2	N	N	N	N	N
22	PCR, ELISA	50	F	1.52	63	27.26801	2	N	N	N	N	N
23	PCR, ELISA	50	M	1.72	75	25.35154	2	N	N	N	N	N
24	PCR, ELISA	51	F	1.65	58	21.30395	2	N	N	N	N	N
25	PCR, ELISA	52	M	1.75	74	24.16327	2	N	N	N	N	N
26	PCR, ELISA	53	F	1.65	75	27.54821	2	N	N	N	N	N
27	PCR, ELISA	54	F	1.63	47.5	17.87798	3	N	N	N	N	N
28	PCR, ELISA	57	F	1.59	67	26.50212	2	N	N	N	N	N
29	PCR, ELISA	57	M	1.61	74	28.54828	2	N	N	N	N	N
30	PCR, ELISA	59	F	1.6	59	23.04688	2	N	N	N	N	N
31	PCR, ELISA	63	M	1.76	66	21.30682	2	N	N	N	N	N
32	PCR, ELISA	64	F	1.65	55	20.20202	2	N	N	N	N	N
33	PCR, ELISA	64	M	1.68	59	20.9042	2	N	N	N	N	N
34	PCR, ELISA	65	M	1.78	95	29.98359	3	N	N	N	N	N
35	PCR, ELISA	66	F	1.61	71	27.39092	2	N	N	N	N	N
36	PCR, ELISA	66	F	1.65	70	25.71166	2	N	N	N	N	N
37	PCR, ELISA	68	M	1.78	91	28.72112	3	N	N	N	N	N
38	PCR, ELISA	71	M	1.7	72	24.91349	2	N	N	N	N	N
39	PCR, ELISA	73	F	1.6	60.5	23.63281	3	N	N	N	N	N
40	PCR, ELISA	73	M	1.75	95	31.02041	2	N	N	N	N	N
41	PCR, ELISA	77	M	1.73	58	19.3792	2	N	N	N	N	N
42	Double immunofluorescence	31	F	1.56	57	23.42209	2	N	N	N	N	N
43	Double immunofluorescence	19	M	1.72	45	15.21092	2	N	N	N	N	N
44	Double immunofluorescence	32	F	1.6	48	18.75	2	N	N	N	N	N
45	Double immunofluorescence	38	M	1.72	74	25.01352	2	N	N	N	N	N
46	Double immunofluorescence	20	F	1.54	55	23.19109	2	N	N	N	N	N
47	Double immunofluorescence	34	M	1.72	60	20.28123	2	N	N	N	N	N
48	Double immunofluorescence	40	F	1.6	70	27.34375	2	N	N	N	N	N
49	Double immunofluorescence	34	F	1.75	85	27.7551	2	N	N	N	N	N
50	Double immunofluorescence	28	F	1.63	54	20.32444	3	N	N	N	N	N
51	Double immunofluorescence	31	F	1.6	50	19.53125	2	N	N	N	N	N
52	Double immunofluorescence	33	F	1.62	60	22.86237	2	N	N	N	N	N
53	Double immunofluorescence	33	F	1.65	34	12.48852	2	N	N	N	N	N
54	Double immunofluorescence	67	F	1.58	58	23.23346	2	N	N	N	N	N
55	Double immunofluorescence	67	M	1.73	60	20.04745	2	N	N	N	N	N
56	Double immunofluorescence	69	M	1.68	68	24.09297	2	N	N	N	N	N
57	Double immunofluorescence	71	M	1.7	71	24.56747	2	N	N	N	N	N
58	Double immunofluorescence	70	F	1.56	64	26.29849	2	N	N	N	N	N
59	Double immunofluorescence	77	F	1.52	52.5	22.72334	3	N	N	N	N	N
60	Double immunofluorescence	75	M	1.65	65	23.87511	3	N	N	N	N	N
62	Double immunofluorescence	72	F	1.66	56	20.32225	2	N	N	N	N	N
63	Double immunofluorescence	73	F	1.6	59	23.04688	3	N	N	N	N	N
64	Double immunofluorescence	72	F	1.6	63	24.60938	3	N	N	N	N	N
65	Double immunofluorescence	71	M	1.78	80	25.24934	3	N	N	N	N	N
66	Double immunofluorescence	71	F	1.54	71	29.93759	3	N	N	N	N	N
67	Double immunofluorescence	78	M	1.7	56	19.37716	2	N	N	N	N	N
68	Double immunofluorescence	72	M	1.7	78	26.98962	2	N	N	N	N	N
69	WB	39	F	1.7	54	18.68512	2	N	N	N	N	N
70	WB	32	F	1.55	52.5	21.85224	2	N	N	N	N	N
71	WB	30	F	1.55	52.5	21.85224	1	N	N	N	N	N
72	WB	13	M	1.75	52	16.97959	2	N	N	N	N	N
73	WB	32	F	1.64	49	18.21832	2	N	N	N	N	N
74	WB	40	M	1.78	80	25.24934	2	N	N	N	N	N
75	WB	66	F	1.67	73	26.17519	2	N	N	N	N	N
76	WB	76	M	1.7	67	23.18339	2	N	N	N	N	N
77	WB	70	M	1.75	75	24.4898	5	N	N	N	N	N
78	WB	68	M	1.65	70	25.71166	3	N	N	N	N	N
79	WB	67	M	1.75	75	24.4898	3	N	N	N	N	N
80	WB	72	M	1.73	72	24.05693	2	N	N	N	N	N

Supplementary Table S3. Differentially expressed genes (DEGs) between control and senescent endothelial cells (GSE228491)

	p_val	avg_log2FC
Gm49595	4.55E-11	8.05055301
Gm11651	8.71E-11	7.92177191
Gm47551	3.12E-09	7.75620511
Gm37640	1.12E-07	7.4578995
Gm26759	7.94E-07	7.25240995
Gm49173	2.94E-06	7.06054398
Slc24a5	2.94E-06	7.05935575
Gm29237	4.11E-28	7.04539658
Gm17546	2.12E-06	7.04020996
Synpr	7.86E-06	7.03179706
Wfdc10	1.52E-05	6.99670501
Gm16064	1.09E-05	6.97459479
C1qtnf12	7.86E-06	6.90782155
Gm45509	1.52E-05	6.74250669
Gm26964	5.67E-05	6.69015427
Gm17089	2.93E-05	6.64772794
)30545L23F	9.78E-20	6.63641099
Gm28153	5.67E-05	6.58733192
Kctd4	5.67E-05	6.55502005
Slc10a1	4.08E-05	6.49907283
'00015H07F	0.000153	6.48554961
Gm16043	0.000214	6.46268418
Gm42161	0.00011	6.43807232
30422M22F	0.001143	6.38253253
Gm16183	0.000214	6.37365768
Gm17359	0.000299	6.36333301
Gm9844	0.000299	6.32416925
Gm44759	0.00011	6.3036494
Gm22146	0.000214	6.28551308
Gm26724	7.11E-18	6.27812935
Gm47471	0.000299	6.24869326
Gm29358	0.000214	6.24357236
Gm45345	0.000299	6.24142485
Gm29682	0.000583	6.20846697
Gm17545	0.000214	6.17524076
Gm45075	0.000816	6.16804322
Gm16031	0.001603	6.14823461
Gm26979	0.000214	6.14325581
Gm16185	0.000583	6.14230378
Gm16000	0.000299	6.13982998
Vmn2r9	0.000816	6.04356781
Gm13580	0.002252	6.03631161
Gm15965	0.001603	6.01092717
Gm16572	0.002252	6.0052218
'00047K16F	0.001603	6.00170988
Hoxa4	0.001603	5.99521384
Gm48727	0.000583	5.98741995
Gm15591	0.003168	5.9632864
Gm36992	0.001143	5.95881889
Acy3	0.002252	5.92022484
Gm38287	0.000816	5.91913111
Gm28192	0.001143	5.91895942
Ascl4	0.001143	5.91272073
Gm20492	0.001143	5.90912585
;30011L22F	2.43E-12	5.8902731
Hist2h2aa1	0.003168	5.88232644
;31405J19R	0.001603	5.83686106

Gm11713	1.17E-30	5.83309671
Pitpnm2os1	0.002252	5.8138743
Arhgap20os	0.001603	5.81195488
Gm44020	0.002252	5.76733164
Cst9	0.003168	5.75741546
Spag11b	0.003168	5.74348975
Gm28085	0.001143	5.71135137
Gm49958	0.001603	5.69831726
700007J10R	0.003168	5.69343034
Gm50196	0.003168	5.69016555
Gm10602	0.003168	5.67941645
Klk10	0.003168	5.66867625
Ecrg4	0.006304	5.65790974
Gm15889	0.004464	5.65573645
Gchfr	0.018035	5.65200131
H2-Q1	0.002252	5.64637526
Gm50132	0.004464	5.63926183
Gm48855	0.003168	5.63562288
Anxa11os	0.003168	5.62265384
Smpd5	0.006304	5.62020259
Lipf	0.003168	5.60378115
Gm48683	0.006304	5.60057346
Gm41442	0.003168	5.57242933
Gm9889	0.006304	5.57100793
Gm50130	0.004464	5.54914046
Plppr3	0.004464	5.53799352
Gm16150	0.003168	5.53298362
Gm8237	0.008922	5.50898683
Sap18b	0.006304	5.50567779
Gm49227	0.008922	5.49053805
Tnnt2	0.012664	5.48616283
330454E08R	0.006304	5.48032766
Dlx1as	0.012664	5.47807876
Gm27240	0.004464	5.47157419
Glp2r	0.003168	5.46607434
Sstr2	0.008922	5.46343976
Gm47320	0.004464	5.46283923
30586N03R	0.006304	5.43923169
310063I02R	0.008922	5.43313722
Gm16156	0.008922	5.43203193
Gm28723	0.012664	5.38812144
Gm12705	0.008922	5.38376201
30415G19R	0.006304	5.38035071
Gm12802	0.008922	5.36061375
Gm15821	0.008922	5.35790319
410080I02R	0.008922	5.35458275
Gm34552	0.006304	5.34807923
Colec11	0.008922	5.34365247
Gm26952	0.018035	5.34004527
Platr8	0.018035	5.31513326
Gm11627	0.012664	5.3035153
Gm42439	5.85E-08	5.28803658
330009N23R	0.012664	5.2846118
700063J08R	0.012664	5.28026743
Gm16178	0.012664	5.25891266
Gm16283	0.012664	5.25285943
Gm43118	0.012664	5.25273626
Lingo1	0.018035	5.25247373

Gm20089	0.008922	5.24708274
Hic1	0.012664	5.24471094
Gm20456	0.012664	5.21421912
30013A20F	5.85E-08	5.21370296
Prl2c3	0.012664	5.20726799
Vps25	0.018035	5.20500787
30427M07F	0.012664	5.20289841
Gm37850	0.018035	5.19781309
Gm4632	0.012664	5.19772549
Amelx	0.018035	5.18702697
Retn	0.018035	5.17995137
'00016C15F	0.018035	5.15239364
Gm15594	0.012664	5.14392272
Gm15243	0.018035	5.13139231
Slc22a20	0.018035	5.12873887
Gm17638	0.012664	5.11276296
Nptx1	0.012664	5.1062087
Angptl8	0.012664	5.10487253
Gm26648	0.018035	5.10327404
Gm50340	0.018035	5.09278941
130051J06F	0.037048	5.0923254
Olfr655	0.025785	5.08619675
Gm16725	0.018035	5.07871613
Gm16253	0.012664	5.07763097
Gm26852	0.037048	5.06827335
Gm9984	3.12E-11	5.06612852
Gm10532	0.018035	5.06104861
Ddi1	6.09E-09	5.06003284
Camp	0.018035	5.05548596
Gm47272	0.012664	5.05288781
Gm15342	0.018035	5.04648289
BC065403	0.012664	5.04433604
Gm10575	2.30E-09	5.03886942
Gm16712	0.012664	5.02510401
Gm7480	6.42E-13	5.0237841
Gm16268	0.037048	5.01649323
Gm43150	0.025785	5.00922401
Gm16033	0.018035	5.00282661
Gm5767	0.018035	5.0008068
Bpifa1	4.27E-231	4.99009591
Serpine3	0.018035	4.98850848
Gm44129	0.018035	4.98603794
Celf3	0.012664	4.96924082
30111J21Ri	0.018035	4.95478326
Gm38804	0.025785	4.95060358
Gm16239	0.018035	4.94342562
Gm13179	0.037048	4.94238922
Gm27200	0.018035	4.93910737
Pcsk1n	0.037048	4.93768338
'21516A02F	0.025785	4.93266379
Gm17080	0.025785	4.92761388
Hbb-bt	5.61E-290	4.91775085
Gm26879	0.037048	4.9105911
30578M07F	0.025785	4.9093881
Gm15890	0.018035	4.90446694
Gm7467	0.025785	4.90216808
Gm15674	0.025785	4.89688312
Gpr179	0.037048	4.88920683

Ccdc7a	0.025785	4.88315745
Hbb-bs	0	4.87667574
Gm15551	0.025785	4.87485033
Gm45820	0.037048	4.87222525
30455H04F	0.025785	4.86719889
Gm40117	0.025785	4.86216418
Gm15527	0.018035	4.86194902
Lrfn3	0.037048	4.85964194
Gsg1	0.025785	4.85830665
Macrod2os1	0.037048	4.85688328
Gm15813	0.037048	4.84543587
Sln	0.025785	4.84150107
Gm27252	0.037048	4.83036834
Gm10432	0.037048	4.82841271
Gm50237	0.025785	4.82525029
C79798	1.13E-07	4.82509365
Gm44686	3.60E-15	4.8225772
Gm12158	0.025785	4.80760032
Gm43091	0.037048	4.80666687
Gm26688	0.025785	4.8013459
Lrrc14b	0.037048	4.79095366
Capn12	0.025785	4.78607527
Gm49475	0.025785	4.77829318
Gm10382	0.025785	4.77626895
Lbx2	0.037048	4.77415646
Xkr8	0.025785	4.76493668
Gm32688	0.025785	4.76010446
Gm28258	0.025785	4.7564351
)31425F14R	0.037048	4.75299621
Gm50145	0.025785	4.75236425
Gm20631	3.60E-44	4.7504393
Ngb	7.82E-06	4.74799798
Gm28809	2.12E-06	4.74078424
Gm7247	0.037048	4.73790347
Gm32849	0.025785	4.73236724
Gm15672	0.025785	4.73120508
Rhd	0.025785	4.73059166
Gm38293	0.037048	4.71413558
Spink4	0.037048	4.71147145
Ufsp1	0.025785	4.70856141
Gstm7	0.037048	4.70451116
Peak1os	0.037048	4.70413736
Gm48682	0.025785	4.70310335
Slc46a2	0.037048	4.68015211
Gm34006	0.025785	4.67233744
Hspb7	0.025785	4.66792498
Gm20544	0.037048	4.62964811
Gm15423	0.025785	4.61819086
530001N23F	0.037048	4.61299044
Gm43514	0.037048	4.60943232
Plac9b	0.037048	4.60892596
Gm50138	1.58E-07	4.60796228
Gstm6	0.037048	4.6072601
Rab42	0.037048	4.59764913
Gm49926	0.025785	4.59428413
Pard3bos3	8.01E-07	4.59356671
Gm16063	0.025785	4.59305365
Crtac1	0.037048	4.58918497

Gm16316	0.037048	4.58507337
Krtap17-1	0.037048	4.57709199
Itgb2l	0.037048	4.57003288
700108J01R	0.037048	4.56516727
Gm16137	0.037048	4.56481322
Hist1h4d	1.78E-15	4.54248398
Gm7580	2.13E-06	4.52074722
Gm50250	5.99E-08	4.52069879
Gm45235	0.037048	4.51835051
Carmil3	0.037048	4.51133859
Padi1	0.037048	4.50687224
Gm11714	6.21E-11	4.49139867
Hsbp1l1	0.037048	4.46436069
30478M13R	0.037048	4.44594374
Gm44036	0.037048	4.44125138
33428G20R	0.037048	4.4336914
Sncg	0.037048	4.42472945
730049H05R	4.02E-05	4.39339031
Gm16982	2.91E-05	4.38519043
Gm48228	4.11E-06	4.38215835
Gm48383	2.38E-19	4.31026356
Uchl4	2.01E-07	4.30211649
Gm32250	3.20E-17	4.29313758
Gm16193	0.000108	4.27657839
Rpl10	5.38E-158	4.25962827
Rnaseh2c	1.27E-19	4.25354683
Gm20616	8.15E-25	4.22186546
Atad3aos	0.000291	4.20427692
Rapgef4os1	1.09E-10	4.15170132
Slurp1	4.98E-130	4.14228173
10008P14R	1.42E-22	4.05191579
Gm16267	0.001533	4.03067989
Gm50232	2.57E-11	4.01813659
Gm32036	7.25E-24	3.9916041
Gm46367	1.13E-62	3.95655216
Cst8	0.000566	3.95543667
00061H18R	1.49E-07	3.9552412
30579G18R	1.56E-08	3.93405638
Hist1h4h	7.94E-06	3.93195846
Angptl3	1.53E-05	3.90542055
Ldhd	0.0011	3.90126693
30447N08R	2.82E-07	3.89666239
Mfap2	1.63E-18	3.88728669
Gm41764	1.38E-08	3.87318815
Gm26936	6.92E-21	3.87068414
Gm15738	2.32E-38	3.86965241
Rpl35	2.42E-172	3.85169604
Gm13708	4.23E-07	3.84019991
Gm11209	2.12E-05	3.83977855
Myl7	1.14E-13	3.83931822
Gm31763	7.17E-14	3.83134882
Gm49267	8.19E-09	3.81157333
Gm10097	0.00021	3.80749423
Gm11808	1.37E-05	3.7683286
Pard3bos1	3.96E-48	3.76198021
Gm15336	1.37E-13	3.731414
30028H03R	1.35E-41	3.72813902
Gm13748	4.98E-05	3.72758871

Gm50397	0.008252	3.71203391
Gm44040	0.002147	3.70097346
Rps27	3.22E-239	3.69553846
Bmyc	2.21E-08	3.69217534
Gm45479	0.002997	3.68415898
Lsm10	1.69E-06	3.67130955
Gm48250	4.87E-10	3.65695749
Gm20652	0.000569	3.64987933
Gm10503	2.64E-08	3.64723007
Gng5	2.00E-148	3.6460213
Gm50100	3.61E-05	3.64458944
Apoc1	8.40E-14	3.63427105
Gm19710	6.37E-18	3.62988595
Fabp1	5.24E-57	3.61788848
Gm13684	4.12E-10	3.60839956
Sox5os4	1.92E-05	3.60731933
Gm34455	0.000794	3.60504403
Gm11659	1.54E-10	3.60443967
Gm11716	0.000152	3.60289122
10204G07F	0.002142	3.59755945
Rtl5	0.00215	3.59567006
Gm11250	0.000211	3.59463464
Scgb1c1	2.15E-10	3.57019734
30175M20F	1.85E-41	3.56364634
Pcdhgc3	0.008248	3.56110745
Gm12227	0.000152	3.55463223
Gm46329	0.004205	3.5423897
00085D07F	4.06E-35	3.52799851
Gm12002	0.000793	3.52406702
Gm28370	1.45E-06	3.52093155
Pln	7.25E-06	3.51312044
Fam171a2	9.56E-05	3.51213814
Gm42982	8.40E-06	3.48006339
Rpl15	2.97E-156	3.46573973
Gm46124	9.63E-05	3.46436279
Gm30025	0.000351	3.46297481
H2-Q10	0.001541	3.44932928
Tyrobp	1.15E-96	3.44344645
Rps29	0	3.44105228
Gm20721	3.11E-08	3.43316681
Gm17058	3.92E-07	3.42887376
Gm14211	1.44E-65	3.42823804
Bmp8b	0.004207	3.42305293
Gm15942	1.10E-07	3.40865229
330050I16R	0.008257	3.40822911
030019D19F	0.005887	3.40676384
mt-Nd2	1.26E-186	3.39144579
Plxna4os3	6.09E-06	3.39045122
Gm5149	2.65E-17	3.38621122
Gm28940	0.002155	3.37873752
230059L01F	0.023	3.37612892
Rpl34	1.88E-244	3.37425726
Ttc36	2.09E-07	3.36162612
31440D22F	0.001545	3.36112432
mt-Atp6	0	3.36025235
Gm42413	2.95E-05	3.35898694
Gm19522	9.21E-20	3.35689161
Tchh	1.25E-06	3.35215269

Insyn2a	3.34E-28	3.35048999
Ube4bos1	1.25E-06	3.33772266
)30422I22R	7.49E-14	3.33390544
Gm50071	1.43E-06	3.32858738
Hoxaas3	1.67E-10	3.31549171
Zfp14	6.62E-07	3.31517657
)30549C15F	2.06E-11	3.31440342
'00016K05F	1.84E-19	3.3141713
510009L18F	0.000928	3.31102911
Gm16006	0.004203	3.3086181
Gm16175	0.001545	3.30447219
30517O19F	8.32E-22	3.30379131
Lgals2	1.86E-13	3.30351133
Rd3	0.023012	3.30295546
Gm21798	0.023051	3.30230609
Gm15261	7.42E-35	3.29869601
Gm26531	0.004212	3.29646089
Edf1	1.32E-42	3.28922356
Fcor	1.26E-11	3.28860181
Gm43507	4.34E-40	3.28779299
Il17c	0.005897	3.2837642
S100a9	1.95E-11	3.27424299
Gm16174	0.016322	3.27372216
Gm10791	0.008262	3.27259862
330050J10F	0.000485	3.26415317
30557K07F	1.52E-09	3.25667709
Gm13381	0.011616	3.25448329
Gm16068	1.10E-07	3.24725239
Gm50217	0.016322	3.24657886
Rps2	1.70E-127	3.24533107
Gm15511	0.005897	3.24405323
Gm27206	0.005897	3.24064873
Atxn7l1os1	9.10E-07	3.22707
Rpl30	0	3.22690756
Oxld1	1.17E-05	3.22424334
Hba-a1	0	3.22276073
Lrrc8dos	0.001547	3.22216929
Gm48677	0.005904	3.21870565
230398E01F	0.01634	3.21203634
Gm15346	0.003019	3.20750569
Lysmd4	1.99E-38	3.20418937
Taco1os	0.002466	3.20184558
Tg	4.17E-08	3.19544267
Gm14858	1.34E-07	3.18741436
Prxl2b	0.005904	3.18667577
Gm13703	0.00216	3.18372457
10041H14F	0.005911	3.18349653
Pdap1	6.74E-35	3.18331535
Pmel	0.004219	3.1764914
Gm49226	5.68E-05	3.16062046
Mapkapk5	0.00828	3.1579153
30415O20F	0.004219	3.15529742
Slc16a14	0.008276	3.15511974
Col7a1	0.016358	3.15475648
Gm28379	3.79E-10	3.14729017
Gm13427	1.01E-05	3.14281028
Gm11457	1.14E-07	3.14198274
'00066B19F	4.55E-11	3.13103939

Gm38604	1.28E-09	3.12665227
Sox5os2	0.005911	3.12469734
Gm3848	2.05E-08	3.12395678
)30403P22R	0.016331	3.11843137
Ndufa13	2.05E-83	3.11236488
Gstm5	0.00111	3.11213077
Rpl9	9.46E-224	3.11103732
Smim18	0.046007	3.10945469
C1qtnf2	1.43E-06	3.10779596
Gm16054	0.003019	3.10257353
Mien1	2.24E-21	3.10005054
Timm8b	5.54E-22	3.09929443
Gm36363	0.01161	3.09607139
AW551984	0.004219	3.09552428
Gngt2	1.55E-28	3.09372958
Hrct1	0.046032	3.09018812
Gm17029	0.003016	3.08509036
Gm49969	0.00041	3.08191147
Fxyd3	1.85E-42	3.07899875
Gm15489	3.45E-07	3.07685657
Gm36617	0.004734	3.07242187
Gm42836	4.27E-08	3.07045003
S100a1	1.93E-85	3.06528823
Gm11476	4.85E-12	3.06474287
Dtnbos	1.18E-05	3.06129376
33433G19R	0.000184	3.06013961
Gm16152	2.10E-08	3.05882927
Arl11	1.26E-06	3.05852633
Gm44649	4.26E-10	3.0572563
Ftl1	0	3.0472945
Gm44079	0.000672	3.04403392
B3gnt9	8.41E-08	3.04353761
Fam89a	0.00129	3.04347073
Angptl6	0.032528	3.0431646
Rpl29	2.86E-89	3.04034157
Gm15558	0.001109	3.03797523
Hist1h2ap	0.002461	3.03717568
Tmsb10	8.74E-205	3.03463583
Rpl18a	7.80E-252	3.02858131
Rpl11	6.31E-251	3.02665343
Gm16741	0.004714	3.02526184
700022I11R	0.00829	3.01069359
Gm13402	7.42E-09	3.009301
Gm26632	2.06E-35	3.00222788
mt-Atp8	5.85E-27	2.99051824
Adrb3	0.00829	2.98427943
Gm47460	0.00829	2.98313408
Dynlt1f	4.97E-08	2.9785176
)30484I04R	6.07E-09	2.97711717
Gm46404	0.032528	2.97675057
\rhgap27os	0.000134	2.97203143
Gm5617	1.02E-09	2.97151679
Gm10848	0.016349	2.97041911
Gm27241	0.000489	2.96351796
Rpl23	0	2.95971068
Pcbd1	0.00053	2.95689563
Rps11	1.63E-195	2.95595177
Bola1	1.70E-20	2.95515505

530014C17F	0.023063	2.95325895
mt-Nd4	7.79E-225	2.94419511
Bola3	7.08E-15	2.94223466
Gm15347	0.023038	2.93784034
530016G05F	0.023076	2.93560997
BB365896	0.004736	2.93436816
Nupr1	4.82E-76	2.93383686
Gm37165	0.005917	2.93243398
Map3k6	4.43E-07	2.9319268
Gm43376	5.69E-14	2.92707033
Uba52	1.00E-18	2.92091575
930556J24R	1.58E-06	2.91947815
B3gat2	4.48E-07	2.9157476
Pram1	1.27E-09	2.91287063
Gm31172	5.68E-05	2.90694884
Gm9968	7.18E-08	2.90536291
Vpreb1	0.046083	2.89824332
Cngb1	0.023038	2.89460378
22401L13R	5.74E-10	2.89191328
Atp8b5	7.77E-12	2.89056758
S100g	6.49E-45	2.89038905
Zfand2b	8.40E-07	2.88767339
Maged2	1.27E-06	2.88030722
230217C12F	0.032599	2.86198822
Mettl18	0.000939	2.8607221
Leng9	4.23E-21	2.85933639
Gm26877	0.001291	2.8584766
Gm17227	1.22E-12	2.85674594
30438D12F	3.36E-60	2.85552027
Fbxo2	0.023063	2.85523263
Gm10603	0.011649	2.8550016
G0s2	3.99E-06	2.85224401
mt-Cytb	1.65E-175	2.85217773
Gm44752	0.000134	2.85114504
30409C15F	3.32E-21	2.8494499
Anapc13	6.89E-25	2.84814625
Gm26878	0.001371	2.84713055
Gm17300	0.023025	2.84697433
10035H17F	0.002471	2.84662013
Gm48749	6.29E-05	2.84475962
33413G10F	0.03251	2.84359996
Fth1	0	2.84151059
Dnah2os	0.016363	2.83904397
Gm37229	0.003024	2.83627854
Ifitm2	1.12E-70	2.83492613
Gm17231	9.04E-22	2.82514683
Mannr	0.023089	2.81652796
Pard6a	0.00041	2.81598703
Gm15788	0.003424	2.81293235
Thtpa	0.000491	2.81192519
Gm27008	1.53E-16	2.8049096
Gm17160	0.046058	2.79650539
430095P16F	0.016386	2.79425719
Rps20	0	2.79185035
Gm26771	3.13E-15	2.79157714
Sdf2l1	4.04E-06	2.78908093
Gm26749	5.08E-19	2.78855548
Gm31224	2.06E-17	2.78815678

Gm47167	1.25E-38	2.7846427
Rsf1os1	3.41E-10	2.78169747
Hist1h1e	8.30E-09	2.78041717
Cox7a1	0.008304	2.77723652
Gm10353	1.93E-14	2.77612879
Mmp3	0.00829	2.77348732
30444A19F	1.36E-27	2.7723296
Prss48	0.000207	2.77154874
Gm43713	2.79E-15	2.76640223
Pold4	1.25E-08	2.764082
Gm15418	0.00179	2.7624415
430090L17F	2.13E-09	2.76065647
Hotairm1	1.21E-13	2.7570582
Rps18	1.66E-130	2.75692747
Myo7b	0.003024	2.74937219
30414N17F	0.011643	2.74753381
Nxpe2	1.31E-47	2.7474204
Gapdh	3.45E-72	2.74651745
Cfd	5.80E-08	2.74569817
031403E22F	1.01E-10	2.74379668
Slc4a9	0.032617	2.74369441
Ssr4	1.02E-35	2.74233757
Selenom	1.72E-20	2.73597587
Nkx6-2	0.024107	2.73544725
Gm35188	1.71E-55	2.73419843
Fmod	0.000134	2.72963701
Icam2	1.84E-26	2.72605872
Gm48765	4.60E-16	2.72465368
Gm13008	0.001111	2.72197192
530012P03F	0.0013	2.71946797
Gm15581	1.51E-49	2.71532529
Gm48742	4.74E-05	2.70956363
00061G19F	0.032564	2.70600645
Atp5g1	7.86E-35	2.70585112
Gm47093	0.032599	2.70501429
330024N08F	1.33E-12	2.70349397
Ccn4	0.023089	2.70069069
Gm9828	0.000404	2.6987231
Gm34280	0.016386	2.69853623
Snapc5	6.53E-12	2.69547576
Rapgef4os2	0.006553	2.69291715
30405A10F	8.69E-06	2.69287755
Cdpf1	1.33E-08	2.69197756
Slc39a3	2.36E-10	2.69197653
Gm26788	0.008308	2.69151681
Cox5b	4.08E-35	2.69095734
Rpl6	1.41E-210	2.69023508
30019P16F	8.63E-06	2.68863887
Ddt	1.69E-13	2.68856639
Smim1	4.91E-36	2.68625996
30556N13F	3.07E-06	2.68481953
mt-Nd5	2.25E-69	2.68469388
Gm15563	9.05E-18	2.68103117
Gm26854	0.011669	2.68083379
Ndufb11	1.29E-49	2.67566035
Gm16541	9.56E-21	2.67424851
Rarres2	1.99E-191	2.67345065
Slfn5os	0.024107	2.6727473

Elob	8.67E-76	2.6694582
Pin4	1.18E-22	2.66754742
Tspan17	3.01E-08	2.66752233
BC028777	0.003432	2.65933598
Gm15886	2.75E-14	2.65706253
Dnlz	3.68E-08	2.65685468
33417O13F	4.13E-09	2.65623595
Cyp2j9	0.005927	2.65426951
Plk3	8.65E-07	2.65262966
30414N06F	5.82E-05	2.6508417
Smim27	2.19E-10	2.65055766
Gm28501	1.97E-09	2.64900803
Sftpc	0	2.64746513
Gapdhs	0.000288	2.64636692
Gm48708	1.03E-07	2.64619504
Patl2	0.002483	2.64414165
Gm50242	0.016404	2.64405729
Rtp3	1.24E-06	2.64127695
Krt10	0.000405	2.64037781
Mia	1.45E-06	2.63766336
Atp5j	7.60E-68	2.6355525
Usp46os1	0.006572	2.63359936
Mrpl11	9.74E-11	2.63332328
Gm29361	0.016404	2.63207366
Rpl37	3.13E-279	2.6290022
Rpl28	1.24E-199	2.62743118
Rpl32	5.88E-194	2.62075223
Gm40155	0.004763	2.61439327
30565N06F	4.63E-05	2.61377181
Rpl36a	6.54E-118	2.61207876
Inka1	0.032599	2.60831931
Rps3	4.19E-163	2.60767512
30512B01F	1.27E-06	2.6052455
Gm16599	4.07E-31	2.60510665
Gm20536	3.91E-16	2.6027676
Gm15492	2.68E-06	2.6000123
Atp5md	3.03E-44	2.59984967
Gm15764	4.06E-06	2.5986679
Rpl18	1.13E-217	2.59853033
Rpl13	0	2.5971836
Gstp1	1.07E-06	2.59640732
Pm20d1	0.032617	2.59517921
Kctd11	1.03E-05	2.59163045
Gm15541	5.32E-13	2.58571702
30409D20F	0.032617	2.58543637
Gng3	0.004755	2.58322145
Gm19557	0.032653	2.57708335
Gm26827	9.99E-29	2.57378807
Gm49521	0.000299	2.57270264
Rps13	1.11E-201	2.57242983
Shisa3	0.032599	2.57181875
Gm42560	0.032599	2.5676556
Gm20406	0.046227	2.56732586
Gm13561	7.26E-09	2.56692399
Gm20528	4.43E-08	2.56565555
Gm43915	6.34E-05	2.56550104
30117O12F	0.046133	2.56407343
Suox	0.012577	2.56136278

Trmt112	5.43E-20	2.56039172
Gm10135	2.73E-12	2.55792935
Mrps24	6.72E-22	2.55757649
Rab26os	0.000151	2.55718408
10021N24R	1.27E-19	2.55388289
Slc6a20b	0.003024	2.55295759
Gm31356	4.01E-20	2.55207004
Alas2	7.57E-06	2.55012438
30431A17R	0.001015	2.54927276
Hba-a2	9.91E-234	2.54832987
Hist1h1b	1.92E-07	2.5475424
Cenpx	2.73E-19	2.54700919
Chst4	8.85E-09	2.54689691
Naa38	2.98E-14	2.54578425
Rps25	6.50E-141	2.54202165
Rpl21	3.44E-205	2.53926798
Rps19bp1	1.40E-07	2.5385168
Dpm3	1.13E-29	2.53686724
Gm50013	3.15E-06	2.5327838
Tmed1	7.96E-05	2.5281267
Platr31	0.005927	2.52552934
Acaa1b	6.38E-10	2.52500417
Gm15614	2.33E-28	2.52449779
Zmynd10	0.000159	2.51943958
Rps19	2.86E-232	2.51844023
Far1os	1.86E-15	2.51398695
Gm3235	6.26E-05	2.51335155
00007K13R	4.61E-11	2.5119527
Clec11a	0.004231	2.5101007
mt-Nd4l	2.38E-49	2.5076882
Ifi27l2a	2.11E-39	2.50683671
Park7	1.39E-39	2.50619374
Rps7	1.27E-209	2.50617945
Lpar2	0.046108	2.50394849
Scrn2	0.009103	2.49987339
Gm11536	0.000152	2.49638332
Ndufb9	3.31E-51	2.49353515
Fam181b	0.002493	2.49334193
Ndufc1	4.88E-45	2.49215822
B9d1	8.32E-08	2.49210395
Fkbp2	1.50E-38	2.48321151
Agrp	4.36E-21	2.48195616
Rpl39	1.43E-215	2.47773008
Dohh	1.29E-06	2.47739909
Txndc17	4.57E-35	2.47663414
Gm17590	0.000539	2.4766128
10009A15R	0.001793	2.47057652
Gm17130	0.004874	2.47035752
Gm10563	5.05E-07	2.46961801
Mrpl57	1.18E-22	2.46902132
Nme2	1.46E-62	2.46811106
Hps6	0.000355	2.46760171
Ndufaf8	8.26E-20	2.46712399
Gm11149	0.046171	2.46592096
Uqcrq	5.14E-65	2.4646779
Ltbp4	6.58E-48	2.4642749
Pkd1l3	0.000221	2.4616235
Gm50163	1.33E-05	2.46090554

Gm17133	0.002499	2.46089177
Gm49959	1.81E-07	2.45988545
Gm830	0.011675	2.45667084
Rps12	1.31E-289	2.45664702
Tmem126a	1.76E-21	2.45603113
'00003E16R	0.006676	2.45036523
Gm14471	0.003448	2.44910927
Gm49700	0.012615	2.44827298
Cd300lg	0.000135	2.44726168
130310I04R	0.000111	2.44676715
Zfp30	0.00011	2.44636476
Aard	0.033465	2.44561637
Ost4	7.36E-21	2.44486092
Lrrc4	1.46E-40	2.44276091
Gm17949	0.003457	2.44211761
Scg3	0.003486	2.44017629
Rps10	4.41E-263	2.43968754
Pla2g2d	0.046158	2.43828933
Rps23	5.45E-252	2.4361951
Ubb	1.57E-107	2.43277678
Bloc1s1	5.63E-06	2.43158169
'30063M14I	0.046133	2.4304723
Parvaos	0.008313	2.42910237
Rpl26	1.02E-201	2.42746148
Gm26693	4.27E-05	2.42628741
)33400F21R	6.38E-06	2.42421263
AU021092	1.24E-41	2.42244372
Rps15a	1.01E-168	2.42067548
Hist1h4c	0.017447	2.41961653
Sec61g	1.06E-74	2.41909829
Gm42620	0.001034	2.41884295
Gm43378	0.017379	2.41873515
AC163685.1	0.046158	2.41780952
Tmem91	0.023127	2.41777491
Paqr4	2.25E-06	2.41523922
Isg20	6.17E-08	2.41495029
Praf2	0.000393	2.41372158
Ctla2a	5.42E-26	2.40941271
Gm13594	0.023127	2.40879511
Cldn3	3.00E-30	2.40506739
Klk8	0.000154	2.40408369
Rundc3b	1.50E-54	2.40363106
Tent5d	8.89E-08	2.40293129
Msantd1	0.016432	2.40078041
Gm10399	0.0019	2.40065194
Gm16048	0.000255	2.39808182
Gm32184	0.046133	2.39762638
Ccdc152	4.89E-05	2.39617237
Rpl31	3.98E-76	2.39255175
Gm10974	3.16E-09	2.38940889
)30001D20I	3.07E-08	2.38581463
Gm42556	0.033434	2.38081252
Gm42031	0.032617	2.38076587
Npw	1.43E-08	2.38073347
Gm49915	0.000411	2.37776797
Hddc3	0.04627	2.37736883
Wdr38	7.63E-06	2.37709376
32422M17I	0.000209	2.37564379

Yif1a	2.39E-09	2.3736486
Krt19	6.42E-50	2.37273796
Htr2b	0.000556	2.37238629
Smim6	2.72E-19	2.37214197
700094J05R	0.012488	2.3711419
Tmem179b	3.67E-17	2.3699563
Hyal5	0.032635	2.36726791
Sfta2	4.61E-44	2.36701663
30473A02F	4.66E-05	2.36314758
Clec2g	0.00011	2.35796534
Bcas3os1	5.23E-10	2.35570554
00109K24F	1.39E-08	2.3541586
Gpihbp1	8.90E-19	2.35313262
Aprt	1.08E-45	2.35119255
Enho	0.000556	2.35004389
Wdr17	1.22E-51	2.3479876
Uckl1os	0.00191	2.33984586
Mrpl52	4.16E-52	2.33979056
Gm13856	0.032671	2.33915059
Gm17057	0.000683	2.33908844
mt-Co1	0	2.3356291
Gm15050	0.032671	2.33433423
Gm47827	0.009124	2.33327087
Gm16189	5.26E-06	2.33224069
Gm16751	0.003452	2.33221096
Gm16337	8.03E-11	2.33126521
Rpl10a	5.28E-111	2.33005055
Eno1	2.41E-12	2.32709731
Mrpl54	7.42E-19	2.31507718
Uqcc2	4.13E-21	2.31266638
30506C21F	0.001913	2.31216968
Gm13362	0.032653	2.30788953
Aloxe3	0.032653	2.30556419
Rps17	1.37E-116	2.30482459
Gm36198	1.32E-09	2.30361774
Gm26672	8.46E-16	2.30231399
Ubl5	8.28E-60	2.30191971
Znrd2	1.41E-08	2.30140246
Gm17135	0.000764	2.30091858
Mhrt	0.008318	2.29876067
Fam177a	4.74E-08	2.29777079
Cpsf4l	0.003446	2.29752142
230066G23F	0.046183	2.29570348
Gm30524	1.99E-05	2.29552948
10017D15F	7.78E-27	2.29467997
Arhgap33	0.012472	2.2938293
010003L21F	0.012665	2.2935246
Mgst3	2.00E-13	2.2873921
Serping1	1.78E-51	2.28730676
Gm15706	0.003026	2.28689736
Gm11906	0.000218	2.28649319
Gm13546	0.0242	2.2859506
Ndufa1	1.27E-42	2.28580991
Ptrhd1	5.47E-07	2.28568757
Hyal1	1.79E-06	2.28242058
S100a11	2.42E-180	2.2808116
Angptl7	2.53E-07	2.27625445
Gm17106	2.27E-21	2.27603103

Gpr18	4.65E-05	2.27398965
Gm28376	0.004897	2.2666369
Hist1h3e	0.046209	2.26629118
Gm15441	0.024211	2.26626081
Rnf26	0.002481	2.26567351
30595D18F	1.70E-06	2.26566151
Gm14508	5.81E-06	2.26514235
Tekt4	4.35E-06	2.26092227
Gm37768	6.26E-11	2.24990531
Aplp1	5.84E-08	2.24816056
Stbd1	2.95E-07	2.24734174
10012G03F	1.64E-15	2.24480689
Atpif1	1.86E-93	2.24457655
Fgf18	0.001018	2.24268276
Rpa2	4.94E-05	2.24214414
Gm13091	0.000562	2.24196006
Hspb2	3.07E-05	2.24190091
Fbxl12os	3.59E-05	2.24129453
Gm17036	4.82E-05	2.24004939
Cd68	1.07E-33	2.23694938
Tmem258	6.34E-28	2.23620522
Atp5d	1.15E-31	2.23491955
31428L18F	0.032671	2.23377661
AC163638.2	3.43E-05	2.22988492
Ndufa5	5.33E-31	2.22605861
Xlr	5.92E-06	2.22465449
Rpl36	4.36E-139	2.22441094
Gm48678	2.08E-58	2.22314054
Mir133a-1hg	1.23E-07	2.22051956
Gm39326	4.91E-05	2.21919255
Dydc2	0.010702	2.21887744
Chrd	0.003461	2.21863627
Myl9	0.000746	2.21303688
Tstd1	9.12E-10	2.21215092
Uqcr10	1.18E-49	2.21154736
Churc1	6.18E-14	2.21058523
Fmc1	2.23E-10	2.20780966
Gm15883	2.52E-20	2.20505221
Cox8a	9.13E-103	2.2049295
Folr1	0.009195	2.20360492
Rpl12	6.88E-105	2.20349642
Tmcc3os	0.003509	2.20219278
Car11	0.016432	2.20019819
Prdx5	2.01E-59	2.19976842
Trappc1	6.61E-08	2.19655604
Rabac1	1.36E-72	2.19425085
Ssna1	7.33E-10	2.19179595
Rps27l	2.53E-69	2.19129132
Rpp21	8.81E-17	2.19087458
Gm45767	0.001036	2.19029672
530036H23F	0.004781	2.19022477
30455C13F	1.35E-09	2.18992275
Cox7c	3.44E-123	2.18891307
Dnajc4	1.07E-05	2.18733079
Cox6c	1.22E-128	2.18505801
Mrps34	5.19E-11	2.1831073
Apobec2	0.032689	2.17984588
Uqcrb	1.64E-61	2.17942421

Ccl27a	0.000742	2.17829073
Wfdc21	4.97E-43	2.17778822
Tmem160	1.34E-33	2.17541693
H2afj	7.41E-63	2.17351545
'30085K08F	0.002499	2.16979896
31422A03F	1.34E-06	2.16774143
Car14	4.74E-11	2.16653854
Nsa2	3.11E-40	2.16638787
Gpr153	0.033513	2.16631913
Gm47096	0.004902	2.16551179
Gm16573	0.000561	2.16235863
Gm9902	0.005931	2.16231342
Scgb3a2	0	2.16220485
Rspo1	2.77E-19	2.1611595
Mrpl34	6.53E-16	2.16055865
Atp5h	1.17E-93	2.15954435
Cavin3	4.73E-17	2.15937573
Gm43221	0.00495	2.15936371
Rpl19	2.16E-232	2.15183266
Rpl41	0	2.15093814
Eif3g	1.40E-14	2.15059387
Apobr	4.04E-07	2.14954816
BC051226	0.000412	2.14759976
Gm28375	2.32E-13	2.14702119
Mfap5	2.73E-08	2.14623495
Orc1	0.000212	2.14582106
Dancr	5.45E-07	2.14120991
Dalrd3	8.48E-17	2.14111251
Atp5e	3.29E-94	2.13857363
10030D12F	0.017532	2.13670088
Ndufb4	7.52E-35	2.13222961
Fbxo6	4.45E-05	2.12677038
Zfp85	0.011675	2.12665463
Gpr22	0.008727	2.12482709
Apex1	7.99E-06	2.12308982
Tmem139	0.001025	2.12233352
Ndufb10	7.68E-41	2.12165074
Mrpl53	1.15E-14	2.11840676
D8Ertd738e	9.38E-34	2.11770442
Lrrc18	0.006716	2.11407991
Gm43660	0.012696	2.11354424
Calr3	0.032671	2.11192841
Gm10389	0.006729	2.11157952
Cd300c2	7.44E-13	2.10986943
Cnmd	0.046209	2.10783085
Hist2h2ac	0.008318	2.10666323
Tmsb15b2	0.031854	2.1052327
Gm42726	0.000559	2.10521645
10013P06F	1.51E-11	2.10450404
Oprm1	0.012528	2.10169711
Tmsb4x	0	2.10033183
Gm12709	0.006619	2.09878408
Gm14295	0.033609	2.09860575
Gspt2	0.003514	2.09748795
33417C18F	0.046259	2.0974832
'00084C06F	1.59E-19	2.09628567
Imp3	1.08E-19	2.09614025
Ndufs8	2.53E-10	2.09498235

Ppcs	1.45E-05	2.09256189
Thap7	3.00E-07	2.09221237
Fbxo44	0.043005	2.09173432
Mrps14	7.96E-21	2.08964553
Ccnq	0.00186	2.08712859
Pam16	1.22E-09	2.08681858
Aqp1	6.93E-137	2.08607253
Mrps21	1.11E-28	2.08447672
Slc6a8	8.98E-05	2.0844177
Tff2	7.74E-40	2.08324012
Rpp38	0.000966	2.08184802
Slc6a4	6.01E-06	2.08130599
Ifitm3	3.00E-81	2.07959412
Gm31683	0.001049	2.07904252
Hey2	0.000769	2.07866366
Zcchc9	7.64E-15	2.0726026
Snrpg	8.25E-41	2.07199094
Cytl1	3.35E-08	2.07032881
Rex1bd	2.50E-22	2.07029965
Proca1	0.008719	2.07014763
Gm47350	7.10E-07	2.06776153
Yif1b	9.36E-08	2.06707891
Serf2	2.65E-113	2.06593754
Stmn3	0.032671	2.06533507
Ahnak2	3.50E-17	2.06281114
Tpm2	0.000745	2.06277799
Rpl22	1.15E-144	2.05933007
Gmpr	3.82E-10	2.05640865
Lamtor2	4.22E-39	2.05244542
S100a10	9.54E-84	2.05013644
Gm15910	1.96E-05	2.04904966
Eif3k	4.75E-31	2.04800718
Myh6	0.033609	2.04455598
Etnk2	4.58E-06	2.04337472
Krtcap2	2.86E-29	2.04059783
Zfp61	0.001404	2.03948646
Psme1	4.10E-54	2.03518169
Tbcc	8.68E-08	2.03400432
Lyz2	0	2.03306052
Cryab	7.44E-35	2.03255926
Mrps16	2.91E-13	2.02771366
Resp18	0.006632	2.02641325
Gm2087	2.65E-05	2.02612798
Rps6	1.74E-108	2.02556007
Rpl17	3.67E-164	2.02510007
Gm20663	0.000416	2.02401022
Cd248	8.86E-05	2.02304453
Ccdc166	0.016422	2.02231916
Gm39214	1.47E-05	2.02188068
Gm31243	5.03E-05	2.02182785
Tulp2	1.97E-05	2.0164229
Rps5	6.17E-139	2.01638895
Supt4a	1.07E-16	2.01635454
Wfdc1	1.39E-12	2.01471275
Sox7	8.78E-16	2.01363816
Tmem42	2.04E-05	2.01317919
Siva1	7.07E-15	2.01231131
Gfra4	0.046234	2.01207581

Mcee	7.29E-17	2.01079314
Rpusd4	2.66E-05	2.00983432
10046G10F	0.006491	2.00945914
Gins2	0.000744	2.0086146
Gabrr2	0.033625	2.00761887
Tent5b	0.008779	2.00692297
Hint1	9.10E-79	2.00449055
Rps28	8.32E-274	2.00422077
Ciao2b	3.15E-06	2.0027142
00018L02F	0.009176	2.00192457
Gm47969	0.032671	2.00132291
Rps3a1	5.20E-257	2.0009622
Polr3h	1.02E-08	2.00027144
Carmn	0.000416	1.99814273
Scgb1a1	0	1.99739445
U2af1l4	0.000243	1.99601074
Fau	1.78E-281	1.99529873
Ccdc153	2.66E-41	1.99328493
Tgif2	0.008078	1.99321544
Smim8	1.22E-10	1.99243491
Mrpl40	4.98E-10	1.9916624
Gps2	9.32E-21	1.99121711
Slpi	1.31E-78	1.98935996
Dipk1b	0.011845	1.98924937
00012B09F	0.003351	1.98883279
Gm20470	4.19E-06	1.98818043
Dda1	1.32E-07	1.98680588
Snrnp27	8.54E-23	1.98610189
Ica1l	1.93E-05	1.98446381
AI463229	0.006626	1.98357258
Rps4x	1.82E-161	1.98343034
Rab1b	3.25E-12	1.98215973
Zbtb11os1	0.046422	1.98203928
Hspb1	2.99E-36	1.98133649
Chmp2a	8.13E-33	1.97711461
32443L11F	0.0025	1.97413644
Nfkbib	1.00E-06	1.9728002
Gm42917	0.004134	1.96992073
Spsb2	2.36E-05	1.96904243
00015L24F	0.000763	1.96802939
Gm4651	0.005931	1.96254667
Gm16984	0.004765	1.96223034
Egfl8	0.01255	1.96124359
00113A16F	0.001847	1.96035027
Mypop	0.002325	1.95959824
C4b	1.96E-84	1.9591398
Gm20219	0.031786	1.95826593
Rps16	1.54E-208	1.9546434
Serf1	4.81E-07	1.95390788
Lst1	1.28E-05	1.95344342
10039H08F	2.82E-07	1.95119563
Mrpl27	2.66E-09	1.95065912
Id3	2.01E-36	1.95031712
Ccdc102a	2.76E-05	1.94997371
Gm4356	0.009135	1.94899538
Rps27a	4.60E-281	1.94848659
Sphk1	0.006081	1.94847447
Rpl24	1.35E-165	1.94518293

Cox7a2	6.08E-54	1.9431199
Brd9	4.59E-28	1.94084229
Gcnt7	5.47E-11	1.93958737
Tmem223	2.54E-15	1.93918644
Gm47271	0.004537	1.93902452
Rpl35a	1.75E-265	1.93882502
Zc3hav1l	0.00251	1.93589484
Gm16552	5.63E-07	1.93555449
Gm2000	1.10E-05	1.93529549
Gm4189	0.017128	1.93522835
Gm15402	0.00057	1.93304883
Commd4	3.51E-07	1.93278747
Rbp1	2.78E-19	1.93245468
Acot2	5.59E-05	1.92860529
Klhdc8a	0.016432	1.92822153
Selenoh	1.98E-12	1.92739024
Sfrp1	9.78E-13	1.92710009
Eif1	4.10E-211	1.92705915
Rpl23a	5.07E-113	1.92662961
Erf	3.88E-09	1.92490774
30408C22R	0.000114	1.92419486
Vegfb	3.10E-07	1.92400258
Gm17146	0.033593	1.92320005
Surf2	6.64E-05	1.92174256
510017J16R	0.021422	1.92121325
10009K17R	0.000259	1.92049555
Skida1	1.89E-06	1.92007278
Lamb2	1.47E-20	1.91930617
Crip1	0	1.91809684
Cd81	2.03E-63	1.91583933
Fcgr1	1.48E-05	1.91502963
Bet1l	5.69E-13	1.91502631
B3galt4	0.012562	1.91074352
Tlr6	0.008318	1.91058165
Sap18	5.30E-17	1.91049326
Dusp23	1.32E-08	1.90850153
Grip1os3	0.046259	1.90410624
Scpep1os	0.023153	1.9031669
Appbp2os	0.006527	1.90205525
Erich5	0.032671	1.90046718
Tmem198b	0.000689	1.89837033
Wdr24	0.004528	1.89776318
Gm43569	0.001925	1.89774532
Cd59a	0.000732	1.8973175
Mea1	9.03E-08	1.894763
Gm10076	8.05E-81	1.89409189
Gm10800	0.014506	1.8937816
Rpl4	3.29E-62	1.89254453
Calca	0.038486	1.88960527
Gm48914	0.002652	1.88634922
Ndufaf3	2.59E-07	1.88629653
Dchs1	0.014478	1.88254334
Vmac	8.87E-05	1.88162092
Gm49961	0.00099	1.88120735
Gm14167	0.000306	1.87869842
00004A13R	1.05E-13	1.87532256
Zfp773	0.031731	1.87426323
Aebp1	4.19E-43	1.87309524

Rps24	6.68E-284	1.87198017
Lrrc19	0.004806	1.87066244
Ndn	6.72E-08	1.87003685
Coro6	0.000312	1.86936056
Mroh5	0.032689	1.86661087
Ddx18	4.77E-18	1.86543409
Sap30	0.000153	1.86508555
Tomm5	9.83E-17	1.86412955
Wnt2b	0.004897	1.86217112
Gm50368	0.000114	1.86188528
Col18a1	0.001625	1.86138702
Polr2i	1.32E-15	1.86025134
Txn1	5.06E-85	1.85923001
Fabp3	0.009225	1.85819001
Ramp2	2.40E-42	1.85795755
Gm13571	0.003619	1.85769717
Tomm7	1.98E-55	1.85612884
mt-Nd1	5.69E-73	1.85601634
Dctpp1	0.006076	1.85469595
Gm28410	3.48E-05	1.85262858
Tmem115	0.000204	1.85102369
Rpl3	4.00E-112	1.84805058
Armcx4	0.001321	1.84182638
Pltp	1.07E-70	1.8392098
Gdf2	0.023364	1.83899491
Atp5k	2.45E-59	1.8384718
Fam57b	0.021452	1.83791992
Borcs8	6.62E-07	1.83578095
Tmem234	4.36E-38	1.83283537
Naxe	2.41E-16	1.83256496
Cst3	9.35E-162	1.83028974
Ang	1.78E-11	1.83008064
Ip6k3	0.000408	1.83005175
Sac3d1	0.000305	1.82958324
Ccne2	6.71E-05	1.82906544
Nap1l3	0.043132	1.82627051
Gm47423	0.021487	1.82271215
Smdt1	4.41E-48	1.8219935
Tax1bp3	9.30E-12	1.82197227
Chchd10	1.35E-69	1.8214029
Rnasek	1.36E-32	1.8205844
Hcfc1r1	3.52E-24	1.81883307
Cdkn1c	6.09E-06	1.81873579
Timm10b	2.70E-27	1.81831325
30550D23F	0.007323	1.81776405
Degs2	0.003392	1.81679638
Tmem238	3.82E-12	1.81600113
Atp5j2	1.45E-53	1.81559023
Cox4i1	8.63E-134	1.81549713
Ndufb1-ps	3.37E-74	1.81479853
Rpl5	3.20E-116	1.81266795
Tmem79	0.021496	1.81212282
Gadd45gip1	5.82E-12	1.80960522
Hsd17b10	3.49E-08	1.80903484
Ndufb2	3.06E-23	1.807928
Mt1	1.04E-84	1.80769349
Rgp1	1.60E-07	1.80293662
Atox1	4.46E-40	1.80220556

30309D02F	2.40E-06	1.80202873
230083G16F	0.004934	1.80080152
Gm36756	5.02E-05	1.80067811
Psmc3ip	0.019276	1.79980437
Nedd8	2.46E-43	1.79834884
Rpl36a-ps1	0.033689	1.79804534
Prcd	0.017204	1.797102
Lrpap1	3.27E-22	1.79409475
Gm13056	0.000215	1.79336677
Gm4793	2.32E-05	1.79248508
Fpr1	8.35E-14	1.79179633
Fam173a	3.04E-15	1.79134674
10009B22F	2.04E-05	1.79123032
Ucp2	2.72E-60	1.78623839
Pim3	2.21E-12	1.78594609
Dynlt1c	0.012776	1.78551265
Mmp11	0.001121	1.78388795
Ucp3	4.52E-06	1.78372338
Gm15345	6.50E-05	1.78353123
Ndufa6	3.59E-38	1.78336062
Rpl27a	7.37E-202	1.78309799
Igf2	0.046259	1.78278234
Btbd6	0.014433	1.78215018
Akr1b8	3.61E-06	1.77687099
Gm36738	7.11E-07	1.77679756
Calml4	4.98E-13	1.77465009
Apcdd1	0.023369	1.77350879
Plb1	0.006096	1.7728154
Map1lc3b	1.86E-46	1.77208377
Elof1	3.34E-31	1.77201364
Cd151	1.16E-19	1.77127171
Ppib	6.59E-31	1.77046182
10038B12F	0.005739	1.77008624
Iscu	2.55E-19	1.76979898
Rec114	4.87E-05	1.769103
Iffo1	2.57E-13	1.76907645
Rplp0	5.66E-110	1.76852444
Dad1	2.37E-40	1.76491503
Sbsn	1.90E-22	1.76470872
Rab34	0.000305	1.76424815
Tspo	1.32E-49	1.76364139
10009A05F	7.85E-10	1.76306559
Mdk	0.006098	1.76185873
310405F17R	1.88E-06	1.76116477
Slc16a4	2.14E-06	1.75990927
Hsbp1	7.03E-30	1.75891811
Abhd8	0.001044	1.75884226
Pebp1	2.07E-43	1.75689149
Gsdme	2.41E-09	1.75638615
Car3	0.000121	1.75634963
Gm45051	0.006639	1.75628678
Hspe1	7.64E-32	1.75620639
10027K06F	1.75E-05	1.75593631
10012L19F	0.000125	1.75513311
Aurkaip1	3.50E-15	1.754426
Gm27017	4.81E-05	1.75318028
Coa3	1.74E-15	1.75313676
310010J17R	2.08E-05	1.7510651

Gm15891	0.033625	1.75081574
Ushbp1	5.64E-32	1.74604866
Sh3bgrl3	4.84E-53	1.7453958
Mrpl49	2.49E-05	1.74537035
Ppp1r14b	6.60E-24	1.74531058
Sptbn5	0.024375	1.74511695
310033P09R	3.11E-09	1.74280738
Insyn2b	6.96E-08	1.74213249
Fcer1g	1.18E-53	1.7419556
Ankrd61	0.00882	1.73988124
700016K19R	6.36E-16	1.73978282
Abhd17a	5.15E-14	1.7396955
Fndc5	0.046209	1.73954028
Gm12758	2.08E-05	1.73883342
10009B15R	2.34E-06	1.7347202
Pfdn5	2.94E-72	1.73464886
Sdhaf1	0.000194	1.73452387
Clu	1.22E-57	1.73351498
921511I17R	0.028896	1.73345183
Gm49066	1.65E-06	1.73166096
Tnni2	0.024393	1.73148428
10040G24R	0.028862	1.73036909
Rassf7	6.40E-06	1.72993797
Mrpl18	1.81E-13	1.72954333
Atg16l2	8.04E-21	1.7294393
Spata2l	0.046574	1.72890669
Fibin	8.47E-11	1.72620278
Gm28198	3.30E-06	1.72502616
Gal	9.11E-05	1.723103
Gm13449	0.000208	1.72253262
Tmem151a	7.78E-10	1.72231068
Gm16141	0.002646	1.72222353
Cox4i2	0.000845	1.72161503
Hist1h2ae	0.01192	1.72118116
Best1	0.019305	1.72064684
Gm11084	0.031909	1.71918591
Nt5c	1.27E-11	1.71778617
Gpatch11	1.57E-08	1.71670033
Atp5o	1.27E-36	1.71545433
Gm5914	0.046259	1.71530381
730034F03R	0.003143	1.71489804
Higd1b	0.000293	1.71139668
Ficd	0.006547	1.71131223
Ptcd1	2.77E-05	1.70951963
Trappc6a	2.90E-09	1.7089524
Mrps33	1.73E-24	1.70865998
Pcolce	5.66E-20	1.70833103
Ndufv3	7.49E-22	1.70725892
Frmd3	0.008883	1.70447748
Ooep	0.046487	1.70368254
Mrpl36	1.64E-11	1.70282828
Ift27	8.11E-06	1.70155775
330026I12R	1.49E-09	1.70126321
P2ry13	0.003377	1.70102445
Gm43672	0.012613	1.70096354
Bgn	7.51E-67	1.69808489
Tnfrsf1a	1.32E-18	1.69668185
Selenok	2.58E-36	1.6960897

Etfb	4.32E-35	1.69549681
33438C02F	0.001927	1.69541966
Gm34868	0.001894	1.69441921
Gm31323	5.88E-05	1.69422888
Cd3d	0.009774	1.69293243
Ndufv1	7.14E-09	1.69038539
Rps21	2.04E-223	1.6878258
Polr2f	4.27E-16	1.68728101
Rinl	1.13E-08	1.68699952
Cox6b2	7.55E-08	1.68659463
Enpp6	0.000856	1.68651985
Usp50	4.19E-25	1.68644237
Gm49380	0.032689	1.68613664
Dbi	1.98E-67	1.68529236
Fam122a	2.13E-06	1.6842779
Rpl38	5.81E-227	1.68333177
Flywch2	0.024305	1.68214526
Ndufc2	4.15E-42	1.68212646
Nkiras2	7.80E-08	1.68203873
30053A07F	0.003366	1.68200929
Nkapl	0.00612	1.68057186
Gm49189	0.000389	1.68055418
Gm28609	0.00262	1.68050691
Ccdc96	0.001764	1.67792432
Coa6	8.02E-06	1.67775154
Atp13a2	8.55E-16	1.6776337
Ppp1r14a	4.24E-06	1.67703617
Psmb5	1.21E-20	1.6769392
Acp5	6.71E-12	1.67688957
Rps8	9.63E-275	1.67170908
Bag5	2.04E-07	1.66968974
00035N22F	0.00057	1.66744271
Sf3b5	9.85E-20	1.66700557
Sowahb	0.012727	1.66662455
Trip6	0.000186	1.66459475
Fgfbp1	0.005584	1.66246675
Pafah1b3	8.47E-07	1.66193527
Pet100	1.27E-25	1.66081274
Unc45bos	0.003395	1.66060863
Lrrc55	0.033844	1.66023762
Svbp	8.06E-12	1.66004767
310061I04R	1.74E-05	1.65854119
930012L18F	0.008587	1.6583301
Ddx24	1.07E-27	1.65405828
Gm36371	0.046574	1.65184735
Bola2	6.52E-25	1.6507001
Tmem100	6.25E-72	1.65060605
Impdh2	2.70E-08	1.6505602
00056N10F	0.038726	1.6504687
Isyna1	4.51E-05	1.64965965
Gtf3c6	1.22E-10	1.6473966
Ncr1	0.046259	1.64404225
Gm15396	0.003115	1.64311578
Grin2c	0.033641	1.64283354
Pusl1	0.021444	1.64277737
Phlda3	8.40E-07	1.64245341
Mpst	0.000256	1.64242616
Smim26	1.26E-06	1.64204544

Tril	0.00655	1.64154266
Cdc42ep5	1.72E-06	1.6403688
Fam183b	7.23E-24	1.64016417
Trp53rkb	0.001954	1.63803766
Hilpda	7.66E-05	1.63748977
Micos13	5.14E-19	1.63746143
700012I11R	0.011379	1.63489275
Gm17354	0.001114	1.63392579
Snai2	0.022451	1.63319518
Ndufb8	2.43E-30	1.63243553
Ankrd1	0.000571	1.63208941
Ptprr	3.16E-05	1.63159924
Igfbp6	5.35E-128	1.63129403
Adh1	9.34E-08	1.63051605
Inf2	1.73E-15	1.62912751
Ackr4	0.000851	1.62897684
Clic3	9.76E-17	1.62886539
Uqcc3	1.99E-14	1.62869961
Oxa1l	7.57E-12	1.62267811
Kti12	0.000488	1.62267336
Gm15991	3.49E-06	1.62117995
Tceal8	3.85E-08	1.62088827
Gm28523	0.029103	1.62005258
Dok1	5.83E-05	1.61880306
Nnmt	1.02E-06	1.61856525
Chd5	0.001639	1.61856135
Rpl27	5.38E-109	1.61855838
Prpf19	1.19E-07	1.61789685
Znhit2	0.000451	1.61661746
530402F18R	0.021461	1.61571651
Plcd1	0.000232	1.61531799
Gm9917	2.60E-07	1.61520415
Rpl14	3.58E-78	1.61485389
Epop	0.01269	1.61479326
Gm15401	0.033641	1.61397656
Gm15283	4.54E-10	1.61356855
Naalad2	0.000313	1.61215385
S100b	0.011925	1.61190754
00002D01R	4.60E-06	1.61106131
mt-Co2	7.86E-156	1.61104804
Gm17494	0.00017	1.61097685
Gm48696	0.009767	1.6099471
Angptl4	9.26E-05	1.60964784
Lyrm9	0.002171	1.60727244
Gm11465	0.006782	1.60665971
Plet1os	9.63E-08	1.60584327
Gm17090	0.003627	1.60571582
Iglc1	3.01E-05	1.60311791
Pfn2	0.000164	1.60239475
Zfand2a	4.55E-07	1.60141967
Pop5	1.07E-09	1.60105139
Pcolce2	3.79E-10	1.60095956
Tmem221	0.046487	1.60064794
Gm15587	0.00578	1.60055675
Smim11	2.21E-16	1.59889843
Gm43573	0.043404	1.59694419
Hspa1a	4.55E-07	1.59619295
Rps14	9.00E-193	1.59609387

10065P20R	6.32E-05	1.59589556
Qpctl	2.57E-05	1.59585605
Slc48a1	1.29E-15	1.59470787
Tubb4b	1.55E-50	1.59438464
Gm10851	0.012983	1.59377457
Eva1b	3.56E-10	1.58961627
Ly6c1	2.71E-23	1.58953153
S100a6	1.97E-208	1.58632907
Slc52a3	0.000209	1.58608053
Zfp300	0.046574	1.58508967
Emp3	7.72E-27	1.58495636
Gamt	0.033993	1.58452858
mt-Nd3	2.85E-30	1.58336902
Arpc1b	9.97E-59	1.5814795
Gm12359	0.011436	1.58134955
Epha1	0.025838	1.58092728
Tmem35b	0.00388	1.58066561
Comtd1	2.66E-05	1.57989615
Dynll1	4.97E-76	1.57888893
Fam227b	0.017609	1.57855338
Use1	6.68E-18	1.57800995
Tmem14a	0.007552	1.57797842
Pigyl	4.84E-12	1.57621727
Plekho1	1.73E-18	1.57506355
Flacc1	0.046465	1.57377876
Gm12592	1.34E-07	1.5735535
Armcx2	0.000278	1.57295404
Tmsb15b1	0.009199	1.57275546
Cebpzos	2.40E-06	1.57249347
Kazald1	2.60E-06	1.57074754
Slc9a3r2	8.65E-49	1.56999948
Mrpl4	1.74E-09	1.56992444
10006H16R	4.48E-14	1.56744925
Gm11747	0.004802	1.5666311
Mfsd4a	4.48E-18	1.56569488
Oas1c	0.003186	1.56517912
Gm27201	1.60E-05	1.56306631
Zbtb12	0.021626	1.56272976
Naa10	5.62E-06	1.56218937
Rpl37a	3.95E-256	1.56030226
30408M09R	0.046259	1.55958627
Gng11	1.20E-19	1.55894563
Pkd2l2	0.001973	1.55775911
Rcn3	9.86E-08	1.55398599
10009E02R	0.001228	1.55358985
30001M01R	0.002403	1.55277388
Gm10550	2.37E-05	1.55233265
Znhit3	0.00288	1.55178985
Emilin1	2.56E-05	1.55063805
Fndc9	0.021583	1.55061362
Gm20404	1.78E-08	1.54952535
Crym	0.000322	1.54933308
Ifi27	6.06E-18	1.54747633
Arsa	4.93E-05	1.54694598
Ccdc85b	3.05E-17	1.54671105
Slirp	4.70E-11	1.54527643
30001N04R	0.00653	1.54444832
Lynx1	2.76E-06	1.54444162

Dolk	0.001554	1.5441189
Cand2	0.046259	1.54311864
Gm38134	1.82E-07	1.54270405
10011C24R	0.01725	1.5421622
Ppdpf	1.12E-12	1.54084665
30048N14R	3.39E-05	1.53970893
Trim47	5.80E-11	1.53868853
Nthl1	7.13E-05	1.53760562
Gm14798	1.13E-10	1.53750809
Vps28	1.11E-14	1.53556823
Dctn3	3.81E-14	1.53438746
Pole3	1.34E-05	1.53341049
BC029722	1.29E-05	1.53334359
Rbm42	1.46E-13	1.53305877
Lmo1	0.009292	1.53232153
Rpl22l1	8.93E-36	1.53181388
Dnah17	0.043404	1.53100427
Mrpl16	2.96E-10	1.53086601
Higd2a	9.23E-15	1.52766069
Gm33370	0.008568	1.52572707
Ppp1r11	6.31E-17	1.52435985
Gm4673	0.000482	1.52412695
Pcif1	2.16E-11	1.52410458
Rbis	3.29E-12	1.52344945
Plcxd1	0.004195	1.52006272
Mrpl23	2.47E-18	1.51994603
Lsm3	2.85E-05	1.51898891
Cox7b	6.20E-39	1.51826167
Cbr1	4.27E-08	1.51803765
Bdkrb2	0.021574	1.51720164
9-Mar	0.038892	1.51695447
Dnajc19	2.16E-23	1.5159368
Pdcd5	8.86E-25	1.51485008
Gm11290	5.83E-08	1.51325901
Lrrn4	1.18E-26	1.51319937
Efcab10	1.37E-08	1.51302644
Ufc1	3.22E-15	1.51212673
Kif26a	1.00E-06	1.51134556
33423P22R	0.001036	1.51070569
Rac3	6.60E-06	1.51068305
Arl6ip4	3.10E-12	1.51017187
Tlcd2	0.029433	1.50754008
Fam50a	9.15E-11	1.50738627
Vash1	0.029352	1.50638342
Eef1b2	9.43E-57	1.50595366
Mrps18c	3.66E-14	1.50529654
00024G13R	1.45E-08	1.50519226
Rsph1	4.41E-15	1.50427372
510507I01R	0.004428	1.50335951
Mbd3	3.45E-10	1.50309699
Tuba1a	4.31E-40	1.50230253
Tgfb1i1	2.44E-08	1.50188546
Rfc5	0.007477	1.50022443
Meox1	0.004865	1.50015908
30077J02R	7.03E-05	1.4992789
Siglecf	6.46E-10	1.49893516
Gm17251	0.009292	1.49810645
Pla2g1b	9.38E-07	1.49770571

Ppia	8.80E-106	1.49579887
Cxcl12	1.80E-32	1.49416216
Mars	0.000113	1.49399249
Eif4ebp1	7.05E-06	1.49036939
Aup1	1.81E-13	1.48979015
Msln	5.96E-69	1.48937336
Brsk1	0.043313	1.48892003
Eif2b1	0.000822	1.48888742
Rbm19	4.50E-05	1.48860377
Rpl7a	1.60E-64	1.48637935
Stoml1	1.12E-05	1.48635216
Eif2s2	4.33E-46	1.48628811
She	2.17E-15	1.48412548
Gm21057	6.28E-05	1.48298952
Mrpl58	1.98E-07	1.48266385
Snhg18	2.56E-11	1.4824766
Crlf2	2.28E-07	1.48216571
S100a13	1.65E-29	1.48200766
Epb41l4aos	6.80E-06	1.48132785
Pdrg1	2.03E-06	1.48000299
Bex2	1.26E-05	1.4778563
190007I07R	0.002975	1.47662421
Romo1	8.18E-39	1.47531375
Zfp335os	7.30E-05	1.47332117
Hspa8	2.03E-135	1.47323455
Polr2l	3.99E-08	1.47312243
30469K13F	0.006561	1.47206208
Ddx41	2.38E-07	1.47078066
Gm26760	0.009863	1.46881922
Surf1	1.39E-05	1.46779825
Copz2	5.83E-07	1.46765562
Mrpl20	3.73E-17	1.46757495
Cdc42ep1	7.01E-23	1.46674418
30020D12F	5.89E-11	1.46546617
Gm15478	6.06E-17	1.46539699
Hmg20b	1.74E-15	1.46457583
Rfesd	0.000529	1.46445686
Ccdc73	2.17E-05	1.46400459
Tceal1	0.019378	1.46392081
Sharpin	1.75E-05	1.46335333
Gm47689	0.033673	1.46174272
00093K20F	1.06E-05	1.46168269
Sgtb	0.000792	1.46079609
Tma7	2.11E-31	1.46016221
Hscb	6.21E-07	1.45995379
4-Sep	8.68E-19	1.45916454
Cox6a2	0.000557	1.45821482
Scgb3a1	0	1.45659551
Rsf1os2	0.000537	1.45562808
AI480526	1.25E-07	1.45497463
30043A13F	0.006622	1.45334824
Ptn	0.000651	1.45310249
Triap1	6.97E-05	1.45252027
Taf10	5.61E-16	1.45251075
Slc35a4	2.08E-07	1.45199021
Nit2	0.000492	1.45099523
Gm10138	0.007415	1.45035241
Ndufa2	1.62E-46	1.44929052

Stk32a	0.000413	1.44908231
Gm26981	0.01605	1.44881269
Smpd2	0.000226	1.44827064
Perp	1.15E-07	1.44791879
Gm14964	0.002399	1.44756075
Uqcr11	6.27E-56	1.44498347
Gm4013	0.004854	1.44399358
Reg3g	8.90E-100	1.44395806
Abracl	1.79E-25	1.4438958
Knop1	5.50E-13	1.44324107
Ndufs7	5.55E-16	1.44306282
Sec61b	1.35E-38	1.44262025
Hoxaas2	0.034074	1.44215025
Stk11ip	0.003821	1.44211545
B3gnt3	0.002247	1.44168593
Scand1	7.96E-33	1.44141912
Rpl36al	1.40E-73	1.4405433
BC028528	1.33E-28	1.44020746
Gm48882	0.038677	1.43895099
Gm43113	0.000489	1.4362132
Des	0.014579	1.43530273
Trim46	0.003891	1.43382693
Ndufa7	1.23E-37	1.43274712
Las1l	9.57E-11	1.43164722
Tbrg1	4.05E-15	1.42837155
Retsat	3.77E-05	1.42734043
Gm26532	6.62E-14	1.42637276
Adap2os	0.000217	1.42510744
310058I24R	1.08E-13	1.42491915
Rac2	5.46E-07	1.42395823
Dbp	8.82E-25	1.42283049
Zfp105	0.005037	1.4221842
Gm43331	0.000223	1.42161978
Man2b1	4.66E-23	1.4211791
Ackr2	9.12E-06	1.42100146
Ltc4s	4.96E-23	1.4208349
Sft2d3	2.19E-06	1.42049945
H2afz	1.76E-63	1.42038681
Gm48512	3.80E-05	1.42024762
Necap2	2.56E-10	1.42009763
Trim65	2.55E-07	1.4194326
Zdhhc12	0.001582	1.4166298
Prelp	4.71E-15	1.41573893
Ndufs5	2.47E-32	1.41402154
Upk3b	5.04E-60	1.41168697
Prdx4	6.95E-09	1.41079594
Nol7	2.40E-24	1.41055835
Lamtor4	5.17E-18	1.40991843
Tfip11	0.000529	1.40899354
Banf1	3.82E-14	1.40861058
Trp53i11	1.49E-05	1.40834919
Pamr1	5.23E-07	1.4073606
Irf3	5.90E-14	1.40574255
Slc25a5	3.11E-33	1.40513286
Nubp1	1.76E-05	1.40405831
Dusp7	2.02E-08	1.4036635
Cd3eap	3.12E-05	1.40274894
Gm3336	0.02441	1.40233235

Iqcd	0.010989	1.40185413
Rita1	0.01101	1.40105436
Gm47484	0.016176	1.40061958
Tm2d2	6.27E-09	1.40010231
Vim	1.82E-109	1.40002478
Tpt1	2.46E-217	1.39972916
Rab13	0.000604	1.3995106
Rab17	0.019393	1.39831175
Spaca9	6.17E-09	1.39831048
Coq6	0.000277	1.3979338
Uqcrh	1.07E-65	1.39779493
Ech1	2.83E-12	1.39696177
Tbl3	0.000292	1.39507822
Mybbp1a	1.65E-11	1.39421855
Mfng	5.61E-05	1.39358286
Mturn	6.26E-06	1.39335515
00015A07F	1.95E-05	1.3922974
Kcnb1	4.48E-07	1.39067265
Gatc	0.003029	1.38979529
Fabp5	2.14E-43	1.38847297
Kcnh4	0.000493	1.38820035
Mrpl21	1.31E-08	1.38756978
Gm29093	0.011985	1.38746177
Nudt22	0.002325	1.38737033
Trmt2a	2.82E-07	1.38731854
Slc15a3	1.96E-10	1.38676208
Omd	0.02167	1.38637144
Snhg3	2.02E-06	1.38553567
Ilk	4.14E-18	1.38525318
Gm10658	0.001522	1.3845857
'00023F06F	0.043459	1.38368316
Lyl1	0.000459	1.38345254
Adh5	2.63E-07	1.38286068
Pfdn6	4.87E-10	1.38152033
Gm13205	0.046661	1.38106412
Msrb1	1.29E-38	1.38019527
Hint2	1.19E-14	1.38008951
Gm15545	0.001362	1.37932823
Arhgef25	0.000724	1.37912541
Tmco1	4.24E-28	1.37864907
Sigmar1	1.51E-06	1.37828297
Tmem216	2.17E-08	1.3775366
Gm16282	0.046259	1.37660211
Ogn	2.74E-18	1.37623124
30221H12F	0.038922	1.37573133
Masp2	0.017635	1.37560464
Lsm7	1.05E-06	1.37515103
Kcnrg	2.24E-15	1.37505763
Arsj	0.002646	1.37501955
Bcam	5.26E-54	1.37401762
Slc25a4	3.69E-18	1.37280773
Tomm6	3.56E-33	1.37249851
Phgdh	6.99E-09	1.37230673
Tmem204	9.66E-17	1.37197591
Dynlrb1	5.05E-19	1.37138695
Ss18l2	9.08E-06	1.37084665
Abhd14b	2.11E-07	1.3703653
Tbx3os1	1.80E-09	1.37031603

Smyd1	0.000478	1.3692204
Pnkd	8.52E-15	1.36879177
Lxn	1.75E-06	1.36725556
Pomt1	0.011228	1.36718245
[illegible]10020C07R	1.49E-14	1.36701615
Riox1	0.000136	1.36659301
Acot1	1.74E-06	1.36529
Il18	1.13E-19	1.36498392
Snrpf	3.78E-16	1.3638844
Gm45435	0.009863	1.36266562
Dph3	2.09E-07	1.36262109
Ppm1m	2.98E-05	1.36251976
Zfp449	0.038566	1.36046478
Scx	0.038907	1.36014304
Mrpl33	2.49E-38	1.35894318
Zfp408	1.28E-07	1.35837573
Gm16192	0.00049	1.35758978
Chrac1	7.56E-08	1.35757932
Psmd10	8.23E-05	1.35632523
Cetn3	3.50E-18	1.35629894
AC166172.1	0.025416	1.35553164
Cklf	1.18E-05	1.3545188
Cstb	2.67E-33	1.35387507
Raly	1.26E-11	1.35224585
Arhgef15	1.82E-36	1.35191794
Men1	0.000119	1.35118932
Gm36975	2.30E-14	1.35110859
Blvrb	1.45E-08	1.35097277
Tmem14c	8.73E-23	1.35037262
Gja4	4.14E-05	1.35017291
Gm42477	7.33E-05	1.34894168
Unc119	1.76E-08	1.3474517
Gadd45b	6.30E-10	1.34678516
Sftpa1	1.72E-219	1.34665102
Ccdc74a	0.007694	1.34629919
Ndufa4	1.68E-50	1.34558338
Ager	2.76E-55	1.34516674
Aldoa	1.39E-54	1.34484892
Ace	1.68E-47	1.34372398
[illegible]700034P13R	0.012669	1.34362917
Nudt8	0.005614	1.34273213
Srrd	0.01144	1.34209432
Rwdd1	3.33E-12	1.34197188
Hmgn5	6.24E-07	1.34191683
Impdh1	1.19E-09	1.34095104
Bcs1l	0.001937	1.34024643
Ccdc137	3.07E-05	1.33988938
Gm39822	0.000168	1.33938583
Mlx	0.003455	1.33896893
Cdh13	2.20E-08	1.3385972
Alkbh6	2.25E-05	1.33818265
Rab25	0.000375	1.33758029
Borcs7	3.15E-05	1.33381879
Wdr55	0.046222	1.3331097
Rpl7	4.02E-115	1.33309754
Fkbp11	0.012698	1.33164958
Aspn	0.045111	1.33120596
Bri3	1.05E-56	1.33119022

Mtg2	1.20E-05	1.33106481
Pifo	9.58E-08	1.33085431
Rrp8	3.35E-06	1.33047373
Olfml3	0.0014	1.33033951
Llph	3.73E-19	1.33009329
Mpg	0.000496	1.32989158
Gpr34	0.021696	1.32867732
Gatd3a	0.00039	1.32842232
Ino80b	3.08E-07	1.32797695
Dpysl5	0.018543	1.32752762
510528J11R	0.011975	1.32741764
Igha	9.86E-15	1.32673937
Foxo4	3.58E-05	1.32660853
Ntan1	7.48E-15	1.32541105
Actr1b	7.45E-07	1.32370532
Eif2b2	4.83E-06	1.32362457
Cycs	4.47E-12	1.32309609
Haspin	0.029023	1.32185222
Hras	4.95E-11	1.32144375
Bex4	1.79E-09	1.32091159
Arl16	0.001976	1.31983251
Zfp593	0.011578	1.3188067
Fmo1	4.24E-21	1.3172671
Prelid1	1.26E-11	1.31573354
Zyx	2.84E-14	1.31567306
Gmppb	0.004316	1.31546395
Gm16066	0.000127	1.31511955
Lmna	1.88E-33	1.31499701
Bace2	1.43E-07	1.314297
Dhrs4	1.23E-07	1.3141435
Ccl5	8.51E-06	1.31385848
Nbl1	3.69E-19	1.31303179
AV356131	0.004531	1.31295422
Ssbp4	5.23E-11	1.31231486
Atp6v1f	2.08E-25	1.31150733
530082P21R	1.26E-06	1.31054368
Psma4	7.80E-18	1.31008854
Aldoc	1.75E-05	1.30990852
Tor4a	0.036263	1.3093844
Cox6b1	1.03E-51	1.30829892
Ppp1ca	1.67E-23	1.30778554
530028O10R	4.75E-06	1.30676566
Akr1b3	9.13E-22	1.30622493
Ccdc34	6.23E-09	1.30620202
30111J21Ri	1.20E-18	1.30595504
Adgre5	5.74E-53	1.30594892
Sem1	8.57E-62	1.30531385
Chrdl1	7.32E-05	1.30524164
Sub1	1.71E-39	1.30351293
Ccpg1os	5.01E-06	1.30237671
Nhp2	3.82E-10	1.30173982
Srxn1	0.003056	1.30118266
Gm26691	0.017311	1.3004481
Nmnat1	0.005883	1.30041996
Meig1	2.65E-07	1.29900947
Gm17178	0.000313	1.29873196
Gm13889	0.034142	1.29859598
Rraga	2.70E-06	1.29755671

Cyb561d2	0.005093	1.29722961
Vstm5	0.015879	1.29658885
C2cd4b	0.008773	1.29640605
Ier3ip1	5.72E-16	1.29367156
Thnsl2	0.044999	1.29349901
Adrm1	2.10E-06	1.29165438
Ffar2	0.003444	1.29103738
Bmx	3.26E-09	1.28965688
Trmt5	0.036061	1.28891651
Ndufa11	4.30E-20	1.28855283
Slc26a10	5.08E-11	1.28828826
Ssr2	4.54E-10	1.28789595
D17H6S53E	0.001261	1.28656115
Grap	5.84E-08	1.28224367
Wdr18	0.000137	1.28140052
Plec	6.95E-36	1.28139979
Gpx8	1.14E-07	1.2809161
Gimap1	3.19E-12	1.28083668
Inafm2	0.000431	1.27913457
Gm11831	0.003026	1.27689034
Gtf3a	4.36E-05	1.27650388
Rgs19	6.32E-05	1.27581269
Ear1	1.11E-22	1.27573308
Gins4	0.002251	1.2744234
Galt	0.033084	1.27422562
Zfp511	4.13E-05	1.27348696
Prorsd1	1.78E-06	1.27319257
10010K14R	0.00072	1.27236474
Pmm1	0.002932	1.27222427
Crispld1	0.000139	1.27113171
310037I17R	1.29E-52	1.27106981
Acadvl	3.51E-08	1.27079362
Cox14	1.29E-18	1.27071306
Eid2	0.007533	1.27004099
Apoe	1.35E-33	1.26961726
Lgals4	0.028064	1.26890964
Gas5	1.30E-25	1.26765145
Ndufa12	9.55E-16	1.26719129
Nfic	2.24E-21	1.26680563
Gnai2	6.42E-41	1.26676763
Sod3	3.30E-05	1.26668298
Zfp955b	5.73E-05	1.26521046
Zfp566	0.015066	1.26450865
Fmo5	7.41E-09	1.26450473
210016L21R	2.05E-06	1.26377392
Clec4b1	0.003443	1.26357223
Flii	3.06E-17	1.26302347
Ap1m1	3.84E-05	1.2630115
Eln	5.57E-47	1.26280659
Ankrd24	0.000502	1.26232248
Rtraf	1.78E-20	1.2621558
Nelfe	1.68E-05	1.2601566
Ccl21a	2.59E-10	1.25949915
030018B13R	0.00232	1.2590005
Fxyd5	5.36E-47	1.25851989
Smim4	5.65E-13	1.25820398
Rtl8b	3.25E-05	1.25708502
Lamtor5	8.74E-10	1.25642307

Foxc1	0.000805	1.25638888
10320M18F	0.001384	1.25557571
Pabpc4	6.25E-14	1.25540982
Tnfsf12	5.88E-09	1.25396049
Cnpy2	3.28E-13	1.25393742
Gm47889	0.001541	1.25249757
Gm11837	0.014744	1.25244296
Gstm2	9.06E-14	1.25022095
Pcdh12	1.41E-05	1.24955825
Rpl13a	5.11E-97	1.24861561
Psme2	3.33E-15	1.24756032
Eef1d	1.60E-18	1.24738937
Bcl2a1a	1.21E-07	1.24720512
30439K02F	0.002902	1.24714485
Cdc37	6.04E-13	1.2470329
Fam53b	1.17E-12	1.2443638
Ubxn1	9.77E-21	1.2436508
Calm1	1.59E-120	1.24248881
Os9	1.23E-17	1.24214473
Syf2	4.88E-16	1.24141367
Tie1	6.27E-30	1.24130577
F8a	0.011242	1.24128466
Amigo2	0.000228	1.24106559
Gm30054	0.000128	1.24050369
Cyren	3.88E-05	1.24023039
Napsa	2.41E-28	1.23908682
L3mbtl2	0.000122	1.23857275
Nop10	4.55E-15	1.23814087
Chchd1	6.11E-13	1.23786972
Cnih4	1.87E-11	1.23669028
Dmkn	1.08E-08	1.23667693
Tmem203	0.000562	1.23591761
Cidec	3.04E-09	1.23567091
Avpi1	1.49E-11	1.23443879
B3galt2	0.001142	1.23436048
Trappc4	2.30E-11	1.23410984
Bex3	5.80E-08	1.23355041
Fcgr3	3.23E-10	1.23320465
10002D19F	0.033024	1.23286366
Ppp2r5b	5.63E-05	1.23249729
Aldh2	5.84E-43	1.23247193
Grpel1	4.07E-06	1.2306351
Kcnj13	0.003465	1.23034613
Wscd1	9.47E-06	1.22923661
Psmc3	4.53E-19	1.22848189
Caskin2	4.62E-08	1.22794759
Homer3	5.26E-08	1.22769267
530033B14F	0.001531	1.22758839
Leng1	0.000152	1.22723771
Fgd5	3.79E-56	1.2262017
Tbca	1.08E-38	1.22533048
Smtnl2	5.22E-05	1.22495732
Dnal4	0.000838	1.22445722
Ppp1r18os	3.82E-05	1.22388027
Myct1	1.26E-10	1.22354039
Nsun5	0.013461	1.22238724
Ndufs6	6.43E-18	1.22233579
Ear2	1.84E-42	1.22176978

Fbxl15	0.002904	1.22107237
Tubb2a	1.18E-07	1.2207343
Sra1	5.74E-07	1.2206187
Idh3g	8.79E-10	1.22034452
Akt1	7.32E-10	1.2200511
C5ar2	0.001604	1.21786696
Cenpb	1.15E-14	1.21720894
Cep68	1.52E-17	1.21687793
Aimp1	1.60E-15	1.21618654
Sbds	3.14E-16	1.21563739
Serpinb1a	4.39E-10	1.21478626
Ret	0.00081	1.21471287
Mrpl30	8.68E-11	1.21352344
Cox20	7.83E-09	1.21309218
Cetn2	3.76E-21	1.21242387
Zfp46	1.94E-06	1.21193858
Psma7	1.86E-31	1.21189952
Arpc3	2.59E-28	1.21172819
Glis2	4.98E-11	1.21111043
Gm26873	0.00347	1.21104308
10131K14R	0.011224	1.21103821
Trappc2l	5.30E-11	1.20998701
Gm35853	3.95E-07	1.2092066
Gm11520	0.0136	1.20896299
Zfpm1	0.00066	1.20845089
Riiad1	5.24E-13	1.20676896
Ptpn7	0.000419	1.20635682
Mest	0.004485	1.20611671
Hist1h2bc	1.21E-20	1.20550067
Fadd	0.022756	1.20532228
Mgst1	6.21E-67	1.20376401
Mast1	0.026007	1.20282384
Xrcc6	3.61E-09	1.20250365
Smim12	0.003427	1.20179652
Eme2	0.029138	1.20155456
Rnf186	4.33E-05	1.20092274
Cdh5	1.23E-41	1.2002305
Tsc22d4	2.10E-20	1.19951446
Ephb6	0.029742	1.19829691
Armcx5	0.039258	1.19742757
Gfer	0.000383	1.19720681
Gm10785	2.53E-06	1.19667309
Cd320	0.046699	1.19568289
Spink2	0.002377	1.19547955
10043K17R	4.69E-10	1.19518879
Calm3	1.08E-18	1.19269511
Cd74	2.63E-131	1.19226339
Nans	2.20E-06	1.19202427
Ndufa3	7.16E-29	1.19149088
Nkain4	3.85E-16	1.19110865
Lrrc32	0.004088	1.19092146
Sfrp2	1.72E-14	1.18981191
Carhsp1	8.19E-09	1.1895296
Pilra	4.06E-08	1.1883422
Kank3	2.31E-38	1.18705019
Akap14	0.00057	1.18628025
Ccdc86	0.000169	1.18615285
030581F22R	1.20E-05	1.1860237

Creg1	1.38E-31	1.18584859
Gm16093	0.000122	1.18501187
Gm2a	2.03E-19	1.1838777
Ly6e	3.26E-85	1.18341223
Ttc32	0.000258	1.18311661
Atp6v1e1	3.15E-35	1.18303795
Srm	0.046334	1.18254849
Krt8	4.36E-26	1.18222947
[illegible]10024B03F	0.007072	1.18202046
Cfap298	3.85E-10	1.1819671
Mrpl43	8.40E-09	1.18195931
Kcnt2	0.003777	1.18165098
Pdzd11	0.000307	1.18156422
Gm14221	0.014497	1.18073368
Psmb6	1.09E-13	1.18016322
Aldh1a2	3.43E-14	1.17970443
Prkd2	5.89E-33	1.17930916
Pin1	0.001037	1.17878102
Ndufb6	6.84E-11	1.1777421
Tmem205	1.81E-10	1.17741525
Fastkd1	0.002357	1.17726898
Pkm	2.63E-23	1.17720891
Mcf2l	1.89E-24	1.17703932
Selenow	1.23E-25	1.17672709
Zcrb1	3.94E-18	1.17644857
Gm47754	0.025681	1.17643374
Trpc3	0.008184	1.17591442
Spa17	6.58E-10	1.17501709
Cyp2j6	0.001809	1.17448333
Rplp2	2.72E-102	1.17431114
Nkd2	0.002143	1.17379799
Hsp90ab1	1.34E-115	1.17353263
Cx3cr1	5.87E-05	1.17320516
Prr15	0.00061	1.17197127
Mrps11	0.00438	1.17193988
Sparcl1	3.76E-33	1.1717447
Mettl23	2.06E-07	1.1715774
Hcar2	0.004531	1.17105015
Zkscan14	0.007764	1.17023255
Efr3b	7.76E-06	1.16953498
Pgp	4.72E-09	1.16812686
Fam180a	0.000375	1.16761884
Ndufab1	1.71E-17	1.16736504
Nfe2l1	1.73E-29	1.16691159
Zfp692	1.57E-05	1.16649382
Eif6	5.37E-07	1.16601466
Ccdc160	0.005237	1.16597489
Tekt2	0.008717	1.16492313
Rbx1	1.67E-26	1.1634015
Chst15	0.001706	1.1631928
Slx4	0.03901	1.16306108
Glp1r	2.10E-14	1.16277674
Castor1	0.001057	1.16239305
Alox5ap	6.66E-21	1.16221276
Rrp1	1.12E-10	1.16205351
Zfpl1	3.92E-05	1.16186729
Ocel1	3.58E-06	1.16131604
Zdhhc24	0.001066	1.16097148

Ybey	0.005665	1.15919428
Itm2a	0.004045	1.15906769
Sdr39u1	0.020231	1.15875249
Iqcg	1.44E-05	1.15874762
Emc7	3.85E-17	1.15846372
Kat2a	0.011294	1.15795089
LTO1	9.93E-06	1.15749014
Chchd5	0.001374	1.15706773
Med22	0.001256	1.15666558
Efs	0.043587	1.15583505
Cyb5r3	1.78E-24	1.15369497
Gpr65	0.011582	1.15345944
Mob3c	6.57E-05	1.15286996
Gsta4	4.97E-12	1.15249286
Zfp637	0.000284	1.15171506
Atp5l	2.97E-53	1.15069863
Polr2d	0.000493	1.14980914
Rpgrip1	2.89E-08	1.14934848
S100a8	0.000533	1.14837968
Nenf	1.14E-25	1.14816738
Jun	2.69E-18	1.1481373
Tnfaip8l2	1.33E-05	1.14810092
Pomgnt1	9.92E-07	1.14588892
Pcp4l1	2.41E-11	1.14532849
Bvht	0.0003	1.1451799
Cops9	1.49E-15	1.14496835
Tst	6.89E-11	1.14392437
Polr2c	1.91E-07	1.14364971
Gabarapl2	1.61E-19	1.14265688
Mrps5	7.67E-07	1.14255985
Ginm1	1.25E-07	1.14137712
700016J18R	0.043495	1.14074803
Asl	0.000199	1.14055206
Gm50020	0.029323	1.14022916
Thap11	0.005568	1.14022582
Gm49708	2.24E-06	1.14010614
Rbp4	0.003546	1.13946178
Ighg2b	0.008775	1.13937146
Zfp319	0.00052	1.13900825
Sdhaf4	8.28E-07	1.13891809
Nop53	1.10E-12	1.13866321
Ubald1	4.33E-05	1.13857652
10006K23F	0.001475	1.13822338
Tmem60	1.19E-05	1.13800975
Acaa2	4.59E-14	1.1372594
Cetn4	2.44E-07	1.13575521
Stmn2	2.24E-16	1.13553561
Kank4	3.11E-05	1.13510975
Col6a2	8.19E-10	1.13461105
Rbm8a	8.99E-14	1.1323627
700029J07R	3.52E-05	1.13200975
Gpsm3	1.14E-08	1.13167595
Mr1	3.53E-05	1.1314923
Sri	2.29E-18	1.13102465
Snrpa1	5.97E-06	1.12997774
Rps26	9.59E-97	1.12989879
Tinagl1	2.83E-05	1.12983599
Map1lc3a	1.74E-18	1.1297875

Tusc2	0.00046	1.12941631
Abhd14a	0.000898	1.12811402
Phf5a	4.63E-08	1.1259259
Mcm4	0.002536	1.12572737
Lgals3	1.46E-49	1.12459374
Clec9a	7.85E-10	1.12455482
Tecpr1	7.35E-17	1.1245122
Snhg14	0.005289	1.12377916
Gm38948	0.000153	1.12315056
Phpt1	0.004599	1.1227063
Snrpe	2.81E-23	1.11979431
Fancb	0.002669	1.1189427
Syt7	7.62E-08	1.11836662
Aldh3a1	0.049741	1.11798537
Nudt19	0.000941	1.11713018
Adprhl2	0.000102	1.11699341
Abhd15	0.003977	1.1161421
Atp6v1g2	0.00067	1.11540666
Il27ra	0.017129	1.1145493
Ctla2b	0.001532	1.11395461
Bcl10	7.38E-10	1.11243461
Gm37240	1.08E-41	1.11172527
Tmem256	5.53E-16	1.11144602
Fstl1	3.11E-13	1.11083571
Btg4	0.024017	1.11040692
Rad51	0.013567	1.10954241
Paqr3	0.004585	1.10865903
Rab5if	4.07E-11	1.1078001
Abhd4	3.72E-05	1.10745935
Anapc11	5.75E-10	1.10724986
Anxa8	4.57E-09	1.10698073
Snapin	8.13E-09	1.10689683
Gm16062	0.003454	1.10688161
P4htm	0.00508	1.10643199
Atrip	0.001211	1.10582812
Pfdn1	2.50E-13	1.10366211
Polr3e	3.15E-06	1.1036608
Ecscr	6.87E-08	1.10307694
Znhit6	9.21E-08	1.10091077
Mtln	8.29E-11	1.10068035
Edn1	2.30E-06	1.10025694
Snx21	1.08E-08	1.10009266
Pigs	9.69E-07	1.0998454
Cd52	1.25E-19	1.09852784
Slc31a2	5.60E-10	1.09836741
Mgp	1.33E-127	1.09812094
)30025P20R	0.025655	1.09640857
Gabarap	3.71E-50	1.09639263
Rab8a	9.05E-08	1.0955869
Lrrc61	0.030904	1.09534801
Slc41a1	7.72E-06	1.0951963
Coa4	0.002407	1.09502111
Zfp882	0.038922	1.09457886
Ninj1	0.024234	1.09362852
Lrp10	8.22E-16	1.09334244
Ndufa8	2.48E-11	1.09319118
Igf2r	9.19E-20	1.09302625
Tpcn1	8.11E-11	1.09302095

Plod3	5.11E-08	1.09293773
Mycbp	9.70E-13	1.09120866
Trf	3.71E-64	1.09064868
Mtif3	0.000458	1.08952632
Eef2	2.29E-49	1.08919213
Cd27	9.58E-08	1.08893912
Cfap157	0.00405	1.08814193
Nanos1	0.019826	1.08782722
Emcn	9.85E-30	1.08710058
Hspa1b	3.92E-05	1.08554647
10059G10F	2.07E-05	1.08414411
Gm9856	7.67E-06	1.08396046
Lsm4	2.77E-10	1.0837778
Lbp	1.38E-15	1.08368667
Gm36279	0.000341	1.08327394
Krt80	2.14E-08	1.08318019
Tal1	5.33E-08	1.08229814
Htra1	5.65E-06	1.08221515
Prr29	0.007709	1.08211777
Gstm1	1.79E-43	1.08138423
P3h4	1.59E-07	1.08091828
Got2	8.28E-08	1.08069713
Hacd4	2.70E-10	1.08059736
Tmem184a	0.010156	1.07951065
Mrps7	5.06E-05	1.07902843
Exosc8	5.21E-05	1.07886618
Cldn5	3.97E-22	1.07750237
Snn	0.000505	1.07745666
Mfsd5	1.08E-08	1.07670158
Cox17	2.17E-24	1.07651464
Ctsd	4.98E-100	1.07649561
Uqcrc1	5.92E-09	1.076367
Cox19	2.51E-05	1.07525828
C2	3.55E-20	1.07522108
Rfxap	4.00E-05	1.07489938
Notch4	5.50E-08	1.07211227
Acta2	0.003402	1.07190811
Cfb	1.13E-20	1.07167062
Dusp19	0.005919	1.0713041
Psenen	2.51E-16	1.07119262
Lrp1	1.10E-33	1.0711688
Ttyh3	1.63E-07	1.07071827
Mrpl55	4.53E-05	1.06975768
Fdx2	3.72E-05	1.06959191
Psmb8	1.23E-09	1.0692334
Gstt2	3.51E-06	1.06784554
Lgals1	5.14E-14	1.0676403
Clec1b	0.000284	1.06759646
Pirb	8.27E-10	1.06710742
Btf3	1.66E-34	1.06635646
Card19	1.41E-05	1.06623236
Gadd45g	1.89E-05	1.06486798
Lig3	1.56E-06	1.06479632
Gm15417	0.005666	1.06319096
Gm11423	0.014692	1.06305361
Sytl1	0.038999	1.06276403
Ifi30	2.64E-26	1.06247063
mt-Co3	9.63E-117	1.06208434

Anxa5	3.08E-42	1.06206039
Zfp128	0.036403	1.06187889
Tbcb	1.16E-11	1.06185052
Ptp4a3	1.63E-08	1.06129738
00010M07F	2.92E-11	1.06082585
Klf10	1.87E-14	1.0606508
Timm44	3.97E-05	1.06031406
Slc39a4	5.77E-08	1.05899417
Aarsd1	1.63E-05	1.05854175
Tbxa2r	0.019698	1.05845588
Plpp2	0.036578	1.05760307
Chmp7	0.003766	1.05745163
'30409E04F	0.001436	1.0571994
Atraid	6.03E-10	1.05691777
Ttll1	0.002551	1.05518697
Xylt2	7.42E-05	1.05355666
Serpine1	0.000196	1.05322942
Prpf8	1.35E-09	1.05301572
Krt79	4.56E-14	1.05135923
Prx	1.46E-22	1.05114126
Nos3	3.16E-08	1.05061984
Rapgef3	5.44E-14	1.04971602
Tma16	0.001103	1.04922531
Atoh8	0.007581	1.04917515
Tead2	4.12E-05	1.04879929
Mob3a	2.28E-05	1.04796809
Eif3i	1.04E-08	1.04748078
Klf15	2.82E-05	1.04729906
Ltf	6.12E-13	1.04692974
Hmgb1	1.12E-47	1.04676647
Med10	4.05E-05	1.0467444
Sparc	6.91E-49	1.04486088
Eif5a	8.29E-19	1.04420443
Eef1g	2.38E-23	1.0433143
Ppm1f	3.84E-09	1.04289915
Cks1b	0.001747	1.0419442
Fis1	2.02E-20	1.04146042
Arap3	8.13E-24	1.04064055
Tle5	7.71E-24	1.04056581
Eef1a1	9.08E-126	1.0403227
Slc29a1	1.14E-08	1.03980452
Chst12	0.000274	1.03951254
Snhg10	0.014732	1.03885395
Sema3b	0.002386	1.03794751
Ift20	8.87E-16	1.03746641
Rbm4b	3.30E-09	1.03697759
Gnas	9.12E-79	1.03670616
Idh3b	1.60E-07	1.03515536
Fam89b	0.00047	1.0350636
Alkbh7	0.004048	1.03355675
Oaz1	1.47E-49	1.03316388
Tbkbp1	2.04E-05	1.0329604
Mrps12	2.19E-08	1.03275669
Ncl	4.27E-34	1.03120225
St6galnac6	0.000645	1.03110876
Gpr4	0.032727	1.03101021
Tbata	0.009607	1.03007263
Casp9	0.001521	1.029197

10019D21F	0.009895	1.02915398
Ehd4	1.33E-48	1.02851471
Atp5b	1.03E-29	1.02832814
Ebp	1.92E-11	1.02789936
S1pr1	1.52E-22	1.02732262
Lsm2	0.000109	1.02718013
Mrpl17	7.09E-07	1.02711851
Ano8	0.04553	1.02688575
Tpm4	1.77E-24	1.026632
Bmp2	0.021832	1.02627235
Mrps36	8.95E-06	1.02596799
Cd93	1.75E-16	1.02563591
Gpx7	0.008865	1.02556325
Gm16278	0.039007	1.02461734
Gm20416	0.004612	1.02406873
Gpr182	0.04646	1.02300355
Dusp3	3.41E-20	1.02248748
Fuz	0.002016	1.02244835
Psmd7	4.07E-12	1.02208036
Gpx1	1.40E-52	1.02194455
Srp14	8.38E-21	1.0213757
Gstt3	0.000494	1.01996542
Exosc3	1.39E-05	1.01957335
Sec62	1.12E-46	1.0195491
Cyp2d22	3.55E-06	1.01933654
AI413582	0.000732	1.01900178
Rad1	0.007685	1.01890749
Maf1	1.80E-05	1.01859291
Npm1	2.97E-43	1.01851375
Polr2j	3.07E-07	1.0184278
Atp6v0c	4.26E-24	1.0179129
Pcbp4	0.017393	1.01675322
Phb	0.000428	1.01596237
Pycard	1.98E-05	1.0156792
Tirap	1.05E-05	1.01564007
Aplnr	0.002358	1.01548252
Mark4	5.32E-07	1.01497857
Dock6	1.85E-22	1.01492034
Acd	0.010807	1.01488901
Eif2b4	1.33E-05	1.01395022
Tubg1	0.01483	1.01282463
Snhg8	8.03E-11	1.01268003
Def6	4.04E-09	1.01252969
Npdc1	1.04E-07	1.01232536
Tprgl	5.65E-10	1.01155268
Dhodh	0.005591	1.01129958
Rbmxl1	9.95E-05	1.01116256
Coq10a	0.000433	1.01038884
Tpbgl	0.011636	1.01038036
Parvb	3.10E-21	1.00994755
Phykpl	3.48E-08	1.00933279
Ldhb	1.16E-10	1.00910944
Ptprb	6.53E-65	1.00799855
Fabp4	6.15E-10	1.00768759
Mir142hg	1.57E-15	1.0073028
Acbd4	0.006043	1.00556856
Odc1	1.13E-12	1.00550953
Tln1	1.29E-22	1.00506591

Ly6g6d	0.026052	1.00491614
Spr	6.23E-06	1.00445645
Chd3	4.32E-17	1.00369703
Acot13	5.97E-07	1.0026274
Cygb	9.79E-07	1.00235105
Mdh2	8.99E-12	1.00190724
530005A16R	0.039001	1.00074947
Apip	0.000437	1.00016311
Rgs10	3.46E-05	1.00002372
Cic	3.08E-06	0.99988339
Mettl26	4.27E-06	0.99970355
Fam161b	0.002105	0.9993164
Mrpl35	4.70E-06	0.99908243
Inmt	9.46E-14	0.99905753
Lyar	0.000138	0.99871857
Mast3	0.000121	0.99870731
Mtss2	3.57E-05	0.99707573
Zfp623	0.043144	0.99665642
921511C10R	1.18E-08	0.9962462
Cnot3	4.56E-16	0.99575035
30405H02R	3.60E-09	0.9955988
Chac2	2.58E-05	0.99533158
AW209491	0.003427	0.99505866
Rxylt1	6.66E-08	0.99485839
Lamp1	1.17E-49	0.99463484
Zfp74	1.37E-05	0.99340951
Pon1	5.98E-11	0.99308176
Pdcd11	3.44E-08	0.99305101
700062C07R	0.000237	0.99303795
130317F20R	0.032004	0.99244593
Timm13	1.13E-13	0.98998976
Saysd1	1.40E-05	0.98976626
Fam189b	0.014733	0.98928898
Abi3	6.30E-09	0.98919151
Shisa5	4.66E-19	0.98865102
Kcnab1	2.67E-05	0.98804421
Ergic3	2.02E-09	0.98804196
Ptov1	1.65E-10	0.98771004
Tmem242	0.000692	0.98724239
Gsdmd	3.86E-05	0.98688894
Pofut2	0.000403	0.9865673
Ctbp2	4.76E-08	0.9863081
700037C18R	6.62E-05	0.9858314
Sirt7	1.01E-07	0.98560607
Nt5dc2	0.014715	0.98521983
30043M19R	3.02E-06	0.98443459
Nop56	1.17E-06	0.98412333
Ctdsp1	1.51E-07	0.98406925
Hirip3	0.024594	0.98394442
700094D03R	3.85E-13	0.9836062
Rras	3.90E-08	0.9831306
Gm26631	0.009841	0.98291593
Cryz	0.045337	0.98283525
Accs	0.001099	0.98229337
Thoc7	3.81E-13	0.98227957
Gm49602	0.005913	0.98196066
Spata24	0.002876	0.98138726
Snrpd1	4.34E-13	0.98097484

Fendrr	5.89E-38	0.98094725
Prpf40b	6.97E-06	0.98092825
Yipf3	1.58E-09	0.98070337
Smtn	0.000212	0.98053299
00012H06F	2.04E-05	0.98042516
333403J16R	0.033453	0.97890757
Zfp758	0.001535	0.97870639
Dmac1	0.00067	0.97851384
Klhdc4	1.23E-19	0.97833275
Pak4	0.000122	0.97791354
30020H09F	0.046868	0.97785583
Capn10	0.001049	0.97725364
Unc119b	2.23E-05	0.97720726
H2-T23	2.19E-09	0.97663626
Swi5	1.42E-21	0.97648457
Lilra6	0.039209	0.97637663
Gtf2h5	5.80E-13	0.97576063
10040N11F	0.001499	0.97575616
Cplx2	0.022025	0.9757055
Polr1b	0.005687	0.9756111
10032A03F	1.17E-05	0.97548308
Cltb	2.20E-10	0.97548125
Atf5	1.62E-05	0.97444383
Cdc42ep3	1.26E-25	0.97419006
Kxd1	5.43E-08	0.97386852
Pard6g	1.74E-10	0.97366276
Rps9	2.06E-103	0.97359547
730020E08F	0.03443	0.97348305
Angpt2	0.009088	0.97332863
Ubxn8	4.87E-06	0.97217831
Polr2k	2.96E-11	0.9718448
Abca2	0.000185	0.97173697
Ccdc106	0.003868	0.97113221
Higd1a	6.82E-13	0.97098824
Amz2	8.67E-08	0.97075065
Naglu	0.000345	0.97061863
Pla2g15	8.10E-10	0.97036686
Rpp25l	0.000502	0.97015603
Nudc	2.05E-14	0.96994136
Bax	1.82E-10	0.96975977
Pdlim2	5.07E-07	0.9688651
00052L18F	0.000571	0.96875894
Mesd	7.28E-09	0.9685662
Zfp646	7.09E-07	0.96748625
Gas2l1	3.34E-08	0.96576436
Muc16	7.34E-13	0.96569002
Lrrc26	0.024232	0.96564341
Dctn6	2.76E-12	0.96551276
Mrnip	0.004592	0.96520614
Gps1	8.71E-05	0.96459261
Cript	2.04E-10	0.96424374
Gmfg	1.06E-09	0.96416984
Cpe	1.78E-12	0.96359931
Star	0.000365	0.96337445
Qars	2.55E-05	0.96259926
Pnp	2.34E-12	0.96178088
Paqr7	2.88E-06	0.96170836
Slc10a3	0.043832	0.96147414

Srp9	1.09E-14	0.96092655
Cavin4	0.010005	0.95990634
Snu13	1.05E-09	0.95976885
Myl6	1.36E-92	0.95963101
Ankrd29	0.000742	0.95940063
Pink1	1.62E-06	0.95907226
Dpp3	2.27E-06	0.95893767
Dsel	0.000759	0.95860751
Ydjc	0.034581	0.95698652
Dnttip2	3.13E-07	0.9568729
Ets2	2.51E-12	0.95645751
Dkk3	0.000874	0.95598182
Epcam	1.78E-11	0.95575262
Urod	0.00034	0.95504851
N4bp3	2.04E-05	0.95503904
110082I17R	9.09E-11	0.95470176
Glrx2	3.12E-08	0.95447446
B4galt4	8.50E-10	0.95417632
Ecm1	3.34E-11	0.95351065
Exoc7	1.54E-05	0.95275521
Gemin7	2.38E-08	0.95237385
Cacybp	4.72E-13	0.95124975
Morn2	0.000438	0.95085286
Tmem98	2.21E-05	0.95071463
Nxt2	0.010598	0.95058881
Thap3	1.86E-15	0.95043797
Hyal2	5.38E-05	0.9500556
Babam1	3.15E-08	0.94950404
Cops6	1.70E-07	0.9495008
Eif4a3	4.13E-08	0.94930446
Scn3b	5.95E-14	0.94922181
Rrp36	7.90E-05	0.949094
Gm34408	0.007418	0.94898547
Selenop	5.14E-33	0.94877574
Pradc1	0.00162	0.94866852
Dhrs1	2.01E-05	0.94861221
Rpl8	4.57E-72	0.94859795
Dnajc27	0.017185	0.94859583
Wdr83os	4.69E-08	0.94852615
Pdcd2	0.011734	0.94847984
Lilra5	1.37E-08	0.94774779
Hypk	2.38E-06	0.94773008
Ndufb7	6.71E-22	0.94629186
Chpf2	2.23E-05	0.94607669
Tmem37	0.007682	0.94551639
Ypel1	0.001839	0.94467428
Capg	6.41E-10	0.94463202
Kctd12b	9.18E-05	0.94400066
Psme2b	0.0008	0.94389357
Gm36660	0.005743	0.94364567
Iah1	2.68E-09	0.94316873
Ggact	4.11E-05	0.94286557
Utp3	5.61E-08	0.94132907
Nr1h2	2.05E-07	0.94126304
Trp53rka	0.003513	0.94056525
Gm12353	1.12E-11	0.93951226
Tmem176b	9.81E-26	0.93945
Bace1	6.80E-06	0.93914291

Clta	6.31E-23	0.93900488
Eif5b	2.87E-25	0.93874508
Abcb1a	2.98E-16	0.93834816
Myh10	1.85E-07	0.9367974
Lgalsl	0.002513	0.93623458
Donson	0.00208	0.93605228
Upk1b	1.96E-10	0.9359989
Ubl4a	7.59E-05	0.93535088
Tmed3	2.98E-08	0.93499694
Camk1	3.10E-10	0.93352432
Ercc1	0.008914	0.93302941
Acyp1	2.73E-05	0.93300977
Zfp316	0.039472	0.93242886
Mterf1a	0.016153	0.93211437
.10004F10R	9.97E-13	0.93205058
Spi1	1.18E-14	0.93189048
Man2c1os	2.97E-07	0.93182187
Dlec1	0.026055	0.93167042
Dkc1	0.000141	0.93161607
Tesk1	0.00212	0.93155986
Mrpl51	2.57E-12	0.93155559
Cav2	5.02E-13	0.93140356
Pgls	1.29E-12	0.93131226
BC003965	1.56E-05	0.93007094
Dcdc2b	0.001625	0.92963457
Hmgn2	4.08E-06	0.92947572
Igfbp7	1.57E-31	0.92773438
Fbln5	4.08E-11	0.92764376
Fzd4	6.45E-09	0.92746688
Ccdc84	0.009025	0.927028
Tuba4a	6.47E-09	0.92668549
Rxra	3.55E-07	0.92476566
Cox15	0.036599	0.92422709
Plod1	3.70E-06	0.92347348
Alox5	3.29E-07	0.92318017
Trim56	6.78E-11	0.92300197
Cav1	1.67E-47	0.92285734
Sirt2	7.38E-11	0.92222387
Timm8a1	0.009197	0.92213487
Emp1	1.12E-10	0.9214338
Smim19	7.07E-05	0.92142562
Ncoa4	2.00E-14	0.92133263
Fam110a	0.001212	0.92119998
H3f3b	1.44E-63	0.92056012
Slc3a2	3.06E-16	0.9204834
Mettl7a1	7.31E-23	0.91979037
Eif3f	1.70E-14	0.91944152
Sh3tc1	3.59E-09	0.91928718
Med19	2.43E-05	0.91901805
Retnlg	0.00731	0.91841516
H3f3a	4.62E-47	0.91831051
Plbd2	0.00012	0.91829639
H2-DMa	1.35E-08	0.91723974
Tomm40	6.66E-07	0.91665022
Egfl7	9.38E-42	0.91580433
Cep164	6.00E-06	0.91571484
Fkbp7	0.000323	0.91537622
Ddx54	8.70E-07	0.91533047

Ccdc12	1.56E-11	0.91446063
Arpp19	1.11E-18	0.91370295
E2f4	0.001024	0.91368765
Ebpl	0.000278	0.91364461
Sema6b	0.003796	0.91354415
Eif3c	2.38E-24	0.91243101
Hdac5	5.78E-05	0.91237576
Cpsf1	0.002375	0.91177862
Xpa	2.93E-07	0.91164181
Hp	1.28E-44	0.91120187
Trim17	0.039567	0.91103143
Mfap1a	3.03E-06	0.91095658
Pold2	0.013191	0.9108077
Snrnp35	0.013555	0.91065419
Bcl2a1b	1.11E-05	0.91041851
Nicn1	0.017208	0.91008592
Ptms	4.94E-37	0.90989701
Slc25a1	0.000192	0.90849905
Heg1	2.55E-77	0.90812764
Cacna1a	9.33E-09	0.90809933
Dnajb1	8.79E-06	0.90794374
Tmem101	0.00467	0.90627056
Mthfsd	0.000726	0.90606843
Abhd16a	2.96E-08	0.90572252
Ildr2	4.43E-06	0.90559602
Atp5g2	1.57E-30	0.90500235
Sh2d3c	2.71E-14	0.90491907
Lypd2	1.20E-18	0.90453992
Slc43a3	3.77E-30	0.90378544
Gabarapl1	2.55E-19	0.90337538
Haus2	0.00019	0.90321128
Mrpl42	1.43E-06	0.90293152
Ahsa1	1.04E-06	0.90120724
Zrsr1	0.000635	0.90042336
Rtl8a	2.00E-05	0.90020858
Washc1	0.000284	0.89920044
Stambp	0.000283	0.89842576
Emc4	0.005087	0.89838139
Gm1673	4.44E-06	0.89746279
Pecam1	1.47E-59	0.89638367
Fads2	2.25E-06	0.89561224
Acvrl1	1.97E-24	0.89541096
Snrpd2	4.80E-09	0.89514035
Rack1	2.19E-53	0.89410624
Bcl6b	5.31E-05	0.89389297
Fastk	0.000121	0.89344307
Ccdc124	3.90E-05	0.89322225
Lyrm2	4.91E-06	0.89295009
Fes	1.47E-06	0.89176881
Psmc5	5.83E-09	0.89104716
Eif1b	9.30E-10	0.89102712
Aimp2	0.00523	0.89084564
Exosc4	0.00017	0.89054221
Fbxl8	0.002637	0.89021858
Ccm2l	5.60E-13	0.88962179
Mphosph10	0.000173	0.88914232
Ppif	0.011714	0.88842106
Nop14	1.60E-05	0.88814263

Atp5c1	2.13E-16	0.88806543
Prss23	8.43E-10	0.88776228
Malat1	0	0.88737114
Amd1	1.77E-08	0.88613251
Podxl	4.93E-35	0.88594788
Cuta	2.11E-08	0.88580995
Spns2	7.40E-09	0.88543815
Rnf215	1.03E-06	0.8853616
Khk	0.047392	0.88479636
Plvap	0.000243	0.88373381
AI837181	0.001173	0.88367018
Slc52a2	0.019779	0.88204992
Tbx3	2.57E-15	0.88177963
Jagn1	0.000226	0.88140514
Dynll2	2.05E-11	0.88130844
[illegible]30014A01[illegible]	0.002042	0.88090242
Lgals9	1.12E-11	0.88084832
Emd	1.07E-05	0.8805507
Dtymk	3.02E-07	0.87970989
Cox5a	1.23E-26	0.87937538
Il2rg	8.17E-05	0.87925129
Fuca1	8.31E-14	0.8789816
Rab3d	8.86E-06	0.87895501
Cfap57	0.022494	0.87886934
Mpc2	1.36E-11	0.87819789
Cdh26	0.026104	0.8781852
Col6a1	2.28E-12	0.87818233
Piezo1	7.79E-20	0.87809181
Ccl6	7.26E-59	0.87744102
Cct2	6.20E-13	0.87736372
Nmt1	6.15E-16	0.87707747
Itgb1bp1	5.05E-05	0.87648283
Cdc25b	6.10E-06	0.87644404
Fbxo22	9.45E-06	0.87585999
Bicd2	3.72E-09	0.87572857
B3gnt8	0.007262	0.87458019
C3	3.69E-76	0.87437518
Cdk16	2.17E-08	0.87415242
Clec1a	1.78E-27	0.87374875
Slfn5	3.28E-18	0.87373751
Angptl2	0.001391	0.87308134
Adrb1	2.07E-11	0.87305057
Lims2	9.21E-20	0.87289547
Tmcc2	1.24E-09	0.87276353
Nudt2	0.005335	0.87245415
Ranbp1	3.72E-09	0.87212706
Oplah	0.000524	0.87179951
Dvl2	0.020884	0.87161674
Mcemp1	8.72E-11	0.87121962
Dmap1	0.002962	0.87100263
Fgd1	0.031063	0.87018601
Sycp3	0.022032	0.86925146
Mrpl22	0.00012	0.86826485
Rps6ka2	2.39E-10	0.8678669
Iqcc	0.016605	0.86786355
Lsm6	5.04E-08	0.86720196
Gm16272	0.039149	0.86703211
[illegible]30028I04R	0.035416	0.86666073

Nynrin	0.000319	0.86647342
Zfp358	0.002953	0.86592879
Gm29538	0.001037	0.86471236
Brf2	0.02871	0.86379035
Wbp1	0.000295	0.86360403
Tcea3	5.81E-08	0.86341706
Fbxl14	0.000258	0.86291237
Mmrn2	1.59E-06	0.86195404
Ltb4r1	0.019928	0.86011215
Tbc1d20	3.68E-08	0.86008053
Upf1	1.28E-05	0.85992534
Zfp830	0.001605	0.85956473
Atp5mpl	2.81E-21	0.85953824
Ripor1	4.03E-09	0.85927594
Rasgrp2	4.70E-06	0.85921087
700102P08R	0.032141	0.85915914
Mrfap1	5.45E-12	0.85905891
Rgs2	2.19E-05	0.85891095
Hist1h1c	2.16E-08	0.85836033
Icmt	0.002928	0.85802019
Plp2	0.000181	0.85770647
Hlf	2.63E-13	0.85743039
Rps15	1.68E-52	0.8567985
B3galt6	0.042266	0.85633138
530039A03R	0.022225	0.85511111
Tm6sf1	3.59E-15	0.85474635
B4gat1	0.002599	0.85451325
Slc35b2	0.001025	0.85441484
Osgin1	0.002376	0.85440669
Mtx1	0.000218	0.85422323
30548M08R	2.62E-11	0.85422319
Nrgn	0.035139	0.85394788
Tnfrsf25	0.032261	0.85385562
Bhlhb9	0.004642	0.85363917
Acp6	0.000657	0.85350196
Aip	2.15E-05	0.85317551
Fam124b	0.029434	0.85317035
Ccdc61	0.000141	0.85293906
Wbp2	1.87E-10	0.85272532
Ston1	9.84E-11	0.85239253
Rhbdf1	0.000753	0.85232813
Iglc2	2.49E-05	0.85225595
Rplp1	5.03E-113	0.85186838
Rpp30	0.001039	0.85129257
Jcad	2.69E-20	0.84977299
Psmb3	2.32E-12	0.84949407
Mydgf	9.80E-06	0.84946335
300002I08R	0.002651	0.84936985
Tmem128	0.000113	0.84931678
Txndc15	3.14E-07	0.84916694
Mxd4	3.60E-23	0.84847479
Trmt10b	0.001138	0.84842689
Matn2	6.47E-05	0.8483962
Fam210b	0.001379	0.84715661
Kri1	3.81E-05	0.84713638
Cyc1	8.07E-06	0.84672586
Ramac	0.000114	0.8467017
Trmt10c	0.004905	0.84636188

Itm2b	9.55E-59	0.84535095
Pop7	0.001287	0.84522231
Stk16	5.23E-05	0.8450945
Ccdc9b	0.000411	0.84487375
Mpv17	8.84E-05	0.84369506
Ndufa9	1.48E-05	0.84356627
Rrp7a	0.000558	0.84335044
Cbr3	0.014295	0.84271626
Mrps26	0.000218	0.84259611
Ipo4	0.023391	0.84246614
Set	1.11E-15	0.84233355
Fuom	0.000133	0.84211122
Thumpd1	0.000776	0.84105603
Itga5	6.98E-10	0.84070649
Zhx1	1.20E-05	0.83974947
10001G20F	0.003092	0.83922454
Tek	1.05E-40	0.8380257
Kat8	0.000547	0.83785306
Cald1	7.98E-29	0.8372918
Surf6	0.003351	0.83637507
Emc9	0.012711	0.83615408
Ehd2	2.24E-15	0.83586155
Cxcl14	0.019907	0.83572903
Rac1	6.16E-28	0.8354663
Clec14a	1.33E-09	0.83447137
Aamdc	2.42E-05	0.83413981
10034E14F	3.93E-06	0.83342043
Rnase4	7.54E-37	0.83294582
Nes	3.55E-05	0.83187949
Jup	7.50E-18	0.83151019
Gpkow	2.75E-05	0.83128092
Hr	0.020037	0.83125419
Arhgef10	1.01E-06	0.83093296
Nsg1	0.001673	0.83089118
Commd6	2.48E-05	0.83034028
Eng	1.04E-24	0.83025042
Orc6	5.59E-07	0.83015248
Trappc5	6.31E-05	0.8295954
Vps51	0.016662	0.82919634
Asnsd1	4.12E-05	0.82907504
Aldh9a1	1.43E-05	0.82902782
Prdx1	6.95E-41	0.82882942
Bckdk	0.001831	0.82857465
P2rx7	3.40E-08	0.82853563
Coa5	0.000338	0.82838948
Wdr74	0.031047	0.82768475
Crnkl1	8.25E-06	0.82766454
Zfp414	0.007318	0.82758628
BC031181	1.40E-08	0.82647074
Ndufaf1	0.031026	0.8264489
Abcb10	0.000162	0.82632501
Cnppd1	2.21E-06	0.82618678
Mrps23	0.000172	0.82565646
Sirt4	0.022451	0.82560581
Trp53i13	0.001641	0.8255018
Bloc1s4	0.043604	0.82527684
Lsm8	0.00016	0.82518209
BC005624	1.15E-08	0.82496462

Clic1	2.24E-28	0.82471632
Hmgb3	0.023726	0.82457915
Gm15787	0.000805	0.82427902
Ddx21	1.06E-11	0.82420652
Akap12	1.68E-08	0.8232063
Zer1	0.001785	0.82280035
Ifit3	0.013247	0.82152091
Dhrs7	1.45E-10	0.82116666
Ogfod3	0.030969	0.82050022
Jpt1	5.88E-13	0.82023204
Plet1	1.58E-29	0.81986762
Axin2	0.000842	0.81929399
Rap2b	2.55E-07	0.81921614
Naca	1.67E-35	0.81815643
Sfmbt2	0.000629	0.81797574
Rxrb	0.000102	0.8174055
Mmp28	1.11E-07	0.81731714
Nucb1	1.80E-10	0.81683708
AU040972	3.36E-13	0.81598712
Snw1	5.56E-12	0.81582698
Lin37	0.027003	0.81550305
Taf7	0.001871	0.81546811
Ccdc59	8.43E-08	0.81471332
Fxyd6	0.001267	0.81406095
Nfyb	0.00062	0.81379485
Hoxb4	0.000786	0.81375217
Nol12	0.000377	0.81348409
Bmp6	5.34E-24	0.81302228
Nagk	2.96E-08	0.81267984
Mrps18b	0.004208	0.8126004
Zmat5	3.67E-05	0.81259773
Nme9	2.94E-05	0.8124454
Mical1	0.000152	0.81174045
Sox17	6.39E-07	0.81125827
Plxnb2	1.86E-09	0.81124984
Ccdc163	0.029719	0.81024694
Vti1b	2.15E-12	0.80993058
Dek	1.42E-15	0.80956337
Taf6	0.000449	0.80918671
Dyrk1b	0.0034	0.80783834
Mrpl41	2.58E-05	0.80768337
Nradd	0.003509	0.80729069
230032D23F	0.004396	0.80700305
Plpp1	1.00E-33	0.80689191
Col5a1	0.049832	0.80631675
Rbm15b	0.000654	0.80417722
Klf4	2.65E-23	0.80411187
Bad	3.23E-05	0.80261887
Ufm1	2.00E-07	0.80205326
Parvg	0.003126	0.80187074
Sult1d1	0.000407	0.80147543
Aqp5	4.16E-10	0.80132731
Vps72	8.39E-06	0.80128079
Tle2	0.000331	0.8011807
Poldip2	2.31E-06	0.80106125
Mvp	5.39E-08	0.80044827
Dnajc30	0.035236	0.79921478
Zc3h15	5.80E-12	0.79784028

Clec4n	1.01E-09	0.79781475
Capn2	1.69E-13	0.79767879
Fam107a	0.010882	0.79639063
Bag6	5.76E-05	0.79595522
Lage3	4.63E-05	0.7957572
Pfkl	0.006737	0.79565834
Prr18	0.001086	0.79553305
Trem2	0.008916	0.79505712
Lrrc14	0.001027	0.79456049
Pdgfb	9.24E-14	0.79437654
Cox6a1	1.65E-21	0.79419389
Dok3	0.00012	0.79332004
Mphosph8	8.54E-13	0.79126788
Smpdl3a	9.45E-15	0.79101668
Fam162a	7.20E-08	0.7906446
Czib	0.000262	0.7905537
Gm2245	0.01694	0.79039446
Nipal3	1.96E-06	0.79035701
Tspan7	4.55E-32	0.79014095
Hspb11	2.57E-05	0.78969119
Eny2	7.86E-10	0.78960538
Agr2	4.70E-05	0.789278
Rnf126	0.018265	0.78902445
Dpt	0.006727	0.78883709
Scube1	0.000259	0.78799857
Cdr2	7.51E-05	0.78797637
Fkbp3	8.83E-06	0.78703799
Cyba	3.17E-27	0.78697992
Saraf	5.67E-11	0.78673714
Ubald2	1.00E-06	0.78657966
Tmem176a	7.96E-22	0.78618142
Pde4a	0.00665	0.78436382
Ttc4	4.70E-05	0.78371165
Dync1i2	1.80E-28	0.78354016
Colgalt2	7.82E-07	0.78334655
Cbfa2t3	2.95E-18	0.78321626
Mrps15	5.25E-05	0.78238211
Tmem107	0.000115	0.78211536
Art3	0.044619	0.78179115
Id1	2.38E-10	0.78171303
Pgam1	3.94E-08	0.7813381
Scp2	1.12E-26	0.78058896
Mrpl39	0.005003	0.78022384
Phax	3.55E-05	0.77964015
Ebna1bp2	6.83E-05	0.77951113
Cbr2	7.05E-163	0.77938312
Smagp	9.83E-06	0.7791274
Grhpr	0.02875	0.77912242
Shfl	0.001193	0.77892182
Ampd3	4.67E-06	0.77783855
Gpi1	2.88E-16	0.77752413
Dpf2	1.53E-08	0.77750727
Csrp1	5.66E-13	0.77711348
Gdpd3	6.84E-06	0.77655077
Dele1	4.11E-05	0.77610267
Myh7	0.020023	0.77585293
Usp1	1.30E-05	0.77556239
Psmd12	7.59E-08	0.77388613

Larp7	4.13E-05	0.77312517
Cd300ld	0.042183	0.77290497
Dapk3	3.83E-05	0.7727844
Zfp747	0.019713	0.77228267
Dnaja1	9.20E-30	0.77164172
Nipsnap1	0.004761	0.77079557
Wbp11	6.27E-09	0.77074356
Ces2g	0.035912	0.77039691
Ly6a	7.87E-19	0.77035075
Scaf1	3.74E-08	0.77020046
Adcy4	2.65E-06	0.7697219
Ado	0.000179	0.76901672
Adgra2	1.52E-05	0.76879913
Gtf2ird1	6.78E-08	0.7687165
Cpne5	6.11E-05	0.76825785
Gm48321	0.031274	0.76817041
Lysmd2	0.000214	0.76705626
Ccdc167	0.030967	0.76663359
Selenon	8.31E-05	0.76654851
00026A02R	1.16E-11	0.76634764
Hmcn1	9.55E-38	0.76545825
Mrps17	1.34E-08	0.76491818
C1qbp	4.88E-06	0.76378156
Dcxr	3.37E-07	0.76314365
Cog1	0.00036	0.76241119
Cyb561a3	1.05E-12	0.76109743
Exoc8	0.009318	0.76101063
Tmem199	0.002623	0.76026636
Mxra7	8.89E-10	0.75917626
1-Sep	0.000219	0.75916894
Scrn1	0.01498	0.75908535
Hectd3	0.006824	0.75902522
Map2k2	5.49E-08	0.7589443
Mak16	0.000849	0.75881513
Stard9	2.45E-26	0.7587818
Micos10	1.54E-16	0.75835005
Psmd6	4.69E-07	0.75756772
Capn5	0.003225	0.75736756
Itgb4	0.000427	0.7572254
Anapc7	0.003982	0.75719765
Pigp	1.14E-05	0.75611485
Sesn2	0.001783	0.75604056
Slc12a4	0.000191	0.75568868
Bnip3l	1.62E-18	0.75534342
Ruvbl2	0.000797	0.75515679
Arl10	0.000402	0.75384586
Snx33	0.008994	0.75329485
Tmem147	5.35E-06	0.75187182
Mlst8	0.006316	0.75164834
Trappc3	1.74E-05	0.75158922
Gm26590	0.007712	0.75155757
Adamts2	9.79E-05	0.75122091
Slc16a3	0.008743	0.75089491
33412E12R	0.049211	0.75059303
Myo1c	2.62E-11	0.74985257
Gm17018	2.21E-06	0.74970082
Mettl3	0.002123	0.74935867
Gpc3	5.87E-37	0.74932736

Ltbr	6.99E-05	0.74895707
Npepl1	0.000581	0.74878798
Pno1	0.005635	0.74878401
Tmem219	4.76E-08	0.74816886
Pdcl3	4.17E-05	0.74804543
Hmgcl	3.35E-06	0.74782844
Slc25a11	2.27E-05	0.74779035
Gtf2a2	1.64E-08	0.74774373
Tomm22	1.94E-07	0.74719212
Gm10501	0.004092	0.74715353
Prdx2	1.26E-10	0.74604859
Srgn	2.84E-15	0.74582347
Fam3a	0.000118	0.74475416
Mfap4	1.89E-09	0.74456909
Mfsd10	0.000565	0.74433702
Ddah2	0.000272	0.74363704
Urm1	0.002826	0.74307223
)30037H05I	0.000667	0.74300905
Mrps25	0.000392	0.74260466
Npc2	8.47E-48	0.74251241
P3h1	0.003109	0.74212487
Setd4	0.004528	0.74192534
Ppp5c	0.000519	0.74132092
Unc45b	0.000774	0.74105436
Hmgn1	1.09E-15	0.74085347
Cyp4b1	6.62E-11	0.74020267
Gm15265	0.045531	0.73977264
Shank3	3.23E-24	0.73948882
30554G24F	1.49E-09	0.73941347
Epha4	7.37E-16	0.73912997
Dctn2	1.37E-06	0.73901154
Zbtb8os	3.71E-05	0.73843938
Ephb4	3.22E-11	0.73822737
Pdlim3	0.032331	0.73814749
Fxyd1	0.000194	0.73683041
Atxn1l	0.001124	0.7367815
Pop4	0.042388	0.73676311
Nup85	0.001402	0.73650429
Gm44148	0.000102	0.73620548
Myzap	1.10E-19	0.73507618
Crip2	1.04E-18	0.73491413
Uxt	0.024524	0.73430545
Nom1	3.25E-06	0.73428032
2-Mar	3.95E-08	0.73412601
Zdhhc8	0.001531	0.7341034
Psmc2	3.17E-07	0.73386914
Smim5	0.000861	0.7338051
Lsm5	0.000938	0.73367796
Nudt14	0.004173	0.73367261
Hvcn1	1.58E-09	0.73362848
Plin2	2.35E-22	0.73358849
Zfp54	0.043382	0.73352924
Arl1	1.31E-07	0.7331853
Ptpmt1	0.000576	0.73302236
Efnb2	1.61E-17	0.73268246
Smpd4	0.003967	0.73257534
Kbtbd4	0.017052	0.73215756
Gm15559	0.009704	0.73188381

Coro2b	5.44E-11	0.73184365
Ppm1e	0.005938	0.73152145
Fundc2	1.44E-05	0.73100128
Snrpc	0.000165	0.730322
Rnf5	0.011105	0.72988044
Magoh	4.67E-05	0.72934692
Stk40	4.53E-07	0.72927935
H2-Ke6	0.000275	0.7292188
Ftsj3	0.004267	0.72915467
Gm17167	0.035314	0.72905099
Cemip2	9.56E-33	0.72826146
Nr6a1os	0.00286	0.72791645
Psmb4	1.17E-14	0.7264212
Mrpl46	0.016179	0.72640829
Tnxb	6.18E-05	0.72631638
Pxdn	3.60E-07	0.72629434
Tspyl4	0.019122	0.72594076
Stx19	0.000534	0.72429644
Fam92a	0.000302	0.72396649
Ncapd3	3.99E-05	0.72288241
Gm49767	0.013959	0.72208852
Cib2	0.002072	0.72194132
Pip5k1c	2.61E-07	0.72124728
Alad	7.95E-05	0.7203914
Bcar1	0.000851	0.72008319
Mis12	1.93E-05	0.71976401
Scly	0.000202	0.71919299
Rnf7	7.34E-08	0.71918405
Igfbp5	9.67E-77	0.7188905
Cdc42se1	4.03E-05	0.71864902
Ddx27	0.000396	0.71841821
Ccdc71	0.017559	0.71836463
Kars	6.81E-05	0.71832938
Mrps10	0.000168	0.71746329
Ddx23	1.25E-06	0.71732972
Slc38a10	4.40E-06	0.71696661
Ccdc78	0.002921	0.71663886
Clmp	0.001496	0.71645531
Chmp6	0.020761	0.71614837
Sart1	2.35E-06	0.71569859
Lrrc47	0.006972	0.71529221
Apba3	0.033373	0.71527269
Fam32a	3.33E-07	0.71472401
Akr1a1	6.78E-19	0.71436285
Gxylt2	0.00123	0.7143335
Xpc	0.000987	0.71418259
Tmod2	1.93E-05	0.71373544
Ctbs	0.008944	0.71313293
Smad7	5.27E-13	0.71297277
Csk	8.01E-07	0.71271722
Dpy30	2.47E-08	0.71259502
Rhoj	6.99E-24	0.71234071
Agpat2	0.010137	0.71126656
Mettl5	0.004884	0.71091999
Fmnl1	1.52E-05	0.71039079
Ciao2a	3.07E-05	0.71030676
Sncaip	6.56E-05	0.70974066
Zfyve21	0.000525	0.70963468

Calr	1.29E-24	0.70958718
Slc35e4	0.007279	0.70954378
Stmn1	1.14E-10	0.70927469
Cdk4	0.000359	0.70891324
Cd14	0.00048	0.70870609
Ifi35	1.67E-07	0.70832557
Pja1	0.007625	0.70689284
Fcgrt	1.56E-08	0.70685143
Gm16136	0.014445	0.70642188
Twf1	1.04E-06	0.70554141
Zbtb7a	1.12E-12	0.70540199
Brat1	0.003843	0.70465487
Psmg4	0.001565	0.70446198
Dbndd2	0.000638	0.70415697
Timm9	0.000771	0.70335285
Adgrl1	9.50E-06	0.70302306
Adamts10	5.72E-10	0.70254096
510030E20F	1.60E-06	0.70181183
Nars	4.51E-09	0.70158259
Srsf9	6.85E-07	0.70113801
Hagh	0.000668	0.70096116
10062O18F	0.006629	0.7007568
Mpeg1	3.33E-28	0.70068082
Pcyox1	2.11E-08	0.70050105
Sar1a	1.27E-07	0.70042877
Elp5	0.000444	0.70037462
Mgat4b	1.04E-07	0.6996095
Copb2	3.07E-08	0.69950936
Ndufb5	1.25E-10	0.69926678
10454H06F	0.027351	0.69812176
Cotl1	2.07E-15	0.69701633
Suclg1	6.77E-07	0.69678191
Fmo2	1.41E-47	0.69643506
Qsox2	0.006299	0.69639155
Cyth2	0.002862	0.69616479
Col3a1	5.22E-14	0.69615971
Timp2	5.02E-33	0.69582515
Grasp	0.002139	0.69580631
Amdhd2	0.001595	0.6955079
Abcg1	6.82E-28	0.69543307
Rnh1	2.04E-12	0.69496965
Echdc2	0.025341	0.69480039
Nxt1	0.013675	0.6947966
Nid1	4.49E-05	0.69430153
Clcn4	8.70E-07	0.69307475
Rnf10	6.56E-09	0.69151952
Trim35	8.94E-10	0.69123067
Gnao1	1.42E-06	0.69104982
Mrps18a	0.000291	0.69102296
Ramp1	2.00E-08	0.69099825
Thsd7a	0.000338	0.69067934
Tada2b	0.028197	0.69011543
Sh3d21	0.015734	0.68953844
Tpgs1	0.003715	0.68928165
Zbtb9	0.002953	0.68842204
Arglu1	5.24E-18	0.68774981
Prdm11	2.72E-05	0.68768603
Hes6	0.001291	0.68726751

Adgrl4	1.54E-40	0.6870267
Asna1	0.006479	0.68694275
Det1	0.011675	0.68692355
Gm26787	0.03021	0.68682674
Mpped2	5.20E-07	0.68640445
Ncln	5.55E-05	0.68633537
Vat1	3.88E-08	0.68572501
Ywhab	1.42E-18	0.68563104
Rin3	1.48E-20	0.68543862
P3h3	0.011569	0.68439145
Bag1	7.60E-18	0.68433459
Gipc1	0.010827	0.68425745
Rmnd1	0.004229	0.68376535
Scimp	0.010175	0.68304527
Cacnb3	0.015989	0.68287833
Hpgds	0.006213	0.68266306
Hspa2	0.001531	0.68178369
Nbn	8.25E-05	0.68166574
Fzd2	0.017845	0.68136708
Lman2	3.88E-09	0.68131014
Myl4	0.023575	0.68112022
Zdhhc5	5.83E-06	0.68080437
Vkorc1	5.32E-05	0.68054774
Sec13	7.29E-05	0.67992634
Anp32b	1.62E-14	0.67991975
Atxn7l3b	9.35E-14	0.67991671
Ntmt1	0.00386	0.67976046
Pacc1	0.025985	0.67951604
Gm13919	0.004211	0.67919446
Cebpz	2.07E-09	0.67904402
Hsd11b1	0.000265	0.67877102
Drg2	0.006401	0.67876011
N6amt1	0.009954	0.67815026
Reep5	1.91E-26	0.67776827
Gm12258	0.021331	0.67740313
Tmem18	0.009306	0.67713957
Cdk2ap1	0.020366	0.67671067
Synm	0.004547	0.67622681
Tfpi	9.73E-06	0.67607779
Cthrc1	0.018449	0.67534568
Uqcrfs1	1.87E-06	0.67530313
Irak1bp1	0.005251	0.67524763
Sema6d	2.00E-14	0.67508706
Mal	4.35E-05	0.67506823
Dysf	9.50E-10	0.67502164
Fgfr3	5.70E-07	0.67444598
Mill2	2.30E-16	0.67429605
Arrb1	1.18E-11	0.67418628
Rps6ka1	2.15E-06	0.67347072
Napa	1.72E-05	0.67336311
Cby1	0.016397	0.67323004
Zfas1	0.002168	0.67316929
Pld3	2.76E-11	0.67217422
Lsp1	8.12E-15	0.67217289
Sntn	3.42E-06	0.67209742
Pias4	0.001408	0.67204189
Dus4l	0.016321	0.67187127
Gsta3	1.02E-16	0.67168104

Tagln2	5.98E-20	0.67153721
Npr3	0.003087	0.6714274
Cdc42bpg	0.000361	0.67122415
Nudt7	7.33E-05	0.67106477
Zxdb	0.0181	0.67086887
Psmd3	1.21E-05	0.67076124
Chchd4	0.001706	0.67056559
Lyve1	0.000276	0.66872726
Lipa	7.45E-13	0.66857233
Mras	0.000154	0.66854079
Rnasel	0.000108	0.66851048
Manf	6.30E-13	0.66830485
Aste1	0.000195	0.66828027
Scyl1	0.002293	0.66821633
Ccdc9	0.001173	0.66706369
Map7d3	0.008588	0.66593026
Gde1	1.97E-09	0.6658952
Metap2	1.71E-14	0.66585945
Hspd1	5.50E-15	0.66549356
Pea15a	1.07E-08	0.66412206
Me3	0.013719	0.66384842
Tgfb1	3.74E-10	0.66375029
Pkn3	0.000636	0.66338381
Lrrc74b	0.033378	0.66291217
Casp1	0.002931	0.66206798
Chchd7	0.002966	0.6616974
Igsf6	0.014776	0.66097149
Bend6	0.001537	0.66052069
Ldb2	3.53E-47	0.66041311
Hpgd	4.06E-14	0.65994663
Cfap97	7.86E-05	0.65922197
Tmem59	5.60E-14	0.6586121
Gm14966	0.002676	0.65854273
Fads1	0.000195	0.65846988
Zfp422	0.007126	0.65836134
Gm9929	0.02537	0.65820453
Cope	5.38E-07	0.65815294
Ccng1	6.68E-07	0.65787687
Ybx1	2.44E-20	0.65742906
Nostrin	8.81E-20	0.65741414
Themis2	0.000724	0.65739726
Wdr53	0.004621	0.6573022
Bin2	5.19E-06	0.65726863
Plekha8	0.000128	0.65697997
Calm2	1.92E-22	0.65620921
Dhrs7b	1.96E-06	0.65616857
Afmid	0.021147	0.65595585
Crat	0.001148	0.65593247
Cyb5a	6.01E-25	0.6556801
Gem	0.002394	0.65557795
Ccdc107	6.73E-05	0.65546134
Cfl2	2.19E-08	0.65511049
Dnpep	0.000806	0.65480435
Cep41	0.032037	0.65459823
Cdkn1a	8.16E-08	0.65422995
Zc3hc1	0.019907	0.65377258
Noa1	0.036169	0.65352584
Il17rd	6.08E-10	0.65324348

Atp5a1	2.63E-22	0.6527211
'00081O15F	0.000606	0.65265447
Tlr2	1.94E-05	0.65239583
Syngr1	0.022426	0.65199244
Camk2n1	0.000406	0.65162484
Spcs1	3.20E-10	0.65139167
Rccd1	0.016976	0.65110658
Reep6	0.025235	0.65091438
Bcdin3d	0.042833	0.65029054
BC005561	4.98E-07	0.6493936
Slc25a44	0.000651	0.64933022
Cdv3	5.46E-13	0.64908289
Robo4	0.000625	0.64889226
Snta1	0.005161	0.64831349
Sys1	5.42E-07	0.64829908
Nnat	0.001139	0.64787502
Polr1d	3.61E-10	0.64740507
Bscl2	0.000126	0.64725407
Sf3b2	1.86E-15	0.64696009
Ripply3	0.000302	0.64684011
Ddrgk1	5.85E-09	0.64609216
Zfp598	0.004282	0.64588995
Sult1a1	0.001725	0.64579237
Loxl2	0.028347	0.64523904
Pou6f1	0.000463	0.64491267
Snhg12	0.000868	0.64438628
Pcdh17	9.06E-14	0.64425021
Mkrn2	0.003276	0.64422658
Plekhb1	0.000916	0.64421895
Asap2	4.80E-15	0.64411758
Piezo2	4.45E-20	0.6422505
Ier2	2.41E-05	0.64183777
Sdhb	5.23E-05	0.64117953
Pdcd2l	0.019477	0.64043384
Ubc	1.15E-22	0.64031816
Sorbs2os	0.038943	0.64026769
Als2cl	8.48E-05	0.64022779
Prss12	0.002959	0.6399487
Stab1	0.000154	0.63985335
Coro1a	7.76E-11	0.63957105
)20021L13F	0.002769	0.63952442
Wfdc2	1.80E-62	0.63952372
Slco2a1	5.00E-40	0.63929601
Ccdc113	1.97E-05	0.63900588
Ethe1	0.035207	0.63843188
Emp2	3.54E-19	0.63787364
Emc6	9.22E-07	0.63721357
Gatad1	1.11E-06	0.63681617
Timm17a	2.57E-05	0.63608081
Mylk3	0.024766	0.6358892
Gtpbp3	0.031481	0.635638
Ccz1	6.11E-09	0.63560081
Kctd17	0.003447	0.63546225
Gm16091	0.002817	0.63535561
Smpd1	6.69E-05	0.63527702
Rasip1	1.82E-10	0.63519258
Tgfb3	0.007242	0.63490435
Ephx1	1.51E-11	0.63471403

Snx17	8.08E-06	0.63448156
Txnl4a	0.002092	0.63440676
Cox7a2l	7.35E-12	0.63420217
Brip1os	2.51E-07	0.63406314
Gm47644	0.008611	0.63349562
C5ar1	0.002797	0.63318209
Toe1	0.021101	0.6318123
Cited2	6.31E-06	0.63175096
Eci1	0.000183	0.63172191
Tmem64	0.002571	0.63149829
Ran	1.12E-10	0.63122503
Myrip	6.37E-07	0.63085007
210016F16R	0.000982	0.63015161
Aaas	0.032151	0.62966042
Rexo2	1.85E-08	0.62951329
Afap1l2	0.000175	0.628038
Eapp	1.66E-06	0.6274963
Rprm	0.029334	0.62651607
Ccdc97	0.009133	0.62639469
Tprkb	0.008529	0.62597975
Elk3	5.07E-23	0.62586414
Znrd1	0.000453	0.62568548
Slc16a11	7.84E-07	0.62546649
Jam2	3.48E-08	0.62542476
Phldb1	2.09E-06	0.62509763
Chil1	6.78E-21	0.62480606
Gucd1	0.000944	0.62429845
Ttc38	0.011936	0.62401595
Ints1	0.027914	0.62399264
Hcst	0.008368	0.62376722
Lrrc10b	0.002148	0.62328507
Prkcsh	3.11E-06	0.62294947
Csrp2	1.64E-05	0.62266899
Rnf113a2	0.04829	0.62224136
Fzd1	0.024376	0.62163728
Arl3	1.49E-07	0.62146822
Pllp	0.01153	0.62029825
Zfp267	0.021432	0.62011873
Tsacc	0.009704	0.61894644
Zkscan5	0.031672	0.61875532
Ddb1	2.79E-08	0.61839862
Cdc42ep2	0.014009	0.61819226
Timm22	0.014182	0.61768133
Sema3g	4.81E-07	0.61738088
C2cd2l	3.37E-05	0.61729082
Gar1	0.032212	0.61715506
330041H03R	0.014349	0.61709588
Rps6kb2	0.027612	0.61679367
Ptbp1	1.82E-07	0.61678833
Ccdc28b	0.002694	0.61639508
Il16	0.00446	0.61583959
Wrap73	0.003817	0.61547136
Agr3	0.003233	0.6154257
30017K11R	0.021206	0.61520367
Dhcr7	0.015789	0.61512618
Wnt5b	0.008297	0.61311667
Bbip1	2.64E-06	0.61249617
Pgam5	0.003211	0.61216691

Lpl	4.99E-29	0.61189868
Gm10125	0.007196	0.61154413
Atp5g3	1.72E-18	0.61102995
Cd2bp2	0.037819	0.61094965
Mknk2	6.69E-06	0.61073048
Cptp	0.045353	0.6104821
Dipk2b	2.50E-07	0.6098124
Bud23	0.006129	0.60946071
Akap5	1.23E-06	0.60936026
Lats2	1.17E-11	0.60891377
Rpsa	1.19E-37	0.60872114
Dusp8	0.016543	0.60871155
00002E11F	0.001653	0.60805353
Esrp2	0.028337	0.60792039
30430F08F	0.046981	0.60777415
Reep1	3.43E-07	0.6077116
Fbl	0.000287	0.60767693
Dtx3	7.00E-05	0.60736258
Mocs2	0.000819	0.60711195
Spock2	1.54E-09	0.6070835
Mkrn1	2.15E-06	0.60701847
Capns1	1.26E-12	0.60685051
Cfap36	9.86E-08	0.60633586
Gm10484	0.037773	0.60620423
C1s1	1.36E-08	0.6058075
Traf7	2.77E-06	0.60569013
Mtch1	6.80E-12	0.60486159
Pard6b	0.0044	0.60473059
Prr12	1.16E-05	0.60449358
Gulp1	9.82E-13	0.60425502
Ighm	1.29E-11	0.60422216
Prickle1	5.85E-17	0.6041526
Slc16a9	0.005079	0.60410408
Prkcg	0.000274	0.60377691
Cc2d1a	0.023355	0.60358207
Dph7	0.029293	0.60351306
BC051019	0.000234	0.60293135
Arrdc2	0.022389	0.60247547
Zdhhc4	0.019774	0.60140189
Stoml2	0.025324	0.60136081
Sod1	4.75E-19	0.60117572
Thoc3	0.006074	0.60093642
Chmp1b	0.000128	0.60044537
Hint3	9.08E-05	0.59996734
Polr1c	0.000581	0.59975199
Atp6v1g1	7.50E-19	0.59968334
Rufy1	2.86E-06	0.59917968
Tppp3	4.86E-20	0.59912466
Wdsub1	0.003242	0.59844066
Sec11c	6.75E-09	0.59842075
Gskip	0.000123	0.59813499
Med25	0.000807	0.59799175
AW112010	9.81E-10	0.59779614
Lmf2	0.001802	0.59773911
Espn	0.013538	0.59743457
Hdx	0.018881	0.59691553
Atp6v0e	7.50E-13	0.59663096
Alg5	0.000429	0.59651868

Zbtb17	0.026678	0.59618962
Rgs3	4.33E-08	0.59559193
Nfkbil1	0.019214	0.59523058
Tnks1bp1	1.49E-05	0.59517002
Rhpn2	0.000103	0.59509383
Coq10b	4.39E-06	0.59485521
Adgrf5	1.28E-29	0.59473084
Cggbp1	5.09E-08	0.59435098
Utp11	2.84E-06	0.59402356
Dguok	5.28E-05	0.59374829
H2-T24	0.001648	0.59347891
Kifc3	5.10E-07	0.59342519
Sulf1	3.93E-15	0.59296481
Nup43	0.032855	0.59289707
Vamp5	8.64E-09	0.59262969
Syap1	2.44E-05	0.59262235
Mapt	7.69E-17	0.5919208
Mtrr	0.045589	0.59148157
Gba	0.00056	0.59121915
Nqo2	0.002359	0.59099712
Vgll3	0.007642	0.59076225
Plk2	0.003801	0.59020939
Gm30382	5.55E-13	0.59005187
Gm7706	2.51E-05	-7.2770893
Nccrp1	0.000138	-7.0089643
Vsig1	5.88E-05	-6.7823057
Mefv	0.000327	-6.5622908
Ptgdr	0.00452	-6.3063525
Olfr57	0.000779	-6.2718692
Gm28187	0.011106	-6.2293018
Cnr1	0.001866	-6.0548714
Gm31107	0.00452	-5.9895651
Gm31392	0.000779	-5.9890327
30459C07R	0.000779	-5.9846759
Ccl1	0.011106	-5.9827366
Cacng4	0.00452	-5.9180587
Ccdc169	0.027886	-5.9063351
Gm10440	0.011106	-5.8599608
Vmn2r71	0.011106	-5.8299993
Ms4a14	0.001866	-5.818451
Gm48747	0.001866	-5.8173563
21504E06R	0.011106	-5.7549761
30440I19R	0.011106	-5.7514149
Luzp4	0.027886	-5.669719
Gm9774	0.011106	-5.667785
Gm47405	0.001866	-5.6465821
Cers3	0.011106	-5.6464162
Il13	0.011106	-5.6311662
Il17f	0.011106	-5.6300268
Gm12729	0.027886	-5.5800123
Gm44982	0.00452	-5.577438
Aox4	0.011106	-5.5561025
Gm15245	0.011106	-5.5197673
Gm17266	0.027886	-5.5166884
Xkr5	0.027886	-5.5136027
Gm13660	0.011106	-5.5092305
Clec4b2	0.027886	-5.45174
Gm9008	0.011106	-5.4498177

Hmga1b	0.027886	-5.3791654
Ptpn5	0.027886	-5.3635096
Calcr	0.027886	-5.3574574
Hormad1	0.027886	-5.34536
AU015336	0.027886	-5.2964636
30412B13F	0.027886	-5.2759207
Slc17a8	0.011106	-5.2663295
Lhb	0.011106	-5.2578797
Izumo1r	0.027886	-5.2531091
Il10	2.60E-05	-5.245544
Gm39383	0.011106	-5.2448586
Fndc3c1	0.027886	-5.2410065
Gm49086	0.027886	-5.2244457
Capn9	1.42E-08	-5.2131175
Gm49162	0.027886	-5.2056192
Fcrlb	0.027886	-5.2010143
Gm19272	0.027886	-5.1988104
30616G12F	0.027886	-5.1786964
Gm47214	0.027886	-5.1547193
Sv2c	0.027886	-5.1471486
Gm35657	0.027886	-5.1433399
Gm12953	0.027886	-5.14008
Ido2	0.027886	-5.1175415
Gm29340	0.027886	-5.1104925
Gm50019	0.027886	-5.0959991
Vmn2r113	0.027886	-5.0797654
Gm14102	0.027886	-5.0513013
Gm43016	0.027886	-5.0305048
Pla2g10os	0.027886	-5.0252187
00028N14F	0.027886	-5.0165457
30080O11F	0.011106	-5.0143356
Gm8378	0.027886	-4.9715857
Gm8953	0.027886	-4.9225446
11-Mar	0.027886	-4.8981877
Scn2b	0.027886	-4.8874625
Tfap2c	0.027886	-4.863923
Acod1	1.33E-17	-4.8234418
Gm26805	0.027886	-4.8181407
Gm35721	0.027886	-4.8022948
Gm13219	0.027886	-4.7889427
Dmrt1	0.027886	-4.7774184
Csprs	0.027886	-4.7560993
Mcpt4	0.027886	-4.7411004
Palm3	0.027886	-4.7406523
30432O09F	0.027886	-4.7277743
Olfr267	0.027886	-4.7216335
Wnt16	0.027886	-4.6679611
Gm11455	0.027886	-4.6521944
Ccdc36	0.027886	-4.6337723
Gm31693	0.027886	-4.6334729
Gm48522	0.027886	-4.6311897
Hmcn2	6.03E-05	-4.5715883
Pgbd5	0.027886	-4.4797804
Gm48202	0.027886	-4.4750175
Scin	1.12E-23	-4.4593537
F11	2.61E-05	-4.412697
30542D17F	4.97E-05	-4.2009147
Gm35769	0.001774	-4.0676822

Dcstamp	7.27E-11	-4.0669337
Slco1a6	0.001775	-4.0667582
Mpp4	0.001777	-4.0589733
Ocstamp	0.001777	-4.0582108
Gm14280	2.94E-07	-4.0355764
Lrrc7	0.009789	-4.0247233
Gm20632	0.00417	-4.0080184
Cxcl1	0.009805	-3.9852527
)20031H02F	0.009789	-3.982035
Ccl24	6.64E-15	-3.9803509
Trim30c	0.000141	-3.9359207
'00113H08F	0.02313	-3.9316844
Gm49223	0.009789	-3.9167603
Nrg3	0.009789	-3.8857193
Cldn2	0.00178	-3.8801392
Gm6209	9.03E-14	-3.8647818
Gm10685	0.023149	-3.8269119
Gm47662	0.02313	-3.7354658
Qprt	0.023149	-3.6462846
Slc26a4	1.26E-165	-3.6176948
Ms4a4a	0.02313	-3.6113878
Il1f9	0.002947	-3.5681717
Gm48099	6.39E-85	-3.5664414
Gm50061	0.023149	-3.5134811
Gm32391	0.02313	-3.4950992
)30032O16F	7.46E-06	-3.4942742
Il2	0.009814	-3.4472682
Adra2a	5.00E-07	-3.4345663
Grid1	1.08E-10	-3.4320474
Gm5432	0.000179	-3.3971564
Fer1l6	2.64E-65	-3.3776293
Gm20711	0.000181	-3.3758181
Gm45740	0.000114	-3.3722309
Tnip3	2.39E-27	-3.3545732
Vmn1r13	2.42E-06	-3.3497623
Naa11	0.023168	-3.3373545
Chil4	4.81E-66	-3.3325583
Asxl3	7.18E-11	-3.3116324
Gm14643	0.001309	-3.2979646
Cd209f	0.000328	-3.293697
Iqcm	0.001307	-3.2911698
Vsig4	0.00018	-3.2846741
Penk	0.000398	-3.2708237
230112J17R	0.000181	-3.2529031
)30441J16R	0.009814	-3.2492495
Gm27188	0.000258	-3.2325025
Aspg	0.004085	-3.2284889
'00110K17F	0.009822	-3.2187156
Hs6st3	1.01E-135	-3.2184545
Zfp804b	7.53E-12	-3.2136095
Ankrd55	1.65E-07	-3.2123434
Srrm4	0.023168	-3.2096339
Trpc4	0.023168	-3.207374
Gm33251	0.006602	-3.1983058
Gm17749	0.000399	-3.1982128
Gm28404	0.006592	-3.1920457
Mc1r	3.43E-07	-3.1841316
)30017D23F	0.001309	-3.1823412

D7Ertd443e	0.009822	-3.1745689
10053B23F	0.00417	-3.1609629
Cdh8	0.004177	-3.1604874
Olfr111	2.39E-11	-3.1562396
Zbtb8b	0.023149	-3.1445116
Cxcl5	7.89E-06	-3.1360236
Ccdc150	2.19E-07	-3.1315024
Gm33843	1.15E-07	-3.1181532
Vmn2r96	3.59E-05	-3.1151331
Gm30849	0.006606	-3.0914984
Tarm1	3.63E-05	-3.0881268
Ptprt	1.12E-15	-3.0744124
Vmn2r103	0.009822	-3.0691432
Rnase2a	3.62E-05	-3.0446795
Gm28905	0.000873	-3.0438493
Mmp12	1.31E-162	-3.0358298
530023F18F	0.023149	-3.0109494
Gm46332	0.014707	-3.0078994
Gm2788	0.023187	-2.9867195
Ppp1r3a	0.03246	-2.9857684
Egf	0.000874	-2.9845757
Prss35	0.014676	-2.984508
Dscam	0.00409	-2.9774597
Lin28b	0.004096	-2.9738847
Gm33525	0.009822	-2.9702257
Gm44949	0.008786	-2.9615537
Slc38a11	3.68E-06	-2.9577229
Gfi1	0.002956	-2.9538865
Dock3	0.000123	-2.9478311
Il1a	2.26E-07	-2.9347195
Sorcs1	0.000874	-2.9186331
Gabrb1	0.000259	-2.9063264
Gm37381	1.88E-09	-2.904804
Lrr1	0.00983	-2.9009998
Acp7	0.014738	-2.9005452
Fam83f	7.94E-06	-2.8937022
530052E02F	9.81E-07	-2.8810147
Sntg1	9.57E-16	-2.869204
Gm28307	2.35E-24	-2.8543553
Ptpn20	0.000259	-2.8480741
Clca1	1.59E-251	-2.8472607
Gm34045	0.023168	-2.8461439
Gm5860	0.004181	-2.8168131
Htr7	7.68E-19	-2.8166207
Spock1	0.000552	-2.8074516
St18	7.52E-07	-2.8074062
Dgki	0.023206	-2.7986595
Klhl14	1.28E-06	-2.7869444
Ctcflos	0.002984	-2.7869428
Oas1g	0.014697	-2.7838472
Gpc5	0.002953	-2.7834762
Sult2a8	0.014697	-2.7803164
Phex	2.58E-05	-2.7766655
Gatm	5.39E-15	-2.7648885
532411P08F	0.006616	-2.7599984
Cdc20b	4.76E-07	-2.7515999
Camk1d	0	-2.7500514
Kcnq3	0.004112	-2.7441204

Abca13	0.008775	-2.7335717
Col24a1	5.60E-05	-2.7310493
700029J03R	1.56E-06	-2.7279009
Hcn1	0.006022	-2.7265973
Gm43024	5.16E-06	-2.7237688
Crhr2	0.023187	-2.7213067
Eid3	0.014717	-2.7043856
Olfr171	4.31E-05	-2.6921708
AA467197	5.01E-43	-2.6906343
Agbl4	9.85E-52	-2.6892606
Dpp6	6.10E-32	-2.6878664
Trpm2	8.00E-06	-2.6833784
Inhba	1.58E-16	-2.6821596
Asb14	2.20E-09	-2.6818305
Gm15859	1.09E-05	-2.6725143
Slc7a11	7.50E-28	-2.6646971
930512J16R	0.00146	-2.6607652
Sdk2	0.032548	-2.6406465
Tgtp1	1.28E-05	-2.62965
Gpr176	0.001469	-2.6273564
Gm15958	0.032504	-2.6272379
Oas3	0.038822	-2.620639
Dcc	0.002972	-2.6191244
Tacr1	4.64E-29	-2.6047453
Gm47814	0.023187	-2.5976121
Grik1	2.20E-31	-2.5853856
Arg1	1.97E-09	-2.5799487
Atp2b2	2.84E-15	-2.5789777
530023P12R	0.018641	-2.5786417
Ctla4	9.26E-09	-2.5732534
Galntl6	3.74E-49	-2.5722197
Gm36107	0.032548	-2.5682865
Mroh8	0.014738	-2.5620008
Fam228b	0.001731	-2.5562216
Sh2d1a	0.000292	-2.5393748
Gm26535	0.004131	-2.5392872
30422H06R	5.42E-06	-2.5388081
Kcng3	0.032548	-2.5385605
Fabp7	0.038868	-2.5318214
Gm36486	0.010596	-2.5254222
Sgcz	0.00172	-2.5235318
Fbp1	5.15E-09	-2.5224871
Myo18b	0.008803	-2.5220853
Slc6a20a	6.21E-37	-2.5209237
Gm38575	0.000208	-2.5088921
Gm1604a	0.014759	-2.5075512
Fat3	7.46E-58	-2.5047061
Il2ra	1.16E-09	-2.4951135
Gm49497	0.032504	-2.4929007
Chl1	4.62E-09	-2.479424
Mcidas	0.032592	-2.475757
Plch1	6.33E-27	-2.472323
Syngap1	7.44E-05	-2.4661744
Gm42705	7.81E-05	-2.4511568
Slc22a22	1.92E-12	-2.4473934
Slco5a1	0.01064	-2.4466811
Platr16	0.010627	-2.4455235
Gm10415	1.58E-08	-2.4430411

Gm37168	5.93E-07	-2.4397533
Gm50306	6.17E-05	-2.4341278
Hectd2	6.87E-12	-2.42431
Tmem267	2.21E-05	-2.4110909
Mapk4	0.007618	-2.4041125
Eldr	0.001914	-2.4000492
Naaladl2	6.05E-264	-2.397744
Cnga2	0.006631	-2.3964824
Rimkla	0.005166	-2.3927536
Gm44767	0.03257	-2.3864693
Asb15	0.032548	-2.3821244
H2-M2	6.04E-08	-2.3819652
)30016O22F	0.021488	-2.3761191
30502A04F	2.61E-05	-2.3657184
Irf4	3.61E-10	-2.36379
Gm42722	3.77E-27	-2.3635208
Gm32061	0.014749	-2.3608045
Gbp2b	0.03257	-2.3573443
Gm19951	5.28E-32	-2.3465528
Slc1a2	0.00043	-2.3441152
Aqp9	0.000297	-2.3424999
330012L14F	0.038913	-2.3402706
Ccr5	5.44E-25	-2.3367238
Mccc1os	0.023352	-2.3331636
Cib4	0.032614	-2.3328458
Il23r	0.001126	-2.3299081
33405D12F	0.000293	-2.3287752
Gm44662	0.03257	-2.3158419
BC147527	0.018653	-2.315625
Mab21l3	7.82E-06	-2.315399
AI429214	0.03257	-2.3149026
Gm14051	8.67E-15	-2.3109111
Gm15564	1.13E-106	-2.3093434
Gm26901	0.001003	-2.3058786
Gm15675	6.13E-25	-2.3045338
Ccdc148	8.13E-10	-2.3028471
Klhl32	1.61E-10	-2.298591
30567H12F	0.042705	-2.2985557
Vat1l	0.018688	-2.2951996
Gm765	0.000149	-2.2951058
Gm12296	0.000347	-2.2891807
Gm20110	0.021512	-2.2889583
Slc27a6	0.021464	-2.2888842
Dmrt2	0.00665	-2.285124
Cdh10	0.038936	-2.2755462
Slc26a8	0.010608	-2.2723452
Gm49980	1.11E-16	-2.2715532
Ifi205	0.000508	-2.2654119
Syt9	0.002163	-2.264343
Gm10062	1.47E-07	-2.2601
Impg2	0.000131	-2.2599076
Acot6	0.018769	-2.2535093
Slc44a5	0.001186	-2.2517229
Ccl3	0.010621	-2.2501843
Nkain3	2.57E-05	-2.2501749
Itgb8	3.88E-07	-2.24883
Gm28638	0.001	-2.245678
Slc5a1	3.08E-11	-2.2415283

Gm48357	0.006734	-2.2415252
Timd4	2.44E-09	-2.2410023
Cfap61	4.45E-26	-2.2351302
Arg2	8.33E-14	-2.2345246
Usp9y	0.021488	-2.2308527
Gm41625	0.03257	-2.2257204
Serpinb2	0.000256	-2.2218596
Pakap	2.33E-21	-2.218437
Frmpd1	3.40E-05	-2.212544
Arhgef33	0.000193	-2.2060022
Aoah	7.12E-08	-2.2001248
Tnfrsf13c	0.0041	-2.1991409
Gm30667	0.042705	-2.1961414
Galnt17	0.00015	-2.192639
Gm36264	0.001128	-2.1886078
330018L16R	7.00E-12	-2.187489
Gm16685	1.01E-28	-2.1839098
Ankfn1	2.53E-39	-2.1782257
Gm20429	0.002158	-2.1754229
Troap	0.00677	-2.1741079
Tnfrsf8	0.00381	-2.1681308
Rassf6	3.34E-06	-2.1652954
Ralyl	5.94E-05	-2.1600973
Nek2	3.23E-07	-2.1522754
Nwd2	3.41E-21	-2.1507034
Sox9	0.002174	-2.1468867
Acnat1	0.004109	-2.1413839
Mark1	0.001387	-2.14031
Ffar1	0.032592	-2.1393564
Gm11867	1.19E-13	-2.13378
930020B18R	4.20E-06	-2.1334066
Efcab11	8.16E-10	-2.1334048
Adamts20	0.000196	-2.1266795
Fer1l4	0.023461	-2.1256735
Cntn5	0.006738	-2.1256488
10032G03R	0.006087	-2.1248606
Epsti1	6.51E-37	-2.124476
Gm5600	0.042773	-2.1239433
Ccl2	0.000151	-2.1213656
Cnksr2	1.17E-38	-2.1203574
Lypd6	2.16E-05	-2.1186909
Vwc2l	0.010655	-2.1148889
Sgo2a	0.000435	-2.1120044
Corin	0.001479	-2.1093479
Bfsp2	0.038959	-2.1054028
Pde1b	2.51E-05	-2.104701
Gpr39	5.21E-30	-2.1001447
Tgfbr3l	0.001399	-2.0986136
Map3k21	0.000583	-2.0961553
Gm19950	0.023448	-2.0957949
Gm37273	0.001323	-2.0929686
Pdcd1lg2	8.32E-16	-2.082837
Tnni3k	0.038982	-2.0790168
Kynu	3.91E-64	-2.0783498
Pcdh15	2.70E-09	-2.0761972
Hs6st2	4.90E-20	-2.070613
Il1r2	0.045031	-2.0703032
Ifi211	1.33E-06	-2.0687523

Ptprn2	3.38E-11	-2.0682859
Gpr157	9.87E-06	-2.06772
Crtam	0.03257	-2.067533
30593A02F	0.001245	-2.0661826
Gm43579	0.018722	-2.0644287
Fbxl13	4.03E-08	-2.0642241
Cntnap2	1.73E-06	-2.0622383
Cfap299	7.53E-55	-2.0617957
Itgam	9.48E-13	-2.0577465
Bmpr1b	1.61E-109	-2.0562197
C3ar1	0.001132	-2.0530846
10020O05F	0.002341	-2.048777
Kctd16	3.05E-05	-2.0444528
Olfr920	0.000589	-2.0432359
Rgs22	1.77E-29	-2.042808
Zfp711	0.001501	-2.0422739
Ncam2	2.45E-06	-2.0391848
Dab1	1.78E-05	-2.0369307
Itprid1	1.06E-54	-2.0352551
Nebl	4.23E-212	-2.0333511
Dtl	0.00028	-2.0239696
Gpr173	0.012025	-2.0235183
Gm12166	0.042864	-2.0097596
Ifngas1	0.018688	-2.0083542
Fstl4	5.25E-15	-2.0051626
Lrp1b	5.42E-39	-2.0037998
Fcna	9.13E-11	-1.9969812
Ect2l	6.94E-09	-1.9955132
Gm12695	7.56E-20	-1.9943288
Bmp5	3.37E-41	-1.9941431
Thrb	1.90E-95	-1.990342
Fam3b	0.032658	-1.9854746
Slamf8	0.001241	-1.9844802
Gm29488	1.65E-06	-1.9830584
Ikzf4	0.014002	-1.9823232
Gm17767	0.044988	-1.9822022
Gm47798	0.025419	-1.9805734
Cd5l	5.18E-05	-1.9792102
Nos2	1.01E-05	-1.9786656
Gm32950	0.03268	-1.9767276
Ch25h	3.95E-16	-1.9740375
Fut9	1.65E-10	-1.9700598
Glt1d1	0.000235	-1.9694113
Mtus2	2.10E-19	-1.9684602
Fcrl5	0.00768	-1.9662802
Gm44748	0.002488	-1.9549655
Ksr2	1.74E-06	-1.9542372
St8sia6	6.55E-17	-1.9526108
Gm32569	0.00114	-1.9511371
Lnx1	1.33E-17	-1.9480835
Erc2	1.64E-36	-1.9448511
30404E10F	8.99E-05	-1.9435355
Pcx	7.33E-41	-1.9418101
Armc2	3.83E-07	-1.9388257
Frmpd4	5.33E-07	-1.9356719
Ptx3	0.004128	-1.9351439
Tnfrsf4	0.002571	-1.9293471
Ccl4	0.000281	-1.9270403

Tmc5	1.59E-09	-1.9267932
Acvr1c	7.75E-05	-1.9266352
Ttc6	5.07E-23	-1.9222966
Ms4a7	0.00017	-1.9204821
Rmi2	4.93E-05	-1.9179832
Gm13481	0.000278	-1.9172185
Awat2	0.025343	-1.9134675
130043K22I	8.70E-05	-1.9104317
30622O22F	8.29E-64	-1.9088374
Spag16	7.54E-32	-1.9060243
Gm2895	0.039005	-1.9031319
30427N15F	0.018734	-1.9026748
Ank1	0.004147	-1.901385
Gm36723	1.05E-11	-1.9013637
Hkdc1	1.06E-05	-1.8972363
Grik4	0.0253	-1.8962656
Klkb1	0.001526	-1.8955563
Gm20658	0.045119	-1.8954803
Plau	0.000235	-1.8936847
Fcgbp	1.10E-18	-1.8931182
Gm43259	5.35E-09	-1.8912428
Lypd6b	3.66E-19	-1.8842954
32428C04F	0.014193	-1.8818093
Ikzf2	1.26E-40	-1.8761059
Gm6213	2.42E-11	-1.8758146
Ankub1	3.88E-05	-1.8753939
Xlr5a	3.72E-14	-1.8730329
Nek11	3.66E-11	-1.872406
Il23a	0.045053	-1.8722472
Cd55b	0.004343	-1.8699489
00042K21F	0.0449	-1.8698698
Gm5086	0.010677	-1.8658569
Gm47140	0.042954	-1.8635534
Zfp811	0.010683	-1.8609637
Platr22	1.18E-07	-1.8593862
Muc5ac	1.19E-06	-1.8589805
Depdc1b	0.045713	-1.8583589
Adgb	2.91E-14	-1.8582971
Tgfa	9.69E-09	-1.8524779
030444F02F	0.023545	-1.8501187
Elmod1	3.32E-10	-1.8500211
Itga7	2.08E-06	-1.8459966
Rp1	1.07E-30	-1.8453856
Batf3	1.57E-09	-1.8452696
Igsf11	0.007764	-1.8433944
430402P08F	0.042977	-1.8430802
Myo16	0.007325	-1.8422227
Dnah7b	1.39E-12	-1.8414461
510040J01R	4.71E-45	-1.8395705
Wfdc13	0.004162	-1.8342439
Crb1	5.64E-05	-1.8329748
4-Mar	4.52E-08	-1.832833
Cacna1i	0.025404	-1.8318232
Slco1a5	4.59E-10	-1.8277004
Sema6c	0.032658	-1.8275199
Gm48653	0.004614	-1.8243528
Wdr49	5.53E-09	-1.8239488
Slamf1	0.000473	-1.8220411

Gm1968	0.045192	-1.8210032
Gm12474	2.79E-09	-1.8206344
Fat2	0.045045	-1.815922
Zfp385b	4.06E-26	-1.8153055
Tnfrsf9	0.0008	-1.8140936
Ska1	0.024825	-1.8130328
Cacnb4	3.13E-06	-1.8129009
Saa3	2.52E-12	-1.812112
Taco1	7.34E-52	-1.8071381
Panct2	3.56E-11	-1.8069387
Ccl20	0.001494	-1.8047141
Gm14286	0.004512	-1.8029778
Gfra1	3.60E-18	-1.8024123
Clnk	1.04E-05	-1.7994922
Zbbx	4.22E-11	-1.7975817
Slc12a5	0.018734	-1.7971508
Sybu	1.26E-07	-1.7951728
Dpp10	0.004647	-1.7948394
Gm4107	0.008207	-1.7922117
Mthfs	2.86E-32	-1.7915541
Vmn2r53	0.044879	-1.7910645
Kif5c	0.002619	-1.7877734
Inava	0.014263	-1.7872111
Agbl1	3.78E-08	-1.7833294
Btbd11	2.52E-08	-1.7831805
30509H03F	8.41E-05	-1.7799347
Atp10b	3.03E-05	-1.7798664
Gm42717	0.006097	-1.7795646
Trp73	1.95E-05	-1.777485
Lrig1	2.18E-17	-1.776192
Csmd1	3.67E-10	-1.7720706
Galnt5	0.000159	-1.7706245
Dnah7c	3.60E-10	-1.7691051
Shank2	4.53E-30	-1.766964
Caps2	0.00267	-1.7668562
Ifi206	0.001521	-1.7647528
Rubcnl	1.13E-05	-1.7639046
Igfbp3	0.045922	-1.7631554
Depdc1a	0.000441	-1.7629333
Arnt2	0.025485	-1.762002
Gm26973	8.76E-31	-1.7608056
Clip4	6.07E-44	-1.7605385
Rgs16	0.045733	-1.7597188
Uhrf1	0.000881	-1.7563248
Efhb	5.32E-09	-1.7554616
Dusp2	0.002651	-1.7538489
Prrx1	0.000859	-1.7527267
Kif6	6.28E-10	-1.7508338
Greb1l	1.16E-09	-1.7466693
Ank2	5.19E-14	-1.74295
00112D23F	8.40E-05	-1.7422472
Nrg1	8.44E-23	-1.7398523
Socs1	3.34E-05	-1.7395999
Gm29266	3.45E-05	-1.7379685
Cd86	3.29E-09	-1.7371686
Gm10115	0.012653	-1.7348624
Fign	4.18E-37	-1.7329506
Chrna7	0.004151	-1.7286697

Kctd8	1.45E-53	-1.7274003
Gm5431	0.036962	-1.7266626
Junos	5.15E-45	-1.7262974
Gria4	0.019026	-1.7255602
Msr1	1.57E-09	-1.7251015
Rhox8	0.039143	-1.719215
Sh3rf3	1.99E-19	-1.7190374
Syt10	0.007839	-1.7181585
Vmn2r29	0.02334	-1.7172198
Gm42418	0	-1.7171516
Ankrd45	5.63E-05	-1.7169017
Cd72	0.040941	-1.7158677
Gm43042	0.045031	-1.7148271
Rad54b	0.000887	-1.7134197
Mob3b	1.03E-145	-1.7131258
Slc7a2	1.18E-121	-1.711739
Ctnna2	0.004167	-1.7104076
Cfap47	7.86E-16	-1.7068479
Camk2a	0.02363	-1.7050682
Rasgrf1	0.000513	-1.7022354
Fpr3	3.11E-05	-1.6954255
Efhc2	0.025617	-1.693345
730090N16R	0.024908	-1.6907475
Gm31793	9.97E-05	-1.6906087
330416I19R	9.98E-05	-1.6883924
Cysltr2	0.001358	-1.6876227
00002D24R	0.045961	-1.6872617
Anks1b	3.96E-07	-1.6863285
Sec14l5	0.045706	-1.686205
Adgrv1	1.14E-09	-1.6846632
330002G04R	3.19E-16	-1.6830958
Tmem56	4.88E-24	-1.6823339
Lpar3	8.04E-25	-1.6821778
Hs3st3a1	1.97E-05	-1.6818534
730522E02R	0.000175	-1.6814703
Rbm47	1.66E-84	-1.6768772
Cxcl10	0.000269	-1.6758485
230057D06R	1.25E-19	-1.6743873
Trpm6	6.06E-61	-1.674322
Pglyrp2	0.044082	-1.6742698
Esrrg	1.53E-29	-1.6715862
Hnf4g	0.025427	-1.6700962
030562C15R	2.99E-08	-1.669924
130230L23R	0.001228	-1.6693868
Gm17268	0.002976	-1.6646716
Mfap3l	1.24E-11	-1.6630672
Ros1	6.09E-06	-1.6630595
Bora	2.01E-05	-1.6623593
Slc18a1	0.002364	-1.6616882
Gli3	2.51E-12	-1.661042
Il18rap	1.78E-05	-1.659595
Cd163	0.001407	-1.6588894
Adamts3	0.01322	-1.6572163
Tmem232	6.06E-14	-1.6549262
Ccser1	1.92E-124	-1.6538
Npas2	0.000502	-1.6533505
Armc4	4.15E-11	-1.6526845
Sema3e	5.54E-57	-1.6513546

Cap2	2.73E-07	-1.6496977
Ppargc1b	8.99E-14	-1.6489781
Eya1	2.64E-10	-1.6483533
Ppp2r2b	4.64E-25	-1.6450065
Mreg	3.05E-09	-1.6391232
Gm37245	2.20E-11	-1.6379025
Rasef	2.26E-17	-1.6351255
Hmgcll1	1.71E-19	-1.635004
Cntn3	0.025595	-1.6344189
Spata18	1.20E-09	-1.6334374
Nlrp4e	0.025415	-1.6329845
)30001A20F	0.004675	-1.6310263
Rian	0.007973	-1.6308291
Agmo	1.02E-71	-1.6292088
Ccdc170	5.35E-17	-1.6291632
Map2	1.08E-08	-1.6281733
Ppp1r42	0.002564	-1.6275014
Esrp1	6.42E-16	-1.6237105
Gm15998	0.013943	-1.6226331
Cadm2	3.63E-51	-1.621537
Fam83b	3.25E-06	-1.6191557
Gm43260	6.45E-09	-1.6185494
Tmem26	0.046066	-1.6183763
Foxp2	1.34E-78	-1.6175758
Pkhd1	4.64E-07	-1.6155776
Trmt44	0.004254	-1.6152579
Gm11337	0.023691	-1.6146793
Dusp10	2.01E-05	-1.6139813
T2	3.43E-09	-1.6119857
Susd4	1.62E-06	-1.6093687
H2-DMb2	5.92E-05	-1.6092147
Catspere2	4.73E-26	-1.6050229
Ap1s3	4.34E-11	-1.6048457
Egln3	0.000905	-1.6034188
Epha3	0.001538	-1.6029463
Spata48	0.013196	-1.5990213
Kcnj15	4.46E-48	-1.5983568
Gm32364	0.0003	-1.5969771
Parpbp	0.008038	-1.5967765
C1qc	2.11E-11	-1.5960079
Ctnna3	1.54E-05	-1.594449
Dsc2	0.045972	-1.5914608
Vit	0.000909	-1.5872092
Inpp4b	1.21E-107	-1.5871938
Pak6	0.008245	-1.58716
Kcnmb2	8.46E-13	-1.5870633
Opcml	0.001541	-1.5869408
Dthd1	0.006828	-1.5864496
Nek10	7.57E-10	-1.5863265
Rad51b	2.77E-61	-1.585721
Mafb	9.66E-08	-1.584702
Gk	2.07E-14	-1.5839685
Cd300lb	0.007226	-1.5824102
Wdr72	1.24E-09	-1.581286
Ccl8	0.002678	-1.5787767
Stx11	7.39E-07	-1.57471
Lrguk	1.23E-06	-1.5728969
Prr16	3.98E-06	-1.5726405

Cd244a	2.20E-06	-1.5725897
Edar	0.000903	-1.5709209
Cers6	6.85E-46	-1.5706126
Gm28856	0.003654	-1.5691256
Pigr	2.87E-49	-1.5673804
Frmpd2	2.94E-08	-1.5646343
Rims1	0.045119	-1.5645815
Il1b	0.00135	-1.5643069
Plekha6	1.14E-08	-1.5637882
Diaph3	9.58E-20	-1.5620631
330093E20F	2.14E-07	-1.5619371
Il1rapl1	4.01E-09	-1.5619092
730002L08F	0.002373	-1.5613461
530023A22F	0.045882	-1.5602613
Frem2	0.000837	-1.5596326
Rab3c	1.27E-07	-1.5582575
Lrriq1	4.22E-18	-1.5582363
Rbfox1	2.72E-08	-1.5570352
Mecom	0	-1.5570191
330182L06F	2.66E-24	-1.5563317
Tenm2	0.004541	-1.5551747
Gclc	1.25E-130	-1.5549622
Fam20c	1.54E-05	-1.554349
Malt1	6.27E-64	-1.5521235
Lrrc6	1.83E-09	-1.5500468
Sox6	3.68E-34	-1.5495474
Shh	0.000304	-1.5482283
Alcam	0	-1.5470831
Mgat3	8.47E-15	-1.5459958
Tc2n	2.45E-51	-1.5457899
Gbp6	1.48E-09	-1.5450058
Knl1	6.21E-06	-1.5437393
Xkr4	4.02E-06	-1.5432013
Gm42659	4.58E-06	-1.5413686
Col4a6	1.62E-14	-1.5404471
Cfap221	0.000875	-1.5403641
Nkx2-1	8.78E-46	-1.5396578
Lpar1	3.67E-07	-1.5357247
Dck	1.05E-06	-1.532686
Ccdc60	1.06E-05	-1.5314253
Slamf7	4.64E-08	-1.5311112
Eya2	7.07E-33	-1.5310983
Arhgap19	0.007823	-1.5307041
Adam28	9.57E-15	-1.5303361
Tnfsf8	6.78E-06	-1.5297957
Igf1	2.05E-13	-1.5281897
Gm38393	0.025589	-1.5281669
Ano2	0.001485	-1.5272077
Rab27b	6.08E-37	-1.5237068
Il27	0.004055	-1.5231003
Csmd3	0.008238	-1.5221125
Spata17	1.63E-09	-1.5219529
Katnal2	0.014438	-1.5218257
Macc1	2.20E-34	-1.5212828
Ngef	1.25E-12	-1.520504
Fmn1	4.93E-17	-1.5200469
Crnde	4.46E-08	-1.5190586
Galm	1.51E-05	-1.5177442

Ly6i	0.001828	-1.5175127
Mtmr7	1.21E-15	-1.5159229
Nkain2	3.54E-05	-1.5156495
Slc17a9	1.39E-07	-1.5140778
Enah	2.22E-42	-1.5126648
Gm47578	0.004573	-1.5121543
Tnc	0.021143	-1.5108222
Shisal2a	0.032724	-1.5103542
Cracr2a	2.44E-34	-1.5099414
Dnah3	3.62E-18	-1.5081352
Spag17	3.07E-15	-1.5077972
Pde6a	0.003999	-1.5069419
Pms1	6.63E-05	-1.505648
Cdca2	0.002085	-1.5048176
C1qa	5.79E-15	-1.504047
Plcxd3	0.004305	-1.5017239
Pclo	6.59E-12	-1.5002624
Slco4c1	1.71E-46	-1.49994
Rnf17	0.022801	-1.4976079
Kcnb2	1.05E-05	-1.4951014
Aass	5.15E-05	-1.4935298
Sobp	9.44E-06	-1.4929041
Bcl11a	1.16E-11	-1.492831
Dtna	7.41E-31	-1.4919288
310014F06R	0.00084	-1.4911018
AW046200	0.004373	-1.4906226
Cep128	6.54E-27	-1.4887823
Sestd1	1.59E-18	-1.488437
Lrrc4c	0.012447	-1.4861613
Zfp827	2.66E-12	-1.4856377
Garem1	2.96E-16	-1.4855503
Dnah12	1.15E-32	-1.4833137
Gbp5	5.26E-12	-1.4786191
Gm10778	0.007792	-1.4782578
Grip1	4.03E-68	-1.477764
Slc35f3	3.83E-06	-1.4769326
Pmaip1	0.00703	-1.4761359
Ccdc122	8.87E-08	-1.4746761
Map7	6.00E-33	-1.4738534
Mis18bp1	0.001453	-1.4724722
Gca	4.47E-10	-1.4721135
Gm47121	3.58E-05	-1.4714274
230072E10R	0.000512	-1.4681523
Frk	9.87E-20	-1.467878
Ust	5.05E-13	-1.4670188
Dmc1	0.022949	-1.4659107
Gm43256	7.21E-06	-1.4649989
Dnajc5b	0.043045	-1.4625198
Hpn	0.000522	-1.4623064
Iigp1	9.46E-37	-1.4585376
Fam13a	1.82E-47	-1.4572982
Plcl2	1.52E-22	-1.4572324
Chsy3	3.37E-12	-1.4553038
Lrriq3	0.004688	-1.4540525
Kifc1	0.014367	-1.4534803
Myo5c	3.93E-44	-1.4517947
Adora2a	0.001548	-1.4491697
Sema3a	4.06E-31	-1.4475733

Gm14636	0.000144	-1.4468344
Pdzrn3	2.88E-20	-1.4465661
Veph1	1.68E-10	-1.4462002
Gm42595	0.046191	-1.4455392
Pla2g4f	0.000789	-1.4450406
Dclk1	1.51E-14	-1.4447095
Lekr1	1.99E-07	-1.4435085
Akap6	0.000768	-1.4431734
Gm26797	0.007756	-1.4398743
Gm29650	0.037509	-1.4397253
Ror1	7.27E-59	-1.4389286
Mbnl3	3.37E-17	-1.4387772
Fhit	1.07E-54	-1.438685
Cdk8	1.09E-109	-1.4383703
Gm14634	0.031969	-1.4380246
Gm28055	0.004737	-1.4372415
Rnf150	2.89E-13	-1.4368161
Osbpl6	1.68E-57	-1.4367987
Shisa6	0.041238	-1.4358308
Pla2g4c	0.024531	-1.4356885
Rcan2	3.04E-09	-1.434064
530024D03R	4.33E-06	-1.4323508
Dact1	0.044254	-1.4322312
Magi2	1.59E-25	-1.431609
Plch2	0.013774	-1.4308778
Slc16a12	5.54E-09	-1.4280272
Gfra2	0.002713	-1.4270356
Asic2	0.007438	-1.427021
Dlg2	0.004066	-1.4257684
Robo1	1.41E-10	-1.4217443
Myo5b	1.39E-29	-1.4214153
Zfp618	0.000851	-1.4212848
Ank3	0	-1.4205442
Arhgap10	3.57E-27	-1.4199777
Bmper	0.000104	-1.4194044
Gm42701	0.002153	-1.419385
Gm35154	0.000895	-1.4193523
Rnf128	2.89E-10	-1.417109
Acsl4	1.42E-89	-1.4161476
Vtcn1	5.01E-08	-1.4157993
Ttc29	5.55E-10	-1.4149365
Gm39526	0.003245	-1.4148013
Arhgap24	5.37E-42	-1.4141318
Fam169b	3.45E-06	-1.414104
Sema4b	3.67E-06	-1.4139319
Map3k9	0.000482	-1.4133809
Enox1	0.018249	-1.411917
Col23a1	1.88E-42	-1.410675
Rnf19b	1.68E-20	-1.4097526
Ifi204	8.59E-06	-1.4096424
Rsph14	0.041295	-1.4083733
310064F22R	2.28E-06	-1.4074594
Wdr95	0.000763	-1.406398
Cacna1e	1.50E-09	-1.4055201
230072F16R	1.95E-05	-1.4054155
Gm50334	0.046118	-1.4052426
Epb41l5	4.09E-35	-1.4052073
Arhgap44	1.07E-36	-1.4043568

Gm20755	0.002733	-1.4037926
Prkn	1.30E-88	-1.4026671
Me1	2.62E-28	-1.4011423
B3galt1	4.12E-11	-1.398821
Gm6566	0.023821	-1.3975539
Cacna1d	2.65E-11	-1.3971355
Pacrg	4.76E-22	-1.3956094
Abca9	0.002284	-1.3955462
Nfkbid	0.000453	-1.3946326
Il5ra	0.041	-1.3942252
Gm48027	5.35E-06	-1.393971
Osbpl3	3.62E-25	-1.3917344
Acss3	5.46E-10	-1.3906405
Ssh3	0.000921	-1.3888077
Slc37a2	0.002584	-1.3882186
Tmem30b	0.004736	-1.386227
Nexmif	4.27E-21	-1.3850071
Slc1a1	1.60E-07	-1.3846014
Mir155hg	0.013342	-1.3843887
Gimap7	0.013156	-1.3828498
Shcbp1	0.013435	-1.3823175
Gm19325	0.004086	-1.3822328
Gm32857	0.033837	-1.381317
Ccl9	5.53E-09	-1.37844
Gpc4	1.27E-06	-1.3755923
Aig1	3.70E-46	-1.3749903
Zfp51	0.007109	-1.3744176
Frem1	0.000138	-1.3728632
Lgmn	4.57E-27	-1.3698131
Nsun7	1.15E-08	-1.3690305
Spc24	0.018126	-1.3682748
Gm35330	4.03E-06	-1.3666206
Tsga10	1.19E-11	-1.3661524
Steap2	6.08E-06	-1.3656354
Lca5	1.17E-07	-1.3640697
Cfap44	1.70E-15	-1.3637513
930009F23F	0.028789	-1.3627092
Rrm2	0.004331	-1.3619748
Ptpro	0.000767	-1.3594161
Car8	2.25E-75	-1.3588259
Gm31227	0.01391	-1.3568528
Plekha7	5.07E-11	-1.355547
Pak1	8.03E-09	-1.3552421
Clec10a	0.041164	-1.3550693
Neo1	2.41E-16	-1.3548188
Csf3r	0.000423	-1.3547773
Ms4a1	5.05E-06	-1.3537262
Igf2bp2	3.54E-12	-1.3528555
Nxf1	6.62E-20	-1.3526885
Rfx3	4.05E-53	-1.3509252
Lama2	1.18E-07	-1.3502014
Tmprss2	1.93E-10	-1.3499681
Pcdh11x	0.030243	-1.3488888
Sel1l3	0.000776	-1.3483455
Plcxd2	0.000209	-1.3483361
Tec	3.34E-13	-1.348274
Cftr	1.93E-13	-1.348062
Ptafr	0.00058	-1.3478277

Map11	3.91E-05	-1.3471395
Gm31718	0.01049	-1.344754
Cacna2d1	1.28E-18	-1.3434976
Stk33	4.26E-10	-1.3434589
Ly86	1.34E-08	-1.3433144
P3h2	1.66E-09	-1.3426207
Galnt13	1.31E-19	-1.3415013
Dach2	0.035596	-1.3390899
Actn2	0.021215	-1.3375487
Ppef2	0.005522	-1.3352008
Fhl2	0.000841	-1.3336522
Sytl2	2.16E-07	-1.3326184
Trim7	0.004946	-1.3321361
Ntm	0.002743	-1.3317579
Lrrc71	2.42E-05	-1.3317432
Klhl29	1.17E-28	-1.3315877
Ccdc180	1.52E-06	-1.3300241
Ormdl1	0.000126	-1.3298026
Gm42869	0.007365	-1.3298004
AI182371	0.007633	-1.3265489
Fcrl1	0.039135	-1.3253726
Igsf9	0.002178	-1.3252629
Irf6	0.013478	-1.3249186
Tnfaip8l3	0.007058	-1.3247243
Ahcyl2	1.36E-42	-1.3239959
'00054A03F	2.86E-10	-1.3239088
Adamdec1	0.000405	-1.3225837
Rab38	1.17E-07	-1.3221915
C6	1.38E-13	-1.3218817
Cpeb2	1.53E-13	-1.3216771
Epha7	9.74E-23	-1.3210583
Stk36	0.000473	-1.3205585
Ildr1	2.47E-05	-1.3199701
Nell1	0.013915	-1.3199231
Mitf	7.29E-92	-1.3196155
Myo3b	0.006665	-1.3179669
Ect2	0.000359	-1.3174951
Etv1	1.20E-05	-1.3171894
Galnt7	2.74E-30	-1.3167334
Rnf24	6.43E-07	-1.3137502
Arhgap8	4.25E-06	-1.3133812
Cdcp1	1.83E-07	-1.3130985
Gm43577	0.011492	-1.3129684
Gm15987	8.11E-10	-1.3117758
Ttc8	1.87E-06	-1.3102439
Mctp2	6.26E-12	-1.3089326
Muc5b	1.40E-28	-1.3074925
Ccdc141	7.70E-72	-1.3072456
Ifnlr1	0.002529	-1.3070515
Slfn4	0.046256	-1.3063277
Cd96	0.018445	-1.3041081
Ccdc88c	4.86E-27	-1.3031034
Rgs20	0.042739	-1.3026603
Gm2682	7.36E-18	-1.3013752
Dpyd	2.07E-07	-1.3004103
Cdh1	3.08E-50	-1.2996777
Arhgef28	1.34E-32	-1.2987961
Ptpn13	8.81E-42	-1.298312

Racgap1	0.000173	-1.2974999
Tmem108	9.69E-11	-1.2965786
Fam110b	1.63E-06	-1.2965386
Dse	5.84E-14	-1.2963191
Togaram2	8.32E-08	-1.2947171
Atp8a2	0.004218	-1.2945402
Spata13	1.01E-12	-1.2944752
Rmdn2	1.51E-21	-1.2937611
Ddhd1	5.93E-58	-1.2934534
Lrmda	2.35E-257	-1.2918901
Thsd4	1.98E-69	-1.2911355
Lin52	5.15E-28	-1.2888393
Rpl9-ps6	0.011722	-1.2880937
Btc	0.001194	-1.2879982
Fzr1	2.35E-05	-1.2877959
Prkcb	6.58E-22	-1.2867724
Grm8	0.007991	-1.2851637
Zfat	1.08E-05	-1.2851598
Eps8	4.17E-41	-1.2849409
Gramd3	8.86E-14	-1.2846291
Gramd1b	1.04E-25	-1.2832519
Gm26740	2.83E-29	-1.2817273
Serpina3g	0.008582	-1.2805995
A2ml1	2.09E-13	-1.2805096
Rbm45	3.18E-05	-1.2802407
H2-K1	1.23E-169	-1.2786212
Mir17hg	0.001986	-1.2784497
Hnmt	3.49E-06	-1.2779321
Gm42702	1.01E-06	-1.2770223
Rnf43	1.90E-12	-1.2740776
Ssbp2	9.32E-32	-1.2735111
P2ry10	0.000544	-1.2728855
Adamtsl1	1.16E-50	-1.2726622
Lrrc40	0.000449	-1.2714313
Ms4a6b	8.89E-05	-1.2711676
Acaca	1.50E-38	-1.2700055
Mir99ahg	3.18E-69	-1.2696752
Cyp7b1	3.54E-07	-1.2693203
Cacnb2	3.97E-24	-1.2682854
Slc4a7	2.19E-15	-1.2679163
Gm43728	1.50E-07	-1.2678771
Lilr4b	1.09E-07	-1.2673425
Cfap69	2.05E-13	-1.2647331
Gm13986	0.025224	-1.2617502
Gm32618	0.022053	-1.2611216
Arhgap15	7.82E-86	-1.2607142
Exph5	7.43E-08	-1.2606393
Ptgs2	0.008082	-1.2577267
Cdkl5	2.91E-23	-1.2576344
Egfr	1.27E-12	-1.2569593
Gm32633	0.009821	-1.2560279
Cfap46	3.55E-05	-1.2554547
Atp11a	7.28E-69	-1.2544733
Lpxn	1.26E-05	-1.2542542
Cacna1c	3.55E-52	-1.2539499
Grhl2	4.94E-11	-1.2534184
Vdr	4.42E-05	-1.2520782
Ttc39c	3.83E-06	-1.2518179

Dennd1b	4.27E-53	-1.2509566
Gm43920	0.004229	-1.2498101
Diaph2	6.02E-123	-1.2494541
Ak9	3.58E-10	-1.248122
Klhl15	1.23E-08	-1.246781
Gm30211	0.013435	-1.246212
Gm49890	0.00355	-1.2457675
Neil3	0.000619	-1.2457141
Shroom3	4.40E-101	-1.2442167
Pkib	1.20E-07	-1.2425482
Ppp1r14c	5.39E-92	-1.2422519
Dnah6	1.08E-14	-1.24165
Ipcef1	5.45E-06	-1.2413644
Bnc2	2.06E-20	-1.2412609
Tesk2	0.000277	-1.2407967
Arfgef3	3.40E-12	-1.2387744
Tmem132d	5.43E-45	-1.2381108
Crocc2	0.032004	-1.2378322
Grem2	0.000129	-1.2378269
Fam122b	0.001203	-1.2363438
Osmr	6.42E-21	-1.2361011
Cdk1	0.003807	-1.236077
Gm43466	0.022255	-1.2359096
Gm15832	0.018468	-1.2351741
Satb1	4.69E-11	-1.2334797
Slc2a3	0.002859	-1.2330141
Gm43258	0.009025	-1.2328651
Tmem131l	1.03E-20	-1.231565
Celf2	1.38E-108	-1.2312593
Ranbp17	6.23E-06	-1.2305655
Pheta1	0.006624	-1.2303023
Mex3d	0.024051	-1.2291712
Gm20732	1.53E-36	-1.2291441
Kcnn2	0.015532	-1.228967
Spata5	3.96E-28	-1.228406
330076H08R	7.04E-05	-1.2254769
Erich3	1.80E-05	-1.2254483
Rapgefl1	0.044288	-1.2249914
Atp13a4	0.002012	-1.2218047
Atp2c2	0.028753	-1.2211488
Slit3	1.58E-14	-1.2208222
1-Mar	9.26E-23	-1.2202696
Gramd2	5.43E-24	-1.2201231
Gm31641	9.08E-17	-1.2197687
030443J06R	0.000276	-1.2181721
Matn4	7.62E-07	-1.2178548
Il10ra	4.19E-05	-1.2175604
Cfap54	8.82E-15	-1.2171722
Gm16675	0.000104	-1.2168256
Vrk2	9.70E-16	-1.2168081
Aldh1a7	4.25E-18	-1.2161696
Bank1	3.35E-27	-1.21578
Zfp474	1.80E-05	-1.2144536
Daw1	0.002123	-1.2119497
Vwa3a	5.50E-05	-1.2117716
Nrbp2	1.07E-08	-1.2106568
10019D14R	1.47E-09	-1.2090342
Mboat1	1.40E-06	-1.2075615

Slc28a2	0.025485	-1.2072991
Chka	1.29E-47	-1.2066471
Elf3	0.000278	-1.2060116
Gm26917	3.71E-43	-1.2056768
Gm4951	3.61E-14	-1.2045365
Sorcs2	6.64E-11	-1.2038192
Prim2	1.04E-09	-1.2036115
Ltb	7.85E-05	-1.2032056
Dnah9	3.99E-13	-1.2021551
Gm43462	0.006591	-1.2007524
Notch2	8.32E-30	-1.1996878
Tenm4	0.007483	-1.1987556
Cpeb3	4.21E-37	-1.1966492
Slc16a7	5.79E-18	-1.1965684
Msmo1	6.23E-12	-1.196273
Lbh	8.48E-20	-1.1958998
Lrpprc	1.22E-11	-1.1956368
St14	2.44E-06	-1.1950634
Lrrc36	1.31E-08	-1.1944802
Kif27	0.000894	-1.1933558
Parp8	6.11E-26	-1.1929612
Hs2st1	3.36E-37	-1.1914955
30427O19F	0.006577	-1.1913703
Slc25a13	3.58E-07	-1.1911618
Apex2	6.97E-08	-1.1908734
10300C02F	1.32E-07	-1.1904051
Cpeb1	1.26E-06	-1.1903943
Ptpn3	7.32E-15	-1.1901782
Hk3	0.034077	-1.1901344
Fabp12	0.003356	-1.1900788
Bcl6	3.36E-12	-1.1883761
Ppm1l	8.69E-17	-1.1881274
Clec4a1	0.01218	-1.1880252
Kif21b	0.01609	-1.187782
Wnt3a	0.011656	-1.1867522
Samhd1	1.44E-45	-1.1844
Aox1	1.13E-11	-1.1838971
Cacna1b	0.023098	-1.1836513
Plekhh2	1.55E-07	-1.1831671
Rspo3	0.022737	-1.1831544
Prdm16	7.06E-19	-1.1824335
210408I21R	3.30E-31	-1.1813251
Adora2b	0.000585	-1.1809624
Osbpl10	4.49E-13	-1.1801306
Aplf	0.003008	-1.1794943
Mccc2	2.33E-06	-1.1793818
Lrba	6.54E-59	-1.1792093
Kndc1	0.020467	-1.1772554
Frmd5	1.48E-06	-1.1767424
Evi2	0.042787	-1.1758083
Zfp619	0.034125	-1.1737453
Tespa1	0.000748	-1.1729155
Pym1	4.23E-05	-1.1728066
Tfec	6.43E-18	-1.1726057
30036O11F	1.16E-14	-1.172056
Wdr7	5.28E-20	-1.1711447
Arid5b	5.44E-108	-1.1709267
Sdc3	2.74E-08	-1.1696972

Met	6.80E-23	-1.1678331
Lat2	0.005127	-1.1628843
Armc9	3.48E-05	-1.1608646
Ddo	1.17E-05	-1.1605726
Gm44751	0.015518	-1.1603239
Mif4gd	9.06E-07	-1.159668
Atp8a1	1.13E-204	-1.1593071
Rasgef1b	1.89E-11	-1.1584208
Srgap3	1.92E-06	-1.157686
Gna14	2.09E-29	-1.1576852
Cfap43	1.66E-14	-1.1569191
Ctnnd2	5.77E-21	-1.1568945
Npas3	2.94E-05	-1.1568626
Kif11	0.000595	-1.1555803
Rims2	0.003517	-1.1555735
Sfta3-ps	1.61E-172	-1.1547729
Slc1a3	0.00016	-1.1543969
Usp43	0.002921	-1.1543417
Pbx1	7.99E-184	-1.1542186
430402I18R	0.000412	-1.1540051
Dnah5	9.98E-22	-1.153614
Lonrf1	2.68E-05	-1.1532542
Ppm1b	4.24E-25	-1.1530163
Ccdc30	1.15E-06	-1.1519849
Rtkn2	6.10E-36	-1.1518368
Adamts15	0.017726	-1.1504712
Il3ra	4.05E-05	-1.1501285
Dock5	4.92E-23	-1.1498231
Gm20045	1.11E-06	-1.1497762
Tinag	9.70E-10	-1.149583
Dapk1	2.74E-69	-1.1478791
Spaar	0.021445	-1.1475941
Mtcl1	1.61E-15	-1.1458106
Zswim5	0.000556	-1.1442158
Fam49b	6.53E-63	-1.1439384
Timp1	0.000518	-1.1438035
Cd55	8.64E-16	-1.1437312
Patj	4.78E-30	-1.1431123
Flt3	0.001677	-1.1428468
St7	1.38E-22	-1.1426831
Znrf3	2.74E-35	-1.1422943
Serinc5	1.36E-10	-1.1417338
Col4a5	1.21E-17	-1.1409196
Slc27a1	2.83E-05	-1.1408718
Epb41l1	8.95E-08	-1.1407311
Gypc	0.004863	-1.1407062
Gm26671	1.47E-06	-1.1401809
Slx4ip	4.37E-13	-1.1394707
Kcnd2	0.006063	-1.1393474
Fank1	0.000657	-1.139301
Hydin	2.85E-08	-1.138823
Tmem39a	6.43E-08	-1.1385938
Rnf149	1.46E-08	-1.1376617
Mfsd7a	0.033294	-1.1371997
Gm43149	0.006071	-1.1370669
Rbms3	1.19E-112	-1.1365435
Taf4	4.49E-08	-1.1364413
Ift80	1.30E-07	-1.1355087

Ccdc18	0.044403	-1.1351016
Aph1c	0.00512	-1.1342779
Jaml	0.047949	-1.1342513
Piwil4	0.020123	-1.1341553
Gm42984	0.001313	-1.1331539
Arhgef38	1.23E-53	-1.1316713
Ube2cbp	0.004731	-1.1305098
Ppp2r3d	2.81E-05	-1.1299516
Jazf1	3.36E-15	-1.1298611
Ppp1r9a	7.88E-43	-1.1294339
30417C22F	4.75E-20	-1.1289037
Ryr2	3.17E-08	-1.1286076
Tmprss4	0.000316	-1.1283158
Arhgef4	0.007936	-1.1282133
Bhlhe40	1.77E-12	-1.1278903
Shq1	5.13E-06	-1.1278785
Slc6a15	0.010842	-1.127777
Nmnat3	0.000181	-1.1275897
Fam43a	6.95E-06	-1.1262931
Ptpn2	2.11E-19	-1.1260845
Fut8	4.35E-45	-1.1258361
Rttn	6.41E-11	-1.125739
Zfp57	0.005824	-1.1256523
Pfkfb2	4.84E-20	-1.1235408
Kif16b	1.85E-20	-1.1233943
Slc9a9	6.06E-19	-1.1232278
Ttpa	0.009203	-1.1230519
Tox	2.59E-13	-1.1226206
Lrp4	2.55E-14	-1.1214392
Hs3st3b1	0.003899	-1.1211204
Cth	0.016798	-1.1206447
Scel	2.95E-06	-1.1199315
Adam22	6.82E-11	-1.1198942
Snd1	2.10E-66	-1.1195914
Blnk	2.65E-07	-1.1191432
Cp	2.34E-43	-1.1171249
Sox5	5.27E-46	-1.1161363
Fggy	8.85E-07	-1.1160836
Zfp90	0.000286	-1.1149102
Fam221a	0.004739	-1.1148953
00018A04F	0.024236	-1.1147892
Apobec1	4.81E-08	-1.11398
Rarb	2.61E-19	-1.1138648
Peli1	9.36E-44	-1.1136806
Tmem38b	2.03E-05	-1.1134224
Dapk2	2.97E-20	-1.1127049
Mgmt	0.000653	-1.112414
30097N15F	0.043712	-1.1119443
Magi3	1.90E-80	-1.1118286
Cxadr	6.24E-21	-1.1112293
Rassf2	5.65E-08	-1.1101205
Glcci1	6.13E-26	-1.1093354
Prdm5	1.08E-05	-1.1084189
Gm28791	0.002694	-1.1081431
Ints6	3.35E-18	-1.107968
Gli2	0.041377	-1.1076997
32491K20F	0.044443	-1.1074906
Gm29642	0.005822	-1.1073746

Tes	2.85E-10	-1.1070257
Srp54a	1.64E-06	-1.1069737
Ccdc138	4.03E-05	-1.1068466
Igsf9b	0.028004	-1.1065775
Tpk1	9.66E-17	-1.1060979
Barx2	0.029379	-1.1059536
Cdk2	0.00118	-1.1057662
Cradd	1.33E-30	-1.1054532
Snx25	1.57E-69	-1.1048521
Txk	0.008143	-1.1043102
Gm281	0.012321	-1.1035266
Akap17b	0.002283	-1.1034173
Alg12	0.000444	-1.1023491
Retnla	1.76E-197	-1.1023084
Spef2	2.29E-10	-1.102091
Gm46401	0.04361	-1.1019336
Btla	4.57E-06	-1.1018978
Kcnk13	0.010486	-1.101462
Srsf2	3.40E-43	-1.101258
Clcn5	2.81E-12	-1.1008766
Epb41l4a	2.11E-12	-1.1008259
30428M24I	5.36E-06	-1.0994738
Dlg4	0.001679	-1.0992324
Mbip	1.97E-18	-1.0990287
Upp2	2.31E-11	-1.0984579
Ero1lb	6.00E-06	-1.0984207
Tcf20	1.04E-42	-1.0980565
Myo5a	3.22E-13	-1.0975285
Rab28	1.02E-15	-1.097292
Hnf1b	0.00013	-1.0968193
Adk	1.94E-88	-1.0964984
Icos	0.004024	-1.0962515
Snx7	2.38E-16	-1.0938666
Il33	1.69E-34	-1.0935545
Atg10	5.44E-17	-1.0934128
Parp11	1.07E-09	-1.0933393
Adcy2	2.35E-07	-1.0925269
Kif26b	0.002619	-1.0924151
Xylt1	1.95E-10	-1.0923282
Epb41l4b	2.84E-06	-1.0922054
Wdr93	0.007381	-1.0919504
Nr6a1	4.17E-38	-1.0913274
Chuk	1.50E-16	-1.0911253
Zdhhc21	1.72E-11	-1.0906336
Pla2g4a	9.79E-14	-1.0896497
Gm5150	0.00038	-1.0882623
Tnfrsf11a	4.23E-06	-1.0882305
Lrrk2	9.30E-84	-1.0880239
Myo1b	1.35E-26	-1.0878501
Runx3	0.006584	-1.086463
Tex11	0.000323	-1.086085
Mrc2	0.000312	-1.0857515
Cd177	0.001205	-1.0854407
Kif24	0.000725	-1.0843094
Vps50	5.13E-13	-1.0835958
Nup93	1.16E-06	-1.081474
Taf4b	2.81E-06	-1.0813709
Zeb2	6.91E-99	-1.0795207

Kif23	0.001729	-1.0793805
Dnah7a	0.001607	-1.0764426
Gpm6b	7.82E-05	-1.0761781
Pof1b	7.78E-05	-1.0761638
Unc5cl	0.044616	-1.0759307
Rnf138	2.20E-12	-1.0758814
Unc5d	2.44E-06	-1.0748405
Prom1	9.52E-09	-1.0747743
Ccdc171	4.80E-11	-1.0730872
Khdrbs3	0.001247	-1.0730251
Ptpn22	7.80E-08	-1.0728825
Tdp2	0.000243	-1.0726934
Gm29724	0.002048	-1.0711356
Atl2	7.82E-10	-1.0708302
Gm28403	0.005796	-1.070714
Gramd1c	0.001452	-1.0699962
Sdk1	5.96E-09	-1.0695506
Pikfyve	3.73E-18	-1.0688621
Rap1gap2	0.000144	-1.0682697
Robo2	1.08E-12	-1.0677713
Rbbp5	1.79E-06	-1.0669335
Dock2	2.16E-47	-1.0669075
Fbxl5	4.21E-24	-1.0656274
St8sia1	0.003987	-1.0650933
Plxdc2	8.82E-43	-1.0644572
Erbb4	0.02026	-1.0637077
Slc41a2	6.41E-09	-1.062787
Tomm34	2.59E-07	-1.0620241
430106G13F	0.000256	-1.0610731
Fgd4	1.38E-15	-1.0607125
Stk39	5.76E-17	-1.0606522
Zc3h12a	0.000502	-1.0605156
Carmil1	2.44E-26	-1.0602183
Ak7	0.000193	-1.059697
Cecr2	8.24E-11	-1.0563672
Slc7a5	0.031075	-1.0543673
Gsap	9.06E-77	-1.0542051
Sfxn2	1.79E-05	-1.0541809
Fastkd2	0.022129	-1.0528489
Phldb2	1.30E-84	-1.0522186
Tent2	1.47E-27	-1.0521246
Kctd1	4.50E-06	-1.050755
Ldlrad3	1.47E-09	-1.0487777
Nrg4	0.000169	-1.0487184
Tasp1	2.12E-13	-1.0485767
Jak2	7.95E-16	-1.0476892
Zfp956	0.031223	-1.0472846
Tsc22d2	3.30E-25	-1.0460712
Iqch	0.019677	-1.0454833
Naip6	0.017724	-1.0435091
Iqck	0.013189	-1.0423184
Itgb6	0.001995	-1.0421079
Tbl1xr1	2.19E-31	-1.042095
Gm20619	0.032614	-1.0418901
Bicd1	2.43E-10	-1.0414168
Ppara	0.003865	-1.0408032
Nfkb2	0.006775	-1.0404172
Pign	3.61E-16	-1.0403961

Arhgap32	4.95E-12	-1.0403231
Sidt1	2.02E-07	-1.040179
Bend3	0.0242	-1.040041
Slc20a2	1.12E-07	-1.0400409
Tspan11	1.57E-14	-1.0395765
Ehf	1.13E-08	-1.0386866
Tmem163	1.68E-51	-1.0373784
Chchd3	1.01E-31	-1.0372331
Rnaset2a	0.007143	-1.036849
Acsm1	0.005631	-1.0365585
Zmat4	5.20E-05	-1.036078
Jarid2	1.79E-27	-1.0360695
Tanc2	2.90E-53	-1.0355415
Liph	8.02E-05	-1.0351777
Rbpj	1.67E-29	-1.0346136
Akr1c14	0.014023	-1.0346117
Rerg	6.81E-08	-1.0345495
Mapk13	0.025017	-1.0335438
Rfx5	0.013705	-1.033097
Cblb	4.20E-42	-1.0328727
Fig4	1.97E-08	-1.0324571
Atp13a3	5.37E-56	-1.0319053
Satb2	0.006715	-1.0316568
Stk38l	2.21E-08	-1.0314654
Lacc1	0.023772	-1.0311493
Haus3	0.008672	-1.0304473
Memo1	1.30E-19	-1.0302647
Pip5k1b	1.74E-35	-1.0301211
Pvt1	1.22E-24	-1.0297864
Tfrc	0.000159	-1.0296078
Ikzf1	2.01E-20	-1.0290355
Pls1	0.000289	-1.0285103
Zranb3	0.001215	-1.027868
Smim3	0.000721	-1.0278464
Slc25a33	0.023541	-1.0278193
Pou2f1	2.37E-21	-1.0269567
Aff1	1.12E-51	-1.0269312
Slc30a7	5.59E-16	-1.0268787
Setbp1	7.68E-40	-1.0267453
Syde2	0.000542	-1.0262983
Tom1l1	1.63E-06	-1.0261361
Trps1	2.64E-68	-1.0251597
Cstf3	1.73E-28	-1.0243268
Cpeb4	2.03E-24	-1.0221024
Ankrd6	0.000267	-1.0220916
Bard1	0.010468	-1.0216745
Yipf6	1.47E-07	-1.0215331
Stim1	1.01E-23	-1.0214408
Pik3cd	1.67E-06	-1.0213622
Tank	1.38E-16	-1.02086
Zc3h6	1.28E-06	-1.0198775
Igsf5	3.94E-10	-1.019744
10301B20F	7.72E-05	-1.0193222
Cldn12	0.016702	-1.0189014
Lrrc43	0.016846	-1.0187648
Ccdc162	6.08E-12	-1.0175939
Gm15337	0.036404	-1.0175302
Vill	0.033625	-1.0167844

Elmod2	0.000163	-1.0162113
Piga	0.009348	-1.0161472
Nxph1	2.73E-05	-1.0160093
Myb	0.001978	-1.0147983
Klf5	1.33E-09	-1.014573
Rai14	2.84E-40	-1.0137642
Ccdc187	0.000105	-1.0135425
Slc7a8	0.024051	-1.0135254
Ccl25	1.96E-06	-1.0120073
Clic6	2.44E-06	-1.0102025
Fbxo16	0.021796	-1.0098985
Isoc2a	0.003182	-1.0096253
Cd226	0.000642	-1.0089792
Arntl	0.000218	-1.0082415
Magi1	6.08E-88	-1.0076588
Lars2	1.73E-64	-1.0076387
Vhl	0.007892	-1.0070279
Fgfr1op	7.03E-07	-1.0064623
Prmt8	5.15E-12	-1.005585
Cep70	5.68E-07	-1.0048248
32404H12F	0.02967	-1.0048008
Cckar	4.97E-05	-1.0046817
Cyp4f15	0.024123	-1.0045565
Dcps	4.74E-05	-1.0041763
Sgpp2	3.31E-12	-1.0036376
Iqcb1	1.25E-06	-1.0028072
Atrnl1	6.46E-73	-1.0024532
Rpgrip1l	4.25E-08	-1.0013839
Fam126b	2.67E-14	-1.0011305
Pola1	2.04E-10	-1.0004347
Dnah2	0.000331	-1.0000823
Mgat5	4.84E-29	-0.9996415
Cd28	0.025018	-0.9992725
Slit2	1.53E-11	-0.9988839
Hibadh	5.69E-21	-0.9983759
Lemd3	1.97E-14	-0.9969059
Scnn1b	1.54E-08	-0.9965864
St3gal5	5.50E-09	-0.9962808
Hif1a	9.95E-15	-0.9948457
Tead1	1.85E-31	-0.9946765
Pde1c	4.06E-11	-0.9946064
Plagl1	0.003101	-0.9943331
Smyd4	0.012979	-0.9943273
Pex1	0.005355	-0.994236
Prkcd	7.59E-11	-0.993849
530020G20I	0.001831	-0.9919127
Nfxl1	1.83E-06	-0.9913892
Rhbdd3	0.038291	-0.9902341
031425E22F	2.03E-22	-0.9889151
C7	8.69E-08	-0.988759
Pstpip2	1.54E-07	-0.987618
Gtf2f2	9.00E-11	-0.9873452
Prkca	1.85E-26	-0.9868485
10021A01F	0.001018	-0.9854397
Nav3	5.96E-07	-0.9845513
Ptbp2	1.06E-15	-0.9843176
Lrp2	9.92E-14	-0.9840989
Prdm9	0.039372	-0.98398

'00025G04F	5.61E-11	-0.9836399
Supt3	1.47E-14	-0.9835575
Zmym6	4.58E-05	-0.9833284
D1Ertd622e	3.77E-06	-0.9832423
Pdzrn4	0.036091	-0.9832158
Traf4	0.018989	-0.9827985
Pax5	0.00014	-0.9822699
Tnfaip3	0.000134	-0.9818836
Cobll1	8.85E-25	-0.9807317
Jag1	9.19E-11	-0.9806951
Gbp4	1.89E-12	-0.9806169
Pde4d	6.10E-224	-0.9792331
Nt5c2	3.77E-13	-0.9783682
Gabrp	2.88E-08	-0.9780841
Nampt	4.95E-13	-0.9768626
Slf1	1.62E-10	-0.9765353
Tle1	5.70E-20	-0.9762837
Brip1	0.000424	-0.9755116
Tbc1d1	2.95E-10	-0.9745374
Fbln1	0.003144	-0.9735832
Lclat1	2.63E-10	-0.9722265
Slc25a15	0.041626	-0.9721733
Usp47	5.23E-34	-0.9720242
Ckap4	3.56E-05	-0.9718444
Slc12a2	9.75E-26	-0.9717074
Slc35e3	0.001254	-0.9717019
Etnk1	8.36E-20	-0.9711092
Eif2ak4	6.27E-10	-0.9705637
Lpp	4.51E-83	-0.9701773
Braf	1.98E-34	-0.9700989
Fbxo8	7.47E-09	-0.9698348
Alkal1	0.000214	-0.9696562
Alpk1	5.26E-06	-0.9688262
St8sia2	0.048767	-0.9685483
Maob	3.00E-05	-0.9679844
Gm16083	0.000335	-0.9675671
Glis3	3.30E-30	-0.9671704
Pid1	1.84E-24	-0.9670212
Osbpl1a	1.17E-05	-0.9658114
Ctps	5.43E-06	-0.9652125
Ppm1h	6.99E-28	-0.9651832
Stau2	7.57E-06	-0.9648931
Acsl1	2.36E-10	-0.9644924
Abhd3	0.00771	-0.9640848
Zfp951	0.000873	-0.9640383
Mars2	0.010953	-0.9637188
Zmynd12	0.010148	-0.9635549
Ercc8	0.001722	-0.9634792
Srcin1	0.000695	-0.9630777
Zdhhc1	3.23E-09	-0.9622897
Cul3	7.03E-25	-0.9621463
Tap1	2.77E-06	-0.9618519
Slc4a4	8.81E-12	-0.9616629
Pla2g7	0.000669	-0.9615635
Slc23a2	9.38E-32	-0.9609709
Ptges	0.026514	-0.9598547
Rwdd3	0.020313	-0.9597327
Gm671	0.038005	-0.9592542

Gm8113	0.029299	-0.9591897
Spry4	0.002011	-0.9590187
Man1a	4.28E-25	-0.9589612
Mtx2	8.70E-08	-0.9584608
P2ry10b	0.003178	-0.9584381
Tnpo2	7.36E-07	-0.9579178
Eed	2.91E-07	-0.9576007
Tm9sf1	0.000401	-0.9573787
Rngtt	2.96E-12	-0.9571097
Dmac2l	0.023604	-0.9564409
Nelfcd	0.038624	-0.9562184
Pcdhga12	0.002061	-0.9556037
Mnat1	9.75E-18	-0.9551656
Bend7	3.60E-17	-0.9549156
Rabgap1l	1.39E-72	-0.954737
Homer1	3.67E-06	-0.9537786
Lyplal1	0.029422	-0.9536415
Rab43	8.34E-12	-0.9536045
Sinhcaf	0.000545	-0.9525175
Dennd1a	1.81E-53	-0.9511449
Borcs5	0.001042	-0.9496953
Zfp384	1.73E-06	-0.9496644
Kif20b	0.009704	-0.9495879
Tbxas1	1.24E-12	-0.9484842
Pkp2	3.86E-06	-0.9473434
Dtwd2	0.000568	-0.946825
Ralgps2	5.32E-06	-0.946112
Cfp	0.001191	-0.9453504
Cobl	1.55E-17	-0.9451329
Spopl	1.43E-18	-0.944916
Fsip1	0.002428	-0.9437684
AA386476	0.002022	-0.9434735
Zcwpw2	0.007419	-0.942656
Samd8	2.76E-08	-0.9423398
10051M20F	3.63E-12	-0.9419428
Cd274	2.45E-09	-0.9414476
Anpep	0.000219	-0.9408373
Vps13b	2.69E-51	-0.9407976
Gm16536	0.026555	-0.9399391
Lrmp	0.003205	-0.9398652
D5Ertd615e	3.02E-05	-0.9392944
Etv3	4.87E-05	-0.9391938
Setdb2	0.000188	-0.938933
F13a1	0.000524	-0.9389021
Ercc6l2	2.99E-08	-0.9384757
Ttc39b	1.12E-12	-0.9381
Wdr63	0.000489	-0.9379683
F5	0.044614	-0.9372621
Cspp1	1.67E-25	-0.9359492
Stat1	3.13E-17	-0.9359413
Uri1	1.92E-15	-0.9358216
Gm14296	0.01693	-0.9357929
Nek1	8.99E-06	-0.9357643
Vps54	5.39E-29	-0.9357421
Smpdl3b	0.011123	-0.9354795
Eepd1	0.000157	-0.9353296
Ppp1r16a	0.000297	-0.9352809
Eda	4.31E-05	-0.9351335

Sh3rf2	0.005368	-0.9345285
C2cd5	5.63E-11	-0.9340258
Intu	0.007809	-0.9336252
Ctsc	2.76E-44	-0.9324988
Ckb	0.000637	-0.9320432
Med12l	0.004253	-0.9308971
Cln6	0.008902	-0.9308058
Enpep	6.61E-07	-0.9307267
Picalm	3.86E-43	-0.9293454
Myo1e	4.55E-44	-0.9290139
430033K04[illegible]	0.009375	-0.9289023
Cbarp	0.042247	-0.9288373
Clec12a	0.039878	-0.9287975
Aim2	0.000113	-0.9283423
Usp6nl	5.44E-14	-0.9281562
[illegible]30307A14[illegible]	1.09E-09	-0.9277662
Tatdn1	0.000825	-0.9270533
Gm13483	0.007887	-0.9268438
Cd200r1	7.14E-09	-0.9262302
Lama3	1.35E-05	-0.9255113
Rnaset2b	0.031006	-0.9254083
Cacfd1	0.012795	-0.9252761
Psmd14	2.90E-14	-0.9249194
Nos1ap	3.42E-19	-0.9244862
Pim1	1.17E-09	-0.9243987
Cldn1	0.032261	-0.9241613
Slc25a40	0.004717	-0.9239397
Fam49a	1.92E-06	-0.9235803
[illegible]10026B05[illegible]	4.88E-15	-0.9228079
Kiz	2.77E-13	-0.9226607
Ikbke	0.018757	-0.9225108
Maf	2.12E-07	-0.9222967
Wwc1	1.85E-26	-0.9219505
Fmo3	2.57E-09	-0.9215927
Tmem164	3.16E-65	-0.9204754
Ptk7	0.000329	-0.9202103
Fntb	3.28E-05	-0.9201638
Pawr	4.69E-16	-0.9199091
Etfbkmt	5.89E-05	-0.9193628
Slc8b1	0.000318	-0.9189954
Wdr66	0.00692	-0.9186719
Ercc6	3.19E-07	-0.9180007
E2f3	2.17E-06	-0.9169366
Dcp2	5.43E-07	-0.9166395
Gas2l3	0.000614	-0.9165977
Itpr1	4.02E-32	-0.9165133
Map2k4	1.24E-18	-0.915999
Zfp53	1.35E-06	-0.91593
Arhgap6	8.94E-28	-0.9157721
Arrdc4	0.00023	-0.9155225
Spred1	1.69E-16	-0.9154555
Mbd5	2.86E-36	-0.9140427
Mllt3	1.25E-38	-0.9139973
Skap1	4.99E-19	-0.9139915
Slamf6	0.001959	-0.9139573
Celf1	3.17E-38	-0.9136872
Exoc1	9.52E-06	-0.9131148
Abcb1b	0.006678	-0.9121022

Commd10	6.10E-10	-0.9117725
Dennd2d	0.00532	-0.9115975
Slc44a3	7.07E-06	-0.9114235
Mob1b	1.43E-13	-0.9113116
Gnb5	0.006682	-0.9112544
Slc1a4	0.009902	-0.9110399
Kdm2b	2.68E-05	-0.9104441
Pde1a	0.048187	-0.9103969
Ralgps1	4.10E-05	-0.9101906
Abca8b	0.027547	-0.9097618
Tpcn2	0.01322	-0.9091995
BC005537	1.23E-22	-0.9081697
Sh3kbp1	1.03E-10	-0.9081305
Mindy3	1.08E-09	-0.907527
Nlrc5	3.69E-13	-0.9072607
Cd83	0.005198	-0.9066792
Acvr2a	1.66E-12	-0.9066728
Lmbrd2	2.05E-06	-0.9062767
Syk	1.06E-11	-0.906046
Myef2	3.53E-09	-0.9051529
Fsd1l	1.36E-06	-0.9041124
Fam151b	0.024672	-0.9040291
Map3k5	5.57E-26	-0.9039229
Alms1	3.46E-07	-0.903525
Hivep2	9.41E-37	-0.9030518
Wdfy4	4.97E-11	-0.9026234
Astn2	0.000398	-0.9021818
Dph5	0.014405	-0.9021732
Zfp974	0.026775	-0.9021356
Exoc4	6.29E-72	-0.9019789
Stk35	0.002718	-0.9018782
Chsy1	1.53E-14	-0.9016004
Il34	9.23E-07	-0.9014687
Ankrd42	0.014916	-0.9013453
Mdm2	2.36E-09	-0.9012509
Itga4	2.30E-09	-0.9008335
Zcchc2	1.65E-06	-0.9004404
Ddx11	0.045534	-0.9004329
Ext1	3.66E-74	-0.9003897
Atxn7l1	3.23E-24	-0.89945
Ints6l	1.06E-12	-0.8986071
Pcca	4.81E-14	-0.8970507
Atp6v1h	3.95E-15	-0.8970189
Ube2e2	1.80E-34	-0.8963854
Nr3c2	2.22E-29	-0.8962723
Gigyf1	6.27E-10	-0.8960573
Gm32200	0.016091	-0.8959843
Svep1	0.002961	-0.8953207
Abcd3	2.08E-19	-0.8952425
Eml4	1.42E-22	-0.8939911
Ppp2r5e	4.04E-30	-0.8932647
Gas7	7.42E-06	-0.8932157
Ifi207	0.001566	-0.8931324
Clybl	1.43E-05	-0.8928959
Stambpl1	0.000923	-0.8926698
Ankrd17	8.58E-42	-0.892383
Aftph	8.40E-20	-0.8920454
Gucy1a2	0.002906	-0.8915745

Pik3c2g	1.11E-06	-0.8913895
B4galt3	0.00013	-0.8903429
Smad3	3.61E-08	-0.8900595
Map3k20	1.57E-14	-0.8898901
Fam155a	0.014765	-0.8892905
Amy1	0.000113	-0.8890627
Adam19	1.59E-24	-0.8889258
Wdr41	0.000153	-0.8881573
Etv5	2.88E-22	-0.8879891
Pde7a	8.16E-22	-0.8874463
Slc10a6	0.001088	-0.8871907
Sntb1	1.34E-31	-0.8870772
Umad1	6.79E-14	-0.8870661
Slain1	0.021268	-0.886991
Slc6a14	0.001472	-0.8867767
Immp2l	1.95E-20	-0.8864413
Pspc1	2.89E-12	-0.8863141
Zfp407	8.62E-31	-0.8860681
Tsix	0.010486	-0.8856345
Dock10	6.87E-60	-0.8851278
Lyst	3.23E-16	-0.8850839
Rnf180	6.45E-08	-0.8850279
Traf2	0.000931	-0.8841899
H2-M3	0.010076	-0.88319
Bbx	4.26E-19	-0.8829014
D5Ertd579e	2.28E-14	-0.8822916
Ccdc88b	0.013006	-0.8810732
Mdm4	8.07E-17	-0.8809024
30072M11I	0.005347	-0.8805054
Ms4a8a	7.21E-07	-0.8804256
Klhl28	4.42E-07	-0.8799538
Cdc14a	4.39E-14	-0.8795353
Scamp1	8.47E-10	-0.878984
Scfd2	1.74E-10	-0.8784375
Aox3	1.52E-22	-0.8783654
Cask	8.20E-19	-0.8779946
Vps45	0.000127	-0.8772931
Htt	2.36E-10	-0.8767988
Bckdhb	4.69E-10	-0.8767443
Unc5c	0.000943	-0.8764574
Tll1	6.60E-08	-0.8763696
Palld	6.40E-05	-0.8756188
Ttll4	3.41E-05	-0.8752384
Enox2	4.36E-22	-0.8731317
Mettl14	0.007023	-0.8719953
Pigu	2.03E-08	-0.8719761
Slc31a1	4.20E-06	-0.8714669
Usp3	1.95E-20	-0.8714331
Asxl2	1.49E-17	-0.870853
Slc39a12	0.018398	-0.8704733
Acss2	0.000267	-0.8696711
Faf1	8.09E-34	-0.8696499
Mypopos	0.009871	-0.86905
Phkb	7.84E-17	-0.8687911
Fam149a	0.004	-0.8687906
Vbp1	0.001653	-0.8685143
Bmp1	9.74E-09	-0.8674153
Lgr4	0.011558	-0.8673487

Pex6	0.000629	-0.8672889
Dnajc24	0.000359	-0.8670466
Ikzf3	8.64E-08	-0.8664503
Steap4	0.026924	-0.8664474
Ifrd1	2.21E-12	-0.8662012
Hook1	0.004743	-0.8661118
Ncoa1	5.53E-30	-0.8649964
Abca5	0.000956	-0.864874
Pik3r5	3.36E-06	-0.864616
Slc25a47	0.03753	-0.8644539
Lcorl	5.42E-16	-0.8642432
Hdac3	0.003939	-0.8641027
Traf3	1.09E-12	-0.8637632
Rab3il1	0.030531	-0.8635866
Sdccag8	2.46E-12	-0.8632562
Cdkn2c	0.041101	-0.8630698
Fnip2	1.34E-18	-0.8628895
Dzank1	0.043419	-0.8622386
Cachd1	3.39E-10	-0.8618146
BC002059	0.000584	-0.8618029
Il1rap	1.25E-06	-0.8609517
Phka1	3.32E-09	-0.8608914
Slc2a12	0.023925	-0.8601328
Lrwd1	0.026963	-0.8595658
Rasgrp1	0.036265	-0.8592875
Ralgapa2	7.56E-80	-0.8590293
Prdm6	0.00075	-0.8587673
Rabgef1	6.28E-08	-0.8587017
Fgf13	1.53E-08	-0.8584919
Rusc1	0.003639	-0.8582785
Cdyl	1.48E-09	-0.8575304
Atp9b	3.64E-20	-0.8572212
Zfp654	3.52E-09	-0.8571635
Mal2	7.89E-08	-0.8566365
Sec24d	4.21E-08	-0.8558532
Retreg1	8.09E-10	-0.8551051
Trim23	0.001056	-0.8550751
Ddx31	0.000345	-0.8549338
Cry2	5.77E-07	-0.8545014
F8	0.013861	-0.8540136
Tmem117	0.003302	-0.853918
Ccdc149	0.003381	-0.8537052
Tfb1m	0.026758	-0.8528375
Slc6a9	0.033913	-0.8527505
Runx1	1.24E-45	-0.8524508
Tarbp2	0.019136	-0.8517016
Cdc73	4.19E-12	-0.8514681
Adgrd1	0.005781	-0.8514349
Arhgef10l	0.018344	-0.8503147
Snx29	7.63E-23	-0.8502385
Grb14	6.59E-26	-0.8501048
AA0111838	9.52E-08	-0.84925
Igf1r	9.77E-31	-0.8489449
Negr1	0.010972	-0.8487504
Wdr78	9.71E-06	-0.8486372
Map3k2	3.32E-13	-0.8486272
Angpt1	4.15E-06	-0.8482434
Mccc1	0.002406	-0.8479944

Smad2	3.06E-11	-0.8479293
Lurap1l	4.98E-10	-0.8477031
Cbfa2t2	4.78E-12	-0.8476117
'00012D14F	0.000205	-0.8475619
Snhg1	0.001375	-0.8472199
Slmap	8.99E-20	-0.8470095
930007I19F	0.00012	-0.8469914
Nup98	9.08E-15	-0.846764
Prg4	0.002925	-0.8466378
Fam117a	9.84E-05	-0.8466292
Zbtb18	0.000435	-0.8462329
Casp4	9.23E-06	-0.8454316
Lnpk	5.35E-05	-0.8446525
Msl2	1.78E-09	-0.844102
Ism1	0.000162	-0.8436742
Dusp6	0.002326	-0.8435192
Vapa	2.05E-20	-0.8422361
Arhgef6	5.56E-05	-0.8422245
Sema4d	0.003535	-0.842058
Trim33	1.54E-13	-0.8415783
Marco	4.65E-05	-0.8414937
Anxa4	1.44E-13	-0.8409645
Trim30d	0.002818	-0.840884
Ift88	0.000204	-0.8408292
Ap1b1	0.000238	-0.8408117
Mme	1.91E-07	-0.8405567
Wdhd1	0.029302	-0.840403
Sik2	4.16E-13	-0.8397852
Parm1	0.048071	-0.8393414
Fnip1	7.07E-24	-0.8392036
Slc9a7	2.92E-05	-0.8384897
Kcnk2	2.75E-08	-0.838235
Fgf12	0.002313	-0.8381358
Saxo2	0.004495	-0.8376175
Pex11a	0.030533	-0.8372426
Arhgap39	3.60E-09	-0.836989
Morrbid	3.16E-13	-0.8364573
Nbas	4.21E-12	-0.8361387
Slc22a23	1.38E-19	-0.8360088
Chd7	3.53E-18	-0.83568
Cacul1	1.98E-10	-0.8355055
Gm39469	0.00919	-0.8354036
Ddx39b	1.14E-13	-0.8352817
Bbs9	1.96E-12	-0.835257
Slc38a1	6.88E-06	-0.8349728
Fam184a	6.55E-06	-0.8342556
Cnot2	1.34E-17	-0.8339939
Dock7	9.63E-13	-0.8324957
Ezh2	1.34E-07	-0.8319688
Micu3	3.46E-07	-0.8297019
Kcnq5	1.80E-21	-0.8293447
Gpatch2l	1.68E-05	-0.8292753
Kansl2	8.55E-06	-0.8290281
Ankrd28	4.06E-10	-0.8286799
B4galt1	3.97E-19	-0.828103
Lrrfip2	1.30E-17	-0.8277818
Lactb2	5.73E-06	-0.8268587
Kif18a	0.023672	-0.8266812

Sorl1	3.91E-10	-0.8266521
Cnnm2	5.41E-08	-0.8264941
Rp2	0.002523	-0.8260999
Agps	6.36E-14	-0.8260625
Mms22l	0.005655	-0.8257734
Nabp1	4.71E-09	-0.824793
Ablim3	0.000203	-0.824626
Pdk3	0.003675	-0.8245507
Brms1l	0.000695	-0.8242163
Arsb	5.14E-05	-0.8240352
Ogt	5.82E-33	-0.8238826
Fars2	5.02E-19	-0.8236281
Farp1	1.47E-18	-0.8235039
Lmo7	1.81E-36	-0.8232591
Plce1	4.03E-10	-0.8229311
Dgkd	3.37E-10	-0.8229131
Dapp1	4.37E-06	-0.8226793
Ascc2	8.33E-10	-0.8214902
Snx13	2.99E-17	-0.820981
Spop	4.48E-27	-0.8204233
Gpn1	0.047498	-0.8203362
Ing3	0.00114	-0.8200794
Mdfic	2.59E-20	-0.8194874
Lin9	0.016623	-0.8191929
Galk2	7.61E-08	-0.8183724
Bin3	5.01E-05	-0.8177544
Eif4e3	0.011984	-0.8173554
Hccs	0.048582	-0.8171754
Pi4k2b	1.15E-21	-0.8171728
Trp53bp2	1.81E-07	-0.8170513
Phf20	3.58E-10	-0.816849
Prrg3	0.005236	-0.8168273
Ern1	1.21E-07	-0.8165533
Mtx3	0.041641	-0.815737
Ric8b	4.65E-07	-0.8154698
Mib1	2.25E-16	-0.8151561
Cstf2t	0.041266	-0.8151489
Cyfip2	0.00111	-0.8149749
Gpatch2	4.58E-10	-0.8144205
Atxn2	8.99E-17	-0.8143446
Pick1	0.048243	-0.8142253
Far1	5.38E-14	-0.814214
Zcchc7	4.30E-56	-0.8140007
Phip	8.90E-23	-0.8138346
Atf6	1.19E-15	-0.8130857
Ubxn10	0.048803	-0.8127946
Baiap2l1	4.07E-13	-0.8127745
Srek1	5.00E-17	-0.8125117
Uap1	7.10E-06	-0.8124866
Kctd3	0.001026	-0.8122355
Snx8	0.000288	-0.8120799
Ggt5	0.036176	-0.8106646
Nedd4l	7.44E-52	-0.8106457
Pbx3	1.01E-26	-0.8105754
Slc33a1	3.09E-05	-0.81027
Tmem170b	5.11E-05	-0.8102155
Vps37a	1.61E-05	-0.81021
Uhrf1bp1l	9.26E-13	-0.8100154

Chrm3	1.72E-06	-0.8091923
Dusp16	8.16E-12	-0.8090174
Gm49539	0.003897	-0.8088664
Slc7a6	0.01423	-0.8085189
Ip6k2	0.000892	-0.8084072
Mcub	0.000448	-0.8082713
Stau1	1.05E-06	-0.8074631
Themis	0.000126	-0.8069167
Acad11	0.002024	-0.806892
Sfpq	2.37E-29	-0.8068266
Mpp7	7.38E-20	-0.8063482
Cd48	0.028394	-0.8061275
Dym	1.71E-17	-0.8057586
Cop1	1.89E-16	-0.8056407
Ccr2	0.006109	-0.805053
Map3k13	0.005194	-0.8048904
Rbm4	8.07E-06	-0.8041206
BE692007	0.009893	-0.8039142
Ppargc1a	0.026972	-0.8039136
Zmat1	0.014523	-0.8035732
Copz1	7.16E-06	-0.8034155
Ddx17	2.60E-39	-0.8033025
Efna5	5.37E-24	-0.8032704
Strn	2.04E-07	-0.8032402
Atad2b	5.50E-19	-0.8030235
Gm26549	0.0468	-0.8028418
Fbxw7	7.76E-17	-0.801881
Rcor3	8.90E-05	-0.8009487
Tial1	3.14E-06	-0.8007089
Fyb	1.24E-05	-0.8006149
Tent5c	3.89E-05	-0.800586
Xpo6	2.14E-06	-0.8004398
Pde8b	1.86E-12	-0.7995171
Abhd17b	1.66E-19	-0.7995124
Hook2	0.001481	-0.7992443
Ppp3ca	2.58E-103	-0.7989977
Csgalnact2	9.16E-06	-0.7989857
Gm20629	6.80E-09	-0.7985967
Dlg1	6.17E-20	-0.7981987
Ccdc85c	0.00307	-0.7978934
Tollip	0.000249	-0.7978503
Celsr1	0.001289	-0.797624
Foxo3	2.77E-18	-0.7970214
Zfp609	9.06E-16	-0.7967796
Gas2	0.021622	-0.7959228
Ifi47	0.033631	-0.7956165
Abl2	1.83E-15	-0.7955036
Maml3	1.41E-41	-0.7950737
Atp11b	2.37E-07	-0.7949032
Vasn	0.042155	-0.7948582
Eaf1	0.03395	-0.7946064
Foxa2	0.002847	-0.794565
Ube2q2	7.44E-06	-0.7942231
Dnah10	0.01906	-0.7923044
Sergef	0.032643	-0.7915946
Sp3	1.01E-09	-0.7914855
Tdrd3	2.56E-13	-0.79146
Snx15	0.002432	-0.7913953

Cdc27	2.02E-06	-0.7913061
Ubtd2	3.33E-05	-0.7909917
Bmpr1a	5.45E-09	-0.7907523
Tmod1	0.0108	-0.7901606
Cenpp	0.000197	-0.7898787
Sik1	0.00035	-0.7896788
Fcgr2b	0.000201	-0.7896766
Mtfr1	0.001966	-0.7895636
Zfx	1.12E-10	-0.7893681
Zfp607a	0.017441	-0.7891782
Rpap2	0.00924	-0.7890078
Lcp2	3.20E-06	-0.7886742
Dnajc14	0.001104	-0.788668
Wwox	1.24E-44	-0.7884977
Prune2	0.011105	-0.7882989
Nt5dc3	0.000692	-0.7882947
Bmp2k	3.16E-10	-0.7877548
Fbxo34	6.67E-06	-0.7875808
Optn	0.001522	-0.7869997
Arih1	4.41E-41	-0.7867496
Cwc27	1.26E-09	-0.7867
Snx24	5.63E-15	-0.786302
Cep44	0.030047	-0.786041
Phf8	8.26E-08	-0.7852117
Dcaf8	6.03E-11	-0.784393
Mynn	0.013428	-0.7834284
Zswim6	1.21E-31	-0.7831687
Zbtb37	2.84E-05	-0.7830479
Spred2	6.62E-07	-0.7829578
Zfp992	0.013594	-0.7829341
Lmln	0.014731	-0.7827007
Ap3b1	3.13E-17	-0.782681
Slc9a6	0.003824	-0.7825055
Tada1	0.007486	-0.7821696
Cnot4	2.74E-20	-0.7813582
Arhgap12	1.45E-10	-0.7811014
Minpp1	0.017442	-0.7808875
Dcaf15	0.046898	-0.7805976
Sec23b	4.26E-07	-0.780329
Slc25a37	6.74E-05	-0.7801636
Kif15	0.012503	-0.7797732
Ythdf3	5.75E-18	-0.7793276
Zbtb44	7.39E-07	-0.7786775
Cept1	1.14E-15	-0.7777179
Zfp507	0.012145	-0.7775016
Rsrc1	2.24E-14	-0.7765105
Hnrnpll	8.79E-09	-0.7761898
Klhl21	0.006039	-0.7757518
Trp53bp1	0.000186	-0.7756894
530001G21I	0.014479	-0.7755771
Msantd2	1.65E-08	-0.7754909
Kcnip4	5.32E-108	-0.775413
Larp4b	3.56E-24	-0.7749712
Arfgap1	0.004882	-0.7748468
Hnrnph1	1.21E-16	-0.7747192
Ccdc15	0.046222	-0.7743922
Sec22a	3.21E-06	-0.7743201
Arap1	0.000636	-0.7742984

Pde3b	3.18E-08	-0.7742759
Cdk13	3.49E-20	-0.7740936
Slc25a12	1.73E-05	-0.7740351
Phf14	6.34E-20	-0.7724435
Ammecr1	1.79E-05	-0.7719505
Ranbp6	0.022885	-0.771534
Tubgcp3	0.000475	-0.7712664
Trmt1l	5.80E-07	-0.7706772
Clmn	0.002797	-0.770303
Vti1a	2.11E-31	-0.7702425
Lockd	0.006286	-0.7698709
Tbc1d5	3.26E-36	-0.7696847
Zfand3	5.36E-45	-0.7693772
Vps13a	1.56E-15	-0.7693565
Fam107b	4.59E-08	-0.7688174
Chd2	4.18E-23	-0.7684653
Flvcr1	0.004405	-0.7684579
Mto1	0.009617	-0.7682221
Pde7b	5.25E-22	-0.7681309
Hbs1l	7.97E-05	-0.7680537
Adam9	6.42E-05	-0.7680333
R3hdm4	0.00074	-0.7678437
Sprtn	0.041761	-0.7662697
Mmp14	0.000468	-0.7661313
Morc2a	1.28E-05	-0.7660174
Slc25a36	2.71E-13	-0.7644051
Nup88	0.001063	-0.7642804
Creb5	1.24E-16	-0.7638163
Metap1	0.000224	-0.7633638
Neurl4	0.002276	-0.7632177
Nav2	4.17E-47	-0.7630575
Caap1	7.15E-08	-0.7630481
Txndc16	0.000496	-0.7628093
Ric1	3.90E-07	-0.7625853
Arid1b	5.61E-26	-0.7621916
Tbc1d8b	0.000997	-0.7616627
Ngly1	2.82E-05	-0.7614347
.30008F23R	0.045956	-0.7613917
Tbc1d14	2.26E-05	-0.7611891
Macrod2	8.56E-12	-0.76111
Ddb2	0.022599	-0.761043
Ulk4	0.009761	-0.7600533
Cnot7	0.000181	-0.7599191
Mtm1	1.96E-08	-0.7598711
Rasa3	8.19E-05	-0.7598094
Tox3	7.17E-09	-0.7596568
Erbb2	0.01225	-0.7589576
Znrf2	4.38E-10	-0.7588814
Gpam	2.40E-07	-0.7588433
Ugdh	0.007559	-0.7588283
Fat1	4.12E-05	-0.758297
Smyd3	4.75E-26	-0.7577199
Arhgap35	3.26E-09	-0.7574913
Rdh10	0.007896	-0.757289
Grk2	8.96E-05	-0.7572458
Pex7	6.57E-05	-0.7572192
Gtf2a1	9.34E-06	-0.7565629
Btbd10	1.44E-05	-0.756507

Rhobtb1	0.003579	-0.7561427
Cox10	0.001063	-0.7553799
Tob1	8.34E-05	-0.7551582
Dnajc6	0.022245	-0.7550737
Mcph1	0.001507	-0.7549904
Mir22hg	0.000583	-0.7540816
Lrp12	1.65E-05	-0.7535937
Nectin3	3.15E-09	-0.7522678
Taz	0.000295	-0.7507943
Dram1	5.67E-27	-0.7506058
Ppm1k	0.00534	-0.7505274
Naa30	0.003587	-0.7504312
Herc3	0.000155	-0.7498913
Emb	1.05E-08	-0.7498497
Bmt2	9.58E-07	-0.7493982
Sugct	7.33E-07	-0.7491847
Smim20	0.014841	-0.7487278
Bcat2	0.027153	-0.7483599
Ttc7	0.000109	-0.7479578
Rfx7	7.17E-11	-0.7477643
Dhx35	0.020668	-0.7476946
Zfp36	0.007849	-0.7470673
Taf15	8.90E-13	-0.7470304
3-Mar	1.00E-10	-0.7465801
Erp44	2.29E-07	-0.7460551
Col4a4	1.28E-10	-0.7460017
Hif1an	0.009259	-0.745955
Ppp3cb	2.47E-11	-0.7458695
Tnks	2.56E-11	-0.7456466
Strn3	7.82E-37	-0.745493
Src	0.010663	-0.7454023
Mpp6	5.88E-07	-0.7452691
Gstcd	0.007634	-0.7450703
Ccdc146	0.003605	-0.7449676
Tfcp2l1	0.00144	-0.7448311
Nol10	0.004503	-0.7447645
Mpp5	2.67E-09	-0.7442062
Tmem30a	1.45E-14	-0.743992
Dcun1d1	8.02E-07	-0.7439141
7-Mar	6.72E-13	-0.7436132
Rps6kc1	7.46E-05	-0.7432724
Ubr3	5.11E-18	-0.743165
Gpt2	0.035446	-0.7424367
Pkia	4.24E-05	-0.7421318
Fndc3b	1.39E-22	-0.7413811
Ehbp1	5.88E-15	-0.741317
Naaa	5.36E-07	-0.7409238
Mfsd6	2.30E-05	-0.7407927
Rnf121	0.026018	-0.7406875
Atf1	6.73E-05	-0.7404316
Tcf12	2.40E-50	-0.7400592
Lamp3	7.04E-28	-0.7400333
[illegible]30029C05[illegible]	0.002389	-0.7399752
Scarf2	0.015402	-0.739952
Relch	4.57E-12	-0.7399499
Tmem62	0.033248	-0.7397568
Rasa2	3.72E-08	-0.7396357
Lrrc23	0.010773	-0.7393922

Zfp626	0.023404	-0.7393902
33427G06F	0.01189	-0.7390541
Nlrp1b	0.030253	-0.7390214
Fbxw11	1.06E-12	-0.7389254
Scaper	2.16E-12	-0.7387675
Hsf2	0.005628	-0.7385995
Plpp3	9.75E-15	-0.7385292
Man2a1	5.12E-19	-0.7384582
Pard3	2.72E-40	-0.7383807
Mbnl1	1.50E-77	-0.7382442
Hps3	0.002733	-0.7380268
Tmem68	0.001144	-0.738007
Osbpl8	1.07E-16	-0.7379164
Mettl8	0.000379	-0.7374702
Fancl	0.000483	-0.7373678
Nfkb1	8.14E-23	-0.7372917
Cdkal1	1.27E-13	-0.7370317
Lcor	4.67E-20	-0.7370215
Mcm9	0.000791	-0.7369217
Tusc3	3.73E-08	-0.7369076
Rufy3	5.31E-08	-0.7365389
Foxp4	0.001769	-0.7364835
Abhd17c	0.000509	-0.7361698
Dnal1	0.038534	-0.7360925
B3gnt2	7.49E-06	-0.7360375
Nox4	9.74E-05	-0.7360087
Fubp1	5.07E-14	-0.7357692
Kdm2a	2.71E-17	-0.7355157
Arid2	4.30E-16	-0.7352818
Abcc1	0.00064	-0.73527
Usp54	0.000125	-0.7349337
Ubac2	2.36E-06	-0.7344635
Nemp2	0.016334	-0.7340916
Nt5e	0.002514	-0.7339977
Dyrk1a	9.04E-16	-0.7334008
Usf1	0.025216	-0.733127
Atp8b4	0.0015	-0.7325881
Nudcd1	0.013251	-0.7324459
Spry2	0.018508	-0.7323703
Nek5	0.012063	-0.732053
Smg7	3.37E-09	-0.731912
Mapre3	0.001488	-0.7316533
Hps5	0.004869	-0.7312773
Wdr44	3.62E-06	-0.7307772
Foxj3	1.05E-07	-0.7306946
Map3k14	0.000126	-0.7306587
Gigyf2	8.97E-13	-0.7301443
Elfn1	0.046503	-0.7298485
Smg1	3.97E-20	-0.7295984
Wnk1	6.42E-39	-0.7288954
Slc11a2	0.02895	-0.7284637
N4bp2l1	0.010992	-0.7272712
Rhoh	0.013541	-0.7269796
Acbd3	1.67E-06	-0.7269139
Trip12	4.83E-26	-0.7268545
Ids	0.002033	-0.7264532
Dedd	0.004228	-0.7262698
Rsrp1	1.33E-34	-0.7261541

00054A10F	0.000273	-0.7258685
Cd24a	1.72E-11	-0.7256217
Mtf2	1.12E-05	-0.7254832
Pias2	5.08E-09	-0.72491
Limch1	2.22E-42	-0.7249048
Cdc42se2	3.99E-08	-0.7248664
Pld1	2.65E-11	-0.7248603
Slc39a8	4.33E-11	-0.7245641
Zc3h12c	0.00023	-0.7245554
Hivep1	1.73E-05	-0.7242738
Gtf3c1	0.000112	-0.7240755
Hnrnpc	2.45E-16	-0.7236083
Cd84	2.51E-05	-0.7234821
Rars2	0.000254	-0.7233038
Nck2	6.33E-06	-0.722237
Yrdc	0.017118	-0.722179
Atg2b	0.02434	-0.7220756
Mcu	1.64E-12	-0.7217373
Snx30	0.000142	-0.7216832
Lrif1	5.21E-05	-0.7211646
Arl5a	0.000756	-0.7210998
Bbof1	0.009458	-0.7202067
Nrp2	6.35E-13	-0.7198632
Trio	3.69E-15	-0.7196801
Klf11	0.038511	-0.7189236
Atxn7	9.85E-13	-0.7188595
Megf9	9.16E-08	-0.7181653
Snrnp40	0.010945	-0.7180634
Snx2	3.36E-09	-0.7179544
Sdhaf3	0.044751	-0.7178801
Phf12	1.19E-05	-0.717333
Ipo7	0.000109	-0.7165817
Hacd3	5.60E-06	-0.7163931
Sf1	1.80E-06	-0.7162748
Gm42941	0.034825	-0.7160822
Tiam1	0.000234	-0.7155192
Dcaf6	6.04E-11	-0.7153834
Efcab7	0.014155	-0.7151251
Lilrb4a	0.000147	-0.7150086
Pcid2	0.017862	-0.7148926
Reln	1.94E-08	-0.7146836
Disp1	0.048294	-0.7145792
Zfp516	0.000973	-0.7145075
Dip2c	2.85E-42	-0.7143332
Dmd	1.87E-07	-0.7142268
Wdyhv1	0.00799	-0.7141966
Acpp	0.034899	-0.7139797
Rnf146	0.000885	-0.7136996
Psme4	8.75E-18	-0.7132907
Rbm12b2	0.026496	-0.7131893
Tuft1	0.027967	-0.7131131
310013L24R	8.25E-10	-0.7130056
Tpd52	4.72E-07	-0.7128938
Ptpn9	7.32E-07	-0.7127268
Nfia	5.43E-59	-0.7117027
Esyt2	9.28E-20	-0.7113478
Usf3	0.000169	-0.7113398
Elf1	3.10E-17	-0.7112235

Il18r1	1.51E-07	-0.710945
330032B11F	0.040558	-0.7105254
Dcaf5	1.42E-07	-0.7104956
Ppp6c	6.55E-05	-0.710091
Por	3.75E-22	-0.7099465
Swt1	1.36E-11	-0.7097157
Plekhm3	7.45E-06	-0.7096499
Crppa	0.013453	-0.7096166
Scpep1	0.029166	-0.7092669
Atp6v1c2	8.55E-09	-0.7091984
Nsmce2	1.26E-14	-0.708905
Fbxl17	4.12E-46	-0.7088597
Itsn1	4.22E-06	-0.7081659
Larp1b	5.88E-06	-0.7078648
Togaram1	2.76E-06	-0.7077457
10037D02F	9.02E-07	-0.7075493
Rbm6	2.94E-26	-0.7074701
Plekha1	1.71E-07	-0.7073417
10403D21F	0.005653	-0.7069264
Ptcd3	0.001585	-0.7066011
Cdc26	9.30E-05	-0.7065989
Tns3	1.14E-09	-0.7062105
Grb2	1.67E-13	-0.7061046
Cyp4v3	0.007904	-0.7058247
Cyp4f13	0.004199	-0.705603
Zc2hc1a	3.29E-06	-0.7053377
Gbf1	4.09E-16	-0.7048972
Pwwp2a	1.32E-06	-0.7047107
Tnfrsf1b	0.002763	-0.7036215
Slc39a9	0.000947	-0.7031543
Usp38	0.000289	-0.703152
Srcap	4.43E-08	-0.7027235
Tiparp	4.31E-05	-0.7018027
Abtb2	5.30E-07	-0.7016227
Cd180	0.043647	-0.701407
Slc43a2	6.66E-05	-0.7014035
330023F24F	8.63E-11	-0.7012263
Nfyc	1.69E-06	-0.7006669
BC017158	0.014762	-0.7004669
Ep300	1.29E-11	-0.7003941
Rmdn3	0.028811	-0.7000123
Fam172a	2.60E-25	-0.6998474
Dennd4c	8.92E-09	-0.6996618
Phc3	1.75E-09	-0.6983273
Zfp568	0.008757	-0.6972492
Sfmbt1	3.01E-05	-0.6965303
Ppp2r3c	0.036578	-0.6958625
Zfp26	0.005526	-0.6955335
Gbp3	0.007128	-0.6954009
Cabcoco1	0.00985	-0.6950132
Gphn	8.46E-58	-0.6949613
Sh3rf1	1.22E-20	-0.6949269
Ogdh	4.08E-10	-0.6947388
Ice2	0.016341	-0.6941104
Acoxl	8.62E-20	-0.6940131
Rnf145	2.23E-05	-0.6938992
Ago4	0.013056	-0.6936335
Iqgap2	1.89E-06	-0.6930309

Ccdc57	0.001518	-0.6930286
Ibtk	0.000158	-0.6921576
Abcb7	8.05E-06	-0.6921249
Tbc1d9	2.13E-06	-0.6918882
Adnp	1.00E-08	-0.6918333
Clpb	0.001128	-0.6914521
Stard7	0.000161	-0.6914143
Pex5	0.038544	-0.6913039
Cmtm6	0.018682	-0.690891
Rai1	8.72E-08	-0.6908867
Dyrk2	0.000564	-0.6907338
Xpo1	1.50E-10	-0.6904146
Bcl2l2	0.027999	-0.6899888
Pdp1	0.021731	-0.6898225
Lnpep	1.36E-17	-0.6898131
Hmg20a	1.46E-07	-0.6890014
Lrrc1	0.000358	-0.6889013
Pepd	0.011589	-0.6883402
Rab9	0.026987	-0.6882627
Stat4	0.026839	-0.6875206
Rabgap1	9.08E-15	-0.6875141
Wwp1	3.91E-12	-0.6856914
Exoc5	3.09E-08	-0.6852358
Batf2	0.046088	-0.6848711
Heca	6.74E-06	-0.6848525
Dync2h1	2.50E-07	-0.6843616
Ranbp2	2.63E-10	-0.6842835
Ippk	0.007207	-0.6841179
Prkaa2	0.000251	-0.6835978
Tgfbr1	3.42E-12	-0.682665
Dis3l2	4.88E-09	-0.6826411
Agtpbp1	4.20E-07	-0.6826339
Bcl11b	0.002351	-0.6824498
Plbd1	0.024853	-0.6817608
Tnfrsf21	0.00021	-0.6817509
Azin2	0.015874	-0.6815933
Samd4	3.63E-12	-0.681342
Gtf2b	0.002361	-0.6807667
Pgm1	0.00099	-0.6800995
Apbb1ip	1.57E-07	-0.6793694
Ythdc2	0.00133	-0.678416
Ctdp1	0.010498	-0.6779471
Ap3s1	4.99E-06	-0.6778583
Birc3	1.94E-06	-0.6775142
Cbl	3.38E-09	-0.6774255
Dtnbp1	0.000537	-0.6771707
Srbd1	9.37E-06	-0.6766365
Pum1	1.04E-15	-0.6763762
Tlr4	1.25E-05	-0.6763426
Bcl2	7.61E-10	-0.6760814
Vps41	1.14E-05	-0.6760134
Atp7a	7.91E-05	-0.6756973
Gatb	0.016995	-0.6756004
Sec63	1.35E-11	-0.6753502
Ap1g1	2.14E-08	-0.6752497
Mrtfa	7.32E-11	-0.6751352
Acss1	2.75E-05	-0.6745242
Myof	3.44E-11	-0.6742728

Tnfaip8	2.07E-10	-0.6741397
Ppp2ca	6.79E-07	-0.674104
Fbxo3	0.003897	-0.673791
Pias1	3.20E-14	-0.6734487
AY036118	2.28E-05	-0.6731049
Hsd17b12	0.0003	-0.6730289
Stimate	0.000186	-0.6729798
Asxl1	1.49E-12	-0.6726485
Dlg5	0.011514	-0.672455
Dock8	1.20E-11	-0.6717029
Usp8	9.37E-08	-0.671089
Pus10	0.007196	-0.6690509
Ralgapb	6.02E-08	-0.6686419
Bcap29	0.000251	-0.668548
Gsk3b	9.97E-19	-0.6683493
Fip1l1	1.49E-08	-0.6681046
Etv6	3.04E-25	-0.6675464
Babam2	2.31E-14	-0.667543
Pex14	0.000143	-0.6674049
Brwd3	0.000338	-0.6671608
Wsb1	0.000196	-0.667123
Iqsec1	3.84E-06	-0.667003
Nab1	0.001009	-0.6669513
Tspan31	0.029617	-0.6665153
Scai	9.19E-05	-0.666165
Stk3	5.44E-15	-0.6661393
Hivep3	4.97E-12	-0.6658591
Fan1	0.049165	-0.6657137
Smarcad1	0.00045	-0.6653422
Fbxo32	0.001923	-0.6650915
Alg13	0.025735	-0.664933
Lpcat2	0.025497	-0.6644727
30462N17F	9.75E-05	-0.6643164
Camk2d	3.29E-35	-0.6640449
Ireb2	1.99E-06	-0.6627287
Cog3	5.23E-05	-0.6624641
Elp4	2.15E-08	-0.6621504
Ubn2	1.09E-25	-0.6620084
Tle3	0.009943	-0.6619898
Ubp1	0.001344	-0.6619386
Nars2	0.00139	-0.6619176
Edrf1	0.00726	-0.6610035
Syncrip	6.92E-11	-0.6606913
Katnbl1	0.000938	-0.6600557
Kansl1	1.36E-36	-0.6600463
Slc4a5	0.011258	-0.6600461
Pon3	1.01E-06	-0.6599689
Nlgn1	0.037369	-0.659815
Usp39	0.021723	-0.6596855
Tcp11l2	0.000775	-0.6595503
Txndc12	0.028477	-0.6594043
Eri3	2.63E-07	-0.6593153
Lnx2	4.61E-06	-0.6590147
Traf1	0.039166	-0.6589909
Plekhj1	0.01338	-0.6589602
Max	0.008769	-0.6584701
Ofd1	0.020766	-0.6567995
Larp4	2.69E-05	-0.6565801

Slc1a5	0.00139	-0.6561112
Fra10ac1	0.042357	-0.6558401
Eefsec	0.000243	-0.655692
Gfpt1	8.60E-07	-0.6554438
32427E13R	1.22E-06	-0.6553732
Mtss1	9.75E-23	-0.6552792
Cep295	0.01029	-0.6549162
Fnbp4	4.95E-06	-0.6546791
Arfgef1	5.61E-14	-0.654449
Rps6ka3	1.76E-16	-0.6544419
Nt5dc1	0.000103	-0.6544261
Cog5	3.69E-12	-0.654342
Lpgat1	0.000198	-0.6543146
Tra2a	2.34E-16	-0.6539521
Gtdc1	2.72E-10	-0.6537478
Zfp930	0.0331	-0.6536737
Tlk1	1.11E-09	-0.6535624
Snx19	0.014083	-0.6534248
Ripk2	0.005754	-0.6531374
Nsmaf	6.82E-05	-0.653083
Kmt5c	0.008645	-0.6528482
Pdgfc	0.031986	-0.6527938
Cnot6	1.39E-05	-0.6523429
Dhx15	8.30E-07	-0.6522609
Kdm4c	1.32E-07	-0.6520576
Ptprc	1.93E-18	-0.6519103
R3hcc1l	2.11E-07	-0.6512814
Shroom4	7.12E-09	-0.6503014
Ptprj	6.01E-43	-0.6502836
Ints7	0.002918	-0.6500871
Nup205	0.03387	-0.6498029
Lhfpl2	0.00074	-0.6493282
Tapbp	1.78E-11	-0.6493041
Jade3	0.017168	-0.6492998
Ssr3	0.000146	-0.6490603
Arhgap25	0.001719	-0.6490564
Edem2	0.00349	-0.6490061
Mrps9	0.001219	-0.6489888
Akap7	6.81E-05	-0.6488095
Eva1c	6.16E-05	-0.648682
Slc8a1	4.61E-27	-0.6474181
Ank	0.012086	-0.647306
Gng2	0.00078	-0.6471058
Gls	5.01E-12	-0.6466458
Gm27003	0.018716	-0.6465457
Zdhhc9	0.013533	-0.6464297
Fam189a2	4.56E-05	-0.6462862
Ube2f	0.000382	-0.6462686
Kmt2d	0.000194	-0.6460891
Tbc1d30	0.00067	-0.6458891
Il11ra1	0.010482	-0.6456153
Atxn1	1.28E-52	-0.6456063
Gopc	0.000287	-0.6454588
Csnk1g1	3.39E-10	-0.6451321
Zfp207	3.04E-12	-0.6448755
Grk3	0.000413	-0.6448529
Shprh	8.68E-05	-0.6447338
Clint1	3.02E-11	-0.6443083

Enpp2	0.024458	-0.6441105
Ccdc25	0.010778	-0.6440933
Edem3	9.22E-05	-0.6440013
Col4a3	1.81E-05	-0.6437015
Mtmr3	5.66E-17	-0.6433921
Wdr11	0.001149	-0.6432665
Tmem260	6.11E-05	-0.6428669
Plaa	0.000628	-0.6426041
Gm26569	0.035235	-0.6424573
Ecpas	7.71E-11	-0.6423894
Odf2l	0.024596	-0.6419923
Slc37a1	0.002694	-0.6418821
Sil1	1.45E-08	-0.6418774
Erbin	1.47E-17	-0.6417151
Tmem161b	1.14E-05	-0.6407294
Med13	1.16E-11	-0.6406878
Ccdc91	0.003516	-0.640356
Tspan5	1.09E-09	-0.6400687
Cds1	0.01063	-0.6398505
Brcc3	0.003338	-0.6398132
Rere	8.23E-34	-0.6394697
Soat1	1.82E-11	-0.6390592
Kpna1	0.000756	-0.6390108
Tmem11	0.046288	-0.638578
Rad18	0.021403	-0.63852
Cpne1	2.05E-07	-0.6381443
Tmem87a	2.60E-05	-0.6376298
Ano1	0.000982	-0.637389
Rev1	0.000105	-0.6371166
Zfc3h1	9.79E-07	-0.6370827
Med14	0.000144	-0.6368298
Surf4	0.000158	-0.6366554
Sesn3	0.005105	-0.6364369
Rc3h2	6.32E-06	-0.6362976
Dtnb	1.28E-05	-0.6359745
Srsf4	2.06E-06	-0.6351606
Irf1	3.73E-05	-0.6349417
Akna	0.024386	-0.6348626
Npepps	1.91E-09	-0.6341025
Ppp4c	0.005057	-0.6336189
Itch	1.15E-13	-0.6336176
Ell2	8.92E-06	-0.6334942
Scmh1	1.08E-10	-0.6334829
Akap11	0.000213	-0.6334183
Pigl	0.032595	-0.633157
Blcap	0.033591	-0.6326679
Abcd2	0.000217	-0.6325388
Wdr20	1.62E-05	-0.6322458
Yap1	9.71E-05	-0.6316581
Prkaa1	0.001671	-0.6307932
Cyb5r1	0.028892	-0.6306981
Mecp2	1.29E-07	-0.6305536
Smchd1	3.70E-06	-0.6305157
Reps1	1.14E-05	-0.6304352
Atf4	0.001042	-0.6304108
Rlim	0.000561	-0.6303921
Rnf38	3.42E-09	-0.6302979
Adam10	1.52E-11	-0.6299171

Otud7b	2.51E-08	-0.6294707
Dop1a	2.49E-05	-0.6292827
Zfr	5.57E-15	-0.6290372
Plxnc1	0.027506	-0.62867
Mtus1	3.37E-10	-0.6285881
Thada	2.86E-08	-0.6284642
Tcf7l2	1.30E-27	-0.6284356
Srsf5	4.04E-15	-0.6282741
Dcbld2	0.024283	-0.6282198
Hyou1	0.008123	-0.6280207
Wdpcp	0.001535	-0.6278162
Usp32	1.04E-07	-0.627578
Tnrc6a	2.47E-11	-0.6272162
Rcc2	0.03548	-0.6271098
Nhs	0.001352	-0.6263862
Laptm4b	0.047941	-0.6260293
Paip1	0.000744	-0.6254219
Tbc1d32	0.000552	-0.6251406
Tmem135	9.57E-08	-0.6246716
Zmym2	4.57E-09	-0.6240087
Erlin1	0.034667	-0.6239419
Plagl2	0.035125	-0.6235494
Hexb	6.06E-06	-0.6233769
Lmbr1	1.23E-05	-0.6233387
Mbp	0.016405	-0.6232977
Zfp280d	3.11E-10	-0.6232298
Uvrag	1.98E-17	-0.623137
Rsbn1	2.03E-06	-0.6227494
Azin1	1.72E-05	-0.6226526
Amfr	0.00375	-0.6225939
Tmco4	0.019706	-0.6222249
Prorp	0.038312	-0.6221454
Rel	0.032478	-0.622016
Morc3	0.005012	-0.6219541
30480H06F	0.011283	-0.6217614
Mllt10	5.29E-22	-0.6215754
Xkr6	0.001825	-0.6215303
Fam120a	4.56E-09	-0.6214183
Rlf	4.32E-06	-0.6211935
Ncoa2	4.78E-20	-0.6206882
Dcun1d4	0.018064	-0.6204375
Suz12	1.18E-05	-0.6203624
Epc2	1.93E-07	-0.6202397
Itgav	0.000222	-0.6201467
Cyp39a1	0.011097	-0.6197947
Ube3a	1.68E-11	-0.6195977
8-Mar	0.001593	-0.6186117
Ube2k	4.73E-11	-0.6185218
N4bp2	0.015504	-0.6180269
Plcl1	1.10E-17	-0.6177785
Srpk2	1.13E-08	-0.6172235
Pan3	2.36E-27	-0.616843
Zfp369	0.010477	-0.6167109
Hacd2	0.016143	-0.6162496
Impact	0.000501	-0.6161663
Ubxn7	0.00101	-0.6159354
Nek7	2.52E-12	-0.6151279
Zbtb11	0.002006	-0.6148806

Cep126	0.039589	-0.6142945
Kdm5d	0.033427	-0.6140611
Nrf1	2.09E-09	-0.6139047
Sgms1	1.23E-25	-0.6132716
Txndc11	0.000103	-0.6127547
Tmem245	6.81E-09	-0.6121617
Abcc4	0.009473	-0.6120009
Hc	3.15E-16	-0.6118366
Mmaa	0.012753	-0.6116339
Zbtb25	0.046771	-0.6114629
Atp10a	0.000172	-0.6114383
Atp2a2	2.05E-09	-0.6112705
Tcerg1	0.000161	-0.6103406
Tex10	0.003444	-0.6103134
Camk4	0.000752	-0.6101367
Cpq	2.96E-06	-0.6097683
Rnf4	0.020067	-0.6092973
Sh2d4a	0.009553	-0.6092811
Tab2	5.64E-06	-0.6091112
Gvin1	0.001271	-0.6090606
Snx27	0.000233	-0.6088879
Zfp395	0.019128	-0.6088182
Cd44	1.20E-23	-0.6085231
Gpbp1	6.46E-09	-0.6082863
Unc93b1	0.005553	-0.6081789
)30021J03R	1.04E-07	-0.6078733
Mical2	3.74E-08	-0.6077583
Fbxw4	0.00069	-0.6075631
Wdr91	0.044056	-0.6066043
Large1	6.56E-14	-0.6065237
Cdon	0.027426	-0.6063579
Rhbdd1	0.001072	-0.6062718
Ehmt1	1.87E-09	-0.6058142
Il20rb	0.017266	-0.605728
Zc3h14	0.000647	-0.6056503
Ino80	2.44E-05	-0.6055364
Phkg2	0.034947	-0.6051763
Tiprl	0.013885	-0.604858
Kif21a	0.000999	-0.6045666
Gm16759	0.033553	-0.6038008
Adcy9	6.13E-06	-0.603765
Fhod1	0.006367	-0.6037543
Exoc6b	3.12E-17	-0.6037445
Zfand6	2.59E-10	-0.603666
Luc7l	0.003446	-0.6031823
Fcho2	1.43E-17	-0.6030364
Trim41	0.022301	-0.6024245
Gga1	0.003827	-0.601805
Xrcc4	1.36E-05	-0.6013265
Npnt	1.61E-08	-0.6010873
Azi2	0.000626	-0.6008185
Rictor	8.66E-08	-0.6007453
Zfp217	0.044781	-0.6004716
Crem	0.004116	-0.6003916
Olr1	0.012953	-0.5999502
Smg5	0.005338	-0.5993695
Ubap2l	1.06E-05	-0.5988456
Gm46218	0.00934	-0.5987344

Phtf2	0.004807	-0.5986952
Rprd1a	0.000559	-0.5986873
Shb	0.000297	-0.598659
Spin1	0.001662	-0.5986339
Wapl	2.79E-11	-0.5983377
Ppip5k2	0.011777	-0.5983129
Nbeal1	5.89E-13	-0.5981533
Cpsf6	3.92E-07	-0.598022
Ly75	0.000177	-0.5978961
Ranbp9	2.60E-07	-0.5975041
Sp4	0.000177	-0.5956947
Dock11	0.000305	-0.5954858
Mapk1ip1l	6.12E-05	-0.5951409
Slc10a7	4.97E-08	-0.5948511
Nktr	9.47E-10	-0.5947289
Ptprd	1.96E-05	-0.5942864
Mok	0.043805	-0.5940909
Cyb5b	2.63E-06	-0.5938183
Kantr	0.023221	-0.5937885
Stx18	0.003497	-0.5933053
Slf2	0.000437	-0.5932459
Setd5	2.19E-18	-0.5931778
Luc7l2	7.78E-32	-0.5929506
Zfp148	6.10E-11	-0.5926657
6-Mar	7.22E-06	-0.5924142
Clk4	3.63E-05	-0.592305
Ripor2	5.89E-06	-0.5921601
Srgap2	3.21E-17	-0.5921208
Map3k1	2.40E-05	-0.5917511
Dennd4a	6.80E-08	-0.591146
Kdm6a	1.18E-08	-0.591014
Fgd6	2.84E-05	-0.5908866
Clcn3	3.52E-05	-0.590684
Nmd3	0.014209	-0.5906164
Cyp51	0.020318	-0.5905709
Ubr1	2.04E-07	-0.5905491
Irak2	0.000178	-0.590387
Frs2	7.78E-07	-0.5903478
Trappc9	1.24E-06	-0.5903038
P2ry14	0.001626	-0.5902028

Supplementary Table S4. Differentially expressed genes (DEGs) between control and senescent endothelial cells (GSE197366)

Gene ID	Transcript	Total reads	P-value (Old and d-flow vs. Young and d-flow)	FDR step up (Old and d-flow vs. Young and d-flow)	Ratio (Old and d-flow vs. Young and d-flow)	Fold change (Old and d-flow vs. Young and d-flow)	LSMean(Old and d-flow) (Old and d-flow vs. Young and d-flow)	LSMean(Young and d-flow) (Old and d-flow vs. Young and d-flow)	Log_2ratio
Erdr1	Erdr1-204	3405.6	0.000003	0.000	159272.328	159272.328	31.358	0.000	17.28114
Rbpms	Rbpms-004	2489.3	0.000001	0.000	132422.714	132422.714	24.716	0.000	17.01479
Sec14l1	Sec14l1-001	2703.0	0.000069	0.001	122704.739	122704.739	20.024	0.000	16.90483
Setd1b	Setd1b-002	3247.7	0.000000	0.000	92089.597	92089.597	20.958	0.000	16.49075
Nufip2	Nufip2-004	6032.8	0.000006	0.000	70652.414	70652.414	45.531	0.001	16.10845
Trim47	Trim47-001	4174.1	0.000001	0.000	64705.624	64705.624	33.832	0.001	15.9816
Nfat5	Nfat5-004	13357.7	0.000001	0.000	57573.123	57573.123	85.391	0.001	15.81311
Clstn1	Clstn1-002	5492.6	0.000002	0.000	57168.260	57168.260	41.543	0.001	15.80293
Atp2b4	Atp2b4-002	6473.7	0.000019	0.000	38951.025	38951.025	45.592	0.001	15.24937
Stk11	Stk11-007	2295.7	0.000000	0.000	34831.957	34831.957	21.105	0.001	15.08812
Drap1	Drap1-001	3766.1	0.000005	0.000	23271.329	23271.329	32.641	0.001	14.50627
Glg1	Glg1-003	8761.3	0.000167	0.001	19490.473	19490.473	69.702	0.004	14.25048
Foxp1	Foxp1-021	6111.4	0.000001	0.000	19248.202	19248.202	35.393	0.002	14.23244
Eva1b	Eva1b-002	1072.8	0.000489	0.003	14859.768	14859.768	7.555	0.001	13.85912
Ubtf	Ubtf-011	4358.7	0.000003	0.000	14114.461	14114.461	28.871	0.002	13.78489
Aff1	Aff1-002	5117.1	0.000001	0.000	13070.537	13070.537	33.704	0.003	13.67403
Pard3	Pard3-001	1739.2	0.000015	0.000	10836.223	10836.223	16.152	0.001	13.40357
Prrg2	Prrg2-001	872.7	0.000178	0.001	10354.312	10354.312	6.432	0.001	13.33794
Amotl2	Amotl2-001	4017.0	0.000003	0.000	10004.494	10004.494	36.109	0.004	13.28836
Wnk1	Wnk1-005	1622.9	0.000001	0.000	8156.523	8156.523	13.818	0.002	12.99374
Asap2	Asap2-006	17068.4	0.000005	0.000	7570.515	7570.515	124.430	0.016	12.88618
Setd1b	Setd1b-201	4925.5	0.000000	0.000	7330.767	7330.767	44.973	0.006	12.83975
Vmac	Vmac-201	681.6	0.000018	0.000	5579.148	5579.148	6.078	0.001	12.44583
Fgfr1op2	Fgfr1op2-004	2398.8	0.000000	0.000	5290.604	5290.604	19.708	0.004	12.36922
Col6a3	Col6a3-202	1646.4	0.000012	0.000	4281.550	4281.550	5.551	0.001	12.06392
Acin1	Acin1-003	4985.6	0.000003	0.000	2756.595	2756.595	36.035	0.013	11.42867
Pan3	Pan3-003	807.5	0.000267	0.002	2728.453	2728.453	10.403	0.004	11.41387
Nf2	Nf2-002	897.2	0.001514	0.006	2436.772	2436.772	5.302	0.002	11.25076
Rn7s6	Rn7s6-201	692644.0	0.000000	0.000	2394.014	2394.014	6163.467	2.575	11.22522
Exoc6b	Exoc6b-006	2719.9	0.000003	0.000	2348.932	2348.932	17.936	0.008	11.19779
Hnrnpa3	Hnrnpa3-002	1443.3	0.000049	0.000	2080.874	2080.874	17.323	0.008	11.02297
Hdac4	Hdac4-201	2878.7	0.002181	0.008	2035.619	2035.619	23.349	0.011	10.99125
Mbnl1	Mbnl1-015	2707.7	0.000006	0.000	2004.430	2004.430	23.145	0.012	10.96898
Gm26461	Gm26461-201	171000.0	0.000000	0.000	1687.452	1687.452	1538.155	0.912	10.72063
Pfkfb3	Pfkfb3-015	649.8	0.000016	0.000	1483.781	1483.781	8.840	0.006	10.53506
Rpph1	Rpph1-201	379214.0	0.000000	0.000	1431.854	1431.854	3075.995	2.148	10.48367
Synj1	Synj1-003	1947.0	0.000001	0.000	1398.120	1398.120	14.202	0.010	10.44927
Rn7s1	Rn7s1-201	1583952.0	0.000000	0.000	1352.027	1352.027	13953.129	10.320	10.40091
Rnu3a	Rnu3a-201	22857.3	0.000000	0.000	1322.935	1322.935	218.339	0.165	10.36953
Rn7s2	Rn7s2-201	1612739.0	0.000000	0.000	1312.600	1312.600	14229.445	10.841	10.35821
Sgsh	Sgsh-006	531.5	0.001079	0.005	1299.134	1299.134	3.186	0.002	10.34333
Psmb8	Psmb8-003	1074.2	0.004333	0.014	1294.443	1294.443	7.612	0.006	10.33812
Cul4a	Cul4a-004	2211.9	0.000040	0.000	1250.035	1250.035	17.770	0.014	10.28775
Ino80e	Ino80e-003	513.3	0.002752	0.010	1239.107	1239.107	3.514	0.003	10.27508
10038B12R	0038B12Rik-	12853.7	0.000504	0.003	1231.095	1231.095	108.630	0.088	10.26573
Epha4	Epha4-001	747.5	0.000111	0.001	1162.291	1162.291	2.384	0.002	10.18276
Zxdc	Zxdc-003	439.4	0.000023	0.000	1131.433	1131.433	3.986	0.004	10.14393
Mecom	Mecom-014	631.3	0.000067	0.001	1120.289	1120.289	5.478	0.005	10.12966
Smarcb1	Smarcb1-004	1355.3	0.001872	0.007	1112.125	1112.125	11.392	0.010	10.1191
Fubp3	Fubp3-002	729.1	0.000040	0.000	1101.679	1101.679	6.322	0.006	10.10549
Myo18a	Myo18a-022	6086.4	0.000000	0.000	1101.590	1101.590	38.608	0.035	10.10537
Zhx3	Zhx3-201	2111.2	0.000464	0.003	1061.579	1061.579	14.691	0.014	10.052
Nt5c2	Nt5c2-001	3344.5	0.001510	0.006	1061.437	1061.437	20.968	0.020	10.0518
Kif1b	Kif1b-003	5848.0	0.000113	0.001	1023.506	1023.506	44.240	0.043	9.999303
Epha4	Epha4-003	1630.3	0.001237	0.005	1007.522	1007.522	12.831	0.013	9.976595
Rmrp	Rmrp-201	259493.2	0.000000	0.000	870.738	870.738	2309.956	2.653	9.766095
Lmo2	Lmo2-002	4218.8	0.000001	0.000	847.856	847.856	43.050	0.051	9.727675
Dmwd	Dmwd-001	404.5	0.000362	0.002	824.809	824.809	2.532	0.003	9.687916
Polg	Polg-003	1781.4	0.000010	0.000	819.990	819.990	13.650	0.017	9.679463
Adamts10	Adamts10-00(	3106.4	0.000000	0.000	807.844	807.844	18.599	0.023	9.657932
Klc2	Klc2-002	483.2	0.002381	0.009	792.526	792.526	4.395	0.006	9.630315
Mapk8ip3	Mapk8ip3-00	672.9	0.000082	0.001	790.271	790.271	5.699	0.007	9.626204
Prr3	Prr3-001	446.0	0.006960	0.021	759.900	759.900	4.134	0.005	9.569665
Otud5	Otud5-002	520.8	0.005704	0.018	743.995	743.995	4.336	0.006	9.539149
Kifc3	Kifc3-201	887.7	0.000000	0.000	729.921	729.921	5.639	0.008	9.511597
AA474408	AA474408-20	771389.0	0.000000	0.000	720.734	720.734	6550.945	9.089	9.493324
Gm28635	Gm28635-001	2428.8	0.000002	0.000	715.517	715.517	20.154	0.028	9.482843
Nfic	Nfic-002	4827.7	0.000079	0.001	688.883	688.883	39.737	0.058	9.428115
Kifc3	Kifc3-203	439.9	0.000021	0.000	688.281	688.281	2.224	0.003	9.426854
Tcf7l2	Tcf7l2-001	771.4	0.000117	0.001	670.975	670.975	4.442	0.007	9.390116
Srrm1	Srrm1-015	9378.8	0.000000	0.000	670.577	670.577	78.878	0.118	9.38926
Cnot2	Cnot2-006	529.6	0.001719	0.007	655.287	655.287	4.351	0.007	9.355982
Ttbk2	Ttbk2-003	1010.1	0.000842	0.004	599.961	599.961	7.457	0.012	9.228724
Pik3r4	Pik3r4-007	1035.6	0.000558	0.003	588.891	588.891	4.586	0.008	9.201857
Fyn	Fyn-003	512.0	0.003158	0.011	579.427	579.427	1.717	0.003	9.178483
Herpud1	Herpud1-003	616.4	0.004608	0.015	558.817	558.817	4.197	0.008	9.126231
Xpo4	Xpo4-002	327.8	0.009710	0.027	552.605	552.605	1.876	0.003	9.110104
Cic	Cic-015	1301.5	0.000236	0.002	542.473	542.473	12.582	0.023	9.083408
Matr3	Matr3-008	1700.1	0.000229	0.001	539.958	539.958	19.387	0.036	9.076705
Snora73b	Snora73b-201	6342.2	0.000082	0.001	528.687	528.687	54.938	0.104	9.046269
Entpd4	Entpd4-201	657.4	0.001363	0.006	504.217	504.217	6.297	0.012	8.9779
Vegfa	Vegfa-201	5267.0	0.000249	0.002	501.635	501.635	38.526	0.077	8.970495
Ralgds	Ralgds-002	593.4	0.000244	0.002	469.291	469.291	6.523	0.014	8.874339
Sipa1l3	Sipa1l3-014	473.3	0.003922	0.013	431.356	431.356	3.713	0.009	8.752736
Gatad2b	Gatad2b-201	1367.9	0.005334	0.017	411.950	411.950	12.337	0.030	8.686325
Fam107a	Fam107a-003	6334.0	0.000109	0.001	403.949	403.949	28.078	0.070	8.658029
Cdan1	Cdan1-002	968.7	0.014478	0.037	396.960	396.960	6.286	0.016	8.63285
Cbfa2t3	Cbfa2t3-002	8660.2	0.000390	0.002	392.204	392.204	58.301	0.149	8.615462
Ctage5	Ctage5-008	1967.1	0.000004	0.000	390.984	390.984	18.451	0.047	8.610966
Slc4a3	Slc4a3-010	1793.6	0.003659	0.013	389.644	389.644	10.226	0.026	8.606015
Map4k4	Map4k4-004	322.5	0.000452	0.002	383.589	383.589	3.695	0.010	8.583418
Ldb1	Ldb1-007	691.6	0.000021	0.000	374.149	374.149	5.310	0.014	8.547468
Samd4	Samd4-001	2517.6	0.000082	0.001	364.823	364.823	30.677	0.084	8.511055
Morf4l1	Morf4l1-013	9626.4	0.000134	0.001	346.986	346.986	78.361	0.226	8.438732
U3	U3.4-201	7480.8	0.000000	0.000	345.835	345.835	71.793	0.208	8.433941
Far1	Far1-201	1580.8	0.000428	0.002	341.055	341.055	11.741	0.034	8.413859
Arhgap12	Arhgap12-002	382.2	0.000237	0.002	335.823	335.823	3.179	0.009	8.391557
P23-446O1	23-446O11.1-	630565.0	0.000000	0.000	329.703	329.703	4991.418	15.139	8.365024
Smagp	Smagp-201	239.9	0.000564	0.003	327.624	327.624	1.097	0.003	8.355898
Mapk3	Mapk3-003	287.2	0.008693	0.025	323.125	323.125	3.721	0.012	8.335949

Mir6240	Mir6240-201	69095.0	0.000000	0.000	320.333	320.333	609.300	1.902	8.323429
Bnip2	Bnip2-201	2949.4	0.000817	0.004	311.606	311.606	21.967	0.070	8.283577
Gan	Gan-201	740.5	0.000739	0.004	298.743	298.743	4.577	0.015	8.222762
Matr3	Matr3-007	1376.1	0.000525	0.003	295.393	295.393	12.226	0.041	8.206492
Morc3	Morc3-002	404.9	0.000363	0.002	289.469	289.469	2.303	0.008	8.177264
Psmc3	Psmc3-010	2909.2	0.001939	0.008	284.701	284.701	20.141	0.071	8.153303
Snora68	Snora68-201	8157.0	0.000000	0.000	265.082	265.082	70.083	0.264	8.050292
Plrg1	Plrg1-002	660.6	0.000881	0.004	250.891	250.891	7.462	0.030	7.970915
Gse1	Gse1-001	2289.8	0.000165	0.001	242.450	242.450	15.689	0.065	7.921542
Ehd4	Ehd4-004	7659.2	0.000000	0.000	233.467	233.467	56.557	0.242	7.867078
Snord22	Snord22-201	48164.0	0.000000	0.000	229.317	229.317	421.635	1.839	7.841198
Asap1	Asap1-008	2295.6	0.000039	0.000	225.236	225.236	16.600	0.074	7.815291
D5Ertd579e	5Ertd579e-00	1030.6	0.004432	0.015	221.660	221.660	8.111	0.037	7.792205
Rcan2	Rcan2-202	220.0	0.000074	0.001	218.399	218.399	1.832	0.008	7.770821
Mul1	Mul1-001	950.7	0.000315	0.002	212.103	212.103	8.684	0.041	7.728624
Tpt1	Tpt1-006	48115.6	0.000000	0.000	210.010	210.010	480.938	2.290	7.714312
Tial1	Tial1-004	1173.6	0.000009	0.000	209.705	209.705	13.663	0.065	7.71222
Rtn4	Rtn4-006	5556.7	0.002145	0.008	204.665	204.665	45.374	0.222	7.677121
Ehmt2	Ehmt2-002	471.1	0.000130	0.001	203.576	203.576	4.654	0.023	7.669423
Tns1	Tns1-020	11875.0	0.000003	0.000	199.506	199.506	90.042	0.451	7.640292
Stx3	Stx3-201	362.9	0.000495	0.003	197.805	197.805	3.929	0.020	7.627931
Thap4	Thap4-002	799.8	0.000651	0.003	196.915	196.915	8.156	0.041	7.621432
Luc7l2	Luc7l2-007	8639.6	0.000616	0.003	191.065	191.065	67.379	0.353	7.577921
10403A07R	0403A07Rik-	1936.9	0.002379	0.009	190.795	190.795	15.421	0.081	7.575876
Il6st	Il6st-007	5481.4	0.000127	0.001	190.056	190.056	54.552	0.287	7.570282
Map7	Map7-201	715.8	0.005920	0.018	185.677	185.677	5.886	0.032	7.536648
Zeb2	Zeb2-009	633.2	0.000142	0.001	183.070	183.070	5.232	0.029	7.516254
Gm25117	Gm25117-201	3475.0	0.000000	0.000	182.603	182.603	28.818	0.158	7.512563
Polr3gl	Polr3gl-002	1176.5	0.000135	0.001	181.737	181.737	10.356	0.057	7.505712
Golim4	Golim4-201	21029.5	0.000000	0.000	179.635	179.635	156.140	0.869	7.488928
Dnm2	Dnm2-001	4161.9	0.000097	0.001	179.260	179.260	29.255	0.163	7.485913
Rnf214	Rnf214-001	310.0	0.005632	0.018	175.858	175.858	1.499	0.009	7.458267
Ywhae	Ywhae-002	7998.0	0.000000	0.000	175.786	175.786	72.856	0.414	7.45768
Ewsr1	Ewsr1-009	5565.7	0.007954	0.023	175.702	175.702	46.191	0.263	7.456988
Hipk1	Hipk1-005	1672.6	0.002551	0.009	172.972	172.972	18.004	0.104	7.434393
Hnrnph1	Hnrnph1-008	2881.6	0.000000	0.000	171.432	171.432	24.720	0.144	7.421495
Timp2	Timp2-002	15037.7	0.000000	0.000	170.746	170.746	112.398	0.658	7.415711
Snord17	Snord17-201	10923.0	0.000000	0.000	170.318	170.318	93.539	0.549	7.412084
Ramp2	Ramp2-001	6414.5	0.000968	0.004	168.253	168.253	31.853	0.189	7.394489
Prpf40b	Prpf40b-006	522.4	0.004624	0.015	166.970	166.970	3.328	0.020	7.383443
Nmnat2	Nmnat2-002	1545.2	0.034473	0.073	161.147	161.147	3.952	0.025	7.332231
Vrk2	Vrk2-201	718.4	0.000104	0.001	159.529	159.529	4.671	0.029	7.317672
Zcchc9	Zcchc9-003	401.2	0.014909	0.038	158.853	158.853	3.266	0.021	7.311547
Nynrin	Nynrin-201	3147.8	0.000001	0.000	157.962	157.962	27.166	0.172	7.303434
Sfi1	Sfi1-001	800.0	0.007645	0.022	157.263	157.263	6.112	0.039	7.297038
Usf2	Usf2-011	1611.5	0.010957	0.030	154.122	154.122	13.458	0.087	7.267926
Arhgap12	Arhgap12-001	622.4	0.001809	0.007	153.045	153.045	5.458	0.036	7.257816
Foxp1	Foxp1-007	295.4	0.000260	0.002	147.567	147.567	2.746	0.019	7.205227
Gm26397	Gm26397-201	3597.0	0.000000	0.000	144.616	144.616	30.269	0.209	7.176088
Capn5	Capn5-002	1184.6	0.003360	0.012	144.349	144.349	4.413	0.031	7.173417
Asap1	Asap1-007	1778.5	0.006657	0.020	143.161	143.161	16.343	0.114	7.161492
Brcc3	Brcc3-008	297.4	0.002713	0.010	142.960	142.960	3.140	0.022	7.159467
Myh9	Myh9-005	4124.8	0.012988	0.034	141.521	141.521	38.404	0.271	7.144869
Arhgef40	Arhgef40-005	780.9	0.000203	0.001	140.778	140.778	4.364	0.031	7.137275
Ikbip	Ikbip-005	877.8	0.000558	0.003	138.964	138.964	5.667	0.041	7.118567
Cul1	Cul1-002	1430.6	0.000024	0.000	138.334	138.334	15.021	0.109	7.112009
Apoe	Apoe-002	48924.7	0.002292	0.009	138.155	138.155	450.532	3.261	7.110149
Nudc	Nudc-003	1787.2	0.001145	0.005	137.858	137.858	18.237	0.132	7.10704
Fn1	Fn1-017	3921.3	0.009454	0.026	137.809	137.809	38.055	0.276	7.106528
Sclt1	Sclt1-002	335.1	0.014099	0.036	137.796	137.796	4.464	0.032	7.106392
Ica1	Ica1-001	763.6	0.003785	0.013	135.663	135.663	6.648	0.049	7.083884
Ephb4	Ephb4-004	973.6	0.003765	0.013	133.415	133.415	9.771	0.073	7.059777
Plec	Plec-015	18313.0	0.001998	0.008	132.291	132.291	89.571	0.677	7.047568
Cyb5a	Cyb5a-005	6035.9	0.018875	0.045	131.509	131.509	45.965	0.350	7.039014
Hdgf	Hdgf-006	4364.2	0.000000	0.000	130.786	130.786	35.727	0.273	7.031065
Bsg	Bsg-005	12499.5	0.013430	0.035	127.734	127.734	88.237	0.691	6.996996
Cald1	Cald1-007	6568.0	0.010698	0.029	127.201	127.201	77.020	0.605	6.990961
R3hdm2	R3hdm2-001	1579.8	0.000017	0.000	122.703	122.703	8.060	0.066	6.939022
Dhx16	Dhx16-002	2900.3	0.000009	0.000	120.738	120.738	27.764	0.230	6.915738
Add1	Add1-004	5592.8	0.000000	0.000	120.376	120.376	44.766	0.372	6.911404
P4hb	P4hb-002	10428.4	0.000000	0.000	119.255	119.255	72.319	0.606	6.897911
Tnks1bp1	Tnks1bp1-001	4221.0	0.000539	0.003	117.742	117.742	31.341	0.266	6.879487
Fryl	Fryl-201	2848.9	0.000002	0.000	116.678	116.678	28.340	0.243	6.866387
Sfmbt1	Sfmbt1-004	237.3	0.002307	0.009	116.608	116.608	1.325	0.011	6.865518
Actg1	Actg1-007	6652.4	0.000361	0.002	116.168	116.168	44.727	0.385	6.860074
Gnb2l1	Gnb2l1-003	4502.4	0.000700	0.003	116.097	116.097	44.342	0.382	6.859188
Gnas	Gnas-018	1258.9	0.000010	0.000	115.910	115.910	7.784	0.067	6.856859
Skil	Skil-003	5863.2	0.008221	0.024	115.704	115.704	50.631	0.438	6.854298
Ube2h	Ube2h-002	1449.0	0.000004	0.000	115.367	115.367	17.811	0.154	6.850084
Tfe3	Tfe3-003	301.0	0.013213	0.034	114.872	114.872	1.558	0.014	6.843883
Usp19	Usp19-001	1254.4	0.000892	0.004	114.319	114.319	13.019	0.114	6.836923
Lrrc49	Lrrc49-005	631.1	0.002720	0.010	114.041	114.041	2.510	0.022	6.83341
Wdr36	Wdr36-202	1143.0	0.000638	0.003	112.743	112.743	10.398	0.092	6.816897
Cdc34	Cdc34-201	330.1	0.006346	0.019	112.147	112.147	2.721	0.024	6.809252
Ehmt2	Ehmt2-001	490.8	0.003098	0.011	110.959	110.959	4.015	0.036	6.793883
Sh3glb1	Sh3glb1-005	5282.1	0.000000	0.000	109.932	109.932	39.924	0.363	6.780471
Psme1	Psme1-006	2286.7	0.000087	0.001	109.724	109.724	23.168	0.211	6.777735
Hnrnpk	Hnrnpk-011	1055.9	0.004070	0.014	109.673	109.673	10.853	0.099	6.777063
Egfl7	Egfl7-008	8018.9	0.000000	0.000	108.795	108.795	62.269	0.572	6.765473
Egfl7	Egfl7-002	24374.1	0.001872	0.007	108.404	108.404	162.088	1.495	6.760269
Dab2ip	Dab2ip-006	10923.5	0.000000	0.000	108.134	108.134	51.152	0.473	6.756671
Zbtb20	Zbtb20-012	6527.6	0.002324	0.009	107.776	107.776	44.591	0.414	6.751891
Clu	Clu-007	4247.8	0.000001	0.000	105.398	105.398	37.951	0.360	6.719707
Gm24270	Gm24270-201	7371.0	0.000002	0.000	101.434	101.434	63.927	0.630	6.664395
Atp2c1	Atp2c1-006	199.6	0.011823	0.031	101.168	101.168	2.576	0.025	6.660605
Rbm39	Rbm39-020	2630.6	0.001505	0.006	100.936	100.936	21.194	0.210	6.657293
Dis3l	Dis3l-003	204.0	0.009460	0.026	100.552	100.552	2.356	0.023	6.651792
Tef	Tef-006	3144.3	0.016231	0.040	98.834	98.834	27.296	0.276	6.626941
Apoe	Apoe-007	8168.7	0.022421	0.052	98.740	98.740	62.602	0.634	6.625568
Eln	Eln-003	22396.1	0.000193	0.001	98.497	98.497	123.031	1.249	6.622011
Nfat5	Nfat5-007	2363.1	0.000071	0.001	97.537	97.537	26.081	0.267	6.607871
Stoml2	Stoml2-009	97.1	0.009812	0.027	97.037	97.037	0.921	0.009	6.600466
Anp32a	Anp32a-010	1722.6	0.000323	0.002	96.841	96.841	13.552	0.140	6.597551
Zeb2	Zeb2-011	693.0	0.000700	0.003	96.586	96.586	6.113	0.063	6.593738
Gripap1	Gripap1-002	612.9	0.023501	0.054	96.480	96.480	4.122	0.043	6.592152
Htra1	Htra1-001	1298.3	0.000192	0.001	96.196	96.196	9.353	0.097	6.587912
Gm24245	Gm24245-201	5879.0	0.000000	0.000	95.645	95.645	50.189	0.525	6.579615

Rbm39	Rbm39-004	237.3	0.001577	0.007	94.575	94.575	2.387	0.025	6.563394
Pogk	Pogk-003	1999.1	0.005492	0.017	94.431	94.431	16.956	0.180	6.561187
Odf2	Odf2-004	1328.4	0.005439	0.017	93.385	93.385	12.601	0.135	6.545122
Nat9	Nat9-001	449.8	0.014472	0.037	92.650	92.650	3.702	0.040	6.533722
Arhgef40	Arhgef40-006	1662.0	0.000264	0.002	91.888	91.888	8.844	0.096	6.521803
Safb2	Safb2-004	2331.8	0.000040	0.000	90.684	90.684	16.274	0.179	6.502771
Trappc6a	Trappc6a-001	244.0	0.002006	0.008	89.615	89.615	1.237	0.014	6.485661
Gm24119	Gm24119-201	6250.0	0.000000	0.000	89.315	89.315	52.033	0.583	6.480827
Ergic2	Ergic2-015	1255.9	0.001573	0.006	89.116	89.116	9.354	0.105	6.477616
Odf2	Odf2-005	545.1	0.034896	0.074	88.924	88.924	1.642	0.018	6.474497
Mypop	Mypop-201	457.2	0.000081	0.001	87.785	87.785	4.457	0.051	6.455906
Adar	Adar-002	2698.8	0.003953	0.013	87.674	87.674	16.922	0.193	6.45407
Rrp9	Rrp9-001	765.5	0.000432	0.002	86.456	86.456	6.814	0.079	6.433891
Pls3	Pls3-004	10972.8	0.000002	0.000	86.212	86.212	99.065	1.149	6.429811
Dnajb12	Dnajb12-005	1045.1	0.002878	0.010	86.072	86.072	8.968	0.104	6.427473
Denr	Denr-002	667.0	0.001466	0.006	85.358	85.358	5.913	0.069	6.415456
Psen2	Psen2-001	2262.7	0.005110	0.016	85.164	85.164	22.563	0.265	6.412165
Lmo2	Lmo2-005	7594.3	0.000003	0.000	85.101	85.101	67.683	0.795	6.411099
Slc6a8	Slc6a8-001	1342.2	0.000700	0.003	84.959	84.959	12.340	0.145	6.408688
Trappc13	Trappc13-003	636.5	0.002100	0.008	84.852	84.852	5.552	0.065	6.406882
Tbc1d17	Tbc1d17-001	564.9	0.002439	0.009	84.586	84.586	5.112	0.060	6.402345
Tln1	Tln1-005	1992.7	0.000593	0.003	84.123	84.123	17.173	0.204	6.394434
Bicd1	Bicd1-003	547.0	0.019192	0.046	83.904	83.904	3.539	0.042	6.390662
Atp2c1	Atp2c1-012	600.5	0.000061	0.001	83.844	83.844	2.763	0.033	6.389641
Myo18a	Myo18a-002	5648.8	0.000000	0.000	83.428	83.428	41.002	0.491	6.382454
Rbbp5	Rbbp5-005	900.3	0.000645	0.003	82.935	82.935	7.516	0.091	6.373912
Ube3a	Ube3a-002	3921.6	0.000790	0.004	82.309	82.309	29.689	0.361	6.362976
Otud5	Otud5-006	1391.5	0.009378	0.026	81.261	81.261	9.917	0.122	6.344496
St6galnac6	St6galnac6-00	146.9	0.041430	0.084	81.189	81.189	0.870	0.011	6.343221
Scp2	Scp2-007	2918.8	0.001785	0.007	80.739	80.739	21.874	0.271	6.335198
Cav1	Cav1-009	4082.9	0.002753	0.010	79.219	79.219	23.115	0.292	6.307775
Fhl1	Fhl1-008	3368.7	0.000000	0.000	78.564	78.564	17.482	0.223	6.295793
Gnb2	Gnb2-001	3747.9	0.003610	0.012	78.256	78.256	26.694	0.341	6.290133
Ahnak	Ahnak-201	38580.5	0.000000	0.000	77.806	77.806	228.434	2.936	6.281803
Mdfic	Mdfic-004	1985.8	0.001717	0.007	75.902	75.902	14.603	0.192	6.246072
Zkscan1	Zkscan1-002	1115.6	0.007246	0.021	75.733	75.733	10.380	0.137	6.242852
Tns1	Tns1-004	6678.3	0.000000	0.000	75.507	75.507	48.653	0.644	6.238547
Ddx26b	Ddx26b-201	329.1	0.002236	0.009	75.429	75.429	3.622	0.048	6.237045
Gpatch2	Gpatch2-201	1004.5	0.017774	0.043	74.565	74.565	7.897	0.106	6.220436
Ablim1	Ablim1-002	5974.6	0.012450	0.033	74.180	74.180	57.263	0.772	6.212952
Oplah	Oplah-201	1834.6	0.000022	0.000	74.154	74.154	15.375	0.207	6.212448
Ivns1abp	Ivns1abp-004	742.0	0.000274	0.002	73.846	73.846	7.820	0.106	6.206448
Epha5	Epha5-002	1092.4	0.000005	0.000	73.109	73.109	8.448	0.116	6.191985
Rtn4	Rtn4-005	5622.4	0.000181	0.001	72.820	72.820	44.675	0.614	6.186263
Gm26035	Gm26035-201	9361.0	0.000002	0.000	72.731	72.731	84.183	1.157	6.184499
Hsph1	Hsph1-002	941.3	0.001595	0.007	72.363	72.363	9.046	0.125	6.177182
Cspp1	Cspp1-017	945.3	0.001570	0.006	70.941	70.941	7.766	0.109	6.148557
Rn7sk	Rn7sk-201	439544.0	0.000000	0.000	70.201	70.201	3816.435	54.364	6.133424
Ly6c1	Ly6c1-003	2378.5	0.002335	0.009	69.983	69.983	9.919	0.142	6.128924
Shc4	Shc4-002	902.8	0.002711	0.010	69.953	69.953	7.700	0.110	6.128313
Git2	Git2-006	2345.2	0.002923	0.011	69.707	69.707	22.262	0.319	6.123223
Dlg4	Dlg4-001	790.3	0.000538	0.003	69.198	69.198	4.890	0.071	6.112668
Cggbp1	Cggbp1-002	1335.4	0.000000	0.000	68.830	68.830	10.493	0.152	6.104964
Ccdc23	Ccdc23-001	364.8	0.000325	0.002	68.830	68.830	3.124	0.045	6.104962
Arhgap12	Arhgap12-006	903.5	0.001799	0.007	68.669	68.669	8.510	0.124	6.101586
Actb	Actb-003	22342.5	0.000001	0.000	68.486	68.486	241.149	3.521	6.09774
Gm23935	Gm23935-201	6013.0	0.000000	0.000	68.389	68.389	52.021	0.761	6.095695
Igsf8	Igsf8-001	1428.0	0.000008	0.000	68.131	68.131	13.785	0.202	6.09023
Arpc1b	Arpc1b-003	3660.9	0.000004	0.000	67.990	67.990	28.128	0.414	6.08726
Atp8b2	Atp8b2-002	3308.9	0.000930	0.004	67.642	67.642	18.905	0.279	6.079843
Ccnyl1	Ccnyl1-003	2488.0	0.000000	0.000	67.608	67.608	12.669	0.187	6.079132
Snora30	Snora30-201	5641.0	0.000000	0.000	67.386	67.386	48.222	0.716	6.074382
Gpr174	Gpr174-001	2018.0	0.000002	0.000	65.963	65.963	17.445	0.264	6.043581
Ralgapa2	Ralgapa2-007	1176.4	0.003013	0.011	65.785	65.785	6.821	0.104	6.039698
Ern1	Ern1-003	2953.6	0.002084	0.008	65.395	65.395	18.027	0.276	6.031101
Dst	Dst-009	36727.0	0.000001	0.000	64.248	64.248	299.582	4.663	6.005578
Rps15a	Rps15a-003	4283.7	0.000000	0.000	64.012	64.012	39.533	0.618	6.00026
Tle1	Tle1-001	2506.5	0.009965	0.028	63.626	63.626	21.486	0.338	5.991539
Pygo2	Pygo2-001	1460.3	0.000000	0.000	63.263	63.263	13.602	0.215	5.983286
Nab1	Nab1-002	1807.2	0.006284	0.019	62.643	62.643	12.846	0.205	5.969075
Fez1	Fez1-201	3078.5	0.000016	0.000	61.521	61.521	29.627	0.482	5.943016
Sharpin	Sharpin-001	609.0	0.012965	0.034	61.074	61.074	6.253	0.102	5.932488
Ddx23	Ddx23-005	976.3	0.000001	0.000	60.691	60.691	7.881	0.130	5.9234
Srsf2	Srsf2-201	553.1	0.000045	0.000	59.576	59.576	6.370	0.107	5.896668
Zfp467	Zfp467-002	1362.8	0.031887	0.069	59.360	59.360	9.877	0.166	5.891419
Pard3	Pard3-015	333.8	0.014281	0.036	59.212	59.212	2.284	0.039	5.887828
Gm24455	Gm24455-201	1779.0	0.000001	0.000	59.064	59.064	14.196	0.240	5.884205
Trnau1ap	Trnau1ap-011	259.7	0.045652	0.091	58.987	58.987	2.894	0.049	5.882325
Cald1	Cald1-004	674.7	0.000004	0.000	58.807	58.807	7.626	0.130	5.877915
H2-Q4	H2-Q4-001	12504.5	0.000000	0.000	58.262	58.262	102.373	1.757	5.864475
Tom1l1	Tom1l1-002	760.7	0.000409	0.002	58.081	58.081	2.613	0.045	5.859991
Adar	Adar-005	1179.5	0.000002	0.000	57.783	57.783	7.210	0.125	5.85258
Palmd	Palmd-003	2524.3	0.000526	0.003	57.775	57.775	8.599	0.149	5.852369
Adck1	Adck1-202	214.1	0.003996	0.013	57.603	57.603	2.599	0.045	5.848077
Gm26493	Gm26493-201	1912.0	0.000002	0.000	57.421	57.421	15.339	0.267	5.843502
Rhoa	Rhoa-002	7265.5	0.000731	0.004	57.213	57.213	45.110	0.788	5.838277
Plekhh3	Plekhh3-001	1215.6	0.002888	0.010	56.889	56.889	7.329	0.129	5.830082
Nr6a1	Nr6a1-001	710.8	0.000011	0.000	56.609	56.609	5.086	0.090	5.822966
Lrrfip1	Lrrfip1-006	418.7	0.002614	0.010	56.149	56.149	3.942	0.070	5.811187
Scfd2	Scfd2-003	2454.5	0.000001	0.000	56.023	56.023	22.665	0.405	5.807938
Itgb1	Itgb1-002	11822.8	0.000000	0.000	55.959	55.959	90.459	1.617	5.806303
Cic	Cic-201	232.8	0.009253	0.026	55.889	55.889	3.297	0.059	5.804482
Elac2	Elac2-003	731.3	0.003012	0.011	55.843	55.843	8.630	0.155	5.803302
Ptma	Ptma-006	4321.9	0.000001	0.000	55.629	55.629	37.115	0.667	5.79777
Usp7	Usp7-002	4378.7	0.000026	0.000	55.356	55.356	33.547	0.606	5.790665
Pfkfb3	Pfkfb3-006	590.9	0.034772	0.074	55.289	55.289	6.439	0.116	5.788922
D32441J04Ri	2441J04Rik-(	8985.1	0.000005	0.000	54.918	54.918	77.634	1.414	5.779209
Ndufa10	Ndufa10-006	1236.2	0.005272	0.017	54.801	54.801	10.951	0.200	5.776138
Xpo4	Xpo4-201	1104.2	0.000678	0.003	53.865	53.865	9.010	0.167	5.751283
Nptn	Nptn-007	547.6	0.034403	0.073	53.163	53.163	3.739	0.070	5.732346
Hsp90aa1	Hsp90aa1-004	2994.5	0.005128	0.016	52.963	52.963	21.944	0.414	5.726917
Cdv3	Cdv3-002	2739.3	0.000000	0.000	52.916	52.916	18.577	0.351	5.72562
Mtdh	Mtdh-010	991.6	0.002153	0.008	52.601	52.601	5.755	0.109	5.717017
Serpina9	Serpina9-001	1463.0	0.000000	0.000	52.332	52.332	12.578	0.240	5.70962
Pkn1	Pkn1-001	3613.0	0.000002	0.000	51.894	51.894	26.414	0.509	5.697496
Rbm42	Rbm42-001	1571.5	0.000103	0.001	51.653	51.653	15.539	0.301	5.690779
Men1	Men1-003	353.4	0.036550	0.077	51.306	51.306	3.846	0.075	5.681057

Nrf1	Nrf1-002	1140.8	0.000304	0.002	51.289	51.289	11.158	0.218	5.680574
Ccnj	Ccnj-002	321.2	0.005867	0.018	51.183	51.183	2.606	0.051	5.677597
Paf1	Paf1-010	2126.6	0.003737	0.013	51.103	51.103	15.562	0.305	5.675341
Slco2b1	Slco2b1-002	794.2	0.005520	0.017	51.060	51.060	9.896	0.194	5.674114
Atp6v0c	Atp6v0c-003	557.1	0.004128	0.014	50.787	50.787	5.985	0.118	5.666379
Ddx26b	Ddx26b-001	825.7	0.000712	0.004	50.617	50.617	6.785	0.134	5.661552
Tardbp	Tardbp-011	1581.6	0.006268	0.019	50.555	50.555	15.724	0.311	5.659777
Gm24447	Gm24447-201	103196.0	0.000000	0.000	50.177	50.177	860.047	17.140	5.648958
Emx2os	Emx2os-001	1224.0	0.000023	0.000	49.706	49.706	11.872	0.239	5.635341
Sqstm1	Sqstm1-007	5506.3	0.028948	0.063	49.608	49.608	43.493	0.877	5.632495
Fbxw11	Fbxw11-001	4093.5	0.002552	0.009	49.423	49.423	31.643	0.640	5.6271
Zic1	Zic1-001	970.0	0.000002	0.000	49.180	49.180	7.840	0.159	5.620003
Ncln	Ncln-004	702.7	0.008679	0.025	49.139	49.139	6.088	0.124	5.618788
Zfp467	Zfp467-001	1225.0	0.001066	0.005	49.055	49.055	6.393	0.130	5.61633
Nfib	Nfib-007	18298.3	0.000026	0.000	48.547	48.547	121.162	2.496	5.601309
Gm22009	Gm22009-201	12797.0	0.000000	0.000	48.352	48.352	109.601	2.267	5.59549
Arpp19	Arpp19-009	3024.7	0.047769	0.095	48.311	48.311	27.102	0.561	5.594294
Fbxo6	Fbxo6-001	514.2	0.000064	0.001	47.255	47.255	3.789	0.080	5.562382
Kat7	Kat7-007	1042.6	0.000001	0.000	47.107	47.107	7.594	0.161	5.557862
Nlgn2	Nlgn2-001	2452.3	0.000048	0.000	47.070	47.070	21.477	0.456	5.556724
Rpl13a	Rpl13a-006	9221.1	0.008130	0.023	46.669	46.669	89.619	1.920	5.544393
Rab6a	Rab6a-002	1055.5	0.000019	0.000	46.339	46.339	10.519	0.227	5.534157
Tmem38b	Tmem38b-001	92444.7	0.000000	0.000	46.203	46.203	820.033	17.748	5.529927
Map2k7	Map2k7-010	959.4	0.000008	0.000	46.117	46.117	7.335	0.159	5.527227
Man1a2	Man1a2-006	1674.1	0.026909	0.060	45.839	45.839	13.952	0.304	5.518515
Ttn	Ttn-202	921.3	0.027083	0.060	45.550	45.550	12.430	0.273	5.509386
Atp2a1	Atp2a1-001	575.5	0.000946	0.004	45.450	45.450	6.998	0.154	5.506212
Zeb2	Zeb2-007	4185.7	0.001015	0.005	45.373	45.373	31.870	0.702	5.503772
Brd2	Brd2-004	846.3	0.003581	0.012	45.083	45.083	6.728	0.149	5.494525
Nos1ap	Nos1ap-004	3156.2	0.022231	0.052	44.758	44.758	18.044	0.403	5.484084
Stau1	Stau1-002	1831.0	0.000204	0.001	44.649	44.649	19.805	0.444	5.480552
Kri1	Kri1-003	1589.8	0.013463	0.035	44.401	44.401	15.011	0.338	5.472535
Calm2	Calm2-002	8341.4	0.000000	0.000	44.398	44.398	64.988	1.464	5.472438
Pdzd2	Pdzd2-009	701.2	0.003986	0.013	44.012	44.012	5.717	0.130	5.459818
Dvl1	Dvl1-001	1632.6	0.000747	0.004	43.768	43.768	13.189	0.301	5.451794
Cp	Cp-007	13105.7	0.000209	0.001	43.565	43.565	146.247	3.357	5.445087
Saa4	Saa4-201	1205.0	0.000030	0.000	43.388	43.388	8.010	0.185	5.439215
Gm14914	Gm14914-001	1906.0	0.000002	0.000	43.103	43.103	17.172	0.398	5.429733
Lmo4	Lmo4-001	825.0	0.000152	0.001	43.010	43.010	8.851	0.206	5.426601
Clu	Clu-002	5065.9	0.000000	0.000	42.562	42.562	41.134	0.966	5.411496
Eif5	Eif5-201	4751.2	0.000004	0.000	42.412	42.412	41.950	0.989	5.406396
Med11	Med11-003	442.2	0.005123	0.016	42.377	42.377	4.533	0.107	5.405222
Rabep1	Rabep1-008	1267.8	0.001192	0.005	42.243	42.243	8.957	0.212	5.400637
Cebpg	Cebpg-002	1276.4	0.000007	0.000	41.930	41.930	10.061	0.240	5.389895
Podn	Podn-001	133.7	0.024194	0.055	41.755	41.755	1.557	0.037	5.383868
Lonrf2	Lonrf2-001	1069.0	0.000003	0.000	41.664	41.664	8.790	0.211	5.380737
Slc39a3	Slc39a3-001	868.4	0.000004	0.000	41.472	41.472	7.751	0.187	5.374055
Lrif1	Lrif1-006	611.2	0.001601	0.007	41.468	41.468	5.955	0.144	5.373931
Shprh	Shprh-002	2449.9	0.028232	0.062	41.125	41.125	18.967	0.461	5.361935
Fam57a	Fam57a-005	357.9	0.028982	0.063	40.960	40.960	2.222	0.054	5.356137
Slc38a10	Slc38a10-013	1182.2	0.000018	0.000	40.927	40.927	10.300	0.252	5.35499
Spag1	Spag1-202	584.9	0.003754	0.013	40.847	40.847	5.264	0.129	5.352149
Aktip	Aktip-003	996.6	0.003382	0.012	40.814	40.814	6.213	0.152	5.351
Rps3	Rps3-003	9975.6	0.000000	0.000	40.653	40.653	108.272	2.663	5.3453
Mapk9	Mapk9-005	861.7	0.000044	0.000	39.572	39.572	5.022	0.127	5.306424
Pfkfb3	Pfkfb3-004	1082.1	0.008323	0.024	39.481	39.481	6.712	0.170	5.303091
Ctnnd1	Ctnnd1-028	1871.4	0.015403	0.039	39.050	39.050	15.532	0.398	5.287242
Polr2a	Polr2a-201	14787.3	0.000043	0.000	39.002	39.002	124.764	3.199	5.285473
P4ha2	P4ha2-001	2021.4	0.000037	0.000	38.781	38.781	9.226	0.238	5.277276
Svil	Svil-005	2202.0	0.033008	0.071	38.679	38.679	12.508	0.323	5.273477
Bag6	Bag6-002	798.2	0.001752	0.007	38.664	38.664	8.587	0.222	5.272901
Dstyk	Dstyk-004	610.7	0.020997	0.049	38.571	38.571	5.307	0.138	5.269463
Arl1	Arl1-003	1109.2	0.000014	0.000	38.211	38.211	8.280	0.217	5.255915
Mif4gd	Mif4gd-001	1098.2	0.006487	0.020	37.751	37.751	9.556	0.253	5.238453
Stag2	Stag2-201	358.3	0.009705	0.027	37.703	37.703	3.790	0.101	5.236621
Mark2	Mark2-005	336.2	0.006419	0.019	37.610	37.610	3.026	0.080	5.233028
Alkbh6	Alkbh6-001	401.6	0.012591	0.033	37.468	37.468	3.491	0.093	5.227597
Gm11027	Gm11027-201	1523.0	0.000000	0.000	37.324	37.324	12.920	0.346	5.222049
Gm26315	Gm26315-201	58237.0	0.000000	0.000	37.127	37.127	513.122	13.821	5.214401
Trim2	Trim2-005	3048.6	0.036965	0.077	37.001	37.001	29.051	0.785	5.209498
Lhx5	Lhx5-001	575.0	0.000055	0.001	36.564	36.564	4.892	0.134	5.192371
Zfp384	Zfp384-003	495.5	0.000083	0.001	36.543	36.543	4.435	0.121	5.191535
Tmem214	Tmem214-00	577.2	0.034850	0.074	36.512	36.512	3.911	0.107	5.190292
Immt	Immt-001	1288.5	0.001598	0.007	36.401	36.401	11.535	0.317	5.185905
10403A07R	10403A07Rik-	835.1	0.000732	0.004	36.094	36.094	6.434	0.178	5.173675
Atn1	Atn1-003	10448.9	0.000780	0.004	36.009	36.009	73.741	2.048	5.170274
St6galnac6	St6galnac6-20	481.1	0.018294	0.044	35.847	35.847	4.452	0.124	5.163772
Pkn3	Pkn3-004	2134.3	0.000001	0.000	35.828	35.828	16.080	0.449	5.163025
Src	Src-001	462.0	0.004980	0.016	35.793	35.793	4.448	0.124	5.161604
Cgnl1	Cgnl1-001	99475.3	0.000000	0.000	35.744	35.744	436.808	12.220	5.159633
Lmo2	Lmo2-001	11712.6	0.000001	0.000	35.627	35.627	122.609	3.441	5.15491
G3bp2	G3bp2-002	1130.7	0.007563	0.022	35.433	35.433	7.379	0.208	5.14703
Rnf165	Rnf165-003	1974.9	0.000032	0.000	35.306	35.306	16.168	0.458	5.141825
Abcf1	Abcf1-003	551.6	0.000015	0.000	35.241	35.241	4.001	0.114	5.139201
Gtpbp3	Gtpbp3-003	436.4	0.003267	0.011	34.840	34.840	4.233	0.122	5.122659
Qdpr	Qdpr-001	1128.6	0.005642	0.018	34.755	34.755	9.657	0.278	5.119141
Srsf2	Srsf2-002	470.7	0.000180	0.001	34.748	34.748	4.982	0.143	5.118839
Gapvd1	Gapvd1-201	3750.4	0.000004	0.000	34.651	34.651	32.272	0.931	5.114827
Myh9	Myh9-007	3687.2	0.000084	0.001	34.605	34.605	34.039	0.984	5.112928
Fam89b	Fam89b-003	737.0	0.000020	0.000	34.582	34.582	3.406	0.098	5.111951
Rpl5	Rpl5-002	2415.6	0.006146	0.019	34.503	34.503	24.987	0.724	5.108655
Gm26138	Gm26138-201	1461.0	0.000005	0.000	34.081	34.081	11.888	0.349	5.090895
Thnsl2	Thnsl2-001	147.7	0.018476	0.045	33.782	33.782	1.076	0.032	5.078167
Dctn1	Dctn1-003	347.6	0.004730	0.015	33.705	33.705	3.236	0.096	5.074908
Nmt2	Nmt2-003	1669.9	0.026377	0.059	33.652	33.652	16.103	0.479	5.072608
Gcm1	Gcm1-201	1598.0	0.000078	0.001	33.499	33.499	16.013	0.478	5.066057
Strn	Strn-002	995.3	0.005746	0.018	33.470	33.470	7.477	0.223	5.064789
Zfp532	Zfp532-007	764.6	0.042022	0.085	33.071	33.071	5.956	0.180	5.0475
Iqsec2	Iqsec2-002	3369.5	0.019379	0.046	32.965	32.965	24.849	0.754	5.042861
Usp22	Usp22-004	1614.7	0.001599	0.007	32.786	32.786	12.171	0.371	5.034999
Rxra	Rxra-003	548.9	0.006572	0.020	32.723	32.723	3.281	0.100	5.032252
Auts2	Auts2-013	2528.6	0.004785	0.015	32.574	32.574	27.489	0.844	5.025659
Piezo1	Piezo1-001	5420.2	0.000078	0.001	32.507	32.507	35.194	1.083	5.022656
Mtcl1	Mtcl1-002	191.1	0.044169	0.089	32.220	32.220	1.428	0.044	5.009879
Mtmr3	Mtmr3-003	663.5	0.004943	0.016	32.188	32.188	5.858	0.182	5.008459
Cav1	Cav1-010	6797.0	0.000009	0.000	32.170	32.170	49.550	1.540	5.007634
Gtf2h1	Gtf2h1-002	957.8	0.013809	0.036	32.094	32.094	5.692	0.177	5.00425

Shisa9	Shisa9-002	665.7	0.032398	0.069	32.086	32.086	6.357	0.198	5.003872
Osbpl9	Osbpl9-013	2243.2	0.006667	0.020	31.839	31.839	13.346	0.419	4.992716
Gm16551	Gm16551-001	1819.0	0.000005	0.000	31.773	31.773	16.026	0.504	4.989707
Asph	Asph-005	2464.2	0.000001	0.000	31.695	31.695	19.374	0.611	4.986206
Amotl2	Amotl2-002	9146.6	0.000223	0.001	31.617	31.617	86.993	2.752	4.982606
Frmd5	Frmd5-004	628.3	0.009182	0.026	31.470	31.470	6.119	0.194	4.975926
Rapgef1	Rapgef1-007	2340.9	0.000167	0.001	31.466	31.466	15.193	0.483	4.975731
Itsn1	Itsn1-004	2933.8	0.016780	0.041	31.436	31.436	29.807	0.948	4.974336
Fgfr1op2	Fgfr1op2-006	1067.6	0.006723	0.020	31.368	31.368	7.988	0.255	4.971242
Yam1	Yam1-001	1006590.5	0.000000	0.000	31.352	31.352	9508.815	303.295	4.970471
Gm37357	Gm37357-001	5884.0	0.000004	0.000	31.319	31.319	49.149	1.569	4.968972
Ipo9	Ipo9-002	1264.9	0.014007	0.036	31.181	31.181	11.488	0.368	4.962594
Srsf11	Srsf11-010	5311.5	0.000048	0.000	31.153	31.153	46.195	1.483	4.961309
Kdm1a	Kdm1a-007	510.3	0.009572	0.027	31.113	31.113	6.570	0.211	4.959467
Tcf3	Tcf3-011	718.0	0.000450	0.002	31.058	31.058	4.488	0.145	4.956913
Parp10	Parp10-004	243.0	0.020591	0.049	30.936	30.936	2.423	0.078	4.951226
Bmx	Bmx-002	7691.5	0.000000	0.000	30.873	30.873	52.693	1.707	4.948252
Lats2	Lats2-002	7674.2	0.000002	0.000	30.823	30.823	61.801	2.005	4.945959
Arhgap6	Arhgap6-003	796.8	0.000310	0.002	30.816	30.816	7.958	0.258	4.945617
Dab2ip	Dab2ip-010	3252.2	0.003447	0.012	30.661	30.661	26.129	0.852	4.938311
Brox	Brox-003	716.4	0.008048	0.023	30.597	30.597	6.503	0.213	4.935306
Tnk2	Tnk2-003	1342.0	0.000045	0.000	30.536	30.536	10.536	0.345	4.932456
Dhx30	Dhx30-001	321.0	0.008011	0.023	30.474	30.474	3.082	0.101	4.929504
Ocln	Ocln-002	960.1	0.015212	0.038	30.429	30.429	9.352	0.307	4.927365
Alx4	Alx4-001	1460.2	0.000000	0.000	30.414	30.414	12.142	0.399	4.926682
Dnase2a	Dnase2a-001	1008.0	0.002900	0.010	30.196	30.196	7.773	0.257	4.916282
Akap1	Akap1-006	2299.9	0.003969	0.013	30.137	30.137	18.334	0.608	4.913487
Thbs3	Thbs3-003	244.4	0.004302	0.014	30.050	30.050	2.093	0.070	4.909297
Psap	Psap-001	19043.4	0.000000	0.000	30.019	30.019	140.952	4.695	4.907797
Cadps2	Cadps2-007	823.7	0.018298	0.044	29.918	29.918	6.146	0.205	4.902924
Fam133b	Fam133b-001	977.2	0.000232	0.001	29.877	29.877	7.938	0.266	4.900971
Rnf6	Rnf6-003	2189.5	0.004357	0.014	29.768	29.768	13.292	0.447	4.895672
Flywch1	Flywch1-202	2939.3	0.015788	0.039	29.742	29.742	22.245	0.748	4.894418
Cbx4	Cbx4-002	1207.2	0.008190	0.024	29.720	29.720	9.223	0.310	4.893376
Adnp	Adnp-001	1384.6	0.004175	0.014	29.559	29.559	8.978	0.304	4.885546
Helz2	Helz2-003	4811.9	0.000215	0.001	29.539	29.539	25.548	0.865	4.884526
Sptbn1	Sptbn1-007	106435.1	0.000000	0.000	29.527	29.527	620.808	21.025	4.883962
Slitrk4	Slitrk4-001	584.0	0.000017	0.000	29.512	29.512	4.704	0.159	4.883206
Glce	Glce-003	1804.1	0.003137	0.011	29.510	29.510	13.723	0.465	4.883127
Pias3	Pias3-004	691.3	0.011066	0.030	29.459	29.459	3.661	0.124	4.880623
Sel1l	Sel1l-006	4456.8	0.000220	0.001	29.374	29.374	37.620	1.281	4.876445
Sdf4	Sdf4-004	1897.9	0.000005	0.000	29.333	29.333	15.705	0.535	4.874464
Lsm14b	Lsm14b-003	326.4	0.016410	0.041	29.326	29.326	3.640	0.124	4.874112
Ralgapa2	Ralgapa2-008	3058.8	0.006816	0.020	29.303	29.303	24.772	0.845	4.872986
Fam214b	Fam214b-005	1799.4	0.000000	0.000	29.220	29.220	11.570	0.396	4.868883
Ylpm1	Ylpm1-004	72.9	0.021897	0.051	29.182	29.182	0.484	0.017	4.867018
Ap4e1	Ap4e1-007	347.3	0.009650	0.027	29.153	29.153	3.796	0.130	4.865584
Gak	Gak-002	415.2	0.001123	0.005	29.132	29.132	2.656	0.091	4.864544
Kcnc1	Kcnc1-001	331.0	0.000091	0.001	29.080	29.080	2.313	0.080	4.861932
Camsap1	Camsap1-202	1583.0	0.006963	0.021	29.070	29.070	9.151	0.315	4.861475
Mpdu1	Mpdu1-005	138.0	0.017029	0.042	29.064	29.064	0.644	0.022	4.86114
Crlf2	Crlf2-001	1714.1	0.021702	0.051	28.703	28.703	14.637	0.510	4.843146
Sirt2	Sirt2-001	496.7	0.000254	0.002	28.679	28.679	4.776	0.167	4.841929
Gm10125	Gm10125-003	1175.4	0.043577	0.088	28.555	28.555	9.121	0.319	4.83567
Mapkapk5	Mapkapk5-006	648.0	0.034245	0.073	28.508	28.508	6.392	0.224	4.833284
Crebzf	Crebzf-001	1900.7	0.005244	0.017	28.506	28.506	20.127	0.706	4.833209
Csrp2bp	Csrp2bp-001	1387.5	0.001596	0.007	28.362	28.362	14.348	0.506	4.825896
Meis3	Meis3-002	358.9	0.025717	0.058	28.345	28.345	2.896	0.102	4.825036
Rpl34	Rpl34-004	7073.8	0.029227	0.064	28.279	28.279	68.950	2.438	4.821664
Zeb1	Zeb1-003	6516.5	0.000003	0.000	28.199	28.199	52.778	1.872	4.817572
Nptn	Nptn-201	1436.9	0.003861	0.013	28.127	28.127	11.169	0.397	4.813896
Nos3	Nos3-002	18452.0	0.000000	0.000	28.059	28.059	72.411	2.581	4.810413
Pear1	Pear1-004	3076.7	0.038820	0.080	28.052	28.052	30.374	1.083	4.810057
Gm21738	Gm21738-201	1209.0	0.000022	0.000	28.002	28.002	14.161	0.506	4.807441
Mcmbp	Mcmbp-002	2822.2	0.000001	0.000	27.981	27.981	21.487	0.768	4.806357
Fbxl19	Fbxl19-003	882.2	0.010277	0.028	27.965	27.965	5.725	0.205	4.805564
Pxn	Pxn-002	5222.1	0.000000	0.000	27.942	27.942	43.547	1.558	4.80437
Gak	Gak-009	1382.2	0.035636	0.075	27.860	27.860	10.850	0.389	4.800146
Mir6236	Mir6236-201	101164.0	0.000010	0.000	27.747	27.747	886.874	31.963	4.794268
Dusp3	Dusp3-005	693.1	0.005814	0.018	27.743	27.743	3.865	0.139	4.79403
Fnip2	Fnip2-006	3440.7	0.000033	0.000	27.708	27.708	28.590	1.032	4.792214
Fnbp1	Fnbp1-007	864.0	0.003426	0.012	27.699	27.699	5.209	0.188	4.791786
Cttnbp2nl	Cttnbp2nl-004	1693.3	0.000006	0.000	27.658	27.658	15.090	0.546	4.789646
Tanc1	Tanc1-001	13005.9	0.000000	0.000	27.613	27.613	104.981	3.802	4.787263
Cnot6l	Cnot6l-201	944.2	0.010234	0.028	27.588	27.588	9.062	0.328	4.785963
Gm38269	Gm38269-001	284.0	0.000070	0.001	27.526	27.526	2.190	0.080	4.782724
Gm17767	Gm17767-002	393.0	0.000040	0.000	27.404	27.404	3.646	0.133	4.776311
810428I15R	0428I15Rik-0	462.9	0.027278	0.061	27.306	27.306	3.097	0.113	4.771166
Rit1	Rit1-001	1157.0	0.016514	0.041	27.180	27.180	8.377	0.308	4.764491
Gm24507	Gm24507-201	793.0	0.000004	0.000	27.053	27.053	7.184	0.266	4.757735
Arhgef10l	Arhgef10l-002	1594.4	0.014657	0.037	27.044	27.044	17.088	0.632	4.757259
Ywhab	Ywhab-002	2370.5	0.000002	0.000	27.015	27.015	14.124	0.523	4.755715
Sh3tc1	Sh3tc1-001	589.5	0.049534	0.097	26.861	26.861	5.864	0.218	4.74742
Ddr1	Ddr1-004	4442.1	0.000000	0.000	26.684	26.684	36.452	1.366	4.737909
Ssna1	Ssna1-003	487.1	0.014686	0.037	26.290	26.290	3.412	0.130	4.716437
Parp10	Parp10-001	934.7	0.004411	0.015	26.280	26.280	6.353	0.242	4.715913
Actn2	Actn2-002	395.7	0.000035	0.000	26.223	26.223	3.469	0.132	4.712735
Celf1	Celf1-202	2510.1	0.000003	0.000	26.089	26.089	18.376	0.704	4.705378
Ramp2	Ramp2-008	4433.0	0.000002	0.000	26.036	26.036	25.876	0.994	4.70243
Clta	Clta-003	92.9	0.003623	0.012	25.950	25.950	1.043	0.040	4.697667
Lhfp	Lhfp-001	3638.7	0.001104	0.005	25.924	25.924	25.768	0.994	4.696201
Ttc33	Ttc33-002	408.5	0.002622	0.010	25.753	25.753	3.211	0.125	4.68668
Ncoa3	Ncoa3-001	15067.9	0.000001	0.000	25.723	25.723	77.436	3.010	4.685009
Cuedc1	Cuedc1-006	1101.8	0.000034	0.000	25.551	25.551	9.382	0.367	4.675325
Stk11	Stk11-004	703.9	0.005439	0.017	25.545	25.545	7.632	0.299	4.674989
Hist1h4a	Hist1h4a-001	322.0	0.000122	0.001	25.513	25.513	3.354	0.131	4.673145
9-Sep	Sept9-002	7972.7	0.000017	0.000	25.460	25.460	30.656	1.204	4.670167
Igf2	Igf2-002	9595.4	0.003730	0.013	25.457	25.457	34.937	1.372	4.670012
Snora23	Snora23-201	15116.0	0.000000	0.000	25.373	25.373	133.383	5.257	4.665218
Kalrn	Kalrn-012	8331.0	0.000010	0.000	25.345	25.345	66.412	2.620	4.663624
Gne	Gne-002	277.4	0.005511	0.017	25.318	25.318	2.868	0.113	4.662113
Ptgis	Ptgis-002	6139.9	0.000000	0.000	25.312	25.312	34.962	1.381	4.661733
Htra1	Htra1-003	3398.2	0.000006	0.000	25.239	25.239	24.890	0.986	4.657611
Celsr2	Celsr2-002	332.6	0.006777	0.020	25.227	25.227	2.893	0.115	4.656902
Sgms1	Sgms1-004	6244.5	0.000000	0.000	25.192	25.192	51.413	2.041	4.6549
33421A08R	3421A08Rik-	530.0	0.000061	0.001	25.140	25.140	3.326	0.132	4.651939
Ankrd33b	Ankrd33b-004	1026.9	0.038499	0.080	25.042	25.042	5.857	0.234	4.64627

Rab11a	Rab11a-005	2464.5	0.000001	0.000	24.934	24.934	17.479	0.701	4.640065
Agrn	Agrn-004	305.1	0.006974	0.021	24.902	24.902	1.362	0.055	4.638199
Csgalnact2	sgalnact2-00	2728.9	0.000936	0.004	24.748	24.748	18.215	0.736	4.629229
Gm15584	Gm15584-001	668.0	0.000006	0.000	24.735	24.735	5.237	0.212	4.62851
Matr3	Matr3-006	498.0	0.001170	0.005	24.721	24.721	5.549	0.224	4.627661
Brd8	Brd8-003	2148.6	0.005382	0.017	24.703	24.703	18.214	0.737	4.626594
Hes6	Hes6-005	746.4	0.000771	0.004	24.670	24.670	6.020	0.244	4.624706
Gm23734	Gm23734-201	1495.0	0.000040	0.000	24.636	24.636	12.301	0.499	4.622696
Sp100	Sp100-001	1580.1	0.000002	0.000	24.577	24.577	13.770	0.560	4.619246
Coro1c	Coro1c-008	2240.8	0.007001	0.021	24.487	24.487	18.651	0.762	4.613946
Sox12	Sox12-002	453.4	0.014140	0.036	24.419	24.419	5.136	0.210	4.609953
Dmd	Dmd-007	616.0	0.005372	0.017	24.275	24.275	2.879	0.119	4.601387
Lzts3	Lzts3-201	910.0	0.004806	0.016	24.208	24.208	5.109	0.211	4.597405
Gm25395	Gm25395-201	3537.0	0.000082	0.001	24.155	24.155	29.658	1.228	4.594269
C77080	C77080-005	3276.2	0.000000	0.000	24.151	24.151	21.690	0.898	4.594001
Ccdc85b	Ccdc85b-201	1086.0	0.000006	0.000	24.103	24.103	8.231	0.341	4.591111
Khk	Khk-005	414.7	0.013338	0.035	23.917	23.917	3.421	0.143	4.579991
Agps	Agps-007	1043.5	0.049222	0.097	23.887	23.887	8.686	0.364	4.57813
Rps8	Rps8-007	197.0	0.003034	0.011	23.885	23.885	1.736	0.073	4.578019
Gm22620	Gm22620-201	1967.0	0.000007	0.000	23.734	23.734	17.505	0.738	4.568871
Gm10719	Gm10719-201	352.6	0.000105	0.001	23.715	23.715	3.788	0.160	4.567724
Fgf13	Fgf13-006	166.9	0.007660	0.022	23.649	23.649	2.068	0.087	4.563723
Hip1r	Hip1r-005	589.6	0.001416	0.006	23.633	23.633	4.709	0.199	4.56271
Irak1	Irak1-007	901.7	0.001727	0.007	23.519	23.519	7.699	0.327	4.55578
Gm12498	Gm12498-001	496.7	0.000100	0.001	23.470	23.470	3.635	0.155	4.552773
Mtcl1	Mtcl1-011	446.2	0.036374	0.076	23.463	23.463	6.414	0.273	4.552303
Zfp40	Zfp40-002	246.3	0.028732	0.063	23.427	23.427	2.615	0.112	4.550125
Ddx5	Ddx5-004	882.7	0.000025	0.000	23.396	23.396	8.417	0.360	4.548168
Hspa5	Hspa5-007	1498.7	0.010454	0.029	23.304	23.304	11.428	0.490	4.542502
Sncaip	Sncaip-005	6228.6	0.001202	0.005	22.952	22.952	49.361	2.151	4.520543
Pak1ip1	Pak1ip1-001	1825.2	0.004368	0.014	22.906	22.906	13.143	0.574	4.517632
Naa10	Naa10-001	340.4	0.008069	0.023	22.901	22.901	3.305	0.144	4.51734
Klhl18	Klhl18-001	1229.4	0.011094	0.030	22.829	22.829	9.093	0.398	4.512772
Luc7l3	Luc7l3-009	8493.1	0.000000	0.000	22.823	22.823	70.032	3.069	4.512395
Fhl1	Fhl1-010	1357.0	0.000178	0.001	22.762	22.762	11.148	0.490	4.508567
Man2c1	Man2c1-001	335.5	0.005654	0.018	22.701	22.701	2.873	0.127	4.504686
Znrf1	Znrf1-003	670.8	0.000259	0.002	22.607	22.607	7.101	0.314	4.498666
Vim	Vim-002	32050.2	0.000000	0.000	22.497	22.497	223.490	9.934	4.491632
BC021785	C021785-00	1168.0	0.000022	0.000	22.418	22.418	9.567	0.427	4.486568
Dmtf1	Dmtf1-004	388.1	0.014726	0.037	22.319	22.319	4.343	0.195	4.480177
Nfrkb	Nfrkb-001	1234.2	0.020293	0.048	22.257	22.257	13.549	0.609	4.476178
Jdp2	Jdp2-201	1345.6	0.000298	0.002	22.215	22.215	8.512	0.383	4.473474
Hdac7	Hdac7-009	4372.8	0.011752	0.031	22.173	22.173	32.775	1.478	4.470719
Med16	Med16-003	464.6	0.001686	0.007	22.016	22.016	2.876	0.131	4.460493
Hnrnpk	Hnrnpk-008	2955.0	0.001366	0.006	21.917	21.917	25.829	1.178	4.453999
Tbc1d4	Tbc1d4-006	303.1	0.002516	0.009	21.912	21.912	2.403	0.110	4.453654
Eif4a2	Eif4a2-022	2546.1	0.049292	0.097	21.885	21.885	22.473	1.027	4.451903
Hmgb1	Hmgb1-002	3971.2	0.000013	0.000	21.881	21.881	38.018	1.738	4.451593
Gm4070	Gm4070-004	6187.0	0.000000	0.000	21.824	21.824	46.570	2.134	4.447847
RP24-79A6.	P24-79A6.8-0	1073.0	0.000026	0.000	21.808	21.808	9.799	0.449	4.446782
Kank1	Kank1-002	12900.7	0.000012	0.000	21.757	21.757	134.087	6.163	4.443411
Mbnl1	Mbnl1-010	671.3	0.019565	0.047	21.749	21.749	5.619	0.258	4.442909
Inpp4a	Inpp4a-006	334.8	0.021159	0.050	21.730	21.730	4.760	0.219	4.441619
AW554918	W554918-00	621.2	0.007095	0.021	21.610	21.610	3.671	0.170	4.433598
Tmod2	Tmod2-203	1106.0	0.010642	0.029	21.543	21.543	10.039	0.466	4.429172
Ctsd	Ctsd-004	2934.0	0.003551	0.012	21.519	21.519	18.276	0.849	4.427538
Synj2	Synj2-201	1245.7	0.002583	0.010	21.477	21.477	11.606	0.540	4.424738
Jmjd6	Jmjd6-002	449.6	0.015457	0.039	21.296	21.296	5.468	0.257	4.412533
Cyth3	Cyth3-004	370.3	0.000242	0.002	21.242	21.242	3.000	0.141	4.408842
Irak1	Irak1-002	1496.3	0.002181	0.008	21.234	21.234	13.286	0.626	4.408288
Clcn3	Clcn3-002	873.7	0.001181	0.005	21.209	21.209	8.444	0.398	4.406604
Cst3	Cst3-003	4775.0	0.000199	0.001	21.188	21.188	28.073	1.325	4.405186
Nadk2	Nadk2-003	716.9	0.017672	0.043	21.168	21.168	6.600	0.312	4.403787
Gm26917	Gm26917-001	927232.1	0.000000	0.000	21.144	21.144	7791.380	368.487	4.402192
Emc1	Emc1-002	3785.0	0.000009	0.000	21.014	21.014	27.311	1.300	4.393309
Fkbp8	Fkbp8-003	2093.1	0.000188	0.001	20.963	20.963	19.332	0.922	4.389806
Scaf1	Scaf1-003	5973.4	0.000000	0.000	20.952	20.952	58.510	2.793	4.389047
Flot2	Flot2-001	5002.0	0.000001	0.000	20.837	20.837	36.155	1.735	4.381067
Dhx30	Dhx30-003	420.9	0.005368	0.017	20.736	20.736	4.185	0.202	4.374033
Ubl3	Ubl3-201	3045.5	0.000015	0.000	20.715	20.715	22.719	1.097	4.372615
Mrpl38	Mrpl38-001	801.3	0.005769	0.018	20.705	20.705	8.492	0.410	4.371942
Kctd17	Kctd17-007	784.4	0.032015	0.069	20.676	20.676	5.441	0.263	4.369851
Dhx30	Dhx30-004	1230.5	0.025851	0.058	20.662	20.662	10.063	0.487	4.368882
Gm10172	Gm10172-201	642.0	0.000026	0.000	20.638	20.638	5.432	0.263	4.367234
Nr2e1	Nr2e1-001	434.0	0.000657	0.003	20.547	20.547	4.333	0.211	4.360845
Gm6304	Gm6304-201	228.0	0.000507	0.003	20.533	20.533	1.633	0.080	4.359858
Canx	Canx-003	11287.9	0.000168	0.001	20.515	20.515	81.687	3.982	4.358616
Fam129b	Fam129b-006	723.1	0.023687	0.054	20.398	20.398	5.978	0.293	4.350379
Zrsr2	Zrsr2-004	421.1	0.003593	0.012	20.387	20.387	4.107	0.201	4.34956
Fgfr3	Fgfr3-001	783.4	0.000031	0.000	20.358	20.358	2.191	0.108	4.347497
Chtf8	Chtf8-005	1231.8	0.000008	0.000	20.245	20.245	7.580	0.374	4.339497
Trim47	Trim47-002	2558.1	0.000055	0.001	20.243	20.243	24.445	1.208	4.339347
Scrib	Scrib-001	399.2	0.005406	0.017	20.209	20.209	3.723	0.184	4.33693
Gabbr1	Gabbr1-002	1881.9	0.000099	0.001	20.195	20.195	13.367	0.662	4.335941
Tet3	Tet3-003	4812.2	0.000001	0.000	20.185	20.185	34.803	1.724	4.335184
Clcn3	Clcn3-004	1449.6	0.001818	0.007	20.142	20.142	11.079	0.550	4.332102
Rapgef3	Rapgef3-007	2189.5	0.024917	0.056	20.108	20.108	6.105	0.304	4.32971
Schip1	Schip1-009	1413.1	0.041139	0.084	20.081	20.081	14.146	0.704	4.327738
Plekhm2	Plekhm2-001	608.4	0.017469	0.043	20.076	20.076	4.949	0.247	4.327436
Sema3f	Sema3f-010	9929.7	0.000077	0.001	19.985	19.985	49.711	2.487	4.320881
Pde5a	Pde5a-002	463.2	0.000408	0.002	19.894	19.894	4.241	0.213	4.314298
Nrgn	Nrgn-001	1157.2	0.000053	0.001	19.877	19.877	9.518	0.479	4.313058
Arpc2	Arpc2-002	15569.5	0.000009	0.000	19.846	19.846	118.251	5.959	4.310746
Ddrgk1	Ddrgk1-003	3262.1	0.000008	0.000	19.791	19.791	22.258	1.125	4.306739
Ythdf2	Ythdf2-002	2795.8	0.049019	0.097	19.743	19.743	25.895	1.312	4.303296
Nfe2l1	Nfe2l1-003	1770.2	0.008770	0.025	19.732	19.732	12.551	0.636	4.302501
Ncor2	Ncor2-008	2415.7	0.000001	0.000	19.683	19.683	21.453	1.090	4.298893
Trdc	Trdc-001	1105.0	0.000008	0.000	19.628	19.628	8.803	0.448	4.294877
Man1c1	Man1c1-001	3806.6	0.000000	0.000	19.548	19.548	26.721	1.367	4.288956
Fmo2	Fmo2-002	1193.0	0.012636	0.033	19.489	19.489	9.180	0.471	4.284582
Abcc1	Abcc1-004	1013.9	0.021443	0.050	19.451	19.451	9.390	0.483	4.281766
Clip1	Clip1-004	526.7	0.017153	0.042	19.334	19.334	5.127	0.265	4.273093
Stat3	Stat3-004	713.6	0.000146	0.001	19.299	19.299	7.216	0.374	4.270478
Papola	Papola-003	681.8	0.031794	0.068	19.293	19.293	6.346	0.329	4.269995
Nisch	Nisch-002	2574.8	0.003726	0.013	19.253	19.253	17.926	0.931	4.267016
Fam3c	Fam3c-005	6532.3	0.000000	0.000	19.251	19.251	47.672	2.476	4.266876
Phf8	Phf8-202	362.7	0.000093	0.001	19.248	19.248	4.248	0.221	4.266602

Fgf10	Fgf10-001	829.0	0.000681	0.003	19.217	19.217	6.145	0.320	4.264306
Ptms	Ptms-004	1359.1	0.019148	0.046	19.147	19.147	7.704	0.402	4.259026
Itga6	Itga6-002	367.8	0.010137	0.028	19.107	19.107	2.821	0.148	4.256057
Mef2c	Mef2c-006	655.0	0.025612	0.058	19.101	19.101	5.596	0.293	4.255598
Shroom1	Shroom1-001	443.4	0.003619	0.012	19.030	19.030	2.310	0.121	4.250203
D2Wsu81e	D2Wsu81e-00	500.2	0.008078	0.023	18.973	18.973	4.689	0.247	4.245902
Prickle2	Prickle2-002	1689.1	0.000093	0.001	18.938	18.938	8.411	0.444	4.243233
Srcap	Srcap-003	1438.1	0.000070	0.001	18.909	18.909	10.248	0.542	4.241024
Znfx1	Znfx1-003	3688.8	0.000028	0.000	18.901	18.901	24.446	1.293	4.240424
Baiap2	Baiap2-001	620.3	0.001239	0.005	18.832	18.832	3.891	0.207	4.235078
Serinc3	Serinc3-002	11349.7	0.000000	0.000	18.799	18.799	93.470	4.972	4.23258
Tinagl1	Tinagl1-004	2590.1	0.000002	0.000	18.779	18.779	17.865	0.951	4.231019
Itsn1	Itsn1-005	2240.0	0.020305	0.048	18.766	18.766	18.840	1.004	4.230043
Hnrnpd	Hnrnpd-005	1956.8	0.000004	0.000	18.747	18.747	16.852	0.899	4.228617
Ppp1r12c	Ppp1r12c-001	2839.3	0.000001	0.000	18.709	18.709	17.562	0.939	4.225668
Nucks1	Nucks1-001	3367.5	0.000000	0.000	18.610	18.610	24.956	1.341	4.218
Ahnak2	Ahnak2-003	6226.0	0.000001	0.000	18.454	18.454	46.514	2.521	4.205839
P23-319P12	23-319P12.1-	1004.0	0.000020	0.000	18.423	18.423	7.815	0.424	4.203426
Gm21887	Gm21887-20	4555.2	0.000088	0.001	18.282	18.282	38.616	2.112	4.192323
Epb4.1	Epb4.1-002	849.6	0.026314	0.059	18.269	18.269	6.681	0.366	4.191334
Dbp	Dbp-001	3098.8	0.000669	0.003	18.246	18.246	18.730	1.026	4.189539
Camk2d	Camk2d-012	2642.9	0.000001	0.000	18.211	18.211	21.564	1.184	4.186749
Rasa4	Rasa4-004	352.1	0.001275	0.006	18.202	18.202	2.928	0.161	4.186049
Id3	Id3-002	1083.3	0.000157	0.001	18.185	18.185	11.363	0.625	4.184688
Pik3r1	Pik3r1-003	1324.5	0.014138	0.036	18.164	18.164	10.519	0.579	4.183019
Ramp2	Ramp2-004	2630.2	0.000001	0.000	18.145	18.145	10.705	0.590	4.1815
Ctbp2	Ctbp2-001	2963.0	0.001755	0.007	18.127	18.127	18.136	1.000	4.180099
Thsd7a	Thsd7a-001	5640.6	0.000585	0.003	18.123	18.123	58.726	3.240	4.179737
Mapk9	Mapk9-006	567.6	0.002561	0.009	18.108	18.108	4.729	0.261	4.178557
Uqcc1	Uqcc1-012	293.5	0.017020	0.042	18.105	18.105	2.447	0.135	4.178294
Spg11	Spg11-002	364.9	0.014245	0.036	18.082	18.082	2.868	0.159	4.176456
Mid1	Mid1-201	1305.7	0.000021	0.000	18.068	18.068	13.203	0.731	4.175392
Cc2d2a	Cc2d2a-004	498.3	0.001991	0.008	18.029	18.029	2.056	0.114	4.172271
Fam131a	Fam131a-001	350.7	0.000095	0.001	17.950	17.950	2.365	0.132	4.165879
Jade1	Jade1-003	1872.5	0.000341	0.002	17.919	17.919	17.482	0.976	4.163441
Ehmt1	Ehmt1-014	1088.9	0.000710	0.004	17.888	17.888	7.370	0.412	4.160939
Flna	Flna-003	33859.9	0.000000	0.000	17.864	17.864	260.456	14.580	4.158943
Synpo	Synpo-008	3570.6	0.018424	0.045	17.801	17.801	21.704	1.219	4.153927
Als2cl	Als2cl-002	1802.7	0.000937	0.004	17.800	17.800	13.908	0.781	4.153795
Fbrsl1	Fbrsl1-002	1226.1	0.024806	0.056	17.796	17.796	4.114	0.231	4.153469
Plscr3	Plscr3-002	2277.2	0.000167	0.001	17.795	17.795	16.742	0.941	4.153434
Stag2	Stag2-001	9353.7	0.000000	0.000	17.784	17.784	72.744	4.090	4.152541
Klc3	Klc3-002	312.2	0.033180	0.071	17.779	17.779	2.104	0.118	4.152121
Ube2d3	Ube2d3-004	476.5	0.002561	0.009	17.778	17.778	4.629	0.260	4.152014
Kctd10	Kctd10-002	1570.4	0.000016	0.000	17.772	17.772	10.696	0.602	4.151558
Ddx24	Ddx24-201	6314.8	0.000000	0.000	17.772	17.772	56.381	3.172	4.151521
Tcf12	Tcf12-018	2791.0	0.000980	0.004	17.767	17.767	23.534	1.325	4.151091
Uqcrc1	Uqcrc1-004	1715.0	0.002288	0.009	17.765	17.765	13.648	0.768	4.150939
Camk2d	Camk2d-019	1247.4	0.031329	0.068	17.638	17.638	11.428	0.648	4.140619
Cops7b	Cops7b-003	345.8	0.033150	0.071	17.608	17.608	2.616	0.149	4.138138
Gapvd1	Gapvd1-202	1263.2	0.000350	0.002	17.606	17.606	9.909	0.563	4.137999
Gm36987	Gm36987-001	457.0	0.000019	0.000	17.589	17.589	3.260	0.185	4.13664
Stat3	Stat3-010	5246.8	0.000007	0.000	17.468	17.468	37.490	2.146	4.126631
Dnm2	Dnm2-003	2576.0	0.000832	0.004	17.457	17.457	20.271	1.161	4.125736
Ppp1cc	Ppp1cc-002	665.5	0.027771	0.061	17.451	17.451	4.522	0.259	4.125266
Zscan22	Zscan22-004	278.3	0.049835	0.098	17.439	17.439	1.903	0.109	4.12428
Rgs3	Rgs3-016	1417.4	0.018390	0.044	17.437	17.437	10.423	0.598	4.124087
Rab4b	Rab4b-002	468.1	0.002635	0.010	17.432	17.432	3.624	0.208	4.123645
Pear1	Pear1-001	211.4	0.015922	0.040	17.404	17.404	0.747	0.043	4.121357
Ythdc1	Ythdc1-002	1729.6	0.001786	0.007	17.379	17.379	15.164	0.873	4.119297
Zfyve27	Zfyve27-003	674.3	0.012129	0.032	17.335	17.335	5.924	0.342	4.115593
Raly	Raly-004	428.1	0.028651	0.063	17.328	17.328	4.606	0.266	4.115013
Notch4	Notch4-012	11195.1	0.000001	0.000	17.317	17.317	80.325	4.639	4.114116
Pkp4	Pkp4-003	5344.2	0.000000	0.000	17.290	17.290	31.685	1.833	4.111883
Man2c1	Man2c1-019	599.4	0.015721	0.039	17.236	17.236	6.420	0.372	4.107327
Hnrnph1	Hnrnph1-009	7293.5	0.000240	0.002	17.221	17.221	58.864	3.418	4.106101
Metap2	Metap2-009	3212.1	0.000010	0.000	17.152	17.152	25.407	1.481	4.100292
Chst12	Chst12-002	1027.5	0.000003	0.000	17.107	17.107	7.685	0.449	4.096528
Rasd1	Rasd1-001	1839.0	0.010863	0.029	17.051	17.051	9.455	0.555	4.09176
Prkd3	Prkd3-002	2635.1	0.004705	0.015	17.044	17.044	17.768	1.042	4.091194
Taf10	Taf10-002	447.9	0.000338	0.002	17.031	17.031	4.075	0.239	4.090114
Cep131	Cep131-001	997.2	0.000410	0.002	17.025	17.025	8.467	0.497	4.089603
Als2cl	Als2cl-003	1934.3	0.000291	0.002	17.018	17.018	11.692	0.687	4.088985
Mta1	Mta1-003	3369.5	0.000026	0.000	16.958	16.958	24.670	1.455	4.083874
Crocc	Crocc-001	660.7	0.003306	0.012	16.930	16.930	5.049	0.298	4.08153
Mast4	Mast4-002	710.2	0.046961	0.093	16.884	16.884	5.995	0.355	4.077548
Ptgs1	Ptgs1-002	1026.6	0.013339	0.035	16.780	16.780	4.730	0.282	4.068649
Zfp711	Zfp711-201	135.3	0.035354	0.074	16.777	16.777	1.597	0.095	4.068405
Hnrnpd	Hnrnpd-002	2482.8	0.020021	0.048	16.754	16.754	19.971	1.192	4.066429
Gm26870	Gm26870-201	1131.7	0.000613	0.003	16.634	16.634	11.781	0.708	4.056058
Nploc4	Nploc4-003	312.7	0.009941	0.027	16.616	16.616	2.622	0.158	4.054473
Lbp	Lbp-001	2232.5	0.012023	0.032	16.608	16.608	26.839	1.616	4.053796
Bag6	Bag6-003	3329.0	0.000035	0.000	16.541	16.541	23.592	1.426	4.047982
Gm10608	Gm10608-201	1479.0	0.000000	0.000	16.495	16.495	11.803	0.716	4.043981
Exoc6b	Exoc6b-007	1075.8	0.000050	0.000	16.479	16.479	8.095	0.491	4.042528
Rph3al	Rph3al-002	581.6	0.000004	0.000	16.460	16.460	5.191	0.315	4.040897
Qars	Qars-001	3579.3	0.000001	0.000	16.386	16.386	31.435	1.918	4.034355
Sfmbt1	Sfmbt1-002	480.7	0.004474	0.015	16.369	16.369	4.352	0.266	4.032883
Igsf9	Igsf9-002	293.9	0.013998	0.036	16.322	16.322	2.053	0.126	4.028705
Adgra2	Adgra2-002	863.3	0.006212	0.019	16.295	16.295	8.617	0.529	4.026318
Phldb1	Phldb1-004	716.1	0.002818	0.010	16.260	16.260	6.335	0.390	4.023271
10038B21R	0038B21Rik-	638.0	0.000238	0.002	16.140	16.140	5.625	0.348	4.012554
Lrp5	Lrp5-001	23135.5	0.000023	0.000	16.081	16.081	155.373	9.662	4.007315
Prex1	Prex1-003	948.8	0.010503	0.029	16.080	16.080	8.763	0.545	4.007228
Ywhaz	Ywhaz-007	5337.6	0.000000	0.000	16.071	16.071	41.723	2.596	4.006382
Ssfa2	Ssfa2-001	32003.3	0.000000	0.000	16.025	16.025	224.123	13.986	4.002266
Col4a3bp	Col4a3bp-20	3534.7	0.012225	0.032	16.018	16.018	29.254	1.826	4.001584
Cbx1	Cbx1-002	903.4	0.000163	0.001	16.017	16.017	6.958	0.434	4.001528
Afap1l1	Afap1l1-002	3935.9	0.000013	0.000	15.984	15.984	27.731	1.735	3.998513
Slc9a3r2	Slc9a3r2-201	21420.1	0.000000	0.000	15.965	15.965	77.618	4.862	3.996836
Cald1	Cald1-010	4952.3	0.000201	0.001	15.955	15.955	53.579	3.358	3.995909
Cep170b	Cep170b-203	1852.7	0.000135	0.001	15.940	15.940	13.799	0.866	3.994591
Magt1	Magt1-201	3080.1	0.021008	0.049	15.878	15.878	22.718	1.431	3.988938
Cd200	Cd200-010	1701.7	0.009392	0.026	15.826	15.826	16.018	1.012	3.984266
Snora33	Snora33-201	2464.0	0.000001	0.000	15.790	15.790	23.235	1.471	3.980974
Inpp4a	Inpp4a-004	748.1	0.020156	0.048	15.738	15.738	4.000	0.254	3.976183
Limch1	Limch1-016	2765.3	0.000117	0.001	15.718	15.718	28.572	1.818	3.974376

Gtf2ird1	Gtf2ird1-004	220.3	0.006469	0.020	15.700	15.700	1.567	0.100	3.972671
Mta1	Mta1-004	2036.0	0.002539	0.009	15.664	15.664	18.159	1.159	3.969422
Zfp592	Zfp592-004	460.9	0.000950	0.004	15.634	15.634	3.052	0.195	3.96665
Trim25	Trim25-003	1374.5	0.002266	0.009	15.629	15.629	8.486	0.543	3.966187
Pou1f1	Pou1f1-201	2695.6	0.000066	0.001	15.616	15.616	20.986	1.344	3.964966
Ube2d3	Ube2d3-014	507.8	0.005219	0.017	15.597	15.597	4.220	0.271	3.963181
Cald1	Cald1-009	847.3	0.017968	0.044	15.582	15.582	8.911	0.572	3.961766
Hnrnph1	Hnrnph1-011	3602.5	0.000000	0.000	15.533	15.533	27.550	1.774	3.957254
Fam96b	Fam96b-001	871.1	0.014220	0.036	15.528	15.528	6.876	0.443	3.956758
Trmt2a	Trmt2a-002	316.8	0.030255	0.066	15.525	15.525	2.780	0.179	3.956509
Dgkz	Dgkz-004	1080.4	0.000363	0.002	15.508	15.508	12.204	0.787	3.954956
Gpatch2	Gpatch2-008	693.5	0.020472	0.048	15.439	15.439	3.090	0.200	3.948507
Gle1	Gle1-004	393.1	0.000309	0.002	15.414	15.414	2.932	0.190	3.946126
Fam81a	Fam81a-002	535.7	0.000061	0.001	15.399	15.399	4.048	0.263	3.944777
Vwc2	Vwc2-201	480.0	0.004414	0.015	15.393	15.393	3.783	0.246	3.944241
Eml1	Eml1-004	1806.0	0.011459	0.031	15.380	15.380	13.469	0.876	3.942947
Mroh1	Mroh1-001	3009.0	0.000523	0.003	15.330	15.330	30.668	2.001	3.938265
Fmo2	Fmo2-004	3363.5	0.031711	0.068	15.328	15.328	27.956	1.824	3.938067
Cad	Cad-003	449.6	0.006886	0.021	15.313	15.313	6.470	0.423	3.936668
Ssr4	Ssr4-004	563.2	0.000028	0.000	15.259	15.259	5.796	0.380	3.931587
Ubl3	Ubl3-003	1306.8	0.000062	0.001	15.247	15.247	9.699	0.636	3.930476
Snx11	Snx11-001	835.1	0.012037	0.032	15.239	15.239	7.397	0.485	3.929742
Slc50a1	Slc50a1-001	1183.2	0.003789	0.013	15.192	15.192	13.385	0.881	3.925236
Map3k10	Map3k10-001	921.6	0.004233	0.014	15.184	15.184	7.093	0.467	3.924508
Fmr1	Fmr1-001	2601.1	0.000001	0.000	15.175	15.175	21.354	1.407	3.923623
Ptbp3	Ptbp3-006	886.2	0.004239	0.014	15.172	15.172	6.926	0.457	3.923367
Slc52a3	Slc52a3-002	651.1	0.005010	0.016	15.154	15.154	3.427	0.226	3.921617
Camsap2	Camsap2-003	6176.1	0.001830	0.007	15.126	15.126	45.095	2.981	3.918946
Adamts14	\damts14-001	113.0	0.015491	0.039	15.123	15.123	0.810	0.054	3.91865
Phldb1	Phldb1-007	3497.3	0.002487	0.009	15.075	15.075	24.595	1.631	3.914116
Alpk1	Alpk1-201	476.5	0.002172	0.008	15.066	15.066	3.113	0.207	3.913191
Calml4	Calml4-201	701.0	0.003458	0.012	15.034	15.034	2.678	0.178	3.910156
Zfp946	Zfp946-002	360.6	0.034244	0.073	15.025	15.025	1.228	0.082	3.909302
Tmem259	Tmem259-002	1509.4	0.001799	0.007	15.005	15.005	12.372	0.825	3.907366
Foxf2	Foxf2-001	293.0	0.000859	0.004	14.976	14.976	2.357	0.157	3.904576
Ptov1	Ptov1-009	335.8	0.048997	0.097	14.968	14.968	1.670	0.112	3.90382
Kmt2a	Kmt2a-002	3476.2	0.000039	0.000	14.967	14.967	23.898	1.597	3.903755
Fgf13	Fgf13-008	1407.2	0.001972	0.008	14.945	14.945	11.667	0.781	3.901598
Nras	Nras-003	786.2	0.000045	0.000	14.931	14.931	5.597	0.375	3.900239
Gm26638	Gm26638-001	680.0	0.000034	0.000	14.918	14.918	5.558	0.373	3.899024
Slfn5	Slfn5-003	993.0	0.019978	0.047	14.896	14.896	4.003	0.269	3.8969
Rpl7	Rpl7-001	12830.2	0.000066	0.001	14.888	14.888	119.920	8.055	3.896095
Nr1h2	Nr1h2-001	2952.6	0.000001	0.000	14.876	14.876	25.957	1.745	3.894926
Csnk1a1	Csnk1a1-002	1470.0	0.000836	0.004	14.873	14.873	12.245	0.823	3.894646
Spata5l1	Spata5l1-002	478.7	0.002286	0.009	14.861	14.861	3.633	0.244	3.893495
Ccdc12	Ccdc12-003	562.7	0.015857	0.040	14.857	14.857	3.292	0.222	3.893045
Agpat2	Agpat2-003	1180.1	0.000134	0.001	14.803	14.803	9.531	0.644	3.887837
Svil	Svil-013	16958.1	0.000026	0.000	14.776	14.776	125.294	8.480	3.885154
Pam	Pam-011	8089.6	0.000004	0.000	14.743	14.743	59.344	4.025	3.882004
Gm22392	Gm22392-201	1121.0	0.000009	0.000	14.694	14.694	8.537	0.581	3.877179
Gmeb2	Gmeb2-002	414.7	0.046879	0.093	14.653	14.653	3.062	0.209	3.873076
932702P03R	2702P03Rik-	2012.8	0.000113	0.001	14.641	14.641	16.972	1.159	3.871927
Fbxo34	Fbxo34-205	2415.4	0.000004	0.000	14.641	14.641	14.375	0.982	3.871895
Slc4a4	Slc4a4-006	1612.3	0.000154	0.001	14.637	14.637	6.362	0.435	3.871581
Acvrl1	Acvrl1-005	2628.4	0.000610	0.003	14.633	14.633	17.984	1.229	3.871124
Sft2d2	Sft2d2-002	3565.0	0.000077	0.001	14.621	14.621	25.209	1.724	3.870008
Kdm5c	Kdm5c-002	3739.6	0.000041	0.000	14.591	14.591	27.611	1.892	3.86696
Bmpr1a	Bmpr1a-003	2251.8	0.000033	0.000	14.588	14.588	18.155	1.244	3.866707
Mapkapk5	Mapkapk5-001	394.1	0.022769	0.053	14.581	14.581	1.924	0.132	3.866048
Hba-a1	Hba-a1-001	9496.8	0.000259	0.002	14.516	14.516	135.200	9.314	3.85955
Cdr2	Cdr2-001	1207.2	0.000300	0.002	14.511	14.511	12.744	0.878	3.859107
Trim47	Trim47-007	413.7	0.001619	0.007	14.499	14.499	3.899	0.269	3.857866
Tfeb	Tfeb-003	585.8	0.003373	0.012	14.498	14.498	3.100	0.214	3.857779
931406B18R	1406B18Rik-	984.2	0.000013	0.000	14.463	14.463	8.131	0.562	3.854334
Agrn	Agrn-001	3939.5	0.000641	0.003	14.434	14.434	19.013	1.317	3.851355
Crk	Crk-005	1256.3	0.000160	0.001	14.423	14.423	8.943	0.620	3.850287
Il3ra	Il3ra-201	846.0	0.000111	0.001	14.402	14.402	6.129	0.426	3.848236
Surf1	Surf1-001	794.6	0.000144	0.001	14.400	14.400	6.234	0.433	3.848033
Nadk	Nadk-002	6164.7	0.000007	0.000	14.399	14.399	45.907	3.188	3.847865
Ccdc61	Ccdc61-001	1260.4	0.002413	0.009	14.384	14.384	7.740	0.538	3.846417
Mapk8ip3	Mapk8ip3-007	945.3	0.000900	0.004	14.366	14.366	6.152	0.428	3.844615
Ralgds	Ralgds-003	1280.4	0.001005	0.005	14.346	14.346	15.295	1.066	3.842571
C1qa	C1qa-001	1285.0	0.000867	0.004	14.323	14.323	12.728	0.889	3.840255
Taok2	Taok2-004	504.2	0.000406	0.002	14.319	14.319	2.810	0.196	3.839878
Dlgap4	Dlgap4-007	1585.4	0.002152	0.008	14.318	14.318	10.982	0.767	3.839792
Mknk2	Mknk2-001	13799.9	0.000000	0.000	14.312	14.312	71.174	4.973	3.839155
Gm25021	Gm25021-201	436.0	0.000320	0.002	14.311	14.311	3.429	0.240	3.83901
932428N05R	2428N05Rik-	1070.9	0.000081	0.001	14.306	14.306	7.310	0.511	3.838532
Galnt11	Galnt11-001	649.0	0.003780	0.013	14.294	14.294	5.972	0.418	3.837378
Adam10	Adam10-002	11600.1	0.000010	0.000	14.151	14.151	74.776	5.284	3.822825
Dhdds	Dhdds-002	940.6	0.001592	0.007	14.140	14.140	6.103	0.432	3.821689
Zfp219	Zfp219-202	985.7	0.018273	0.044	14.079	14.079	11.792	0.838	3.815439
00017D01R	0017D01Rik-	529.0	0.000199	0.001	14.040	14.040	4.447	0.317	3.811515
Abcc5	Abcc5-001	3134.0	0.000963	0.004	14.033	14.033	21.205	1.511	3.810728
Adrbk1	Adrbk1-001	7211.7	0.000004	0.000	13.996	13.996	57.701	4.123	3.806942
Fam20a	Fam20a-001	3211.0	0.000441	0.002	13.993	13.993	10.026	0.717	3.806609
Slc17a8	Slc17a8-001	1309.0	0.000010	0.000	13.985	13.985	12.238	0.875	3.805853
P4ha2	P4ha2-007	1345.0	0.001303	0.006	13.985	13.985	5.950	0.425	3.805828
Nub1	Nub1-001	1495.7	0.000171	0.001	13.957	13.957	12.277	0.880	3.80288
Tmem8b	Tmem8b-009	561.6	0.015632	0.039	13.953	13.953	7.098	0.509	3.802533
Evi5l	Evi5l-002	158.3	0.013656	0.035	13.906	13.906	1.858	0.134	3.797585
Impdh1	Impdh1-002	1077.2	0.003648	0.012	13.904	13.904	6.500	0.468	3.797421
Rbms3	Rbms3-006	514.5	0.010905	0.029	13.897	13.897	5.035	0.362	3.79675
Sulf1	Sulf1-010	2473.4	0.000049	0.000	13.883	13.883	19.258	1.387	3.795278
Fan1	Fan1-001	332.5	0.030128	0.066	13.881	13.881	3.124	0.225	3.794995
Gnb2	Gnb2-004	1025.7	0.000112	0.001	13.877	13.877	5.701	0.411	3.794619
Ddx3x	Ddx3x-003	2846.1	0.000011	0.000	13.867	13.867	23.854	1.720	3.793568
Scrib	Scrib-002	882.3	0.011455	0.031	13.827	13.827	6.632	0.480	3.789369
E2f5	E2f5-201	515.3	0.049567	0.097	13.825	13.825	4.284	0.310	3.789215
Rab11a	Rab11a-002	3258.1	0.008132	0.023	13.824	13.824	17.990	1.301	3.789087
Adcy9	Adcy9-002	1583.3	0.004471	0.015	13.802	13.802	10.488	0.760	3.786836
Sgms1	Sgms1-008	2168.3	0.000001	0.000	13.800	13.800	15.148	1.098	3.786579
Stat5b	Stat5b-001	5217.6	0.002089	0.008	13.796	13.796	42.333	3.069	3.786134
Gnas	Gnas-201	39473.3	0.000000	0.000	13.778	13.778	285.788	20.743	3.784257
Phldb1	Phldb1-006	563.8	0.015831	0.039	13.765	13.765	4.318	0.314	3.782893
Maml2	Maml2-002	414.5	0.013744	0.035	13.765	13.765	3.892	0.283	3.782883
Zfp335	Zfp335-003	1620.6	0.014767	0.037	13.683	13.683	14.855	1.086	3.774353

Ahcyl2	Ahcyl2-002	990.4	0.000997	0.005	13.681	13.681	9.296	0.679	3.774118
Golga1	Golga1-002	726.6	0.046599	0.093	13.617	13.617	5.571	0.409	3.76733
Mtmr11	Mtmr11-001	974.7	0.018677	0.045	13.612	13.612	8.620	0.633	3.766808
Islr	Islr-201	236.8	0.006977	0.021	13.559	13.559	1.447	0.107	3.761204
Usp33	Usp33-010	236.6	0.038528	0.080	13.553	13.553	1.888	0.139	3.760593
Dab2ip	Dab2ip-008	6136.5	0.000066	0.001	13.531	13.531	42.108	3.112	3.758146
Aldh2	Aldh2-005	6773.2	0.000194	0.001	13.517	13.517	41.407	3.063	3.756735
Zfp93	Zfp93-001	336.4	0.026151	0.059	13.460	13.460	4.168	0.310	3.750648
Skp1a	Skp1a-005	1418.9	0.000011	0.000	13.426	13.426	13.891	1.035	3.746971
Myo1d	Myo1d-002	2880.4	0.002018	0.008	13.418	13.418	22.873	1.705	3.746149
Ubp1	Ubp1-203	1577.7	0.000739	0.004	13.379	13.379	16.081	1.202	3.741852
Set	Set-005	2046.6	0.000044	0.000	13.366	13.366	16.319	1.221	3.740505
Pkig	Pkig-001	804.0	0.012573	0.033	13.350	13.350	9.654	0.723	3.738729
Rdx	Rdx-201	12824.4	0.000000	0.000	13.316	13.316	103.796	7.795	3.735099
Ambra1	Ambra1-002	2452.5	0.000063	0.001	13.297	13.297	18.564	1.396	3.732989
Ppp1r9b	Ppp1r9b-001	17309.0	0.000000	0.000	13.295	13.295	139.569	10.498	3.73282
Stard7	Stard7-001	1124.9	0.037850	0.079	13.294	13.294	10.637	0.800	3.732743
Pax6	Pax6-024	2507.9	0.000001	0.000	13.268	13.268	20.736	1.563	3.729867
Shisa5	Shisa5-003	1918.4	0.000167	0.001	13.266	13.266	11.105	0.837	3.729689
Ndufv1	Ndufv1-001	1309.7	0.001507	0.006	13.250	13.250	8.654	0.653	3.727903
Gm7416	Gm7416-001	2849.0	0.000002	0.000	13.203	13.203	25.546	1.935	3.722746
Hdac7	Hdac7-007	2345.3	0.000327	0.002	13.163	13.163	18.100	1.375	3.718398
Mgat1	Mgat1-201	403.8	0.005303	0.017	13.155	13.155	3.511	0.267	3.717508
Tle2	Tle2-004	166.9	0.047646	0.094	13.098	13.098	1.052	0.080	3.71124
Ywhae	Ywhae-003	5853.3	0.000001	0.000	13.074	13.074	41.548	3.178	3.708633
Crip2	Crip2-003	1459.9	0.000005	0.000	13.065	13.065	9.704	0.743	3.707611
Ncor2	Ncor2-006	1022.1	0.000295	0.002	13.065	13.065	9.451	0.723	3.707607
Bzw1	Bzw1-002	1027.1	0.016763	0.041	13.054	13.054	7.651	0.586	3.706414
Arid1b	Arid1b-002	10945.3	0.000000	0.000	13.046	13.046	80.036	6.135	3.705566
Phldb1	Phldb1-001	2492.2	0.009403	0.026	13.021	13.021	15.476	1.189	3.702789
Kdm2b	Kdm2b-001	398.7	0.043916	0.088	12.975	12.975	4.200	0.324	3.697681
Hyal1	Hyal1-001	2738.2	0.016597	0.041	12.959	12.959	18.868	1.456	3.695895
Iffo2	Iffo2-001	773.8	0.000023	0.000	12.940	12.940	6.111	0.472	3.693738
Klhl18	Klhl18-005	644.8	0.015173	0.038	12.923	12.923	4.762	0.369	3.691855
Gse1	Gse1-003	507.4	0.004283	0.014	12.917	12.917	1.559	0.121	3.691194
Nfib	Nfib-002	5470.3	0.000011	0.000	12.903	12.903	38.945	3.018	3.689594
Myc	Myc-202	1689.6	0.040441	0.083	12.885	12.885	17.340	1.346	3.687585
Rrp7a	Rrp7a-005	400.6	0.002261	0.009	12.868	12.868	3.267	0.254	3.685709
Galnt2	Galnt2-001	4921.9	0.000047	0.000	12.850	12.850	33.326	2.593	3.683732
Gria2	Gria2-007	797.0	0.000007	0.000	12.817	12.817	6.453	0.503	3.680042
Sorbs2	Sorbs2-023	545.8	0.026822	0.060	12.815	12.815	5.191	0.405	3.679721
Por	Por-003	785.8	0.035672	0.075	12.807	12.807	5.037	0.393	3.678829
Supt20	Supt20-001	1351.5	0.000134	0.001	12.798	12.798	13.348	1.043	3.677887
Anapc2	Anapc2-002	1413.7	0.000008	0.000	12.794	12.794	10.957	0.856	3.677398
Ddx19a	Ddx19a-003	749.9	0.000016	0.000	12.788	12.788	6.417	0.502	3.67671
Klhdc3	Klhdc3-201	1283.2	0.005343	0.017	12.759	12.759	12.969	1.016	3.673474
Armcx1	Armcx1-001	606.3	0.004727	0.015	12.752	12.752	5.001	0.392	3.672685
Kbtbd4	Kbtbd4-001	937.3	0.017230	0.042	12.723	12.723	6.991	0.549	3.669405
Rpl12	Rpl12-003	20469.2	0.000001	0.000	12.722	12.722	166.505	13.088	3.669208
Chd3	Chd3-201	9013.7	0.000000	0.000	12.697	12.697	68.355	5.384	3.666374
Ccm2l	Ccm2l-003	1443.6	0.000083	0.001	12.648	12.648	7.088	0.560	3.660868
Phactr2	Phactr2-003	4241.3	0.000005	0.000	12.630	12.630	30.663	2.428	3.658813
Rptor	Rptor-003	2073.8	0.000004	0.000	12.618	12.618	15.456	1.225	3.657401
Spr	Spr-002	1017.8	0.001186	0.005	12.615	12.615	7.615	0.604	3.657099
Slc4a4	Slc4a4-003	650.5	0.016419	0.041	12.611	12.611	2.344	0.186	3.656603
Zzz3	Zzz3-002	2370.6	0.013519	0.035	12.599	12.599	17.844	1.416	3.655256
Sap18	Sap18-005	1468.0	0.018734	0.045	12.595	12.595	11.899	0.945	3.654737
Aftph	Aftph-001	1112.8	0.000003	0.000	12.592	12.592	8.250	0.655	3.654492
Rnf146	Rnf146-001	588.6	0.003129	0.011	12.525	12.525	3.191	0.255	3.646772
Rit1	Rit1-006	662.6	0.000849	0.004	12.519	12.519	4.933	0.394	3.646029
Jade2	Jade2-001	1922.4	0.004592	0.015	12.496	12.496	12.233	0.979	3.643382
Cp	Cp-008	3034.8	0.001247	0.005	12.481	12.481	28.598	2.291	3.641633
Sptan1	Sptan1-012	674.4	0.000411	0.002	12.475	12.475	2.212	0.177	3.640981
Vps8	Vps8-201	463.2	0.000199	0.001	12.456	12.456	3.963	0.318	3.63876
Tcf4	Tcf4-022	1389.1	0.017893	0.043	12.437	12.437	11.410	0.917	3.636512
Snora31	Snora31-201	1689.0	0.000035	0.000	12.432	12.432	15.353	1.235	3.635992
Lincpint	Lincpint-002	961.8	0.000160	0.001	12.431	12.431	6.198	0.499	3.635909
Gm17786	Gm17786-001	192.0	0.001134	0.005	12.332	12.332	1.984	0.161	3.624281
Rhbdf1	Rhbdf1-006	1694.6	0.014147	0.036	12.310	12.310	10.736	0.872	3.621781
Zfp704	Zfp704-002	2037.0	0.000108	0.001	12.294	12.294	14.618	1.189	3.619923
Plekha1	Plekha1-001	908.9	0.000672	0.003	12.293	12.293	7.797	0.634	3.619735
Rgs20	Rgs20-002	1591.9	0.000021	0.000	12.289	12.289	11.977	0.975	3.619295
Nt5c2	Nt5c2-002	581.8	0.000044	0.000	12.277	12.277	4.927	0.401	3.617903
Itgb4	Itgb4-005	5409.8	0.000001	0.000	12.273	12.273	27.641	2.252	3.617427
Mtmr12	Mtmr12-001	430.9	0.046641	0.093	12.252	12.252	4.122	0.336	3.614903
Lrp6	Lrp6-003	706.3	0.022962	0.053	12.230	12.230	6.056	0.495	3.61232
Fxr1	Fxr1-005	1453.9	0.000392	0.002	12.208	12.208	11.475	0.940	3.609793
Ltbp4	Ltbp4-004	31820.6	0.000000	0.000	12.198	12.198	207.327	16.997	3.608558
Tma7	Tma7-004	775.6	0.000269	0.002	12.163	12.163	5.997	0.493	3.604485
Lpin3	Lpin3-004	1419.2	0.000692	0.003	12.158	12.158	13.948	1.147	3.603864
Tnrc18	Tnrc18-004	1965.0	0.000379	0.002	12.145	12.145	14.442	1.189	3.602242
Rnf216	Rnf216-008	433.2	0.025574	0.057	12.140	12.140	4.265	0.351	3.601732
Jade2	Jade2-004	4269.9	0.004938	0.016	12.139	12.139	38.861	3.201	3.601608
Gm26545	Gm26545-201	530.0	0.000107	0.001	12.135	12.135	4.483	0.369	3.601087
Ide	Ide-002	1296.0	0.000060	0.001	12.125	12.125	10.247	0.845	3.599856
Lamb2	Lamb2-007	1042.6	0.000362	0.002	12.112	12.112	7.338	0.606	3.598378
Pdlim7	Pdlim7-001	7904.9	0.000040	0.000	12.070	12.070	58.001	4.805	3.593399
Clpb	Clpb-006	402.8	0.005091	0.016	12.057	12.057	3.055	0.253	3.591767
Inpp1	Inpp1-006	314.8	0.028738	0.063	12.053	12.053	3.236	0.268	3.591267
Lrpap1	Lrpap1-005	4764.3	0.005131	0.016	12.010	12.010	30.351	2.527	3.586223
Arhgef1	Arhgef1-002	480.4	0.001860	0.007	12.006	12.006	3.520	0.293	3.585653
Dopey1	Dopey1-017	924.1	0.004770	0.015	12.000	12.000	6.744	0.562	3.584952
Prdx1	Prdx1-004	152.7	0.040309	0.083	11.998	11.998	1.187	0.099	3.584726
Ino80d	Ino80d-004	937.0	0.016508	0.041	11.945	11.945	6.165	0.516	3.57831
Glrx2	Glrx2-001	1212.2	0.002223	0.009	11.944	11.944	8.582	0.719	3.578214
Ptk2b	Ptk2b-007	4444.7	0.001988	0.008	11.932	11.932	38.769	3.249	3.576746
Adam15	Adam15-002	21876.1	0.003075	0.011	11.924	11.924	57.241	4.800	3.575847
Hist2h2ac	Hist2h2ac-001	3723.1	0.000973	0.004	11.917	11.917	33.542	2.815	3.574936
Hipk3	Hipk3-002	4214.2	0.000043	0.000	11.909	11.909	44.492	3.736	3.573924
Gm15417	Gm15417-00[illegible]	218.8	0.003764	0.013	11.895	11.895	1.735	0.146	3.572226
Snrk	Snrk-003	1165.3	0.000090	0.001	11.875	11.875	8.174	0.688	3.569889
Scn1b	Scn1b-003	1982.1	0.020148	0.048	11.838	11.838	13.714	1.158	3.565412
Fgfr3	Fgfr3-202	1039.6	0.000556	0.003	11.838	11.838	1.390	0.117	3.565341
Phf14	Phf14-001	2861.6	0.039362	0.081	11.827	11.827	20.851	1.763	3.564038
Ahcyl2	Ahcyl2-005	915.5	0.026966	0.060	11.824	11.824	5.266	0.445	3.563672
Calu	Calu-007	1824.5	0.000007	0.000	11.820	11.820	10.914	0.923	3.563175
Pold1	Pold1-002	1022.6	0.005356	0.017	11.811	11.811	4.143	0.351	3.562007

Klc1	Klc1-009	1268.5	0.000256	0.002	11.810	11.810	8.603	0.728	3.561938
Gm27195	Gm27195-001	184.0	0.001042	0.005	11.783	11.783	1.563	0.133	3.558608
Gpr107	Gpr107-002	876.4	0.000355	0.002	11.770	11.770	6.269	0.533	3.557051
Rdx	Rdx-202	2519.1	0.000000	0.000	11.764	11.764	17.751	1.509	3.556319
.P23-425F8.:	23-425F8.1-0	4807.0	0.000016	0.000	11.757	11.757	42.992	3.657	3.555498
Adamts10	Adamts10-002	5405.9	0.000333	0.002	11.751	11.751	37.221	3.168	3.554672
Gm9945	Gm9945-201	296.0	0.001328	0.006	11.689	11.689	2.492	0.213	3.547074
Gm7367	Gm7367-201	661.0	0.000020	0.000	11.647	11.647	6.456	0.554	3.541825
Snap23	Snap23-009	1254.0	0.000471	0.003	11.638	11.638	11.736	1.008	3.54082
Gm29236	Gm29236-002	135.1	0.031381	0.068	11.637	11.637	1.627	0.140	3.540617
Tle2	Tle2-002	563.1	0.004716	0.015	11.627	11.627	2.576	0.222	3.539398
Acap3	Acap3-201	1361.1	0.013416	0.035	11.626	11.626	8.358	0.719	3.539266
Heg1	Heg1-005	236723.1	0.000000	0.000	11.603	11.603	1536.168	132.395	3.53641
Phactr1	Phactr1-201	973.7	0.023487	0.054	11.600	11.600	9.216	0.795	3.535996
Fgfrl1	Fgfrl1-001	2751.6	0.001460	0.006	11.599	11.599	25.338	2.185	3.535899
230029F24R	0029F24Rik-	960.9	0.000027	0.000	11.589	11.589	7.909	0.682	3.534737
Smarcal1	Smarcal1-002	697.9	0.038190	0.079	11.582	11.582	5.474	0.473	3.533863
230029F24R	0029F24Rik-	91.1	0.006915	0.021	11.575	11.575	0.750	0.065	3.532881
Syne3	Syne3-201	2098.2	0.010498	0.029	11.563	11.563	14.423	1.247	3.531415
Fbf1	Fbf1-001	1471.8	0.000717	0.004	11.558	11.558	9.749	0.844	3.530824
Map3k5	Map3k5-002	901.6	0.008659	0.025	11.456	11.456	6.896	0.602	3.51798
Rnpepl1	Rnpepl1-002	1140.2	0.023300	0.054	11.424	11.424	6.806	0.596	3.513971
Dennd3	Dennd3-008	2170.4	0.001503	0.006	11.419	11.419	19.254	1.686	3.513423
Ube2e3	Ube2e3-006	346.9	0.000369	0.002	11.381	11.381	2.971	0.261	3.508556
Hand2	Hand2-001	200.8	0.002065	0.008	11.359	11.359	2.011	0.177	3.505784
Nfe2l1	Nfe2l1-005	1568.6	0.000810	0.004	11.312	11.312	11.855	1.048	3.49974
Ccdc85c	Ccdc85c-201	1348.0	0.000053	0.000	11.306	11.306	11.102	0.982	3.498964
Gm17382	Gm17382-001	711.0	0.000247	0.002	11.279	11.279	6.249	0.554	3.495562
Plekhg2	Plekhg2-002	205.8	0.012657	0.033	11.239	11.239	2.182	0.194	3.490505
Trim39	Trim39-002	621.8	0.009514	0.027	11.208	11.208	5.483	0.489	3.486482
Gm25791	Gm25791-201	1310.9	0.000005	0.000	11.188	11.188	10.606	0.948	3.48393
Ace	Ace-002	39729.6	0.000004	0.000	11.174	11.174	282.730	25.302	3.482105
Plcb4	Plcb4-005	4964.6	0.000011	0.000	11.171	11.171	29.224	2.616	3.481657
Fstl1	Fstl1-002	2127.8	0.000001	0.000	11.154	11.154	14.228	1.276	3.479476
Lhfpl4	Lhfpl4-001	2642.0	0.000000	0.000	11.150	11.150	18.368	1.647	3.47894
Cdk8	Cdk8-003	934.7	0.000149	0.001	11.109	11.109	8.581	0.772	3.473649
Dhx15	Dhx15-004	6273.7	0.000001	0.000	11.081	11.081	50.532	4.560	3.469965
Tmcc3	Tmcc3-003	2569.4	0.000115	0.001	11.060	11.060	24.033	2.173	3.467275
32438A13R	2438A13Rik-	587.2	0.003512	0.012	11.036	11.036	4.217	0.382	3.4642
Tnfrsf22	Tnfrsf22-202	9462.7	0.007057	0.021	11.014	11.014	71.579	6.499	3.461302
Trim56	Trim56-002	371.4	0.003248	0.011	11.010	11.010	3.019	0.274	3.460777
Grrp1	Grrp1-001	874.4	0.003031	0.011	11.005	11.005	8.413	0.764	3.460027
Abhd12	Abhd12-001	1565.6	0.002029	0.008	11.000	11.000	14.147	1.286	3.459445
Hdlbp	Hdlbp-003	22261.1	0.001931	0.008	10.995	10.995	176.930	16.092	3.45874
Gm12021	Gm12021-001	270.0	0.000780	0.004	10.992	10.992	1.756	0.160	3.458384
Sf1	Sf1-012	4036.4	0.001780	0.007	10.983	10.983	31.973	2.911	3.457155
Cped1	Cped1-005	2227.9	0.000004	0.000	10.963	10.963	15.021	1.370	3.454598
Echdc1	Echdc1-002	499.6	0.027116	0.060	10.943	10.943	2.502	0.229	3.451887
Cadm2	Cadm2-001	184.0	0.003387	0.012	10.938	10.938	1.154	0.105	3.451246
Rpa1	Rpa1-005	2276.7	0.000222	0.001	10.932	10.932	14.195	1.298	3.450529
Mical3	Mical3-002	2995.1	0.001035	0.005	10.918	10.918	21.050	1.928	3.448702
Rgs7bp	Rgs7bp-201	3245.0	0.000002	0.000	10.905	10.905	28.009	2.569	3.446859
Golga2	Golga2-003	5293.4	0.000009	0.000	10.894	10.894	38.987	3.579	3.445451
Tnxb	Tnxb-001	6898.7	0.000000	0.000	10.877	10.877	47.118	4.332	3.443175
Kat2a	Kat2a-001	437.9	0.022059	0.051	10.874	10.874	2.907	0.267	3.442803
Atp2b2	Atp2b2-003	11161.0	0.000899	0.004	10.842	10.842	46.979	4.333	3.438498
Cdv3	Cdv3-007	935.8	0.000157	0.001	10.815	10.815	7.094	0.656	3.434957
Plekhg1	Plekhg1-002	589.9	0.036593	0.077	10.788	10.788	4.257	0.395	3.431295
Dgke	Dgke-002	648.1	0.000126	0.001	10.786	10.786	5.235	0.485	3.431155
Acvr1	Acvr1-004	5067.4	0.001789	0.007	10.782	10.782	38.427	3.564	3.430525
30018O15R	0018O15Rik-	374.0	0.000050	0.000	10.773	10.773	3.132	0.291	3.429306
Parva	Parva-004	1030.6	0.001904	0.008	10.762	10.762	9.606	0.893	3.427819
Chd7	Chd7-201	2894.4	0.006338	0.019	10.759	10.759	25.311	2.353	3.427406
Lrch3	Lrch3-006	770.7	0.037903	0.079	10.716	10.716	4.529	0.423	3.421692
Nav2	Nav2-008	1803.2	0.000078	0.001	10.688	10.688	16.124	1.509	3.417884
Zfp523	Zfp523-001	534.6	0.015998	0.040	10.687	10.687	4.390	0.411	3.417809
Vax2os	Vax2os-003	780.0	0.000026	0.000	10.686	10.686	6.274	0.587	3.417601
Specc1	Specc1-006	4820.4	0.000002	0.000	10.677	10.677	21.768	2.039	3.416404
Rhoa	Rhoa-004	5611.8	0.000000	0.000	10.654	10.654	34.936	3.279	3.413308
Mfsd10	Mfsd10-002	217.4	0.047002	0.093	10.650	10.650	0.785	0.074	3.412834
Dgcr2	Dgcr2-003	774.9	0.001315	0.006	10.650	10.650	8.370	0.786	3.412797
Kdm2b	Kdm2b-003	319.8	0.035368	0.075	10.639	10.639	3.668	0.345	3.41126
Map7d1	Map7d1-002	4969.8	0.000004	0.000	10.608	10.608	30.562	2.881	3.407026
Zfp111	Zfp111-002	522.2	0.009130	0.026	10.605	10.605	4.108	0.387	3.406671
Inppl1	Inppl1-002	592.6	0.006958	0.021	10.588	10.588	3.530	0.333	3.404295
Cdk15	Cdk15-001	8575.0	0.000001	0.000	10.587	10.587	74.214	7.010	3.404188
Naa15	Naa15-008	656.5	0.021672	0.051	10.559	10.559	5.907	0.559	3.400398
Fbxl16	Fbxl16-201	1333.0	0.000003	0.000	10.559	10.559	11.193	1.060	3.400334
Sncg	Sncg-001	4692.0	0.000002	0.000	10.552	10.552	33.633	3.187	3.399401
Hba-a2	Hba-a2-001	12719.0	0.000417	0.002	10.550	10.550	178.902	16.957	3.399208
Ly75	Ly75-201	1507.4	0.018928	0.046	10.536	10.536	13.539	1.285	3.397234
Sox17	Sox17-003	1231.1	0.011857	0.032	10.507	10.507	9.473	0.902	3.39323
Chrd	Chrd-001	1169.7	0.021556	0.050	10.504	10.504	3.182	0.303	3.392856
Tmem59	Tmem59-003	4803.3	0.000000	0.000	10.502	10.502	35.748	3.404	3.392607
Clasp1	Clasp1-003	5750.9	0.015731	0.039	10.484	10.484	44.908	4.284	3.390063
Tapt1	Tapt1-003	195.5	0.028070	0.062	10.480	10.480	1.844	0.176	3.389619
00002D01R	0002D01Rik-	1761.0	0.002194	0.008	10.472	10.472	12.223	1.167	3.388464
Tpm4	Tpm4-002	6365.9	0.000168	0.001	10.472	10.472	40.878	3.904	3.388408
Slc30a9	Slc30a9-009	179.3	0.004819	0.016	10.456	10.456	1.270	0.121	3.386301
Rnf215	Rnf215-004	420.6	0.000380	0.002	10.450	10.450	1.999	0.191	3.385395
30159F19R	0159F19Rik-	886.0	0.000043	0.000	10.442	10.442	6.616	0.634	3.384268
Trp53bp1	Trp53bp1-007	787.8	0.026554	0.059	10.426	10.426	6.289	0.603	3.382162
Rasgrf2	Rasgrf2-003	3362.9	0.000018	0.000	10.398	10.398	19.904	1.914	3.378295
Rplp2	Rplp2-001	19100.2	0.000012	0.000	10.396	10.396	178.593	17.179	3.377957
Tango2	Tango2-001	225.4	0.010815	0.029	10.363	10.363	1.744	0.168	3.373379
Epha5	Epha5-008	3722.7	0.000002	0.000	10.347	10.347	24.281	2.347	3.371194
Epha5	Epha5-010	170.6	0.002242	0.009	10.339	10.339	1.113	0.108	3.370093
Gtf2a2	Gtf2a2-002	238.6	0.022379	0.052	10.303	10.303	1.353	0.131	3.364964
Hk1	Hk1-013	2046.7	0.005385	0.017	10.280	10.280	17.431	1.696	3.361698
Dab2ip	Dab2ip-004	605.0	0.000340	0.002	10.266	10.266	2.775	0.270	3.359743
Dctn1	Dctn1-001	20755.5	0.000001	0.000	10.256	10.256	142.392	13.883	3.358437
Dpp8	Dpp8-202	5463.5	0.000000	0.000	10.241	10.241	41.085	4.012	3.356255
Srp72	Srp72-002	3032.1	0.000003	0.000	10.240	10.240	23.398	2.285	3.356197
Lats2	Lats2-010	618.8	0.000517	0.003	10.218	10.218	4.055	0.397	3.353078
Gpsm1	Gpsm1-001	777.9	0.009404	0.026	10.201	10.201	6.066	0.595	3.350693
Mkln1	Mkln1-009	709.7	0.015343	0.039	10.167	10.167	4.632	0.456	3.345829
Dlgap4	Dlgap4-008	727.5	0.016180	0.040	10.161	10.161	4.581	0.451	3.344949

Mef2d	Mef2d-003	2323.9	0.000015	0.000	10.118	10.118	18.363	1.815	3.338857
Mecom	Mecom-007	1812.9	0.001313	0.006	10.115	10.115	15.335	1.516	3.338475
H2-Q7	H2-Q7-001	975.5	0.023336	0.054	10.109	10.109	5.615	0.555	3.337598
Tirap	Tirap-004	1261.9	0.000471	0.003	10.103	10.103	10.034	0.993	3.336737
Gpx4	Gpx4-001	3424.4	0.000789	0.004	10.071	10.071	27.470	2.728	3.332087
Pnrc2	Pnrc2-002	1012.1	0.001545	0.006	10.064	10.064	7.880	0.783	3.331103
Lor	Lor-001	984.0	0.000244	0.002	10.062	10.062	7.935	0.789	3.330892
Ahsg	Ahsg-201	1489.0	0.003708	0.013	10.056	10.056	15.816	1.573	3.330007
Ankle2	Ankle2-002	2372.0	0.003448	0.012	10.041	10.041	17.928	1.785	3.327893
Alyref	Alyref-003	456.8	0.001760	0.007	10.037	10.037	3.661	0.365	3.327193
Nfic	Nfic-004	2801.5	0.000015	0.000	10.035	10.035	15.667	1.561	3.327029
Hist1h4c	Hist1h4c-001	970.0	0.000066	0.001	10.033	10.033	9.218	0.919	3.326725
Gpaa1	Gpaa1-001	504.2	0.019199	0.046	10.033	10.033	4.042	0.403	3.326685
9-Sep	Sept9-007	1356.3	0.025977	0.058	10.026	10.026	6.486	0.647	3.32565
Vezt	Vezt-002	589.0	0.021669	0.051	10.014	10.014	4.613	0.461	3.323886
C4a	C4a-201	58.4	0.016322	0.040	9.995	9.995	0.593	0.059	3.321166
C4a	C4a-001	81.5	0.011277	0.030	9.993	9.993	0.521	0.052	3.320865
Zfp664	Zfp664-001	2330.6	0.000056	0.001	9.990	9.990	20.714	2.073	3.320506
Tmf1	Tmf1-002	2443.6	0.000124	0.001	9.962	9.962	17.621	1.769	3.316467
Cers2	Cers2-006	388.9	0.002044	0.008	9.941	9.941	1.600	0.161	3.313344
Kat2b	Kat2b-005	322.7	0.000666	0.003	9.931	9.931	3.202	0.322	3.312006
Sdf4	Sdf4-002	8026.0	0.016627	0.041	9.886	9.886	58.363	5.904	3.305347
Mybbp1a	Mybbp1a-001	1816.3	0.001187	0.005	9.876	9.876	19.576	1.982	3.303916
Itgb1bp1	Itgb1bp1-006	842.0	0.000368	0.002	9.863	9.863	7.145	0.724	3.302041
Fgfr1	Fgfr1-007	1033.8	0.000602	0.003	9.860	9.860	8.735	0.886	3.301563
Hnrnpk	Hnrnpk-012	2485.1	0.009777	0.027	9.849	9.849	20.713	2.103	3.300005
Ptgr2	Ptgr2-002	487.7	0.004327	0.014	9.839	9.839	3.690	0.375	3.29844
Cbx6	Cbx6-004	250.9	0.001007	0.005	9.829	9.829	2.471	0.251	3.297107
Nfat5	Nfat5-005	324.8	0.006136	0.019	9.806	9.806	1.816	0.185	3.293708
Tbp	Tbp-011	532.9	0.003053	0.011	9.802	9.802	4.161	0.425	3.293101
Gls	Gls-004	1227.8	0.000060	0.001	9.789	9.789	8.159	0.833	3.291146
Bnip3l	Bnip3l-005	3559.6	0.000207	0.001	9.785	9.785	25.645	2.621	3.290617
Pgrmc2	Pgrmc2-002	486.8	0.000508	0.003	9.780	9.780	2.904	0.297	3.289773
Fastk	Fastk-005	1417.6	0.019302	0.046	9.777	9.777	10.853	1.110	3.289336
Zkscan17	Zkscan17-001	1591.9	0.018060	0.044	9.767	9.767	16.373	1.676	3.287883
Chp1	Chp1-005	541.4	0.024453	0.056	9.765	9.765	3.382	0.346	3.28759
Pkp4	Pkp4-012	1712.5	0.000002	0.000	9.764	9.764	10.407	1.066	3.287471
Gstm5	Gstm5-003	389.4	0.000641	0.003	9.744	9.744	3.550	0.364	3.284489
Prkcd	Prkcd-202	334.4	0.011467	0.031	9.739	9.739	1.935	0.199	3.283825
Rpap1	Rpap1-002	574.0	0.000322	0.002	9.727	9.727	6.392	0.657	3.281934
Prpf40b	Prpf40b-010	1180.9	0.010735	0.029	9.721	9.721	9.827	1.011	3.281089
Chmp4b	Chmp4b-003	753.0	0.003199	0.011	9.717	9.717	6.318	0.650	3.28046
Ptch1	Ptch1-007	1942.5	0.000051	0.000	9.713	9.713	14.287	1.471	3.279895
Sf3b1	Sf3b1-002	348.5	0.026931	0.060	9.710	9.710	2.613	0.269	3.279504
Tle2	Tle2-006	593.1	0.049415	0.097	9.702	9.702	6.268	0.646	3.278323
Gga3	Gga3-009	281.0	0.024023	0.055	9.696	9.696	2.097	0.216	3.277414
Add1	Add1-001	12864.7	0.000001	0.000	9.689	9.689	93.618	9.663	3.276304
Usp19	Usp19-002	1573.2	0.000017	0.000	9.668	9.668	12.967	1.341	3.273203
Ubc	Ubc-003	3873.7	0.003914	0.013	9.662	9.662	29.205	3.023	3.27231
Cdk2ap2	Cdk2ap2-001	449.6	0.029911	0.065	9.637	9.637	5.222	0.542	3.268575
Pdxdc1	Pdxdc1-013	420.7	0.008633	0.025	9.636	9.636	2.867	0.298	3.268361
Ftl1	Ftl1-001	28446.2	0.000102	0.001	9.631	9.631	236.303	24.535	3.267747
Mtmr10	Mtmr10-004	2040.5	0.000001	0.000	9.613	9.613	21.760	2.264	3.264919
Nfe2l1	Nfe2l1-002	1244.2	0.000553	0.003	9.600	9.600	10.907	1.136	3.263088
Vsig10	Vsig10-005	1042.7	0.000725	0.004	9.596	9.596	9.636	1.004	3.26243
Impdh1	Impdh1-001	2055.6	0.000887	0.004	9.590	9.590	12.343	1.287	3.261605
Pkn3	Pkn3-003	5418.8	0.000035	0.000	9.570	9.570	17.564	1.835	3.258568
Srsf1	Srsf1-007	331.7	0.001549	0.006	9.566	9.566	3.792	0.396	3.257874
Ski	Ski-001	5215.3	0.000001	0.000	9.561	9.561	37.551	3.928	3.257151
Scarna3b	Scarna3b-201	782.0	0.000097	0.001	9.546	9.546	5.768	0.604	3.254916
Itgb4	Itgb4-201	994.5	0.000168	0.001	9.495	9.495	4.943	0.521	3.247096
P23-326H18	23-326H18.1-	822.0	0.000111	0.001	9.481	9.481	6.810	0.718	3.245036
Snora26	Snora26-001	1051.1	0.000518	0.003	9.480	9.480	10.397	1.097	3.244905
Psmf1	Psmf1-002	356.0	0.043092	0.087	9.473	9.473	3.857	0.407	3.243818
Snora26	Snora26-201	91.3	0.013946	0.036	9.472	9.472	0.903	0.095	3.243607
Tulp4	Tulp4-008	5298.3	0.000003	0.000	9.471	9.471	34.808	3.675	3.243558
Lats1	Lats1-201	2180.0	0.000011	0.000	9.467	9.467	16.014	1.692	3.242882
Rpl19-ps12	pl19-ps12-00	195.0	0.000838	0.004	9.466	9.466	1.759	0.186	3.242727
Sox17	Sox17-006	1108.4	0.000624	0.003	9.465	9.465	5.818	0.615	3.242534
P24-230J14	24-230J14.4-(	7168.0	0.000001	0.000	9.461	9.461	67.137	7.096	3.242017
Rab11fip3	Rab11fip3-004	5383.6	0.000003	0.000	9.391	9.391	40.625	4.326	3.231344
Qrich1	Qrich1-001	7218.1	0.000008	0.000	9.363	9.363	53.798	5.746	3.227035
Nr1h3	Nr1h3-004	306.8	0.004215	0.014	9.351	9.351	3.628	0.388	3.225121
Apc	Apc-003	1265.0	0.000501	0.003	9.348	9.348	7.897	0.845	3.224731
Gm23547	Gm23547-201	588.0	0.000114	0.001	9.344	9.344	4.060	0.434	3.224046
Tpr	Tpr-004	633.7	0.000051	0.000	9.325	9.325	7.165	0.768	3.22112
Tbrg4	Tbrg4-001	340.3	0.017741	0.043	9.315	9.315	4.143	0.445	3.219484
Rhox8	Rhox8-001.1	3562.0	0.000000	0.000	9.298	9.298	26.872	2.890	3.216904
Ptov1	Ptov1-006	1227.7	0.014387	0.037	9.293	9.293	10.012	1.077	3.216101
Tef	Tef-010	2779.8	0.012326	0.032	9.292	9.292	19.040	2.049	3.216035
Pkp4	Pkp4-005	1492.9	0.031815	0.068	9.276	9.276	7.225	0.779	3.213452
Metap2	Metap2-011	2935.0	0.000050	0.000	9.268	9.268	22.307	2.407	3.21227
Rgs3	Rgs3-014	787.5	0.004855	0.016	9.257	9.257	5.443	0.588	3.210499
Mapk7	Mapk7-002	564.1	0.003770	0.013	9.251	9.251	5.328	0.576	3.209636
Setd8	Setd8-002	560.3	0.007394	0.022	9.242	9.242	2.907	0.315	3.208283
Cdkn1b	Cdkn1b-002	2050.5	0.000168	0.001	9.233	9.233	16.267	1.762	3.206748
Ldha	Ldha-006	444.7	0.001350	0.006	9.228	9.228	3.685	0.399	3.205944
Cc2d1b	Cc2d1b-001	2254.8	0.001865	0.007	9.224	9.224	16.339	1.771	3.205441
Nr2c2	Nr2c2-003	649.2	0.037703	0.078	9.223	9.223	5.812	0.630	3.205166
Cnn3	Cnn3-008	2608.8	0.000021	0.000	9.217	9.217	20.997	2.278	3.204302
Prkrir	Prkrir-002	1012.1	0.001146	0.005	9.210	9.210	7.385	0.802	3.203238
Gm10501	Gm10501-001	698.9	0.031190	0.067	9.209	9.209	4.976	0.540	3.203056
Klc1	Klc1-001	11986.7	0.000000	0.000	9.203	9.203	65.131	7.077	3.202126
Whsc1l1	Whsc1l1-004	3929.3	0.000008	0.000	9.193	9.193	26.273	2.858	3.200505
Micall2	Micall2-003	1926.9	0.000526	0.003	9.192	9.192	10.626	1.156	3.200315
Gnao1	Gnao1-003	819.4	0.000076	0.001	9.191	9.191	5.714	0.622	3.200231
Ski	Ski-003	27297.9	0.000000	0.000	9.177	9.177	163.372	17.803	3.197945
Rarg	Rarg-001	3691.0	0.000630	0.003	9.169	9.169	21.234	2.316	3.196748
Col5a2	Col5a2-004	519.3	0.002497	0.009	9.154	9.154	2.750	0.300	3.194366
Mdga1	Mdga1-201	417.5	0.003891	0.013	9.146	9.146	3.557	0.389	3.193134
RP24-86E4.	P24-86E4.1-0	123.0	0.007507	0.022	9.122	9.122	1.207	0.132	3.189288
Raph1	Raph1-201	1555.2	0.003227	0.011	9.121	9.121	15.614	1.712	3.189114
Gm25776	Gm25776-201	2227.0	0.000000	0.000	9.106	9.106	18.346	2.015	3.18677
Dapk3	Dapk3-202	3005.6	0.000627	0.003	9.095	9.095	24.719	2.718	3.185048
Lgmn	Lgmn-001	11701.3	0.001395	0.006	9.067	9.067	128.505	14.173	3.180651
Gm28447	Gm28447-001	324.0	0.001539	0.006	9.063	9.063	3.131	0.345	3.180031
Helz2	Helz2-002	1154.9	0.000095	0.001	9.063	9.063	5.569	0.615	3.18

Scaf1	Scaf1-001	3387.1	0.000036	0.000	9.043	9.043	16.714	1.848	3.176764
Arl16	Arl16-009	251.4	0.014101	0.036	9.043	9.043	1.790	0.198	3.176733
Dvl2	Dvl2-001	363.2	0.005481	0.017	9.038	9.038	3.132	0.347	3.176051
Kdm5a	Kdm5a-008	393.0	0.001829	0.007	9.034	9.034	3.144	0.348	3.175313
Cd300lg	Cd300lg-001	476.6	0.005172	0.016	9.020	9.020	4.249	0.471	3.173071
Whsc1l1	Whsc1l1-006	1136.0	0.000946	0.004	9.016	9.016	7.890	0.875	3.172551
Ctbp1	Ctbp1-004	522.4	0.000464	0.003	9.013	9.013	5.028	0.558	3.171982
Uimc1	Uimc1-001	377.3	0.028888	0.063	8.996	8.996	2.929	0.326	3.169293
Tcf7l1	Tcf7l1-006	1124.0	0.004698	0.015	8.993	8.993	7.674	0.853	3.168754
Ubtf	Ubtf-003	1242.7	0.001213	0.005	8.992	8.992	9.729	1.082	3.168706
Anxa3	Anxa3-003	5042.0	0.000001	0.000	8.976	8.976	34.447	3.838	3.166112
Tnfrsf14	Tnfrsf14-005	681.2	0.000305	0.002	8.951	8.951	6.788	0.758	3.161997
Rpl13a	Rpl13a-005	150.6	0.022593	0.052	8.947	8.947	1.150	0.129	3.161438
Unc13b	Unc13b-002	642.6	0.037405	0.078	8.928	8.928	6.585	0.738	3.158374
Adrm1	Adrm1-001	2285.4	0.033041	0.071	8.927	8.927	17.016	1.906	3.158128
Ramp2	Ramp2-002	12138.8	0.000000	0.000	8.925	8.925	48.535	5.438	3.157874
Fabp4	Fabp4-001	4662.7	0.000587	0.003	8.915	8.915	27.762	3.114	3.156179
Atxn2l	Atxn2l-001	4037.4	0.000344	0.002	8.884	8.884	26.032	2.930	3.151288
Snap23	Snap23-008	594.2	0.006320	0.019	8.878	8.878	4.332	0.488	3.150263
C4a	C4a-002	350.3	0.004987	0.016	8.860	8.860	3.137	0.354	3.147378
Aldh7a1	Aldh7a1-002	556.4	0.015026	0.038	8.845	8.845	3.053	0.345	3.144941
Dctn1	Dctn1-004	435.2	0.003559	0.012	8.806	8.806	4.128	0.469	3.138432
Kcna2	Kcna2-001	535.6	0.000137	0.001	8.802	8.802	3.977	0.452	3.137776
Kcna2	Kcna2-003	434.4	0.000246	0.002	8.801	8.801	3.226	0.367	3.137694
Bnip2	Bnip2-004	3237.4	0.000011	0.000	8.797	8.797	16.925	1.924	3.137029
Plxdc2	Plxdc2-003	4169.8	0.001927	0.008	8.784	8.784	35.041	3.989	3.13495
Mpp1	Mpp1-003	2365.5	0.000006	0.000	8.778	8.778	17.437	1.986	3.133964
Gnb1	Gnb1-201	8512.2	0.000000	0.000	8.770	8.770	53.525	6.103	3.132566
Hnrnpl	Hnrnpl-004	3650.4	0.005015	0.016	8.731	8.731	28.218	3.232	3.126211
Furin	Furin-001	9083.1	0.000000	0.000	8.719	8.719	72.874	8.358	3.124108
Gyg	Gyg-001	271.2	0.031269	0.068	8.710	8.710	1.557	0.179	3.122676
Erdr1	Erdr1-203	13996.8	0.000015	0.000	8.707	8.707	116.800	13.414	3.122181
Top3a	Top3a-002	701.7	0.001752	0.007	8.694	8.694	6.321	0.727	3.120015
Arhgef40	Arhgef40-003	2951.4	0.004169	0.014	8.693	8.693	22.536	2.593	3.119801
Mbd3	Mbd3-001	857.3	0.049246	0.097	8.688	8.688	10.126	1.165	3.119054
Anxa3	Anxa3-005	814.8	0.001871	0.007	8.642	8.642	6.065	0.702	3.111347
Phf12	Phf12-002	2731.9	0.000138	0.001	8.636	8.636	20.475	2.371	3.110355
Adgrl2	Adgrl2-006	880.1	0.002869	0.010	8.620	8.620	9.646	1.119	3.107686
Pkn2	Pkn2-007	1310.6	0.010447	0.029	8.604	8.604	10.009	1.163	3.105002
Tnks2	Tnks2-008	556.0	0.001462	0.006	8.598	8.598	3.944	0.459	3.103996
Gm6457	Gm6457-201	337.0	0.000865	0.004	8.574	8.574	2.748	0.321	3.100019
Tceb1	Tceb1-008	515.4	0.000343	0.002	8.569	8.569	4.024	0.470	3.099091
Tfap2a	Tfap2a-202	812.2	0.000056	0.001	8.564	8.564	6.105	0.713	3.098248
Rcor3	Rcor3-005	1123.3	0.006903	0.021	8.562	8.562	7.139	0.834	3.098008
Slc4a4	Slc4a4-007	6724.4	0.035008	0.074	8.543	8.543	29.104	3.407	3.094816
Arhgef10	Arhgef10-001	7348.9	0.000171	0.001	8.538	8.538	44.592	5.222	3.093978
Agfg1	Agfg1-002	812.9	0.012731	0.033	8.538	8.538	7.227	0.846	3.09393
Ninj1	Ninj1-001	3835.3	0.000195	0.001	8.534	8.534	27.076	3.173	3.09316
Apaf1	Apaf1-003	1110.0	0.035469	0.075	8.529	8.529	8.545	1.002	3.09246
Dvl1	Dvl1-002	1520.7	0.004225	0.014	8.520	8.520	14.270	1.675	3.09089
Sox6	Sox6-205	291.6	0.005193	0.016	8.513	8.513	1.433	0.168	3.089608
Lrrc7	Lrrc7-005	1228.0	0.000016	0.000	8.513	8.513	9.712	1.141	3.089607
H2-D1	H2-D1-005	1159.8	0.000045	0.000	8.495	8.495	7.345	0.865	3.086696
Slc43a3	Slc43a3-005	1553.5	0.001030	0.005	8.488	8.488	10.138	1.194	3.085427
Osbpl2	Osbpl2-002	897.3	0.015742	0.039	8.464	8.464	8.356	0.987	3.081274
Osbpl7	Osbpl7-001	767.6	0.023421	0.054	8.457	8.457	4.128	0.488	3.080128
Rps27a	Rps27a-003	12657.8	0.000484	0.003	8.455	8.455	120.378	14.238	3.079797
Gm17174	Gm17174-001	189.0	0.003786	0.013	8.450	8.450	1.114	0.132	3.078867
Pfkfb3	Pfkfb3-002	5361.0	0.000312	0.002	8.430	8.430	47.850	5.676	3.075597
Stk38l	Stk38l-002	458.4	0.030446	0.066	8.425	8.425	2.631	0.312	3.074642
Surf2	Surf2-004	268.3	0.002708	0.010	8.422	8.422	2.078	0.247	3.074149
Zfp579	Zfp579-002	894.0	0.000654	0.003	8.420	8.420	7.656	0.909	3.073769
Acsm2	Acsm2-201	430.6	0.000251	0.002	8.391	8.391	3.383	0.403	3.068907
Lag3	Lag3-001	165.7	0.002423	0.009	8.391	8.391	1.285	0.153	3.068829
Acsm2	Acsm2-202	79.4	0.017286	0.042	8.383	8.383	0.624	0.074	3.067518
Zc3h15	Zc3h15-004	644.8	0.001778	0.007	8.382	8.382	6.937	0.828	3.067246
Llgl2	Llgl2-001	648.7	0.000965	0.004	8.378	8.378	5.817	0.694	3.066599
Oxr1	Oxr1-206	4195.1	0.000019	0.000	8.376	8.376	42.247	5.044	3.066196
Id1	Id1-002	1037.3	0.011006	0.030	8.367	8.367	9.214	1.101	3.064793
Hnrnpdl	Hnrnpdl-002	4467.3	0.000011	0.000	8.361	8.361	36.255	4.336	3.063637
Hr	Hr-001	1275.6	0.000454	0.002	8.358	8.358	3.763	0.450	3.06314
Nde1	Nde1-006	2538.5	0.004909	0.016	8.356	8.356	20.620	2.468	3.062731
Sat1	Sat1-005	405.1	0.027188	0.060	8.337	8.337	2.720	0.326	3.059567
Snap23	Snap23-005	1271.7	0.001330	0.006	8.328	8.328	9.603	1.153	3.058027
Hnrnpr	Hnrnpr-009	585.4	0.027994	0.062	8.322	8.322	4.479	0.538	3.056995
Ago3	Ago3-006	134.1	0.030443	0.066	8.319	8.319	1.260	0.151	3.056447
Coro7	Coro7-003	353.1	0.002672	0.010	8.264	8.264	2.147	0.260	3.046925
Klc2	Klc2-001	492.9	0.005540	0.017	8.263	8.263	4.115	0.498	3.046616
Ncor2	Ncor2-003	17686.0	0.000000	0.000	8.259	8.259	126.535	15.321	3.04592
Ncor1	Ncor1-008	4573.4	0.019562	0.047	8.251	8.251	33.200	4.024	3.044615
Fxyd5	Fxyd5-008	1101.6	0.000115	0.001	8.250	8.250	7.254	0.879	3.044424
Cep85	Cep85-005	424.9	0.000130	0.001	8.248	8.248	3.405	0.413	3.044126
Ywhah	Ywhah-003	3917.3	0.000082	0.001	8.228	8.228	25.736	3.128	3.040625
Tma7-ps	Tma7-ps-001	393.0	0.001425	0.006	8.228	8.228	2.806	0.341	3.040531
Ncaph2	Ncaph2-001	1579.8	0.002260	0.009	8.224	8.224	14.749	1.793	3.039803
Tmbim6	Tmbim6-006	563.2	0.000561	0.003	8.221	8.221	4.199	0.511	3.039262
230317F20R	30317F20Rik-	415.0	0.000598	0.003	8.204	8.204	4.146	0.505	3.036247
Inppl1	Inppl1-001	1425.5	0.026514	0.059	8.198	8.198	10.651	1.299	3.035268
Fyco1	Fyco1-001	60470.8	0.000008	0.000	8.183	8.183	439.435	53.704	3.032544
Uggt1	Uggt1-002	8662.5	0.000010	0.000	8.165	8.165	63.380	7.762	3.029497
Tuba1a	Tuba1a-002	461.1	0.000905	0.004	8.139	8.139	3.748	0.461	3.024842
Smarca2	Smarca2-002	9036.2	0.000024	0.000	8.131	8.131	60.958	7.497	3.023495
Arhgef40	Arhgef40-007	1162.9	0.002091	0.008	8.130	8.130	10.180	1.252	3.023228
Qsox1	Qsox1-001	2023.5	0.001711	0.007	8.126	8.126	11.745	1.445	3.022606
Tmem136	Tmem136-201	1112.0	0.000417	0.002	8.123	8.123	9.316	1.147	3.022086
Ap3m1	Ap3m1-002	987.1	0.024848	0.056	8.108	8.108	6.798	0.838	3.019346
Plac9b	Plac9b-003	741.2	0.000575	0.003	8.103	8.103	6.105	0.753	3.018461
Gm20628	Gm20628-001	1171.7	0.000475	0.003	8.100	8.100	11.917	1.471	3.017932
Actg1	Actg1-003	1483.5	0.000066	0.001	8.097	8.097	9.510	1.174	3.017467
Suco	Suco-003	306.6	0.001670	0.007	8.085	8.085	2.165	0.268	3.015249
Nfkb2	Nfkb2-202	984.4	0.003447	0.012	8.084	8.084	11.618	1.437	3.015072
Gabarap	Gabarap-004	1320.5	0.000709	0.003	8.062	8.062	10.906	1.353	3.01113
Agrn	Agrn-006	6899.4	0.000002	0.000	8.051	8.051	41.355	5.137	3.009111
Fkbp8	Fkbp8-004	1161.1	0.000008	0.000	8.046	8.046	8.979	1.116	3.008323
Atp11a	Atp11a-004	3048.7	0.000002	0.000	8.035	8.035	25.048	3.117	3.006296
Thrap3	Thrap3-015	481.7	0.010612	0.029	8.034	8.034	5.116	0.637	3.006145
00052L18R	0052L18Rik-	634.0	0.001420	0.006	8.015	8.015	4.737	0.591	3.00262

Lrrfip1	Lrrfip1-009	830.4	0.000162	0.001	8.010	8.010	8.009	1.000	3.001836
Trps1	Trps1-201	2307.2	0.000013	0.000	8.006	8.006	16.423	2.051	3.000997
Thra	Thra-004	3237.4	0.000011	0.000	7.998	7.998	23.814	2.978	2.999597
Rbbp7	Rbbp7-002	982.4	0.002101	0.008	7.996	7.996	7.364	0.921	2.999223
Gm5111	Gm5111-002	426.0	0.001459	0.006	7.990	7.990	3.168	0.396	2.998203
Atp1a2	Atp1a2-001	3043.0	0.000003	0.000	7.980	7.980	22.687	2.843	2.996317
Gm25848	Gm25848-201	564.0	0.000160	0.001	7.970	7.970	5.051	0.634	2.994524
Eya3	Eya3-006	399.3	0.003400	0.012	7.966	7.966	2.097	0.263	2.99383
Arhgef10l	Arhgef10l-007	214.5	0.031196	0.067	7.963	7.963	0.963	0.121	2.993331
Eps15	Eps15-005	2821.4	0.000810	0.004	7.956	7.956	18.598	2.338	2.991997
Pcnxl3	Pcnxl3-003	554.5	0.034697	0.073	7.954	7.954	4.118	0.518	2.991721
Ilk	Ilk-005	939.2	0.017637	0.043	7.954	7.954	7.325	0.921	2.99163
Hoxaas2	Hoxaas2-001	206.0	0.024739	0.056	7.937	7.937	1.045	0.132	2.988549
Map1lc3a	Map1lc3a-001	941.0	0.000030	0.000	7.935	7.935	9.210	1.161	2.988252
Ahr	Ahr-002	268.8	0.043751	0.088	7.930	7.930	2.323	0.293	2.987387
Boc	Boc-001	3989.1	0.000827	0.004	7.929	7.929	14.379	1.813	2.987198
Hsp90ab1	Hsp90ab1-008	519.2	0.043427	0.088	7.929	7.929	4.500	0.567	2.987158
Gm10709	Gm10709-201	12064.0	0.000000	0.000	7.928	7.928	106.281	13.405	2.987027
Nfkbil1	Nfkbil1-001	656.4	0.000165	0.001	7.901	7.901	5.617	0.711	2.982089
Akap13	Akap13-010	683.7	0.002185	0.008	7.901	7.901	3.593	0.455	2.98198
Ccdc12	Ccdc12-001	2657.9	0.000001	0.000	7.884	7.884	19.147	2.429	2.978938
Setx	Setx-003	320.7	0.014422	0.037	7.878	7.878	2.350	0.298	2.977867
Tcf4	Tcf4-007	2485.0	0.002654	0.010	7.873	7.873	21.142	2.686	2.976851
Mical2	Mical2-002	1351.7	0.000168	0.001	7.862	7.862	7.548	0.960	2.974931
Ehbp1	Ehbp1-201	763.9	0.016015	0.040	7.858	7.858	5.948	0.757	2.974151
Hdac5	Hdac5-019	930.3	0.034932	0.074	7.834	7.834	7.108	0.907	2.969707
Rapgef6	Rapgef6-002	1134.2	0.000586	0.003	7.833	7.833	6.129	0.782	2.969599
Wdfy3	Wdfy3-004	15068.3	0.000000	0.000	7.809	7.809	119.570	15.312	2.965161
Trim47	Trim47-004	1203.9	0.000943	0.004	7.803	7.803	10.644	1.364	2.964027
Jmjd1c	Jmjd1c-007	269.4	0.029984	0.065	7.802	7.802	2.344	0.300	2.96379
Gsk3b	Gsk3b-003	1789.2	0.008064	0.023	7.792	7.792	12.022	1.543	2.96204
Mknk1	Mknk1-001	499.7	0.012279	0.032	7.790	7.790	4.555	0.585	2.961602
Macf1	Macf1-008	9895.8	0.000036	0.000	7.785	7.785	93.757	12.044	2.960633
Thap7	Thap7-002	459.9	0.009216	0.026	7.773	7.773	3.258	0.419	2.95845
Fam134a	Fam134a-002	989.2	0.046752	0.093	7.771	7.771	5.389	0.693	2.958121
Ric3	Ric3-001	564.1	0.007274	0.021	7.761	7.761	3.635	0.468	2.956329
Rsrc2	Rsrc2-009	2721.6	0.004949	0.016	7.758	7.758	18.372	2.368	2.95576
Tnnt1	Tnnt1-012	466.6	0.000200	0.001	7.756	7.756	3.526	0.455	2.95526
Tbc1d4	Tbc1d4-005	336.2	0.025063	0.057	7.754	7.754	1.808	0.233	2.954991
Spock2	Spock2-001	18605.3	0.001868	0.007	7.722	7.722	41.600	5.387	2.948936
Nr2f6	Nr2f6-001	1045.7	0.000037	0.000	7.714	7.714	9.360	1.213	2.947564
Krt83	Krt83-201	123.0	0.022008	0.051	7.699	7.699	0.821	0.107	2.944582
Nr3c1	Nr3c1-004	2425.7	0.011663	0.031	7.672	7.672	12.987	1.693	2.939551
Emilin1	Emilin1-001	17006.7	0.000000	0.000	7.671	7.671	129.677	16.904	2.939486
Mical1	Mical1-201	1273.2	0.008344	0.024	7.671	7.671	10.757	1.402	2.939408
Tsc22d3	Tsc22d3-001	6350.8	0.003345	0.012	7.671	7.671	27.159	3.541	2.939354
Becn1	Becn1-003	1202.8	0.000983	0.004	7.670	7.670	8.744	1.140	2.939308
Chd3	Chd3-005	480.5	0.005030	0.016	7.663	7.663	2.920	0.381	2.937977
Gmeb1	Gmeb1-003	591.8	0.028907	0.063	7.648	7.648	5.340	0.698	2.935079
Fkbp1a	Fkbp1a-010	4287.4	0.000073	0.001	7.648	7.648	35.945	4.700	2.935077
Capg	Capg-001	1271.0	0.006787	0.020	7.640	7.640	10.078	1.319	2.933665
Agpat1	Agpat1-001	1787.8	0.003897	0.013	7.632	7.632	14.434	1.891	2.932128
Cpne8	Cpne8-002	1857.6	0.000085	0.001	7.628	7.628	18.248	2.392	2.931324
Atxn2	Atxn2-001	1262.3	0.000214	0.001	7.627	7.627	9.595	1.258	2.931024
Kif1c	Kif1c-002	20997.4	0.000382	0.002	7.626	7.626	129.958	17.042	2.930908
Pqlc1	Pqlc1-005	867.4	0.000248	0.002	7.616	7.616	3.687	0.484	2.92911
Megf11	Megf11-003	2215.9	0.000031	0.000	7.607	7.607	19.020	2.500	2.927321
Snapc4	Snapc4-201	416.0	0.012875	0.034	7.605	7.605	3.964	0.521	2.927013
Limch1	Limch1-009	1182.8	0.000670	0.003	7.572	7.572	9.114	1.204	2.920641
Ppm1m	Ppm1m-001	768.9	0.001366	0.006	7.563	7.563	6.083	0.804	2.918947
Ddx5	Ddx5-007	1280.4	0.002332	0.009	7.562	7.562	9.769	1.292	2.9187
Npdc1	Npdc1-001	2908.3	0.033390	0.071	7.545	7.545	17.922	2.375	2.91558
Ift140	Ift140-002	693.5	0.034379	0.073	7.542	7.542	4.714	0.625	2.915038
Huwe1	Huwe1-010	137.4	0.023387	0.054	7.526	7.526	1.510	0.201	2.911918
Hspa1b	Hspa1b-001	6256.0	0.000006	0.000	7.512	7.512	50.762	6.758	2.90912
Pear1	Pear1-005	1598.0	0.001383	0.006	7.511	7.511	11.392	1.517	2.909049
Mkx	Mkx-003	665.0	0.000535	0.003	7.511	7.511	4.780	0.636	2.908965
Zbtb12	Zbtb12-001	289.2	0.009401	0.026	7.506	7.506	2.791	0.372	2.908112
Oxr1	Oxr1-204	856.1	0.005001	0.016	7.494	7.494	5.956	0.795	2.905751
Adgrl2	Adgrl2-003	585.4	0.002506	0.009	7.476	7.476	3.930	0.526	2.902194
Polrmt	Polrmt-001	663.9	0.046223	0.092	7.466	7.466	4.832	0.647	2.900262
Sypl	Sypl-002	2943.5	0.000046	0.000	7.445	7.445	23.117	3.105	2.896276
Lmbr1l	Lmbr1l-001	848.8	0.003233	0.011	7.444	7.444	5.542	0.744	2.89612
Arhgap24	Arhgap24-001	3028.3	0.012226	0.032	7.438	7.438	33.801	4.544	2.894881
Eva1b	Eva1b-003	1057.2	0.024786	0.056	7.437	7.437	10.039	1.350	2.894741
Rpl37rt	Rpl37rt-001	6702.6	0.000000	0.000	7.435	7.435	63.068	8.483	2.894295
Rplp2	Rplp2-003	2408.1	0.000120	0.001	7.412	7.412	20.078	2.709	2.889958
Cacng4	Cacng4-001	1812.0	0.000001	0.000	7.412	7.412	12.561	1.695	2.889841
15-Sep	Sep15-003	3478.3	0.004402	0.014	7.405	7.405	24.481	3.306	2.888535
Xiap	Xiap-004	3781.3	0.000021	0.000	7.398	7.398	22.263	3.010	2.887062
Trmt1	Trmt1-003	715.4	0.033576	0.072	7.394	7.394	7.533	1.019	2.886394
Ppp1r18	Ppp1r18-201	7022.6	0.000064	0.001	7.389	7.389	55.319	7.487	2.885339
Alg1	Alg1-002	885.8	0.001911	0.008	7.387	7.387	7.674	1.039	2.885047
Eif1a	Eif1a-202	1092.1	0.035344	0.074	7.384	7.384	9.810	1.329	2.884386
Tax1bp3	Tax1bp3-001	1111.2	0.001337	0.006	7.381	7.381	8.953	1.213	2.88375
H2-K2	H2-K2-001	459.0	0.000715	0.004	7.375	7.375	4.709	0.638	2.882551
Prrc2c	Prrc2c-008	5170.0	0.000062	0.001	7.366	7.366	38.668	5.249	2.880939
Gabbr1	Gabbr1-010	204.3	0.010167	0.028	7.356	7.356	1.823	0.248	2.878934
Pmpca	Pmpca-002	735.7	0.000774	0.004	7.354	7.354	6.009	0.817	2.878607
Pnisr	Pnisr-004	1502.9	0.000729	0.004	7.352	7.352	9.507	1.293	2.878223
Dag1	Dag1-002	5917.9	0.001052	0.005	7.338	7.338	41.231	5.619	2.875439
Cyth1	Cyth1-001	1398.0	0.006155	0.019	7.330	7.330	9.206	1.256	2.873862
Syt11	Syt11-004	231.2	0.010363	0.028	7.321	7.321	2.495	0.341	2.872042
Golga2	Golga2-007	5876.3	0.000571	0.003	7.316	7.316	41.305	5.646	2.871125
Osbpl5	Osbpl5-001	8693.2	0.000000	0.000	7.282	7.282	66.707	9.161	2.864301
Hmgb1-ps1	Hmgb1-ps1-00	1820.0	0.000006	0.000	7.282	7.282	15.703	2.157	2.864293
Tmc6	Tmc6-001	2034.3	0.000017	0.000	7.278	7.278	9.405	1.292	2.863548
Spata6	Spata6-001	2169.0	0.000943	0.004	7.270	7.270	17.447	2.400	2.86192
Clk1	Clk1-005	1130.4	0.000558	0.003	7.266	7.266	8.069	1.111	2.861121
Shank3	Shank3-006	557.3	0.000896	0.004	7.256	7.256	3.180	0.438	2.859126
Uty	Uty-012	13523.0	0.000005	0.000	7.251	7.251	111.761	15.414	2.85812
Arhgap22	Arhgap22-004	564.0	0.005172	0.016	7.249	7.249	5.461	0.753	2.857711
Map7d1	Map7d1-001	21131.0	0.000003	0.000	7.245	7.245	140.416	19.381	2.857023
Slc50a1	Slc50a1-005	1105.1	0.000035	0.000	7.245	7.245	10.050	1.387	2.856937
Dnajb5	Dnajb5-001	533.6	0.008375	0.024	7.234	7.234	6.723	0.929	2.854834
Rilp	Rilp-001	1040.7	0.030158	0.066	7.233	7.233	6.244	0.863	2.854671
Pcdhga12	Pcdhga12-001	346.5	0.037606	0.078	7.227	7.227	2.767	0.383	2.853493

Actg1	Actg1-001	37404.2	0.000309	0.002	7.226	7.226	264.993	36.675	2.853105
Apc	Apc-002	1245.0	0.001199	0.005	7.224	7.224	9.990	1.383	2.852826
Pabpn1	Pabpn1-001	2426.0	0.000005	0.000	7.221	7.221	19.268	2.668	2.852121
Ccdc67	Ccdc67-002	168.9	0.023459	0.054	7.212	7.212	1.007	0.140	2.85049
Gm2710	Gm2710-001	197.0	0.001807	0.007	7.208	7.208	1.710	0.237	2.849615
Arfgef1	Arfgef1-001	2294.0	0.005086	0.016	7.208	7.208	15.195	2.108	2.849596
Gm5812	Gm5812-001	953.8	0.023626	0.054	7.201	7.201	6.104	0.848	2.848275
Flna	Flna-010	854.0	0.001997	0.008	7.201	7.201	8.264	1.148	2.848113
Foxp2	Foxp2-012	430.8	0.000423	0.002	7.192	7.192	3.338	0.464	2.846418
Ankrd24	Ankrd24-003	865.2	0.038444	0.080	7.192	7.192	6.691	0.930	2.846302
Snx11	Snx11-006	181.6	0.013945	0.036	7.183	7.183	1.039	0.145	2.844594
Gm11889	Gm11889-001	926.0	0.000008	0.000	7.182	7.182	6.667	0.928	2.844438
Ubxn1	Ubxn1-202	4239.5	0.000005	0.000	7.182	7.182	32.694	4.552	2.8444
Mrto4	Mrto4-003	376.9	0.001758	0.007	7.178	7.178	2.288	0.319	2.843599
Rrp8	Rrp8-002	856.4	0.027349	0.061	7.174	7.174	5.520	0.769	2.842817
Apbb2	Apbb2-017	4359.0	0.000003	0.000	7.172	7.172	27.172	3.788	2.842458
Lincpint	Lincpint-004	1201.5	0.002835	0.010	7.162	7.162	8.837	1.234	2.840359
Dnaja1	Dnaja1-007	1464.3	0.008859	0.025	7.161	7.161	11.471	1.602	2.840155
Egfl7	Egfl7-013	543.1	0.002697	0.010	7.154	7.154	4.514	0.631	2.838751
Dcaf7	Dcaf7-002	405.2	0.011899	0.032	7.151	7.151	2.339	0.327	2.838143
Xkrx	Xkrx-001	1923.0	0.000065	0.001	7.150	7.150	14.291	1.999	2.83789
Trpv4	Trpv4-005	1399.8	0.008467	0.024	7.144	7.144	9.977	1.397	2.836677
Lats2	Lats2-003	1770.2	0.000068	0.001	7.107	7.107	16.267	2.289	2.829149
Eef1a1	Eef1a1-008	1436.6	0.000073	0.001	7.106	7.106	14.748	2.075	2.82905
H2afy	H2afy-003	1493.0	0.003552	0.012	7.105	7.105	11.806	1.662	2.828923
10002H16R	0002H16Rik-	1100.4	0.000570	0.003	7.090	7.090	9.508	1.341	2.825701
Baz2b	Baz2b-011	138.6	0.022837	0.053	7.086	7.086	1.280	0.181	2.82501
Sgk1	Sgk1-003	8819.6	0.000001	0.000	7.078	7.078	78.530	11.094	2.823419
Cyth3	Cyth3-002	2967.5	0.000009	0.000	7.071	7.071	20.109	2.844	2.821868
Tnfrsf1a	Tnfrsf1a-005	1937.8	0.000250	0.002	7.061	7.061	15.310	2.168	2.819887
Ehd1	Ehd1-003	849.5	0.003387	0.012	7.052	7.052	4.968	0.704	2.817975
Ramp2	Ramp2-005	6420.3	0.000032	0.000	7.046	7.046	27.680	3.929	2.816737
Ago1	Ago1-005	784.3	0.038696	0.080	7.043	7.043	6.013	0.854	2.816274
Gm5829	Gm5829-001	251.0	0.002755	0.010	7.035	7.035	2.032	0.289	2.814504
Phldb2	Phldb2-003	6685.2	0.002799	0.010	7.035	7.035	55.898	7.946	2.814451
S100a16	S100a16-001	3139.6	0.000840	0.004	7.032	7.032	23.801	3.384	2.814002
Tapt1	Tapt1-001	1830.9	0.000189	0.001	7.023	7.023	13.187	1.878	2.812132
Brf2	Brf2-201	642.0	0.000350	0.002	7.019	7.019	4.778	0.681	2.811168
Uba52	Uba52-003	1911.6	0.001336	0.006	7.012	7.012	17.261	2.462	2.809901
Slc17a9	Slc17a9-002	517.0	0.003926	0.013	6.993	6.993	3.322	0.475	2.805822
Ryr2	Ryr2-201	240.0	0.005509	0.017	6.986	6.986	1.678	0.240	2.804457
Ryr2	Ryr2-202	240.0	0.005509	0.017	6.986	6.986	1.678	0.240	2.804457
Nedd4	Nedd4-008	2700.2	0.000115	0.001	6.981	6.981	16.797	2.406	2.803408
Tnip2	Tnip2-007	478.3	0.004369	0.014	6.978	6.978	3.826	0.548	2.802734
Stx18	Stx18-005	715.3	0.028390	0.062	6.971	6.971	5.618	0.806	2.801463
Znfx1	Znfx1-002	1553.0	0.024379	0.055	6.965	6.965	12.479	1.792	2.800148
Myo18a	Myo18a-202	298.9	0.007569	0.022	6.965	6.965	2.058	0.295	2.80014
Rbm6	Rbm6-003	658.8	0.023738	0.054	6.954	6.954	4.848	0.697	2.797921
Zfp12	Zfp12-001	775.1	0.002087	0.008	6.949	6.949	6.877	0.990	2.796885
Ece1	Ece1-003	3707.7	0.000229	0.001	6.938	6.938	30.074	4.335	2.794496
Cul7	Cul7-001	726.7	0.022821	0.053	6.936	6.936	5.330	0.768	2.794023
Rtn4rl1	Rtn4rl1-001	753.0	0.000542	0.003	6.934	6.934	3.145	0.454	2.793645
Usp17la	Usp17la-001	4823.0	0.000005	0.000	6.931	6.931	35.933	5.184	2.793053
Lmna	Lmna-003	4015.5	0.033898	0.072	6.931	6.931	30.335	4.377	2.793041
Hsd17b10	Hsd17b10-001	493.6	0.022357	0.052	6.930	6.930	4.882	0.705	2.792783
Gm10408	Gm10408-001	519.0	0.000098	0.001	6.911	6.911	3.287	0.476	2.788791
Mapk14	Mapk14-002	3730.6	0.000006	0.000	6.900	6.900	23.568	3.415	2.786684
Ambra1	Ambra1-003	666.1	0.013326	0.035	6.892	6.892	4.616	0.670	2.785001
Ddx3y	Ddx3y-002	3404.5	0.004474	0.015	6.874	6.874	24.771	3.604	2.781137
Hal	Hal-001	920.3	0.000705	0.003	6.868	6.868	7.767	1.131	2.779839
Setd3	Setd3-002	3626.6	0.000017	0.000	6.856	6.856	28.273	4.124	2.777373
P23-125B15	23-125B15.1-	698.0	0.000124	0.001	6.856	6.856	5.286	0.771	2.777308
Map3k6	Map3k6-001	11420.5	0.000182	0.001	6.846	6.846	68.725	10.038	2.775331
Gm13132	Gm13132-001	478.0	0.003300	0.012	6.837	6.837	1.643	0.240	2.773363
Pex19	Pex19-001	1444.2	0.009782	0.027	6.837	6.837	9.644	1.411	2.773272
Vps18	Vps18-002	390.6	0.045037	0.090	6.835	6.835	2.646	0.387	2.773011
Cdk2ap1	Cdk2ap1-002	673.9	0.031922	0.069	6.835	6.835	3.834	0.561	2.772939
Fam210a	Fam210a-002	153.0	0.008605	0.024	6.832	6.832	1.860	0.272	2.772217
Ncor1	Ncor1-013	1650.3	0.010947	0.030	6.813	6.813	13.534	1.987	2.768246
Etl4	Etl4-019	3641.5	0.001684	0.007	6.794	6.794	29.467	4.337	2.764356
Upf3b	Upf3b-003	979.4	0.003022	0.011	6.791	6.791	6.816	1.004	2.763546
Mink1	Mink1-006	1485.9	0.001085	0.005	6.788	6.788	10.025	1.477	2.763093
Dgkz	Dgkz-002	1763.6	0.000714	0.004	6.786	6.786	12.433	1.832	2.762532
Lifr	Lifr-201	12812.4	0.000747	0.004	6.778	6.778	90.161	13.301	2.760931
Acin1	Acin1-011	221.0	0.005401	0.017	6.778	6.778	2.573	0.380	2.760795
Pick1	Pick1-011	322.0	0.049348	0.097	6.776	6.776	2.498	0.369	2.760497
Tbkbp1	Tbkbp1-002	866.6	0.000342	0.002	6.771	6.771	7.171	1.059	2.759289
Stxbp6	Stxbp6-001	4995.5	0.000001	0.000	6.770	6.770	32.052	4.734	2.759164
Srsf4	Srsf4-001	4002.8	0.015688	0.039	6.755	6.755	25.890	3.833	2.755874
Rbm3	Rbm3-005	313.3	0.012824	0.033	6.747	6.747	2.445	0.362	2.754232
Akt2	Akt2-013	1011.3	0.001113	0.005	6.745	6.745	8.632	1.280	2.753799
Ldb1	Ldb1-001	5289.2	0.000105	0.001	6.741	6.741	38.061	5.646	2.752932
Slc35f1	Slc35f1-001	690.0	0.000444	0.002	6.738	6.738	5.139	0.763	2.752407
Rpl7a	Rpl7a-002	2687.4	0.000443	0.002	6.736	6.736	21.568	3.202	2.751798
Arap1	Arap1-002	5722.7	0.000520	0.003	6.729	6.729	38.770	5.761	2.750475
Ptp4a3	Ptp4a3-201	2871.8	0.001225	0.005	6.726	6.726	13.221	1.966	2.749782
Ewsr1	Ewsr1-003	2056.0	0.016657	0.041	6.724	6.724	14.411	2.143	2.749237
Edf1	Edf1-002	788.3	0.013274	0.034	6.714	6.714	2.419	0.360	2.74722
Scn2a1	Scn2a1-201	661.9	0.038766	0.080	6.687	6.687	2.906	0.435	2.741402
Plp1	Plp1-002	807.9	0.016542	0.041	6.687	6.687	8.015	1.199	2.741263
Camk1	Camk1-001	2912.6	0.038417	0.080	6.686	6.686	15.823	2.367	2.741081
Scn2a1	Scn2a1-001	205.7	0.038801	0.080	6.684	6.684	0.903	0.135	2.740779
Nr1h2	Nr1h2-005	554.1	0.015950	0.040	6.683	6.683	2.807	0.420	2.740491
30444P10R	0444P10Rik-	314.0	0.013574	0.035	6.677	6.677	1.779	0.266	2.739116
Paip1	Paip1-003	731.3	0.012761	0.033	6.673	6.673	5.620	0.842	2.73826
Phc3	Phc3-004	853.9	0.021267	0.050	6.672	6.672	5.332	0.799	2.738136
Arrb1	Arrb1-002	5476.4	0.014751	0.037	6.671	6.671	37.802	5.667	2.737912
Pink1	Pink1-001	5436.6	0.000046	0.000	6.668	6.668	47.048	7.056	2.737178
Mus81	Mus81-001	393.6	0.043391	0.088	6.653	6.653	4.800	0.721	2.734094
Ankib1	Ankib1-002	611.1	0.000816	0.004	6.649	6.649	4.500	0.677	2.733119
Pa2g4	Pa2g4-002	2455.8	0.003712	0.013	6.648	6.648	19.907	2.995	2.732817
Zfp367	Zfp367-003	804.4	0.005790	0.018	6.643	6.643	5.599	0.843	2.731753
Dctn6	Dctn6-009	625.1	0.024429	0.055	6.642	6.642	5.393	0.812	2.731622
Fyn	Fyn-004	1410.7	0.010186	0.028	6.635	6.635	7.716	1.163	2.730161
Fyn	Fyn-005	1101.0	0.007499	0.022	6.635	6.635	5.176	0.780	2.730119
Smtn	Smtn-003	3750.2	0.002672	0.010	6.624	6.624	32.041	4.837	2.727671
Ocel1	Ocel1-001	333.7	0.007634	0.022	6.622	6.622	2.332	0.352	2.727304

Gm12129	Gm12129-001	259.0	0.006492	0.020	6.618	6.618	2.103	0.318	2.726312
Bicd2	Bicd2-203	2802.6	0.000561	0.003	6.598	6.598	16.513	2.503	2.72212
Pou1f1	Pou1f1-001	1593.4	0.000180	0.001	6.592	6.592	13.394	2.032	2.720682
Zmiz1	Zmiz1-002	15410.2	0.000001	0.000	6.583	6.583	104.575	15.885	2.71881
Plxna2	Plxna2-005	3185.0	0.000247	0.002	6.581	6.581	25.139	3.820	2.718344
Ppp2r5b	Ppp2r5b-001	3658.5	0.000003	0.000	6.575	6.575	19.775	3.007	2.717074
L3mbtl2	L3mbtl2-001	899.2	0.003729	0.013	6.560	6.560	7.108	1.084	2.713616
Tbc1d22b	Tbc1d22b-00	340.3	0.020978	0.049	6.557	6.557	3.390	0.517	2.713121
Ncor1	Ncor1-201	1640.1	0.005301	0.017	6.555	6.555	12.123	1.849	2.712661
Myo18a	Myo18a-009	632.8	0.002471	0.009	6.553	6.553	2.729	0.416	2.712132
Mgll	Mgll-201	1093.3	0.002425	0.009	6.553	6.553	1.958	0.299	2.712127
Pou3f2	Pou3f2-001	498.0	0.001518	0.006	6.526	6.526	3.625	0.555	2.706182
Col25a1	Col25a1-006	254.0	0.008492	0.024	6.524	6.524	2.054	0.315	2.70567
Tm9sf1	Tm9sf1-006	778.2	0.020634	0.049	6.522	6.522	7.783	1.193	2.705419
Keap1	Keap1-004	1268.3	0.000404	0.002	6.522	6.522	7.193	1.103	2.70531
Kank1	Kank1-004	1113.9	0.000418	0.002	6.517	6.517	9.809	1.505	2.704314
Fam171a1	am171a1-00	8308.4	0.000000	0.000	6.515	6.515	63.847	9.800	2.703804
Tmem176b	mem176b-20	6312.2	0.000167	0.001	6.513	6.513	48.055	7.379	2.703265
Ppp1r18	Ppp1r18-001	2097.1	0.043708	0.088	6.508	6.508	21.573	3.315	2.702136
Rnh1	Rnh1-201	1385.9	0.001692	0.007	6.507	6.507	12.334	1.896	2.701938
Mylk4	Mylk4-201	3111.3	0.000008	0.000	6.500	6.500	23.520	3.619	2.70034
St3gal6	St3gal6-002	1004.5	0.013208	0.034	6.498	6.498	6.487	0.998	2.699932
Rasd2	Rasd2-001	2298.6	0.000523	0.003	6.468	6.468	9.685	1.497	2.693365
Ncoa2	Ncoa2-002	2663.4	0.000427	0.002	6.467	6.467	18.119	2.801	2.693193
Arl2bp	Arl2bp-201	3112.7	0.000008	0.000	6.466	6.466	23.839	3.687	2.692945
Ccdc80	Ccdc80-001	22789.0	0.000001	0.000	6.450	6.450	173.733	26.935	2.689313
Hdlbp	Hdlbp-004	3769.5	0.010631	0.029	6.441	6.441	28.679	4.452	2.687335
Dennd4b	Dennd4b-005	673.0	0.023163	0.053	6.438	6.438	4.963	0.771	2.686506
Gm17300	Gm17300-001	4075.5	0.000243	0.002	6.436	6.436	40.940	6.361	2.686118
Zzz3	Zzz3-005	473.1	0.022926	0.053	6.433	6.433	3.422	0.532	2.68552
Ndufb10	Ndufb10-001	3125.3	0.000096	0.001	6.423	6.423	23.501	3.659	2.683178
Smarcc1	Smarcc1-005	2264.9	0.010649	0.029	6.415	6.415	16.424	2.560	2.681474
Smarce1	Smarce1-005	564.3	0.001353	0.006	6.410	6.410	4.891	0.763	2.680355
Cgnl1	Cgnl1-005	8980.8	0.004754	0.015	6.403	6.403	32.550	5.084	2.678695
Zfp423	Zfp423-001	6992.8	0.000003	0.000	6.391	6.391	42.548	6.658	2.675976
Zscan25	Zscan25-001	881.2	0.001758	0.007	6.390	6.390	6.550	1.025	2.675867
G6pc3	G6pc3-002	354.8	0.016967	0.042	6.386	6.386	2.097	0.328	2.674853
Gramd4	Gramd4-002	3223.8	0.001745	0.007	6.381	6.381	25.322	3.969	2.673698
Ephx3	Ephx3-001	300.0	0.000602	0.003	6.377	6.377	2.197	0.345	2.672766
Pacsin2	Pacsin2-201	5473.6	0.000031	0.000	6.375	6.375	40.707	6.386	2.672315
Mzf1	Mzf1-006	2888.1	0.000090	0.001	6.374	6.374	24.088	3.779	2.672247
Gm17484	Gm17484-201	242.6	0.021150	0.050	6.359	6.359	2.167	0.341	2.668897
Hgs	Hgs-001	3620.6	0.000294	0.002	6.352	6.352	30.878	4.861	2.66719
Tex264	Tex264-001	592.5	0.006766	0.020	6.350	6.350	6.046	0.952	2.66687
Mbd3	Mbd3-002	2824.1	0.000273	0.002	6.350	6.350	21.991	3.463	2.666779
Chst15	Chst15-002	5166.9	0.000036	0.000	6.338	6.338	44.919	7.087	2.664065
Ism1	Ism1-001	925.3	0.035222	0.074	6.318	6.318	10.562	1.672	2.659368
Cdv3	Cdv3-001	3643.7	0.000027	0.000	6.316	6.316	26.242	4.155	2.65911
Flot2	Flot2-004	326.0	0.018549	0.045	6.309	6.309	2.652	0.420	2.657481
Snrpc	Snrpc-001	876.3	0.000364	0.002	6.303	6.303	8.017	1.272	2.656046
Elf2	Elf2-005	873.0	0.002764	0.010	6.302	6.302	6.873	1.091	2.655699
Gm8050	Gm8050-001	793.0	0.000038	0.000	6.297	6.297	4.984	0.791	2.654721
P23-54L16.	23-54L16.2-0	2295.0	0.000176	0.001	6.291	6.291	19.651	3.124	2.653216
Gm10575	Gm10575-001	290.2	0.006424	0.019	6.286	6.286	2.106	0.335	2.652052
Ilkap	Ilkap-003	791.9	0.003667	0.013	6.285	6.285	4.390	0.698	2.651924
Gtpbp2	Gtpbp2-010	585.9	0.001501	0.006	6.284	6.284	3.669	0.584	2.651599
Cbx3	Cbx3-002	3331.7	0.000018	0.000	6.283	6.283	27.400	4.361	2.651436
Igfbp4	Igfbp4-006	661.6	0.002422	0.009	6.271	6.271	6.481	1.033	2.648743
Large	Large-001	1729.2	0.000623	0.003	6.268	6.268	10.108	1.613	2.648011
Ypel3	Ypel3-002	888.5	0.000633	0.003	6.267	6.267	8.112	1.294	2.647824
Gtf2a1	Gtf2a1-002	2288.3	0.021800	0.051	6.266	6.266	14.919	2.381	2.647569
Bckdha	Bckdha-201	2149.0	0.000193	0.001	6.265	6.265	18.841	3.008	2.647225
Raf1	Raf1-001	3028.3	0.000245	0.002	6.252	6.252	22.242	3.558	2.644228
Pacsin3	Pacsin3-004	638.4	0.004260	0.014	6.249	6.249	4.599	0.736	2.643699
Actg1	Actg1-006	526.3	0.002761	0.010	6.248	6.248	3.586	0.574	2.643297
Tcf12	Tcf12-019	100.2	0.041778	0.085	6.243	6.243	0.997	0.160	2.642314
Ccnd3	Ccnd3-003	571.4	0.032874	0.070	6.241	6.241	5.972	0.957	2.641709
Cdc42bpa	Cdc42bpa-00	6002.7	0.000015	0.000	6.227	6.227	43.545	6.993	2.638607
Mum1	Mum1-008	994.3	0.021988	0.051	6.227	6.227	7.293	1.171	2.638436
Xpa	Xpa-001	975.7	0.018641	0.045	6.223	6.223	7.371	1.184	2.637583
Bcl2l1	Bcl2l1-003	1952.9	0.003234	0.011	6.223	6.223	9.540	1.533	2.637544
Rpl13a	Rpl13a-003	2903.4	0.000024	0.000	6.217	6.217	25.763	4.144	2.636245
Shroom4	Shroom4-004	7336.7	0.000279	0.002	6.206	6.206	45.800	7.380	2.633764
P2rx7	P2rx7-003	517.0	0.002836	0.010	6.206	6.206	4.507	0.726	2.633574
Srsf6	Srsf6-002	3320.1	0.001953	0.008	6.204	6.204	26.416	4.258	2.633155
Anks1	Anks1-003	1215.3	0.002478	0.009	6.204	6.204	10.163	1.638	2.6331
Tln2	Tln2-201	5282.4	0.048759	0.096	6.203	6.203	25.420	4.098	2.632887
Fry	Fry-010	3173.3	0.000353	0.002	6.190	6.190	24.538	3.964	2.629862
Mef2a	Mef2a-001	3037.6	0.000102	0.001	6.187	6.187	26.034	4.208	2.629262
Wdr44	Wdr44-003	461.3	0.002320	0.009	6.181	6.181	3.836	0.621	2.627826
Inpp5d	Inpp5d-005	1052.5	0.024636	0.056	6.178	6.178	6.013	0.973	2.62711
Mtcl1	Mtcl1-001	940.4	0.043421	0.088	6.175	6.175	9.170	1.485	2.626377
Pbx1	Pbx1-005	522.2	0.007244	0.021	6.168	6.168	1.866	0.303	2.624741
Phactr1	Phactr1-005	2615.2	0.004225	0.014	6.162	6.162	23.661	3.840	2.62335
Rpl26	Rpl26-004	2906.7	0.000217	0.001	6.154	6.154	26.747	4.346	2.621551
Golga5	Golga5-202	2910.5	0.001104	0.005	6.153	6.153	15.613	2.537	2.621343
Fam221b	Fam221b-002	4392.9	0.000001	0.000	6.147	6.147	30.171	4.908	2.619797
Clec3b	Clec3b-201	1176.0	0.001787	0.007	6.142	6.142	7.442	1.212	2.61861
Osbpl1a	Osbpl1a-005	247.7	0.008723	0.025	6.131	6.131	1.134	0.185	2.616097
Efnb2	Efnb2-002	1890.0	0.000565	0.003	6.116	6.116	9.771	1.598	2.612537
Srrt	Srrt-018	2374.5	0.000004	0.000	6.110	6.110	19.300	3.159	2.611109
Arhgap21	Arhgap21-00	2979.9	0.001848	0.007	6.106	6.106	27.395	4.487	2.610216
Gm8894	Gm8894-201	5645.0	0.000001	0.000	6.106	6.106	40.691	6.664	2.610172
Stxbp1	Stxbp1-001	4504.8	0.000081	0.001	6.105	6.105	28.993	4.749	2.610075
Trim28	Trim28-002	562.8	0.013792	0.035	6.097	6.097	5.116	0.839	2.608108
Tom1l2	Tom1l2-201	693.6	0.017710	0.043	6.091	6.091	2.958	0.486	2.606652
Fbxw2	Fbxw2-002	1309.7	0.004101	0.014	6.078	6.078	9.249	1.522	2.603649
Cab39	Cab39-002	1603.0	0.000897	0.004	6.078	6.078	10.723	1.764	2.603623
Rpl38	Rpl38-001	1159.1	0.009536	0.027	6.067	6.067	12.005	1.979	2.601047
Kirrel	Kirrel-002	1813.7	0.005435	0.017	6.067	6.067	10.711	1.766	2.600985
Myh11	Myh11-201	10420.0	0.000069	0.001	6.065	6.065	63.744	10.511	2.600411
Atp1b1	Atp1b1-004	748.9	0.004557	0.015	6.057	6.057	2.647	0.437	2.598586
Specc1l	Specc1l-202	8829.4	0.000001	0.000	6.057	6.057	69.141	11.416	2.598521
Ece1	Ece1-004	301.0	0.002595	0.010	6.046	6.046	3.165	0.523	2.596064
Slc39a3	Slc39a3-002	874.0	0.004631	0.015	6.043	6.043	3.795	0.628	2.59534
Gm10698	Gm10698-001	3436.0	0.000003	0.000	6.039	6.039	25.586	4.237	2.594225
Tlk2	Tlk2-002	1918.2	0.000018	0.000	6.032	6.032	15.012	2.489	2.592655

Cxcl12	Cxcl12-001	2420.7	0.024679	0.056	6.027	6.027	31.142	5.167	2.591441
Ppp2r3d	Ppp2r3d-003	298.0	0.010439	0.029	6.016	6.016	2.089	0.347	2.588885
Clasp2	Clasp2-202	1587.1	0.001822	0.007	6.016	6.016	10.638	1.768	2.588712
Cbfa2t3	Cbfa2t3-003	3742.5	0.000008	0.000	6.013	6.013	22.548	3.750	2.587967
Lrrcc1	Lrrcc1-002	888.3	0.015065	0.038	6.009	6.009	7.193	1.197	2.587209
Paqr8	Paqr8-002	745.2	0.044835	0.090	6.009	6.009	5.031	0.837	2.58704
Rps3	Rps3-002	6719.5	0.000000	0.000	6.000	6.000	58.351	9.726	2.5849
Tnrc6c	Tnrc6c-001	13580.3	0.000013	0.000	5.999	5.999	76.658	12.778	2.584811
Rnf31	Rnf31-001	2593.4	0.000075	0.001	5.999	5.999	19.268	3.212	2.584797
Kmt2b	Kmt2b-001	1125.1	0.004590	0.015	5.997	5.997	11.033	1.840	2.584342
Cracr2b	Cracr2b-001	4717.0	0.000001	0.000	5.993	5.993	32.225	5.377	2.583345
St8sia1	St8sia1-001	1418.8	0.005439	0.017	5.991	5.991	13.257	2.213	2.582682
Ndel1	Ndel1-002	1022.0	0.001859	0.007	5.986	5.986	4.979	0.832	2.581487
Kalrn	Kalrn-008	201.7	0.025649	0.058	5.985	5.985	1.320	0.221	2.581351
Atp6v1f	Atp6v1f-001	4318.4	0.000092	0.001	5.984	5.984	33.418	5.585	2.581044
Cul9	Cul9-001	2377.7	0.000236	0.002	5.982	5.982	16.600	2.775	2.580586
Cdc25b	Cdc25b-003	256.5	0.013594	0.035	5.981	5.981	2.257	0.377	2.580422
Vipr1	Vipr1-001	3107.4	0.000153	0.001	5.959	5.959	9.706	1.629	2.575024
Raly	Raly-002	2721.6	0.019070	0.046	5.952	5.952	18.377	3.088	2.573334
Sumo2	Sumo2-001	6108.2	0.000278	0.002	5.949	5.949	51.127	8.593	2.572767
Smg1	Smg1-005	10983.3	0.000026	0.000	5.946	5.946	83.921	14.115	2.571808
Wnk4	Wnk4-001	5002.8	0.030299	0.066	5.931	5.931	22.474	3.789	2.568219
Apbb2	Apbb2-001	8772.3	0.000001	0.000	5.930	5.930	55.470	9.354	2.568099
Entpd5	Entpd5-001	1487.8	0.000193	0.001	5.930	5.930	7.961	1.343	2.56799
Mprip	Mprip-003	12064.9	0.005787	0.018	5.930	5.930	107.847	18.188	2.567914
Aebp2	Aebp2-002	2223.4	0.000170	0.001	5.929	5.929	16.757	2.826	2.567756
Leng8	Leng8-011	650.4	0.007510	0.022	5.919	5.919	6.415	1.084	2.56533
Tns1	Tns1-011	4087.5	0.000001	0.000	5.904	5.904	24.970	4.229	2.561651
Skp1a	Skp1a-003	2670.5	0.000180	0.001	5.903	5.903	22.945	3.887	2.561502
Plekhg5	Plekhg5-001	4851.5	0.005756	0.018	5.901	5.901	36.551	6.194	2.560926
Stau1	Stau1-004	1374.7	0.035165	0.074	5.895	5.895	10.689	1.813	2.559547
Rnf44	Rnf44-001	1295.3	0.014186	0.036	5.889	5.889	11.166	1.896	2.55809
Atp9a	Atp9a-201	886.7	0.000435	0.002	5.885	5.885	4.751	0.807	2.557005
Gm28035	Gm28035-001	1212.0	0.003362	0.012	5.876	5.876	11.297	1.922	2.554899
Krt7	Krt7-004	330.6	0.003080	0.011	5.870	5.870	2.447	0.417	2.55332
Cntrl	Cntrl-202	1928.4	0.027849	0.062	5.869	5.869	7.311	1.246	2.553128
Arih2	Arih2-009	1243.3	0.002525	0.009	5.868	5.868	9.186	1.566	2.552746
Kdsr	Kdsr-002	852.8	0.000141	0.001	5.862	5.862	6.535	1.115	2.551367
P24-176O2	24-176O2.2-(	317.0	0.005561	0.017	5.854	5.854	2.344	0.400	2.549483
Evc2	Evc2-003	123.0	0.032219	0.069	5.854	5.854	0.937	0.160	2.549304
Zfp652	Zfp652-201	2190.9	0.027789	0.061	5.837	5.837	20.428	3.500	2.545188
Pwp1	Pwp1-002	313.3	0.011726	0.031	5.837	5.837	2.997	0.513	2.545164
Usp48	Usp48-003	1796.5	0.009533	0.027	5.835	5.835	12.426	2.130	2.544714
Usf1	Usf1-201	934.0	0.009005	0.025	5.832	5.832	7.673	1.316	2.544046
Kdm4b	Kdm4b-001	2004.8	0.002630	0.010	5.830	5.830	13.076	2.243	2.543389
Creld1	Creld1-001	1879.4	0.002989	0.011	5.828	5.828	10.910	1.872	2.54305
Gm37486	Gm37486-001	111.0	0.014050	0.036	5.825	5.825	0.775	0.133	2.542268
Pex5	Pex5-002	578.7	0.029216	0.064	5.824	5.824	6.470	1.111	2.541929
Ufc1	Ufc1-004	341.1	0.049646	0.097	5.816	5.816	2.587	0.445	2.540137
Mt1	Mt1-201	12892.0	0.003194	0.011	5.811	5.811	115.634	19.898	2.538873
Btbd10	Btbd10-002	454.7	0.007343	0.022	5.806	5.806	2.750	0.474	2.537478
Gm16142	Gm16142-001	191.0	0.008864	0.025	5.805	5.805	1.245	0.214	2.537331
Cpsf6	Cpsf6-002	2153.4	0.008452	0.024	5.801	5.801	16.127	2.780	2.536415
Ppp1r18	Ppp1r18-004	4444.1	0.001108	0.005	5.794	5.794	14.367	2.479	2.534676
Eif4g1	Eif4g1-004	5152.5	0.000311	0.002	5.794	5.794	41.686	7.195	2.534572
Pltp	Pltp-001	2246.0	0.029192	0.064	5.793	5.793	13.307	2.297	2.534344
Sik3	Sik3-005	4069.3	0.006125	0.019	5.793	5.793	23.968	4.138	2.534212
Syp	Syp-001	348.5	0.025906	0.058	5.791	5.791	1.633	0.282	2.533756
Pfdn2	Pfdn2-003	1835.5	0.000145	0.001	5.788	5.788	15.339	2.650	2.532958
Srsf5	Srsf5-008	2099.7	0.000026	0.000	5.785	5.785	16.590	2.868	2.532194
Ceacam2	Ceacam2-001	547.2	0.005057	0.016	5.784	5.784	3.042	0.526	2.532034
Sertad4	Sertad4-001	4196.6	0.000002	0.000	5.774	5.774	30.944	5.360	2.529488
Adh5	Adh5-007	606.2	0.039786	0.082	5.772	5.772	5.015	0.869	2.529058
Frrs1l	Frrs1l-001	1173.0	0.000390	0.002	5.767	5.767	9.428	1.635	2.527918
Odf2	Odf2-008	2209.4	0.002654	0.010	5.767	5.767	16.122	2.796	2.527792
00020D05R	0020D05Rik-	244.0	0.006094	0.019	5.767	5.767	2.138	0.371	2.527777
Grm1	Grm1-001	387.8	0.002998	0.011	5.765	5.765	2.495	0.433	2.52724
Rpl5	Rpl5-003	1918.1	0.000166	0.001	5.757	5.757	17.144	2.978	2.525241
Bcas3	Bcas3-002	1091.9	0.048602	0.096	5.754	5.754	12.324	2.142	2.524632
Polr2b	Polr2b-002	309.8	0.004520	0.015	5.750	5.750	2.626	0.457	2.523621
Vps72	Vps72-001	2398.3	0.000065	0.001	5.749	5.749	17.168	2.986	2.523297
Pdap1	Pdap1-002	822.6	0.001694	0.007	5.746	5.746	6.108	1.063	2.522441
Gm13152	Gm13152-201	1100.2	0.003830	0.013	5.741	5.741	6.656	1.159	2.521247
Fam219b	Fam219b-202	351.5	0.036465	0.076	5.739	5.739	3.852	0.671	2.520872
Ptgs1	Ptgs1-005	1027.9	0.000341	0.002	5.737	5.737	4.261	0.743	2.520308
Cdan1	Cdan1-001	305.4	0.048422	0.096	5.737	5.737	2.499	0.436	2.520227
Ankrd17	Ankrd17-001	2488.2	0.006660	0.020	5.733	5.733	15.288	2.667	2.519255
Gm15185	Gm15185-001	317.0	0.008819	0.025	5.732	5.732	2.406	0.420	2.51905
Arid1a	Arid1a-002	5491.4	0.027722	0.061	5.728	5.728	40.289	7.034	2.517952
Btnl10	Btnl10-003	253.7	0.007169	0.021	5.724	5.724	1.808	0.316	2.517127
Synj2	Synj2-006	853.5	0.010253	0.028	5.724	5.724	5.769	1.008	2.517042
30403L08R	0403L08Rik-	714.9	0.000187	0.001	5.724	5.724	4.338	0.758	2.516979
Ubfd1	Ubfd1-005	237.9	0.003516	0.012	5.720	5.720	1.907	0.333	2.515982
Btbd2	Btbd2-003	448.6	0.035881	0.075	5.716	5.716	3.968	0.694	2.515031
Dcaf8	Dcaf8-005	3111.7	0.014448	0.037	5.716	5.716	23.979	4.195	2.514884
Psma7	Psma7-002	241.2	0.041790	0.085	5.709	5.709	2.030	0.355	2.513308
Crebl2	Crebl2-002	189.6	0.037718	0.078	5.702	5.702	1.669	0.293	2.511498
Macf1	Macf1-005	1773.0	0.000586	0.003	5.702	5.702	10.101	1.771	2.511478
Ctgf	Ctgf-001	61815.4	0.001427	0.006	5.695	5.695	885.177	155.423	2.509763
Lppr4	Lppr4-001	1489.0	0.000119	0.001	5.674	5.674	10.249	1.806	2.504276
Lsr	Lsr-002	3205.8	0.018449	0.045	5.659	5.659	18.077	3.194	2.500605
Chmp1a	Chmp1a-002	202.8	0.035365	0.075	5.658	5.658	1.799	0.318	2.500166
L3mbtl2	L3mbtl2-002	525.9	0.047387	0.094	5.654	5.654	3.555	0.629	2.499367
Gm15501	Gm15501-002	7641.1	0.000002	0.000	5.651	5.651	68.173	12.063	2.498623
Gigyf2	Gigyf2-024	153.0	0.024291	0.055	5.651	5.651	0.892	0.158	2.498621
Ktn1	Ktn1-011	1573.1	0.000955	0.004	5.648	5.648	12.161	2.153	2.497622
Exd1	Exd1-201	1284.9	0.000027	0.000	5.647	5.647	9.066	1.606	2.49744
Vars	Vars-002	2391.5	0.000217	0.001	5.646	5.646	23.523	4.166	2.497196
Ubr3	Ubr3-004	354.6	0.031385	0.068	5.633	5.633	2.379	0.422	2.493858
Hps5	Hps5-001	813.4	0.028487	0.063	5.628	5.628	5.909	1.050	2.49269
Ptpn14	Ptpn14-002	13749.3	0.001090	0.005	5.627	5.627	97.749	17.371	2.492361
Hid1	Hid1-005	505.8	0.006426	0.019	5.627	5.627	3.243	0.576	2.492297
30577N17R	0577N17Rik-	366.6	0.005712	0.018	5.620	5.620	2.196	0.391	2.490555
Pdgfra	Pdgfra-006	1790.7	0.000016	0.000	5.619	5.619	15.218	2.708	2.490404
Hspb7	Hspb7-001	1840.0	0.000157	0.001	5.618	5.618	13.372	2.380	2.489959
Fgf9	Fgf9-003	2932.1	0.000001	0.000	5.617	5.617	19.509	3.474	2.489689
Zfpm1	Zfpm1-001	9378.9	0.000000	0.000	5.616	5.616	48.007	8.548	2.489498

Ndfip2	Ndfip2-003	462.1	0.032658	0.070	5.615	5.615	4.163	0.741	2.489365
Rnpepl1	Rnpepl1-001	1136.0	0.009745	0.027	5.609	5.609	6.599	1.177	2.487646
Prkag3	Prkag3-004	1139.0	0.000115	0.001	5.606	5.606	8.508	1.518	2.486857
Prkag3	Prkag3-201	1139.0	0.000115	0.001	5.606	5.606	8.508	1.518	2.486857
Atp2a3	Atp2a3-201	36566.8	0.000021	0.000	5.605	5.605	145.021	25.873	2.486741
Kalrn	Kalrn-011	2488.3	0.009128	0.026	5.605	5.605	14.721	2.627	2.486678
Acly	Acly-001	2508.1	0.000423	0.002	5.599	5.599	20.901	3.733	2.485251
Bscl2	Bscl2-201	1076.0	0.000841	0.004	5.596	5.596	8.346	1.491	2.484385
Yipf1	Yipf1-002	1895.6	0.000704	0.003	5.590	5.590	18.574	3.323	2.482916
Abi2	Abi2-007	324.8	0.020122	0.048	5.576	5.576	1.718	0.308	2.479113
Stk11	Stk11-001	5593.5	0.000489	0.003	5.566	5.566	45.688	8.209	2.476567
Psen2	Psen2-007	453.1	0.002470	0.009	5.564	5.564	4.810	0.865	2.476026
Patz1	Patz1-012	913.4	0.044870	0.090	5.559	5.559	7.737	1.392	2.474865
Serping1	Serping1-003	999.8	0.000624	0.003	5.557	5.557	8.913	1.604	2.474388
Brd2	Brd2-009	309.2	0.006076	0.019	5.557	5.557	2.189	0.394	2.474306
Raly	Raly-001	3114.1	0.000452	0.002	5.556	5.556	23.343	4.201	2.474153
Pdia6	Pdia6-002	1438.4	0.003652	0.013	5.556	5.556	10.835	1.950	2.473972
Eml1	Eml1-002	372.1	0.027620	0.061	5.553	5.553	2.775	0.500	2.473373
Qsox1	Qsox1-002	8975.6	0.000006	0.000	5.553	5.553	43.185	7.778	2.473147
Gm11836	Gm11836-001	358.0	0.003366	0.012	5.549	5.549	2.473	0.446	2.472241
Hist2h2ab	Hist2h2ab-001	231.3	0.014176	0.036	5.545	5.545	1.912	0.345	2.471241
P23-323D23	23-323D23.1-	355.0	0.001058	0.005	5.542	5.542	2.484	0.448	2.470513
00076A07R	0076A07Rik-	263.3	0.005646	0.018	5.529	5.529	1.823	0.330	2.466962
Nfxl1	Nfxl1-003	401.7	0.022679	0.052	5.526	5.526	2.603	0.471	2.466138
Calr	Calr-004	21296.1	0.001404	0.006	5.524	5.524	151.324	27.392	2.46582
Psen1	Psen1-201	2756.6	0.002344	0.009	5.509	5.509	18.954	3.441	2.461733
Tnks1bp1	Tnks1bp1-003	7549.8	0.000087	0.001	5.502	5.502	44.092	8.014	2.459936
Socs6	Socs6-003	826.6	0.017111	0.042	5.499	5.499	5.471	0.995	2.459243
Eif2ak4	Eif2ak4-001	2285.0	0.005144	0.016	5.497	5.497	17.141	3.118	2.458687
Ick	Ick-004	2079.0	0.001417	0.006	5.491	5.491	13.784	2.510	2.457056
Mier3	Mier3-004	742.8	0.044839	0.090	5.483	5.483	4.707	0.858	2.455021
Setdb1	Setdb1-001	1538.3	0.003465	0.012	5.480	5.480	11.525	2.103	2.454292
Cdc27	Cdc27-002	1124.2	0.012222	0.032	5.468	5.468	7.944	1.453	2.451124
Safb	Safb-006	5368.8	0.000077	0.001	5.459	5.459	39.957	7.319	2.448635
Rapgefl1	Rapgefl1-002	306.9	0.006370	0.019	5.454	5.454	2.291	0.420	2.447229
Ddx54	Ddx54-001	3537.3	0.000019	0.000	5.448	5.448	31.984	5.871	2.44573
Micu1	Micu1-004	689.3	0.023155	0.053	5.443	5.443	7.535	1.384	2.444427
Endov	Endov-001	390.5	0.004784	0.015	5.441	5.441	3.581	0.658	2.44399
H2-Q5	H2-Q5-001	692.1	0.003822	0.013	5.441	5.441	5.328	0.979	2.443743
10067B10R	0067B10Rik-	1882.2	0.035983	0.076	5.438	5.438	7.429	1.366	2.443157
Gm10157	Gm10157-001	707.0	0.000118	0.001	5.435	5.435	5.309	0.977	2.442187
Erbb2	Erbb2-002	2269.2	0.036473	0.076	5.432	5.432	16.118	2.967	2.441544
Gm5814	Gm5814-201	129.0	0.023415	0.054	5.417	5.417	1.142	0.211	2.437456
Cops6	Cops6-004	779.7	0.003252	0.011	5.410	5.410	5.545	1.025	2.435615
Tns2	Tns2-201	8921.4	0.000038	0.000	5.403	5.403	47.275	8.750	2.43366
Wiz	Wiz-001	1024.9	0.005548	0.017	5.400	5.400	7.736	1.432	2.43304
Zfp740	Zfp740-002	2893.9	0.001319	0.006	5.398	5.398	20.482	3.795	2.432351
Pam	Pam-013	480.4	0.001170	0.005	5.388	5.388	3.430	0.637	2.429636
Gm26705	Gm26705-201	1062.0	0.000169	0.001	5.385	5.385	7.767	1.442	2.42891
Osbpl10	Osbpl10-003	417.6	0.038894	0.080	5.379	5.379	2.295	0.427	2.42747
Uqcc2	Uqcc2-001	1439.4	0.001129	0.005	5.375	5.375	10.432	1.941	2.426251
Smad4	Smad4-002	2576.5	0.017244	0.042	5.372	5.372	23.628	4.398	2.42552
Itgb6	Itgb6-002	275.0	0.007005	0.021	5.370	5.370	1.720	0.320	2.425028
Dab2ip	Dab2ip-011	2786.6	0.001101	0.005	5.364	5.364	17.568	3.275	2.423399
Syn2	Syn2-201	397.7	0.005003	0.016	5.361	5.361	1.353	0.252	2.422592
Gpr37	Gpr37-002	1812.0	0.000016	0.000	5.358	5.358	12.655	2.362	2.421564
Tpm1	Tpm1-012	1328.4	0.025746	0.058	5.355	5.355	13.324	2.488	2.421012
Igfbp6	Igfbp6-001	740.0	0.006777	0.020	5.353	5.353	4.546	0.849	2.42043
Syncrip	Syncrip-011	281.5	0.020635	0.049	5.350	5.350	1.859	0.347	2.41944
Auts2	Auts2-007	2194.5	0.004010	0.013	5.349	5.349	18.466	3.452	2.419207
Casp12	Casp12-004	1575.1	0.001049	0.005	5.342	5.342	14.388	2.693	2.417472
Vwf	Vwf-003	2385.5	0.000209	0.001	5.341	5.341	20.279	3.797	2.417101
Mars	Mars-201	160.2	0.029329	0.064	5.337	5.337	2.235	0.419	2.415979
Nrarp	Nrarp-001	3170.2	0.001586	0.007	5.332	5.332	16.441	3.084	2.414616
Adgrl3	Adgrl3-027	445.7	0.007678	0.022	5.331	5.331	3.471	0.651	2.414513
Tanc2	Tanc2-006	915.0	0.001928	0.008	5.330	5.330	5.829	1.094	2.414216
Pfn2	Pfn2-001	349.8	0.009284	0.026	5.322	5.322	3.122	0.587	2.41184
Acer3	Acer3-002	507.8	0.006149	0.019	5.321	5.321	3.413	0.641	2.411819
Flna	Flna-002	32604.4	0.000009	0.000	5.318	5.318	284.657	53.527	2.410884
Ehd4	Ehd4-002	156.4	0.044277	0.089	5.318	5.318	0.921	0.173	2.410809
Lmo7	Lmo7-006	9477.3	0.000019	0.000	5.312	5.312	61.656	11.607	2.409289
C1qtnf1	C1qtnf1-003	1006.8	0.000129	0.001	5.310	5.310	8.245	1.553	2.408728
Cdyl	Cdyl-202	1446.6	0.011823	0.031	5.291	5.291	8.918	1.685	2.403593
00014E21R	R0014E21Rik-	322.0	0.001195	0.005	5.290	5.290	2.387	0.451	2.403242
Taok3	Taok3-002	4715.1	0.000842	0.004	5.286	5.286	32.382	6.126	2.402258
Gnai2	Gnai2-003	19806.8	0.000000	0.000	5.285	5.285	113.035	21.387	2.401956
Pigr	Pigr-001	1898.0	0.000009	0.000	5.282	5.282	12.700	2.404	2.401074
Phrf1	Phrf1-001	3260.1	0.003420	0.012	5.270	5.270	31.968	6.066	2.397765
Ctbp1	Ctbp1-001	3055.7	0.000903	0.004	5.269	5.269	23.752	4.508	2.397597
Cbx7	Cbx7-007	2424.6	0.003252	0.011	5.269	5.269	18.152	3.445	2.397504
Golim4	Golim4-005	513.8	0.006169	0.019	5.268	5.268	4.086	0.776	2.397256
Hmgb1	Hmgb1-004	5894.8	0.000174	0.001	5.267	5.267	50.789	9.644	2.396852
P23-257P11	23-257P11.1-	226.0	0.007877	0.023	5.265	5.265	1.945	0.369	2.396378
Brsk2	Brsk2-007	537.2	0.033887	0.072	5.263	5.263	2.874	0.546	2.395997
Srgn	Srgn-004	7444.8	0.000276	0.002	5.263	5.263	27.572	5.239	2.395921
Dot1l	Dot1l-005	428.6	0.012351	0.033	5.262	5.262	4.018	0.764	2.395648
Ccdc124	Ccdc124-201	4440.0	0.000100	0.001	5.248	5.248	32.133	6.123	2.391744
Dek	Dek-005	1217.8	0.019986	0.047	5.247	5.247	7.874	1.501	2.391571
Procr	Procr-003	731.9	0.002278	0.009	5.244	5.244	6.005	1.145	2.390788
Hsp90ab1	Hsp90ab1-005	1705.9	0.000017	0.000	5.243	5.243	13.054	2.490	2.390484
Fgfr1	Fgfr1-002	3082.8	0.024828	0.056	5.238	5.238	17.349	3.312	2.388981
Pcmt1	Pcmt1-004	681.4	0.007702	0.022	5.232	5.232	6.762	1.292	2.387489
Mcfd2	Mcfd2-002	3406.3	0.000237	0.002	5.231	5.231	11.395	2.178	2.387063
Ndufv2	Ndufv2-001	1192.6	0.000150	0.001	5.225	5.225	11.350	2.172	2.385429
Usp45	Usp45-004	354.0	0.036094	0.076	5.223	5.223	2.826	0.541	2.384936
Rrbp1	Rrbp1-006	273.1	0.002569	0.010	5.219	5.219	2.112	0.405	2.383763
Oxsr1	Oxsr1-002	500.4	0.005552	0.017	5.215	5.215	3.034	0.582	2.382795
Ptprs	Ptprs-001	2775.3	0.000086	0.001	5.204	5.204	20.050	3.853	2.379538
Mex3d	Mex3d-001	2127.0	0.000062	0.001	5.193	5.193	18.027	3.471	2.376661
Ncam1	Ncam1-005	2821.4	0.000444	0.002	5.193	5.193	10.683	2.057	2.37645
Ptch1	Ptch1-001	2750.1	0.007112	0.021	5.193	5.193	19.697	3.793	2.376433
Hdac2	Hdac2-004	560.3	0.011558	0.031	5.191	5.191	3.014	0.581	2.375918
Mrps26	Mrps26-002	1167.1	0.012707	0.033	5.183	5.183	8.557	1.651	2.373709
Smpd4	Smpd4-003	512.4	0.018237	0.044	5.181	5.181	4.878	0.941	2.37325
Rgl2	Rgl2-008	349.5	0.008167	0.023	5.181	5.181	2.850	0.550	2.373154
Sirt7	Sirt7-001	465.4	0.049774	0.098	5.176	5.176	3.807	0.736	2.371813
P24-285A14	24-285A14.2-	813.0	0.000231	0.001	5.176	5.176	5.764	1.114	2.371728

Zfp691	Zfp691-002	560.9	0.022535	0.052	5.173	5.173	3.374	0.652	2.37103
Phrf1	Phrf1-003	1776.8	0.002144	0.008	5.170	5.170	16.129	3.119	2.370295
Sel1l	Sel1l-005	1534.2	0.001301	0.006	5.168	5.168	10.175	1.969	2.369602
P23-17D18	23-17D18.3-(	137.0	0.009478	0.026	5.167	5.167	1.081	0.209	2.369206
Rps4x-ps	Rps4x-ps-001	310.0	0.002466	0.009	5.165	5.165	2.740	0.531	2.368636
Mier2	Mier2-001	737.2	0.012922	0.034	5.159	5.159	6.644	1.288	2.367068
Aven	Aven-003	483.1	0.042562	0.086	5.157	5.157	2.353	0.456	2.366537
Ppp1r37	Ppp1r37-201	8797.0	0.000000	0.000	5.151	5.151	58.810	11.418	2.364783
Ceacam20	Ceacam20-20	308.0	0.010659	0.029	5.147	5.147	2.338	0.454	2.363655
Phf21a	Phf21a-002	584.4	0.008493	0.024	5.146	5.146	5.452	1.059	2.36345
Arhgef2	Arhgef2-028	211.8	0.018487	0.045	5.136	5.136	1.072	0.209	2.360765
Blvrb	Blvrb-001	1087.2	0.003118	0.011	5.127	5.127	8.056	1.571	2.358103
Rpl41	Rpl41-001	885.7	0.011093	0.030	5.125	5.125	7.086	1.383	2.35764
Rilpl2	Rilpl2-002	2315.0	0.000010	0.000	5.125	5.125	16.092	3.140	2.357436
Acvrl1	Acvrl1-003	4739.8	0.012362	0.033	5.117	5.117	22.168	4.332	2.355308
Tbkbp1	Tbkbp1-005	3651.1	0.000070	0.001	5.114	5.114	23.965	4.686	2.354378
Lpar1	Lpar1-001	896.1	0.024201	0.055	5.110	5.110	10.152	1.987	2.353373
Gmcl1	Gmcl1-003	458.3	0.004503	0.015	5.106	5.106	4.195	0.822	2.352173
Ssbp4	Ssbp4-201	3808.0	0.000001	0.000	5.093	5.093	27.843	5.467	2.348454
Crip2	Crip2-004	297.4	0.014993	0.038	5.089	5.089	1.682	0.331	2.347269
Mgat4b	Mgat4b-001	3117.0	0.000212	0.001	5.086	5.086	24.354	4.788	2.346632
Scand1	Scand1-201	923.9	0.006522	0.020	5.086	5.086	6.722	1.322	2.346398
Ica1	Ica1-006	1016.8	0.004623	0.015	5.074	5.074	7.071	1.394	2.343018
Fut8	Fut8-201	709.3	0.041554	0.085	5.065	5.065	5.214	1.029	2.340536
Pdgfc	Pdgfc-001	403.9	0.046811	0.093	5.062	5.062	1.886	0.373	2.339706
Plec	Plec-201	18744.1	0.000037	0.000	5.059	5.059	84.915	16.785	2.338875
Adcy5	Adcy5-201	10640.0	0.000307	0.002	5.058	5.058	70.869	14.010	2.33869
Eps8l2	Eps8l2-001	842.2	0.005591	0.017	5.058	5.058	5.044	0.997	2.338515
Cyfip1	Cyfip1-005	3912.3	0.000145	0.001	5.056	5.056	26.608	5.263	2.338013
Dnajc10	Dnajc10-004	1224.2	0.006159	0.019	5.056	5.056	6.414	1.269	2.337988
Tead2	Tead2-001	2221.7	0.014481	0.037	5.056	5.056	12.419	2.457	2.337883
Kpnb1	Kpnb1-003	1046.5	0.003476	0.012	5.054	5.054	7.316	1.448	2.337539
Gm5526	Gm5526-001	4641.0	0.000003	0.000	5.050	5.050	32.834	6.502	2.336312
Tia1	Tia1-003	273.4	0.026070	0.058	5.044	5.044	3.708	0.735	2.334526
Acbd4	Acbd4-002	1222.8	0.001061	0.005	5.041	5.041	9.588	1.902	2.333598
Tmsb4x	Tmsb4x-003	1573.0	0.000271	0.002	5.040	5.040	14.585	2.894	2.333284
Nmt2	Nmt2-002	5408.0	0.001058	0.005	5.039	5.039	39.674	7.873	2.333128
E4f1	E4f1-201	3031.0	0.000001	0.000	5.038	5.038	20.582	4.085	2.332879
Gm3033	Gm3033-001	427.0	0.002175	0.008	5.037	5.037	2.927	0.581	2.332687
Ube4b	Ube4b-011	651.4	0.008997	0.025	5.036	5.036	5.252	1.043	2.332147
Pick1	Pick1-006	2672.2	0.008914	0.025	5.028	5.028	22.600	4.495	2.330065
Gm8325	Gm8325-002	654.5	0.000311	0.002	5.027	5.027	4.440	0.883	2.329739
Tor1aip2	Tor1aip2-009	763.5	0.023727	0.054	5.026	5.026	7.293	1.451	2.329466
Eef1b2	Eef1b2-008	1621.9	0.012213	0.032	5.025	5.025	11.928	2.374	2.329055
Rprd1b	Rprd1b-008	1455.9	0.000069	0.001	5.023	5.023	7.575	1.508	2.328688
Cox4i1	Cox4i1-007	1708.4	0.000189	0.001	5.023	5.023	14.117	2.811	2.328546
Syne3	Syne3-202	2525.9	0.000701	0.003	5.017	5.017	15.353	3.060	2.3268
Per1	Per1-002	2602.6	0.044314	0.089	5.016	5.016	14.692	2.929	2.32647
Nr3c1	Nr3c1-003	567.9	0.011581	0.031	5.011	5.011	4.160	0.830	2.325146
Tap1	Tap1-001	757.4	0.011301	0.030	5.010	5.010	5.545	1.107	2.324787
Ciz1	Ciz1-012	2029.2	0.000090	0.001	5.000	5.000	15.543	3.109	2.321882
Txnip	Txnip-004	823.4	0.000700	0.003	4.990	4.990	5.313	1.065	2.319023
Phf3	Phf3-010	7039.5	0.001629	0.007	4.987	4.987	52.054	10.439	2.318094
Med25	Med25-201	4183.0	0.000009	0.000	4.985	4.985	35.187	7.059	2.317516
Fbxl17	Fbxl17-001	3596.8	0.000112	0.001	4.978	4.978	23.415	4.704	2.31555
H2-K1	H2-K1-008	841.7	0.003213	0.011	4.977	4.977	5.499	1.105	2.31518
Tcirg1	Tcirg1-002	649.7	0.039653	0.082	4.976	4.976	6.956	1.398	2.315095
Iffo2	Iffo2-002	2965.5	0.000638	0.003	4.973	4.973	21.433	4.310	2.314232
Chkb	Chkb-002	318.0	0.043548	0.088	4.969	4.969	1.809	0.364	2.313014
Lmo7	Lmo7-014	1642.4	0.000324	0.002	4.968	4.968	11.813	2.378	2.312611
Mrps12	Mrps12-001	583.3	0.008349	0.024	4.967	4.967	5.931	1.194	2.312232
Dmtf1	Dmtf1-009	467.1	0.031417	0.068	4.966	4.966	4.083	0.822	2.312153
Nkd1	Nkd1-201	694.5	0.017089	0.042	4.962	4.962	3.904	0.787	2.311012
Evi5	Evi5-013	947.8	0.000897	0.004	4.960	4.960	7.002	1.412	2.310341
Mecom	Mecom-012	2676.3	0.003842	0.013	4.955	4.955	19.181	3.871	2.308864
Cebpd	Cebpd-201	6224.0	0.000150	0.001	4.951	4.951	61.299	12.380	2.307847
Stat1	Stat1-009	1118.2	0.020023	0.048	4.948	4.948	6.918	1.398	2.306795
Klf15	Klf15-001	1805.7	0.012618	0.033	4.946	4.946	23.054	4.661	2.306343
Dusp1	Dusp1-001	2108.1	0.002995	0.011	4.945	4.945	9.614	1.944	2.306085
Aamp	Aamp-001	5333.9	0.000010	0.000	4.944	4.944	39.785	8.047	2.305703
Zfp777	Zfp777-201	2528.9	0.000024	0.000	4.943	4.943	17.274	3.495	2.305297
Gm11737	Gm11737-001	412.0	0.003790	0.013	4.940	4.940	2.224	0.450	2.304453
Mum1	Mum1-001	1629.5	0.007462	0.022	4.937	4.937	13.136	2.660	2.303748
Rhobtb1	Rhobtb1-201	1467.6	0.013423	0.035	4.937	4.937	9.378	1.900	2.303646
Fam129a	Fam129a-002	3286.8	0.000322	0.002	4.931	4.931	20.457	4.148	2.301992
Micu1	Micu1-005	724.4	0.016766	0.041	4.931	4.931	5.520	1.119	2.301985
Zfyve27	Zfyve27-007	159.8	0.040915	0.084	4.931	4.931	1.105	0.224	2.301873
Cnih1	Cnih1-002	536.5	0.041581	0.085	4.927	4.927	4.739	0.962	2.300761
Rnf10	Rnf10-001	9341.4	0.000045	0.000	4.927	4.927	67.787	13.760	2.300579
Mdm4	Mdm4-002	918.5	0.009864	0.027	4.925	4.925	7.071	1.436	2.300107
Zfp157	Zfp157-004	213.8	0.013201	0.034	4.923	4.923	1.337	0.272	2.299395
Hbb-bs	Hbb-bs-001	5287.0	0.004128	0.014	4.919	4.919	68.467	13.918	2.298496
Tbc1d14	Tbc1d14-003	530.2	0.034051	0.072	4.916	4.916	4.681	0.952	2.297588
Gm21798	Gm21798-201	390.0	0.001780	0.007	4.906	4.906	2.604	0.531	2.294425
Wtap	Wtap-007	1011.7	0.000420	0.002	4.893	4.893	8.316	1.700	2.290574
St8sia3	St8sia3-001	251.0	0.005320	0.017	4.891	4.891	2.072	0.424	2.290104
P23-245J24	23-245J24.1-(	1511.0	0.000005	0.000	4.881	4.881	9.809	2.010	2.287122
Nol3	Nol3-201	1361.4	0.000045	0.000	4.879	4.879	11.756	2.410	2.286528
Reln	Reln-006	203.4	0.010533	0.029	4.874	4.874	1.423	0.292	2.285235
Mink1	Mink1-005	6993.0	0.000352	0.002	4.873	4.873	42.985	8.821	2.284795
Fgfr3	Fgfr3-008	1242.1	0.020512	0.048	4.871	4.871	3.051	0.626	2.284248
Lrp3	Lrp3-001	1730.3	0.003658	0.013	4.867	4.867	13.168	2.706	2.283012
Dvl3	Dvl3-001	785.1	0.002115	0.008	4.860	4.860	7.594	1.563	2.280995
Banp	Banp-003	242.8	0.047389	0.094	4.854	4.854	2.876	0.592	2.279294
D10Jhu81e	D10Jhu81e-20	1466.0	0.000064	0.001	4.852	4.852	11.063	2.280	2.27856
Ptprj	Ptprj-201	64538.6	0.000122	0.001	4.848	4.848	207.797	42.865	2.277312
Cd82	Cd82-002	2066.1	0.000355	0.002	4.846	4.846	18.067	3.728	2.276922
30046B11R	0046B11Rik-	886.0	0.000580	0.003	4.846	4.846	6.357	1.312	2.27689
Chmp4b	Chmp4b-001	9449.6	0.000008	0.000	4.845	4.845	75.585	15.600	2.276566
Rbm3	Rbm3-009	1530.3	0.000085	0.001	4.843	4.843	12.063	2.491	2.275859
Zpr1	Zpr1-003	992.1	0.002805	0.010	4.842	4.842	8.896	1.837	2.275658
Gm15807	Gm15807-001	1190.0	0.000051	0.000	4.837	4.837	6.677	1.380	2.274244
Ap2a1	Ap2a1-001	315.8	0.037187	0.078	4.837	4.837	1.980	0.409	2.274027
Thoc2	Thoc2-010	1354.1	0.010383	0.028	4.826	4.826	9.165	1.899	2.27077
Sh3bp5l	Sh3bp5l-001	796.7	0.021937	0.051	4.825	4.825	5.529	1.146	2.270429
Plec	Plec-205	15361.6	0.000122	0.001	4.818	4.818	85.585	17.762	2.268577
Cers5	Cers5-004	235.8	0.035368	0.075	4.817	4.817	1.360	0.282	2.268175

Jund	Jund-201	9629.7	0.000000	0.000	4.813	4.813	64.355	13.371	2.266991
Prlr	Prlr-005	153.0	0.020863	0.049	4.812	4.812	1.024	0.213	2.266648
Map4k5	Map4k5-201	1091.8	0.001794	0.007	4.812	4.812	8.774	1.824	2.266527
Pcyt2	Pcyt2-001	775.1	0.000990	0.005	4.811	4.811	7.652	1.591	2.266257
Calcoco1	Calcoco1-001	9706.9	0.000046	0.000	4.808	4.808	74.537	15.502	2.265468
Guk1	Guk1-006	595.9	0.010013	0.028	4.808	4.808	5.327	1.108	2.265327
L1cam	L1cam-001	7666.5	0.000301	0.002	4.805	4.805	24.731	5.147	2.264395
Mrpl2	Mrpl2-001	618.2	0.049912	0.098	4.801	4.801	5.683	1.184	2.263468
Sema3d	Sema3d-001	2133.5	0.000491	0.003	4.801	4.801	11.231	2.339	2.263462
Plscr1	Plscr1-001	945.5	0.006052	0.019	4.798	4.798	8.728	1.819	2.262474
Pde4dip	Pde4dip-006	1553.3	0.001074	0.005	4.796	4.796	11.513	2.400	2.261855
Son	Son-001	7056.5	0.000000	0.000	4.796	4.796	53.887	11.236	2.261827
Wscd1	Wscd1-001	4663.0	0.000221	0.001	4.788	4.788	23.357	4.878	2.259419
Ppp1r12c	Ppp1r12c-011	2107.4	0.000007	0.000	4.783	4.783	10.206	2.134	2.258057
Pqlc3	Pqlc3-001	1679.0	0.001356	0.006	4.779	4.779	6.265	1.311	2.256846
Uvssa	Uvssa-003	574.5	0.003520	0.012	4.778	4.778	4.162	0.871	2.256327
Pik3r2	Pik3r2-001	2082.9	0.000038	0.000	4.776	4.776	15.840	3.317	2.255689
Fbxo34	Fbxo34-201	1143.1	0.024856	0.056	4.773	4.773	3.470	0.727	2.254983
Fbxw11	Fbxw11-004	425.8	0.002253	0.009	4.767	4.767	3.104	0.651	2.253213
Rabep2	Rabep2-002	1491.9	0.000375	0.002	4.767	4.767	7.740	1.624	2.253087
Hs3st1	Hs3st1-004	882.3	0.005418	0.017	4.766	4.766	6.298	1.321	2.252853
Kif1c	Kif1c-003	843.1	0.018390	0.044	4.766	4.766	5.327	1.118	2.252667
Gm6142	Gm6142-001	453.0	0.001642	0.007	4.762	4.762	3.302	0.693	2.251611
Rpl19	Rpl19-002	1224.2	0.000062	0.001	4.762	4.762	9.149	1.921	2.251527
Lrrc45	Lrrc45-004	560.3	0.007381	0.022	4.760	4.760	3.230	0.679	2.251062
Mark2	Mark2-015	244.8	0.027455	0.061	4.756	4.756	0.836	0.176	2.249643
Tug1	Tug1-001	2006.4	0.018754	0.045	4.753	4.753	13.773	2.898	2.248759
Setd3	Setd3-005	1971.9	0.021708	0.051	4.747	4.747	14.159	2.983	2.247166
Pecam1	Pecam1-001	250845.7	0.000003	0.000	4.741	4.741	1473.253	310.777	2.245053
Timm44	Timm44-001	1021.9	0.014090	0.036	4.726	4.726	6.141	1.299	2.240756
Sdf4	Sdf4-003	2327.6	0.042667	0.086	4.721	4.721	15.937	3.376	2.239161
Gm37633	m37633-001	1116.0	0.000121	0.001	4.719	4.719	9.267	1.964	2.238591
Gm10136	m10136-001	4483.0	0.000001	0.000	4.715	4.715	39.081	8.289	2.237204
Gm2244	Gm2244-001	495.0	0.000786	0.004	4.711	4.711	2.874	0.610	2.23611
Bbx	Bbx-009	1222.9	0.007272	0.021	4.710	4.710	7.884	1.674	2.235789
Mrip-ps	Mrip-ps-001	3207.0	0.000008	0.000	4.709	4.709	26.947	5.722	2.235512
Plvap	Plvap-201	20904.0	0.002941	0.011	4.709	4.709	81.765	17.363	2.235487
Pabpn1	Pabpn1-003	2143.1	0.008842	0.025	4.709	4.709	14.988	3.183	2.235315
H36-12I15.	I36-12I15.4-0	1938.0	0.000011	0.000	4.707	4.707	15.800	3.357	2.234796
Gm5803	Gm5803-001	349.0	0.001229	0.005	4.704	4.704	2.722	0.579	2.23381
Tmem104	mem104-00	844.1	0.001045	0.005	4.702	4.702	7.693	1.636	2.23326
Slc46a3	Slc46a3-004	1690.7	0.001118	0.005	4.702	4.702	13.421	2.855	2.233204
Uimc1	Uimc1-003	2381.9	0.000014	0.000	4.696	4.696	20.726	4.413	2.231563
Tpm3	Tpm3-009	316.2	0.014411	0.037	4.695	4.695	2.970	0.633	2.23117
Csf1	Csf1-001	9663.9	0.003128	0.011	4.694	4.694	65.302	13.912	2.230754
Cad	Cad-012	401.3	0.046220	0.092	4.689	4.689	1.934	0.412	2.229362
Itga3	Itga3-001	21739.6	0.000000	0.000	4.687	4.687	164.374	35.071	2.228652
Plcb4	Plcb4-201	11504.2	0.000375	0.002	4.684	4.684	75.399	16.096	2.227836
Pkig	Pkig-002	2392.3	0.001666	0.007	4.684	4.684	14.336	3.061	2.227599
Sec63	Sec63-002	4327.4	0.002930	0.011	4.680	4.680	32.854	7.020	2.226471
Tubg1	Tubg1-002	269.6	0.026841	0.060	4.678	4.678	1.757	0.376	2.225818
Inpp5f	Inpp5f-002	1979.1	0.000293	0.002	4.675	4.675	15.404	3.295	2.224891
Os9	Os9-202	9596.4	0.000016	0.000	4.672	4.672	75.082	16.070	2.224075
Rab3c	Rab3c-201	2896.0	0.000002	0.000	4.671	4.671	16.949	3.629	2.223729
Sqstm1	Sqstm1-001	10040.8	0.000078	0.001	4.669	4.669	78.204	16.751	2.22302
Erbb2ip	Erbb2ip-005	3302.4	0.001720	0.007	4.666	4.666	20.620	4.419	2.222067
Zfp326	Zfp326-004	1620.4	0.000137	0.001	4.662	4.662	9.199	1.973	2.221053
Mapk14	Mapk14-003	704.5	0.003385	0.012	4.661	4.661	3.808	0.817	2.220647
Cpsf6	Cpsf6-005	2520.1	0.000885	0.004	4.638	4.638	20.011	4.315	2.213447
Irf3	Irf3-004	981.8	0.014315	0.037	4.638	4.638	8.272	1.784	2.213364
Apeh	Apeh-002	515.3	0.000928	0.004	4.637	4.637	4.538	0.979	2.213085
Actn4	Actn4-004	3403.4	0.001804	0.007	4.628	4.628	25.393	5.487	2.210289
Suco	Suco-004	206.3	0.010981	0.030	4.627	4.627	1.282	0.277	2.209923
Mink1	Mink1-013	2216.2	0.000574	0.003	4.612	4.612	14.573	3.160	2.205524
Gm3755	Gm3755-001	2525.0	0.000012	0.000	4.611	4.611	14.917	3.235	2.205228
Hp1bp3	Hp1bp3-010	1173.1	0.014994	0.038	4.611	4.611	8.335	1.808	2.205051
Rapgef3	Rapgef3-004	2782.7	0.001942	0.008	4.604	4.604	16.964	3.685	2.202935
Tial1	Tial1-201	733.5	0.034941	0.074	4.604	4.604	2.344	0.509	2.202786
Ttc17	Ttc17-201	3763.5	0.000110	0.001	4.602	4.602	18.612	4.044	2.202343
Prkd2	Prkd2-202	3348.9	0.001037	0.005	4.601	4.601	26.073	5.667	2.202018
Ahdc1	Ahdc1-003	663.7	0.044897	0.090	4.598	4.598	3.230	0.703	2.201002
P24-210N1	24-210N1.3-(	196.0	0.023715	0.054	4.597	4.597	1.454	0.316	2.200542
Aldh3a1	Aldh3a1-002	1476.7	0.004844	0.016	4.591	4.591	9.416	2.051	2.19881
Ogdh	Ogdh-004	2690.6	0.006391	0.019	4.587	4.587	16.887	3.682	2.197411
Ybx1	Ybx1-003	573.1	0.001111	0.005	4.578	4.578	3.412	0.745	2.194636
Phex	Phex-001	815.8	0.001117	0.005	4.576	4.576	5.164	1.128	2.194242
Arl14ep	Arl14ep-004	295.1	0.033218	0.071	4.573	4.573	2.004	0.438	2.193164
Rbck1	Rbck1-001	3931.0	0.003295	0.012	4.572	4.572	28.990	6.341	2.192851
Sulf2	Sulf2-003	430.5	0.000571	0.003	4.566	4.566	2.454	0.538	2.190832
Lrig1	Lrig1-001	558.8	0.033046	0.071	4.564	4.564	5.129	1.124	2.190264
S100a16	S100a16-003	944.3	0.026342	0.059	4.563	4.563	6.980	1.530	2.190008
Zfp191	Zfp191-002	706.0	0.025473	0.057	4.563	4.563	5.396	1.183	2.189889
Clcn6	Clcn6-201	556.1	0.020719	0.049	4.559	4.559	2.197	0.482	2.188871
Epn1	Epn1-001	6888.6	0.000005	0.000	4.557	4.557	51.578	11.318	2.188104
Shisa5	Shisa5-019	583.9	0.046465	0.093	4.554	4.554	3.930	0.863	2.18722
Gm15013	m15013-001	377.0	0.003052	0.011	4.551	4.551	3.269	0.718	2.186309
Ubxn4	Ubxn4-004	1476.7	0.000032	0.000	4.551	4.551	12.063	2.651	2.186248
Gm25635	m25635-201	350.0	0.008804	0.025	4.551	4.551	2.052	0.451	2.186138
Tinagl1	Tinagl1-001	28978.6	0.000001	0.000	4.550	4.550	195.680	43.004	2.185943
Csnk1g1	Csnk1g1-002	975.1	0.007515	0.022	4.550	4.550	5.317	1.169	2.185879
Usp22	Usp22-005	1525.8	0.001787	0.007	4.550	4.550	9.797	2.153	2.185797
Mtdh	Mtdh-005	2419.1	0.001879	0.007	4.550	4.550	17.065	3.751	2.185717
Mob2	Mob2-201	7385.0	0.000025	0.000	4.548	4.548	20.262	4.455	2.185286
Mprip	Mprip-004	15057.8	0.004470	0.015	4.544	4.544	122.863	27.037	2.184057
30088H06F	0088H06Rik-	2701.0	0.000427	0.002	4.538	4.538	20.441	4.504	2.182048
Ephb4	Ephb4-001	9494.3	0.000024	0.000	4.535	4.535	37.606	8.292	2.181117
Lsm14a	Lsm14a-003	936.1	0.001094	0.005	4.533	4.533	6.721	1.483	2.180521
Myh9	Myh9-001	375702.5	0.000000	0.000	4.522	4.522	3070.978	679.068	2.177071
Map4k2	Map4k2-005	1352.3	0.000376	0.002	4.521	4.521	9.870	2.183	2.176623
Gm13597	m13597-001	264.0	0.008083	0.023	4.515	4.515	1.794	0.397	2.17467
Chd2	Chd2-007	1596.2	0.002944	0.011	4.513	4.513	11.625	2.576	2.173963
Arid1b	Arid1b-201	379.8	0.019452	0.046	4.511	4.511	2.173	0.482	2.173542
Coro7	Coro7-013	510.1	0.003891	0.013	4.510	4.510	3.371	0.747	2.173145
Fkbp5	Fkbp5-001	13226.5	0.002007	0.008	4.508	4.508	94.119	20.879	2.172408
Iars2	Iars2-005	281.2	0.011001	0.030	4.507	4.507	1.802	0.400	2.172024
Thrap3	Thrap3-014	723.1	0.001380	0.006	4.506	4.506	4.843	1.075	2.171936
Cacnb4	Cacnb4-002	674.0	0.001795	0.007	4.503	4.503	5.244	1.165	2.17102

Dbnl	Dbnl-003	1082.1	0.009086	0.026	4.494	4.494	5.363	1.193	2.167954
Dennd2d	Dennd2d-001	464.0	0.013220	0.034	4.494	4.494	5.230	1.164	2.16789
Ctcf	Ctcf-002	1285.8	0.008733	0.025	4.493	4.493	8.159	1.816	2.167567
Hba-a1	Hba-a1-002	4888.1	0.010118	0.028	4.491	4.491	63.503	14.139	2.167172
Eif4g1	Eif4g1-201	5592.5	0.001753	0.007	4.491	4.491	41.751	9.296	2.167165
Papln	Papln-002	213.7	0.020242	0.048	4.485	4.485	1.035	0.231	2.165006
Diap1	Diap1-002	14793.4	0.000156	0.001	4.483	4.483	95.825	21.375	2.164494
Prrc2b	Prrc2b-003	23823.9	0.000030	0.000	4.478	4.478	167.160	37.331	2.162792
Gm5913	Gm5913-001	381.0	0.007198	0.021	4.476	4.476	3.206	0.716	2.16233
Hip1r	Hip1r-001	23508.8	0.000003	0.000	4.472	4.472	130.279	29.132	2.160926
Celf1	Celf1-201	1021.9	0.007923	0.023	4.470	4.470	7.271	1.627	2.160177
Sptbn5	Sptbn5-004	236.0	0.006589	0.020	4.469	4.469	1.310	0.293	2.159957
Chp1	Chp1-004	2017.1	0.000217	0.001	4.468	4.468	15.036	3.365	2.159744
Ubxn6	Ubxn6-002	1237.8	0.041041	0.084	4.468	4.468	7.312	1.637	2.159606
Vprbp	Vprbp-003	11401.0	0.000081	0.001	4.468	4.468	78.931	17.668	2.159489
Gm7308	Gm7308-001	331.0	0.017746	0.043	4.465	4.465	2.248	0.503	2.158672
Gm16350	Gm16350-001	243.9	0.008731	0.025	4.452	4.452	1.415	0.318	2.154348
Gprasp1	Gprasp1-001	2152.5	0.039934	0.082	4.447	4.447	17.222	3.873	2.152778
Pip5k1a	Pip5k1a-005	341.2	0.026295	0.059	4.446	4.446	2.855	0.642	2.152382
Trerf1	Trerf1-201	3184.8	0.000097	0.001	4.445	4.445	13.859	3.118	2.152159
Clcn7	Clcn7-007	1013.3	0.000655	0.003	4.442	4.442	6.953	1.565	2.151251
Mmp3	Mmp3-201	1151.0	0.044511	0.089	4.440	4.440	12.460	2.806	2.150509
Plin4	Plin4-001	854.1	0.000917	0.004	4.439	4.439	4.570	1.029	2.150313
Hdac7	Hdac7-005	6335.4	0.000121	0.001	4.438	4.438	40.907	9.218	2.149838
Smap1	Smap1-003	1531.9	0.046451	0.093	4.435	4.435	10.089	2.275	2.148936
Mrps9	Mrps9-002	1552.1	0.000600	0.003	4.428	4.428	12.598	2.845	2.146671
P23-438D3	23-438D3.3-(	137.0	0.029958	0.065	4.427	4.427	1.059	0.239	2.146284
Ier5l	Ier5l-001	1554.0	0.000075	0.001	4.417	4.417	10.495	2.376	2.143165
Tfeb	Tfeb-002	1593.7	0.000998	0.005	4.413	4.413	11.186	2.535	2.141843
Ap2a1	Ap2a1-202	2221.8	0.000125	0.001	4.413	4.413	15.169	3.437	2.141758
Prpsap1	Prpsap1-007	277.3	0.016800	0.041	4.408	4.408	2.294	0.520	2.140226
Cask	Cask-010	1170.8	0.001352	0.006	4.404	4.404	9.036	2.052	2.138886
Akap2	Akap2-201	4906.9	0.000345	0.002	4.402	4.402	28.922	6.570	2.138302
Fgfr1	Fgfr1-201	1450.5	0.007662	0.022	4.401	4.401	13.150	2.988	2.13798
Ube4b	Ube4b-012	981.1	0.028649	0.063	4.396	4.396	7.585	1.725	2.136308
Npr2	Npr2-002	3843.8	0.000203	0.001	4.393	4.393	24.395	5.553	2.135339
Pecam1	Pecam1-006	5983.9	0.000106	0.001	4.389	4.389	33.372	7.604	2.133877
Itga11	Itga11-001	296.0	0.012339	0.032	4.389	4.389	1.746	0.398	2.13376
Clpb	Clpb-003	569.0	0.021369	0.050	4.383	4.383	4.246	0.969	2.131915
Fkbp8	Fkbp8-002	2005.5	0.012642	0.033	4.380	4.380	16.220	3.703	2.13094
Sh3glb2	Sh3glb2-003	601.1	0.010830	0.029	4.380	4.380	4.375	0.999	2.130939
Shpk	Shpk-001	269.4	0.026449	0.059	4.372	4.372	2.112	0.483	2.1284
Ksr1	Ksr1-004	679.1	0.004478	0.015	4.370	4.370	4.542	1.039	2.127558
Gm10819	Gm10819-001	577.0	0.002949	0.011	4.369	4.369	4.262	0.976	2.127379
Nt5c3	Nt5c3-001	1820.1	0.000189	0.001	4.368	4.368	15.266	3.495	2.127034
Rbm5	Rbm5-016	1680.1	0.000763	0.004	4.367	4.367	10.897	2.495	2.12668
Tbc1d2	Tbc1d2-001	2641.1	0.000023	0.000	4.356	4.356	19.371	4.447	2.122879
Pdgfd	Pdgfd-202	5466.1	0.000097	0.001	4.355	4.355	45.174	10.373	2.122719
Azi2	Azi2-009	1043.5	0.001525	0.006	4.355	4.355	8.247	1.894	2.122625
Plp1	Plp1-001	3302.8	0.000047	0.000	4.352	4.352	22.739	5.225	2.121687
Adra2c	Adra2c-001	534.0	0.021674	0.051	4.351	4.351	1.861	0.428	2.121324
Hras	Hras-001	1302.3	0.000901	0.004	4.348	4.348	9.717	2.235	2.120294
Mum1	Mum1-003	2116.0	0.003160	0.011	4.348	4.348	14.670	3.374	2.120258
Alas2	Alas2-001	1564.6	0.012407	0.033	4.342	4.342	19.727	4.543	2.118497
Npr1	Npr1-004	2799.5	0.000958	0.004	4.342	4.342	19.420	4.472	2.118434
Junb	Junb-201	10143.0	0.001592	0.007	4.342	4.342	95.483	21.992	2.118262
Myl12a	Myl12a-001	9067.3	0.000001	0.000	4.341	4.341	75.551	17.403	2.11808
Tulp4	Tulp4-011	1119.8	0.002190	0.008	4.341	4.341	9.634	2.219	2.118071
Sptan1	Sptan1-010	41338.3	0.000006	0.000	4.341	4.341	249.273	57.426	2.117957
Snrnp200	Snrnp200-002	4575.0	0.000016	0.000	4.339	4.339	32.024	7.380	2.117375
Arfgap2	Arfgap2-001	1886.0	0.041780	0.085	4.338	4.338	13.624	3.140	2.11713
Tek	Tek-003	50058.2	0.000000	0.000	4.338	4.338	232.925	53.693	2.117051
Arhgef1	Arhgef1-003	10634.9	0.000231	0.001	4.336	4.336	63.264	14.592	2.116234
D11Wsu47e	11Wsu47e-0(	458.4	0.013748	0.035	4.335	4.335	3.500	0.807	2.115944
Arhgap23	Arhgap23-003	13792.4	0.000001	0.000	4.335	4.335	71.726	16.547	2.115892
Pbrm1	Pbrm1-002	1737.6	0.000802	0.004	4.332	4.332	11.095	2.561	2.115159
C4b	C4b-001	3646.0	0.000356	0.002	4.326	4.326	33.076	7.646	2.113035
Tnks1bp1	Tnks1bp1-201	17157.0	0.000027	0.000	4.326	4.326	117.444	27.150	2.112959
Elk3	Elk3-004	1421.5	0.001726	0.007	4.313	4.313	10.534	2.442	2.108846
Acox1	Acox1-001	6357.6	0.000142	0.001	4.311	4.311	39.425	9.145	2.108141
Ncdn	Ncdn-003	518.5	0.043928	0.088	4.307	4.307	4.109	0.954	2.106694
Gm5939	Gm5939-001	98.0	0.036784	0.077	4.301	4.301	0.681	0.158	2.104713
Spen	Spen-004	738.7	0.001776	0.007	4.301	4.301	5.792	1.347	2.104704
Txndc5	Txndc5-001	17381.9	0.000192	0.001	4.300	4.300	180.293	41.928	2.104373
Gps1	Gps1-001	622.2	0.033894	0.072	4.291	4.291	5.986	1.395	2.101388
S100a1	S100a1-001	2289.8	0.000025	0.000	4.284	4.284	12.642	2.951	2.099036
Sirpa	Sirpa-006	1414.3	0.001596	0.007	4.284	4.284	10.445	2.438	2.098925
Gm22786	Gm22786-201	254.0	0.023828	0.054	4.283	4.283	1.680	0.392	2.098705
Smoc1	Smoc1-003	13452.5	0.000036	0.000	4.283	4.283	112.344	26.231	2.098606
Tle3	Tle3-014	2600.8	0.016943	0.042	4.281	4.281	20.032	4.679	2.098067
Nfib	Nfib-003	6807.5	0.000515	0.003	4.271	4.271	51.805	12.128	2.094741
Ccdc94	Ccdc94-201	1302.0	0.000842	0.004	4.269	4.269	9.917	2.323	2.093853
Slc9b2	Slc9b2-001	21189.9	0.000003	0.000	4.263	4.263	129.329	30.340	2.091731
Gm5121	Gm5121-201	268.0	0.013337	0.035	4.262	4.262	2.258	0.530	2.091554
Nr1h2	Nr1h2-010	191.7	0.032822	0.070	4.260	4.260	1.465	0.344	2.090792
Bcor	Bcor-001	2233.3	0.009824	0.027	4.259	4.259	20.725	4.866	2.090585
Lzts2	Lzts2-202	2429.6	0.008519	0.024	4.259	4.259	15.543	3.650	2.090383
Tcf3	Tcf3-007	773.8	0.027861	0.062	4.258	4.258	7.339	1.724	2.090011
Ilk	Ilk-002	1572.5	0.009045	0.025	4.256	4.256	9.240	2.171	2.089426
Usf2	Usf2-001	3304.2	0.000154	0.001	4.253	4.253	26.681	6.273	2.088556
Gnaq	Gnaq-003	848.0	0.000168	0.001	4.251	4.251	5.309	1.249	2.087668
Acacb	Acacb-003	2209.8	0.027560	0.061	4.250	4.250	15.670	3.687	2.0874
Rps14	Rps14-001	19852.3	0.000009	0.000	4.250	4.250	166.419	39.161	2.087319
Unc45bos	Unc45bos-001	406.4	0.015569	0.039	4.249	4.249	1.354	0.319	2.08721
Npdc1	Npdc1-011	1076.1	0.000343	0.002	4.247	4.247	8.064	1.899	2.086537
Npepl1	Npepl1-001	2369.8	0.000091	0.001	4.246	4.246	19.486	4.589	2.086028
Csde1	Csde1-002	19904.2	0.000005	0.000	4.245	4.245	126.598	29.826	2.085611
Smarcd3	Smarcd3-001	1636.5	0.000023	0.000	4.243	4.243	8.128	1.915	2.085175
30068E07R	0068E07Rik-	691.9	0.027672	0.061	4.243	4.243	5.814	1.370	2.084981
Rchy1	Rchy1-001	1445.0	0.000944	0.004	4.239	4.239	10.445	2.464	2.083677
Hyou1	Hyou1-003	2563.7	0.039259	0.081	4.236	4.236	20.153	4.758	2.082652
Map4k4	Map4k4-005	27287.1	0.000000	0.000	4.225	4.225	252.049	59.650	2.079109
Aldoa	Aldoa-001	17543.0	0.000006	0.000	4.224	4.224	124.136	29.388	2.078612
Arhgef2	Arhgef2-011	457.7	0.015813	0.039	4.222	4.222	2.300	0.545	2.077864
Ccnt2	Ccnt2-002	4071.4	0.011466	0.031	4.220	4.220	26.736	6.336	2.077176
Lmod1	Lmod1-001	1588.0	0.000813	0.004	4.217	4.217	11.260	2.670	2.076183
Evc2	Evc2-002	967.6	0.032267	0.069	4.217	4.217	7.157	1.697	2.076084

Map7d1	Map7d1-006	6725.2	0.000065	0.001	4.215	4.215	41.533	9.854	2.075488
Thbs3	Thbs3-001	704.9	0.039838	0.082	4.214	4.214	4.560	1.082	2.075067
Vps8	Vps8-202	1470.0	0.019102	0.046	4.213	4.213	9.607	2.280	2.074957
Ubald1	Ubald1-001	2477.7	0.003637	0.012	4.211	4.211	19.614	4.658	2.074151
Prr14	Prr14-008	831.9	0.001186	0.005	4.207	4.207	5.070	1.205	2.07287
P24-496C22	24-496C22.3-	12988.0	0.000001	0.000	4.197	4.197	91.703	21.849	2.069394
Hrct1	Hrct1-001	3026.0	0.002328	0.009	4.181	4.181	25.683	6.143	2.063847
Ppp2r3d	Ppp2r3d-201	342.1	0.007109	0.021	4.179	4.179	2.878	0.689	2.063017
P23-237H9	23-237H9.3-(	173.0	0.035079	0.074	4.176	4.176	1.538	0.368	2.062243
Zkscan6	Zkscan6-002	970.8	0.003936	0.013	4.176	4.176	8.401	2.012	2.062174
Arhgap21	Arhgap21-001	1444.5	0.005338	0.017	4.175	4.175	7.988	1.913	2.061921
Mybbp1a	Mybbp1a-008	3854.4	0.000017	0.000	4.173	4.173	31.329	7.507	2.061153
Gm9817	Gm9817-201	1435.1	0.001497	0.006	4.172	4.172	9.207	2.207	2.060757
Gm3550	Gm3550-201	833.0	0.000806	0.004	4.168	4.168	6.687	1.604	2.05928
Gm15453	Gm15453-001	639.0	0.000669	0.003	4.166	4.166	5.811	1.395	2.058722
Ndufa3	Ndufa3-001	1391.5	0.001385	0.006	4.163	4.163	10.143	2.437	2.057604
Atoh8	Atoh8-201	1580.0	0.000740	0.004	4.160	4.160	18.148	4.363	2.056554
Rpl10a	Rpl10a-003	205.6	0.021768	0.051	4.159	4.159	2.047	0.492	2.056086
Map2k2	Map2k2-002	3134.7	0.000787	0.004	4.153	4.153	22.081	5.317	2.05415
Pip5k1c	Pip5k1c-003	4207.5	0.000157	0.001	4.151	4.151	36.047	8.683	2.053613
Ubxn2a	Ubxn2a-003	400.4	0.026068	0.058	4.146	4.146	1.626	0.392	2.051724
Herc1	Herc1-007	1208.7	0.009922	0.027	4.145	4.145	8.310	2.005	2.051505
Nfatc1	Nfatc1-202	3203.0	0.002288	0.009	4.145	4.145	22.259	5.370	2.051429
Arl8a	Arl8a-001	3702.8	0.000458	0.002	4.142	4.142	29.576	7.140	2.050446
Snhg4	Snhg4-003	1496.3	0.006264	0.019	4.141	4.141	13.900	3.357	2.049973
Gm8101	Gm8101-001	99.0	0.034812	0.074	4.140	4.140	0.878	0.212	2.04965
Vps54	Vps54-006	455.6	0.003641	0.012	4.138	4.138	2.643	0.639	2.049018
Klhl13	Klhl13-002	205.5	0.020528	0.048	4.133	4.133	1.328	0.321	2.047263
Gm10676	Gm10676-201	353.2	0.003858	0.013	4.127	4.127	1.992	0.483	2.045006
Gm37477	Gm37477-001	626.0	0.001334	0.006	4.127	4.127	4.382	1.062	2.044928
Pkn3	Pkn3-008	2003.9	0.009831	0.027	4.125	4.125	7.556	1.832	2.044532
Ppp1r16b	Ppp1r16b-002	7338.1	0.000083	0.001	4.125	4.125	29.058	7.045	2.04428
Samd14	Samd14-001	3163.7	0.000000	0.000	4.119	4.119	22.520	5.468	2.042244
Ccr4	Ccr4-201	522.0	0.003356	0.012	4.103	4.103	3.596	0.876	2.036697
Calm1	Calm1-003	5434.4	0.000185	0.001	4.102	4.102	26.852	6.546	2.036297
Cnot6	Cnot6-201	1101.9	0.007302	0.022	4.096	4.096	8.297	2.026	2.034073
Lrp8	Lrp8-009	2229.4	0.007460	0.022	4.094	4.094	16.511	4.032	2.033666
Bcl7c	Bcl7c-001	928.8	0.006808	0.020	4.094	4.094	7.204	1.760	2.033441
Gm19203	Gm19203-001	345.0	0.010276	0.028	4.093	4.093	2.500	0.611	2.033049
Snx17	Snx17-001	1851.1	0.024946	0.056	4.092	4.092	11.912	2.911	2.032846
Cltb	Cltb-002	1958.9	0.000200	0.001	4.092	4.092	10.144	2.479	2.032834
Pfkm	Pfkm-201	2040.6	0.000290	0.002	4.088	4.088	13.448	3.289	2.031527
Gm15800	Gm15800-003	670.4	0.003512	0.012	4.085	4.085	4.479	1.097	2.030326
Tfap4	Tfap4-201	1423.0	0.001526	0.006	4.082	4.082	13.501	3.308	2.029108
Slu7	Slu7-002	3112.4	0.000415	0.002	4.078	4.078	19.626	4.813	2.027874
Aes	Aes-201	16831.0	0.000000	0.000	4.077	4.077	108.573	26.633	2.027382
Ptms	Ptms-001	33432.7	0.000001	0.000	4.075	4.075	196.841	48.302	2.026886
Ncaph2	Ncaph2-002	1304.6	0.027571	0.061	4.075	4.075	10.546	2.588	2.02684
Gm5615	Gm5615-001	563.0	0.000684	0.003	4.075	4.075	4.112	1.009	2.026688
Abcf3	Abcf3-004	349.9	0.034926	0.074	4.074	4.074	1.853	0.455	2.026513
00037H04R	0037H04Rik-	118.4	0.034611	0.073	4.073	4.073	0.622	0.153	2.026146
Trabd2b	Trabd2b-001	462.0	0.024689	0.056	4.073	4.073	3.059	0.751	2.02598
Hagh	Hagh-001	621.0	0.011785	0.031	4.065	4.065	5.732	1.410	2.023293
Zeb1	Zeb1-007	2714.0	0.004663	0.015	4.061	4.061	18.271	4.499	2.021996
Leng8	Leng8-008	573.9	0.007155	0.021	4.054	4.054	3.751	0.925	2.01919
Cad	Cad-011	300.0	0.042593	0.086	4.052	4.052	1.728	0.426	2.01877
Cnot2	Cnot2-014	438.6	0.025021	0.057	4.050	4.050	3.057	0.755	2.01793
Ncoa5	Ncoa5-003	590.7	0.016774	0.041	4.047	4.047	2.914	0.720	2.016932
Gm16204	Gm16204-001	306.0	0.008774	0.025	4.046	4.046	2.269	0.561	2.016468
Scaf4	Scaf4-201	4643.2	0.000576	0.003	4.044	4.044	34.272	8.474	2.015878
Ugt8a	Ugt8a-001	2859.6	0.001680	0.007	4.042	4.042	26.892	6.653	2.015147
Nfia	Nfia-005	1339.7	0.017713	0.043	4.041	4.041	8.419	2.083	2.014842
Hdac5	Hdac5-003	244.0	0.041502	0.085	4.039	4.039	2.093	0.518	2.013932
Cdo1	Cdo1-201	4492.0	0.004392	0.014	4.038	4.038	50.334	12.464	2.013706
Uba52	Uba52-007	18593.5	0.000001	0.000	4.032	4.032	152.765	37.888	2.011481
Csnk1g2	Csnk1g2-201	4746.5	0.000405	0.002	4.024	4.024	34.440	8.559	2.008477
Unc13d	Unc13d-001	973.6	0.004048	0.014	4.022	4.022	5.337	1.327	2.008086
Magi2	Magi2-001	883.4	0.008382	0.024	4.020	4.020	6.047	1.504	2.007236
Il10rb	Il10rb-001	2560.1	0.003476	0.012	4.014	4.014	19.999	4.982	2.004977
Gm6030	Gm6030-201	1228.0	0.000065	0.001	4.012	4.012	10.579	2.637	2.004241
Bmp7	Bmp7-001	264.0	0.010715	0.029	4.011	4.011	1.713	0.427	2.004015
Pecam1	Pecam1-007	35465.8	0.000030	0.000	4.011	4.011	227.802	56.795	2.003949
Clasrp	Clasrp-201	3467.0	0.000005	0.000	4.009	4.009	25.130	6.268	2.003212
Cnn3	Cnn3-001	10993.3	0.000001	0.000	4.005	4.005	87.622	21.879	2.001729
Lgals4	Lgals4-001	567.7	0.003292	0.012	4.000	4.000	4.450	1.112	2.00007
Ubn2	Ubn2-002	3665.9	0.007154	0.021	3.999	3.999	23.504	5.877	1.999624
810058I24R	0058I24Rik-(	654.9	0.003417	0.012	3.997	3.997	4.242	1.061	1.998988
Gm37964	Gm37964-001	326.0	0.018941	0.046	3.995	3.995	1.482	0.371	1.998032
Prr13	Prr13-003	3089.0	0.001577	0.007	3.993	3.993	15.831	3.964	1.997598
Nup85	Nup85-006	323.3	0.035938	0.075	3.993	3.993	2.355	0.590	1.99756
Rbm5	Rbm5-002	4119.6	0.000126	0.001	3.993	3.993	32.546	8.151	1.997477
Hoxd11	Hoxd11-002	205.0	0.046806	0.093	3.992	3.992	1.481	0.371	1.996948
Tnrc18	Tnrc18-003	27610.5	0.000000	0.000	3.991	3.991	198.260	49.681	1.996614
Hsp90ab1	Hsp90ab1-006	5698.2	0.000013	0.000	3.989	3.989	35.829	8.982	1.996078
Plac9b	Plac9b-001	9986.6	0.000638	0.003	3.987	3.987	44.298	11.111	1.995271
Tesk1	Tesk1-001	855.9	0.022886	0.053	3.982	3.982	6.390	1.605	1.99336
Slmap	Slmap-001	3264.4	0.005869	0.018	3.980	3.980	25.504	6.408	1.992805
Lrwd1	Lrwd1-003	718.4	0.004083	0.014	3.979	3.979	5.035	1.266	1.992233
Fbxo11	Fbxo11-001	4736.4	0.000072	0.001	3.978	3.978	35.674	8.968	1.992031
Zwint	Zwint-003	1438.4	0.007960	0.023	3.978	3.978	12.079	3.037	1.99188
Zc3h18	Zc3h18-001	2150.9	0.009874	0.027	3.974	3.974	15.884	3.997	1.990507
Ptpn14	Ptpn14-005	9413.2	0.015369	0.039	3.973	3.973	55.552	13.981	1.990374
Cnga1	Cnga1-005	521.0	0.004940	0.016	3.970	3.970	3.689	0.929	1.988969
Mapk1ip1	Mapk1ip1-001	2667.3	0.007656	0.022	3.969	3.969	21.142	5.326	1.988939
Srrt	Srrt-002	1925.8	0.004988	0.016	3.969	3.969	11.331	2.855	1.988866
Wnk4	Wnk4-002	453.0	0.041304	0.084	3.968	3.968	1.835	0.462	1.988388
Ccnl2	Ccnl2-003	1035.8	0.002333	0.009	3.963	3.963	6.298	1.589	1.986488
Cyb561	Cyb561-002	630.4	0.002512	0.009	3.960	3.960	5.011	1.265	1.985509
Trappc9	Trappc9-202	1588.2	0.005842	0.018	3.959	3.959	9.038	2.283	1.985177
Ovgp1	Ovgp1-002	594.9	0.014041	0.036	3.958	3.958	4.011	1.013	1.984624
Prorsd1	Prorsd1-001	571.1	0.038052	0.079	3.957	3.957	4.896	1.237	1.984463
Npnt	Npnt-003	898.9	0.013022	0.034	3.956	3.956	6.040	1.527	1.98411
Rpsa-ps4	Rpsa-ps4-001	137.0	0.022578	0.052	3.956	3.956	1.247	0.315	1.984043
Gm12331	Gm12331-001	91.0	0.039561	0.081	3.949	3.949	0.835	0.211	1.981559
Glns-ps1	Glns-ps1-001	411.0	0.002308	0.009	3.947	3.947	2.303	0.583	1.980911
Tmtc1	Tmtc1-002	1164.7	0.012153	0.032	3.943	3.943	8.696	2.205	1.979235
Gm9780	Gm9780-001	24440.4	0.000311	0.002	3.942	3.942	113.743	28.855	1.978873

Smtn	Smtn-005	314.1	0.008783	0.025	3.935	3.935	1.419	0.361	1.976405
Klk12	Klk12-201	249.6	0.012675	0.033	3.928	3.928	0.999	0.254	1.973949
Slx4	Slx4-006	372.4	0.043108	0.087	3.928	3.928	3.461	0.881	1.973946
Mau2	Mau2-201	3838.1	0.000011	0.000	3.928	3.928	30.664	7.806	1.973866
Tbce	Tbce-003	311.9	0.038994	0.080	3.924	3.924	2.554	0.651	1.972441
Hras	Hras-003	341.6	0.022354	0.052	3.922	3.922	2.278	0.581	1.971701
Clip2	Clip2-001	4412.6	0.000052	0.000	3.921	3.921	30.135	7.685	1.971396
Rrbp1	Rrbp1-008	1511.9	0.002293	0.009	3.921	3.921	11.734	2.993	1.971103
31425E22R	1425E22Rik-	1988.0	0.000030	0.000	3.920	3.920	14.021	3.577	1.97093
Stbd1	Stbd1-001	2647.9	0.001941	0.008	3.920	3.920	18.912	4.825	1.970778
10010F05R	0010F05Rik-	1206.3	0.012846	0.034	3.920	3.920	9.188	2.344	1.970713
Itch	Itch-004	2109.5	0.000761	0.004	3.918	3.918	13.414	3.423	1.970223
Clcn2	Clcn2-002	262.3	0.012961	0.034	3.915	3.915	1.838	0.469	1.968891
Agfg2	Agfg2-001	647.4	0.005982	0.018	3.912	3.912	6.150	1.572	1.967893
Unc13b	Unc13b-015	955.0	0.002143	0.008	3.912	3.912	5.331	1.363	1.967868
Gfm1	Gfm1-002	1407.9	0.000364	0.002	3.910	3.910	9.490	2.427	1.967287
P3h3	P3h3-002	748.1	0.049512	0.097	3.910	3.910	8.120	2.077	1.96709
Hes7	Hes7-001	1098.3	0.001987	0.008	3.910	3.910	8.351	2.136	1.967016
Sptan1	Sptan1-011	470.1	0.014959	0.038	3.908	3.908	3.080	0.788	1.966574
Ctbp2	Ctbp2-017	167.4	0.047897	0.095	3.908	3.908	0.724	0.185	1.966327
Uqcrq	Uqcrq-001	224.9	0.021569	0.050	3.907	3.907	1.678	0.429	1.965879
Cer1	Cer1-001	132.0	0.023576	0.054	3.906	3.906	0.933	0.239	1.965599
Rec8	Rec8-001	179.7	0.031054	0.067	3.900	3.900	1.362	0.349	1.963352
Gm15725	Gm15725-001	5430.8	0.023328	0.054	3.900	3.900	43.782	11.227	1.963292
Impa1	Impa1-005	266.2	0.033597	0.072	3.899	3.899	2.053	0.527	1.963279
Sox6	Sox6-203	843.0	0.010289	0.028	3.896	3.896	6.699	1.719	1.962031
Hmgn3	Hmgn3-002	1581.5	0.011478	0.031	3.890	3.890	12.419	3.192	1.959871
Hsd17b12	Hsd17b12-004	1064.9	0.019675	0.047	3.890	3.890	9.936	2.554	1.95987
Fam219a	Fam219a-003	3679.9	0.000550	0.003	3.889	3.889	22.374	5.753	1.959368
Opa1	Opa1-008	3034.6	0.023312	0.054	3.889	3.889	25.257	6.495	1.95932
Ephx1	Ephx1-002	3100.7	0.002182	0.008	3.886	3.886	16.841	4.334	1.958331
Rpl26	Rpl26-001	49881.5	0.000000	0.000	3.880	3.880	423.632	109.173	1.956193
310006J02R	0006J02Rik-(	255.6	0.013490	0.035	3.875	3.875	2.113	0.545	1.954304
Gm13216	Gm13216-001	351.0	0.004418	0.015	3.872	3.872	2.983	0.770	1.953054
Unc45a	Unc45a-201	1175.2	0.049576	0.097	3.871	3.871	7.438	1.921	1.952801
Ccdc86	Ccdc86-001	578.3	0.012132	0.032	3.866	3.866	6.043	1.563	1.950718
Hmg20b	Hmg20b-012	864.0	0.020362	0.048	3.866	3.866	5.105	1.321	1.950659
Dmtn	Dmtn-203	4811.7	0.000181	0.001	3.857	3.857	25.113	6.511	1.947594
Ehmt2	Ehmt2-004	3073.0	0.003014	0.011	3.854	3.854	23.411	6.074	1.946395
Dlg1	Dlg1-006	587.1	0.012706	0.033	3.853	3.853	4.680	1.215	1.945979
Ccnd3	Ccnd3-201	479.9	0.046910	0.093	3.852	3.852	3.355	0.871	1.945765
AU040320	AU040320-00	628.8	0.020426	0.048	3.852	3.852	3.056	0.793	1.945593
Flna	Flna-201	25606.3	0.000059	0.001	3.848	3.848	211.053	54.844	1.944192
Zc3h7a	Zc3h7a-015	1601.5	0.005052	0.016	3.845	3.845	10.328	2.686	1.942965
30416O09R	0416O09Rik-	2088.0	0.001324	0.006	3.844	3.844	12.929	3.363	1.942649
Gigyf1	Gigyf1-001	7887.8	0.000003	0.000	3.841	3.841	56.716	14.767	1.94134
Med13l	Med13l-005	9578.6	0.000031	0.000	3.829	3.829	73.097	19.089	1.937053
Foxd2os	Foxd2os-001	343.8	0.032294	0.069	3.828	3.828	2.661	0.695	1.936693
Fnbp4	Fnbp4-002	2722.5	0.021504	0.050	3.826	3.826	21.135	5.524	1.935843
Scfd2	Scfd2-001	983.4	0.001350	0.006	3.824	3.824	7.070	1.849	1.935013
Cdk12	Cdk12-001	4105.8	0.000496	0.003	3.824	3.824	21.311	5.573	1.935003
Rplp1	Rplp1-201	35133.0	0.000117	0.001	3.820	3.820	291.804	76.396	1.933441
Zmynd11	Zmynd11-002	1368.5	0.010996	0.030	3.819	3.819	12.979	3.398	1.933255
Man2c1	Man2c1-023	634.1	0.023856	0.054	3.811	3.811	4.323	1.134	1.930346
Cdh13	Cdh13-004	483.4	0.036549	0.077	3.808	3.808	2.292	0.602	1.928865
Cyb5r1	Cyb5r1-003	381.8	0.031649	0.068	3.806	3.806	2.406	0.632	1.928458
Agap3	Agap3-001	2268.3	0.002772	0.010	3.801	3.801	15.233	4.008	1.926252
Pf4	Pf4-001	1281.5	0.003049	0.011	3.800	3.800	15.474	4.072	1.926081
Mtmr3	Mtmr3-201	3770.0	0.000709	0.003	3.795	3.795	22.228	5.857	1.924161
Gm2026	Gm2026-003	326.1	0.010371	0.028	3.789	3.789	2.381	0.628	1.921869
Ndufv3	Ndufv3-002	1988.6	0.000676	0.003	3.789	3.789	13.640	3.600	1.92165
Hist1h1c	Hist1h1c-001	9889.0	0.001012	0.005	3.787	3.787	74.667	19.718	1.920934
Sf3a2	Sf3a2-001	1678.9	0.002382	0.009	3.786	3.786	13.779	3.639	1.920854
Atp5j	Atp5j-009	492.9	0.010987	0.030	3.785	3.785	4.096	1.082	1.920289
Hist1h1e	Hist1h1e-001	8548.0	0.012561	0.033	3.784	3.784	67.824	17.922	1.920072
Rabep2	Rabep2-001	2723.0	0.000251	0.002	3.781	3.781	19.805	5.238	1.918904
Tatdn2	Tatdn2-001	2654.7	0.000017	0.000	3.779	3.779	18.378	4.863	1.918144
Tbc1d8	Tbc1d8-004	4434.8	0.031524	0.068	3.779	3.779	40.287	10.661	1.917919
Mast2	Mast2-201	2494.3	0.002807	0.010	3.777	3.777	10.896	2.885	1.917306
Cerk	Cerk-001	2479.6	0.015775	0.039	3.776	3.776	20.501	5.429	1.916978
Rexo1	Rexo1-002	740.3	0.013860	0.036	3.774	3.774	6.285	1.665	1.91595
Atp2a3	Atp2a3-203	16096.9	0.001549	0.006	3.771	3.771	66.441	17.617	1.915132
Rsrp1	Rsrp1-002	1644.6	0.046390	0.092	3.771	3.771	10.752	2.851	1.914824
Ctnnd1	Ctnnd1-001	10166.4	0.000955	0.004	3.769	3.769	71.721	19.032	1.914003
Pikfyve	Pikfyve-006	1331.0	0.021589	0.050	3.768	3.768	12.409	3.293	1.91396
Unk	Unk-001	1164.3	0.004957	0.016	3.768	3.768	9.609	2.550	1.913707
Tigit	Tigit-201	1387.0	0.002121	0.008	3.764	3.764	8.997	2.390	1.912238
Erbb2ip	Erbb2ip-004	2164.5	0.003803	0.013	3.761	3.761	15.821	4.206	1.911296
Gm7857	Gm7857-001	168.0	0.034485	0.073	3.761	3.761	1.395	0.371	1.911169
Tug1	Tug1-005	804.5	0.010140	0.028	3.760	3.760	5.146	1.369	1.910637
Mad1l1	Mad1l1-001	2826.3	0.000999	0.005	3.759	3.759	17.204	4.576	1.910506
Gem	Gem-001	1221.8	0.012444	0.033	3.759	3.759	11.633	3.095	1.910327
Pmepa1	Pmepa1-002	1262.0	0.025394	0.057	3.758	3.758	8.371	2.228	1.909929
Gm4617	Gm4617-001	3821.0	0.001071	0.005	3.758	3.758	27.569	7.336	1.909911
Ogfr	Ogfr-001	3401.6	0.000128	0.001	3.757	3.757	25.354	6.749	1.909543
Hist1h2bn	Hist1h2bn-001	290.0	0.031879	0.069	3.751	3.751	2.551	0.680	1.907273
Gm6404	Gm6404-201	165.0	0.017676	0.043	3.750	3.750	1.211	0.323	1.906869
Zgpat	Zgpat-003	1945.9	0.000672	0.003	3.750	3.750	14.060	3.750	1.906801
Anapc5	Anapc5-018	976.1	0.010942	0.030	3.748	3.748	7.159	1.910	1.905966
32411N23R	2411N23Rik-	332.0	0.006522	0.020	3.747	3.747	3.073	0.820	1.905654
Pex14	Pex14-001	1556.9	0.000123	0.001	3.745	3.745	11.958	3.193	1.904845
Gm22579	Gm22579-201	220.0	0.012948	0.034	3.744	3.744	1.581	0.422	1.904419
Ppp2r3a	Ppp2r3a-003	3666.6	0.000138	0.001	3.743	3.743	28.369	7.579	1.904182
Cygb	Cygb-001	1518.3	0.005169	0.016	3.743	3.743	11.483	3.068	1.904173
Xab2	Xab2-003	756.2	0.044659	0.090	3.742	3.742	7.049	1.884	1.903643
Snhg11	Snhg11-001	1868.8	0.000184	0.001	3.741	3.741	12.936	3.458	1.903363
Nov	Nov-001	1466.0	0.007996	0.023	3.731	3.731	8.256	2.213	1.899672
Hdac7	Hdac7-002	1157.5	0.023597	0.054	3.729	3.729	8.771	2.352	1.898799
Sun2	Sun2-002	9904.2	0.000020	0.000	3.728	3.728	74.087	19.872	1.898511
P24-86C15.	24-86C15.1-(	119.0	0.032868	0.070	3.727	3.727	0.978	0.262	1.898122
Smim7	Smim7-003	399.1	0.020879	0.049	3.726	3.726	2.378	0.638	1.897704
Eif4e2	Eif4e2-006	1823.2	0.015080	0.038	3.724	3.724	14.190	3.810	1.896856
Stam2	Stam2-002	1060.4	0.022516	0.052	3.723	3.723	9.041	2.428	1.896648
Obscn	Obscn-003	937.2	0.013351	0.035	3.721	3.721	3.501	0.941	1.895645
Pds5a	Pds5a-009	905.7	0.008603	0.024	3.721	3.721	6.748	1.814	1.895569
Fcho2	Fcho2-204	1983.0	0.001996	0.008	3.720	3.720	15.211	4.089	1.895155
P23-27B23.	23-27B23.9-(	3757.0	0.000101	0.001	3.719	3.719	28.261	7.600	1.894837

Plec	Plec-005	25313.0	0.000143	0.001	3.719	3.719	107.974	29.035	1.894825
Gm3788	Gm3788-001	204.0	0.027388	0.061	3.719	3.719	1.859	0.500	1.894802
Nr1h2	Nr1h2-002	1574.2	0.028977	0.063	3.714	3.714	9.910	2.668	1.892946
Plec	Plec-204	10779.8	0.000186	0.001	3.707	3.707	43.360	11.698	1.890072
Srsf1	Srsf1-004	1561.5	0.045995	0.092	3.704	3.704	11.540	3.115	1.889221
Il16	Il16-001	1419.0	0.016371	0.041	3.692	3.692	9.570	2.592	1.884443
00033N17R	0033N17Rik-	215.0	0.030560	0.066	3.692	3.692	1.979	0.536	1.884331
Fam76b	Fam76b-003	322.0	0.018655	0.045	3.692	3.692	3.164	0.857	1.88422
Add1	Add1-015	4465.9	0.000009	0.000	3.691	3.691	28.219	7.645	1.884047
Hhex	Hhex-001	5179.4	0.000027	0.000	3.690	3.690	39.115	10.600	1.88363
Hsd3b7	Hsd3b7-004	1581.0	0.011702	0.031	3.689	3.689	9.068	2.458	1.883038
Ino80e	Ino80e-002	1195.5	0.027182	0.060	3.689	3.689	10.358	2.808	1.883035
00021K19R	0021K19Rik-	763.5	0.022624	0.052	3.685	3.685	5.302	1.439	1.881511
Gm6180	Gm6180-201	1003.0	0.005417	0.017	3.684	3.684	7.914	2.148	1.881374
Asph	Asph-001	3309.8	0.000104	0.001	3.681	3.681	21.772	5.914	1.880256
Zbtb20	Zbtb20-007	1158.8	0.001957	0.008	3.680	3.680	8.033	2.183	1.879607
Gm37017	Gm37017-001	365.0	0.009741	0.027	3.680	3.680	2.428	0.660	1.879517
U2af2	U2af2-201	8950.6	0.000041	0.000	3.680	3.680	65.115	17.697	1.879515
Ppp1r16b	Ppp1r16b-001	800.0	0.005716	0.018	3.678	3.678	3.781	1.028	1.878984
Rpl21	Rpl21-005	172.9	0.031096	0.067	3.676	3.676	1.226	0.334	1.878051
Evc	Evc-001	3712.3	0.001813	0.007	3.675	3.675	24.514	6.670	1.877859
Adipor1	Adipor1-002	2663.0	0.001933	0.008	3.675	3.675	17.934	4.880	1.877634
Ssh2	Ssh2-002	1993.0	0.032797	0.070	3.674	3.674	13.492	3.673	1.87723
Endog	Endog-001	359.4	0.044022	0.089	3.672	3.672	3.552	0.967	1.876519
Synrg	Synrg-002	1000.4	0.008158	0.023	3.670	3.670	7.912	2.156	1.875684
Zfp524	Zfp524-201	795.0	0.000265	0.002	3.665	3.665	3.679	1.004	1.873976
Srrm2	Srrm2-010	603.8	0.006300	0.019	3.665	3.665	3.488	0.952	1.873678
Spryd3	Spryd3-001	3014.7	0.002636	0.010	3.662	3.662	23.512	6.421	1.872455
Git2	Git2-012	1656.5	0.013026	0.034	3.661	3.661	12.663	3.459	1.872194
Sh2d4b	Sh2d4b-001	405.2	0.005684	0.018	3.660	3.660	2.866	0.783	1.871741
Gm14291	Gm14291-001	264.0	0.035540	0.075	3.659	3.659	1.358	0.371	1.871389
Gm16286	Gm16286-001	3338.8	0.000036	0.000	3.657	3.657	27.960	7.645	1.870835
Gm7729	Gm7729-001	1233.0	0.000391	0.002	3.655	3.655	9.904	2.710	1.86977
Bcor	Bcor-003	1444.2	0.007841	0.023	3.654	3.654	8.790	2.406	1.869291
Bcorl1	Bcorl1-001	2617.5	0.001187	0.005	3.652	3.652	14.700	4.025	1.868783
Dctn1	Dctn1-011	523.0	0.028765	0.063	3.648	3.648	2.863	0.785	1.8671
Gm26387	Gm26387-201	579.0	0.006259	0.019	3.647	3.647	5.221	1.432	1.866675
Eif4g1	Eif4g1-011	1759.3	0.017233	0.042	3.646	3.646	7.542	2.068	1.866506
Rai1	Rai1-201	1710.9	0.040265	0.083	3.645	3.645	12.714	3.489	1.865743
Abcc5	Abcc5-011	604.7	0.016977	0.042	3.641	3.641	3.314	0.910	1.864496
Tada2b	Tada2b-002	795.5	0.044798	0.090	3.641	3.641	5.208	1.431	1.864328
00034H15R	0034H15Rik-	2592.4	0.000241	0.002	3.638	3.638	16.016	4.402	1.863191
Yif1b	Yif1b-002	879.1	0.003430	0.012	3.637	3.637	6.474	1.780	1.86288
Fblim1	Fblim1-009	884.9	0.010714	0.029	3.637	3.637	8.165	2.245	1.862865
230050P20R	0050P20Rik-	3890.1	0.000389	0.002	3.634	3.634	28.430	7.823	1.861687
Tnr	Tnr-001	365.0	0.008975	0.025	3.631	3.631	2.500	0.688	1.860365
Pcdhga1	Pcdhga1-001	327.7	0.020271	0.048	3.631	3.631	2.794	0.770	1.860188
Igfbp5	Igfbp5-002	2629.7	0.040650	0.083	3.630	3.630	10.356	2.853	1.860106
Fbrsl1	Fbrsl1-008	2580.1	0.000129	0.001	3.628	3.628	15.162	4.179	1.859312
Gm16348	Gm16348-001	876.0	0.001697	0.007	3.627	3.627	6.164	1.699	1.858822
Ssfa2	Ssfa2-005	2795.1	0.005454	0.017	3.626	3.626	15.608	4.304	1.85852
Ube4a	Ube4a-005	1829.4	0.022005	0.051	3.624	3.624	13.797	3.807	1.857668
30001P10R	0001P10Rik-	671.0	0.000964	0.004	3.624	3.624	5.106	1.409	1.857498
Pea15a	Pea15a-002	2453.5	0.001050	0.005	3.622	3.622	11.196	3.091	1.856865
Arfgap2	Arfgap2-007	174.3	0.047035	0.093	3.621	3.621	1.178	0.325	1.856582
Polr3d	Polr3d-201	1404.4	0.021895	0.051	3.620	3.620	12.771	3.528	1.855973
Prdx5	Prdx5-004	1166.8	0.039813	0.082	3.618	3.618	9.708	2.683	1.855232
Zfp362	Zfp362-201	2245.7	0.005748	0.018	3.617	3.617	14.421	3.987	1.85485
Rasa4	Rasa4-003	520.8	0.014504	0.037	3.614	3.614	4.407	1.219	1.853725
P2ry1	P2ry1-003	726.5	0.009619	0.027	3.611	3.611	2.592	0.718	1.852247
Smad5	Smad5-004	956.7	0.031888	0.069	3.609	3.609	8.663	2.400	1.851695
Tsc22d2	Tsc22d2-002	3363.7	0.001074	0.005	3.603	3.603	23.336	6.477	1.84928
Gm37396	Gm37396-001	2177.0	0.000033	0.000	3.602	3.602	13.591	3.774	1.848616
Pdlim4	Pdlim4-002	415.6	0.033029	0.071	3.596	3.596	2.784	0.774	1.846469
Lipe	Lipe-001	892.2	0.021668	0.051	3.594	3.594	4.178	1.162	1.845556
Ace	Ace-003	4730.0	0.007706	0.022	3.594	3.594	31.699	8.820	1.845527
Rps6ka4	Rps6ka4-201	3110.1	0.003400	0.012	3.591	3.591	17.185	4.786	1.844283
Rp9	Rp9-201	4407.0	0.000149	0.001	3.591	3.591	34.591	9.634	1.844197
Hmgcl	Hmgcl-001	1376.9	0.005233	0.017	3.590	3.590	9.592	2.672	1.843922
Gm13127	Gm13127-001	200.0	0.014841	0.038	3.589	3.589	1.437	0.400	1.843775
Camta1	Camta1-006	507.9	0.024411	0.055	3.589	3.589	5.206	1.451	1.843603
Bcl2l2	Bcl2l2-002	1588.2	0.004061	0.014	3.588	3.588	9.697	2.702	1.843255
Gnas	Gnas-002	902.6	0.030632	0.066	3.587	3.587	5.358	1.494	1.842692
Gimap1	Gimap1-003	294.5	0.037271	0.078	3.586	3.586	0.983	0.274	1.842378
Kansl3	Kansl3-001	3851.7	0.004399	0.014	3.585	3.585	25.929	7.233	1.84189
Cttnbp2	Cttnbp2-001	788.4	0.003777	0.013	3.584	3.584	2.877	0.803	1.841762
Smarcd2	Smarcd2-001	1820.7	0.000493	0.003	3.583	3.583	11.737	3.275	1.841288
Gm15772	Gm15772-001	2355.0	0.000002	0.000	3.573	3.573	18.946	5.302	1.83726
Stk19	Stk19-004	424.5	0.014861	0.038	3.573	3.573	3.289	0.920	1.837214
Tns1	Tns1-012	592.7	0.007512	0.022	3.566	3.566	3.184	0.893	1.834352
P4htm	P4htm-001	887.3	0.004389	0.014	3.566	3.566	7.268	2.038	1.834242
H2-T22	H2-T22-002	3826.0	0.012186	0.032	3.566	3.566	22.079	6.192	1.834133
Hdac7	Hdac7-004	1849.0	0.019003	0.046	3.565	3.565	9.479	2.659	1.833891
Crip1	Crip1-004	3458.6	0.000322	0.002	3.565	3.565	16.798	4.713	1.833718
Gm26672	Gm26672-002	1265.5	0.030361	0.066	3.563	3.563	9.981	2.801	1.832928
Arhgef6	Arhgef6-007	344.7	0.016605	0.041	3.563	3.563	2.666	0.748	1.832898
Rhot2	Rhot2-002	685.1	0.011247	0.030	3.559	3.559	5.045	1.417	1.831588
Map4k2	Map4k2-003	1711.1	0.015424	0.039	3.557	3.557	9.009	2.533	1.830573
Slc1a3	Slc1a3-007	671.1	0.001284	0.006	3.555	3.555	4.972	1.399	1.829994
Zfp865	Zfp865-002	1388.2	0.031095	0.067	3.555	3.555	9.945	2.797	1.829853
Mib2	Mib2-002	1638.2	0.010262	0.028	3.554	3.554	12.672	3.565	1.82953
Zbtb7b	Zbtb7b-001	2233.6	0.022257	0.052	3.554	3.554	13.677	3.848	1.829476
Smurf1	Smurf1-003	1781.9	0.002302	0.009	3.552	3.552	13.429	3.781	1.828728
Pfkfb3	Pfkfb3-001	1213.3	0.001266	0.005	3.551	3.551	11.207	3.156	1.828164
Vps39	Vps39-003	1935.0	0.042883	0.087	3.550	3.550	13.716	3.863	1.827893
Sphk2	Sphk2-003	266.1	0.034687	0.073	3.543	3.543	2.436	0.688	1.824852
Psmd4	Psmd4-001	3243.2	0.022871	0.053	3.541	3.541	25.212	7.121	1.824084
Ocln	Ocln-003	1847.6	0.018571	0.045	3.537	3.537	11.586	3.276	1.822581
Taok2	Taok2-001	18487.8	0.000010	0.000	3.535	3.535	112.897	31.937	1.821702
Loxl1	Loxl1-201	2456.0	0.000796	0.004	3.535	3.535	21.957	6.212	1.821548
Atraid	Atraid-001	1133.3	0.019224	0.046	3.534	3.534	5.969	1.689	1.821495
Arl4d	Arl4d-001	5729.0	0.002215	0.008	3.534	3.534	12.556	3.552	1.821489
Cd99l2	Cd99l2-001	3573.7	0.002455	0.009	3.531	3.531	21.368	6.052	1.820053
Gm16245	Gm16245-001	3073.0	0.000021	0.000	3.530	3.530	19.237	5.449	1.819779
Gtf2a2	Gtf2a2-001	775.4	0.000362	0.002	3.530	3.530	4.955	1.404	1.819539
Numb	Numb-009	863.1	0.047413	0.094	3.528	3.528	4.184	1.186	1.818873
Dlx6	Dlx6-001	1512.8	0.000723	0.004	3.525	3.525	9.810	2.783	1.817483

Gabra2	Gabra2-002	8201.0	0.000012	0.000	3.522	3.522	53.646	15.230	1.816531
Pabpn1	Pabpn1-008	2429.9	0.015985	0.040	3.522	3.522	16.691	4.740	1.816238
Fgl1	Fgl1-002	212.4	0.020638	0.049	3.519	3.519	1.409	0.400	1.815222
Bcl7b	Bcl7b-001	3787.5	0.000006	0.000	3.518	3.518	24.411	6.938	1.814847
Ldb1	Ldb1-004	1561.0	0.000158	0.001	3.518	3.518	11.484	3.265	1.814568
Smu1	Smu1-002	1929.8	0.000044	0.000	3.517	3.517	13.235	3.763	1.814325
Gltscr2	Gltscr2-001	4842.4	0.000002	0.000	3.516	3.516	38.423	10.927	1.814004
Sox13	Sox13-002	9937.0	0.000018	0.000	3.516	3.516	35.582	10.121	1.813779
Flna	Flna-006	754.0	0.003042	0.011	3.512	3.512	5.189	1.477	1.812361
Akap13	Akap13-008	5191.1	0.001386	0.006	3.510	3.510	29.190	8.316	1.811446
Zfp871	Zfp871-002	701.9	0.006639	0.020	3.509	3.509	4.775	1.361	1.810869
Sv2c	Sv2c-001	2097.0	0.000184	0.001	3.508	3.508	14.814	4.224	1.810451
Atp6v0e2	Atp6v0e2-201	807.0	0.005687	0.018	3.506	3.506	6.018	1.717	1.80968
Git2	Git2-004	674.9	0.018617	0.045	3.503	3.503	5.910	1.687	1.808595
Gimap4	Gimap4-004	2969.5	0.012309	0.032	3.503	3.503	19.288	5.506	1.80853
Vti1b	Vti1b-001	5552.1	0.000121	0.001	3.502	3.502	39.493	11.277	1.808245
5-Sep	Sept5-201	491.6	0.013546	0.035	3.499	3.499	4.777	1.366	1.806801
Gm28727	Gm28727-001	6892.4	0.000167	0.001	3.497	3.497	53.513	15.303	1.806016
Senp3	Senp3-001	2789.1	0.003514	0.012	3.494	3.494	20.870	5.973	1.804841
Capns1	Capns1-001	14941.8	0.000002	0.000	3.493	3.493	73.558	21.059	1.804475
Ttc14	Ttc14-006	2537.9	0.013816	0.036	3.492	3.492	18.184	5.207	1.804106
Zcchc8	Zcchc8-001	1475.0	0.023962	0.055	3.492	3.492	10.601	3.036	1.80397
Kctd6	Kctd6-201	774.4	0.004865	0.016	3.491	3.491	6.860	1.965	1.803559
Gm21887	Gm21887-202	599.0	0.043656	0.088	3.483	3.483	5.217	1.498	1.800441
Rfwd2	Rfwd2-005	640.1	0.003477	0.012	3.482	3.482	5.248	1.507	1.800091
Dnmt3a	Dnmt3a-004	1726.2	0.010466	0.029	3.479	3.479	8.903	2.559	1.798482
Golga2	Golga2-002	2671.5	0.043285	0.087	3.478	3.478	18.115	5.208	1.79841
Aff4	Aff4-006	202.6	0.029839	0.065	3.478	3.478	2.302	0.662	1.798195
Psme1	Psme1-003	2449.5	0.009663	0.027	3.474	3.474	14.153	4.073	1.796799
Tymp	Tymp-201	400.3	0.002905	0.010	3.474	3.474	2.757	0.794	1.796733
Flna	Flna-013	4445.4	0.002705	0.010	3.473	3.473	36.736	10.577	1.796243
Rpl13	Rpl13-002	1814.0	0.000341	0.002	3.472	3.472	14.232	4.099	1.79585
Itfg3	Itfg3-001	1735.0	0.001302	0.006	3.472	3.472	11.909	3.430	1.79574
Cc2d1a	Cc2d1a-002	540.0	0.019872	0.047	3.471	3.471	3.998	1.152	1.795148
230208H11R	0208H11Rik-	1114.6	0.000903	0.004	3.462	3.462	10.103	2.918	1.791556
Sephs2	Sephs2-201	641.0	0.017162	0.042	3.462	3.462	5.699	1.646	1.791498
Rnf10	Rnf10-003	1937.8	0.009897	0.027	3.456	3.456	13.099	3.790	1.789176
Srsf5	Srsf5-001	1800.4	0.008305	0.024	3.453	3.453	11.195	3.242	1.787859
Jam2	Jam2-002	1290.8	0.008137	0.023	3.451	3.451	5.369	1.556	1.78703
Sirt1	Sirt1-201	954.3	0.045624	0.091	3.443	3.443	7.699	2.237	1.783463
Ulk3	Ulk3-201	7796.0	0.000253	0.002	3.441	3.441	56.221	16.339	1.782819
Hnrnpl	Hnrnpl-005	1594.4	0.009433	0.026	3.440	3.440	9.420	2.739	1.782226
Brap	Brap-001	2653.3	0.035203	0.074	3.440	3.440	22.128	6.433	1.78222
Ppt2	Ppt2-006	889.6	0.042406	0.086	3.436	3.436	7.357	2.141	1.78092
Tmco1	Tmco1-003	5114.5	0.000005	0.000	3.435	3.435	32.945	9.592	1.780142
Celf1	Celf1-001	2162.3	0.000589	0.003	3.433	3.433	15.620	4.550	1.779347
Sap30l	Sap30l-001	3220.0	0.000081	0.001	3.430	3.430	21.533	6.277	1.778327
31434O11R	1434O11Rik-	457.8	0.035400	0.075	3.429	3.429	3.066	0.894	1.777791
Tlk2	Tlk2-006	1213.5	0.000520	0.003	3.429	3.429	7.646	2.230	1.777757
Col6a3	Col6a3-201	2594.7	0.007958	0.023	3.428	3.428	4.508	1.315	1.777182
Zbtb16	Zbtb16-201	18791.0	0.025701	0.058	3.427	3.427	121.610	35.486	1.776947
Sepp1	Sepp1-005	2125.5	0.002062	0.008	3.422	3.422	22.276	6.510	1.774817
Shb	Shb-001	1140.9	0.000303	0.002	3.420	3.420	7.653	2.237	1.774069
Ech1	Ech1-001	2230.7	0.000296	0.002	3.415	3.415	17.173	5.029	1.771929
Arrb1	Arrb1-007	1249.8	0.005137	0.016	3.403	3.403	6.900	2.027	1.766962
Myo18a	Myo18a-018	2891.5	0.019060	0.046	3.403	3.403	16.783	4.932	1.766742
Ncor1	Ncor1-009	1794.8	0.001121	0.005	3.399	3.399	12.533	3.688	1.765005
Kcnb1	Kcnb1-001	885.2	0.013214	0.034	3.397	3.397	1.376	0.405	1.764216
Ston1	Ston1-201	1993.2	0.022689	0.052	3.395	3.395	19.150	5.640	1.76362
Plxna3	Plxna3-001	306.2	0.011130	0.030	3.393	3.393	2.241	0.660	1.762667
Rbpj	Rbpj-007	1341.5	0.001193	0.005	3.385	3.385	9.283	2.742	1.759365
Cdk5rap3	Cdk5rap3-001	1239.8	0.026401	0.059	3.385	3.385	9.817	2.900	1.759308
Gm6170	Gm6170-001	2208.0	0.000017	0.000	3.380	3.380	18.845	5.575	1.757059
Sorbs2	Sorbs2-201	1105.0	0.041493	0.085	3.379	3.379	7.958	2.355	1.756641
Parp3	Parp3-005	1138.1	0.028623	0.063	3.375	3.375	7.905	2.342	1.754817
Rgs3	Rgs3-005	5367.2	0.010690	0.029	3.372	3.372	32.212	9.552	1.753663
Rn18s-rs5	Rn18s-rs5-001	17974703.1	0.001884	0.007	3.366	3.366	132964.229	39496.491	1.751242
Pcbp4	Pcbp4-001	1644.3	0.000430	0.002	3.365	3.365	11.974	3.558	1.7508
Dusp22	Dusp22-002	639.0	0.028123	0.062	3.363	3.363	4.703	1.399	1.749678
Gm13050	Gm13050-001	390.0	0.004937	0.016	3.362	3.362	3.209	0.955	1.749292
Fam102b	Fam102b-201	3440.3	0.006284	0.019	3.358	3.358	26.228	7.810	1.747706
Pak4	Pak4-001	3518.3	0.001344	0.006	3.358	3.358	13.733	4.090	1.747587
Ankrd13a	Ankrd13a-001	10552.3	0.000036	0.000	3.356	3.356	78.709	23.450	1.746907
Myo1c	Myo1c-002	18521.3	0.000033	0.000	3.354	3.354	98.259	29.294	1.745963
Ern1	Ern1-002	2132.6	0.001655	0.007	3.353	3.353	10.814	3.225	1.745466
Pkn3	Pkn3-001	15759.3	0.000042	0.000	3.352	3.352	66.031	19.700	1.74494
Glrb	Glrb-006	1491.0	0.000240	0.002	3.351	3.351	10.001	2.985	1.744515
Cdk9	Cdk9-001	2632.5	0.000312	0.002	3.347	3.347	21.544	6.436	1.743084
Stat5a	Stat5a-001	995.4	0.038796	0.080	3.345	3.345	9.727	2.908	1.741805
Gm29408	Gm29408-001	1904.0	0.002448	0.009	3.340	3.340	14.662	4.390	1.73966
Gltscr2	Gltscr2-002	5880.0	0.010513	0.029	3.338	3.338	46.083	13.807	1.738853
Sh3bp5l	Sh3bp5l-201	1311.1	0.013450	0.035	3.337	3.337	8.627	2.585	1.738636
Atxn7l3	Atxn7l3-001	1702.6	0.012041	0.032	3.335	3.335	11.445	3.431	1.737852
Mlph	Mlph-001	6456.1	0.015588	0.039	3.334	3.334	27.332	8.198	1.737262
Scara5	Scara5-001	2556.8	0.005380	0.017	3.333	3.333	16.005	4.802	1.736758
Acin1	Acin1-024	582.2	0.035077	0.074	3.331	3.331	4.755	1.428	1.735926
Cfb	Cfb-002	2041.7	0.000203	0.001	3.330	3.330	17.530	5.265	1.735466
Ctdsp2	Ctdsp2-202	21532.9	0.000017	0.000	3.328	3.328	158.170	47.524	1.734752
Kif5b	Kif5b-004	5221.3	0.000719	0.004	3.327	3.327	31.985	9.612	1.734438
Plec	Plec-013	22124.0	0.000055	0.001	3.327	3.327	88.697	26.658	1.734298
Gsn	Gsn-005	7121.9	0.000163	0.001	3.327	3.327	39.745	11.946	1.734236
Hist1h3e	Hist1h3e-001	396.0	0.034959	0.074	3.325	3.325	3.925	1.180	1.733291
Sost	Sost-001	857.0	0.010129	0.028	3.324	3.324	4.671	1.405	1.73295
Snrpa	Snrpa-001	1258.6	0.021711	0.051	3.323	3.323	10.840	3.262	1.73248
Atp6ap1	Atp6ap1-002	2377.3	0.000532	0.003	3.322	3.322	13.025	3.921	1.732083
Tnxb	Tnxb-002	2066.3	0.041319	0.084	3.320	3.320	9.921	2.988	1.731353
Bin2	Bin2-001	299.4	0.046445	0.093	3.317	3.317	3.938	1.187	1.72976
Pkp4	Pkp4-201	11167.3	0.000563	0.003	3.315	3.315	62.577	18.874	1.729199
Uspl1	Uspl1-012	772.9	0.009816	0.027	3.315	3.315	7.018	2.117	1.728954
Anxa11	Anxa11-001	6680.4	0.000019	0.000	3.310	3.310	42.461	12.829	1.726691
Ercc3	Ercc3-001	2468.8	0.000046	0.000	3.307	3.307	17.191	5.198	1.725718
Maf1	Maf1-001	1980.1	0.001265	0.005	3.307	3.307	15.989	4.836	1.725312
Pitpnm2	Pitpnm2-003	1912.9	0.002018	0.008	3.306	3.306	12.043	3.643	1.725149
Hist2h4	Hist2h4-001	1532.0	0.016444	0.041	3.306	3.306	11.129	3.367	1.724887
Pum1	Pum1-003	3616.8	0.005039	0.016	3.296	3.296	26.894	8.158	1.720918
Rhot1	Rhot1-001	978.7	0.022618	0.052	3.295	3.295	5.905	1.792	1.720398
Zfp672	Zfp672-001	1696.4	0.000555	0.003	3.295	3.295	10.390	3.154	1.720104

Klk14	Klk14-001	1096.7	0.000902	0.004	3.294	3.294	7.238	2.198	1.719681
Wipf1	Wipf1-003	645.7	0.047739	0.095	3.293	3.293	3.591	1.090	1.719401
Stub1	Stub1-201	6854.1	0.000007	0.000	3.292	3.292	48.802	14.825	1.718889
Sptbn5	Sptbn5-001	1327.0	0.016167	0.040	3.289	3.289	7.569	2.301	1.717739
Nat14	Nat14-201	371.0	0.015752	0.039	3.289	3.289	2.348	0.714	1.717726
Pdlim2	Pdlim2-002	1840.9	0.037657	0.078	3.288	3.288	5.775	1.756	1.717379
Rbm39	Rbm39-008	666.3	0.017116	0.042	3.287	3.287	4.421	1.345	1.716926
Kansl2	Kansl2-202	2209.9	0.000870	0.004	3.285	3.285	17.164	5.226	1.715766
Parvaos	Parvaos-001	164.0	0.048043	0.095	3.282	3.282	1.210	0.369	1.714624
Rps6ka4	Rps6ka4-202	3216.9	0.000718	0.004	3.279	3.279	18.636	5.683	1.713433
430010I23R	30010I23Rik-	1743.7	0.001337	0.006	3.278	3.278	12.387	3.779	1.712756
Tfeb	Tfeb-011	353.7	0.022226	0.052	3.277	3.277	2.357	0.719	1.712308
Ip6k2	Ip6k2-009	410.0	0.010658	0.029	3.277	3.277	3.099	0.946	1.712211
Ngfr	Ngfr-001	235.0	0.017854	0.043	3.275	3.275	1.715	0.524	1.711449
Cmtm8	Cmtm8-201	511.0	0.004176	0.014	3.275	3.275	2.594	0.792	1.711351
Por	Por-001	12883.2	0.000122	0.001	3.275	3.275	74.154	22.645	1.711347
Gnb1	Gnb1-003	968.4	0.001966	0.008	3.273	3.273	5.811	1.775	1.710742
Dag1	Dag1-003	1849.5	0.012676	0.033	3.267	3.267	14.182	4.341	1.707899
Tpt1-ps6	Tpt1-ps6-001	4550.0	0.000004	0.000	3.266	3.266	36.356	11.131	1.707683
Necap2	Necap2-001	2737.6	0.017172	0.042	3.266	3.266	20.708	6.340	1.707613
Son	Son-013	19351.3	0.000017	0.000	3.265	3.265	129.057	39.528	1.707057
Dnaaf3	Dnaaf3-001	361.6	0.021908	0.051	3.263	3.263	2.507	0.768	1.70633
Csde1	Csde1-003	4206.1	0.003210	0.011	3.262	3.262	31.535	9.667	1.705757
Srrm1	Srrm1-011	608.0	0.048384	0.096	3.262	3.262	3.351	1.027	1.705674
Mbd6	Mbd6-002	1909.9	0.002406	0.009	3.260	3.260	13.035	3.999	1.704663
Ddx39b	Ddx39b-005	3192.5	0.000068	0.001	3.259	3.259	23.211	7.122	1.704379
Atp2c1	Atp2c1-011	1617.7	0.010618	0.029	3.259	3.259	12.863	3.947	1.704258
Gm5525	Gm5525-001	393.0	0.029748	0.065	3.257	3.257	3.157	0.969	1.7037
Gtf2i	Gtf2i-202	2637.0	0.001357	0.006	3.257	3.257	16.926	5.197	1.703599
Git2	Git2-201	508.8	0.045657	0.091	3.255	3.255	5.060	1.555	1.70271
Psmd2	Psmd2-007	587.5	0.022821	0.053	3.254	3.254	4.269	1.312	1.702196
Frmd5	Frmd5-007	308.9	0.022342	0.052	3.254	3.254	2.906	0.893	1.702042
Selo	Selo-001	1178.8	0.003015	0.011	3.253	3.253	7.625	2.344	1.70156
Anpep	Anpep-001	11052.0	0.000332	0.002	3.250	3.250	75.022	23.082	1.700527
Map4k5	Map4k5-001	1438.0	0.000860	0.004	3.249	3.249	9.091	2.798	1.700151
Gria3	Gria3-005	1455.9	0.004249	0.014	3.249	3.249	9.132	2.810	1.700142
Gm15204	Gm15204-001	264.0	0.020000	0.048	3.248	3.248	1.888	0.581	1.699486
Snapc4	Snapc4-001	1191.7	0.000974	0.004	3.247	3.247	10.176	3.134	1.699243
Gli1	Gli1-201	339.0	0.010101	0.028	3.245	3.245	2.158	0.665	1.698113
Gm13204	Gm13204-001	279.0	0.044635	0.090	3.242	3.242	1.568	0.484	1.697084
Eif4h	Eif4h-004	1694.0	0.000175	0.001	3.241	3.241	10.969	3.384	1.696495
Szrd1	Szrd1-003	2261.7	0.004471	0.015	3.238	3.238	17.111	5.284	1.695316
Sptbn5	Sptbn5-002	1220.0	0.000684	0.003	3.235	3.235	6.812	2.106	1.693574
Ube2i	Ube2i-013	371.4	0.017088	0.042	3.232	3.232	2.816	0.871	1.692646
Cul3	Cul3-006	396.4	0.036109	0.076	3.230	3.230	2.843	0.880	1.691664
Efemp2	Efemp2-001	542.4	0.046384	0.092	3.229	3.229	4.707	1.457	1.691302
Ggact	Ggact-001	984.8	0.002756	0.010	3.223	3.223	8.181	2.538	1.688432
Rnf25	Rnf25-002	993.4	0.012926	0.034	3.222	3.222	6.316	1.960	1.687806
Man1c1	Man1c1-002	775.5	0.023104	0.053	3.221	3.221	6.316	1.961	1.687546
Pak6	Pak6-002	220.4	0.039917	0.082	3.217	3.217	1.264	0.393	1.685619
Srrt	Srrt-201	3334.4	0.002076	0.008	3.216	3.216	22.076	6.864	1.685428
N4bp2l2	N4bp2l2-008	289.4	0.022085	0.051	3.216	3.216	1.474	0.458	1.685151
Flcn	Flcn-004	1097.1	0.032215	0.069	3.215	3.215	7.346	2.285	1.684872
Arl6ip5	Arl6ip5-001	9692.0	0.000001	0.000	3.213	3.213	57.910	18.022	1.684085
Srgap2	Srgap2-004	7475.3	0.000030	0.000	3.213	3.213	46.135	14.360	1.683785
Grin2d	Grin2d-001	640.6	0.018098	0.044	3.212	3.212	3.333	1.038	1.683389
A4galt	A4galt-202	1028.2	0.003721	0.013	3.204	3.204	8.380	2.615	1.680095
Pfdn6	Pfdn6-001	685.2	0.014419	0.037	3.204	3.204	5.319	1.660	1.679944
00097C17R	0097C17Rik-	2787.0	0.000006	0.000	3.202	3.202	18.840	5.883	1.679091
Zfp512	Zfp512-005	321.6	0.009523	0.027	3.201	3.201	2.288	0.715	1.678521
Gm15288	Gm15288-001	375.0	0.006270	0.019	3.199	3.199	2.629	0.822	1.677792
Hk1	Hk1-014	8012.5	0.000257	0.002	3.198	3.198	48.764	15.249	1.677108
Hcfc1r1	Hcfc1r1-002	2119.6	0.000808	0.004	3.196	3.196	9.794	3.065	1.676226
Serf2	Serf2-006	4709.6	0.000698	0.003	3.195	3.195	33.592	10.512	1.676007
Gas2l1	Gas2l1-007	514.7	0.009060	0.026	3.193	3.193	3.026	0.948	1.674844
Zfp384	Zfp384-010	1631.5	0.015859	0.040	3.188	3.188	7.518	2.358	1.672515
Pgm5	Pgm5-002	348.5	0.016313	0.040	3.186	3.186	2.701	0.848	1.671801
Crispld1	Crispld1-002	1687.7	0.032939	0.070	3.184	3.184	4.106	1.289	1.670974
Palld	Palld-003	8481.6	0.015404	0.039	3.183	3.183	62.474	19.627	1.670396
Usp54	Usp54-201	10275.5	0.001015	0.005	3.181	3.181	85.726	26.953	1.669275
Kcnj16	Kcnj16-001	442.4	0.002840	0.010	3.180	3.180	2.920	0.918	1.669203
Kcnj16	Kcnj16-002	273.8	0.009760	0.027	3.180	3.180	1.808	0.568	1.669136
Kcnj16	Kcnj16-201	255.4	0.011479	0.031	3.180	3.180	1.686	0.530	1.669135
Kcnj16	Kcnj16-202	203.4	0.018933	0.046	3.180	3.180	1.343	0.422	1.669052
Tgm2	Tgm2-003	1255.2	0.007858	0.023	3.180	3.180	4.680	1.472	1.668908
Gm7909	Gm7909-001	590.0	0.022588	0.052	3.178	3.178	4.407	1.387	1.668191
Per2	Per2-001	14153.1	0.013059	0.034	3.176	3.176	89.811	28.279	1.667186
Actn1	Actn1-202	35885.8	0.000002	0.000	3.175	3.175	249.398	78.553	1.666712
Pnpla7	Pnpla7-009	365.0	0.016387	0.041	3.173	3.173	2.889	0.910	1.665897
Limch1	Limch1-004	6034.1	0.017616	0.043	3.171	3.171	38.972	12.289	1.665081
Sertad3	Sertad3-201	1218.0	0.001316	0.006	3.171	3.171	7.773	2.451	1.664781
Frmd3	Frmd3-001	481.0	0.011100	0.030	3.170	3.170	3.054	0.963	1.664675
Acvrl1	Acvrl1-001	5448.5	0.000748	0.004	3.170	3.170	26.411	8.330	1.66467
Plcb1	Plcb1-007	196.4	0.028062	0.062	3.170	3.170	1.332	0.420	1.664658
Iqce	Iqce-002	924.5	0.013970	0.036	3.167	3.167	5.524	1.744	1.663276
Rin3	Rin3-001	5552.2	0.000636	0.003	3.164	3.164	34.501	10.903	1.661898
Sema3f	Sema3f-012	8104.8	0.000877	0.004	3.161	3.161	32.818	10.381	1.660518
Snrk	Snrk-006	1458.9	0.004701	0.015	3.161	3.161	9.191	2.908	1.660377
Plec	Plec-203	8457.3	0.000146	0.001	3.159	3.159	32.476	10.280	1.659563
Ppp1r15a	Ppp1r15a-001	2070.3	0.002404	0.009	3.158	3.158	12.652	4.007	1.658935
Sbno1	Sbno1-013	729.2	0.021510	0.050	3.155	3.155	5.711	1.810	1.657841
Nkx2-3	Nkx2-3-001	1425.0	0.047508	0.094	3.155	3.155	9.361	2.968	1.657426
Alas1	Alas1-001	1835.6	0.047302	0.094	3.153	3.153	12.891	4.088	1.656781
Ntm	Ntm-001	1040.0	0.001899	0.008	3.153	3.153	7.026	2.228	1.656723
Zbtb38	Zbtb38-005	484.5	0.024853	0.056	3.152	3.152	3.794	1.204	1.65624
Lrg1	Lrg1-201	4796.0	0.042914	0.087	3.151	3.151	37.025	11.750	1.655879
Rps6-ps2	Rps6-ps2-201	178.0	0.040037	0.082	3.147	3.147	1.410	0.448	1.65397
Arfrp1	Arfrp1-004	478.2	0.023554	0.054	3.145	3.145	2.997	0.953	1.65324
Lpl	Lpl-001	6969.9	0.000031	0.000	3.144	3.144	49.845	15.856	1.652458
Gsn	Gsn-001	31207.8	0.000090	0.001	3.141	3.141	150.834	48.027	1.651057
Dbnl	Dbnl-001	3404.2	0.002922	0.011	3.140	3.140	20.317	6.470	1.65082
Actn1	Actn1-201	45852.6	0.000001	0.000	3.138	3.138	329.578	105.034	1.649767
Lincpint	Lincpint-003	333.7	0.039171	0.081	3.134	3.134	2.892	0.923	1.648051
Eln	Eln-005	49564.1	0.000204	0.001	3.133	3.133	210.873	67.307	1.647548
Vwf	Vwf-201	126195.4	0.000304	0.002	3.129	3.129	930.153	297.270	1.645696
Ier2	Ier2-201	7860.0	0.002859	0.010	3.127	3.127	54.303	17.366	1.644735
P23-370G17	23-370G17.3-	191.0	0.031211	0.067	3.127	3.127	1.638	0.524	1.644652

Zfp13	Zfp13-202	831.6	0.015022	0.038	3.126	3.126	6.652	2.128	1.644264
Acp6	Acp6-003	286.6	0.042357	0.086	3.124	3.124	2.052	0.657	1.643586
Tmem8b	Tmem8b-001	1912.4	0.008647	0.025	3.123	3.123	9.698	3.106	1.642823
Gas5	Gas5-025	794.6	0.042345	0.086	3.121	3.121	7.059	2.262	1.641969
Eif4a1	Eif4a1-009	643.1	0.009611	0.027	3.119	3.119	5.032	1.613	1.641172
Pcnp	Pcnp-005	626.9	0.020858	0.049	3.119	3.119	4.000	1.283	1.641031
Dnajb12	Dnajb12-003	1592.5	0.006735	0.020	3.118	3.118	10.840	3.476	1.640766
Shank3	Shank3-005	956.0	0.001090	0.005	3.117	3.117	5.497	1.764	1.640097
Spata13	Spata13-001	624.6	0.015824	0.039	3.117	3.117	3.131	1.005	1.639967
Adh1	Adh1-001	18663.9	0.010425	0.029	3.115	3.115	126.209	40.515	1.639301
Tbx1	Tbx1-201	5886.0	0.002191	0.008	3.115	3.115	39.531	12.692	1.639098
Snhg12	Snhg12-007	2633.4	0.000876	0.004	3.114	3.114	22.046	7.079	1.638965
Dynll2	Dynll2-001	3198.6	0.000910	0.004	3.110	3.110	24.209	7.783	1.637049
Kl	Kl-002	3140.3	0.000920	0.004	3.108	3.108	22.112	7.114	1.636037
Fmo2	Fmo2-001	20718.7	0.012781	0.033	3.106	3.106	167.703	53.988	1.635186
Dda1	Dda1-001	937.8	0.020269	0.048	3.101	3.101	8.418	2.715	1.63256
Atp2b4	Atp2b4-005	1159.2	0.001459	0.006	3.100	3.100	6.959	2.245	1.632215
Dnajc7	Dnajc7-003	705.7	0.002887	0.010	3.095	3.095	4.371	1.412	1.63013
Usp9x	Usp9x-002	1236.3	0.008134	0.023	3.095	3.095	6.915	2.234	1.629931
Mkrn1	Mkrn1-001	2960.8	0.000471	0.003	3.093	3.093	23.381	7.560	1.628908
Tsc22d1	Tsc22d1-001	12048.1	0.000095	0.001	3.093	3.093	67.951	21.971	1.628905
Gabarapl2	Gabarapl2-002	1143.0	0.023624	0.054	3.092	3.092	9.921	3.209	1.62834
Ift140	Ift140-001	2443.8	0.003755	0.013	3.091	3.091	16.241	5.254	1.628174
Cald1	Cald1-014	5751.5	0.006277	0.019	3.090	3.090	56.305	18.222	1.627597
Ctcf	Ctcf-006	564.6	0.006159	0.019	3.090	3.090	4.440	1.437	1.627444
Phc2	Phc2-008	986.4	0.005579	0.017	3.089	3.089	4.876	1.579	1.626978
Kank4	Kank4-001	3001.4	0.015570	0.039	3.087	3.087	16.051	5.200	1.626095
Trove2	Trove2-002	1488.9	0.003087	0.011	3.086	3.086	9.770	3.166	1.62567
Rpl10	Rpl10-010	1465.1	0.001728	0.007	3.082	3.082	12.479	4.049	1.623984
Eri3	Eri3-001	1153.1	0.016686	0.041	3.082	3.082	8.279	2.687	1.623649
Lrsam1	Lrsam1-001	1123.2	0.016862	0.042	3.080	3.080	7.175	2.330	1.622794
Plac9a	Plac9a-001	10210.8	0.001806	0.007	3.079	3.079	43.254	14.048	1.622517
Cnksr3	Cnksr3-001	1915.5	0.000770	0.004	3.079	3.079	12.978	4.215	1.622473
Map4	Map4-007	896.9	0.007614	0.022	3.077	3.077	4.053	1.317	1.621353
Map6	Map6-201	4137.4	0.000661	0.003	3.075	3.075	22.050	7.170	1.620763
Nabp2	Nabp2-002	1919.4	0.002451	0.009	3.075	3.075	12.450	4.049	1.620504
'00028E10R	0028E10Rik-	2168.9	0.004986	0.016	3.071	3.071	16.105	5.245	1.618555
Huwe1	Huwe1-003	3455.7	0.045779	0.091	3.068	3.068	29.626	9.656	1.617309
Pam	Pam-008	10355.8	0.004115	0.014	3.068	3.068	70.741	23.059	1.617243
Kdm2a	Kdm2a-008	1490.5	0.005548	0.017	3.067	3.067	9.636	3.142	1.617039
Crebrf	Crebrf-002	3598.0	0.000345	0.002	3.067	3.067	26.618	8.678	1.616966
Cdkn2c	Cdkn2c-001	562.9	0.010268	0.028	3.067	3.067	3.656	1.192	1.616917
Plac9a	Plac9a-002	1268.7	0.001727	0.007	3.066	3.066	6.209	2.025	1.616433
Gm12715	Gm12715-001	22735.0	0.000289	0.002	3.064	3.064	157.340	51.356	1.615283
Carkd	Carkd-001	1052.0	0.036480	0.076	3.061	3.061	7.251	2.369	1.614152
Cenpb	Cenpb-001	16242.0	0.000000	0.000	3.057	3.057	108.128	35.365	1.612337
Smad6	Smad6-001	11062.9	0.000653	0.003	3.057	3.057	82.739	27.068	1.61198
Hist1h4i	Hist1h4i-001	195.0	0.042620	0.086	3.051	3.051	1.763	0.578	1.609487
Gm15666	Gm15666-001	877.0	0.001514	0.006	3.050	3.050	5.116	1.677	1.608851
Gm7114	Gm7114-001	256.0	0.022125	0.051	3.046	3.046	2.210	0.725	1.606875
Uchl1	Uchl1-001	579.5	0.009338	0.026	3.045	3.045	4.552	1.495	1.606561
Nckap5l	Nckap5l-001	2829.1	0.004645	0.015	3.044	3.044	21.530	7.073	1.605898
Man1a2	Man1a2-003	690.9	0.015566	0.039	3.042	3.042	3.901	1.282	1.604862
Cuedc1	Cuedc1-001	3169.8	0.002672	0.010	3.042	3.042	15.515	5.101	1.604805
Spopl	Spopl-002	539.8	0.047336	0.094	3.041	3.041	2.714	0.892	1.604768
Mapk8ip1	Mapk8ip1-001	1533.6	0.026366	0.059	3.041	3.041	9.833	3.234	1.604392
Prrt4	Prrt4-001	7712.0	0.017570	0.043	3.037	3.037	16.399	5.400	1.602519
Slc4a2	Slc4a2-005	601.4	0.027822	0.061	3.036	3.036	3.811	1.255	1.602264
Jup	Jup-007	755.5	0.029024	0.064	3.028	3.028	2.993	0.989	1.5982
Stx8	Stx8-003	806.4	0.006922	0.021	3.026	3.026	5.737	1.896	1.597483
Gps2	Gps2-001	2289.2	0.003265	0.011	3.023	3.023	18.913	6.255	1.596182
Shfm1	Shfm1-001	10532.0	0.000009	0.000	3.023	3.023	72.379	23.944	1.595914
Nrip1	Nrip1-003	6866.8	0.004920	0.016	3.022	3.022	43.754	14.477	1.595652
Rarg	Rarg-002	4136.7	0.004179	0.014	3.022	3.022	29.050	9.615	1.595268
Rtn1	Rtn1-001	2157.0	0.007851	0.023	3.020	3.020	15.170	5.023	1.594499
Dyrk1a	Dyrk1a-001	4029.2	0.006285	0.019	3.019	3.019	25.026	8.289	1.594086
Plec	Plec-001	20686.7	0.000126	0.001	3.018	3.018	86.894	28.788	1.593776
Gpr17	Gpr17-001	6620.2	0.005852	0.018	3.016	3.016	24.114	7.994	1.592844
Slc12a4	Slc12a4-001	8135.1	0.000001	0.000	3.016	3.016	53.549	17.756	1.592587
Drosha	Drosha-001	7099.2	0.010335	0.028	3.011	3.011	38.303	12.720	1.590339
Stbd1	Stbd1-002	2319.0	0.004991	0.016	3.010	3.010	15.222	5.058	1.589532
Cacng2	Cacng2-001	347.0	0.012944	0.034	3.004	3.004	2.307	0.768	1.58696
Gata2	Gata2-201	19097.9	0.000152	0.001	2.999	2.999	116.010	38.684	1.584432
P24-80L24.	24-80L24.2-0	879.0	0.004830	0.016	2.999	2.999	6.463	2.155	1.584347
Bahcc1	Bahcc1-201	1625.8	0.032359	0.069	2.999	2.999	12.250	4.085	1.584327
Samd1	Samd1-201	1840.0	0.003076	0.011	2.998	2.998	13.764	4.592	1.58382
Lrrc61	Lrrc61-003	1687.8	0.001338	0.006	2.997	2.997	13.964	4.658	1.583736
Zmynd11	Zmynd11-007	5098.6	0.000294	0.002	2.995	2.995	31.897	10.648	1.582753
Gm10076	Gm10076-201	23384.0	0.000005	0.000	2.994	2.994	179.766	60.052	1.581849
Cds2	Cds2-001	16672.6	0.000001	0.000	2.993	2.993	105.538	35.262	1.581592
Gbp7	Gbp7-002	2213.5	0.035197	0.074	2.992	2.992	11.920	3.983	1.581289
Tspan4	Tspan4-001	1946.5	0.009125	0.026	2.991	2.991	16.568	5.539	1.580581
Pgm2	Pgm2-001	1998.9	0.032040	0.069	2.990	2.990	14.130	4.726	1.580186
Gxylt1	Gxylt1-002	561.7	0.026520	0.059	2.989	2.989	3.567	1.193	1.579781
Anapc1	Anapc1-009	431.1	0.025337	0.057	2.986	2.986	2.077	0.695	1.578384
Wibg	Wibg-202	705.6	0.012427	0.033	2.983	2.983	5.963	1.999	1.576692
Amotl2	Amotl2-003	1180.7	0.003483	0.012	2.982	2.982	10.586	3.550	1.576451
Arid3b	Arid3b-201	1153.5	0.024807	0.056	2.980	2.980	8.176	2.744	1.575142
Mtcp1	Mtcp1-004	384.0	0.010475	0.029	2.980	2.980	3.430	1.151	1.57514
Anp32e	Anp32e-007	2269.8	0.016705	0.041	2.979	2.979	14.979	5.029	1.574768
Brd3	Brd3-202	3155.1	0.006606	0.020	2.976	2.976	23.492	7.894	1.573388
Adam15	Adam15-004	1741.3	0.029941	0.065	2.974	2.974	6.144	2.066	1.572388
Zfp92	Zfp92-001	202.0	0.027433	0.061	2.972	2.972	1.717	0.578	1.571504
Mid2	Mid2-005	1118.2	0.026555	0.059	2.970	2.970	7.553	2.543	1.570228
Cenpc1	Cenpc1-002	6606.4	0.000006	0.000	2.969	2.969	39.415	13.274	1.570153
Kif7	Kif7-001	287.8	0.032977	0.070	2.969	2.969	1.965	0.662	1.570024
Gm25000	Gm25000-201	652.0	0.012551	0.033	2.968	2.968	3.983	1.342	1.569311
Tm4sf1	Tm4sf1-002	10455.1	0.008352	0.024	2.963	2.963	102.441	34.571	1.567181
Inpp5b	Inpp5b-002	1911.0	0.014387	0.037	2.962	2.962	11.304	3.816	1.566725
Etv6	Etv6-004	994.1	0.037398	0.078	2.962	2.962	6.925	2.338	1.566415
Fbxw7	Fbxw7-001	2622.8	0.025254	0.057	2.953	2.953	13.285	4.499	1.562304
Ryk	Ryk-003	2392.0	0.001827	0.007	2.953	2.953	15.366	5.204	1.562075
Klf2	Klf2-001	35822.0	0.027764	0.061	2.952	2.952	69.593	23.573	1.561837
Tead4	Tead4-001	2274.6	0.016609	0.041	2.952	2.952	15.462	5.238	1.561639
30403L08R	0403L08Rik-	735.7	0.003284	0.012	2.952	2.952	4.299	1.457	1.561542
Mir143hg	Mir143hg-002	313.7	0.033060	0.071	2.952	2.952	1.901	0.644	1.561502
Samhd1	Samhd1-009	837.5	0.021254	0.050	2.948	2.948	7.213	2.446	1.559793

Tspan11	Tspan11-001	3698.8	0.010424	0.029	2.948	2.948	8.884	3.014	1.559732
Trim14	Trim14-003	599.2	0.012489	0.033	2.947	2.947	4.388	1.489	1.559168
Hmgb1-ps9	Hmgb1-ps9-20	477.0	0.003926	0.013	2.944	2.944	3.881	1.318	1.557592
Rnpepl1	Rnpepl1-006	583.8	0.026186	0.059	2.941	2.941	3.499	1.190	1.556304
Osmr	Osmr-005	1146.9	0.023419	0.054	2.941	2.941	9.251	3.146	1.556088
Ssu72	Ssu72-002	777.3	0.039326	0.081	2.939	2.939	5.634	1.917	1.555511
Rps15-ps2	Rps15-ps2-00	394.0	0.008559	0.024	2.939	2.939	2.974	1.012	1.555175
Tagln	Tagln-201	16895.0	0.003746	0.013	2.936	2.936	194.773	66.339	1.553858
Timp4	Timp4-201	924.0	0.025626	0.058	2.936	2.936	4.414	1.504	1.553745
Btf3	Btf3-004	4445.5	0.000265	0.002	2.936	2.936	32.777	11.165	1.553664
Abca7	Abca7-001	2921.5	0.016856	0.042	2.932	2.932	16.087	5.487	1.55174
Safb2	Safb2-010	603.1	0.016990	0.042	2.926	2.926	4.463	1.525	1.549139
Slc3a2	Slc3a2-201	20338.1	0.000500	0.003	2.923	2.923	176.429	60.358	1.547483
Gm26573	Gm26573-201	3411.0	0.002087	0.008	2.923	2.923	25.164	8.609	1.547448
Ptma	Ptma-005	1501.0	0.018281	0.044	2.922	2.922	10.276	3.517	1.546787
Srebf1	Srebf1-005	4662.7	0.000320	0.002	2.920	2.920	40.626	13.914	1.545849
Plcd3	Plcd3-001	4676.3	0.001049	0.005	2.918	2.918	32.170	11.026	1.544798
Btbd19	Btbd19-007	257.6	0.031709	0.068	2.915	2.915	1.609	0.552	1.543743
Rps19	Rps19-007	4186.4	0.028177	0.062	2.914	2.914	37.867	12.995	1.542932
Arhgap22	Arhgap22-001	797.3	0.016938	0.042	2.913	2.913	6.810	2.337	1.542724
Nlgn3	Nlgn3-002	1336.5	0.001871	0.007	2.913	2.913	8.851	3.039	1.542304
Akap8l	Akap8l-201	6453.0	0.000832	0.004	2.912	2.912	40.234	13.818	1.541876
Slc43a2	Slc43a2-201	648.8	0.042163	0.086	2.909	2.909	7.330	2.520	1.540579
Casc3	Casc3-003	1304.5	0.033881	0.072	2.909	2.909	9.125	3.137	1.540547
Rnf39	Rnf39-002	380.9	0.044427	0.089	2.908	2.908	2.062	0.709	1.540183
Clk1	Clk1-013	1139.7	0.013223	0.034	2.907	2.907	6.959	2.394	1.539629
Agpat3	Agpat3-201	1322.1	0.037549	0.078	2.907	2.907	8.900	3.062	1.539297
Shank3	Shank3-001	8115.8	0.001522	0.006	2.905	2.905	37.679	12.972	1.538376
Tufm	Tufm-202	924.3	0.014597	0.037	2.904	2.904	6.186	2.131	1.537878
Dus1l	Dus1l-201	1075.3	0.016138	0.040	2.904	2.904	7.348	2.531	1.537826
Bad	Bad-002	900.2	0.004494	0.015	2.902	2.902	5.964	2.055	1.537067
Nfe2l1	Nfe2l1-201	16615.1	0.001319	0.006	2.902	2.902	96.353	33.208	1.536804
Trp53i13	Trp53i13-001	713.2	0.012303	0.032	2.900	2.900	3.792	1.307	1.536296
Trf	Trf-009	859.9	0.009282	0.026	2.899	2.899	6.658	2.297	1.535417
Ssfa2	Ssfa2-004	17548.5	0.000269	0.002	2.898	2.898	112.070	38.677	1.534852
Atp6v1a	Atp6v1a-002	2509.0	0.009165	0.026	2.897	2.897	17.830	6.154	1.534684
Arid4b	Arid4b-007	408.2	0.029248	0.064	2.889	2.889	3.101	1.073	1.530511
Rpl7a	Rpl7a-004	4691.7	0.001869	0.007	2.885	2.885	40.558	14.056	1.528791
Itpr3	Itpr3-001	49112.3	0.000003	0.000	2.885	2.885	263.857	91.449	1.528711
Rap1gap2	Rap1gap2-201	3482.3	0.000022	0.000	2.885	2.885	23.544	8.160	1.528633
Itga3	Itga3-004	1116.9	0.048064	0.095	2.885	2.885	9.741	3.377	1.528362
Per1	Per1-201	3351.5	0.015690	0.039	2.884	2.884	22.181	7.690	1.528258
Shroom3	Shroom3-005	12765.2	0.000238	0.002	2.883	2.883	88.899	30.840	1.52737
Tead1	Tead1-009	619.3	0.013069	0.034	2.882	2.882	4.102	1.423	1.527286
Vwf	Vwf-002	4086.5	0.001861	0.007	2.880	2.880	27.610	9.588	1.525938
Mbd2	Mbd2-005	3797.4	0.000240	0.002	2.877	2.877	29.756	10.341	1.524759
Sp110	Sp110-001	7575.5	0.000945	0.004	2.877	2.877	36.315	12.623	1.524509
Trmt112	Trmt112-002	1325.6	0.015014	0.038	2.872	2.872	7.580	2.639	1.521977
Srp14	Srp14-001	1520.4	0.024715	0.056	2.869	2.869	11.267	3.927	1.520568
Cog7	Cog7-001	2812.9	0.000078	0.001	2.863	2.863	18.501	6.462	1.51755
Zfp651	Zfp651-201	2077.0	0.000111	0.001	2.862	2.862	15.019	5.248	1.517048
Mark4	Mark4-201	3976.0	0.000049	0.000	2.856	2.856	22.083	7.732	1.514021
Fbxl17	Fbxl17-003	117.2	0.024384	0.055	2.852	2.852	0.651	0.228	1.512076
Faf2	Faf2-006	2315.9	0.010696	0.029	2.850	2.850	15.825	5.552	1.511166
Athl1	Athl1-003	975.8	0.011165	0.030	2.849	2.849	8.642	3.033	1.510656
Fmo2	Fmo2-003	2215.0	0.005496	0.017	2.848	2.848	20.795	7.301	1.510076
Ppp2r4	Ppp2r4-001	2447.4	0.005835	0.018	2.845	2.845	16.711	5.873	1.508521
Capn1	Capn1-201	2982.5	0.000774	0.004	2.844	2.844	15.977	5.618	1.507802
Khdrbs1	Khdrbs1-001	6503.7	0.001770	0.007	2.840	2.840	45.153	15.900	1.505809
Dcaf8	Dcaf8-001	6413.1	0.001059	0.005	2.839	2.839	43.198	15.218	1.505197
Bco1	Bco1-001	1075.9	0.009670	0.027	2.839	2.839	6.008	2.117	1.505135
Firre	Firre-001	4421.5	0.000969	0.004	2.837	2.837	28.227	9.949	1.504411
Chrac1	Chrac1-001	1853.3	0.000967	0.004	2.836	2.836	14.638	5.162	1.503755
Abcg2	Abcg2-004	1344.2	0.021767	0.051	2.835	2.835	8.053	2.840	1.50357
Crtc2	Crtc2-001	1625.3	0.010030	0.028	2.834	2.834	10.158	3.584	1.502904
Agpat3	Agpat3-005	663.3	0.027457	0.061	2.834	2.834	5.443	1.921	1.502881
Zfp280d	Zfp280d-002	1982.9	0.003371	0.012	2.833	2.833	11.181	3.947	1.502141
Map2k7	Map2k7-001	1595.7	0.007171	0.021	2.832	2.832	10.016	3.536	1.502015
Rps16	Rps16-002	3097.7	0.013468	0.035	2.830	2.830	26.880	9.498	1.500774
Dgcr8	Dgcr8-002	798.1	0.016756	0.041	2.829	2.829	5.534	1.956	1.500094
Slc25a10	Slc25a10-001	897.3	0.004309	0.014	2.828	2.828	5.667	2.004	1.499771
4-Sep	Sept4-003	731.8	0.025436	0.057	2.826	2.826	8.102	2.867	1.498755
Rbm19	Rbm19-001	1459.1	0.025740	0.058	2.825	2.825	10.587	3.747	1.498367
Arhgap44	Arhgap44-004	4064.4	0.000173	0.001	2.824	2.824	29.489	10.441	1.49794
Slc20a1	Slc20a1-004	1056.7	0.007196	0.021	2.822	2.822	7.454	2.641	1.496905
Tap2	Tap2-002	4899.6	0.000239	0.002	2.822	2.822	32.231	11.423	1.496514
Tubb2b	Tubb2b-201	4986.0	0.000045	0.000	2.821	2.821	39.023	13.834	1.496078
Chd4	Chd4-006	3295.1	0.014489	0.037	2.818	2.818	20.955	7.435	1.494883
Mapre3	Mapre3-006	956.8	0.014709	0.037	2.817	2.817	5.936	2.107	1.49434
Sptbn5	Sptbn5-003	540.0	0.021849	0.051	2.817	2.817	3.593	1.275	1.494021
P24-164P24	24-164P24.5-	517.0	0.010932	0.030	2.815	2.815	2.318	0.824	1.493164
Cables1	Cables1-202	3063.3	0.000497	0.003	2.813	2.813	22.097	7.856	1.492091
Krba1	Krba1-002	821.8	0.049468	0.097	2.812	2.812	4.379	1.557	1.491482
Myo6	Myo6-001	2520.2	0.001037	0.005	2.811	2.811	17.026	6.057	1.491059
Tbc1d14	Tbc1d14-006	786.0	0.028400	0.062	2.809	2.809	5.660	2.015	1.49012
Hmgb1-ps2	Hmgb1-ps2-00	2303.0	0.000232	0.001	2.809	2.809	17.059	6.074	1.489807
Mon1b	Mon1b-201	2239.6	0.013447	0.035	2.808	2.808	14.903	5.307	1.489761
Wars	Wars-001	2390.5	0.013187	0.034	2.807	2.807	17.429	6.208	1.489187
Smarcc2	Smarcc2-203	4456.3	0.020096	0.048	2.806	2.806	39.969	14.243	1.488624
Smc1a	Smc1a-006	419.0	0.041806	0.085	2.803	2.803	2.785	0.993	1.487098
Akr7a5	Akr7a5-001	651.2	0.018893	0.045	2.803	2.803	5.220	1.863	1.48672
Sptb	Sptb-001	393.9	0.016228	0.040	2.801	2.801	2.721	0.972	1.48577
Atp6v0a1	Atp6v0a1-001	1029.2	0.024097	0.055	2.801	2.801	5.384	1.923	1.485702
30042M11	0042M11Rik-	760.0	0.003055	0.011	2.801	2.801	5.279	1.885	1.485702
Nfix	Nfix-002	2339.8	0.003053	0.011	2.799	2.799	17.118	6.115	1.484977
Plec	Plec-202	19174.3	0.000179	0.001	2.798	2.798	78.375	28.012	1.484339
Pygo1	Pygo1-001	802.0	0.012625	0.033	2.798	2.798	5.302	1.895	1.484183
Tom1	Tom1-202	696.9	0.005278	0.017	2.796	2.796	5.126	1.833	1.48355
Smad7	Smad7-001	17932.3	0.000513	0.003	2.796	2.796	124.016	44.357	1.483285
Ppan	Ppan-001	790.2	0.028091	0.062	2.794	2.794	8.012	2.867	1.48249
Ccdc109b	Ccdc109b-001	969.3	0.032284	0.069	2.794	2.794	6.496	2.325	1.482219
Zyx	Zyx-202	10742.6	0.005070	0.016	2.794	2.794	72.105	25.810	1.482181
Kdm4a	Kdm4a-001	3544.9	0.013255	0.034	2.793	2.793	19.864	7.112	1.481821
Dzip1l	Dzip1l-001	323.9	0.047668	0.094	2.787	2.787	2.355	0.845	1.478653
Palm	Palm-201	4479.4	0.001598	0.007	2.786	2.786	16.321	5.858	1.478313
Lfng	Lfng-003	1754.2	0.009290	0.026	2.786	2.786	9.011	3.235	1.478135
Rpl23a	Rpl23a-001	12639.2	0.000114	0.001	2.784	2.784	105.436	37.877	1.476983

Abcd1	Abcd1-002	324.5	0.033076	0.071	2.783	2.783	2.021	0.726	1.476523
Gm2260	Gm2260-002	2930.3	0.000185	0.001	2.780	2.780	17.384	6.252	1.475289
Bag6	Bag6-001	2949.1	0.002713	0.010	2.779	2.779	18.172	6.539	1.474543
Smtn	Smtn-001	5866.3	0.021546	0.050	2.778	2.778	51.256	18.448	1.474248
Sulf2	Sulf2-007	354.3	0.029795	0.065	2.777	2.777	1.701	0.612	1.473703
Eif4enif1	Eif4enif1-001	1538.7	0.011369	0.030	2.777	2.777	11.920	4.293	1.473533
Wdr13	Wdr13-007	420.4	0.022491	0.052	2.777	2.777	2.545	0.917	1.473336
Pank4	Pank4-001	1267.6	0.019918	0.047	2.776	2.776	9.694	3.492	1.472873
Mboat7	Mboat7-001	825.0	0.025044	0.057	2.775	2.775	5.523	1.990	1.472534
Gm16437	Gm16437-201	407.0	0.010546	0.029	2.774	2.774	2.798	1.009	1.472107
Klf16	Klf16-001	1026.0	0.029517	0.064	2.772	2.772	7.217	2.604	1.470682
Tulp4	Tulp4-009	1764.1	0.003435	0.012	2.771	2.771	8.899	3.212	1.470237
Mllt6	Mllt6-001	11898.1	0.000079	0.001	2.769	2.769	69.896	25.242	1.46938
Foxo4	Foxo4-001	2219.0	0.000234	0.001	2.768	2.768	14.531	5.250	1.468788
Gipc2	Gipc2-008	492.0	0.008528	0.024	2.767	2.767	3.530	1.276	1.46845
Plin4	Plin4-201	840.9	0.046881	0.093	2.765	2.765	2.870	1.038	1.467404
Tnfsfm13	Tnfsfm13-003	1515.3	0.003492	0.012	2.764	2.764	10.247	3.708	1.466617
Cul7	Cul7-003	1656.9	0.013841	0.036	2.763	2.763	13.450	4.868	1.466173
Ubc	Ubc-001	64806.2	0.000008	0.000	2.759	2.759	454.286	164.678	1.463955
Efnb1	Efnb1-001	1052.4	0.017319	0.042	2.757	2.757	5.728	2.078	1.463033
Parp9	Parp9-001	2960.0	0.000875	0.004	2.757	2.757	21.520	7.807	1.462852
Ibtk	Ibtk-003	2444.6	0.029042	0.064	2.756	2.756	19.154	6.949	1.462695
Eif4a2	Eif4a2-016	7493.1	0.000146	0.001	2.755	2.755	50.147	18.202	1.462066
Flt4	Flt4-002	529.4	0.018472	0.045	2.753	2.753	3.432	1.247	1.460848
Kdm4b	Kdm4b-002	1308.1	0.044672	0.090	2.752	2.752	10.286	3.737	1.460674
Caml	Caml-001	846.5	0.011702	0.031	2.752	2.752	6.321	2.297	1.460425
Npr1	Npr1-001	21371.6	0.000055	0.001	2.749	2.749	129.952	47.271	1.458946
Rgs3	Rgs3-013	1191.0	0.021290	0.050	2.743	2.743	7.933	2.892	1.455728
Cap1	Cap1-007	329.0	0.044045	0.089	2.738	2.738	2.911	1.063	1.453168
Pkd2	Pkd2-003	1438.6	0.002293	0.009	2.736	2.736	12.106	4.424	1.45224
Fars2	Fars2-201	1995.3	0.000545	0.003	2.735	2.735	11.873	4.341	1.451789
Ndufa6	Ndufa6-201	4686.0	0.000403	0.002	2.734	2.734	33.365	12.203	1.451071
Zfp771	Zfp771-001	553.0	0.024425	0.055	2.734	2.734	4.313	1.578	1.450876
Stx12	Stx12-002	757.3	0.012288	0.032	2.732	2.732	6.135	2.245	1.450143
Igfbp5	Igfbp5-001	5770.3	0.014288	0.036	2.732	2.732	19.197	7.027	1.449974
Scarf2	Scarf2-201	3507.0	0.000144	0.001	2.732	2.732	23.029	8.430	1.44995
Fbxl5	Fbxl5-008	674.6	0.027157	0.060	2.731	2.731	5.082	1.861	1.449336
Arhgef1	Arhgef1-012	783.4	0.021369	0.050	2.729	2.729	5.055	1.853	1.448136
Plec	Plec-006	17877.1	0.000104	0.001	2.726	2.726	71.200	26.120	1.446718
Fam107a	Fam107a-001	38076.7	0.024623	0.056	2.723	2.723	157.526	57.842	1.4454
Upf1	Upf1-201	9653.0	0.000004	0.000	2.723	2.723	63.812	23.435	1.44514
Tomm34	Tomm34-002	1331.0	0.003667	0.013	2.722	2.722	8.415	3.091	1.444869
Plec	Plec-003	34884.1	0.000655	0.003	2.721	2.721	147.285	54.120	1.444382
Acbd6	Acbd6-003	539.5	0.030120	0.066	2.720	2.720	3.882	1.427	1.443821
Ppp1r15a	Ppp1r15a-002	10756.3	0.006108	0.019	2.720	2.720	75.413	27.727	1.44354
Mical2	Mical2-004	1736.9	0.009648	0.027	2.718	2.718	12.192	4.485	1.442646
Clip1	Clip1-013	1448.7	0.019638	0.047	2.716	2.716	8.042	2.962	1.441236
Dap	Dap-004	615.3	0.025975	0.058	2.713	2.713	3.369	1.242	1.439913
'00084E18R	0084E18Rik-	557.6	0.019050	0.046	2.712	2.712	4.522	1.667	1.43956
Rbm43	Rbm43-001	1286.7	0.010299	0.028	2.712	2.712	5.824	2.147	1.439351
Sfxn3	Sfxn3-201	8536.2	0.001065	0.005	2.712	2.712	45.524	16.788	1.439197
'23-265F20.	.3-265F20.14-	519.3	0.026039	0.058	2.709	2.709	2.481	0.916	1.437559
Ktn1	Ktn1-009	1080.6	0.035553	0.075	2.706	2.706	7.643	2.824	1.436308
Ypel3	Ypel3-008	303.9	0.046935	0.093	2.706	2.706	1.864	0.689	1.436167
Rhoj	Rhoj-004	903.3	0.003404	0.012	2.704	2.704	5.067	1.874	1.434998
Sbno2	Sbno2-201	12622.0	0.001937	0.008	2.704	2.704	104.016	38.474	1.434851
Trerf1	Trerf1-005	3597.5	0.000086	0.001	2.703	2.703	14.455	5.348	1.434375
Tmbim6	Tmbim6-003	513.4	0.039319	0.081	2.701	2.701	2.103	0.779	1.43362
Cyb5d1	Cyb5d1-002	975.7	0.001686	0.007	2.701	2.701	6.697	2.480	1.433415
Fam3c	Fam3c-006	812.6	0.005072	0.016	2.700	2.700	5.883	2.179	1.43299
Bahcc1	Bahcc1-001	2808.6	0.006908	0.021	2.700	2.700	16.207	6.003	1.432791
Gm15716	Gm15716-001	1655.0	0.001134	0.005	2.695	2.695	11.035	4.094	1.430339
Tcf7l1	Tcf7l1-003	4775.9	0.000426	0.002	2.695	2.695	24.562	9.115	1.430155
Prpf31	Prpf31-002	564.6	0.020117	0.048	2.693	2.693	5.130	1.905	1.429238
Id1	Id1-001	4457.7	0.022238	0.052	2.693	2.693	16.898	6.275	1.429161
Gm8399	Gm8399-201	6793.0	0.001117	0.005	2.690	2.690	48.271	17.947	1.427416
Klc1	Klc1-002	5966.5	0.000051	0.000	2.689	2.689	29.810	11.085	1.427268
I30110L20R	0110L20Rik-	642.9	0.006799	0.020	2.688	2.688	2.996	1.115	1.426357
Tnks1bp1	Tnks1bp1-002	3285.6	0.035065	0.074	2.688	2.688	22.773	8.474	1.426289
Rps6	Rps6-003	1431.9	0.001298	0.006	2.686	2.686	11.727	4.367	1.425201
Nrp2	Nrp2-002	2108.8	0.000104	0.001	2.685	2.685	15.244	5.678	1.424804
Ssh3	Ssh3-201	2076.2	0.000661	0.003	2.685	2.685	14.256	5.310	1.424781
Cpeb2	Cpeb2-005	920.7	0.029524	0.064	2.684	2.684	5.410	2.016	1.424281
Cd200	Cd200-003	2209.5	0.031037	0.067	2.683	2.683	19.352	7.213	1.423743
Gm11209	Gm11209-001	540.0	0.011360	0.030	2.681	2.681	3.832	1.429	1.42256
Otud3	Otud3-001	873.0	0.007493	0.022	2.680	2.680	6.173	2.303	1.422302
Fbxo34	Fbxo34-202	2213.6	0.023226	0.053	2.679	2.679	7.475	2.790	1.421877
E2f1	E2f1-002	10929.9	0.000494	0.003	2.678	2.678	75.577	28.224	1.421011
Rnaseh2c	Rnaseh2c-001	798.3	0.026961	0.060	2.676	2.676	5.925	2.214	1.420174
Morc3	Morc3-003	2277.9	0.020397	0.048	2.675	2.675	16.721	6.251	1.419484
Wfdc1	Wfdc1-201	842.0	0.032474	0.070	2.674	2.674	2.987	1.117	1.419235
Per1	Per1-001	6089.1	0.009710	0.027	2.674	2.674	44.227	16.541	1.418902
Gfra2	Gfra2-201	437.0	0.032081	0.069	2.671	2.671	3.144	1.177	1.417233
Def6	Def6-001	2080.0	0.001597	0.007	2.670	2.670	11.088	4.152	1.417044
Ppp1r10	Ppp1r10-002	982.4	0.018442	0.045	2.669	2.669	6.883	2.579	1.416553
Cdkal1	Cdkal1-011	902.5	0.020569	0.049	2.664	2.664	4.491	1.686	1.413794
Rbm33	Rbm33-003	8109.9	0.002452	0.009	2.664	2.664	52.176	19.586	1.413607
Mxd4	Mxd4-001	17029.9	0.000729	0.004	2.663	2.663	87.970	33.040	1.412792
Hsp25-ps1	Hsp25-ps1-20	3290.9	0.019630	0.047	2.662	2.662	20.129	7.562	1.412406
Rpl36-ps3	Rpl36-ps3-201	2655.0	0.000830	0.004	2.655	2.655	20.527	7.731	1.408837
Asph	Asph-006	2222.1	0.003432	0.012	2.654	2.654	16.582	6.247	1.408419
Mlip	Mlip-005	716.0	0.005406	0.017	2.652	2.652	4.512	1.701	1.407318
Gm15387	Gm15387-001	1432.0	0.001576	0.006	2.648	2.648	10.819	4.086	1.404672
Aebp1	Aebp1-001	31847.2	0.005103	0.016	2.647	2.647	241.817	91.348	1.404475
BC017643	BC017643-00	1302.0	0.038336	0.079	2.642	2.642	8.874	3.358	1.401888
Akap7	Akap7-007	453.1	0.015666	0.039	2.642	2.642	2.807	1.062	1.401675
Rpsa-ps2	Rpsa-ps2-201	1730.0	0.002721	0.010	2.642	2.642	14.224	5.384	1.401667
Pigt	Pigt-001	3394.0	0.000965	0.004	2.637	2.637	20.233	7.672	1.398979
Yam1	Yam1-001.1	7338813.7	0.000005	0.000	2.635	2.635	50788.766	19274.142	1.397843
Dnajc2	Dnajc2-001	2288.4	0.007552	0.022	2.635	2.635	15.250	5.787	1.397836
Rpl29	Rpl29-002	7320.7	0.001912	0.008	2.635	2.635	54.600	20.724	1.39763
Spr	Spr-001	1170.6	0.033029	0.071	2.633	2.633	9.963	3.784	1.396642
Trrap	Trrap-006	4827.6	0.010866	0.029	2.629	2.629	28.181	10.721	1.394324
Frmd5	Frmd5-001	1400.3	0.043003	0.087	2.626	2.626	10.216	3.890	1.393005
Trafd1	Trafd1-001	2321.2	0.031323	0.068	2.626	2.626	16.447	6.264	1.392734
Erc1	Erc1-007	6931.2	0.006703	0.020	2.624	2.624	36.622	13.957	1.391733
Cic	Cic-002	3550.5	0.013553	0.035	2.624	2.624	20.763	7.914	1.391593

Ccdc22	Ccdc22-001	1347.6	0.007640	0.022	2.623	2.623	8.396	3.201	1.391304
Ncor2	Ncor2-005	3866.3	0.038062	0.079	2.616	2.616	21.172	8.094	1.387267
Rasal2	Rasal2-002	2417.9	0.005006	0.016	2.615	2.615	17.329	6.627	1.386876
Adgrv1	Adgrv1-008	2365.0	0.000735	0.004	2.615	2.615	15.664	5.991	1.386637
Zbtb14	Zbtb14-002	2860.3	0.009893	0.027	2.613	2.613	19.634	7.513	1.385825
Lxn	Lxn-001	2155.6	0.006050	0.019	2.612	2.612	12.546	4.802	1.385386
Vwf	Vwf-001	317880.6	0.000437	0.002	2.612	2.612	2233.325	855.124	1.384988
Lox	Lox-201	22185.1	0.000060	0.001	2.610	2.610	131.572	50.402	1.384309
Sort1	Sort1-004	5423.5	0.004592	0.015	2.610	2.610	22.640	8.675	1.383863
P23-396K2	23-396K2.2-(	1498.0	0.001577	0.007	2.609	2.609	10.039	3.847	1.383653
Aldh1a1	Aldh1a1-001	1773.0	0.017781	0.043	2.608	2.608	12.785	4.901	1.383169
Plec	Plec-002	12396.0	0.000894	0.004	2.607	2.607	48.293	18.521	1.382639
Ppp1r7	Ppp1r7-002	607.9	0.041212	0.084	2.604	2.604	4.682	1.798	1.380484
Mrps5	Mrps5-002	787.6	0.030068	0.065	2.601	2.601	5.734	2.205	1.37892
Phf23	Phf23-001	1759.9	0.003684	0.013	2.600	2.600	13.610	5.234	1.3787
Bcam	Bcam-001	74480.3	0.001265	0.005	2.600	2.600	211.141	81.204	1.378584
Rere	Rere-004	630.5	0.011395	0.031	2.599	2.599	4.446	1.711	1.377894
Gga1	Gga1-201	3328.0	0.000047	0.000	2.597	2.597	20.431	7.869	1.376569
30102H24F	0102H24Rik-	803.1	0.013194	0.034	2.596	2.596	5.719	2.203	1.376221
Gm10094	Gm10094-201	2977.0	0.000222	0.001	2.592	2.592	20.843	8.041	1.374179
Fnbp1	Fnbp1-006	3775.0	0.040392	0.083	2.590	2.590	29.382	11.343	1.37313
Cby1	Cby1-201	1120.0	0.004935	0.016	2.590	2.590	6.970	2.691	1.373098
Myl12a	Myl12a-002	18583.1	0.000425	0.002	2.589	2.589	149.735	57.839	1.372302
Rhbdf1	Rhbdf1-008	3913.5	0.002406	0.009	2.583	2.583	24.645	9.541	1.369153
Gm26619	Gm26619-201	4318.8	0.000528	0.003	2.583	2.583	35.489	13.741	1.368894
Gm12353	Gm12353-001	323.8	0.037255	0.078	2.583	2.583	1.852	0.717	1.368824
Cox4i1	Cox4i1-001	5755.7	0.003830	0.013	2.582	2.582	40.551	15.706	1.368397
Rpl35	Rpl35-001	6402.4	0.000776	0.004	2.581	2.581	51.668	20.015	1.368177
Tll1	Tll1-201	1192.0	0.023827	0.054	2.579	2.579	8.881	3.443	1.367032
Cyfip1	Cyfip1-002	7225.2	0.004041	0.014	2.575	2.575	38.949	15.126	1.364518
Cuedc1	Cuedc1-002	1437.6	0.008787	0.025	2.570	2.570	8.938	3.478	1.361545
Dhx16	Dhx16-003	7952.5	0.000027	0.000	2.567	2.567	48.527	18.902	1.360249
Ctnnb1	Ctnnb1-009	469.7	0.011751	0.031	2.566	2.566	2.043	0.796	1.359498
Gm28587	Gm28587-001	531.0	0.010783	0.029	2.566	2.566	2.996	1.168	1.35929
Gm16378	Gm16378-001	219.0	0.046832	0.093	2.565	2.565	1.908	0.744	1.359021
Gm16165	Gm16165-001	472.0	0.024291	0.055	2.564	2.564	3.581	1.396	1.358444
Lnp	Lnp-004	211.5	0.046019	0.092	2.564	2.564	1.265	0.493	1.358367
Scn1b	Scn1b-001	2868.2	0.023360	0.054	2.564	2.564	12.317	4.805	1.358127
Zc3h11a	Zc3h11a-201	11227.8	0.000089	0.001	2.563	2.563	75.887	29.608	1.357856
Rb1cc1	Rb1cc1-012	3861.0	0.000387	0.002	2.562	2.562	27.229	10.628	1.357276
Scrib	Scrib-003	4141.6	0.001291	0.006	2.561	2.561	24.143	9.426	1.356973
Klhl29	Klhl29-201	1214.0	0.030514	0.066	2.561	2.561	5.353	2.090	1.356468
Ccm2	Ccm2-001	1599.4	0.000738	0.004	2.560	2.560	9.269	3.620	1.356395
St6galnac2	t6galnac2-00	1530.2	0.031666	0.068	2.559	2.559	5.534	2.162	1.355706
Cc2d1a	Cc2d1a-001	2531.4	0.008944	0.025	2.557	2.557	15.078	5.896	1.354512
Spg20	Spg20-001	5713.6	0.009012	0.025	2.555	2.555	38.985	15.260	1.353178
Slmap	Slmap-008	1012.6	0.003382	0.012	2.555	2.555	4.988	1.953	1.353086
Babam1	Babam1-201	4909.0	0.001144	0.005	2.554	2.554	31.141	12.193	1.352718
Uba52	Uba52-001	3852.0	0.010629	0.029	2.554	2.554	24.610	9.636	1.352715
Gm29371	Gm29371-001	757.0	0.029295	0.064	2.552	2.552	5.479	2.147	1.35143
Atf7	Atf7-007	188.0	0.042964	0.087	2.551	2.551	1.222	0.479	1.351133
Anapc5	Anapc5-007	4721.5	0.014827	0.038	2.551	2.551	33.072	12.965	1.351001
Gm16066	Gm16066-001	1298.1	0.006540	0.020	2.551	2.551	7.521	2.949	1.350886
Gm6117	Gm6117-001	1178.0	0.003218	0.011	2.549	2.549	7.260	2.849	1.34976
Ifi27l2b	Ifi27l2b-001	906.0	0.025251	0.057	2.546	2.546	5.699	2.239	1.348004
Tbcel	Tbcel-001	2192.1	0.028717	0.063	2.545	2.545	16.822	6.609	1.347858
Xbp1	Xbp1-002	1874.9	0.047872	0.095	2.545	2.545	16.599	6.523	1.34751
Mustn1	Mustn1-201	3596.0	0.010156	0.028	2.543	2.543	17.938	7.054	1.346504
Cep164	Cep164-001	3234.7	0.000930	0.004	2.542	2.542	21.829	8.587	1.346038
Kmt2b	Kmt2b-003	9636.8	0.001635	0.007	2.542	2.542	66.354	26.106	1.345798
Gm5518	Gm5518-201	1951.0	0.001154	0.005	2.537	2.537	14.825	5.843	1.343219
Cox4i1	Cox4i1-003	708.3	0.026077	0.058	2.537	2.537	5.243	2.066	1.343171
Kcnq2	Kcnq2-002	1157.8	0.000905	0.004	2.536	2.536	7.066	2.786	1.342767
Ict1	Ict1-002	996.2	0.018079	0.044	2.534	2.534	6.597	2.603	1.341638
Smoc1	Smoc1-001	11492.8	0.002715	0.010	2.533	2.533	86.972	34.340	1.340676
Ube3b	Ube3b-002	4956.1	0.035085	0.074	2.531	2.531	30.177	11.922	1.339877
Sardh	Sardh-001	1737.3	0.007907	0.023	2.531	2.531	10.639	4.203	1.339782
Serpina1e	Serpina1e-002	1360.6	0.049715	0.098	2.527	2.527	3.198	1.265	1.337703
Gm26704	Gm26704-201	710.0	0.042593	0.086	2.527	2.527	5.075	2.008	1.33716
Klhl36	Klhl36-201	887.0	0.008584	0.024	2.525	2.525	6.773	2.683	1.336044
Calu	Calu-002	7476.4	0.005574	0.017	2.524	2.524	42.916	17.005	1.335546
Sdf2	Sdf2-001	1428.2	0.007503	0.022	2.523	2.523	10.371	4.110	1.335282
Ppp6r2	Ppp6r2-201	4075.0	0.000810	0.004	2.523	2.523	24.735	9.803	1.335241
Plec	Plec-016	14328.7	0.000111	0.001	2.518	2.518	53.263	21.156	1.332027
Kank3	Kank3-001	5782.6	0.000162	0.001	2.517	2.517	17.507	6.956	1.331527
Stk11	Stk11-002	1150.8	0.014976	0.038	2.517	2.517	7.938	3.154	1.331459
Mrgpre	Mrgpre-201	10692.0	0.000080	0.001	2.514	2.514	62.074	24.687	1.330228
Cyp20a1	Cyp20a1-002	456.2	0.013946	0.036	2.514	2.514	3.571	1.420	1.330112
Tnrc18	Tnrc18-001	9484.0	0.000056	0.001	2.512	2.512	64.777	25.782	1.329099
Apoe	Apoe-001	40522.7	0.004281	0.014	2.512	2.512	300.455	119.619	1.328705
Ube2d3	Ube2d3-005	1924.1	0.028460	0.063	2.509	2.509	14.449	5.759	1.327131
Gm527	Gm527-201	426.0	0.044828	0.090	2.509	2.509	2.076	0.827	1.327107
Atrx	Atrx-006	2217.0	0.000287	0.002	2.508	2.508	14.152	5.643	1.326497
Gm15287	Gm15287-001	1962.0	0.008565	0.024	2.508	2.508	12.655	5.046	1.326468
Rbfa	Rbfa-201	1034.0	0.009002	0.025	2.507	2.507	7.976	3.181	1.326128
P23-345O17	23-345O17.5-	604.0	0.005773	0.018	2.506	2.506	4.101	1.636	1.325593
Taf11	Taf11-001	335.2	0.042616	0.086	2.502	2.502	2.362	0.944	1.323273
Prpf40b	Prpf40b-003	1008.4	0.046496	0.093	2.502	2.502	6.622	2.647	1.322839
Rps5	Rps5-002	1251.5	0.004688	0.015	2.497	2.497	10.048	4.023	1.320404
Trim2	Trim2-003	5687.2	0.011528	0.031	2.497	2.497	40.851	16.358	1.320335
P24-309H3	24-309H3.6-(	1010.0	0.002329	0.009	2.491	2.491	5.821	2.336	1.317012
Zfp62	Zfp62-001	1503.9	0.049747	0.098	2.491	2.491	9.989	4.009	1.31695
Elmo2	Elmo2-008	1872.4	0.025016	0.057	2.488	2.488	12.100	4.864	1.314948
Fam219a	Fam219a-002	478.7	0.019905	0.047	2.486	2.486	2.946	1.185	1.314027
Kctd2	Kctd2-002	985.7	0.040662	0.083	2.485	2.485	6.706	2.698	1.313293
Pawr	Pawr-201	2475.0	0.002785	0.010	2.485	2.485	24.220	9.747	1.313145
Zscan26	Zscan26-001	3195.4	0.002491	0.009	2.484	2.484	19.538	7.865	1.312828
Actr5	Actr5-004	994.3	0.047328	0.094	2.483	2.483	6.940	2.795	1.311861
Tmem115	Tmem115-001	1986.0	0.001506	0.006	2.480	2.480	13.266	5.350	1.310135
Zfp276	Zfp276-001	1371.5	0.001703	0.007	2.479	2.479	8.139	3.283	1.310011
Gm14420	Gm14420-002	2815.9	0.000058	0.001	2.477	2.477	17.470	7.053	1.308502
Itgb5	Itgb5-001	4778.0	0.011261	0.030	2.476	2.476	40.808	16.482	1.308
Ppp1r16b	Ppp1r16b-201	4454.7	0.000187	0.001	2.475	2.475	21.178	8.555	1.307655
Pfkp	Pfkp-005	3487.9	0.007745	0.023	2.473	2.473	23.499	9.502	1.306343
Ttc7	Ttc7-001	918.4	0.030796	0.067	2.470	2.470	5.436	2.201	1.304512
Gm6133	Gm6133-201	446.0	0.015550	0.039	2.469	2.469	3.651	1.478	1.30417
Pi16	Pi16-001	9150.9	0.002273	0.009	2.469	2.469	35.677	14.450	1.303937

Ankrd50	Ankrd50-002	8367.6	0.000139	0.001	2.466	2.466	53.034	21.504	1.302305
Nbeal2	Nbeal2-003	2382.0	0.012978	0.034	2.465	2.465	11.652	4.727	1.301515
Rusc2	Rusc2-004	8596.6	0.001969	0.008	2.463	2.463	63.237	25.672	1.300552
Smarca4	Smarca4-001	1679.0	0.029466	0.064	2.459	2.459	10.085	4.102	1.297995
Trip10	Trip10-201	10106.0	0.000142	0.001	2.458	2.458	60.529	24.625	1.297498
Gm12505	Gm12505-001	639.0	0.025124	0.057	2.458	2.458	4.885	1.988	1.297269
Gpr146	Gpr146-002	3631.5	0.014044	0.036	2.457	2.457	23.380	9.516	1.296776
Ntng2	Ntng2-002	437.1	0.033166	0.071	2.453	2.453	2.189	0.893	1.294519
Eng	Eng-005	6392.8	0.014509	0.037	2.453	2.453	38.845	15.836	1.294512
Git2	Git2-003	710.1	0.007201	0.021	2.451	2.451	5.276	2.153	1.293125
Hs3st1	Hs3st1-003	1705.2	0.026497	0.059	2.451	2.451	10.664	4.352	1.293087
Rbm39	Rbm39-025	2722.8	0.001027	0.005	2.450	2.450	18.142	7.404	1.292918
Rgs3	Rgs3-006	946.6	0.045962	0.092	2.450	2.450	7.317	2.986	1.29281
Emg1	Emg1-002	528.0	0.024341	0.055	2.450	2.450	3.055	1.247	1.292514
Zfp687	Zfp687-005	2617.4	0.006944	0.021	2.449	2.449	19.770	8.072	1.292411
Tenm1	Tenm1-003	663.0	0.010716	0.029	2.449	2.449	4.669	1.906	1.292259
Nsmaf	Nsmaf-001	3116.7	0.041924	0.085	2.448	2.448	22.643	9.251	1.291406
Scx	Scx-201	2185.0	0.003670	0.013	2.447	2.447	14.612	5.971	1.291194
Lama5	Lama5-001	56085.0	0.000035	0.000	2.447	2.447	229.017	93.589	1.291035
Nub1	Nub1-003	5140.0	0.000094	0.001	2.446	2.446	34.178	13.972	1.29056
Svep1	Svep1-001	1960.6	0.023692	0.054	2.446	2.446	14.141	5.781	1.290466
Ccdc157	Ccdc157-001	1492.1	0.023942	0.055	2.446	2.446	7.606	3.109	1.290441
Gm6525	Gm6525-001	2799.0	0.000060	0.001	2.443	2.443	21.770	8.910	1.288934
Dlg4	Dlg4-003	1789.4	0.014007	0.036	2.441	2.441	11.342	4.646	1.287709
Bahcc1	Bahcc1-003	2534.0	0.013203	0.034	2.439	2.439	17.383	7.128	1.28607
Gnai2	Gnai2-007	619.9	0.024934	0.056	2.439	2.439	5.886	2.414	1.286067
Kansl1	Kansl1-002	7789.6	0.001330	0.006	2.439	2.439	45.497	18.657	1.286041
Rnf41	Rnf41-202	2165.2	0.002335	0.009	2.438	2.438	12.469	5.115	1.285455
Gm17907	Gm17907-001	357.0	0.021659	0.051	2.437	2.437	1.872	0.768	1.285099
Gm13092	Gm13092-001	1180.0	0.008568	0.024	2.437	2.437	9.222	3.784	1.285073
Zfpl1	Zfpl1-001	665.2	0.047707	0.095	2.435	2.435	5.147	2.114	1.284011
Nacad	Nacad-001	1170.2	0.019713	0.047	2.432	2.432	7.103	2.921	1.282093
Zfp319	Zfp319-201	2305.4	0.012464	0.033	2.432	2.432	15.806	6.500	1.282064
Nudcd3	Nudcd3-004	8167.7	0.000028	0.000	2.431	2.431	51.536	21.198	1.281643
Gm9910	Gm9910-001	2351.5	0.023364	0.054	2.431	2.431	15.141	6.228	1.28158
Synpo	Synpo-007	2555.4	0.001726	0.007	2.429	2.429	18.800	7.739	1.280574
Gfpt2	Gfpt2-001	2349.0	0.019582	0.047	2.429	2.429	19.308	7.950	1.280111
Thsd7b	Thsd7b-004	348.0	0.023314	0.054	2.425	2.425	2.314	0.954	1.278209
Nfyc	Nfyc-001	854.2	0.021851	0.051	2.424	2.424	5.122	2.113	1.277628
Osbpl3	Osbpl3-001	2250.2	0.035856	0.075	2.424	2.424	17.250	7.117	1.277152
Myh9	Myh9-002	2424.1	0.038734	0.080	2.423	2.423	16.788	6.929	1.2768
030624J02Ri	0624J02Rik-0	1045.3	0.014947	0.038	2.419	2.419	7.100	2.935	1.274192
Lrrc49	Lrrc49-201	672.2	0.031463	0.068	2.415	2.415	3.308	1.370	1.272053
Col6a1	Col6a1-001	1080.6	0.030517	0.066	2.415	2.415	7.645	3.166	1.271908
Prkg1	Prkg1-002	3162.4	0.039511	0.081	2.413	2.413	27.322	11.324	1.270681
Abca3	Abca3-201	6169.2	0.005912	0.018	2.412	2.412	40.782	16.911	1.269972
Mgll	Mgll-001	3922.0	0.014142	0.036	2.411	2.411	12.211	5.064	1.269742
Nab1	Nab1-001	4342.2	0.004314	0.014	2.411	2.411	27.885	11.567	1.269483
Naa30	Naa30-001	1395.3	0.003837	0.013	2.410	2.410	10.564	4.384	1.26877
Hist1h2bg	Hist1h2bg-001	649.0	0.023864	0.054	2.407	2.407	4.119	1.711	1.267233
Dcbld2	Dcbld2-007	15729.2	0.000021	0.000	2.406	2.406	93.167	38.730	1.266368
Vars2	Vars2-003	709.8	0.042088	0.086	2.402	2.402	4.912	2.045	1.264462
Hlx	Hlx-001	1683.1	0.026294	0.059	2.402	2.402	12.804	5.330	1.264305
Cd22	Cd22-007	825.9	0.016067	0.040	2.402	2.402	5.809	2.419	1.26408
Irf2bp1	Irf2bp1-201	2448.0	0.001153	0.005	2.401	2.401	16.747	6.974	1.263755
Sprtn	Sprtn-201	1659.0	0.003312	0.012	2.401	2.401	10.664	4.442	1.263389
Zfp236	Zfp236-003	1467.9	0.037329	0.078	2.399	2.399	9.316	3.883	1.262606
Myo18a	Myo18a-004	1768.7	0.036084	0.076	2.398	2.398	9.125	3.805	1.261783
Dennd5a	Dennd5a-001	24262.0	0.000300	0.002	2.396	2.396	166.698	69.568	1.260736
Tmem30a	Tmem30a-002	1960.1	0.013186	0.034	2.395	2.395	11.409	4.763	1.260095
Spice1	Spice1-003	610.1	0.020199	0.048	2.392	2.392	3.548	1.483	1.258344
430105I19R	30105I19Rik-	9047.0	0.000555	0.003	2.392	2.392	43.587	18.224	1.258019
Park7	Park7-001	4433.3	0.004761	0.015	2.391	2.391	29.975	12.535	1.257846
Lrrc42	Lrrc42-001	890.1	0.040203	0.082	2.387	2.387	6.593	2.762	1.254932
Mlf2	Mlf2-202	7341.6	0.000274	0.002	2.387	2.387	49.610	20.788	1.254905
Zfp503	Zfp503-001	807.0	0.026685	0.059	2.386	2.386	4.123	1.728	1.254794
Os9	Os9-201	17879.6	0.000012	0.000	2.386	2.386	103.663	43.441	1.25477
Rps16	Rps16-001	6232.5	0.014958	0.038	2.386	2.386	50.824	21.305	1.25432
Abt1	Abt1-001	1562.0	0.000925	0.004	2.384	2.384	11.149	4.676	1.253586
Rnf13	Rnf13-002	1101.2	0.021043	0.049	2.384	2.384	6.384	2.678	1.253469
Acin1	Acin1-001	602.4	0.037934	0.079	2.381	2.381	4.156	1.745	1.251584
Osbpl1a	Osbpl1a-001	1493.5	0.044625	0.090	2.381	2.381	9.333	3.920	1.251457
Slc38a10	Slc38a10-201	5363.0	0.003871	0.013	2.381	2.381	36.706	15.418	1.251376
Arhgef2	Arhgef2-025	406.0	0.029816	0.065	2.381	2.381	1.605	0.674	1.251343
Acad12	Acad12-001	1162.3	0.015622	0.039	2.379	2.379	5.037	2.117	1.250438
Ikbke	Ikbke-001	1384.5	0.044360	0.089	2.376	2.376	6.101	2.567	1.248653
Sub1	Sub1-004	2516.0	0.005763	0.018	2.374	2.374	17.923	7.551	1.247093
Pard3b	Pard3b-002	4087.7	0.008190	0.024	2.373	2.373	22.664	9.550	1.246845
Aqp1	Aqp1-001	70748.0	0.002772	0.010	2.371	2.371	329.777	139.066	1.245726
Myo1c	Myo1c-009	4296.9	0.000438	0.002	2.371	2.371	23.979	10.114	1.24542
Sulf1	Sulf1-002	64752.2	0.000213	0.001	2.371	2.371	356.434	150.348	1.245327
Rab6a	Rab6a-001	6514.1	0.000141	0.001	2.370	2.370	40.797	17.211	1.245157
Kcnh7	Kcnh7-001	1528.8	0.003178	0.011	2.370	2.370	10.133	4.275	1.245067
Pten	Pten-003	852.8	0.006964	0.021	2.370	2.370	5.514	2.326	1.245038
Azi2	Azi2-005	890.8	0.007816	0.023	2.370	2.370	4.938	2.084	1.244789
Ndufa10	Ndufa10-001	1920.6	0.009452	0.026	2.369	2.369	13.373	5.645	1.244431
Sfpq	Sfpq-002	5484.2	0.008557	0.024	2.369	2.369	36.831	15.547	1.24428
Copg1	Copg1-002	1003.7	0.023874	0.054	2.368	2.368	6.954	2.937	1.24355
Rps6kl1	Rps6kl1-201	1162.0	0.021982	0.051	2.367	2.367	5.261	2.223	1.242762
Rpl13a	Rpl13a-008	37403.4	0.000004	0.000	2.366	2.366	289.366	122.296	1.242522
Oscp1	Oscp1-001	647.0	0.006744	0.020	2.364	2.364	4.024	1.702	1.241473
Rbm39	Rbm39-015	1029.1	0.034057	0.072	2.363	2.363	6.552	2.772	1.240786
Six2	Six2-001	1946.2	0.002010	0.008	2.361	2.361	10.066	4.264	1.239097
Wdr33	Wdr33-202	911.9	0.027967	0.062	2.360	2.360	6.171	2.615	1.238774
Nfkbia	Nfkbia-201	11076.0	0.000395	0.002	2.358	2.358	76.682	32.519	1.237589
Cyp4a31	Cyp4a31-001	1099.3	0.009911	0.027	2.356	2.356	8.008	3.399	1.236406
Hist1h2bc	Hist1h2bc-001	3037.0	0.002604	0.010	2.354	2.354	18.585	7.895	1.235096
Ascc1	Ascc1-201	1215.7	0.006394	0.019	2.351	2.351	8.171	3.475	1.233323
Ddx27	Ddx27-001	5342.5	0.000579	0.003	2.350	2.350	37.520	15.964	1.232874
Sp3	Sp3-001	2703.0	0.022180	0.051	2.349	2.349	15.966	6.795	1.232352
Sin3b	Sin3b-201	5603.6	0.000085	0.001	2.347	2.347	36.127	15.394	1.230738
Prr12	Prr12-201	12398.0	0.000066	0.001	2.347	2.347	78.064	33.264	1.23069
Gm20400	Gm20400-001	1177.0	0.038488	0.080	2.341	2.341	8.721	3.725	1.227328
Zfp811	Zfp811-001	253.0	0.049326	0.097	2.340	2.340	1.768	0.756	1.226619
Psmd2	Psmd2-001	10488.8	0.012204	0.032	2.338	2.338	69.761	29.836	1.22535
Rgag4	Rgag4-001	2569.0	0.023541	0.054	2.338	2.338	14.241	6.091	1.225295
Arid1a	Arid1a-003	2751.5	0.012888	0.034	2.336	2.336	17.729	7.588	1.22431

Pcnxl3	Pcnxl3-001	5157.3	0.007110	0.021	2.336	2.336	34.208	14.645	1.223926
Itgb4	Itgb4-202	3000.6	0.020672	0.049	2.335	2.335	12.349	5.288	1.22345
Usp48	Usp48-012	974.0	0.018831	0.045	2.332	2.332	5.720	2.452	1.221783
Nek7	Nek7-001	6786.4	0.003197	0.011	2.331	2.331	35.191	15.094	1.221252
Ccdc149	Ccdc149-002	2884.6	0.000685	0.003	2.331	2.331	20.019	8.589	1.220839
Zc3h7a	Zc3h7a-004	1338.8	0.014230	0.036	2.330	2.330	9.122	3.915	1.220301
Gm4980	Gm4980-001	3462.0	0.026657	0.059	2.330	2.330	18.290	7.851	1.220125
Arhgef11	Arhgef11-001	3813.5	0.005752	0.018	2.329	2.329	26.676	11.454	1.219725
Kat7	Kat7-001	3819.0	0.023146	0.053	2.328	2.328	21.409	9.196	1.2192
Erlec1	Erlec1-005	1423.8	0.022305	0.052	2.327	2.327	7.685	3.302	1.218606
Irf6	Irf6-004	2174.2	0.039469	0.081	2.327	2.327	16.665	7.163	1.21823
Rnf220	Rnf220-005	499.4	0.044219	0.089	2.327	2.327	2.939	1.263	1.218197
Khdrbs3	Khdrbs3-201	2219.0	0.002963	0.011	2.326	2.326	14.906	6.408	1.217897
Gm5453	Gm5453-201	1579.0	0.001422	0.006	2.326	2.326	12.323	5.299	1.21764
Cacna1e	Cacna1e-003	3084.0	0.000508	0.003	2.325	2.325	17.986	7.735	1.217468
Lamb2	Lamb2-001	62158.1	0.009690	0.027	2.325	2.325	341.395	146.815	1.217447
Meis2	Meis2-013	4750.3	0.001051	0.005	2.325	2.325	29.936	12.876	1.217156
C1rb	C1rb-001	1878.0	0.023319	0.054	2.324	2.324	13.458	5.790	1.21678
Erf	Erf-001	6456.1	0.000331	0.002	2.323	2.323	41.952	18.063	1.215696
Erg	Erg-005	5167.7	0.002909	0.011	2.321	2.321	31.171	13.431	1.214576
Cdc37	Cdc37-201	14062.0	0.000031	0.000	2.320	2.320	93.680	40.377	1.214215
Rexo1	Rexo1-001	3981.7	0.000179	0.001	2.319	2.319	27.096	11.686	1.213308
Gapdh	Gapdh-001	6117.1	0.005543	0.017	2.316	2.316	47.076	20.328	1.21151
Esam	Esam-005	1562.6	0.005820	0.018	2.313	2.313	10.638	4.600	1.209514
Smoc1	Smoc1-002	18311.6	0.002606	0.010	2.312	2.312	137.122	59.297	1.209424
Eif4g2	Eif4g2-008	5789.4	0.000208	0.001	2.312	2.312	33.303	14.403	1.209303
Snx3	Snx3-001	7136.5	0.002963	0.011	2.312	2.312	39.134	16.925	1.209251
Gm4735	Gm4735-001	1262.0	0.008312	0.024	2.312	2.312	8.919	3.858	1.209012
Serpinb6b	Serpinb6b-002	6992.6	0.005705	0.018	2.311	2.311	51.467	22.275	1.208221
Tanc2	Tanc2-002	4857.0	0.013446	0.035	2.307	2.307	26.691	11.571	1.205828
Pkp2	Pkp2-003	381.1	0.035570	0.075	2.305	2.305	3.463	1.503	1.204729
Uba1	Uba1-005	1079.4	0.019022	0.046	2.304	2.304	6.528	2.833	1.204298
Adcy4	Adcy4-202	15464.4	0.000103	0.001	2.303	2.303	92.336	40.096	1.203423
Mamld1	Mamld1-201	9615.5	0.000826	0.004	2.301	2.301	81.306	35.333	1.202359
Dgcr2	Dgcr2-004	5295.9	0.013443	0.035	2.300	2.300	28.110	12.223	1.201507
Vat1	Vat1-001	16282.6	0.000042	0.000	2.300	2.300	88.276	38.385	1.20148
Synj1	Synj1-001	1705.1	0.009563	0.027	2.297	2.297	10.030	4.366	1.199991
Mrpl20	Mrpl20-001	743.8	0.037112	0.077	2.296	2.296	6.113	2.662	1.199241
Sbf1	Sbf1-003	3338.4	0.020161	0.048	2.296	2.296	31.047	13.524	1.19891
App	App-201	110173.0	0.000222	0.001	2.295	2.295	660.013	287.541	1.198727
Spsb1	Spsb1-001	6236.8	0.014315	0.037	2.294	2.294	43.507	18.964	1.198
Cox5b	Cox5b-001	5041.1	0.002509	0.009	2.293	2.293	31.567	13.766	1.1973
Phf1	Phf1-201	4990.0	0.000711	0.004	2.292	2.292	29.097	12.697	1.196405
Mllt4	Mllt4-008	1404.6	0.013122	0.034	2.291	2.291	6.438	2.810	1.196103
Abhd5	Abhd5-002	1556.0	0.018074	0.044	2.289	2.289	9.559	4.176	1.194547
Ick	Ick-006	1700.7	0.042842	0.087	2.289	2.289	6.664	2.912	1.1945
Atp13a2	Atp13a2-004	1179.9	0.049597	0.097	2.287	2.287	9.092	3.976	1.193313
Slc4a2	Slc4a2-001	5320.4	0.010492	0.029	2.286	2.286	28.722	12.564	1.192893
Gm38297	Gm38297-001	2018.0	0.000830	0.004	2.284	2.284	12.735	5.577	1.191333
Gm10167	Gm10167-001	427.0	0.018243	0.044	2.278	2.278	2.701	1.186	1.187505
Tle3	Tle3-201	2448.8	0.006957	0.021	2.276	2.276	16.037	7.045	1.186692
Arhgef1	Arhgef1-005	2329.3	0.039269	0.081	2.276	2.276	14.283	6.277	1.186193
Cyhr1	Cyhr1-003	1585.6	0.010808	0.029	2.274	2.274	11.111	4.885	1.18553
Ppme1	Ppme1-201	4249.0	0.000196	0.001	2.274	2.274	25.887	11.383	1.185416
Stoml1	Stoml1-201	1355.0	0.005215	0.017	2.274	2.274	8.756	3.851	1.185148
Myl9	Myl9-001	3195.1	0.037842	0.079	2.269	2.269	27.630	12.175	1.182361
Plac9b	Plac9b-004	1054.3	0.014092	0.036	2.267	2.267	5.826	2.570	1.180909
Azin1	Azin1-006	793.5	0.028228	0.062	2.267	2.267	2.181	0.962	1.18059
Nvl	Nvl-002	2157.0	0.008952	0.025	2.264	2.264	14.404	6.362	1.178963
Ttc4	Ttc4-001	758.5	0.046949	0.093	2.264	2.264	4.876	2.154	1.17885
Tomm40l	Tomm40l-004	888.9	0.027588	0.061	2.263	2.263	5.969	2.638	1.178088
Fmnl3	Fmnl3-001	7836.3	0.007364	0.022	2.263	2.263	44.752	19.778	1.178058
Gm2274	Gm2274-002	2854.7	0.000736	0.004	2.262	2.262	17.228	7.616	1.177766
Sec63	Sec63-004	1224.1	0.049861	0.098	2.260	2.260	5.933	2.625	1.176523
Zfp526	Zfp526-201	2987.0	0.008098	0.023	2.260	2.260	20.076	8.882	1.17649
Emc10	Emc10-002	3099.4	0.000371	0.002	2.259	2.259	20.731	9.175	1.175945
Map3k6	Map3k6-002	5392.4	0.023813	0.054	2.259	2.259	23.763	10.521	1.175468
Sqrdl	Sqrdl-008	3043.0	0.021480	0.050	2.258	2.258	18.304	8.106	1.175034
Rps20	Rps20-001	14306.3	0.000034	0.000	2.257	2.257	106.988	47.397	1.174583
Grsf1	Grsf1-001	1323.1	0.007545	0.022	2.257	2.257	8.189	3.629	1.174204
Stxbp5	Stxbp5-002	4917.3	0.016923	0.042	2.257	2.257	29.248	12.961	1.17418
Gm12693	Gm12693-001	870.0	0.003975	0.013	2.256	2.256	6.153	2.728	1.173656
Ankrd11	Ankrd11-002	22743.3	0.000003	0.000	2.252	2.252	150.233	66.711	1.171203
Triobp	Triobp-005	13174.2	0.000022	0.000	2.252	2.252	65.429	29.057	1.171042
Tmpo	Tmpo-204	2644.1	0.020771	0.049	2.251	2.251	8.370	3.719	1.170418
Tbc1d9b	Tbc1d9b-001	7621.8	0.005981	0.018	2.250	2.250	48.984	21.767	1.170153
Csnk2b	Csnk2b-001	3249.2	0.006161	0.019	2.250	2.250	23.453	10.422	1.1701
Wbp5	Wbp5-001	4360.2	0.010159	0.028	2.250	2.250	30.259	13.448	1.169958
Actb	Actb-005	3092.1	0.028119	0.062	2.250	2.250	23.917	10.631	1.169672
Ppp1r12b	Ppp1r12b-001	5683.0	0.010865	0.029	2.249	2.249	48.582	21.603	1.169225
BE692007	BE692007-001	560.9	0.037453	0.078	2.247	2.247	3.931	1.750	1.167893
C1qc	C1qc-001	1465.0	0.008640	0.025	2.247	2.247	13.802	6.144	1.167719
Eif4a2	Eif4a2-019	900.3	0.040461	0.083	2.241	2.241	6.755	3.014	1.164231
Katnb1	Katnb1-201	1119.0	0.003578	0.012	2.240	2.240	7.763	3.466	1.163318
030018B13R	0018B13Rik-	513.0	0.025640	0.058	2.239	2.239	3.753	1.676	1.163017
Map3k3	Map3k3-001	10728.7	0.000029	0.000	2.239	2.239	71.988	32.153	1.162805
Tdrd7	Tdrd7-002	1729.2	0.030499	0.066	2.238	2.238	10.400	4.647	1.162215
Ndufs1	Ndufs1-006	1583.2	0.016208	0.040	2.238	2.238	8.386	3.747	1.162188
Ranbp3	Ranbp3-201	3848.0	0.000589	0.003	2.238	2.238	26.670	11.919	1.161969
Maz	Maz-201	3937.0	0.000243	0.002	2.237	2.237	25.363	11.336	1.161842
Hddc2	Hddc2-001	454.8	0.038975	0.080	2.236	2.236	3.577	1.600	1.160924
Svil	Svil-004	1724.9	0.028601	0.063	2.236	2.236	8.432	3.771	1.160764
Iqsec3	Iqsec3-002	606.4	0.011593	0.031	2.235	2.235	4.174	1.867	1.160547
Aldh3b1	Aldh3b1-201	1058.0	0.011461	0.031	2.235	2.235	8.075	3.613	1.160245
Ctnnd1	Ctnnd1-202	7764.8	0.005935	0.018	2.234	2.234	49.905	22.335	1.159906
Snrpb	Snrpb-001	4665.4	0.001015	0.005	2.234	2.234	33.218	14.868	1.159746
Ptp4a2	Ptp4a2-001	16200.3	0.000262	0.002	2.233	2.233	107.400	48.101	1.158866
Cttn	Cttn-001	12534.8	0.000051	0.000	2.233	2.233	73.938	33.115	1.158855
Wasf2	Wasf2-006	963.2	0.012870	0.034	2.230	2.230	5.660	2.539	1.156801
Dtnbp1	Dtnbp1-201	2021.8	0.012042	0.032	2.229	2.229	17.163	7.699	1.156468
Sfn	Sfn-001	909.0	0.023552	0.054	2.228	2.228	5.005	2.247	1.155645
Opa1	Opa1-005	1059.5	0.034631	0.073	2.228	2.228	6.104	2.740	1.155529
Csnk1e	Csnk1e-003	389.3	0.025665	0.058	2.226	2.226	2.523	1.133	1.154713
Gm11808	Gm11808-001	7105.0	0.000075	0.001	2.225	2.225	51.975	23.358	1.153931
Pld1	Pld1-004	5571.5	0.041568	0.085	2.224	2.224	34.071	15.317	1.153397
Gm15576	Gm15576-001	835.0	0.034804	0.074	2.224	2.224	5.376	2.418	1.152955
Hmgb1-ps6	Hmgb1-ps6-00	326.0	0.043715	0.088	2.223	2.223	2.112	0.950	1.152468

Ilk	Ilk-001	6659.6	0.000196	0.001	2.222	2.222	46.648	20.993	1.151943
Pnpla2	Pnpla2-002	10456.7	0.001088	0.005	2.222	2.222	71.287	32.081	1.151901
Gm5527	Gm5527-001	1040.0	0.002820	0.010	2.217	2.217	8.044	3.628	1.14878
Sympk	Sympk-001	5629.4	0.004125	0.014	2.213	2.213	35.361	15.982	1.145757
Glyr1	Glyr1-202	3596.7	0.004499	0.015	2.211	2.211	23.951	10.830	1.145019
Celf4	Celf4-201	1196.7	0.014211	0.036	2.211	2.211	7.274	3.290	1.144699
Celf4	Celf4-202	611.3	0.026689	0.059	2.211	2.211	3.715	1.680	1.144686
10036O22R	0036O22Rik-	7732.0	0.000133	0.001	2.210	2.210	51.297	23.214	1.143858
Itgb4	Itgb4-003	13673.1	0.008615	0.024	2.208	2.208	46.892	21.240	1.142532
Ndufa12	Ndufa12-201	3935.5	0.020567	0.049	2.206	2.206	26.615	12.064	1.141577
Foxp1	Foxp1-023	4479.7	0.021134	0.050	2.206	2.206	20.779	9.420	1.141272
Ap1b1	Ap1b1-201	3167.6	0.034374	0.073	2.205	2.205	24.878	11.285	1.14049
Card10	Card10-001	10480.8	0.000265	0.002	2.202	2.202	53.808	24.432	1.139034
Alyref	Alyref-002	1511.0	0.032523	0.070	2.200	2.200	10.250	4.659	1.13755
Elf1	Elf1-201	5858.5	0.000968	0.004	2.200	2.200	35.695	16.226	1.137382
Chst12	Chst12-001	6206.5	0.000283	0.002	2.200	2.200	29.333	13.335	1.137295
Nos1	Nos1-001	1238.0	0.002370	0.009	2.199	2.199	7.923	3.602	1.136978
Gm6611	Gm6611-001	1074.0	0.011947	0.032	2.199	2.199	6.001	2.729	1.136827
Ppm1h	Ppm1h-002	657.0	0.043217	0.087	2.198	2.198	4.837	2.200	1.136499
Ctnnb1	Ctnnb1-003	1454.4	0.049313	0.097	2.197	2.197	7.238	3.295	1.135293
Tle4	Tle4-202	2339.9	0.041805	0.085	2.196	2.196	13.511	6.153	1.134808
Zfp646	Zfp646-001	8307.0	0.000281	0.002	2.195	2.195	55.795	25.419	1.134241
Pcbp2	Pcbp2-202	3764.4	0.030211	0.066	2.195	2.195	32.979	15.028	1.133901
Psmg4	Psmg4-001	818.6	0.012477	0.033	2.192	2.192	6.015	2.743	1.132557
Naa38	Naa38-001	731.8	0.042745	0.087	2.192	2.192	5.066	2.311	1.132329
Zcchc24	Zcchc24-001	3857.2	0.001122	0.005	2.189	2.189	25.109	11.469	1.130504
Fgfr1	Fgfr1-003	6948.1	0.016621	0.041	2.189	2.189	47.743	21.814	1.130044
Rrbp1	Rrbp1-002	32233.8	0.000197	0.001	2.189	2.189	212.760	97.216	1.129962
Abi3	Abi3-001	4194.5	0.029424	0.064	2.187	2.187	26.577	12.154	1.128725
Bud13	Bud13-002	5026.1	0.001064	0.005	2.185	2.185	32.390	14.826	1.127447
Ubb	Ubb-001	61234.9	0.000216	0.001	2.184	2.184	372.822	170.716	1.126892
Hnrnpm	Hnrnpm-002	9735.6	0.001788	0.007	2.184	2.184	69.352	31.760	1.126717
Hmgb1-ps7	mgb1-ps7-00	576.0	0.015387	0.039	2.183	2.183	4.548	2.083	1.126333
Apba3	Apba3-201	1836.0	0.003469	0.012	2.182	2.182	12.796	5.865	1.125537
Evc2	Evc2-001	4610.5	0.009717	0.027	2.178	2.178	31.678	14.541	1.123321
Fhl1	Fhl1-002	9243.8	0.001209	0.005	2.178	2.178	50.343	23.111	1.123207
Fbxo3	Fbxo3-001	3088.4	0.024243	0.055	2.178	2.178	14.989	6.882	1.123085
Ampd3	Ampd3-003	4822.5	0.003657	0.013	2.177	2.177	27.119	12.458	1.12225
33434E20R	3434E20Rik-	1318.6	0.018823	0.045	2.173	2.173	9.989	4.597	1.119708
Ssfa2	Ssfa2-003	27532.0	0.000694	0.003	2.172	2.172	142.719	65.696	1.119295
Mmp28	Mmp28-003	4254.0	0.006091	0.019	2.171	2.171	20.056	9.237	1.118553
Csde1	Csde1-001	12587.7	0.004417	0.015	2.170	2.170	78.685	36.261	1.117645
Dapk1	Dapk1-202	6959.0	0.000292	0.002	2.167	2.167	38.738	17.878	1.115538
Acin1	Acin1-025	1714.2	0.008502	0.024	2.166	2.166	10.541	4.867	1.114834
Atg4c	Atg4c-201	2997.3	0.004772	0.015	2.165	2.165	23.468	10.838	1.114684
Rab11b-ps2	ab11b-ps2-20	751.0	0.025556	0.057	2.163	2.163	5.236	2.420	1.11319
Akap2	Akap2-004	4671.3	0.026937	0.060	2.163	2.163	19.658	9.088	1.11309
L1cam	L1cam-010	1736.9	0.047727	0.095	2.160	2.160	9.490	4.394	1.110862
Abhd8	Abhd8-201	1152.0	0.010582	0.029	2.159	2.159	7.429	3.441	1.110514
Pan3	Pan3-001	2587.1	0.013515	0.035	2.159	2.159	15.001	6.949	1.110111
Mrps18c	Mrps18c-002	876.3	0.018189	0.044	2.156	2.156	6.022	2.793	1.108196
Kmt2c	Kmt2c-008	2558.0	0.045357	0.091	2.155	2.155	15.442	7.167	1.107453
Myh10	Myh10-002	4824.2	0.011066	0.030	2.154	2.154	30.856	14.327	1.106827
Aldh2	Aldh2-001	13669.1	0.000164	0.001	2.150	2.150	68.039	31.647	1.104288
Il18r1	Il18r1-202	468.6	0.039759	0.082	2.150	2.150	3.384	1.574	1.104218
Tmem259	Tmem259-001	4513.2	0.000360	0.002	2.147	2.147	29.313	13.653	1.102311
Mxra8	Mxra8-006	1748.4	0.031348	0.068	2.147	2.147	8.723	4.064	1.101996
Bcl2l1	Bcl2l1-201	5414.9	0.037004	0.077	2.146	2.146	35.386	16.488	1.101739
Anxa7	Anxa7-202	5513.7	0.024681	0.056	2.146	2.146	28.684	13.366	1.101729
Cactin	Cactin-201	3030.0	0.000285	0.002	2.141	2.141	19.839	9.265	1.09846
Luzp1	Luzp1-004	7691.2	0.028728	0.063	2.138	2.138	41.058	19.202	1.096356
Chic2	Chic2-002	1150.3	0.017262	0.042	2.138	2.138	9.539	4.462	1.096075
Slc23a2	Slc23a2-002	800.7	0.029063	0.064	2.137	2.137	5.762	2.697	1.095463
Arhgap29	Arhgap29-002	8120.5	0.004837	0.016	2.137	2.137	54.979	25.731	1.095359
Mid1ip1	Mid1ip1-002	3115.0	0.049168	0.097	2.137	2.137	18.384	8.604	1.095328
Mtss1l	Mtss1l-001	1268.0	0.031225	0.067	2.136	2.136	7.222	3.382	1.0946
Zfp366	Zfp366-001	1338.0	0.017585	0.043	2.135	2.135	7.540	3.531	1.094537
Hspa1a	Hspa1a-001	8575.3	0.027676	0.061	2.135	2.135	58.545	27.424	1.094087
Pde4dip	Pde4dip-001	1168.4	0.018035	0.044	2.135	2.135	6.492	3.041	1.093995
Klc4	Klc4-201	2653.0	0.027528	0.061	2.133	2.133	13.428	6.295	1.092989
Tapbp	Tapbp-003	1094.0	0.021975	0.051	2.131	2.131	6.855	3.217	1.091441
Impdh2	Impdh2-001	3169.8	0.000926	0.004	2.131	2.131	22.918	10.757	1.091227
Supt5	Supt5-201	17894.0	0.000036	0.000	2.128	2.128	113.619	53.387	1.089645
Cct6a	Cct6a-201	2575.6	0.008657	0.025	2.128	2.128	17.355	8.157	1.089326
Rab1b	Rab1b-201	5318.0	0.000416	0.002	2.124	2.124	32.452	15.278	1.086844
Hist1h4d	Hist1h4d-001	3993.0	0.033929	0.072	2.121	2.121	30.085	14.185	1.084672
Nme2	Nme2-001	4002.1	0.017927	0.044	2.120	2.120	24.792	11.692	1.084355
Ccdc28b	Ccdc28b-001	821.9	0.044473	0.089	2.120	2.120	4.375	2.064	1.084033
Gm27013	Gm27013-001	567.0	0.041089	0.084	2.119	2.119	3.194	1.507	1.08368
Mxi1	Mxi1-201	1427.4	0.039162	0.081	2.118	2.118	10.855	5.124	1.082966
430042O09F	0042O09Rik-	2680.0	0.001851	0.007	2.116	2.116	15.592	7.368	1.081519
Rheb	Rheb-004	943.1	0.030866	0.067	2.115	2.115	5.109	2.415	1.080729
Fam20c	Fam20c-001	1384.1	0.038213	0.079	2.112	2.112	6.049	2.864	1.078698
Myo9b	Myo9b-203	3513.9	0.010961	0.030	2.111	2.111	19.122	9.059	1.077879
Chd6	Chd6-001	2727.2	0.004587	0.015	2.111	2.111	17.577	8.327	1.077846
Gpi1	Gpi1-002	2461.0	0.015004	0.038	2.110	2.110	15.769	7.473	1.077333
Erbb2ip	Erbb2ip-006	955.3	0.035314	0.074	2.109	2.109	5.595	2.652	1.076749
P24-537K9	24-537K9.1-(	1079.0	0.003920	0.013	2.109	2.109	7.749	3.674	1.076542
Adamtsl3	Adamtsl3-001	1328.5	0.020240	0.048	2.109	2.109	6.204	2.942	1.076335
Snhg17	Snhg17-003	2302.1	0.009284	0.026	2.109	2.109	17.488	8.294	1.076249
Rabac1	Rabac1-201	4818.0	0.003040	0.011	2.108	2.108	32.309	15.326	1.075993
Dbi	Dbi-003	1757.6	0.015798	0.039	2.108	2.108	13.413	6.363	1.075783
Sepp1	Sepp1-001	39957.9	0.010284	0.028	2.106	2.106	362.502	172.102	1.074725
Rcan1	Rcan1-202	791.9	0.039514	0.081	2.106	2.106	4.918	2.335	1.074366
Ddrgk1	Ddrgk1-001	4027.0	0.008749	0.025	2.101	2.101	29.495	14.037	1.071236
Cldn5	Cldn5-201	15238.0	0.005710	0.018	2.101	2.101	59.938	28.535	1.070743
Psme2	Psme2-001	3071.0	0.000435	0.002	2.100	2.100	19.440	9.258	1.070236
Sh3glb1	Sh3glb1-002	2481.6	0.024379	0.055	2.099	2.099	13.544	6.452	1.069863
Cd3d	Cd3d-201	536.0	0.022202	0.052	2.098	2.098	3.354	1.599	1.068719
Caskin2	Caskin2-001	17882.1	0.000237	0.002	2.097	2.097	67.764	32.316	1.068272
Sult1a1	Sult1a1-001	5109.6	0.023528	0.054	2.095	2.095	23.561	11.247	1.066933
Xylt2	Xylt2-001	1486.8	0.014738	0.037	2.095	2.095	10.698	5.107	1.066699
Whsc1l1	Whsc1l1-001	1122.0	0.033660	0.072	2.094	2.094	4.528	2.162	1.066515
Cep350	Cep350-003	1415.7	0.049856	0.098	2.094	2.094	8.830	4.217	1.066295
BC002163	3C002163-00	530.0	0.034303	0.073	2.093	2.093	3.264	1.559	1.065876
Tab1	Tab1-201	1399.0	0.011281	0.030	2.091	2.091	8.201	3.922	1.064251
Gabbr1	Gabbr1-005	4840.4	0.023910	0.055	2.091	2.091	27.223	13.021	1.064004

Pam	Pam-201	22300.5	0.015089	0.038	2.090	2.090	131.484	62.899	1.063767
Ptov1	Ptov1-001	1964.1	0.001696	0.007	2.089	2.089	14.084	6.742	1.062937
Npr1	Npr1-003	776.4	0.044323	0.089	2.087	2.087	4.123	1.975	1.06137
Nckipsd	Nckipsd-001	789.2	0.047923	0.095	2.087	2.087	5.224	2.503	1.06122
Cyth1	Cyth1-202	2833.9	0.005615	0.018	2.084	2.084	15.158	7.273	1.059475
Slc35e4	Slc35e4-201	1608.3	0.031997	0.069	2.084	2.084	8.551	4.103	1.059305
Usp4	Usp4-004	5887.7	0.002269	0.009	2.083	2.083	31.885	15.309	1.058526
Gm5884	Gm5884-201	716.0	0.008788	0.025	2.082	2.082	4.407	2.116	1.058248
Pcgf2	Pcgf2-201	1536.3	0.013658	0.035	2.079	2.079	9.026	4.341	1.056175
Ddx49	Ddx49-005	705.0	0.018676	0.045	2.079	2.079	4.345	2.090	1.055885
Ube3a	Ube3a-006	663.2	0.034971	0.074	2.079	2.079	3.655	1.758	1.055703
Eva1a	Eva1a-002	393.5	0.044863	0.090	2.079	2.079	2.672	1.286	1.055613
Prkcdbp	Prkcdbp-201	10258.0	0.000252	0.002	2.076	2.076	57.544	27.719	1.053779
Rpl8	Rpl8-201	37876.0	0.001536	0.006	2.075	2.075	269.772	129.981	1.053437
Ppp1r12c	Ppp1r12c-006	846.5	0.049641	0.097	2.074	2.074	3.988	1.923	1.052505
Ddr2	Ddr2-001	3678.4	0.017942	0.044	2.074	2.074	21.856	10.538	1.052438
Tmem176a	mem176a-20	3833.3	0.017139	0.042	2.073	2.073	25.614	12.358	1.051412
Tubb2a	Tubb2a-201	13025.0	0.000158	0.001	2.072	2.072	89.036	42.971	1.051035
Abca3	Abca3-001	6930.2	0.000046	0.000	2.072	2.072	42.178	20.358	1.050895
Ptbp1	Ptbp1-008	3505.7	0.028525	0.063	2.070	2.070	24.364	11.769	1.049739
Ergic3	Ergic3-001	5552.4	0.004057	0.014	2.070	2.070	34.778	16.803	1.04942
Rpl34-ps1	Rpl34-ps1-00	1944.0	0.001546	0.006	2.068	2.068	14.892	7.200	1.048571
Numa1	Numa1-001	46588.1	0.001280	0.006	2.067	2.067	311.886	150.904	1.047383
Txlna	Txlna-201	4112.8	0.046074	0.092	2.064	2.064	30.801	14.920	1.045704
Nrde2	Nrde2-201	2298.0	0.012920	0.034	2.063	2.063	14.951	7.248	1.044509
Rps3a3	Rps3a3-001	3711.0	0.000161	0.001	2.061	2.061	28.169	13.669	1.043171
Gm10335	Gm10335-001	10038.0	0.000085	0.001	2.060	2.060	78.288	38.011	1.042372
Zfp768	Zfp768-201	1341.0	0.004612	0.015	2.058	2.058	9.214	4.477	1.041352
Gm37108	Gm37108-001	1256.0	0.001794	0.007	2.057	2.057	9.843	4.786	1.04031
Pigk	Pigk-001	17555.4	0.000392	0.002	2.055	2.055	102.536	49.891	1.039271
Eif3b	Eif3b-001	11906.1	0.003468	0.012	2.055	2.055	79.773	38.816	1.039227
Pecam1	Pecam1-002	17124.8	0.000197	0.001	2.054	2.054	76.251	37.116	1.038737
Camkk2	Camkk2-006	1385.4	0.013009	0.034	2.054	2.054	8.638	4.205	1.0386
Irf2bpl	Irf2bpl-201	8743.0	0.002595	0.010	2.052	2.052	62.419	30.421	1.036905
Lfng	Lfng-001	2262.2	0.013859	0.036	2.052	2.052	13.642	6.649	1.036883
Zfp106	Zfp106-011	1311.5	0.035304	0.074	2.052	2.052	6.617	3.225	1.036867
Nnt	Nnt-004	1442.9	0.043338	0.088	2.050	2.050	9.779	4.771	1.035404
Cnpy3	Cnpy3-001	2467.1	0.046025	0.092	2.047	2.047	16.385	8.004	1.033604
Pi4kb	Pi4kb-001	930.6	0.047537	0.094	2.046	2.046	6.096	2.979	1.032969
Pi4ka	Pi4ka-012	4357.1	0.017387	0.043	2.045	2.045	28.648	14.010	1.032004
Myh10	Myh10-001	9056.2	0.017219	0.042	2.044	2.044	70.867	34.677	1.031114
Il6st	Il6st-004	25738.8	0.006140	0.019	2.043	2.043	225.440	110.352	1.030629
Pigb	Pigb-003	624.1	0.043945	0.089	2.043	2.043	2.781	1.361	1.03061
Xrn1	Xrn1-201	2612.9	0.030140	0.066	2.042	2.042	15.767	7.720	1.03023
Unkl	Unkl-002	1880.2	0.021582	0.050	2.041	2.041	11.319	5.545	1.029544
Rnf115	Rnf115-001	3418.0	0.004021	0.013	2.036	2.036	19.861	9.757	1.025415
Zc3h7b	Zc3h7b-201	12804.0	0.000240	0.002	2.034	2.034	75.162	36.949	1.024479
Olfm1	Olfm1-004	897.5	0.047065	0.093	2.034	2.034	4.268	2.098	1.024408
Rab27a	Rab27a-003	11663.7	0.013865	0.036	2.034	2.034	62.857	30.905	1.024226
Tsc22d4	Tsc22d4-003	2057.6	0.004979	0.016	2.034	2.034	11.846	5.825	1.024149
Ppp1r13l	Ppp1r13l-001	1236.4	0.006280	0.019	2.033	2.033	9.613	4.729	1.02354
Rp2h	Rp2h-003	1088.5	0.041340	0.084	2.033	2.033	8.135	4.002	1.023335
Dtx3	Dtx3-004	894.5	0.022868	0.053	2.031	2.031	5.936	2.923	1.02219
Rpl19	Rpl19-001	37952.0	0.000012	0.000	2.025	2.025	277.451	136.986	1.018202
Klk10	Klk10-201	36476.0	0.006522	0.020	2.025	2.025	26.276	12.978	1.017684
23-396K22	23-396K22.3-	10736.0	0.000222	0.001	2.023	2.023	67.924	33.570	1.016756
Dctn2	Dctn2-201	8389.0	0.000239	0.002	2.022	2.022	50.391	24.927	1.015472
Med23	Med23-003	1351.0	0.002161	0.008	2.021	2.021	8.087	4.001	1.01534
Tmem63a	Tmem63a-002	4347.7	0.021845	0.051	2.020	2.020	25.729	12.737	1.014325
C1ra	C1ra-201	7666.0	0.017132	0.042	2.020	2.020	51.808	25.649	1.014269
Gm9762	Gm9762-001	570.0	0.023934	0.055	2.018	2.018	3.570	1.769	1.013058
Spata13	Spata13-201	2602.0	0.037775	0.079	2.018	2.018	14.661	7.266	1.012839
Ubap2l	Ubap2l-005	5606.8	0.036693	0.077	2.017	2.017	36.827	18.259	1.012132
Dhx16	Dhx16-001	3331.8	0.024229	0.055	2.014	2.014	16.795	8.339	1.010163
Arfip1	Arfip1-003	1412.4	0.026978	0.060	2.014	2.014	8.027	3.985	1.010134
Gstm2	Gstm2-001	5459.5	0.004508	0.015	2.014	2.014	34.802	17.279	1.010109
Fam50a	Fam50a-001	4666.3	0.000522	0.003	2.014	2.014	30.319	15.056	1.009914
200014J11Ri	0014J11Rik-	17223.1	0.000147	0.001	2.014	2.014	113.579	56.402	1.00986
Synpo	Synpo-004	2953.1	0.035389	0.075	2.012	2.012	24.426	12.138	1.008875
Hdac5	Hdac5-001	5140.1	0.002894	0.010	2.012	2.012	27.871	13.851	1.008807
Acsf3	Acsf3-201	1443.0	0.013822	0.036	2.006	2.006	8.959	4.467	1.003968
Stim1	Stim1-201	10089.0	0.002128	0.008	2.001	2.001	55.074	27.520	1.000907
Gm9581	Gm9581-001	6682.0	0.004861	0.016	2.001	2.001	45.109	22.545	1.000586
Plxnb2	Plxnb2-001	8354.8	0.001319	0.006	1.998	1.998	64.419	32.241	0.998597
Cdc42ep1	Cdc42ep1-201	6891.0	0.002745	0.010	1.998	1.998	43.799	21.922	0.998515
Tk2	Tk2-201	1460.0	0.015952	0.040	1.996	1.996	9.697	4.857	0.997337
Aida	Aida-005	1079.4	0.035197	0.074	1.996	1.996	6.509	3.260	0.997281
Wdtc1	Wdtc1-001	4452.4	0.009001	0.025	1.996	1.996	29.311	14.686	0.997005
Rhobtb2	Rhobtb2-201	1680.0	0.023188	0.053	1.995	1.995	13.033	6.533	0.996255
Nrp2	Nrp2-003	1689.3	0.033898	0.072	1.993	1.993	10.142	5.090	0.994669
Stk40	Stk40-001	6318.2	0.027649	0.061	1.993	1.993	35.465	17.799	0.994596
Kif26a	Kif26a-001	16763.1	0.003264	0.011	1.990	1.990	57.095	28.689	0.992863
Kif5b	Kif5b-002	5909.5	0.007689	0.022	1.989	1.989	35.185	17.686	0.992341
Gm19721	Gm19721-001	1538.0	0.037353	0.078	1.989	1.989	9.253	4.652	0.992184
Ppp6r1	Ppp6r1-201	10769.0	0.000144	0.001	1.988	1.988	69.086	34.748	0.991481
Fau	Fau-202	11356.1	0.000381	0.002	1.987	1.987	80.846	40.681	0.990825
Noc2l	Noc2l-201	5723.3	0.001963	0.008	1.987	1.987	43.445	21.867	0.990448
Pja2	Pja2-001	7250.3	0.012275	0.032	1.986	1.986	47.204	23.766	0.990032
Cic	Cic-001	8494.2	0.004180	0.014	1.985	1.985	51.294	25.837	0.989369
Gripap1	Gripap1-010	2438.8	0.028617	0.063	1.984	1.984	14.422	7.268	0.988535
Ndufb7	Ndufb7-001	4763.4	0.002397	0.009	1.983	1.983	30.869	15.564	0.987988
Gm7808	Gm7808-201	1087.0	0.003197	0.011	1.983	1.983	7.299	3.680	0.987921
Dyrk1b	Dyrk1b-003	2113.4	0.026502	0.059	1.982	1.982	10.193	5.143	0.986728
Nat6	Nat6-001	2435.6	0.016814	0.041	1.981	1.981	12.173	6.146	0.985876
Epb4.1	Epb4.1-004	7247.2	0.000433	0.002	1.980	1.980	55.077	27.821	0.98531
Tfip11	Tfip11-001	3870.9	0.002475	0.009	1.979	1.979	24.970	12.615	0.985129
Zcchc8	Zcchc8-201	1756.6	0.026542	0.059	1.978	1.978	11.093	5.608	0.984021
Rnd1	Rnd1-001	3603.6	0.018333	0.044	1.977	1.977	27.887	14.108	0.983141
Nsmce4a	Nsmce4a-001	2644.0	0.003064	0.011	1.976	1.976	17.465	8.838	0.982691
Gng12	Gng12-002	665.7	0.023406	0.054	1.976	1.976	5.099	2.581	0.982505
Leng8	Leng8-003	4871.1	0.038237	0.079	1.975	1.975	30.057	15.216	0.982145
Arrdc2	Arrdc2-201	900.0	0.020135	0.048	1.975	1.975	7.721	3.909	0.981993
Gm15682	Gm15682-001	1836.0	0.005885	0.018	1.974	1.974	12.651	6.408	0.981285
Gadd45gip1	add45gip1-00	1319.2	0.020174	0.048	1.972	1.972	10.947	5.552	0.97942
Wdr81	Wdr81-001	5209.4	0.000273	0.002	1.971	1.971	34.899	17.705	0.978992
Col26a1	Col26a1-012	1080.0	0.029859	0.065	1.971	1.971	6.668	3.383	0.978805
Smyd4	Smyd4-001	1334.4	0.033688	0.072	1.970	1.970	8.971	4.554	0.978063

Ppp2r4	Ppp2r4-004	1051.2	0.036069	0.076	1.969	1.969	7.331	3.723	0.977507
Dst	Dst-203	99821.3	0.000150	0.001	1.968	1.968	649.916	330.284	0.976548
Rpl21	Rpl21-006	10604.0	0.000958	0.004	1.967	1.967	77.317	39.301	0.97621
Gm2000	Gm2000-001	9643.0	0.001447	0.006	1.967	1.967	74.030	37.638	0.97591
Eif2b5	Eif2b5-001	3903.6	0.000283	0.002	1.967	1.967	25.509	12.971	0.975715
Lars2	Lars2-201	4012998.0	0.001003	0.005	1.965	1.965	25175.086	12813.147	0.974372
Man1a	Man1a-003	1966.3	0.040216	0.082	1.965	1.965	11.011	5.604	0.974328
Fam134c	Fam134c-002	3410.4	0.001451	0.006	1.962	1.962	22.499	11.470	0.972002
Zfyve9	Zfyve9-001	6259.0	0.015520	0.039	1.961	1.961	42.188	21.510	0.971798
Pam	Pam-001	40585.0	0.004625	0.015	1.960	1.960	213.171	108.734	0.971209
'24-132L16.	4-132L16.13-	636.0	0.024446	0.056	1.960	1.960	3.782	1.929	0.971162
Nav2	Nav2-010	759.5	0.019737	0.047	1.959	1.959	4.844	2.472	0.970465
Srrm2	Srrm2-002	93514.6	0.000064	0.001	1.959	1.959	586.660	299.516	0.969892
Leng1	Leng1-001	1801.3	0.015435	0.039	1.957	1.957	11.024	5.632	0.9689
Scarf1	Scarf1-003	2980.1	0.008953	0.025	1.957	1.957	18.695	9.555	0.96838
Setbp1	Setbp1-201	37889.0	0.038893	0.080	1.957	1.957	217.652	111.243	0.968315
Cyb5r4	Cyb5r4-001	1372.0	0.008146	0.023	1.955	1.955	9.259	4.735	0.967474
Crtap	Crtap-001	7403.8	0.000725	0.004	1.955	1.955	47.958	24.530	0.967204
Rcn3	Rcn3-002	5847.9	0.013589	0.035	1.954	1.954	38.784	19.844	0.966729
Pfkl	Pfkl-001	4546.0	0.005803	0.018	1.954	1.954	30.173	15.442	0.966335
Ankrd17	Ankrd17-004	5032.0	0.038459	0.080	1.954	1.954	29.477	15.088	0.966186
P23-387L12	23-387L12.3-	1738.0	0.003055	0.011	1.953	1.953	12.442	6.369	0.966022
Gm9770	Gm9770-201	1442.0	0.012285	0.032	1.953	1.953	9.803	5.018	0.965979
Hip1	Hip1-001	30243.0	0.000273	0.002	1.953	1.953	161.346	82.634	0.965351
Sfswap	Sfswap-002	4821.0	0.007926	0.023	1.952	1.952	25.513	13.067	0.965245
Uba52	Uba52-005	4528.7	0.000655	0.003	1.952	1.952	31.481	16.130	0.964708
Snhg20	Snhg20-001	841.4	0.048859	0.096	1.950	1.950	5.704	2.926	0.963335
Plec	Plec-004	122654.9	0.000094	0.001	1.948	1.948	388.912	199.598	0.962348
Gadd45g	Gadd45g-001	2719.8	0.033297	0.071	1.946	1.946	22.446	11.532	0.960816
Tm9sf4	Tm9sf4-001	3223.4	0.016212	0.040	1.945	1.945	21.001	10.799	0.959537
Map2k2	Map2k2-001	1783.8	0.014178	0.036	1.944	1.944	10.326	5.311	0.959307
Usp19	Usp19-004	3223.2	0.036618	0.077	1.944	1.944	20.919	10.760	0.959103
Zfp282	Zfp282-001	2577.1	0.003782	0.013	1.943	1.943	15.728	8.093	0.9585
Grasp	Grasp-201	10863.0	0.000883	0.004	1.943	1.943	38.511	19.821	0.9582
Dpy19l3	Dpy19l3-003	4138.0	0.004003	0.013	1.943	1.943	27.423	14.115	0.958168
Atp8b1	Atp8b1-201	19277.0	0.003842	0.013	1.940	1.940	136.033	70.109	0.956291
Nfx1	Nfx1-007	2984.9	0.010745	0.029	1.939	1.939	16.964	8.750	0.955142
Gid8	Gid8-001	3882.4	0.000572	0.003	1.938	1.938	25.199	13.005	0.954302
Gm10521	Gm10521-201	2594.0	0.027217	0.060	1.938	1.938	17.368	8.963	0.954282
Megf8	Megf8-001	10758.0	0.002091	0.008	1.935	1.935	60.538	31.281	0.952572
Bop1	Bop1-201	2424.0	0.035149	0.074	1.933	1.933	18.081	9.356	0.950596
Pigp	Pigp-004	723.6	0.023091	0.053	1.933	1.933	3.875	2.005	0.950587
Ccar2	Ccar2-201	5722.0	0.002842	0.010	1.932	1.932	36.883	19.089	0.950212
Baz2b	Baz2b-007	2012.4	0.038771	0.080	1.928	1.928	11.595	6.014	0.947232
Fxr2	Fxr2-006	3888.4	0.000433	0.002	1.928	1.928	26.113	13.543	0.947206
Gm37967	Gm37967-001	1306.0	0.019849	0.047	1.928	1.928	7.876	4.085	0.947176
Elmo1	Elmo1-003	1403.2	0.042094	0.086	1.927	1.927	8.655	4.492	0.946109
Gm14420	Gm14420-001	1992.1	0.031951	0.069	1.927	1.927	12.440	6.457	0.946055
Rbfox2	Rbfox2-204	6922.5	0.007034	0.021	1.925	1.925	35.946	18.677	0.944565
Zbtb22	Zbtb22-001	3111.6	0.015608	0.039	1.924	1.924	17.984	9.346	0.944258
Mir99ahg	Mir99ahg-009	2931.0	0.013121	0.034	1.924	1.924	16.698	8.681	0.943805
Snx29	Snx29-006	1585.6	0.048230	0.095	1.922	1.922	9.928	5.164	0.942933
Pwwp2b	Pwwp2b-201	3664.6	0.017017	0.042	1.920	1.920	16.057	8.362	0.941265
Akt1	Akt1-001	20488.5	0.000404	0.002	1.918	1.918	121.582	63.377	0.939906
Anxa3	Anxa3-002	15183.4	0.001574	0.006	1.918	1.918	85.616	44.649	0.939269
Rnf181	Rnf181-001	1321.8	0.020657	0.049	1.917	1.917	9.208	4.803	0.938916
Ube2o	Ube2o-001	2913.5	0.041708	0.085	1.916	1.916	20.773	10.841	0.938258
Slc12a6	Slc12a6-003	6505.3	0.001250	0.005	1.916	1.916	43.101	22.497	0.938001
Clip1	Clip1-011	4162.4	0.002936	0.011	1.915	1.915	21.110	11.026	0.937016
Sf3b2	Sf3b2-201	32180.0	0.000036	0.000	1.914	1.914	197.490	103.199	0.936353
Ctsh	Ctsh-001	25269.4	0.001940	0.008	1.913	1.913	102.672	53.664	0.936011
Mospd2	Mospd2-003	2591.6	0.006716	0.020	1.910	1.910	18.016	9.430	0.933887
Nkx2-1	Nkx2-1-001	2730.7	0.007420	0.022	1.910	1.910	15.493	8.113	0.933375
Actg-ps1	Actg-ps1-201	1804.0	0.014286	0.036	1.909	1.909	11.905	6.236	0.93282
Hsd3b7	Hsd3b7-003	6235.9	0.041551	0.085	1.909	1.909	34.784	18.226	0.932474
Shank3	Shank3-201	23177.5	0.000812	0.004	1.904	1.904	111.912	58.769	0.929243
Ranbp1	Ranbp1-001	4577.8	0.029904	0.065	1.904	1.904	31.308	16.447	0.928672
Arpc3	Arpc3-001	7974.0	0.018158	0.044	1.902	1.902	56.586	29.754	0.927366
Itgb1	Itgb1-003	4277.6	0.005574	0.017	1.902	1.902	24.059	12.651	0.927308
Sik1	Sik1-201	12624.0	0.000310	0.002	1.902	1.902	87.123	45.816	0.927211
Sun1	Sun1-001	5544.0	0.005155	0.016	1.901	1.901	31.195	16.411	0.926691
Ap3d1	Ap3d1-201	17928.0	0.000010	0.000	1.901	1.901	107.744	56.689	0.926451
Krt80	Krt80-201	34166.0	0.005562	0.017	1.898	1.898	120.710	63.604	0.924357
Atp5h	Atp5h-001	2551.5	0.022449	0.052	1.898	1.898	16.288	8.583	0.924316
Clta	Clta-201	10007.0	0.009203	0.026	1.897	1.897	63.002	33.210	0.923758
Plekhm2	Plekhm2-002	2419.3	0.018110	0.044	1.896	1.896	15.806	8.337	0.922905
Ccdc102a	Ccdc102a-001	2433.0	0.007634	0.022	1.895	1.895	17.613	9.292	0.922524
Grik5	Grik5-201	4169.0	0.000478	0.003	1.894	1.894	21.764	11.489	0.921762
Abl1	Abl1-001	5163.2	0.019340	0.046	1.894	1.894	34.096	17.999	0.921704
Pdxdc1	Pdxdc1-010	4385.4	0.004996	0.016	1.893	1.893	29.372	15.516	0.920685
Ddx23	Ddx23-001	9487.4	0.001266	0.005	1.892	1.892	62.134	32.833	0.920218
Gm37352	Gm37352-001	6231.0	0.005058	0.016	1.891	1.891	30.231	15.990	0.918904
Unc45a	Unc45a-002	2690.6	0.003622	0.012	1.890	1.890	18.976	10.038	0.918736
Gltscr2	Gltscr2-004	1526.1	0.043919	0.088	1.890	1.890	10.186	5.388	0.918723
Ctps	Ctps-001	15372.8	0.002661	0.010	1.889	1.889	143.420	75.911	0.917878
Arhgef15	Arhgef15-001	21689.4	0.005778	0.018	1.888	1.888	94.115	49.853	0.916742
Rpl28	Rpl28-001	20861.1	0.011900	0.032	1.887	1.887	160.827	85.213	0.916361
AI661453	AI661453-002	1930.7	0.005602	0.017	1.884	1.884	9.109	4.834	0.914058
Zfp523	Zfp523-002	1230.2	0.042513	0.086	1.884	1.884	8.114	4.307	0.913772
Heg1	Heg1-003	25564.1	0.015217	0.038	1.884	1.884	131.717	69.926	0.913537
Arhgef2	Arhgef2-001	15549.8	0.007062	0.021	1.882	1.882	74.002	39.325	0.912101
Scyl1	Scyl1-201	8006.2	0.000102	0.001	1.882	1.882	51.289	27.259	0.911907
Cdk11b	Cdk11b-001	8179.8	0.004593	0.015	1.881	1.881	53.713	28.552	0.911673
Hdac3	Hdac3-001	3161.3	0.019212	0.046	1.880	1.880	23.302	12.392	0.911028
Ybx1	Ybx1-001	71787.5	0.000007	0.000	1.880	1.880	365.597	194.451	0.910845
Optn	Optn-001	3533.2	0.046719	0.093	1.878	1.878	22.497	11.980	0.909076
Fermt3	Fermt3-201	3080.9	0.004753	0.015	1.876	1.876	20.744	11.057	0.907805
P4ha2	P4ha2-002	3170.6	0.044321	0.089	1.875	1.875	13.966	7.448	0.906963
Zfp628	Zfp628-201	2391.0	0.009676	0.027	1.873	1.873	13.907	7.425	0.905409
Lrrc3b	Lrrc3b-201	554.4	0.035353	0.074	1.872	1.872	2.167	1.157	0.904403
AU040320	AU040320-002	4823.8	0.018245	0.044	1.872	1.872	27.701	14.800	0.904368
Rpsa	Rpsa-201	47837.0	0.004191	0.014	1.872	1.872	358.703	191.659	0.904253
Crip2	Crip2-001	21545.8	0.000926	0.004	1.867	1.867	118.155	63.284	0.900768
Trpv4	Trpv4-002	2412.7	0.039622	0.081	1.865	1.865	9.611	5.153	0.899143
Ankrd13a	Ankrd13a-005	4911.7	0.009700	0.027	1.863	1.863	33.085	17.756	0.897883
Lonp1	Lonp1-201	6295.0	0.000623	0.003	1.861	1.861	40.751	21.894	0.896323
Pim3	Pim3-201	8311.0	0.031297	0.068	1.860	1.860	75.894	40.806	0.895202

Strn4	Strn4-002	3329.9	0.035172	0.074	1.859	1.859	18.350	9.870	0.89468
Bcar3	Bcar3-001	1753.4	0.017765	0.043	1.858	1.858	13.386	7.204	0.893954
Brms1	Brms1-201	2376.0	0.002396	0.009	1.858	1.858	14.393	7.746	0.893827
Zfp64	Zfp64-002	1803.4	0.014217	0.036	1.858	1.858	11.350	6.110	0.893388
Gm9843	Gm9843-201	16946.0	0.000260	0.002	1.857	1.857	122.712	66.085	0.892872
Gm3052	Gm3052-002	2653.0	0.007435	0.022	1.857	1.857	15.778	8.497	0.892829
Herc3	Herc3-007	488.6	0.034166	0.073	1.856	1.856	3.252	1.752	0.892304
Ptrf	Ptrf-001	134313.0	0.000230	0.001	1.856	1.856	576.586	310.650	0.892243
Nfib	Nfib-001	13248.7	0.004745	0.015	1.855	1.855	81.341	43.839	0.891759
Fkbp15	Fkbp15-003	3879.0	0.014731	0.037	1.855	1.855	21.002	11.320	0.891689
P23-50E10.	23-50E10.1-(	2908.0	0.018704	0.045	1.854	1.854	17.143	9.246	0.890761
Ppp1r9b	Ppp1r9b-002	14422.1	0.014488	0.037	1.853	1.853	89.952	48.555	0.889519
Acox1	Acox1-002	1326.8	0.040321	0.083	1.852	1.852	7.415	4.004	0.888996
Rpl7	Rpl7-003	31395.5	0.000069	0.001	1.849	1.849	224.847	121.572	0.887131
Pja2	Pja2-002	5189.7	0.023541	0.054	1.849	1.849	31.051	16.797	0.886457
Mob3a	Mob3a-201	1989.4	0.012468	0.033	1.848	1.848	15.403	8.335	0.885945
Pcdh7	Pcdh7-004	17121.2	0.002046	0.008	1.847	1.847	103.283	55.913	0.885336
Hoxa4	Hoxa4-001	1183.1	0.031077	0.067	1.847	1.847	6.202	3.358	0.885192
Map2k7	Map2k7-002	1712.4	0.024710	0.056	1.845	1.845	11.123	6.027	0.883883
Actn4	Actn4-002	37632.4	0.000367	0.002	1.845	1.845	221.895	120.276	0.883535
Uqcrc1	Uqcrc1-001	4316.8	0.004023	0.013	1.844	1.844	25.031	13.573	0.883021
Spen	Spen-001	17309.0	0.021885	0.051	1.841	1.841	114.738	62.323	0.880501
Gm12183	Gm12183-001	4218.0	0.002099	0.008	1.840	1.840	25.530	13.878	0.879391
Cd200	Cd200-011	187.4	0.031982	0.069	1.839	1.839	0.582	0.316	0.879225
Akap10	Akap10-001	3248.2	0.010655	0.029	1.839	1.839	21.129	11.491	0.8787
Ksr1	Ksr1-001	6101.4	0.014238	0.036	1.839	1.839	37.187	20.225	0.878663
Rab3gap1	Rab3gap1-001	8680.5	0.003523	0.012	1.838	1.838	53.604	29.160	0.878362
Fbxo46	Fbxo46-201	1038.8	0.047140	0.094	1.836	1.836	6.236	3.395	0.876942
Sorbs1	Sorbs1-201	8974.7	0.042381	0.086	1.836	1.836	57.141	31.119	0.876757
Tmem220	Tmem220-001	2237.0	0.027023	0.060	1.833	1.833	12.913	7.045	0.874062
Ano10	Ano10-201	2568.0	0.006282	0.019	1.832	1.832	15.573	8.501	0.873368
Gm12174	Gm12174-001	1969.0	0.002063	0.008	1.829	1.829	13.671	7.473	0.871376
Gm6483	Gm6483-003	2874.5	0.006939	0.021	1.829	1.829	17.171	9.387	0.871176
Mmrn2	Mmrn2-201	94249.0	0.008133	0.023	1.829	1.829	421.292	230.391	0.870739
Mast4	Mast4-008	7518.1	0.005964	0.018	1.828	1.828	55.781	30.518	0.870119
Slc25a30	Slc25a30-201	2181.0	0.004203	0.014	1.827	1.827	11.985	6.560	0.8695
Keap1	Keap1-001	3695.8	0.034101	0.072	1.826	1.826	19.484	10.671	0.868534
Sulf2	Sulf2-002	5309.9	0.011515	0.031	1.826	1.826	23.827	13.051	0.868434
Sptan1	Sptan1-004	29301.3	0.010082	0.028	1.825	1.825	146.854	80.456	0.868106
430203G13F	0203G13Rik-	1522.0	0.007303	0.022	1.825	1.825	8.657	4.743	0.867934
Ntn1	Ntn1-201	58060.4	0.005562	0.017	1.825	1.825	226.880	124.324	0.867818
Sart1	Sart1-201	9432.0	0.000417	0.002	1.823	1.823	60.173	33.012	0.866119
Tpgs1	Tpgs1-201	1058.0	0.020749	0.049	1.818	1.818	6.691	3.680	0.862603
Usp3	Usp3-004	2488.2	0.025195	0.057	1.818	1.818	15.232	8.378	0.86252
Prkcz	Prkcz-001	1514.2	0.049888	0.098	1.817	1.817	8.712	4.794	0.861674
Taf1	Taf1-001	4945.8	0.033881	0.072	1.817	1.817	29.446	16.207	0.861418
Tie1	Tie1-001	46284.7	0.000627	0.003	1.817	1.817	220.353	121.286	0.861409
Fam189a2	Fam189a2-201	8848.0	0.048499	0.096	1.817	1.817	39.774	21.893	0.861391
Pcbp1	Pcbp1-001	12002.0	0.009725	0.027	1.816	1.816	82.774	45.570	0.861089
Mtm1	Mtm1-007	917.5	0.030784	0.067	1.815	1.815	5.172	2.850	0.860046
Zc3h18	Zc3h18-002	4814.5	0.031531	0.068	1.815	1.815	29.036	15.997	0.860029
Rps24	Rps24-202	13173.6	0.001976	0.008	1.814	1.814	84.569	46.615	0.859346
Jun	Jun-001	38249.4	0.020238	0.048	1.814	1.814	221.942	122.346	0.859219
Sil1	Sil1-201	2086.0	0.011125	0.030	1.812	1.812	14.412	7.956	0.857211
Nfkb1	Nfkb1-009	1864.0	0.044805	0.090	1.808	1.808	12.127	6.707	0.85447
Tmem184b	Tmem184b-20	17430.8	0.000128	0.001	1.808	1.808	81.553	45.118	0.854031
Nenf	Nenf-001	2406.0	0.002515	0.009	1.806	1.806	12.888	7.138	0.852406
Sptan1	Sptan1-001	33206.2	0.023292	0.053	1.804	1.804	171.145	94.887	0.850933
Ehd1	Ehd1-001	15415.3	0.001258	0.005	1.803	1.803	77.323	42.875	0.850754
Nfix	Nfix-001	31670.0	0.000306	0.002	1.802	1.802	186.651	103.603	0.849286
Ythdc1	Ythdc1-001	5487.9	0.011374	0.031	1.800	1.800	38.908	21.611	0.848334
Zfp236	Zfp236-001	4929.1	0.016589	0.041	1.798	1.798	25.711	14.304	0.845997
Xrcc1	Xrcc1-201	2656.0	0.027872	0.062	1.797	1.797	18.162	10.105	0.845802
Atg4d	Atg4d-201	1332.7	0.036617	0.077	1.796	1.796	7.760	4.321	0.844665
Ppia	Ppia-004	3370.7	0.016824	0.041	1.792	1.792	20.077	11.204	0.841569
Fis1	Fis1-001	3434.5	0.032176	0.069	1.789	1.789	18.139	10.137	0.839421
Nelfb	Nelfb-001	3279.8	0.028486	0.063	1.787	1.787	21.559	12.064	0.837561
Abcf1	Abcf1-001	12806.2	0.000365	0.002	1.786	1.786	78.695	44.063	0.836704
Pi16	Pi16-004	3924.1	0.017347	0.042	1.785	1.785	13.459	7.539	0.83617
Crbn	Crbn-001	2298.3	0.034966	0.074	1.785	1.785	13.113	7.348	0.83566
Zfp212	Zfp212-001	2810.5	0.012096	0.032	1.784	1.784	15.776	8.841	0.835488
Rarb	Rarb-201	1515.0	0.049617	0.097	1.784	1.784	9.044	5.070	0.834888
Pin1	Pin1-201	2038.0	0.043065	0.087	1.779	1.779	11.696	6.576	0.830708
Nhsl2	Nhsl2-007	3283.1	0.023946	0.055	1.778	1.778	12.708	7.148	0.83014
Rnf39	Rnf39-001	2842.6	0.031015	0.067	1.777	1.777	16.924	9.524	0.829386
Cltb	Cltb-001	10538.8	0.001658	0.007	1.776	1.776	40.936	23.046	0.828819
Srf	Srf-201	7905.0	0.006302	0.019	1.775	1.775	63.488	35.769	0.827785
Ctsa	Ctsa-201	2181.3	0.011004	0.030	1.774	1.774	13.278	7.483	0.827266
Sipa1l3	Sipa1l3-007	2107.8	0.006871	0.021	1.774	1.774	12.765	7.195	0.827131
Plcb1	Plcb1-003	4436.2	0.008114	0.023	1.774	1.774	26.448	14.908	0.827048
Map2k5	Map2k5-201	3932.0	0.002720	0.010	1.774	1.774	21.052	11.868	0.826886
Ap2s1	Ap2s1-001	1349.0	0.049493	0.097	1.773	1.773	9.567	5.395	0.826265
Tbc1d10b	Tbc1d10b-001	4808.6	0.006720	0.020	1.773	1.773	29.249	16.501	0.825821
Rnf40	Rnf40-201	7982.0	0.004717	0.015	1.772	1.772	51.034	28.802	0.825283
Prkcsh	Prkcsh-201	5528.5	0.046428	0.093	1.772	1.772	37.731	21.297	0.825095
Zc3h3	Zc3h3-201	1737.0	0.031161	0.067	1.771	1.771	10.425	5.886	0.824675
Krt18	Krt18-201	5633.0	0.005130	0.016	1.771	1.771	46.845	26.450	0.824597
Anxa3	Anxa3-007	3135.6	0.008865	0.025	1.771	1.771	16.839	9.508	0.824581
Nfu1	Nfu1-001	1063.0	0.025674	0.058	1.766	1.766	6.350	3.595	0.820748
Tlk2	Tlk2-004	143.7	0.047151	0.094	1.764	1.764	0.749	0.425	0.819122
Zmiz2	Zmiz2-004	6467.1	0.003117	0.011	1.764	1.764	34.526	19.571	0.819008
Sppl2b	Sppl2b-201	1389.0	0.011186	0.030	1.763	1.763	7.971	4.521	0.818165
Gm10132	Gm10132-001	6753.0	0.001676	0.007	1.762	1.762	50.475	28.652	0.816963
Prdm16	Prdm16-007	3230.1	0.031487	0.068	1.761	1.761	14.931	8.480	0.816238
Rela	Rela-001	14914.0	0.007423	0.022	1.759	1.759	106.565	60.572	0.815018
Lrrfip2	Lrrfip2-003	3670.1	0.024466	0.056	1.759	1.759	23.023	13.088	0.814886
Cxx1b	Cxx1b-001	2217.0	0.006162	0.019	1.759	1.759	9.554	5.431	0.814865
Cdc42bpg	Cdc42bpg-001	1770.5	0.010131	0.028	1.758	1.758	12.054	6.855	0.814195
Hdac8	Hdac8-001	799.6	0.038349	0.079	1.758	1.758	4.755	2.704	0.814177
Capn15	Capn15-201	2023.0	0.027963	0.062	1.757	1.757	11.790	6.711	0.813081
Bcl9l	Bcl9l-201	27215.0	0.006847	0.020	1.754	1.754	103.563	59.039	0.810767
Ctdsp1	Ctdsp1-001	7957.4	0.031470	0.068	1.754	1.754	52.640	30.012	0.810633
Zbtb7c	Zbtb7c-201	3830.4	0.029134	0.064	1.753	1.753	5.506	3.141	0.809903
Cept1	Cept1-003	2577.8	0.025245	0.057	1.753	1.753	11.818	6.742	0.809695
Utp18	Utp18-001	1756.3	0.025470	0.057	1.751	1.751	12.566	7.175	0.808435
Sdccag3	Sdccag3-003	2731.9	0.029360	0.064	1.748	1.748	17.066	9.763	0.805805
Vps13d	Vps13d-002	29709.0	0.001431	0.006	1.747	1.747	151.246	86.597	0.804515

Tie1	Tie1-002	7977.5	0.024027	0.055	1.743	1.743	38.748	22.229	0.801713
Hint1	Hint1-001	5710.1	0.006308	0.019	1.741	1.741	40.403	23.203	0.800157
Terf1	Terf1-002	1180.3	0.038920	0.080	1.740	1.740	7.989	4.591	0.799083
Rbms1	Rbms1-002	5849.5	0.020869	0.049	1.738	1.738	34.662	19.945	0.797319
Adamts10	Adamts10-012	1311.7	0.034493	0.073	1.738	1.738	7.332	4.219	0.797313
Safb	Safb-004	4582.8	0.009979	0.028	1.735	1.735	26.691	15.383	0.795039
Nf1	Nf1-003	5972.5	0.022058	0.051	1.734	1.734	32.374	18.671	0.794043
Gm12060	Gm12060-001	1925.0	0.009174	0.026	1.733	1.733	12.705	7.333	0.792982
Rpl36a	Rpl36a-003	3493.0	0.011190	0.030	1.732	1.732	23.172	13.379	0.792457
Nt5c2	Nt5c2-202	3058.9	0.037313	0.078	1.731	1.731	18.582	10.737	0.791356
Fam207a	Fam207a-201	2009.5	0.027331	0.061	1.730	1.730	13.854	8.009	0.790522
Rhbdf1	Rhbdf1-007	1729.5	0.018760	0.045	1.729	1.729	11.277	6.522	0.790035
Rwdd1	Rwdd1-001	2450.9	0.010182	0.028	1.728	1.728	15.222	8.809	0.789132
Atp2a2	Atp2a2-002	6960.1	0.037921	0.079	1.726	1.726	44.374	25.710	0.787383
Afap1l1	Afap1l1-001	4376.9	0.023398	0.054	1.723	1.723	23.716	13.768	0.784513
Mical1	Mical1-003	4389.0	0.026119	0.058	1.721	1.721	25.499	14.814	0.783456
Ehbp1l1	Ehbp1l1-201	23146.1	0.000601	0.003	1.721	1.721	96.118	55.864	0.782891
Rasip1	Rasip1-001	27170.2	0.001089	0.005	1.718	1.718	129.783	75.537	0.780851
Mon1a	Mon1a-002	1121.5	0.027345	0.061	1.717	1.717	6.355	3.700	0.780204
Ctnnb1	Ctnnb1-001	17730.1	0.001508	0.006	1.717	1.717	102.972	59.986	0.779559
Vim	Vim-006	109035.0	0.028728	0.063	1.716	1.716	586.715	341.846	0.779314
Gdpd5	Gdpd5-201	2700.0	0.037270	0.078	1.716	1.716	16.771	9.776	0.778653
Lemd2	Lemd2-201	2033.0	0.003225	0.011	1.715	1.715	11.331	6.606	0.778466
Tbc1d13	Tbc1d13-001	3916.2	0.017225	0.042	1.713	1.713	23.086	13.473	0.776927
Psmd3	Psmd3-001	5171.2	0.020055	0.048	1.711	1.711	30.540	17.853	0.77459
Zfp358	Zfp358-201	3628.0	0.002278	0.009	1.709	1.709	17.270	10.103	0.773536
Tpt1	Tpt1-001	42481.5	0.003825	0.013	1.709	1.709	278.679	163.062	0.773185
Gm11560	Gm11560-001	2749.0	0.003240	0.011	1.708	1.708	13.168	7.711	0.771966
Pgrmc1	Pgrmc1-001	8498.0	0.007592	0.022	1.707	1.707	47.176	27.635	0.771583
Mbd6	Mbd6-001	4310.7	0.042948	0.087	1.706	1.706	24.684	14.466	0.77087
Dock6	Dock6-201	64649.0	0.027310	0.061	1.703	1.703	263.908	154.958	0.768157
Gm7536	Gm7536-001	5001.0	0.002848	0.010	1.700	1.700	36.082	21.220	0.765885
Klk8	Klk8-201	6916.6	0.031657	0.068	1.699	1.699	17.078	10.053	0.764483
Sf1	Sf1-006	1501.9	0.023485	0.054	1.698	1.698	10.171	5.990	0.763751
Rabl6	Rabl6-001	6845.4	0.006052	0.019	1.697	1.697	38.569	22.725	0.763188
Nos3	Nos3-001	63452.2	0.016372	0.041	1.697	1.697	189.159	111.456	0.763124
Hnrnpf	Hnrnpf-002	5816.6	0.023831	0.054	1.697	1.697	32.778	19.320	0.762585
Dusp3	Dusp3-001	12380.6	0.029047	0.064	1.694	1.694	59.427	35.086	0.760235
Cdc42bpb	Cdc42bpb-201	46535.2	0.000063	0.001	1.692	1.692	243.238	143.730	0.759003
Foxc1	Foxc1-001	31652.0	0.037570	0.078	1.691	1.691	162.944	96.350	0.758011
031414P19R	031414P19Rik-	1358.0	0.049216	0.097	1.690	1.690	9.795	5.795	0.757361
Cdc42ep5	Cdc42ep5-001	1514.3	0.049945	0.098	1.688	1.688	6.632	3.928	0.755615
Ghdc	Ghdc-001	2680.3	0.020608	0.049	1.687	1.687	12.430	7.368	0.754444
Ahnak2	Ahnak2-001	39447.0	0.043081	0.087	1.686	1.686	210.255	124.707	0.753592
Edc4	Edc4-001	5477.1	0.012941	0.034	1.686	1.686	31.307	18.570	0.753505
Als2cl	Als2cl-004	7254.9	0.027765	0.061	1.681	1.681	26.896	16.000	0.749304
Mtmr9	Mtmr9-201	3379.0	0.015841	0.040	1.678	1.678	17.224	10.262	0.747087
Xpc	Xpc-001	3132.0	0.008294	0.024	1.678	1.678	22.420	13.358	0.747052
Abca2	Abca2-001	6251.6	0.006270	0.019	1.678	1.678	34.797	20.735	0.746919
Myl6	Myl6-201	26943.0	0.001660	0.007	1.678	1.678	151.409	90.228	0.746794
Pxn	Pxn-003	2705.0	0.039483	0.081	1.677	1.677	15.449	9.212	0.745863
Chd2	Chd2-005	2839.2	0.044770	0.090	1.677	1.677	18.620	11.105	0.745585
Plin3	Plin3-201	4205.0	0.005683	0.018	1.675	1.675	24.849	14.834	0.744282
Tbc1d22a	Tbc1d22a-201	4753.0	0.002575	0.010	1.675	1.675	25.252	15.079	0.743838
Hnrnpab	Hnrnpab-003	10580.6	0.027873	0.062	1.672	1.672	72.413	43.321	0.741193
Cabp1	Cabp1-001	174.3	0.020872	0.049	1.670	1.670	0.510	0.305	0.739791
Stom	Stom-001	4147.3	0.037048	0.077	1.670	1.670	26.500	15.873	0.739469
Ptpra	Ptpra-001	13118.3	0.010189	0.028	1.669	1.669	70.957	42.524	0.738651
Col4a1	Col4a1-001	69884.9	0.019694	0.047	1.667	1.667	486.556	291.860	0.737328
Ddx41	Ddx41-201	2957.0	0.019410	0.046	1.666	1.666	19.796	11.879	0.736778
Arhgef7	Arhgef7-001	5772.5	0.018941	0.046	1.666	1.666	36.495	21.901	0.736742
Pigyl	Pigyl-001	1541.9	0.047664	0.094	1.666	1.666	8.973	5.387	0.736039
Mbd1	Mbd1-201	5381.0	0.013513	0.035	1.665	1.665	30.148	18.103	0.735845
Anapc2	Anapc2-001	9171.2	0.004589	0.015	1.663	1.663	48.378	29.094	0.733637
Dohh	Dohh-001	1625.6	0.046637	0.093	1.663	1.663	9.800	5.894	0.733635
Eif2ak3	Eif2ak3-001	4593.1	0.017342	0.042	1.657	1.657	27.148	16.385	0.728479
Kif13b	Kif13b-201	11418.0	0.016725	0.041	1.655	1.655	63.965	38.640	0.727163
Ephb4	Ephb4-002	4007.6	0.049172	0.097	1.653	1.653	20.748	12.553	0.724905
Sh3bp4	Sh3bp4-001	10426.7	0.013145	0.034	1.652	1.652	62.148	37.623	0.724087
Fcgrt	Fcgrt-201	3645.0	0.006845	0.020	1.650	1.650	22.299	13.512	0.722732
Gm9774	Gm9774-001	1468.2	0.033613	0.072	1.650	1.650	8.721	5.285	0.722554
Pkn1	Pkn1-004	5404.9	0.010617	0.029	1.650	1.650	27.126	16.443	0.722145
Uqcrh	Uqcrh-001	7603.7	0.002315	0.009	1.646	1.646	49.555	30.113	0.718644
Clec2d	Clec2d-001	20801.0	0.024124	0.055	1.644	1.644	177.402	107.879	0.717615
Ccdc88c	Ccdc88c-201	9083.6	0.006313	0.019	1.644	1.644	40.253	24.481	0.71742
Cep250	Cep250-001	6743.7	0.015223	0.038	1.644	1.644	29.083	17.691	0.717175
Wbp2	Wbp2-001	10914.2	0.003824	0.013	1.643	1.643	67.567	41.133	0.716018
Aurkaip1	Aurkaip1-006	2807.4	0.012230	0.032	1.642	1.642	16.962	10.328	0.715797
Gm13611	Gm13611-001	4795.0	0.021068	0.049	1.638	1.638	34.482	21.052	0.711872
Tmem109	Tmem109-201	11369.2	0.003408	0.012	1.637	1.637	56.761	34.664	0.711444
Idh2	Idh2-001	3456.7	0.037832	0.079	1.636	1.636	23.260	14.221	0.709789
Ube2s	Ube2s-201	2432.0	0.042815	0.087	1.631	1.631	15.627	9.579	0.706133
Plcb3	Plcb3-201	17169.0	0.001665	0.007	1.631	1.631	89.547	54.911	0.705556
Rps27rt	Rps27rt-001	16897.0	0.002904	0.010	1.630	1.630	119.464	73.298	0.704718
Hook3	Hook3-002	1187.4	0.035183	0.074	1.630	1.630	7.261	4.456	0.704533
Mapre2	Mapre2-001	5642.3	0.018975	0.046	1.619	1.619	31.985	19.750	0.695526
Efna1	Efna1-001	3751.4	0.023457	0.054	1.616	1.616	29.037	17.973	0.692028
Wash1	Wash1-201	2233.8	0.042291	0.086	1.615	1.615	12.232	7.573	0.691673
H13	H13-002	6619.0	0.017995	0.044	1.614	1.614	40.020	24.789	0.691026
Dnmbp	Dnmbp-201	4592.0	0.008065	0.023	1.612	1.612	27.730	17.202	0.688894
Sh2b1	Sh2b1-201	4100.0	0.015253	0.038	1.611	1.611	25.384	15.752	0.688327
Ppp1r12c	Ppp1r12c-004	6630.7	0.033981	0.072	1.610	1.610	30.874	19.174	0.687195
Tysnd1	Tysnd1-201	1209.0	0.042389	0.086	1.610	1.610	7.943	4.933	0.687156
Suclg1	Suclg1-001	3054.1	0.027814	0.061	1.606	1.606	16.972	10.567	0.6836
Daglb	Daglb-001	2223.1	0.040883	0.084	1.606	1.606	16.094	10.021	0.6834
Trim28	Trim28-001	5520.2	0.026561	0.059	1.604	1.604	38.522	24.024	0.681234
Stab1	Stab1-001	73013.3	0.013067	0.034	1.603	1.603	412.097	257.044	0.680968
Slc27a4	Slc27a4-001	1963.9	0.031550	0.068	1.603	1.603	11.268	7.029	0.680862
Col5a1	Col5a1-001	29326.3	0.003369	0.012	1.600	1.600	149.196	93.244	0.67812
Ndufs3	Ndufs3-001	1813.9	0.048886	0.096	1.599	1.599	10.932	6.836	0.677323
Ajuba	Ajuba-201	5617.0	0.036238	0.076	1.598	1.598	42.659	26.691	0.676517
Rin1	Rin1-201	5620.0	0.009599	0.027	1.598	1.598	24.750	15.490	0.67606
Wdr89	Wdr89-001	14072.8	0.001569	0.006	1.598	1.598	97.216	60.854	0.675841
Gm13653	Gm13653-001	761.0	0.048910	0.096	1.594	1.594	5.258	3.299	0.672227
Cyba	Cyba-201	2952.0	0.036313	0.076	1.592	1.592	19.827	12.450	0.671275
Wdfy2	Wdfy2-201	2043.0	0.042244	0.086	1.592	1.592	12.988	8.157	0.671102
Macf1	Macf1-009	47326.6	0.036480	0.076	1.590	1.590	294.890	185.485	0.668869

Sod3	Sod3-001	45755.0	0.029775	0.065	1.589	1.589	207.721	130.737	0.667977
Fam193a	Fam193a-001	6961.2	0.021658	0.051	1.587	1.587	34.926	22.003	0.666572
Fam193b	Fam193b-201	8438.0	0.009666	0.027	1.581	1.581	53.280	33.695	0.661088
Cmklr1	Cmklr1-001	22851.8	0.014730	0.037	1.581	1.581	48.126	30.447	0.660535
Ep400	Ep400-001	14040.4	0.004920	0.016	1.577	1.577	75.427	47.818	0.657528
Dgcr14	Dgcr14-201	2986.0	0.016639	0.041	1.574	1.574	17.494	11.117	0.654051
Aup1	Aup1-201	4314.6	0.012816	0.033	1.572	1.572	25.837	16.434	0.652748
Gon4l	Gon4l-202	8006.0	0.001990	0.008	1.572	1.572	45.902	29.198	0.652677
Ubtf	Ubtf-201	1800.7	0.027542	0.061	1.569	1.569	10.261	6.539	0.650147
Mgat2	Mgat2-001	5028.0	0.016483	0.041	1.569	1.569	30.667	19.550	0.649519
Egln2	Egln2-001	4918.7	0.005256	0.017	1.568	1.568	25.123	16.025	0.64864
Gm1821	Gm1821-001	5291.0	0.021893	0.051	1.568	1.568	30.132	19.221	0.648581
Gm5564	Gm5564-001	2812.0	0.009283	0.026	1.566	1.566	16.821	10.742	0.646931
Atp11a	Atp11a-001	15942.4	0.019290	0.046	1.564	1.564	97.773	62.505	0.645475
S100a13	S100a13-001	5158.0	0.034385	0.073	1.563	1.563	26.419	16.897	0.644776
Sars	Sars-002	4800.1	0.014667	0.037	1.563	1.563	27.421	17.543	0.644433
D8Ertd738e	8Ertd738e-20	4170.0	0.019028	0.046	1.563	1.563	27.300	17.466	0.644321
Sec24d	Sec24d-001	6724.8	0.006330	0.019	1.563	1.563	44.602	28.536	0.644318
Usp53	Usp53-010	14956.4	0.004608	0.015	1.563	1.563	81.619	52.220	0.644302
Plod1	Plod1-006	3059.1	0.033475	0.071	1.558	1.558	20.099	12.897	0.640018
Pcdhgb6	Pcdhgb6-001	7350.0	0.017962	0.044	1.555	1.555	41.713	26.828	0.636731
Zyx	Zyx-201	101736.3	0.017538	0.043	1.553	1.553	622.162	400.523	0.635406
Phip	Phip-010	4509.4	0.039124	0.081	1.553	1.553	26.512	17.068	0.635367
Tacc2	Tacc2-202	12002.7	0.014879	0.038	1.553	1.553	54.183	34.899	0.63468
Mpdz	Mpdz-001	7531.7	0.007986	0.023	1.552	1.552	42.553	27.425	0.633754
Prkag1	Prkag1-201	4280.4	0.014192	0.036	1.551	1.551	23.814	15.357	0.632924
Srpk2	Srpk2-001	3697.4	0.011134	0.030	1.550	1.550	19.245	12.413	0.632642
Wbp11	Wbp11-001	7914.1	0.008084	0.023	1.547	1.547	48.302	31.219	0.62963
Gcn1l1	Gcn1l1-001	11862.9	0.012513	0.033	1.543	1.543	77.418	50.159	0.62616
Rpa1	Rpa1-002	6325.3	0.021941	0.051	1.543	1.543	34.273	22.211	0.625787
Gm9493	Gm9493-201	8085.0	0.012366	0.033	1.542	1.542	57.670	37.388	0.625257
H13	H13-001	4356.5	0.026172	0.059	1.540	1.540	23.457	15.229	0.62322
Rpl19-ps1	Rpl19-ps1-00	3518.0	0.004649	0.015	1.538	1.538	23.834	15.495	0.621223
Med22	Med22-001	1999.3	0.031621	0.068	1.538	1.538	12.836	8.347	0.620821
Ppia	Ppia-001	3571.5	0.037502	0.078	1.536	1.536	18.936	12.329	0.619067
Ltbr	Ltbr-001	3868.8	0.027151	0.060	1.535	1.535	23.654	15.408	0.618376
Rpl31	Rpl31-006	3847.6	0.015712	0.039	1.535	1.535	24.824	16.174	0.618044
Habp4	Habp4-201	2786.0	0.014087	0.036	1.534	1.534	17.181	11.201	0.617169
Ric8	Ric8-001	3183.5	0.021478	0.050	1.533	1.533	15.718	10.253	0.616377
Ptk2	Ptk2-202	4310.7	0.017690	0.043	1.532	1.532	20.822	13.591	0.615431
Taok2	Taok2-002	16738.5	0.025567	0.057	1.532	1.532	90.493	59.068	0.615427
Ccdc91	Ccdc91-001	3908.5	0.023936	0.055	1.532	1.532	21.761	14.205	0.615395
Pcif1	Pcif1-001	6818.0	0.021368	0.050	1.527	1.527	36.264	23.746	0.610827
Frg1	Frg1-201	2398.0	0.048828	0.096	1.527	1.527	15.591	10.209	0.610797
Rgmb	Rgmb-201	3122.0	0.031446	0.068	1.525	1.525	17.630	11.558	0.609112
Phc2	Phc2-004	6177.5	0.008986	0.025	1.524	1.524	36.736	24.099	0.608221
Sptan1	Sptan1-002	37620.1	0.015321	0.039	1.516	1.516	188.365	124.246	0.600334
Gm10275	Gm10275-002	12588.1	0.004776	0.015	1.512	1.512	91.701	60.648	0.59646
Pelp1	Pelp1-001	4153.3	0.020858	0.049	1.508	1.508	24.564	16.292	0.59241
Notch1	Notch1-001	65947.0	0.000313	0.002	1.507	1.507	315.930	209.650	0.591625
Rpl21-ps15	Rpl21-ps15-00	3524.0	0.018950	0.046	1.506	1.506	23.595	15.668	0.59065
Fkbp1a	Fkbp1a-002	28161.185	2.05078E-09	1.31534E-06	3.1753E-07	-3149307.677	0.000149172	469.7877371	-21.5866
Mecom	Mecom-005	4425.6897	3.24645E-07	1.82142E-05	6.67649E-06	-149779.2168	0.000395765	59.27733123	-17.19248
Fryl	Fryl-009	10260.665	2.40113E-07	1.55755E-05	1.48209E-05	-67472.23691	0.00232331	156.7589346	-16.04201
Mkrn1	Mkrn1-004	1881.8752	1.60355E-07	1.25679E-05	1.55549E-05	-64288.50437	0.000371028	23.85286209	-15.97227
Tcf4	Tcf4-008	3096.2811	3.35691E-07	1.84549E-05	1.56854E-05	-63753.55918	0.000716087	45.6530659	-15.96022
Gnptg	Gnptg-202	2112.6263	9.37715E-05	0.00076168	1.62577E-05	-61509.195	0.000374112	23.01135191	-15.90851
Park7	Park7-009	695.06791	0.000127311	0.00095812	1.83749E-05	-54422.01435	0.000194125	10.56466634	-15.7319
Ndel1	Ndel1-003	708.66485	9.93686E-05	0.00079431	1.92101E-05	-52055.93608	0.000208608	10.85926099	-15.66778
Ndufa10	Ndufa10-004	2422.5689	0.000193351	0.001299069	3.21837E-05	-31071.64169	0.001175222	36.51608127	-14.92331
Tfdp2	Tfdp2-003	739.52861	0.000167087	0.001168858	4.10926E-05	-24335.30483	0.00054067	13.1573643	-14.57076
Csde1	Csde1-006	14689.952	1.0178E-07	9.62769E-06	4.43284E-05	-22558.87623	0.009380258	211.6080736	-14.46141
Psmd1	Psmd1-002	587.5392	1.94038E-07	1.39912E-05	5.06875E-05	-19728.74209	0.000420428	8.294516778	-14.26801
Adgrl2	Adgrl2-201	573.34212	0.00055841	0.002913702	6.76194E-05	-14788.65668	0.000544502	8.052458014	-13.8522
Gigyf2	Gigyf2-020	1214.2214	0.000383966	0.002182874	7.36856E-05	-13571.17147	0.001192567	16.18452964	-13.72826
Tor1aip2	Tor1aip2-005	1736.3482	0.000512431	0.002722737	8.45138E-05	-11832.38263	0.001953474	23.11425388	-13.53045
Tm9sf1	Tm9sf1-008	374.03937	8.18589E-06	0.000135117	8.70574E-05	-11486.67918	0.00050155	5.761140207	-13.48767
Ergic3	Ergic3-006	2290.8774	2.31526E-06	5.97571E-05	0.000101592	-9843.273584	0.002640662	25.99276139	-13.26492
Plcd3	Plcd3-002	830.48372	0.001009952	0.004591301	0.000102507	-9755.475753	0.000796079	7.766128363	-13.252
Nop56	Nop56-014	1563.5117	2.82373E-07	1.66746E-05	0.000104268	-9590.66804	0.002662856	25.53856679	-13.22742
Otud4	Otud4-004	663.54213	0.000614897	0.003135826	0.000106143	-9421.288742	0.001028231	9.687262517	-13.20171
Git2	Git2-007	792.38184	7.76974E-06	0.000130512	0.000107233	-9325.518305	0.001310036	12.21676748	-13.18697
Slmap	Slmap-007	247.08833	1.78875E-06	4.98835E-05	0.000109358	-9144.250415	0.000286922	2.623686906	-13.15865
Ankhd1	Ankhd1-016	1333.312	0.000261148	0.001625649	0.000145116	-6891.049021	0.002156847	14.86293811	-12.75051
Trps1	Trps1-002	945.89558	6.441E-06	0.000115319	0.00014556	-6870.005833	0.001594779	10.95614319	-12.7461
Tmem175	Tmem175-001	1337.1157	0.000223219	0.001447819	0.000181433	-5511.670649	0.002303032	12.69355507	-12.42827
Me1	Me1-002	993.75027	4.81743E-07	2.2951E-05	0.00020228	-4943.643747	0.001820287	8.998849812	-12.27136
Ube2k	Ube2k-010	805.91798	0.002332694	0.008831193	0.000227504	-4395.532096	0.002393065	10.51879513	-12.10182
Rps2	Rps2-005	4132.485	1.97946E-05	0.000250357	0.000228385	-4378.572951	0.015639621	68.47922232	-12.09625
Slc25a3	Slc25a3-005	3303.0583	2.33507E-07	1.55294E-05	0.000231699	-4315.939221	0.010400319	44.88714455	-12.07546
Zhx1	Zhx1-002	1192.6012	0.000515976	0.002737326	0.000239872	-4168.883692	0.004940599	20.59678344	-12.02545
Hnrnpr	Hnrnpr-007	1236.6113	9.14029E-06	0.000145607	0.000259291	-3856.675503	0.00567598	21.89041462	-11.91314
Chn1	Chn1-002	302.84109	0.000307287	0.001842216	0.000282381	-3541.313635	0.000768379	2.721072589	-11.79007
Syt11	Syt11-003	3825.2565	7.67677E-06	0.000129394	0.000286371	-3491.978516	0.015390154	53.74208805	-11.76983
Rorb	Rorb-001	215607.28	1.86576E-12	1.27804E-08	0.000298979	-3344.72103	0.863625143	2888.585179	-11.70767
Rorb	Rorb-005	14971.75	1.84984E-11	7.03967E-08	0.000299194	-3342.310928	0.060002423	200.5467551	-11.70663
Dnmt3a	Dnmt3a-005	247.45585	0.000405782	0.002274563	0.000322006	-3105.53576	0.00160053	4.970502929	-11.60063
Sgk3	Sgk3-001	639.1147	0.000389691	0.002207559	0.000330096	-3029.42426	0.002316764	7.018462165	-11.56483
Hnrnpk	Hnrnpk-009	1411.0373	6.2122E-06	0.000112457	0.000330663	-3024.222798	0.008227886	24.88296026	-11.56235
Mrto4	Mrto4-005	623.22172	0.001079811	0.004833184	0.000353343	-2830.114621	0.003676833	10.40585943	-11.46664
Dnajb5	Dnajb5-002	471.34499	2.30992E-05	0.000279415	0.000384478	-2600.925846	0.001220284	3.173867518	-11.34481
33404O12R	3404O12Rik-	351.27555	8.43843E-06	0.000138087	0.000395223	-2530.220086	0.002256237	5.708776076	-11.30505
Fbxw17	Fbxw17-002	198.20251	1.00995E-05	0.000155534	0.000456162	-2192.20378	0.000784866	1.720585478	-11.09817
Fbln2	Fbln2-003	10216.858	5.58325E-05	0.000521906	0.000457138	-2187.523085	0.033411128	73.08761291	-11.09508
Kalrn	Kalrn-007	258.30558	0.001129885	0.005014717	0.000459489	-2176.330563	0.001349304	2.936532398	-11.08768
Macf1	Macf1-010	852.47093	3.90268E-05	0.000402948	0.000474239	-2108.64306	0.005348236	11.27752099	-11.0421
Ube2d3	Ube2d3-009	2086.8126	2.87735E-05	0.000325816	0.000484714	-2063.070351	0.012895794	26.60493045	-11.01058
Chtop	Chtop-007	348.43142	0.000223368	0.001448183	0.000492027	-2032.4085	0.00127635	2.594065113	-10.98897
Ttc3	Ttc3-003	501.53349	3.88986E-05	0.000401894	0.000501343	-1994.642561	0.005478018	10.92668859	-10.96191
Adh1	Adh1-004	16199.123	0.000201832	0.0013428	0.000514489	-1943.67443	0.107060323	208.0904129	-10.92457
Tcf7l2	Tcf7l2-010	1572.9698	0.0001667	0.001167342	0.000521575	-1917.268206	0.012001315	23.00974016	-10.90484
Gnb1	Gnb1-008	20281.71	1.36495E-08	3.05553E-06	0.00053539	-1867.798042	0.121936008	227.7518369	-10.86712
Aebp1	Aebp1-002	5086.0397	1.34713E-05	0.000190343	0.000536209	-1864.944379	0.040705221	75.91297322	-10.86492
Megf11	Megf11-201	726.45904	1.7151E-05	0.000225889	0.000553601	-1806.354357	0.004210538	7.60572361	-10.81887
Ncor2	Ncor2-007	1564.565	0.000297657	0.001798965	0.000588015	-1700.637799	0.016525172	28.10333266	-10.73186
Ivns1abp	Ivns1abp-002	4827.7575	0.000114016	0.000880504	0.000588766	-1698.468595	0.063251296	107.4303391	-10.73002
Ccne2	Ccne2-001	244.60951	0.00016559	0.001161705	0.000617918	-1618.337282	0.002519765	4.077829444	-10.6603

10203C20R	0203C20Rik-	1432.1439	1.15446E-06	3.84258E-05	0.000941826	-1061.767249	0.017483238	18.56312966	-10.05225
Slc50a1	Slc50a1-007	552.23029	0.0001469	0.00106355	0.000618948	-1615.645108	0.003894331	6.291857542	-10.65789
Nisch	Nisch-006	437.88466	3.92307E-06	8.3044E-05	0.000657098	-1521.843715	0.00494229	7.521392917	-10.5716
Arf4	Arf4-004	6903.5359	0.000726976	0.003561569	0.000715784	-1397.068799	0.069101983	96.54022403	-10.44819
Ilk	Ilk-012	696.78128	0.000508305	0.002705011	0.00075229	-1329.274174	0.003888184	5.168462762	-10.37642
Klhl12	Klhl12-201	304.31842	8.50677E-05	0.000709761	0.00075291	-1328.179615	0.003280533	4.357137123	-10.37523
Csnk2b	Csnk2b-011	1427.4673	0.000965043	0.00442473	0.000799058	-1251.473043	0.015034303	18.81502554	-10.28941
Ipo11	Ipo11-003	1765.7294	4.63108E-05	0.000455603	0.000849054	-1177.781466	0.023530941	27.71430675	-10.20186
Rab7	Rab7-002	9716.4144	4.79085E-07	2.28852E-05	0.000872674	-1145.903206	0.099920755	114.4995141	-10.16227
Atf2	Atf2-015	4343.8618	1.27778E-05	0.000183419	0.000897656	-1114.013079	0.049741329	55.41249056	-10.12155
Tnpo3	Tnpo3-201	3139.7593	2.43945E-06	6.17525E-05	0.000952801	-1049.536812	0.044304684	46.49939663	-10.03554
Mbd5	Mbd5-008	219.11308	1.45768E-05	0.000201394	0.000965841	-1035.367349	0.001751771	1.813726265	-10.01593
Slc37a3	Slc37a3-004	166.32838	0.00441946	0.014518177	0.000971578	-1029.253888	0.002933603	3.01942257	-10.00738
Mmadhc	Mmadhc-003	1387.7533	7.12639E-05	0.000626004	0.000991	-1009.081368	0.021278178	21.47141269	-9.978827
Agbl5	Agbl5-006	260.41966	2.89043E-05	0.000326508	0.000995308	-1004.713747	0.002766853	2.779895469	-9.972569
Pja1	Pja1-004	458.99031	0.000552507	0.002890828	0.001026208	-974.4614646	0.007232236	7.047535755	-9.928461
Baz1b	Baz1b-002	1344.5386	0.000102801	0.000814274	0.001046195	-955.8449367	0.023964388	22.90623916	-9.900633
Trim2	Trim2-001	2783.3546	8.3472E-06	0.000136921	0.001056942	-946.1258605	0.040974644	38.76717066	-9.885888
Eif4enif1	Eif4enif1-012	1140.1361	0.004820725	0.015542674	0.001058472	-944.7576864	0.010898117	10.29608016	-9.883801
Prpf31	Prpf31-201	251.9812	0.00090213	0.004212506	0.001077311	-928.2373623	0.004242768	3.938295472	-9.85835
Nfix	Nfix-005	3562.198	0.00101147	0.00459759	0.001083165	-923.2199941	0.043180479	39.86508191	-9.850531
Kmt2b	Kmt2b-002	693.46683	0.000182391	0.001245145	0.001118028	-894.4318208	0.009333835	8.348479344	-9.804828
Zscan21	Zscan21-002	383.32969	7.6853E-05	0.000661246	0.001129	-885.7393887	0.005998092	5.312746353	-9.790738
Acsl4	Acsl4-004	541.0871	5.75051E-06	0.00010652	0.001152318	-867.8156964	0.010458104	9.075706427	-9.761245
Qdpr	Qdpr-005	198.30872	0.000833344	0.003952093	0.00115917	-862.6861318	0.002400842	2.071172833	-9.752692
Mbnl1	Mbnl1-007	5288.7611	1.30452E-06	4.12936E-05	0.001199191	-833.8954283	0.080384618	67.0323653	-9.703723
Eef1d	Eef1d-001	881.36961	0.001653354	0.006741355	0.001218101	-820.9500524	0.013182046	10.82180095	-9.681151
Cpeb3	Cpeb3-008	1086.5988	0.001903357	0.007528582	0.001224213	-816.8515753	0.018469355	15.08672149	-9.67393
Metap1	Metap1-007	227.33048	1.56989E-05	0.000211688	0.001261854	-792.4846576	0.002286335	1.811885541	-9.630239
Pard3	Pard3-004	247.62284	2.1519E-05	0.000266074	0.001282721	-779.5925185	0.004084767	3.184454067	-9.606576
Psen2	Psen2-005	250.05485	0.001964475	0.007731045	0.001318204	-758.6078646	0.004904039	3.720242543	-9.567211
Tmed2	Tmed2-002	5921.7037	0.000171008	0.001188037	0.001410715	-708.8603435	0.124892155	88.53109613	-9.469358
Synpo	Synpo-006	16051.327	0.000220664	0.001436287	0.001410817	-708.8093374	0.306554703	217.2888359	-9.469254
Armcx1	Armcx1-003	441.59592	0.001182922	0.005199575	0.001445069	-692.0082821	0.007793607	5.393240311	-9.434645
Srsf2	Srsf2-003	7231.0728	0.001193515	0.005233377	0.001445967	-691.5787996	0.141384083	97.77823472	-9.43375
Dlg1	Dlg1-002	1578.1779	5.86928E-06	0.000108083	0.00144867	-690.2882117	0.033433911	23.07903485	-9.431055
Fgd5	Fgd5-002	11895.435	0.000166973	0.001168535	0.0014652	-682.5007704	0.180058035	122.8897474	-9.414687
Plcb4	Plcb4-009	4127.3612	0.000702654	0.00347522	0.001478391	-676.4109028	0.063295775	42.81395204	-9.401756
Haus2	Haus2-002	1636.022	0.001945706	0.007672167	0.001489288	-671.4619823	0.03186493	21.39608925	-9.391162
Rpn2	Rpn2-002	714.75179	0.000768122	0.003711649	0.001515982	-659.638336	0.00896097	5.910999233	-9.365531
Cul4a	Cul4a-006	5554.8156	0.000186701	0.001266742	0.001531625	-652.9012362	0.106471849	69.51560179	-9.350721
Gne	Gne-009	156.65832	0.002793647	0.010161243	0.001549797	-645.2456689	0.003318944	2.141534413	-9.333705
Rbm43	Rbm43-201	722.56605	0.000566112	0.002945365	0.001593122	-627.6983006	0.019513569	12.24863408	-9.293927
Nsmf	Nsmf-009	669.52054	0.000247239	0.001562637	0.00160443	-623.2743785	0.015230402	9.492719424	-9.283724
Klhl12	Klhl12-001	229.36796	0.003306864	0.011590269	0.001643911	-608.305514	0.004321412	2.628738461	-9.248652
Ezh2	Ezh2-003	308.06001	0.000203585	0.001350271	0.001716011	-582.7468182	0.010249928	5.97311272	-9.186725
Bfar	Bfar-002	942.1215	0.000149918	0.001079706	0.001751955	-570.7909676	0.021641438	12.35273731	-9.156819
Tor1aip1	Tor1aip1-201	15291.539	2.03971E-10	3.493E-07	0.001762466	-567.386688	0.33168245	188.1922066	-9.148188
Calcoco1	Calcoco1-003	3614.1812	0.002761342	0.010065557	0.001804842	-554.0650807	0.074826519	41.45876149	-9.113912
Stk26	Stk26-002	169.76042	5.07466E-05	0.000485766	0.001806935	-553.4232466	0.003926248	2.172877022	-9.112239
Fxyd5	Fxyd5-011	2662.8174	2.37935E-05	0.000284542	0.001818589	-549.8767577	0.042290337	23.25447339	-9.102964
Mitd1	Mitd1-004	312.33382	0.005956941	0.018340945	0.001835533	-544.8009684	0.007024195	3.82678837	-9.089585
Pnisr	Pnisr-005	3440.7807	1.6109E-05	0.000215437	0.001858322	-538.119909	0.072959408	39.26091022	-9.071784
Rufy3	Rufy3-003	1097.4563	2.83777E-05	0.00032226	0.00190748	-524.2519605	0.018329481	9.609266566	-9.034117
Slc39a3	Slc39a3-201	555.08654	0.00017247	0.001194121	0.001921886	-520.3221622	0.012956974	6.741800513	-9.023261
Ap1b1	Ap1b1-002	450.24499	0.001016958	0.004612744	0.002012034	-497.0094881	0.011884774	5.906845296	-8.95713
Tsc22d1	Tsc22d1-005	6055.43	0.000156965	0.001116755	0.00203102	-492.3635598	0.145293548	71.53724871	-8.94358
Acsl1	Acsl1-002	543.19457	0.000911791	0.004244782	0.002037204	-490.868969	0.015566587	7.641154472	-8.939194
Oxnad1	Oxnad1-002	189.03387	0.000169799	0.001181314	0.002083819	-479.8880362	0.002893766	1.388683569	-8.906554
Luc7l2	Luc7l2-010	4325.397	0.000271505	0.001673696	0.002224583	-449.5223553	0.138996264	62.48192795	-8.812249
Mettl8	Mettl8-002	285.5227	0.003170585	0.011226356	0.002258366	-442.7979762	0.010867009	4.811889441	-8.790505
Sik3	Sik3-003	1321.6722	0.000580308	0.002998578	0.002280342	-438.5307459	0.038522368	16.8932429	-8.776534
Ppt2	Ppt2-001	224.87277	0.012411912	0.032637848	0.002286769	-437.298226	0.003745606	1.637946859	-8.772474
Hnrnpd	Hnrnpd-006	1157.46	0.000673103	0.003358652	0.002348546	-425.7954062	0.044160251	18.80323202	-8.734017
Dda1	Dda1-004	356.57174	0.000686501	0.003411634	0.00238268	-419.6954205	0.005448199	2.286584137	-8.713199
Eif2a	Eif2a-002	5619.4306	0.002086901	0.008095051	0.002394348	-417.6501646	0.174167527	72.74109616	-8.706151
Hnrnpd	Hnrnpd-010	1787.4117	0.001088449	0.00486422	0.002447709	-408.545319	0.066770676	27.27884697	-8.674352
Atp5b	Atp5b-005	9768.6104	0.003707513	0.01266404	0.002465982	-405.5179009	0.326335467	132.3348735	-8.663622
Pdik1l	Pdik1l-004	614.36694	0.000359183	0.002071744	0.002541914	-393.4043725	0.021751349	8.557075934	-8.619869
Mettl25	Mettl25-002	322.95304	0.013345963	0.034576342	0.002582376	-387.2402585	0.007514429	2.909889565	-8.597085
Abi1	Abi1-002	591.78147	0.000142636	0.001042084	0.002591398	-385.892162	0.025654584	9.899902839	-8.592054
Arl2bp	Arl2bp-202	2046.3342	4.95822E-05	0.000478094	0.002594516	-385.4283754	0.070257037	27.07905558	-8.590319
Capns1	Capns1-006	2749.7806	0.000495385	0.002652742	0.002600561	-384.5324474	0.070152913	26.9760714	-8.586962
Errfi1	Errfi1-002	3792.2091	0.003157245	0.011186147	0.002619281	-381.7842287	0.22548698	86.08737269	-8.576614
Mib2	Mib2-009	228.64383	3.39916E-05	0.0003653	0.002683783	-372.608341	0.003170452	1.18133672	-8.541516
Tpd52	Tpd52-002	1233.5995	0.0014847	0.00620891	0.002786959	-358.8140796	0.063856409	22.91257851	-8.487093
Ephb4	Ephb4-003	973.35112	3.76476E-05	0.00039264	0.002807184	-356.2288757	0.022706695	8.088780366	-8.476661
Chtop	Chtop-002	864.74936	0.000138684	0.001022065	0.002817126	-354.9716817	0.038661311	13.72367064	-8.47156
Ggnbp2	Ggnbp2-201	3221.5809	2.6601E-06	6.53097E-05	0.002895204	-345.3988359	0.13015084	44.95394868	-8.432119
Helz	Helz-003	788.60525	0.0006982	0.003458907	0.002902672	-344.510206	0.033902274	11.67967941	-8.428403
Tanc1	Tanc1-006	5092.8441	0.008050395	0.023240976	0.002940081	-340.126738	0.174519271	59.3586703	-8.409929
Chtop	Chtop-004	1281.7651	1.05583E-06	3.61984E-05	0.003142294	-318.2388237	0.06061359	19.2895977	-8.313966
Sgk3	Sgk3-002	255.16048	0.006203915	0.018946419	0.003160522	-316.4034762	0.00933042	2.95217724	-8.305622
Lgals8	Lgals8-002	950.79707	0.000293172	0.001776562	0.003179969	-314.4684395	0.055345212	17.40432258	-8.296771
Arpc5	Arpc5-003	9815.673	3.89935E-05	0.000402753	0.003305554	-302.5211508	0.457987146	138.5507983	-8.240892
Fbxo34	Fbxo34-204	1136.4179	4.17199E-07	2.08905E-05	0.003393055	-294.719612	0.038670449	11.39693973	-8.203199
Gapdh	Gapdh-003	842.07561	9.93757E-05	0.00079431	0.003409501	-293.2980312	0.052672501	15.4487407	-8.196224
Smarca2	Smarca2-010	2498.3646	0.002251902	0.008596484	0.003452516	-289.6438073	0.111883646	32.40640514	-8.178136
P4ha2	P4ha2-016	2025.0445	5.57555E-06	0.000104752	0.00345619	-289.3359273	0.04193072	12.13206384	-8.176602
Taf6	Taf6-005	297.16602	0.021653381	0.05055063	0.003482932	-287.1144341	0.01180743	3.390083507	-8.165482
Scmh1	Scmh1-002	515.83527	0.000367637	0.002105616	0.003503775	-285.4064414	0.025067692	7.154480708	-8.156874
Bbs9	Bbs9-004	855.73059	0.000196878	0.001317155	0.003547941	-281.8536234	0.039154945	11.03596302	-8.138802
Wdr61	Wdr61-001	279.59865	0.011318659	0.030376436	0.003563501	-280.622889	0.013688501	3.841306779	-8.132489
Nde1	Nde1-002	280.99546	0.000236997	0.001513828	0.003602281	-277.6018977	0.008140816	2.259906029	-8.116874
42434	March5-004	796.53627	0.001348092	0.005753538	0.00361223	-276.8372884	0.036304331	10.05039261	-8.112894
Armcx1	Armcx1-005	576.38643	0.01151205	0.030817519	0.003651988	-273.8234314	0.024227776	6.634132878	-8.097102
Stag1	Stag1-015	572.25786	0.024944695	0.056365812	0.003655639	-273.5499516	0.014852569	4.062919654	-8.09566
Cnot7	Cnot7-006	1023.6996	0.000377589	0.002153967	0.003674549	-272.1422613	0.049803198	13.55355484	-8.088217
Ncf1	Ncf1-002	200.04122	0.000895107	0.004188744	0.003687973	-271.1516459	0.014638034	3.969127117	-8.082956
Scmh1	Scmh1-201	333.46697	0.000807882	0.003860744	0.003824132	-261.4972283	0.019742727	5.162668326	-8.030652
Vamp4	Vamp4-001	2217.8797	3.41464E-05	0.000365779	0.003832463	-260.9288006	0.132796269	34.65037121	-8.027512
Ube3b	Ube3b-005	2201.132	7.01777E-05	0.000618618	0.00384772	-259.8941859	0.111966814	29.09952408	-8.021781
Taf5l	Taf5l-201	632.78792	0.000179669	0.001230489	0.003932673	-254.2799478	0.025887359	6.582636268	-7.990274
Nfam1	Nfam1-001	498.69362	0.000141211	0.001035871	0.003948926	-253.2334053	0.047726946	12.08605693	-7.984324
Tpst1	Tpst1-002	1226.2748	0.000159508	0.00113085	0.003952623	-252.9965833	0.062186674	15.73301609	-7.982974
Dlg1	Dlg1-201	1183.7738	6.29116E-05	0.000570897	0.003980153	-251.2466142	0.053277507	13.38579326	-7.97296
Cwc22	Cwc22-001	75.250197	0.001802216	0.007212656	0.003999915	-250.0053011	0.004817606	1.204427128	-7.965815

Gapdh	Gapdh-201	2088.655	9.84606E-06	0.000152938	0.004030446	-248.1115096	0.14597474	36.21801312	-7.954845
Srr	Srr-002	141.05514	0.00119602	0.005241002	0.004106397	-243.5224556	0.009626444	2.344255306	-7.927911
Son	Son-012	8417.3318	0.000725786	0.003556756	0.004293411	-232.9150179	0.513679334	119.6436313	-7.86366
Zcchc9	Zcchc9-006	2033.9968	0.000363086	0.002086903	0.004348878	-229.9443529	0.121553076	27.95044349	-7.845141
Fam161b	Fam161b-002	291.18247	0.000354169	0.002048687	0.004390817	-227.7480499	0.013296061	3.028152025	-7.831295
Nipa2	Nipa2-007	1745.0522	0.000125895	0.000950384	0.00442163	-226.1609617	0.111701968	25.26262451	-7.821206
Clk4	Clk4-004	3534.2358	2.69669E-06	6.57575E-05	0.004499213	-222.2610833	0.206207342	45.83186723	-7.796112
Specc1	Specc1-004	383.37161	0.006472998	0.019560629	0.004506779	-221.8879468	0.009927999	2.202903263	-7.793687
Plekha1	Plekha1-003	1866.0445	0.001413685	0.005976976	0.00455882	-219.3549859	0.107652889	23.614198	-7.777124
Slco2a1	Slco2a1-002	15167.594	0.005358047	0.016916768	0.004567768	-218.9252855	0.891433472	195.1573274	-7.774295
Gm26751	Gm26751-201	3403.6419	0.000232766	0.001492928	0.004576446	-218.5101707	0.150902503	32.97373161	-7.771557
Hspg2	Hspg2-002	2750.2993	0.000164203	0.001154577	0.004601248	-217.3323523	0.155091163	33.70632718	-7.763759
Slmap	Slmap-201	9684.0427	0.000235003	0.001504576	0.004620771	-216.4140964	0.584105997	126.4087715	-7.757651
Arf4	Arf4-002	2589.8739	0.001366389	0.005817857	0.004830336	-207.0249458	0.167799099	34.73859935	-7.693661
Abi1	Abi1-007	287.95486	0.008964021	0.025288926	0.004833749	-206.8787751	0.015997215	3.309484272	-7.692642
Plxna2	Plxna2-003	1382.8716	0.000114381	0.000882359	0.004846165	-206.3487204	0.079427962	16.38985835	-7.688941
Atf2	Atf2-201	1575.3463	0.000132192	0.000985106	0.004917335	-203.3621916	0.090046775	18.31210955	-7.667908
Add1	Add1-013	6320.5124	0.000292178	0.001771794	0.004986254	-200.551351	0.351681344	70.53016868	-7.647828
Tm9sf1	Tm9sf1-005	681.5987	0.000142699	0.001042323	0.00504284	-198.3009652	0.033305513	6.604515444	-7.631548
Trp53bp1	Trp53bp1-006	226.31127	0.009624089	0.026737916	0.005054769	-197.8329924	0.010855717	2.147618962	-7.628139
Cadps2	Cadps2-202	1357.6602	8.26082E-05	0.000695405	0.005087276	-196.5688602	0.087253033	17.15122921	-7.618891
Tm6sf1	Tm6sf1-002	2290.0272	0.002734017	0.00999894	0.005092297	-196.3750456	0.124642558	24.47668809	-7.617468
Eml1	Eml1-008	1348.9197	0.003089663	0.010992101	0.005108099	-195.7675469	0.080217221	15.70392863	-7.612998
Smarca2	Smarca2-006	7785.3051	0.000144423	0.0010511	0.005146403	-194.3104832	0.581830146	113.0556968	-7.60222
Nfia	Nfia-004	8271.4801	0.000734228	0.003586836	0.005195523	-192.4733961	0.526334798	101.3054461	-7.588515
Plekhg1	Plekhg1-201	844.67593	0.000127689	0.000959046	0.00523671	-190.9595878	0.054237523	10.35717503	-7.577124
Ankrd13a	Ankrd13a-004	1156.806	0.004549612	0.014863048	0.005300664	-188.6555945	0.083488957	15.75065881	-7.559611
Anpep	Anpep-201	9376.0042	2.97463E-06	7.01178E-05	0.005429027	-184.195084	0.621382418	114.4555867	-7.525091
Pcdhga8	Pcdhga8-003	729.18254	0.000457764	0.002498152	0.005458356	-183.2053561	0.053665027	9.83172034	-7.517318
Apobec1	Apobec1-002	1512.3079	0.000621248	0.003161627	0.00548499	-182.3157477	0.170187453	31.02785282	-7.510295
Aifm1	Aifm1-004	777.06968	0.000354317	0.002049199	0.005510816	-181.4613381	0.056446636	10.24288208	-7.503518
Cpped1	Cpped1-005	1376.4762	6.44696E-08	7.43463E-06	0.005513184	-181.3833849	0.10135967	18.38496006	-7.502898
Cct6a	Cct6a-006	3262.9353	0.001566809	0.006468562	0.005539773	-180.512802	0.290101031	52.36695004	-7.495957
P24-230L5.	24-230L5.2-0	940	9.81295E-07	3.44358E-05	0.005645044	-177.1465493	0.066202625	11.72756653	-7.4688
Nadk	Nadk-007	3427.0815	1.71454E-06	4.82711E-05	0.00572206	-174.762227	0.235123281	41.09066825	-7.44925
Plxdc2	Plxdc2-004	4239.9103	0.010118504	0.027840519	0.005728027	-174.5801826	0.36635999	63.95919393	-7.447746
Rps6kc1	Rps6kc1-005	862.97672	0.0024964	0.009307827	0.005741295	-174.1767257	0.051388632	8.950703661	-7.444408
Trim2	Trim2-004	5742.8333	0.004528734	0.014811796	0.00579746	-172.4893268	0.46883675	80.86933543	-7.430363
Ddr1	Ddr1-002	539.86141	0.00023102	0.001485801	0.005840359	-171.2223579	0.040773905	6.98140424	-7.419727
Tbrg4	Tbrg4-008	149.53203	0.001625069	0.006648185	0.005974847	-167.3683098	0.013494617	2.258571209	-7.386883
Nras	Nras-002	6453.3392	0.004972209	0.015938058	0.005993392	-166.8504139	0.519913216	86.74773534	-7.382411
Lig1	Lig1-005	164.39848	0.006231608	0.019012344	0.006017334	-166.1865412	0.00496927	0.825825723	-7.37666
Snx14	Snx14-006	735.56913	6.3692E-05	0.000575913	0.006036432	-165.6607821	0.041109569	6.810243383	-7.372088
Armcx3	Armcx3-003	2979.6692	0.001372586	0.005836882	0.006051013	-165.261598	0.220936629	36.51234033	-7.368608
Gm10275	Gm10275-001	830.92864	6.05229E-05	0.000553823	0.006107831	-163.7242456	0.093321047	15.27891801	-7.355124
Snrpb2	Snrpb2-003	1349.8286	0.007921898	0.022956682	0.006142078	-162.8113554	0.12284089	19.99989173	-7.347058
Myo18a	Myo18a-015	1009.0393	2.99016E-06	7.03387E-05	0.006148407	-162.6437521	0.077100951	12.53998794	-7.345572
Lcp1	Lcp1-003	7159.5471	0.001115186	0.004964919	0.006194163	-161.4423049	1.105582908	178.4878529	-7.334875
Tuba4a	Tuba4a-002	412.27965	0.01501275	0.037891005	0.006280144	-159.2320268	0.041501176	6.6083164	-7.314987
Agrn	Agrn-003	682.59138	0.00011604	0.000892718	0.006327266	-158.0461433	0.032529519	5.141165018	-7.304202
Rsrp1	Rsrp1-004	4759.9292	0.00015296	0.001096918	0.006394838	-156.3761259	0.407839429	63.77634996	-7.288876
Tm9sf2	Tm9sf2-002	3184.0557	0.000706552	0.003487953	0.006404775	-156.1335084	0.257518542	40.20727347	-7.286636
Kdm5d	Kdm5d-008	260.4399	5.05103E-05	0.00048431	0.006442334	-155.223245	0.027459847	4.262406487	-7.278201
Ppm1b	Ppm1b-204	276.15138	0.006206385	0.018950584	0.006488202	-154.1259069	0.006276062	0.967303702	-7.267966
Cdh13	Cdh13-007	4618.9489	0.02103832	0.049414516	0.00650985	-153.6133588	0.358228187	55.02863502	-7.26316
Add1	Add1-002	4364.4256	1.63822E-09	1.16894E-06	0.006520063	-153.3727588	0.299471015	45.93069578	-7.260898
Erlin1	Erlin1-002	1239.2137	1.50048E-06	4.4805E-05	0.00655691	-152.5108737	0.11996552	18.29604631	-7.252768
Eri2	Eri2-007	299.61222	0.000560667	0.002923696	0.006591115	-151.7193899	0.016242385	2.464284816	-7.245262
Fam188a	Fam188a-019	845.62943	0.001842985	0.007339793	0.006661661	-150.112709	0.065594977	9.846639721	-7.229902
Usp7	Usp7-001	1625.8555	7.44407E-06	0.000126423	0.006709606	-149.0400479	0.161436073	24.0604401	-7.219556
10032A03R	0032A03Rik-	412.11066	0.000186143	0.001264498	0.006720827	-148.7912203	0.026492826	3.941899848	-7.217146
Cnot2	Cnot2-004	1088.2649	0.000510053	0.002712206	0.006724967	-148.6996172	0.111482308	16.57737658	-7.216257
Tmem176a	mem176a-20	3535.6933	0.008730422	0.024753059	0.006729378	-148.6021412	0.309656001	46.01554484	-7.215311
Iffo2	Iffo2-201	3594.8357	0.000215023	0.001407594	0.006782821	-147.4312766	0.379337218	55.92617031	-7.203899
Mysm1	Mysm1-003	192.86445	0.008835111	0.024981635	0.007002147	-142.8133413	0.0201217	2.873647162	-7.157987
Ktn1	Ktn1-003	233.52813	0.000239417	0.001524452	0.007019713	-142.4559598	0.021302853	3.034718355	-7.154372
Stim2	Stim2-002	657.22133	0.007831296	0.022748018	0.007057174	-141.6997785	0.035714905	5.060794184	-7.146694
C77080	C77080-002	1025.2705	0.000277758	0.001703044	0.007213371	-138.631432	0.064409636	8.929200084	-7.115111
Ttc3	Ttc3-004	396.6142	0.00250627	0.009335482	0.007361822	-135.8359296	0.036750692	4.992064469	-7.085721
Clip1	Clip1-005	3035.5826	2.39556E-07	1.55689E-05	0.007446929	-134.2835378	0.210092634	28.21198223	-7.069139
Hnrnpk	Hnrnpk-021	2669.8002	0.002905217	0.010493954	0.007478161	-133.7227121	0.293356204	39.22838724	-7.063101
Vrk1	Vrk1-201	367.99284	0.004299493	0.014212686	0.007496284	-133.3994253	0.061687043	8.22901604	-7.059609
Fam188a	Fam188a-002	389.25456	0.005792682	0.017944951	0.007650305	-130.7137368	0.039987047	5.22685639	-7.030267
Rab23	Rab23-003	942.2821	9.12772E-07	3.32933E-05	0.007705294	-129.780905	0.083895467	10.88802969	-7.019934
Ctnnd1	Ctnnd1-023	4867.0896	0.002424384	0.009098444	0.007713474	-129.6432752	0.506479539	65.6616663	-7.018404
Vwf	Vwf-006	12775.186	8.30889E-06	0.00013662	0.007720565	-129.524203	1.140865235	147.7696604	-7.017078
Leprot	Leprot-002	5237.8239	2.98777E-05	0.000334634	0.007774979	-128.6177074	0.516583646	66.4418042	-7.006945
Gnas	Gnas-001	16002.326	3.04522E-07	1.73677E-05	0.007856688	-127.2800908	1.363191221	173.5071023	-6.991863
Mgat4a	Mgat4a-201	3155.788	5.15394E-06	9.96068E-05	0.008076191	-123.8207525	0.395453738	48.96537938	-6.952109
Pls3	Pls3-005	6489.3951	4.16236E-08	5.58022E-06	0.008106127	-123.3634754	0.728002654	89.80893757	-6.946772
P23-173N1	23-173N16.8-	2768.9988	2.22491E-06	5.83932E-05	0.008211488	-121.7806124	0.312082184	38.00555947	-6.928141
Socs2	Socs2-202	890.60186	2.62967E-05	0.000305932	0.008228837	-121.5238545	0.082589447	10.03658797	-6.925096
Echs1	Echs1-002	836.86996	0.000407407	0.00228188	0.008241162	-121.3421069	0.090023301	10.92361702	-6.922936
Nisch	Nisch-012	111.80633	0.000560784	0.002923863	0.008294937	-120.5554615	0.003547973	0.427727566	-6.913553
Hdac2	Hdac2-003	2326.6873	0.009497587	0.026474513	0.008312833	-120.2959377	0.271460592	32.65560645	-6.910444
Nxf1	Nxf1-006	1155.8309	0.010969171	0.029633548	0.008332116	-120.0175392	0.134360804	16.1256531	-6.907101
Eif2s2	Eif2s2-201	2561.1907	6.71534E-05	0.000599427	0.00838418	-119.2722474	0.339626321	40.5079946	-6.898115
Zfp583	Zfp583-001	189.24679	0.000457058	0.002495492	0.008386326	-119.2417287	0.014487448	1.727508338	-6.897745
Tmem39a	Tmem39a-003	2288.0302	0.001259263	0.005459464	0.008433369	-118.5765794	0.278331246	33.00356713	-6.889675
Lin54	Lin54-003	292.12113	9.96403E-05	0.000795682	0.00843805	-118.5107949	0.015874261	1.88127131	-6.888875
Snap23	Snap23-003	5381.3558	3.02914E-07	1.73524E-05	0.008453689	-118.2915534	0.693446904	82.02891142	-6.886203
Arid4a	Arid4a-002	470.17105	0.00756626	0.022154776	0.008465919	-118.120672	0.047735805	5.638585349	-6.884118
Nfyc	Nfyc-003	468.60418	0.001204429	0.005270434	0.008479417	-117.9326294	0.070433452	8.306402231	-6.881819
10035C23R	0035C23Rik-	1728.8294	0.00822992	0.02360958	0.008500902	-117.6345795	0.186671361	21.95900706	-6.878168
Cetn2	Cetn2-001	506.49164	0.000953378	0.00438651	0.008527666	-117.2653793	0.062087902	7.280761432	-6.873633
Eif4a2	Eif4a2-006	3802.2935	6.4388E-07	2.67632E-05	0.008551146	-116.9433839	0.423026573	49.47015897	-6.869666
Serpine1	Serpine1-201	18951.406	0.01191787	0.031639955	0.008575278	-116.614292	4.036615024	470.7270033	-6.865601
Wnk1	Wnk1-015	1087.6362	0.000528264	0.002786545	0.008606935	-116.1853826	0.100328708	11.65672936	-6.860285
Pard3	Pard3-007	209.14551	0.008159681	0.023461573	0.008716601	-114.7236191	0.020530825	2.355370499	-6.842019
Erbb2ip	Erbb2ip-002	6246.6187	5.42568E-07	2.43727E-05	0.008733603	-114.5002802	0.751550615	86.05275607	-6.839207
Ddhd1	Ddhd1-004	784.49857	0.000298752	0.001804632	0.008792221	-113.7368999	0.091854964	10.44729886	-6.829557
Wfs1	Wfs1-002	1311.9567	0.000185841	0.001263408	0.008808024	-113.5328444	0.151173382	17.16314405	-6.826966
Arl5b	Arl5b-002	2627.2017	2.07061E-05	0.000259148	0.008809836	-113.5094967	0.304238083	34.53391165	-6.826669
Sipa1l3	Sipa1l3-017	303.82602	0.001155273	0.005104245	0.008844724	-113.0617498	0.033161476	3.749294451	-6.820967
Pisd	Pisd-008	345.56521	0.002113273	0.008169256	0.008867668	-112.7692151	0.060805281	6.856963839	-6.817229
Wasf2	Wasf2-003	2387.0458	0.001297289	0.005591764	0.008899838	-112.3615997	0.23534167	26.44336655	-6.812005
Map2k7	Map2k7-006	2399.4489	8.68721E-05	0.000719383	0.00895068	-111.723358	0.313206175	34.99244566	-6.803787

Nat9	Nat9-003	402.63748	0.003299641	0.011571986	0.00895394	-111.6826773	0.045101683	5.03707674	-6.803262
Swt1	Swt1-003	225.74759	0.016796604	0.041384409	0.009084923	-110.0724852	0.022557848	2.482998342	-6.78231
Ociad2	Ociad2-001	93.440531	0.002940863	0.010590369	0.009139979	-109.4094391	0.011176082	1.222768814	-6.773593
Otud5	Otud5-003	2282.121	0.005062359	0.016152953	0.009183945	-108.8856712	0.280660635	30.55992163	-6.76667
Nup205	Nup205-005	913.04078	0.001880225	0.007455166	0.009245826	-108.1569133	0.093677205	10.13183731	-6.756982
Uhrf1	Uhrf1-004	602.84057	0.003183353	0.01126225	0.009334496	-107.1295144	0.111884768	11.98616091	-6.743212
Chtop	Chtop-006	4569.065	3.22486E-06	7.39401E-05	0.009367603	-106.7508909	0.544156278	58.08916748	-6.738104
Ogt	Ogt-005	3176.1988	0.000746894	0.003632136	0.009413609	-106.2291835	0.362761902	38.53590068	-6.731036
Fam135a	Fam135a-005	699.98137	1.60334E-05	0.000214919	0.009417265	-106.1879468	0.042570184	4.52044046	-6.730476
Ndufv1	Ndufv1-003	825.54981	0.00518155	0.016456611	0.009426027	-106.089231	0.111032733	11.77937725	-6.729134
Dpy19l4	Dpy19l4-002	2710.464	1.25252E-05	0.000180932	0.009446366	-105.8608116	0.322373482	34.12671842	-6.726025
Mtf2	Mtf2-005	320.5398	0.02423237	0.055124779	0.009461089	-105.6960737	0.038081056	4.025018116	-6.723778
Tns1	Tns1-007	4032.3515	0.018466428	0.044597034	0.009497032	-105.2960548	0.278152354	29.28834553	-6.718308
Cox4i1	Cox4i1-006	2358.0709	0.006500425	0.019615184	0.009525393	-104.9825466	0.306051049	32.13001853	-6.714006
Rtn4	Rtn4-008	13339.93	9.05955E-08	8.98218E-06	0.00954555	-104.7608545	1.920595274	201.203202	-6.710956
Adam15	Adam15-009	4263.114	0.001634132	0.006676489	0.009586702	-104.3111643	0.189617343	19.77920587	-6.70475
Brd3	Brd3-201	1543.884	0.000237245	0.001514567	0.009624048	-103.906385	0.183320774	19.04819887	-6.69914
Tardbp	Tardbp-025	3071.2479	0.003793618	0.012878523	0.009639885	-103.7356748	0.408467004	42.37260029	-6.696768
Fnbp1l	Fnbp1l-005	2695.518	0.013523333	0.034927543	0.00971786	-102.9033129	0.329873019	33.94502644	-6.685146
Lgals8	Lgals8-006	2778.7537	0.002404173	0.009041717	0.009797603	-102.0657785	0.362766657	37.02606125	-6.673355
Zfp207	Zfp207-006	3144.5871	0.012344462	0.032497911	0.009829011	-101.7396406	0.494963025	50.35736032	-6.668738
Fkbp14	Fkbp14-004	479.87198	0.003217959	0.011354705	0.009860172	-101.4181142	0.042013787	4.260959092	-6.664172
Rmnd5a	Rmnd5a-005	2480.8873	0.001015155	0.004607003	0.009867023	-101.3476947	0.360488736	36.53470237	-6.663169
Acadvl	Acadvl-005	1114.9681	6.71036E-07	2.75005E-05	0.009911526	-100.8926421	0.128673128	12.98217183	-6.656677
Bgn	Bgn-009	25109.404	4.89787E-07	2.31674E-05	0.01008355	-99.17141811	2.515879664	249.503354	-6.631852
Nat10	Nat10-003	298.06286	0.016996259	0.041762097	0.010094141	-99.06736728	0.026445078	2.619844247	-6.630338
Arhgef10	Arhgef10-007	3607.8624	0.003118343	0.011069987	0.010118055	-98.83322825	0.244704778	24.18496313	-6.626924
Cxcl12	Cxcl12-002	11276.355	0.029239548	0.063962095	0.010319033	-96.90830825	2.525124923	244.7055844	-6.598548
Rab6a	Rab6a-003	8270.0305	4.24304E-06	8.77031E-05	0.010546436	-94.81876246	1.155236906	109.5381337	-6.567101
Pdcd10	Pdcd10-002	404.49442	0.000450383	0.002467652	0.010639594	-93.98854719	0.054867305	5.156898291	-6.554413
Ktn1	Ktn1-201	1142.4025	1.07843E-05	0.000163218	0.010655553	-93.84778158	0.180668568	16.95534428	-6.552251
Rcor3	Rcor3-006	386.74147	0.001660328	0.006758527	0.010658429	-93.82246141	0.050295547	4.718851977	-6.551861
Atp11a	Atp11a-003	8919.4942	1.38927E-05	0.000194691	0.010700943	-93.44970714	1.369341325	127.9645458	-6.546118
Prrc2c	Prrc2c-009	3817.7917	0.001965771	0.007733477	0.010714728	-93.32947984	0.589238777	54.99334856	-6.544261
Laptm5	Laptm5-010	1419.1061	0.005033105	0.016083584	0.010746823	-93.05075159	0.357561695	33.27138445	-6.539946
Gpaa1	Gpaa1-002	569.70001	0.001031718	0.004666874	0.01074821	-93.03874515	0.083186772	7.739592873	-6.53976
Hmox2	Hmox2-002	1201.8011	0.000347184	0.00201824	0.010801451	-92.58015003	0.13717759	12.69992188	-6.532631
Wnk1	Wnk1-002	11937.493	0.01896395	0.045560838	0.010880707	-91.90578906	1.500797445	137.9319734	-6.522084
Slc25a39	Slc25a39-012	282.55477	0.000131955	0.000983776	0.011001194	-90.89922476	0.024162018	2.19630872	-6.506196
Prdx5	Prdx5-003	1017.7644	0.011794321	0.031392253	0.011034633	-90.62376603	0.182291379	16.5199313	-6.501818
Lig1	Lig1-008	694.50627	0.000225968	0.00146197	0.011043505	-90.55096405	0.132588191	12.00598849	-6.500658
Uap1	Uap1-005	2802.7624	2.50328E-07	1.58908E-05	0.011048889	-90.50683631	0.297508705	26.92657165	-6.499955
Rtn4	Rtn4-009	14988.038	3.07267E-08	4.59559E-06	0.01114449	-89.73044329	2.355672273	211.3755173	-6.487526
Tpx2	Tpx2-201	471.75365	0.024307483	0.055266284	0.011157839	-89.62309004	0.092760632	8.313494459	-6.485799
Wscd1	Wscd1-003	1939.1518	0.00061123	0.003122706	0.011167762	-89.54345442	0.199385072	17.85362808	-6.484516
Fbxl5	Fbxl5-004	1601.6586	0.001404779	0.005946566	0.011268492	-88.74302022	0.280226204	24.86811971	-6.471562
Pfkp	Pfkp-011	1095.3062	0.002213712	0.008481893	0.011317278	-88.36047234	0.213284961	18.84595987	-6.465329
Zc3h15	Zc3h15-003	3153.761	0.002821079	0.010241886	0.011434465	-87.45489863	0.507769239	44.40690731	-6.450467
Gm8865	Gm8865-001	519	1.18895E-06	3.90495E-05	0.011490782	-87.02627855	0.067618821	5.884614395	-6.443379
Zeb2	Zeb2-002	4097.1103	2.82695E-05	0.000321769	0.01151704	-86.82786348	0.846067074	73.46219636	-6.440086
Gm37376	Gm37376-001	1445284.4	6.4414E-10	7.11671E-07	0.011517318	-86.82577191	180.9184042	15708.3801	-6.440051
Zfp93	Zfp93-002	900.42777	0.000329112	0.001940116	0.011581859	-86.34192169	0.094013869	8.117338121	-6.431989
Cit	Cit-001	970.98933	0.006302807	0.019152795	0.011650883	-85.83040247	0.185691709	15.93799413	-6.423417
Rapgef1	Rapgef1-002	7387.6771	0.000653593	0.003286196	0.011796609	-84.77012512	0.81956947	69.47500651	-6.405484
Fmr1	Fmr1-009	128.56872	0.006582322	0.019814075	0.011819577	-84.60539946	0.018079397	1.529614574	-6.402678
Il1b	Il1b-001	5840.2427	0.009024466	0.025426783	0.011964428	-83.58109807	1.771873684	148.0951482	-6.385105
Sncaip	Sncaip-010	492.28636	0.001314841	0.005645563	0.011964663	-83.57945125	0.024823228	2.074711758	-6.385076
Gpatch2	Gpatch2-001	1047.2124	0.004035593	0.013513124	0.012024003	-83.16698197	0.152824151	12.70992338	-6.377939
Morf4l2	Morf4l2-203	542.75493	0.000242917	0.001541296	0.012106269	-82.60182994	0.099759953	8.240354657	-6.368102
Cmklr1	Cmklr1-002	7280.7769	0.000579379	0.002995731	0.012133222	-82.41834184	0.532239659	43.86631019	-6.364894
Slc25a36	Slc25a36-004	461.01809	0.018285054	0.044227619	0.012214546	-81.869599	0.064677661	5.295134158	-6.355256
Dnm2	Dnm2-006	1794.771	0.018243211	0.044165633	0.012217976	-81.8466153	0.22383305	18.31997754	-6.354851
Samd4	Samd4-002	3209.9932	1.13169E-05	0.000168596	0.012254473	-81.60285325	0.671229412	54.77423518	-6.350548
Apbb2	Apbb2-011	1070.3305	0.000252738	0.001588016	0.012257632	-81.58182694	0.134336726	10.95943552	-6.350176
Nipa2	Nipa2-004	656.583	0.00317839	0.011249341	0.012276147	-81.45878189	0.091721679	7.471536255	-6.347998
Tcf7l2	Tcf7l2-006	1964.6717	0.002122457	0.008194584	0.012319024	-81.17526509	0.325967706	26.46051492	-6.342968
Snrpa	Snrpa-201	337.52361	0.006028125	0.01852187	0.012367159	-80.85931437	0.089358201	7.225442901	-6.337342
Pcmtd1	Pcmtd1-005	3360.334	0.002754217	0.010050097	0.012619642	-79.24154871	0.412504342	32.68748292	-6.308185
Gm20721	Gm20721-201	19531.273	1.16039E-09	9.64042E-07	0.012688012	-78.81455036	2.735279999	215.5798632	-6.30039
Osbpl9	Osbpl9-019	248.67076	0.002773416	0.010099892	0.012730393	-78.55216741	0.048512477	3.810760233	-6.295579
Plxnd1	Plxnd1-002	1698.8128	0.002360258	0.00891048	0.01289299	-77.56152681	0.354841537	27.52205138	-6.277269
Trappc13	Trappc13-002	1049.8364	0.001133122	0.005026475	0.012933857	-77.31645601	0.158685884	12.2690302	-6.272704
Bbs7	Bbs7-002	314.78269	0.003060848	0.010919076	0.012976626	-77.06163605	0.037749623	2.909047743	-6.267941
Eif5a	Eif5a-005	1851.6491	0.031307423	0.067621823	0.013041919	-76.67583237	0.410823658	31.50024597	-6.2607
Sdf4	Sdf4-006	311.73962	0.006311136	0.019171586	0.01305044	-76.62576755	0.052747835	4.041843332	-6.259758
Cnp	Cnp-002	144.21075	0.030165687	0.065631736	0.013075586	-76.47841016	0.015912309	1.216948131	-6.256981
Mtus1	Mtus1-001	2514.507	3.61253E-06	7.93643E-05	0.013098858	-76.34253511	0.425591977	32.49077044	-6.254415
Psmb2	Psmb2-003	354.77165	0.002756627	0.010053719	0.013171222	-75.92310085	0.031910473	2.422742062	-6.246467
Zfp106	Zfp106-009	795.16819	0.001799005	0.007204854	0.013352215	-74.89393933	0.158294339	11.85528662	-6.226777
Med7	Med7-004	757.74546	8.94472E-05	0.000735325	0.013547332	-73.8152709	0.092583877	6.83410398	-6.205847
Gar1	Gar1-005	1783.428	0.021539227	0.050352777	0.013626038	-73.38890473	0.401757172	29.48451881	-6.19749
Gng10	Gng10-002	555.02175	0.018206546	0.04409066	0.013685563	-73.06970376	0.092854685	6.784864335	-6.191201
Abcd3	Abcd3-004	2172.9137	5.17113E-09	1.84491E-06	0.013707443	-72.95306698	0.377445506	27.53580726	-6.188897
Poc1b	Poc1b-001	797.2032	0.04897422	0.096500232	0.013718528	-72.89411788	0.123910027	9.032312109	-6.18773
Cox16	Cox16-001	668.72212	0.002919279	0.010533638	0.013761626	-72.66583045	0.071920201	5.226141132	-6.183205
Fam214b	Fam214b-003	329.99382	0.002383892	0.0089832	0.013946754	-71.70127434	0.040809024	2.926059042	-6.163927
Mpzl1	Mpzl1-002	550.66145	0.007888071	0.022883826	0.013964235	-71.61151401	0.133766184	9.579198978	-6.16212
Epn2	Epn2-006	361.34233	0.000792159	0.003798327	0.013987429	-71.49276792	0.083641983	5.979796877	-6.159725
Stab1	Stab1-007	3667.4095	0.007826985	0.022738419	0.014220748	-70.3197908	0.680597656	47.85948476	-6.135859
Psmb9	Psmb9-006	623.83567	0.011662394	0.031123851	0.014280273	-70.02667132	0.110962045	7.770302688	-6.129833
Set	Set-001	2350.6713	0.008537939	0.024310545	0.014309021	-69.8859823	0.675231937	47.18924721	-6.126931
Socs2	Socs2-201	425.66052	0.001374542	0.005842402	0.014343539	-69.71780163	0.068446332	4.771927786	-6.123455
Ergic2	Ergic2-014	302.19112	0.002648808	0.009751875	0.014411288	-69.39005095	0.066536185	4.616949258	-6.116657
Cers5	Cers5-002	2101.092	0.002046834	0.007970902	0.014416362	-69.36562773	0.321984166	22.33463383	-6.116149
Gp49a	Gp49a-002	5649.4908	0.000469175	0.002547033	0.0144258	-69.32024499	2.036153264	141.1466431	-6.115205
Psme1	Psme1-009	3761.8649	1.80363E-06	5.01414E-05	0.014429012	-69.30481483	0.683499461	47.36980359	-6.114884
Fbxl17	Fbxl17-002	2087.067	0.000775058	0.003737078	0.014449865	-69.20479679	0.451099332	31.2182376	-6.1128
Ifitm1	Ifitm1-001	1114.4659	0.010268392	0.028168884	0.014477688	-69.07180216	0.355218357	24.5355721	-6.110025
Dnajc13	Dnajc13-004	428.64933	0.010147267	0.027906206	0.014568302	-68.64217809	0.103182752	7.082688844	-6.101023
Grb2	Grb2-001	564.2008	0.007827327	0.022738419	0.014633083	-68.3383009	0.082523644	5.639525635	-6.094622
Nrbp1	Nrbp1-006	1903.6864	0.018653134	0.044980731	0.014644868	-68.28330608	0.268979319	18.36679716	-6.093461
Atp2c1	Atp2c1-001	3313.1573	0.001369658	0.005828149	0.014690724	-68.07016475	0.562270859	38.27386999	-6.088951
Ccni	Ccni-006	1304.0301	0.000159895	0.001132217	0.014696871	-68.04169295	0.220951821	15.03393599	-6.088347
Dleu2	Dleu2-007	2993.3064	0.000291807	0.001770797	0.014731779	-67.88046224	0.586663918	39.8230179	-6.084924
Rnf20	Rnf20-002	1451.715	1.57053E-05	0.00021169	0.014762483	-67.73928049	0.251930817	17.06561227	-6.081921
Cnot11	Cnot11-002	1099.2373	0.003492588	0.012080504	0.014786857	-67.62762392	0.229095835	15.493207	-6.079541
Smco4	Smco4-201	897.6961	0.000330421	0.001945825	0.014801476	-67.56082909	0.155503389	10.5059379	-6.078115

Ttc7	Ttc7-007	701.94237	0.010193869	0.028007382	0.01486663	-67.26473903	0.179210731	12.05456307	-6.071779
Sema6d	Sema6d-004	351.13542	0.001166148	0.005141018	0.014870339	-67.24796208	0.078807852	5.299667464	-6.071419
Gpr35	Gpr35-004	1435.5123	0.002786952	0.010140562	0.014907916	-67.07845561	0.520445869	34.91070515	-6.067778
Tm6sf1	Tm6sf1-001	318.36976	0.000142062	0.00103944	0.014937974	-66.94348453	0.030491907	2.041234508	-6.064872
Uqcrh	Uqcrh-005	2193.3556	2.4803E-06	6.24176E-05	0.015157908	-65.97216406	0.439193363	28.97453661	-6.043786
10004B18R	0004B18Rik-	285.6991	0.016131497	0.040059729	0.015170714	-65.9164759	0.0786327	5.183190443	-6.042567
Nfia	Nfia-002	4093.4085	8.17783E-06	0.000135114	0.015198445	-65.79620306	0.726997247	47.83365848	-6.039932
Trp53	Trp53-202	590.04051	0.006960467	0.020731889	0.015225261	-65.68032064	0.141399807	9.287184651	-6.037389
Tfpi	Tfpi-007	534.49589	0.03488219	0.073729636	0.01535159	-65.13983261	0.108481364	7.066457917	-6.025468
Ahcyl1	Ahcyl1-004	5307.6914	3.3114E-07	1.83224E-05	0.015437946	-64.77545553	1.211184155	78.45500538	-6.017375
Abcc9	Abcc9-201	395.17215	7.14194E-05	0.000626727	0.015456563	-64.69743685	0.063179799	4.087571063	-6.015637
Klf3	Klf3-008	2394.6884	0.002373211	0.00895083	0.015579147	-64.18836565	0.319374697	20.50013985	-6.00424
Mef2a	Mef2a-003	1161.3669	0.000489396	0.002630128	0.015590997	-64.13958071	0.234827539	15.06173989	-6.003143
Zfp788	Zfp788-002	300.68289	0.004813257	0.015524442	0.015599563	-64.10435976	0.059278514	3.80001116	-6.002351
Zmym3	Zmym3-013	99.351054	0.006217901	0.018977287	0.015647828	-63.9066321	0.020572035	1.314689489	-5.997894
Hagh	Hagh-201	117.73989	0.010729625	0.029119625	0.015705868	-63.67046932	0.017050637	1.085622036	-5.992552
Atp6v1a	Atp6v1a-004	3317.9287	0.017937976	0.043569653	0.015791341	-63.32584594	0.627682293	39.74851216	-5.984723
Idh3g	Idh3g-008	877.48989	0.008026372	0.023188802	0.015810871	-63.24762274	0.144257214	9.123925845	-5.982939
Hspa8	Hspa8-006	3037.3724	0.005094291	0.016230648	0.01586176	-63.04470812	0.782105114	49.30758863	-5.978303
Stxbp1	Stxbp1-002	2618.9624	0.000296634	0.001794052	0.015960495	-62.65469839	0.340482348	21.33281879	-5.969351
Rbpj	Rbpj-012	2873.1682	0.001413001	0.005975462	0.016008114	-62.46832197	0.721003598	45.03988489	-5.965053
Tpm3	Tpm3-201	1988.1521	1.37653E-05	0.000193451	0.016070858	-62.22443123	0.480187178	29.87937401	-5.959409
Pecam1	Pecam1-005	1066.0452	0.001135593	0.005034828	0.016260771	-61.49770015	0.151085215	9.291393272	-5.942461
Gprasp1	Gprasp1-201	1492.28	0.000963493	0.004421766	0.016417166	-60.91185355	0.468137213	28.51510537	-5.928651
Luc7l2	Luc7l2-005	3768.5374	0.000958831	0.004403912	0.016541375	-60.45446484	0.858272829	51.88642456	-5.917777
Gtf2h2	Gtf2h2-001	535.8663	0.007791983	0.022653036	0.01655785	-60.39431553	0.105118033	6.348531674	-5.916341
Clcn3	Clcn3-003	930.22422	0.012041729	0.031897077	0.016596874	-60.25231043	0.154602718	9.315170958	-5.912945
Rtn4	Rtn4-001	1063.123	6.8946E-05	0.000611286	0.016597283	-60.25082589	0.271212991	16.3408067	-5.912909
Sec11a	Sec11a-004	1492.119	0.010294009	0.028223647	0.016652332	-60.05164747	0.344852993	20.70899036	-5.908132
Stt3a	Stt3a-005	1092.6383	0.002212001	0.008476287	0.016694871	-59.8986375	0.290000203	17.37061705	-5.904451
Elmod2	Elmod2-201	145.61565	0.020858523	0.049069608	0.016746268	-59.71479602	0.025818044	1.541719228	-5.900017
Bnip2	Bnip2-002	15451.376	1.17481E-06	3.87268E-05	0.016931556	-59.06131867	2.701915354	159.5786838	-5.884142
St6gal1	St6gal1-201	3265.613	1.09189E-05	0.000164673	0.017027771	-58.72759402	0.743007181	43.63502409	-5.875967
Prpf38b	Prpf38b-005	826.63982	0.002488934	0.009288078	0.017107321	-58.45450546	0.152606803	8.920555181	-5.869242
Pld1	Pld1-003	474.00651	0.000445693	0.00244592	0.017125759	-58.39157111	0.085241054	4.977359084	-5.867688
Rad50	Rad50-002	2484.4924	9.83981E-05	0.000787783	0.017205013	-58.12259335	0.499113292	29.00975891	-5.861027
Arhgef11	Arhgef11-004	3412.8529	3.81228E-05	0.000395693	0.017211582	-58.10041068	0.684371223	39.7622491	-5.860476
Csde1	Csde1-004	11494.832	5.76966E-05	0.00053524	0.017234421	-58.02341657	2.533127253	146.9806978	-5.858563
Vrk2	Vrk2-001	528.68808	0.017703858	0.043129465	0.017240421	-58.00322491	0.100253757	5.815041239	-5.858061
Lrrc8a	Lrrc8a-002	7951.2965	1.96408E-06	5.33425E-05	0.017375323	-57.55288811	1.892994311	108.9472898	-5.846816
Snx3	Snx3-003	2549.7536	0.019081705	0.04576349	0.017391439	-57.4995544	0.513264488	29.51247937	-5.845479
Whsc1	Whsc1-006	1164.5692	0.001035412	0.004677245	0.017476802	-57.21870675	0.303274375	17.35296755	-5.838415
Phf8	Phf8-006	993.73664	0.000923458	0.004285696	0.017479633	-57.20943777	0.253731593	14.51584179	-5.838181
Rnf168	Rnf168-201	3971.4422	3.79356E-05	0.000394802	0.017566909	-56.92521172	0.854522665	48.64388365	-5.830996
Apobec3	Apobec3-201	171.65648	0.003217644	0.011354705	0.017596046	-56.83094868	0.049051616	2.787649887	-5.828605
Creb1	Creb1-004	3492.6995	0.001616826	0.006621581	0.017841118	-56.050299	0.740439373	41.50184824	-5.80865
Sumo2	Sumo2-003	3088.2897	0.005860621	0.018107917	0.017856696	-56.00140083	0.845133389	47.3286537	-5.807391
Slc38a1	Slc38a1-202	961.75348	0.000339333	0.001984804	0.017882407	-55.92088331	0.412129891	23.04666755	-5.805315
BC029214	3C029214-01(	170.03079	0.00128932	0.005565116	0.017940703	-55.73917549	0.019413728	1.082105168	-5.80062
Pfkfb2	Pfkfb2-201	181.21673	0.008759617	0.024815324	0.01812911	-55.15990444	0.054818292	3.023771725	-5.785548
Ccm2	Ccm2-201	685.94649	0.001516383	0.006303691	0.018437193	-54.23819071	0.191871956	10.40678775	-5.761237
St7	St7-005	116.05007	0.007817433	0.022717385	0.018533249	-53.95708041	0.028656025	1.546195438	-5.75374
Pcolce	Pcolce-002	128.35524	0.03869975	0.080021245	0.018596677	-53.77304875	0.023618133	1.270019034	-5.748811
E2f7	E2f7-001	800.39935	0.001439984	0.006064117	0.01862485	-53.69170625	0.163822242	8.795895673	-5.746627
Ranbp17	Ranbp17-002	886.27935	5.24823E-07	2.38407E-05	0.018834084	-53.09522756	0.213576909	11.33991458	-5.73051
Gpx4	Gpx4-006	534.46924	0.002959805	0.010641752	0.018883288	-52.95687796	0.110452325	5.849210281	-5.726746
Ctdspl2	Ctdspl2-006	1158.2787	0.003393826	0.011812859	0.018901176	-52.90676232	0.192219464	10.16970949	-5.72538
)30015E06F	0015E06Rik-	2564.7064	5.37638E-08	6.57708E-06	0.01893245	-52.81936634	0.552054491	29.15916839	-5.722995
Actr3	Actr3-201	9566.1563	0.000733974	0.003586617	0.018960093	-52.74235683	3.279409161	172.9637682	-5.72089
Cacnb4	Cacnb4-001	429.10141	0.030339969	0.065939714	0.019067788	-52.44446935	0.113079329	5.930385383	-5.712719
Bcl7b	Bcl7b-003	129.42415	0.012646296	0.033116878	0.019106577	-52.33799754	0.037587442	1.967251442	-5.709787
Vegfa	Vegfa-008	2488.5435	5.22318E-05	0.000496789	0.019188439	-52.11471239	0.398758066	20.78116193	-5.703619
Hira	Hira-002	507.95351	0.003071453	0.010948925	0.019278071	-51.87240938	0.139080832	7.214457867	-5.696895
Ccdc6	Ccdc6-003	783.20038	0.003979618	0.013368175	0.019313263	-51.77789064	0.185542667	9.607007918	-5.694264
Zfp446	Zfp446-006	359.97124	0.00441725	0.014515094	0.019331442	-51.72919758	0.108074412	5.590602594	-5.692907
Dlg4	Dlg4-006	520.04109	0.003384787	0.01179439	0.019385655	-51.5845352	0.133503125	6.886696648	-5.688867
Mast4	Mast4-013	796.41377	0.012065023	0.031943959	0.019389998	-51.57298208	0.245287084	12.65018637	-5.688544
Tbcel	Tbcel-005	1887.7953	0.001212722	0.005300627	0.019411483	-51.51589837	0.381889588	19.67338519	-5.686946
Ran	Ran-002	2272.1308	0.000265156	0.001644983	0.019492502	-51.30177842	0.771097444	39.55867019	-5.680937
Slbp	Slbp-002	1370.7082	0.007365288	0.021687377	0.019493162	-51.30004074	0.401342009	20.58886142	-5.680888
Clk1	Clk1-004	9376.4986	5.07311E-06	9.86679E-05	0.019549228	-51.15291424	2.560083648	130.9557393	-5.676745
Rpl7	Rpl7-006	2610.2399	0.005400284	0.017032852	0.019653366	-50.8818682	0.784111862	39.89707641	-5.66908
Rnf2	Rnf2-002	561.0311	0.001670891	0.006790224	0.019676651	-50.82165541	0.186363608	9.471307049	-5.667371
Cadps2	Cadps2-002	854.00543	0.000493886	0.002647612	0.019691001	-50.78462037	0.170829332	8.675502788	-5.66632
Slmap	Slmap-006	1857.0336	0.000944919	0.00436048	0.019737018	-50.66621563	0.506646997	25.66988602	-5.662952
Tgfb1i1	Tgfb1i1-010	113.00466	0.038817262	0.080186442	0.019782225	-50.55043199	0.025033702	1.265464472	-5.659652
Srek1ip1	Srek1ip1-001	825.66462	0.000798273	0.003822824	0.019814273	-50.46866996	0.189067017	9.541960886	-5.657316
Morf4l2	Morf4l2-204	459.64517	0.004226293	0.014023496	0.019829551	-50.42978543	0.145562468	7.340684034	-5.656204
Klhl13	Klhl13-004	128.63029	0.006286933	0.019119823	0.019847967	-50.38299267	0.034225033	1.724359569	-5.654865
Timp1	Timp1-001	1412.1296	0.025837073	0.057959114	0.019919932	-50.20097488	0.570424245	28.63585318	-5.649643
Rnf38	Rnf38-017	6354.8771	0.000410097	0.002292819	0.019952497	-50.11903942	1.637909518	82.09045169	-5.647287
Tmem248	Гmem248-201	3647.6579	0.000120118	0.000916522	0.019995029	-50.01243084	0.977801154	48.90221258	-5.644215
Fry	Fry-006	2713.0474	0.005339073	0.016872418	0.020020238	-49.94945643	0.641822038	32.05866191	-5.642397
Tpm1	Tpm1-017	1688.3113	0.000838136	0.003971138	0.020053619	-49.86630991	0.48614891	24.2424522	-5.639994
Ctnnd1	Ctnnd1-003	3503.5442	2.41663E-07	1.55824E-05	0.020093759	-49.76669707	0.840393044	41.82358602	-5.637109
Itgbl1	Itgbl1-003	4259.8952	0.001491904	0.006228389	0.020227668	-49.43723509	0.689389178	34.08149488	-5.627526
Raph1	Raph1-005	2260.0001	0.029683981	0.06476058	0.020232707	-49.4249245	0.740433125	36.5958513	-5.627167
Itga9	Itga9-002	10395.65	9.295E-06	0.00014691	0.020241809	-49.40269865	1.726117002	85.27483811	-5.626518
Rbms1	Rbms1-004	616.86636	0.003344031	0.011689565	0.020308465	-49.24055115	0.133328049	6.565146635	-5.621775
Pdrg1	Pdrg1-003	1127.2436	0.008406569	0.024016402	0.020314767	-49.22527559	0.322023426	15.85169189	-5.621327
Palb2	Palb2-004	154.0875	0.030553932	0.066320563	0.020506433	-48.76518501	0.034869012	1.700393831	-5.60778
Synj1	Synj1-011	2027.4353	0.008021368	0.023178254	0.020551028	-48.65936546	0.555686365	27.03934591	-5.604646
Ppt2	Ppt2-002	215.31973	0.019836102	0.047223123	0.020646928	-48.43335438	0.047130666	2.282696231	-5.597929
Caprin1	Caprin1-013	3113.5885	1.14468E-06	3.81412E-05	0.02064707	-48.4330223	0.973109658	47.13064176	-5.597919
Sell1	Sell1-201	9567.1319	5.68428E-07	2.48495E-05	0.020649278	-48.42784336	2.350564758	113.8327819	-5.597765
Tmem243	Гmem243-002	669.03019	0.006478563	0.019573994	0.020653516	-48.41790683	0.209296735	10.13370981	-5.597469
Ckap5	Ckap5-202	3231.5522	4.98993E-07	2.3248E-05	0.020657222	-48.4092187	0.920690457	44.56990571	-5.59721
Fry	Fry-009	1416.8988	0.015187353	0.038236317	0.020672288	-48.37393971	0.454042576	21.96382817	-5.596158
Gpc6	Gpc6-004	6745.9119	0.000305141	0.001832558	0.020694406	-48.32223724	1.393943232	67.35845554	-5.594615
Tnfrsf23	Tnfrsf23-001	2700.7142	0.000699072	0.003461502	0.020802854	-48.07032662	0.96410211	46.34470333	-5.587075
Malat1	Malat1-004	65449.526	7.31443E-08	7.97832E-06	0.020807577	-48.05941577	14.00165736	672.9114725	-5.586747
Gas5	Gas5-005	5011.123	1.11369E-05	0.000166713	0.020874604	-47.9051004	1.734956652	83.11327257	-5.582107
Klhl7	Klhl7-001	1024.2488	0.002982174	0.010703076	0.020899672	-47.84763977	0.286429452	13.70497324	-5.580376
Rsrc2	Rsrc2-010	643.24605	0.003573616	0.012304851	0.020952613	-47.72674352	0.147957948	7.061551013	-5.576726
Lrp5	Lrp5-004	5021.8386	0.000228267	0.001472621	0.021007439	-47.60218435	0.809947267	38.55525912	-5.572956
Zc3h14	Zc3h14-202	160.84594	0.002911946	0.010512716	0.021012102	-47.59162057	0.042003953	1.999036173	-5.572636
Grb10	Grb10-009	8488.1446	8.76753E-05	0.000723759	0.021024342	-47.56391465	2.151492613	102.333411	-5.571796

Arhgap24	Arhgap24-201	198.33455	0.000178656	0.001225757	0.0210385	-47.53190516	0.062087944	2.951158263	-5.570824
42622	Sept9-004	1344.9687	0.003010533	0.0107879	0.021216852	-47.13234599	0.361551252	17.0407587	-5.558646
Cd99l2	Cd99l2-002	617.68227	0.00429758	0.014209369	0.021218719	-47.12819902	0.165643418	7.806475971	-5.558519
Ctsa	Ctsa-001	336.08866	0.022759552	0.052517326	0.021282148	-46.98773827	0.082673207	3.884627027	-5.554212
Atp6v0b	Atp6v0b-002	1224.015	0.005739147	0.017812939	0.021295989	-46.95719853	0.346444599	16.26806783	-5.553274
Ambra1	Ambra1-005	1832.2741	0.004942073	0.015857786	0.02140076	-46.72731315	0.502145689	23.46391888	-5.546194
Lpp	Lpp-202	17686.112	5.24938E-05	0.000498728	0.021431135	-46.66108455	4.885992862	227.985726	-5.544148
Dcakd	Dcakd-002	362.28583	0.014170595	0.036252562	0.021441212	-46.63915429	0.123680936	5.768374272	-5.54347
Fxr1	Fxr1-007	2679.9406	0.010696718	0.029060252	0.021677968	-46.12978557	0.755500845	34.85109196	-5.527627
Cd200	Cd200-004	15796.859	2.01893E-08	3.56435E-06	0.021682673	-46.11977514	4.515516983	208.2546279	-5.527314
Oaz2	Oaz2-006	2226.9462	0.046173852	0.092105037	0.021911852	-45.63740279	0.641632215	29.28242785	-5.512145
Arhgap25	Arhgap25-004	1060.5374	0.033167232	0.070781837	0.021927878	-45.60404842	0.401363911	18.30381923	-5.51109
Psme1	Psme1-007	4068.4157	0.029623842	0.064649967	0.022053702	-45.34386142	1.075789086	48.78043123	-5.502835
Pafah1b1	Pafah1b1-006	5066.2736	4.16564E-05	0.000422313	0.022092404	-45.26442722	1.215450141	55.01665443	-5.500306
Dpm1	Dpm1-001	383.93303	6.4092E-06	0.000114929	0.022122928	-45.20197404	0.09836482	4.446284039	-5.498314
Ttc3	Ttc3-010	825.26381	0.001801341	0.007211443	0.022346441	-44.74985623	0.202438033	9.059072878	-5.483811
Slc17a5	Slc17a5-002	1047.2529	0.002040235	0.007953342	0.022416971	-44.60906034	0.352469949	15.72335322	-5.479265
Fam184b	Fam184b-002	192.59017	0.002177607	0.008363204	0.022525414	-44.39429964	0.039145851	1.737852631	-5.472303
Ccni	Ccni-004	2415.6713	1.32247E-05	0.000188258	0.022606993	-44.23410107	0.724097195	32.02978849	-5.467087
Atf2	Atf2-008	1318.7514	0.000266817	0.001652531	0.02260883	-44.23050642	0.30911313	13.67223028	-5.46697
33434E20R	3434E20Rik-	361.05103	0.000480258	0.002590368	0.022642109	-44.16549757	0.087653996	3.871282332	-5.464848
Gas5	Gas5-019	204.46376	0.004035788	0.013513124	0.02274185	-43.97179624	0.036530758	1.606323031	-5.458507
Mertk	Mertk-002	2912.7087	0.006915223	0.020618894	0.022863383	-43.73806014	0.655527665	28.67150844	-5.450817
Jade1	Jade1-001	3639.5914	7.84577E-09	2.31621E-06	0.022872197	-43.72120519	1.030502369	45.05480551	-5.450261
Ube2j2	Ube2j2-004	265.83492	0.000225138	0.001457103	0.022874569	-43.7166713	0.102791897	4.493719572	-5.450112
Dcun1d2	Dcun1d2-202	276.01356	0.005880976	0.018159342	0.022913992	-43.64145675	0.074217601	3.238964235	-5.447627
Ap2b1	Ap2b1-006	298.79104	0.001580543	0.00651192	0.023000494	-43.47732777	0.051014024	2.217953463	-5.442191
Atrx	Atrx-005	2908.7442	2.66197E-06	6.53097E-05	0.023092817	-43.30350932	0.927934915	40.18283823	-5.436412
Prpf8	Prpf8-004	2778.0784	0.000716845	0.003527577	0.023097342	-43.29502444	0.806327943	34.90998798	-5.436129
Stt3a	Stt3a-006	734.78679	0.005582625	0.017446797	0.023100032	-43.28998414	0.25064109	10.8502488	-5.435961
Npepps	Npepps-020	869.48338	0.003474307	0.012031851	0.02317268	-43.1542664	0.241792619	10.43438309	-5.431431
Aebp2	Aebp2-005	1720.2541	0.02424766	0.055152234	0.023189907	-43.12220858	0.499394982	21.53501458	-5.430359
Ccser2	Ccser2-002	620.43403	4.96205E-05	0.000478194	0.023291246	-42.93458533	0.177063897	7.602164987	-5.424068
Zscan21	Zscan21-004	70.303191	0.015521588	0.038894819	0.023331205	-42.86105279	0.012982578	0.556446943	-5.421595
Dab2ip	Dab2ip-005	1935.2158	4.28946E-05	0.000432164	0.02337172	-42.78675245	0.351200276	15.02671927	-5.419092
Cd59a	Cd59a-201	673.40946	0.000119263	0.000911775	0.023381536	-42.76879077	0.082070112	3.510039461	-5.418487
Slc25a3	Slc25a3-002	3801.5683	0.003616541	0.012411477	0.023382211	-42.76755511	1.182610206	50.57734717	-5.418445
Pou2f1	Pou2f1-008	451.67474	0.036296289	0.076094014	0.023642985	-42.29584422	0.147779417	6.250455218	-5.402444
Fxr1	Fxr1-008	403.61728	0.000105471	0.000828527	0.023644518	-42.29310083	0.137843481	5.82982824	-5.40235
Trip12	Trip12-009	2527.2149	9.9101E-06	0.000153584	0.024171306	-41.37136858	0.848674569	35.11082839	-5.370561
Slc43a1	Slc43a1-201	653.7576	0.023998332	0.054698954	0.024283163	-41.1807974	0.181501306	7.474368502	-5.3639
Phf21a	Phf21a-202	1425.4689	1.89225E-05	0.000242733	0.024326828	-41.10687992	0.460937296	18.94769408	-5.361308
Ncor1	Ncor1-003	3216.6801	7.01164E-07	2.81865E-05	0.024355157	-41.05906683	0.933541815	38.33035575	-5.359629
Sfxn3	Sfxn3-202	8491.9594	2.24189E-05	0.00027345	0.024465684	-40.87357636	1.722062448	70.38685097	-5.353097
Hnrnpll	Hnrnpll-201	297.86287	0.022621982	0.052270315	0.024478975	-40.8513842	0.094847281	3.874642706	-5.352313
Rcbtb2	Rcbtb2-202	526.54857	0.009169757	0.025757743	0.024491972	-40.82970462	0.209730766	8.563245221	-5.351547
Sema6d	Sema6d-001	2131.2998	9.66029E-05	0.000777685	0.024578134	-40.68657091	0.525774325	21.39195437	-5.346481
Nrbp1	Nrbp1-002	414.91576	0.000565302	0.002942047	0.024578194	-40.68647266	0.075153984	3.057750527	-5.346477
Ppp2r4	Ppp2r4-003	1156.8501	0.000323249	0.001912012	0.024797446	-40.32673284	0.340471454	13.73010138	-5.333665
Tnrc6c	Tnrc6c-201	2295.3763	0.000455596	0.002489891	0.024828471	-40.27634286	0.416670492	16.78196361	-5.331861
Ctage5	Ctage5-013	2343.3881	0.000726932	0.003561569	0.024847269	-40.24587203	0.864464542	34.79112933	-5.330769
Nabp1	Nabp1-001	259.3212	0.002073063	0.0080529	0.024947991	-40.08338733	0.138368982	5.546297485	-5.324933
Tbc1d1	Tbc1d1-007	314.92947	0.00722097	0.021351829	0.024968284	-40.05081049	0.096673387	3.871847486	-5.32376
Dhx35	Dhx35-001	288.26243	0.001459107	0.006128821	0.024996265	-40.00597628	0.098807644	3.952896262	-5.322144
Athl1	Athl1-001	226.00329	0.006034248	0.018537358	0.025032586	-39.94793055	0.074351718	2.970197284	-5.320049
Atg7	Atg7-006	160.44931	0.002555267	0.009480867	0.025095104	-39.84841031	0.060623253	2.415740277	-5.31645
Shoc2	Shoc2-202	9511.6321	1.30942E-05	0.000186554	0.025229725	-39.63578745	2.557533259	101.3698446	-5.308732
30216N24F	0216N24Rik-	141.54091	0.016509719	0.040800771	0.025297666	-39.52933805	0.04733357	1.871064685	-5.304852
Hnrnph2	Hnrnph2-003	2761.2463	0.000694519	0.003444789	0.025316477	-39.49996606	1.046665228	41.34324098	-5.30378
Rpl41	Rpl41-007	233.78639	0.001339212	0.005724199	0.025352791	-39.4433894	0.068553923	2.703999093	-5.301712
Lrch3	Lrch3-012	548.27199	0.000161055	0.001137668	0.025397299	-39.37426507	0.133160197	5.243084887	-5.299181
Pxylp1	Pxylp1-001	445.52283	0.001725499	0.006965827	0.025419912	-39.33923909	0.135157379	5.316988438	-5.297897
Exoc3l2	Exoc3l2-201	117.69317	0.012039642	0.031897077	0.025461647	-39.27475655	0.036605243	1.437662024	-5.29553
Asap1	Asap1-006	1508.8426	0.000268427	0.001660396	0.025467903	-39.26510893	0.694945473	27.28710972	-5.295176
Bend4	Bend4-004	1005.0316	4.23307E-05	0.000427894	0.025527294	-39.17375633	0.374860755	14.68470386	-5.291816
Cldn12	Cldn12-201	768.52376	0.007249104	0.021422071	0.025547936	-39.1421058	0.262506201	10.27504549	-5.290649
Gna11	Gna11-002	839.95323	1.02279E-05	0.000156947	0.025679446	-38.94165013	0.178768476	6.961539437	-5.283242
Gatad2b	Gatad2b-001	9978.4943	3.29938E-09	1.51021E-06	0.025763218	-38.8150275	3.237350234	125.6578384	-5.278543
Fn1	Fn1-012	11717.181	3.80432E-06	8.16753E-05	0.025776578	-38.79490865	4.764846355	184.8517791	-5.277795
Ddr1	Ddr1-001	39.918903	0.017785217	0.043266117	0.025781696	-38.78720761	0.016845688	0.653397203	-5.277509
Clpx	Clpx-201	361.38244	0.001885681	0.007470743	0.025786675	-38.77971902	0.133884222	5.191992504	-5.27723
Ptp4a2	Ptp4a2-201	31990.208	5.80613E-07	2.51721E-05	0.025823189	-38.72488469	9.253894627	358.3560024	-5.275189
Prpf38b	Prpf38b-002	2139.8391	0.019356213	0.046308346	0.025869507	-38.65554862	0.654458671	25.29845897	-5.272604
Rps14	Rps14-004	678.73484	0.000143831	0.001047683	0.025891996	-38.62197382	0.308628286	11.91983357	-5.27135
Prorsd1	Prorsd1-002	751.88571	0.007666072	0.022374347	0.026023165	-38.42730152	0.283816368	10.90629714	-5.26406
Mta1	Mta1-001	1384.6789	5.49067E-05	0.000515785	0.026027221	-38.42131255	0.471335656	18.10933457	-5.263835
Hnrnph1	Hnrnph1-201	9687.7168	1.91527E-05	0.000244586	0.026075377	-38.35035707	3.663931478	140.5130805	-5.261168
Ier3ip1	Ier3ip1-002	971.10577	0.049641408	0.09741138	0.026112061	-38.29647939	0.260528192	9.977312545	-5.25914
Gm26520	Gm26520-201	386	0.000143739	0.00104746	0.026123806	-38.27926118	0.102659512	3.929730292	-5.258491
Mob1a	Mob1a-004	4091.8006	0.00209802	0.008124964	0.026148852	-38.24259644	1.761821154	67.3766154	-5.257109
Lrrc57	Lrrc57-002	1458.6194	7.11321E-06	0.000122788	0.026239942	-38.10984038	0.334677676	12.75451282	-5.252092
10010F05R	0010F05Rik-	760.78256	0.00013783	0.001017387	0.026250335	-38.09475139	0.271640624	10.34808204	-5.25152
Nsmaf	Nsmaf-010	716.05702	0.000709673	0.003499828	0.026359388	-37.93714738	0.175198738	6.64654035	-5.245539
Preb	Preb-013	1017.9348	0.002049743	0.0079786	0.026379385	-37.90838962	0.292913253	11.10386971	-5.244445
Acsl3	Acsl3-001	596.19034	0.000906833	0.004225911	0.026419367	-37.85101979	0.154269352	5.839252303	-5.24226
Dnajb4	Dnajb4-001	573.20606	0.001445457	0.006083424	0.026534258	-37.68712896	0.26917932	10.14459575	-5.236
Taf13	Taf13-003	1695.3243	0.005654004	0.017612517	0.026564492	-37.64423541	0.698892249	26.30926436	-5.234357
Rnf31	Rnf31-004	1275.2686	0.006251625	0.019054111	0.026732285	-37.4079514	0.459341847	17.18303749	-5.225273
Ddx60	Ddx60-006	396.54636	0.028313086	0.062341575	0.026787894	-37.33029536	0.138731535	5.178889161	-5.222275
Mef2c	Mef2c-007	695.09116	0.002600457	0.009611055	0.026845359	-37.25038607	0.319299067	11.89401353	-5.219183
Lgals8	Lgals8-005	2295.4708	0.000314688	0.001873142	0.026902286	-37.17156288	0.780234046	29.00251892	-5.216127
Adss	Adss-003	1248.782	3.38455E-05	0.000364417	0.027026848	-37.00024556	0.37711354	13.95329359	-5.209463
Mapre2	Mapre2-006	7375.1791	5.31504E-07	2.40794E-05	0.027080958	-36.92631621	2.261331741	83.50265093	-5.206577
Tgif1	Tgif1-011	1053.8742	0.005051418	0.016129659	0.027081463	-36.92562737	0.505184624	18.6542592	-5.206551
Gcfc2	Gcfc2-001	384.16267	0.014076174	0.036064404	0.027145033	-36.8391517	0.150465744	5.543030364	-5.203168
Parvg	Parvg-201	222.87662	0.003733774	0.01272648	0.027161996	-36.81614505	0.087947062	3.237871802	-5.202267
Arpc1b	Arpc1b-009	3321.4441	1.79629E-08	3.32556E-06	0.027171822	-36.80283215	1.221262787	44.94592937	-5.201745
Itpr2	Itpr2-006	2552.236	3.35256E-06	7.55785E-05	0.027252122	-36.69438982	0.581722122	21.34593832	-5.197488
Bach2	Bach2-201	2941.6261	0.001635534	0.006680623	0.027353021	-36.55903365	0.989422415	36.17232738	-5.192156
Pxn	Pxn-004	4958.8657	1.57409E-06	4.61977E-05	0.027495239	-36.36993355	1.585878473	57.67829467	-5.184674
Prrc2c	Prrc2c-004	909.33405	2.56232E-06	6.36908E-05	0.027572657	-36.26781411	0.432957224	15.70241212	-5.180618
Zfp62	Zfp62-010	1687.7125	0.000698047	0.003458907	0.027625994	-36.19779237	0.565906124	20.48455237	-5.17783
Pex5	Pex5-003	69.10417	0.002317627	0.008795426	0.027638478	-36.18144216	0.034474022	1.247319832	-5.177178
Dock11	Dock11-004	605.07039	0.008421361	0.024047993	0.027669142	-36.14134511	0.269346503	9.734544901	-5.175578
Tbcb	Tbcb-002	616.95587	0.005198609	0.01650008	0.027684775	-36.1209366	0.2183847	7.888259892	-5.174763
Zfp119b	Zfp119b-002	178.16851	0.010643035	0.028955751	0.027734963	-36.05557414	0.063971547	2.306530844	-5.17215
Pink1	Pink1-003	4304.3771	0.042572945	0.086325461	0.027742971	-36.04516658	1.475524951	53.18554266	-5.171734

Gm26625	Gm26625-201	462.10322	0.000285162	0.001738484	0.027744188	-36.04358569	0.202926297	7.314191393	-5.171671
Ccdc125	Ccdc125-202	365.10251	0.002399728	0.009032223	0.02782951	-35.93308015	0.106141852	3.814003675	-5.167241
Mbd3	Mbd3-004	671.87878	0.017206641	0.042158056	0.027876397	-35.87264111	0.307758176	11.0400986	-5.164812
Akt3	Akt3-201	7529.4919	1.84535E-06	5.09293E-05	0.027927617	-35.80685035	2.147557916	76.89728492	-5.162164
Mettl21b	Mettl21b-001	6552	1.59788E-08	3.19391E-06	0.028008094	-35.70396459	2.056092518	73.41065446	-5.158012
Sntg2	Sntg2-002	166.7069	0.001071763	0.004804067	0.028052773	-35.64710032	0.064428844	2.296701463	-5.155713
Crl1	Crl1-010	699.46585	0.011862873	0.031530608	0.028085455	-35.60561851	0.192344496	6.848544765	-5.154033
Rpf2	Rpf2-002	575.3024	0.006726885	0.020164172	0.028100054	-35.58711966	0.277343375	9.869851856	-5.153283
Zfr	Zfr-004	1773.9436	4.27825E-06	8.81118E-05	0.028126903	-35.55314968	0.661285875	23.51079568	-5.151905
Gm13510	Gm13510-001	389	0.000292597	0.001773706	0.028155723	-35.51675855	0.146993201	5.220722046	-5.150428
Tmsb4x	Tmsb4x-002	28862.513	3.01984E-08	4.55917E-06	0.0281849	-35.47999045	12.87325271	456.7428834	-5.148934
Cnot3	Cnot3-004	1142.6245	5.2632E-05	0.000499533	0.028188314	-35.47569365	0.38047842	13.49773588	-5.148759
Pcnp	Pcnp-001	1226.6844	0.000438687	0.002417543	0.028384841	-35.23007206	0.478578738	16.86036342	-5.138736
Hs3st1	Hs3st1-001	1819.0131	0.001617443	0.006623317	0.028423563	-35.18207735	0.344133852	12.10734381	-5.136769
P24-309H3	24-309H3.3-(	869	1.79113E-06	4.98835E-05	0.028567836	-35.00440205	0.369902405	12.9482125	-5.129464
Calcrl	Calcrl-201	4802.8208	3.26525E-07	1.82142E-05	0.028624237	-34.93542951	0.967295371	33.79287924	-5.126619
Lancl2	Lancl2-003	2882.851	0.000395553	0.002231893	0.028673075	-34.87592407	0.820986496	28.63266269	-5.12416
Kif1b	Kif1b-011	777.36511	0.016630761	0.04104955	0.028676953	-34.87120872	0.421253908	14.68963294	-5.123964
Rbm5	Rbm5-027	2117.2639	0.000399933	0.002249212	0.028685459	-34.86086782	0.766593375	26.72411034	-5.123537
Gm26768	Gm26768-201	219.49504	2.71771E-05	0.000313512	0.028687053	-34.85893062	0.084472694	2.944627785	-5.123456
Celf1	Celf1-004	1288.9774	4.57957E-05	0.000452538	0.02869132	-34.85374644	0.418894677	14.60004884	-5.123242
Ttc3	Ttc3-008	284.26779	0.036164444	0.075851778	0.028860631	-34.64927727	0.084765998	2.937080567	-5.114753
Cbfb	Cbfb-002	255.46552	0.013458072	0.034798352	0.028902927	-34.59857249	0.056382282	1.95074647	-5.112641
Alg8	Alg8-001	1263.8725	3.03781E-05	0.000337809	0.028978785	-34.50800264	0.654710184	22.59274077	-5.108859
Srebf1	Srebf1-001	524.79528	0.010664883	0.028999066	0.029374658	-34.04295011	0.239037942	8.137556727	-5.089284
Cpeb2	Cpeb2-002	3887.0535	0.000136582	0.001010087	0.029396491	-34.01766567	1.474477982	50.15829903	-5.088212
Slfn4	Slfn4-001	1042.6053	0.022890175	0.052719285	0.029414086	-33.99731732	0.74293481	25.25779047	-5.087349
Gnb4	Gnb4-003	954.34124	0.006439301	0.01948459	0.029492308	-33.90714598	0.368502518	12.49486868	-5.083517
Tango2	Tango2-002	214.94942	0.008175726	0.023493465	0.029533633	-33.85970164	0.050314982	1.703650265	-5.081497
Psma3	Psma3-007	4040.798	0.007426003	0.021833685	0.029548861	-33.84225155	1.719692248	58.19825766	-5.080754
Papolg	Papolg-002	1291.6354	4.33569E-06	8.87837E-05	0.029628406	-33.75139328	0.440959952	14.88301277	-5.076875
Atp8a1	Atp8a1-002	1899.9664	0.003331473	0.011659813	0.029642346	-33.73552139	0.852507143	28.75977294	-5.076197
Tyw5	Tyw5-011	478.44719	0.006353001	0.019267714	0.02968253	-33.68985047	0.190049763	6.402748098	-5.074242
Gm26735	Gm26735-201	2369.2153	0.030403348	0.066056501	0.029684236	-33.68791487	1.615324818	54.41692495	-5.074159
Fam111a	Fam111a-001	269.58886	0.014931688	0.037755317	0.029759595	-33.60260757	0.144979563	4.871691348	-5.070501
Stau2	Stau2-007	323.21447	0.008140583	0.023420159	0.029807118	-33.54903294	0.10587383	3.551964615	-5.068199
Ermp1	Ermp1-002	5431.9442	0.003239021	0.011407871	0.029813275	-33.5421055	1.879706869	63.04932612	-5.067901
Ramp2	Ramp2-007	15011.87	5.32815E-09	1.88133E-06	0.02998062	-33.35488013	2.376497533	79.26779035	-5.059826
Plec	Plec-010	138.82369	0.007271381	0.02146753	0.030098358	-33.22440349	0.01044692	0.347092689	-5.054171
Arhgap12	Arhgap12-008	2348.3465	5.75282E-07	2.50361E-05	0.030106408	-33.21552042	0.918965184	30.52390682	-5.053786
Arhgdib	Arhgdib-003	5343.6979	0.000334596	0.001963324	0.030151665	-33.16566471	2.238103552	74.22819198	-5.051619
Hsdl2	Hsdl2-002	669.60451	0.003436097	0.011926056	0.030446461	-32.84453959	0.219241949	7.200900863	-5.037582
St3gal6	St3gal6-014	518.09353	0.00115851	0.005113267	0.030563358	-32.71891766	0.149236846	4.88286808	-5.032053
Tagln2	Tagln2-003	10029.549	0.00021877	0.001427216	0.030583797	-32.69705188	4.510572611	147.4824267	-5.031089
Phf21a	Phf21a-205	140.99428	0.003728532	0.012716951	0.030636191	-32.64113351	0.057362933	1.872391159	-5.028619
Caml	Caml-002	667.44269	0.003907798	0.013181219	0.030685451	-32.58873367	0.270469525	8.81425933	-5.026301
Tmbim4	Tmbim4-009	1453.0224	0.003218082	0.011354705	0.030763066	-32.50651308	0.520917527	16.9332124	-5.022657
Setd8	Setd8-010	3343.7721	5.8985E-05	0.000543805	0.030763853	-32.50568177	1.39223854	45.25566294	-5.02262
Matr3	Matr3-002	3146.0881	2.32649E-05	0.000280868	0.030884283	-32.37892886	1.323060577	42.83928429	-5.016983
Zkscan6	Zkscan6-001	494.17264	0.015700648	0.039231574	0.030884946	-32.37823356	0.206488315	6.685726881	-5.016952
Mrps17	Mrps17-006	150.05372	0.019908927	0.047359406	0.031039887	-32.21661209	0.027244557	0.877727332	-5.009733
Btf3	Btf3-005	661.69051	1.56822E-06	4.60648E-05	0.031122206	-32.13139836	0.257356324	8.26921858	-5.005912
Kdm5d	Kdm5d-006	360.76009	0.022004431	0.051168147	0.031151781	-32.10089371	0.090103494	2.892402684	-5.004542
Malsu1	Malsu1-003	685.48996	0.006734418	0.020182406	0.031167227	-32.08498438	0.314044618	10.07611667	-5.003826
Acox1	Acox1-004	4701.6011	0.001364087	0.005811664	0.031169042	-32.08311672	1.908713247	61.2374699	-5.003742
30015E06R	0015E06Rik-	355.71379	0.001780495	0.007148276	0.031202828	-32.04837763	0.215899385	6.919225033	-5.002179
Fam206a	Fam206a-002	1283.7099	2.20772E-05	0.000270825	0.031239005	-32.01126301	0.453731355	14.52451375	-5.000508
Tial1	Tial1-005	1767.1665	0.001214434	0.005306759	0.031248149	-32.00189544	0.832118168	26.62935862	-5.000085
Raet1d	Raet1d-001	311.4124	0.039840598	0.081831514	0.031261988	-31.98772927	0.151787948	4.855351773	-4.999447
Rhot2	Rhot2-003	315.80208	0.043182963	0.087309119	0.031340344	-31.9077546	0.076225642	2.432189094	-4.995835
Cul2	Cul2-002	584.31516	0.005269191	0.016693165	0.031393767	-31.85345712	0.244250826	7.780233207	-4.993378
Fam134a	Fam134a-001	270.47546	0.001246801	0.005421761	0.031395915	-31.85127718	0.092937533	2.960179111	-4.993279
Kif14	Kif14-201	361.60503	0.019979087	0.047476842	0.031491332	-31.75476984	0.142072289	4.511472842	-4.988901
Hps3	Hps3-003	788.5968	0.0064213	0.019440423	0.031522491	-31.72338096	0.31412035	9.964959524	-4.987475
Selp	Selp-003	14729.474	1.4585E-08	3.14941E-06	0.031540204	-31.7055652	7.084607219	224.6214761	-4.986664
Cd44	Cd44-201	3652.6812	1.8528E-05	0.000238655	0.031551425	-31.69428916	2.021466555	64.06894553	-4.986151
Ptprc	Ptprc-008	2205.8258	0.000719848	0.003535244	0.031602021	-31.64354581	1.666065401	52.72021683	-4.983839
Pitpna	Pitpna-202	850.04968	0.003433315	0.011920023	0.031612955	-31.63260119	0.323202218	10.22372686	-4.98334
Comt	Comt-201	32127.389	1.64944E-07	1.26693E-05	0.031625817	-31.61973621	13.25853327	419.2313244	-4.982753
Dock10	Dock10-001	1294.829	0.009535646	0.026551207	0.031632171	-31.61338538	0.99871054	31.57262117	-4.982464
Kif1b	Kif1b-009	1086.1594	0.019908469	0.047359406	0.031917637	-31.33064014	0.374595753	11.73632473	-4.969502
Fam214b	Fam214b-001	573.55566	0.000766961	0.003706718	0.031943217	-31.30555065	0.166690918	5.218350969	-4.968347
Mospd2	Mospd2-001	1102.655	0.005229629	0.016587773	0.031992961	-31.2568752	0.489747819	15.30798647	-4.966102
Tatdn3	Tatdn3-009	815.8231	1.14101E-05	0.000169542	0.032003953	-31.24614052	0.389847171	12.1812195	-4.965606
Lztfl1	Lztfl1-004	282.37752	0.023919168	0.054579048	0.032024233	-31.22635242	0.077469647	2.419094493	-4.964692
Mios	Mios-003	204.87918	0.000197791	0.00132131	0.032074239	-31.17766857	0.108618953	3.386485709	-4.962441
Asb7	Asb7-002	2412.4726	0.016794244	0.041384409	0.032090392	-31.16197503	0.841127622	26.21119795	-4.961715
Stradb	Stradb-002	305.36637	0.000100252	0.000798674	0.03209602	-31.15651124	0.105106884	3.274763807	-4.961462
Bsg	Bsg-006	7663.7303	2.11823E-05	0.000263433	0.032142816	-31.11115049	2.470685734	76.86587568	-4.95936
Gm10012	Gm10012-002	1379.9959	1.99049E-07	1.41479E-05	0.032442478	-30.82378612	0.613826663	18.92046178	-4.945972
Fgfr1op	Fgfr1op-001	264.83946	0.006449771	0.019507651	0.032468286	-30.79928515	0.162527586	5.005733461	-4.944825
Ube2d2a	Ube2d2a-201	890.86115	7.18192E-07	2.86691E-05	0.032518221	-30.75198963	0.337286437	10.372229	-4.942608
Srrt	Srrt-020	1819.7018	0.021282611	0.049865196	0.032613471	-30.66217669	0.849987049	26.06245308	-4.938388
Phf21a	Phf21a-001	2892.8274	2.05237E-05	0.000257391	0.032728186	-30.55470256	1.098141019	33.55337221	-4.933323
Plod2	Plod2-003	1963.8468	2.56842E-07	1.60262E-05	0.032783127	-30.50349656	1.044745509	31.86839105	-4.930903
Ubxn2a	Ubxn2a-002	2569.4607	7.67455E-06	0.000129394	0.032795782	-30.49172597	1.105123649	33.69712746	-4.930346
Rad18	Rad18-003	515.88616	0.001688464	0.006843774	0.032814834	-30.47402255	0.275330471	8.390426968	-4.929508
H2-T22	H2-T22-003	430.76583	0.048585268	0.0959104	0.032854527	-30.43720582	0.062807648	1.911689308	-4.927764
Agps	Agps-002	944.36199	0.009640767	0.026777737	0.032905163	-30.39036743	0.474436384	14.41829603	-4.925542
Mapk14	Mapk14-005	10872.114	5.58929E-07	2.46375E-05	0.032920759	-30.37597069	3.145695853	95.55356503	-4.924859
Epc2	Epc2-002	1000.9778	0.034047624	0.072327179	0.032977268	-30.32391919	0.367708018	11.15034824	-4.922384
Rasgrp3	Rasgrp3-202	738.11377	0.010854169	0.029385447	0.033125534	-30.18819221	0.499696689	15.08493969	-4.915912
Gm28875	Gm28875-001	2030	6.21366E-07	2.61447E-05	0.033127566	-30.18634077	0.717599009	21.66168823	-4.915824
Pja2	Pja2-201	3434.3408	7.84086E-06	0.000131192	0.033140286	-30.17475476	1.246507701	37.61306419	-4.91527
Acadsb	Acadsb-002	2270.9092	0.001403496	0.005944495	0.033247406	-30.07753462	0.970158752	29.17998345	-4.910614
Hsp90b1	Hsp90b1-005	782.54198	0.000282694	0.001727434	0.033319344	-30.01259599	0.312237937	9.371071053	-4.907496
Cep170	Cep170-005	4308.6246	7.06309E-07	2.82937E-05	0.033449561	-29.89575898	2.203947519	65.88868384	-4.901869
Zfp229	Zfp229-001	661.47372	3.22202E-06	7.39401E-05	0.033484819	-29.86427983	0.321192638	9.59218683	-4.900349
Myo18a	Myo18a-017	2523.1166	0.000987318	0.004504546	0.03351507	-29.8373239	0.858434608	25.61339145	-4.899046
Arhgap11a	rhgap11a-00	1791.5683	0.005323251	0.016834844	0.033553085	-29.80351871	1.254434891	37.38657375	-4.897411
Lztfl1	Lztfl1-007	226.14657	0.001528372	0.00634219	0.033574589	-29.78443037	0.06365518	1.895933277	-4.896486
Idh1	Idh1-201	604.29776	0.006073991	0.018646068	0.033580331	-29.77933733	0.219568079	6.538591899	-4.89624
Map4k5	Map4k5-002	2733.9972	0.000969297	0.004438293	0.033597543	-29.76408154	1.132952031	33.72127663	-4.8955
00002E11R	0002E11Rik-	4822.9445	9.41115E-08	9.20948E-06	0.033598925	-29.76285696	1.557224953	46.34746354	-4.895441
Ptprg	Ptprg-004	1162.1586	0.003432374	0.011918261	0.033599514	-29.76233488	0.284392099	8.464172884	-4.895416
Morf4l2	Morf4l2-001	1804.1823	0.001255546	0.005449144	0.033660906	-29.70805333	0.845473801	25.11738077	-4.892782
Synj2	Synj2-007	84.678838	0.014258509	0.036419861	0.033683184	-29.688405	0.050652528	1.50379278	-4.891828

Eya1	Eya1-201	824.23409	0.018352416	0.044368622	0.033698247	-29.67513404	0.478214664	14.19108426	-4.891183
Myo1h	Myo1h-002	404.10919	0.044122818	0.088821355	0.033740826	-29.63768596	0.169905147	5.035595382	-4.889361
Cdca8	Cdca8-003	243.88859	0.000465174	0.002529727	0.03379846	-29.58714686	0.10385595	3.072801256	-4.886899
Gnpda2	Gnpda2-006	174.06185	0.005972734	0.018378055	0.033806404	-29.5801942	0.081152068	2.400493933	-4.88656
Phldb2	Phldb2-006	1727.5359	0.034246964	0.072678514	0.033878566	-29.51718809	0.755431772	22.2982217	-4.883483
Smim1	Smim1-014	114.38776	0.007886993	0.022882634	0.033913456	-29.48682098	0.051050892	1.505328513	-4.881998
M6pr	M6pr-002	2208.8973	4.09229E-05	0.000417021	0.034007415	-29.40535149	1.067303176	31.38442504	-4.878007
Ap1g2	Ap1g2-201	109.09805	0.023255211	0.053416336	0.034023791	-29.39119898	0.023057564	0.677689448	-4.877312
Zfp740	Zfp740-001	1476.4453	0.00014729	0.001065628	0.034086158	-29.33742168	0.619911992	18.18661953	-4.87467
Lmo7	Lmo7-001	4549.0381	0.001783494	0.007159479	0.034133583	-29.29666035	1.474030881	43.18418208	-4.872664
Morf4l1	Morf4l1-014	816.23323	0.025436547	0.057260136	0.034162197	-29.272122	0.42424217	12.41846857	-4.871455
Adam12	Adam12-001	3643.6647	0.001478627	0.006189979	0.034174978	-29.26117437	2.970694025	86.92599585	-4.870916
Paics	Paics-007	1336.0281	9.49658E-05	0.000769169	0.034263732	-29.18537873	0.331579343	9.677268711	-4.867174
Suv420h1	Suv420h1-01	370.7267	0.00039312	0.002222208	0.034300052	-29.15447467	0.151749351	4.4241726	-4.865645
Rnf214	Rnf214-004	364.8384	0.020012549	0.047529978	0.034310406	-29.14567669	0.174292812	5.079881953	-4.86521
Abcg2	Abcg2-006	352.38133	0.000224622	0.001454311	0.034690317	-28.82648758	0.143493435	4.136411717	-4.849323
Stam	Stam-001	2261.0159	0.000784617	0.003770608	0.034695672	-28.82203907	0.879884994	25.36007966	-4.849101
Agtpbp1	Agtpbp1-013	1890.6375	0.00087743	0.004120094	0.034724687	-28.79795554	0.657040282	18.92141682	-4.847894
Agpat1	Agpat1-002	1193.0382	0.000982397	0.004486877	0.034780808	-28.75148807	0.498226603	14.32475623	-4.845565
Nbr1	Nbr1-015	643.91173	0.002093098	0.008110487	0.034800964	-28.73483584	0.236594395	6.798501092	-4.844729
Tcf19	Tcf19-002	69.614654	0.012916587	0.033680481	0.034817741	-28.72098993	0.028211456	0.810260955	-4.844034
Preb	Preb-011	1452.2413	0.013365698	0.034611762	0.03483009	-28.71080764	0.583587642	16.75527253	-4.843522
Ddr1	Ddr1-201	79.60454	0.007433972	0.021845863	0.034846675	-28.69714261	0.039996609	1.147788383	-4.842835
Fas	Fas-202	721.42062	2.72157E-06	6.61091E-05	0.034859078	-28.68693174	0.286995991	8.233034404	-4.842322
Zfp207	Zfp207-201	1051.4412	0.001071076	0.004801617	0.034871706	-28.67654344	0.348258672	9.986854935	-4.841799
Spata6	Spata6-005	2076.5087	0.002981696	0.010703076	0.034882865	-28.66737034	0.839763703	24.07381708	-4.841338
Itgav	Itgav-002	965.88242	3.27484E-05	0.000356173	0.034884236	-28.66624331	0.474737877	13.60895148	-4.841281
Ism1	Ism1-201	232.67734	0.007502059	0.022009904	0.034946504	-28.61516571	0.102143893	2.922864425	-4.838708
Pspc1	Pspc1-004	791.27518	0.000165973	0.001163677	0.034953636	-28.60932656	0.379785962	10.86542062	-4.838414
F13a1	F13a1-202	675.96584	0.012335959	0.032484269	0.035014473	-28.55961863	0.471384116	13.46255057	-4.835905
Rpl31	Rpl31-001	1728.5787	4.87113E-05	0.000472065	0.035024533	-28.55141604	1.029620298	29.3971175	-4.83549
Papola	Papola-019	3345.4778	0.034068681	0.072358447	0.035038462	-28.54006587	1.6386074	46.76596312	-4.834917
Srpk1	Srpk1-001	62871.431	3.90464E-12	2.2289E-08	0.035106747	-28.48455316	29.00975355	826.3298673	-4.832108
Gclm	Gclm-002	1065.8851	0.000429842	0.00237903	0.035123286	-28.47113987	0.417403632	11.88395718	-4.831428
Prkd3	Prkd3-001	5363.0248	1.25497E-06	4.02278E-05	0.035131909	-28.46415219	2.385410306	67.89868199	-4.831074
Strbp	Strbp-005	416.30896	0.020108061	0.047703892	0.035158955	-28.44225602	0.177660472	5.053064622	-4.829964
Bid	Bid-004	278.10575	0.007713704	0.022465507	0.035167711	-28.43517461	0.150446354	4.277968347	-4.829605
Ppp4r1	Ppp4r1-004	1055.5231	0.019018132	0.045668584	0.035187563	-28.41913207	0.417926995	11.87712246	-4.828791
P24-295P14	24-295P14.2-	1504	3.99451E-07	2.03932E-05	0.035220268	-28.3927422	0.561062264	15.93009621	-4.82745
Ndufb5	Ndufb5-007	1209.8702	0.007757275	0.022569375	0.035299709	-28.32884583	0.591584634	16.7589099	-4.8242
P23-306G1.	23-306G1.11-	459	0.000860766	0.004054632	0.035304283	-28.32517546	0.179201499	5.075913901	-4.824013
Pacrgl	Pacrgl-003	167.5373	0.004785867	0.015447737	0.035354042	-28.28530861	0.065635825	1.856529568	-4.821981
Slk	Slk-004	568.96501	2.2723E-06	5.91386E-05	0.035377483	-28.2665673	0.232446997	6.570478692	-4.821025
Pogz	Pogz-002	4645.9436	0.002932742	0.010567745	0.03548631	-28.17988133	2.349938756	66.22099527	-4.816594
Nt5c3	Nt5c3-003	2508.2342	0.007552468	0.022123847	0.035500934	-28.16827273	1.012633807	28.52414525	-4.815999
Cd302	Cd302-002	228.98928	0.017730415	0.043184946	0.035533777	-28.14223789	0.034702227	0.976598315	-4.814665
Nxf1	Nxf1-201	345.6584	0.006843227	0.020448486	0.035542298	-28.13549075	0.141211035	3.973041779	-4.814319
Commd3	Commd3-006	480.93254	0.000668178	0.003341172	0.035573453	-28.1108498	0.230740737	6.48631821	-4.813055
Chd2	Chd2-008	725.47599	0.014170782	0.036252562	0.035597931	-28.0915204	0.295681226	8.306135178	-4.812063
Gm12942	Gm12942-002	684.28369	0.047391703	0.094031156	0.035657133	-28.04487941	0.330985802	9.282456907	-4.809665
Nup107	Nup107-202	1017.0406	0.00063136	0.003200722	0.035751178	-27.97110631	0.583555471	16.32269212	-4.805865
Lbr	Lbr-002	1880.3575	1.3523E-07	1.12653E-05	0.035779705	-27.94880506	0.879211621	24.57291419	-4.804715
P24-478I22	24-478I22.8-	156	4.53417E-05	0.00044935	0.035793043	-27.93838986	0.082576606	2.307057425	-4.804177
Malat1	Malat1-003	27558.801	1.48417E-07	1.19607E-05	0.0357953	-27.93662886	10.0858536	281.7647488	-4.804086
Clip1	Clip1-007	5440.4155	0.000903167	0.004216092	0.035839281	-27.9023453	1.853538403	51.71806855	-4.802314
Nt5c2	Nt5c2-201	2739.7998	1.99675E-06	5.39767E-05	0.035892141	-27.86125277	1.13689064	31.67519749	-4.800188
Bend4	Bend4-002	850.19466	1.84307E-05	0.000237849	0.035920481	-27.83927122	0.381494388	10.62052574	-4.79905
Slc31a1	Slc31a1-002	829.47642	0.011753817	0.031308776	0.035972349	-27.79912983	0.377989043	10.50776648	-4.796968
Cog6	Cog6-001	1226.4411	0.000385179	0.002187426	0.036015496	-27.76582591	0.538913696	14.96338387	-4.795238
Surf1	Surf1-002	247.86049	0.002962786	0.010648548	0.036123855	-27.68253812	0.090621548	2.508634469	-4.790904
Zfp27	Zfp27-001	446.58617	0.014081312	0.03607487	0.036145904	-27.66565215	0.131810329	3.646618723	-4.790024
Ewsr1	Ewsr1-002	4382.6677	6.78487E-06	0.000119106	0.036257065	-27.58083121	2.147244813	59.22279677	-4.785594
Ergic3	Ergic3-008	901.34604	0.039397457	0.081101268	0.036351708	-27.50902356	0.382809824	10.53072446	-4.781833
Hnrnph3	Hnrnph3-006	2403.2662	1.09386E-06	3.70571E-05	0.036453292	-27.43236503	1.115225949	30.59328532	-4.777807
Zfp62	Zfp62-201	918.39146	0.000119758	0.00091511	0.036458403	-27.4285191	0.507071693	13.90822562	-4.777605
Platr14	Platr14-006	245.55235	0.000263532	0.001637812	0.036465236	-27.42337946	0.118817063	3.258365411	-4.777334
Cfh	Cfh-005	55623.918	0.000147361	0.001065916	0.036551419	-27.35871906	32.75763187	896.2068475	-4.773929
Nat6	Nat6-006	1879.7381	0.003935014	0.01325014	0.036625047	-27.30371889	0.364335163	9.947704875	-4.771026
Fam172a	Fam172a-202	412.62184	0.007000444	0.020822994	0.036654489	-27.28178833	0.212920324	5.808847215	-4.769866
Rhbdd1	Rhbdd1-002	695.84175	0.0015631	0.006456364	0.0366683	-27.27151257	0.305746111	8.338158909	-4.769323
Stk17b	Stk17b-001	2752.8319	0.000123528	0.000937267	0.036695339	-27.2514176	2.336361896	63.66917369	-4.768259
Slc25a3	Slc25a3-008	2321.4655	0.019927356	0.047380212	0.036701803	-27.24661766	1.283635394	34.97472281	-4.768005
Cald1	Cald1-001	8839.9677	3.38779E-07	1.85948E-05	0.03676018	-27.20334881	4.884686718	132.8798366	-4.765712
Xpo1	Xpo1-202	2256.3604	0.001555396	0.006428418	0.036846134	-27.13988946	0.820017552	22.25518571	-4.762343
Asph	Asph-013	3690.3884	0.000663976	0.003326191	0.036908433	-27.09407935	1.709311831	46.31223039	-4.759906
Mtdh	Mtdh-006	4049.8733	6.11787E-06	0.000111219	0.036927872	-27.07981639	2.181614524	59.07772074	-4.759146
Brwd1	Brwd1-016	411.46634	0.013957051	0.035804733	0.036959394	-27.05672063	0.211683646	5.727465281	-4.757915
Maf1	Maf1-004	685.4412	0.000302551	0.001821292	0.037009905	-27.01979364	0.235375241	6.359790444	-4.755945
Srsf5	Srsf5-004	5347.6625	0.000234545	0.001502369	0.037023357	-27.00997638	2.815333189	76.04208293	-4.75542
Cmtr1	Cmtr1-002	1715.7274	0.019023846	0.045673251	0.037211892	-26.87312996	0.757483568	20.35595437	-4.748092
Scml2	Scml2-002	79.280194	0.005868328	0.01812519	0.037212165	-26.87293279	0.020746951	0.557531414	-4.748082
Agrn	Agrn-002	3518.9816	2.16126E-07	1.48423E-05	0.037288477	-26.8179362	0.996948887	26.73611164	-4.745126
Hspa8	Hspa8-007	1320.5201	0.000355077	0.002053155	0.037298926	-26.81042333	0.979719471	26.26669377	-4.744722
Sqstm1	Sqstm1-005	2691.2894	4.8149E-06	9.53238E-05	0.037404795	-26.73454056	1.319100111	35.26553543	-4.740633
Slc25a51	Slc25a51-001	3032.5123	1.2786E-05	0.000183461	0.037410365	-26.73055949	1.346945835	36.00461578	-4.740418
Nrg4	Nrg4-006	517.30191	1.76319E-05	0.000230846	0.037515297	-26.65579311	0.257455452	6.862679259	-4.736377
Raph1	Raph1-002	507.68735	0.001086077	0.004855501	0.037542686	-26.63634645	0.268913833	7.162882008	-4.735324
Mfap5	Mfap5-005	1143.7471	0.008623601	0.0245009	0.037577198	-26.61188281	0.344252424	9.161205173	-4.733999
Ubtf	Ubtf-006	603.74568	0.000775613	0.003738473	0.037587107	-26.60486772	0.321820967	8.562004251	-4.733618
Cep135	Cep135-201	1040.367	0.00097986	0.004479471	0.037614225	-26.58568689	0.487558437	12.96207595	-4.732578
Mrps33	Mrps33-002	1028.2936	1.6598E-07	1.26693E-05	0.037815356	-26.44428371	0.519274543	13.73184333	-4.724884
Larp4	Larp4-201	3810.7337	1.27794E-07	1.09423E-05	0.0378204	-26.44075693	2.027326786	53.60405477	-4.724692
Map4k4	Map4k4-201	3962.6726	0.000468205	0.002542976	0.037845834	-26.42298774	2.660993493	70.31139843	-4.723722
Cdk8	Cdk8-001	1184.7401	0.000806272	0.003855114	0.037849991	-26.42008533	0.61262721	16.18566317	-4.723563
Prr5l	Prr5l-201	1223.4967	0.000216135	0.001413256	0.037962897	-26.34150903	0.645531712	17.00427941	-4.719266
Ssbp1	Ssbp1-007	1335.8364	1.1335E-05	0.000168793	0.038014185	-26.30596982	0.646790708	17.01445683	-4.717318
Asph	Asph-003	5007.1994	1.16279E-07	1.04255E-05	0.038022504	-26.30021409	2.193509257	57.68976306	-4.717003
Lig3	Lig3-001	111.98668	0.002326365	0.008819793	0.038050948	-26.28055385	0.027090417	0.711951159	-4.715924
Map3k7	Map3k7-004	1365.1672	0.000133364	0.000991688	0.038177175	-26.19366143	0.741950201	19.43439236	-4.711146
Casp8	Casp8-002	2872.1247	0.000140713	0.001033326	0.038300422	-26.10937268	1.743914073	45.53250246	-4.706496
Svil	Svil-012	754.36008	0.027309958	0.060627823	0.038358507	-26.06983637	0.26990894	7.036481891	-4.70431
Zmynd11	Zmynd11-001	728.38476	0.002765414	0.010077181	0.038424201	-26.02526447	0.391106555	10.17865153	-4.701841
Kdm4c	Kdm4c-006	1830.6077	0.000641286	0.003239055	0.038500316	-25.97381262	0.659240625	17.12299247	-4.698986
Sdf2	Sdf2-002	2132.0449	0.007692929	0.022429797	0.038515276	-25.96372408	0.955216895	24.80098789	-4.698425
030018L15R	0018L15Rik-	346.08979	1.3776E-05	0.000193451	0.038531121	-25.95304696	0.165419977	4.293152438	-4.697832
Ints3	Ints3-002	547.58208	0.008149516	0.023439782	0.038634835	-25.88337671	0.169822687	4.395584581	-4.693954
Ahcyl1	Ahcyl1-003	4824.4328	1.88273E-05	0.000241602	0.038640591	-25.87952166	2.547885109	65.93804787	-4.693739

)30018L15R	0018L15Rik-	35.910332	0.008286306	0.023754344	0.038724358	-25.82353985	0.017253581	0.445548527	-4.690615
Tmem59	Tmem59-002	9260.4663	2.22247E-08	3.78705E-06	0.039265545	-25.46762043	3.877042203	98.7390392	-4.670592
Calm1	Calm1-002	1621.3216	0.020197759	0.047852789	0.039326932	-25.42786682	0.638841338	16.24437246	-4.668339
Atp11c	Atp11c-001	525.94699	0.00020345	0.0013499	0.039401588	-25.37968774	0.170247991	4.320840852	-4.665602
Mapk6	Mapk6-201	3581.2215	0.000296993	0.001795274	0.03941308	-25.3722874	2.293975558	58.20340714	-4.665182
Whsc1	Whsc1-007	1381.7858	0.003625255	0.012430171	0.039420983	-25.36720108	0.784031652	19.88868858	-4.664892
Gnb2	Gnb2-015	1068.0548	0.001606066	0.006591477	0.039564686	-25.27506496	0.534842925	13.51818968	-4.659643
Cep112os1	Cep112os1-00	99	0.003704048	0.012654727	0.039606049	-25.24866845	0.066472458	1.678341047	-4.658135
Zkscan1	Zkscan1-201	5855.2181	0.000171973	0.001192233	0.039644026	-25.22448131	2.71787672	68.55703053	-4.656753
Arl8a	Arl8a-003	1776.1329	0.019431329	0.046435842	0.039656417	-25.21659973	0.898610562	22.65990284	-4.656302
Cd53	Cd53-001	2792	0.000765327	0.00370128	0.03969551	-25.19176586	2.617501428	65.93948313	-4.65488
Rps27a	Rps27a-002	1547.2618	8.57106E-06	0.000139325	0.039713491	-25.18036005	0.811552143	20.43517517	-4.654227
Ppp1r16b	Ppp1r16b-004	7757.1702	0.000158864	0.001126987	0.039753673	-25.15490863	2.730309266	68.68068012	-4.652768
Cacul1	Cacul1-202	725.29204	0.005432851	0.017104067	0.039773457	-25.14239575	0.3409872	8.573235131	-4.65205
Maf1	Maf1-003	137.61707	0.033888579	0.072056359	0.039811657	-25.1182715	0.038139041	0.957986777	-4.650665
Ola1	Ola1-001	1037.517	0.002307908	0.008763859	0.039882664	-25.07355095	0.474838783	11.90589442	-4.648094
Nudcd3	Nudcd3-003	3691.0374	3.20825E-05	0.000351287	0.0399425	-25.03598917	1.863098039	46.64450233	-4.645932
Abhd5	Abhd5-003	2165.6896	0.00146926	0.00615863	0.039974756	-25.01578752	0.886029688	22.1647304	-4.644767
Xiap	Xiap-005	10601.54	7.4355E-05	0.000646282	0.04007378	-24.95397224	5.034300597	125.6257973	-4.641198
Wdr37	Wdr37-201	1441.8131	0.001264519	0.005476664	0.040156921	-24.90230739	0.724276676	18.03616041	-4.638208
Nabp1	Nabp1-006	618.25224	0.01605019	0.039892527	0.040189712	-24.88198952	0.488275268	12.1492601	-4.63703
Rlf	Rlf-201	1045.6512	0.003779634	0.012841231	0.040263135	-24.8366157	0.680369438	16.89807427	-4.634397
Eif4enif1	Eif4enif1-009	692.46478	6.4613E-05	0.000582061	0.040320422	-24.80132769	0.364624432	9.043170016	-4.632345
Eftud1	Eftud1-002	348.56254	0.005587832	0.017457196	0.040325302	-24.79832633	0.165035802	4.092611668	-4.632171
Dnaja1	Dnaja1-008	2855.2029	0.015000434	0.037874298	0.040353001	-24.78130446	1.61945069	40.1321006	-4.63118
Reps1	Reps1-002	860.12299	0.00016736	0.001170506	0.040564035	-24.65238005	0.386151159	9.519545138	-4.623655
Dyrk1a	Dyrk1a-201	3202.9729	0.048184083	0.095299278	0.04063066	-24.61195567	1.734408459	42.68718411	-4.621287
Tmcc1	Tmcc1-008	929.48906	0.008064068	0.023270227	0.040644485	-24.60358373	0.605728278	14.90308642	-4.620797
Rpl22l1	Rpl22l1-009	1369.0348	3.07726E-07	1.75077E-05	0.040718937	-24.55859764	0.743805035	18.26680857	-4.618156
Gas5	Gas5-006	2428.9051	0.000271116	0.001672197	0.040853254	-24.47785412	1.61794157	39.60373772	-4.613405
Emg1	Emg1-004	1349.2391	9.10796E-05	0.000745216	0.040863934	-24.47145703	0.784369416	19.19466246	-4.613028
Gm37062	Gm37062-001	711	1.45475E-05	0.00020107	0.040887645	-24.45726551	0.393276657	9.618471626	-4.612191
Synpo	Synpo-011	1334.2254	0.015417894	0.038694478	0.040928555	-24.43281968	0.751650929	18.36495162	-4.610748
Tle3	Tle3-001	1437.392	8.21514E-05	0.000693325	0.040936349	-24.42816778	0.705168805	17.22598187	-4.610474
Fbxo7	Fbxo7-004	1162.5911	0.036939042	0.077181685	0.040958231	-24.41511673	0.599674884	14.6411323	-4.609703
Ythdc1	Ythdc1-004	4595.8959	3.82425E-06	8.16753E-05	0.040964353	-24.41146847	2.354954095	57.48788763	-4.609487
Elovl5	Elovl5-002	182.82478	0.002802434	0.010187154	0.040998756	-24.39098402	0.13925105	3.396470126	-4.608276
Itga6	Itga6-009	3866	4.83996E-09	1.8062E-06	0.04118804	-24.27889286	2.13905544	51.93389786	-4.601631
Fbxo28	Fbxo28-002	1596.2489	0.00955593	0.026593856	0.041266266	-24.23286873	0.936944513	22.70485339	-4.598893
AI314180	AI314180-00(	176.10347	0.001835111	0.007317795	0.04130988	-24.20728392	0.066148405	1.601273212	-4.597369
10002F23R	0002F23Rik-	319.97464	0.006545515	0.019727551	0.041311776	-24.20617336	0.210569335	5.097077829	-4.597303
Zfp800	Zfp800-001	272.58035	0.008093849	0.023331095	0.041368161	-24.17317998	0.199999787	4.834630844	-4.595335
Gpatch2	Gpatch2-005	525.05945	0.001694642	0.006861507	0.04137855	-24.16711065	0.276742479	6.688066102	-4.594973
Dhx9	Dhx9-002	1072.0103	0.032548485	0.069738505	0.041525098	-24.08182147	0.679373348	16.36054768	-4.589873
Tmc5	Tmc5-004	3008.0885	1.2484E-08	2.92862E-06	0.041654337	-24.00710381	1.484434862	35.63698184	-4.585389
Rpl30	Rpl30-005	6389.9305	8.55415E-07	3.19847E-05	0.041669797	-23.99819724	4.023129018	96.54784368	-4.584854
Lrrfip2	Lrrfip2-002	1123.0227	0.00121892	0.005324324	0.04167843	-23.99322636	0.721891164	17.3204981	-4.584555
Rsu1	Rsu1-008	2036.7889	0.000190394	0.001283411	0.041679396	-23.99267014	0.929796414	22.30829865	-4.584522
Gm15787	Gm15787-004	284.62487	0.000518531	0.00274705	0.04186479	-23.8864213	0.155220549	3.707663427	-4.578119
Dpy19l1	Dpy19l1-001	2177.4865	0.005933939	0.018283322	0.041892322	-23.87072292	1.269722669	30.30919803	-4.57717
Snhg4	Snhg4-001	979.58453	0.000778526	0.003747646	0.041993998	-23.81292702	0.774267277	18.43757015	-4.573673
Slc8b1	Slc8b1-003	914.93882	0.005928799	0.018274061	0.042032489	-23.79112044	0.429068273	10.20801495	-4.572351
Gak	Gak-005	2582.4973	0.049080697	0.096665547	0.042035984	-23.7891421	1.414774815	33.65627912	-4.572231
Snapc3	Snapc3-004	487.88175	0.015952758	0.039707993	0.04206	-23.77555845	0.185259849	4.404656366	-4.571407
Celf1	Celf1-203	2170.4568	8.19707E-05	0.000692953	0.042065512	-23.7724433	1.185649127	28.18577664	-4.571218
Fam135a	Fam135a-006	5670.0321	0.000204679	0.001354639	0.04206832	-23.77085653	1.465302846	34.83150373	-4.571122
Rdh5	Rdh5-001	122.27613	0.013333467	0.034559652	0.042086561	-23.76055378	0.049461904	1.175242235	-4.570497
Fgfr1op2	Fgfr1op2-005	343.18849	0.021196068	0.049716842	0.042095944	-23.75525765	0.163471999	3.883319458	-4.570175
Hmcn1	Hmcn1-001	787.17212	0.001153489	0.005098681	0.042183741	-23.70581595	0.437793831	10.37825998	-4.567169
Luc7l3	Luc7l3-001	5694.5284	6.38981E-05	0.000577088	0.042197516	-23.69807759	3.562701254	84.42917073	-4.566698
Tnip2	Tnip2-003	314.01114	0.004325674	0.014281313	0.042252215	-23.66739842	0.210199888	4.974884501	-4.564829
10008L16R	0008L16Rik-	750.24379	0.04942806	0.097137425	0.042267164	-23.65902747	0.522282663	12.35669988	-4.564319
Nmnat3	Nmnat3-001	300.25999	0.036572302	0.076541482	0.042327566	-23.62526609	0.211039405	4.985862103	-4.562259
Rnf144b	Rnf144b-201	569.65077	0.016896287	0.041582081	0.042383954	-23.59383464	0.23416021	5.524737282	-4.560338
Gm27177	Gm27177-002	325.49792	3.1592E-05	0.000347583	0.042482889	-23.53888905	0.201943691	4.753530148	-4.556974
Dync2h1	Dync2h1-201	1105.0572	0.001284068	0.005545945	0.042493067	-23.53325068	0.535496192	12.60196613	-4.556629
32438A13R	2438A13Rik-	827.77554	1.7161E-05	0.000225889	0.042507033	-23.52551868	0.411938245	9.691060888	-4.556155
Tubgcp4	Tubgcp4-001	2102.5366	0.019836469	0.047223123	0.042512374	-23.52256303	1.195443928	28.11990514	-4.555973
Ctbs	Ctbs-002	767.58348	0.003835615	0.012987623	0.042546702	-23.5035842	0.157264303	3.696274786	-4.554809
Adam15	Adam15-013	1062.6886	0.004365116	0.014382416	0.042558199	-23.49723481	0.148775629	3.495815899	-4.554419
Gm29095	Gm29095-001	746	2.77344E-06	6.68946E-05	0.042631205	-23.4569959	0.363091254	8.517030062	-4.551946
Camk2d	Camk2d-023	2398.9382	5.71743E-05	0.000531547	0.042652706	-23.44517115	1.349726224	31.64456232	-4.551219
P2rx7	P2rx7-002	578.70197	0.0093088	0.026054294	0.042688589	-23.42546385	0.27591027	6.463326056	-4.550006
Ckap5	Ckap5-201	710.14357	0.000162529	0.00114492	0.042689813	-23.42479201	0.445386579	10.43308798	-4.549964
Ctdsp2	Ctdsp2-201	21749.069	3.45351E-07	1.88049E-05	0.042697185	-23.4207477	11.2819505	264.2317162	-4.549715
Ppp1cb	Ppp1cb-006	2578.0363	2.5857E-06	6.41276E-05	0.04271174	-23.41276681	1.408910663	32.98649681	-4.549224
Mybbp1a	Mybbp1a-005	522.16142	0.000139095	0.001024073	0.042725313	-23.40532864	0.387216937	9.062939654	-4.548765
)30048N14R	0048N14Rik-	190.68922	0.003101953	0.011023231	0.042750656	-23.39145391	0.17070293	3.992989712	-4.54791
Rabgef1	Rabgef1-002	93.467751	0.00840747	0.024016402	0.042819798	-23.35368346	0.049718391	1.16110757	-4.545578
Zbtb20	Zbtb20-003	557.31892	0.002689592	0.00986491	0.042894242	-23.31315212	0.231978881	5.408158944	-4.543072
Supt20	Supt20-007	1605.6597	0.005173717	0.01644088	0.042916829	-23.30088259	0.863258425	20.1146832	-4.542313
Tsga10	Tsga10-005	1593.8894	0.008838345	0.024986654	0.042939307	-23.28868508	0.757415232	17.63920481	-4.541557
Gm37809	Gm37809-001	358	3.87076E-05	0.000400645	0.042939688	-23.28847855	0.202926297	4.725844725	-4.541544
Raet1e	Raet1e-202	412.09968	0.001760536	0.00708559	0.043023609	-23.24305252	0.211565417	4.917426108	-4.538728
Nrros	Nrros-201	1129.976	0.000465727	0.00253193	0.043035681	-23.23653273	0.930649637	21.62507074	-4.538323
Gm38357	Gm38357-001	2185	2.74713E-06	6.64941E-05	0.043317134	-23.08555341	1.90879542	44.0655986	-4.528918
Huwe1	Huwe1-021	3187.8281	9.45496E-08	9.22599E-06	0.043506538	-22.98505116	1.664681806	38.26279646	-4.522624
Tnk2	Tnk2-004	604.66286	0.001609565	0.006600528	0.043624556	-22.92286958	0.169219931	3.879006417	-4.518716
Mapk9	Mapk9-004	606.10053	0.037230723	0.077639569	0.043628844	-22.92061671	0.325720864	7.465723079	-4.518574
l7Rn6	l7Rn6-001	1144.6516	0.007707875	0.02245426	0.043686391	-22.89042375	0.556828252	12.74603464	-4.516672
Fnip2	Fnip2-002	2586.5037	0.002748321	0.010042677	0.043768012	-22.84773639	1.467920676	33.53866464	-4.513979
P24-232H5	24-232H5.3-(	248	0.000325013	0.00191928	0.043777175	-22.8429542	0.133721446	3.054592872	-4.513677
Atp5o	Atp5o-006	263.43976	0.007117454	0.021108557	0.043836054	-22.81227243	0.168531378	3.844583699	-4.511738
Slc25a14	Slc25a14-003	191.26089	0.000567421	0.002951117	0.043977892	-22.73869799	0.135488576	3.080833805	-4.507078
Rmnd1	Rmnd1-008	768.97382	0.007785043	0.022642851	0.04398788	-22.7335341	0.376682391	8.563321983	-4.50675
Ccdc63	Ccdc63-006	739	1.04339E-06	3.59868E-05	0.044001025	-22.72674348	0.398334746	9.052851601	-4.506319
Zfx	Zfx-002	4190.9084	0.000360593	0.002078128	0.044053289	-22.69978077	2.526628894	57.35392199	-4.504606
Ska3	Ska3-201	365	0.004471038	0.014660056	0.044189873	-22.62961902	0.249485682	5.645765938	-4.50014
Ntng2	Ntng2-005	354.41782	0.02047946	0.048393921	0.044204189	-22.62229014	0.174038141	3.937141312	-4.499673
Exo5	Exo5-001	465.85261	4.79157E-05	0.000466888	0.044240161	-22.6038962	0.318565853	7.200829478	-4.4985
Tpcn1	Tpcn1-002	734.85698	0.004713201	0.015267863	0.044263269	-22.59209547	0.269963201	6.099034416	-4.497746
Igfbp7	Igfbp7-002	188.08469	0.002255821	0.008606647	0.044278953	-22.58409313	0.061173901	1.381557083	-4.497235
H2afz	H2afz-001	8869.1642	9.52951E-06	0.000149787	0.044394486	-22.52531983	6.587589308	148.3875561	-4.493476
H2-K1	H2-K1-004	8381.966	4.58384E-06	9.22965E-05	0.044398883	-22.5230891	4.947279506	111.4280171	-4.493333
Tm4sf1	Tm4sf1-004	60508.602	4.24559E-10	5.19327E-07	0.044480178	-22.48192439	40.09242409	901.3548472	-4.490694
Ccnl2	Ccnl2-006	2429.5062	5.60891E-05	0.00052359	0.044493207	-22.47534103	1.301772443	29.25777959	-4.490271
Gngt2	Gngt2-004	2062.6371	0.012878915	0.033600154	0.044499753	-22.472035	1.224037443	27.50661226	-4.490059

Fbxw7	Fbxw7-003	1743.2949	4.70384E-05	0.000460726	0.044507698	-22.46802357	0.732218743	16.45150798	-4.489801
Cenpe	Cenpe-001	2433.4526	0.045776853	0.091478533	0.044515286	-22.46419378	2.076222309	46.64066029	-4.489555
Strip2	Strip2-004	84.625961	0.028503718	0.062664634	0.044569428	-22.43690475	0.0114715	0.257384948	-4.487802
Mecom	Mecom-201	1679.0994	0.008021196	0.023178254	0.044659143	-22.39183159	0.865848032	19.38792331	-4.484901
Erdr1	Erdr1-201	728.11548	0.006295633	0.019137785	0.044698917	-22.37190691	0.444494454	9.944188547	-4.483616
Gp49a	Gp49a-001	2414.9572	0.035977185	0.075531358	0.044740805	-22.35096137	2.54981286	56.99076874	-4.482265
Ywhaq	Ywhaq-003	9042.0156	0.000263775	0.001639024	0.044747138	-22.34779784	5.239078859	117.0818752	-4.482061
30112A20F	0112A20Rik-	2445	2.19509E-07	1.49728E-05	0.044755234	-22.34375524	1.590017472	35.52696124	-4.4818
Ap1g1	Ap1g1-004	226.15977	4.83901E-05	0.000469907	0.044800896	-22.32098193	0.113885204	2.542029585	-4.480329
Map4k5	Map4k5-202	330.99573	0.002332982	0.008831193	0.045018777	-22.21295353	0.170028585	3.776837048	-4.473329
Enpp6	Enpp6-002	13042.009	8.93567E-08	8.98218E-06	0.045040404	-22.20228771	5.943965541	131.9696331	-4.472636
Uba6	Uba6-001	4730.6389	3.789E-09	1.6427E-06	0.045081094	-22.18224796	3.249735619	72.0864413	-4.471334
Catip	Catip-001	108.98623	0.021143648	0.049617674	0.045219941	-22.11413759	0.095926482	2.121331421	-4.466897
Atg13	Atg13-001	414.8989	0.011251631	0.030229712	0.045249678	-22.0996047	0.255453323	5.645417451	-4.465949
B4galt4	B4galt4-003	869.18643	0.005642064	0.017588122	0.045253833	-22.09757558	0.451468243	9.976353624	-4.465816
Atp11b	Atp11b-005	1131.8864	0.003379285	0.011783803	0.045294323	-22.07782183	0.83287807	18.38813363	-4.464526
Rab9	Rab9-002	919.51835	0.018093956	0.043858315	0.045336336	-22.0573624	0.555086103	12.24373534	-4.463188
Sdccag8	Sdccag8-006	2282.0384	4.27875E-05	0.000431402	0.045387344	-22.03257373	1.168499987	25.74506211	-4.461566
Txk	Txk-007	248.61634	8.20727E-05	0.000693216	0.045507296	-21.97449839	0.132663755	2.915219471	-4.457758
Trim30a	Trim30a-003	1120.7772	8.93031E-06	0.000143261	0.045522956	-21.96693892	0.616868724	13.55071758	-4.457262
Tubgcp3	Tubgcp3-010	1042.9088	0.008201964	0.02355898	0.04554778	-21.95496701	0.507214747	11.13588303	-4.456475
42624	Sept11-003	648.59696	2.08632E-05	0.000260505	0.045596242	-21.93163203	0.279984449	6.140515916	-4.454941
Lrrc40	Lrrc40-001	1070.8445	0.015237401	0.038328508	0.045603336	-21.92822024	0.581439391	12.74993102	-4.454717
Rab11a	Rab11a-008	3035.0412	3.65807E-05	0.000384439	0.045630488	-21.91517233	1.331165967	29.17273157	-4.453858
Rbm41	Rbm41-001	511.43709	0.000139753	0.001028038	0.045689168	-21.88702576	0.306100342	6.699626064	-4.452004
Olr1	Olr1-001	1972.6927	0.018655512	0.044980731	0.045728077	-21.86840252	2.017046867	44.10959278	-4.450776
Gm15191	Gm15191-001	345	1.37283E-05	0.000193099	0.045786319	-21.84058499	0.232572034	5.079509286	-4.44894
Kif15	Kif15-201	1094	0.033378067	0.071174124	0.045860273	-21.80536518	0.84011934	18.319109	-4.446611
Sub1	Sub1-002	5050.0147	9.72521E-07	3.4339E-05	0.045865871	-21.80270361	3.286223764	71.6485627	-4.446435
Cacnb4	Cacnb4-201	299.9576	0.016601929	0.040999069	0.045985864	-21.74581311	0.133630027	2.905893592	-4.442666
Gtf2a2	Gtf2a2-008	977.15416	0.005165433	0.016423698	0.046041135	-21.7197078	0.455027262	9.883059161	-4.440933
Ssrp1	Ssrp1-201	2440.409	9.80092E-06	0.000152721	0.046121896	-21.68167593	1.456606126	31.58166199	-4.438404
Usp21	Usp21-004	193.36886	0.010888987	0.029463406	0.046138555	-21.67384746	0.067046734	1.453160694	-4.437883
Slc41a3	Slc41a3-002	484.92532	0.039006018	0.080488981	0.046139669	-21.67332406	0.265535345	5.755033573	-4.437849
Uba1	Uba1-001	9473.9828	4.0482E-07	2.05608E-05	0.046196063	-21.64686653	5.753042689	124.5353472	-4.436086
Gm21974	Gm21974-001	255.02633	0.028393534	0.062486572	0.046259241	-21.61730248	0.140671365	3.040935448	-4.434115
Tm9sf4	Tm9sf4-005	1805.3387	0.025628834	0.057586273	0.046316758	-21.5904574	1.105201908	23.86181473	-4.432322
Ddx58	Ddx58-004	359.43971	0.004287479	0.014181182	0.046342766	-21.57834068	0.208830883	4.506223931	-4.431512
Cbll1	Cbll1-002	470.03229	0.043464411	0.08775901	0.046354061	-21.5730827	0.288937532	6.233273282	-4.43116
Hmcn1	Hmcn1-003	1542.8447	1.64973E-06	4.74002E-05	0.046397317	-21.55297052	0.772080296	16.64062385	-4.429815
Tmem254c	Tmem254c-00	498.13006	0.006810257	0.020365956	0.046436207	-21.5349201	0.238023663	5.125820566	-4.428606
Ccna2	Ccna2-001	1763.9706	0.036098699	0.075744681	0.046525475	-21.49360098	1.447794983	31.11832766	-4.425835
Slc29a1	Slc29a1-003	752.87849	0.002097121	0.008123318	0.046533289	-21.48999189	0.290906203	6.251571941	-4.425593
Psme2	Psme2-201	1495.9089	0.012545156	0.032912652	0.046717071	-21.40545166	0.838653356	17.95175387	-4.419906
Plekhb1	Plekhb1-201	52.968947	0.034848993	0.073666248	0.046786737	-21.3735786	0.041218654	0.880990136	-4.417757
Scn3b	Scn3b-003	2525.429	0.000324598	0.0019178	0.046875941	-21.3329052	0.860910155	18.36571473	-4.415009
H2-Aa	H2-Aa-004	1269.4523	0.002600154	0.009610972	0.046895437	-21.32403617	1.197784058	25.54159059	-4.414409
Nrd1	Nrd1-009	3814.1397	2.95816E-07	1.71608E-05	0.04694194	-21.30291168	1.928955723	41.09237342	-4.412979
Gm10552	Gm10552-001	472	3.53126E-06	7.80295E-05	0.047048679	-21.25458183	0.281963813	5.993022929	-4.409702
Gm20483	Gm20483-001	292.78989	0.000113313	0.000876258	0.04719885	-21.18695658	0.165776976	3.512309587	-4.405104
Tmem159	Tmem159-00	145.01572	0.012633263	0.033100534	0.047223236	-21.17601606	0.066813778	1.41484963	-4.404359
Anp32e	Anp32e-002	2070.01	6.20014E-05	0.000564023	0.047487909	-21.05799165	1.032370988	21.73965965	-4.396296
Myo6	Myo6-003	1451.9503	0.000800969	0.003834126	0.047535126	-21.03707461	0.809055231	17.02015526	-4.394862
Mier1	Mier1-201	1467.6	9.02528E-07	3.30252E-05	0.047602926	-21.00711195	0.836714956	17.57696475	-4.392806
Tex13	Tex13-002	218	7.33208E-05	0.000639805	0.047714268	-20.95809164	0.099827119	2.092185908	-4.389435
Emp1	Emp1-003	4803.5159	0.005257428	0.016658978	0.047722596	-20.95443424	3.106467982	65.09427905	-4.389184
Pole	Pole-001	989.60023	0.009391192	0.026248436	0.047743438	-20.94528682	0.890231456	18.64615319	-4.388554
Ccdc149	Ccdc149-001	1224.4044	0.010928748	0.029554647	0.04778853	-20.92552345	0.618121445	12.9345148	-4.387192
Thrap3	Thrap3-009	479.78024	0.011610639	0.031014223	0.047823794	-20.91009346	0.305643813	6.391040703	-4.386128
Trerf1	Trerf1-004	830.00035	0.009292469	0.026012838	0.047842871	-20.90175557	0.343583303	7.181494221	-4.385552
Nxpe2	Nxpe2-003	601.0133	0.010541304	0.028749775	0.047862072	-20.89337062	0.199074348	4.159334142	-4.384973
Rcbtb1	Rcbtb1-010	2750.0928	9.57632E-08	9.29147E-06	0.047895279	-20.8788845	1.562003187	32.61288413	-4.383973
610010F05R	0010F05Rik-	1561.3717	0.012637322	0.033103501	0.04797958	-20.8421998	0.905324139	18.86894659	-4.381436
Gm16548	Gm16548-001	1960.5719	4.59252E-07	2.21853E-05	0.048025861	-20.82211518	1.369855667	28.52329248	-4.380045
P24-491C2	24-491C20.4-	1262.7489	7.79094E-05	0.000667266	0.048029245	-20.8206479	0.839483175	17.47858361	-4.379943
Glce	Glce-201	9817.1008	5.99619E-06	0.000109472	0.048201294	-20.74633111	5.023618907	104.2216612	-4.374784
Eif4a2	Eif4a2-005	3629.4556	0.02228082	0.05167026	0.048271395	-20.71620279	2.530016518	52.41233525	-4.372688
Gnas	Gnas-017	11349.151	4.92743E-06	9.68163E-05	0.048352728	-20.68135653	5.749127323	118.8997519	-4.370259
Foxp1	Foxp1-024	1252.985	0.001682079	0.006825972	0.048380177	-20.66962255	0.53643197	11.08784635	-4.36944
Trp53inp1	Trp53inp1-00	430.30281	0.011278487	0.030287609	0.048664831	-20.5487201	0.224248921	4.608028318	-4.360977
Ybx1	Ybx1-004	17637.667	1.93882E-07	1.39912E-05	0.048671665	-20.54583494	9.197606745	188.97251	-4.360774
Srsf5	Srsf5-012	1264.7701	0.008081	0.023307306	0.048728827	-20.52173354	0.705673435	14.4816422	-4.359081
Cdh17	Cdh17-002	385	0.00032303	0.0019115	0.048753841	-20.51120433	0.198407874	4.06958445	-4.35834
Zeb1	Zeb1-009	2555.4304	0.000575082	0.00297936	0.048783298	-20.49881891	1.384539868	28.38143203	-4.357469
10059E24R	0059E24Rik-	1469.6341	5.17888E-05	0.000493536	0.048798121	-20.49259243	0.750352047	15.37665869	-4.357031
Limk2	Limk2-004	867.4394	0.001082891	0.004843395	0.048826863	-20.48052926	0.614805656	12.59154523	-4.356181
Cpne1	Cpne1-001	550.17723	0.000211455	0.001389549	0.048922509	-20.44048865	0.18783182	3.839374185	-4.353358
Slu7	Slu7-201	2461.291	6.45901E-05	0.000582008	0.048940589	-20.43293775	1.382224	28.24289695	-4.352825
Tctn2	Tctn2-002	767.50734	0.001766036	0.007101881	0.048951229	-20.42849624	0.328050818	6.701584904	-4.352511
Ptafr	Ptafr-001	2109	0.008229231	0.02360958	0.048955318	-20.42678988	2.282131211	46.61661676	-4.352391
410089E03R	0089E03Rik-	930.26023	0.000188327	0.001274744	0.048957747	-20.42577649	0.528313692	10.7912174	-4.352319
Kirrel3	Kirrel3-004	417.16083	0.041717023	0.084941921	0.04898514	-20.41435406	0.153781346	3.139346853	-4.351512
Aktip	Aktip-001	1101.6496	0.004660184	0.01513198	0.04908666	-20.37213357	0.525694868	10.70952606	-4.348525
00012H06R	0012H06Rik-	1427.8035	0.001249306	0.005429967	0.049141585	-20.34936418	0.753258343	15.32832835	-4.346912
Ppp6c	Ppp6c-002	1382.9806	1.27334E-05	0.00018309	0.049189258	-20.32964187	0.857581863	17.43433215	-4.345513
Hnrnpab	Hnrnpab-002	6880.7069	1.33404E-05	0.000189275	0.049279227	-20.29252591	5.476608068	111.1342111	-4.342877
Lrig2	Lrig2-006	3326.0246	0.000808831	0.003863665	0.049293352	-20.28671118	2.006548332	40.70626647	-4.342463
Jkamp	Jkamp-001	1191.9047	0.000939684	0.004341587	0.049316336	-20.2772568	0.596784585	12.10115429	-4.341791
Txndc5	Txndc5-003	4043.5556	0.000289653	0.00176105	0.049325203	-20.27361135	3.524053464	71.44529031	-4.341531
Smarca2	Smarca2-019	2893.1807	0.012243302	0.032293654	0.049353371	-20.26204054	1.900017922	38.49824015	-4.340708
Agl	Agl-004	270.0255	0.00932071	0.026079103	0.049380065	-20.25108697	0.141165777	2.858760428	-4.339927
Rps16	Rps16-003	5497.8034	2.42781E-05	0.000288423	0.049410104	-20.23877534	3.934098367	79.62133301	-4.33905
Farp1	Farp1-004	724.55981	0.007986184	0.023094894	0.049530777	-20.18946722	0.315742358	6.374669977	-4.335531
Epc1	Epc1-004	508.36761	0.034312322	0.072784163	0.04953785	-20.18658452	0.282266763	5.698001868	-4.335325
Mapre2	Mapre2-201	1232.2526	0.039069404	0.08060015	0.049561126	-20.17710394	0.591458152	11.9339126	-4.334647
Ctsh	Ctsh-003	16723.635	5.95389E-09	2.01902E-06	0.049745251	-20.10242143	6.003944408	120.6938208	-4.329297
Gm37277	Gm37277-001	367	9.78423E-05	0.00078516	0.049771635	-20.09176531	0.262824469	5.280607554	-4.328532
Usp14	Usp14-007	281.6393	0.000467796	0.002542022	0.049798466	-20.08093996	0.210958892	4.236252849	-4.327755
Lpin2	Lpin2-001	1872.9627	5.82527E-06	0.000107556	0.049800618	-20.08007229	1.53525222	30.82797556	-4.327693
Csf2rb	Csf2rb-201	4862	0.015889096	0.039601334	0.049869809	-20.0522122	5.742707656	115.1539925	-4.32569
P23-386C1	23-386C10.5-	1134	1.38725E-05	0.000194488	0.049874498	-20.05032695	0.67412817	13.51649021	-4.325554
Gngt2	Gngt2-007	557.47614	0.001941691	0.007660744	0.049912843	-20.03492372	0.302715486	6.064881676	-4.324445
Sh3bp5	Sh3bp5-003	790.57944	0.015012529	0.037891005	0.049966214	-20.01352365	0.560220252	11.21198126	-4.322903
H2-Q1	H2-Q1-003	288.53164	0.009932156	0.027424729	0.050002398	-19.99904099	0.171869772	3.437230623	-4.321859
Zbtb20	Zbtb20-020	342.02379	0.0003095	0.001851502	0.05001793	-19.99283056	0.202192575	4.04240189	-4.321411
Poglut1	Poglut1-005	264.47649	0.002282446	0.008688974	0.050107352	-19.95715131	0.176059616	3.513648388	-4.318834
Gm13622	Gm13622-001	503	3.42337E-05	0.000366181	0.050153227	-19.93889631	0.328590229	6.551726507	-4.317514

Rbbp6	Rbbp6-005	3646.7446	7.00722E-07	2.81865E-05	0.050179882	-19.92830506	2.615861959	52.12969511	-4.316747
10026B05R	0026B05Rik-	7968.51	2.32224E-09	1.34808E-06	0.050261615	-19.89589856	5.154407413	102.551567	-4.314399
Ube4a	Ube4a-004	1379.9262	0.001369189	0.00582688	0.050451662	-19.82095276	0.632568622	12.53811278	-4.308954
Ankrd50	Ankrd50-201	1670.6713	0.001166684	0.005142718	0.050484011	-19.80825161	1.068714091	21.16935762	-4.30803
Lamb1	Lamb1-001	2377.5238	0.01541128	0.0386835	0.050599035	-19.76322267	2.433754219	48.09882657	-4.304746
Pja1	Pja1-003	273.98809	0.004530673	0.014815309	0.050607763	-19.75981419	0.160588681	3.1732025	-4.304497
Eif3s6-ps1	Eif3s6-ps1-00	1176	2.96425E-07	1.71608E-05	0.050742801	-19.70722905	0.82332778	16.22550915	-4.300653
Gtpbp3	Gtpbp3-002	428.47958	0.003582995	0.012325992	0.050761551	-19.69994957	0.251581406	4.956141012	-4.30012
Creb1	Creb1-005	673.33685	0.01330533	0.034502389	0.050774673	-19.69485862	0.286923298	5.650913793	-4.299747
Mxra8	Mxra8-005	7773.3002	0.000403739	0.002265781	0.050817853	-19.67812369	3.087084999	60.74804045	-4.298521
Prdx2	Prdx2-201	1063.9693	0.014677711	0.037259977	0.050862156	-19.66098331	0.626038873	12.30853984	-4.297264
Golim4	Golim4-001	12203.36	6.1027E-07	2.59327E-05	0.050877761	-19.65495299	6.745742953	132.5872606	-4.296821
Huwe1	Huwe1-018	2020.5732	0.00157967	0.006509889	0.050954723	-19.62526633	1.382557912	27.13306724	-4.29464
Atf7ip	Atf7ip-007	1661.3387	0.001510589	0.006289041	0.05101845	-19.60075229	1.152475158	22.58938009	-4.292837
Mllt10	Mllt10-004	480.07741	0.00581257	0.017991914	0.051029798	-19.59639333	0.220135595	4.313863706	-4.292516
Myo1f	Myo1f-003	476.90618	0.011901588	0.031607591	0.051195462	-19.53298142	0.548146008	10.70692579	-4.28784
Mterf4	Mterf4-002	2305.0449	0.018133941	0.043936579	0.051242783	-19.51494304	1.195142346	23.32313481	-4.286507
H2-T10	H2-T10-201	299.67729	0.024748322	0.056023136	0.051350222	-19.47411264	0.132781294	2.585797878	-4.283486
Stradb	Stradb-003	125.88638	0.041445436	0.084499445	0.051432411	-19.44299288	0.065700504	1.27741443	-4.281178
Hnrnpr	Hnrnpr-010	1151.9378	4.14752E-05	0.000421021	0.05149293	-19.42014192	0.78994444	15.34083314	-4.279482
RP24-95O4.	P24-95O4.1-0	423	0.000145375	0.001056015	0.05154829	-19.39928554	0.276716604	5.368104409	-4.277932
Sumo3	Sumo3-002	2020.9532	0.000685945	0.003409813	0.051638	-19.36558363	1.107668059	21.45063844	-4.275423
Gm36638	Gm36638-001	1693	6.84796E-07	2.77894E-05	0.051675894	-19.35138261	1.172090035	22.68156273	-4.274365
Rap2a	Rap2a-001	2254.9685	0.000227139	0.001467282	0.051710599	-19.33839504	1.598786823	30.91797117	-4.273396
Ggta1	Ggta1-003	3561.9502	0.001036847	0.004681665	0.051787453	-19.30969643	2.696656126	52.07161117	-4.271254
Krcc1	Krcc1-202	1795.6849	0.024602975	0.055789983	0.051908987	-19.26448683	1.14727934	22.10174775	-4.267872
Rtfdc1	Rtfdc1-002	825.29882	0.043757376	0.088217278	0.051935199	-19.25476417	0.529239072	10.19037352	-4.267144
Dnaja1	Dnaja1-002	5883.1603	1.20961E-07	1.06644E-05	0.051940616	-19.25275572	3.820340412	73.55208074	-4.266993
Rbm5	Rbm5-011	2111.8173	0.004075311	0.013609537	0.051947009	-19.2503865	1.262327377	24.3002899	-4.266816
Casp4	Casp4-005	520.85145	0.000106733	0.000835636	0.052034508	-19.21801574	0.317389735	6.099600924	-4.264387
Atl3	Atl3-202	10711.29	2.63636E-07	1.61538E-05	0.052320992	-19.11278755	6.24936695	119.4428228	-4.256466
Senp3	Senp3-008	1113.2139	1.68519E-05	0.000223366	0.052435905	-19.07090187	0.67488955	12.87075238	-4.253301
Mob1a	Mob1a-002	12474.827	0.000421745	0.002344551	0.052437218	-19.07042443	8.382581336	159.8593839	-4.253265
Ifi203	Ifi203-201	668.4648	0.000223596	0.001449037	0.052440183	-19.06934626	0.450888621	8.598151244	-4.253183
Gm6483	Gm6483-002	2672.0515	3.02751E-05	0.000337059	0.05247218	-19.05771795	1.879553266	35.81999601	-4.252303
Wdfy1	Wdfy1-008	611.01412	0.003228314	0.011379	0.052540059	-19.03309608	0.348718885	6.637200036	-4.250438
Pus10	Pus10-001	1002.2522	0.018271776	0.04421205	0.052553676	-19.02816478	0.376381642	7.161851909	-4.250065
Samhd1	Samhd1-008	601.22086	0.004110804	0.013707801	0.052563865	-19.0244764	0.47364795	9.010904239	-4.249785
Sult6b1	Sult6b1-201	197.84099	0.003860065	0.013049771	0.052645912	-18.99482735	0.131968385	2.506716684	-4.247535
Rab1	Rab1-201	17877.404	2.66712E-09	1.47337E-06	0.052694975	-18.97714158	11.31328945	214.6938956	-4.246191
Kif11	Kif11-201	2054	0.045312222	0.090671879	0.052714219	-18.97021388	1.768201066	33.54315241	-4.245664
00076A07R	0076A07Rik-	112.62609	0.002979034	0.010695168	0.052727027	-18.96560577	0.047379083	0.898573006	-4.245314
Gas5	Gas5-020	478.09636	0.014757981	0.037416601	0.052871041	-18.91394585	0.45517976	8.609245334	-4.241378
Lmo4	Lmo4-002	2342.4912	0.010747934	0.029150835	0.05294886	-18.88614785	1.973258945	37.26726019	-4.239257
Gm27232	Gm27232-001	549.72733	0.013561583	0.035007854	0.052975039	-18.87681493	0.307614487	5.806781735	-4.238543
Klhl4	Klhl4-002	1014.752	0.000766259	0.003705261	0.053100909	-18.8320692	0.553267437	10.41917066	-4.23512
Atp2c1	Atp2c1-009	902.2553	0.006083576	0.018667129	0.053112088	-18.82810561	0.591918387	11.14470191	-4.234816
Lrrc47	Lrrc47-004	1073.702	0.001316188	0.005649051	0.053230474	-18.7862313	0.827801533	15.55127107	-4.231604
Cdk5rap3	Cdk5rap3-01(	558.09199	0.010552619	0.028766888	0.05334843	-18.74469412	0.279446427	5.238137807	-4.22841
Stag1	Stag1-007	2230.4085	0.004610128	0.015016347	0.053364329	-18.73910953	1.288720506	24.14947472	-4.22798
Rpl36a	Rpl36a-004	1388.3887	0.002643664	0.009735029	0.053368726	-18.73756538	1.063058311	19.9191246	-4.227862
Rnmtl1	Rnmtl1-001	156.5403	0.013190971	0.034268109	0.053407052	-18.72411923	0.093532354	1.751310945	-4.226826
Umps	Umps-002	879.04681	0.022754066	0.052508205	0.053442384	-18.71174003	0.912485162	17.07418513	-4.225872
Fam92a	Fam92a-005	363.85906	0.00197109	0.007750841	0.053473743	-18.70076672	0.156579971	2.928165518	-4.225026
Scamp3	Scamp3-003	101.91814	0.038033094	0.078947483	0.05353204	-18.68040163	0.023763	0.443902393	-4.223454
Arhgap44	Arhgap44-003	3016.9806	0.007897217	0.022906478	0.053542697	-18.67668353	1.799084691	33.60093543	-4.223166
Vamp4	Vamp4-006	995.48869	0.027338304	0.060667157	0.053708338	-18.61908291	0.764719578	14.23837722	-4.21871
Rps27	Rps27-002	1475.8278	0.024044919	0.05477153	0.053727685	-18.61237843	1.207915583	22.48218195	-4.218191
Syncrip	Syncrip-006	659.03285	0.003009253	0.01078666	0.053739365	-18.6083331	0.410533361	7.639341534	-4.217877
Abi2	Abi2-003	536.9834	0.025324168	0.057070191	0.053755537	-18.60273496	0.431686167	8.030543358	-4.217443
Itga6	Itga6-005	1592.8487	8.36692E-05	0.000701339	0.053770373	-18.59760224	1.320383041	24.5559586	-4.217045
Fam175a	Fam175a-003	633.32999	0.002134147	0.00823044	0.053820045	-18.58043784	0.431217925	8.012217859	-4.215713
Tctex1d2	Tctex1d2-001	578.30334	0.022750643	0.052503842	0.054009159	-18.51537805	0.424513634	7.860030417	-4.210652
Dhx57	Dhx57-201	1062.6913	0.003460084	0.011992298	0.054127026	-18.47505895	0.641979703	11.86061286	-4.207507
Tpm3	Tpm3-001	6103.7869	1.61623E-08	3.19391E-06	0.054183732	-18.45572402	4.693723373	86.62606319	-4.205996
Tm9sf1	Tm9sf1-004	1992.3395	0.042504753	0.086217815	0.054195258	-18.45179879	1.365618744	25.19812228	-4.20569
Rps15	Rps15-002	4553.2686	5.13157E-09	1.84491E-06	0.054217964	-18.44407133	3.253514988	60.00806251	-4.205085
Lcorl	Lcorl-005	301.32609	0.013682723	0.035248834	0.054233136	-18.43891171	0.258913335	4.77408012	-4.204682
Pbrm1	Pbrm1-012	1608.3913	0.00044065	0.002427054	0.054312295	-18.41203733	0.917683602	16.89642474	-4.202577
Apbb2	Apbb2-008	5029.9062	2.27787E-05	0.000276623	0.05434591	-18.4006489	2.842409557	52.30218027	-4.201685
P24-503L4.	24-503L4.4-(	2008	2.47765E-06	6.23966E-05	0.054363245	-18.39478132	1.375131109	25.29523605	-4.201225
Inhba	Inhba-202	768.41176	0.013327659	0.034552443	0.054460263	-18.3620121	0.772901765	14.19203156	-4.198652
Mbtd1	Mbtd1-002	2480.1367	6.73624E-06	0.000118559	0.054529198	-18.33879888	1.751530127	32.12095872	-4.196827
Arpc2	Arpc2-001	21872.583	7.49386E-10	7.67255E-07	0.054582668	-18.32083391	14.93031022	273.5357338	-4.195413
Gm37401	Gm37401-001	1366.0063	0.000100695	0.000800745	0.05459144	-18.31789028	0.720387306	13.19597564	-4.195181
Fam188a	Fam188a-014	286.5281	0.009654946	0.026805427	0.054596514	-18.31618765	0.164792876	3.018377245	-4.195047
Pcnxl3	Pcnxl3-006	1082.8854	0.003370051	0.011759986	0.054690387	-18.28474908	0.838121754	15.32484598	-4.192569
Prpf40b	Prpf40b-002	199.85855	0.036888181	0.077103638	0.054698751	-18.28195299	0.059250465	1.083214224	-4.192348
Procr	Procr-002	25650.597	2.09035E-06	5.59331E-05	0.054700568	-18.28134574	20.33887977	371.8220931	-4.1923
Birc6	Birc6-018	2891.0081	2.17019E-06	5.75747E-05	0.054879669	-18.22168441	2.097411534	38.21836741	-4.187584
Qrich1	Qrich1-002	5530.3137	9.11075E-05	0.000745266	0.054890751	-18.21800542	3.908041311	71.19671778	-4.187293
P23-14C20.	23-14C20.2-(	234	0.00012685	0.000955282	0.055070508	-18.15853946	0.148342366	2.693680714	-4.182576
Snx7	Snx7-002	728.01303	0.002561318	0.009498175	0.055083941	-18.15411133	0.541788214	9.835683546	-4.182224
Gm20775	Gm20775-001	934	1.30909E-06	4.13237E-05	0.055179733	-18.12259578	0.615848996	11.16078242	-4.179718
Gm15982	Gm15982-001	476	2.26509E-05	0.000275495	0.055203245	-18.11487712	0.362754389	6.571251188	-4.179103
10009B22R	0009B22Rik-	1391.4376	1.12314E-06	3.77874E-05	0.055306728	-18.0809829	0.858459667	15.52179457	-4.176401
Acvr2a	Acvr2a-002	665.71898	0.047653769	0.094474507	0.055336645	-18.07120766	0.46330893	8.372551889	-4.175621
Ccdc138	Ccdc138-201	363	0.000619714	0.003154755	0.055345628	-18.06827437	0.313295579	5.660710487	-4.175387
Nsmaf	Nsmaf-002	2338.7772	0.000582876	0.003009565	0.055436324	-18.03871427	1.603234109	28.92028201	-4.173025
Eef1b2	Eef1b2-009	8625.3423	2.12094E-08	3.6337E-06	0.055561291	-17.99814193	7.318831516	131.7253684	-4.169776
Snx10	Snx10-002	3543.9407	4.6392E-09	1.8062E-06	0.055564135	-17.99722078	2.699580122	48.58493948	-4.169702
Gm26582	Gm26582-201	3550.797	1.24393E-05	0.000179993	0.055574668	-17.99380963	2.601150276	46.80460289	-4.169429
Bgn	Bgn-201	1690.1153	0.00387318	0.013085069	0.055632278	-17.97517634	0.823906478	14.80986424	-4.167934
Zfp457	Zfp457-201	2059	1.79684E-07	1.33171E-05	0.055703326	-17.95224926	1.394945888	25.04241629	-4.166093
Zfp280c	Zfp280c-001	1284.7044	0.004408554	0.014494861	0.055709335	-17.95031308	0.984895145	17.67917621	-4.165937
Larp4b	Larp4b-003	623.19933	0.004424463	0.014531825	0.056066918	-17.83582963	0.532457473	9.496820774	-4.156706
S100pbp	S100pbp-002	2235.2657	0.00077417	0.003733503	0.056067688	-17.83558471	1.177338387	20.99851854	-4.156687
P2ry1	P2ry1-002	2031.9429	0.01754472	0.042823973	0.056069687	-17.83494884	0.991665593	17.68630512	-4.156635
Phf3	Phf3-005	928.41451	4.33701E-07	2.13117E-05	0.05611517	-17.82049299	0.681888322	12.15158606	-4.155465
Ccnc	Ccnc-004	840.58002	0.000227397	0.001468671	0.056192699	-17.79590631	0.674916162	12.01074478	-4.153474
Ndrg1	Ndrg1-007	90.878643	0.01248302	0.032789587	0.056194406	-17.79536547	0.043176918	0.768349038	-4.15343
Ttc8	Ttc8-002	297.71007	0.002948906	0.010611477	0.056234934	-17.7825406	0.174745088	3.107411627	-4.15239
Tbc1d19	Tbc1d19-001	698.60146	0.000858408	0.004047981	0.056247868	-17.77845151	0.233363257	4.148837358	-4.152058
Tor1aip2	Tor1aip2-006	1280.1519	0.022107059	0.051343783	0.056254703	-17.77629145	0.782790645	13.91511465	-4.151882
Rbbp5	Rbbp5-002	2167.526	0.00091347	0.004251439	0.056272116	-17.77079078	1.568259233	27.86920673	-4.151436
Use1	Use1-002	459.86743	0.000582933	0.003009565	0.056318412	-17.75618258	0.285486687	5.06915374	-4.15025
Atf7ip	Atf7ip-003	5666.8882	0.000717236	0.003527981	0.056382567	-17.7359786	3.937884811	69.84224073	-4.148607

Gm37342	Gm37342-001	216	0.001558844	0.006441116	0.05641763	-17.72495598	0.237697155	4.213171619	-4.14771
Tmem167b	mem167b-00	1711.6866	2.35423E-06	6.0223E-05	0.056461102	-17.71130851	1.069732285	18.94635853	-4.146599
Mxi1	Mxi1-203	1144.8313	0.006711046	0.020127262	0.056503924	-17.69788587	0.952804489	16.8626251	-4.145505
Alg11	Alg11-003	511.84767	5.77581E-05	0.000535635	0.05654905	-17.68376317	0.394949507	6.984193555	-4.144353
Elf4	Elf4-201	6030.8233	8.56021E-05	0.000712683	0.056579941	-17.67410815	3.677577415	64.99790095	-4.143566
Vps53	Vps53-013	137.87683	0.03754512	0.078145738	0.05670179	-17.63612767	0.087035956	1.534977227	-4.140462
Cks1b	Cks1b-001	1140.1533	0.000276369	0.00169605	0.056766092	-17.61615012	1.074380008	18.92643951	-4.138827
Cd180	Cd180-001	564.1942	0.001853121	0.007368166	0.056788175	-17.60929967	0.718753463	12.65674512	-4.138266
Tet2	Tet2-006	5958.905	3.85831E-08	5.30712E-06	0.056828883	-17.59668598	4.627007089	81.41999075	-4.137232
Odf2l	Odf2l-004	552.39183	0.041291817	0.084266504	0.056885649	-17.57912615	0.476407446	8.374826594	-4.135791
Chfr	Chfr-008	607.39994	0.002464918	0.009223582	0.056969669	-17.55320008	0.392465182	6.88901987	-4.133662
Amotl2	Amotl2-004	1678.8954	0.002963574	0.010648548	0.057108716	-17.51046206	0.857965859	15.02337862	-4.130145
Mrps14	Mrps14-003	1014.7115	0.000920983	0.004276528	0.057133898	-17.50274418	0.769596311	13.47004736	-4.129509
Rbmx	Rbmx-005	1780.5849	0.000433578	0.00239478	0.057233836	-17.47218217	1.378508844	24.08555764	-4.126988
Fam214b	Fam214b-002	850.46613	0.001426201	0.006020135	0.057250166	-17.46719811	0.301621567	5.268483675	-4.126576
Chtf8	Chtf8-002	1294.5098	0.000161521	0.001140403	0.057260255	-17.46412054	0.783455221	13.68235642	-4.126322
Guf1	Guf1-004	359.47571	0.000204246	0.001352754	0.057299273	-17.4522285	0.251778665	4.3940988	-4.125339
Arid1a	Arid1a-004	1136.1977	0.000160374	0.00113441	0.057317194	-17.44677178	0.696846974	12.15773012	-4.124888
510002J02R	0002J02Rik-2	654.33592	0.04227547	0.08587479	0.057426643	-17.41352005	0.51260351	8.926231499	-4.122136
Trp53bp1	Trp53bp1-002	3869.5464	8.22835E-06	0.000135621	0.057451374	-17.40602401	2.875364973	50.04867175	-4.121515
Tardbp	Tardbp-005	720.19611	0.026212802	0.058632999	0.057463108	-17.4024697	0.661652427	11.51438631	-4.12122
Angel2	Angel2-007	3410.1635	2.74372E-06	6.64585E-05	0.057488984	-17.39463699	2.007626097	34.92192716	-4.120571
Ccpg1	Ccpg1-005	6654.0212	0.000255165	0.00159828	0.057534233	-17.38095662	4.029212838	70.03157355	-4.119436
00002E11R	0002E11Rik-	5358.5462	2.88168E-05	0.00032595	0.057542442	-17.37847692	3.530717643	61.35849507	-4.11923
Sptlc2	Sptlc2-005	869.63851	1.7544E-05	0.000229884	0.057557587	-17.37390426	0.531707189	9.2378298	-4.11885
AC160635.1	C160635.1-20	190	0.000166134	0.001164573	0.057583851	-17.36597979	0.135137643	2.346797577	-4.118192
Itga2	Itga2-201	810	0.029788309	0.064955088	0.057587793	-17.36479109	1.029390368	17.87514869	-4.118093
Zc3h11a	Zc3h11a-001	2882.8599	5.00227E-07	2.3248E-05	0.057621029	-17.35477523	1.809302846	31.4000442	-4.117261
Exosc3	Exosc3-003	219.6394	0.007484045	0.021971745	0.057644105	-17.34782774	0.139964394	2.428078203	-4.116683
Inpp5d	Inpp5d-001	1513.1637	3.70699E-05	0.000388204	0.057652929	-17.34517263	1.547049738	26.83384478	-4.116462
Vps8	Vps8-009	211.14556	0.012853141	0.033545689	0.057756602	-17.3140381	0.173656964	3.006703296	-4.11387
P23-429E19	23-429E19.4-	944	3.05597E-06	7.16694E-05	0.057897556	-17.2718861	0.738274932	12.75140053	-4.110354
Mrpl52	Mrpl52-002	504.4829	0.01218771	0.032196611	0.057937108	-17.26009519	0.395156775	6.820443547	-4.109369
Gcnt1	Gcnt1-201	17737.223	2.12615E-07	1.47864E-05	0.057938065	-17.25981005	13.81700663	238.4789098	-4.109345
Papola	Papola-005	1698.8066	1.55154E-05	0.000210456	0.058090618	-17.21448387	1.192724848	20.53214265	-4.105551
Csrp1	Csrp1-002	7828.6163	7.07852E-08	7.84593E-06	0.058151962	-17.19632445	5.424415111	93.28000222	-4.104028
Pcnxl3	Pcnxl3-005	1723.2352	0.002318811	0.008798943	0.058175311	-17.18942241	1.304622163	22.42570144	-4.103449
Fntb	Fntb-002	346.34584	0.012147828	0.032108589	0.058205133	-17.18061544	0.199548372	3.428363841	-4.10271
Add3	Add3-201	2529.7541	0.001068578	0.00479293	0.058309499	-17.14986427	1.376730841	23.61074706	-4.100125
Fbxw11	Fbxw11-002	4051.9645	1.44688E-05	0.000200287	0.058355905	-17.13622649	2.367179729	40.56452797	-4.098978
P23-156K2	23-156K23.4-	273	0.000196076	0.001312669	0.058475538	-17.10116794	0.196654813	3.363026978	-4.096023
Mthfsl	Mthfsl-006	1208.3967	1.10678E-05	0.000165791	0.058504323	-17.09275413	1.079588868	18.45314708	-4.095313
Crk	Crk-002	7534.6661	5.09165E-08	6.36457E-06	0.058537716	-17.08300347	5.467787942	93.4062404	-4.09449
Glud1	Glud1-005	9318.2703	3.87049E-07	2.01638E-05	0.058575085	-17.07210492	6.077723304	103.7595299	-4.093569
Stx6	Stx6-006	755.0008	0.015193482	0.038248936	0.058598513	-17.06527932	0.732041171	12.49248705	-4.092992
Nol11	Nol11-002	831.64446	6.80166E-05	0.000605555	0.058637005	-17.05407716	0.651298408	11.1072933	-4.092045
Spns2	Spns2-002	1455.3811	0.005580997	0.0174438	0.058670488	-17.04434422	0.982025187	16.73797533	-4.091221
430110L20R	0110L20Rik-	447.12953	0.000222452	0.001443812	0.058781816	-17.01206382	0.426683147	7.258760919	-4.088486
Abr	Abr-001	242.44031	0.022963191	0.052864359	0.058956142	-16.96176109	0.068735935	1.165882513	-4.084214
Gm19967	Gm19967-001	238	0.000474829	0.002570695	0.059036634	-16.93863511	0.182033892	3.083405683	-4.082246
Alkbh1	Alkbh1-004	1743.3223	0.000564894	0.002940368	0.059084909	-16.92479536	1.462965122	24.76038531	-4.081066
Snrpg	Snrpg-201	2162	3.59441E-07	1.9146E-05	0.059156397	-16.90434258	1.739932187	29.41240975	-4.079322
Rbck1	Rbck1-002	1235.6042	4.44844E-05	0.000443549	0.05923284	-16.88252671	0.615599646	10.39287747	-4.077459
Klhdc2	Klhdc2-004	1954.4202	1.6219E-07	1.26693E-05	0.059276607	-16.8700614	1.43079635	24.13762228	-4.076393
Hnrnpll	Hnrnpll-001	2878.5324	0.001245301	0.005417606	0.059462736	-16.81725499	2.184209274	36.73240431	-4.07187
Spag9	Spag9-004	317.96772	0.015890702	0.039602454	0.059566965	-16.78782867	0.224406123	3.767291542	-4.069344
Snapc4	Snapc4-004	305.70203	0.012341585	0.032492834	0.059592725	-16.78057176	0.268807888	4.51075006	-4.06872
Tmem126a	mem126a-00	622.32717	0.012689967	0.033205849	0.059609907	-16.77573502	0.410092515	6.87960337	-4.068304
Mid2	Mid2-201	2699.9304	9.73859E-07	3.43508E-05	0.059734333	-16.74079132	1.660589368	27.79958008	-4.065296
Gm12976	Gm12976-001	44.832771	0.012942951	0.033733105	0.059771194	-16.73046731	0.046442395	0.777002976	-4.064406
P4ha3	P4ha3-004	323.56407	0.018475051	0.044610408	0.059783179	-16.7271132	0.354827446	5.935238861	-4.064117
Cxadr	Cxadr-002	427.64357	0.003184752	0.011264933	0.059807138	-16.72041225	0.290106706	4.850703723	-4.063539
Lsm6	Lsm6-001	358.99275	0.016538338	0.040862651	0.059993256	-16.66854028	0.291640753	4.861225645	-4.059056
Ppapdc1b	Ppapdc1b-006	916.7455	0.017879554	0.043461657	0.060022522	-16.66041295	0.762079191	12.69655403	-4.058352
Uhmk1	Uhmk1-002	26759.126	1.13062E-10	2.03809E-07	0.060051929	-16.65225445	18.51358053	308.2928538	-4.057646
Nedd4l	Nedd4l-201	1279.1233	0.001438936	0.006061293	0.060154514	-16.62385644	1.086471345	18.06134367	-4.055183
Pkp4	Pkp4-013	13590.608	4.07023E-05	0.000415279	0.060253638	-16.59650819	5.213540292	86.52656417	-4.052808
Senp6	Senp6-003	1030.0248	0.00046789	0.002542073	0.060266741	-16.59289978	0.826030783	13.706246	-4.052494
Pilra	Pilra-001	1646.272	0.002923341	0.010542895	0.060341402	-16.57236932	2.226927019	36.90545701	-4.050708
Wdr13	Wdr13-001	953.34904	0.000329897	0.001943751	0.060530799	-16.52051545	0.634503727	10.48232862	-4.046187
Crebzf	Crebzf-004	634.82814	2.44236E-05	0.00028975	0.06066896	-16.48289335	0.456763015	7.528776063	-4.042898
Mif4gd	Mif4gd-003	458.65841	0.017438347	0.042607333	0.060744719	-16.46233635	0.300271977	4.943178279	-4.041097
Kat6b	Kat6b-006	1036.5432	0.020409425	0.0482484	0.060787865	-16.45065167	0.646597642	10.63695258	-4.040073
42622	Sept9-005	714.14093	0.01174708	0.031300566	0.060788519	-16.45047467	0.408378235	6.718015804	-4.040057
Arhgap12	Arhgap12-007	452.94919	0.000123824	0.000938238	0.060801554	-16.44694806	0.21518517	3.539139307	-4.039748
Rpe	Rpe-002	557.33441	0.01465778	0.037217657	0.060827052	-16.44005379	0.333917267	5.489617828	-4.039143
Ebf1	Ebf1-003	2619.5173	0.008538147	0.024310545	0.060847195	-16.4346114	1.777665273	29.21523796	-4.038665
Med12	Med12-005	2444.8335	0.000746682	0.003631616	0.060921768	-16.41449399	1.811760333	29.73912911	-4.036898
Csf1	Csf1-004	14416.489	0.000164199	0.001154577	0.061017039	-16.38886487	11.36322197	186.2303093	-4.034644
Rai14	Rai14-201	4499.747	0.00017841	0.001224805	0.0610531	-16.37918468	3.139789052	51.42718473	-4.033792
Cxcl2	Cxcl2-002	366.59037	0.008388536	0.023976246	0.061137414	-16.35659625	0.412077087	6.740178537	-4.031801
Mios	Mios-002	488.76853	0.000727937	0.003565767	0.061180817	-16.34499248	0.289061479	4.724707693	-4.030777
Kcnt2	Kcnt2-006	3352.0221	0.00257555	0.009532375	0.061207654	-16.33782607	2.673954925	43.68661047	-4.030144
Gm27813	Gm27813-201	269	0.000427668	0.002369783	0.061313131	-16.30971989	0.148679231	2.424916615	-4.02766
Fnbp4	Fnbp4-003	534.63936	0.01636912	0.040532269	0.061381444	-16.2915685	0.391034824	6.37057062	-4.026054
Gm14548	Gm14548-001	607	0.019665219	0.046896934	0.061414494	-16.28280137	0.86241254	14.0425201	-4.025277
Smg5	Smg5-009	752.9117	0.001911727	0.007557322	0.06158224	-16.23844797	0.533043643	8.655801469	-4.021342
Smarca2	Smarca2-018	15795.085	9.02592E-06	0.000144389	0.061607779	-16.23171638	12.05137452	195.6144932	-4.020744
Actr3	Actr3-010	1694.4911	1.58517E-05	0.000213412	0.061611099	-16.23084179	1.801770019	29.24424411	-4.020666
Pign	Pign-201	1405.8951	1.12817E-06	3.78374E-05	0.061628358	-16.22629639	1.240406493	20.1272034	-4.020262
Kdm1a	Kdm1a-008	1451.5965	1.63002E-06	4.69933E-05	0.061684706	-16.2114739	0.898794016	14.57077573	-4.018943
Urb2	Urb2-001	2199.9012	0.000419263	0.002333783	0.061701349	-16.20710114	1.936259349	31.38115109	-4.018554
Oasl2	Oasl2-008	3544.9917	1.75739E-07	1.31708E-05	0.061797426	-16.18190374	3.03991822	49.19166402	-4.016309
Dram2	Dram2-003	484.83825	0.016372726	0.040532409	0.061830703	-16.17319485	0.147858652	2.391346797	-4.015533
Gm37072	Gm37072-001	154	0.011532214	0.030855272	0.061839166	-16.17098122	0.148139565	2.395562128	-4.015335
Zzz3	Zzz3-201	4539.4845	2.08426E-07	1.46469E-05	0.061876487	-16.16122767	3.638474096	58.80220824	-4.014465
Oasl2	Oasl2-007	7189.7759	3.12872E-05	0.00034528	0.061901346	-16.15473761	4.90247028	79.19812104	-4.013885
Ripk1	Ripk1-003	1568.7154	6.36448E-06	0.000114247	0.061919361	-16.15003747	1.256608307	20.29427125	-4.013466
Zfp433	Zfp433-003	891.38404	0.000555964	0.002904035	0.061939485	-16.14479038	0.677832932	10.94347059	-4.012997
Gm13202	Gm13202-001	152	0.00025162	0.001582443	0.061975171	-16.13549393	0.116201101	1.874962156	-4.012166
Trp53bp1	Trp53bp1-005	1140.2092	2.60607E-06	6.43995E-05	0.062058799	-16.11375056	0.884919649	14.25937449	-4.01022
BC004004	BC004004-002	2204.2705	0.007696565	0.022436577	0.062078004	-16.10876539	1.661600762	26.76633685	-4.009774
Lztfl1	Lztfl1-006	652.9202	0.004869348	0.015665405	0.062094915	-16.10437837	0.472210446	7.604655692	-4.009381
Ptbp1	Ptbp1-005	4192.5107	0.000307909	0.001845299	0.062156228	-16.08849227	3.103348742	49.92820225	-4.007957
Lipe	Lipe-003	458.50696	0.000496315	0.002656477	0.062309152	-16.04900679	0.471625639	7.56912309	-4.004412
Iars2	Iars2-002	1553.1837	0.003325871	0.011643779	0.062312815	-16.04806342	0.93383234	14.98620061	-4.004327
Fubp3	Fubp3-005	1040.7776	2.3932E-07	1.55689E-05	0.062348654	-16.03883853	0.764394026	12.25999236	-4.003498

Tug1	Tug1-003	7188.4641	2.39603E-05	0.000285838	0.062366283	-16.03430499	5.672089459	90.9480123	-4.00309
Csde1	Csde1-005	14172.218	7.81056E-05	0.000668445	0.062389169	-16.02842307	10.94234161	175.3884808	-4.002561
Gm12439	Gm12439-001	109	0.00105782	0.004756508	0.062391524	-16.02781804	0.067618821	1.083782167	-4.002506
Npr2	Npr2-003	1075.62	0.000396527	0.002235565	0.06239808	-16.0261342	0.71669402	11.48583455	-4.002355
Tspan8	Tspan8-202	95.650699	0.038656613	0.079955855	0.06250396	-15.99898626	0.055223555	0.883520892	-3.999909
P24-363K2(	24-363K20.7-	1128	1.7545E-05	0.000229884	0.062512679	-15.99675472	0.90120525	14.41635934	-3.999707
Pum2	Pum2-202	11820.582	8.18257E-09	2.33544E-06	0.062533285	-15.99148361	9.531808542	152.4277601	-3.999232
Rps27a	Rps27a-004	3456.779	0.003880754	0.013109364	0.062541108	-15.98948335	3.012593568	48.16981471	-3.999051
RP23-409I5.	P23-409I5.4-0	562	1.09389E-05	0.000164816	0.062633492	-15.9658988	0.417877986	6.671797643	-3.996922
Cald1	Cald1-016	1491.8627	0.021614246	0.050479912	0.06269374	-15.95055577	1.411593982	22.51570853	-3.995535
Mbnl1	Mbnl1-006	6671.3482	1.59613E-06	4.64858E-05	0.062699574	-15.94907173	4.425248605	70.57860744	-3.995401
Ptpmt1	Ptpmt1-201	373.655	0.011912656	0.031633467	0.062744256	-15.9377138	0.265926804	4.2382653	-3.994373
Vps13d	Vps13d-010	218.43138	0.007312297	0.021553028	0.062803585	-15.92265796	0.063070807	1.004254882	-3.993009
Ddb2	Ddb2-002	322.69195	0.008010563	0.023154203	0.062856318	-15.90929975	0.136403688	2.170087153	-3.991798
Mtmr2	Mtmr2-004	2330.152	4.60278E-05	0.000454047	0.062867388	-15.90649832	1.486961577	23.65235182	-3.991544
Eps15	Eps15-003	190.50255	0.000660635	0.003313331	0.062889645	-15.90086895	0.109031811	1.733700537	-3.991034
Arfrp1	Arfrp1-005	1007.1281	0.000269922	0.001666634	0.062899135	-15.89846984	0.750750481	11.93578388	-3.990816
Nek7	Nek7-002	17226.247	6.086E-05	0.000556152	0.06293732	-15.88882391	10.9456488	173.9134863	-3.98994
Cep170	Cep170-201	2861.8247	1.18209E-05	0.000173087	0.06294179	-15.88769558	2.616663976	41.57276068	-3.989838
30548M08F	0548M08Rik-	411.49714	0.007072932	0.021002942	0.062944404	-15.8870358	0.305118429	4.847427404	-3.989778
Rgs2	Rgs2-001	1367.304	0.006130106	0.018781727	0.063120335	-15.84275485	1.872571667	29.66669385	-3.985751
Ndufb10	Ndufb10-006	270.98152	0.002655993	0.009772024	0.063157934	-15.83332358	0.234880079	3.718932295	-3.984892
Sertad4	Sertad4-201	6789.3676	0.000120622	0.000918741	0.063168443	-15.83068946	4.495371605	71.16483188	-3.984652
Zfp426	Zfp426-008	1521.5064	0.006606655	0.019871602	0.063171174	-15.83000498	0.906122722	14.3439272	-3.98459
Rpl10	Rpl10-007	919.88562	1.4191E-05	0.000197417	0.063312014	-15.79479053	0.754931624	11.92398687	-3.981377
330018L16F	0018L16Rik-	174	0.000791898	0.003797609	0.063469153	-15.75568519	0.16903197	2.66321451	-3.977801
Ccnl1	Ccnl1-011	215.65017	0.014780769	0.03746051	0.063469592	-15.75557624	0.180997155	2.851714474	-3.977791
Cd84	Cd84-002	792.18902	0.001336308	0.005714765	0.063535353	-15.73926879	1.020787505	16.06644891	-3.976297
Ift140	Ift140-006	178.33844	0.008407494	0.024016402	0.063611324	-15.72047144	0.132183145	2.077981348	-3.974573
Tnfaip3	Tnfaip3-002	3292.3814	0.003082313	0.010973933	0.06363026	-15.71579318	3.528956043	55.46034333	-3.974143
Gm38344	Gm38344-001	346	3.30563E-05	0.000357944	0.063698643	-15.69892167	0.304069613	4.77356504	-3.972594
Amdhd2	Amdhd2-006	174.32779	0.00789794	0.022906635	0.063724109	-15.69264786	0.104209886	1.635329046	-3.972017
Pi4k2b	Pi4k2b-004	327.81663	0.000380463	0.002166393	0.063732213	-15.69065244	0.306721982	4.812668007	-3.971833
Arhgap24	Arhgap24-003	535.30061	0.04701854	0.093435706	0.063735272	-15.68989935	0.427554539	6.70828768	-3.971764
Hnrnpdl	Hnrnpdl-006	1060.2111	4.10326E-06	8.57454E-05	0.063752667	-15.68561829	0.943239532	14.79529526	-3.97137
Ddr1	Ddr1-202	531.18888	0.001003718	0.00456781	0.063780159	-15.67885712	0.351791208	5.515684092	-3.970748
Gm26821	Gm26821-201	1320.2029	3.80417E-05	0.000395299	0.063780778	-15.67870488	0.695054444	10.89755351	-3.970734
P23-262J12	23-262J12.3-(	883.94527	0.000367025	0.002102809	0.06387368	-15.65590076	0.688204321	10.77445855	-3.968635
Syngr2	Syngr2-003	865.86973	0.000513528	0.002727718	0.063900316	-15.64937489	0.550204989	8.610364141	-3.968033
Gm15356	Gm15356-001	394	0.02009482	0.047680923	0.063930923	-15.64188261	0.552645796	8.644420676	-3.967342
Svil	Svil-006	1596.3185	0.000650362	0.003274276	0.063963887	-15.63382172	0.872599945	13.64207197	-3.966599
Nfkbiz	Nfkbiz-002	2095.4082	0.000561584	0.002926698	0.064079558	-15.6056007	1.824587073	28.47377729	-3.963992
Nfat5	Nfat5-010	8961.4587	1.06731E-06	3.64823E-05	0.064235427	-15.56773337	5.624134747	87.55503018	-3.960487
Cadm1	Cadm1-008	347.55918	0.037148099	0.077506806	0.064302967	-15.551382	0.216933728	3.373619272	-3.958971
Apoo	Apoo-001	183.69071	0.002645688	0.009741432	0.064307075	-15.55038843	0.132794809	2.065010867	-3.958879
N4bp2	N4bp2-001	5907.3387	1.66666E-06	4.76145E-05	0.064491051	-15.50602735	5.069769983	78.61199202	-3.954757
Rhno1	Rhno1-003	240.50453	0.041689611	0.084906237	0.064502974	-15.50316124	0.280134167	4.342965164	-3.954491
Afap1l1	Afap1l1-003	3346.1455	1.37705E-05	0.000193451	0.064583935	-15.48372664	2.43803687	37.74989643	-3.952681
Cope	Cope-006	406.01784	0.031136376	0.067341557	0.064681265	-15.4604274	0.342938831	5.301980897	-3.950508
Galnt1	Galnt1-201	14686.538	3.80256E-05	0.000395299	0.064683487	-15.45989637	16.43790482	254.1283049	-3.950459
Mff	Mff-004	1880.982	0.034634367	0.073318936	0.064700162	-15.45591173	1.370811249	21.18713766	-3.950087
Bnip2	Bnip2-006	2266.4194	0.019083634	0.04576491	0.064724747	-15.45004116	1.631670821	25.20938134	-3.949539
Ext2	Ext2-003	1230.1403	0.000120246	0.000917246	0.064741563	-15.44602815	1.091687979	16.86224326	-3.949164
Entpd1	Entpd1-010	152.12942	0.036678801	0.076722178	0.064747172	-15.44468991	0.079106443	1.221774478	-3.949039
Cd160	Cd160-201	363.28977	0.009777423	0.027076061	0.064755302	-15.44275091	0.236700967	3.655314073	-3.948858
Usp4	Usp4-010	1146.2527	0.010383104	0.028426963	0.064801841	-15.43166035	0.644849212	9.951094013	-3.947821
Nacc2	Nacc2-002	495.11276	0.000569156	0.002957609	0.06480489	-15.43093438	0.31930637	4.927195633	-3.947754
R3hdm1	R3hdm1-011	477.88408	0.049080235	0.096665547	0.064971325	-15.39140544	0.42920661	6.606092947	-3.944053
Gm10827	Gm10827-201	1760	1.59339E-06	4.6473E-05	0.065044841	-15.37400939	1.271561003	19.5489908	-3.942422
Ldlrad3	Ldlrad3-002	1253.3891	0.012759215	0.033348835	0.065062989	-15.36972107	1.282987723	19.71916344	-3.942019
Setd2	Setd2-002	1756.1092	1.71198E-06	4.82711E-05	0.065069309	-15.36822828	1.41652652	21.76950293	-3.941879
Eef1d	Eef1d-004	594.83781	8.05688E-05	0.000685075	0.06507664	-15.36649707	0.608413472	9.34918383	-3.941716
Taok3	Taok3-005	868.98255	2.4418E-05	0.00028975	0.065090802	-15.36315378	0.804561092	12.36059579	-3.941403
RP23-1A12.	P23-1A12.5-0	202	0.004110216	0.01370739	0.065123612	-15.35541354	0.170448167	2.617302091	-3.940675
Thsd7a	Thsd7a-202	3066.7192	0.000251278	0.001581449	0.065258066	-15.32377627	2.805211548	42.98643416	-3.9377
Nsun6	Nsun6-202	61.915987	0.024760429	0.056046836	0.065271216	-15.32068888	0.053589766	0.821032129	-3.937409
Tecr	Tecr-203	947.49248	0.002751976	0.010046384	0.06527762	-15.31918589	0.394183281	6.038566948	-3.937268
Gm20663	Gm20663-001	789.45529	0.000364457	0.002091949	0.065411853	-15.28774908	0.60593863	9.263437729	-3.934304
Thsd1	Thsd1-001	1007.7005	0.014455982	0.036814438	0.065425507	-15.28455861	0.701491943	10.72199471	-3.934003
Phip	Phip-012	6001.908	4.0087E-07	2.04313E-05	0.065431597	-15.28313605	4.770786119	72.91257331	-3.933869
Sqrdl	Sqrdl-002	1460.2827	0.005408843	0.017048856	0.06545979	-15.27655373	0.725121032	11.0773504	-3.933247
Rpf1	Rpf1-002	1112.2075	0.02025992	0.04795786	0.0655342	-15.25920811	1.042848948	15.91304913	-3.931608
Gm6483	Gm6483-006	2345.032	1.30843E-06	4.13237E-05	0.065534239	-15.259199	1.802937464	27.51138154	-3.931607
Mef2c	Mef2c-013	1002.6243	0.000113176	0.000875399	0.065555261	-15.25430594	0.906447115	13.8272216	-3.931145
Angel2	Angel2-002	482.46326	0.049629462	0.097399099	0.065594772	-15.2451173	0.302650001	4.61393477	-3.930275
Palmd	Palmd-002	2841.1138	1.4082E-07	1.15661E-05	0.065723234	-15.21531953	1.26679849	19.27474381	-3.927453
Pramel4	Pramel4-002	153.68303	0.000347164	0.00201824	0.065732223	-15.21323864	0.114818129	1.746755602	-3.927255
Fxn	Fxn-001	1751.4089	6.69295E-08	7.59514E-06	0.065771709	-15.20410554	1.313365017	19.96854033	-3.926389
Dip2a	Dip2a-004	1213.0047	0.00229082	0.008713003	0.065868586	-15.1817439	1.02903098	15.62248481	-3.924266
Sri	Sri-001	1021.2371	0.000320193	0.001899309	0.065871153	-15.18115211	0.667309189	10.1305223	-3.924209
Ahcyl2	Ahcyl2-023	440	0.00027404	0.001685072	0.065897106	-15.17517315	0.344357513	5.225684888	-3.923641
Kpna4	Kpna4-001	11260.254	1.18282E-09	9.64042E-07	0.065938143	-15.16572894	10.11259021	153.3648019	-3.922743
Hnrnph1	Hnrnph1-007	7507.4388	5.55491E-06	0.000104538	0.065970147	-15.15837165	6.806553068	103.176261	-3.922043
Cds2	Cds2-002	12336.692	3.49376E-06	7.75876E-05	0.066083978	-15.13226101	9.257564453	140.0878816	-3.919556
Tmem47	Tmem47-201	2769.3691	3.89438E-08	5.3353E-06	0.06614684	-15.11788025	2.421696227	36.61091357	-3.918184
Zfp595	Zfp595-001	525.63036	0.022865938	0.052695356	0.066273083	-15.08908232	0.357936947	5.400940054	-3.915433
Setd1b	Setd1b-005	2447.9609	6.28499E-07	2.638E-05	0.066275846	-15.08845324	2.062006696	31.11249162	-3.915373
Ctnnd1	Ctnnd1-004	14762.252	3.40868E-08	4.88483E-06	0.066344118	-15.07292636	11.42690903	172.2369584	-3.913888
Gm28551	Gm28551-001	306.37753	0.034704269	0.073435143	0.066427558	-15.05399315	0.255148309	3.841000898	-3.912074
Cd276	Cd276-201	463.81197	0.022702221	0.052406218	0.066493151	-15.03914292	0.541445551	8.142877017	-3.91065
Ulk4	Ulk4-004	2708.1543	1.23203E-06	3.99216E-05	0.066500433	-15.03749602	2.048070668	30.79785452	-3.910492
Pml	Pml-001	72.592097	0.032718102	0.07003281	0.066513626	-15.03451337	0.038754738	0.582658633	-3.910206
Lsg1	Lsg1-002	592.6268	0.000768999	0.003714318	0.066514555	-15.0343033	0.619317927	9.311013545	-3.910186
933436I20R	3436I20Rik-(	283.30062	0.000387321	0.002197769	0.06655207	-15.02582866	0.235387225	3.53688811	-3.909373
Wipf3	Wipf3-202	2271.5969	0.009521164	0.026529441	0.066561939	-15.02360073	1.108284901	16.65042985	-3.909159
Sycp1	Sycp1-004	251	0.000363583	0.002088682	0.066708309	-14.99063623	0.196991677	2.953030578	-3.90599
Lrrc1	Lrrc1-002	382.40797	0.012794987	0.033421928	0.066782237	-14.97404158	0.277393761	4.153705707	-3.904392
Uxs1	Uxs1-008	211.64795	0.033181285	0.070803003	0.06678852	-14.97263311	0.158519172	2.373449408	-3.904256
Sqrdl	Sqrdl-009	727.58405	0.004284601	0.014174401	0.066979984	-14.92983326	0.459178399	6.85545693	-3.900126
Jak2	Jak2-201	4970.0279	1.2269E-05	0.000178359	0.067104765	-14.90207136	4.74781651	70.75230045	-3.897441
Cdv3	Cdv3-003	8045.8608	9.32212E-07	3.36087E-05	0.067120998	-14.8984675	6.796018875	101.2502664	-3.897092
Usp16	Usp16-002	1518.3574	7.2847E-05	0.000636482	0.06718327	-14.884658	1.367304928	20.35186624	-3.895754
330027C09F	0027C09Rik-	182.5625	0.019774134	0.047091092	0.067326637	-14.85296219	0.186910839	2.776179619	-3.892679
Psmd2	Psmd2-002	3411.0141	2.30847E-05	0.000279383	0.067334674	-14.85118947	2.989867746	44.40309238	-3.892507
Papola	Papola-004	16203.106	1.34546E-08	3.03171E-06	0.067373725	-14.84258136	13.43903566	199.4699802	-3.89167
Ube2c	Ube2c-001	963.2767	0.036905576	0.077121171	0.067437268	-14.82859592	1.190973227	17.66046073	-3.89031
Galk2	Galk2-003	350.64915	0.018112661	0.043891228	0.067470547	-14.82128198	0.315281111	4.672870249	-3.889598

Casc5	Casc5-004	1094.3517	0.004999621	0.016001547	0.067486003	-14.8178874	1.074122289	15.91622314	-3.889268
Pla2g4a	Pla2g4a-002	1861.1192	0.00101578	0.004608619	0.067488932	-14.81724439	2.095507894	31.04965258	-3.889205
Serpinb8	Serpinb8-001	3825.086	0.000156373	0.001113813	0.067537667	-14.80655241	2.298040325	34.02605452	-3.888164
Gm28582	Gm28582-001	517	0.000282679	0.001727434	0.067566367	-14.80026299	0.231425671	3.42516079	-3.887551
Dido1	Dido1-007	2723.7321	1.00476E-06	3.50438E-05	0.067584321	-14.79633132	2.229020259	32.98132226	-3.887168
Sh3glb1	Sh3glb1-001	17507.021	2.48607E-06	6.24561E-05	0.067598322	-14.79326657	13.53675999	200.252899	-3.886869
Asap1	Asap1-003	1044.0587	0.004017398	0.013467347	0.067630783	-14.7861663	1.175109311	17.37536169	-3.886176
Banp	Banp-002	696.40125	0.003766209	0.012805785	0.06774523	-14.76118677	0.400944765	5.918420567	-3.883737
Csf3r	Csf3r-201	2927.7735	0.0039619	0.013321888	0.067748019	-14.7605791	4.011268852	59.20865117	-3.883677
Diap1	Diap1-202	3166.1016	0.000107238	0.000838754	0.067748286	-14.76052094	3.345419018	49.38012747	-3.883672
Pabpc1	Pabpc1-006	33578.344	8.5713E-11	1.72687E-07	0.067764233	-14.75704739	33.9245359	500.6259841	-3.883332
Tsc1	Tsc1-006	1225.4664	0.011852578	0.031515471	0.06786799	-14.73448684	0.891762874	13.13966833	-3.881125
Stxbp3	Stxbp3-002	1909.4077	1.2735E-07	1.09317E-05	0.067935494	-14.71984576	1.526903465	22.47578349	-3.879691
Eno3	Eno3-002	102.88452	0.011062962	0.029835155	0.067957238	-14.71513595	0.076263997	1.12223509	-3.879229
Cdv3	Cdv3-004	2059.1374	0.001818607	0.007263824	0.067983492	-14.70945337	1.442605262	21.21993483	-3.878672
Ehmt1	Ehmt1-001	2458.7932	1.5218E-05	0.000207573	0.06799588	-14.7067735	1.958965755	28.81006565	-3.878409
Rfx7	Rfx7-006	238.81621	0.005673126	0.017664052	0.068148756	-14.6737822	0.195058508	2.862246055	-3.875169
Arid1a	Arid1a-201	3219.477	1.26634E-05	0.000182466	0.068179326	-14.66720283	2.341154765	34.33819178	-3.874522
Fmr1	Fmr1-008	125.87639	0.046209831	0.092166708	0.068181435	-14.66674905	0.027006802	0.39610199	-3.874477
Tmem216	Tmem216-001	241.67466	0.041786755	0.085053565	0.068238279	-14.65453133	0.177458504	2.600571213	-3.873275
Dhtkd1	Dhtkd1-004	3995.9129	1.21978E-08	2.8812E-06	0.068258901	-14.65010397	3.285162013	48.12796504	-3.872839
Rad1	Rad1-006	543.0004	0.012056482	0.031928749	0.06843932	-14.61148347	0.510154238	7.45411022	-3.869031
Rnf4	Rnf4-006	960.98875	0.000270629	0.001670401	0.068493114	-14.60000789	0.717453669	10.47482922	-3.867897
Nme2	Nme2-002	5617.4189	0.000199495	0.00132984	0.068528654	-14.59243601	4.106063308	59.91746609	-3.867149
Tia1	Tia1-001	2129.9512	4.64109E-05	0.00045638	0.068613326	-14.57442839	1.870915261	27.26752049	-3.865367
Ogt	Ogt-003	2511.3499	0.018357759	0.044376025	0.068671948	-14.5619869	2.02890246	29.54485105	-3.864135
H2afz	H2afz-005	307.66736	0.006661569	0.020005151	0.068704185	-14.55515414	0.380416742	5.537024312	-3.863458
Ssbp3	Ssbp3-007	199.35233	0.029620825	0.064647503	0.068772387	-14.54071972	0.187197265	2.721982967	-3.862027
Med12	Med12-006	1584.4093	4.59808E-05	0.000453715	0.068774941	-14.54017959	1.191793936	17.32889786	-3.861973
Gm29237	Gm29237-001	2922.6201	3.75529E-06	8.10452E-05	0.068921221	-14.50931937	1.842742361	26.73693744	-3.858908
Morc3	Morc3-005	1080.1524	6.88264E-05	0.00061071	0.068924512	-14.50862652	0.949465411	13.77543904	-3.858839
Pdcd6ip	Pdcd6ip-003	1096.8181	1.56257E-06	4.59776E-05	0.068965401	-14.50002435	1.006659359	14.59658522	-3.857983
Vps25	Vps25-001	2414.8257	0.000181771	0.001241408	0.069037097	-14.48496599	1.736082823	25.14710065	-3.856484
Sypl	Sypl-001	15986.728	3.41564E-09	1.53928E-06	0.069064072	-14.47930853	11.66389616	168.8851511	-3.855921
Diap2	Diap2-006	9557.2737	3.66011E-05	0.000384537	0.069066022	-14.47889953	6.825815621	98.83029862	-3.85588
Gm28049	Gm28049-005	269.31382	0.015940681	0.039686583	0.069141403	-14.46311402	0.131648681	1.904049885	-3.854306
Tor3a	Tor3a-002	1646.2296	0.000183988	0.001253724	0.069141764	-14.46303857	1.586873441	22.95101177	-3.854299
Nras	Nras-001	23573.39	2.35446E-11	8.06404E-08	0.069174105	-14.45627669	18.89904429	273.2098133	-3.853624
Crtc2	Crtc2-006	678.59509	2.33801E-06	5.99827E-05	0.069190281	-14.45289695	0.664444642	9.603149947	-3.853287
Zeb2	Zeb2-016	2525.8385	6.82359E-06	0.000119483	0.069201336	-14.45058799	2.936915302	42.44015299	-3.853056
Azin1	Azin1-004	828.89595	7.5422E-07	2.95223E-05	0.069217879	-14.44713429	0.496920201	7.179070419	-3.852711
Ubap2l	Ubap2l-002	791.9922	0.013151355	0.034181517	0.069256945	-14.43898509	0.638968421	9.226055496	-3.851897
Gm37494	Gm37494-001	6057.5708	3.30704E-09	1.51021E-06	0.069347172	-14.42019874	4.880227938	70.37385678	-3.850019
Suv39h1	Suv39h1-001	225.42091	0.047868559	0.094813704	0.069396117	-14.4100282	0.180116374	2.595482034	-3.849001
Phf21a	Phf21a-014	1830.5384	0.004560788	0.014892456	0.069525432	-14.38322606	1.542452989	22.18545002	-3.846315
Gigyf2	Gigyf2-007	1537.0916	9.13404E-05	0.000746815	0.069579472	-14.372055	0.914002152	13.13608921	-3.845194
Arfrp1	Arfrp1-003	502.71356	0.037052795	0.07734387	0.069587785	-14.37033805	0.344032576	4.943864416	-3.845022
Nek7	Nek7-004	6209.3139	0.011655207	0.031110001	0.069722617	-14.34254821	4.370355904	62.68204025	-3.842229
Pfas	Pfas-004	1530.0688	0.024934005	0.056347388	0.069777649	-14.3312367	1.613890618	23.12904846	-3.841091
Azin1	Azin1-003	6163.5242	2.63158E-06	6.48896E-05	0.069839722	-14.31849924	3.756929748	53.79359575	-3.839808
10417H13R	0417H13Rik-	1222.897	0.001170896	0.005155313	0.069869203	-14.31245744	1.297626413	18.57222282	-3.839199
Nlk	Nlk-002	2609.8957	1.80008E-06	5.00836E-05	0.069878923	-14.31046676	2.004585897	28.68655984	-3.838999
Dmxl1	Dmxl1-002	1287.4625	1.39176E-06	4.29054E-05	0.069995193	-14.28669545	0.969833658	13.85571812	-3.8366
Qsox1	Qsox1-008	683.98529	0.000322696	0.001909857	0.070008507	-14.28397832	0.318720548	4.552597399	-3.836326
Grsf1	Grsf1-002	3029.2332	0.018921628	0.045497457	0.07002088	-14.2814543	3.103056589	44.31616086	-3.836071
Zfp280d	Zfp280d-020	2733.2443	0.001550409	0.006408581	0.070199835	-14.24504779	2.282334105	32.51195839	-3.832389
Gsap	Gsap-001	620.44466	0.000386438	0.002193488	0.070219097	-14.24114013	0.762198857	10.85458073	-3.831993
Gm608	Gm608-201	10624.904	0.000247887	0.001565576	0.070307443	-14.22324518	7.762913273	110.4138188	-3.830179
30071D04R	0071D04Rik-	1511	0.006405694	0.019401753	0.070376323	-14.20932428	2.273230234	32.30106556	-3.828766
Dstyk	Dstyk-006	646	9.23012E-05	0.000752854	0.070458864	-14.19267852	0.413022698	5.861898379	-3.827075
Tbpl1	Tbpl1-002	517.00479	0.027361623	0.060699242	0.070566587	-14.17101279	0.320195103	4.537488905	-3.824871
Atrx	Atrx-007	722.34075	0.010755087	0.029163308	0.070597897	-14.16472792	0.519606647	7.360086783	-3.824231
Tyrobp	Tyrobp-201	2963	0.005042042	0.016107634	0.070613413	-14.16161538	4.732338443	67.01755687	-3.823914
Rpl9	Rpl9-010	305.69707	0.004737268	0.0153213	0.070651464	-14.15398833	0.262515109	3.715635794	-3.823137
Hhex	Hhex-002	5198.5311	7.12712E-06	0.000122883	0.070686104	-14.1470521	5.129284666	72.56425738	-3.82243
Cep170b	Cep170b-201	1046.192	0.00954677	0.026574843	0.070776935	-14.12889666	0.827826549	11.69627577	-3.820577
Dock11	Dock11-003	3293.3749	0.000522641	0.00276327	0.070872586	-14.10982803	3.463636862	48.87132049	-3.818628
Pbrm1	Pbrm1-006	3486.5781	0.000421565	0.00234425	0.070936011	-14.09721213	1.972332151	27.80438471	-3.817338
Ubap2l	Ubap2l-012	1369.114	8.18744E-08	8.50945E-06	0.070981154	-14.08824655	1.285176508	18.10588351	-3.81642
Idh3b	Idh3b-002	1211.1884	1.24265E-06	4.0076E-05	0.071069721	-14.0706898	1.078345211	15.17306096	-3.814621
Slc4a1ap	Slc4a1ap-003	791.78326	0.004830887	0.015566644	0.071129518	-14.05886084	0.712984038	10.02374337	-3.813408
Slc19a1	Slc19a1-008	237.65436	0.00631123	0.019171586	0.071238977	-14.03725949	0.303442311	4.259498463	-3.811189
Gm37639	Gm37639-001	1663	4.20578E-05	0.000425674	0.071245452	-14.03598367	1.779491301	24.97691085	-3.811058
Gm13882	Gm13882-001	156	0.001460656	0.006132319	0.071343951	-14.01660526	0.148139565	2.07641381	-3.809065
Siglecg	Siglecg-004	176.08637	0.004499585	0.014731938	0.071375189	-14.01047069	0.220694039	3.092027365	-3.808434
Ttc1	Ttc1-201	2873.9163	0.000163761	0.001152179	0.071408686	-14.00389854	2.004526413	28.07118452	-3.807757
Prrg1	Prrg1-002	379.2028	0.000477426	0.002581472	0.071447502	-13.99629059	0.317274337	4.440663814	-3.806973
Ss18	Ss18-202	2179.2762	4.8685E-06	9.61073E-05	0.071559517	-13.97438159	1.80635607	25.24270901	-3.804713
Gm37195	Gm37195-001	649	0.000923804	0.00428672	0.071568156	-13.97269469	0.512021032	7.154313551	-3.804538
Nup160	Nup160-002	1143.9099	6.24589E-07	2.62481E-05	0.071576883	-13.97099118	1.014655906	14.1757374	-3.804362
Rbm5	Rbm5-003	581.65311	0.001103408	0.004921437	0.071596369	-13.9671887	0.353596735	4.938752323	-3.80397
Gm26606	Gm26606-201	263	0.000715158	0.003520793	0.071653833	-13.95598757	0.282300677	3.939784745	-3.802812
Ski	Ski-002	26242.406	2.17679E-05	0.000267894	0.071703999	-13.94622349	17.23015854	240.2956418	-3.801803
Cnot6l	Cnot6l-005	882.50901	3.96526E-05	0.000407716	0.071788076	-13.9298899	0.705193713	9.823270785	-3.800112
Zmym6	Zmym6-002	569.24344	0.001548069	0.006402001	0.071793964	-13.92874756	0.489452386	6.817458724	-3.799994
Zfp639	Zfp639-001	276.463	0.04062818	0.083140058	0.071881124	-13.91185811	0.267182832	3.717009646	-3.798243
Ccnl1	Ccnl1-006	2495.2369	1.19683E-08	2.8812E-06	0.071931317	-13.90215053	2.150587965	29.89779762	-3.797236
Naa50	Naa50-003	1805.5496	9.78139E-05	0.000785124	0.071960904	-13.89643471	1.891498024	26.28507879	-3.796643
Ss18	Ss18-201	2169.2241	0.00238846	0.008996978	0.072441038	-13.80433017	1.415517091	19.54026528	-3.787049
Capn2	Capn2-004	4407.276	9.67837E-07	3.43066E-05	0.072537119	-13.78604526	2.62234302	36.15173957	-3.785137
Pole3	Pole3-002	274.27594	0.045691387	0.091338626	0.072546021	-13.78435357	0.145275552	2.002529577	-3.78496
Celf2	Celf2-202	426.06572	0.020104668	0.047699146	0.072588754	-13.77623867	0.492920658	6.790592632	-3.78411
Plgrkt	Plgrkt-006	595.16319	3.92E-05	0.000404033	0.072605609	-13.77304053	0.517821584	7.13197766	-3.783775
L2hgdh	L2hgdh-201	1679	0.000105634	0.000829616	0.072733847	-13.7487571	2.042188408	28.07755237	-3.781229
Lmo2	Lmo2-201	43135.64	6.69356E-05	0.00059764	0.072780383	-13.73996621	41.21992376	566.3603597	-3.780307
Dut	Dut-002	294.14439	0.03105362	0.067209816	0.072787888	-13.73854946	0.26870071	3.691557996	-3.780158
Naa30	Naa30-002	579.1967	0.020063258	0.047617392	0.072792128	-13.73774914	0.564932387	7.760899414	-3.780074
Ppapdc1b	Ppapdc1b-007	569.42714	0.01355174	0.034987719	0.072801203	-13.73603671	0.529296656	7.270438297	-3.779894
Lhfpl2	Lhfpl2-003	766.09943	0.04290774	0.08688088	0.072820609	-13.73237622	0.856321851	11.75933382	-3.779509
Fam185a	Fam185a-001	305.23835	0.016141715	0.040076386	0.072963515	-13.70548013	0.313144275	4.291792636	-3.776681
Ostc	Ostc-003	622.15844	0.019231774	0.046062115	0.072983235	-13.70177694	0.67972061	9.313380175	-3.776291
Fbxw11	Fbxw11-201	3890.6365	0.000106161	0.000832425	0.073001309	-13.69838456	3.29698918	45.16342568	-3.775934
Zfp715	Zfp715-004	222.76346	0.001978598	0.007769566	0.073009743	-13.69680217	0.154470719	2.115754884	-3.775767
Odf2	Odf2-001	575.57319	0.000163664	0.001151732	0.073050498	-13.68916055	0.426521343	5.838719136	-3.774962
Gm7180	Gm7180-001	337	0.000281726	0.001722441	0.073064474	-13.68654204	0.289381661	3.960634271	-3.774686
Hmbox1	Hmbox1-201	1649.7592	0.000127475	0.00095883	0.0730908	-13.68161239	1.180079109	16.14538496	-3.774166
Pramel7	Pramel7-002	140	0.00085586	0.00403763	0.073124881	-13.67523597	0.102659512	1.403893058	-3.773494

Kdr	Kdr-001	1423.2612	0.0032016	0.011311615	0.073130753	-13.67413791	1.567091853	21.42863011	-3.773378
Psmd9	Psmd9-007	95.796716	0.02362505	0.054035793	0.073140327	-13.67234792	0.065724551	0.898608926	-3.773189
Xrn1	Xrn1-008	508.50568	0.02156036	0.050384983	0.073204804	-13.66030572	0.430171524	5.876274528	-3.771918
Rap1gds1	Rap1gds1-004	383.91781	0.012907366	0.033658999	0.073237718	-13.65416657	0.343994188	4.696953942	-3.771269
Arhgap12	Arhgap12-002	2147.4683	0.004455585	0.01462072	0.07326079	-13.6498665	1.92951755	26.33765696	-3.770815
Gbp9	Gbp9-001	10234.601	0.003601976	0.012376371	0.07326934	-13.64827367	5.905545165	80.60049658	-3.770647
Ttc28	Ttc28-002	1470.4542	5.05867E-06	9.84428E-05	0.073279394	-13.64640107	1.367616696	18.66304594	-3.770449
Wac	Wac-004	4093.4769	0.000584857	0.003016318	0.073329892	-13.63700367	3.021210625	41.20026038	-3.769455
Syn3	Syn3-003	3903.389	3.83415E-06	8.1745E-05	0.073389753	-13.62588037	1.820954125	24.81210308	-3.768278
Smchd1	Smchd1-010	678.00173	0.000505037	0.002693058	0.073456602	-13.61348032	0.659171067	8.973612345	-3.766964
Fus	Fus-001	4771.642	2.22936E-05	0.00027266	0.073658811	-13.57610827	4.142194379	56.23487937	-3.762998
Exoc5	Exoc5-002	2413.4791	2.11327E-06	5.65022E-05	0.073718907	-13.56504103	2.413160017	32.73461463	-3.761822
Hif1a	Hif1a-003	4810.983	0.001481754	0.006198862	0.073780034	-13.55380235	5.110736333	69.26991014	-3.760626
Setd2	Setd2-006	792.02297	0.002477294	0.009258766	0.073793662	-13.55129938	0.595331585	8.067516539	-3.760359
Prokr2	Prokr2-003	1282.3334	0.000493352	0.002645992	0.073852362	-13.54052827	0.76380542	10.34232888	-3.759212
Gm15719	Gm15719-001	150	0.001001007	0.004557897	0.073899098	-13.53196491	0.131428719	1.778488809	-3.758299
Akap13	Akap13-009	586.93844	5.40936E-06	0.000102529	0.073957918	-13.52120265	0.449155732	6.073125668	-3.757152
Cxcr2	Cxcr2-001	7065.1183	0.000755783	0.003665137	0.073980556	-13.5170652	10.01640105	135.392346	-3.75671
Trpm7	Trpm7-005	784.92678	0.001286291	0.005554846	0.07402287	-13.50933848	0.810879617	10.95444721	-3.755885
Pqlc1	Pqlc1-006	4122.8467	5.65086E-07	2.47813E-05	0.074061496	-13.50229275	2.907295284	39.25515203	-3.755132
Ppie	Ppie-004	95.512482	0.02862941	0.062868328	0.074116789	-13.49221973	0.07377066	0.995329959	-3.754056
Sdcbp	Sdcbp-001	7020.5882	0.003652992	0.012512747	0.074199253	-13.47722466	6.908503677	93.10745612	-3.752452
Zzz3	Zzz3-003	760.26933	0.022646402	0.052316453	0.074334835	-13.45264311	0.64554153	8.684239814	-3.749818
Abcc9	Abcc9-204	308.51754	0.006755915	0.020238791	0.074393737	-13.44199177	0.223037828	2.998072643	-3.748675
Ino80c	Ino80c-002	1203.5079	0.012921615	0.033688011	0.07441602	-13.43796664	1.086154598	14.59570926	-3.748243
10002D24R	0002D24Rik-	924.7251	0.022662931	0.052343745	0.074447789	-13.43223229	0.832533793	11.18278729	-3.747627
Sp100	Sp100-009	594.66117	0.040998432	0.083775543	0.074547897	-13.41419464	0.393671815	5.280790355	-3.745689
Itga3	Itga3-006	1650.6177	0.022367271	0.05183214	0.074575111	-13.4092996	1.649940855	22.12455125	-3.745162
Nvl	Nvl-005	1088.5109	0.003117455	0.011069132	0.074635641	-13.39842454	1.126193701	15.08922131	-3.743991
Mns1	Mns1-001	683.60902	0.013609349	0.035102737	0.074687768	-13.38907326	0.704902084	9.437985646	-3.742984
Morf4l2	Morf4l2-201	503.24537	0.007255895	0.021436592	0.074715148	-13.38416679	0.570452514	7.635031589	-3.742455
Psmf1	Psmf1-005	667.90642	0.008402176	0.024009221	0.074836654	-13.362436	0.515592977	6.88957815	-3.740111
Enpp1	Enpp1-005	724.56327	1.68698E-05	0.000223517	0.074875207	-13.35555565	0.603857668	8.064854692	-3.739368
Usp14	Usp14-006	272.68521	0.014653863	0.037213227	0.074903189	-13.35056635	0.244435251	3.263349036	-3.738829
Herc1	Herc1-003	5823.172	1.59433E-08	3.19391E-06	0.074904518	-13.35032954	5.179623765	69.14968416	-3.738803
Chd8	Chd8-005	1081.7396	0.000360855	0.002079286	0.074948688	-13.34246177	0.970933285	12.95464023	-3.737953
Nfat5	Nfat5-201	17905.554	4.95716E-09	1.8062E-06	0.075035622	-13.32700349	14.13536176	188.3820156	-3.736281
Jazf1	Jazf1-003	434.09913	0.006806017	0.020356833	0.075105972	-13.31452052	0.235499164	3.135558455	-3.734929
10032A03R	0032A03Rik-	460.04507	0.018905688	0.045471898	0.075181074	-13.30121994	0.477633893	6.353113462	-3.733487
Tmco1	Tmco1-002	3092.9416	2.10767E-05	0.0002625	0.075223638	-13.2936937	2.678010317	35.60064889	-3.73267
Kif23	Kif23-201	1379	0.022392483	0.051869507	0.075238888	-13.29099918	1.50748581	20.03599266	-3.732378
Egfl7	Egfl7-017	936.19826	0.000269016	0.00166314	0.075324236	-13.27593958	0.835558944	11.09283005	-3.730742
Phf20	Phf20-004	1411.8448	0.005223493	0.016571382	0.075338346	-13.27345312	1.462085883	19.40692842	-3.730472
Cd300a	Cd300a-002	1582.2646	0.000184901	0.001258193	0.075367575	-13.2683053	2.235577887	29.66232994	-3.729912
Atf7ip	Atf7ip-006	4497.7304	1.08423E-07	9.9407E-06	0.075373013	-13.26734805	4.278996107	56.77093067	-3.729808
Extl1	Extl1-003	1563	2.10514E-08	3.6337E-06	0.075481589	-13.2482637	1.363263575	18.06087534	-3.727731
Stag1	Stag1-017	964.69346	0.000729166	0.003568814	0.07549073	-13.24665959	0.886136058	11.73834271	-3.727557
Rab13	Rab13-002	1082.6035	0.000212758	0.001396236	0.075510503	-13.24319078	1.092025727	14.46190504	-3.727179
Kantr	Kantr-001	831.29888	0.000170173	0.001183438	0.075543505	-13.23740537	0.707896987	9.37071937	-3.726548
Ung	Ung-003	636.40278	0.013213537	0.034304664	0.075549441	-13.23636526	0.780377577	10.32936265	-3.726435
Rpl24	Rpl24-002	5322.3451	1.42506E-05	0.000198006	0.075635016	-13.22138945	5.571493008	73.66287888	-3.724802
Usp24	Usp24-003	516.08331	7.82289E-05	0.000669166	0.075652302	-13.21836849	0.493661514	6.525399801	-3.724472
Cmpk1	Cmpk1-001	9168.1148	1.63695E-08	3.19391E-06	0.075690256	-13.21174018	7.575242596	100.082137	-3.723749
Farsb	Farsb-004	1199.0323	0.017313666	0.042353623	0.075941602	-13.16801302	1.281123329	16.86984868	-3.718966
Ica1	Ica1-004	850.11212	1.79865E-05	0.000233614	0.075977107	-13.16185935	0.770894773	10.14640858	-3.718291
Gm27754	Gm27754-201	122	0.000801983	0.003837911	0.076084716	-13.14324417	0.082306773	1.081778021	-3.71625
Serpinc1	Serpinc1-002	732	3.55542E-05	0.00037689	0.076288848	-13.10807585	0.644159249	8.443688301	-3.712384
Rap2c	Rap2c-001	9829	1.58689E-08	3.19391E-06	0.076488347	-13.07388698	10.27266321	134.3036378	-3.708616
Klhl13	Klhl13-005	407.61514	0.02870903	0.063010848	0.07649609	-13.07256365	0.396096058	5.177990936	-3.70847
33439L19R	3439L19Rik-	2942.1726	1.52306E-06	4.50862E-05	0.076542097	-13.06470601	2.485664006	32.47446949	-3.707603
Fam126b	Fam126b-009	919.24223	8.20261E-05	0.000692993	0.076565929	-13.06063948	0.643455136	8.403935558	-3.707154
Rnf44	Rnf44-003	2033.3337	0.000169061	0.001177616	0.07665383	-13.04566258	1.886186196	24.60654867	-3.705498
Pus7	Pus7-002	186.30084	0.031741575	0.068352654	0.076675131	-13.04203843	0.197189361	2.571751221	-3.705097
Slc37a3	Slc37a3-005	585.59292	0.04584233	0.09155101	0.076699791	-13.03784518	0.365581856	4.76639964	-3.704634
Oasl2	Oasl2-004	485.65242	0.004750191	0.015357186	0.076930454	-12.99875332	0.39783618	5.17137437	-3.700301
Tank	Tank-006	382.93943	0.041004658	0.083780322	0.076966762	-12.99262134	0.446233856	5.797747521	-3.699621
Dmxl1	Dmxl1-201	1329.6235	0.002586763	0.009568703	0.07698982	-12.98873024	1.442256474	18.73308027	-3.699188
Nrf1	Nrf1-001	1919.3513	9.76093E-05	0.000783849	0.077023389	-12.98306939	1.564450943	20.31137514	-3.69856
Hnrnpk	Hnrnpk-020	1376.5864	0.005310291	0.016803165	0.077042259	-12.97988933	1.083347579	14.06173168	-3.698206
Zbtb34	Zbtb34-201	2765.5815	0.000290202	0.001763562	0.07711056	-12.96839236	2.925078563	37.9335665	-3.696928
Tmod2	Tmod2-202	8771.8551	9.80828E-06	0.000152737	0.077142151	-12.96308162	9.691209669	125.6279419	-3.696337
Qars	Qars-003	994.81164	0.00772901	0.022500517	0.077145224	-12.96256529	1.099283423	14.24953315	-3.696279
Pydc3	Pydc3-001	365.15483	0.000128113	0.00096099	0.077177894	-12.95707808	0.338783092	4.389638972	-3.695669
Gm27592	Gm27592-201	189	0.000826066	0.003928457	0.077222052	-12.94966888	0.163637016	2.119045177	-3.694843
Zfp326	Zfp326-001	2204.2317	5.97499E-05	0.000549673	0.077264721	-12.94251743	1.983910472	25.67679586	-3.694046
Gm26983	Gm26983-001	447	0.000537167	0.002826212	0.077282681	-12.93950973	0.392008206	5.072393988	-3.693711
BC028528	BC028528-00	602.41819	0.04670515	0.092932749	0.077318626	-12.93349417	0.600719646	7.769404034	-3.69304
Ccnl1	Ccnl1-004	2603.7567	0.000458114	0.002499032	0.077369692	-12.92495768	2.48377752	32.10271932	-3.692088
Coro1c	Coro1c-010	851.2929	0.000266183	0.001649795	0.077455428	-12.91065101	0.782452208	10.10196739	-3.69049
Mov10	Mov10-006	575.00808	0.000106517	0.000834639	0.077489633	-12.90495203	0.52738876	6.805926648	-3.689853
10315B03R	0315B03Rik-	356.24379	0.001280672	0.005534765	0.077513429	-12.90099028	0.363697952	4.692063744	-3.68941
Pttg1	Pttg1-003	2251.3196	1.18379E-05	0.000173195	0.0775238	-12.89926442	1.980187565	25.54296301	-3.689217
Clec12b	Clec12b-202	93.417671	0.018228737	0.044141278	0.077562795	-12.89277939	0.109170552	1.407511845	-3.688491
E2f7	E2f7-007	250.87316	0.000232572	0.001492241	0.077651503	-12.87805078	0.236834199	3.049962837	-3.686842
Cdc14a	Cdc14a-004	2842.9241	2.6013E-05	0.000304045	0.077685242	-12.87245777	3.053493006	39.30595976	-3.686216
Zfp523	Zfp523-005	281.86168	0.043766915	0.088224167	0.077697757	-12.87038435	0.228660694	2.942951013	-3.685983
Gdi1	Gdi1-003	6905.6633	5.33329E-07	2.41301E-05	0.077756351	-12.86068575	5.57632808	71.7154031	-3.684896
Kansl1	Kansl1-003	5709.6232	8.73593E-09	2.42357E-06	0.077789761	-12.85516227	5.093334369	65.47563979	-3.684276
Bclaf1	Bclaf1-003	7646.7026	4.53313E-09	1.8062E-06	0.077806998	-12.85231439	6.981796835	89.7322479	-3.683956
Prpf40b	Prpf40b-001	90.837662	0.042613137	0.086391614	0.078039026	-12.81410153	0.094360205	1.209141248	-3.67966
Tor1aip2	Tor1aip2-007	469.99046	0.008760688	0.024816274	0.078148396	-12.79616789	0.573091894	7.333380097	-3.67764
Gm10330	Gm10330-201	636	0.00011638	0.000894328	0.078158076	-12.79458317	0.53593495	6.857064292	-3.677461
Zfp637	Zfp637-005	92.296523	0.033264553	0.070958579	0.078173685	-12.79202848	0.067343374	0.861458356	-3.677173
Dhx9	Dhx9-001	3129.6815	0.00017267	0.00119458	0.078206282	-12.7866966	3.240001219	41.42891259	-3.676572
Ndufab1	Ndufab1-005	354.52835	0.01557472	0.038990876	0.078217851	-12.7848054	0.38756037	4.954883918	-3.676358
30001P10R	0001P10Rik-	133	0.002476272	0.009256607	0.07822191	-12.78414197	0.099827119	1.276204062	-3.676283
Rel	Rel-001	5785	5.34574E-06	0.000101661	0.078240811	-12.78105362	7.268614466	92.9005512	-3.675935
Gm15764	Gm15764-001	417.51961	0.000366922	0.002102572	0.07825269	-12.77911346	0.354158337	4.525829569	-3.675716
Cd164	Cd164-004	888.71946	0.019246947	0.046079123	0.078253132	-12.77904123	0.678043818	8.664749902	-3.675708
Robo1	Robo1-001	635	0.000239593	0.001525262	0.078267767	-12.7766517	0.596183668	7.617231074	-3.675438
Rif1	Rif1-004	1652.4022	0.004077594	0.013615833	0.07829751	-12.77179829	2.01966802	25.79479256	-3.67489
Lrrc41	Lrrc41-002	278.34055	0.000129943	0.000971738	0.078324434	-12.76740785	0.267011068	3.409039211	-3.674394
Pex5	Pex5-004	719.77668	0.013177717	0.034244066	0.078361257	-12.76140838	0.561440563	7.164772308	-3.673716
10507B11R	0507B11Rik-	795.97266	0.006143461	0.018810436	0.078376088	-12.75899349	0.7938981	10.12934069	-3.673443
Lipo1	Lipo1-201	625.79683	0.000134547	0.000998105	0.078449617	-12.7470349	0.448108245	5.71205144	-3.67209
Prrg1	Prrg1-004	317.48666	0.021474427	0.050242443	0.078480362	-12.74204111	0.304931266	3.885446725	-3.671524
Lmbr1	Lmbr1-002	1193.8799	0.044245279	0.089031245	0.078537801	-12.73272217	0.909186271	11.57641619	-3.670469

Atrx	Atrx-013	106.61368	0.005050279	0.016127931	0.078615849	-12.72008147	0.055898574	0.711034409	-3.669036
Tcirg1	Tcirg1-008	199.5	0.024003723	0.054706382	0.078625991	-12.71844071	0.222174694	2.825715666	-3.66885
R3hdm1	R3hdm1-007	404.54979	0.045424642	0.09085459	0.078639309	-12.71628665	0.53425461	6.793734768	-3.668606
Krit1	Krit1-006	1015.9099	0.000145573	0.001056999	0.078662475	-12.71254183	0.785461907	9.985217351	-3.668181
N4bp2	N4bp2-008	598.86088	7.17045E-05	0.000628584	0.078867198	-12.6795427	0.6035285	7.652465389	-3.664431
Tmem33	Tmem33-004	1294.6576	0.002372059	0.00894747	0.078910085	-12.67265145	1.184932973	15.01624256	-3.663647
Sorbs2	Sorbs2-202	607.33683	0.020052925	0.047599465	0.07893244	-12.66906223	0.565303899	7.161870283	-3.663238
Gm26763	Gm26763-201	261	0.000508566	0.002705559	0.078939297	-12.66796179	0.286819101	3.633413409	-3.663113
Wsb1	Wsb1-005	3267.0268	1.83993E-06	5.08617E-05	0.078957092	-12.66510672	3.772991642	47.78534178	-3.662787
Blzf1	Blzf1-001	956.11916	0.00683456	0.02042615	0.079020101	-12.65500791	0.934235095	11.82275252	-3.661637
P24-140D11	P24-140D11.6-	453	0.000373184	0.002131327	0.079034941	-12.65263175	0.495916883	6.274653697	-3.661366
Tmprss11d	Tmprss11d-00	255.7005	0.000601239	0.003080396	0.079089598	-12.6438877	0.168834374	2.13472286	-3.660368
Rad54l2	Rad54l2-003	410.92061	0.000683901	0.003401132	0.079166179	-12.63165682	0.318739367	4.026206302	-3.658972
Mum1	Mum1-010	1181.8287	2.88612E-07	1.69341E-05	0.079197274	-12.62669726	0.999919686	12.62568315	-3.658405
Eif2a	Eif2a-004	503.0441	0.006027416	0.018521353	0.079306107	-12.60936949	0.449793164	5.671608201	-3.656424
Fam69a	Fam69a-002	977.30309	8.28847E-06	0.000136415	0.079330499	-12.60549243	0.706801462	8.909580484	-3.655981
Myadm	Myadm-202	14140.591	4.78539E-05	0.000466508	0.079358415	-12.60105819	12.3534931	155.6670854	-3.655473
Hsph1	Hsph1-007	3325.8627	0.00034245	0.001998454	0.079369871	-12.59923935	4.280135478	53.92645135	-3.655265
Ikbkg	Ikbkg-204	388.16068	0.001777706	0.007141309	0.079376969	-12.59811269	0.334674746	4.216270165	-3.655136
Smad5	Smad5-005	1323.7697	0.000476052	0.002576201	0.079399589	-12.59452362	1.219981265	15.36508286	-3.654725
Rnf214	Rnf214-009	305.59963	0.008431131	0.024073885	0.079420246	-12.59124775	0.271105378	3.413554984	-3.654349
Vcan	Vcan-004	4909.4088	0.006785118	0.020308512	0.079423116	-12.59079281	8.454275776	106.4460347	-3.654297
Gm37795	Gm37795-001	573	5.4348E-05	0.000512505	0.079539819	-12.57231933	0.571919203	7.190350858	-3.652179
Zfp532	Zfp532-002	2030.1685	0.000443325	0.002436161	0.079573637	-12.5669761	2.1050617	26.45426007	-3.651566
Gm16286	Gm16286-003	792.76597	0.000452083	0.00247425	0.079595513	-12.56352222	0.770965983	9.686048255	-3.651169
Fut10	Fut10-001	672.35503	0.015558297	0.038964001	0.079610361	-12.56117904	0.710473422	8.924383862	-3.6509
Dixdc1	Dixdc1-002	709.33935	0.020715387	0.04880999	0.07970611	-12.5460897	0.564298906	7.079744688	-3.649166
Hnrnpk	Hnrnpk-004	7665.0843	1.0905E-05	0.000164538	0.079852005	-12.52316709	7.968138835	99.786334	-3.646528
Ubr1	Ubr1-005	438.97027	0.033138624	0.070742824	0.079885735	-12.51787945	0.422753358	5.29197558	-3.645918
Rcbtb2	Rcbtb2-006	356.28249	0.030184772	0.065662847	0.080032774	-12.4948811	0.355455706	4.441376786	-3.643265
Gm12416	Gm12416-001	270	0.000433539	0.00239478	0.08004233	-12.49338946	0.328927094	4.109414288	-3.643093
Raet1e	Raet1e-201	1039.0418	0.023823837	0.054415297	0.080059548	-12.49070255	0.749007275	9.355627083	-3.642783
Apobec3	Apobec3-202	3971.4267	1.18059E-05	0.000172948	0.080084902	-12.48674808	4.304027584	53.74330815	-3.642326
Tfrc	Tfrc-001	817.41186	0.036272421	0.07604863	0.080131939	-12.47941847	1.332457007	16.62828859	-3.641479
Fbxo11	Fbxo11-002	3725.5835	0.000436226	0.002407468	0.080149994	-12.47660731	3.560070785	44.41760519	-3.641154
Tnpo3	Tnpo3-008	1120.9831	0.000147136	0.001064961	0.080153937	-12.47599355	1.024787438	12.78524146	-3.641083
Zfp62	Zfp62-202	490.17309	0.001839739	0.007331982	0.08023905	-12.4627597	0.532989413	6.642518982	-3.639552
Rab22a	Rab22a-002	4626.6901	0.005937713	0.018291658	0.080243928	-12.46200204	4.127011985	51.43083177	-3.639464
Adgre5	Adgre5-002	3853.934	0.000488815	0.002628445	0.080247187	-12.46149602	3.56373134	44.40942392	-3.639405
Gm37833	Gm37833-001	404	0.000597391	0.003066184	0.080264767	-12.45876662	0.477047373	5.94342188	-3.639089
Cers6	Cers6-001	3793.5362	0.000304333	0.00182867	0.080277546	-12.45678336	6.262284725	78.00792416	-3.63886
Capn7	Capn7-003	4412.5317	0.000310164	0.001852975	0.080380357	-12.44085039	3.735624729	46.47434838	-3.637013
Dnaja1	Dnaja1-001	14195.591	3.88957E-07	2.01715E-05	0.080392243	-12.43901103	14.88229634	185.1210484	-3.6368
Zbtb14	Zbtb14-001	2189.9495	2.60663E-05	0.000304388	0.080469263	-12.42710519	1.951923582	24.25675968	-3.635418
Glud1	Glud1-004	406.20842	0.015308963	0.038485906	0.080570063	-12.41155794	0.476274574	5.911309477	-3.633612
Uba3	Uba3-202	2123.167	3.35664E-07	1.84549E-05	0.080601043	-12.40678737	1.724012511	21.38945665	-3.633058
Spata13	Spata13-002	7100.5374	0.000379216	0.002160729	0.080625344	-12.40304787	7.945940784	98.55388389	-3.632623
Ccdc53	Ccdc53-002	178.9922	0.003774306	0.012825676	0.080652903	-12.39880978	0.095666816	1.186154654	-3.63213
Phf21a	Phf21a-204	520.3412	0.014921747	0.037739612	0.080671896	-12.39589067	0.609842427	7.559540054	-3.63179
Ppm1g	Ppm1g-005	652.70975	0.000569129	0.002957609	0.080686729	-12.39361189	0.638399374	7.912074073	-3.631525
Osbpl6	Osbpl6-001	414.99656	0.000497353	0.00265954	0.080728437	-12.38720876	0.416839572	5.163478793	-3.630779
Surf2	Surf2-001	1232.3427	0.00204907	0.007976888	0.080821059	-12.37301287	1.216460885	15.05128619	-3.629125
Hhat	Hhat-001	369.74947	0.000326295	0.001925834	0.080846841	-12.36906706	0.390307421	4.82773866	-3.628665
Lmbrd1	Lmbrd1-006	373.43561	0.000392257	0.002218107	0.08090013	-12.36091952	0.365973882	4.523773698	-3.627714
30519G04R	0519G04Rik-	640	3.93754E-06	8.32476E-05	0.080922434	-12.3575127	0.625019906	7.723691424	-3.627316
Senp7	Senp7-008	2422.1473	1.06843E-07	9.86351E-06	0.081041392	-12.33937338	2.252037112	27.78872678	-3.625197
Pik3ca	Pik3ca-003	446.93763	0.048332301	0.095532163	0.081053514	-12.33752806	0.382042075	4.713454819	-3.624981
Tmem41b	Tmem41b-003	1434.9503	0.007119408	0.021109836	0.081121375	-12.32720724	1.177898459	14.52019842	-3.623774
Arpp19	Arpp19-201	2916.0103	2.26743E-05	0.000275681	0.08116638	-12.32037207	3.032182244	37.35761342	-3.622974
00049A03R	0049A03Rik-	1442.326	0.027539551	0.061026761	0.081254664	-12.30698588	1.665950651	20.50283113	-3.621406
Gm2065	Gm2065-001	273	0.000989064	0.004511913	0.081336737	-12.29456741	0.266466362	3.276088646	-3.619949
Gm15384	Gm15384-001	306	0.001486789	0.006215371	0.081368494	-12.28976902	0.304676311	3.744401489	-3.619386
Ctage5	Ctage5-015	2730.4662	6.2783E-06	0.000113294	0.081371571	-12.28930441	2.692152767	33.08468487	-3.619331
Gm38014	Gm38014-001	479	0.004637657	0.015085931	0.081388566	-12.28673811	0.545092179	6.697404844	-3.61903
Dhx15	Dhx15-001	11446.623	2.70876E-07	1.63625E-05	0.081402262	-12.28467085	11.73975941	144.2190802	-3.618787
Mast2	Mast2-002	1439.5908	0.002353276	0.008891308	0.081623053	-12.2514408	0.912316297	11.1771891	-3.61488
Cep162	Cep162-002	504.88474	0.017524944	0.04278185	0.081640896	-12.24876319	0.592892388	7.262198456	-3.614564
B4galt7	B4galt7-001	293.53933	0.020308714	0.048040157	0.081693363	-12.24089644	0.294464143	3.604505085	-3.613637
Ttc3	Ttc3-007	997.75442	0.049570745	0.097328481	0.081698199	-12.24017189	0.804360364	9.845509116	-3.613552
Gm24081	Gm24081-201	149	0.002080236	0.008075266	0.081746097	-12.233	0.149825595	1.832816503	-3.612706
Igsf6	Igsf6-201	2522	0.013947591	0.035785826	0.081785914	-12.22704443	4.242174372	51.8692545	-3.612004
Stat3	Stat3-009	66.880511	0.022239057	0.051580396	0.081993681	-12.19606173	0.031996634	0.390232922	-3.608343
Aebp2	Aebp2-001	4204.0906	4.64639E-09	1.8062E-06	0.082005217	-12.19434619	3.901277179	47.57352451	-3.608141
Nab1	Nab1-003	5238.3112	1.46206E-08	3.14941E-06	0.082103964	-12.17967988	5.337404092	65.00787322	-3.606404
Gm37609	Gm37609-001	769	0.009894555	0.027338537	0.082110894	-12.17865203	0.567333748	6.909360307	-3.606283
Gm15738	Gm15738-001	265	0.002643211	0.009734937	0.082184241	-12.16778288	0.270175286	3.287434219	-3.604994
Aip	Aip-001	414.37408	0.003961949	0.013321888	0.082300368	-12.15061399	0.299300082	3.636679765	-3.602957
Sorl1	Sorl1-001	5849.1627	0.006709627	0.020124769	0.082324217	-12.14709401	10.33346332	125.5215504	-3.602539
Wdr83os	Wdr83os-001	1126.9573	0.012779666	0.033386999	0.082337773	-12.1450941	0.854380496	10.37653152	-3.602302
N6amt1	N6amt1-001	795.32101	0.026762644	0.059609843	0.082430277	-12.13146469	0.997791933	12.1046776	-3.600682
Golga1	Golga1-006	1562.8355	0.000617784	0.003147292	0.082459393	-12.12718125	1.7123337	20.76578114	-3.600172
Ebf1	Ebf1-002	1291.8998	0.013659062	0.035195822	0.082484257	-12.12352553	1.455413057	17.64473735	-3.599737
Zfp800	Zfp800-005	2381.3645	0.000180187	0.001233045	0.082557104	-12.11282795	2.069499249	25.06748834	-3.598464
Sh3kbp1	Sh3kbp1-002	1372.1507	0.02420188	0.055070049	0.082601308	-12.10634579	1.658637045	20.08003361	-3.597692
Irf4	Irf4-001	234.97366	0.00639863	0.019383785	0.082606433	-12.10559473	0.394596601	4.77682653	-3.597602
Txlng	Txlng-006	2638.3407	1.61874E-06	4.68656E-05	0.082673856	-12.09572226	2.443198162	29.55224639	-3.596425
Gabpb1	Gabpb1-002	282.13973	0.000223569	0.001449037	0.082682614	-12.0944411	0.27614841	3.339860684	-3.596272
Pkig	Pkig-201	3170.5326	2.37721E-06	6.06557E-05	0.082814477	-12.07518349	2.674621028	32.29653969	-3.593973
Gm13477	Gm13477-001	229.88109	0.000931815	0.004312795	0.082906803	-12.06173641	0.220682891	2.661818864	-3.592366
Atp6v1a	Atp6v1a-001	6034.0046	4.19205E-05	0.000424535	0.082910735	-12.06116432	5.454346097	65.78576456	-3.592297
Kdm5b	Kdm5b-005	527.14034	0.000149246	0.00107614	0.082993701	-12.04910714	0.477278916	5.750784794	-3.590854
Ambra1	Ambra1-007	1685.499	0.016049409	0.039892527	0.082998313	-12.04843769	1.552125266	18.70068455	-3.590774
Fbxo3	Fbxo3-003	1018.0523	0.000923997	0.004287036	0.083147612	-12.02680365	0.869086637	10.45233433	-3.588181
Tmem33	Tmem33-003	844.50903	2.16616E-05	0.000266994	0.083183607	-12.02159938	0.864415883	10.39166145	-3.587557
Baz2b	Baz2b-002	1020.4766	6.72557E-05	0.000600028	0.083263439	-12.01007324	1.101121924	13.22455495	-3.586173
Pcdh7	Pcdh7-005	5682.7532	9.82098E-05	0.000787011	0.083330711	-12.0003776	4.790470822	57.48745874	-3.585008
Slx1b	Slx1b-001	7019.7545	1.12493E-08	2.81232E-06	0.08351156	-11.97439019	6.837153688	81.87074608	-3.58188
Fn1	Fn1-011	16899.399	4.78967E-09	1.8062E-06	0.083549732	-11.96891926	17.04043382	203.9555765	-3.581221
Cdc14a	Cdc14a-002	2414.5838	1.05369E-05	0.000160253	0.083568146	-11.96628205	2.77525752	33.20951424	-3.580903
Exo1	Exo1-001	983.14223	7.36777E-06	0.000125608	0.083601037	-11.9615741	1.175003142	14.05488715	-3.580335
Adam17	Adam17-003	5018.1021	6.19884E-05	0.000564023	0.083630974	-11.95729222	3.534961141	42.26856334	-3.579819
Glce	Glce-002	353.94231	0.000814638	0.003883819	0.083680158	-11.95026425	0.297134618	3.550837199	-3.578971
Wdr13	Wdr13-002	1552.786	6.29236E-05	0.000570897	0.083692103	-11.94855868	1.322372879	15.80044995	-3.578765
Ybx1	Ybx1-007	4296.4595	3.22966E-06	7.39401E-05	0.083713801	-11.94546172	3.56844158	42.6266823	-3.578391
Tapt1	Tapt1-002	3349.4919	0.000546167	0.002864658	0.08374032	-11.94167872	2.980514159	35.5923425	-3.577934
P23-336F4.	23-336F4.1-0	286	0.001898303	0.007513917	0.083799707	-11.93321593	0.222469538	2.65477703	-3.576911
Ube2j2	Ube2j2-202	231.46415	0.028797371	0.063156174	0.083897642	-11.9192861	0.233369174	2.781593949	-3.575226

Orc4	Orc4-003	804.91906	0.016852537	0.041504235	0.083899811	-11.91897796	0.816015619	9.726072182	-3.575189
Gm37226	Gm37226-001	623	0.001063296	0.004772988	0.084050725	-11.89757731	0.734983586	8.744524028	-3.572596
P23-327P24	23-327P24.3-	276	0.005396269	0.017023322	0.084058698	-11.89644884	0.201510101	2.397254604	-3.572459
Pfkfb3	Pfkfb3-011	1863.2187	7.34862E-05	0.000640834	0.084093169	-11.89157228	2.257022571	26.83954704	-3.571868
Wdr75	Wdr75-002	1109.8323	0.009090517	0.025579215	0.084124504	-11.88714284	1.418327188	16.85985788	-3.57133
Fip1l1	Fip1l1-007	280.46767	0.049094003	0.096684281	0.084186794	-11.87834752	0.270734096	3.215873672	-3.570262
Pgk1	Pgk1-003	677.72556	0.005139139	0.016352241	0.084259312	-11.86812443	0.917997253	10.89490563	-3.56902
Alcam	Alcam-001	2004.8412	9.99046E-05	0.000797049	0.084325759	-11.85877257	2.477756101	29.38314609	-3.567883
Traf7	Traf7-010	641.01831	0.002458718	0.009202391	0.08432925	-11.85828172	0.662468226	7.855734856	-3.567823
Gm37553	Gm37553-001	2712	2.33313E-08	3.84182E-06	0.084355402	-11.85460535	2.808633924	33.29524675	-3.567376
Gm20517	Gm20517-003	204.19864	0.00074649	0.003631616	0.084391846	-11.84948606	0.177301386	2.100930303	-3.566753
Pdcd4	Pdcd4-202	924.41251	0.011147578	0.029997215	0.084474862	-11.83784112	0.631215288	7.472226296	-3.565334
Pdgfrb	Pdgfrb-002	1316.7614	0.00233614	0.00883631	0.08457156	-11.82430599	1.329004652	15.71455767	-3.563684
Mef2c	Mef2c-014	624.2408	0.001055427	0.004748865	0.084593368	-11.82125765	0.65947371	7.795808642	-3.563312
Ano1	Ano1-001	100.49434	0.044429721	0.089307938	0.084611993	-11.81865556	0.14664515	1.733148522	-3.562994
U2surp	U2surp-001	4887.6772	0.000154303	0.001103054	0.084653092	-11.81291758	5.102239014	60.27232892	-3.562293
Rexo1	Rexo1-007	689.0068	0.037864208	0.078654119	0.084671642	-11.8103296	0.647829919	7.651084863	-3.561977
Slc20a1	Slc20a1-001	2562.4994	0.003855687	0.013039229	0.084971623	-11.76863474	2.546655807	29.970662	-3.556875
P23-479D21	23-479D21.3-	762	1.40197E-05	0.000196115	0.085000008	-11.76470481	0.809449327	9.522932393	-3.556393
Ssbp2	Ssbp2-002	336.21647	0.006874606	0.020515399	0.085030897	-11.76043108	0.306563904	3.605323667	-3.555869
Dab2ip	Dab2ip-002	463.69998	3.83692E-05	0.00039767	0.085075345	-11.7542868	0.334224567	3.928571414	-3.555115
Pcyt1a	Pcyt1a-201	1084.6206	1.55439E-05	0.000210759	0.085111714	-11.74926399	1.077846486	12.6639029	-3.554498
33431K23R	3431K23Rik-	163	0.003249045	0.011434423	0.085259599	-11.72888456	0.117347432	1.376354489	-3.551994
Ticam2	Ticam2-002	880.7933	5.16194E-06	9.96068E-05	0.085403365	-11.70914054	0.901528009	10.55611816	-3.549563
Tsku	Tsku-203	81.842276	0.024786001	0.056090848	0.085403819	-11.70907831	0.099571142	1.165886304	-3.549556
Slc39a10	Slc39a10-001	4853.3978	1.00885E-07	9.59812E-06	0.085413697	-11.70772411	4.474171273	52.38236286	-3.549389
Stx5a	Stx5a-006	1467.9277	0.011024463	0.029750068	0.085439776	-11.70415054	1.224303131	14.32942815	-3.548948
Cp	Cp-005	1793.5171	0.002569964	0.009519929	0.085469226	-11.70011767	2.160317045	25.27596362	-3.548451
Zfp143	Zfp143-202	704.49087	0.00871806	0.024730294	0.085492479	-11.69693531	0.485187526	5.6752071	-3.548059
Eif5	Eif5-202	29766.872	4.39789E-10	5.19406E-07	0.085554284	-11.68848534	33.63079322	393.0930337	-3.547016
Kcnmb4	Kcnmb4-004	202	0.000591231	0.003040492	0.085638294	-11.67701913	0.199217373	2.326265078	-3.5456
Cr1l	Cr1l-001	10029.162	6.03589E-07	2.58089E-05	0.085775181	-11.65838409	9.231672015	107.6263782	-3.543296
Zfp345	Zfp345-001	526	2.66317E-05	0.000308467	0.085815097	-11.65296119	0.539913707	6.291593474	-3.542625
Unc50	Unc50-002	3800.1195	3.20979E-09	1.51021E-06	0.085822332	-11.65197892	3.463916203	40.36147857	-3.542503
Gls	Gls-003	4135.2348	0.000344263	0.002005956	0.086023141	-11.624779	4.350266376	50.57088521	-3.539131
Acp2	Acp2-002	1201.0776	3.83737E-05	0.00039767	0.086053649	-11.62065767	1.290856812	15.00060512	-3.53862
30601H04R	0601H04Rik-	531.55517	0.000506912	0.002699274	0.086081164	-11.61694327	0.266876658	3.100290992	-3.538159
Dek	Dek-006	9824.9142	7.49252E-07	2.94288E-05	0.086201004	-11.60079296	10.81087364	125.4147068	-3.536152
Gbp6	Gbp6-001	6232.3899	0.00641681	0.019430267	0.086225772	-11.59746062	4.151464888	48.14645055	-3.535737
Efcab7	Efcab7-201	473.15817	0.000214542	0.001404717	0.086225935	-11.59743875	0.543043025	6.297908216	-3.535734
Rab11fip2	Rab11fip2-002	1391.6862	3.15324E-05	0.000347151	0.086232125	-11.59660629	1.453010636	16.84999228	-3.535631
Mllt4	Mllt4-001	2097.3645	4.49893E-05	0.000447282	0.086272091	-11.59123403	1.974066966	22.8818722	-3.534962
Mycbp2	Mycbp2-003	1476.4147	1.40513E-05	0.000196427	0.086287827	-11.58912026	2.017907947	23.38577788	-3.534699
Rhot1	Rhot1-003	953.97989	0.033435817	0.071270645	0.086309305	-11.58623629	0.788327682	9.133750796	-3.53434
Stxbp5	Stxbp5-006	807.55307	2.32565E-05	0.000280865	0.086319522	-11.58486491	0.69777014	8.083572806	-3.534169
Gm12088	Gm12088-001	271	0.000330392	0.001945825	0.086323593	-11.58431854	0.264780332	3.06729971	-3.534101
Gm36992	Gm36992-001	296	0.001018821	0.004619968	0.086341018	-11.58198071	0.357426467	4.139706447	-3.53381
Phb	Phb-003	307.0102	0.000504348	0.002690139	0.086381425	-11.57656293	0.350274587	4.054975798	-3.533135
Fbxw5	Fbxw5-008	722.47035	0.02436607	0.055371194	0.086384845	-11.57610455	0.832576572	9.637993442	-3.533078
Ankrd17	Ankrd17-007	1994.4685	2.60086E-07	1.60504E-05	0.086467895	-11.56498607	1.993625922	23.05625602	-3.531692
Fam168b	Fam168b-201	5673.5748	4.63185E-05	0.000455603	0.086520249	-11.55798795	6.10836245	70.60037957	-3.530818
Mavs	Mavs-001	567.85908	0.003598651	0.012368669	0.086532735	-11.55632027	0.529382447	6.1177131	-3.53061
Odf2l	Odf2l-201	191.25584	0.029659759	0.06471598	0.086541798	-11.55511005	0.223021563	2.577038708	-3.530459
Pfdn5	Pfdn5-002	212.47355	0.011817739	0.031442363	0.086589339	-11.54876587	0.212591056	2.455164334	-3.529667
Ktn1	Ktn1-019	390.36759	0.000474203	0.002568224	0.08663253	-11.54300812	0.406664912	4.694136381	-3.528947
Gm5577	Gm5577-003	612.67488	0.005907344	0.018229257	0.086783045	-11.52298822	0.607901052	7.004836656	-3.526443
Gimap4	Gimap4-006	2836.7366	0.000151064	0.001085824	0.086804604	-11.52012624	2.532938806	29.1797748	-3.526085
Nisch	Nisch-007	282.13405	0.006690662	0.020078434	0.086843524	-11.51496334	0.366163628	4.21636075	-3.525438
Tubb5	Tubb5-003	184.67817	0.022074735	0.051293057	0.086869676	-11.51149684	0.080757098	0.929635076	-3.525004
Gm14636	Gm14636-001	1388.4055	4.36072E-05	0.000437349	0.086897475	-11.50781428	1.789096702	20.58859257	-3.524542
Tmem230	Tmem230-001	692.42653	0.012129554	0.032070189	0.086960024	-11.49953682	0.841209804	9.673523113	-3.523504
Gm37718	Gm37718-001	883	2.85804E-06	6.84531E-05	0.086987355	-11.49592377	0.988213961	11.36043237	-3.52305
Tmem8b	Tmem8b-003	385.65615	0.028103585	0.061983887	0.086988892	-11.49572068	0.25567564	2.939175746	-3.523025
Kcnrg	Kcnrg-201	571	5.56356E-05	0.000520918	0.087179224	-11.47062289	0.672105275	7.709466155	-3.519872
Gm26561	Gm26561-201	900.05597	0.000124092	0.000939881	0.087304076	-11.45421893	0.921583748	10.55602201	-3.517807
Ikbkg	Ikbkg-202	1536.9312	0.000179375	0.001228854	0.08737636	-11.44474315	1.154119517	13.20860144	-3.516613
Eif1a	Eif1a-201	6840.916	1.40815E-05	0.000196693	0.087398012	-11.44190791	8.877328596	101.5735763	-3.516256
Ndufaf1	Ndufaf1-001	184.45363	0.035097773	0.074061901	0.087504282	-11.42801221	0.149457698	1.708004393	-3.514503
Usp7	Usp7-012	395.5143	2.48469E-05	0.00029416	0.087507087	-11.42764587	0.444424417	5.078724852	-3.514456
Mb21d1	Mb21d1-001	661.4996	0.000778204	0.003746927	0.087539827	-11.42337188	0.946679005	10.81426632	-3.513917
Pygo2	Pygo2-002	1404.2156	0.004572069	0.014920757	0.087541462	-11.42315861	1.344617036	15.35977366	-3.51389
Tcea1	Tcea1-002	1140.4947	0.000771747	0.003725194	0.087580167	-11.41811021	1.318692529	15.05697663	-3.513252
Cts8	Cts8-201	348	5.68848E-05	0.000529719	0.087634331	-11.41105302	0.33708741	3.846522306	-3.51236
Rint1	Rint1-002	410.64275	0.002455758	0.009194328	0.087689023	-11.40393598	0.282122087	3.217302214	-3.51146
Hsp90ab1	Hsp90ab1-004	558.66483	4.92815E-05	0.000475463	0.087695597	-11.40308104	0.690140699	7.869730317	-3.511352
Aff4	Aff4-003	1367.6286	0.006560552	0.019762436	0.087723722	-11.39942516	1.361847161	15.52427479	-3.510889
N4bp2l2	N4bp2l2-007	4362.9936	8.09303E-09	2.3293E-06	0.087746663	-11.3964448	4.377265715	49.88526709	-3.510512
Mrpl38	Mrpl38-002	695.20856	0.001602835	0.006581595	0.087836633	-11.3847715	0.390647378	4.447431136	-3.509033
Gm22398	Gm22398-201	101	0.001809616	0.007233816	0.087874539	-11.37986054	0.099827119	1.136018692	-3.508411
Gm12915	Gm12915-001	1562	2.3949E-05	0.000285802	0.08789865	-11.37673902	2.096813383	23.85489863	-3.508015
Pds5b	Pds5b-002	399.91618	0.000277699	0.001702987	0.087909238	-11.37536877	0.394380223	4.486220469	-3.507841
Agfg2	Agfg2-002	171.86161	0.026107377	0.058454446	0.087945795	-11.37064027	0.15445095	1.756206195	-3.507242
P23-114F4.	23-114F4.4-	546	3.34376E-05	0.000361046	0.087971056	-11.36737517	0.733703158	8.340279059	-3.506827
Ing4	Ing4-002	1076.0377	5.00875E-05	0.000481205	0.088014876	-11.36171575	0.862087718	9.794795601	-3.506109
Adcy7	Adcy7-202	523.25617	0.010542191	0.028749905	0.088033383	-11.35932716	0.766826366	8.710631569	-3.505805
Dtl	Dtl-003	203.09715	0.041267682	0.084232306	0.088060228	-11.35586436	0.310795294	3.529349203	-3.505366
Tex9	Tex9-008	517.9413	0.000203723	0.001350659	0.088103848	-11.35024209	0.477593565	5.420802582	-3.504651
Rnf19b	Rnf19b-001	1399.8399	0.000951492	0.004380777	0.088113554	-11.34899175	1.853128351	21.03113837	-3.504492
Fuca2	Fuca2-001	2322.8886	7.46352E-06	0.000126673	0.088124677	-11.34755937	2.468836288	28.01526635	-3.50431
Lztr1	Lztr1-003	884.52616	0.000831898	0.003947232	0.088146512	-11.34474837	0.779421025	8.8423354	-3.503953
Hdac7	Hdac7-010	6230.7752	6.83959E-07	2.77884E-05	0.088156972	-11.34340226	5.361439982	60.81697041	-3.503782
Fanca	Fanca-005	137.91268	0.012906986	0.033658999	0.08818436	-11.3398793	0.17564642	1.991809202	-3.503333
Slc35b4	Slc35b4-002	1801.1662	0.000201259	0.001340031	0.088227288	-11.33436176	1.720207132	19.49744994	-3.502631
Ndufs1	Ndufs1-004	683.03571	0.000452215	0.002474573	0.088305837	-11.32427968	0.633553375	7.174535608	-3.501347
Gm23968	Gm23968-201	116	0.002751339	0.010046384	0.088328691	-11.32134976	0.098410922	1.114144471	-3.500974
BC017643	BC017643-001	1014.4984	0.014196851	0.036300273	0.088338318	-11.32011598	1.086997443	12.30493712	-3.500817
Gm26891	Gm26891-201	116.41542	0.003670156	0.012554037	0.088359735	-11.31737208	0.093759548	1.061111692	-3.500467
Fnbp1	Fnbp1-002	1722.2164	0.000140456	0.001031801	0.08838293	-11.31440197	1.988768678	22.50172824	-3.500088
Ninj1	Ninj1-004	1183.3135	0.015557434	0.038964001	0.088449976	-11.3058256	1.07232888	12.1235633	-3.498994
Fbxo43	Fbxo43-201	389	5.37949E-05	0.00050855	0.088530665	-11.29552121	0.349549666	3.948345667	-3.497679
Morc3	Morc3-006	289.90383	0.000958344	0.004402392	0.08855	-11.29305473	0.327604646	3.699657201	-3.497364
P23-422N18	23-422N18.1-	1073	4.52341E-05	0.000448659	0.088557805	-11.29205948	1.117194896	12.61543122	-3.497237
G3bp2	G3bp2-201	6310.6288	1.44687E-06	4.3932E-05	0.088590091	-11.28794417	7.088830946	80.01832793	-3.496711
P23-403P24	23-403P24.3-	466	1.70979E-05	0.000225666	0.088717268	-11.27176276	0.479395156	5.403628469	-3.494641
Mef2d	Mef2d-007	343.98191	0.001668646	0.006784431	0.088751417	-11.26742572	0.483334067	5.445930696	-3.494086
Plekha1	Plekha1-004	562.4617	0.023971706	0.054647958	0.088805685	-11.26054035	0.411857898	4.637742475	-3.493204
Gm37760	Gm37760-001	2047	2.80433E-06	6.75571E-05	0.088855453	-11.25423336	2.247118313	25.28959388	-3.492396

Afmid	Afmid-001	169.18273	0.033522799	0.07143827	0.088933286	-11.24438374	0.103708325	1.166136205	-3.491133
Ube2v2	Ube2v2-001	2782.1484	5.18289E-07	2.36686E-05	0.089027164	-11.23252672	3.254543468	36.55674648	-3.489611
Cd247	Cd247-002	1573.9312	0.015061241	0.037980231	0.089030923	-11.23205252	1.705749629	19.15906942	-3.48955
Adgrl2	Adgrl2-018	238.02299	0.022655371	0.052329812	0.089088197	-11.22483145	0.210196261	2.359417601	-3.488622
Klhdc10	Klhdc10-004	577.2975	0.001321364	0.00566548	0.089132975	-11.21919243	0.775784843	8.703679434	-3.487897
Mocs2	Mocs2-201	560.9149	0.043397244	0.087644061	0.089149378	-11.21712813	0.666109541	7.471836073	-3.487631
Zfp207	Zfp207-005	1461.1917	7.63521E-05	0.000658311	0.08918495	-11.21265411	1.625937049	18.23106974	-3.487056
Ubr1	Ubr1-007	837.96543	0.024478861	0.055552675	0.08918669	-11.21243534	0.761743875	8.541003949	-3.487028
Vcan	Vcan-001	5517.937	0.002237651	0.008558295	0.08927263	-11.20164154	10.05298314	112.6099135	-3.485638
Snrpa1	Snrpa1-002	531.20799	0.015377491	0.038624162	0.089327439	-11.1947685	0.641122574	7.177218793	-3.484753
Pmp22	Pmp22-002	1038.0473	0.009220502	0.025864225	0.089349219	-11.19203969	0.521175975	5.833022202	-3.484401
Gsap	Gsap-002	598.6445	0.012512044	0.032848937	0.089359403	-11.1907641	0.87509996	9.793037219	-3.484237
Scoc	Scoc-201	1505.1092	0.00019778	0.00132131	0.089418358	-11.1833859	1.426428242	15.95229749	-3.483285
Rpl31-ps14	pl31-ps14-00	1566	1.08181E-07	9.9407E-06	0.089423124	-11.18278986	1.834412097	20.51384501	-3.483208
Lpp	Lpp-203	2742.5637	0.023103544	0.05313217	0.089423681	-11.18272012	2.87481919	32.14829841	-3.483199
Dhx30	Dhx30-020	483.24899	2.51101E-05	0.000296457	0.089437324	-11.18101427	0.493359105	5.516255195	-3.482979
Dpp6	Dpp6-008	215.9231	0.000499358	0.002669007	0.089516477	-11.17112778	0.202656464	2.26390126	-3.481703
l7Rn6	l7Rn6-002	179.3292	0.011732249	0.031275184	0.089532056	-11.16918393	0.182778874	2.041490858	-3.481452
Pfkfb3	Pfkfb3-020	1933.5279	0.041761567	0.085017455	0.089561124	-11.16555887	1.893844073	21.14582749	-3.480984
Cflar	Cflar-005	2410.767	4.35319E-05	0.000436764	0.089590912	-11.16184644	2.897737637	32.34410253	-3.480504
Mphosph9	Mphosph9-00	1534.3019	0.000100447	0.000799433	0.089648379	-11.15469135	1.719298646	19.17824573	-3.479579
Dock11	Dock11-002	744.96869	1.1009E-05	0.000165158	0.089659933	-11.15325388	0.907441668	10.1209273	-3.479393
Stag1	Stag1-004	7485.9635	1.28212E-05	0.000183812	0.089665753	-11.15252995	7.633082093	85.12817662	-3.479299
Mccc1	Mccc1-003	525.5378	0.010919045	0.02953074	0.089730235	-11.14451551	0.331960245	3.699536104	-3.478262
Tatdn1	Tatdn1-201	995	2.37466E-05	0.000284181	0.089765966	-11.14007951	1.194613414	13.30808841	-3.477688
Plod1	Plod1-007	145.35974	0.005045843	0.016118274	0.089870471	-11.12712539	0.116880312	1.30054189	-3.476009
Srsf3	Srsf3-003	12395.134	1.40154E-09	1.07321E-06	0.089920357	-11.1209523	14.02762404	156.0005379	-3.475208
Ints7	Ints7-002	2552.2452	2.08233E-05	0.000260101	0.089928467	-11.11994934	2.591938555	28.82222542	-3.475078
Gm13213	Gm13213-001	90	0.018881761	0.045420726	0.08993833	-11.1187298	0.082846439	0.921147175	-3.47492
Zfp260	Zfp260-007	1583.0714	7.9382E-06	0.000132353	0.089989486	-11.11240925	1.786392743	19.85112724	-3.4741
Arhgap23	Arhgap23-001	7954.4691	0.001156889	0.005108186	0.090004883	-11.11050833	6.506322609	72.28855154	-3.473853
Ctps2	Ctps2-001	1361.8549	0.027640238	0.061182585	0.090062249	-11.1034314	1.215228038	13.49320115	-3.472934
Unk	Unk-003	1425.5923	0.001710174	0.006912954	0.090070328	-11.10243544	1.493558909	16.58214137	-3.472804
Mpp1	Mpp1-001	4742.9053	3.19843E-06	7.37188E-05	0.090089039	-11.10012954	5.299443074	58.82450461	-3.472505
Gm13716	Gm13716-001	182	0.002070074	0.008042201	0.090093311	-11.09960317	0.11788713	1.308500368	-3.472436
Ubap2l	Ubap2l-011	504	3.1822E-05	0.000349328	0.090172247	-11.08988672	0.484250444	5.370282568	-3.471173
Gm22002	Gm22002-201	127	0.001997671	0.007828402	0.090270268	-11.07784464	0.131158886	1.452957759	-3.469605
Nfia	Nfia-003	27888.142	6.25942E-06	0.000113138	0.090281102	-11.07651519	27.27605076	302.1235906	-3.469432
Gtf2a1	Gtf2a1-003	250.46296	0.024833292	0.056170933	0.090334757	-11.06993621	0.206491674	2.285849656	-3.468575
H2-DMa	H2-DMa-001	679.64233	0.042214177	0.085791635	0.090427267	-11.05861137	1.098233306	12.14493532	-3.467098
Gm16575	Gm16575-001	201.43313	0.002244104	0.00857596	0.090453513	-11.05540254	0.237460561	2.625222087	-3.46668
Gm12854	Gm12854-001	4718	2.43594E-06	6.17134E-05	0.090458625	-11.05477781	5.217595913	57.67936354	-3.466598
Specc1	Specc1-201	5387.3414	0.000702315	0.003474043	0.090461297	-11.05445127	2.920512022	32.28465785	-3.466556
Zbtb20	Zbtb20-015	2673.3952	3.72229E-06	8.05481E-05	0.090470545	-11.0533213	2.340500128	25.87029991	-3.466408
Ly6e	Ly6e-004	1142.8032	0.000180595	0.001234853	0.090595997	-11.03801528	1.307552775	14.43278752	-3.464409
Arrdc3	Arrdc3-002	3273.1445	0.000118918	0.000910151	0.090652611	-11.0311219	3.967247707	43.76319307	-3.463508
Brd8	Brd8-007	921.60653	2.98008E-05	0.000334117	0.090667405	-11.02932197	1.084316295	11.95927354	-3.463272
Tpd52l2	Tpd52l2-001	561.93215	0.008652761	0.024567444	0.090698931	-11.02548829	0.597520859	6.587959234	-3.462771
Zfp871	Zfp871-001	24570.372	2.78313E-09	1.48816E-06	0.090770855	-11.01675208	24.75610097	272.7318267	-3.461627
Rps10	Rps10-003	111.85502	0.01270316	0.033235389	0.090784191	-11.01513371	0.108723737	1.197606499	-3.461415
Heatr3	Heatr3-002	1551.3144	0.020812797	0.048985589	0.090856324	-11.00638854	1.880890623	20.701813	-3.460269
Gm8935	Gm8935-002	124	0.00116862	0.005149929	0.090884877	-11.0029307	0.135137643	1.486910121	-3.459816
Rchy1	Rchy1-004	503.51776	0.004428931	0.014545103	0.090899008	-11.00122017	0.537122586	5.909003825	-3.459592
Hnrnpl	Hnrnpl-010	366.54034	0.001865112	0.007408104	0.09091947	-10.9987443	0.430654736	4.736661327	-3.459267
P24-196M2	24-196M24.2-	1296	4.62053E-05	0.000454881	0.090922835	-10.99833724	1.011858047	11.12875603	-3.459214
Gnl3l	Gnl3l-007	465.73501	0.034558613	0.073208343	0.090928181	-10.99769061	0.464382459	5.107134614	-3.459129
Vezt	Vezt-010	4022.0809	5.94019E-07	2.54955E-05	0.09110922	-10.9758376	4.152321975	45.57521165	-3.456259
430087J23R	0087J23Rik-0	478	0.000190067	0.001281708	0.091161224	-10.96957631	0.599352926	6.57464766	-3.455436
Parp9	Parp9-003	2780.0142	0.010510207	0.028682773	0.091209916	-10.9637202	2.532103621	27.76127562	-3.454666
Mtus1	Mtus1-003	1801.8593	0.002166236	0.008330741	0.09121893	-10.96263678	1.924391392	21.09640385	-3.454523
Ssbp1	Ssbp1-001	1917.7723	1.39718E-07	1.15309E-05	0.091226793	-10.96169197	2.01908146	22.13254902	-3.454399
Swt1	Swt1-010	463.49755	0.000559618	0.002919558	0.091276754	-10.95569192	0.384456966	4.21199207	-3.453609
H2-Q10	H2-Q10-002	4966.6207	2.64598E-07	1.61538E-05	0.091309567	-10.95175495	5.978822117	65.47859472	-3.45309
Hmga2	Hmga2-002	246.91695	0.015906131	0.039629373	0.091359172	-10.94580845	0.324708463	3.554196637	-3.452307
P23-179A12	23-179A12.1-	789	9.66701E-06	0.000151047	0.091381575	-10.94312498	0.918199611	10.0479731	-3.451953
Pts	Pts-201	1432	5.16754E-06	9.96556E-05	0.09140222	-10.94065334	1.700048184	18.59963783	-3.451627
Ilk	Ilk-201	5882.064	1.21266E-05	0.000176898	0.091410548	-10.93965652	5.47590262	59.90449377	-3.451496
Lrrc3b	Lrrc3b-202	1282.6492	0.000436719	0.002409191	0.091430188	-10.93730662	0.721656378	7.892977075	-3.451186
Cobll1	Cobll1-001	166.74516	0.003298931	0.011570753	0.09143183	-10.93711019	0.134882393	1.475223593	-3.45116
Med14	Med14-003	10052.724	4.58212E-06	9.22965E-05	0.091478048	-10.93158435	12.78876229	139.8014337	-3.450431
Tet2	Tet2-005	3410.7594	1.23943E-06	4.00553E-05	0.091525629	-10.92590139	4.1280824	45.10302121	-3.44968
Gm38057	Gm38057-001	3765	2.9168E-05	0.000328862	0.091620962	-10.91453282	4.075237933	44.47931819	-3.448178
Dmxl1	Dmxl1-004	829.06539	1.54165E-05	0.000209529	0.091672459	-10.90840161	0.924517446	10.0850076	-3.447368
P24-246G1	24-246G10.3-	961.94974	0.000906704	0.004225911	0.091788855	-10.89456883	0.970082623	10.56863191	-3.445537
Aqr	Aqr-004	3154.5847	0.000148917	0.001074225	0.091830336	-10.88964766	3.160911189	34.42120912	-3.444885
Zfp62	Zfp62-002	629.66156	9.28441E-05	0.000756041	0.091832561	-10.88938374	0.680657126	7.411936639	-3.44485
Slc35b3	Slc35b3-201	1804.8251	0.000675234	0.003367296	0.091924259	-10.87852121	2.199429343	23.92653877	-3.443411
Mettl14	Mettl14-002	686.78854	0.006048198	0.018576879	0.092082345	-10.85984507	0.771786404	8.381480777	-3.440932
Gga3	Gga3-002	205.14194	0.009534954	0.026551207	0.092112274	-10.85631649	0.248762635	2.700645895	-3.440463
Ckap2l	Ckap2l-001	728.96971	0.035449069	0.074610127	0.092175582	-10.84886018	0.914223144	9.918279067	-3.439472
Ktn1	Ktn1-015	230.60236	0.000310626	0.001854445	0.092233812	-10.84201096	0.297335321	3.223712813	-3.43856
Tpm1	Tpm1-013	4598.9643	0.000359172	0.002071744	0.092318601	-10.83205318	6.333245085	68.60204756	-3.437235
Whsc1l1	Whsc1l1-201	7858.6395	1.04826E-06	3.6011E-05	0.092319274	-10.8319743	8.539884844	92.50381314	-3.437224
Stam	Stam-006	205.11736	0.02758151	0.061084173	0.092353903	-10.82791267	0.184567493	1.998480692	-3.436683
Klf8	Klf8-002	1628.0513	0.008770241	0.024833064	0.092360659	-10.82712066	0.763198366	8.263240798	-3.436578
Pfkfb3	Pfkfb3-019	5599.1676	6.79282E-05	0.000604925	0.092432887	-10.81866023	6.863326805	74.25200075	-3.43545
33427F10R	3427F10Rik-	522	0.000117093	0.000898392	0.092591558	-10.80012068	0.529866266	5.722619621	-3.432976
Ccnyl1	Ccnyl1-001	1684.6352	7.04074E-06	0.000121975	0.092641141	-10.79434026	1.353983703	14.6153608	-3.432203
Anp32e	Anp32e-001	6167.2517	3.0572E-06	7.16694E-05	0.092667392	-10.79128242	7.612058953	82.14387799	-3.431794
Uso1	Uso1-003	1629.3401	0.007652089	0.022348771	0.092785328	-10.77756602	2.134023712	22.99958144	-3.429959
Gtf2h3	Gtf2h3-001	1154.692	0.000295224	0.001786577	0.092812334	-10.77443001	1.297470765	13.97950795	-3.42954
Sh2d4b	Sh2d4b-002	547.79041	0.022873675	0.052699003	0.092872547	-10.76744457	0.691721274	7.448070477	-3.428604
Invs	Invs-003	759.10455	7.02472E-05	0.000618978	0.092918182	-10.76215634	0.787732525	8.477700591	-3.427895
Epn2	Epn2-202	948.52534	0.004087139	0.013640141	0.093001929	-10.75246517	1.290494963	13.87600214	-3.426596
Alg6	Alg6-001	626.71339	0.000153787	0.001101301	0.093010432	-10.7514822	0.657453854	7.068603402	-3.426464
Agtpbp1	Agtpbp1-009	715.00028	0.001190641	0.005225454	0.093028949	-10.74934216	0.830840329	8.930986982	-3.426176
Pogk	Pogk-002	4814.5065	1.50363E-06	4.48599E-05	0.093186235	-10.73119867	5.247423511	56.31114422	-3.423739
Ttc3	Ttc3-028	985.02557	1.42608E-05	0.000198066	0.093315447	-10.71633942	1.0669192	11.43346828	-3.42174
Gins2	Gins2-002	1506.978	0.005156942	0.016399745	0.093335165	-10.71407541	1.977831112	21.19063169	-3.421435
Psmf1	Psmf1-001	700.91995	0.001104666	0.004923843	0.093429779	-10.70322558	0.990338113	10.59981222	-3.419974
Ralb	Ralb-002	1633.5267	5.26538E-07	2.3886E-05	0.093471235	-10.69847848	2.198906553	23.52495443	-3.419334
BC016548	BC016548-00	414	7.89109E-05	0.000673705	0.09357126	-10.68704218	0.387355719	4.139686906	-3.417791
Mdm4	Mdm4-012	1828.6052	6.66464E-05	0.000595368	0.093574987	-10.68661647	1.676363312	17.91465178	-3.417733
Gm37124	Gm37124-001	284	0.001234162	0.005379898	0.09359275	-10.68458828	0.300900355	3.214996406	-3.417459
Atf1	Atf1-001	10766.781	5.46928E-09	1.9073E-06	0.093596965	-10.68410708	13.34624494	142.5927101	-3.417394
Plaa	Plaa-003	776.07177	5.31178E-05	0.00050326	0.093620079	-10.6814693	0.934505767	9.981894655	-3.417038
Zc3hav1l	Zc3hav1l-001	3875	1.05806E-06	3.62386E-05	0.093644636	-10.67866827	4.958540699	52.95061121	-3.41666

Smurf2	Smurf2-001	28081.252	4.83331E-09	1.8062E-06	0.093794408	-10.66161641	31.1845265	332.4774596	-3.414354
Cnep1r1	Cnep1r1-001	4099.0115	0.000332121	0.001952812	0.093798382	-10.66116475	3.715757382	39.61430163	-3.414293
Sub1	Sub1-001	2149.2667	9.37853E-07	3.36703E-05	0.093813851	-10.65940684	2.226998416	23.73848214	-3.414055
Eif4g2	Eif4g2-014	490.45866	3.9789E-05	0.000408383	0.093919107	-10.64746068	0.618028334	6.580432388	-3.412437
Necab1	Necab1-002	164.47144	0.000752507	0.003655279	0.093921527	-10.64718633	0.16903197	1.799714883	-3.4124
Dmd	Dmd-013	2227.2288	2.24711E-06	5.8706E-05	0.094005981	-10.63762103	1.937538997	20.61080559	-3.411104
Sh3pxd2a	Sh3pxd2a-202	2938.1106	0.003347201	0.011698124	0.094031483	-10.63473599	3.363087481	35.76554747	-3.410712
Nsun3	Nsun3-005	207.59596	0.025167143	0.056783573	0.094080469	-10.62919867	0.244194689	2.595593868	-3.409961
Srsf9	Srsf9-002	306.84499	0.003288297	0.011539362	0.09411276	-10.62555178	0.388865873	4.131914471	-3.409466
Dhfr	Dhfr-201	761.7029	0.005070003	0.016169865	0.094124961	-10.62417444	1.22662834	13.03191346	-3.409279
Nfe2l1	Nfe2l1-204	7517.42	1.63345E-06	4.70132E-05	0.094167903	-10.61932965	7.450300251	79.11719434	-3.408621
Fam107b	Fam107b-001	3220.0436	0.00022682	0.001465771	0.094188582	-10.61699816	5.589935169	59.34833143	-3.408304
Rbbp8	Rbbp8-202	671.64391	0.029434706	0.064302762	0.09425142	-10.60991976	0.814896155	8.645982812	-3.407342
Smarca5	Smarca5-005	477.35741	3.59598E-05	0.000379661	0.094255045	-10.60951168	0.532198104	5.646361996	-3.407286
Dkk2	Dkk2-001	3928	7.49833E-06	0.000127138	0.094259159	-10.6090486	4.936098364	52.36730742	-3.407223
Orc3	Orc3-001	2399.2864	0.015812921	0.039461729	0.094305397	-10.60384695	2.97333424	31.52878121	-3.406516
Mmadhc	Mmadhc-001	2442.9022	3.26342E-05	0.000355396	0.094340462	-10.59990572	2.633178289	27.9114416	-3.40598
Tcn2	Tcn2-004	524.73957	0.001313493	0.005641019	0.094370702	-10.59650905	0.328090983	3.476619068	-3.405517
Cald1	Cald1-015	11907.407	0.022232936	0.051577285	0.094449514	-10.587667	17.66540975	187.0354758	-3.404313
Fkbp7	Fkbp7-002	391.85234	0.013332124	0.034558787	0.094480767	-10.58416474	0.373771442	3.95605852	-3.403836
Zfp788	Zfp788-006	965.6339	0.00027812	0.001704893	0.094512833	-10.58057371	0.791215942	8.371518603	-3.403346
Alg3	Alg3-001	598.81417	0.033413661	0.071236719	0.094527095	-10.57897739	0.83625728	8.846746858	-3.403128
Ino80dos	Ino80dos-001	547.59413	0.005034944	0.016087958	0.094543026	-10.57719472	0.595728384	6.301135125	-3.402885
Hdac2	Hdac2-002	963.39297	1.10021E-05	0.000165128	0.094576414	-10.57346075	1.033131204	10.92377223	-3.402376
Gucy1a3	Gucy1a3-003	74.772516	0.033283475	0.07099452	0.094615693	-10.56907119	0.092603373	0.978731641	-3.401777
Fth-ps2	Fth-ps2-001	577	2.19983E-05	0.000270113	0.094626491	-10.56786514	0.79798569	8.43300515	-3.401612
Sass6	Sass6-003	325.53116	0.000210568	0.001384784	0.094670817	-10.56291715	0.358068352	3.782246336	-3.400936
Bivm	Bivm-003	170.64563	0.030145076	0.065595226	0.094674871	-10.56246487	0.141809469	1.497857531	-3.400875
Nsun3	Nsun3-002	1449.0683	9.40452E-05	0.000763462	0.094686578	-10.56115897	1.584457157	16.73370391	-3.400696
Mllt4	Mllt4-016	783.18445	0.02320682	0.053326642	0.094735204	-10.5557381	0.608064245	6.418566925	-3.399956
Smyd3	Smyd3-002	404.87096	0.006797139	0.020336288	0.094778969	-10.55086392	0.509918204	5.380077587	-3.399289
Mkl1	Mkl1-002	837.82843	0.008322105	0.023829557	0.094789613	-10.54967912	1.275283257	13.45382915	-3.399127
Slc22a4	Slc22a4-001	248.29637	0.002400111	0.009032223	0.094868911	-10.54086099	0.189450109	1.996967262	-3.397921
P24-230L5.	24-230L5.3-(	547	0.001242856	0.005409668	0.094879935	-10.5396362	0.662286293	6.980256588	-3.397753
Rspry1	Rspry1-002	812.50857	2.36338E-05	0.000283623	0.094940735	-10.53288661	0.91494188	9.636979083	-3.396829
530001G21I	0001G21Rik-	381.59344	0.00730467	0.021534251	0.094945011	-10.53241225	0.555804219	5.853959165	-3.396764
Dnajc10	Dnajc10-002	731.03842	2.42676E-05	0.000288399	0.094945707	-10.53233506	0.594151094	6.257798399	-3.396753
Nfe2l1	Nfe2l1-202	5961.943	9.2069E-06	0.000146108	0.095003852	-10.52588901	5.79798709	61.02896857	-3.39587
Surf1	Surf1-007	376.41031	0.005314393	0.016814591	0.095067279	-10.51886628	0.338757712	3.563347076	-3.394907
Cpne1	Cpne1-002	472.44513	0.019622636	0.046821463	0.0951978	-10.50444446	0.392293835	4.120828802	-3.392928
P24-450N1(	24-450N10.3-	373	0.002351817	0.008886775	0.095237757	-10.50003733	0.412618802	4.332512821	-3.392323
Atf2	Atf2-004	1445.5508	1.87193E-05	0.000240486	0.095241001	-10.49967961	1.694175145	17.78829624	-3.392273
30144P21R	0144P21Rik-	993.96818	2.72529E-05	0.000313978	0.09529395	-10.49384563	0.891701586	9.357378791	-3.391472
Golga1	Golga1-003	728.65513	0.003010211	0.010787877	0.095429185	-10.47897459	0.483748003	5.069183033	-3.389426
Gm12294	Gm12294-001	349	0.004341507	0.014318402	0.095467809	-10.47473504	0.376026145	3.938774233	-3.388842
Ndfip2	Ndfip2-001	3464.9535	2.3739E-07	1.55689E-05	0.095480816	-10.47330802	2.997860523	31.39751668	-3.388645
Gm26631	Gm26631-201	691.42875	0.000920762	0.004276081	0.095515956	-10.46945493	0.59823896	6.263235831	-3.388114
Dclre1c	Dclre1c-008	352.79742	0.046952656	0.093333051	0.095614013	-10.45871803	0.314623452	3.290557976	-3.386634
Tbce	Tbce-013	688.64133	0.000576755	0.002984867	0.095632594	-10.45668589	0.715973173	7.48670657	-3.386354
Ubfd1	Ubfd1-003	459.11922	0.000781292	0.003757266	0.09573652	-10.44533476	0.615575294	6.429890018	-3.384787
Adh5	Adh5-005	675.7023	0.003240168	0.011407871	0.095752612	-10.44357931	0.695452834	7.263016826	-3.384544
Fsd1l	Fsd1l-201	2890.452	2.0676E-05	0.000258923	0.095754259	-10.44339975	2.803337957	29.27637891	-3.38452
Birc6	Birc6-010	314.74298	0.018660357	0.044989246	0.095789806	-10.43952419	0.31496686	3.288104154	-3.383984
Ythdc2	Ythdc2-004	274.44425	0.000460331	0.002509364	0.095808197	-10.43752025	0.272824894	2.847615355	-3.383707
Ccdc77	Ccdc77-001	920.71216	9.45733E-05	0.000767022	0.09586914	-10.43088524	1.157117836	12.06976335	-3.38279
Ano1	Ano1-201	101.20618	0.044319603	0.089145329	0.095952005	-10.42187709	0.160267102	1.670284035	-3.381543
Iqcb1	Iqcb1-002	650.61098	0.010491008	0.028649101	0.09600957	-10.41562837	0.767757263	7.996674335	-3.380678
Trmt2a	Trmt2a-001	1496.0136	6.48941E-06	0.000115754	0.096017789	-10.4147368	1.549595199	16.13862615	-3.380554
Mospd2	Mospd2-004	991.90168	0.003365924	0.011749267	0.096031023	-10.41330159	1.139849745	11.86959916	-3.380356
Sucla2	Sucla2-001	2024.5822	2.56251E-06	6.36908E-05	0.096034177	-10.41295956	2.070544056	21.56049152	-3.380308
00097O09R	0097O09Rik-	575.20982	4.10075E-05	0.000417511	0.096034572	-10.41291668	0.903480177	9.407863808	-3.380302
Gm24303	Gm24303-201	149	0.00065021	0.003273992	0.096069522	-10.40912846	0.150095428	1.562362591	-3.379777
P24-383P24	24-383P24.2-(	772	0.001395019	0.005916219	0.096075189	-10.40851456	0.782151374	8.141033962	-3.379692
Cnot7	Cnot7-004	374.63302	0.000301221	0.00181602	0.09607816	-10.4081927	0.407726611	4.243697134	-3.379648
10203C20R	0203C20Rik-	9786.1531	1.32159E-08	3.01762E-06	0.096095724	-10.40629032	11.02589217	114.738635	-3.379384
Srsf3	Srsf3-005	10079.523	3.7252E-06	8.05481E-05	0.096201768	-10.39481938	13.34679065	138.737478	-3.377793
P4hb	P4hb-003	2049.1487	2.38626E-07	1.55689E-05	0.096220618	-10.39278299	2.111882439	21.94833588	-3.37751
Fgd5	Fgd5-201	345.76	0.000916839	0.004260172	0.096224568	-10.3923563	0.307316115	3.193738561	-3.377451
Rai1	Rai1-005	1027.4538	0.001725898	0.006965827	0.096294206	-10.38484082	1.513453719	15.71697596	-3.376407
Prrc2a	Prrc2a-006	933.63542	0.000155948	0.001112059	0.096311092	-10.38302002	1.158371837	12.02739797	-3.376154
Ints2	Ints2-008	595.64	0.000550924	0.002883429	0.096444309	-10.36867817	0.647705184	6.715846602	-3.37416
Gcsh	Gcsh-001	2369.0042	4.33071E-06	8.87652E-05	0.096464431	-10.36651526	3.521194614	36.50251768	-3.373859
Trim36	Trim36-201	331.92539	0.016254002	0.040302737	0.096478207	-10.36503508	0.362266982	3.754909975	-3.373653
Fuca1	Fuca1-003	838.85916	0.020066516	0.047621826	0.096485628	-10.3642379	0.897793249	9.304942813	-3.373542
Alg11	Alg11-001	5151.6335	2.27841E-05	0.000276623	0.096557785	-10.35649271	6.291714562	65.16009597	-3.372464
Ttc3	Ttc3-027	954.42712	0.000382741	0.002177554	0.096571461	-10.35502613	1.129777603	11.6988766	-3.372259
Usp3	Usp3-008	190.54239	0.002233976	0.008547104	0.096643808	-10.34727443	0.194752078	2.015153197	-3.371179
RP23-407I6.	23-407I6.7-0	190	0.000693531	0.003441521	0.096658387	-10.34571371	0.184529421	1.909088562	-3.370961
Anln	Anln-201	3074	0.045358181	0.090735411	0.096668456	-10.34463614	4.413967804	45.66089088	-3.370811
Snapc3	Snapc3-005	2986.7179	2.10914E-05	0.000262588	0.096679534	-10.34345077	2.974734667	30.7690216	-3.370646
Xpo6	Xpo6-001	1769.5336	0.003617301	0.012412594	0.096745612	-10.33638609	1.94414015	20.0953832	-3.36966
Gm16310	Gm16310-001	235	0.010442079	0.028558749	0.096824265	-10.32798962	0.31484584	3.251724564	-3.368488
Obfc1	Obfc1-002	539.0828	0.00724663	0.021417781	0.096834328	-10.32691626	0.388740246	4.014487971	-3.368338
Dclre1c	Dclre1c-004	1076.6947	5.7955E-05	0.000537056	0.09685663	-10.32453844	1.647025896	17.00478217	-3.368005
Rnf181	Rnf181-003	1226.2803	0.036434881	0.076328501	0.096913879	-10.3184395	1.355130173	13.9828287	-3.367153
Wac	Wac-007	4284.3999	1.21056E-06	3.94874E-05	0.09695404	-10.31416534	4.547813442	46.90689979	-3.366555
Aff1	Aff1-201	4825.0375	0.000460431	0.002509509	0.096955615	-10.31399783	5.843408707	60.26890473	-3.366532
Gtf3c5	Gtf3c5-001	420.86738	0.023088668	0.053115724	0.096999662	-10.30931423	0.425301477	4.384566574	-3.365876
Itga6	Itga6-001	2194.1857	0.001542865	0.006387422	0.097013203	-10.30787526	2.49829142	25.75207632	-3.365675
Crybg3	Crybg3-002	2580.9895	3.0168E-06	7.08192E-05	0.097067883	-10.30206868	3.088959323	31.8226711	-3.364862
Raph1	Raph1-003	3285.2138	6.02062E-06	0.000109801	0.097153866	-10.2929512	4.47874799	46.09953448	-3.363585
Pde7b	Pde7b-001	1445.5099	9.26056E-07	3.35538E-05	0.097181475	-10.29002695	1.23671892	12.72587102	-3.363175
Gm16230	Gm16230-001	109	0.018618307	0.044913157	0.097200713	-10.28799033	0.132305249	1.361155127	-3.362889
Sel1l	Sel1l-003	589.26529	0.002335544	0.0088357	0.097202599	-10.28779074	0.710769492	7.312247798	-3.362861
Ywhaz	Ywhaz-002	86761.092	6.76187E-09	2.13967E-06	0.097227225	-10.285185	94.45893099	971.5275801	-3.362496
Nde1	Nde1-008	355.22057	0.010482517	0.028635045	0.097246656	-10.28312993	0.410516698	4.221396542	-3.362208
Large	Large-002	2350.9625	2.80485E-06	6.75571E-05	0.097308622	-10.27658166	2.06161004	21.18630392	-3.361289
Birc6	Birc6-014	1184.4749	0.028340952	0.062390898	0.097345075	-10.27273336	1.392176271	14.30145561	-3.360748
Kat7	Kat7-002	394.47618	0.006979824	0.020769676	0.097346865	-10.27254446	0.47115988	4.840010817	-3.360722
Eif2a	Eif2a-001	3727.8671	3.67913E-07	1.95365E-05	0.097361579	-10.27099196	4.323810798	44.40982594	-3.360504
Gm26836	Gm26836-201	1112	5.01423E-06	9.81274E-05	0.097368129	-10.27030113	1.225257769	12.58376625	-3.360407
Hsd17b10	Hsd17b10-002	1109.8899	0.005299041	0.016776868	0.097369846	-10.27011996	1.275688592	13.10147487	-3.360381
Gemin8	Gemin8-001	587.74327	4.14565E-05	0.000420956	0.097390442	-10.26794801	0.687723937	7.061513628	-3.360076
P23-239A1	23-239A13.2-	597	2.2332E-05	0.000272876	0.097453967	-10.26125493	0.670499959	6.880171009	-3.359135
Ankrd6	Ankrd6-006	826	6.13327E-05	0.000559426	0.097508784	-10.25548636	0.842455148	8.639787277	-3.358324
Med8	Med8-201	411.58421	0.019476072	0.046520481	0.097610863	-10.24476137	0.374722456	3.838942145	-3.356814
Gm10566	Gm10566-001	505	4.83346E-05	0.0004695	0.097619244	-10.24388178	0.680265591	6.968560292	-3.356691

Psma4	Psma4-005	459.02423	0.000203651	0.001350446	0.097619924	-10.2438105	0.541320783	5.545187525	-3.356681
Gm21975	Gm21975-001	2201.4864	0.000729065	0.003568814	0.097623211	-10.24346551	3.721616267	38.12224788	-3.356632
Hmgb3	Hmgb3-001	1004.3771	0.014793959	0.037482845	0.097666965	-10.23887657	1.391729714	14.24974877	-3.355986
Gm15939	Gm15939-001	108.70092	0.002454599	0.009193003	0.097678098	-10.23770955	0.12890495	1.319691438	-3.355821
Myef2	Myef2-007	1693.7971	4.34453E-05	0.000436364	0.097685168	-10.23696864	1.796308231	18.38875103	-3.355717
Pip4k2a	Pip4k2a-001	5511.1491	1.18137E-06	3.88683E-05	0.097711748	-10.23418393	8.598784986	88.00154712	-3.355324
Cobll1	Cobll1-201	137.21967	0.005227896	0.016583812	0.097735445	-10.23170248	0.118277504	1.210180231	-3.354974
Asph	Asph-019	190.9316	0.01008629	0.02777866	0.097797611	-10.22519866	0.170406354	1.742438826	-3.354057
Oaz1	Oaz1-201	625.68726	0.00586816	0.01812519	0.09782262	-10.22258447	0.629289476	6.432964819	-3.353688
Tex264	Tex264-010	629.63979	0.028002795	0.061817321	0.097874723	-10.21714262	0.640684278	6.54596264	-3.35292
Abr	Abr-006	4031.0603	0.001104566	0.004923843	0.097936358	-10.21071254	4.122625683	42.09494577	-3.352012
Abcb1b	Abcb1b-006	267.35656	0.022992504	0.052915823	0.097969879	-10.20721884	0.299398111	3.056022036	-3.351518
Ido1	Ido1-002	255.12756	0.014448497	0.036798114	0.098056124	-10.19824121	0.161930735	1.651408692	-3.350248
Gm27209	Gm27209-001	430	0.00027342	0.001682543	0.098097356	-10.19395468	0.373274464	3.805142975	-3.349642
Ints10	Ints10-001	993.31441	0.00076868	0.0037133	0.098105967	-10.19305994	1.33678275	13.6259067	-3.349515
Fyttd1	Fyttd1-201	340.27355	0.034892961	0.073743301	0.098119735	-10.19162969	0.347515414	3.541748417	-3.349313
Hnrnph1	Hnrnph1-015	1233.8168	0.003477428	0.012036574	0.098214911	-10.18175341	1.533810068	15.6168759	-3.347914
Diablo	Diablo-004	558.44109	0.041050618	0.083855679	0.098284677	-10.17452596	0.72509059	7.377453026	-3.34689
Hmgb3	Hmgb3-002	2048.6898	0.000629552	0.003192975	0.098396086	-10.16300592	2.972431374	30.20883764	-3.345255
Sgol1	Sgol1-001	696.5745	0.003037954	0.010864562	0.098396162	-10.16299805	0.875349151	8.896171715	-3.345254
00037H04R	00037H04Rik-	120.3523	0.026583312	0.05928752	0.098431002	-10.15940075	0.154037857	1.56493232	-3.344743
Sirt2	Sirt2-005	2261.8795	8.97555E-07	3.29397E-05	0.098433725	-10.15911978	2.048435473	20.81030134	-3.344704
00008O03R	0008O03Rik-	609	0.000154331	0.001103054	0.098463198	-10.15607878	0.65284555	6.630350832	-3.344272
Ccser2	Ccser2-008	1121.9879	0.000490135	0.00263286	0.098475297	-10.15483098	0.788561307	8.007706794	-3.344094
Prex1	Prex1-002	232.40216	0.003421696	0.011892946	0.098498952	-10.15239232	0.395517132	4.015445095	-3.343748
Ubap2l	Ubap2l-020	299.95063	0.000104632	0.000823828	0.098647353	-10.1371194	0.413197204	4.188629396	-3.341576
Psmc3	Psmc3-002	461.67413	0.001197769	0.005247327	0.098761543	-10.12539869	0.229155971	2.320295569	-3.339907
Rbm28	Rbm28-005	155.0763	0.004217211	0.014000143	0.098803158	-10.12113396	0.211800276	2.143658962	-3.339299
Rmi1	Rmi1-201	2818	4.05246E-07	2.05608E-05	0.098808334	-10.12060377	3.341852963	33.82156969	-3.339223
Zfp182	Zfp182-001	1060.0952	0.004418636	0.014516862	0.098835431	-10.11782905	0.960644305	9.719634852	-3.338828
Leng8	Leng8-004	3992.6111	0.000166829	0.001168011	0.098870235	-10.1142675	4.726963426	47.80977255	-3.33832
Was	Was-002	354.26487	0.000255877	0.001601864	0.098939734	-10.10716284	0.370249953	3.742176561	-3.337306
Itgax	Itgax-201	2113	0.025697555	0.05770283	0.098956444	-10.1054561	4.219107585	42.6360065	-3.337063
Smg7	Smg7-005	1596.896	2.56214E-05	0.000300628	0.098965012	-10.10458116	1.649463165	16.66713442	-3.336938
Stag1	Stag1-016	4896.9737	1.04478E-06	3.59868E-05	0.098978056	-10.10324955	4.743589834	47.92567186	-3.336747
Oaz1	Oaz1-004	1361.2138	0.00416921	0.013878456	0.098986924	-10.1023444	1.514014363	15.29509453	-3.336618
Ube2j1	Ube2j1-003	1750.3453	1.37143E-05	0.000192981	0.098988181	-10.10221611	2.1604408	21.82523986	-3.3366
Kmt2e	Kmt2e-007	6184.5109	0.00011283	0.000873318	0.099027262	-10.09822728	6.772927146	68.39455763	-3.33603
Man1c1	Man1c1-201	6762.8249	0.003084992	0.010980046	0.099292629	-10.07124107	8.143446655	82.0146144	-3.33217
Phka1	Phka1-005	547.57251	0.012545246	0.032912652	0.099292697	-10.07123413	0.616229818	6.206194772	-3.332169
30428H23R	30428H23Rik-	1591.1262	3.48992E-05	0.000371326	0.099376586	-10.06273253	1.875018333	18.86780797	-3.33095
Mesdc2	Mesdc2-001	11714.118	3.07733E-09	1.51021E-06	0.099419016	-10.05843787	13.84778794	139.2871146	-3.330334
Lrrc8e	Lrrc8e-201	387	0.000114462	0.000882759	0.099420127	-10.0583255	0.481890685	4.847013364	-3.330318
Ssb	Ssb-004	175.31056	0.026068742	0.058394036	0.099442237	-10.05608911	0.153011329	1.538695556	-3.329997
Fbxl12	Fbxl12-002	661.44892	0.022939834	0.052826552	0.099445454	-10.05576384	0.771362206	7.756636179	-3.329951
Atp2a2	Atp2a2-003	6653.5334	1.46667E-07	1.18755E-05	0.099467858	-10.05349893	7.426101454	74.65830302	-3.329626
Asap1	Asap1-004	3870.8941	5.49859E-05	0.000516247	0.09953174	-10.04704634	5.353471501	53.78657623	-3.3287
Ubap2l	Ubap2l-006	1851.4904	0.001519404	0.006313187	0.099593348	-10.0408312	2.539327246	25.49695623	-3.327807
Uap1	Uap1-007	331.51105	0.004679066	0.01518026	0.099612035	-10.03894761	0.120289258	1.207577562	-3.327536
Huwe1	Huwe1-006	1580.4006	0.000398344	0.002242856	0.099619525	-10.03819283	2.092004764	20.99994723	-3.327428
Cops5	Cops5-007	1398.3001	0.000142251	0.001040154	0.099651909	-10.0349307	1.783755636	17.8998642	-3.326959
Mff	Mff-015	687.89016	7.74729E-05	0.000664358	0.099668465	-10.03326374	0.704475993	7.068193433	-3.326719
Me3	Me3-004	373.00966	0.001192525	0.00523102	0.099676779	-10.03242695	0.449244114	4.507008758	-3.326599
Phtf1	Phtf1-002	2284.7951	0.037061263	0.077356833	0.099717126	-10.02836762	2.870346579	28.78489069	-3.326015
Ryk	Ryk-002	1984.2498	2.63556E-05	0.000306409	0.099723413	-10.02773541	1.89910702	19.04374272	-3.325924
Entpd5	Entpd5-202	2743.9606	0.001999204	0.007832259	0.099732316	-10.02684026	3.028421733	30.36550097	-3.325795
Cdc14a	Cdc14a-003	1099.696	4.49728E-05	0.000447247	0.099752834	-10.02477781	1.340495549	13.43817004	-3.325498
Mrpl33	Mrpl33-004	518.11722	0.000598999	0.003071674	0.099774695	-10.02258139	0.611900957	6.132827147	-3.325182
Tyms	Tyms-003	579.75252	0.011920669	0.031644818	0.09979878	-10.02016258	0.820874641	8.22529736	-3.324834
)30518I15Ri	0518I15Rik-2	943	9.21494E-06	0.000146108	0.099831397	-10.01688882	0.939011303	9.405971823	-3.324363
Ifi44	Ifi44-001	348.65903	0.001161659	0.005125186	0.099842586	-10.0157662	0.426034967	4.267066625	-3.324201
Gm22174	Gm22174-201	108	0.003510696	0.012132109	0.100007873	-9.999212772	0.100366785	1.003588838	-3.321815
Morc2a	Morc2a-004	295.67659	0.001254047	0.005444431	0.100156683	-9.984356184	0.351101702	3.505524446	-3.319669
Lcorl	Lcorl-007	606.91869	0.001024869	0.004643704	0.100319546	-9.968147161	0.747357996	7.449774482	-3.317325
Adrm1	Adrm1-008	382.38661	0.022743253	0.052490325	0.100324497	-9.967655235	0.522525728	5.208356305	-3.317254
Celf1	Celf1-003	1254.6978	0.000788741	0.003786179	0.100412775	-9.958892172	1.544359095	15.3801057	-3.315985
Kif20b	Kif20b-201	1786	0.013912937	0.035720996	0.100441209	-9.956072876	2.852357174	28.39827589	-3.315577
Tmem39a	Tmem39a-014	639.11949	0.001227337	0.005356305	0.100452203	-9.954983278	0.742209251	7.388680686	-3.315419
Trim43b	Trim43b-201	279	0.000300604	0.001812938	0.1005352	-9.946764912	0.290258192	2.887129999	-3.314227
Suv39h2	Suv39h2-001	494.47658	0.003009022	0.01078666	0.100538535	-9.946434945	0.756892914	7.528386127	-3.31418
Gtf2i	Gtf2i-013	237.71923	0.001428031	0.006024396	0.100554989	-9.944807382	0.295420218	2.937897164	-3.313943
Arid4b	Arid4b-201	983.25453	0.033782365	0.07187514	0.100594337	-9.940917404	0.772958803	7.683919619	-3.313379
Rsrc2	Rsrc2-012	2765.1124	1.19168E-06	3.9095E-05	0.100602476	-9.940113176	3.361976284	33.41842476	-3.313262
Gyg	Gyg-201	2595.9583	0.00023707	0.001514012	0.100608687	-9.939499603	3.018188929	29.99928766	-3.313173
Ep300	Ep300-002	1509.2453	6.48619E-06	0.000115754	0.100615613	-9.938815388	2.002680829	19.90427504	-3.313074
Nfia	Nfia-006	2265.9792	0.00244858	0.009174473	0.100685341	-9.931932364	2.558408736	25.40994253	-3.312074
Sbno1	Sbno1-011	124	0.005957996	0.018340945	0.100693863	-9.931091847	0.151781458	1.507355597	-3.311952
Cytip	Cytip-001	1762.145	0.040458908	0.082866656	0.100694405	-9.93103841	3.709080806	36.83502395	-3.311945
Gm16286	Gm16286-002	402.75462	0.011559065	0.030905385	0.100706439	-9.929851688	0.490257581	4.868185064	-3.311772
Dll1	Dll1-006	2699.267	2.17478E-05	0.000267744	0.100737897	-9.926750788	0.601547065	5.971407801	-3.311322
Gm37618	Gm37618-001	451	0.005020856	0.016052648	0.100747388	-9.925815664	0.509580559	5.058002696	-3.311186
33428G20R	33428G20Rik-	986.78734	5.59842E-05	0.000522896	0.10077256	-9.923336311	0.904447934	8.975141024	-3.310825
Caprin1	Caprin1-008	10794.673	1.55191E-07	1.23039E-05	0.100836068	-9.917086422	13.44338834	133.3192439	-3.309916
Snapc4	Snapc4-003	897.57842	0.000488631	0.002628077	0.100836464	-9.917047465	0.964437554	9.56437182	-3.309911
RP23-409I5.	P23-409I5.1-0	649	0.001367941	0.00582374	0.100853634	-9.915359097	0.785265576	7.786190172	-3.309665
Mier1	Mier1-004	1111.199	1.52054E-06	4.50505E-05	0.100906624	-9.910152163	1.369784474	13.57477257	-3.308907
Dlg1	Dlg1-008	634.52799	0.004018537	0.013469848	0.100962374	-9.904679983	0.76942142	7.620872934	-3.30811
Asah1	Asah1-004	5193.6546	0.000149662	0.001078687	0.10096534	-9.904388957	6.708217247	66.44079283	-3.308068
H3f3a	H3f3a-001	5121.6171	1.11796E-05	0.000167161	0.100966022	-9.904322066	5.508697008	54.55990933	-3.308058
Sec23b	Sec23b-002	911.66235	9.01583E-06	0.000144295	0.100988863	-9.902082008	1.174027906	11.6253206	-3.307732
Galnt11	Galnt11-005	3733.0551	1.98124E-07	1.41379E-05	0.100990444	-9.901927002	4.812745258	47.65545222	-3.307709
P24-500I24	24-500I24.2-0	1211	1.36825E-06	4.26277E-05	0.101053057	-9.895791715	1.41474528	14.00002462	-3.306815
Srsf5	Srsf5-009	108.54857	0.013591518	0.035066269	0.101063308	-9.894787968	0.129795179	1.284295776	-3.306669
Caprin1	Caprin1-014	555.37101	0.020533658	0.048498571	0.101102118	-9.890989589	0.612591559	6.059136728	-3.306115
Timm8a1	Timm8a1-001	1066	3.00482E-05	0.000335767	0.101169034	-9.884447449	1.730233587	17.10240296	-3.30516
Atp2a2	Atp2a2-005	463.35583	0.000941506	0.004348245	0.101471931	-9.85494206	0.523513895	5.159199102	-3.300847
Gnb2l1	Gnb2l1-005	448.324	0.000894175	0.004185525	0.101508885	-9.851354432	0.522585599	5.148175958	-3.300322
Gm14461	Gm14461-001	244	0.000254141	0.001593614	0.101519333	-9.850340572	0.300697554	2.961973313	-3.300174
Dnajc2	Dnajc2-006	2752.6547	0.000456958	0.002495347	0.101546376	-9.847717223	3.973432721	39.12924184	-3.299789
Mpdz	Mpdz-008	611.36486	0.001182044	0.005197716	0.101600064	-9.842513458	0.592561756	5.832297058	-3.299027
Casp4	Casp4-003	1333.3075	0.031435	0.067816122	0.101641967	-9.838455777	1.374194825	13.51995501	-3.298432
Topbp1	Topbp1-003	208.75057	0.007100015	0.021072403	0.101644751	-9.838186312	0.248749687	2.447245762	-3.298392
Fbxw2	Fbxw2-007	3252.356	1.79184E-08	3.32556E-06	0.101669428	-9.835798425	3.649894441	35.899626	-3.298042
Gria3	Gria3-006	566.12576	0.015665142	0.039168066	0.1016757	-9.835191652	0.443122267	4.358192421	-3.297953
Scyl2	Scyl2-005	853.53268	2.83004E-06	6.80203E-05	0.101684863	-9.834305467	1.041638445	10.24379066	-3.297823
Akap10	Akap10-002	240.75493	0.02446415	0.055533649	0.101731242	-9.829821947	0.169649112	1.667620567	-3.297165
P23-77D22	23-77D22.2-0	189	0.001086215	0.004855501	0.101786879	-9.82444903	0.230886005	2.268327785	-3.296377

Pja2	Pja2-202	3403.6348	9.57103E-06	0.000150164	0.101946756	-9.809041922	3.141838471	30.81842527	-3.294112
10010F05R	0010F05Rik-	510.62916	0.032552102	0.069738505	0.101948716	-9.808853295	0.674077543	6.611927731	-3.294084
Tcf4	Tcf4-030	4085.7355	5.39607E-05	0.000509554	0.101972275	-9.806587091	6.055469315	59.38348721	-3.293751
Rian	Rian-007	2261.4493	3.08467E-05	0.000341577	0.101974841	-9.8063404	1.929527411	18.92160261	-3.293715
Ctnnd1	Ctnnd1-005	10026.745	0.000111667	0.000866276	0.101982835	-9.805571725	10.00306198	98.08574169	-3.293602
AB041806	AB041806-00	297	0.000253585	0.001591489	0.101985867	-9.805280202	0.332905851	3.264235152	-3.293559
Gm38082	Gm38082-001	4217	6.74138E-08	7.59514E-06	0.10201562	-9.802420477	5.19676027	50.94082928	-3.293138
Stim2	Stim2-003	260.89294	0.001340952	0.005730207	0.102033252	-9.800726546	0.1675917	1.642520425	-3.292889
Gm24576	Gm24576-201	609	6.93229E-06	0.000120646	0.102133655	-9.791091897	0.597182287	5.847066648	-3.29147
Snapin	Snapin-005	3360.7952	7.4373E-05	0.000646282	0.102178807	-9.786765239	3.718296283	36.39009281	-3.290832
Arhgef10l	Arhgef10l-00	602.98596	0.026786672	0.059651723	0.102195327	-9.785183261	0.744801703	7.288021156	-3.290599
Mtif2	Mtif2-001	317.55812	0.001653702	0.006741971	0.102272053	-9.777842217	0.375847269	3.674975293	-3.289516
Nop56	Nop56-004	1248.7289	0.000390176	0.002209212	0.102320454	-9.773216975	2.088588363	20.41222724	-3.288834
Nmd3	Nmd3-007	979.66533	0.000400848	0.002253249	0.1023212	-9.773145708	1.297885214	12.68442131	-3.288823
Rap1a	Rap1a-004	1696.5678	0.000190851	0.001285982	0.102322426	-9.773028613	1.863275931	18.20984899	-3.288806
Gm14681	Gm14681-001	1842	8.49166E-07	3.18758E-05	0.102347473	-9.770636922	2.9936957	29.25031374	-3.288453
Whsc1l1	Whsc1l1-009	1679.1134	0.000204355	0.001352754	0.10242448	-9.763290948	1.993636219	19.46445045	-3.287368
Hdac10	Hdac10-004	196.2262	0.012732645	0.033292091	0.102456785	-9.760212542	0.14507147	1.415928385	-3.286913
30025C20R	0025C20Rik-	624	0.00017545	0.001209088	0.102498114	-9.756277112	0.880326527	8.588709543	-3.286331
Lppr3	Lppr3-002	1519.617	5.15715E-06	9.96068E-05	0.102531798	-9.75307189	1.84989226	18.0421322	-3.285857
Dab2ip	Dab2ip-012	4630.7175	0.000131521	0.000981395	0.10254752	-9.751576655	4.898204671	47.76521832	-3.285635
Gm37034	Gm37034-001	617	0.000121634	0.000924948	0.10257174	-9.749274017	0.753921834	7.350190543	-3.285295
Eif2s2	Eif2s2-002	1751.5825	4.22448E-06	8.75838E-05	0.102652945	-9.741561735	2.410802164	23.48497811	-3.284153
Ufd1l	Ufd1l-006	524.60931	2.14302E-05	0.000265648	0.102673306	-9.739629918	0.568935936	5.541225465	-3.283867
Tbce	Tbce-014	657.66683	0.000187408	0.00127028	0.102743176	-9.733006486	0.719484418	7.002746504	-3.282886
Gm8121	Gm8121-001	394	0.001062674	0.004772073	0.102783905	-9.729149715	0.394153188	3.834775376	-3.282314
Slc4a1ap	Slc4a1ap-009	859.06015	2.34965E-05	0.000282362	0.102821495	-9.725592911	0.956860758	9.306038207	-3.281786
Pkn2	Pkn2-006	1241.8241	0.008610765	0.024474581	0.102881239	-9.719945133	1.060592278	10.30889875	-3.280948
Atg16l1	Atg16l1-003	88.408093	0.041985067	0.085401387	0.102924334	-9.715875326	0.160096381	1.555476474	-3.280344
Atxn7l1os1	Atxn7l1os1-00	461	2.62238E-05	0.000305602	0.102957218	-9.712772196	0.618478588	6.007141635	-3.279883
Med14	Med14-002	1335.2441	0.002819994	0.010239034	0.102978291	-9.71078461	1.528355856	14.84153452	-3.279588
Abcc9	Abcc9-202	358.54423	0.000755253	0.003665042	0.103037751	-9.705180735	0.330875338	3.211204957	-3.278755
Cyfip1	Cyfip1-001	8229.4799	1.54609E-05	0.000209909	0.103059954	-9.703089938	7.79713808	75.65633205	-3.278444
Zfp354c	Zfp354c-001	1553.9411	6.55915E-05	0.000589171	0.103075472	-9.70162914	1.905939305	18.4907163	-3.278227
Ccr5	Ccr5-001	1058.9048	0.020139662	0.047759013	0.103123001	-9.697157685	2.396345286	23.23773811	-3.277562
Ric8	Ric8-003	3138.1353	5.72115E-06	0.000106453	0.103183451	-9.691476593	3.233157592	31.33407113	-3.276716
Naa50	Naa50-002	2385.9987	0.000498745	0.002666563	0.103199417	-9.689977179	2.916490666	28.260728	-3.276493
Atf2	Atf2-005	1394.355	0.016465664	0.040715305	0.103322723	-9.67841314	1.862788182	18.02883361	-3.274771
Pqlc3	Pqlc3-004	4442.2649	2.0851E-08	3.6337E-06	0.103380962	-9.672960842	3.310777657	32.02502263	-3.273958
Anapc1	Anapc1-007	499.55947	0.000266926	0.001652903	0.103390044	-9.672111151	0.480427247	4.646745735	-3.273831
Cenpa	Cenpa-010	1302.8659	2.09577E-05	0.000261399	0.103399224	-9.671252449	1.801057436	17.41848114	-3.273703
Myo6	Myo6-004	3233.2668	8.14747E-05	0.000690303	0.103465096	-9.6650952	3.425158886	33.1044867	-3.272784
Mrpl2	Mrpl2-003	468.26562	0.033419137	0.071243959	0.103480638	-9.663643504	0.438932845	4.24169054	-3.272567
Msh2	Msh2-002	986.687	0.003536063	0.012198859	0.103491559	-9.662623831	1.312876564	12.68583237	-3.272415
Ppip5k2	Ppip5k2-004	746.28955	0.000416653	0.002321892	0.103526434	-9.659368715	1.078025511	10.41304589	-3.271929
Plagl2	Plagl2-002	2049.0566	0.000927206	0.004296105	0.103545833	-9.657559059	2.830972935	27.34028831	-3.271659
P23-434M2	23-434M21.2-	644	7.60296E-05	0.000656088	0.103565671	-9.655709173	0.673994106	6.507891073	-3.271382
Hist2h2be	Hist2h2be-001	4478	0.000127545	0.000958875	0.103568161	-9.655476996	5.10513841	49.29254648	-3.271348
Ints8	Ints8-004	2094.7727	1.17927E-05	0.000172903	0.103612193	-9.651373752	2.69016485	25.96378643	-3.270734
Acvr1	Acvr1-001	6721.009	1.20184E-05	0.00017561	0.103637984	-9.648971923	7.534389045	72.69910836	-3.270375
Dhx15	Dhx15-007	363.92952	0.002196448	0.008424179	0.103645124	-9.64830728	0.463508419	4.472071651	-3.270276
Nfkb1	Nfkb1-007	2739.8076	3.17018E-07	1.79173E-05	0.103742426	-9.639257882	3.882174183	37.4212781	-3.268922
Dpy30	Dpy30-202	462.62117	0.016112492	0.040015435	0.103746274	-9.638900425	0.594916311	5.734339079	-3.268869
Alkbh1	Alkbh1-010	1694.9229	1.70228E-05	0.000224761	0.103765276	-9.637135247	2.422362659	23.34463657	-3.268604
Senp7	Senp7-001	1594.8582	0.011187714	0.030083945	0.10377941	-9.635822777	1.584213057	15.26519626	-3.268408
Hltf	Hltf-004	1601.2276	7.90525E-07	3.04904E-05	0.103854577	-9.628848657	1.98401283	19.10375928	-3.267363
Uchl5	Uchl5-003	92.81646	0.021015784	0.049368355	0.103948463	-9.620151905	0.11887613	1.143606426	-3.26606
Myef2	Myef2-009	1046.3414	0.024878806	0.056247877	0.103950389	-9.619973593	1.530180835	14.72029922	-3.266033
Far2	Far2-002	827.39356	4.04523E-05	0.00041358	0.103979357	-9.617293594	0.889147123	8.551188934	-3.265631
Pitpnm3	Pitpnm3-005	2072.901	0.004321962	0.01427181	0.104017922	-9.61372788	1.434277685	13.78875537	-3.265096
Cdyl	Cdyl-201	1158.4243	0.024073772	0.054829544	0.104040336	-9.611656801	1.567742572	15.06860356	-3.264785
Sncaip	Sncaip-008	164.78982	0.019924203	0.047379293	0.104042237	-9.611481112	0.176094152	1.692525616	-3.264759
Pan3	Pan3-007	1132.5507	0.016962392	0.041708803	0.104087487	-9.607302752	1.541796008	14.81250103	-3.264131
230398E01F	0398E01Rik-	726.95069	0.00068651	0.003411634	0.104102562	-9.605911522	0.871327853	8.36989826	-3.263923
P2ry4	P2ry4-001	368	0.000915513	0.004256357	0.104137571	-9.602682193	0.41202408	3.956536294	-3.263437
Irf4	Irf4-002	202.02667	0.0044973	0.014728678	0.104162701	-9.600365466	0.40619938	3.899662496	-3.263089
Nup43	Nup43-001	701.55198	9.58565E-05	0.000773631	0.104164361	-9.600212513	1.325063465	12.72089086	-3.263066
Suv420h1	Suv420h1-20	3728.9497	1.15265E-05	0.000170754	0.104209823	-9.596024334	4.070385404	39.05951738	-3.262437
Gm15401	Gm15401-00	543.55212	5.85723E-05	0.000540919	0.104277922	-9.58975768	0.607324175	5.824091673	-3.261494
Gm12112	Gm12112-001	315	8.56875E-05	0.000712683	0.104311149	-9.586702943	0.41646179	3.992495465	-3.261035
Usp50	Usp50-004	348.52242	8.85773E-05	0.000729272	0.104323341	-9.585582538	0.376195033	3.606048542	-3.260866
Arhgap19	Arhgap19-001	376.557	0.001769601	0.007114548	0.104348541	-9.583267637	0.594182769	5.694212497	-3.260518
Zfp280d	Zfp280d-021	623.96574	0.004996389	0.015999094	0.104412987	-9.577352697	0.870389144	8.336023812	-3.259627
Dusp22	Dusp22-001	1001.5781	9.6714E-05	0.00077812	0.104458672	-9.573164006	1.353123888	12.9536769	-3.258996
Eps15	Eps15-001	15153.151	3.15833E-09	1.51021E-06	0.104470512	-9.572079057	17.24138271	165.0358784	-3.258832
Gm38374	Gm38374-001	1697	5.06158E-08	6.36457E-06	0.104526331	-9.566967428	1.921933111	18.38707148	-3.258062
Hnrnpu	Hnrnpu-003	794.5188	0.00023481	0.001503785	0.104629381	-9.557544871	1.072812923	10.25345765	-3.25664
Ift20	Ift20-005	721.43572	0.03571663	0.075057958	0.104660745	-9.554680721	0.687032344	6.564374692	-3.256208
Gm37598	Gm37598-001	766	0.001702004	0.006885616	0.104686765	-9.55230586	0.814426703	7.779652969	-3.255849
Atr	Atr-008	573.1584	0.000802824	0.003839251	0.104722	-9.549091922	0.673236881	6.428800862	-3.255364
Mfsd4	Mfsd4-003	391.86376	0.000285366	0.001738798	0.104777779	-9.544008376	0.404550503	3.861033385	-3.254595
Mid1	Mid1-004	853.49518	0.0003945	0.002227672	0.10482235	-9.539950219	1.334452114	12.73060673	-3.253982
Sag	Sag-007	712.58678	1.49968E-06	4.4805E-05	0.104823048	-9.539886655	0.868134103	8.281900948	-3.253972
Ncor1	Ncor1-007	6470.8232	0.00120693	0.005278681	0.104826994	-9.5395276	6.842835055	65.27741387	-3.253918
Igf2	Igf2-008	1622.9725	0.003349706	0.011705687	0.104831996	-9.539072381	0.556692716	5.310332108	-3.253849
Sptan1	Sptan1-008	3468.7494	4.37256E-06	8.92024E-05	0.104874438	-9.535211961	3.072200801	29.29408582	-3.253265
Gm9797	Gm9797-201	838	0.000135817	0.001005343	0.104895146	-9.533329593	1.380648151	13.16217388	-3.25298
Sbno1	Sbno1-002	2045.9238	8.509E-06	0.00013858	0.104911109	-9.531878988	2.297527282	21.89975203	-3.252761
RP23-50I16.	P23-50I16.3-0	259	0.014966871	0.037816908	0.104966803	-9.526821516	0.396581685	3.778162929	-3.251995
Rrnad1	Rrnad1-003	101.50648	0.024163014	0.054999882	0.104972303	-9.526322412	0.084752036	0.807375223	-3.251919
Ube2r2	Ube2r2-003	3522.7252	2.95645E-06	6.98335E-05	0.105252415	-9.500969637	4.419619261	41.9906684	-3.248075
Zranb2	Zranb2-002	1323.0977	0.008358414	0.023907953	0.105254537	-9.500778083	1.940400968	18.43531899	-3.248046
Dao	Dao-004	554	0.001132493	0.005024339	0.105275401	-9.498895202	0.617952604	5.869867023	-3.24776
Rsrc2	Rsrc2-201	1738.2084	0.029277586	0.064020769	0.105278361	-9.49862813	2.507265771	23.81558518	-3.247719
Nptn	Nptn-005	2323.6615	1.28565E-06	4.09614E-05	0.105303738	-9.496339057	3.040196113	28.87073309	-3.247371
30221H12F	0221H12Rik-	233.71094	0.016784785	0.041367633	0.105307945	-9.495959656	0.393438266	3.736073898	-3.247314
Mettl7a2	Mettl7a2-001	880.491	0.000470391	0.002552019	0.105308248	-9.495932356	0.654788786	6.217830015	-3.24731
Adal	Adal-005	179.96742	0.036367913	0.076227836	0.105414529	-9.486358353	0.151870582	1.440698764	-3.245854
Slc12a6	Slc12a6-001	4217.2945	6.02611E-06	0.000109843	0.105464986	-9.481819877	4.292780882	40.7033751	-3.245164
Cyb5a	Cyb5a-002	1418.4098	0.000383995	0.002182874	0.105513419	-9.477467489	1.571125055	14.89028663	-3.244502
Dennd2c	Dennd2c-006	487.72949	1.59034E-05	0.000213856	0.105548469	-9.474320256	0.561289938	5.31784063	-3.244022
Lipa	Lipa-202	5914.1165	0.003396055	0.011818217	0.105588627	-9.470716978	7.554731223	71.54872126	-3.243474
Fbxl3	Fbxl3-005	1826.4443	9.24597E-05	0.00075327	0.105641404	-9.465985475	1.841953174	17.43590199	-3.242753
Mex3d	Mex3d-002	971.53088	3.34955E-05	0.000361443	0.105743376	-9.456857116	1.178621539	11.14605549	-3.241361
Ly6a	Ly6a-201	32231.975	7.43874E-05	0.000646282	0.105776812	-9.453867866	21.02161342	198.7355556	-3.240905
Idh3g	Idh3g-007	511.54122	0.000712391	0.003510201	0.105872402	-9.44533215	0.559065398	5.280558378	-3.239602
Xrn1	Xrn1-007	1761.3388	1.43277E-06	4.36587E-05	0.105889558	-9.443801809	2.081300482	19.65538926	-3.239368

Ulk1	Ulk1-005	907.34509	0.00027881	0.001708104	0.105946085	-9.438763124	1.185875906	11.19320177	-3.238598
10035C23R	0035C23Rik-	4838.1859	1.81379E-06	5.0383E-05	0.105999264	-9.434027756	5.814570361	54.85481818	-3.237874
Ppp1r15b	Ppp1r15b-002	3698.7313	4.99398E-05	0.00048019	0.106061796	-9.428465649	5.182850889	48.86633157	-3.237023
Mecom	Mecom-008	2249.4229	0.000984768	0.004495308	0.106099771	-9.425091043	2.696950369	25.41900276	-3.236507
Ica1l	Ica1l-005	1230	3.33078E-07	1.83999E-05	0.106167728	-9.419058153	1.289743102	12.14816528	-3.235583
Ide	Ide-005	874.36575	0.004925132	0.015812316	0.10619798	-9.416374939	1.013375975	9.542328136	-3.235172
Gm14540	Gm14540-001	249.05097	0.018021743	0.043720406	0.106208334	-9.415456951	0.290398263	2.734232346	-3.235031
P23-132D7	23-132D7.2-(	460	0.000499866	0.002670569	0.106233711	-9.413207792	0.63910116	6.015992021	-3.234686
Gm26789	Gm26789-201	2628	8.98967E-07	3.29397E-05	0.106268143	-9.410157811	3.555575869	33.45853004	-3.234219
Celf2	Celf2-011	1091.458	0.000505302	0.002694052	0.106323385	-9.405268649	1.517126264	14.26898008	-3.233469
Ocrl	Ocrl-004	680.07414	0.007259561	0.021443376	0.106358915	-9.402126728	0.569773179	5.357079636	-3.232987
10044O15R	0044O15Rik8	1749.8793	1.82655E-05	0.000236608	0.106386556	-9.399683941	2.0578812	19.34343287	-3.232612
Cct6a	Cct6a-004	314.96514	0.005339768	0.016873056	0.106408568	-9.397739497	0.566031944	5.319420756	-3.232314
P23-403P24	23-403P24.6-(	876.75359	1.52366E-05	0.000207662	0.106417496	-9.396951021	1.107730406	10.40928837	-3.232193
Glb1l	Glb1l-002	593.05157	0.022585081	0.052223807	0.106443293	-9.394673629	0.415369649	3.902262285	-3.231843
St3gal6	St3gal6-001	4368.5504	0.000746549	0.003631616	0.106464643	-9.392789679	4.804356648	45.12631153	-3.231554
Rps10	Rps10-001	783.76269	0.004721262	0.015285303	0.106465616	-9.392703881	1.263633922	11.86893925	-3.231541
Gm38021	Gm38021-001	628	4.88611E-05	0.000472791	0.106481285	-9.391321697	0.754932428	7.08981329	-3.231328
Snx8	Snx8-003	162.46156	0.036494654	0.076406987	0.106486183	-9.390889696	0.149594808	1.404828337	-3.231262
Bcl2l11	Bcl2l11-001	4106.522	8.87307E-06	0.000142691	0.106537245	-9.386388792	7.605916951	71.39209363	-3.23057
Gm37018	Gm37018-001	317	0.008759628	0.024815324	0.106548993	-9.385353806	0.278928618	2.617843766	-3.230411
Zdhhc24	Zdhhc24-004	2279.2577	2.447E-06	6.18395E-05	0.106567219	-9.383748684	2.500499766	23.46406139	-3.230164
Hat1	Hat1-201	2640.1284	3.15764E-05	0.000347523	0.106579359	-9.382679769	3.941890131	36.98549278	-3.23
Mrps2	Mrps2-004	525.4788	0.042441867	0.086100453	0.106706601	-9.371491417	0.890192933	8.342435434	-3.228279
Cflar	Cflar-006	2261.1516	1.69518E-06	4.81028E-05	0.106753411	-9.367382199	2.293208435	21.48135987	-3.227646
Mastl	Mastl-001	1023.8901	0.001076307	0.00481939	0.106768658	-9.366044516	1.43086311	13.40152759	-3.22744
Ltbp4	Ltbp4-001	21345.164	9.66643E-05	0.000777902	0.106859359	-9.358094712	20.75290449	194.2076458	-3.226215
Calm2	Calm2-001	26181.336	3.51711E-07	1.89107E-05	0.106876323	-9.356609328	36.19652283	338.6767231	-3.225986
Pan3	Pan3-010	662.38617	0.000127635	0.000958875	0.106877657	-9.356492572	0.717390393	6.712257879	-3.225968
Ptpmt1	Ptpmt1-003	932.22242	0.003579926	0.012320383	0.106914674	-9.353253074	1.206518299	11.28487099	-3.225468
Gm7162	Gm7162-001	699	0.000320349	0.001899904	0.106928176	-9.352071984	0.708089529	6.622104244	-3.225286
Mocs2	Mocs2-001	993.79083	4.70153E-05	0.000460726	0.106949897	-9.35017267	0.901365039	8.427918755	-3.224993
Cox17	Cox17-003	2485.7544	5.04879E-05	0.000484238	0.106977464	-9.347763199	3.355531444	31.36671335	-3.224621
Ly6a	Ly6a-001	3260.2543	2.72081E-05	0.000313696	0.106986988	-9.346931071	2.014038099	18.82507528	-3.224493
Rnf4	Rnf4-003	1790.5843	1.39556E-05	0.000195412	0.106989358	-9.346724022	2.429561606	22.70844182	-3.224461
Gm8349	Gm8349-001	175	0.001240493	0.005401332	0.106991343	-9.346550587	0.250632046	2.342545096	-3.224434
Sh3d19	Sh3d19-006	902.2233	2.6183E-05	0.000305335	0.107023567	-9.34373641	0.689809982	6.445402643	-3.224
Usp36	Usp36-201	918.63418	0.008202746	0.023559248	0.10705314	-9.341155216	1.190829874	11.12372669	-3.223601
Platr1	Platr1-001	622	2.38344E-05	0.000284888	0.107113124	-9.335924145	0.741310126	6.920815109	-3.222793
Slc29a1	Slc29a1-001	382.81142	0.001255729	0.005449144	0.107193346	-9.32893726	0.22686558	2.11641476	-3.221713
30028B13R	0028B13Rik-	207	0.001256602	0.005450821	0.107305713	-9.319168304	0.274154043	2.55488767	-3.220201
Synj2	Synj2-002	7389.3811	0.000193414	0.001299159	0.107349287	-9.315385558	8.316066341	77.46736429	-3.219615
Tmem60	Tmem60-002	234.81107	0.005586736	0.017456958	0.10736866	-9.313704737	0.317375865	2.955945099	-3.219355
Rpl31-ps13	pl31-ps13-00	1620	1.29513E-07	1.09569E-05	0.107378631	-9.312839906	2.266945068	21.11169649	-3.219221
Tpr	Tpr-009	4457.6744	7.65948E-06	0.000129294	0.107476781	-9.304335271	5.713732644	53.16248417	-3.217903
Ndufa3	Ndufa3-006	532.99485	0.000172303	0.001193642	0.107548002	-9.298173705	0.73496505	6.833832702	-3.216947
Dmtf1	Dmtf1-002	1040.5233	0.00256428	0.009507101	0.107597992	-9.293853727	1.271513296	11.81725859	-3.216277
Gm37052	Gm37052-001	1499	4.84105E-06	9.57309E-05	0.10759943	-9.293729488	1.711025506	15.9018082	-3.216258
P23-293K21	23-293K21.1-	4595	8.77437E-09	2.42357E-06	0.107689596	-9.285948129	5.822286873	54.0654539	-3.215049
Tfg	Tfg-006	1434.3293	0.011431976	0.030644533	0.107770865	-9.278945673	1.758599423	16.31794851	-3.213961
Gm38143	Gm38143-001	168	0.000961074	0.004413027	0.107881241	-9.269452152	0.200093904	1.854760869	-3.212484
10019D14R	0019D14Rik-	317.89719	0.027027897	0.060106842	0.107883718	-9.269239286	0.444421497	4.119449199	-3.212451
Il1f9	Il1f9-001	916	0.008034096	0.023202603	0.107887656	-9.268900955	1.694184007	15.70322376	-3.212398
Rad54b	Rad54b-003	214.85021	0.029372209	0.064186702	0.107930994	-9.265179187	0.405385715	3.755971291	-3.211819
Ldb2	Ldb2-006	1515.9934	2.51474E-07	1.58908E-05	0.107937825	-9.264592816	1.492473214	13.82715661	-3.211728
Slc35a4	Slc35a4-001	3916.0941	0.008599501	0.024450682	0.107952051	-9.263371957	4.76999339	44.18622301	-3.211537
Supt20	Supt20-002	6660.7192	9.6974E-09	2.51618E-06	0.108131193	-9.248025237	8.081746115	74.74019203	-3.209145
Rnpepl1	Rnpepl1-005	2532.1936	1.4461E-06	4.3932E-05	0.108169761	-9.244727812	2.996229167	27.69932311	-3.208631
Pan3	Pan3-022	2489.5704	1.70491E-06	4.82585E-05	0.108199323	-9.242201973	2.956026732	27.32019609	-3.208237
Vps53	Vps53-008	1205.7301	0.000456902	0.002495347	0.108233749	-9.239262369	1.351345985	12.48544011	-3.207778
Siah1b	Siah1b-001	73.232885	0.023094583	0.053122193	0.108331358	-9.230937543	0.108126171	0.998105934	-3.206477
Disc1	Disc1-004	529.27669	8.62108E-05	0.000715869	0.108374884	-9.227230189	0.671134728	6.192714626	-3.205898
Gm26563	Gm26563-201	388.91436	0.000362741	0.002085684	0.108388104	-9.226104739	0.58009144	5.351984385	-3.205722
Rabl3	Rabl3-003	339.17711	0.00357557	0.012307865	0.108421075	-9.22329907	0.392859748	3.623462951	-3.205283
Copa	Copa-007	1984.4981	6.11689E-05	0.000558527	0.108454299	-9.220473606	2.590723722	23.8876997	-3.204841
Gm27634	Gm27634-201	140	0.003758808	0.012786965	0.108503434	-9.216298207	0.16903197	1.557849044	-3.204187
Abi1	Abi1-009	881.97872	0.004241852	0.014064224	0.108559057	-9.211575997	0.736998423	6.788916983	-3.203448
Metap2	Metap2-001	2823.5405	9.24874E-06	0.000146449	0.108579871	-9.209810177	3.74102178	34.45410047	-3.203171
Gm7665	Gm7665-001	2177	5.73666E-06	0.000106494	0.108676077	-9.201657136	2.698668539	24.83222262	-3.201894
Tm9sf1	Tm9sf1-013	305.52438	0.000930527	0.004307415	0.108755946	-9.194899557	0.402709318	3.702871726	-3.200834
Whsc1	Whsc1-004	2831.4982	9.1846E-06	0.000145973	0.10878002	-9.192864654	3.834315372	35.24834226	-3.200515
Elf5	Elf5-002	363	8.91648E-05	0.000733228	0.108835369	-9.188189517	0.449546618	4.130519523	-3.199781
Man1a2	Man1a2-007	621.80081	0.012095721	0.032012068	0.108849238	-9.187018866	0.805316068	7.398453912	-3.199597
Golga3	Golga3-003	587.83214	0.000119086	0.000911234	0.10886694	-9.185525027	0.684163666	6.284402475	-3.199362
Hectd2	Hectd2-008	491.07654	0.009616478	0.026724032	0.108870155	-9.185253771	0.686384064	6.304611816	-3.19932
Gm17275	Gm17275-201	6230.7197	1.7068E-08	3.24767E-06	0.108917078	-9.18129659	7.4615261	68.50648414	-3.198698
Dgat1	Dgat1-002	665.96618	0.000453425	0.002480401	0.108951151	-9.178425268	0.984555095	9.036665366	-3.198247
Tia1	Tia1-011	2835.7327	3.14524E-07	1.78352E-05	0.109052839	-9.169866736	4.006148967	36.73585215	-3.196901
Armc10	Armc10-003	565.683	0.023927323	0.054590382	0.109060385	-9.169232293	0.753169672	6.90598768	-3.196801
Timeless	Timeless-013	224.0817	0.001058173	0.004757472	0.109149063	-9.161782703	0.294762934	2.700553948	-3.195628
Qprt	Qprt-001	413	8.46502E-05	0.000707657	0.109163975	-9.160531237	0.485194007	4.444634855	-3.195431
Pdcd6ip	Pdcd6ip-005	2267.3798	0.001637395	0.006687429	0.10918889	-9.15844091	2.853244522	26.13127135	-3.195102
Iars	Iars-202	4773.1758	0.000816614	0.003891612	0.109202518	-9.157297998	6.849926536	62.72681856	-3.194922
Gprasp1	Gprasp1-002	524.41153	0.004779181	0.015430521	0.109246134	-9.153642034	0.819017116	7.496989497	-3.194346
Gm26647	Gm26647-001	313	0.003014326	0.010794715	0.109254165	-9.152969141	0.336817577	3.082880886	-3.19424
Cdk8	Cdk8-002	842.15232	0.028952854	0.063438275	0.109259656	-9.152509172	1.413796625	12.93978658	-3.194167
Ipo11	Ipo11-006	2605.0774	0.00035413	0.002048687	0.109262191	-9.152296826	3.203526213	29.31962279	-3.194134
P23-132D7	23-132D7.4-(	581	7.13088E-06	0.000122883	0.109317611	-9.147656936	0.694953543	6.357196597	-3.193402
Pum1	Pum1-202	3562.0023	0.000304055	0.00182796	0.109352701	-9.144721511	5.394414012	49.33041386	-3.192939
Dusp11	Dusp11-002	1815.6127	2.65232E-06	6.52599E-05	0.109355191	-9.144513285	2.031239737	18.57469876	-3.192906
Gm38302	Gm38302-001	475	0.000192063	0.00129262	0.109380175	-9.142424605	0.641785809	5.867478368	-3.192577
Psme2	Psme2-008	2224.7413	0.038865647	0.08027187	0.109448242	-9.136738799	2.44820903	22.36864643	-3.191679
Eif4a2	Eif4a2-002	19444.681	1.28924E-07	1.09569E-05	0.1094699	-9.134931171	26.58789389	242.8785806	-3.191394
Tnfrsf18	Tnfrsf18-001	394.75078	0.0040576	0.013566401	0.109486815	-9.133519837	0.396768485	3.623892828	-3.191171
Syne2	Syne2-004	2170.0872	7.15697E-05	0.000627885	0.109517065	-9.130997103	2.671273295	24.39138872	-3.190772
Trim21	Trim21-202	1380.7533	0.000245384	0.001553495	0.109534298	-9.129560458	1.518159281	13.86012694	-3.190545
Tmem219	Tmem219-002	1248.5457	3.3923E-05	0.000364792	0.109552105	-9.12807651	1.162338876	10.60991819	-3.190311
Gm17057	Gm17057-001	1061	0.000119602	0.000914165	0.10956593	-9.126924746	0.932887563	8.514394586	-3.190129
Itga3	Itga3-002	9290.3691	0.000205317	0.001358071	0.109573229	-9.126316755	11.09132272	101.2229244	-3.190033
Prr11	Prr11-001	1156	0.002758555	0.010058609	0.109592982	-9.12467187	1.773245474	16.18028309	-3.189773
Ghr	Ghr-001	1291.3487	0.034509619	0.073118124	0.109698756	-9.11587365	1.189520304	10.84351679	-3.188381
Kat7	Kat7-005	981.58596	0.001209657	0.005289259	0.109705001	-9.115354727	1.19823806	10.92236497	-3.188299
Medag	Medag-003	870.23797	0.000956625	0.00439909	0.109844054	-9.103815496	1.602031973	14.5846035	-3.186471
Nmd3	Nmd3-009	715.85099	0.013933926	0.035761481	0.109900424	-9.099145982	0.943723938	8.587081879	-3.185731
Gm17669	Gm17669-201	1079	7.99896E-06	0.000133023	0.110012438	-9.089881233	1.638127118	14.89038094	-3.184261
Nmral1	Nmral1-004	441.00099	0.00031853	0.001892069	0.110078383	-9.0844358	0.683098574	6.20556514	-3.183397
Eef1d	Eef1d-011	1499.2744	0.016771871	0.041347196	0.110111991	-9.081663069	2.145884199	19.48819728	-3.182957

Trf	Trf-011	395.22522	0.00398882	0.013391112	0.110145034	-9.078938555	0.664849943	6.036131784	-3.182524
P23-357D17	23-357D17.3-	482	0.000131764	0.000982994	0.110163894	-9.077384263	0.551836297	5.009230122	-3.182277
Gm26682	Gm26682-201	327	0.000645352	0.003253837	0.110169294	-9.076939358	0.402650368	3.654832977	-3.182206
Hnrnpd	Hnrnpd-004	2806.5461	3.56533E-05	0.000377688	0.110213089	-9.073332482	3.775670886	34.25791729	-3.181633
Gm26799	Gm26799-201	356	0.000140476	0.001031801	0.110307056	-9.065603229	0.446714224	4.049733916	-3.180403
Gnai1	Gnai1-001	887	2.62777E-05	0.000305825	0.110307894	-9.065534341	1.232137658	11.16998625	-3.180392
Gm28933	Gm28933-001	4227	2.76989E-06	6.68562E-05	0.110308276	-9.065502958	4.534515179	41.10766077	-3.180387
Npm1	Npm1-004	278.49431	0.00631218	0.019172068	0.110310658	-9.065307158	0.374932255	3.398876059	-3.180356
Aftph	Aftph-008	496.32479	0.00269416	0.009880605	0.110351109	-9.061984105	0.606040388	5.491928367	-3.179827
Rsbn1	Rsbn1-001	5649.6032	4.99491E-06	9.78134E-05	0.110357781	-9.061436275	7.446341007	67.47454452	-3.17974
Ahrr	Ahrr-005	412.24252	4.79818E-05	0.000467267	0.110393124	-9.058535167	0.487149869	4.412864224	-3.179278
Mbnl1	Mbnl1-016	4257.3555	3.19046E-05	0.000349899	0.110394059	-9.058458455	4.479770416	40.5798142	-3.179266
Rian	Rian-003	1636.8287	2.67055E-07	1.62462E-05	0.110397358	-9.058187806	1.580130866	14.31312215	-3.179222
Arhgap6	Arhgap6-007	1382.9311	0.000844205	0.003991994	0.110401283	-9.057865768	1.131612664	10.24999561	-3.179171
Alg6	Alg6-002	1265.3129	4.33679E-06	8.87837E-05	0.110424041	-9.055998932	1.272911162	11.52748212	-3.178874
P24-360B3	24-360B3.2-(	3165	1.92038E-06	5.23254E-05	0.110434015	-9.055181048	3.671161255	33.24302982	-3.178743
Kansl3	Kansl3-005	2025.7758	5.87277E-06	0.000108083	0.110436162	-9.055005041	2.687714067	24.33726443	-3.178715
Rpl7	Rpl7-002	1425.8411	0.003959774	0.013317188	0.110479486	-9.051454091	1.921458261	17.39199124	-3.17815
Dmtf1	Dmtf1-005	1645.9995	1.16385E-05	0.000171615	0.110510289	-9.048931158	1.746126998	15.800583	-3.177747
Gm37160	Gm37160-001	690	0.003028484	0.010836355	0.110597977	-9.04175673	0.934384473	8.448477099	-3.176603
Mff	Mff-003	396.18539	0.037736683	0.07845107	0.110609893	-9.040782596	0.512406984	4.632560145	-3.176448
Ppp1cb	Ppp1cb-004	3422.048	1.24547E-07	1.08038E-05	0.11063321	-9.038877166	3.927264262	35.49805926	-3.176144
Zfyve9	Zfyve9-002	4621.0205	0.000127592	0.000958875	0.110658507	-9.036810875	5.297074326	47.86865887	-3.175814
Elf2	Elf2-001	2950.4087	4.37214E-05	0.000438109	0.11069084	-9.034171183	4.130284827	37.31370016	-3.175392
Gm26543	Gm26543-201	3200	2.98768E-07	1.72269E-05	0.110720992	-9.031711019	3.87645913	35.01105864	-3.174999
Phka1	Phka1-003	684.75825	0.000524015	0.002768822	0.110731706	-9.030837104	0.834830404	7.53921739	-3.17486
Cp	Cp-011	437.10965	0.006222535	0.018989736	0.110732339	-9.030785503	0.58129694	5.249567982	-3.174851
Ahcyl2	Ahcyl2-003	2634.9893	6.00612E-05	0.000551243	0.110736822	-9.030419883	3.151106303	28.45581301	-3.174793
Gm26522	Gm26522-201	276	0.005447747	0.017136099	0.110738645	-9.030271238	0.610264922	5.510857774	-3.174769
Spata6	Spata6-002	2208.943	1.63231E-05	0.00021762	0.110782633	-9.026685588	2.877355644	25.97298472	-3.174196
Gm28192	Gm28192-001	247.02193	0.010184196	0.027992032	0.110845496	-9.021566366	0.229216216	2.067889307	-3.173378
Sik2	Sik2-002	1066.4408	0.032002155	0.068775415	0.110924418	-9.015147613	1.316837353	11.87148312	-3.172351
Brd8	Brd8-001	2035.3259	9.81583E-05	0.000786782	0.110961418	-9.01214148	2.114361582	19.05492571	-3.17187
Mecom	Mecom-015	120.07786	0.007533148	0.022080473	0.111027524	-9.006775675	0.14441268	1.300692615	-3.171011
Apbb2	Apbb2-020	8760.5894	4.76699E-05	0.000465023	0.111111471	-8.999970834	10.36985957	93.32843367	-3.16992
Abi3	Abi3-004	820.97118	3.28727E-05	0.000357085	0.111148296	-8.996988999	1.053796779	9.480998029	-3.169442
Stau2	Stau2-005	303.7794	0.039539397	0.081320144	0.111197722	-8.992989973	0.434628179	3.908606856	-3.168801
Zdhhc4	Zdhhc4-002	367.2482	0.011420116	0.030617533	0.111231093	-8.990291927	0.430360706	3.86906838	-3.168368
Slc2a12	Slc2a12-001	233.00907	0.001417111	0.005987669	0.111235443	-8.989940396	0.345955333	3.110117824	-3.168312
Ormdl1	Ormdl1-002	313.35701	0.00132369	0.00567201	0.111347752	-8.980872836	0.354821358	3.186605493	-3.166856
Uevld	Uevld-004	1434.1177	3.58977E-06	7.89151E-05	0.111362833	-8.979656637	1.738739546	15.61328411	-3.16666
Brox	Brox-001	2142.3601	0.004045639	0.013538168	0.111422685	-8.974833095	2.237069424	20.0773247	-3.165885
Flt1	Flt1-002	8176.1912	0.001372416	0.005836882	0.111508033	-8.967963752	12.84705827	115.2119529	-3.16478
Fam60a	Fam60a-008	546.15628	0.000161771	0.001141491	0.111527232	-8.966419948	0.69242635	6.208585438	-3.164532
Phtf2	Phtf2-001	1175.972	0.00011844	0.00090695	0.111547323	-8.964805	1.307011443	11.71710272	-3.164272
Gm26407	Gm26407-201	289	0.008340636	0.023869215	0.111548278	-8.964728256	0.370778936	3.323932402	-3.16426
Map9	Map9-201	523.55805	0.00015392	0.001101434	0.111637629	-8.957553167	0.860505575	7.708024443	-3.163105
Poglut1	Poglut1-004	334.10949	0.000629674	0.00319312	0.11163805	-8.95751944	0.424705967	3.80431196	-3.163099
Arhgap24	Arhgap24-004	508.87267	0.01932311	0.046242071	0.11164351	-8.957081307	0.707294189	6.33529156	-3.163029
Mga	Mga-006	582.21064	8.28549E-05	0.00069673	0.111650288	-8.956537542	0.872779554	7.817082838	-3.162941
Fxyd5	Fxyd5-012	1682.4091	0.000568211	0.002953593	0.111690082	-8.953346488	1.566179797	14.02255038	-3.162427
N4bp2	N4bp2-007	712.10905	3.15319E-05	0.000347151	0.111704378	-8.952200635	0.883091888	7.905615764	-3.162242
Yes1	Yes1-005	1119.5884	8.63824E-05	0.000716541	0.111711374	-8.951639957	1.264800982	11.322043	-3.162152
Nme7	Nme7-006	295.7336	0.000896648	0.004194036	0.111738827	-8.949440638	0.439122059	3.9298968	-3.161798
Odf2l	Odf2l-007	173.30088	0.005299944	0.016778175	0.11178353	-8.945861712	0.265527137	2.375369046	-3.16122
Zfp369	Zfp369-004	1108.1197	5.44312E-06	0.000103055	0.111850739	-8.940486312	1.460477555	13.05737959	-3.160353
Cxcl12	Cxcl12-004	3669.1646	1.4488E-05	0.000200392	0.11190497	-8.936153585	7.158186634	63.96665515	-3.159654
Chkb	Chkb-008	832.6246	0.001296144	0.005588234	0.111945037	-8.932955168	0.996558361	8.902211164	-3.159138
Apbb2	Apbb2-009	770.0143	0.004627702	0.015059267	0.111957698	-8.931945002	0.762975572	6.814855849	-3.158974
Gm5590	Gm5590-002	868	4.88146E-06	9.62522E-05	0.111959259	-8.931820441	1.079849502	9.645021858	-3.158954
Fam228a	Fam228a-201	144.64543	0.010763287	0.029180921	0.112017042	-8.92721307	0.181427195	1.619639224	-3.15821
Dnah14	Dnah14-003	890.58775	0.000597606	0.003066825	0.112032051	-8.926017055	1.042000753	9.300916491	-3.158017
Spata2	Spata2-002	135.77768	0.035074673	0.074040494	0.112126251	-8.918518089	0.103874504	0.92640664	-3.156804
Dennd2c	Dennd2c-001	280.07423	0.026647257	0.059399191	0.112143721	-8.917128756	0.333065058	2.969984009	-3.156579
Atp10d	Atp10d-003	1342.1599	0.001460295	0.00613231	0.112152739	-8.916411751	1.345304851	11.99529198	-3.156463
Lins	Lins-010	637.15013	0.003421365	0.011892946	0.112156877	-8.916082784	0.939948326	8.380657092	-3.15641
Terf2	Terf2-004	1136.2455	0.000103021	0.000815645	0.112198021	-8.912813144	1.276056521	11.37325334	-3.155881
Gm28424	Gm28424-001	631	7.36648E-05	0.000641989	0.112201956	-8.912500586	0.715845948	6.37997743	-3.15583
Garnl3	Garnl3-003	502.94772	0.001243618	0.005412188	0.112266083	-8.90740973	0.648031225	5.772279639	-3.155006
Exph5	Exph5-003	445	3.4071E-05	0.00036558	0.112283131	-8.906057287	0.515649243	4.592401696	-3.154787
Pds5a	Pds5a-002	3774.0956	4.48583E-07	2.18238E-05	0.112328407	-8.90246759	5.027678187	44.75874211	-3.154205
Dyrk1a	Dyrk1a-005	642.60787	0.000120402	0.000917819	0.11234097	-8.901472029	0.653091378	5.813474632	-3.154044
Tcn2	Tcn2-007	467.09953	0.004824883	0.015551689	0.112356426	-8.900247524	0.211269414	1.880350081	-3.153845
Pakap	Pakap-002	2628.6152	1.25278E-06	4.02135E-05	0.112418091	-8.895365423	3.009449697	26.77015477	-3.153054
Nup210l	Nup210l-201	274.69681	0.000708573	0.003496872	0.112423967	-8.894900537	0.445044278	3.958624586	-3.152978
Hsph1	Hsph1-010	1741.31	0.000860653	0.004054632	0.11248487	-8.890084533	2.831354371	25.1709797	-3.152197
Ptbp3	Ptbp3-001	21681.631	2.20902E-06	5.82441E-05	0.112493535	-8.889399715	28.46892095	253.0716177	-3.152086
Slc26a2	Slc26a2-004	399.13459	0.001186081	0.005210116	0.11252522	-8.886896663	0.459190162	4.080775521	-3.15168
Fbxo11	Fbxo11-003	1863.2356	0.002078446	0.008069232	0.112574333	-8.883019577	2.191238055	19.46481054	-3.15105
Usp48	Usp48-004	3754.8859	2.76584E-07	1.65612E-05	0.112696142	-8.873418205	4.340407155	38.51424787	-3.14949
Parvb	Parvb-002	457.82318	9.33001E-05	0.000758492	0.112743963	-8.869654507	0.452165176	4.010548892	-3.148878
Rab19	Rab19-001	241	0.002040124	0.007953342	0.112755828	-8.868721142	0.366666115	3.251859526	-3.148726
Cops7a	Cops7a-007	640.95447	0.028045251	0.061883124	0.11282248	-8.863481855	0.473432812	4.196263143	-3.147874
Tlk2	Tlk2-003	2248.8611	0.001372681	0.005836882	0.112846941	-8.861560525	2.406874534	21.32866436	-3.147561
Birc6	Birc6-006	313.40505	0.024523387	0.055631607	0.112894555	-8.857823114	0.417297068	3.69634361	-3.146952
P24-481E4	24-481E4.4-(	846	3.3268E-06	7.53092E-05	0.112896194	-8.857694535	1.002901913	8.883398798	-3.146931
Rpia	Rpia-002	7570.5878	4.28558E-09	1.80128E-06	0.11293203	-8.85488378	9.65615277	85.50411054	-3.146473
Nsl1	Nsl1-001	1545.6219	1.77556E-05	0.000231677	0.112933586	-8.8547618	2.025112379	17.93188773	-3.146453
Rbm12	Rbm12-002	3190.9362	6.38552E-05	0.000577056	0.112942403	-8.854070492	4.490084273	39.75552267	-3.146341
Cntn2	Cntn2-003	221.88996	0.001316046	0.005649051	0.112942758	-8.854042704	0.278944909	2.469790139	-3.146336
Cntn2	Cntn2-002	147.9314	0.003531202	0.012186989	0.112976521	-8.851396692	0.144340598	1.277615888	-3.145905
Cntn2	Cntn2-001	100.44072	0.005481799	0.017221759	0.112985996	-8.850654391	0.126321812	1.1180307	-3.145784
Cdk6	Cdk6-001	8924.0015	6.72532E-08	7.59514E-06	0.112989025	-8.85041708	13.82285926	122.3380697	-3.145745
Cycs	Cycs-002	206.94744	0.03185432	0.068539418	0.113152692	-8.837615605	0.402802917	3.559817345	-3.143657
Rab23	Rab23-009	171.99423	0.031318697	0.067637642	0.113190925	-8.83463049	0.179930064	1.589615633	-3.14317
Asap2	Asap2-001	15039.972	4.212E-06	8.75182E-05	0.113287314	-8.827113692	17.4393506	153.9391304	-3.141942
Gm37730	Gm37730-001	235	0.016834076	0.041461752	0.113337029	-8.823241727	0.394503734	3.480801809	-3.141309
Pan3	Pan3-023	3092.384	3.81131E-07	2.00025E-05	0.113359197	-8.821516284	4.067702805	35.88330653	-3.141027
Mecom	Mecom-013	112.61493	0.016259733	0.040310955	0.113369428	-8.82072014	0.129542887	1.142661556	-3.140896
Pabpc1	Pabpc1-002	930.31563	0.000103831	0.000819427	0.113395907	-8.818660421	1.343253939	11.84570034	-3.14056
Agpat1	Agpat1-008	444.74293	0.012535267	0.032896552	0.113423746	-8.816495992	0.606925394	5.350955302	-3.140205
Rbm6	Rbm6-006	160.35818	0.001975704	0.007761577	0.113477522	-8.812317919	0.243693912	2.147508226	-3.139522
Gm37569	Gm37569-001	591	6.39093E-05	0.000577088	0.113484114	-8.811806057	0.887880145	7.823827636	-3.139438
Epb4.1	Epb4.1-011	1038.9584	0.002045566	0.007969586	0.11349841	-8.810696143	1.370921654	12.07877413	-3.139256
Capg	Capg-002	222.90191	0.033603478	0.071565672	0.113554262	-8.806362586	0.378159066	3.33020585	-3.138546
AV026068	AV026068-00	147.68839	0.004321782	0.01427181	0.113557961	-8.806075717	0.213380006	1.879040491	-3.138499
Themis2	Themis2-001	1375	0.046315395	0.092327763	0.113561215	-8.805823331	3.229553476	28.43887735	-3.138458

Tead1	Tead1-201	14809.682	0.000127101	0.00095696	0.113619767	-8.801285468	23.01090015	202.5255011	-3.137714
Gm28017	Gm28017-201	124	0.005886578	0.018173364	0.113619919	-8.801273672	0.135407476	1.191758253	-3.137712
Cox5b	Cox5b-002	1202.5464	0.000576494	0.00298397	0.113620582	-8.80122232	1.586069206	13.9593477	-3.137704
Gm5345	Gm5345-001	674	0.006588794	0.019830069	0.11362467	-8.800905673	0.923933136	8.131448377	-3.137652
Gm12114	Gm12114-001	64	0.02336596	0.05359883	0.113630095	-8.800485432	0.100096952	0.880901768	-3.137583
Polg2	Polg2-003	873.59409	5.14476E-05	0.000491652	0.113668616	-8.797503075	1.006253055	8.852514344	-3.137094
Cops4	Cops4-003	429.44184	0.001249447	0.005429967	0.113684332	-8.796286892	0.675690659	5.94356889	-3.136895
Git1	Git1-001	1911.1617	4.40453E-05	0.000440324	0.113703844	-8.794777438	1.922428099	16.90732727	-3.136647
Ifi47	Ifi47-201	2156.8979	0.000509159	0.002708292	0.113755464	-8.790786491	3.425790441	30.11539233	-3.135992
Supt7l	Supt7l-004	677.42399	3.27957E-05	0.000356475	0.113755661	-8.790771286	0.801745982	7.047965557	-3.13599
Ndrg3	Ndrg3-002	591.58879	0.048158034	0.095264679	0.113769681	-8.789687987	0.414843811	3.64634766	-3.135812
Pick1	Pick1-005	500.00084	0.014927077	0.037750306	0.113787099	-8.788342487	0.517817604	4.550758451	-3.135591
Ppid	Ppid-001	4128.7081	3.43469E-05	0.000366931	0.113800606	-8.78729944	6.52851872	57.36804889	-3.13542
Kdm5a	Kdm5a-001	12340.527	7.68934E-08	8.28176E-06	0.11384866	-8.783590417	15.953877	140.1323212	-3.134811
Dennd5a	Dennd5a-002	10761.755	8.1535E-06	0.000134777	0.113862148	-8.782549929	13.70063204	120.3264849	-3.13464
Atp2a3	Atp2a3-002	5233.3389	0.002233015	0.008544381	0.113870783	-8.781883938	3.929177108	34.50557734	-3.13453
Gm4335	Gm4335-001	238	0.001306214	0.005619624	0.113885377	-8.780758538	0.398738643	3.501227744	-3.134346
Cchcr1	Cchcr1-003	294.04506	0.000484542	0.002609777	0.113917014	-8.778320015	0.379745544	3.333527912	-3.133945
Jmjd1c	Jmjd1c-004	1491.0454	4.64424E-06	9.30909E-05	0.113944383	-8.776211459	1.944184655	17.06257564	-3.133598
Sun2	Sun2-006	2526.4302	0.01535907	0.038589214	0.113957962	-8.775165701	2.918260823	25.60822228	-3.133426
Zfp456	Zfp456-001	653.67578	0.000146462	0.001061658	0.11395985	-8.775020306	0.854714728	7.500139097	-3.133402
Ndufaf1	Ndufaf1-201	200.82173	0.027129947	0.060314208	0.114050479	-8.768047313	0.239014449	2.095690001	-3.132256
Fbxo3	Fbxo3-005	1933.8378	0.00285953	0.010358423	0.114071986	-8.76639426	2.374602151	20.81669866	-3.131984
Ptbp3	Ptbp3-012	1992.2559	0.000206259	0.001363515	0.114107968	-8.76362992	2.908175456	25.48617344	-3.131529
Lamp2	Lamp2-004	2117.4494	8.47676E-06	0.000138252	0.114156718	-8.759887433	3.355020151	29.38959886	-3.130912
Ttbk2	Ttbk2-002	2974.7505	1.71078E-05	0.000225709	0.114165292	-8.759229581	3.293725719	28.85049975	-3.130804
Tsc22d3	Tsc22d3-003	3850.0948	0.000209078	0.001377101	0.11423854	-8.753613302	2.955461059	25.87096324	-3.129879
Zfp407	Zfp407-006	977.117	0.000350662	0.002034246	0.114242984	-8.753272745	1.195480752	10.46436908	-3.129823
Taf1	Taf1-201	1293.9989	0.000235602	0.001507864	0.11426003	-8.751966894	1.70201434	14.89597315	-3.129607
Rps9	Rps9-008	218.91247	0.016435505	0.040652564	0.114315676	-8.747706637	0.295973333	2.58908789	-3.128905
Med23	Med23-004	668.14068	6.44143E-05	0.000580729	0.114331066	-8.746529133	0.721697488	6.312348107	-3.128711
P23-446B13	23-446B13.7-	2261	2.34487E-07	1.55411E-05	0.11434747	-8.745274345	2.723055091	23.81386383	-3.128504
RP23-42O8.	23-42O8.7-0	722	1.32694E-05	0.000188658	0.114361371	-8.744211351	0.910862476	7.964774002	-3.128328
Zc3hc1	Zc3hc1-007	2904.8159	1.16135E-07	1.04255E-05	0.11437513	-8.743159455	3.554925643	31.08128175	-3.128155
Rps5	Rps5-004	259.37991	0.032075279	0.068906624	0.114391287	-8.741924544	0.426569174	3.729035533	-3.127951
Gm23681	Gm23681-201	180	0.001061262	0.00476698	0.114520179	-8.732085534	0.231425671	2.020828751	-3.126326
Srp19	Srp19-002	1546.3878	0.000194958	0.001306973	0.114528986	-8.731414065	2.110259138	18.42554632	-3.126215
P23-182M12	23-182M12.4-	431	0.000196344	0.001314206	0.114544275	-8.730248666	0.756618458	6.605467281	-3.126023
Prrc2c	Prrc2c-003	8577.9746	9.23063E-05	0.000752854	0.114548583	-8.729920312	10.12251003	88.36870588	-3.125968
Tmx1	Tmx1-003	428.34561	0.009962145	0.027496451	0.114561704	-8.72892046	0.60105481	5.24655963	-3.125803
Tcf12	Tcf12-003	1629.1669	0.014298416	0.036500018	0.114564941	-8.728673845	2.570646375	22.43833378	-3.125762
Sbno1	Sbno1-010	650.49113	0.001253354	0.00544418	0.114732629	-8.715916409	0.845647631	7.370594067	-3.123652
Gbe1	Gbe1-201	427.47822	0.010488198	0.028643712	0.114781973	-8.712169438	0.620838719	5.408852112	-3.123032
Atp7a	Atp7a-003	540.62417	8.00013E-05	0.000680926	0.114804705	-8.710444378	0.693826279	6.043535212	-3.122746
Btbd10	Btbd10-003	315.85912	0.005456859	0.017160723	0.114835481	-8.708110016	0.336697679	2.932000435	-3.12236
Gm22907	Gm22907-201	155	0.00098559	0.004498461	0.114916839	-8.701944864	0.201510101	1.753529786	-3.121338
Ubap2l	Ubap2l-022	303.24885	0.003566354	0.012284787	0.11497085	-8.69785687	0.449828964	3.912547948	-3.12066
Becn1	Becn1-001	1128.0949	1.52652E-05	0.000207968	0.114985659	-8.696736684	1.443453649	12.5533363	-3.120474
Ttll4	Ttll4-009	545.3235	0.002349123	0.008880515	0.11498884	-8.696496093	0.839261633	7.298635514	-3.120434
Ttf1	Ttf1-001	1761.7052	0.00069459	0.003444789	0.115005559	-8.695231868	1.761170377	15.31378478	-3.120224
30416P10R	0416P10Rik-	470.0027	0.001073031	0.004808493	0.115032503	-8.693195212	0.818552201	7.115834075	-3.119887
Gm37558	Gm37558-001	2730	7.00321E-08	7.79249E-06	0.115056223	-8.691402964	3.399645821	29.54769177	-3.119589
30555F03R	0555F03Rik-	683	9.87754E-06	0.000153288	0.115126389	-8.686105833	0.855658166	7.432337383	-3.11871
Bcl10	Bcl10-001	3986.4013	9.21126E-06	0.000146108	0.11514777	-8.684492972	6.808635795	59.12954971	-3.118442
Zfyve27	Zfyve27-002	566.70106	0.00033872	0.001981747	0.115191423	-8.681201931	0.832937075	7.230894943	-3.117895
Cd300a	Cd300a-001	2099.16	0.0043999	0.014471966	0.115254462	-8.676453641	4.583429185	39.76791084	-3.117105
Ube3a	Ube3a-007	610.2273	7.23791E-05	0.000633175	0.115293197	-8.673538628	0.764983942	6.635117771	-3.116621
Tcf4	Tcf4-035	3035.3039	9.22296E-06	0.000146108	0.115319262	-8.671578201	5.083020199	44.07780715	-3.116295
Brap	Brap-008	1503.9371	1.10464E-07	1.00356E-05	0.115385545	-8.666596811	1.936649567	16.78416096	-3.115466
Enthd2	Enthd2-004	923.17966	2.49307E-05	0.000294554	0.115389594	-8.666292698	1.017019387	8.813787687	-3.115415
Gm37768	Gm37768-001	319	0.000797052	0.00381858	0.115392068	-8.666106898	0.440239938	3.815166368	-3.115384
Atf7ip	Atf7ip-005	969	3.44985E-06	7.69755E-05	0.115406648	-8.665012118	1.332079554	11.54248547	-3.115202
Aldh18a1	Aldh18a1-007	533.58096	0.021131925	0.049598981	0.11541251	-8.664571964	1.053773216	9.130493867	-3.115128
Eif2s3y	Eif2s3y-003	2780.5893	4.70576E-06	9.40877E-05	0.115470258	-8.66023871	3.752355502	32.49629437	-3.114407
Gm38365	Gm38365-001	1469	3.45554E-06	7.70022E-05	0.115495502	-8.658345817	2.181959251	18.89215775	-3.114091
10051M20R	0051M20Rik-	898.54603	7.46918E-05	0.000647644	0.115580289	-8.65199428	1.184944595	10.25213385	-3.113033
Rcbtb1	Rcbtb1-003	1629.8544	0.008326698	0.023836287	0.115603623	-8.650247933	2.004338476	17.33802476	-3.112741
Stat1	Stat1-012	852.38676	0.000397605	0.002240167	0.115609242	-8.649827486	1.024739232	8.863817572	-3.112671
Paxbp1	Paxbp1-001	5531.4709	5.24843E-07	2.38407E-05	0.11566801	-8.645432732	6.962862614	60.19696035	-3.111938
Gm37049	Gm37049-001	198	0.001402094	0.005941813	0.115794334	-8.636001111	0.265656863	2.294212962	-3.110363
Wdr26	Wdr26-002	37082.167	6.80585E-10	7.28439E-07	0.115802059	-8.635425046	43.52217612	375.8324897	-3.110267
Usp34	Usp34-201	16598.54	6.85895E-09	2.13967E-06	0.115821878	-8.633947404	19.53725172	168.6836038	-3.11002
Trim24	Trim24-008	1626.0324	6.42246E-06	0.000115107	0.115833784	-8.633059906	2.353331684	20.3164534	-3.109872
Gm2445	Gm2445-001	401	5.73982E-05	0.000532761	0.115909023	-8.627456039	0.56416449	4.867304337	-3.108935
Depdc5	Depdc5-201	382.33247	0.001984562	0.007787723	0.115920355	-8.626612665	0.487627731	4.206575561	-3.108794
Ino80d	Ino80d-005	927.84283	5.1064E-05	0.000488395	0.115955521	-8.623996444	1.278853718	11.02882992	-3.108357
Cep295	Cep295-012	1145.3808	7.01883E-05	0.000618618	0.115957372	-8.623858811	1.451757354	12.51975045	-3.108334
Apbb2	Apbb2-014	718.01923	0.033525886	0.071440403	0.116089943	-8.614010572	1.025385891	8.83268491	-3.106685
Prokr2	Prokr2-001	1229.1966	0.002169189	0.008338353	0.116130304	-8.611016805	1.558482023	13.42011489	-3.106184
Polg2	Polg2-004	227.16138	0.005535009	0.017342793	0.116134953	-8.610672121	0.301795213	2.598659631	-3.106126
Ehmt1	Ehmt1-012	189	0.014611633	0.037130763	0.116182335	-8.60716044	0.296112099	2.548684342	-3.105537
Nf1	Nf1-007	1038.9368	0.000210422	0.00138426	0.116223091	-8.604142219	1.577870598	13.57622303	-3.105031
Tet1	Tet1-002	872.86838	0.005628956	0.017556848	0.116231713	-8.603503969	1.098646682	9.452211092	-3.104924
Ddit3	Ddit3-003	516.40215	0.000394499	0.002227672	0.116265507	-8.601003255	0.847512982	7.289461914	-3.104505
Psmb4	Psmb4-005	268.04316	0.003771859	0.012819905	0.116275794	-8.600242271	0.38492824	3.310476119	-3.104377
Prrc2c	Prrc2c-015	1254.2074	7.78141E-06	0.00013058	0.116367171	-8.59348893	1.560062862	13.40638293	-3.103244
Supt20	Supt20-006	324.86719	0.001541359	0.006385043	0.116405243	-8.590678378	0.441946347	3.796618923	-3.102772
Mgst1	Mgst1-002	1732.5214	0.012035835	0.031895995	0.116411323	-8.590229651	2.362294898	20.29265568	-3.102697
Fam73a	Fam73a-005	605.31575	8.20002E-05	0.000692953	0.116432259	-8.588685037	0.803119607	6.897741349	-3.102437
Cd300ld	Cd300ld-001	1421	0.029249031	0.063970581	0.116446391	-8.587642668	3.383850844	29.05930189	-3.102262
Cisd3	Cisd3-002	263.03314	0.021191945	0.049710576	0.116455401	-8.58697831	0.286966246	2.464172931	-3.102151
Ctcfl	Ctcfl-001	1791.0342	1.24599E-07	1.08038E-05	0.116468172	-8.586036676	2.3303471	20.00844567	-3.101992
Syne2	Syne2-005	964.77402	8.37841E-05	0.000701958	0.116472747	-8.585699467	1.212819963	10.41290771	-3.101936
Kbtbd2	Kbtbd2-002	2525.7442	0.001259122	0.005459464	0.116479466	-8.585204211	2.88193763	24.74202308	-3.101852
Cyp20a1	Cyp20a1-003	3878.2792	5.08098E-07	2.34487E-05	0.116515586	-8.582542781	5.196138659	44.59608233	-3.101405
Cd99l2	Cd99l2-006	5983.4384	4.32572E-05	0.000434858	0.116557328	-8.579469179	6.846105252	58.73594901	-3.100888
P23-434H17	23-434H17.3-	1047	4.57967E-06	9.22965E-05	0.116567749	-8.57870216	1.298510116	11.13953153	-3.100759
230072C01R	0072C01Rik-	467.80928	9.2726E-05	0.00075526	0.116576042	-8.578091867	0.638391093	5.476177446	-3.100657
Ube2k	Ube2k-008	1886.5902	2.21506E-06	5.82686E-05	0.11667977	-8.57046595	2.878083951	24.6665205	-3.099374
Tnpo1	Tnpo1-002	5796.9828	3.56736E-05	0.000377688	0.116759258	-8.564631308	8.302044982	71.10395438	-3.098391
Trim25	Trim25-002	4547.7678	1.32085E-07	1.1088E-05	0.116769757	-8.563861281	5.126084611	43.89907752	-3.098261
Mbtd1	Mbtd1-005	1058.2273	0.033735538	0.071793349	0.116791181	-8.56229033	1.822823724	15.60754594	-3.097997
Cript	Cript-001	3855.3351	4.17091E-08	5.58022E-06	0.116834425	-8.559121202	4.521278807	38.6981733	-3.097463
Rbm28	Rbm28-003	397.4933	0.001647706	0.006720725	0.116853157	-8.557749119	0.533602577	4.566436985	-3.097231
Mapk1ip1l	Mapk1ip1l-20	9496.6253	2.82298E-07	1.66746E-05	0.116865043	-8.556878708	13.35232497	114.2542252	-3.097085
Rps15a-ps5	ps15a-ps5-00	878	1.23925E-06	4.00553E-05	0.116919942	-8.552860896	1.300667074	11.12442455	-3.096407
Ublcp1	Ublcp1-005	855.73616	0.038771937	0.080136313	0.116926921	-8.552350395	1.173695034	10.03785119	-3.096321

Snx14	Snx14-002	1115.3106	1.2906E-05	0.000184333	0.117020552	-8.545507475	1.453563923	12.42144137	-3.095166
Prx	Prx-002	237.63297	0.009914762	0.027385531	0.117061967	-8.542484181	0.161349837	1.378328426	-3.094656
Gm11599	Gm11599-001	124	0.009472639	0.026428631	0.117171795	-8.534477119	0.18176406	1.551261207	-3.093303
Ralgps1	Ralgps1-001	258.02016	0.00726637	0.021456433	0.117187767	-8.533313919	0.576787731	4.921910772	-3.093106
AI314180	AI314180-008	907.99453	0.000155648	0.001110382	0.117271899	-8.527192002	1.057557032	9.017991867	-3.092071
Cul1	Cul1-008	889.98479	0.001026639	0.004649879	0.117353742	-8.521245119	1.073516916	9.147700781	-3.091064
P24-104D12	24-104D12.2-	234	0.003393269	0.01181212	0.117359541	-8.520824084	0.40440343	3.445850486	-3.090993
Zcchc7	Zcchc7-005	989.99665	0.010262314	0.028163802	0.117422503	-8.516255221	1.272831942	10.83976167	-3.090219
Atp8a1	Atp8a1-001	945.65173	0.014600381	0.037104922	0.117426204	-8.515986765	1.475804049	12.56792775	-3.090174
Ints1	Ints1-006	441.42722	0.025765219	0.057813074	0.117497974	-8.51078506	0.566738375	4.823388497	-3.089292
Cd320	Cd320-002	357.43031	0.035470874	0.074628696	0.117552691	-8.506823568	0.633693776	5.39072115	-3.088621
Pik3c3	Pik3c3-002	1972.8314	8.59906E-05	0.000714849	0.117588294	-8.504247872	2.673492649	22.73604417	-3.088184
Nf1	Nf1-006	2171.5145	5.39281E-06	0.000102329	0.117617309	-8.502149968	2.748451831	23.36774965	-3.087828
Pikfyve	Pikfyve-003	952.46409	4.97789E-05	0.000479316	0.117644009	-8.500220332	1.431715374	12.16989613	-3.0875
Nbeal2	Nbeal2-004	1310.9202	0.045811312	0.091515744	0.117696648	-8.496418692	1.277068562	10.8505092	-3.086855
Hnrnpd	Hnrnpd-003	4457.3908	0.000398328	0.002242856	0.117761488	-8.491740557	7.299141075	61.98241229	-3.08606
Bcas2	Bcas2-002	2319.8537	2.93995E-05	0.00033109	0.117765859	-8.491425364	3.049923613	25.89819873	-3.086007
Nsun6	Nsun6-201	84.39219	0.012118164	0.032054921	0.117771187	-8.491041172	0.058357271	0.495513994	-3.085941
42624	Sept11-005	1295.1501	0.002433032	0.009123205	0.117853706	-8.485095948	0.96717657	8.206585997	-3.084931
Ggct	Ggct-003	816.88592	0.00212145	0.008191618	0.11785447	-8.485040935	1.437717345	12.19909052	-3.084922
Pnpt1	Pnpt1-002	1598.0711	4.81546E-05	0.00046855	0.117859892	-8.484650602	2.261945365	19.1918161	-3.084855
Rbm39	Rbm39-001	8600.7601	0.000194856	0.001306541	0.117888875	-8.482564599	12.35131594	104.7708353	-3.084501
Snhg6	Snhg6-004	390.55958	0.003015904	0.01079811	0.11793363	-8.479345567	0.630803053	5.348797069	-3.083953
R3hdm1	R3hdm1-002	855.63805	0.021172343	0.049667996	0.118015698	-8.473448979	0.873434326	7.401001197	-3.082949
D5Ertd579e	5Ertd579e-00	763.9623	4.90739E-05	0.000474127	0.118131495	-8.465143005	0.846564871	7.166292697	-3.081534
Acer3	Acer3-003	615.25693	0.021340971	0.049984836	0.118160788	-8.463044448	0.906593527	7.672541314	-3.081177
Gabarap	Gabarap-002	3001.0219	0.000117742	0.000902969	0.11818971	-8.460973486	4.238547267	35.86223604	-3.080824
00038G22R	0038G22Rik-	556	3.40484E-05	0.000365452	0.11832638	-8.451200841	0.947981118	8.011578818	-3.079156
Erc1	Erc1-201	4833.5786	2.6108E-05	0.000304593	0.118353558	-8.449260172	5.593578432	47.26159947	-3.078825
Gm27956	Gm27956-201	380	0.000377975	0.002155811	0.11836216	-8.448646069	0.502040623	4.241563533	-3.07872
Pbx3	Pbx3-002	970.75771	0.000249436	0.001573251	0.118391274	-8.446568471	1.394220125	11.77637575	-3.078365
Atad1	Atad1-201	8592	8.39426E-09	2.36288E-06	0.11843373	-8.443540552	11.69454952	98.74340314	-3.077848
Gm37436	Gm37436-001	543	7.89562E-05	0.000673705	0.118451878	-8.442246888	0.814899337	6.879581395	-3.077627
Ophn1	Ophn1-006	330.27097	0.013691809	0.035266937	0.118453304	-8.442145298	0.210108872	1.773769625	-3.07761
Gulp1	Gulp1-001	1744.2052	2.28087E-05	0.000276824	0.118475186	-8.440586039	2.239387223	18.90174053	-3.077343
Osbpl3	Osbpl3-003	4498.7682	1.08978E-05	0.000164538	0.118475308	-8.440577355	7.499226334	63.29779997	-3.077342
Mbnl1	Mbnl1-024	242.83818	0.005470548	0.017193772	0.11856002	-8.434546483	0.166199781	1.401819776	-3.07631
Gm14120	Gm14120-001	183	0.003732634	0.01272648	0.11857215	-8.433683605	0.246450487	2.078485436	-3.076163
Gigyf2	Gigyf2-018	168.9566	0.001693253	0.006858314	0.118580968	-8.433056506	0.217996189	1.838374179	-3.076056
Ckap5	Ckap5-002	1870.0031	0.003719123	0.012692303	0.118600792	-8.431646925	1.849243683	15.59216981	-3.075814
Desi2	Desi2-001	11509.451	2.929E-09	1.51021E-06	0.118665463	-8.42705176	14.50591236	122.2420743	-3.075028
Hey1	Hey1-001	638.95663	0.001516244	0.006303691	0.118677915	-8.42616756	0.75963656	6.400824942	-3.074877
Cask	Cask-003	4578.2251	4.52893E-06	9.17303E-05	0.11868633	-8.425570124	5.364786829	45.20138763	-3.074774
Dalrd3	Dalrd3-002	697.81023	5.602E-05	0.000523087	0.118710713	-8.423839525	0.799037606	6.73096457	-3.074478
Ubr3	Ubr3-005	437.41845	0.007703352	0.02244872	0.118729112	-8.422534159	0.543363317	4.5764961	-3.074254
Rpap1	Rpap1-006	731.36382	0.000141376	0.001036855	0.118739407	-8.421803903	0.907183296	7.640119825	-3.074129
Mrgprb1	Mrgprb1-001	62	0.01295251	0.033750747	0.118780609	-8.418882547	0.050098476	0.421773185	-3.073629
Rbm25	Rbm25-010	142.71618	0.010107335	0.027820962	0.118811093	-8.416722524	0.236509487	1.990634729	-3.073259
Abi2	Abi2-011	580.74272	0.000140291	0.001031111	0.118814479	-8.416482664	0.865490791	7.284388241	-3.073217
P23-72O23	23-72O23.4-(	850	0.001316387	0.0056492	0.118837371	-8.414861364	1.078378249	9.074403467	-3.07294
Tm4sf1	Tm4sf1-005	13278.149	2.26975E-09	1.34808E-06	0.118926922	-8.408525054	23.52186624	197.7842016	-3.071853
Arhgap9	Arhgap9-201	824	0.007276977	0.02147395	0.11899127	-8.403977865	1.942784142	16.32711492	-3.071072
Fam65b	Fam65b-006	1384.4421	0.001031859	0.004666874	0.118998979	-8.403433434	2.082516368	17.50028768	-3.070979
Oxr1	Oxr1-201	2038.452	0.000260187	0.001622563	0.119011253	-8.402566794	3.247382073	27.28634477	-3.07083
Nfrkb	Nfrkb-006	2154.6069	5.24273E-06	0.000100601	0.119087966	-8.39715407	2.860611126	24.02099236	-3.0699
Mmp16	Mmp16-002	396.16029	0.030017285	0.065366989	0.119118425	-8.395006892	0.479686961	4.026975341	-3.069532
Birc6	Birc6-017	709.13584	0.000100448	0.000799433	0.119135114	-8.393830881	1.106936752	9.291439888	-3.069329
Rnps1	Rnps1-202	870.16928	0.000808776	0.003863665	0.119161787	-8.391952058	1.127941781	9.465633347	-3.069006
Mdm4	Mdm4-007	738.02487	2.00804E-05	0.00025313	0.119195655	-8.38956755	0.940378386	7.88936799	-3.068596
Sema6d	Sema6d-005	2351.4717	3.726E-06	8.05481E-05	0.11923637	-8.38670283	3.053536049	25.60909943	-3.068104
Immt	Immt-007	1839.6813	6.50526E-06	0.000115754	0.119278491	-8.383741234	2.457500759	20.60305045	-3.067594
Sun1	Sun1-002	1278.9368	0.002771916	0.01009712	0.119329235	-8.380176048	1.67241319	14.01511696	-3.066981
Gm20616	Gm20616-002	130.33967	0.021966145	0.051117032	0.119355001	-8.378366985	0.170274251	1.42662016	-3.066669
Zeb2	Zeb2-001	8327.7032	4.27963E-07	2.11533E-05	0.119395138	-8.375550405	12.56666099	105.2527025	-3.066184
Zcchc7	Zcchc7-001	6088.5556	9.0006E-06	0.000144119	0.119414786	-8.374172382	8.76959205	73.43807555	-3.065947
Gm2415	Gm2415-001	1021.6933	0.003753786	0.012774204	0.119428517	-8.373209523	1.250668598	10.47211021	-3.065781
Exoc1	Exoc1-007	1127.2489	0.000126355	0.000952602	0.119469103	-8.370365039	1.753405426	14.67664348	-3.065291
Ubn2	Ubn2-005	861.38912	0.003973337	0.013349696	0.119523338	-8.366566895	1.10381296	9.235124969	-3.064636
Rsu1	Rsu1-003	504.92642	0.000212943	0.001397183	0.119584202	-8.362308607	0.672690352	5.625244318	-3.063901
Gm26740	Gm26740-002	276.62616	0.016000857	0.03979879	0.119611125	-8.360426311	0.588685106	4.92165845	-3.063577
Csnk1e	Csnk1e-010	336.19034	0.000611901	0.003124271	0.11961827	-8.359926953	0.421578631	3.52436656	-3.06349
Sorbs2	Sorbs2-001	1322.8769	0.012543744	0.032912652	0.119643735	-8.358147623	1.920424662	16.05119282	-3.063183
Il6st	Il6st-003	681.49111	0.006498324	0.019611209	0.11968529	-8.355245634	1.242783003	10.38375726	-3.062682
Sccpdh	Sccpdh-002	244.88441	0.000463277	0.002521409	0.119733658	-8.351870434	0.317196184	2.649181433	-3.062099
Morc4	Morc4-006	485.4861	0.033866639	0.072034213	0.119851459	-8.343661435	0.651398184	5.435045905	-3.060681
H2-Q7	H2-Q7-002	752.56024	0.000666166	0.003334725	0.119872035	-8.342229293	0.863818645	7.206173207	-3.060433
Fip1l1	Fip1l1-001	1803.4015	0.005178906	0.016451265	0.119988774	-8.334113014	2.2186866	18.49078487	-3.059029
Gm38147	Gm38147-001	988	2.66844E-05	0.000308972	0.120009077	-8.332703008	1.300667074	10.83807244	-3.058785
Zeb2	Zeb2-006	1616.4457	3.23174E-05	0.000353294	0.120060638	-8.329124458	2.642661738	22.01105851	-3.058165
Ywhae	Ywhae-001	58049.588	5.28699E-08	6.53717E-06	0.12007642	-8.328029795	86.45409786	719.9923021	-3.057975
Hnrnpu	Hnrnpu-004	4881.9103	1.21734E-05	0.00017727	0.120082492	-8.32760864	7.497718719	62.43806718	-3.057902
Rnf38	Rnf38-004	11324.583	0.000168754	0.001175715	0.120103774	-8.326133003	14.77481161	123.0170466	-3.057647
Srsf11	Srsf11-002	2099.8956	0.013742351	0.035386477	0.120137668	-8.323784031	3.297412886	27.44695272	-3.05724
Gm37198	Gm37198-001	1198	4.94154E-06	9.69902E-05	0.120149809	-8.322942869	1.580777825	13.15672352	-3.057094
Fxr1	Fxr1-006	1295.9505	3.35878E-06	7.55836E-05	0.120152235	-8.322774868	1.46490163	12.19204647	-3.057065
Gm12447	Gm12447-001	283	0.000246883	0.001561353	0.120154787	-8.322598105	0.349549666	2.909161388	-3.057034
430110C17R	0110C17Rik-	2021	2.11519E-06	5.65094E-05	0.120168279	-8.321663666	2.720370443	22.63800787	-3.056872
Prpf38b	Prpf38b-008	3039.3002	4.23827E-06	8.76574E-05	0.12018127	-8.320764099	4.495494618	37.40595022	-3.056716
Erc1	Erc1-009	6120.6872	1.77559E-05	0.000231677	0.120186309	-8.320415281	7.037853946	58.55786752	-3.056656
Skint8	Skint8-001	488	0.000494582	0.002650421	0.120222129	-8.317936195	0.657700838	5.470713603	-3.056226
30006D01R	0006D01Rik-	820	2.41526E-05	0.000287631	0.120249554	-8.316039138	1.086861748	9.038384837	-3.055897
Mllt10	Mllt10-201	3810.6474	0.007352439	0.021656437	0.120376231	-8.307287872	4.633924391	38.4953439	-3.054378
Pls3	Pls3-003	5022.0959	0.00026065	0.001624464	0.120420783	-8.304214381	7.903038732	65.6285279	-3.053844
Tmem39a	Tmem39a-007	623.04645	0.001194988	0.005238147	0.120428103	-8.303709667	0.885553386	7.353378211	-3.053756
Pltp	Pltp-002	1496.7269	0.041141351	0.083994472	0.120477663	-8.300293829	0.832379254	6.908992382	-3.053162
Ocrl	Ocrl-005	2302.0681	0.007500353	0.022007297	0.120483987	-8.299858172	3.049309604	25.30883724	-3.053087
Zfp951	Zfp951-201	293.36073	0.015303517	0.038475039	0.120559882	-8.294633203	0.390902789	3.242395252	-3.052178
Heg1	Heg1-006	49575.023	1.47598E-06	4.44277E-05	0.120566462	-8.294180486	47.84456985	396.8314976	-3.052099
Aff4	Aff4-002	1522.3302	1.09465E-05	0.000164816	0.120568816	-8.294018605	2.000164268	16.58939965	-3.052071
Pot1a	Pot1a-001	2318.9568	0.000231047	0.001485801	0.120574147	-8.293651901	3.088611837	25.61587143	-3.052007
Vamp3	Vamp3-001	17037.753	3.25718E-09	1.51021E-06	0.120580922	-8.293185871	18.6307646	154.5083938	-3.051926
P24-362N9	24-362N9.3-(	514	3.60586E-05	0.000380236	0.120643506	-8.288883757	0.646304232	5.35714065	-3.051178
700034J05R	0034J05Rik-(	557.59662	0.000425849	0.002361995	0.120667276	-8.287250983	0.889668224	7.372903864	-3.050894
Smc2	Smc2-001	2534.0389	0.001770372	0.007116812	0.120725632	-8.283245109	4.120495176	34.13107152	-3.050196
Tlk1	Tlk1-004	677.52255	0.041385307	0.084412028	0.120754663	-8.28125371	0.861933166	7.13788723	-3.049849
Rnf4	Rnf4-001	3751.3629	0.000107359	0.000839511	0.120795731	-8.278438241	4.975561367	41.18987749	-3.049359
Eif2a	Eif2a-006	943.61508	4.09604E-05	0.00041721	0.120797813	-8.278295536	1.193311622	9.878586277	-3.049334

Txndc16	Txndc16-003	740.05054	0.021806679	0.050825367	0.120833	-8.275884907	1.079832041	8.936565687	-3.048914
Maged2	Maged2-002	511.03706	0.009790999	0.027104891	0.120851287	-8.274632626	0.457614852	3.786594789	-3.048695
Rsf1	Rsf1-201	3996.0255	1.21746E-07	1.06644E-05	0.120866096	-8.273618738	5.921281512	48.99042567	-3.048518
Cpne1	Cpne1-003	1974.2363	0.025605383	0.05755616	0.120883208	-8.272447584	2.352539213	19.46125733	-3.048314
Ndufc2	Ndufc2-001	1408.2785	2.56483E-05	0.00030084	0.120887512	-8.272153033	1.910335622	15.80258861	-3.048263
Ccnt2	Ccnt2-007	356.34287	0.004198573	0.013950438	0.120977555	-8.265996087	0.419356522	3.466399368	-3.047189
Kctd9	Kctd9-002	2637.3933	0.005319589	0.016827415	0.120992434	-8.264979596	2.572172603	21.25895408	-3.047011
Rapgef2	Rapgef2-002	2179.0054	4.98553E-05	0.000479648	0.121034005	-8.26214086	2.203542596	18.20597931	-3.046516
Lamp2	Lamp2-005	1515.0097	4.70779E-05	0.000460726	0.121078146	-8.259128745	2.115069708	17.46863302	-3.04599
Zfand1	Zfand1-003	2722.6632	1.67417E-08	3.20336E-06	0.121082319	-8.258844123	3.601159832	29.74141771	-3.04594
Gm16794	Gm16794-00	234.63125	0.009542984	0.026566463	0.121117151	-8.256468961	0.256940788	2.121423639	-3.045525
Ankrd44	Ankrd44-002	317.86443	0.036448625	0.076343286	0.12114194	-8.254779495	0.390317599	3.221985716	-3.04523
Zfp273	Zfp273-003	226	0.001283786	0.005545424	0.121225599	-8.249082749	0.179201499	1.478247994	-3.044234
200014J11R	00014J11Rik-	756.64422	0.001787084	0.007166331	0.121254795	-8.247096545	1.179808853	9.729997514	-3.043886
Srrm1	Srrm1-001	3061.0814	0.000276692	0.00169742	0.12129503	-8.244360845	5.034529815	41.50648048	-3.043408
Dph5	Dph5-008	463.20733	0.000151891	0.001090165	0.12130503	-8.243681256	0.852477239	7.027550639	-3.043289
Dhodh	Dhodh-005	1216.604	1.53752E-05	0.000209217	0.121308167	-8.243468035	1.631390663	13.44831679	-3.043251
Adam10	Adam10-004	366.35121	0.025937622	0.058146587	0.121359588	-8.239975229	0.325907156	2.685466896	-3.04264
Ktn1	Ktn1-012	394.58587	0.000259252	0.001618197	0.121393295	-8.237687245	0.462841714	3.812745282	-3.042239
Mdm4	Mdm4-001	27726.828	3.92536E-10	5.12675E-07	0.121438753	-8.234603635	38.33253437	315.6532269	-3.041699
Zfp280d	Zfp280d-004	1695.456	0.000162166	0.001143073	0.121468745	-8.232570475	2.245358709	18.48507381	-3.041343
Ppip5k2	Ppip5k2-006	335.434	0.00799892	0.023123145	0.121516931	-8.229305904	0.379574663	3.123636018	-3.040771
Camk2d	Camk2d-027	1734.695	7.62807E-06	0.000128848	0.121523201	-8.228881317	2.471518216	20.33783007	-3.040696
Fryl	Fryl-002	2462.0321	4.56012E-05	0.000451139	0.121534357	-8.228125981	4.27725762	35.19381455	-3.040564
Gm37419	Gm37419-001	232	0.008432603	0.024075472	0.121582838	-8.224845015	0.375959113	3.092205436	-3.039988
Nucb2	Nucb2-002	1435.9881	9.79123E-05	0.000785346	0.121609915	-8.223013748	1.720464149	14.14740035	-3.039667
Pid1	Pid1-002	1007.1078	0.001692977	0.006858096	0.121704999	-8.216589335	1.846015846	15.16795411	-3.03854
Wls	Wls-005	1222.308	4.03662E-05	0.000413316	0.1217435	-8.213990888	1.346767779	11.06233826	-3.038083
Itgb1bp1	Itgb1bp1-001	2791.4983	1.94764E-05	0.000247431	0.121770054	-8.212199713	3.265314174	26.81541212	-3.037769
Zfp62	Zfp62-008	480.86807	0.001952609	0.007694084	0.121840209	-8.207471146	0.61644779	5.059477448	-3.036938
Eci2	Eci2-001	760.92626	0.019409542	0.046394749	0.121841664	-8.207373165	0.986950101	8.100267771	-3.036921
Col27a1	Col27a1-003	348.06006	0.003412456	0.011868057	0.121917142	-8.202292026	0.587402743	4.818048833	-3.036027
Tlr13	Tlr13-001	1586	0.011642011	0.031085903	0.121973503	-8.198501928	4.03202462	33.05656163	-3.03536
Rassf2	Rassf2-002	623.03002	0.011591026	0.030966665	0.121996792	-8.196936831	1.240284245	10.16653161	-3.035085
Ptbp2	Ptbp2-011	1146.1128	0.002244292	0.00857596	0.122073963	-8.191755014	1.705169659	13.9683321	-3.034173
Rpl5	Rpl5-001	21590.359	3.1415E-09	1.51021E-06	0.122135929	-8.187598906	32.50404885	266.1301148	-3.03344
Pyroxd1	Pyroxd1-001	2184.2076	3.11514E-05	0.000344062	0.122148987	-8.186723623	3.234335965	26.47861465	-3.033286
Cc2d1b	Cc2d1b-008	119.53849	0.01690188	0.041592858	0.122150068	-8.186651174	0.140506466	1.150277422	-3.033273
St6galnac3	St6galnac3-00	5223.5499	1.30172E-05	0.000185688	0.122217431	-8.182138922	8.884505178	72.69425562	-3.032478
Gm29151	Gm29151-001	746	0.000167554	0.001171141	0.122301064	-8.176543745	1.241296592	10.14951589	-3.031491
Gm26835	Gm26835-201	4110.6106	9.50848E-06	0.000149594	0.122313179	-8.17573388	5.04646556	41.25855945	-3.031348
Zfp280c	Zfp280c-004	183.98786	0.008916341	0.025173909	0.122318252	-8.175394821	0.259894735	2.124742072	-3.031288
Lamb2	Lamb2-003	364.38209	0.023543119	0.053886115	0.122356149	-8.172862643	0.410316097	3.353457101	-3.030841
Npl	Npl-002	561	2.85116E-05	0.000323351	0.122448143	-8.166722469	0.70655295	5.770221852	-3.029757
Dlg1	Dlg1-005	1000.799	0.000573782	0.002976227	0.122457921	-8.166070338	1.248312805	10.19381017	-3.029642
Rps18	Rps18-008	1491.9446	0.017116078	0.041990371	0.122494195	-8.163652193	2.245639918	18.33262324	-3.029215
Magt1	Magt1-001	11020.877	9.18466E-09	2.47697E-06	0.122516148	-8.162189346	13.89700592	113.4299937	-3.028956
Xpo1	Xpo1-201	5189.6123	8.8315E-05	0.000727638	0.122537699	-8.160753844	9.177769058	74.89751412	-3.028702
Zfr	Zfr-009	706.47663	0.000254995	0.001597803	0.122632627	-8.15443677	0.936872706	7.639669242	-3.027585
Rfc5	Rfc5-001	1318.8416	3.46656E-05	0.000369759	0.122650139	-8.153272444	1.989174923	16.21828509	-3.027379
Shmt1	Shmt1-001	615.07759	0.000376238	0.002146616	0.122651601	-8.153175285	1.095392438	8.930926557	-3.027362
Gm6483	Gm6483-005	2965.364	3.69317E-07	1.95806E-05	0.122670919	-8.151891306	4.41672081	36.00462797	-3.027135
Serpinb6a	Serpinb6a-00	1714.6026	0.000265166	0.001644983	0.122737518	-8.147467985	2.290649118	18.66299035	-3.026352
Eif5a	Eif5a-201	1926.2186	0.000643062	0.00324468	0.122740606	-8.147263011	3.172420418	25.84654353	-3.026315
Elmo2	Elmo2-012	247.52954	0.02384312	0.054441791	0.122758394	-8.146082451	0.339297938	2.76394898	-3.026106
Gcc2	Gcc2-005	2417.6167	4.27229E-05	0.000431135	0.12277894	-8.144719261	3.450500351	28.10335667	-3.025865
Tm2d3	Tm2d3-201	969.04871	0.000547199	0.002868755	0.122843233	-8.140456538	1.336151041	10.87687947	-3.02511
Vps13d	Vps13d-008	1368.538	1.22714E-06	3.98678E-05	0.122851832	-8.139886734	1.463033909	11.90893031	-3.025009
Pds5a	Pds5a-003	940.85493	2.21101E-05	0.000271127	0.122915644	-8.135660931	1.512984217	12.30912658	-3.02426
Atg3	Atg3-002	5126.7995	6.79168E-06	0.000119106	0.122916252	-8.135620691	7.232523488	58.84106774	-3.024252
Gm16580	Gm16580-001	3627	6.58544E-08	7.54352E-06	0.122971171	-8.131987308	5.976420779	48.60017793	-3.023608
Hspb11	Hspb11-001	285.04616	0.023921845	0.054581519	0.123098452	-8.123579002	0.374186835	3.039736318	-3.022115
Zbtb11	Zbtb11-003	1598.0767	3.24064E-05	0.000353931	0.123108858	-8.122892341	2.158063585	17.52971816	-3.021994
Gm29398	Gm29398-001	127	0.009263266	0.0259523	0.123122993	-8.121959793	0.130889053	1.063075624	-3.021828
Pitpnm2os2	itpnm2os2-00	370.00711	0.001896182	0.007508006	0.123127654	-8.121652349	0.398559458	3.23696136	-3.021773
Ralgapb	Ralgapb-006	1050.9061	0.000250204	0.001576675	0.123181926	-8.118074061	1.348895731	10.95043545	-3.021138
Gm4853	Gm4853-001	600	0.000742922	0.003619507	0.123195496	-8.117179844	0.899722022	7.303205459	-3.020979
Zfp655	Zfp655-002	965.76514	4.78616E-05	0.000466508	0.123264317	-8.112647874	1.243245648	10.08601416	-3.020173
Tiprl	Tiprl-001	2171.6627	5.12419E-06	9.94917E-05	0.123316373	-8.109223245	3.228823109	26.18324741	-3.019564
Thg1l	Thg1l-002	148.04242	0.038250455	0.079302548	0.123318964	-8.109052859	0.245537401	1.991075764	-3.019533
230025D16R	0025D16Rik-	1938.5784	7.81749E-05	0.000668871	0.123354617	-8.106709123	3.01547704	24.44559523	-3.019116
Prokr2	Prokr2-005	203.00091	0.002711584	0.009934931	0.123374352	-8.105412408	0.294769929	2.389231839	-3.018886
Abi1	Abi1-015	288.3231	0.001229714	0.005363263	0.123405684	-8.103354437	0.37096854	3.006089569	-3.018519
930016D06R	0016D06Rik-	2003.0118	3.9807E-06	8.38756E-05	0.12341364	-8.102832096	2.870155601	23.25638893	-3.018426
Dhx9	Dhx9-006	577.70389	8.24084E-05	0.000694339	0.123415804	-8.102689984	0.817336221	6.622622014	-3.018401
P24-470E5.	24-470E5.4-	2838	8.2883E-05	0.000696795	0.123494206	-8.097545897	2.573126695	20.83601151	-3.017485
Tubgcp6	Tubgcp6-006	161.13405	0.044785752	0.089833793	0.123551139	-8.093814489	0.267503087	2.165120359	-3.01682
Gm13535	Gm13535-001	253	0.005669251	0.017655196	0.123560674	-8.093189886	0.363900753	2.945117895	-3.016708
Srrm1	Srrm1-007	4750.3864	0.001665014	0.006772969	0.123603346	-8.090395872	7.066751471	57.17281693	-3.01621
Sun2	Sun2-001	7588.8595	1.55559E-06	4.58116E-05	0.123621773	-8.089189912	10.36619754	83.85414054	-3.015995
Dkc1	Dkc1-001	6899.482	4.83807E-08	6.18299E-06	0.123650834	-8.087288754	11.78248595	95.2883661	-3.015656
Gapvd1	Gapvd1-007	1396.2963	0.011970206	0.031744448	0.123710013	-8.08342003	1.701918665	13.75732343	-3.014966
Rnf214	Rnf214-003	1541.0549	0.010554422	0.028769397	0.123739597	-8.081487436	2.275926888	18.39287455	-3.014621
Ctnnd1	Ctnnd1-010	343.01802	0.00250573	0.009335104	0.123844618	-8.074634277	0.40723983	3.288312692	-3.013397
30525G20R	0525G20Rik-	276	0.001698555	0.006873969	0.123881858	-8.072207004	0.404606231	3.266065254	-3.012963
Phip	Phip-018	2104.2816	4.58859E-06	9.23116E-05	0.123929631	-8.069095306	2.907442757	23.4604327	-3.012407
Plscr3	Plscr3-001	1937.2839	0.000752725	0.003655818	0.123988556	-8.065260451	1.927597821	15.54657847	-3.011721
Srcap	Srcap-007	1390.7751	5.83869E-05	0.00053989	0.124033275	-8.062352632	1.831897922	14.76940703	-3.011201
Gm37233	Gm37233-001	887	0.000870391	0.004092654	0.124136095	-8.055674697	1.495549538	12.04766057	-3.010005
Ginm1	Ginm1-003	961.21562	0.012776638	0.033381635	0.124218014	-8.050362191	1.475917354	11.88166926	-3.009054
Ppm1m	Ppm1m-002	890.80038	0.001845057	0.007346354	0.124256774	-8.047850994	1.053592331	8.479154086	-3.008604
Tsc1	Tsc1-201	1324.4363	0.018005503	0.04369958	0.124274245	-8.04671958	1.732319844	13.93949201	-3.008401
Ica1l	Ica1l-002	437.45694	0.01193223	0.031665805	0.124299795	-8.045065564	0.683223098	5.496574616	-3.008104
Tmem230	Tmem230-00	605.2311	0.046850049	0.093169876	0.124322207	-8.043615261	0.811934368	6.530887674	-3.007844
Gatad1	Gatad1-003	956.22628	0.001401196	0.005938741	0.124339532	-8.042494465	1.251537405	10.06548265	-3.007643
Vdac3	Vdac3-202	1652.4926	5.61673E-06	0.000105295	0.124358317	-8.041279624	2.121354474	17.0584045	-3.007425
Adh7	Adh7-003	2167.5747	0.004210147	0.013980759	0.124370065	-8.040520036	2.618337791	21.05279747	-3.007289
Bdp1	Bdp1-006	228.03237	0.000663025	0.003322399	0.124657273	-8.021994804	0.336682826	2.700867881	-3.003961
Dph6	Dph6-002	721.41305	0.019107014	0.045814563	0.124710186	-8.0185912	1.268391963	10.17071663	-3.003349
Gm13854	Gm13854-001	219	0.004651507	0.015113756	0.124753284	-8.015821045	0.293009872	2.3487147	-3.00285
Mpc2	Mpc2-003	813.99576	0.039621838	0.081451243	0.12482164	-8.011431355	1.099030113	8.804804311	-3.00206
Nsmaf	Nsmaf-003	808.66059	0.004654438	0.015120411	0.124860153	-8.008960231	1.112235338	8.907848589	-3.001615
Klc1	Klc1-007	1861.3346	5.49285E-06	0.00010371	0.124927869	-8.004619051	1.947671249	15.59036638	-3.000833
Txndc11	Txndc11-008	274.07377	0.000933542	0.004320102	0.124948249	-8.003313408	0.263535408	2.109156465	-3.000597
Birc5	Birc5-002	1140.2027	0.000144707	0.001052497	0.124967933	-8.002052793	1.790974577	14.33147312	-3.00037
Hmga2	Hmga2-001	560.64231	0.000784331	0.003769765	0.125106563	-7.993185783	0.982355156	7.852147264	-2.998771
Rbm17	Rbm17-003	1055.9715	3.52579E-05	0.000374561	0.125166412	-7.989363765	1.421323355	11.35546931	-2.998081

P24-555M2(	24-555M20.4-	671	0.00017601	0.001211972	0.125183348	-7.988282895	0.716263526	5.721715673	-2.997885
RP24-544J6.	P24-544J6.6-0	281	0.00081426	0.003883098	0.12519204	-7.987728263	0.393694235	3.14472257	-2.997785
Ptprg	Ptprg-006	1064.9461	0.00052597	0.002776142	0.125441275	-7.971857759	0.900342059	7.177398826	-2.994916
Gm7816	Gm7816-001	1685	3.55476E-06	7.83465E-05	0.12547314	-7.96983322	2.439572281	19.4429842	-2.99455
Scamp3	Scamp3-002	1510.4817	0.000138156	0.001019379	0.12549634	-7.968359873	1.709222785	13.61970225	-2.994283
Gm37829	Gm37829-001	1386.9766	1.8425E-05	0.000237849	0.125517072	-7.967043747	1.927150921	15.3536957	-2.994044
Stat1	Stat1-007	119.19528	0.041980748	0.085397673	0.125576241	-7.963289786	0.202580317	1.613205768	-2.993365
Bmp2k	Bmp2k-002	1266.2101	0.009475398	0.026432022	0.125719697	-7.954203074	2.809981462	22.35116318	-2.991717
Gm37800	Gm37800-002	1247.9394	3.35618E-06	7.55785E-05	0.12572673	-7.953758135	1.617167681	12.8625606	-2.991637
Fam169b	Fam169b-001	633.54624	9.9199E-05	0.000793408	0.125769679	-7.951041976	0.816956546	6.495655789	-2.991144
Arl5b	Arl5b-005	836	1.7388E-05	0.000228093	0.125872425	-7.944551789	1.250128932	9.93171404	-2.989966
Nrk	Nrk-001	1721.6429	0.000180177	0.001233045	0.125872691	-7.944535018	1.34037185	10.6486311	-2.989963
Scaf4	Scaf4-202	7776.8123	2.81135E-06	6.7666E-05	0.125889452	-7.943477267	9.660653635	76.73918254	-2.989771
Wdr61	Wdr61-004	379.44555	0.030589115	0.0663759	0.125908664	-7.942265227	0.516294802	4.100550256	-2.989551
Ascc1	Ascc1-202	293.31546	0.026966677	0.060005762	0.12593963	-7.940312383	0.206358496	1.638550925	-2.989196
Rps15a-ps6	ps15a-ps6-00	1013	1.63389E-05	0.000217746	0.125940015	-7.940288059	1.634079623	12.97506292	-2.989191
Apbb2	Apbb2-005	1286.2391	0.000138233	0.001019703	0.125976678	-7.937977242	1.192768583	9.468169866	-2.988771
Tcf12	Tcf12-021	705.86629	0.021666677	0.050565791	0.12598278	-7.937592745	0.663152813	5.263836956	-2.988702
Tcn2	Tcn2-003	826.70569	0.000399	0.002245589	0.125993818	-7.936897351	0.687587843	5.457314127	-2.988575
Dph6	Dph6-001	1455.1919	0.029677252	0.064750023	0.126008891	-7.935947935	2.649035516	21.02260794	-2.988403
Cdk12	Cdk12-005	7891.6577	3.81035E-05	0.000395693	0.12604706	-7.933544808	11.12406569	88.25327359	-2.987966
Gm25735	Gm25735-201	438	3.27396E-05	0.000356173	0.126081164	-7.931398848	0.684851046	5.431826797	-2.987575
Ankhd1	Ankhd1-013	512.83082	0.0001651	0.001159222	0.126103803	-7.929974965	0.677345451	5.371332468	-2.987316
Cc2d1a	Cc2d1a-003	183.16995	0.045798667	0.091495821	0.126112699	-7.92941558	0.209524768	1.661408956	-2.987215
Nek7	Nek7-003	1920.9892	0.00012614	0.000951673	0.126124937	-7.928646169	2.354732166	18.66983816	-2.987075
Phf11d	Phf11d-004	2422.7796	0.000189331	0.001278254	0.126191519	-7.924462832	2.795344219	22.15160137	-2.986313
Cct8	Cct8-002	1303.9088	0.004451389	0.014609052	0.126221977	-7.922550611	2.542499651	20.14308216	-2.985965
Snx25	Snx25-002	631.73567	0.037660195	0.078325339	0.126229179	-7.922098562	1.172711516	9.290336211	-2.985883
Sox5	Sox5-003	580.28112	0.01251249	0.032848937	0.126231748	-7.921937366	1.03696541	8.214775029	-2.985853
Mrps35	Mrps35-002	660.79264	0.000241089	0.001532308	0.126278782	-7.91898671	0.980711581	7.766241978	-2.985316
Paics	Paics-002	18798.566	1.29563E-07	1.09569E-05	0.126313252	-7.916825721	19.34946535	153.186345	-2.984922
Pkp4	Pkp4-010	8523.1537	0.000406015	0.002275198	0.126317832	-7.916538641	7.335279953	58.0700272	-2.98487
Mia3	Mia3-005	388.45031	0.000655506	0.003291947	0.126350693	-7.914479711	0.594826004	4.707738343	-2.984495
Ctnnd2	Ctnnd2-201	135	0.018919311	0.045495078	0.126355866	-7.914155736	0.25177841	1.992613545	-2.984435
Slc29a3	Slc29a3-001	4323.9983	3.92177E-06	8.3044E-05	0.126399259	-7.911438812	7.084609685	56.04945603	-2.98394
Mlh1	Mlh1-002	1045.0558	0.000948914	0.004372433	0.126415277	-7.910436317	1.664580293	13.1675564	-2.983757
Clec4a1	Clec4a1-001	568	0.049613197	0.097379545	0.126432344	-7.909368477	1.476827773	11.68077503	-2.983563
Pola1	Pola1-003	235.80919	0.002904958	0.010493954	0.126446722	-7.90846916	0.311386683	2.462591981	-2.983398
Crtc2	Crtc2-008	147.5176	0.034045492	0.072327138	0.126477342	-7.906554517	0.242859307	1.92018035	-2.983049
Isca2	Isca2-201	2315	3.24964E-07	1.82142E-05	0.126499393	-7.905176283	3.401089381	26.88621111	-2.982798
Igsf10	Igsf10-002	575.01072	0.000405835	0.002274563	0.126518918	-7.903956284	0.977825284	7.728688302	-2.982575
Gm21917	Gm21917-001	838	5.14623E-06	9.96068E-05	0.126528395	-7.903364315	1.120958877	8.859346386	-2.982467
Mtpn	Mtpn-001	22642.954	3.1043E-08	4.59588E-06	0.126689033	-7.89334309	31.61518098	249.5494703	-2.980636
30408A02R	0408A02Rik-	1308.683	2.34535E-05	0.000281952	0.126738099	-7.89028721	1.475682496	11.64355872	-2.980078
Polq	Polq-007	171.50449	0.009873609	0.027285066	0.126768058	-7.888422511	0.326904199	2.578758446	-2.979737
Ppt1	Ppt1-002	1432.0943	0.006760138	0.020249671	0.126781249	-7.887601731	1.978046493	15.60204295	-2.979587
Tmem55b	Tmem55b-001	1200.4006	0.001606203	0.006591477	0.126797644	-7.886581842	2.093338143	16.50928259	-2.9794
Acp1	Acp1-202	3956.6645	0.000224073	0.001451307	0.126887003	-7.881027809	5.500149839	43.34683383	-2.978384
Csn3	Csn3-004	549	4.94346E-05	0.000476805	0.12692984	-7.878368088	0.803581739	6.330912728	-2.977897
Yme1l1	Yme1l1-006	544.09522	0.001103903	0.004921721	0.126948986	-7.87717987	0.596994425	4.702632465	-2.977679
Gm20537	Gm20537-002	106.16858	0.023249733	0.053408798	0.126962306	-7.876353457	0.144273816	1.136351567	-2.977528
Nfl	Nfl-004	896.70095	5.73794E-05	0.000532731	0.127090098	-7.868433626	1.246414865	9.807332635	-2.976076
30511E19R	0511E19Rik-	1907.1137	0.000100272	0.000798674	0.127168161	-7.863603559	2.92523161	23.0028617	-2.975191
Mfap3	Mfap3-002	3656.0837	1.15322E-05	0.000170765	0.127230725	-7.859736683	4.366800752	34.32190405	-2.974481
Klc1	Klc1-008	1752.8593	3.10418E-05	0.000343227	0.127278221	-7.85680371	2.171981252	17.06483036	-2.973943
Tet1	Tet1-201	3699.7957	0.000560048	0.002921359	0.127394219	-7.849649725	4.561257033	35.80427001	-2.972628
Brca2	Brca2-008	307.61395	0.000183397	0.00125027	0.127472557	-7.844825794	0.384793158	3.018635293	-2.971741
C5ar2	C5ar2-201	1081.7262	1.85363E-06	5.10344E-05	0.127523436	-7.841695861	1.690275207	13.25462409	-2.971166
C5ar2	C5ar2-202	397.27328	4.71499E-05	0.000461263	0.127535211	-7.840971832	0.620833635	4.867939044	-2.971032
30562O15R	0562O15Rik-	200.57624	0.007995786	0.023116982	0.12754819	-7.840173981	0.328652421	2.57669216	-2.970886
Spire1	Spire1-203	935.41195	0.022970584	0.052872481	0.127694664	-7.831180809	1.648985692	12.91350511	-2.96923
Gm10433	Gm10433-001	349	0.000124766	0.00094415	0.127727087	-7.829192857	0.450153316	3.524337124	-2.968864
Cox7c	Cox7c-001	5501.2743	2.63928E-05	0.000306737	0.127808663	-7.824195781	8.497722014	66.48784073	-2.967942
Ddah1	Ddah1-001	4178.2835	0.002520664	0.00937177	0.127856266	-7.821282671	8.67888498	67.88001269	-2.967405
Gm4221	Gm4221-201	553.57585	1.98042E-05	0.000250385	0.127868145	-7.820556082	0.64153758	5.017180624	-2.967271
Rassf1	Rassf1-006	1469.8496	0.001547763	0.006401506	0.127896803	-7.818803732	1.774703663	13.87605962	-2.966948
Trim33	Trim33-001	4044.0567	7.2211E-07	2.87584E-05	0.127928311	-7.816878023	5.492314482	42.93275237	-2.966593
Ipp	Ipp-001	343.364	0.012882215	0.033606206	0.127956885	-7.815132443	0.38246227	2.988993294	-2.96627
Tstd3	Tstd3-001	1454.8717	0.000209668	0.001380722	0.128058209	-7.808948833	2.301592132	17.97301519	-2.965128
Hdac5	Hdac5-005	1264.3526	0.002565671	0.009510198	0.128068041	-7.808349288	0.755813307	5.901654299	-2.965018
Srrm2	Srrm2-001	53159.908	1.74618E-09	1.19613E-06	0.128091804	-7.806900729	77.82246081	607.552226	-2.96475
Prr14	Prr14-001	2188.2858	0.006323726	0.019197626	0.128107115	-7.805967656	2.600558609	20.29987639	-2.964577
Pex11a	Pex11a-001	296.47635	0.012249782	0.032305769	0.128153842	-7.803121529	0.576306817	4.496992127	-2.964051
Ak2	Ak2-001	4027.9155	1.98357E-05	0.000250657	0.128175511	-7.801802342	6.797249364	53.03079601	-2.963807
Sf1	Sf1-013	283.73101	0.004263602	0.014121299	0.128260957	-7.796604866	0.564346179	4.399984162	-2.962846
010107E04R	0107E04Rik-	1173	2.52553E-05	0.000297406	0.128269475	-7.796087107	1.905773907	14.85757938	-2.96275
Zc3h11a	Zc3h11a-006	3064.1816	2.0599E-07	1.45768E-05	0.128302547	-7.794077571	4.336361109	33.79793486	-2.962378
Odf2l	Odf2l-006	567.39737	0.00014591	0.001058326	0.128378575	-7.789461769	0.815055817	6.348846123	-2.961524
P23-15K13	23-15K13.2-(	2376	1.19583E-06	3.91559E-05	0.12839846	-7.788255408	3.389275197	26.39654088	-2.9613
10474O19R	0474O19Rik-	788.18187	0.009589917	0.026664609	0.128408384	-7.787653495	1.263923765	9.843000328	-2.961189
Gm38134	Gm38134-001	199	0.028926258	0.063390129	0.128435048	-7.786036722	0.198407874	1.544810995	-2.960889
Gm37518	Gm37518-001	296	0.007822252	0.022729459	0.128460909	-7.784469255	0.415585259	3.235110671	-2.960599
Setd8	Setd8-001	2470.4091	0.000347891	0.002021938	0.128505297	-7.781780366	3.761190724	29.26876013	-2.9601
Ube2t	Ube2t-003	707.4696	3.91778E-05	0.000404033	0.128523438	-7.780682007	1.101416881	8.569774507	-2.959897
Tmem263	Tmem263-201	8986	5.43539E-08	6.62499E-06	0.128538422	-7.779774957	12.76736408	99.32721935	-2.959728
Cdc6	Cdc6-002	631.28862	0.00016426	0.001154745	0.128592453	-7.776506128	1.027297322	7.988783923	-2.959122
Ilf2	Ilf2-005	5160.9844	1.01486E-07	9.62769E-06	0.128600929	-7.775993615	6.720252286	52.25663887	-2.959027
030037D09R	0037D09Rik-	211.37518	0.014668396	0.037239089	0.128621461	-7.774752323	0.213006108	1.65606973	-2.958797
Axl	Axl-001	2473.2131	0.010114776	0.02783647	0.128628441	-7.774330425	4.10272343	31.89592759	-2.958718
30020A03R	0020A03Rik-	674	0.000216732	0.001416076	0.128669088	-7.771874443	0.909028701	7.06485693	-2.958263
Gm11575	Gm11575-001	357	0.004580507	0.014945447	0.128690568	-7.770577236	0.562896038	4.374027143	-2.958022
U2surp	U2surp-002	4850.1707	1.10785E-08	2.78999E-06	0.12869285	-7.770439442	8.056368245	62.60152158	-2.957996
Fchsd2	Fchsd2-004	3731.3897	2.82888E-05	0.000321769	0.128731493	-7.768106915	4.062403307	31.55718322	-2.957563
Rabepk	Rabepk-001	852.30006	0.000383614	0.002182157	0.128776892	-7.76536835	1.332618525	10.34827372	-2.957054
Ncoa6	Ncoa6-006	225.32869	0.001610179	0.006602255	0.128783367	-7.764977905	0.341495016	2.651701254	-2.956982
Mars	Mars-005	397.02484	0.005411625	0.017053369	0.128788096	-7.764692776	0.732728401	5.689410922	-2.956929
Rufy2	Rufy2-005	155.65541	0.006480639	0.01957681	0.128848541	-7.761050226	0.191573516	1.486811681	-2.956252
Mettl7a2	Mettl7a2-002	744.03585	0.000548105	0.002872187	0.128868357	-7.759856801	0.654788786	5.081067211	-2.95603
Phc1	Phc1-202	897.84665	0.000258908	0.001617085	0.128879448	-7.759189059	1.435193792	11.13593997	-2.955906
Mdm2	Mdm2-002	1049.3287	0.005426508	0.01708881	0.128908142	-7.75746187	1.21895826	9.456022224	-2.955585
Gm10284	Gm10284-001	778	0.000123675	0.000937761	0.12892936	-7.756185235	1.292169893	10.02230905	-2.955347
Rnf170	Rnf170-003	408.13773	0.030709651	0.066603695	0.128945021	-7.755243219	0.449691869	3.487469819	-2.955172
Atad5	Atad5-001	968.51432	0.002164553	0.008325203	0.12895917	-7.754392335	1.547511634	12.00001235	-2.955014
Wnk1	Wnk1-012	1151.695	8.45317E-06	0.00013813	0.129022698	-7.750574249	1.749452127	13.55925861	-2.954303
Cyp4f13	Cyp4f13-003	1248.4267	0.000865767	0.004075946	0.129086992	-7.746713948	1.436620733	11.12908987	-2.953584
Fip1l1	Fip1l1-006	766.09369	0.000577717	0.002989393	0.129091024	-7.746471973	1.155025238	8.947370638	-2.953539
Gmppa	Gmppa-011	333.47379	0.033537045	0.071459735	0.129107553	-7.745480255	0.456412076	3.535130724	-2.953355

Cdkl5	Cdkl5-003	3339.1312	2.55466E-06	6.36343E-05	0.129118527	-7.74482195	4.330218533	33.53677154	-2.953232
Usp45	Usp45-009	3066.5071	2.54217E-05	0.000298796	0.129143071	-7.743350014	4.13432985	32.0135631	-2.952958
Rps27a	Rps27a-001	326.78008	0.003251591	0.011438685	0.12915408	-7.742689961	0.494525442	3.828957175	-2.952835
Oxr1	Oxr1-202	228.74397	0.018730191	0.045109981	0.129161054	-7.742271931	0.410339223	3.176957849	-2.952757
Fubp1	Fubp1-010	8225.5842	7.91505E-08	8.3929E-06	0.129198603	-7.740021741	12.87682289	99.66688912	-2.952338
Hdac3	Hdac3-002	904.81328	0.000301938	0.001819066	0.129212324	-7.739199866	1.437709577	11.12672177	-2.952184
Nsun4	Nsun4-002	841.09253	1.89335E-05	0.000242783	0.129236205	-7.737769751	1.156697049	8.950255438	-2.951918
32427E13R	2427E13Rik-	5299.0584	6.17475E-09	2.07338E-06	0.129239765	-7.737556635	7.571034923	58.58131151	-2.951878
Igf2bp2	Igf2bp2-009	1699.7085	0.002602991	0.009619382	0.129245906	-7.737188958	2.491628039	19.27819695	-2.95181
Rnaseh2a	Rnaseh2a-201	536.16106	2.99641E-05	0.000335163	0.129295204	-7.734238924	0.88587146	6.851541529	-2.951259
Gm12734	Gm12734-001	334	0.000908821	0.004233828	0.129354646	-7.730684862	0.51807774	4.005095739	-2.950596
Cnep1r1	Cnep1r1-003	1281.7382	7.34798E-06	0.00012552	0.129358498	-7.730454609	1.95662801	15.12562402	-2.950553
Wbp1l	Wbp1l-001	7830.1466	3.19975E-07	1.80249E-05	0.129363487	-7.730156512	9.580136882	74.0559575	-2.950498
Rnpep	Rnpep-002	446.8565	0.018575034	0.044824556	0.129394304	-7.728315482	0.616105973	4.76146133	-2.950154
P24-421E18	24-421E18.7-	698	8.7984E-06	0.000142009	0.129407539	-7.727525058	0.901555796	6.966795008	-2.950006
Luc7l3	Luc7l3-007	478.15028	0.001943112	0.007663703	0.129490644	-7.722565638	0.732441959	5.656331104	-2.94908
30430E12R	0430E12Rik-	172.86227	0.002443246	0.009157493	0.129558147	-7.718542012	0.253542185	1.956976008	-2.948328
Gm8203	Gm8203-001	1295	0.002416637	0.009078624	0.129578062	-7.717355727	2.497866842	19.27692698	-2.948107
Gm5920	Gm5920-001	68	0.013575767	0.035033906	0.129601764	-7.715944337	0.098950588	0.763497231	-2.947843
Pag1	Pag1-001	1737.6625	0.000768503	0.003712969	0.12960979	-7.715466574	3.725344086	28.74276777	-2.947753
Gm4994	Gm4994-001	381	0.000205665	0.001360113	0.129653682	-7.712854606	0.559793812	4.317608281	-2.947265
Col1a2	Col1a2-004	515.34722	0.001082941	0.004843395	0.129693828	-7.710467155	0.886127019	6.832453278	-2.946818
Bora	Bora-201	444	0.0253642	0.057137848	0.12970144	-7.710014639	0.705203785	5.437131505	-2.946734
Tgfb1i1	Tgfb1i1-007	4513.8694	0.001042867	0.004702206	0.12970776	-7.709638965	6.119238831	47.17712213	-2.946663
Matr3	Matr3-012	438.95757	0.000172682	0.00119458	0.129731641	-7.708219757	0.786754617	6.064477485	-2.946398
Meis2	Meis2-011	723.48771	0.039735185	0.081654172	0.129761031	-7.7064739	0.995242525	7.669810542	-2.946071
Pgap2	Pgap2-007	172.01429	0.014353836	0.036608748	0.129774543	-7.705671512	0.261076796	2.011772027	-2.945921
Fchsd2	Fchsd2-001	3967.4645	0.00019426	0.001303822	0.129780213	-7.70533489	4.144511133	31.93484624	-2.945858
Cetn4	Cetn4-004	455.95628	0.000198875	0.001326998	0.129811514	-7.703476917	0.708392594	5.457085997	-2.94551
Svil	Svil-009	1077.3574	3.91739E-06	8.30358E-05	0.129877236	-7.699578671	1.448569068	11.1533715	-2.94478
Aim1l	Aim1l-002	1450.9888	2.22241E-05	0.000272139	0.129908694	-7.697714233	1.829030825	14.07935661	-2.94443
Mrps9	Mrps9-005	852.80467	6.29594E-05	0.000571069	0.129915993	-7.697281747	1.303278563	10.0317023	-2.944349
Slirp	Slirp-004	726.12594	0.003909686	0.013183689	0.129932888	-7.696280854	1.24149175	9.554869182	-2.944161
Pds5a	Pds5a-010	2789.4933	2.90781E-06	6.91523E-05	0.1299363	-7.696078744	4.034589477	31.05051832	-2.944124
Usp46	Usp46-001	1388.5504	0.017920698	0.043543127	0.129954219	-7.695017565	2.275263621	17.50819353	-2.943925
P23-357D17	23-357D17.4-	2304	7.48339E-05	0.000648511	0.129983966	-7.693256538	3.359804568	25.84783846	-2.943594
Mier1	Mier1-013	1137.6192	0.003497962	0.012096646	0.130047487	-7.689498837	1.468292883	11.29043642	-2.94289
Sgms1	Sgms1-001	13364.83	0.000261032	0.001625246	0.130061934	-7.688644729	18.21428091	140.0431349	-2.942729
Nhs	Nhs-201	449.04938	0.002987098	0.010719355	0.13014044	-7.684006596	0.456649478	3.508897602	-2.941859
Sptbn1	Sptbn1-005	3813.9331	0.002401944	0.00903631	0.130207213	-7.680066067	3.916315377	30.07756083	-2.941119
Zdhhc15	Zdhhc15-001	387	0.000533489	0.002810211	0.130223308	-7.679116834	0.661801683	5.082052444	-2.94094
00011K16F	00011K16Rik-	1115	3.76686E-05	0.000392739	0.1302295	-7.678751769	1.532585761	11.76834562	-2.940872
Gpcpd1	Gpcpd1-201	303.326	0.005815552	0.017996264	0.1302699	-7.676370367	0.51609234	3.961715944	-2.940424
Trib1	Trib1-002	422.96965	0.019884133	0.047313572	0.130325903	-7.673071739	0.947215006	7.268048693	-2.939804
Rabggtb	Rabggtb-008	2916.0628	4.64503E-06	9.30909E-05	0.130332262	-7.672697368	4.844775325	37.17249488	-2.939734
Txn1	Txn1-001	10008	4.37619E-06	8.92169E-05	0.130340555	-7.672209158	17.04826022	130.7978182	-2.939642
Metap2	Metap2-007	5528.3437	1.00048E-05	0.000154609	0.130377048	-7.670061703	8.689600896	66.64977505	-2.939238
Mtmr11	Mtmr11-003	1650.0761	0.00497466	0.015944424	0.130494216	-7.663174875	2.129245782	16.31678278	-2.937942
Slc31a1	Slc31a1-001	3616.5263	6.303E-05	0.000571559	0.130520615	-7.661624966	6.757257546	51.77157311	-2.93765
Far1os	Far1os-001	154.46102	0.020572341	0.048566494	0.130612756	-7.656220022	0.186001706	1.424069988	-2.936632
Gm3534	Gm3534-001	587	5.50931E-05	0.000516828	0.130707625	-7.650663088	0.997776792	7.633654076	-2.935585
Psma3	Psma3-003	735.49192	0.000227683	0.001470239	0.13075515	-7.647882349	1.149537074	8.791524301	-2.93506
Ncam1	Ncam1-003	441.92409	0.000946366	0.004363042	0.130815801	-7.644336458	0.395706409	3.024912926	-2.934391
Med1	Med1-002	3553.0412	0.006629709	0.019926946	0.130836157	-7.643147157	4.355914876	33.2928984	-2.934167
Nav1	Nav1-005	639.03061	0.003723488	0.01270467	0.130867872	-7.641294894	1.011596766	7.729909205	-2.933817
200014J11R	0014J11Rik-(	195.27954	0.000882606	0.004139251	0.130869146	-7.641220476	0.256389765	1.959130724	-2.933803
Sirt7	Sirt7-008	325.52109	0.018038546	0.043754973	0.130928147	-7.637777103	0.377762854	2.88526848	-2.933153
B2m	B2m-001	118275	1.9323E-09	1.27272E-06	0.130933838	-7.637445091	163.6645768	1249.979219	-2.93309
Mast4	Mast4-009	1153.5697	0.000621939	0.003163263	0.131000042	-7.633585366	2.01824401	15.40643794	-2.932361
Supt20	Supt20-009	367.22013	0.00125121	0.005435959	0.131069038	-7.629566935	0.538492006	4.108460801	-2.931601
Arih2	Arih2-006	484.46617	0.000257162	0.001608796	0.131076879	-7.629110547	0.827767677	6.315131117	-2.931515
Rbpj	Rbpj-006	1329	2.37389E-05	0.000284181	0.131088333	-7.628443948	2.278140578	17.3786677	-2.931389
Lclat1	Lclat1-001	2823.8683	1.34045E-05	0.000189713	0.131202803	-7.621788403	4.353459238	33.18114513	-2.93013
Tmbim6	Tmbim6-008	551.74387	0.00143896	0.006061293	0.131203292	-7.621759975	0.685234372	5.222691911	-2.930124
P23-94F17.	23-94F17.2-(	369	0.000742754	0.003619507	0.131224082	-7.620552427	0.508109306	3.872073607	-2.929896
Gm28262	Gm28262-001	1330	3.47883E-06	7.737E-05	0.131279423	-7.617340006	1.878219802	14.30703884	-2.929287
Wbp11	Wbp11-005	476.74306	0.006484308	0.019586166	0.131302864	-7.615980117	0.807760593	6.151888617	-2.92903
P23-293K2	23-293K21.4-	482	0.000409805	0.00229231	0.131306692	-7.615758084	0.906870037	6.906502817	-2.928988
Gm14418	Gm14418-001	332.90254	0.001702633	0.006886534	0.131355612	-7.612921809	0.444945435	3.387334809	-2.92845
Lbhd1	Lbhd1-001	773.61697	0.000841965	0.003983053	0.13144191	-7.607923554	1.276931743	9.714799085	-2.927503
Pcmt1	Pcmt1-002	2285.0246	2.55087E-05	0.000299716	0.131460638	-7.606839708	3.470831704	26.40206043	-2.927297
Gatad2b	Gatad2b-008	607	5.85537E-05	0.000540919	0.131495706	-7.604811045	0.83881155	6.379003338	-2.926912
P23-372C7.	23-372C7.7-(	412	0.006026625	0.018520583	0.131505506	-7.604244346	0.563894657	4.287992758	-2.926805
Tmed5	Tmed5-004	2137.1348	0.000829105	0.003937442	0.131512954	-7.603813708	3.187871272	24.23997928	-2.926723
Cd81	Cd81-004	1579.2587	0.000382589	0.002177054	0.131534891	-7.602545544	1.647781898	12.52733693	-2.926483
Ube2b	Ube2b-001	7266.4862	1.90094E-07	1.38526E-05	0.131553786	-7.601453596	10.41179604	79.14478445	-2.926275
Gm7809	Gm7809-001	9374	9.07397E-08	8.98218E-06	0.131562194	-7.600967804	12.59794921	95.75660633	-2.926183
Klhdc10	Klhdc10-002	1478.7752	0.026042417	0.058354777	0.131601879	-7.598675704	2.222407007	16.88735013	-2.925748
20431F20R	0431F20Rik-	513.49582	0.000583686	0.003012491	0.131620586	-7.597595736	0.781463907	5.937246851	-2.925543
Birc6	Birc6-001	5065.051	1.15677E-05	0.000170994	0.131626453	-7.59725709	6.756657991	51.33206783	-2.925479
Gsk3a	Gsk3a-002	1271.5715	0.000755821	0.003665137	0.13163764	-7.596611416	1.632818217	12.40388551	-2.925356
Gm36940	Gm36940-001	1113	4.07034E-05	0.000415279	0.131651035	-7.595838493	1.720359548	13.06757328	-2.925209
Mef2c	Mef2c-001	4040.5898	0.000261401	0.001626336	0.13170269	-7.592859325	5.367300675	40.75315898	-2.924643
Add1	Add1-014	1395.0442	0.000218497	0.001425724	0.131747317	-7.590287381	1.616811747	12.2720658	-2.924155
30012L14F	0012L14Rik-	903	0.000698726	0.003460792	0.131757282	-7.58971333	0.930257971	7.060391322	-2.924045
Zfp451	Zfp451-002	1304.6595	0.023096223	0.053122398	0.131759829	-7.589566617	1.355137586	10.28490699	-2.924018
Skil	Skil-004	3841.6836	2.69685E-06	6.57575E-05	0.131766359	-7.589190484	4.927112034	37.39279176	-2.923946
Fastkd5	Fastkd5-001	1256.3607	0.000506068	0.002696036	0.13182311	-7.585923279	1.896303568	14.38521338	-2.923325
Gm14869	Gm14869-001	185	0.014982717	0.037843516	0.131853589	-7.584169769	0.261678105	1.984611177	-2.922991
Cnr2	Cnr2-001	256.39972	0.007185327	0.021262955	0.131857938	-7.583919575	0.505489327	3.833590406	-2.922944
Kdm1a	Kdm1a-005	1295.2964	2.1249E-05	0.000263958	0.13189363	-7.581867321	1.497644539	11.35494219	-2.922553
Fam222b	Fam222b-002	4602.0054	0.000157086	0.001117384	0.131916645	-7.580544526	6.167096204	46.74994737	-2.922301
Cabp1	Cabp1-006	358.45232	0.014266088	0.036433787	0.131943509	-7.57900112	0.218188018	1.653647235	-2.922008
Yipf6	Yipf6-001	12706.639	3.06035E-09	1.51021E-06	0.131985238	-7.576604908	16.82980459	127.5127801	-2.921552
31425E22R	1425E22Rik-	1858.2369	0.001612448	0.006608563	0.131994138	-7.576094036	2.622411731	19.86763788	-2.921454
Apaf1	Apaf1-002	3754.4381	0.000412354	0.002301309	0.132018503	-7.574695784	5.747671917	43.53686623	-2.921188
Rps15a	Rps15a-004	13693.972	3.0217E-08	4.55917E-06	0.13206018	-7.572305289	23.45566365	177.6134459	-2.920733
32438A13F	2438A13Rik-	538.96556	0.001721329	0.006950662	0.132112959	-7.569280173	0.725712678	5.493122588	-2.920156
Disp1	Disp1-002	986.30002	0.000567476	0.002951117	0.132218179	-7.563256478	1.349848759	10.20925237	-2.919008
Rps15a	Rps15a-201	961.59686	0.000500085	0.002670813	0.1322926	-7.559001772	1.725203845	13.04081892	-2.918196
Dsn1	Dsn1-007	328.72134	0.000323569	0.001913369	0.132346686	-7.555912676	0.462481508	3.494469892	-2.917606
Cep57l1	Cep57l1-006	592.93184	7.41249E-05	0.000644851	0.132354601	-7.555460775	0.715643147	5.407013724	-2.91752
Phip	Phip-001	1781.7514	7.70969E-06	0.000129694	0.132413448	-7.552102992	2.530329726	19.10931069	-2.916878
Pdlim1	Pdlim1-005	3085.1593	9.74808E-06	0.000152078	0.132441654	-7.550494663	5.01222434	37.84477313	-2.916571
42436	March7-004	2784.9521	1.09768E-06	3.71497E-05	0.132460056	-7.54944571	3.948144678	29.8063039	-2.916371
Pcdhga1	Pcdhga1-002	317.20316	0.036738889	0.076824405	0.13248982	-7.547749689	0.492869918	3.720058767	-2.916047
Npm1	Npm1-006	15420.874	4.77786E-07	2.2855E-05	0.132495739	-7.547412536	29.17485823	220.1946907	-2.915982

Sharpin	Sharpin-009	276.90995	0.01047511	0.028623944	0.132582335	-7.542482961	0.401876089	3.031143551	-2.91504
Accs	Accs-001	1183.7393	5.46709E-05	0.000514418	0.132640311	-7.539186186	1.976187522	14.89884567	-2.914409
Sag	Sag-005	524.06214	5.79814E-05	0.000537155	0.132663091	-7.537891611	0.605668585	4.565464147	-2.914161
Gm26670	Gm26670-001	487	3.0641E-05	0.00033996	0.132704912	-7.535516103	0.678039895	5.109380549	-2.913706
Emd	Emd-001	2095.2856	0.003737465	0.012732063	0.132720198	-7.53464822	3.549089997	26.74114463	-2.91354
AU040972	AU040972-00	429	0.001040144	0.004694285	0.132757836	-7.532512082	0.696247652	5.244493849	-2.913131
Zfp934	Zfp934-201	183.70001	0.029864872	0.065088888	0.132810586	-7.529520302	0.273090002	2.056236712	-2.912558
Sbf1	Sbf1-007	498.04468	0.001668673	0.006784431	0.132859569	-7.526744277	0.607072898	4.569282461	-2.912026
Qrich1	Qrich1-011	176.96375	0.012772244	0.033372702	0.132860621	-7.526684652	0.278879395	2.099037258	-2.912015
Pan3	Pan3-005	3996.1587	5.18091E-07	2.36686E-05	0.132971568	-7.520404673	5.76101629	43.32517383	-2.91081
Trip12	Trip12-001	12095.991	2.12187E-08	3.6337E-06	0.132971718	-7.520396179	16.48454096	123.9702788	-2.910809
Adgrg6	Adgrg6-201	380	0.001156911	0.005108186	0.133014292	-7.517989119	0.591584531	4.44752607	-2.910347
Sdcbp	Sdcbp-004	2041.7927	3.16195E-06	7.31363E-05	0.133055353	-7.515669042	3.212532619	24.14433196	-2.909902
Huwe1	Huwe1-020	170.35738	0.014380522	0.036671347	0.133127086	-7.511619381	0.252474919	1.896495495	-2.909124
P24-500I24	24-500I24.5-(	825	8.92822E-06	0.000143261	0.133149683	-7.510344554	1.255119989	9.426383574	-2.908879
30230L23R	0230L23Rik-	415.56948	0.000446389	0.002448905	0.133265732	-7.503804478	0.673198289	5.051548333	-2.907622
Camk2d	Camk2d-024	659.12791	0.013656044	0.035194119	0.133339733	-7.49964001	0.891907388	6.688984336	-2.906821
Gm38241	Gm38241-001	226	0.00124021	0.005400785	0.133457232	-7.493037176	0.2331117	1.746714638	-2.905551
Hdac3	Hdac3-003	803.61676	0.001026891	0.004649949	0.133482791	-7.491602401	1.324810976	9.92495709	-2.905274
Sp1	Sp1-001	22548.593	1.54184E-08	3.19391E-06	0.133592759	-7.485435662	32.42787911	242.7368027	-2.904086
Nop56	Nop56-017	2094.4926	1.41651E-05	0.000197137	0.1336363	-7.482996778	3.771452712	28.22176849	-2.903616
Epb4.1	Epb4.1-201	566.00028	0.026452055	0.059071715	0.133649551	-7.482254863	0.966254779	7.229764518	-2.903473
Hnrnph3	Hnrnph3-005	1270.1516	0.001253912	0.005444431	0.133668086	-7.481217324	1.710085242	12.79351934	-2.903273
Gm37588	Gm37588-001	474	0.000643974	0.003248799	0.133671809	-7.481008931	0.679995758	5.087054338	-2.903233
Atf5	Atf5-002	417.00491	0.000443002	0.002435442	0.133675421	-7.480806833	0.70389137	5.265675368	-2.903194
Gm8624	Gm8624-002	1558	3.84688E-07	2.00847E-05	0.133681043	-7.480492213	2.660673366	19.9031464	-2.903133
Agtpbp1	Agtpbp1-001	2064.8811	0.002310731	0.008773143	0.133681431	-7.480470509	2.627202649	19.65271194	-2.903129
Ska2	Ska2-001	1199.7649	3.52104E-06	7.79545E-05	0.133698294	-7.479526991	1.950749916	14.59068664	-2.902947
Csde1	Csde1-015	395.85463	0.000393554	0.002224294	0.133725306	-7.478016173	0.647746134	4.843856063	-2.902656
00049G17R	00049G17Rik-	357.21765	0.00021906	0.001428564	0.133732755	-7.477599613	0.468932714	3.506491083	-2.902575
Cep170	Cep170-011	1686.9208	0.000444301	0.002439455	0.133793013	-7.474231848	2.628776361	19.648084	-2.901925
Zfp788	Zfp788-001	1434.9611	0.00055476	0.002900159	0.133864508	-7.470240008	1.870463259	13.97280947	-2.901155
P24-434E24	24-434E24.5-	250	0.00084406	0.003991861	0.133872637	-7.469786367	0.296921598	2.217940902	-2.901067
Wdr90	Wdr90-002	503.93583	0.008052915	0.023245879	0.133930874	-7.466538299	0.737909499	5.509629537	-2.90044
Brwd1	Brwd1-002	1340.5117	0.000641908	0.003241173	0.13393821	-7.466129352	2.029417215	15.15189144	-2.900361
Zscan2	Zscan2-003	230.86803	0.001149955	0.005087677	0.133978956	-7.463858718	0.310124562	2.314725915	-2.899922
Mrpl20	Mrpl20-003	462.81649	0.043653464	0.088068042	0.133991455	-7.463162482	0.780112732	5.822108072	-2.899787
Nedd4	Nedd4-002	2529.5826	0.000310451	0.001854135	0.13399968	-7.462704367	4.439249037	33.12874273	-2.899699
Gm19325	Gm19325-001	372	0.009084864	0.02556751	0.13401596	-7.461797807	0.734512657	5.480784935	-2.899523
Golga7	Golga7-001	12759.287	4.33326E-08	5.71384E-06	0.134037902	-7.460576347	20.81602246	155.2995248	-2.899287
Rsf1	Rsf1-001	4421.9895	2.81448E-08	4.38777E-06	0.134047704	-7.460030776	7.279517424	54.30542401	-2.899182
Hnrnpll	Hnrnpll-002	791.92123	0.000146119	0.001059615	0.134071404	-7.458712068	1.16571284	8.694716429	-2.898927
Snx5	Snx5-008	306.03706	0.000260003	0.001621765	0.134082086	-7.458117876	0.532659656	3.972638503	-2.898812
Gm9392	Gm9392-001	458	0.000505756	0.002695811	0.134084758	-7.457969239	0.751223504	5.602601782	-2.898783
Neto2	Neto2-201	1031	8.19817E-05	0.000692953	0.134118935	-7.456068757	2.2008681	16.40982388	-2.898415
Plscr2	Plscr2-003	539.87203	0.000154399	0.00110331	0.134158302	-7.453880874	0.809902935	6.036919997	-2.897992
Mettl2	Mettl2-005	224.94726	0.001145224	0.005072085	0.134166292	-7.453436967	0.304695698	2.271030176	-2.897906
Srsf10	Srsf10-003	1077.4106	0.007740707	0.022530739	0.13419703	-7.451729769	1.696748719	12.64371294	-2.897575
Gm10184	Gm10184-201	1306	0.008132543	0.023405608	0.134214106	-7.450781648	2.503248114	18.65115511	-2.897392
Gm12949	Gm12949-001	76	0.017117926	0.04199176	0.134218545	-7.450535255	0.11788713	0.878322222	-2.897344
Erc1	Erc1-011	10867.588	4.74564E-09	1.8062E-06	0.134233294	-7.449716616	14.01493036	104.4072596	-2.897186
Gm25731	Gm25731-201	125	0.003053107	0.010901131	0.134252105	-7.448672783	0.163637016	1.218878589	-2.896983
Gltscr2	Gltscr2-003	5916.6101	5.29916E-06	0.000101168	0.134299363	-7.446051689	10.18903476	75.86807951	-2.896476
30565N06R	0565N06Rik-	2015	5.20645E-06	0.000100124	0.134315615	-7.445150746	2.938410677	21.87691044	-2.896301
Ccdc34	Ccdc34-002	1168.851	0.019926022	0.047380212	0.13432984	-7.444362303	2.128295982	15.84380638	-2.896148
Zfp182	Zfp182-004	825.83499	0.000259091	0.001617549	0.134411866	-7.439819358	1.103923141	8.212988756	-2.895268
Rnf7	Rnf7-001	7707.7015	4.90119E-09	1.8062E-06	0.134431929	-7.43870899	10.9237683	81.25873344	-2.895052
Zfp58	Zfp58-001	356.60994	0.001298345	0.005593501	0.134475032	-7.436324705	0.656847719	4.884532917	-2.89459
Dap3	Dap3-012	412.31061	0.045693315	0.091338626	0.134491792	-7.435397993	0.688297284	5.117764244	-2.89441
Pphln1	Pphln1-201	1684.0784	0.000379685	0.002163042	0.134529168	-7.433332234	2.680111508	19.92215926	-2.894009
Gm24809	Gm24809-201	586	8.20011E-05	0.000692953	0.134532741	-7.433134806	0.818608262	6.084825562	-2.893971
Nod1	Nod1-202	5188.8678	0.000759346	0.003679626	0.1345367	-7.432916063	7.830671014	58.20472037	-2.893928
Apbb2	Apbb2-006	1392.1835	0.001185228	0.005208374	0.134536826	-7.432909107	2.197905059	16.33682853	-2.893927
Gm5580	Gm5580-001	123	0.007432717	0.021844049	0.134649507	-7.426688894	0.180347863	1.339387469	-2.892719
Arfip1	Arfip1-001	5304.8369	9.95947E-07	3.48429E-05	0.134657456	-7.42625049	7.560728586	56.14786437	-2.892634
RP23-339I6.	P23-339I6.1-0	285	0.006270743	0.019090929	0.134689575	-7.424479571	0.441602785	3.278670855	-2.89229
Erbb2ip	Erbb2ip-001	7578.7071	4.01486E-05	0.000411334	0.134772599	-7.419905872	12.05714658	89.46289269	-2.891401
Senp1	Senp1-004	2502.9924	5.7887E-07	2.51284E-05	0.134791968	-7.418839701	3.257473599	24.16667446	-2.891194
Supt3	Supt3-004	353.32885	0.001201146	0.005259169	0.134823706	-7.417093278	0.558052643	4.139128508	-2.890854
Rcor3	Rcor3-011	2005	5.26116E-06	0.000100729	0.134849179	-7.415692187	2.529399704	18.75724962	-2.890581
00017E21R	0017E21Rik-	279.58493	0.001686212	0.006838396	0.134858803	-7.415162948	0.337158649	2.500086322	-2.890478
E2f3	E2f3-001	1922.6892	5.83661E-05	0.000539843	0.13486856	-7.414626513	3.670554216	27.21578861	-2.890374
Zeb1	Zeb1-002	3258.2263	3.01294E-06	7.07801E-05	0.134876383	-7.414196445	4.784570457	35.47374528	-2.89029
Rpl30-ps8	Rpl30-ps8-20	1363	1.8095E-07	1.33171E-05	0.134950168	-7.410142691	2.20757288	16.35843004	-2.889501
Exoc1	Exoc1-006	1153.7005	1.03503E-06	3.57718E-05	0.134960567	-7.409571736	1.61384928	11.95793201	-2.88939
Med20	Med20-001	2307.2142	9.55129E-06	0.000149923	0.134973152	-7.408880862	3.193764237	23.66221873	-2.889256
Cep295	Cep295-010	153.17163	0.004485104	0.014694357	0.135001336	-7.407334119	0.168075338	1.244990187	-2.888954
P23-200E11	23-200E11.1-	879	0.001238948	0.005397351	0.135047274	-7.404814426	1.472149629	10.90099481	-2.888464
Tmem104	Tmem104-002	1990.3454	0.02612344	0.05847512	0.135081667	-7.40292909	2.720808019	20.14194883	-2.888096
Tardbp	Tardbp-001	6117.1237	0.001737949	0.007009508	0.135143344	-7.399550537	10.78261174	79.78648048	-2.887438
Aqr	Aqr-002	812.72724	8.4703E-06	0.000138252	0.135178959	-7.397600977	0.959334532	7.096774068	-2.887057
Tnfrsf26	Tnfrsf26-201	5329	6.89103E-07	2.78981E-05	0.135190897	-7.396947764	10.09237056	74.65273785	-2.88693
Itsn1	Itsn1-003	1537.4969	0.00049465	0.002650421	0.135197716	-7.396574658	2.081708962	15.39751575	-2.886857
Spag9	Spag9-203	1322.6517	0.032103505	0.068945638	0.135224425	-7.395113714	2.43206952	17.98543066	-2.886572
Atp7a	Atp7a-001	3608.5009	0.008810282	0.024917601	0.135250945	-7.393663673	5.566459269	41.15652768	-2.886289
Rps8	Rps8-005	2497.1182	0.00014845	0.001071984	0.135347554	-7.388386223	4.303082002	31.79283178	-2.885259
Nqo2	Nqo2-201	704.14724	0.035218236	0.074265766	0.135355808	-7.387935659	0.867090982	6.406012387	-2.885171
Ifi47	Ifi47-001	2258.0241	0.00020695	0.001367292	0.135376791	-7.386790557	3.747312588	27.68061324	-2.884948
Akr1e1	Akr1e1-001	2167.379	0.002009527	0.007857782	0.135405051	-7.385248862	3.309057248	24.43821127	-2.884647
Rbm3	Rbm3-002	3467.1002	0.002222878	0.008510346	0.135437532	-7.383477743	6.39676168	47.23034749	-2.884301
Dhdds	Dhdds-006	347.7901	0.031102966	0.067286293	0.135520953	-7.378932786	0.616927785	4.55226866	-2.883412
Clcc1	Clcc1-004	177.95727	0.019771098	0.047087137	0.135529082	-7.3784902	0.177634809	1.3106767	-2.883326
Gm14253	Gm14253-001	475	3.78672E-05	0.00039433	0.135613884	-7.373876252	0.743535822	5.482741141	-2.882423
Alms1	Alms1-003	248.59961	0.007405917	0.021787722	0.135627816	-7.373118788	0.42970235	3.168246469	-2.882275
Kdm1a	Kdm1a-001	2170.3796	0.010518969	0.028699647	0.135677165	-7.370437042	3.268422634	24.08970325	-2.88175
Ccnd1	Ccnd1-001	14140.763	0.001254001	0.005444431	0.135765472	-7.365643013	25.85326514	190.4259217	-2.880811
Pex3	Pex3-004	1414.2815	0.002167384	0.008334219	0.135777255	-7.365003816	1.770305176	13.03830438	-2.880686
Gdap10	Gdap10-201	5124	3.81543E-06	8.16753E-05	0.13580284	-7.363616272	6.958306336	51.23829777	-2.880414
Tlr3	Tlr3-201	5250.8706	9.14172E-07	3.33089E-05	0.135840187	-7.361591727	7.065870306	52.01605239	-2.880018
Synj2bp	Synj2bp-002	248.21033	0.002688621	0.009862404	0.135848235	-7.361155653	0.388116243	2.856984076	-2.879932
Bak1	Bak1-001	1977.195	0.011094073	0.029893601	0.135879543	-7.359459556	3.042829084	22.39357758	-2.8796
Frrs1	Frrs1-005	3121.2111	0.002102234	0.008133927	0.13592171	-7.357176408	5.092779581	37.46847779	-2.879152
Arl14ep	Arl14ep-001	4314.2322	8.45304E-07	3.1832E-05	0.135932833	-7.356574383	7.184599125	52.85403787	-2.879034
Smim10l1	Smim10l1-00	637.89691	0.021795124	0.050805349	0.135956945	-7.355269725	0.940412975	6.916991082	-2.878778
Gm28893	Gm28893-001	164	0.006016739	0.018495182	0.135964494	-7.354861338	0.236820625	1.741782857	-2.878698
P23-120C8	23-120C8.4-(	184	0.002733316	0.009998513	0.135997928	-7.353053187	0.217344416	1.598145054	-2.878343
Mpdz	Mpdz-004	406.90724	0.038056484	0.078976889	0.136031431	-7.351242243	0.618388711	4.545925211	-2.877988

Gm2546	Gm2546-001	244	0.002572643	0.009525731	0.136048741	-7.350306877	0.447590755	3.289929406	-2.877804
Exoc7	Exoc7-004	383.22971	0.005082524	0.016202202	0.136098004	-7.347646318	0.519805624	3.819347876	-2.877282
Dennd1b	Dennd1b-008	990.27165	8.83778E-05	0.00072798	0.136264601	-7.338663086	1.399913571	10.27349405	-2.875517
920006O11Rik	20006O11Rik-	234	0.014896657	0.037692856	0.136281742	-7.337740065	0.50177079	3.681863627	-2.875336
Tars2	Tars2-007	77.608752	0.020893421	0.049128083	0.136311965	-7.336113156	0.148654473	1.090546032	-2.875016
RP23-342N8	23-342N8.2-0	1620.279	2.16453E-05	0.000266994	0.13636771	-7.333114292	1.714682786	12.57396484	-2.874426
Kifc3	Kifc3-202	462.42331	0.005401971	0.017035033	0.136375454	-7.332697864	0.611318707	4.482615379	-2.874344
Gm26823	Gm26823-201	1707	1.53834E-06	4.54992E-05	0.136384408	-7.332216462	2.693153203	19.74678225	-2.874249
Gm13696	Gm13696-001	55.710577	0.0207128	0.04880999	0.136404417	-7.331140906	0.097622592	0.715684979	-2.874038
Rfwd2	Rfwd2-002	4589.7902	3.01669E-08	4.55917E-06	0.136423059	-7.330139124	7.519146739	55.11639169	-2.873841
Gm27177	Gm27177-005	138.31184	0.015718715	0.039270988	0.136480095	-7.327075783	0.197382553	1.446236924	-2.873238
Krr1	Krr1-001	4698.8957	1.20935E-06	3.94853E-05	0.136483116	-7.326913586	7.810528497	57.22706736	-2.873206
Mapk1	Mapk1-004	185.47278	0.010081157	0.027770664	0.136556322	-7.322985729	0.259833224	1.902754992	-2.872432
Mapkapk5	Mapkapk5-20	1347.5043	0.000793001	0.003801833	0.136574513	-7.322010366	1.889787899	13.83704658	-2.87224
Gm28906	Gm28906-001	666	2.99544E-05	0.000335163	0.136583843	-7.321510211	0.987877097	7.23275225	-2.872141
Btf3	Btf3-003	748.6867	0.034673755	0.07338871	0.136590876	-7.321133216	1.091051817	7.987735696	-2.872067
Dopey1	Dopey1-008	1608.5959	0.000937656	0.004333971	0.136603149	-7.320475434	1.964550055	14.38144042	-2.871937
Tmem57	Tmem57-004	317.65152	0.024045101	0.05477153	0.136625588	-7.319273188	0.16276367	1.191311767	-2.8717
Imp4	Imp4-008	402.3646	0.000801128	0.003834354	0.136653487	-7.317778889	0.660585124	4.834015875	-2.871406
00028K03Rik	0028K03Rik-	441.38013	0.008117773	0.023381862	0.136694658	-7.315574844	0.582764168	4.263254885	-2.870971
Slc35a1	Slc35a1-001	3909.9391	5.72525E-06	0.000106453	0.13669977	-7.315301272	5.515394733	40.3467741	-2.870917
Gm22906	Gm22906-201	626	0.000676187	0.003370112	0.136853509	-7.307083382	0.907234265	6.62923642	-2.869296
Gm37503	Gm37503-001	147	0.021204767	0.049723625	0.136887296	-7.305279814	0.164176682	1.199356602	-2.86894
Crtc3	Crtc3-003	258.27827	0.005742789	0.017821012	0.136916203	-7.303737445	0.397273819	2.901583671	-2.868635
Mon1b	Mon1b-202	2362.6921	0.000199154	0.001328337	0.136937746	-7.30258843	3.084997485	22.52846694	-2.868408
Ceacam2	Ceacam2-002	1049.501	0.02774271	0.061381641	0.137039985	-7.29714033	1.240712347	9.053652102	-2.867331
Chek2	Chek2-006	613.76435	5.12935E-05	0.000490316	0.137077676	-7.295133899	0.901210364	6.574450276	-2.866934
Tmem184b	Tmem184b-20	7872.0701	2.28855E-06	5.93809E-05	0.137096872	-7.294112424	7.673765132	55.97330559	-2.866732
Cul5	Cul5-005	1291.6543	7.51331E-05	0.000649826	0.137229024	-7.287088196	1.477563475	10.76713536	-2.865342
Hdhd2	Hdhd2-007	218.08396	0.036812394	0.076959318	0.137271116	-7.284853732	0.236907658	1.725837635	-2.8649
530040E14Rik	0040E14Rik-	2663.6881	5.18122E-08	6.42959E-06	0.137334319	-7.281501156	4.073115803	29.65839743	-2.864236
Mrps2	Mrps2-001	706.09932	0.01724478	0.04221526	0.137369952	-7.27961233	1.197444678	8.71693304	-2.863862
Gfm1	Gfm1-001	2303.2579	5.58046E-05	0.000521788	0.137372959	-7.279453017	3.715281668	27.04521835	-2.86383
Ifi203	Ifi203-202	1128.5734	0.000893125	0.004181753	0.137385392	-7.278794265	1.727769253	12.57607693	-2.863699
Trappc1	Trappc1-002	1249.5311	0.0051247	0.016316908	0.137418908	-7.277018977	2.058246506	14.97789888	-2.863348
Foxj3	Foxj3-003	938.79391	6.95586E-05	0.000614967	0.137489143	-7.273301571	1.265047434	9.201071488	-2.86261
Ftsj3	Ftsj3-003	388.13518	0.002031832	0.007930512	0.137510938	-7.272148779	0.619436198	4.504632189	-2.862382
700034I23Rik	00034I23Rik-0	375	0.027620494	0.061158645	0.137535081	-7.270872235	0.587282591	4.270056684	-2.862128
Bcl2	Bcl2-002	565.54972	0.000463016	0.002520388	0.137550705	-7.270046321	1.188745009	8.64223128	-2.861965
Zcwpw1	Zcwpw1-005	737	8.23575E-06	0.000135678	0.137595915	-7.267657596	1.029782289	7.484105073	-2.86149
Gm16596	Gm16596-201	298.97339	0.028459361	0.062598264	0.137662999	-7.264116072	0.374789266	2.722512729	-2.860787
Gbas	Gbas-004	185.58841	0.002328282	0.008822179	0.13769924	-7.262204226	0.24290568	1.764030653	-2.860408
RP23-393E10	23-393E10.2-0	449	0.000520978	0.002757032	0.137712358	-7.261512428	0.645831598	4.689714173	-2.86027
Alox5ap	Alox5ap-003	494.3649	0.044741195	0.089770705	0.137733843	-7.2603797	0.946448428	6.871574951	-2.860045
Srcap	Srcap-006	801.42743	0.02121606	0.049739889	0.137758907	-7.25905875	1.389483016	10.08633885	-2.859782
530001G21Rik	0001G21Rik-	194.87455	0.003159249	0.011190845	0.137759698	-7.259017078	0.310071265	2.250812608	-2.859774
Gm16459	Gm16459-001	153	0.001805014	0.007219883	0.137767867	-7.258586635	0.229739641	1.667585088	-2.859689
Gm38319	Gm38319-001	709	5.39343E-05	0.000509446	0.137818717	-7.255908506	1.122442105	8.14433722	-2.859156
Eif4a1	Eif4a1-012	8730.0301	1.40928E-05	0.000196771	0.137863261	-7.253564108	17.05158857	123.6847908	-2.85869
Ncapg2	Ncapg2-201	2564	0.00181652	0.007258872	0.137889557	-7.252180802	4.576545871	33.1899381	-2.858415
Usp33	Usp33-001	373.77258	0.01587105	0.039564996	0.137912212	-7.250989504	0.655187298	4.75075622	-2.858178
Gm38178	Gm38178-001	428	0.000338661	0.001981747	0.137949634	-7.249022495	0.570435975	4.135103214	-2.857786
Thap6	Thap6-001	757.48668	3.12975E-06	7.26245E-05	0.137953971	-7.248794578	1.219529332	8.840117607	-2.857741
AC132393.1	AC132393.1-20	425.18455	0.008130091	0.023405608	0.138000977	-7.246325527	0.701348622	5.082200422	-2.85725
RP23-234P16	23-234P16.4-0	360	0.001033841	0.004672864	0.138036748	-7.244447663	0.639843627	4.635313671	-2.856876
Kpna2	Kpna2-007	756.04189	0.047337758	0.093947271	0.138083762	-7.24198115	1.488080518	10.77665106	-2.856384
Gcnt4	Gcnt4-201	191	0.019406027	0.046394749	0.138122569	-7.239946402	0.265252966	1.920417258	-2.855979
Gm4707	Gm4707-013	84.440755	0.026067834	0.058394036	0.138155418	-7.238224988	0.115594403	0.836698296	-2.855636
Sirt2	Sirt2-201	1371.4234	0.001528597	0.00634219	0.138209864	-7.235373608	1.446329034	10.46473092	-2.855068
Lsm2	Lsm2-001	976.94151	0.035000985	0.073921424	0.138241839	-7.233700047	1.55646692	11.25901483	-2.854734
Zfp882	Zfp882-001	1346.8038	1.02148E-05	0.000156886	0.138260015	-7.232749109	2.073244983	14.9952608	-2.854544
Gm38047	Gm38047-001	1003	4.39887E-06	8.95195E-05	0.138313163	-7.229969854	1.497032767	10.82350177	-2.85399
Ppdpf	Ppdpf-006	1356.65	0.008101552	0.023344206	0.138321022	-7.229559061	1.580740525	11.42805698	-2.853908
RP23-382I10	23-382I10.1-0	2398	2.7841E-07	1.66125E-05	0.138340898	-7.2285204	4.13083587	29.85983135	-2.8537
Krit1	Krit1-004	1756.5996	2.98181E-06	7.02387E-05	0.138361288	-7.227455132	2.48755862	17.97871832	-2.853488
Ttc14	Ttc14-001	14231.336	2.27637E-08	3.80321E-06	0.138375762	-7.226699123	21.09242996	152.4286451	-2.853337
Pacsin3	Pacsin3-002	127.51449	0.042153494	0.085698175	0.138396785	-7.225601381	0.162045638	1.170877185	-2.853118
Rbm25	Rbm25-016	325.0655	0.001453706	0.006110634	0.138415304	-7.224634618	0.572975352	4.139537567	-2.852925
Fuca2	Fuca2-006	530.13758	0.030956061	0.067044712	0.138449852	-7.222831867	0.883612998	6.382188121	-2.852565
Mamstr	Mamstr-003	1080.2184	0.028923998	0.063389233	0.138539832	-7.218140713	0.777727675	5.613747791	-2.851627
Rap1gds1	Rap1gds1-005	2853.2274	0.004055643	0.013566342	0.138569975	-7.216570519	3.898425209	28.13326044	-2.851313
Pbx1	Pbx1-002	33386.701	1.06067E-07	9.81834E-06	0.138577743	-7.216166019	47.49969403	342.7656779	-2.851233
Ppp2r3a	Ppp2r3a-002	3215.0925	8.98753E-08	8.98218E-06	0.138600113	-7.215001316	5.603598676	40.42997182	-2.851
Suds3	Suds3-004	551.27018	0.000409664	0.002292273	0.138630618	-7.213413704	1.039293191	7.496851749	-2.850682
Ccdc171	Ccdc171-001	490.32526	0.030108713	0.065536918	0.138632566	-7.213312367	0.87949798	6.344093657	-2.850662
Tmem87b	Tmem87b-003	2688.6511	2.19969E-06	5.81321E-05	0.138651015	-7.212352561	4.17393625	30.1038998	-2.85047
Gm38257	Gm38257-001	1258	1.77399E-05	0.000231677	0.138735896	-7.207939911	1.72119674	12.40628268	-2.849587
C5ar1	C5ar1-201	1052.4734	0.043044313	0.087075054	0.138745196	-7.20745676	2.917441249	21.02733165	-2.84949
910305D13Rik	0305D13Rik-	2023.3202	3.64415E-05	0.000383447	0.138794198	-7.204912108	3.060614168	22.05145608	-2.848981
Slc15a1	Slc15a1-201	264	0.000657101	0.003298507	0.138888221	-7.200034646	0.408301474	2.939784759	-2.848004
Gm38384	Gm38384-001	450	6.3494E-05	0.000574701	0.138934695	-7.197626179	0.550959767	3.965602441	-2.847521
Suco	Suco-002	450.34757	0.000709651	0.003499828	0.138992856	-7.194614354	0.670676932	4.825261884	-2.846917
030039L03Rik	0039L03Rik-	810.13708	9.19919E-06	0.000146108	0.139068492	-7.190701388	1.138998363	8.190197107	-2.846132
Ifi203	Ifi203-002	1761.3406	0.002989517	0.010724936	0.139074408	-7.190395535	2.549941831	18.33509035	-2.846071
Smc4	Smc4-002	16562.208	1.69494E-06	4.81028E-05	0.139106931	-7.188714405	25.74100235	185.0447144	-2.845734
Lrrcc1	Lrrcc1-001	2913.3476	0.000213787	0.001400745	0.13915114	-7.186430532	3.97690972	28.57978544	-2.845275
AY074887	AY074887-00	183.39504	0.00411114	0.013707801	0.13919049	-7.184398891	0.219162033	1.574547468	-2.844867
Lrrk2	Lrrk2-004	719.32055	4.91751E-05	0.000474837	0.139219024	-7.182926389	1.061993033	7.628217778	-2.844572
Lyz2	Lyz2-201	31628	0.007449474	0.021887664	0.139252933	-7.181177301	88.57434193	636.0680537	-2.84422
Atad5	Atad5-201	1621.5235	0.001343831	0.005741075	0.139309866	-7.178242472	3.474717378	24.94236386	-2.843631
Vim	Vim-005	9745.837	5.49197E-07	2.4488E-05	0.139322253	-7.177604287	15.93288366	114.359934	-2.843502
Sec63	Sec63-003	1792.697	0.000110122	0.000857395	0.139417234	-7.172714405	3.294773174	23.63246701	-2.842519
Arpc1b	Arpc1b-008	390.60005	0.000409384	0.002291826	0.139544901	-7.166152204	0.516446247	3.700932408	-2.841199
Tubgcp4	Tubgcp4-007	698.50539	0.001189429	0.005222144	0.139572051	-7.164758237	0.876021028	6.276478875	-2.840918
Pphln1	Pphln1-202	3432.3104	0.001330049	0.005694983	0.139608535	-7.16288584	4.459005562	31.9393478	-2.840541
Prim2	Prim2-002	161.30407	0.015903774	0.039629265	0.139662624	-7.160111769	0.234841793	1.681493489	-2.839982
Ppp1r3b	Ppp1r3b-201	790	0.017052494	0.041870236	0.139682738	-7.159080734	1.702395967	12.18759017	-2.839774
Hells	Hells-001	1250.6895	0.000778267	0.003746927	0.139730879	-7.15661426	2.620464695	18.75365501	-2.839277
Gm12940	Gm12940-002	1146.6799	0.000425844	0.002361995	0.139761372	-7.15505284	1.825469124	13.06132804	-2.838962
Pcna-ps2	Pcna-ps2-201	1964	0.000213813	0.001400745	0.139762693	-7.154985207	3.538055524	25.31473493	-2.838949
Cdc42bpa	Cdc42bpa-005	17093.316	0.000359286	0.002071991	0.139763666	-7.154935364	25.49398639	182.4078248	-2.838939
C1qbp	C1qbp-003	216.64918	0.002052537	0.007987658	0.139817807	-7.152164816	0.277294487	1.983255871	-2.83838
Mfsd4	Mfsd4-004	4549.6115	1.02266E-06	3.5458E-05	0.13986574	-7.149713733	6.112430133	43.70212567	-2.837885
RP23-301L9	23-301L9.3-0	336	0.008206857	0.023565148	0.139883133	-7.14882475	0.530943892	3.795624839	-2.837706
Gm27690	Gm27690-201	113	0.016984483	0.041739152	0.139888205	-7.148565549	0.148409398	1.060914311	-2.837654
Vcam1	Vcam1-004	10215.309	1.60922E-08	3.19391E-06	0.139907805	-7.14756408	19.52539225	139.5589923	-2.837452
Tcirg1	Tcirg1-005	1900.2732	3.68673E-05	0.000386502	0.139932704	-7.146292268	3.31258174	23.67267728	-2.837195

Cebpg	Cebpg-001	10293.646	9.47944E-09	2.49747E-06	0.139949469	-7.145436197	15.11162591	107.9791588	-2.837022
Tcf7	Tcf7-001	695.66388	0.03272208	0.070036948	0.140027401	-7.141459383	0.864468916	6.173569649	-2.836219
Cyth1	Cyth1-201	626.63923	0.001709832	0.006912387	0.140038927	-7.140871608	0.614750657	4.389855511	-2.8361
Pld1	Pld1-005	158.42421	0.007219049	0.021347991	0.140040334	-7.140799856	0.215928331	1.541900998	-2.836086
Ostm1	Ostm1-005	799.80171	0.000456644	0.002494429	0.140087694	-7.138385755	1.064551855	7.5991818	-2.835598
Cdadc1	Cdadc1-204	1622.5604	4.10988E-05	0.000418068	0.140101994	-7.137657146	1.926132462	13.74807313	-2.835451
Gtpbp10	Gtpbp10-001	1835.9938	9.12515E-05	0.000746267	0.140104738	-7.137517364	3.173594506	22.65158589	-2.835422
Gm4349	Gm4349-001	640	0.000109968	0.00085639	0.140105765	-7.137465022	1.110304738	7.924761233	-2.835412
Slc3a2	Slc3a2-202	6700.7551	0.000184327	0.001255359	0.140113293	-7.137081569	10.09213227	72.02837124	-2.835334
Itga4	Itga4-001	10155.035	1.41418E-05	0.000197053	0.140121571	-7.136659913	20.12471585	143.6232528	-2.835249
Atf7	Atf7-002	33969.574	1.36032E-07	1.12811E-05	0.140158466	-7.134781266	49.89949908	356.0220113	-2.834869
Mcmbp	Mcmbp-001	8940.7892	1.02872E-07	9.64322E-06	0.140304806	-7.127339595	14.10596489	100.5380021	-2.833364
Usp34	Usp34-007	1725.0262	8.67779E-07	3.22358E-05	0.140373185	-7.123867733	2.849115477	20.29672182	-2.832661
Arhgap39	Arhgap39-008	356.15261	0.001327456	0.005685957	0.140382561	-7.123391891	0.519910634	3.703527196	-2.832564
Ppic	Ppic-201	10262	4.09657E-05	0.00041721	0.14044622	-7.120163134	15.09052668	107.4470118	-2.83191
Tnfrsf22	Tnfrsf22-002	9365.4548	1.51076E-07	1.2118E-05	0.140595797	-7.112588139	15.10578005	107.441192	-2.830375
Thoc2	Thoc2-006	3277.2673	5.77021E-06	0.000106827	0.140598678	-7.112442423	4.893449011	34.80437434	-2.830345
30401A14R	0401A14Rik-	507	0.001472714	0.006170841	0.140605725	-7.112085923	0.830826342	5.908908332	-2.830273
Rnf13	Rnf13-004	649.51726	0.029268047	0.064003998	0.140651428	-7.109774966	0.810819581	5.764744759	-2.829804
Gm15839	Gm15839-001	290	0.000369887	0.002116023	0.140707874	-7.106922792	0.422126577	3.000020989	-2.829225
Ppa2	Ppa2-002	301.12342	0.006249337	0.019049467	0.140760385	-7.10427156	0.216625633	1.538967323	-2.828687
Paxbp1	Paxbp1-003	1077.2127	0.00023155	0.001487914	0.140830005	-7.100759526	1.792922673	12.73111275	-2.827973
Psma3	Psma3-001	1975.8173	3.54002E-05	0.000375606	0.140874008	-7.098541534	3.212236035	22.80219092	-2.827523
Abca3	Abca3-004	310.51439	0.026496416	0.059151771	0.140879275	-7.098276165	0.430126163	3.053154293	-2.827469
Shprh	Shprh-001	7243.5609	1.04686E-06	3.59988E-05	0.140969082	-7.093754076	10.44566323	74.0989661	-2.826549
Nktr	Nktr-002	2547.1262	1.42247E-06	4.34873E-05	0.141071841	-7.088586881	4.15914599	29.48246771	-2.825498
30562O15R	0562O15Rik-	886.56546	0.00384949	0.01302173	0.141124627	-7.085935484	1.841236875	13.04688571	-2.824958
Tpd52	Tpd52-003	2197.318	0.009228076	0.025879112	0.14112751	-7.08579073	4.858817167	34.42856164	-2.824929
Zmym5	Zmym5-201	2901.4073	0.000615365	0.003137749	0.141140818	-7.085122601	4.076830203	28.88484181	-2.824793
P23-461P7.	23-461P7.1-0	809	0.00027979	0.00171305	0.141214498	-7.081425863	1.620876605	11.47811751	-2.82404
Stk38	Stk38-012	572.23139	0.003138474	0.011128763	0.141214998	-7.081400825	1.065829004	7.547562391	-2.824035
Derl2	Derl2-007	853.53101	0.015225117	0.038303237	0.141272948	-7.078496	1.506247348	10.66196582	-2.823443
Naa15	Naa15-003	469.02275	0.000251372	0.001581449	0.141291467	-7.077568224	0.878800748	6.21977225	-2.823254
Ints12	Ints12-001	2306.8667	1.94766E-05	0.000247431	0.14130999	-7.076640495	4.042691059	28.60867126	-2.823065
Mfsd1	Mfsd1-001	7001.576	1.65686E-07	1.26693E-05	0.141364283	-7.073922617	10.76143625	76.12556725	-2.82251
Gm38091	Gm38091-001	574	0.002360692	0.00891048	0.14137464	-7.073404408	0.978448329	6.920960725	-2.822405
Mtm1	Mtm1-009	800.328	0.000135536	0.001003913	0.141412684	-7.071501462	1.303127192	9.215065847	-2.822017
Arid4b	Arid4b-005	1735.877	7.28758E-06	0.0001248	0.141438366	-7.070217401	2.737992181	19.35819996	-2.821755
Wdr83	Wdr83-010	238.01308	0.042383919	0.086023657	0.14146033	-7.069119655	0.435892021	3.081372853	-2.821531
Nr1h2	Nr1h2-004	1760.3448	2.30053E-05	0.000278815	0.141576106	-7.06333879	2.401481606	16.96247818	-2.82035
B3gnt9	B3gnt9-201	299.68282	0.002272399	0.008659287	0.141655301	-7.059389894	0.530874531	3.747650296	-2.819544
Dnajb14	Dnajb14-002	1013.4389	1.84472E-06	5.09293E-05	0.141716392	-7.056346739	1.614853988	11.39496967	-2.818921
P23-18K20	23-18K20.2-0	1532.1669	1.4574E-06	4.40645E-05	0.141717002	-7.056316342	2.434245917	17.17680924	-2.818915
Gm2694	Gm2694-003	669	0.000315726	0.001877688	0.141774273	-7.053465883	0.974672373	6.874818331	-2.818332
Bcor	Bcor-005	3360.2709	0.011183829	0.030078221	0.14183333	-7.050528941	5.719535526	40.32575076	-2.817731
Ccnt2	Ccnt2-005	9257.2833	7.98187E-08	8.43762E-06	0.141867229	-7.048844234	13.96675758	98.44949866	-2.817387
Gpatch4	Gpatch4-201	551.35198	0.022705404	0.052410035	0.141918439	-7.046300743	1.010505255	7.12032393	-2.816866
Lrrc8b	Lrrc8b-001	3186	0.000550015	0.002879989	0.141967272	-7.043877004	6.861908196	48.33443735	-2.81637
Gca	Gca-001	799.14129	0.000176787	0.001216345	0.141990885	-7.042705606	1.750747833	12.33000157	-2.81613
Myadm	Myadm-201	38002.628	4.40145E-07	2.15357E-05	0.142028781	-7.040826478	56.37441631	396.922483	-2.815745
Wdr77	Wdr77-002	682.36715	0.000194716	0.001306114	0.142045415	-7.040001969	1.479405575	10.41501816	-2.815576
Sass6	Sass6-002	1096.4947	2.76087E-05	0.000317084	0.142080636	-7.038256765	1.613461111	11.35595358	-2.815218
Gm13372	Gm13372-001	294.63013	0.012169886	0.032156787	0.142118052	-7.036403806	0.267049788	1.879070142	-2.814838
Zbtb26	Zbtb26-001	1417.9202	1.8864E-06	5.16873E-05	0.142178406	-7.033416859	2.376261221	16.71323573	-2.814226
Mfn1	Mfn1-001	3724.2449	2.68265E-06	6.56313E-05	0.142222581	-7.031232284	5.831611678	41.0034163	-2.813778
Sgpp1	Sgpp1-201	3449	8.73433E-06	0.000141357	0.14222259	-7.031231827	5.934518999	41.72697886	-2.813777
Wbp5	Wbp5-002	639.82326	0.030547119	0.066309978	0.142265368	-7.029117575	1.223261342	8.598447796	-2.813344
Eif2b3	Eif2b3-201	701.49995	0.001458614	0.006128254	0.142265497	-7.029111206	1.094194006	7.691211352	-2.813342
P23-378O9	23-378O9.1-0	567	0.003342692	0.011687945	0.142282873	-7.028252821	0.719432784	5.056355495	-2.813166
Ppp1r21	Ppp1r21-004	135.73537	0.011895693	0.031603124	0.142349827	-7.024947059	0.179493298	1.260930918	-2.812487
Zcchc9	Zcchc9-002	932.70454	1.48477E-05	0.000203822	0.142352445	-7.024817859	1.585505038	11.13788411	-2.812461
Lrrc74b	Lrrc74b-002	380.29808	0.000153578	0.001100434	0.142352608	-7.024809811	0.543642043	3.818981959	-2.812459
Ankrd12	Ankrd12-003	1563.6326	0.000797924	0.003821687	0.142356221	-7.024631562	2.367741286	16.63251017	-2.812423
Tmem123	Tmem123-002	2072.0369	0.005889723	0.018179796	0.142369311	-7.023985678	4.419827884	31.04480776	-2.81229
Oraov1	Oraov1-002	573.60456	0.000314132	0.001870806	0.142411461	-7.021906742	0.619005289	4.34659741	-2.811863
Lrrc74b	Lrrc74b-003	56.701846	0.015471878	0.038804322	0.142480023	-7.018527779	0.08114099	0.569490293	-2.811168
Prr3	Prr3-002	129.07407	0.027954319	0.061730201	0.142520715	-7.016523868	0.147956989	1.038143742	-2.810756
Rc3h2	Rc3h2-004	1372.2631	1.33693E-05	0.000189528	0.142556448	-7.01476515	1.939893254	13.6078956	-2.810395
Zfp961	Zfp961-012	235.52445	0.004460378	0.014632946	0.142574027	-7.013900212	0.401401887	2.815392782	-2.810217
Smco1	Smco1-001	1507	2.70275E-07	1.6355E-05	0.142738847	-7.005801315	2.128131468	14.90926624	-2.80855
Slmapos2	Slmapos2-001	19211	1.45898E-05	0.000201414	0.142740324	-7.005728828	38.13123753	267.1371128	-2.808535
Coq5	Coq5-003	117.14847	0.022667896	0.052351682	0.142812385	-7.002193823	0.155503974	1.088868969	-2.807807
10014B01R	0014B01Rik-	369.65332	0.000833171	0.003951824	0.142905225	-6.997644782	0.52752173	3.691409682	-2.806869
Fam111a	Fam111a-201	3135.8049	0.002008154	0.007855101	0.142940765	-6.995904899	6.932257154	48.49741179	-2.806511
G2e3	G2e3-005	907.9244	0.000970806	0.004444011	0.142950996	-6.995404194	1.484455119	10.38436356	-2.806407
Guf1	Guf1-001	975.51447	0.010802519	0.02926412	0.143008895	-6.992572059	1.72959678	12.09433012	-2.805823
Cd200	Cd200-009	1563.0061	0.000177462	0.001220008	0.143041405	-6.990982775	2.786129192	19.47778119	-2.805495
Ankfy1	Ankfy1-002	1225.2615	0.007301563	0.021531851	0.14308053	-6.989071142	1.460508133	10.20759525	-2.805101
P23-396K22	23-396K22.4-	237	0.002264378	0.008632562	0.143097214	-6.988256258	0.345706678	2.415886857	-2.804933
Egfl7	Egfl7-014	237.38925	0.008766731	0.024830348	0.143122995	-6.986997449	0.102038924	0.712945702	-2.804673
Gm26608	Gm26608-001	585	0.000131804	0.000983076	0.143127436	-6.986780667	0.781826485	5.462450168	-2.804628
Ssh2	Ssh2-005	1980.3014	3.29158E-05	0.000357327	0.14327166	-6.979747413	3.291032122	22.97057294	-2.803175
Sbf2	Sbf2-005	517.85665	0.000624085	0.003170883	0.143286268	-6.979035826	0.749706602	5.232229235	-2.803028
Rap1a	Rap1a-002	4761.6752	1.40141E-05	0.000196115	0.143286798	-6.979010047	8.035238592	56.07801086	-2.803022
Gdpd1	Gdpd1-001	2101	7.47273E-05	0.000647788	0.143299273	-6.978402475	3.554250656	24.80299157	-2.802897
Rasl2-9	Rasl2-9-001	676	0.003600336	0.01237322	0.143310929	-6.9778349	1.454978124	10.15259713	-2.802779
Elmod2	Elmod2-002	1265.3312	1.58461E-06	4.63554E-05	0.143432173	-6.971936501	2.070973052	14.43869262	-2.801559
Gm12251	Gm12251-001	242	0.000538953	0.002832799	0.143477948	-6.969712166	0.380341767	2.650872639	-2.801099
Arih1	Arih1-001	22825.82	7.97993E-09	2.31621E-06	0.143503144	-6.968488441	39.57156226	275.7539742	-2.800846
Sfswap	Sfswap-005	1249.7569	0.000150165	0.00108095	0.143552202	-6.966107021	1.675095833	11.66889684	-2.800353
Rpgrip1l	Rpgrip1l-004	188.6548	0.014561659	0.037031246	0.14359887	-6.963843093	0.290439881	2.022577756	-2.799884
Slc35d1	Slc35d1-002	1752.2107	0.003566859	0.012285288	0.143603532	-6.963617014	2.797974454	19.48402251	-2.799837
Zfp687	Zfp687-003	1065.2468	0.00438973	0.014448227	0.143658346	-6.960960003	1.544330428	10.75002234	-2.799286
RP24-95O4.	P24-95O4.4-0	96	0.025119789	0.056691669	0.143728669	-6.957554147	0.13655384	0.950080734	-2.79858
Ccdc78	Ccdc78-201	258	0.000996648	0.004541672	0.143874314	-6.950510992	0.448603055	3.118020468	-2.797119
Patz1	Patz1-004	586.61921	0.0107244	0.029114673	0.143883829	-6.950051363	0.962667376	6.690587707	-2.797024
Tia1	Tia1-019	234.04714	0.0370319	0.07731439	0.14389924	-6.94930702	0.269997969	1.876298782	-2.796869
Setd2	Setd2-007	1053.3592	0.009691324	0.026881103	0.143913254	-6.948630337	1.152634231	8.009229188	-2.796729
N4bp2l2	N4bp2l2-005	503.81552	0.002897022	0.01047319	0.143959118	-6.94641653	0.755573968	5.248531498	-2.796269
P24-258B22	24-258B22.1-	4607	4.10643E-07	2.06831E-05	0.14399402	-6.944732828	7.116881364	49.42483964	-2.795919
Gm12989	Gm12989-001	709	0.000351331	0.002037779	0.144021059	-6.943429036	1.321358383	9.174758166	-2.795648
Hivep2	Hivep2-005	968.05306	0.011121129	0.029940156	0.1440212	-6.943422218	1.461831263	10.15011167	-2.795647
Dcaf8	Dcaf8-009	614.0789	0.012558194	0.032939053	0.144087517	-6.940226481	1.151706272	7.993102369	-2.794983
Ehbp1	Ehbp1-002	671.59358	0.026487039	0.059140792	0.144122546	-6.938539655	1.185465067	8.225396376	-2.794632
Mef2c	Mef2c-011	394.53974	0.003846067	0.013011437	0.144154445	-6.937004248	0.672187889	4.662970239	-2.794313
Spc24	Spc24-201	2531	3.5242E-07	1.89191E-05	0.14416716	-6.936392437	3.762045765	26.09502579	-2.794186
Gm37648	Gm37648-001	368	0.002440995	0.00915006	0.144205201	-6.934562643	0.77999271	5.408908308	-2.793805

Cspg5	Cspg5-003	641.20141	2.00637E-05	0.000253025	0.144248139	-6.932498453	0.908302143	6.2968032	-2.793375
Tug1	Tug1-002	2647.1388	9.65827E-07	3.43066E-05	0.14431473	-6.929299572	4.234254439	29.34041747	-2.79271
Repin1	Repin1-001	768.80727	0.004807706	0.015507999	0.144381943	-6.926073832	1.168619329	8.093943754	-2.792038
Rpl3	Rpl3-007	2272.923	2.80999E-05	0.000320486	0.144396941	-6.925354487	4.683176866	32.43265992	-2.791888
Plcd4	Plcd4-003	256.48774	0.008484712	0.024196619	0.1444214	-6.924181575	0.331177017	2.293129801	-2.791644
Tbp	Tbp-001	5462.5775	6.41251E-07	2.66863E-05	0.14448229	-6.921263478	7.595694823	52.57180516	-2.791035
Gm27826	Gm27826-201	657	5.53177E-05	0.000518509	0.144513114	-6.919787224	0.992179037	6.865667826	-2.790728
Gm16982	Gm16982-001	667.72461	0.003536905	0.012200524	0.14451726	-6.919588713	1.139677998	7.886103009	-2.790686
Rnf185	Rnf185-002	2447.9722	0.017127584	0.042012444	0.144576026	-6.916776115	3.932571367	27.2007157	-2.7901
Gm28578	Gm28578-002	170	0.002358425	0.008905848	0.144651269	-6.913178218	0.248136517	1.715411966	-2.789349
Ccdc174	Ccdc174-002	1109.7074	0.0007975	0.00382019	0.144701328	-6.910786587	2.215927996	15.31380547	-2.78885
Atp11c	Atp11c-201	3984.5595	0.000169686	0.00118077	0.144710956	-6.910326805	5.23363199	36.16610743	-2.788754
Gm11934	Gm11934-001	633	0.000109793	0.000855224	0.144740488	-6.908916851	0.950071044	6.563961845	-2.78846
Whsc1	Whsc1-201	1371.6326	0.01056748	0.028789071	0.144755572	-6.908196952	2.508462838	17.32895533	-2.788309
Rrp7a	Rrp7a-003	262.10676	0.00517756	0.016450041	0.144783171	-6.90688006	0.30621551	2.114993802	-2.788034
Golim4	Golim4-008	768.24405	0.000573353	0.002974453	0.144809106	-6.905643074	0.948012121	6.546633338	-2.787776
Lcor	Lcor-202	2268.8009	2.92829E-06	6.94075E-05	0.144829646	-6.904663688	3.632986265	25.08454834	-2.787571
Cadm1	Cadm1-009	216.13679	0.011111063	0.029920113	0.144829889	-6.904652125	0.294248816	2.031685716	-2.787569
Palld	Palld-004	1537.3913	0.037206567	0.077603369	0.144849626	-6.903711316	1.533399055	10.58614441	-2.787372
Gm25535	Gm25535-201	305	0.000668234	0.003341172	0.144883683	-6.902088495	0.452379011	3.12235997	-2.787033
Nipa2	Nipa2-010	130.43425	0.01774617	0.043209654	0.144903695	-6.901135267	0.262176182	1.809313296	-2.786834
Rbm39	Rbm39-017	903.38611	0.000132484	0.000986642	0.144978844	-6.89755809	1.466450969	10.11493074	-2.786086
Gm26981	Gm26981-001	1744	3.16248E-06	7.31363E-05	0.144990619	-6.896997913	3.117390088	21.50063293	-2.785969
Smc4	Smc4-004	4555.8936	3.52491E-06	7.79701E-05	0.145003148	-6.896401983	6.985278114	48.17328584	-2.785844
Atf7	Atf7-004	939.49594	2.53176E-05	0.00029788	0.145119295	-6.890882446	1.327005081	9.144236019	-2.784689
Nelfb	Nelfb-002	1835.6425	0.000936724	0.00433036	0.145142719	-6.889770316	2.665348353	18.36363797	-2.784456
Mthfr	Mthfr-005	789.22538	0.008964463	0.025288926	0.14525855	-6.884276355	0.759585801	5.229198573	-2.783305
Ankib1	Ankib1-004	213	0.002055438	0.007997132	0.145303181	-6.882161786	0.296651765	2.041605439	-2.782862
Zmym4	Zmym4-002	5014.8219	0.000260721	0.001624464	0.145340582	-6.880390754	7.862089421	54.09424736	-2.782491
Fam171a1	Fam171a1-004	2272.3391	1.25757E-05	0.000181584	0.14535181	-6.879859294	2.692677204	18.52524029	-2.782379
Negr1	Negr1-001	999.4093	0.017148063	0.04204762	0.145532229	-6.87133021	1.356388079	9.32019038	-2.780589
Hsph1	Hsph1-001	2149.8996	0.021256577	0.049811012	0.145626271	-6.866892878	4.681630156	32.14825278	-2.779657
Nap1l1	Nap1l1-202	16694.221	1.85826E-07	1.36286E-05	0.145651418	-6.865707254	32.35279394	222.124812	-2.779408
Dbf4	Dbf4-004	1771.9894	4.04927E-07	2.05608E-05	0.145658865	-6.86535627	2.748982934	18.87274722	-2.779335
Mcm8	Mcm8-002	333.85601	0.000627314	0.003183981	0.145658935	-6.865352959	0.593174541	4.072352593	-2.779334
RP23-3L20.5	RP23-3L20.5-001	91	0.010561494	0.028777341	0.145660781	-6.86526595	0.131158886	0.900440632	-2.779316
Rbpms	Rbpms-201	3880.1364	0.007296965	0.0215208	0.145724261	-6.862275337	6.594537559	45.25353245	-2.778687
Arhgef15	Arhgef15-002	6064.9655	1.79144E-06	4.98835E-05	0.145781062	-6.859601536	7.857392562	53.89858209	-2.778125
Phip	Phip-011	2165.9124	6.60301E-06	0.000116702	0.145786023	-6.85936815	3.315923813	22.74514219	-2.778076
Tbxas1	Tbxas1-002	1040.4244	6.83033E-05	0.000607002	0.14581054	-6.85821476	1.879451521	12.88968216	-2.777833
Ttc32	Ttc32-201	526	0.007377563	0.02171923	0.145811323	-6.858177945	0.834387521	5.722378095	-2.777825
Gm26822	Gm26822-201	324	0.000634736	0.003213554	0.145925125	-6.852829486	0.495633368	3.396490962	-2.7767
Kcnq3	Kcnq3-001	1544	1.90931E-06	5.21067E-05	0.145928173	-6.852686377	2.255414398	15.45564752	-2.77667
4930428H23Rik	4930428H23Rik-	883.67481	0.000167698	0.001171459	0.145981135	-6.85020018	1.407261172	9.640020735	-2.776146
Exosc10	Exosc10-004	327.28696	0.000926171	0.004292469	0.145988732	-6.849843735	0.384838138	2.636081107	-2.776071
4930550C14Rik	4930550C14Rik-	292	0.004718288	0.015278381	0.146012121	-6.848746487	0.543959496	3.725440689	-2.77584
Agps	Agps-001	9484.2147	1.30383E-06	4.12936E-05	0.146018999	-6.848423884	17.01410459	116.5198002	-2.775772
Gm4575	Gm4575-001	234	0.002783984	0.010131914	0.146056392	-6.846670564	0.466730099	3.195547228	-2.775403
RP23-342N8	RP23-342N8.1-0	4063	2.74113E-08	4.34647E-06	0.146059948	-6.846503864	6.517030304	44.61887316	-2.775367
Lims1	Lims1-001	16774.996	5.91445E-07	2.54485E-05	0.146073107	-6.845887103	26.44809692	181.0606856	-2.775238
Pde4c	Pde4c-001	164.96476	0.002486057	0.009280244	0.146168584	-6.841415387	0.282477497	1.932545894	-2.774295
Gm37604	Gm37604-001	182	0.017055657	0.041875001	0.146171842	-6.841262921	0.344235425	2.355005051	-2.774263
Sec23a	Sec23a-001	5725.9704	1.20752E-06	3.94632E-05	0.146199781	-6.839955549	8.304274814	56.8008706	-2.773987
Mrpl21	Mrpl21-006	1738.5529	0.002189558	0.008402452	0.146203685	-6.839772886	2.74850983	18.79918301	-2.773948
2410001H17Rik	2410001H17Rik-	303.71946	0.008970905	0.025305014	0.146350686	-6.83290274	0.560068837	3.826895894	-2.772499
Psma5	Psma5-001	4648.8011	7.98092E-06	0.000132886	0.14636894	-6.832050579	8.734689806	59.67584255	-2.772319
Ept1	Ept1-004	865.02203	5.46409E-06	0.000103338	0.146391591	-6.830993461	1.594084476	10.88918063	-2.772095
Gm37255	Gm37255-001	220	0.012772007	0.033372702	0.146447802	-6.828371499	0.299821023	2.047289328	-2.771542
4930426P16Rik	4930426P16Rik-	1787.551	0.00047004	0.002550922	0.146467644	-6.827446481	2.31489656	15.80483237	-2.771346
Rnasek	Rnasek-001	4052.6943	8.63711E-06	0.000140067	0.146569174	-6.822717055	6.452827926	44.02581914	-2.770346
Smarca2	Smarca2-009	955.79559	0.035565752	0.07479596	0.146672018	-6.817933047	1.432118723	9.764089572	-2.769334
Cenpl	Cenpl-010	627.56851	0.000384815	0.002185724	0.146691405	-6.817031969	1.049300699	7.153116412	-2.769144
Gm38200	Gm38200-001	1205	0.00024688	0.001561353	0.146698449	-6.816704653	1.814611	12.36966725	-2.769074
4930059G10Rik	4930059G10Rik-	561.25387	0.000210899	0.001386691	0.146700853	-6.816592959	0.765383626	5.217308634	-2.769051
4930058D17Rik	4930058D17Rik-	635	5.42468E-05	0.000511692	0.146722456	-6.815589308	1.117316984	7.615173693	-2.768838
4931428L18Rik	4931428L18Rik-	181	0.001159398	0.005116531	0.146734914	-6.815010638	0.283986707	1.935372431	-2.768716
Rpap2	Rpap2-007	736.68	0.000832889	0.003951031	0.146787506	-6.812568906	1.402237436	9.552839154	-2.768199
Cdc42bpa	Cdc42bpa-006	995.14248	0.000120523	0.000918335	0.146842476	-6.810018664	1.707085672	11.62528529	-2.767659
Rpl5	Rpl5-006	2122.4179	1.17932E-06	3.88382E-05	0.146844496	-6.80992497	4.190814943	28.53913533	-2.767639
Dpf2	Dpf2-007	569.57487	0.000274372	0.001686206	0.146868343	-6.808819257	1.042218414	7.096276805	-2.767405
Sp110	Sp110-003	113.21309	0.014869999	0.037650438	0.146887956	-6.807910119	0.209437838	1.42583398	-2.767212
Mterf3	Mterf3-006	1370.952	1.42181E-05	0.000197714	0.14689289	-6.807681448	2.164669069	14.73637747	-2.767164
Nup85	Nup85-004	909.10025	0.039231925	0.080838252	0.146944786	-6.805277199	1.688971607	11.49391997	-2.766654
Zbtb20	Zbtb20-008	703.17558	0.000461651	0.002514429	0.146953653	-6.80486657	0.896147409	6.098163542	-2.766567
Cyfip1	Cyfip1-004	2273.9472	2.79146E-05	0.000319408	0.146967299	-6.804234742	3.129383017	21.29305665	-2.766433
Gm38309	Gm38309-001	235	0.0102966	0.028226234	0.146996116	-6.802900845	0.354877588	2.414197045	-2.76615
Ccndbp1	Ccndbp1-002	2253.6175	0.000327378	0.001931561	0.146999376	-6.80274995	3.833497203	26.07832291	-2.766118
Ift20	Ift20-001	3235.3204	1.22345E-06	3.97941E-05	0.147006489	-6.80242083	5.075880047	34.52827216	-2.766048
Sgms1	Sgms1-002	4384.1332	4.73388E-06	9.43745E-05	0.147059156	-6.799984632	5.064001833	34.43513465	-2.765531
Pex1	Pex1-011	292	0.005152497	0.016388655	0.147065177	-6.799706248	0.417877866	2.841447555	-2.765472
Arl1	Arl1-001	8119.3637	1.16691E-07	1.04351E-05	0.14708029	-6.79900754	13.26245187	90.17151025	-2.765324
Gm1966	Gm1966-001	2517	0.009062249	0.025516443	0.147114844	-6.79741058	7.254072223	49.30890728	-2.764985
Efr3a	Efr3a-004	495.73103	0.003464046	0.0120036	0.14713729	-6.796373658	0.868392152	5.901917544	-2.764765
Auts2	Auts2-016	657.68955	0.000544371	0.002857428	0.147149685	-6.795801183	1.433066983	9.738838296	-2.764644
Kcnt2	Kcnt2-002	5532.4004	0.005497528	0.017252185	0.147156632	-6.795480336	9.997211582	67.93585472	-2.764576
Glmp	Glmp-005	3089.6612	6.58516E-05	0.000590115	0.14716178	-6.795242618	4.529366234	30.77814247	-2.764525
Srgn	Srgn-201	20496.913	7.02538E-06	0.000121771	0.14716919	-6.7949005	23.47219804	159.4912502	-2.764452
Braf	Braf-001	16607.673	1.9996E-08	3.56435E-06	0.147174449	-6.79465768	27.20088368	184.8206932	-2.764401
Rps15a-ps4	Rps15a-ps4-00	387	0.000674399	0.003364136	0.147180889	-6.794360351	0.6673307	4.534085252	-2.764338
Gm5148	Gm5148-001	148.39888	0.017660342	0.043060205	0.147183154	-6.794255823	0.235905131	1.602799808	-2.764316
Gm28940	Gm28940-001	188	0.013478774	0.034836101	0.147196546	-6.793637673	0.304609279	2.069405075	-2.764184
Gm5148	Gm5148-002	91.819606	0.032542237	0.069730456	0.147211906	-6.792928839	0.146006132	0.991809265	-2.764034
RP23-134H19	RP23-134H19.3-	675.53482	0.030125103	0.065564262	0.147234575	-6.791882947	1.250950388	8.49630861	-2.763812
Guf1	Guf1-008	843.9936	0.040529933	0.082983633	0.147357741	-6.786206103	1.248179208	8.47040136	-2.762605
Aak1	Aak1-002	2864.8499	2.80303E-05	0.000320179	0.14738641	-6.784893838	3.92369254	26.62183734	-2.762326
Zmym3	Zmym3-018	326.37687	0.038307531	0.079411267	0.147390262	-6.784708765	0.628434033	4.263741892	-2.762287
Gapvd1	Gapvd1-013	1192.4646	0.001926696	0.007610348	0.147412241	-6.783697175	1.87124702	12.69397313	-2.762072
Cdh13	Cdh13-006	3854.0035	0.000107574	0.000840452	0.147423573	-6.783175737	5.777606358	39.19051926	-2.761961
Pgk1	Pgk1-001	3987.1793	0.001898635	0.007513917	0.147452854	-6.781828727	8.511908297	57.72630421	-2.761674
Cnksr3	Cnksr3-002	219.47565	0.007262856	0.021449757	0.147456806	-6.781646977	0.265888804	1.803164006	-2.761636
Clta	Clta-004	4568.7561	0.002497565	0.009309129	0.14748342	-6.780423198	7.965002484	54.00608761	-2.761375
Fmr1	Fmr1-006	1969.2857	0.012247652	0.03230264	0.147657596	-6.772425049	3.056424959	20.69940895	-2.759673
Cyb5d2	Cyb5d2-002	1103.9276	0.00064954	0.0032711	0.147681964	-6.77130756	1.702070854	11.52524524	-2.759434
Rnf167	Rnf167-002	1115.7495	0.003825541	0.012960735	0.147783612	-6.766650152	1.301434942	8.806354948	-2.758442
Slc22a18	Slc22a18-001	107.62611	0.010687733	0.029045056	0.147793782	-6.76618451	0.087144691	0.589637057	-2.758343
Crl1	Crl1-004	5371.3884	9.17884E-07	3.34087E-05	0.147821539	-6.76491399	8.433169812	57.04966844	-2.758072
Napb	Napb-003	696.17222	0.002128397	0.008212888	0.147855403	-6.763364621	1.006395023	6.806616491	-2.757741

S100a16	S100a16-002	4355.4947	0.00029196	0.001771214	0.147862234	-6.763052161	8.021519671	54.24995594	-2.757674
Copa	Copa-006	12977.068	8.43582E-05	0.000705905	0.147869936	-6.762699894	20.64804914	139.6365597	-2.757599
Napepld	Napepld-002	1922.7624	0.000438526	0.002417044	0.147916748	-6.760559674	3.734457549	25.24702311	-2.757143
Asap1	Asap1-002	136.07884	0.014725435	0.037367194	0.147933201	-6.759807747	0.308466147	2.085171849	-2.756982
Slk	Slk-003	1346.6356	0.000197343	0.001319342	0.147945586	-6.759241855	1.877283835	12.68901547	-2.756861
Creld1	Creld1-005	1501.7455	0.001872635	0.00743023	0.147988377	-6.757287439	1.748917708	11.81793966	-2.756444
Metap2	Metap2-006	3466.9506	1.34007E-06	4.19154E-05	0.147997798	-6.756857277	6.286941278	42.47996493	-2.756352
Gm37943	Gm37943-001	1006	4.23475E-06	8.76375E-05	0.148006473	-6.75646124	1.480107143	10.00028655	-2.756268
Rbm25	Rbm25-006	857.65588	0.000295242	0.001786577	0.148015824	-6.756034413	1.449910124	9.795642696	-2.756177
Slc6a8	Slc6a8-004	3142.9612	0.000478099	0.002583195	0.148073911	-6.753384147	4.874737636	32.92097587	-2.755611
Micu1	Micu1-002	1500.5598	0.000115723	0.000890676	0.148096918	-6.752334982	2.476852802	16.72453982	-2.755386
Tap1	Tap1-004	1218.4199	0.038654328	0.079955855	0.148138875	-6.750422544	1.889055486	12.75192274	-2.754978
Srsf10	Srsf10-002	3175.7515	1.43369E-05	0.00019874	0.148162472	-6.74934741	5.952185211	40.17336584	-2.754748
G2e3	G2e3-001	1684.6799	2.1142E-05	0.000263109	0.148187805	-6.748193639	3.029230536	20.44183423	-2.754501
Fam118b	Fam118b-001	211.73057	0.045008696	0.090172117	0.148190342	-6.748078104	0.202187635	1.364377954	-2.754477
Gm38190	Gm38190-001	4364	8.63365E-08	8.83565E-06	0.148246448	-6.7455242	6.805396096	45.90596405	-2.753931
Rab7	Rab7-004	864.85452	0.018143209	0.043955925	0.148267839	-6.744551005	1.122304555	7.569440316	-2.753722
Dnm3os	Dnm3os-001	243.80578	0.002569217	0.00951819	0.148314607	-6.742424213	0.46668316	3.146575836	-2.753267
Dnm3os	Dnm3os-201	246.83359	0.002511813	0.009350028	0.148317333	-6.742300299	0.472486167	3.185643623	-2.753241
Gm26530	Gm26530-201	622.94617	0.000305748	0.001834742	0.148348372	-6.740889622	1.044067313	7.037942512	-2.752939
Gm37298	Gm37298-001	421	0.002783495	0.01013121	0.148363207	-6.740215596	0.790512785	5.328226601	-2.752795
Med13l	Med13l-002	991.7751	7.46032E-05	0.000647589	0.148413692	-6.737922824	1.726042631	11.62994204	-2.752304
Sema4d	Sema4d-003	2440.8741	0.002548678	0.009461547	0.148425586	-6.73738286	4.726644568	31.8452141	-2.752188
Celf2	Celf2-004	2809.3183	9.14562E-06	0.000145624	0.148461362	-6.735759317	5.593995377	37.67980648	-2.751841
Adcy3	Adcy3-201	436.57222	0.04171002	0.084932711	0.148473506	-6.73520837	0.664278256	4.474052471	-2.751723
Birc6	Birc6-009	1140.1173	4.04375E-06	8.48642E-05	0.148491485	-6.734392887	1.643954117	11.07103291	-2.751548
Armcx1	Armcx1-004	881.99101	0.018016919	0.043717993	0.14849951	-6.734028966	0.891005978	6.000060065	-2.75147
Ccbl1	Ccbl1-004	426.67923	0.022591523	0.052224363	0.148516334	-6.733266117	0.689862655	4.64502884	-2.751306
Bmpr1a	Bmpr1a-001	24292.574	3.76498E-08	5.22068E-06	0.148527225	-6.732772413	34.85145419	234.6469093	-2.751201
Arhgap29	Arhgap29-004	509.94329	0.005467921	0.017192352	0.148557835	-6.731385128	0.829621196	5.584499782	-2.750903
Tfb2m	Tfb2m-001	1725.3099	8.83563E-06	0.000142343	0.148636072	-6.727841934	3.157973447	21.24634618	-2.750144
Zfp773	Zfp773-201	146	0.005013118	0.016033178	0.148801414	-6.720366233	0.244427593	1.642642942	-2.74854
10057O12R	10057O12Rik-	731.2508	0.005412597	0.017053369	0.148804112	-6.720244395	1.142537564	7.67813166	-2.748514
Gm9964	Gm9964-201	1132	1.17221E-06	3.87268E-05	0.148847637	-6.718279302	1.680101047	11.28738809	-2.748092
Ablim1	Ablim1-005	890.55251	0.003997197	0.013410129	0.148848179	-6.71825484	1.152619581	7.74359208	-2.748087
Zfand5	Zfand5-003	2030.4325	6.78403E-06	0.000119106	0.148869626	-6.717286989	3.447364657	23.15693776	-2.747879
Gm37940	Gm37940-001	1944	0.00017138	0.001189655	0.148889243	-6.716401947	3.040982165	20.42445853	-2.747689
Ints8	Ints8-002	963.56437	0.004844125	0.015602674	0.148927472	-6.714677867	1.38809014	9.32057814	-2.747318
Clock	Clock-001	9587.6275	3.04654E-05	0.000338449	0.148937435	-6.714228684	13.68373391	91.87571872	-2.747222
Nfu1	Nfu1-201	192.45041	0.009153929	0.025728055	0.149033377	-6.709906311	0.353429992	2.371482135	-2.746293
Phka1	Phka1-201	583.59905	0.003157269	0.011186147	0.149097653	-6.70701367	0.937541562	6.288104071	-2.745671
700095J07R	700095J07Rik-	363	0.000104718	0.000824125	0.149148728	-6.704716905	0.56585052	3.793867546	-2.745176
Zranb2	Zranb2-003	1241.8778	0.000384428	0.002184098	0.149162579	-6.704094332	1.550233374	10.39291078	-2.745042
000005J15R	000005J15Rik-	1293.1955	3.42919E-05	0.000366687	0.149177175	-6.703438394	2.212288399	14.82993899	-2.744901
Clint1	Clint1-003	2087.2527	2.1205E-06	5.65631E-05	0.149196459	-6.702571941	3.889043937	26.06659677	-2.744715
30508B09R	0508B09Rik-	332.89766	0.004464451	0.014644905	0.149198309	-6.702488823	0.640998038	4.296282188	-2.744697
Btbd16	Btbd16-201	1942	8.03214E-07	3.07178E-05	0.149198508	-6.702479874	2.766579281	18.54294195	-2.744695
P24-230N16	24-230N16.2-	1214	6.85619E-06	0.00011987	0.149273009	-6.699134722	1.741157558	11.66424906	-2.743975
Rabgap1	Rabgap1-001	4036.9988	3.30984E-05	0.000358193	0.149331867	-6.696494309	5.883104187	39.39617371	-2.743406
Gm20631	Gm20631-002	513.10562	0.000141919	0.001038837	0.149370923	-6.694743381	0.702739098	4.704657926	-2.743029
Atp8a1	Atp8a1-013	204.06354	0.00422265	0.014015482	0.149384851	-6.694119204	0.411096605	2.751929677	-2.742894
30136K24R	0136K24Rik-	1224	6.04623E-07	2.58209E-05	0.149386615	-6.694040151	2.152327195	14.40776466	-2.742877
Gria3	Gria3-001	7582.6565	0.00333497	0.011668477	0.149398704	-6.6934985	7.836027594	52.45043895	-2.74276
Arrdc1	Arrdc1-003	597.33189	0.021116015	0.04956981	0.149416253	-6.692712317	0.837552172	5.605495734	-2.742591
Pcmt1	Pcmt1-006	2333.4143	0.002401588	0.009035967	0.14943341	-6.691943929	3.430944343	22.95968716	-2.742425
Cdnf	Cdnf-001	571.16916	0.000251234	0.001581449	0.149472302	-6.690202713	0.925954325	6.194822134	-2.74205
Brca2	Brca2-006	206.95241	0.006847368	0.020453721	0.149538414	-6.687244904	0.419112544	2.802708225	-2.741412
30151L19R	0151L19Rik-	1450.1856	4.07898E-06	8.53944E-05	0.149549538	-6.686747508	2.717883583	18.17380128	-2.741305
Arhgef40	Arhgef40-008	149.21192	0.019520386	0.046613206	0.149678965	-6.680965476	0.254961202	1.703386991	-2.740057
Immp1l	Immp1l-001	1871	1.33617E-05	0.000189498	0.149713172	-6.67943899	3.040014651	20.30559239	-2.739727
Sqle	Sqle-002	871.14701	1.06941E-05	0.000162211	0.149722792	-6.679009855	1.560895454	10.42523612	-2.739634
Gm5611	Gm5611-201	1845	2.0145E-06	5.42852E-05	0.149751463	-6.677731074	3.950182405	26.37825579	-2.739358
Zfp664	Zfp664-005	17299.871	9.69606E-08	9.35465E-06	0.149805071	-6.675341469	27.77695157	185.4206367	-2.738842
Kdm5c	Kdm5c-003	944.92348	0.000776467	0.003740366	0.149835092	-6.674003963	1.443939902	9.636860631	-2.738553
Skp2	Skp2-002	566.27427	0.025565736	0.057485545	0.149840844	-6.673747796	1.027859254	6.859673435	-2.738497
Stag2	Stag2-003	1202.1661	0.000157626	0.001120464	0.149860133	-6.672888762	2.324925666	15.51397035	-2.738311
Meg3	Meg3-001	7668.9633	1.50948E-06	4.49562E-05	0.149877483	-6.672116299	8.24337657	55.00076717	-2.738144
Snx14	Snx14-004	550.59341	0.000162452	0.001144855	0.149884494	-6.671804224	1.208993042	8.066164886	-2.738077
Bcl6b	Bcl6b-001	10259.217	0.006141336	0.01880577	0.149894164	-6.671373822	17.08518058	113.9816264	-2.737984
S100a4	S100a4-001	8077.2046	0.000309114	0.001850576	0.149897614	-6.671220279	12.18950607	81.31888009	-2.737951
Gtf2i	Gtf2i-009	351.7279	0.043481773	0.087788889	0.149921067	-6.670176661	0.347248609	2.316209568	-2.737725
Gm6277	Gm6277-201	311.55983	0.030208245	0.065707632	0.149928289	-6.669855361	0.498128897	3.322447695	-2.737655
Lmna	Lmna-006	4023.1064	2.67217E-06	6.54662E-05	0.149931133	-6.669728834	4.947325232	32.99731775	-2.737628
Ube2l3	Ube2l3-002	694.05403	0.000722242	0.003544969	0.150001804	-6.666586497	1.141893943	7.612534742	-2.736948
Tnrc6c	Tnrc6c-004	11751.828	2.23222E-07	1.50269E-05	0.150024927	-6.66555898	15.07711451	100.497396	-2.736726
Rara	Rara-001	1059.3748	0.003129884	0.011102904	0.150035902	-6.665071407	1.911977268	12.74346502	-2.73662
Asb18	Asb18-001	878	6.81412E-05	0.000606052	0.15010187	-6.66214218	1.335154417	8.89498856	-2.735986
Celf1	Celf1-002	3305.8898	0.001063022	0.004772381	0.150209403	-6.657372831	5.418921031	36.07577764	-2.734953
Zkscan17	Zkscan17-002	2426.442	3.02109E-05	0.000336714	0.150282484	-6.654135406	2.825758086	18.80297693	-2.734251
Ulbp1	Ulbp1-202	733.01096	0.003514425	0.012140097	0.150286878	-6.653940891	1.670472725	11.11522677	-2.734209
P23-110C17	23-110C17.2-	16951.674	0.001825304	0.007286214	0.150313816	-6.652748428	35.84174707	238.4461265	-2.73395
Rabep1	Rabep1-007	1918.5737	0.002369336	0.008940153	0.150375393	-6.650024167	2.194805896	14.59551225	-2.73336
Morn4	Morn4-201	431	0.001311278	0.005635463	0.15045051	-6.646703938	0.605476666	4.02442414	-2.732639
Mocs2	Mocs2-012	387.64147	0.010729121	0.029119625	0.150467585	-6.6459497	0.594123293	3.948513523	-2.732475
N4bp2l1	N4bp2l1-002	217.26324	0.021528796	0.050338699	0.150571991	-6.641341397	0.463684469	3.079486862	-2.731475
Gm38299	Gm38299-001	836	0.000155349	0.00110894	0.150585409	-6.640749632	1.320481852	8.768989377	-2.731346
Sppl2a	Sppl2a-003	1063.6156	0.000271763	0.001674981	0.15059504	-6.640324942	1.344580131	8.92844898	-2.731254
Hnrnpm	Hnrnpm-001	2167.0258	0.001842254	0.00733859	0.150601774	-6.640028039	3.748774974	24.89197094	-2.731189
Gm37914	Gm37914-001	1031	5.86227E-06	0.000108083	0.150678778	-6.636634661	1.707399001	11.33138339	-2.730452
Luzp1	Luzp1-201	10019.241	1.0215E-06	3.5458E-05	0.150739491	-6.633961643	13.58221347	90.10388316	-2.729871
Ubfd1	Ubfd1-001	5616.8901	1.78798E-07	1.32838E-05	0.150747777	-6.633596995	9.630985572	63.88807695	-2.729791
Kif18a	Kif18a-005	945.95389	1.28617E-05	0.000184085	0.150780164	-6.632172123	1.595584426	10.58219055	-2.729481
Smim7	Smim7-001	7161.3351	3.71113E-08	5.16692E-06	0.150795638	-6.631491554	11.82080449	78.38956511	-2.729333
Gm13827	Gm13827-001	137	0.014252802	0.036410715	0.15085102	-6.629056951	0.21424219	1.420223679	-2.728804
Ccdc132	Ccdc132-007	832.4031	0.017295556	0.042318389	0.150854383	-6.628909157	1.338705131	8.874154699	-2.728771
Apc	Apc-006	10992.12	3.30789E-06	7.503E-05	0.150889001	-6.627388316	16.21442392	107.4592836	-2.72844
Ythdc1	Ythdc1-005	3699.8754	6.19134E-06	0.000112205	0.150947756	-6.624808643	6.329779957	41.93358097	-2.727879
Tmem33	Tmem33-005	1104.6335	0.003358596	0.011729573	0.15095182	-6.624630276	1.704772983	11.29349072	-2.72784
Dnm3	Dnm3-001	517.29255	0.003323987	0.011638373	0.150953258	-6.624567184	0.908023331	6.015261564	-2.727826
Ssx2ip	Ssx2ip-001	964.92799	0.004071758	0.013602976	0.150975342	-6.623598177	1.806129381	11.96307527	-2.727615
Lin54	Lin54-004	608.81286	0.001151409	0.005091772	0.151031726	-6.621125431	0.963679508	6.380642897	-2.727076
Pik3ca	Pik3ca-002	3771.6679	0.001649414	0.00672689	0.151059757	-6.619896788	7.241903346	47.9406527	-2.726809
Zscan22	Zscan22-003	1629.1974	5.52823E-06	0.000104206	0.151091044	-6.618525998	2.731526805	18.07868117	-2.72651
Gcn1l1	Gcn1l1-006	847.06713	7.11634E-06	0.000122788	0.151138628	-6.616442241	1.253044142	8.290694191	-2.726056
Tcf12	Tcf12-001	6319.4443	2.2389E-05	0.00027328	0.151242347	-6.611904801	11.43181664	75.58608335	-2.725066
LP24-511F2	24-511F2.4-0	1639.5066	6.19173E-06	0.000112205	0.151349789	-6.607211044	2.745983309	18.14329125	-2.724041
Med23	Med23-001	419.84436	0.00979757	0.02712089	0.151358654	-6.60682407	0.615060554	4.063596872	-2.723957

Lats1	Lats1-202	12924.983	1.45794E-07	1.18609E-05	0.151414693	-6.604378863	20.36919365	134.525872	-2.723423
Stat3	Stat3-014	2363.806	6.65392E-07	2.73299E-05	0.151417465	-6.604257983	3.617629116	23.89175597	-2.723396
Trim34a	Trim34a-201	680.03602	0.005412926	0.017053369	0.151494462	-6.600901361	0.891894262	5.887306046	-2.722663
30432B10R	0432B10Rik-	381	0.000724844	0.003554175	0.15149876	-6.6007141	0.652305884	4.305684644	-2.722622
Sgk3	Sgk3-005	655.68829	0.000150929	0.001085079	0.151523265	-6.599646584	1.201241218	7.927767502	-2.722389
Trim34a	Trim34a-003	957.24909	0.001513072	0.006294562	0.1515721	-6.59752026	1.375465504	9.074661527	-2.721924
Celf1	Celf1-204	419.08346	0.015158917	0.038178756	0.151639567	-6.594584893	0.452920423	2.986822178	-2.721282
Gpcpd1	Gpcpd1-007	2834.4458	2.83452E-05	0.000322029	0.151654194	-6.593948876	5.520572229	36.40237104	-2.721143
Zfp738	Zfp738-002	185.9479	0.020847315	0.049053348	0.151656265	-6.593858821	0.330457512	2.178990182	-2.721123
C78339	C78339-001	16161.98	5.51306E-09	1.9073E-06	0.15165692	-6.593830339	24.87707425	164.035207	-2.721117
Rbmxl2	Rbmxl2-001	753	0.004532573	0.014818694	0.151673402	-6.593113788	0.683556937	4.506768667	-2.72096
Atf2	Atf2-006	465.67933	0.001466746	0.006148841	0.151730088	-6.590650622	0.440733757	2.904722213	-2.720421
Npm1	Npm1-002	7981.8084	0.001145533	0.005072336	0.151775916	-6.588660635	19.9728787	131.5945197	-2.719985
Paxip1	Paxip1-005	1828.5608	4.3223E-06	8.87018E-05	0.151779581	-6.588501533	2.96252435	19.51859622	-2.71995
Itch	Itch-001	10240.581	4.76425E-05	0.000464887	0.15187337	-6.584432814	16.90960228	111.3401402	-2.719059
Tmem194b	'mem194b-00	1427.7811	0.001042997	0.004702206	0.151911185	-6.582793772	2.016930557	13.27703791	-2.7187
Gm15558	3m15558-001	233	0.010789202	0.029230357	0.152027336	-6.577764405	0.326445247	2.147279924	-2.717597
Whsc1l1	Whsc1l1-002	8035.9589	5.04946E-06	9.84098E-05	0.152034337	-6.577461533	14.79351644	97.30378535	-2.717531
Irgm2	Irgm2-001	11125.968	0.001490672	0.006226392	0.152037889	-6.577307828	16.33376112	107.4321749	-2.717497
Gm25072	3m25072-201	650	9.18419E-05	0.000750363	0.152066798	-6.576057462	0.974065675	6.405511853	-2.717223
Cenpp	Cenpp-201	287	0.007479466	0.021962596	0.152080517	-6.575464223	0.568952746	3.741128429	-2.717093
Phf3	Phf3-013	354.71663	0.002727406	0.009981157	0.152106266	-6.574351131	0.607060677	3.991030049	-2.716849
Nfib	Nfib-006	19265.149	2.81844E-05	0.00032113	0.152144018	-6.572719785	25.38738082	166.8641402	-2.71649
Cyp51	Cyp51-001	1219.5624	0.000448989	0.002460854	0.152267737	-6.567379398	2.170653248	14.25550342	-2.715318
Ptpn18	Ptpn18-007	1079.4834	4.97128E-05	0.000478891	0.152313334	-6.565413374	1.7840217	11.71283993	-2.714886
Adamts10	Adamts10-011	1416.5383	0.004183701	0.013913173	0.152329746	-6.564706028	1.54276657	10.127809	-2.71473
Edem2	Edem2-003	319.71148	0.002265934	0.008636573	0.1523318	-6.564617495	0.574211128	3.76947642	-2.714711
Mau2	Mau2-202	5809.866	1.82252E-06	5.05688E-05	0.152370668	-6.562942941	8.944831691	58.70442	-2.714343
Jmjd1c	Jmjd1c-201	6568.1971	7.61052E-06	0.000128721	0.152379239	-6.562573796	12.65116902	83.02423028	-2.714262
Dhtkd1	Dhtkd1-001	243.56084	0.001385173	0.00587956	0.152381096	-6.562493798	0.476466987	3.126811644	-2.714244
Slc6a2	Slc6a2-201	114	0.010546027	0.028752226	0.15241792	-6.560908327	0.137700204	0.903438412	-2.713896
Rdh9	Rdh9-001	620.84075	0.000170432	0.001184516	0.152507141	-6.557070009	1.053567561	6.908316257	-2.713051
Prdx6	Prdx6-008	2762.1944	3.22031E-06	7.39401E-05	0.152518549	-6.556579536	5.303917326	34.7755558	-2.712943
Zfp940	Zfp940-003	2410.0803	2.01783E-06	5.43323E-05	0.152640594	-6.551337192	3.480595902	22.80255738	-2.711789
Suv420h1	Suv420h1-005	3301.2012	1.27036E-05	0.000182891	0.152654565	-6.550737599	5.478353673	35.88725739	-2.711657
Hsd17b7	Hsd17b7-001	546.60707	8.43884E-05	0.000705985	0.15269501	-6.549002505	1.121539063	7.344962135	-2.711275
Lrrcc1	Lrrcc1-006	655.72558	0.012705595	0.033235389	0.152733477	-6.547353084	1.190868342	7.79703551	-2.710912
Mob4	Mob4-001	4030.8115	8.47508E-06	0.000138252	0.152752865	-6.546522049	6.293517226	41.20064929	-2.710729
Nop58	Nop58-002	3364.1189	0.001675506	0.006805749	0.152790879	-6.54489329	7.707432531	50.44432346	-2.71037
Pirb	Pirb-001	2546.6286	4.65066E-05	0.000456797	0.152823878	-6.543480067	5.823887823	38.10849388	-2.710058
Rhbdd1	Rhbdd1-003	629	0.000361525	0.002081399	0.15287357	-6.541353076	0.958623281	6.270693345	-2.709589
Polr2h	Polr2h-001	1387.9488	0.000949898	0.004375202	0.152918919	-6.539413205	2.699834837	17.65533559	-2.709161
Lnp	Lnp-003	996.66622	0.011613921	0.031020572	0.152947917	-6.538173398	1.560870605	10.20524267	-2.708888
Gm16344	3m16344-001	10834	1.42865E-08	3.11956E-06	0.15298809	-6.536456512	17.75395328	116.0479435	-2.708509
Gm38287	3m38287-001	1860.831	0.005857849	0.018100985	0.153000389	-6.535931094	2.009880437	13.13644004	-2.708393
Cdc27	Cdc27-001	7411.2384	3.04758E-07	1.73677E-05	0.153014569	-6.535325406	11.75028307	76.79192347	-2.708259
930021J03R	0021J03Rik-(	7213.811	5.09368E-07	2.34487E-05	0.153058212	-6.533461925	11.23095434	73.37701254	-2.707848
Blmh	Blmh-006	1803.9235	4.37277E-06	8.92004E-05	0.153065032	-6.53317083	2.732237644	17.85017528	-2.707783
Vprbp	Vprbp-002	4769.0075	1.90553E-05	0.000243734	0.153066657	-6.533101467	8.680961749	56.71360394	-2.707768
Srsf2	Srsf2-001	21045.507	2.33685E-05	0.000281654	0.153173413	-6.528548129	42.21668368	275.6136512	-2.706762
Aff2	Aff2-003	490.06908	0.000587959	0.003028133	0.153186463	-6.527991964	0.64182954	4.189858083	-2.706639
Rbm39	Rbm39-006	2574.8338	4.9261E-06	9.68163E-05	0.153229782	-6.526146471	4.589278007	29.95030047	-2.706231
Dctn6	Dctn6-007	2367.8488	0.001999341	0.007832259	0.153235959	-6.525883399	4.158819208	27.13996923	-2.706173
Stmn1	Stmn1-001	4011.5677	0.009248698	0.025920967	0.153310351	-6.5227168	9.008794366	58.76181436	-2.705473
Ube2i	Ube2i-005	4731.3363	3.01163E-05	0.000335809	0.153345643	-6.521215618	8.95352961	58.38789713	-2.705141
Ssh2	Ssh2-007	1198.1291	2.7821E-05	0.000318888	0.153369378	-6.520206394	1.994889127	13.00708884	-2.704918
Lin9	Lin9-003	366.58093	0.001298081	0.005593501	0.153390785	-6.519296456	0.552510592	3.601980347	-2.704716
Exoc4	Exoc4-004	666.9209	0.043894569	0.088460837	0.153397746	-6.519000628	1.221911232	7.965640087	-2.704651
Cdc42bpa	Cdc42bpa-012	5558.7005	1.62677E-05	0.000217064	0.153475303	-6.515706302	9.007358704	58.68930387	-2.703922
Edrf1	Edrf1-001	1508.5641	8.12463E-05	0.000689125	0.153483271	-6.515368065	2.417559773	15.75129174	-2.703847
Mc1r	Mc1r-001	256	0.001263871	0.005476664	0.153584991	-6.511052897	0.459042417	2.98884946	-2.702891
Kif2a	Kif2a-001	2747.6885	5.97521E-06	0.000109264	0.15368834	-6.506674486	4.782447645	31.11783007	-2.70192
Akap10	Akap10-003	805.92692	8.54953E-05	0.000712114	0.153689278	-6.506634756	1.318209049	8.577104814	-2.701912
Gm37094	3m37094-001	3162	5.86344E-07	2.53244E-05	0.153700507	-6.506159414	4.86282003	31.63828232	-2.701806
Cdk9	Cdk9-005	1732.2266	0.000270801	0.001671001	0.153748355	-6.504134632	2.869681738	18.66479637	-2.701357
Slamf6	Slamf6-002	176.38052	0.006238525	0.019028361	0.153756655	-6.503783532	0.308924901	2.009180686	-2.701279
Rpe	Rpe-001	1121.8544	0.001106371	0.004930616	0.153802331	-6.501852036	1.944409062	12.64226002	-2.700851
Acbd5	Acbd5-201	3848.8771	3.19751E-06	7.37188E-05	0.153809792	-6.501536642	5.005337071	32.54238237	-2.70078
Tcf4	Tcf4-201	18946.005	2.96618E-07	1.71608E-05	0.15393225	-6.496364484	40.85153707	265.3864745	-2.699633
Mapk8	Mapk8-001	3339.3593	6.21028E-07	2.61447E-05	0.153962409	-6.495091939	4.798728659	31.16818383	-2.69935
Dcaf6	Dcaf6-005	338.55397	0.011065416	0.02983693	0.153968756	-6.494824204	0.543628007	3.53076834	-2.69929
Tnfaip8l3	Tnfaip8l3-201	1112	0.0175144	0.042762204	0.153983376	-6.494207523	2.284179863	14.83393805	-2.699153
Stard9	Stard9-006	824.08233	0.000468352	0.002543374	0.153988437	-6.493994099	1.349250775	8.762026569	-2.699106
Adam19	Adam19-001	2073.4283	0.009052045	0.025494	0.154024693	-6.49246546	5.332695293	34.62234	-2.698766
Mapkap1	Mapkap1-004	261.19946	0.032410802	0.069483598	0.154042632	-6.491709402	0.301983963	1.960392131	-2.698598
Nrbp1	Nrbp1-003	786.58365	0.02884414	0.063246594	0.154115782	-6.488628141	0.901581925	5.850029851	-2.697913
Anp32a	Anp32a-006	208.46133	0.019688659	0.046943831	0.154128816	-6.488079405	0.439534792	2.85173663	-2.697791
Cbx5	Cbx5-002	4548.6046	0.000236156	0.001510427	0.154138469	-6.487673118	7.042950458	45.69236036	-2.697701
Gcnt1	Gcnt1-002	1315.9946	0.00067835	0.003376959	0.15419178	-6.485430012	2.38660234	15.47814244	-2.697202
R3hdm1	R3hdm1-005	1524.8557	2.84436E-05	0.000322688	0.154203292	-6.484945859	2.554798077	16.56772721	-2.697095
Kdm1a	Kdm1a-004	596.08214	0.00047227	0.002559376	0.154227286	-6.483936955	0.915682607	5.937228296	-2.69687
Rasa2	Rasa2-003	474.73573	0.0035063	0.012121809	0.15422897	-6.483866143	0.987832964	6.404976708	-2.696854
Gm10481	3m10481-001	722	0.000103597	0.000819068	0.154257798	-6.482654457	1.411643053	9.151194132	-2.696585
Brd4	Brd4-005	3605.2145	1.13735E-06	3.8004E-05	0.154267556	-6.482244385	6.635248493	43.01130229	-2.696493
Mblac2	Mblac2-201	431	0.002073738	0.008054606	0.154300429	-6.480863369	0.840322141	5.446012983	-2.696186
G3bp2	G3bp2-003	2563.8354	0.028967188	0.06345982	0.154441521	-6.474942688	5.537343158	35.8539796	-2.694867
Traf3ip2	Traf3ip2-005	412.537	0.012308876	0.032422655	0.154589096	-6.468761556	0.455149072	2.944250821	-2.69349
Rac2	Rac2-201	1905	0.002942577	0.01059538	0.154610944	-6.467847448	4.732934871	30.61190073	-2.693286
Acp6	Acp6-002	533.56159	0.000525922	0.002776142	0.15464284	-6.466513404	0.838778014	5.42396927	-2.692988
P24-500I24	24-500I24.3-(	1965	2.37126E-07	1.55689E-05	0.154691769	-6.464468043	3.408885469	22.03663118	-2.692532
Cav2	Cav2-002	2906.9272	4.21365E-06	8.75182E-05	0.154709466	-6.463728612	3.843325047	24.84221007	-2.692367
Dctn5	Dctn5-002	2003.6586	1.43124E-05	0.000198622	0.154806286	-6.459686007	3.457326096	22.333241	-2.691464
Camsap2	Camsap2-201	7065.6485	2.06853E-06	5.5436E-05	0.154807374	-6.459640597	8.608594378	55.60842572	-2.691454
Znrf3	Znrf3-002	1505.6219	8.82201E-05	0.000727031	0.154813863	-6.459369848	3.037889022	19.62284875	-2.691393
Smcr8	Smcr8-001	845.5524	0.04286067	0.086816367	0.154821255	-6.459061441	1.563055788	10.09587337	-2.691325
Etfdh	Etfdh-002	1035.5642	0.015519048	0.038893392	0.154830509	-6.458675384	1.6686471	10.77724995	-2.691238
Tiam1	Tiam1-004	111.31127	0.012729835	0.033289826	0.154836165	-6.458439488	0.201858856	1.303693208	-2.691186
Mknk1	Mknk1-003	987.84922	0.00268244	0.009845002	0.154852879	-6.457742382	1.524196161	9.842866146	-2.69103
Rbm12	Rbm12-001	3828.3474	5.89009E-07	2.54075E-05	0.154857425	-6.457552797	6.271016961	40.49542311	-2.690988
Nup54	Nup54-008	186.42152	0.025374342	0.057156936	0.154903556	-6.455629717	0.359828811	2.322921567	-2.690558
Atg16l2	Atg16l2-011	434.58905	0.000565728	0.002943814	0.155002635	-6.451503225	0.633101511	4.084456441	-2.689635
Kif1b	Kif1b-010	258.5565	0.000593127	0.003047495	0.1550114	-6.451138435	0.358419248	2.312212188	-2.689554
Cox17	Cox17-002	604.70712	0.000784886	0.003771372	0.155020126	-6.450775281	1.047800249	6.759123947	-2.689473
Dynll1	Dynll1-006	841.09745	0.000117161	0.000898718	0.155069692	-6.448713382	1.296503316	8.360778282	-2.689011
N6amt1	N6amt1-002	228.19875	0.009302311	0.026038259	0.155071083	-6.44865553	0.46994206	3.030494464	-2.688998
Ttyh1	Ttyh1-001	524.26368	0.000126495	0.000953235	0.155083809	-6.448126364	0.768743849	4.956957481	-2.68888
Celf2	Celf2-017	550.80967	0.001708322	0.006907098	0.155168498	-6.444607075	1.294155175	8.340321598	-2.688092

Safb2	Safb2-007	1112.8168	9.83244E-05	0.000787377	0.155179185	-6.444163244	1.942869112	12.52016572	-2.687993
Malsu1	Malsu1-201	1042.0457	0.000182987	0.001247973	0.155197043	-6.443421715	1.798332625	11.58741549	-2.687827
Zeb2	Zeb2-004	509.25143	0.004863863	0.015650819	0.155204535	-6.443110714	0.711016043	4.581155087	-2.687757
Otub1	Otub1-002	484.01884	0.046246135	0.092223006	0.155245109	-6.441426764	0.711629452	4.583909	-2.68738
Pms2	Pms2-004	471.73176	0.006694732	0.020087128	0.155269513	-6.440414337	0.777673159	5.008537364	-2.687154
Zranb2	Zranb2-004	1818.9833	2.14736E-05	0.000265984	0.155355703	-6.436841257	3.312898952	21.32460465	-2.686353
30400K19R	0400K19Rik-	2326	3.48115E-07	1.88161E-05	0.155393535	-6.435274172	3.662385678	23.56845596	-2.686002
Ssr3	Ssr3-001	45835.743	7.92651E-09	2.31621E-06	0.155426612	-6.433904634	76.45745768	491.9199913	-2.685695
Cmas	Cmas-001	10233.656	8.76376E-07	3.24145E-05	0.155456871	-6.432652295	16.4400237	105.7529562	-2.685414
Ebf1	Ebf1-005	1098.1317	0.002918883	0.010533638	0.155464467	-6.432338002	1.86514774	11.99726068	-2.685343
P23-327P24	23-327P24.2-	460	0.000192876	0.001296564	0.155565023	-6.4281802	0.725205978	4.661754706	-2.68441
H2-DMb2	H2-DMb2-00	1216.4584	3.80289E-06	8.16753E-05	0.155584489	-6.427375952	2.223493488	14.29122858	-2.68423
Aco2	Aco2-006	302.45866	0.049475481	0.097202731	0.155664615	-6.424067526	0.540958888	3.475156427	-2.683487
Cnnm3	Cnnm3-002	907.62314	0.003318796	0.011622572	0.155699544	-6.422626408	1.525646662	9.798658541	-2.683163
Acin1	Acin1-019	858.81273	0.000233109	0.001494847	0.155711143	-6.422147981	1.348171762	8.658158557	-2.683056
42615	Sept2-202	12432.625	2.89887E-06	6.90448E-05	0.155741016	-6.420916132	19.64334102	126.1282452	-2.682779
Reps1	Reps1-008	486.22995	0.004999577	0.016001547	0.15575161	-6.420479375	0.818479344	5.255029749	-2.682681
Rtn4ip1	Rtn4ip1-001	536.3497	0.044413299	0.089290649	0.155766822	-6.419852365	1.031838494	6.6242508	-2.68254
Hnrnph2	Hnrnph2-002	1136.431	0.00922042	0.025864225	0.155779253	-6.419340046	1.549300247	9.945485116	-2.682425
Zfp280d	Zfp280d-017	802.06733	5.36769E-06	0.000102022	0.15580523	-6.418269804	1.239715338	7.956827522	-2.682184
Col5a2	Col5a2-003	995.97289	6.7552E-06	0.000118771	0.155823848	-6.417502925	1.720079153	11.03861299	-2.682012
00082M22R	0082M22Rik-	1078	5.91067E-06	0.000108547	0.155828129	-6.417326602	1.780905792	11.42865412	-2.681972
Dnajb6	Dnajb6-005	306.68429	0.010368913	0.028401734	0.155835174	-6.417036484	0.510742526	3.277453425	-2.681907
Fam102b	Fam102b-001	8112.702	0.002922031	0.010540237	0.155854405	-6.416244708	13.80402508	88.57000288	-2.681729
Adam17	Adam17-002	675.1791	0.00637656	0.019327185	0.155933818	-6.412977084	1.08999114	6.990088204	-2.680994
42621	Sept8-002	1780.8248	0.000178834	0.001226242	0.155962245	-6.411808185	2.847024373	18.25457418	-2.680731
Cyfip1	Cyfip1-006	2977.7334	0.00057856	0.002993306	0.15596823	-6.411562144	4.528554588	29.03510916	-2.680676
Adam15	Adam15-010	1548.7352	0.029356136	0.064167953	0.155988378	-6.410733991	1.103821801	7.076307937	-2.68049
Ly86	Ly86-201	420	0.014334923	0.036574082	0.15606592	-6.40754881	1.208371485	7.742699268	-2.679773
Nsfl1c	Nsfl1c-007	201.99693	0.019437388	0.046444156	0.156071201	-6.407331996	0.230175663	1.474811889	-2.679724
42622	Sept9-001	1758.7644	0.004642621	0.015095261	0.156071763	-6.407308912	1.532536073	9.81943204	-2.679719
Fance	Fance-009	73.644636	0.019489288	0.046545438	0.156178182	-6.40294302	0.11130878	0.712703774	-2.678735
Mrpl15	Mrpl15-007	1588.121	2.7851E-05	0.00031903	0.156196309	-6.402199929	2.823990559	18.07975216	-2.678568
Rps2-ps9	Rps2-ps9-001	527	0.006816666	0.020378007	0.15619726	-6.40216097	0.999935456	6.401747751	-2.678559
Lpcat3	Lpcat3-008	294.79725	0.00578351	0.017922251	0.156254182	-6.39982873	0.378637232	2.423213438	-2.678033
Use1	Use1-005	724.52115	0.004068301	0.013595406	0.156291302	-6.398308727	0.8034045	5.140430024	-2.677691
Lmo7	Lmo7-007	1334.9079	8.79965E-05	0.000725711	0.156337122	-6.396433491	1.9235269	12.30371188	-2.677268
Gm37993	Gm37993-001	109	0.016646401	0.041082234	0.156353345	-6.395769806	0.198677707	1.270696881	-2.677118
Camta1	Camta1-004	659.24787	0.040550486	0.083020751	0.15643156	-6.392571948	1.042398981	6.663610488	-2.676396
Cdc6	Cdc6-001	433.41334	0.038387378	0.079519033	0.156468064	-6.391080547	1.039468268	6.643325424	-2.67606
Ccdc141	Ccdc141-001	769.90849	3.88878E-05	0.000401894	0.156490447	-6.390166437	1.13219285	7.234900753	-2.675854
Mtm1	Mtm1-006	82.775254	0.021133945	0.049598981	0.156501933	-6.389697451	0.111690573	0.713668968	-2.675748
Ddx39	Ddx39-005	324.26808	0.04329038	0.087479823	0.156586809	-6.386233977	0.313872821	2.004465271	-2.674965
Supt20	Supt20-003	282.79259	0.002055362	0.007997132	0.156592533	-6.386000521	0.670458265	4.28154683	-2.674913
Entpd5	Entpd5-201	304.15475	0.015101627	0.038056856	0.156662371	-6.38315376	0.358641418	2.289263314	-2.674269
Ewsr1	Ewsr1-011	1403.5481	0.000165758	0.001162611	0.156669186	-6.382876101	2.635747536	16.82364995	-2.674207
Usp7	Usp7-005	1385.0857	0.000189888	0.001281008	0.15678683	-6.37808673	2.613197014	16.6671972	-2.673124
Cltc	Cltc-005	1354.853	1.21705E-05	0.00017727	0.156836585	-6.37606336	2.271547747	14.48353236	-2.672666
Hus1	Hus1-001	2127.7681	0.001972014	0.007752392	0.156843993	-6.375762172	3.588907097	22.88201811	-2.672598
Ankrd39	Ankrd39-004	488.23459	0.000741191	0.003613924	0.156858407	-6.375176291	0.743070773	4.737207177	-2.672465
Ppp3ca	Ppp3ca-007	500.11522	0.00126419	0.005476664	0.156879141	-6.374333742	0.785748989	5.008626292	-2.672275
Gm17084	Gm17084-002	356.43495	0.018861533	0.04538163	0.156919147	-6.372708603	0.456017856	2.906068912	-2.671907
Etohd2	Etohd2-001	648.70614	7.53067E-05	0.000650506	0.157000528	-6.369405333	0.977601454	6.226739917	-2.671159
Nanog	Nanog-003	221.41936	0.001006601	0.004578262	0.157037245	-6.367916102	0.428542594	2.728923284	-2.670821
Dtymk	Dtymk-003	250.14084	0.038606475	0.079861796	0.157038435	-6.367867851	0.365358006	2.326551503	-2.67081
Nanog	Nanog-002	161.61244	0.002137275	0.00823972	0.157047866	-6.367485452	0.312816001	1.991851337	-2.670724
Gm13694	Gm13694-001	76.210607	0.023009088	0.052950432	0.157057377	-6.36709986	0.111696287	0.71118141	-2.670636
Ttc26	Ttc26-001	509.31598	0.038597702	0.079852993	0.157120723	-6.364532841	1.114422927	7.092781316	-2.670055
Lrrc61	Lrrc61-001	764.4505	0.00457262	0.014921135	0.157129328	-6.364184274	0.755105276	4.805629125	-2.669976
Snx29	Snx29-002	432.4054	0.00208586	0.008093429	0.157132143	-6.364070285	0.470058585	2.991485872	-2.66995
Gm37452	Gm37452-001	805	0.000180385	0.001233974	0.157161787	-6.36286988	1.163269671	7.401733554	-2.669678
Tada1	Tada1-002	841.66472	0.000265565	0.001646265	0.157205026	-6.361119792	1.352136072	8.601099529	-2.669281
Ank2	Ank2-020	202.83544	0.006889315	0.020552133	0.157216091	-6.360672066	0.183285133	1.165816627	-2.669179
Vprbp	Vprbp-007	341.8115	0.00752286	0.022054093	0.157223779	-6.360361039	0.560642297	3.56588742	-2.669109
Gm15650	Gm15650-001	104.0592	0.029733939	0.064861309	0.157236942	-6.359828591	0.230426599	1.465473675	-2.668988
Cct6a	Cct6a-001	10919.989	1.65085E-05	0.00021975	0.157298743	-6.357329872	22.62435958	143.830517	-2.668421
Napb	Napb-001	223.0062	0.039439151	0.081172461	0.15731851	-6.356531109	0.329593171	2.095069246	-2.66824
Rpl23	Rpl23-005	605.55153	0.00432714	0.014282023	0.157358406	-6.354919475	1.09480891	6.957422464	-2.667874
Psmd1	Psmd1-010	261.10099	0.009489337	0.026460133	0.157437618	-6.351722129	0.60260815	3.827599519	-2.667148
Nme1	Nme1-001	1844.657	0.034596052	0.073269527	0.157453344	-6.351087717	4.41050289	28.01149073	-2.667004
Itsn1	Itsn1-006	545.12403	0.017959624	0.043612954	0.157516521	-6.348540435	1.053675042	6.689298608	-2.666425
Pogz	Pogz-005	3159.1069	4.08833E-06	8.55377E-05	0.157516795	-6.348529367	4.855286934	30.82393169	-2.666422
Helb	Helb-003	277.20273	0.015664732	0.039168066	0.157534349	-6.347821978	0.480502679	3.050145466	-2.666262
Gm4654	Gm4654-001	400	0.000515693	0.002736356	0.157563592	-6.346643849	0.746571017	4.738220352	-2.665994
Ncoa3	Ncoa3-003	119.9184	0.007856701	0.022808273	0.15760406	-6.345014201	0.197132603	1.250809166	-2.665623
Atr	Atr-004	223.41012	0.003219156	0.01135607	0.157617768	-6.344462394	0.436088992	2.766750209	-2.665498
Gabpb1	Gabpb1-004	3429.3362	0.001690369	0.006850683	0.157619373	-6.344397781	6.416490124	40.70876571	-2.665483
Gm26931	Gm26931-001	434	0.005352861	0.016905072	0.157680912	-6.34192173	0.665658352	4.221553169	-2.66492
Adrbk1	Adrbk1-002	2158.6779	0.001754468	0.007067786	0.157704636	-6.340967698	3.646239396	23.12068623	-2.664703
10039B14R	0039B14Rik-	1594	4.23397E-05	0.000427894	0.157814548	-6.33655143	2.77109941	17.55921393	-2.663698
Ptchd1	Ptchd1-001	476.7305	0.005797103	0.017955396	0.157839093	-6.335566038	1.008903873	6.391977114	-2.663474
Wdfy1	Wdfy1-003	618.60013	0.022650781	0.052322738	0.157850525	-6.335107206	1.092019761	6.918062255	-2.663369
Nav1	Nav1-201	12759.754	2.54749E-06	6.35018E-05	0.157893142	-6.333397294	20.68451678	131.0032626	-2.66298
Cdc42bpa	Cdc42bpa-01	2265.2299	0.000669063	0.003343982	0.157901679	-6.333054901	4.055971981	25.68669323	-2.662902
Gm15542	Gm15542-001	2911	7.68863E-07	2.99411E-05	0.157943491	-6.331378337	5.181867811	32.8083656	-2.66252
Zscan26	Zscan26-201	15158.502	1.02099E-06	3.5458E-05	0.15796925	-6.330345934	24.73951263	156.6096732	-2.662284
Pdcd10	Pdcd10-001	2791.051	1.45739E-06	4.40645E-05	0.157969731	-6.330326643	5.040307973	31.90679585	-2.66228
Ddx26b	Ddx26b-003	8102.9945	1.29459E-06	4.11315E-05	0.157975573	-6.330092559	15.17418329	96.05398471	-2.662227
Fbxo3	Fbxo3-004	4229.0308	0.00513881	0.016352241	0.157986433	-6.329657443	6.99350232	44.26647401	-2.662127
Tuba1b	Tuba1b-001	31267.963	3.33547E-05	0.000360378	0.15800028	-6.329102716	61.27776962	387.8332981	-2.662001
Rbm6	Rbm6-007	2704.8443	2.23494E-05	0.000272894	0.158038467	-6.327573419	4.681853871	29.6247741	-2.661652
Phospho2	Phospho2-201	1757.1893	2.09966E-05	0.000261694	0.158060134	-6.326706	2.96424476	18.75390511	-2.661455
Inadl	Inadl-011	243.59422	0.002109359	0.008156886	0.158136207	-6.323662483	0.386041308	2.441194939	-2.66076
Rp2h	Rp2h-001	4791.5607	1.61299E-06	4.67779E-05	0.158171529	-6.322250317	8.815766472	55.73548237	-2.660438
Tial1	Tial1-001	3578.991	2.36284E-06	6.03484E-05	0.158177479	-6.322012497	6.198667907	39.18805597	-2.660384
Hhat	Hhat-003	1006	1.3621E-05	0.000191949	0.15818345	-6.321773875	1.593994524	10.07687294	-2.660329
Pan3	Pan3-021	1511.8332	3.44698E-06	7.69616E-05	0.158232578	-6.319811077	2.640470078	16.68727205	-2.659881
Pi4ka	Pi4ka-001	4920.3276	0.000595999	0.003059496	0.158249324	-6.319142318	7.312844832	46.21090724	-2.659729
Phka1	Phka1-004	629.68811	0.000442946	0.002435442	0.158326966	-6.31604349	0.702468993	4.436824713	-2.659021
Gm37499	Gm37499-001	1199	0.00018883	0.001276634	0.158339612	-6.315539022	1.927138946	12.17092121	-2.658906
Usp16	Usp16-007	1956.1982	4.04306E-05	0.00041358	0.15834436	-6.315349657	3.335924563	21.06753004	-2.658863
Cdkl3	Cdkl3-005	395.32304	0.012837504	0.033515094	0.158359973	-6.314727023	0.737328675	4.65602931	-2.65872
Gtf2a2	Gtf2a2-003	554.74772	0.037437352	0.077961289	0.158370476	-6.314308234	0.7035383	4.442357678	-2.658625
00094K13R	0094K13Rik-	331.33971	0.031006678	0.067145848	0.158379393	-6.313952716	0.413520452	2.610948584	-2.658543
Slmo2	Slmo2-001	3841.7217	8.21584E-06	0.00013548	0.158521255	-6.308302326	7.537750553	47.55040935	-2.657252
Gtf2h2	Gtf2h2-006	363.99239	0.009985033	0.027547879	0.158525231	-6.30814408	0.657272584	4.146170158	-2.657216
Ddx24	Ddx24-202	4128.2307	0.000933647	0.004320102	0.15856659	-6.306498738	5.723993854	36.09836001	-2.656839

AW011738	W011738-20	283	0.004335724	0.014304841	0.158594864	-6.305374436	0.580605504	3.6609351	-2.656582
Hnrnpa2b1	Inrnpa2b1-20	41689.806	9.82203E-08	9.39677E-06	0.158613599	-6.304629658	78.73837337	496.416284	-2.656412
Serpinb6a	Serpinb6a-201	690.59657	0.003962833	0.013323554	0.158641405	-6.303524622	1.210463457	7.630186207	-2.656159
Bivm	Bivm-001	594.12044	0.026174514	0.058566479	0.158649214	-6.303214348	0.869117019	5.478230863	-2.656088
Mapk8ip3	Mapk8ip3-00	534.10673	0.004738231	0.01532286	0.15866034	-6.302772312	1.041947618	6.567158597	-2.655987
Nars2	Nars2-001	872.40282	0.000336053	0.001971197	0.15867509	-6.302186433	1.789297571	11.27648688	-2.655852
Csde1	Csde1-009	3505.0959	9.81217E-06	0.000152737	0.158685289	-6.301781368	6.083747838	38.33844877	-2.65576
30551O13R	0551O13Rik-	193	0.017242142	0.042214839	0.158705878	-6.300963842	0.447726525	2.821108643	-2.655573
Gm10259	Gm10259-001	489	0.004285199	0.014175011	0.158751316	-6.29916038	1.035232299	6.52109428	-2.65516
Gm12338	Gm12338-001	711	0.00012635	0.000952602	0.15876093	-6.298778913	1.266300112	7.976144444	-2.655072
P23-375E13	23-375E13.3-	1994	6.11652E-07	2.5948E-05	0.158764958	-6.2986191	3.278905915	20.65257943	-2.655036
Cfap97	Cfap97-201	2528.6156	0.008050538	0.023240976	0.158781281	-6.297971615	4.357695358	27.44464167	-2.654887
Ndufa10	Ndufa10-003	773.66372	0.000170073	0.00118298	0.158803491	-6.297090787	1.138977522	7.172244861	-2.654685
Pcdhb3	Pcdhb3-001	307	0.021843548	0.050896697	0.158803981	-6.297071354	0.558580416	3.51742074	-2.654681
Gm28439	Gm28439-001	140970	6.60244E-09	2.13333E-06	0.158834294	-6.295869592	234.5678204	1476.808408	-2.654406
Sec22b	Sec22b-001	4485.6673	5.90441E-06	0.00010849	0.158837807	-6.29573033	7.47438512	47.05671309	-2.654374
Trmt112	Trmt112-001	1053.2162	0.005349365	0.016897145	0.158870931	-6.294417711	2.299186134	14.47203792	-2.654073
Usp53	Usp53-004	1891.907	9.53317E-05	0.000770395	0.15894755	-6.291383553	3.849108016	24.21621486	-2.653377
Osgepl1	Osgepl1-005	1397.8921	0.000302574	0.001821292	0.159012952	-6.288795889	2.43569234	15.31757197	-2.652784
Vim	Vim-001	78478.192	1.82741E-06	5.05971E-05	0.159017656	-6.288609883	126.2822909	794.1400624	-2.652741
Samhd1	Samhd1-001	6865.1007	3.12557E-05	0.000345103	0.159054177	-6.287165915	15.3011847	96.20108688	-2.65241
Stard7	Stard7-003	832.05254	0.000146559	0.001062134	0.159055994	-6.287094096	1.502526306	9.446524269	-2.652393
Xpr1	Xpr1-007	808.0995	0.00026246	0.001632039	0.159116956	-6.284685348	1.530834677	9.620814267	-2.651841
Senp6	Senp6-007	2006.8856	0.013618154	0.035116832	0.159141189	-6.283728341	3.106445055	19.52005683	-2.651621
Gm20548	Gm20548-00	911.76292	0.002987336	0.010719355	0.159165389	-6.282772935	1.619493936	10.17491267	-2.651401
Hmbox1	Hmbox1-009	1604.5369	1.17301E-06	3.87268E-05	0.159183783	-6.282046951	2.366747587	14.86801946	-2.651235
Yy1	Yy1-002	867.25029	0.000140104	0.001030177	0.159218856	-6.280663158	1.502133577	9.434395017	-2.650917
Trp53inp2	Trp53inp2-00	5040.0453	8.24963E-07	3.12267E-05	0.159230232	-6.280214416	8.278863954	51.99304075	-2.650814
Mast4	Mast4-014	983.9668	0.000877672	0.004120103	0.159234825	-6.280033262	2.279093439	14.31278261	-2.650772
Plekha1	Plekha1-005	2648.5602	2.95424E-06	6.98292E-05	0.159240273	-6.279818422	4.425027406	27.78836862	-2.650723
Rbbp4	Rbbp4-004	1078.9523	0.000494115	0.002648427	0.15926093	-6.279003913	2.236397509	14.04234871	-2.650536
Gm6335	Gm6335-001	685	7.11586E-05	0.000625399	0.159318991	-6.276715606	1.18598217	7.444072793	-2.65001
Fam60a	Fam60a-001	1243.4645	0.005831823	0.018040094	0.159325522	-6.27645832	3.060160628	19.20697063	-2.649951
Ubap2l	Ubap2l-008	1688.7309	5.72981E-06	0.000106453	0.15932973	-6.276292572	2.504348851	15.71802609	-2.649913
Ttc3	Ttc3-026	1270.3737	8.73132E-05	0.000721814	0.159340882	-6.275853313	2.360900954	14.81666808	-2.649812
Tspan18	Tspan18-001	1762.5649	0.000876896	0.004118717	0.159348514	-6.275552712	3.561455189	22.35009977	-2.649743
Lyar	Lyar-003	577.30757	0.034181298	0.072561636	0.159381388	-6.274258323	0.896230399	5.62318104	-2.649445
Klhl20	Klhl20-001	4572.8189	5.92875E-06	0.000108763	0.15938672	-6.274048434	7.551072418	47.37579408	-2.649397
Ankle2	Ankle2-201	2336.3412	0.000592015	0.003043607	0.159441404	-6.271896617	4.274258817	26.80770941	-2.648902
Gm15951	Gm15951-001	147	0.039087978	0.080624105	0.159463424	-6.271030519	0.187698679	1.177064147	-2.648703
Gstm3	Gstm3-001	205	0.02717163	0.060383359	0.159509997	-6.269199542	0.345301076	2.164761346	-2.648281
Klhl32	Klhl32-005	2317.292	3.09362E-06	7.20303E-05	0.159531857	-6.268340489	3.974225927	24.91180129	-2.648084
Camta2	Camta2-006	799.87184	0.00151067	0.006289041	0.159538561	-6.268077108	1.353974292	8.486815265	-2.648023
Rnase4	Rnase4-202	2113.9198	0.002688517	0.009862404	0.15953899	-6.268060247	3.739323553	23.43830531	-2.648019
Gimap1os	Gimap1os-001	698.42839	0.0009504	0.004376469	0.15953933	-6.268046866	1.478565916	9.267720455	-2.648016
00026H17R	0026H17Rik-	2127	6.34718E-07	2.65174E-05	0.159567685	-6.266933059	3.369864315	21.11871408	-2.64776
Usp49	Usp49-001	3799.3488	1.93475E-06	5.26332E-05	0.159630206	-6.264478548	5.435999792	34.05370408	-2.647194
Trip12	Trip12-201	12025.487	8.73139E-08	8.87389E-06	0.159658609	-6.263364097	18.53755658	116.1074664	-2.646938
Garnl3	Garnl3-004	944.37876	8.87633E-05	0.000730628	0.159659105	-6.263344648	1.705523851	10.68228369	-2.646933
Gm37612	Gm37612-001	822	0.000378134	0.002156002	0.159682485	-6.262427574	1.254568347	7.856643412	-2.646722
Bcas2	Bcas2-003	1359.4749	0.002536746	0.009423377	0.159707644	-6.261441043	2.291126026	14.34575053	-2.646495
Tbc1d2	Tbc1d2-002	511.94876	0.000985774	0.0044987	0.15971351	-6.261211072	0.912997928	5.716472735	-2.646442
Fip1l1	Fip1l1-003	4886.0391	1.67198E-07	1.26693E-05	0.159770143	-6.258991688	8.343255878	52.2203692	-2.64593
Slk	Slk-001	34384.04	8.93846E-10	8.50395E-07	0.15987539	-6.254871373	52.53240781	328.5834538	-2.64498
Ubox5	Ubox5-002	324.28182	0.01633689	0.040475875	0.159919251	-6.253155839	0.180470461	1.128509918	-2.644584
Morc2a	Morc2a-005	641.44827	0.001949504	0.007685371	0.159996191	-6.250148802	1.210330246	7.564744136	-2.643891
Mdm4	Mdm4-008	1588.4204	8.63562E-07	3.21361E-05	0.160009608	-6.249624699	2.807398334	17.54518597	-2.64377
Pon2	Pon2-001	18479.121	3.26108E-07	1.82142E-05	0.160011498	-6.24955091	27.46765512	171.660509	-2.643753
Camk2d	Camk2d-001	3630.9416	1.55526E-05	0.000210782	0.16001749	-6.249316871	5.213060613	32.57806764	-2.643698
Mtbp	Mtbp-001	715.06673	0.008088311	0.023320537	0.160110969	-6.245668261	1.2052972	7.527886467	-2.642856
P24-309H3	24-309H3.5-	255	0.001888828	0.007482347	0.160116519	-6.245451807	0.347593803	2.170880347	-2.642806
Cand1	Cand1-004	247.35177	0.009568921	0.026617035	0.160198747	-6.242246074	0.37388802	2.333901022	-2.642065
Il1rl1	Il1rl1-201	104.02788	0.049867724	0.097760262	0.160219644	-6.241431921	0.223204964	1.39311859	-2.641877
Shisa5	Shisa5-001	7816.4507	0.000329899	0.001943751	0.160251882	-6.240176328	11.52631778	71.92625537	-2.641587
Slc45a4	Slc45a4-004	746.28881	0.026992276	0.06004712	0.160252834	-6.240139261	1.387922093	8.660827145	-2.641578
Gm37745	Gm37745-001	1405.4725	1.71924E-05	0.000226129	0.160332897	-6.237023218	2.666349332	16.63008269	-2.640858
Mybl1	Mybl1-001	388.4565	0.002506984	0.009337124	0.160341449	-6.236690545	0.673605304	4.201067833	-2.640781
Cops2	Cops2-001	4474.5622	5.39899E-06	0.000102389	0.16038842	-6.234864085	7.404601778	46.16668569	-2.640358
Tmem165	Tmem165-00	778.29097	0.00061584	0.003138769	0.160418968	-6.233676804	1.359779475	8.476425774	-2.640083
Prim1	Prim1-002	345.7687	0.002531827	0.009407145	0.160448516	-6.23252881	0.800181419	4.987153744	-2.639818
Ube2k	Ube2k-002	2003.2037	0.000455442	0.002489447	0.160480386	-6.231291102	3.929655091	24.4868248	-2.639531
Sgpp2	Sgpp2-001	419.19835	0.000929783	0.0043053	0.160550071	-6.228586479	0.790298008	4.922439488	-2.638905
Blvra	Blvra-002	252.27938	0.010389359	0.028441816	0.160563319	-6.228072548	0.343554483	2.139682246	-2.638786
Gm16701	Gm16701-001	265	0.006358615	0.019280315	0.160584945	-6.227233813	0.343695759	2.140273853	-2.638591
Ppp1r14c	Ppp1r14c-201	437	0.001829414	0.007299327	0.160587989	-6.22711577	0.582628398	3.628094487	-2.638564
Arl6ip6	Arl6ip6-001	1902.0802	3.06071E-05	0.000339694	0.160612654	-6.226159501	3.562068874	22.17800896	-2.638343
00035N22R	0035N22Rik-	284	0.004209154	0.013978814	0.160621448	-6.225818613	0.48209178	3.001415979	-2.638264
H2-Aa	H2-Aa-001	5011.6133	0.036952275	0.077199917	0.160636263	-6.225244408	13.83138119	86.10372841	-2.63813
Mycbp2	Mycbp2-002	4017.0219	4.55243E-06	9.20429E-05	0.160656537	-6.224458826	6.24799768	38.8904043	-2.637948
Tcp1	Tcp1-001	6166.6096	1.04869E-05	0.000159775	0.160708515	-6.222445641	12.10582931	75.32786481	-2.637482
Gm18829	Gm18829-001	183	0.014818192	0.037541467	0.160717109	-6.222112937	0.327174032	2.03571378	-2.637405
Alox8	Alox8-001	1091	2.87141E-06	6.86773E-05	0.160727507	-6.2217104	1.889319212	11.75479699	-2.637311
Rtcb	Rtcb-002	529.20061	0.003747259	0.012757814	0.160777772	-6.219765262	1.039251174	6.465577687	-2.63686
Naa20	Naa20-006	541.19524	0.000154905	0.001106464	0.160784188	-6.219517046	0.7424739	4.617829079	-2.636803
Gm15487	Gm15487-001	509	9.66146E-05	0.000777685	0.1608308	-6.217714521	0.847984166	5.27252346	-2.636384
Ccdc28a	Ccdc28a-002	346.57189	0.011173608	0.030055454	0.160839182	-6.217390493	0.41087333	2.554559938	-2.636309
Tmem258	Tmem258-00	424.41137	0.014314308	0.036528942	0.160839352	-6.217383922	0.857002824	5.328315578	-2.636308
Ilkap	Ilkap-002	1187.548	0.008783547	0.024861412	0.160850312	-6.216960286	2.236246657	13.90265666	-2.636209
Snhg11	Snhg11-003	696.24359	5.94024E-05	0.000546951	0.160855074	-6.21677622	1.105717577	6.873998742	-2.636167
Spag1	Spag1-201	1394.1334	0.009225927	0.025875205	0.160857157	-6.216695733	1.929676033	11.99620876	-2.636148
Kctd3	Kctd3-010	659.48181	9.36839E-06	0.000147865	0.160857209	-6.21669372	1.077413381	6.697948999	-2.636148
Pex19	Pex19-002	2628.1024	1.25078E-06	4.01867E-05	0.160879701	-6.215824571	4.880814291	30.3382854	-2.635946
Nle1	Nle1-004	358.27084	0.000237472	0.001515257	0.160891333	-6.215375186	0.747874047	4.648317794	-2.635841
Gm20236	Gm20236-001	3068	1.33892E-05	0.000189652	0.160905742	-6.214818626	4.998046793	31.0619543	-2.635712
Mettl14	Mettl14-003	740.21792	0.005699142	0.01772089	0.160907224	-6.214761365	1.149274931	7.142469437	-2.635699
Znhit1	Znhit1-002	563.02918	0.031995705	0.068768548	0.16092643	-6.214019662	0.852966769	5.300352271	-2.635527
Frmd4b	Frmd4b-001	4635.3926	0.000483398	0.002604432	0.16094941	-6.213132432	6.307309545	39.18814949	-2.635321
Itch	Itch-005	2777.4178	9.10614E-07	3.325E-05	0.160992038	-6.211487304	4.723444334	29.33961451	-2.634939
Gm2093	Gm2093-201	479	0.00160819	0.006597259	0.161004125	-6.211020998	0.844637763	5.246062883	-2.63483
Mob3c	Mob3c-001	3865.6192	2.69009E-05	0.000310953	0.161050151	-6.209245972	7.095154331	44.05555845	-2.634418
Prim1	Prim1-001	1110.98	0.000621878	0.003163263	0.161051699	-6.209186299	2.302073996	14.29400632	-2.634404
Gm37926	Gm37926-001	1772	5.37562E-06	0.000102116	0.161072377	-6.208389153	2.741518999	17.02041682	-2.634219
Adgrl2	Adgrl2-019	3122.7998	0.005115676	0.016294234	0.161082867	-6.207984849	5.181825253	32.16869266	-2.634125
Stxbp3-ps	Stxbp3-ps-001	359	0.000420785	0.002341112	0.161123922	-6.206403058	0.481148218	2.98619977	-2.633757
Mrps18c	Mrps18c-006	173.34891	0.023537976	0.053884662	0.161136805	-6.205906831	0.279890624	1.736975136	-2.633642
Sike1	Sike1-005	1088.4071	0.000359068	0.002071744	0.161166547	-6.204761581	1.5651804	9.711571214	-2.633376
Gm16072	Gm16072-001	156	0.011696429	0.031206878	0.161227793	-6.202404586	0.184596453	1.144941887	-2.632828

Gm13461	Gm13461-001	796	0.012495802	0.032815612	0.161264559	-6.200990492	1.96418885	12.17991638	-2.632499
Sspn	Sspn-003	621.27738	0.020572076	0.048566494	0.16131822	-6.198927793	1.105759355	6.854522397	-2.632019
Mgst2	Mgst2-010	459.47586	0.000100782	0.000801247	0.161346029	-6.19785939	0.793075345	4.915369477	-2.63177
Pnisr	Pnisr-002	2828.1177	6.86216E-05	0.000609515	0.161381325	-6.196503834	4.834270159	29.95557357	-2.631454
Fbxo22	Fbxo22-002	7178.983	8.43222E-05	0.000705776	0.161400047	-6.195785066	9.63853639	59.71829982	-2.631287
Wdr76	Wdr76-004	2646.1436	7.29982E-07	2.89374E-05	0.161417561	-6.195112827	4.771539456	29.56022529	-2.631131
Pdcd2l	Pdcd2l-001	834.70604	0.000427384	0.002368848	0.161421069	-6.194978177	1.585039511	9.819285181	-2.631099
Syt12	Syt12-001	814.12917	0.005650242	0.017605604	0.161454972	-6.193677341	1.706794002	10.57133133	-2.630796
Naa16	Naa16-001	2007.5553	1.51795E-05	0.000207213	0.161515761	-6.191346224	3.760985048	23.28556057	-2.630253
Pkn3	Pkn3-002	18925.974	9.18314E-05	0.000750363	0.161575309	-6.189064429	17.55074971	108.6227207	-2.629721
Ccser2	Ccser2-005	2052.2699	0.035011864	0.073930724	0.161596314	-6.188259952	2.896865821	17.92655875	-2.629534
Ikbkb	Ikbkb-007	1379.6762	4.04417E-05	0.00041358	0.161602668	-6.188016638	2.295399212	14.20396852	-2.629477
Apol7b	Apol7b-001	347	0.010204609	0.028030144	0.161611769	-6.18766817	0.741526609	4.588320596	-2.629396
Pick1	Pick1-008	816.5586	0.003295299	0.011559195	0.161649223	-6.186234509	1.525911406	9.439645798	-2.629062
Gm15927	Gm15927-003	251.51938	0.019064669	0.045741923	0.161655176	-6.186006702	0.625267079	3.867906339	-2.629008
Rnf20	Rnf20-004	896.77079	3.82064E-06	8.16753E-05	0.16167534	-6.185235169	1.651427398	10.21446682	-2.628828
Asph	Asph-009	602.18268	0.000755021	0.003664888	0.161698869	-6.184335142	1.097408572	6.786742394	-2.628619
Il2rg	Il2rg-001	4319.2036	0.002035685	0.007940124	0.161828783	-6.179370439	8.072076479	49.88035077	-2.62746
Gm13881	Gm13881-001	962	0.032691967	0.069991417	0.161876757	-6.177539123	1.677495406	10.3627935	-2.627032
Col8a1	Col8a1-001	30322	0.01195378	0.031705799	0.161970642	-6.173958353	48.39755579	298.8044938	-2.626196
Ifnar2	Ifnar2-005	2841.7795	1.7658E-05	0.000231041	0.162029264	-6.171724641	5.359322731	33.07626415	-2.625674
Vmn1r-ps63	Vmn1r-ps63-00	82	0.011556209	0.030905385	0.162036503	-6.171448921	0.150095428	0.926306267	-2.625609
Rbms3	Rbms3-004	146.12305	0.03953664	0.081320144	0.162073164	-6.170052936	0.253935246	1.566793909	-2.625283
Gm16185	Gm16185-001	2244	1.95964E-05	0.000248584	0.162156247	-6.166891613	3.932416178	24.25078435	-2.624543
Ero1lb	Ero1lb-201	1613	1.25043E-05	0.000180706	0.162215487	-6.164639493	3.821483278	23.55806674	-2.624017
Ap2b1	Ap2b1-005	6042.6708	0.000162047	0.00114247	0.162236197	-6.163852577	8.549651766	52.69879307	-2.623832
Thoc2	Thoc2-002	218.88807	0.005855559	0.018097175	0.162247723	-6.163414684	0.36226619	2.232796755	-2.62373
Zfp874a	Zfp874a-002	1191.8843	0.000624204	0.003171015	0.162327495	-6.160385845	2.259023387	13.9164557	-2.623021
Mrpl17	Mrpl17-001	1923.4042	0.000302184	0.001820227	0.16236273	-6.15904894	3.325349606	20.48099097	-2.622708
P24-409D4	24-409D4.3-(	535	6.9978E-05	0.000617877	0.16239378	-6.15787132	0.964016529	5.936289734	-2.622432
Brd8	Brd8-005	1471.5141	3.37059E-05	0.000363485	0.162426455	-6.156632567	2.56957233	15.81991269	-2.622141
Gm13650	Gm13650-001	261	0.001396126	0.005919448	0.162456209	-6.155504942	0.509795336	3.13804771	-2.621877
Fyttd1	Fyttd1-001	5979.6229	1.45594E-07	1.18609E-05	0.162499079	-6.153881043	11.44132399	70.40854678	-2.621497
Naa30	Naa30-003	365.92796	0.011858372	0.031523537	0.162519688	-6.153100653	0.661556401	4.070623124	-2.621314
Taok3	Taok3-004	811.5502	5.85159E-05	0.000540791	0.162532842	-6.152602673	1.395499179	8.585951982	-2.621197
Dusp19	Dusp19-004	325.02182	0.006660902	0.020005151	0.162534849	-6.152526726	0.417122371	2.566356537	-2.621179
Nedd4	Nedd4-010	6174.5575	1.08552E-07	9.9407E-06	0.162584072	-6.150663998	10.0632143	61.89544989	-2.620742
Gm17103	Gm17103-001	406.41058	0.004563423	0.0148968	0.162638774	-6.148595273	0.753389444	4.632286775	-2.620257
Ikbkb	Ikbkb-006	749.05563	0.000319679	0.001897305	0.162641785	-6.148481469	1.383454624	8.506145117	-2.62023
P23-148L8.	23-148L8.3-(	2433.2	0.00564147	0.017588122	0.162729716	-6.145159129	5.63315458	34.61663129	-2.61945
Slc25a25	Slc25a25-012	626.34925	0.000729289	0.003568814	0.162858366	-6.140304765	1.340827568	8.233089903	-2.61831
Mast4	Mast4-001	2178.908	0.006366692	0.01930069	0.162864536	-6.140072123	5.059267637	31.06426818	-2.618256
Zfp667	Zfp667-002	609.45989	0.03689927	0.077112697	0.162911713	-6.138294044	0.928342051	5.698436479	-2.617838
Pcna	Pcna-001	5408.4035	0.001994674	0.007820238	0.162924417	-6.137815434	12.15841389	74.62610045	-2.617725
Arhgap42	Arhgap42-001	1161.5743	7.90078E-05	0.000673977	0.16293291	-6.137495489	2.819970404	17.30755563	-2.61765
P24-544J6.	24-544J6.5-0	1873	2.89783E-06	6.90448E-05	0.16293449	-6.13743598	3.40518852	20.89912654	-2.617636
Ube2i	Ube2i-014	2441.5658	4.43082E-05	0.000442179	0.162981632	-6.135660726	4.855740081	29.79317371	-2.617219
Cul9	Cul9-005	533.93859	0.004087887	0.013640141	0.162997642	-6.135058064	0.754909981	4.631416566	-2.617077
Osgepl1	Osgepl1-006	224.79683	0.033758543	0.071836803	0.163004813	-6.134788197	0.330820083	2.029511143	-2.617014
D5Ertd579e	5Ertd579e-00	2109.7323	1.12845E-05	0.00016826	0.163007959	-6.134669784	3.038086151	18.63765531	-2.616986
Fgd4	Fgd4-001	1319.8673	0.000388638	0.002203052	0.163014677	-6.13441697	3.078546929	18.88509052	-2.616926
Gm5507	Gm5507-001	260	0.001255075	0.005448203	0.163043371	-6.133337366	0.536878512	3.292857042	-2.616672
Setdb1	Setdb1-002	3719.691	9.64478E-05	0.000776707	0.163073728	-6.132195617	6.284216276	38.5360435	-2.616404
Hs2st1	Hs2st1-001	5205.0858	2.20142E-07	1.49728E-05	0.163097317	-6.131308718	9.897333359	60.68360631	-2.616195
Cd44	Cd44-002	4491.3939	0.008754713	0.024807554	0.163107581	-6.130922864	11.85328374	72.67156828	-2.616104
Gm37420	Gm37420-001	2414.6148	9.34187E-07	3.36377E-05	0.163184493	-6.128033255	4.256690785	26.08514269	-2.615424
Arpc1a	Arpc1a-006	306.6919	0.002062222	0.008018169	0.1631887	-6.127875271	0.581973798	3.566262848	-2.615387
Mpeg1	Mpeg1-201	6300	0.006259588	0.019070529	0.163218759	-6.126746755	20.13891689	123.3860437	-2.615121
Zfp426	Zfp426-003	1641.0414	5.00098E-05	0.000480728	0.163240131	-6.125944604	2.640752413	16.177103	-2.614932
Rnmt	Rnmt-002	850.53336	0.039543216	0.081323113	0.163242675	-6.125849157	1.704372676	10.44072992	-2.61491
Uty	Uty-006	649.33705	0.020189441	0.047840623	0.163265862	-6.124979131	1.007679841	6.172018	-2.614705
RP23-7E4.3	P23-7E4.3-00	395	0.000152439	0.001093868	0.163277618	-6.124538145	0.700415529	4.289721623	-2.614601
Pole	Pole-002	1004.1583	2.93521E-05	0.000330753	0.163312304	-6.12323737	1.791250731	10.96825341	-2.614295
Shroom2	Shroom2-201	551.3707	0.020626011	0.04866636	0.163321206	-6.122903605	0.84510835	5.17451696	-2.614216
Zeb1	Zeb1-001	18292.666	4.01972E-09	1.72094E-06	0.163331537	-6.122516298	30.93479879	189.3988098	-2.614125
Hdlbp	Hdlbp-001	41963.356	4.83825E-09	1.8062E-06	0.163340456	-6.122182013	72.60236116	444.4848695	-2.614046
Rbm5	Rbm5-008	869.82306	0.020128708	0.047739648	0.163408092	-6.119647984	1.619650215	9.91168917	-2.613449
Rnasel	Rnasel-002	1321.9714	0.003336026	0.011670981	0.16341772	-6.119287433	3.282212437	20.08480131	-2.613364
Gm7584	Gm7584-001	468	0.009596248	0.026680046	0.163420723	-6.11917499	0.813697917	4.979159946	-2.613337
Rnf5	Rnf5-001	497.9297	0.032361689	0.069400027	0.163465575	-6.117495985	0.932023424	5.701649552	-2.612941
Gcc2	Gcc2-001	6170.032	5.01668E-06	9.81274E-05	0.163482298	-6.116870208	10.91522057	66.76698751	-2.612794
Slc28a3	Slc28a3-001	527	3.25844E-05	0.000354967	0.163504495	-6.116039804	0.837139201	5.119976677	-2.612598
Ifi203	Ifi203-004	1520.1627	5.11576E-05	0.000489153	0.16350885	-6.1158769	2.810492501	17.18862617	-2.612559
Pex1	Pex1-003	434.80244	0.000272699	0.001679706	0.163513666	-6.115696779	0.797139883	4.875065813	-2.612517
Sorbs2	Sorbs2-012	882.96229	0.028988132	0.063489163	0.163523298	-6.115336557	1.494547668	9.139661991	-2.612432
Vps26a	Vps26a-202	8782.578	8.24429E-07	3.12267E-05	0.163657097	-6.110336898	15.37215107	93.92902188	-2.611252
Tmem209	Tmem209-010	328.53185	0.002978678	0.010695011	0.163676203	-6.109623642	0.548396167	3.350494184	-2.611084
Srgap2	Srgap2-007	254.46543	0.004249753	0.014084965	0.163685792	-6.109265739	0.463002411	2.828604769	-2.610999
Cnot2	Cnot2-001	2777.852	0.018255991	0.04418853	0.163688476	-6.109165556	4.78283207	29.21911294	-2.610975
Fam213a	Fam213a-002	604.07278	0.023953072	0.054616384	0.163770746	-6.106096639	1.162317705	7.097224229	-2.61025
Pigg	Pigg-001	3655.0036	3.76258E-05	0.000392532	0.163819399	-6.104283181	5.436307786	33.18476219	-2.609822
Proser1	Proser1-002	500.09514	0.000798872	0.003824626	0.1638775	-6.102118948	0.837150084	5.108389389	-2.60931
Ptch1	Ptch1-002	1918.4174	0.046675029	0.092883606	0.163882981	-6.101914861	3.14181731	19.17110173	-2.609262
Birc6	Birc6-022	418.11098	0.043336118	0.087554934	0.163946804	-6.099539474	0.603281245	3.679737766	-2.6087
Chd8	Chd8-012	1263	0.000158748	0.001126398	0.163953056	-6.099306883	1.976193859	12.05341281	-2.608645
Nipsnap3b	Nipsnap3b-00	1020.6751	0.00205568	0.007997165	0.163971487	-6.098621296	1.872995649	11.42269115	-2.608483
Mettl4	Mettl4-201	1876	1.07089E-06	3.65501E-05	0.164035169	-6.096253664	3.379253414	20.600786	-2.607923
Ptrh2	Ptrh2-001	1984.9845	0.01766732	0.043068022	0.164050534	-6.095682683	3.377251034	20.58665064	-2.607788
Prkd3	Prkd3-007	2088.4055	0.001065574	0.004780707	0.164074612	-6.094788141	3.26369061	19.89150282	-2.607576
Airn	Airn-001	3488.3324	3.10458E-05	0.000343227	0.164083661	-6.09445203	4.944591248	30.13457417	-2.607497
Xlr3a	Xlr3a-005	96.705889	0.016138988	0.04007252	0.164085994	-6.094365384	0.183976615	1.121220712	-2.607476
Gm38366	Gm38366-001	759	0.000186027	0.001264173	0.164120268	-6.093092672	1.186454804	7.229179071	-2.607175
Orc1	Orc1-001	743.40316	0.000580955	0.003000948	0.164137868	-6.092439302	1.415906067	8.626321773	-2.60702
Btrc	Btrc-202	1093.0374	0.009137637	0.025692806	0.164139786	-6.092368112	1.282688169	7.814608499	-2.607003
Psmd11	Psmd11-003	1432.9585	3.83991E-05	0.000397812	0.164215874	-6.089545294	2.237288918	13.6240722	-2.606335
Hnrnph2	Hnrnph2-005	1311.6081	0.00015388	0.001101434	0.164223966	-6.089245231	2.575982073	15.68578655	-2.606263
Zfp81	Zfp81-001	3455	7.36341E-06	0.000125596	0.164247679	-6.088366103	6.048879014	36.82778995	-2.606055
Cfap97	Cfap97-002	148.37896	0.011275313	0.030283837	0.164296458	-6.086558499	0.230550755	1.403260657	-2.605627
30016B08R	0016B08Rik-	4611	0.000167928	0.001172587	0.164310162	-6.086050856	7.938247676	48.31257906	-2.605506
Slirp	Slirp-001	758.9357	0.006533952	0.019697902	0.16439814	-6.082793876	1.34326323	8.170793352	-2.604734
Auts2	Auts2-006	1081.0383	0.001660094	0.006758378	0.164444737	-6.081070248	2.058193691	12.51602042	-2.604325
Wnk1	Wnk1-017	1880.071	0.000125428	0.000948113	0.164484962	-6.07958313	3.58644465	21.80408839	-2.603972
P23-451E8.	23-451E8.7-(	431	0.002997681	0.010750847	0.164485071	-6.079579104	0.54886984	3.336897612	-2.603971
Gmds	Gmds-002	647.88573	0.006867628	0.020503509	0.164506498	-6.078787216	1.144334257	6.956164451	-2.603784
Pam	Pam-004	924.31154	0.000445138	0.002443267	0.164509876	-6.078662406	1.712159801	10.40764141	-2.603754
Il6st	Il6st-008	592.64599	0.009420669	0.02631579	0.16451303	-6.078545876	1.387906297	8.436452098	-2.603726
Rbm6	Rbm6-010	1353.7644	6.0843E-05	0.000556144	0.164553356	-6.07705625	2.443947366	14.85200562	-2.603373

Tmem170b	`mem170b-20	7625	5.37688E-08	6.57708E-06	0.164555552	-6.076975161	14.32615771	87.05970453	-2.603353
10020H08R	0020H08Rik-	1015.026	4.91677E-06	9.67257E-05	0.164595469	-6.075501383	1.701276278	10.33610638	-2.603003
Btd	Btd-002	565.33396	0.001190934	0.005226071	0.164602426	-6.075244612	0.681110811	4.137914786	-2.602942
Nudt5	Nudt5-001	3203.4307	4.93106E-07	2.31674E-05	0.164648764	-6.073534814	6.276428716	38.12010831	-2.602536
Erg	Erg-003	42282.763	1.1698E-05	0.000172001	0.164657913	-6.073197334	69.57265041	422.528435	-2.602456
Tatdn3	Tatdn3-004	175.67256	0.044288257	0.08910202	0.164678104	-6.072452723	0.210640913	1.279106987	-2.602279
P23-297I15	23-297I15.1-(	301	0.000413193	0.002305615	0.164695481	-6.07181202	0.494419973	3.002025135	-2.602127
Ubn2	Ubn2-006	4540.9575	3.70888E-06	8.05433E-05	0.164704015	-6.071497397	7.08409863	43.01108639	-2.602052
)30443J06R	0443J06Rik-(	213.20374	0.001142519	0.005062262	0.164707036	-6.071386058	0.350965686	2.130848174	-2.602026
WI1-49P9.2	I1-49P9.2-0(	6168	5.21044E-06	0.000100145	0.164770242	-6.069057048	13.01274469	78.97508986	-2.601472
'23-454A14	3-454A14.10-	6565.9506	0.026520739	0.059178793	0.164778346	-6.06875858	6.440103427	39.08343293	-2.601401
Haus3	Haus3-003	1262.2897	0.040756605	0.083363018	0.164831318	-6.066808267	2.150526188	13.04683006	-2.600938
RP23-86H7.	'23-86H7.3-0	416	0.029499623	0.064422337	0.164876678	-6.065139167	0.653249446	3.962048802	-2.600541
Specc1	Specc1-001	4175.8637	3.96841E-05	0.000407865	0.164940515	-6.062791793	4.00591068	24.28700239	-2.599982
Hnrnpk	Hnrnpk-001	2097.3195	0.001413707	0.005976976	0.164973765	-6.061569845	3.879686988	23.51699365	-2.599691
)30056L22R	0056L22Rik-	1010.3823	0.046249376	0.092224099	0.164984451	-6.061177238	2.048254389	12.41483288	-2.599598
Atg9b	Atg9b-001	1131.1806	0.000998248	0.004546541	0.165002151	-6.060527042	1.238899482	7.508383811	-2.599443
Emc8	Emc8-001	4438.2287	1.0333E-07	9.64322E-06	0.165015953	-6.060020145	7.865027308	47.66222393	-2.599323
Gm12191	Gm12191-001	1404	1.31065E-06	4.13349E-05	0.16503518	-6.059314128	2.808295354	17.01634371	-2.599154
Tma7	Tma7-003	879.05754	0.001449351	0.006098314	0.165074525	-6.057869936	1.663698836	10.07847116	-2.598811
Tbc1d14	Tbc1d14-201	1677.6333	0.003009557	0.01078666	0.165075149	-6.057847022	3.140313147	19.02353665	-2.598805
Irak1	Irak1-004	4141.9707	2.20034E-05	0.000270113	0.165135346	-6.055638748	7.443300188	45.07393703	-2.598279
Gm37893	Gm37893-001	1305	1.22851E-05	0.000178441	0.165203438	-6.053142805	2.477202895	14.99486288	-2.597684
Tpd52l2	Tpd52l2-003	2229.7834	0.011356377	0.03046334	0.165227295	-6.052268789	4.1431517	25.07546772	-2.597476
Elmo1	Elmo1-002	5175.5389	3.7135E-06	8.05433E-05	0.165256063	-6.051215203	8.49955163	51.43261604	-2.597225
Gm3331	Gm3331-001	310	0.007159541	0.02119947	0.165256108	-6.051213553	0.517132471	3.129279019	-2.597224
Tctex1d2	Tctex1d2-003	401.55157	0.004963818	0.01591414	0.165263542	-6.050941349	0.772116994	4.672034646	-2.59716
Gm37137	Gm37137-001	605	0.003641156	0.012479154	0.165263944	-6.050926619	1.12513873	6.808131888	-2.597156
Gm11824	Gm11824-001	906	5.02332E-05	0.000482335	0.165280085	-6.050335691	1.960412894	11.8611561	-2.597015
Fam168a	Fam168a-201	4690.4268	0.001747482	0.007045469	0.165302322	-6.049521809	6.912793289	41.81909376	-2.596821
Srsf1	Srsf1-002	5458.1451	1.9117E-05	0.000244221	0.165368743	-6.047091979	10.92570803	66.06876141	-2.596242
Pcmtd1	Pcmtd1-004	3036.976	1.07151E-05	0.000162458	0.165404907	-6.04576986	5.43116324	32.83556302	-2.595926
Atp5j	Atp5j-012	4679.2263	7.46668E-05	0.000647591	0.165410132	-6.045578878	8.096207829	48.94626304	-2.59588
Ints12	Ints12-002	482.72297	0.005888484	0.01817761	0.165472845	-6.043287639	0.716614163	4.330705511	-2.595334
Gm26781	Gm26781-001	185.25683	0.005930768	0.018276931	0.165476802	-6.043143126	0.303894293	1.836476705	-2.595299
'24-370B22	24-370B22.3-	12130	3.81744E-06	8.16753E-05	0.16549667	-6.042417662	11.69136455	70.64410764	-2.595126
Tlk1	Tlk1-003	706.08747	0.00616578	0.018848444	0.16553421	-6.041047355	1.356724575	8.196037407	-2.594799
Aste1	Aste1-002	833.31386	0.000611593	0.003123163	0.165609067	-6.038316743	1.267458245	7.653314345	-2.594146
10403A07R	0403A07Rik-	2256.9402	1.247E-05	0.000180361	0.165683207	-6.035614686	3.951622159	23.85046874	-2.593501
Gtf2i	Gtf2i-006	158.78756	0.030797237	0.066755607	0.165717331	-6.034371874	0.341250483	2.059232319	-2.593204
Lrrc8c	Lrrc8c-003	326.30736	0.002243528	0.008575102	0.16575047	-6.033165379	0.61901456	3.734617215	-2.592915
Cecr2	Cecr2-003	314.36303	0.005492113	0.017241325	0.165751986	-6.033110227	0.684127433	4.127416215	-2.592902
Pds5b	Pds5b-201	2107.5721	0.015617499	0.039084659	0.165820668	-6.030611341	3.832098533	23.10989687	-2.592304
Gm15530	Gm15530-001	2362	2.57235E-05	0.000301516	0.165896852	-6.027841931	3.553771163	21.42157083	-2.591642
Zfp945	Zfp945-201	414.58411	0.003588508	0.012339999	0.165932064	-6.026562777	0.551469676	3.323466624	-2.591335
Cops5	Cops5-006	496.81639	0.021534797	0.050345856	0.165993992	-6.024314427	1.004745117	6.052900503	-2.590797
Commd8	Commd8-001	1455.6778	0.000537228	0.002826212	0.16600818	-6.023799547	2.578483153	15.53226565	-2.590674
Nudt12	Nudt12-003	778.35821	0.019579353	0.046727951	0.166051125	-6.022241632	1.022861799	6.159920908	-2.590301
Clp1	Clp1-201	659.63666	0.001246665	0.005421761	0.166092585	-6.020738389	0.855562844	5.151120062	-2.58994
Trp53	Trp53-001	3706.2646	0.012580297	0.032988941	0.166127447	-6.019474929	9.266382316	55.77875604	-2.589638
Glt8d1	Glt8d1-202	2554.1784	0.000178744	0.001226115	0.166140985	-6.018984402	3.48843859	20.99685746	-2.58952
Gbp4	Gbp4-002	5213.1016	0.000658019	0.003302146	0.166194307	-6.017053278	4.912264169	29.55735522	-2.589057
30520O04R	0520O04Rik-	706	0.000756988	0.003670278	0.166194757	-6.017036997	1.365004661	8.213283547	-2.589053
Tpp2	Tpp2-008	209.5758	0.028548202	0.062739322	0.16619557	-6.017007558	0.442479144	2.662400352	-2.589046
Mrpl24	Mrpl24-006	2710.3528	0.000103538	0.000818792	0.166211578	-6.016428044	5.113798732	30.76680211	-2.588907
Lrrc57	Lrrc57-004	1138.2978	0.012500308	0.03282493	0.166231522	-6.015706197	1.551405066	9.33279707	-2.588734
Elk4	Elk4-003	1761.6184	0.000300003	0.001811107	0.166261275	-6.014629669	2.984255312	17.94919054	-2.588476
Lsm8	Lsm8-002	740.84609	0.000336723	0.001973776	0.166264592	-6.014509684	1.149773342	6.915322902	-2.588447
Agbl2	Agbl2-202	2963.9952	1.26777E-07	1.09317E-05	0.166310314	-6.012856181	5.258169033	31.61661417	-2.58805
P23-10D15	23-10D15.2-(	367	0.000755329	0.003665042	0.166349913	-6.011424834	0.83881155	5.042452581	-2.587707
Eny2	Eny2-201	5288.9008	9.73195E-08	9.3629E-06	0.16636718	-6.010800936	10.14029825	60.95131419	-2.587557
Fgfr1op	Fgfr1op-002	1951.8852	1.83231E-05	0.000237233	0.166411893	-6.009185908	3.737154926	22.45725872	-2.58717
Pam16	Pam16-002	213.8973	0.021610895	0.050475529	0.166454999	-6.007629734	0.288551847	1.733512657	-2.586796
Rnf10	Rnf10-010	1949.7613	0.000225125	0.001457103	0.166478361	-6.006786655	2.492641716	14.97276699	-2.586593
Hacd2	Hacd2-201	3306	5.07354E-07	2.34487E-05	0.166503031	-6.005896661	5.444269096	32.69771759	-2.58638
Clcf1	Clcf1-003	2110.5807	9.88343E-05	0.000790767	0.166561466	-6.003789609	4.761164826	28.58503191	-2.585873
Siah1b	Siah1b-002	230.04489	0.01373955	0.035381925	0.166567409	-6.003575393	0.413282178	2.481170712	-2.585822
Gm13212	Gm13212-201	207.26604	0.023891207	0.054522511	0.166583493	-6.002995736	0.222375224	1.33491752	-2.585683
Rpf1	Rpf1-003	629.12845	0.00195962	0.007715482	0.166593798	-6.002624405	1.296257003	7.780943922	-2.585593
Cggbp1	Cggbp1-001	9282.5803	2.13017E-07	1.47864E-05	0.166736682	-5.997480497	16.47666892	98.81850049	-2.584357
Gripap1	Gripap1-007	742.2099	0.003654271	0.012515877	0.166736943	-5.997471105	1.391906851	8.347921119	-2.584354
Mbnl1	Mbnl1-001	36820.686	7.84634E-08	8.35206E-06	0.166802539	-5.995112587	53.15559786	318.6737938	-2.583787
Frat2	Frat2-201	781	4.29302E-05	0.000432204	0.166831049	-5.994088055	1.923619141	11.53034252	-2.58354
Lcorl	Lcorl-002	757.19214	0.015994606	0.039794802	0.16694174	-5.990113679	1.276753445	7.647898277	-2.582583
'23-106G24	23-106G24.2-	384	0.003456995	0.011984016	0.166952171	-5.989739431	0.771562561	4.621458696	-2.582493
Fnbp1l	Fnbp1l-003	521.91062	0.005594635	0.017469999	0.166959249	-5.989485486	0.782020368	4.683899643	-2.582432
Gramd1b	Gramd1b-011	8212.647	5.75228E-05	0.000533773	0.166969549	-5.989116019	16.46001344	98.58093017	-2.582343
Trpv2	Trpv2-001	1224.6512	3.56715E-05	0.000377688	0.166989895	-5.988386297	2.480076275	14.85165478	-2.582167
Hspd1	Hspd1-001	16423.267	4.16193E-05	0.000422109	0.166990593	-5.988361286	38.23584241	228.9700384	-2.582161
Gm13270	Gm13270-002	1117	4.14983E-05	0.000421131	0.167012864	-5.987562738	1.83024422	10.9587021	-2.581969
Kdm5d	Kdm5d-005	1554.4766	4.82665E-05	0.000469105	0.167031897	-5.98688046	2.751469467	16.47271879	-2.581804
Rbm26	Rbm26-004	1989.7187	4.55717E-06	9.20843E-05	0.167066633	-5.985635686	3.79383681	22.708525	-2.581504
Lsm2	Lsm2-002	806.70957	0.000129406	0.000968992	0.16707406	-5.985369602	1.575523146	9.430088344	-2.58144
Sat1	Sat1-006	3976.0053	2.91235E-05	0.000328659	0.167096164	-5.984577833	9.090102055	54.40042326	-2.581249
Fnip2	Fnip2-001	9928.2485	1.54322E-06	4.55256E-05	0.167124497	-5.983563266	17.94852959	107.3961623	-2.581005
Sec24b	Sec24b-002	1707.4496	0.003670177	0.012554037	0.167130531	-5.98334722	2.77496256	16.60356452	-2.580953
Ric1	Ric1-002	548.34334	0.005488952	0.017236325	0.167174006	-5.981791215	0.965439272	5.775056157	-2.580578
Phip	Phip-006	352	0.00120365	0.005267698	0.167184461	-5.981417145	0.60768868	3.63483949	-2.580487
Tspyl2	Tspyl2-005	541.08769	0.014264052	0.036431303	0.167228187	-5.979853132	1.081705755	6.468441544	-2.58011
Sncaip	Sncaip-003	2109.6944	0.026705022	0.059500846	0.167230876	-5.979756977	4.197377616	25.09929808	-2.580087
Tmtc3	Tmtc3-001	13348.265	2.28478E-09	1.34808E-06	0.167252481	-5.978984551	22.43764583	134.1543378	-2.5799
Mef2a	Mef2a-002	11997.613	5.42961E-07	2.43727E-05	0.167273522	-5.978232477	21.41133498	128.0019382	-2.579719
Larp4b	Larp4b-005	3304.0909	1.87371E-06	5.14631E-05	0.167280748	-5.977974225	5.191657557	31.03559506	-2.579657
Skp2	Skp2-001	800.24399	0.005531952	0.0173348	0.167285527	-5.977803454	1.780651935	10.64438729	-2.579615
Gm37685	Gm37685-001	1038	1.04002E-05	0.00015878	0.167332903	-5.976110973	1.690808536	10.10445945	-2.579207
Frmd4b	Frmd4b-013	1903.705	1.36906E-06	4.26277E-05	0.16735624	-5.975277657	3.22912503	19.29491865	-2.579006
Pdgfa	Pdgfa-001	3649.3453	0.000252383	0.001586625	0.167372924	-5.974682006	5.712251385	34.12888557	-2.578862
Dopey1	Dopey1-002	733.0713	0.003820871	0.012955527	0.167419591	-5.973016624	1.082195065	6.463969112	-2.57846
Xiap	Xiap-006	789.95728	5.47151E-05	0.000514692	0.167448691	-5.97197861	1.265933686	7.560128896	-2.578209
Madd	Madd-001	127.85777	0.02781299	0.061488063	0.167468561	-5.971270022	0.226874092	1.354726466	-2.578038
Stard7	Stard7-002	2973.0599	0.007555563	0.022130064	0.167472832	-5.971117758	5.702146532	34.04818842	-2.578001
Supt20	Supt20-202	1541.3834	0.000123589	0.000937525	0.167503146	-5.970037126	2.342502759	13.98482844	-2.57774
Lst1	Lst1-001	280	0.042439458	0.086100453	0.167554207	-5.968217813	0.791806894	4.725676008	-2.5773
Hnrnpc	Hnrnpc-202	4527.1435	7.75754E-05	0.000665071	0.167600226	-5.966579081	9.092141549	54.24898157	-2.576904
Fgfr1op2	Fgfr1op2-003	1140.7725	4.58087E-05	0.000452538	0.167715907	-5.962463651	2.000603427	11.92852522	-2.575909
Tinagl1	Tinagl1-005	8090.5054	5.41337E-05	0.000510766	0.167759027	-5.960931095	11.77679502	70.20066365	-2.575538
Ubl4	Ubl4-002	2599.345	0.000594358	0.003052444	0.167771667	-5.96048201	4.144856102	24.70534023	-2.575429

Snapin	Snapin-004	797.54747	0.002161421	0.00831626	0.167775899	-5.960331629	0.937238562	5.586252648	-2.575393
Ash2l	Ash2l-007	264.44052	0.000471646	0.002556395	0.167818797	-5.958808043	0.409373066	2.439375515	-2.575024
Zfp951	Zfp951-001	790.80532	4.16641E-05	0.000422313	0.167835609	-5.958211152	1.427231699	8.503747823	-2.574879
Gm7609	Gm7609-003	1370.038	1.82343E-06	5.05688E-05	0.167850584	-5.957679603	2.308737959	13.75472105	-2.574751
Brpf1	Brpf1-005	714.18552	0.00677547	0.020284951	0.167863915	-5.957206451	1.217572347	7.253329842	-2.574636
Dcdc2c	Dcdc2c-001	2117	9.28935E-06	0.000146888	0.167865173	-5.95716181	3.75896749	22.39277758	-2.574625
Slfn8	Slfn8-001	1084.7097	0.046431741	0.092501579	0.167870767	-5.956963326	2.273257302	13.54171038	-2.574577
Gm9294	Gm9294-001	1449	9.26505E-06	0.00014664	0.167873961	-5.956849962	2.904299867	17.30047855	-2.57455
P24-364E17	24-364E17.1-(	217	0.003614546	0.012405871	0.167965984	-5.953586402	0.399885007	2.380749939	-2.573759
Kctd3	Kctd3-007	125.38666	0.020506084	0.048446808	0.167967312	-5.953539354	0.269460302	1.604242511	-2.573748
Anapc5	Anapc5-019	491.39962	0.034803995	0.073592258	0.167968141	-5.953509962	0.937570551	5.581835615	-2.57374
Haus6	Haus6-001	3401.1817	4.10262E-06	8.57454E-05	0.167968684	-5.953490707	7.374199248	43.9022267	-2.573736
Hprt	Hprt-001	6389	2.77966E-08	4.38726E-06	0.167995699	-5.952533347	10.3628398	61.6851495	-2.573504
Gbp5	Gbp5-002	795.05192	0.032492543	0.069641401	0.168109132	-5.948516835	1.175175441	6.990550895	-2.57253
BC037034	3C037034-002	845.82067	0.019457752	0.046483087	0.168115644	-5.948286407	1.605935856	9.552566421	-2.572474
Dhx36	Dhx36-004	540.66833	0.006005115	0.018466054	0.168136888	-5.947534856	1.171739063	6.968958918	-2.572292
Zfp455	Zfp455-001	356.7009	0.043894856	0.088460837	0.168213748	-5.944817294	0.681570429	4.05181167	-2.571632
Rbm39	Rbm39-026	639.37323	0.001617866	0.006624257	0.168239034	-5.943923823	1.266783991	7.529667544	-2.571416
Gm26876	Gm26876-201	692	5.16355E-05	0.00049276	0.168272465	-5.942742935	1.092459504	6.492205997	-2.571129
Gm21781	Gm21781-001	5992	8.22373E-08	8.50945E-06	0.168308633	-5.94146587	10.16541187	60.39744769	-2.570819
Nxpe2	Nxpe2-005	199.9038	0.024447042	0.055509891	0.168309511	-5.941434901	0.173446939	1.030523697	-2.570811
Med6	Med6-001	837.97591	0.007107068	0.021087852	0.168350112	-5.940002002	1.646331185	9.779210536	-2.570463
Usp16	Usp16-003	630.6673	0.001546376	0.006398087	0.168376736	-5.939062757	1.280597694	7.605550069	-2.570235
Snx5	Snx5-004	936.97068	0.000191387	0.001289086	0.168426606	-5.937304224	2.208918969	13.11502393	-2.569808
Lrrc17	Lrrc17-001	715.20629	0.003693293	0.01262302	0.16845468	-5.936314731	1.689216901	10.02772317	-2.569568
Abcc5	Abcc5-005	1822.0605	0.010056216	0.027717403	0.168516017	-5.934154015	2.742891228	16.276739	-2.569042
Fhl3	Fhl3-001	870.64764	0.000888479	0.00416114	0.168538829	-5.933350813	2.149865796	12.75590797	-2.568847
Pde4b	Pde4b-001	6546.4323	0.000863623	0.004066974	0.16856641	-5.932380019	16.97466333	100.7001535	-2.568611
Map2k4	Map2k4-006	365.373	0.001524407	0.006329368	0.168596231	-5.931330681	0.653842955	3.878158777	-2.568356
Madd	Madd-007	1100.0119	0.002138187	0.008242309	0.16859893	-5.931235756	1.740776104	10.32495347	-2.568333
Mapkap1	Mapkap1-201	362.57316	0.010761096	0.029177291	0.168604092	-5.931054141	0.372050384	2.206650968	-2.568289
Plek	Plek-001	3152.1395	0.020638423	0.04868223	0.168618001	-5.930564915	10.13967219	60.13398414	-2.56817
Atp5g1	Atp5g1-001	1101.2878	0.000143673	0.00104736	0.168679421	-5.92840547	1.655925137	9.816995639	-2.567644
Sgce	Sgce-002	2392.2591	0.007931322	0.022976214	0.168692044	-5.927961849	4.247794513	25.18076381	-2.567536
Itpkb	Itpkb-002	12335.58	1.50594E-05	0.000206067	0.168729067	-5.926661114	21.10481406	125.0810808	-2.56722
Plcd4	Plcd4-008	1432	3.98195E-06	8.38756E-05	0.16873089	-5.92659709	2.617529122	15.51304047	-2.567204
Gm10293	Gm10293-001	618	0.000232176	0.001490817	0.168748017	-5.925995562	1.317243857	7.805981248	-2.567058
Tmem251	Tmem251-201	1517	2.72542E-05	0.000313978	0.168763575	-5.925449244	2.812301474	16.66414964	-2.566925
P23-231L15	23-231L15.4-	1962	0.002674931	0.009822705	0.168813096	-5.923711045	3.327311051	19.71002922	-2.566501
Phkg1	Phkg1-006	3661.939	3.57584E-07	1.90767E-05	0.168880798	-5.921336312	6.335326576	37.5135993	-2.565923
Cntrl	Cntrl-201	3563.8203	0.000110986	0.000861969	0.168950628	-5.918888926	6.749860043	39.95167186	-2.565326
P24-62O16	24-62O16.3-(	408	0.00052186	0.002760299	0.169005797	-5.916956794	0.783161968	4.633935529	-2.564855
Hnrnpa3	Hnrnpa3-003	14940.852	0.000689302	0.003423025	0.169055239	-5.915226328	33.15060943	196.0933577	-2.564433
Gm2308	Gm2308-001	95	0.049022834	0.0965738	0.16905689	-5.915168563	0.186282483	1.101892286	-2.564419
Rb1cc1	Rb1cc1-004	2209.2815	2.33244E-05	0.000281289	0.169091146	-5.913970209	3.810679792	22.53624677	-2.564127
Ncl	Ncl-001	16565.912	3.44276E-07	1.88049E-05	0.169122016	-5.912890718	37.37587343	220.9994551	-2.563864
Tk1	Tk1-006	466.29202	0.049233772	0.096877897	0.169203504	-5.910043093	0.964362445	5.699423607	-2.563169
10403D21R	0403D21Rik-	1176.6917	0.000129595	0.00096998	0.169231817	-5.909054322	1.813389074	10.71541455	-2.562927
Camk2d	Camk2d-017	2105.3625	0.000267064	0.00165346	0.169234512	-5.908960241	3.624096406	21.41464157	-2.562904
Azi2	Azi2-001	4590.5151	1.98541E-08	3.56435E-06	0.169234692	-5.908953947	8.924907949	52.73687005	-2.562903
Gabbr1	Gabbr1-001	2486.3678	0.000179589	0.001320474	0.169240219	-5.908760978	4.189788133	24.75645663	-2.562856
10011F06R	0011F06Rik-	233.93927	0.041892985	0.085234332	0.169241017	-5.90873312	0.538676675	3.18289671	-2.562849
Myo10	Myo10-001	5037.2094	0.023382537	0.053629672	0.169325225	-5.905794617	14.59232645	86.17928298	-2.562131
Tcirg1	Tcirg1-009	1304.5374	6.54736E-06	0.00011613	0.169369197	-5.904261321	2.20119307	12.99641911	-2.561757
10037L13R	0037L13Rik-	4646.149	1.51761E-06	4.50505E-05	0.169394615	-5.903375393	6.873001052	40.57390529	-2.56154
Yif1b	Yif1b-001	469.42867	0.039219707	0.08081794	0.169396172	-5.903321121	0.753085891	4.445707845	-2.561527
Gatad2b	Gatad2b-004	21153.751	2.30304E-08	3.82908E-06	0.169421388	-5.902442495	38.51592662	227.338042	-2.561312
Gm37216	Gm37216-001	233	0.008468017	0.024159065	0.169444041	-5.901653411	0.520707332	3.073034202	-2.561119
Vwa1	Vwa1-001	957.91488	0.00056808	0.00295336	0.169511099	-5.899318737	1.772851653	10.45861698	-2.560548
Rpl36a	Rpl36a-006	77.571262	0.02333263	0.053547479	0.169564816	-5.897449841	0.204590168	1.206560251	-2.560091
Gnai2	Gnai2-004	931.93307	0.000403262	0.002264586	0.169636799	-5.894947342	1.306908058	7.704154182	-2.559479
Apol7e	Apol7e-201	330	0.025297799	0.057022022	0.169659063	-5.894173775	0.702237328	4.139108842	-2.55929
330462I19R	0462I19Rik-(	1516.8158	3.0731E-06	7.1863E-05	0.169711858	-5.892340195	2.595192668	15.29175807	-2.558841
Dynlt3	Dynlt3-001	18447.969	6.53968E-09	2.13318E-06	0.169732307	-5.891630283	25.89456331	152.5611933	-2.558667
Zbtb11	Zbtb11-004	365.42737	0.034588749	0.07326312	0.169838302	-5.887953338	0.674861883	3.973555279	-2.557766
Slc25a36	Slc25a36-005	895.5748	0.000161067	0.001137668	0.169841841	-5.887830672	1.608251697	9.469113668	-2.557736
Csnk1a1	Csnk1a1-006	1357.5029	2.22408E-05	0.000272178	0.169846906	-5.887655086	2.257837894	13.29337076	-2.557693
Phkb	Phkb-003	682.33771	0.000821699	0.003910404	0.169856724	-5.887314764	1.216120425	7.159683733	-2.55761
Ercc8	Ercc8-007	692.64227	0.000414244	0.002311101	0.169881314	-5.886462581	1.567140997	9.224916838	-2.557401
Rbm17	Rbm17-006	149.61397	0.040180584	0.082430967	0.16989382	-5.886029276	0.237954556	1.400607486	-2.557295
Qtrtd1	Qtrtd1-002	838.97933	0.022471863	0.052018202	0.16989777	-5.885892442	1.804293272	10.61987613	-2.557261
Tbl1xr1	Tbl1xr1-004	5574.9262	3.00704E-05	0.000335767	0.169919331	-5.885145574	9.824241497	57.81709137	-2.557078
Ptgr2	Ptgr2-001	2794.682	1.49353E-05	0.000204609	0.169992873	-5.882599565	4.252851804	25.01782417	-2.556454
P24-155C13	24-155C13.6-	221.67485	0.001305192	0.005615933	0.170008259	-5.882067161	0.394949887	2.323121758	-2.556323
Tmem214	Tmem214-001	2225.9502	1.33284E-06	4.18422E-05	0.170024083	-5.881519723	3.827646763	22.51237993	-2.556189
Yme1l1	Yme1l1-002	2962.2706	5.55856E-05	0.000520593	0.170040326	-5.880957919	5.292704305	31.12617129	-2.556051
Muc6	Muc6-002	2212.1382	0.001170134	0.005152623	0.170079117	-5.879616604	3.347856869	19.68411484	-2.555722
D5Ertd579e	5Ertd579e-00	1027.121	0.000664551	0.003328586	0.170079734	-5.87959526	1.791325208	10.5322672	-2.555717
Rpa3	Rpa3-001	745.33968	0.000642996	0.00324468	0.170083977	-5.879448591	1.772337956	10.4203699	-2.555681
Strn4	Strn4-009	477.11099	0.0154376	0.038724023	0.170153562	-5.877044176	0.79786069	4.689062523	-2.555091
Elf1	Elf1-202	8709.5451	8.84565E-05	0.000728452	0.170193785	-5.875655206	16.45360711	96.67572229	-2.55475
Ptpn1	Ptpn1-004	281.21264	0.001228693	0.005360858	0.170195046	-5.875611686	0.449557842	2.641427311	-2.554739
Herc1	Herc1-004	1437.3655	2.14836E-05	0.000265984	0.170207605	-5.875178147	2.428959329	14.27056877	-2.554633
Dusp18	Dusp18-201	1185.8813	0.000191604	0.001289787	0.170225181	-5.874571532	2.037370861	11.96868086	-2.554484
33439K11R	33439K11Rik-	109.45448	0.01420745	0.036324662	0.170229819	-5.874411451	0.220716476	1.296579394	-2.554444
Sp100	Sp100-012	9677.7579	2.27745E-05	0.000276623	0.170244138	-5.87391739	13.93133508	81.83151137	-2.554323
Gm13140	Gm13140-001	301	0.000896198	0.004192704	0.170249747	-5.873723862	0.639370993	3.75548866	-2.554275
Tpd52	Tpd52-001	2123.5072	0.000370585	0.002118955	0.170266183	-5.873156863	5.251535424	30.84309131	-2.554136
Mprip	Mprip-001	7538.2002	9.22855E-08	9.0827E-06	0.170275699	-5.87282638	14.90069215	87.5092116	-2.554056
RP23-48L9.	P23-48L9.1-0	538	3.41536E-05	0.000365779	0.170332827	-5.870858947	0.806616934	4.735534242	-2.553572
Ino80	Ino80-002	1004.5842	0.021200247	0.049719835	0.170377365	-5.869324239	1.827295105	10.72498745	-2.553194
Atp6v1h	Atp6v1h-002	775.33067	3.20042E-05	0.000350653	0.170470158	-5.866129352	1.389492651	8.150943624	-2.552409
Ddr2	Ddr2-202	3956.1739	0.00017502	0.001207339	0.170571247	-5.862652811	5.794875609	33.97334378	-2.551554
P23-110C17	23-110C17.2-	369.26608	0.004922335	0.015804816	0.170580038	-5.862350673	0.784506597	4.599052776	-2.551479
Hk1	Hk1-016	893.75815	0.049403368	0.097100044	0.170586719	-5.862121083	1.422495987	8.338843717	-2.551423
33426K07R	33426K07Rik-	515	0.006464998	0.019543353	0.170620015	-5.860977109	0.968601984	5.676954054	-2.551141
Mtx3	Mtx3-201	4138	4.10914E-06	8.5816E-05	0.170623904	-5.860843523	8.234474552	48.26096684	-2.551108
Tnfsf10	Tnfsf10-001	16692.478	0.005495989	0.017248936	0.170633679	-5.860507776	32.37055709	189.7079015	-2.551026
Mprip	Mprip-007	922.86371	0.001463945	0.006142105	0.170659483	-5.859621629	1.902515457	11.14802072	-2.550808
Smpd4	Smpd4-006	1365.2899	0.0138547	0.03560083	0.17068091	-5.858886044	2.128774829	12.47224914	-2.550626
Npc1	Npc1-003	759.1862	0.042949251	0.086939234	0.170709582	-5.857902003	1.23186621	7.216151539	-2.550384
Mgst2	Mgst2-002	192.07367	0.00849672	0.024220779	0.170776901	-5.855592861	0.446174558	2.61261656	-2.549815
Dpp4	Dpp4-001	254.28357	0.008034537	0.023202603	0.170834155	-5.853630375	0.58278841	3.411427938	-2.549332
Trappc6b	Trappc6b-201	8226	4.94058E-09	1.8062E-06	0.170868627	-5.852449421	13.48774027	78.93631774	-2.549041
Gad2	Gad2-001	562	4.91955E-05	0.000474874	0.170912658	-5.850941698	1.094941351	6.406438007	-2.548669
Ibtk	Ibtk-002	1800.8128	7.13336E-05	0.000626134	0.170916472	-5.850811157	3.760075021	21.99948889	-2.548637
Yod1	Yod1-001	5547.2966	1.63879E-07	1.26693E-05	0.170945353	-5.849822679	9.43856835	55.21395119	-2.548393

Zfp60	Zfp60-003	433.03815	0.000898081	0.004198072	0.170945829	-5.849806365	0.641605511	3.753268004	-2.548389
Gm4787	Gm4787-001	732	0.00126436	0.005476664	0.171012089	-5.847539833	1.186925732	6.940595498	-2.54783
Zfp408	Zfp408-001	2493.8105	0.002587527	0.009570498	0.171039656	-5.846597337	5.204471898	30.42845154	-2.547597
Gm10343	Gm10343-201	286	0.000958372	0.004402392	0.171040483	-5.846569073	0.593810227	3.47175251	-2.54759
Ssr4	Ssr4-007	634.33183	0.001719561	0.00694598	0.171053862	-5.846111807	1.06101177	6.202793436	-2.547477
P23-164N7	23-164N7.3-(	2715	0.000106292	0.000833258	0.171158616	-5.842533814	4.346124424	25.39237891	-2.546594
Vps29	Vps29-009	1483.2561	5.86858E-05	0.00054163	0.171173921	-5.842011405	2.336579867	13.65032623	-2.546465
Gm15276	Gm15276-001	176	0.01210901	0.032040605	0.171196893	-5.84122751	0.333648318	1.948915736	-2.546272
Gm9432	Gm9432-001	136	0.013619273	0.035117074	0.17120182	-5.841059417	0.332568986	1.94255521	-2.54623
Usp14	Usp14-004	421.45746	0.001657112	0.006752659	0.171203229	-5.841011317	0.709151849	4.142163977	-2.546218
Rrbp1	Rrbp1-001	1237.9514	0.004405604	0.014486553	0.171258395	-5.839129807	2.581222517	15.07209334	-2.545753
Xk	Xk-001	461	0.003052264	0.010900943	0.171261868	-5.839011411	1.262321355	7.370708796	-2.545724
30415B17R	0415B17Rik-	180	0.0110763	0.029859369	0.171270579	-5.838714407	0.292740039	1.709225485	-2.545651
42436	March7-002	2514.0125	7.69483E-06	0.000129635	0.17127567	-5.83854089	4.316287312	25.20081997	-2.545608
Pgam1	Pgam1-201	13708	3.81448E-06	8.16753E-05	0.171320831	-5.837001795	27.84030868	162.5039317	-2.545228
P23-424O22	23-424O22.1-	191	0.003351394	0.011710391	0.171410371	-5.833952733	0.42771065	2.495243718	-2.544474
Mob3b	Mob3b-001	450.36764	0.0056967	0.017714907	0.171423406	-5.833509097	1.000093774	5.834056131	-2.544364
Sdcbp	Sdcbp-002	14216.689	8.87392E-06	0.000142691	0.17144832	-5.8326614	28.43144172	165.8309726	-2.544154
Tgs1	Tgs1-001	9061.0573	4.33751E-08	5.71384E-06	0.171464775	-5.832101659	17.52478704	102.2063396	-2.544016
00063D05R	0063D05Rik-	187	0.018718526	0.045008228	0.171537567	-5.829626808	0.354337922	2.06565785	-2.543404
Skil	Skil-001	18362.113	4.19206E-06	8.7193E-05	0.171538812	-5.829584511	31.60025461	184.2163548	-2.543393
Gfm2	Gfm2-006	1065.2078	0.000102181	0.000810397	0.171543155	-5.829436906	1.820189231	10.61067828	-2.543357
Ly6e	Ly6e-002	43344.344	5.03175E-06	9.8366E-05	0.171575579	-5.828335292	74.38162865	433.5210714	-2.543084
Hs2st1	Hs2st1-003	519.03446	0.036872576	0.077075724	0.171582831	-5.828088933	1.014941969	5.915172057	-2.543023
Gm37924	Gm37924-001	222	0.006667359	0.020017271	0.171602558	-5.827418967	0.454537675	2.648781471	-2.542857
Mllt3	Mllt3-001	7224.7356	2.52836E-06	6.32551E-05	0.171628653	-5.826532956	14.77196846	86.06936105	-2.542638
Trps1	Trps1-001	5998.4577	1.00718E-05	0.000155177	0.171630945	-5.826455146	10.29078111	59.95877458	-2.542618
Rfwd2	Rfwd2-001	6185.5933	1.64789E-08	3.19391E-06	0.171662736	-5.825376109	11.18186597	65.1385749	-2.542351
Slco5a1	Slco5a1-001	275.70717	0.018582221	0.04483558	0.171727174	-5.823190211	0.481264581	2.802495195	-2.54181
Znhit6	Znhit6-002	1222.2516	0.005814442	0.017994455	0.171754216	-5.822273368	2.628240013	15.30233183	-2.541583
Phlpp1	Phlpp1-002	381.48983	0.027838702	0.061518521	0.171809749	-5.820391477	0.666146953	3.877236046	-2.541116
Hdac4	Hdac4-008	345.7548	0.006734054	0.020182406	0.171836778	-5.81947597	0.548397206	3.191384364	-2.540889
Dync1i2	Dync1i2-005	1941.0999	0.000250554	0.001577767	0.171857307	-5.818780814	2.25824576	13.1402371	-2.540717
Nsfl1c	Nsfl1c-004	812.12313	0.001819596	0.007266927	0.17188186	-5.817949595	1.374592016	7.997307062	-2.540511
Kdm5d	Kdm5d-009	199.21912	0.002969992	0.01066718	0.171894162	-5.817533247	0.422304214	2.456768806	-2.540408
Cybb	Cybb-001	3836.8395	0.017438528	0.042607333	0.171896036	-5.817469799	12.48964692	72.65814378	-2.540392
Pls3	Pls3-006	565	0.000319744	0.001897305	0.171908928	-5.817033526	1.208883788	7.032117522	-2.540284
Tes	Tes-005	375.13472	0.011571148	0.030932862	0.171916265	-5.816785298	0.698348691	4.062144398	-2.540222
Vopp1	Vopp1-001	717.4607	0.001382949	0.00587376	0.17193017	-5.816314848	1.379314491	8.022527356	-2.540105
Dynlrb1	Dynlrb1-004	1306.4879	0.000226354	0.001463861	0.171936924	-5.816086384	2.201361128	12.80330648	-2.540049
Gm28229	Gm28229-001	683	0.000813093	0.003878241	0.171941168	-5.815942802	1.209976801	7.037155867	-2.540013
Fkbp15	Fkbp15-004	830.35472	0.035370045	0.074506198	0.171953737	-5.815517689	1.518486296	8.830783916	-2.539908
Adnp	Adnp-004	338.20201	0.00230168	0.008746538	0.17195413	-5.815504399	0.594354216	3.456469557	-2.539904
Tmem82	Tmem82-002	874.38849	4.00051E-05	0.000410109	0.171958064	-5.815371372	1.596640528	9.285057616	-2.539871
Ufl1	Ufl1-002	4623.6237	5.11149E-06	9.93015E-05	0.171992711	-5.814199889	8.594798743	49.9718779	-2.539581
Zc3hc1	Zc3hc1-011	400.66756	0.002215459	0.008485739	0.172022931	-5.813178478	0.796955355	4.63284372	-2.539327
Senp3	Senp3-009	117.61467	0.025881462	0.058051085	0.172039794	-5.812608688	0.222401219	1.292731255	-2.539186
Prim2	Prim2-003	1085	0.003942722	0.013271572	0.172118079	-5.809964906	1.729264367	10.04696529	-2.538529
RP23-392I2.	23-392I2.3-0	1329	4.59656E-06	9.23898E-05	0.17214962	-5.80890043	2.170724072	12.60951999	-2.538265
Ttc9c	Ttc9c-001	3474.1943	1.40909E-06	4.32449E-05	0.172166927	-5.808316499	6.737812417	39.13534703	-2.53812
P23-149A1	23-149A1.6-(	1934.4628	4.32661E-07	2.13117E-05	0.172175608	-5.808023624	3.217420297	18.6868531	-2.538047
Ncbp2	Ncbp2-001	3925.5611	4.43728E-07	2.16491E-05	0.172177239	-5.807968609	8.05629222	46.79069232	-2.538034
Rcan1	Rcan1-201	8296.0791	0.0001718	0.001191367	0.172241452	-5.805803354	12.84760599	74.59067395	-2.537496
Adsl	Adsl-001	560.00936	0.004384724	0.014435911	0.172254908	-5.805349827	0.85354922	4.955151816	-2.537383
Mob1b	Mob1b-001	9825.9199	2.24184E-08	3.80114E-06	0.172287375	-5.804255812	18.74037908	108.7739542	-2.537111
Gm37524	Gm37524-001	3190	5.56666E-07	2.4625E-05	0.172316495	-5.80327494	5.666801766	32.88600868	-2.536867
Anxa5	Anxa5-003	3017.8706	4.27181E-06	8.80515E-05	0.172392009	-5.800732923	5.179338093	30.043957	-2.536235
Acadm	Acadm-009	277.94376	0.011320934	0.030380161	0.172401062	-5.800428291	0.453639273	2.631302072	-2.536159
Senp7	Senp7-003	1165.129	0.000150297	0.001081441	0.1724288	-5.799493883	1.886395536	10.94013937	-2.535927
Abi1	Abi1-003	1649.7289	0.014300038	0.036501439	0.172439865	-5.799123075	2.896586283	16.79766035	-2.535835
Srsf6	Srsf6-001	8586.6746	6.4732E-09	2.13318E-06	0.172461926	-5.798381268	17.03168413	98.75619824	-2.53565
Cadps2	Cadps2-009	878.75075	0.032159702	0.069057668	0.172534148	-5.795954097	1.461027956	8.468050965	-2.535046
RP23-76J15.	23-76J15.4-0	4921	3.48303E-07	1.88161E-05	0.172555571	-5.795234503	8.402458172	48.69421551	-2.534867
Ftl1	Ftl1-002	4467.8333	0.016540145	0.040864167	0.172563736	-5.79496032	9.867790859	57.18345648	-2.534799
Pcolce	Pcolce-003	597.4951	0.045103596	0.09032851	0.172564453	-5.794936211	0.957423038	5.548205431	-2.534793
Acp1	Acp1-201	3382.2852	0.000179395	0.001228854	0.172639567	-5.792414906	6.885753002	39.88513833	-2.534165
Dhcr7	Dhcr7-001	594.14876	0.000900316	0.004206796	0.172663561	-5.79160997	1.266920404	7.337508843	-2.533964
Tyr	Tyr-201	2446	3.30849E-07	1.83224E-05	0.172683506	-5.79094103	4.367284956	25.29068964	-2.533798
Mapk8ip3	Mapk8ip3-014	490.57588	0.000173908	0.001201122	0.172686656	-5.790835393	0.873817852	5.060135345	-2.533771
Cetn3	Cetn3-201	9401	2.36883E-08	3.88193E-06	0.172692189	-5.790649857	17.38217139	100.6540683	-2.533725
Mettl8	Mettl8-011	333.24525	0.006323369	0.019197626	0.172712781	-5.789959446	0.707763797	4.097923683	-2.533553
30002G04R	0002G04Rik-	126	0.007985387	0.023094894	0.172731084	-5.789345931	0.228930142	1.325355786	-2.5334
Mfap3	Mfap3-004	527.82016	0.00211429	0.008172264	0.172796671	-5.787148521	0.899365581	5.204762192	-2.532853
P24-465E5	24-465E5.2-(	465	0.002880361	0.010423017	0.172856727	-5.785137899	0.810865524	4.690968874	-2.532351
Gm8113	Gm8113-001	3196	3.2417E-05	0.000353931	0.172885774	-5.784165913	8.20001149	47.43022695	-2.532109
Gm38372	Gm38372-001	6875	1.58099E-06	4.63207E-05	0.172936979	-5.782453264	12.65639151	73.18499242	-2.531682
Sdad1	Sdad1-001	2778.102	7.01259E-05	0.000618618	0.172985883	-5.780818526	6.367094589	36.80701836	-2.531274
30014A18R	0014A18Rik-	572.16823	0.000183471	0.001250521	0.172999486	-5.780363989	1.151052568	6.653502815	-2.53116
Rab11fip3	Rab11fip3-002	2254.7692	0.001373952	0.005840618	0.173100286	-5.776997967	3.38197799	19.53767998	-2.53032
Specc1l	Specc1l-201	8320.5678	0.000525055	0.002773464	0.173151647	-5.77528437	14.20046322	82.01171326	-2.529892
Snap23	Snap23-002	2294.1397	0.005484193	0.017226121	0.173194503	-5.773855289	3.803833894	21.96278645	-2.529535
Rft1	Rft1-003	805.13174	0.027720048	0.06134339	0.173251422	-5.771958394	1.247510739	7.200580082	-2.529061
Npepps	Npepps-006	207.52027	0.006632578	0.019933818	0.173303355	-5.770228733	0.391120309	2.256853644	-2.528629
Morf4l1	Morf4l1-003	2653.3662	7.35862E-06	0.000125577	0.173326957	-5.769443014	4.411776976	25.45349585	-2.528432
Tek	Tek-002	53544.022	3.04544E-07	1.73677E-05	0.173336456	-5.769126843	67.76731855	390.9582565	-2.528353
Terf2	Terf2-005	748.01982	0.004374516	0.014405075	0.173356636	-5.76845525	0.842554613	4.86023858	-2.528185
Gm37465	Gm37465-001	372	0.024830265	0.056170933	0.173414034	-5.766545975	1.042461028	6.011399443	-2.527707
P23-400L4	23-400L4.7-(	579	0.004822038	0.015545332	0.173450388	-5.765337355	0.867338286	5.00049782	-2.527405
Adarb1	Adarb1-005	349.89986	0.043998274	0.088617518	0.173533653	-5.762571009	0.778632965	4.486927751	-2.526713
Gm38376	Gm38376-001	993	0.000101839	0.000808717	0.173620392	-5.759692096	1.887713895	10.8726508	-2.525992
Smpd4	Smpd4-023	934.12203	8.42023E-06	0.000137855	0.173662604	-5.758292111	1.624783111	9.355975769	-2.525641
Egfl7	Egfl7-007	2141.3435	2.62514E-05	0.000305716	0.173669007	-5.758079789	4.244843132	24.44214545	-2.525588
Srgn	Srgn-001	3564.2613	1.18255E-05	0.000173087	0.17368845	-5.757435221	3.36917413	19.3978018	-2.525426
Igsf10	Igsf10-001	686.45997	0.000425511	0.002361529	0.173790257	-5.754062503	1.492805884	8.58969836	-2.524581
Atrn	Atrn-003	1255.275	0.000247237	0.001562637	0.173807217	-5.753501029	2.355494169	13.55233813	-2.52444
Col4a3bp	Col4a3bp-202	13201.755	2.49317E-05	0.000294554	0.173815914	-5.753213134	26.07492765	150.0146162	-2.524368
Ociad2	Ociad2-006	358.37942	0.003226851	0.011375017	0.173850652	-5.752063557	0.598041483	3.439972622	-2.52408
10002D19R	0002D19Rik-	694.5382	0.000771847	0.003725194	0.173867419	-5.751508849	1.373855778	7.901743662	-2.52394
Slc25a40	Slc25a40-006	290.74905	0.000906139	0.004225911	0.173936993	-5.74920827	0.610251241	3.50846148	-2.523363
Camsap1	Camsap1-001	2342.0591	4.71111E-06	9.41397E-05	0.173990599	-5.747436973	4.283821759	24.62099556	-2.522919
Gm37115	Gm37115-001	948	9.79332E-05	0.000785346	0.173992371	-5.747378421	1.769993796	10.17282415	-2.522904
Cox16	Cox16-007	440.03432	0.000473975	0.00256821	0.174045835	-5.745612917	0.699099181	4.016753285	-2.522461
Phtf1	Phtf1-004	646.07516	0.009166238	0.025752083	0.174058268	-5.74520251	0.874726579	5.025481336	-2.522358
Hus1	Hus1-003	670.87993	0.019573756	0.046724362	0.174065994	-5.744947525	1.056672013	6.070525265	-2.522294
Rnf214	Rnf214-011	158.6451	0.019211142	0.046020918	0.17408425	-5.744345035	0.282116346	1.62057363	-2.522142
Gm15789	Gm15789-001	402	0.008129726	0.023405608	0.174105917	-5.743630177	0.75533803	4.338382305	-2.521963
Slx4ip	Slx4ip-003	387.08056	0.013503162	0.034890023	0.17413926	-5.742530441	0.64014947	3.676077817	-2.521687

Cav1	Cav1-002	27664.976	0.000153579	0.001100434	0.17424199	-5.739144754	43.57412561	250.0782144	-2.520836
Rps6kb1	Rps6kb1-001	6383.9057	3.66978E-06	8.00573E-05	0.174250105	-5.738877449	11.15395195	64.0111633	-2.520769
Ncan	Ncan-001	288	0.002301976	0.00874669	0.174263372	-5.738440547	0.517065439	2.967149283	-2.520659
Stat1	Stat1-008	627.03063	0.048186654	0.095299278	0.174338903	-5.735954418	1.164103758	6.677246094	-2.520034
Hnrnpll	Hnrnpll-006	911.53297	0.000186977	0.001267862	0.174381672	-5.734547603	1.814938502	10.40785124	-2.51968
Ccdc50	Ccdc50-002	2225.0093	8.42695E-05	0.000705507	0.174422847	-5.733193871	3.915119669	22.44614009	-2.519339
Rps17	Rps17-003	167.28545	0.022614889	0.052260977	0.174437693	-5.732705951	0.365646253	2.096142453	-2.519216
Prpf4	Prpf4-002	263.65251	0.049997524	0.097953049	0.17448987	-5.730991726	0.405657214	2.324818139	-2.518785
Wdr83os	Wdr83os-003	1888.7102	0.001118574	0.004976122	0.174497448	-5.730742839	3.990862969	22.87060938	-2.518722
Cse1l	Cse1l-001	4407.6965	0.000589106	0.003031384	0.174510266	-5.730321917	9.094556288	52.11473522	-2.518616
Rbm5	Rbm5-013	458.74842	0.030032625	0.065388811	0.174518325	-5.7300573	0.784718627	4.496482695	-2.51855
Fkbp8	Fkbp8-005	193.57988	0.031991194	0.068768548	0.174582051	-5.727965703	0.405698035	2.32382443	-2.518023
Gon4l	Gon4l-201	3360.2737	5.20036E-07	2.37167E-05	0.174596669	-5.727486136	5.456006912	31.24920395	-2.517902
Dst	Dst-016	21055.891	4.45582E-06	9.04099E-05	0.17459961	-5.727389646	41.67047571	238.6630511	-2.517878
Akr1a1	Akr1a1-002	2769.8514	0.000284939	0.001737432	0.174628924	-5.726428244	4.581450049	26.23534496	-2.517636
Tspan2	Tspan2-004	197.88629	0.044376519	0.089235175	0.174651129	-5.72570017	0.32519502	1.861969183	-2.517452
Plxnc1	Plxnc1-001	911.79139	0.008805284	0.02490758	0.174660661	-5.725387699	2.33447265	13.365761	-2.517373
Lyn	Lyn-002	2787.9208	0.0368936	0.077105553	0.174703616	-5.723979964	8.463703838	48.44607119	-2.517019
Ankrd23	Ankrd23-005	339.42587	0.001334653	0.005709764	0.174786459	-5.721266993	0.584103668	3.341813037	-2.516335
Pisd	Pisd-012	1641.4059	0.029977521	0.065288699	0.174788677	-5.721194388	3.240783594	18.54115291	-2.516316
Bcas3os1	Bcas3os1-002	376.4252	0.028353083	0.062409582	0.174789383	-5.721171305	0.757077719	4.331371324	-2.516311
Ing3	Ing3-001	1047.4591	0.001979407	0.007771055	0.174822773	-5.720078584	1.99302544	11.40026214	-2.516035
Hipk1	Hipk1-004	1911.6133	0.000302252	0.001820312	0.174839427	-5.71953372	3.751236132	21.45532155	-2.515898
Lsm6	Lsm6-002	1751.8007	3.66324E-06	7.99656E-05	0.174857451	-5.718944182	3.731562014	21.34059487	-2.515749
Gid8	Gid8-002	181.10472	0.03697283	0.077233436	0.174903304	-5.717444897	0.22240599	1.27159399	-2.515371
Zranb2	Zranb2-005	549.8	9.57568E-05	0.000773142	0.174925469	-5.716720416	1.12005086	6.40301762	-2.515188
Smarce1	Smarce1-002	1313.8068	4.59743E-05	0.000453715	0.174957614	-5.715670071	2.246043599	12.83764418	-2.514923
Gm37906	Gm37906-001	1440	3.81712E-06	8.16753E-05	0.174974517	-5.715117934	2.737418154	15.64466759	-2.514783
Ncstn	Ncstn-006	659.42218	0.00219923	0.008432553	0.174998433	-5.714336851	1.087642953	6.215158208	-2.514586
Ctage5	Ctage5-011	2333.7598	2.59921E-06	6.43229E-05	0.174998739	-5.714326887	4.23659938	24.20931375	-2.514584
Prdx1	Prdx1-001	8994.4558	2.25008E-07	1.50813E-05	0.175033902	-5.713178924	17.04665464	97.39058804	-2.514294
Nfat5	Nfat5-202	17610.402	4.27864E-07	2.11533E-05	0.175034255	-5.713167406	26.90767055	153.7280264	-2.514291
Tcf12	Tcf12-009	1512.5951	0.00050327	0.002686147	0.175087954	-5.711415202	2.820138079	16.1069795	-2.513848
Gm25640	Gm25640-201	93	0.011458855	0.030704568	0.175133652	-5.709924912	0.13486781	0.770085068	-2.513472
Zfp384	Zfp384-012	403.72917	0.003342923	0.011687945	0.175176573	-5.708525872	0.87917603	5.018799115	-2.513118
Ctss	Ctss-201	5158.7976	0.006602414	0.019864206	0.175263158	-5.705705693	17.55681127	100.173998	-2.512405
E2f7	E2f7-003	398.15988	0.001015757	0.004608619	0.175279029	-5.705189065	0.691469608	3.944964846	-2.512275
Bcorl1	Bcorl1-002	1322.6893	0.004175622	0.013895751	0.175417108	-5.700698249	1.435457848	8.183112041	-2.511139
Calu	Calu-001	39707.729	8.00858E-06	0.000133023	0.175464894	-5.699145716	71.57588853	407.9214185	-2.510746
Slc4a2	Slc4a2-015	932.46883	6.06808E-05	0.000555075	0.175487629	-5.698407383	1.782644186	10.15823279	-2.510559
S100pbp	S100pbp-003	1300.6273	0.021455411	0.050204812	0.175497045	-5.698101649	2.784986816	15.86913797	-2.510481
Plod2	Plod2-002	1931.2134	0.010057573	0.027717403	0.175581349	-5.695365741	4.75419804	27.07689664	-2.509788
Ppapdc1b	Ppapdc1b-004	1195.2027	0.018733261	0.0451108	0.175592061	-5.695018298	1.862165764	10.6050681	-2.5097
Slc35a3	Slc35a3-001	8098.9223	2.09189E-08	3.6337E-06	0.175611096	-5.694401002	14.41558277	82.08810898	-2.509544
Snrpc	Snrpc-002	2524.7352	1.35693E-06	4.23267E-05	0.175633726	-5.693667285	4.850096727	27.61483706	-2.509358
Gm27743	Gm27743-201	219	0.004033333	0.013507544	0.175645339	-5.693290837	0.432296105	2.461187456	-2.509263
Gmfb	Gmfb-002	266.88059	0.009868718	0.027278152	0.175671885	-5.692430516	0.494473594	2.814756574	-2.509045
Smim14	Smim14-009	2135.1255	1.32775E-05	0.000188695	0.175677076	-5.69226232	3.497891933	19.91091845	-2.509002
H2-K1	H2-K1-002	10107.756	7.7435E-05	0.000664358	0.175687305	-5.6919309	17.38835057	98.97328991	-2.508918
Smad2	Smad2-001	4493.3503	7.84369E-05	0.000670442	0.175692241	-5.691770972	9.687597784	55.13958786	-2.508878
10005C13R	0005C13Rik-	450.08087	0.000409624	0.002292273	0.175727489	-5.690629304	0.843979475	4.80277433	-2.508588
Commd2	Commd2-002	897.08523	4.06685E-05	0.000415279	0.175735043	-5.690384706	1.735205161	9.873984911	-2.508526
Gsk3b	Gsk3b-001	27079.217	2.30132E-10	3.75335E-07	0.175767329	-5.689339446	44.65043703	254.0314927	-2.508261
Tmem65	Tmem65-001	14339	6.49675E-09	2.13318E-06	0.175821429	-5.687588864	24.0136505	136.5797711	-2.507817
Il15ra	Il15ra-006	2610.3707	8.63374E-05	0.000716341	0.175867505	-5.686098737	4.477836574	25.46142089	-2.507439
Dclre1c	Dclre1c-001	1970.2352	4.37979E-05	0.000438619	0.175900898	-5.68501931	4.125638006	23.45433173	-2.507165
Ankrd17	Ankrd17-012	1718.2058	2.85408E-05	0.000323576	0.175904529	-5.684901955	3.037442977	17.26756552	-2.507135
Xpo7	Xpo7-202	4833.6081	3.43741E-08	4.90547E-06	0.175932661	-5.683992931	8.969700384	50.98371357	-2.506905
Kirrel3	Kirrel3-002	379.43484	0.046345277	0.092361559	0.175950529	-5.683415708	0.696312873	3.957435518	-2.506758
Ogfrl1	Ogfrl1-001	1704.19	0.001664164	0.00677092	0.175951941	-5.683370099	4.998522758	28.40845478	-2.506747
20402A03R	0402A03Rik-	1680	1.84642E-05	0.000238192	0.175976632	-5.682572684	2.784194021	15.82138489	-2.506544
Fastkd2	Fastkd2-002	420.08462	0.026268621	0.058734839	0.176084423	-5.679081112	0.966628401	5.489561093	-2.505658
Sparc	Sparc-002	124605.26	1.4321E-05	0.000198661	0.176085479	-5.679059996	220.1893723	1250.468656	-2.505652
Nr3c1	Nr3c1-201	5974.5117	7.25003E-05	0.000633938	0.176107662	-5.678344638	9.169203195	52.06589579	-2.50547
Hadhb	Hadhb-003	606.98463	0.036618655	0.076608901	0.176131647	-5.677571392	1.343473297	7.627665558	-2.505274
Gm10603	Gm10603-003	550.9679	0.000335613	0.001968952	0.176183721	-5.675893287	1.169688901	6.639029382	-2.504847
Sclt1	Sclt1-006	687.27307	0.000491204	0.00263736	0.176206589	-5.675156677	1.395870946	7.92178632	-2.50466
Uqcc1	Uqcc1-003	440.77542	0.013345502	0.034576342	0.176247372	-5.673843461	0.56904052	3.228646833	-2.504326
Camsap2	Camsap2-004	193.45	0.017279414	0.042287954	0.176302897	-5.672056542	0.205019886	1.162884388	-2.503872
Rngtt	Rngtt-002	1441.3355	0.002526988	0.009391206	0.176305665	-5.671967499	2.887511895	16.37787362	-2.503849
Hnrnpll	Hnrnpll-003	3163.4825	5.57208E-07	2.4625E-05	0.176361397	-5.670175075	5.581419	31.6476229	-2.503393
Ndufb2	Ndufb2-001	1698.0176	9.52662E-05	0.000770089	0.176375655	-5.669716725	2.96216334	16.79462703	-2.503277
Phf6	Phf6-003	629.30816	0.046639974	0.092820915	0.176378515	-5.669624799	1.12926377	6.402501875	-2.503253
Caprin1	Caprin1-010	282.71876	0.004815732	0.015530962	0.176392988	-5.669159603	0.580883554	3.293121581	-2.503135
Reck	Reck-001	8274.7393	0.000617958	0.003147688	0.176433051	-5.667872301	10.41785345	59.047063	-2.502807
P24-309H3	24-309H3.1-(	767	0.000378412	0.002156866	0.176472728	-5.666597969	1.455842679	8.249675166	-2.502483
Phxr4	Phxr4-201	4207	2.68274E-06	6.56313E-05	0.17647506	-5.666524812	7.513024748	42.57273962	-2.502464
Sdhc	Sdhc-002	876.69353	0.001609046	0.00659998	0.176500771	-5.665697632	1.290402306	7.311029289	-2.502254
Slc26a11	Slc26a11-002	1625.4822	3.60068E-05	0.000379865	0.176521123	-5.665044413	3.1189283	17.66886734	-2.502087
Imp4	Imp4-001	6002.9538	7.858E-07	3.04403E-05	0.176523963	-5.664953247	11.26765306	63.8307278	-2.502064
Usp33	Usp33-003	1340.3314	0.00013515	0.001001704	0.176527815	-5.664829648	2.573418727	14.5779787	-2.502033
Gtf3c2	Gtf3c2-007	184.81823	0.038381619	0.079511913	0.176567337	-5.66356167	0.378883163	2.145828157	-2.50171
Rbbp6	Rbbp6-001	15369.158	3.7873E-06	8.16753E-05	0.176592485	-5.662755128	28.09998804	159.1233514	-2.501504
Anapc1	Anapc1-008	419.2117	0.049394334	0.097093431	0.176625593	-5.661693654	0.756781159	4.284663086	-2.501234
Cnih4	Cnih4-003	689.79926	0.01390819	0.035711485	0.176689458	-5.659647233	1.641275249	9.289038923	-2.500712
Nr2c2	Nr2c2-001	15653.875	1.64898E-07	1.26693E-05	0.176702408	-5.659232447	29.11528187	164.7701479	-2.500606
Plekha3	Plekha3-002	458.43051	5.66445E-05	0.000527675	0.176759552	-5.657402882	0.897398195	5.076943135	-2.50014
Adgrl2	Adgrl2-001	501.46026	0.004794114	0.015471441	0.176774197	-5.656934181	1.00121041	5.663781391	-2.50002
Dmxl2	Dmxl2-001	2830.5615	0.030337998	0.065939613	0.1768103	-5.655779102	5.140362698	29.07275593	-2.499726
Gm12504	Gm12504-002	567.43341	0.002002824	0.00784321	0.17682791	-5.655215848	1.203154102	6.804096146	-2.499582
P24-365C1	24-365C10.2-	256	0.015897926	0.039617577	0.176894484	-5.653087519	0.581562748	3.287625111	-2.499039
030033H14R	0033H14Rik-	636	0.000174215	0.001202752	0.17689534	-5.653060179	1.157415765	6.542940969	-2.499032
Topbp1	Topbp1-010	402.48755	0.001165674	0.005140253	0.176912379	-5.652515708	0.763160026	4.313774035	-2.498893
Itgav	Itgav-005	1382.1542	0.009800581	0.027127032	0.176958658	-5.651037421	3.270950486	18.4842636	-2.498516
Eef1g	Eef1g-005	18851.796	6.65494E-07	2.73299E-05	0.176997223	-5.649806144	42.88198812	242.27492	-2.498201
Hypk	Hypk-002	575.57892	0.012450736	0.032722352	0.177030878	-5.648732091	1.145435479	6.470258151	-2.497927
Apbb2	Apbb2-023	4354.83	0.016907106	0.041599739	0.177036081	-5.648566055	6.312254503	35.65518652	-2.497885
Nkiras1	Nkiras1-001	892.85597	1.13646E-05	0.000169087	0.177082303	-5.647091682	1.714543775	9.682185889	-2.497508
Larp1	Larp1-001	3801.1698	0.001486391	0.006214466	0.177118609	-5.645934147	8.502277095	48.00329658	-2.497212
Wdr11	Wdr11-005	1527.768	0.000511636	0.002719357	0.177129877	-5.64557498	2.918393797	16.476011	-2.497121
Ftsj1	Ftsj1-001	1333.5161	1.5594E-05	0.000210864	0.17722008	-5.642701436	2.319466224	13.08805539	-2.496386
Ppfia1	Ppfia1-004	1783.3044	0.000292074	0.001771478	0.177278124	-5.640853903	3.593812449	20.27217098	-2.495914
Timm17a	Timm17a-004	1624.9904	1.73403E-05	0.000227812	0.177307623	-5.63991543	3.201255158	18.05480836	-2.495674
Lrrc48	Lrrc48-003	1262.4576	6.5059E-06	0.000115754	0.177309265	-5.639863217	2.23444895	12.60198644	-2.49566
Ssr3	Ssr3-002	439.73709	0.008592073	0.024435912	0.177329233	-5.639228128	0.958659289	5.406098427	-2.495498
Ptbp1	Ptbp1-004	6838.6322	0.002117388	0.008180414	0.177397628	-5.63705395	14.91445971	84.07361401	-2.494941
Cep63	Cep63-003	1267.8237	8.05921E-06	0.000133799	0.17741995	-5.636344719	1.849973941	10.42709085	-2.49476

Ccdc93	Ccdc93-006	1135.957	0.000127798	0.000959462	0.177422906	-5.636250833	1.920779335	10.82599413	-2.494736
300002I08Ri	00002I08Rik-(	633.26171	0.033187268	0.07081136	0.177430784	-5.63600056	1.19568484	6.738880429	-2.494672
Usp7	Usp7-006	536.23743	0.000706474	0.003487953	0.17745365	-5.635274334	1.108548194	6.246973188	-2.494486
Eif4enif1	Eif4enif1-006	629.97452	0.017545951	0.042823973	0.177459307	-5.635094684	1.200267181	6.76361921	-2.49444
10008F13Ri	0008F13Rik-	969.22702	9.77853E-05	0.000785079	0.177462279	-5.635000335	1.541018217	8.683638168	-2.494416
Nif3l1	Nif3l1-007	1295.8002	1.90271E-05	0.000243706	0.177529785	-5.63285762	2.132145958	12.01007461	-2.493867
Rbms3	Rbms3-001	6171.0023	1.61053E-05	0.000215437	0.177535242	-5.632684459	10.20259012	57.46797081	-2.493823
Ggct	Ggct-001	1109.2651	0.003424778	0.011901242	0.177537209	-5.632622073	2.244021671	12.639726	-2.493807
Itm2a	Itm2a-001	969	0.015294622	0.038458829	0.177537783	-5.632603838	1.758868729	9.907010756	-2.493802
Adgrg3	Adgrg3-201	274	0.038150307	0.079138031	0.177560322	-5.631888851	0.707482831	3.984464668	-2.493619
Adi1	Adi1-001	3346.3595	2.16534E-05	0.000266994	0.177568813	-5.631619571	6.773144083	38.14377078	-2.49355
Setd3	Setd3-007	865.19085	0.014881622	0.037674295	0.177642915	-5.629270384	1.845662369	10.38973251	-2.492948
P23-332O18	23-332O18.2-	1929	5.04374E-06	9.84098E-05	0.177647247	-5.629133114	3.980163301	22.40486904	-2.492913
Tmem41a	Tmem41a-201	938	6.71821E-05	0.000599528	0.177744033	-5.626067907	1.674247141	9.419428106	-2.492127
Wapal	Wapal-001	9856.9493	1.1141E-07	1.00947E-05	0.177804471	-5.624155547	20.13029189	113.2158928	-2.491636
Hus1	Hus1-006	1125.9059	0.000366187	0.002099063	0.177843946	-5.622907185	1.86811792	10.50425368	-2.491316
Rab11b	Rab11b-002	3747.575	7.67098E-05	0.00066046	0.177868514	-5.622130516	6.551471359	36.83322705	-2.491117
Zfp595	Zfp595-002	1119.5877	0.003757442	0.01278359	0.177884447	-5.62162693	1.83173897	10.29735312	-2.490988
St8sia4	St8sia4-004	920.17841	0.00051447	0.002731876	0.177911584	-5.620769479	2.384064749	13.40027838	-2.490768
P23-262J12	23-262J12.2-(	948	0.001898768	0.007513917	0.177956413	-5.619353553	1.798979485	10.10910176	-2.490404
Zfp644	Zfp644-020	6331.1889	1.45372E-06	4.40618E-05	0.177963938	-5.619115926	11.41066811	64.1178669	-2.490343
730013B05R	0013B05Rik-	771.32408	0.000446451	0.002448905	0.177976959	-5.61870483	1.368454598	7.688942459	-2.490238
Fkbp4	Fkbp4-002	1051.9407	0.000856404	0.00403964	0.177990797	-5.618268005	1.895866547	10.6514853	-2.490125
Utp18	Utp18-002	494.90883	0.021239846	0.049786485	0.17801236	-5.617587455	1.318735379	7.408111324	-2.489951
Fam96b	Fam96b-002	532.45422	0.002329241	0.008824688	0.178025409	-5.617175707	0.832027806	4.67364638	-2.489845
Sema6d	Sema6d-008	10373.689	4.38152E-05	0.000438665	0.178047992	-5.61646322	19.21086676	107.8971266	-2.489662
Wdr26	Wdr26-005	571.83516	0.021243503	0.049790595	0.178071035	-5.615736424	0.892149067	5.01007401	-2.489475
610007P14R	0007P14Rik-	1281.2408	0.002195849	0.008422873	0.178079948	-5.615455382	2.694084277	15.12851005	-2.489403
BC085271	BC085271-002	222.23851	0.032191579	0.069106331	0.17814772	-5.61331909	0.617408624	3.465711618	-2.488854
Rbm5	Rbm5-006	20870.958	1.77518E-07	1.32462E-05	0.178197054	-5.611765038	37.59761386	210.988975	-2.488455
Tubgcp4	Tubgcp4-005	1859.8281	1.03999E-06	3.5907E-05	0.17835506	-5.606793556	3.470105104	19.45616294	-2.487176
Ccr5	Ccr5-003	244.13211	0.001744469	0.007034977	0.178372472	-5.606246243	0.453255542	2.541062181	-2.487035
Ccnl1	Ccnl1-003	4400.1088	0.031980112	0.068753731	0.178375929	-5.606137571	9.404036854	52.72032433	-2.487007
Pla2g4a	Pla2g4a-003	790.80166	0.000992843	0.004524933	0.178419329	-5.604773896	2.150410423	12.0525642	-2.486656
Gm23365	Gm23365-201	111	0.013073844	0.034010265	0.178423165	-5.604653397	0.235404428	1.319360227	-2.486625
Asphd1	Asphd1-001	89.280522	0.040846839	0.083507685	0.178488158	-5.602612591	0.219431982	1.229392385	-2.4861
S100pbp	S100pbp-010	1027.3735	0.003656873	0.012522285	0.178498961	-5.602273508	1.703609345	9.544085499	-2.486012
Pdcd6ip	Pdcd6ip-006	644.27286	0.00309694	0.011012131	0.178546621	-5.60077807	0.972491953	5.446711605	-2.485627
Baz2b	Baz2b-003	1839.0561	9.78642E-05	0.00078516	0.178547118	-5.600762472	3.311971363	18.54956492	-2.485623
Cenpq	Cenpq-002	243.44979	0.008043173	0.023225585	0.178573053	-5.599949073	0.46991337	2.631490941	-2.485414
P24-535M14	24-535M14.2-	97	0.047319389	0.093936653	0.178575473	-5.599873156	0.195575481	1.095197885	-2.485394
Tmc6	Tmc6-003	403.90552	0.043249691	0.087423389	0.178582481	-5.599653423	0.644160467	3.607075366	-2.485338
Pdxdc1	Pdxdc1-015	490.65018	0.014517471	0.036929842	0.178597241	-5.599190637	0.502342487	2.812711349	-2.485218
Hnrnpdl	Hnrnpdl-004	11204.498	4.54158E-07	2.20013E-05	0.178618573	-5.598521951	22.27679854	124.7171456	-2.485046
Zcrb1	Zcrb1-006	2904.0013	1.83211E-06	5.06863E-05	0.178648609	-5.59758067	4.976493247	27.8563224	-2.484803
Them4	Them4-001	407.7359	0.00065338	0.003285841	0.178679029	-5.59662767	0.760187699	4.254487512	-2.484558
Ccnt1	Ccnt1-002	1671.3968	8.70873E-05	0.000720469	0.178706549	-5.595765835	3.07360808	17.19919109	-2.484336
Mthfd2l	Mthfd2l-003	1896.2326	1.66493E-06	4.76145E-05	0.178742844	-5.59462958	3.300031395	18.46245326	-2.484043
Gm14335	Gm14335-001	438	0.000743029	0.003619507	0.178774677	-5.593633388	0.836329702	4.678121747	-2.483786
P24-328P2	24-328P2.5-(	1050.1078	0.000590224	0.003036224	0.17883068	-5.591881667	1.298177511	7.259255024	-2.483334
Gm27010	Gm27010-001	2375	4.72854E-06	9.43685E-05	0.178871066	-5.590619117	4.382256422	24.49952653	-2.483008
Galt	Galt-008	613.93798	0.011543706	0.030882654	0.178949063	-5.588182385	0.969245333	5.416319698	-2.482379
Prrg1	Prrg1-202	517.9234	0.016053134	0.039896947	0.178959733	-5.58784921	1.049698056	5.865554453	-2.482293
Ric1	Ric1-001	5835.0508	3.99519E-07	2.03932E-05	0.17897911	-5.587244229	11.78497658	65.84554239	-2.482137
Gls	Gls-017	387.78931	0.007954427	0.023031459	0.178982781	-5.58712963	0.671237383	3.750290274	-2.482107
Clasp1	Clasp1-201	609.06846	0.005537821	0.017350017	0.179007956	-5.586343881	1.175437178	6.56639629	-2.481904
Ssb	Ssb-201	1182.4456	0.000339677	0.00198599	0.179097645	-5.583546347	1.925149051	10.74915895	-2.481182
Prpsap2	Prpsap2-002	691.97393	0.000980485	0.004480954	0.179118596	-5.582893259	1.508658371	8.42267865	-2.481013
Slc25a46	Slc25a46-201	9231.429	5.77654E-08	6.91725E-06	0.17912435	-5.582713903	16.36566373	91.36481846	-2.480967
Tmem164	Tmem164-008	1122.5506	0.004736604	0.0153213	0.17915113	-5.581879398	1.84224346	10.28318082	-2.480751
Larp1	Larp1-003	186.28377	0.017162728	0.04207153	0.179186752	-5.580769716	0.250966144	1.400584257	-2.480464
Hps3	Hps3-002	1508.6227	0.000850838	0.004018919	0.179198839	-5.580393286	3.679842161	20.53496649	-2.480367
P24-121I11	24-121I11.4-(	363	0.008945225	0.025245053	0.179220621	-5.579715075	0.804123111	4.486777844	-2.480191
Gm37663	Gm37663-001	1046	3.47887E-05	0.000370611	0.179230242	-5.57941554	2.179221252	12.15878092	-2.480114
Pum2	Pum2-201	1993.337	0.013503712	0.034890023	0.179296242	-5.577361731	3.050412931	17.01325635	-2.479583
Crtc3	Crtc3-002	2036.3987	1.55947E-05	0.000210864	0.179374752	-5.574920594	4.439999786	24.75264624	-2.478951
Hadha	Hadha-001	2128.8941	7.42244E-06	0.000126351	0.179394706	-5.574300508	3.090637464	17.22814199	-2.478791
Oxr1	Oxr1-205	4590.1651	7.46245E-05	0.000647589	0.179398515	-5.574182155	9.480033588	52.8434335	-2.47876
Ube2d3	Ube2d3-008	16835.932	4.5279E-09	1.8062E-06	0.179532104	-5.570034418	33.84502339	188.5179452	-2.477686
Arhgap29	Arhgap29-005	2328.7745	1.47582E-05	0.00020308	0.17956286	-5.569080385	4.064533375	22.63571309	-2.477439
Senp6	Senp6-001	2258.3478	0.001962229	0.007723978	0.179567969	-5.56892193	4.498909875	25.05407341	-2.477398
Gm5697	Gm5697-001	671	0.000160295	0.001134084	0.179576679	-5.568651807	1.318849173	7.344211831	-2.477328
Brwd3	Brwd3-003	3891.0662	1.76429E-06	4.94088E-05	0.179615415	-5.567450892	8.484138165	47.23502259	-2.477017
Cul5	Cul5-002	4551.9391	1.41057E-05	0.000196791	0.179706081	-5.564641964	9.26742065	51.56987785	-2.476289
Lamp2	Lamp2-002	13034.608	2.87943E-07	1.69341E-05	0.179740761	-5.563568285	23.61317168	131.3734931	-2.47601
Helq	Helq-010	158.63516	0.047840794	0.094773972	0.179791253	-5.562005853	0.309993496	1.72418564	-2.475605
Trip4	Trip4-201	4167.8432	7.14285E-08	7.86633E-06	0.17985621	-5.559997053	7.335259554	40.7840215	-2.475084
Zfp451	Zfp451-004	11185.26	1.47972E-09	1.07831E-06	0.179868119	-5.55962894	21.62922663	120.2504743	-2.474989
Uspl1	Uspl1-008	2442.7416	0.000143374	0.001046139	0.179916951	-5.558119972	4.315726573	23.98732606	-2.474597
Pdlim5	Pdlim5-001	36299.693	7.19577E-08	7.87397E-06	0.179921554	-5.557977779	61.84451911	343.730463	-2.47456
Terf1	Terf1-003	2335.3132	1.78013E-07	1.32542E-05	0.179924838	-5.557876344	4.465246119	24.81728578	-2.474534
Arpc3	Arpc3-005	471.41765	0.001637742	0.006688048	0.179941996	-5.557346368	0.799095062	4.440848041	-2.474396
Mapkap1	Mapkap1-001	2980.7874	2.65716E-05	0.000308006	0.17994371	-5.557293441	5.705804228	31.70882841	-2.474382
Yipf6	Yipf6-003	838.67596	0.000720774	0.003539287	0.179966578	-5.556587276	1.42695611	7.929006165	-2.474199
Nomo1	Nomo1-002	438.5092	0.004756395	0.015372892	0.179979733	-5.556181148	0.733376075	4.074770324	-2.474094
P23-140B23	23-140B23.2-	120	0.007317941	0.021567806	0.179981779	-5.556117985	0.233448565	1.297067772	-2.474077
Sox17	Sox17-001	9798.5595	0.000155049	0.001107262	0.179984205	-5.55604311	17.94190404	99.6859923	-2.474058
Gm10048	Gm10048-001	833.91624	7.9557E-05	0.000677649	0.179987567	-5.555939319	1.43910896	7.995602053	-2.474031
Tmcc3	Tmcc3-006	162.05797	0.02325037	0.053408788	0.180050528	-5.553996495	0.297698997	1.653419189	-2.473526
Setd6	Setd6-001	725.75968	0.001857825	0.007386012	0.180071038	-5.553363893	1.429518332	7.938635489	-2.473362
Rffl	Rffl-001	1815.17	0.048738384	0.096157237	0.180079275	-5.553109863	2.917375949	16.20050916	-2.473296
Gm25534	Gm25534-201	116	0.010002259	0.027587161	0.180106109	-5.552282517	0.204949192	1.137935816	-2.473081
Setd6	Setd6-006	825.71702	3.24736E-05	0.000354437	0.180113273	-5.552061687	1.58471134	8.798415117	-2.473024
Pde7a	Pde7a-001	2140.5188	0.001331731	0.005700047	0.180119255	-5.551877293	4.511946315	25.04977229	-2.472976
Sfi1	Sfi1-020	313.4423	0.020873177	0.049087222	0.180132459	-5.551470332	0.484772218	2.691198586	-2.47287
Hspa4	Hspa4-001	26668.259	1.18049E-05	0.000172948	0.1801418	-5.551182451	62.19016536	345.2289545	-2.472795
Stxbp5	Stxbp5-001	9041.625	2.68565E-06	6.56555E-05	0.180156904	-5.550717065	16.02705922	88.9616711	-2.472674
Ndrg2	Ndrg2-201	755.24207	0.003253946	0.011444615	0.1801783	-5.550057914	1.445409533	8.022106621	-2.472503
P23-85H24	23-85H24.1-(	523	0.003369836	0.011759986	0.180191478	-5.549652033	0.79461363	4.409829148	-2.472397
Mtx2	Mtx2-003	534.87883	0.010531598	0.028730166	0.180212025	-5.549019264	1.108125151	6.149007807	-2.472233
Gbp5	Gbp5-005	1011.3371	0.036475753	0.076385667	0.180273008	-5.547142156	1.57924118	8.760275326	-2.471745
Sms	Sms-001	1497.0332	0.00073449	0.003587605	0.180313141	-5.545907504	2.792145988	15.48498339	-2.471424
Tulp4	Tulp4-012	1870.7758	0.001145949	0.005072753	0.180334191	-5.545260137	4.871669786	27.01467627	-2.471255
Gm4691	Gm4691-002	1806	3.48782E-05	0.000371217	0.180345004	-5.544927643	3.94589244	21.87968807	-2.471169
Auh	Auh-002	1531.6011	1.42683E-05	0.00019809	0.180362452	-5.544391252	3.526187089	19.55056085	-2.471029
Brwd1	Brwd1-201	2894.738	2.52048E-05	0.000297165	0.180391244	-5.5435063	5.583950437	30.95466442	-2.470799
Aldoa	Aldoa-002	7911.583	1.30637E-07	1.09963E-05	0.1804247	-5.542478383	13.96496627	77.40052367	-2.470531

Zfp638	Zfp638-203	550.76974	0.024621087	0.055823662	0.18044273	-5.541924566	0.762845342	4.227631341	-2.470387
Xndc1	Xndc1-003	1639.5007	0.000175152	0.001207689	0.180504164	-5.540038408	3.065972012	16.9856027	-2.469896
Leprotl1	Leprotl1-001	3588	1.24502E-06	4.00771E-05	0.180524708	-5.539407933	7.154227088	39.63018229	-2.469732
Cnpy3	Cnpy3-002	831.19704	0.024198383	0.055070049	0.180539735	-5.538946857	1.575713874	8.727795412	-2.469612
Gnat1	Gnat1-005	280.57441	0.004761078	0.015385122	0.180589528	-5.537419649	0.540642493	2.993764363	-2.469214
Npm1	Npm1-001	6682.1452	0.000423729	0.002353287	0.180598504	-5.537144419	17.29396479	95.75918062	-2.469142
Tfr2	Tfr2-005	61	0.036984492	0.077248358	0.180647877	-5.535631058	0.114784904	0.635406879	-2.468748
Gm7204	Gm7204-201	67	0.037385919	0.07788733	0.180723786	-5.533305942	0.114515071	0.633646923	-2.468142
Ift88	Ift88-002	734.67684	0.009100571	0.025603298	0.180724661	-5.533279167	1.189234247	6.580365082	-2.468135
Skiv2l	Skiv2l-002	1359.0644	0.003274473	0.011503816	0.180736234	-5.532924847	2.264558131	12.52962995	-2.468042
Gas5	Gas5-021	622.34289	0.006200584	0.018937936	0.180744401	-5.532674825	1.474753757	8.159332987	-2.467977
Pear1	Pear1-012	4170.5837	6.27687E-06	0.000113294	0.180799918	-5.530975963	7.662833761	42.38294934	-2.467534
Gm37188	Gm37188-001	528	0.000337761	0.001978319	0.180844384	-5.529616003	1.06752131	5.902982917	-2.467179
Elovl7	Elovl7-201	10329	0.000109731	0.000855132	0.180861511	-5.529092369	15.78583436	87.28133628	-2.467043
Sf1	Sf1-001	6521.9241	3.13892E-06	7.27812E-05	0.18087698	-5.528619496	13.92869279	77.00644251	-2.466919
30020D12F	0020D12Rik-	514.53613	0.000710043	0.003500683	0.180892315	-5.528150811	0.880383929	4.866895131	-2.466797
30020D12F	0020D12Rik-	628.44508	0.00052742	0.002782521	0.180894737	-5.528076796	1.075285668	5.944261751	-2.466778
Pdxdc1	Pdxdc1-002	533.1183	0.000215395	0.001409493	0.180925763	-5.527128833	0.82949813	4.584743033	-2.46653
Rac1	Rac1-002	72866.824	3.53609E-09	1.57287E-06	0.180934462	-5.526863083	115.9054339	640.5934637	-2.466461
Gm15903	Gm15903-002	761.06911	0.000355566	0.002054344	0.180963629	-5.525972281	1.357101316	7.499304252	-2.466228
Abcb8	Abcb8-001	137.0663	0.029029585	0.06357182	0.18102824	-5.524000022	0.278285888	1.53725125	-2.465713
Gm38322	Gm38322-001	163	0.002982117	0.010703076	0.18105252	-5.523259206	0.283986707	1.568532195	-2.46552
P24-555M20	24-555M20.3-	2602	4.9138E-07	2.31674E-05	0.181054053	-5.523212452	4.583193889	25.31395356	-2.465508
42615	Sept2-201	4657.3	0.00011249	0.000871277	0.181069697	-5.522735268	7.814621547	43.15808602	-2.465383
Suco	Suco-001	9927.5443	7.84056E-10	7.67255E-07	0.181070754	-5.522703019	19.37181944	106.9848057	-2.465375
Wdr17	Wdr17-013	471	0.001687849	0.006843712	0.181171201	-5.519641064	0.842803988	4.651975503	-2.464574
P24-68G17	24-68G17.2-(	2165	8.49046E-07	3.18758E-05	0.181183809	-5.51925698	4.303059186	23.74968945	-2.464474
Trip12	Trip12-010	709.00993	5.70138E-05	0.000530631	0.1812502	-5.517235298	1.400327038	7.725933762	-2.463946
Smug1	Smug1-201	1300	3.65947E-06	7.99402E-05	0.181327429	-5.514885436	2.451023942	13.51711624	-2.463331
Nfkbiz	Nfkbiz-201	182.14289	0.017685001	0.04309272	0.181332737	-5.514724012	0.387898015	2.139150495	-2.463289
Tuba1a	Tuba1a-001	16725.852	0.000283292	0.001729843	0.181414275	-5.512245398	35.81115207	197.3998582	-2.46264
Rpl30-ps10	pl30-ps10-00	2047	6.32766E-07	2.64942E-05	0.181415183	-5.512217807	4.421437627	24.37192722	-2.462633
Fzd6	Fzd6-201	8185.4623	0.000172248	0.001193506	0.18141589	-5.512196302	12.10006535	66.6979355	-2.462627
Ephb2	Ephb2-001	272.9121	0.018698706	0.045047476	0.181476176	-5.51036516	0.548190806	3.020731517	-2.462148
Rb1cc1	Rb1cc1-006	1095.41	0.005691161	0.017700897	0.181505514	-5.509474502	1.566761117	8.632030423	-2.461915
Trip12	Trip12-007	1325.5605	4.82663E-05	0.000469105	0.181556286	-5.50793377	2.718254526	14.9719659	-2.461511
Hdx	Hdx-001	377.26312	0.006315854	0.019178827	0.181570916	-5.507489969	0.822599947	4.530460956	-2.461395
Zfp868	Zfp868-001	1930.3343	0.000331616	0.001951175	0.18160115	-5.506573061	3.18829975	17.55660551	-2.461155
Gfpt1	Gfpt1-001	9260.009	2.43015E-06	6.16537E-05	0.181611393	-5.506262494	19.64120881	108.1496514	-2.461073
Cyp4b1	Cyp4b1-002	6278.4234	0.006948994	0.020708522	0.181616295	-5.506113868	14.56180755	80.17897051	-2.461034
Gm17494	Gm17494-001	4387.7071	9.16672E-08	9.04785E-06	0.181637864	-5.50546002	8.353828207	45.99166721	-2.460863
Lrrc3	Lrrc3-001	1189	3.48726E-05	0.000371217	0.181805385	-5.500387118	2.245930575	12.3534876	-2.459533
Mme	Mme-001	495.6658	0.012625236	0.033084636	0.181824025	-5.499823253	0.716367802	3.939896294	-2.459385
Med28	Med28-004	1401.1794	0.000253162	0.001589223	0.181887536	-5.497902835	2.732525841	15.02316157	-2.458881
Lin54	Lin54-001	773.25309	0.008031112	0.023196809	0.181901996	-5.497465777	1.251608955	6.880677394	-2.458767
Rbbp5	Rbbp5-003	1168.5378	0.045590375	0.091170102	0.181903579	-5.497417958	1.956125215	10.75363788	-2.458754
Mtch2	Mtch2-011	436.41728	0.043143815	0.087240268	0.181921407	-5.496879222	0.685492168	3.768067658	-2.458613
Smyd2	Smyd2-004	280.35293	0.048637942	0.095989932	0.181923988	-5.496801218	0.504000526	2.770390708	-2.458592
Tcf4	Tcf4-012	2634.4537	0.001301843	0.005603422	0.181946085	-5.496133644	6.52770871	35.87715946	-2.458417
Cpne8	Cpne8-003	9713.4778	0.004458678	0.01462877	0.18196769	-5.495481084	21.54459056	118.3978899	-2.458246
Gm37262	Gm37262-001	476	0.002216697	0.00848953	0.181977267	-5.495191873	1.043660742	5.735116026	-2.45817
Rfwd3	Rfwd3-201	5858	8.15487E-08	8.50945E-06	0.182014681	-5.494062321	11.93077186	65.54840413	-2.457873
Ccp110	Ccp110-002	1847.3893	2.19413E-05	0.000269738	0.182030377	-5.493588574	4.04720184	22.23366178	-2.457749
Ly6c1	Ly6c1-002	2833.9886	0.01448419	0.036872565	0.182051727	-5.492944335	2.182697194	11.98943419	-2.45758
P23-258C6	23-258C6.2-(	248	0.003983718	0.013379323	0.182060051	-5.492693175	0.428654213	2.35446607	-2.457514
Gm10662	Gm10662-001	280	0.001221814	0.005334921	0.182101534	-5.491441948	0.609644543	3.347827616	-2.457185
30403N20F	0403N20Rik-	150	0.018963087	0.045560838	0.182107401	-5.491265006	0.36087924	1.981683542	-2.457139
Brca2	Brca2-001	769.29494	0.034611061	0.073287719	0.182143362	-5.490180864	2.069029953	11.35934865	-2.456854
Gnb4	Gnb4-004	1468.9597	0.001638818	0.006689254	0.182161295	-5.489640378	2.436801343	13.37716304	-2.456712
Gm37120	Gm37120-001	184	0.021349794	0.050002082	0.182191043	-5.488744037	0.358383711	1.967076458	-2.456476
Ptpn1	Ptpn1-002	4011.5896	0.010545241	0.028752226	0.182259159	-5.486692702	8.689791704	47.67821673	-2.455937
Tnfaip8l1	Tnfaip8l1-001	678.99816	0.046116953	0.092013612	0.182286949	-5.485856263	1.908336467	10.46885956	-2.455717
Eif2b3	Eif2b3-003	478.81995	0.008165068	0.023472686	0.182298237	-5.485516572	0.990360357	5.432638153	-2.455627
Ankib1	Ankib1-001	7281.2249	2.92075E-07	1.70418E-05	0.182307019	-5.485252336	13.79084239	75.64625043	-2.455558
Gm38211	Gm38211-001	10999	8.95267E-09	2.44914E-06	0.182322704	-5.484780421	19.82988266	108.7625522	-2.455434
Gm20548	Gm20548-001	671.98242	0.00111312	0.00495701	0.182358736	-5.483696713	1.065463986	5.842681359	-2.455149
Rps6ka3	Rps6ka3-001	40265.927	7.92453E-09	2.31621E-06	0.182410074	-5.48215336	57.56842135	315.5989146	-2.454743
Tmem131	Tmem131-002	1244.3554	0.000120125	0.000916522	0.182418458	-5.481901397	2.740914437	15.02542268	-2.454676
P24-423B3	24-423B3.2-(	273	0.002636703	0.009717755	0.182432003	-5.481494383	0.522730226	2.8653428	-2.454569
P24-399A1	24-399A15.2-	298	0.003354782	0.011721032	0.182452095	-5.48089076	0.583504929	3.198126774	-2.45441
Hyls1	Hyls1-002	1155.0024	0.000945588	0.00436239	0.182457255	-5.48073575	2.016194338	11.05022839	-2.45437
P23-253G1	23-253G12.9-	2991	0.000177061	0.001217983	0.182481609	-5.480004301	7.480410578	40.99268215	-2.454177
Krt222	Krt222-001	213.14134	0.006269075	0.019090929	0.182573706	-5.477239974	0.411652038	2.254716999	-2.453449
Mrpl19	Mrpl19-001	2733.3354	3.75649E-05	0.000392136	0.182607489	-5.476226656	5.344619489	29.26834771	-2.453182
Olfr1420	Olfr1420-201	232	0.017217597	0.042178865	0.182616434	-5.475958419	0.555533246	3.042076956	-2.453111
Fsd1l	Fsd1l-001	1968.5367	0.02898214	0.063480098	0.182789355	-5.470778091	4.274247151	23.38345767	-2.451746
Ankrd13c	Ankrd13c-001	3878.6199	5.94723E-06	0.000108927	0.182859612	-5.468676145	6.541782733	35.77489118	-2.451192
Gm36933	Gm36933-001	3300	1.68016E-05	0.000222957	0.182894541	-5.467631745	6.558478579	35.85934568	-2.450916
P23-403P24	23-403P24.7-(	234	0.036809108	0.076959318	0.182914418	-5.467037609	0.454860859	2.486741421	-2.450759
St8sia2	St8sia2-001	309.04337	0.006149723	0.018824562	0.182970203	-5.465370761	0.586606116	3.206019915	-2.450319
Srrm1	Srrm1-003	10571.966	1.52844E-07	1.21842E-05	0.182980833	-5.46505327	21.67873634	118.4754489	-2.450236
Gm13785	Gm13785-001	224	0.012223699	0.032257364	0.182988448	-5.464825841	0.286886133	1.56778275	-2.450176
Morf4l2	Morf4l2-002	102.97786	0.027027525	0.060106842	0.182991985	-5.464720221	0.207813797	1.135644261	-2.450148
Il17f	Il17f-003	299.91608	0.002006986	0.00785143	0.182995233	-5.464623235	0.622412297	3.401248698	-2.450122
Bfar	Bfar-006	120.74703	0.012074569	0.03196429	0.182998275	-5.464532379	0.191856937	1.048408445	-2.450098
Ubap2l	Ubap2l-013	512.19055	0.000319061	0.001894564	0.183030131	-5.463581289	1.14692441	6.266314745	-2.449847
St6galnac6	St6galnac6-01	991.32628	3.39546E-05	0.000365017	0.183051465	-5.462944525	1.865449826	10.19084891	-2.449679
Tmem170	Tmem170-201	1484	3.6387E-06	7.97347E-05	0.183051921	-5.462930921	2.763760569	15.09823307	-2.449675
Matr3	Matr3-003	2364.6572	0.002510023	0.009345396	0.183067487	-5.462466428	4.439407421	24.250114	-2.449553
RP23-23C4	P23-23C4.6-0	1236	0.000113885	0.000879696	0.183098995	-5.461526427	2.218617234	12.11703665	-2.449304
Agrn	Agrn-005	1016.8696	0.037421641	0.077942787	0.183156253	-5.459819064	1.074950568	5.869035604	-2.448853
Gm28874	Gm28874-001	2762	4.82644E-07	2.29591E-05	0.183321627	-5.454893758	5.252031612	28.64927445	-2.447551
C2cd3	C2cd3-003	1225.0063	0.007823351	0.022730724	0.183374611	-5.453317649	2.577433264	14.05556231	-2.447134
Mga	Mga-203	10513.817	5.36199E-07	2.4196E-05	0.183401301	-5.452524038	21.38581567	116.606674	-2.446924
Ctnnd1	Ctnnd1-019	653.75957	0.002402819	0.009038612	0.183459074	-5.450806982	1.252049966	6.824682696	-2.44647
Ubap2l	Ubap2l-003	7634.4147	0.000141812	0.001038724	0.183474277	-5.450355313	14.36173989	78.27658535	-2.44635
Dync1h1	Dync1h1-002	151.12597	0.017597222	0.042930753	0.183492883	-5.449802647	0.089435007	0.487403141	-2.446204
Gypc	Gypc-002	1273.0847	0.000919191	0.004269942	0.1835461	-5.448222544	2.00572774	10.92765109	-2.445786
Nrip3	Nrip3-001	118.81947	0.045562698	0.091120075	0.183582768	-5.447134338	0.248178059	1.35185923	-2.445497
Atat1	Atat1-006	220.75579	0.00275831	0.010058609	0.183587375	-5.446997651	0.341879596	1.862217358	-2.445461
30529C04R	0529C04Rik-	105	0.02394733	0.054610562	0.183587902	-5.446982009	0.183450089	0.999249336	-2.445457
Npas2	Npas2-001	1727.7476	0.00113031	0.005015953	0.183674667	-5.44440896	2.477229819	13.48705222	-2.444775
Stat1	Stat1-006	3210.6986	0.000897132	0.004194208	0.183700668	-5.443638346	6.031263004	32.83201457	-2.444571
Ppm1e	Ppm1e-001	1661.3316	6.22947E-06	0.00011271	0.183723491	-5.442962114	3.714101666	20.21571466	-2.444392
Rab4b	Rab4b-005	216.3831	0.001240075	0.005400785	0.183726199	-5.442881891	0.36340561	1.977973812	-2.444371
Gpbp1	Gpbp1-001	2230.0791	0.015932384	0.039668809	0.183738671	-5.442512421	3.772422319	20.53145533	-2.444273
Arhgap6	Arhgap6-004	707.43469	0.005465636	0.017186746	0.183740732	-5.442451377	1.065531545	5.799103623	-2.444257

Fam92a	Fam92a-004	542.05175	0.000309843	0.00185203	0.183749815	-5.44218235	0.924222949	5.029789821	-2.444185
Sar1b	Sar1b-001	3427.6201	3.65163E-06	7.99158E-05	0.183772262	-5.4415176	6.637194684	36.11641169	-2.444009
Stk16	Stk16-013	329.15998	0.001158196	0.005112543	0.183780515	-5.44127325	0.642807173	3.497689477	-2.443944
Gm14212	Gm14212-001	677	0.002104116	0.008139522	0.183794058	-5.440872304	1.37290712	7.469812322	-2.443838
Gm26798	Gm26798-201	1310	0.000303424	0.001824484	0.183819576	-5.440116983	2.162173541	11.762477	-2.443638
Gm15482	Gm15482-001	338	0.011328177	0.030394834	0.183838953	-5.439543612	0.328320396	1.785913114	-2.443486
Xpo4	Xpo4-001	4191.0963	1.55787E-05	0.00021083	0.183864478	-5.438788465	8.615447787	46.85759804	-2.443285
Cd46	Cd46-001	3376.6425	0.000300182	0.001811669	0.183869856	-5.438629363	5.961930656	32.42473113	-2.443243
Nfix	Nfix-004	3211.4845	7.8825E-07	3.04713E-05	0.183891741	-5.437982126	5.80896582	31.5890523	-2.443071
Fam126b	Fam126b-008	731.35254	0.000423391	0.002351791	0.183951467	-5.436216498	1.393717105	7.576547922	-2.442603
Plcb4	Plcb4-003	25243.927	0.000210299	0.00138381	0.183998585	-5.434824398	38.47565165	209.1084104	-2.442233
Slc5a3	Slc5a3-001	5222.5496	4.58779E-07	2.21853E-05	0.184064832	-5.432868355	11.39019842	61.88144855	-2.441714
RP23-7E4.4	RP23-7E4.4-00	191	0.044187344	0.088940794	0.184103608	-5.431724068	0.365114149	1.983199309	-2.44141
Ccnc	Ccnc-002	378.19889	0.000673696	0.003361122	0.184139135	-5.430676116	0.752351816	4.085779036	-2.441132
Gprasp1	Gprasp1-004	1146.7437	9.50103E-05	0.000769169	0.184160276	-5.43005268	1.841531849	9.999614953	-2.440966
Rnf38	Rnf38-008	466.41345	0.000950429	0.004376469	0.184252707	-5.427328687	0.822243534	4.46258592	-2.440242
Tom1l1	Tom1l1-001	1302.7187	0.045317408	0.090671879	0.184261239	-5.427077377	1.867912961	10.13730817	-2.440175
Ltbp4	Ltbp4-006	200.47821	0.019756976	0.047063322	0.18426664	-5.426918284	0.30189607	1.6383653	-2.440133
Lsm12	Lsm12-005	811.11662	0.005172143	0.016437403	0.184268348	-5.426867986	2.055838126	11.15676211	-2.44012
Bbx	Bbx-001	11936.099	5.07575E-08	6.36457E-06	0.184302393	-5.425865526	21.41975941	116.2207342	-2.439853
30428H23R	0428H23Rik-	3180.2068	4.75656E-05	0.000464355	0.184316738	-5.425443236	5.500183522	29.84093349	-2.439741
Prr13	Prr13-001	5682.6617	2.97761E-05	0.000334117	0.184323171	-5.425253898	8.477992847	45.99526374	-2.439691
S100a3	S100a3-005	584	5.2441E-05	0.000498365	0.184336163	-5.42487153	0.801828677	4.349817565	-2.439589
Fdx1	Fdx1-201	1451	2.04229E-05	0.000256597	0.184366697	-5.423973059	2.824738073	15.32130321	-2.43935
Gm37606	Gm37606-001	2570	9.84388E-06	0.000152938	0.184458916	-5.421261383	3.667845958	19.88435165	-2.438629
Slc44a1	Slc44a1-006	13033.501	2.48867E-07	1.58433E-05	0.184482054	-5.420581454	22.91054546	124.1884778	-2.438448
Cfap54	Cfap54-004	578.1279	2.78844E-05	0.000319305	0.184536871	-5.418971261	1.118445204	6.060822416	-2.438019
Tmem202	Tmem202-201	169	0.032896979	0.070337382	0.184547897	-5.41864751	0.462077612	2.503835701	-2.437933
Cnst	Cnst-002	394.59348	0.008770052	0.024833064	0.184571862	-5.417943942	0.844727878	4.57668829	-2.437745
Gm22625	Gm22625-201	142	0.00408918	0.013642551	0.184571994	-5.417940056	0.25009238	1.354985523	-2.437744
Mapk8ip3	Mapk8ip3-00	1676.1366	0.048354526	0.095559548	0.184638994	-5.415974045	2.465778776	13.35459385	-2.437221
Ahctf1	Ahctf1-011	472.48802	0.015065124	0.037982892	0.184726348	-5.413412919	0.819542324	4.436521006	-2.436538
Mpp3	Mpp3-003	231.93464	0.007275045	0.021470942	0.184767738	-5.412200249	0.410190305	2.22003207	-2.436215
Gm13697	Gm13697-001	82.428559	0.028174167	0.062087583	0.184796726	-5.411351272	0.172797937	0.935070337	-2.435989
Fam49a	Fam49a-201	1436.4168	0.017435513	0.042606044	0.184806924	-5.411052667	3.648856862	19.74415666	-2.435909
Sec24a	Sec24a-001	2844.1452	0.000284196	0.001734135	0.184828013	-5.410435278	6.174180311	33.40500297	-2.435745
Gm38099	Gm38099-001	2178	3.0604E-06	7.16954E-05	0.184836118	-5.410198027	4.291365054	23.21713475	-2.435681
Erg	Erg-001	14161.359	0.000207272	0.001368889	0.184837476	-5.410158261	26.72011417	144.5600464	-2.435671
030034I22R	0034I22Rik-	400.94955	0.003806578	0.012913518	0.184863202	-5.409405377	0.896668926	4.85044571	-2.43547
Stk40	Stk40-201	1298.9951	0.015658118	0.039159587	0.184888456	-5.408666511	2.405812502	13.01223751	-2.435273
Smarcc1	Smarcc1-001	4666.4451	0.000201054	0.00133893	0.184912367	-5.407967109	10.44301492	56.47548123	-2.435086
Agfg1	Agfg1-001	9948.1545	6.62578E-07	2.73084E-05	0.184955837	-5.406696091	17.63865967	95.36687228	-2.434747
Gm17383	Gm17383-201	4289	0.000122691	0.000931536	0.18495836	-5.406622341	9.469609649	51.19860308	-2.434728
Ankrd12	Ankrd12-008	145.69118	0.030586578	0.0663759	0.18496167	-5.406525573	0.253117957	1.368488705	-2.434702
Mapkapk5	Mapkapk5-00	520.44148	0.004802077	0.015493657	0.185011164	-5.405079237	0.985835575	5.328519396	-2.434316
Nup155	Nup155-201	8685	7.34289E-07	2.90274E-05	0.185015805	-5.404943653	19.45098868	105.1314978	-2.43428
Calcrl	Calcrl-002	2008.2311	0.041354716	0.08435968	0.185020667	-5.404801605	1.805348363	9.757549728	-2.434242
Sema6d	Sema6d-202	2988.5273	0.001676296	0.006807342	0.185024238	-5.404697315	5.498536114	29.71792337	-2.434214
Meis2	Meis2-005	6072.8449	4.6272E-05	0.000455407	0.185098894	-5.402517432	10.66529133	57.61942235	-2.433632
Mrpl30	Mrpl30-007	437.97089	0.00601827	0.018498228	0.185183848	-5.400038987	0.723967532	3.9094529	-2.43297
Gm13351	Gm13351-001	453	0.00435688	0.014362653	0.185184972	-5.400006228	0.744156201	4.018448122	-2.432961
Gm12976	Gm12976-002	4972.1551	3.19172E-08	4.63205E-06	0.18520494	-5.399424	9.222351897	49.79538816	-2.432806
Ptgr2	Ptgr2-004	415.58745	0.001150209	0.005087777	0.18522193	-5.398928708	0.907756762	4.900914041	-2.432673
Faim	Faim-001	1628.0707	0.005833901	0.018043636	0.185230262	-5.398685874	3.663040394	19.77560443	-2.432608
Gnpat	Gnpat-006	687.84587	0.000339358	0.001984804	0.185284554	-5.397103963	1.28665556	6.944213823	-2.432185
Fkbp1a	Fkbp1a-001	50470.623	0.000235667	0.001507864	0.18536559	-5.394744506	111.3580362	600.7481538	-2.431555
Zfc3h1	Zfc3h1-004	476.64647	0.004559467	0.014889564	0.185384439	-5.394196011	0.965077863	5.205819157	-2.431408
Brd8	Brd8-002	2218.8994	0.007630635	0.022307037	0.185412713	-5.39337342	4.869267113	26.26177582	-2.431188
10037L13R	0037L13Rik-	879.26231	0.003074383	0.010956412	0.18543021	-5.392864365	1.071063654	5.776101014	-2.431052
Gm37285	Gm37285-001	978	0.001382849	0.00587376	0.18546134	-5.391959323	1.663524264	8.969655166	-2.43081
Ssfa2	Ssfa2-002	53422.006	3.13192E-05	0.000345469	0.185506846	-5.39063664	90.32985547	486.9354286	-2.430456
Ahsa2	Ahsa2-008	554.48923	0.03496605	0.073856751	0.185547789	-5.389447146	1.188461464	6.405150247	-2.430137
Fam98b	Fam98b-001	2929.4685	1.42849E-06	4.3567E-05	0.185556343	-5.389198695	6.686826227	36.03663517	-2.430071
Cisd2	Cisd2-003	935.42396	0.030033033	0.065388811	0.185562853	-5.389009623	1.97837036	10.66145691	-2.43002
Agpat5	Agpat5-001	1608.9016	1.35616E-05	0.000191461	0.18561285	-5.387558035	4.298712631	23.15956378	-2.429632
Tomm22	Tomm22-001	5605.4185	8.54534E-07	3.19847E-05	0.185619698	-5.387359261	11.67543413	62.8997582	-2.429578
Caprin1	Caprin1-016	1341.6511	0.000655061	0.003291158	0.185623041	-5.387262238	2.824203524	15.214725	-2.429552
130010J15R	0010J15Rik-	1628.7805	0.001528607	0.00634219	0.185639203	-5.386793221	2.823821888	15.2113446	-2.429427
Ehmt1	Ehmt1-005	2900.3311	1.76603E-05	0.000231041	0.185693082	-5.385230249	5.315850246	28.62707754	-2.429008
Arl6ip1	Arl6ip1-201	6133	2.35555E-06	6.0223E-05	0.185701134	-5.384996722	12.00756967	64.66072329	-2.428945
430218F22R	0218F22Rik-	239.64441	0.003262277	0.011469106	0.18571266	-5.384662516	0.45127243	2.429949741	-2.428856
Dnajb2	Dnajb2-002	1079.0017	0.04455719	0.089506379	0.185755676	-5.383415565	2.140292367	11.52208324	-2.428522
Csnk1g1	Csnk1g1-001	9124.6219	3.07385E-06	7.1863E-05	0.185756456	-5.383392978	17.14738875	92.31113218	-2.428516
Ctnnb1	Ctnnb1-002	77591.476	3.2349E-10	4.75112E-07	0.185846153	-5.380794719	136.9196161	736.7363475	-2.427819
Xpo6	Xpo6-019	1375.2322	2.80935E-05	0.000320486	0.185904774	-5.379098005	2.806502194	15.09645035	-2.427364
Etfa	Etfa-001	6031.2158	1.43929E-06	4.38184E-05	0.185917261	-5.378736736	11.56108564	62.18403605	-2.427267
Nucks1	Nucks1-002	19885.378	5.57762E-08	6.76573E-06	0.185920276	-5.37864949	39.72877977	213.687181	-2.427244
Rell1	Rell1-002	1745.8674	0.002121013	0.008191618	0.185939282	-5.378099729	3.158690594	16.98775303	-2.427097
Mospd1	Mospd1-001	874.52798	0.021890717	0.050979605	0.185957441	-5.377574543	1.482933042	7.974582975	-2.426956
Rps4x	Rps4x-002	444.16578	0.001427465	0.006023988	0.185972417	-5.377141492	0.960135749	5.162785774	-2.426839
P23-336O1	23-336O18.1-	391	0.002818099	0.010234497	0.185992913	-5.376548951	0.943462694	5.07257336	-2.42668
Gm38251	Gm38251-001	187	0.014645924	0.037195825	0.186002278	-5.376278237	0.315251442	1.694879468	-2.426608
Luc7l3	Luc7l3-008	715.07683	0.000219689	0.001432116	0.186019538	-5.375779387	1.139015455	6.123095805	-2.426474
Clcn3	Clcn3-009	955.48322	4.35361E-05	0.000436764	0.186026868	-5.375567577	1.879301054	10.10230981	-2.426417
Nsun6	Nsun6-004	361.02693	0.013258112	0.034400784	0.186101544	-5.373410547	0.463345353	2.489744807	-2.425838
Tspan6	Tspan6-001	14734.81	1.70588E-07	1.28977E-05	0.186114541	-5.373035309	22.78116714	122.4040154	-2.425737
Nus1	Nus1-001	10603.844	2.27672E-06	5.92085E-05	0.186129213	-5.372611774	24.28548635	130.4764899	-2.425624
Uhmk1	Uhmk1-003	5704.9041	0.000346516	0.002015654	0.186132748	-5.372509731	10.92375176	58.68796264	-2.425596
Gm12940	Gm12940-001	450.26981	0.002445759	0.009165911	0.186149257	-5.372033248	0.981763403	5.274065644	-2.425468
Copa	Copa-004	3347.2499	2.031E-06	5.46439E-05	0.186161698	-5.371674251	7.064259982	37.94690345	-2.425372
Psme1	Psme1-011	488.36165	0.043691373	0.088123758	0.186179803	-5.371151885	0.962183347	5.168032897	-2.425232
Gm15443	Gm15443-001	382	0.003563656	0.012279197	0.186214004	-5.370165388	0.800818083	4.300525552	-2.424967
Ppp1r3e	Ppp1r3e-003	285.38485	0.004404605	0.014484658	0.186219893	-5.369995565	0.544349687	2.923155406	-2.424921
Ppm1g	Ppm1g-003	2200.6485	0.024262461	0.055182237	0.186221061	-5.369961888	3.977350679	21.35822156	-2.424912
Cep192	Cep192-201	5788	2.1238E-06	5.65732E-05	0.186238313	-5.36946444	13.26030034	71.20071114	-2.424778
Gm37960	Gm37960-001	1728	3.28683E-06	7.47304E-05	0.186257167	-5.368920923	3.124527834	16.77534286	-2.424632
30159M07R	0159M07Rik-	554.71864	0.011759652	0.031321881	0.186297417	-5.367760947	1.253045225	6.726047225	-2.42432
Dusp1	Dusp1-003	2485.5444	0.016047651	0.039892527	0.186307976	-5.367456722	5.616126392	30.14431535	-2.424239
Gm3272	Gm3272-001	2314	3.25716E-05	0.00035494	0.186315318	-5.367245223	4.960360792	26.62347277	-2.424182
Fam171b	Fam171b-001	207.5686	0.02317779	0.053270655	0.18634194	-5.366478431	0.337154441	1.809332038	-2.423976
Gm17106	Gm17106-001	614.14111	0.000733969	0.003586617	0.18640614	-5.364630144	1.138079378	6.105374938	-2.423479
Usp16	Usp16-001	3282.9768	8.50634E-05	0.000709761	0.186431219	-5.363908492	6.419339065	34.43274733	-2.423285
Brwd1	Brwd1-004	683.86938	0.000489882	0.002632123	0.186527733	-5.361133091	1.296884725	6.952771613	-2.422538
Rnf31	Rnf31-002	1174.6357	5.03023E-05	0.000482727	0.186590798	-5.359321097	2.170107635	11.63030363	-2.42205
Ifngr1	Ifngr1-002	263.0516	0.009043191	0.025475213	0.186612995	-5.358683627	0.595392523	3.190520165	-2.421879
Ndufb5	Ndufb5-008	725.26026	0.000129844	0.000971206	0.186681779	-5.356709182	1.280559141	6.859582907	-2.421347
Luc7l3	Luc7l3-002	7492.3984	4.03865E-05	0.0004134	0.1867449	-5.354898593	13.87212963	74.28384745	-2.420859

Foxj2	Foxj2-201	2025.2328	0.001630304	0.006664826	0.186766107	-5.354290539	4.14020799	22.16787647	-2.420695
Nyx	Nyx-001	303	0.00206916	0.008039561	0.186779129	-5.35391725	0.590438168	3.161157091	-2.420595
Gm37423	Gm37423-002	1723	1.54304E-06	4.55256E-05	0.18681391	-5.352920445	3.32750017	17.81184369	-2.420326
Prex2	Prex2-004	874.96355	0.00010403	0.000820331	0.186846737	-5.351979994	1.738248137	9.303069255	-2.420073
Rbms1	Rbms1-001	17036.356	9.00999E-09	2.44914E-06	0.186869907	-5.351316424	33.76843571	180.7055846	-2.419894
Tnpo3	Tnpo3-010	1197.5462	5.33021E-06	0.000101535	0.186871028	-5.351284317	2.348140631	12.56556813	-2.419885
Lsm8	Lsm8-001	1632.5341	2.48848E-05	0.000294507	0.186903544	-5.350353348	3.635541275	19.45143043	-2.419634
Paf1	Paf1-009	329.30641	0.003375665	0.011773578	0.186921393	-5.349842434	0.647239974	3.462631877	-2.419496
Psma6	Psma6-002	1354.8248	0.032590967	0.069804303	0.187011278	-5.347271102	3.047793615	16.29737872	-2.418803
Mga	Mga-202	5694.0794	3.30386E-07	1.83224E-05	0.187038844	-5.346482992	10.98859295	58.75032529	-2.41859
Gm12668	Gm12668-001	340	0.01234767	0.032503858	0.187041896	-5.346395771	0.617535026	3.30158665	-2.418567
Samhd1	Samhd1-003	4277.9668	0.0001418	0.001038724	0.187089303	-5.345041013	11.18512866	59.78497141	-2.418201
Pde4d	Pde4d-002	506.53629	0.03453881	0.07317092	0.18709601	-5.344849405	1.318783313	7.048698207	-2.418149
Pls3	Pls3-001	1350.1662	0.049173697	0.096763053	0.187134851	-5.343740053	3.092706254	16.52661828	-2.41785
P23-50E10.	23-50E10.7-0	459	0.000208722	0.001375551	0.187151276	-5.343271086	0.970220982	5.184153718	-2.417723
Amz2	Amz2-201	1135.5446	0.01495126	0.037789141	0.187268646	-5.339922202	2.077808976	11.09533828	-2.416819
Ccbl2	Ccbl2-008	1018.993	3.431E-05	0.000366695	0.187287188	-5.339393532	1.962989136	10.4811715	-2.416676
Gm17259	Gm17259-201	909	0.000504154	0.002690025	0.187333864	-5.338063166	1.791896796	9.565258282	-2.416316
Pbrm1	Pbrm1-013	784.39363	9.59935E-05	0.000774324	0.187335432	-5.338018503	1.41897412	7.574510108	-2.416304
Tasp1	Tasp1-201	472.50875	0.001801002	0.007211167	0.1873454 7	-5.337732468	1.039594341	5.54907647	-2.416227
Smarcb1	Smarcb1-003	3199.0551	1.80838E-07	1.33171E-05	0.18739504	-5.336320533	6.010126483	32.07196136	-2.415845
Ythdf1	Ythdf1-001	5677.7755	1.80459E-05	0.000234118	0.187398959	-5.336208941	11.68527697	62.35507945	-2.415815
Gpx8	Gpx8-201	792	9.23245E-05	0.000752854	0.187410798	-5.335871849	1.948232446	10.39551866	-2.415724
Gm5620	Gm5620-001	2518	0.000100993	0.00080274	0.1874991	-5.333358937	5.577161757	29.7450055	-2.415044
Smg1	Smg1-001	37133.486	9.69441E-09	2.51618E-06	0.187511897	-5.332994951	72.07430569	384.3719083	-2.414946
Pbx1	Pbx1-007	7916.8733	1.24432E-06	4.00771E-05	0.187561538	-5.331583485	14.46912585	77.14335241	-2.414564
Trps1	Trps1-006	3348.2337	6.02111E-05	0.000552059	0.187576098	-5.331169635	6.253211959	33.33693372	-2.414452
30308A19R	0308A19Rik-	716.76298	0.005187872	0.016472	0.187623027	-5.32983619	1.93184781	10.29643237	-2.414091
30111J21Ri	0111J21Rik1-	1620.7171	9.94574E-05	0.000794777	0.18765717	-5.328866464	2.080268084	11.08547083	-2.413829
P23-418O21	23-418O21.5-	3131	1.06806E-05	0.000162121	0.187672317	-5.328436364	5.65032312	30.10738718	-2.413712
Nptn	Nptn-012	1184.3052	0.045048106	0.090233208	0.187677087	-5.328300955	1.471267814	7.839357696	-2.413676
Gm12031	Gm12031-001	286	0.001029335	0.004660241	0.187696492	-5.327750067	0.516121877	2.749768365	-2.413526
Tk1	Tk1-007	572.9473	0.00466631	0.015145441	0.187725663	-5.326922194	1.330716013	7.088620662	-2.413302
Zfp27	Zfp27-004	460.92616	0.01858411	0.044836981	0.187746788	-5.326322805	0.744847588	3.967298694	-2.41314
00049G17R	0049G17Rik-	507.42168	0.006963241	0.020736545	0.187758261	-5.32599736	0.914614609	4.871234995	-2.413052
Jak1	Jak1-008	154.40699	0.028228509	0.062175333	0.187769068	-5.325690806	0.375330634	1.998894905	-2.412969
Gm20633	Gm20633-001	376.35315	0.034115712	0.07244486	0.187773738	-5.325558373	0.60245913	3.208431265	-2.412933
Clip1	Clip1-014	1818.5991	2.84317E-05	0.000322659	0.187782984	-5.325296141	2.769007626	14.74578562	-2.412862
Gpcpd1	Gpcpd1-003	2466.1806	0.012371492	0.032551556	0.187865643	-5.322953051	6.222362928	33.12134573	-2.412227
Nudt7	Nudt7-001	853.54529	0.006684714	0.020062344	0.187885629	-5.32238685	1.808872653	9.627520023	-2.412073
Gm37080	Gm37080-001	749	0.000197312	0.001319342	0.187933989	-5.321017271	1.402294999	7.461635909	-2.411702
Wfdc17	Wfdc17-001	1690	0.04614022	0.09205467	0.187934166	-5.321012259	5.643974368	30.03165681	-2.411701
030040B21R	0040B21Rik-	434	0.000164692	0.001157034	0.187975405	-5.319844915	0.762472364	4.056234731	-2.411384
Timm21	Timm21-201	1637	0.000436577	0.002409014	0.188012768	-5.318787717	3.419715046	18.18873838	-2.411097
Gm37412	Gm37412-001	282	0.007832655	0.022750037	0.18811098	-5.316010795	0.559376234	2.973650098	-2.410344
Parp14	Parp14-003	3316.7859	0.00012614	0.000951673	0.188112058	-5.315980342	5.543833408	29.47090942	-2.410336
P23-24K16	23-24K16.2-0	1923	3.14287E-06	7.27812E-05	0.188146353	-5.315011352	3.895545123	20.70486656	-2.410073
Sfswap	Sfswap-003	2486.2776	2.19934E-06	5.81321E-05	0.18817647	-5.314160692	4.571954107	24.2960988	-2.409842
Xpa	Xpa-005	588.76378	0.003339516	0.011679609	0.188267964	-5.311578122	1.060450086	5.632663477	-2.409141
10005L07R	0005L07Rik-	3474.2789	3.46157E-06	7.70362E-05	0.188276862	-5.3113271	7.407458625	39.34343573	-2.409072
Abcb4	Abcb4-003	870	2.60022E-05	0.000304045	0.188316776	-5.31020135	1.472431437	7.818907406	-2.408767
Fktn	Fktn-001	4566.0909	5.69747E-06	0.000106227	0.188319772	-5.310116872	8.282944382	43.98340271	-2.408744
Thoc7	Thoc7-201	3838.7379	1.39319E-06	4.29107E-05	0.188340428	-5.309534513	7.973559497	42.33588934	-2.408585
Micu1	Micu1-009	155.4414	0.02199384	0.051157165	0.188363144	-5.308894191	0.280768693	1.490571283	-2.408411
Commd6	Commd6-201	837.35483	0.002293273	0.00871749	0.188389129	-5.308161925	1.714296307	9.099762387	-2.408212
Hnrnpa3	Hnrnpa3-001	31125.734	1.13434E-08	2.8153E-06	0.188397043	-5.307938923	59.28790652	314.6965867	-2.408152
Dynlt1f	Dynlt1f-201	641	0.000495187	0.002652513	0.188402912	-5.307773573	1.635968454	8.683350125	-2.408107
BC003331	BC003331-00	3758.1928	0.002346849	0.008872897	0.188430198	-5.307004993	7.889344161	41.86878885	-2.407898
P23-134H1	23-134H19.3-	2362.468	0.001612682	0.006608563	0.188434129	-5.306894269	4.301563919	22.82794491	-2.407868
Dlc1	Dlc1-001	263.58171	0.031302235	0.06761488	0.188470375	-5.305873665	0.358038943	1.899709397	-2.40759
Gbf1	Gbf1-004	685.71907	0.000358304	0.002068065	0.188490596	-5.305304464	1.272769221	6.752428232	-2.407436
C3ar1	C3ar1-001	1693	0.025276486	0.056981481	0.188547952	-5.303690585	5.650549873	29.96876816	-2.406997
Hcfc2	Hcfc2-001	4507.5592	1.32546E-06	4.1725E-05	0.188588606	-5.302547273	8.481010093	44.97095694	-2.406686
H2afz	H2afz-006	1495.5196	0.037679347	0.078355217	0.188646597	-5.300917237	3.686358191	19.54107968	-2.406242
Suclg1	Suclg1-002	535.09112	0.000314862	0.001873528	0.188692032	-5.299640841	1.059692743	5.61599094	-2.405895
Stk32a	Stk32a-201	445	0.006677701	0.020046517	0.188725591	-5.29869848	1.169257642	6.195543689	-2.405638
Mcmbp	Mcmbp-004	505.4663	0.007690062	0.022423347	0.188767916	-5.297510417	0.894868991	4.740577803	-2.405315
Top1	Top1-005	1200.6357	5.71364E-05	0.000531484	0.188769644	-5.297461917	2.364575844	12.52625049	-2.405301
AW554918	W554918-20	1911.5304	0.000915287	0.004256357	0.188771921	-5.297398025	3.545400852	18.78139947	-2.405284
Gm16754	Gm16754-001	711.15324	0.000780481	0.003753894	0.188807989	-5.296386045	1.073070108	5.683393545	-2.405008
Rfx3	Rfx3-201	2365.5831	1.0503E-05	0.000159866	0.188829985	-5.295769089	4.855982157	25.71616021	-2.40484
Kat2a	Kat2a-004	2184.916	0.043150584	0.087248804	0.18885291	-5.295126248	5.114064421	27.07961675	-2.404665
Nr2c2	Nr2c2-005	250.30279	0.007291757	0.021507291	0.188887545	-5.294155326	0.452070857	2.393333335	-2.404401
Rhbdd2	Rhbdd2-003	2601.1461	3.30246E-05	0.000357881	0.188890136	-5.294082688	4.824571311	25.54167946	-2.404381
Me2	Me2-201	2248	0.000230236	0.001481976	0.18890071	-5.293786353	5.352348005	28.33418683	-2.4043
Atp6ap1	Atp6ap1-001	22728.967	9.06109E-08	8.98218E-06	0.188936967	-5.292770461	34.72109398	183.7707806	-2.404023
Pld1	Pld1-008	2490.7981	7.4451E-06	0.000126423	0.188940395	-5.292674452	4.22148008	22.34291977	-2.403997
P23-201C9	23-201C9.2-0	427	0.000309911	0.001852111	0.189176587	-5.286066401	0.821574719	4.342898516	-2.402195
Tsn	Tsn-001	6144.8309	4.33633E-07	2.13117E-05	0.189187249	-5.285768504	12.00830303	63.47310997	-2.402113
Slc5a4b	Slc5a4b-001	373	0.002099808	0.008129131	0.18919677	-5.28550249	0.539967057	2.853997227	-2.402041
Gm26624	Gm26624-201	1881	2.03741E-05	0.000256172	0.189232174	-5.284513612	3.816911505	20.1705208	-2.401771
Wac	Wac-011	1061.1231	0.002518096	0.009364254	0.1892391	-5.284320209	1.940030779	10.25174385	-2.401718
Setd5	Setd5-002	6012.6381	8.68677E-05	0.000719383	0.189239924	-5.284297184	12.09147065	63.89492428	-2.401712
Slc2a4rg-ps	lc2a4rg-ps-00	1778.4656	7.46629E-05	0.000647591	0.189278798	-5.283211913	3.188133224	16.84358343	-2.401415
Pphln1	Pphln1-204	913.92924	0.015855838	0.039537523	0.189291511	-5.282857095	1.573146678	8.310709091	-2.401318
Foxo1	Foxo1-001	23667.691	2.57045E-08	4.13324E-06	0.18931669	-5.282154455	49.75033982	262.7889791	-2.401126
Pcdhb16	Pcdhb16-001	1936	1.86143E-05	0.000239497	0.189323489	-5.281964767	3.957127619	20.90140866	-2.401075
Gm10291	Gm10291-001	488	0.000593696	0.003049502	0.189388188	-5.280160358	1.129509408	5.963990798	-2.400582
Dock5	Dock5-201	3552	0.001959098	0.007714314	0.189390972	-5.280082718	10.4168738	55.00195532	-2.400561
Nfat5	Nfat5-003	5833.5271	4.51321E-07	2.19259E-05	0.189401109	-5.279800141	10.29030849	54.33077223	-2.400483
Siah1a	Siah1a-201	3024.6417	1.0328E-05	0.000157987	0.189411848	-5.279500773	5.595056669	29.53910601	-2.400402
Jmjd1c	Jmjd1c-005	1102.8342	0.000130968	0.000978121	0.189449774	-5.278443893	2.22429275	11.74080448	-2.400113
Csnk1a1	Csnk1a1-001	13541.752	1.425E-07	1.16597E-05	0.189455944	-5.278271988	28.30501656	149.401576	-2.400066
Ythdc1	Ythdc1-006	2018.8433	4.11294E-05	0.000418255	0.189463408	-5.278064044	4.587475193	24.21298787	-2.400009
Gm13684	Gm13684-001	494.44353	0.001128397	0.00501006	0.189472742	-5.277804037	0.992418615	5.237790973	-2.399938
Copb2	Copb2-003	556.22597	0.000645003	0.003253034	0.189530319	-5.276200677	1.184505618	6.249689347	-2.399499
Acad12	Acad12-003	794.8595	2.18887E-05	0.000269188	0.189553829	-5.275546295	1.30388544	6.878708	-2.39932
Msn	Msn-002	2850.269	4.54423E-06	9.19315E-05	0.189668991	-5.27234312	7.242159607	38.18315038	-2.398444
Srcap	Srcap-004	1190.5452	7.68705E-05	0.000661246	0.189738969	-5.270398617	2.381856564	12.55333354	-2.397912
Psma8	Psma8-201	283	0.004437341	0.014564344	0.189761841	-5.269763374	0.504063517	2.656295461	-2.397738
Fam53a	Fam53a-006	2389.6046	9.1911E-05	0.000750406	0.189768424	-5.269580571	4.88248572	25.72865189	-2.397688
Tmtc1	Tmtc1-003	14895.456	0.001122544	0.004986657	0.189832601	-5.267799065	22.91795126	120.7271622	-2.3972
Hivep3	Hivep3-001	1213.8168	0.005837666	0.01805002	0.189849341	-5.267334574	3.173743681	16.71716982	-2.397073
Zzef1	Zzef1-003	1647.764	5.62451E-06	0.000105325	0.189855512	-5.267163381	3.14848036	16.58356046	-2.397026
Csnk1a1	Csnk1a1-008	4992.4799	2.09729E-07	1.46596E-05	0.189947368	-5.264616255	10.02956288	52.80179978	-2.396328
Map4	Map4-017	2786.975	5.41209E-07	2.43727E-05	0.189969129	-5.264013192	5.326397913	28.03822888	-2.396163
Exoc1	Exoc1-012	1211.0229	0.000138702	0.001022065	0.19002262	-5.262531374	2.227089503	11.72012838	-2.395757

Map3k12	Map3k12-002	2870.7213	0.00017445	0.001203889	0.190029137	-5.262350907	4.735757318	24.92121682	-2.395707
Tmc4	Tmc4-004	987.95881	3.49907E-05	0.000372068	0.190094901	-5.260530375	1.937541703	10.19249698	-2.395208
Anp32b	Anp32b-001	38402	4.17627E-06	8.69527E-05	0.190127005	-5.259642101	86.33679307	454.1006317	-2.394965
Pds5a	Pds5a-011	2086.2114	0.000172165	0.001193173	0.190148596	-5.259044891	3.448904564	18.13794393	-2.394801
Adprhl2	Adprhl2-002	283.36703	0.044901203	0.089994779	0.190165399	-5.258580192	0.468429715	2.463275219	-2.394673
P24-496C22	24-496C22.4-	356	0.003276003	0.011506831	0.190174232	-5.258335958	0.585797657	3.080320881	-2.394606
Ice2	Ice2-001	1335.6813	0.000132788	0.000988045	0.190252476	-5.256173368	2.643252619	13.89339402	-2.394013
Sec61a1	Sec61a1-002	2232.6043	2.08096E-05	0.000260025	0.190278077	-5.255466175	4.28494406	22.51937857	-2.393819
Trim25	Trim25-001	14659.835	2.71504E-08	4.32512E-06	0.190279632	-5.25542325	27.0470834	142.1438709	-2.393807
Mllt10	Mllt10-009	1116.4292	0.001031981	0.004666874	0.190357496	-5.25327355	2.034613943	10.68838361	-2.393217
Gm13899	Gm13899-001	186	0.013925523	0.035745271	0.190370717	-5.252908718	0.516324678	2.712206403	-2.393117
Mid1	Mid1-006	336.1117	0.005589334	0.017460296	0.19037839	-5.252697001	0.86071217	4.521060236	-2.393058
Ppp2r1b	Ppp2r1b-002	2823.577	2.95195E-06	6.98235E-05	0.190476392	-5.249994457	6.464523769	33.93871396	-2.392316
Noc2l	Noc2l-202	955.83523	0.003217472	0.011354705	0.190488099	-5.249671783	2.033522382	10.67532507	-2.392227
Mcm5	Mcm5-201	1741	0.039930347	0.081978409	0.19051174	-5.249020348	4.614254117	24.22031375	-2.392048
Zfp868	Zfp868-002	579.5166	0.023520832	0.053871104	0.190518451	-5.248835451	1.328118404	6.971074963	-2.391997
Gm17529	Gm17529-201	1054.229	0.000132375	0.000986047	0.190582661	-5.247067043	1.887929084	9.906090474	-2.391511
Zcchc8	Zcchc8-002	1802.2982	3.69651E-05	0.000387409	0.190656813	-5.245026314	3.443768038	18.06265398	-2.39095
Gm16059	Gm16059-001	1675.6031	0.000152931	0.001096918	0.19073493	-5.242878156	3.351986525	17.57405693	-2.390359
Tmem180	Tmem180-004	952.22841	1.68426E-05	0.000223329	0.19076743	-5.241984955	1.906190827	9.992223635	-2.390113
Ttll5	Ttll5-012	294.03913	0.045832434	0.091541921	0.190808995	-5.240843062	0.665880316	3.489774235	-2.389799
Ccdc79	Ccdc79-003	276.52342	0.00824444	0.023643311	0.190894359	-5.23849949	0.477978959	2.503892535	-2.389154
Gm16617	Gm16617-201	548.01228	0.000254984	0.001597803	0.190924038	-5.237685164	0.987832882	5.173957629	-2.388929
L1cam	L1cam-004	275.48261	0.027564632	0.06106195	0.19095948	-5.236713033	0.352884573	1.847955241	-2.388662
Trmt10b	Trmt10b-001	1045.6848	0.003224651	0.011370769	0.190974317	-5.236306198	2.129494541	11.15068547	-2.388549
Rdh9	Rdh9-003	323.12	0.009939502	0.027440588	0.19104519	-5.234363653	0.719768564	3.767530411	-2.388014
Lrrc1	Lrrc1-004	1403.8421	0.002003715	0.007845802	0.191049703	-5.234240012	1.513950561	7.924380604	-2.38798
21507L20R	1507L20Rik-	158.05751	0.017749734	0.04321106	0.191120144	-5.232310844	0.196699525	1.029193057	-2.387448
Jarid2	Jarid2-001	3281.8356	0.00103937	0.004691411	0.191121331	-5.232278344	6.405187971	33.51372631	-2.387439
Kmt2b	Kmt2b-006	364.36037	0.005326581	0.016843819	0.191151505	-5.231452392	0.810595691	4.240592766	-2.387212
Zfp458	Zfp458-201	749	0.000287123	0.001748258	0.191162304	-5.231156864	1.272437533	6.656320337	-2.38713
Sla2	Sla2-002	270.89176	0.008438868	0.024089594	0.191182793	-5.230596251	0.484476595	2.534101463	-2.386975
Prex2	Prex2-003	7276.0609	5.1959E-06	9.99959E-05	0.191242577	-5.228961112	15.12369565	79.08121642	-2.386524
Apc	Apc-201	17944.775	2.47824E-08	4.03392E-06	0.191285133	-5.227797825	34.04829982	177.9976277	-2.386203
Myo5a	Myo5a-001	6536.1134	9.57177E-07	3.40783E-05	0.191332186	-5.226512166	15.11784673	79.01360984	-2.385849
Rpl30-ps1	Rpl30-ps1-001	798	0.000232527	0.001492231	0.191403604	-5.224562007	1.834748962	9.58575972	-2.38531
Rb1cc1	Rb1cc1-011	6271.4556	7.36508E-07	2.90615E-05	0.191494565	-5.222080334	11.57059937	60.42259944	-2.384625
Ick	Ick-002	1083.009	6.98466E-05	0.000617035	0.191540998	-5.220814386	1.341147757	7.001883501	-2.384275
Cnbp	Cnbp-001	35267.741	7.75483E-09	2.31621E-06	0.191577896	-5.219808855	74.70929641	389.9682469	-2.383997
Tvp23b	Tvp23b-001	2511.3291	5.50612E-07	2.4488E-05	0.191604193	-5.219092476	4.59661725	23.99017051	-2.383799
Srebf1	Srebf1-006	1280.3047	0.014408774	0.036729719	0.191628061	-5.218442407	2.08786085	10.8953816	-2.383619
Chm	Chm-003	1188.8959	8.61848E-05	0.000715869	0.191648239	-5.217892978	2.306012925	12.03252865	-2.383467
Isca1	Isca1-002	3855.3273	1.13553E-06	3.79804E-05	0.191673924	-5.217193766	7.789416492	40.63889516	-2.383274
Gm36401	Gm36401-001	807	0.000755504	0.003665137	0.191689577	-5.21676774	1.79033456	9.339759575	-2.383156
Atpaf1	Atpaf1-002	1361.0555	0.008089731	0.023322668	0.191756421	-5.214949231	2.746481776	14.32276303	-2.382653
Rif1	Rif1-002	348.55505	0.002713148	0.009937473	0.191775432	-5.214432265	0.823014269	4.29155216	-2.38251
Zfp518a	Zfp518a-201	5056.6706	3.26915E-06	7.45418E-05	0.191784544	-5.214184524	11.23673119	58.59038985	-2.382442
Traf3	Traf3-002	1111.4662	0.016146975	0.04008654	0.19180118	-5.213732249	2.626063886	13.69159397	-2.382316
Hp1bp3	Hp1bp3-002	1566.0122	0.016167295	0.040131167	0.191813387	-5.213400472	3.121844261	16.27542434	-2.382225
Cdc14a	Cdc14a-007	805.7558	0.001755153	0.007068078	0.191813747	-5.213390675	1.593612086	8.308122389	-2.382222
Aftph	Aftph-002	5962.5308	2.41098E-06	6.1395E-05	0.19187621	-5.211693523	10.82388853	56.41078975	-2.381752
Sdhd	Sdhd-201	4873	4.43837E-06	9.0216E-05	0.191900416	-5.211036124	9.889425917	51.53415569	-2.38157
Slc25a43	Slc25a43-002	727.24271	0.003233151	0.011393029	0.191910799	-5.210754202	1.54454769	8.048258364	-2.381492
Stxbp4	Stxbp4-001	3127.5539	6.46949E-06	0.000115754	0.191912814	-5.210699473	6.52082837	33.97807695	-2.381477
Gm15787	Gm15787-001	301.01669	0.00758561	0.022196253	0.19191416	-5.210662934	0.645898518	3.365559467	-2.381467
Dnajb11	Dnajb11-001	8342.8816	3.81432E-05	0.00039576	0.191928986	-5.210260423	17.28864822	90.07835958	-2.381355
Brd1	Brd1-003	3255.8881	1.40183E-06	4.31263E-05	0.191942293	-5.209899206	6.608247045	34.42830103	-2.381255
Wdr36	Wdr36-201	1925.9808	0.000410647	0.00229397	0.191966415	-5.209244545	4.073181808	21.21820012	-2.381074
Ak6	Ak6-001	1076.1193	0.010558044	0.02877121	0.191989718	-5.20861226	2.533122591	13.19405338	-2.380899
Btf3l4	Btf3l4-001	6009.415	1.03054E-07	9.64322E-06	0.192015898	-5.207902108	11.98602058	62.42202187	-2.380702
Psmc4	Psmc4-003	1108.1634	0.000259305	0.001618197	0.192024553	-5.207667387	2.069149157	10.77544058	-2.380637
Cul3	Cul3-002	16254.207	4.63657E-08	5.97818E-06	0.192024966	-5.207656186	34.19975196	178.1005498	-2.380634
Ost4	Ost4-002	4548.84	2.76164E-05	0.000317084	0.192032039	-5.207464353	9.885075788	51.4761798	-2.380581
Gm37827	Gm37827-001	339	0.047309611	0.093934202	0.192046036	-5.207084821	0.47778984	2.487892222	-2.380476
Osgin2	Osgin2-005	711.07739	0.000342093	0.00199705	0.192060208	-5.206700597	1.469775545	7.652681208	-2.380369
Pnkd	Pnkd-005	375.42473	0.003309321	0.011597693	0.192077431	-5.206233739	0.760001903	3.95674755	-2.38024
Slc38a10	Slc38a10-001	641.87261	0.013046862	0.033963291	0.192116443	-5.205176541	1.302607346	6.780301201	-2.379947
Cpne3	Cpne3-001	23549.688	4.31256E-09	1.80128E-06	0.192161696	-5.203950734	43.25315677	225.0872969	-2.379607
Gm17258	Gm17258-201	201	0.001318019	0.005655385	0.1922174	-5.202442643	0.316464838	1.646390167	-2.379189
Trub2	Trub2-003	1570.6973	0.000496787	0.00265817	0.192227009	-5.202182598	3.516829695	18.29519024	-2.379117
Gm28237	Gm28237-001	283	0.004689872	0.015209557	0.192253434	-5.201467571	0.520707332	2.708442301	-2.378919
Casq1	Casq1-002	356.19969	0.015039282	0.037941618	0.192282263	-5.20068769	0.938158007	4.8790668	-2.378702
Gm20696	Gm20696-001	3669.757	0.00014354	0.001046904	0.192282889	-5.200670777	6.849968905	35.62443311	-2.378698
Neurl3	Neurl3-001	2666.1313	0.006215884	0.018972821	0.19229018	-5.200473584	9.008000535	46.84586883	-2.378643
Cd164	Cd164-001	22949.695	3.01558E-09	1.51021E-06	0.192404986	-5.197370508	44.9602772	233.6752187	-2.377782
Lman2l	Lman2l-001	840.62124	0.000608226	0.003111074	0.1924218	-5.196916367	1.54049324	8.005814534	-2.377656
Timm17a	Timm17a-001	6053.6217	1.66611E-07	1.26693E-05	0.192427268	-5.196768673	12.69878671	65.99265694	-2.377615
Rpl15-ps2	Rpl15-ps2-001	1924	2.69833E-05	0.00031159	0.192479702	-5.195353015	4.443166894	23.08382052	-2.377222
Eri1	Eri1-201	4192	9.60884E-05	0.00077445	0.192510172	-5.194530715	9.725837587	50.52116207	-2.376993
Pcgf2	Pcgf2-002	1668.8622	0.000554114	0.002897469	0.192605204	-5.191967702	3.617400627	18.78142722	-2.376281
Ccnb2-ps	Ccnb2-ps-002	458.36351	0.000268256	0.001659942	0.19264944	-5.190775545	0.776314937	4.029676589	-2.37595
Azi2	Azi2-008	281.78164	0.006493122	0.019600926	0.192657778	-5.19055088	0.534114706	2.772349559	-2.375888
Coro1c	Coro1c-002	2751.1057	0.001149494	0.005086586	0.192823729	-5.186083719	4.06954242	21.10498769	-2.374645
Spg20	Spg20-002	1940.0361	0.010208116	0.028037528	0.192839784	-5.185651933	3.861416535	20.02396212	-2.374525
Lrrc42	Lrrc42-005	914.09541	0.008319964	0.023825983	0.192857084	-5.185186771	1.520838008	7.885829119	-2.374396
Prkacb	Prkacb-003	1272.4886	0.048295265	0.095478631	0.192873676	-5.1847407	3.650185096	18.92526323	-2.374272
Gm37183	Gm37183-001	515.98825	0.000244053	0.001547928	0.192881593	-5.184527909	0.922178368	4.781059489	-2.374213
Camk2d	Camk2d-005	259.20378	0.030962326	0.067054042	0.192912196	-5.183705453	0.468213767	2.427082259	-2.373984
Rbm25	Rbm25-005	1666.6069	2.70463E-06	6.58376E-05	0.192928023	-5.1832802	3.540169308	18.34968948	-2.373865
Naip2	Naip2-201	1952.9478	0.048097487	0.095150403	0.192951549	-5.182648203	3.689706458	19.12245054	-2.373689
Rpl35a	Rpl35a-004	764.04519	0.009165226	0.025751502	0.193071919	-5.179417117	2.068003479	10.71105262	-2.37279
Ubl5	Ubl5-001	1706.1381	0.001163505	0.005132365	0.193085892	-5.179042285	3.535947073	18.31281941	-2.372685
Ttc17	Ttc17-003	1825.2645	0.010065229	0.027734795	0.193091861	-5.178882187	3.120252708	16.15942117	-2.372641
Gm13691	Gm13691-001	65	0.035317661	0.074438762	0.193108573	-5.178434009	0.132305249	0.685134004	-2.372516
00055D18R	0055D18Rik-	617.72028	0.001322815	0.00566897	0.193116436	-5.178223145	1.354243936	7.012577292	-2.372457
Saal1	Saal1-005	1653.4069	0.02844035	0.0625695	0.19320459	-5.175860482	3.416794774	17.68485304	-2.371799
030624J02R	0624J02Rik-0	2012.9642	0.000104044	0.000820331	0.193213386	-5.175624844	4.301403596	22.26245131	-2.371733
Fastkd3	Fastkd3-201	968	0.000841093	0.003982226	0.193221425	-5.175409513	2.266204306	11.72853532	-2.371673
P23-194K16	23-194K16.1-	124	0.032109258	0.06895367	0.193236071	-5.175017248	0.325298883	1.68342733	-2.371564
Gm20899	Gm20899-001	1258	6.35926E-05	0.00057529	0.193305873	-5.17314856	2.870891855	14.85155007	-2.371043
Arhgap31	Arhgap31-002	4043.4287	1.27242E-07	1.09317E-05	0.193334155	-5.172391805	7.250297804	37.50138095	-2.370832
Tbl3	Tbl3-005	352.9954	0.01648582	0.040750529	0.1933356	-5.172353149	0.893981361	4.623987308	-2.370821
30601H04R	0601H04Rik-	348.97219	0.022845189	0.052658168	0.193358853	-5.171731129	0.926894416	4.793648704	-2.370647
Terf1	Terf1-001	1166.4823	0.001169533	0.005152171	0.19337211	-5.171376574	2.112964889	10.92693713	-2.370548
Cox7a2l	Cox7a2l-202	655.99227	0.005903456	0.018220543	0.193389512	-5.170911221	1.395549806	7.216264151	-2.370419
Dgat1	Dgat1-007	225.93121	0.043720565	0.088156679	0.193511424	-5.167653562	0.603081126	3.116514327	-2.369509
Gm37397	Gm37397-001	200.58952	0.011461741	0.030707495	0.193516754	-5.167511227	0.323235145	1.670321239	-2.36947

Cc2d1b	Cc2d1b-007	262.43319	0.002090114	0.008103508	0.193524043	-5.167316592	0.604081939	3.121482624	-2.369415
Rab1	Rab1-004	863.36839	0.00345587	0.01198133	0.193645568	-5.164073781	1.831100988	9.455940602	-2.36851
Gm16587	Gm16587-001	1160.9973	0.000419185	0.002333727	0.19365771	-5.163749992	2.918018542	15.06791822	-2.368419
10403A07R	0403A07Rik-	6787.2599	0.000234302	0.001501096	0.193666513	-5.163515277	12.46347802	64.35535914	-2.368354
Gm37570	Gm37570-001	2798	2.89661E-06	6.90448E-05	0.193674469	-5.163303161	5.58760453	28.85049613	-2.368294
Gm6472	Gm6472-001	452.70356	0.049469088	0.097195746	0.193678292	-5.163201259	0.920967978	4.755143023	-2.368266
Tram2	Tram2-001	20115.569	1.92585E-06	5.24326E-05	0.193712008	-5.162302581	43.60326294	225.0932368	-2.368015
Fgfr1op2	Fgfr1op2-002	5071.7985	0.002330612	0.008827098	0.19373407	-5.161714703	8.818948413	45.52089569	-2.36785
Gm14749	Gm14749-001	89	0.027264889	0.060555246	0.193745537	-5.161409213	0.134261112	0.692976542	-2.367765
Gm8822	Gm8822-001	1402	0.000244278	0.001548497	0.193773896	-5.160653838	3.850644406	19.87184283	-2.367554
Gm24362	Gm24362-001	643	0.000176523	0.001215016	0.193809024	-5.159718478	1.296487221	6.68950907	-2.367292
Erich5	Erich5-201	82	0.024331922	0.055303493	0.193898563	-5.157335807	0.169301803	0.873146252	-2.366626
Ccndbp1	Ccndbp1-001	1892.4598	0.00799058	0.023104884	0.193901855	-5.157248244	3.426400782	17.67079942	-2.366601
Pde1c	Pde1c-206	315.00745	0.049519118	0.097258995	0.193985661	-5.155020206	0.335367767	1.728827618	-2.365978
Rab14	Rab14-002	2346.5268	3.304E-05	0.000357881	0.194036505	-5.153669404	5.162388753	26.60524497	-2.3656
Chd2	Chd2-009	2097.0049	2.24008E-06	5.86452E-05	0.19404271	-5.153504615	4.088100905	21.06804688	-2.365554
Clec16a	Clec16a-201	176.43306	0.025635167	0.057596724	0.194079189	-5.152535964	0.191645009	0.987457803	-2.365283
Clec16a	Clec16a-202	146.5545	0.017450363	0.042633207	0.194079189	-5.152535964	0.191645009	0.987457803	-2.365283
Usp9x	Usp9x-001	59768.463	9.53765E-10	8.82877E-07	0.194084896	-5.15238444	115.410399	594.6387441	-2.36524
Dmtf1	Dmtf1-013	1039.6882	0.003498851	0.012098502	0.194131226	-5.151154827	1.894552135	9.759131375	-2.364896
30017N08R	0017N08Rik-	3311	3.43406E-06	7.68412E-05	0.194239234	-5.148290473	5.253583578	27.04697429	-2.364093
Gtf2ird1	Gtf2ird1-012	353.33643	0.00964835	0.026794452	0.194242536	-5.148202972	0.726654169	3.740963152	-2.364069
Fam98a	Fam98a-001	3712.1806	6.02439E-05	0.000552142	0.194253076	-5.147923623	8.749249819	45.04046982	-2.363991
Gm26269	Gm26269-201	151	0.006484995	0.019586411	0.194254987	-5.147872993	0.303462915	1.562188547	-2.363976
00048O20R	0048O20Rik-	776	0.000534851	0.002816703	0.194320527	-5.146136724	1.49836825	7.710807878	-2.36349
P24-372J15	24-372J15.4-(	2092	1.83276E-05	0.000237233	0.194332516	-5.145819234	3.786983959	19.4871349	-2.363401
Eif2s1	Eif2s1-001	9504	1.28762E-05	0.000184189	0.194364465	-5.144973397	22.97826441	118.2225591	-2.363164
Tspan7	Tspan7-003	1884.8911	0.000403881	0.002266207	0.194398586	-5.144070331	4.346038234	22.35632634	-2.36291
Pp2d1	Pp2d1-201	530.60553	0.007515472	0.022036204	0.194490349	-5.141643288	0.96085498	4.940373562	-2.36223
Nfia	Nfia-201	11453.453	8.67823E-05	0.000719335	0.194495217	-5.141514611	19.20052859	98.71979826	-2.362193
Tmem260	Tmem260-002	792.75475	0.003413225	0.011869525	0.194538941	-5.140359019	1.555751422	7.997120855	-2.361869
Oas3	Oas3-001	1269.9493	0.00206279	0.008019361	0.194540991	-5.140304862	3.25856436	16.75001422	-2.361854
Msl1	Msl1-001	19721.689	2.53006E-09	1.44425E-06	0.19466101	-5.137135587	39.22296448	201.4936867	-2.360964
Tmem167	Tmem167-001	4581.7166	8.12504E-06	0.000134566	0.19467545	-5.136754523	9.957351896	51.14847239	-2.360857
Ube2d1	Ube2d1-002	1005.9263	5.9438E-05	0.000546951	0.194688408	-5.136412635	2.194126155	11.26993731	-2.360761
Mllt4	Mllt4-004	4092.5885	9.365E-07	3.3657E-05	0.19472381	-5.135478803	8.400086392	43.13846561	-2.360499
Mad2l2	Mad2l2-005	861.26089	0.000109511	0.000853798	0.194919651	-5.130319049	1.883398294	9.662434145	-2.359049
Ogfod1	Ogfod1-002	712.00581	0.009715083	0.026931735	0.195043347	-5.127065422	1.526425011	7.826080894	-2.358133
Amt	Amt-001	1298.6067	0.002115093	0.008173524	0.195072654	-5.126295163	2.419242581	12.40175154	-2.357917
Gm15614	Gm15614-001	1862	6.71439E-07	2.75005E-05	0.195081575	-5.126060728	3.760775608	19.27796415	-2.357851
930005H10R	0005H10Rik-	1020.0179	0.001944357	0.007667731	0.195104549	-5.125457129	1.840967379	9.435799377	-2.357681
Gm26581	Gm26581-001	1086	0.012073374	0.031963596	0.195105271	-5.12543816	2.933257863	15.03423178	-2.357675
Ndrg4	Ndrg4-002	1690.9775	0.007877899	0.022858186	0.195148359	-5.124306475	2.378521488	12.18827306	-2.357357
Deptor	Deptor-001	2731.3221	3.96974E-05	0.000407865	0.195213941	-5.122584962	7.121974951	36.48292179	-2.356872
Dock7	Dock7-001	1829.6121	8.36461E-05	0.000701317	0.195344323	-5.119165923	3.225857525	16.51369992	-2.355909
Sacs	Sacs-005	7388.3687	5.19687E-06	9.99959E-05	0.195353091	-5.118936145	15.13839331	77.49246871	-2.355844
Ttl	Ttl-002	256.91471	0.019201652	0.046007052	0.195389245	-5.117988953	0.502231529	2.570415418	-2.355577
Gnpda2	Gnpda2-004	1983.9099	0.000103823	0.000819427	0.195392046	-5.117915588	4.006607368	20.50547831	-2.355556
Btg1	Btg1-201	17527	0.000185696	0.001262673	0.195404637	-5.117585811	48.51346873	248.2718392	-2.355463
Helz	Helz-002	10227.582	1.40688E-07	1.15661E-05	0.19541241	-5.117382259	19.0438883	97.45485613	-2.355406
Ufl1	Ufl1-001	1439.8498	0.001321504	0.00566548	0.195554425	-5.113665921	1.734307134	8.868667286	-2.354358
Gm26773	Gm26773-201	567.76467	0.002368089	0.008936433	0.195561842	-5.113471984	1.201839428	6.145572245	-2.354303
Rmdn1	Rmdn1-002	700.67709	0.004150628	0.013826007	0.195637322	-5.111499115	1.427833362	7.298573427	-2.353746
Ugcg	Ugcg-001	12287.098	8.72034E-07	3.22933E-05	0.195671088	-5.11061706	31.08644977	158.8709405	-2.353497
Tcf4	Tcf4-001	3904.7646	0.000173759	0.00120033	0.195680617	-5.11036819	9.310662842	47.58091522	-2.353427
Arpc5l	Arpc5l-003	1661.939	3.65975E-06	7.99402E-05	0.19580718	-5.107065022	3.705011927	18.92173682	-2.352494
Dopey1	Dopey1-201	3220.4355	0.004642388	0.015095261	0.195895348	-5.104766459	5.512276461	28.13888399	-2.351845
Myl12a	Myl12a-006	12162.073	0.000353431	0.002046495	0.195911492	-5.104345796	28.93908065	147.7150747	-2.351726
Scn2b	Scn2b-201	566.18833	6.48704E-05	0.000583919	0.195916127	-5.104225022	1.073400175	5.478876033	-2.351692
Tiprl	Tiprl-004	1932.2406	5.39987E-05	0.000509773	0.195920885	-5.104101089	3.611151796	18.43168381	-2.351657
Ube2d3	Ube2d3-003	1040.238	0.006923314	0.020639177	0.195946129	-5.103443515	3.084372697	15.74092184	-2.351471
Chml	Chml-001	3500	9.99539E-07	3.49329E-05	0.195986274	-5.102398138	6.179434491	31.52993504	-2.351175
Caprin2	Caprin2-005	680.88531	5.5765E-05	0.000521699	0.196011017	-5.101754045	1.303837862	6.651860087	-2.350993
Gm26804	Gm26804-201	1320	7.07539E-05	0.000622162	0.196051547	-5.10069937	2.789980896	14.2308538	-2.350695
Gabpa	Gabpa-001	7204.8572	2.11874E-07	1.47794E-05	0.196053891	-5.100638378	13.59552177	69.3458401	-2.350678
Gm4279	Gm4279-001	325	0.008688746	0.024657349	0.196090199	-5.099693936	0.497656263	2.537894627	-2.350411
U2surp	U2surp-008	474.04574	0.005665285	0.017644449	0.196168064	-5.097669718	0.75964599	3.87242436	-2.349838
Apoe	Apoe-006	1700.1358	0.021382544	0.050054824	0.196213363	-5.096492843	4.090202816	20.84568938	-2.349505
Mapre1	Mapre1-001	28776.217	5.34128E-08	6.57708E-06	0.196216529	-5.096410609	58.39798427	297.6201066	-2.349482
Pou2f1	Pou2f1-201	4142.5175	3.93755E-06	8.32476E-05	0.196223261	-5.096235764	8.488073246	43.25722245	-2.349432
Capza2	Capza2-002	2703.0385	1.49996E-05	0.00020533	0.196267416	-5.095089253	5.877485846	29.94631497	-2.349107
Anapc5	Anapc5-010	2238.3266	2.01222E-06	5.42665E-05	0.196272192	-5.094965263	4.311981422	21.96939556	-2.349072
Vwf	Vwf-004	959.59575	0.004675727	0.015173741	0.196332019	-5.093412707	2.043341216	10.40758011	-2.348633
Gm37127	Gm37127-001	1350	0.000189698	0.001280227	0.196343952	-5.093103144	2.894687097	14.74293996	-2.348545
30432E09R	0432E09Rik-	533.78808	0.031930792	0.068678073	0.196348894	-5.092974956	1.636655728	8.335446633	-2.348509
Ap3m1	Ap3m1-001	6531.9395	8.63147E-06	0.000140042	0.196482153	-5.089520788	12.97052695	66.01376656	-2.34753
Gm17146	Gm17146-001	610	0.004399628	0.014471966	0.196485395	-5.089436793	0.934695681	4.757074589	-2.347506
Gm12961	Gm12961-001	551	0.000480786	0.002592704	0.196563229	-5.087421504	1.082802278	5.508671593	-2.346935
Tcf4	Tcf4-033	5177.2431	4.88585E-05	0.000472791	0.196623101	-5.085872387	13.46759511	68.49447011	-2.346495
Gm16028	Gm16028-001	138.16476	0.02513998	0.05672976	0.196647068	-5.085252542	0.254869889	1.29607775	-2.346319
Osbpl7	Osbpl7-004	556.68612	0.000399795	0.002248806	0.196732579	-5.083042204	1.008169263	5.124566915	-2.345692
Gm9930	Gm9930-201	139	0.039505053	0.081288559	0.196740057	-5.082848988	0.288841995	1.468140243	-2.345637
Ndufa4	Ndufa4-201	4518	2.4361E-06	6.17134E-05	0.196840305	-5.080260364	8.760489144	44.50556577	-2.344902
Ktn1	Ktn1-025	210.16698	0.007901885	0.022914148	0.196897086	-5.078795325	0.334123834	1.696946567	-2.344486
Slc35a2	Slc35a2-003	1089.2304	7.36155E-05	0.000641723	0.196970533	-5.076901533	2.370958191	12.03712128	-2.343948
Atp6v1h	Atp6v1h-004	2327.275	0.000257328	0.001609478	0.196977132	-5.076731456	4.720107009	23.96271573	-2.3439
Galnt11	Galnt11-003	1388.3271	0.001663991	0.00677092	0.197004452	-5.076027411	2.777826491	14.10032341	-2.3437
Aktip	Aktip-005	2812.8213	1.21272E-05	0.000176898	0.197012166	-5.075828676	6.046179862	30.68937312	-2.343643
Maf	Maf-001	110.64404	0.032953352	0.070439513	0.19710287	-5.073492848	0.25652207	1.301462889	-2.342979
Bbs10	Bbs10-201	592	0.008155658	0.023455479	0.197196637	-5.071080402	1.316367326	6.675404548	-2.342293
Tmem194b	mem194b-00	394.03757	0.019322582	0.046242071	0.197291063	-5.068653308	0.743174392	3.76689334	-2.341602
Efr3a	Efr3a-001	7236.9745	1.62253E-06	4.69356E-05	0.197305147	-5.068291512	13.19671151	66.88478096	-2.3415
Zc3h12b	Zc3h12b-002	2681.6537	4.28761E-05	0.000432164	0.197316885	-5.067990006	5.308892063	26.90541192	-2.341414
Primpol	Primpol-001	1194.3453	0.000378123	0.002156002	0.197319498	-5.067922877	2.681917529	13.5917512	-2.341395
Rnf146	Rnf146-201	3117.4918	0.001658089	0.006754227	0.197376143	-5.066468442	5.664519907	28.69911135	-2.34098
330007P06R	0007P06Rik-	6787.6571	4.73082E-06	9.43685E-05	0.197410811	-5.065578711	12.9750672	65.7262242	-2.340727
Cdc14b	Cdc14b-202	2493.2559	0.000488852	0.002628445	0.197414083	-5.065494733	4.138344183	20.96276066	-2.340703
Arhgap8	Arhgap8-003	1377.2374	0.022332378	0.0517723	0.197434854	-5.064961825	2.391272283	12.11170282	-2.340551
Tmem183a	mem183a-00	3463.3897	1.40862E-06	4.32449E-05	0.197455362	-5.064435774	6.960186388	35.24941694	-2.340402
Fam133b	Fam133b-003	919.3422	0.000742934	0.003619507	0.197466837	-5.064141485	1.862848163	9.433726663	-2.340318
Zcchc4	Zcchc4-004	1146.8025	0.002643064	0.009734937	0.197472338	-5.064000404	1.979583748	10.0246129	-2.340278
Fbxo5	Fbxo5-001	522.88731	0.022536827	0.052133347	0.197486327	-5.063641698	1.123494095	5.688971549	-2.340175
Mms19	Mms19-006	5268.6358	1.71085E-06	4.82711E-05	0.197507855	-5.063089758	10.27131137	52.0045714	-2.340018
Bbs5	Bbs5-004	263.79173	0.00370912	0.012667	0.197571837	-5.061450139	0.54771111	2.772212475	-2.339551
Ptpn14	Ptpn14-003	403.963	0.00544562	0.01713167	0.197582037	-5.06118884	0.924651518	4.679835941	-2.339476
Gapvd1	Gapvd1-008	338.41329	0.012516364	0.032854496	0.197582167	-5.061185495	0.597981085	3.026493191	-2.339475
Rrbp1	Rrbp1-003	14772.291	2.06577E-07	1.45864E-05	0.197594184	-5.06087771	32.42079547	164.0776811	-2.339388

Srsf11	Srsf11-004	5096.7523	5.82352E-08	6.92554E-06	0.197647435	-5.059514179	9.940031479	50.29173021	-2.338999
Cd74	Cd74-201	12946.45	0.005965684	0.018359243	0.19765588	-5.059298012	40.99854851	207.4238749	-2.338937
Kat7	Kat7-006	933.21178	0.003718513	0.012691487	0.197686139	-5.058523591	2.064743469	10.44455354	-2.338716
Nit1	Nit1-001	1041.5006	0.032739859	0.070070622	0.197740227	-5.057139929	2.110101891	10.67108053	-2.338322
Etfdh	Etfdh-004	411.7382	0.016321461	0.04044703	0.197763618	-5.056541805	0.627480633	3.172882053	-2.338151
St5	St5-202	319.27819	0.026316715	0.058819335	0.197788759	-5.055899046	0.377623058	1.909224058	-2.337968
Cklf	Cklf-202	187.378	0.025308198	0.057041707	0.197887936	-5.053365146	0.43856759	2.216242175	-2.337244
Cd47	Cd47-001	53768.594	9.22358E-07	3.35003E-05	0.197915972	-5.052649306	96.62575255	488.2160415	-2.33704
Sumo3	Sumo3-007	118.94412	0.039269432	0.080891239	0.197988731	-5.050792516	0.231492703	1.169221609	-2.33651
Syne3	Syne3-203	650.88057	0.023262154	0.053428699	0.198140949	-5.04691234	1.28536865	6.4871429	-2.335401
Sirpa	Sirpa-009	3374.515	0.000571589	0.002967101	0.198158255	-5.046471579	7.324863685	36.96471641	-2.335275
Epc1	Epc1-001	5167.5209	2.16198E-05	0.000266935	0.198186091	-5.045762761	9.090758426	45.86981034	-2.335072
Ids	Ids-001	27373.371	2.95907E-05	0.000332616	0.198251191	-5.044105883	47.50755219	239.6331235	-2.334599
Crcp	Crcp-001	1051.8645	0.003982935	0.013378007	0.198297871	-5.042918499	2.404977436	12.1281052	-2.334259
Gm26860	Gm26860-201	733.39532	0.004188424	0.013924823	0.198307326	-5.04267804	1.720389181	8.675368744	-2.33419
Blm	Blm-201	605.77805	0.042150354	0.08569688	0.198388724	-5.040609055	1.780202183	8.973303242	-2.333598
Alg10b	Alg10b-201	6420	1.33782E-06	4.19154E-05	0.198421983	-5.039764171	13.42888006	67.67838856	-2.333356
Gm26805	Gm26805-001	230	0.014089374	0.03608908	0.198433722	-5.039466016	0.461146025	2.323929721	-2.333271
Cav1	Cav1-001	144275.94	2.81575E-05	0.000321037	0.198443375	-5.039220882	226.7639859	1142.713813	-2.333201
Ecscr	Ecscr-006	279.41093	0.034016463	0.0722834	0.198461034	-5.038772486	0.283435019	1.428164574	-2.333072
Mndal	Mndal-201	1576.7303	0.000492469	0.00264291	0.198509813	-5.037534345	3.088333645	15.5575868	-2.332718
Dmtf1	Dmtf1-001	1361.9745	0.022149046	0.051423862	0.198549493	-5.036527604	1.760881744	8.868729512	-2.332429
Filip1l	Filip1l-001	742.18749	0.000119864	0.000915347	0.198654945	-5.033854055	1.153560552	5.806855363	-2.331663
Cdk19	Cdk19-201	7885.6947	3.32089E-05	0.00035903	0.198679284	-5.033237386	14.90508457	75.0208289	-2.331487
Katnbl1	Katnbl1-001	3019.5252	3.21954E-06	7.39401E-05	0.198680228	-5.033213469	6.144692488	30.92754899	-2.33148
Lmo4	Lmo4-004	613.50947	0.028116798	0.062001434	0.198745161	-5.031569054	1.389702642	6.992384807	-2.331008
Klhl41	Klhl41-001	1490	8.05309E-07	3.07178E-05	0.198798184	-5.030227027	2.910585033	14.6409035	-2.330624
Snx5	Snx5-003	5907.571	2.03108E-05	0.000255555	0.198798651	-5.030215218	15.64102233	78.67770856	-2.33062
Srp72	Srp72-008	410.02987	0.004770823	0.015410796	0.198814349	-5.029818033	0.815657708	4.102609849	-2.330506
Rad23b	Rad23b-002	453.03231	0.003845104	0.013009465	0.198836172	-5.029266012	0.961229374	4.834278221	-2.330348
Cryz	Cryz-201	139.28997	0.026647251	0.059399191	0.19883743	-5.029234173	0.362348585	1.822335887	-2.330339
Irgm1	Irgm1-002	411.07412	0.010485008	0.028637283	0.198865371	-5.028527557	0.9095364	4.573628853	-2.330136
Lrig2	Lrig2-007	790.64393	0.013052972	0.033976615	0.198895961	-5.027754175	1.17867396	5.926082923	-2.329914
Tulp3	Tulp3-003	281.27026	0.003105455	0.011033386	0.19900162	-5.025084713	0.560725399	2.81769263	-2.329148
Gm13340	Gm13340-001	532760	2.66701E-09	1.47337E-06	0.199012755	-5.024803572	1054.234998	5297.323785	-2.329067
Rpl23	Rpl23-001	8230.4366	2.31883E-06	5.97816E-05	0.199029907	-5.02437054	18.03372737	90.60812855	-2.328943
Cflar	Cflar-013	5892.6596	1.10299E-08	2.78999E-06	0.199070414	-5.023348176	12.56971757	63.14206783	-2.328649
Taz	Taz-018	253.81422	0.014173833	0.036257658	0.199103597	-5.022510966	0.691438647	3.472758187	-2.328409
Ssh2	Ssh2-001	17691.627	7.26782E-06	0.000124586	0.199117132	-5.022169571	43.31642525	217.5424328	-2.328311
Rbm6	Rbm6-005	1883.8388	1.04077E-05	0.000158781	0.199152087	-5.021288069	4.010111806	20.13592657	-2.328057
Pdpk1	Pdpk1-007	3913.8827	0.01519554	0.038250819	0.199256902	-5.018646728	7.901997152	39.65733216	-2.327298
Nans	Nans-002	257.45119	0.026433561	0.059041964	0.199294407	-5.017702281	0.668607536	3.354873559	-2.327027
Gm16588	Gm16588-001	517	0.000145625	0.001057152	0.199318318	-5.017100344	1.056461569	5.300373699	-2.326854
Dnajb6	Dnajb6-002	4518.1452	2.3206E-06	5.97816E-05	0.199410993	-5.014768657	10.08492205	50.57355102	-2.326183
Atf2	Atf2-007	5206.0389	2.24186E-05	0.00027345	0.199440882	-5.014017146	8.917592436	44.71296138	-2.325967
Mecp2	Mecp2-001	8243.2269	5.19392E-06	9.99959E-05	0.199539402	-5.011541531	14.65649008	73.45160871	-2.325254
Gm3222	Gm3222-201	653	0.000155487	0.001109695	0.199576961	-5.010598398	1.470408543	7.367626689	-2.324983
Ccnt1	Ccnt1-001	10852.235	4.12514E-08	5.56245E-06	0.199616833	-5.009597554	24.22629844	121.3640054	-2.324695
Ppp3cb	Ppp3cb-001	507.23058	0.010699041	0.029061953	0.19962323	-5.009437025	0.894682707	4.481856678	-2.324648
BC003965	BC003965-20	2731	2.85342E-06	6.83902E-05	0.199669746	-5.008270011	6.159286259	30.84736866	-2.324312
Ldha	Ldha-002	1049.2465	0.000478475	0.002584006	0.199671735	-5.008220113	2.172513364	10.88042512	-2.324298
Ccdc53	Ccdc53-007	396.72002	0.009232344	0.025888963	0.199687197	-5.007832334	0.66041427	3.307243935	-2.324186
Rnf114	Rnf114-003	5446.5349	1.59373E-06	4.6473E-05	0.199694233	-5.007655887	12.40782921	62.134139	-2.324135
Luc7l2	Luc7l2-011	1593.8703	0.013836259	0.03556145	0.199719378	-5.007025398	3.497304456	17.51109224	-2.323954
Stat1	Stat1-001	2559.843	0.002029248	0.007923134	0.199747597	-5.00631804	4.740194283	23.73092015	-2.32375
Zhx1	Zhx1-201	1374.5548	0.01301241	0.033883909	0.199790172	-5.005251212	2.658336254	13.30564076	-2.323442
Kdm7a	Kdm7a-001	13473.897	2.59326E-06	6.42685E-05	0.199845422	-5.003867451	33.99855887	170.1242821	-2.323044
Tspan2	Tspan2-001	23699.122	1.73054E-08	3.25493E-06	0.199856537	-5.003589156	49.75314678	248.9443057	-2.322963
Ltbp4	Ltbp4-003	10968.005	8.01151E-05	0.000681725	0.199868292	-5.00329488	18.28477737	91.48413299	-2.322878
Arhgap28	Arhgap28-001	1671.043	0.000718955	0.003532883	0.199876644	-5.003085794	5.300165034	26.51718039	-2.322818
Dnajb14	Dnajb14-001	11017.736	3.43823E-07	1.88049E-05	0.199906818	-5.002330633	23.4246049	117.1776186	-2.3226
Gm12286	Gm12286-001	556	0.000922846	0.004284019	0.199954545	-5.001136623	1.29291236	6.466031356	-2.322256
Pgap1	Pgap1-001	2076.3314	5.14086E-06	9.96068E-05	0.199966475	-5.000838263	3.714610818	18.57616791	-2.32217
Ms4a1	Ms4a1-003	1561.3651	1.07142E-06	3.65501E-05	0.200072982	-4.998176116	2.956382198	14.77651889	-2.321402
Nsun7	Nsun7-004	347.09	0.000859718	0.004051926	0.200077576	-4.998061351	0.668541978	3.341413823	-2.321369
Ip6k1	Ip6k1-002	621.76019	0.000410704	0.00229397	0.200122279	-4.996944905	1.510009146	7.545432508	-2.321046
Zfp87	Zfp87-001	2148.5485	1.5564E-05	0.000210782	0.200136743	-4.996583765	4.0358149	20.16528721	-2.320942
Rbm4b	Rbm4b-005	769.47756	0.025012875	0.056509338	0.200228596	-4.994291618	1.171531368	5.850969289	-2.32028
Ttc19	Ttc19-001	341.37929	0.01776483	0.043233347	0.200331219	-4.991733222	0.552870823	2.759783657	-2.319541
Gm15988	Gm15988-001	233	0.013451884	0.034784977	0.200350345	-4.991256689	0.39624482	1.977759609	-2.319403
Gm37625	Gm37625-001	1077	0.000160644	0.001135723	0.200361288	-4.990984077	2.072682982	10.34472776	-2.319324
Gm4790	Gm4790-001	5725	1.93276E-05	0.000246075	0.200376378	-4.990608212	11.96965895	59.73587824	-2.319216
Mccc1	Mccc1-004	948.77825	0.001563619	0.006457729	0.200377079	-4.990590761	1.844141422	9.203355144	-2.319211
Prpf31	Prpf31-003	709.10726	0.008728071	0.024748614	0.200378424	-4.990557256	1.732779224	8.647533929	-2.319201
Gm9892	Gm9892-001	941	0.000211851	0.001391844	0.2004437	-4.988932065	2.245040362	11.20035385	-2.318731
Itih5	Itih5-001	1203	0.017503136	0.042740795	0.200448335	-4.988816684	2.296886295	11.45874467	-2.318698
Cep72	Cep72-201	289	0.006718298	0.020143718	0.200449703	-4.988782653	0.373274464	1.862185173	-2.318688
Ecm2	Ecm2-201	32274	0.020306646	0.048038585	0.200453223	-4.988695032	44.43828702	221.6890617	-2.318662
Gm37978	Gm37978-001	926	0.003957487	0.013310805	0.200459046	-4.98855012	2.153877456	10.74472564	-2.318621
Creb1	Creb1-009	1232.9284	0.000308511	0.001847936	0.200493841	-4.987684377	2.224867421	11.09693647	-2.31837
Fam169b	Fam169b-201	1408.4533	0.000134628	0.00099849	0.200534759	-4.986666666	2.766300977	13.79462087	-2.318076
610015P09R	0015P09Rik-	896.09129	0.006620915	0.01990226	0.200535957	-4.986636897	1.385531798	6.909143984	-2.318067
Tceb1	Tceb1-002	2028.6177	8.29245E-06	0.000136415	0.200536329	-4.986627646	4.295778538	21.42144802	-2.318064
Lef1	Lef1-005	489	0.000133871	0.000994375	0.200708989	-4.98233789	0.859030225	4.27997884	-2.316823
Kif5b	Kif5b-007	177.82076	0.016045288	0.039892527	0.200739355	-4.981584212	0.444697813	2.215299606	-2.316605
Mbtps2	Mbtps2-005	1075.4385	3.73313E-06	8.06178E-05	0.200788839	-4.980356507	2.088985547	10.40389276	-2.316249
Adam28	Adam28-202	1612.8753	1.27486E-05	0.000183211	0.200808222	-4.979875772	3.117608276	15.52530192	-2.31611
Tra2b	Tra2b-002	1506.2842	3.85399E-05	0.00039903	0.200834201	-4.97923159	3.210796216	15.98729795	-2.315923
Ezh2	Ezh2-012	190.37533	0.022767369	0.052531824	0.200881267	-4.978064983	0.423896781	2.11018572	-2.315585
Fcrl6	Fcrl6-001	106	0.014362219	0.036627402	0.200902987	-4.977526783	0.229132943	1.140515362	-2.315429
Armc8	Armc8-001	5916.9261	5.93669E-08	7.0357E-06	0.200922948	-4.977032293	12.39194581	61.67511446	-2.315286
Dram1	Dram1-002	4002.7817	0.006493281	0.019600926	0.200957588	-4.97617437	6.009203098	29.90284244	-2.315037
Ythdf3	Ythdf3-002	2187.5732	0.003909616	0.013183689	0.200986138	-4.975467505	4.916344613	24.46111287	-2.314832
Ddx58	Ddx58-005	3728.1833	0.000151776	0.001090024	0.200994523	-4.975259936	7.003062568	34.84205662	-2.314772
Prpf40b	Prpf40b-015	304.49154	0.038674197	0.079987393	0.201028032	-4.97443062	0.386985694	1.925033486	-2.314531
Prpf38a	Prpf38a-002	745.52047	0.000221038	0.001436813	0.201049375	-4.973902549	1.468404693	7.303701847	-2.314378
Nfatc2	Nfatc2-003	559.38399	0.037162684	0.077530726	0.201056346	-4.973730091	1.234571706	6.140426442	-2.314328
Gm16239	Gm16239-001	314	0.003344069	0.011689565	0.201057172	-4.973709678	0.685120879	3.407592346	-2.314322
Phka2	Phka2-006	1568.4536	0.013397173	0.034674917	0.201083853	-4.973049723	3.612160542	17.96345398	-2.314131
Bclaf1	Bclaf1-007	672.53678	0.000187796	0.001272403	0.201102797	-4.972581262	1.34896873	6.707856629	-2.313995
Gm2574	Gm2574-001	1244	0.000137556	0.001016242	0.201148331	-4.971455615	2.933285556	14.58269895	-2.313668
Zfp82	Zfp82-007	179.7978	0.048753791	0.096176554	0.201169668	-4.970928311	0.346994449	1.724884531	-2.313515
Rpl15	Rpl15-001	9088.4608	5.99348E-06	0.000109472	0.201173976	-4.970821873	18.67984828	92.85419842	-2.313484
Jmjd1c	Jmjd1c-008	10227.141	6.00069E-05	0.000551149	0.201247653	-4.969002046	20.00984505	99.42896101	-2.312956
Ube2g1	Ube2g1-001	3787.5647	0.000179253	0.001228376	0.201267666	-4.96850796	8.660432063	43.02942565	-2.312813
Tsen15	Tsen15-007	501.87122	0.004086302	0.013639593	0.201273163	-4.968372257	0.96683096	4.80357612	-2.312773
Fus	Fus-011	3673.5194	0.002916797	0.01052801	0.201286775	-4.96803628	6.686033031	33.21645466	-2.312676

Srd5a3	Srd5a3-006	317.5075	0.005966084	0.018359243	0.201413786	-4.964903442	0.703670602	3.493656596	-2.311766
Pml	Pml-010	1587.9591	0.040594636	0.083091275	0.201454636	-4.963896685	2.941723877	14.6024134	-2.311473
Mbnl1	Mbnl1-018	5795.1478	0.001546936	0.00639936	0.201581277	-4.960778174	11.25074542	55.8124523	-2.310566
Whsc1	Whsc1-009	890.9934	0.015065623	0.037982892	0.201769939	-4.956139681	1.931487248	9.572720591	-2.309217
Adcy7	Adcy7-204	277.14713	0.03913093	0.080683545	0.201806541	-4.955240775	0.833102234	4.128222161	-2.308955
Gclm	Gclm-001	6704.3379	8.72829E-06	0.000141345	0.201810484	-4.955143949	13.28300856	65.81921947	-2.308927
Dopey1	Dopey1-006	1002	2.47231E-05	0.000292898	0.201841722	-4.954377074	1.84997658	9.165481555	-2.308704
Srp72	Srp72-004	1180.765	0.000633705	0.003209287	0.201878473	-4.953475159	2.595596291	12.85722175	-2.308441
Atxn7l1	Atxn7l1-005	1265.3758	0.009066181	0.025523318	0.201964993	-4.951353137	2.25175142	11.14921646	-2.307823
Asxl2	Asxl2-003	4070.2252	3.3461E-06	7.54966E-05	0.20198997	-4.950740881	8.414479254	41.65790643	-2.307644
Zfp568	Zfp568-001	3420.8945	1.41308E-05	0.00019698	0.202018374	-4.950044792	7.803781828	38.62906959	-2.307442
Armc1	Armc1-001	4749	5.47646E-07	2.4488E-05	0.202026165	-4.949853886	9.512650605	47.08623056	-2.307386
Gm11531	Gm11531-001	132	0.041067214	0.083873111	0.202036351	-4.94960435	0.204342494	1.011414498	-2.307313
Gm26718	Gm26718-201	1331.9723	2.37525E-05	0.000284181	0.202146139	-4.94691615	3.222296232	15.94042927	-2.306529
Mettl3	Mettl3-005	223.08936	0.011710076	0.031231125	0.202165239	-4.946448786	0.364740209	1.804168765	-2.306393
Hykk	Hykk-003	1717.3014	8.75321E-06	0.000141547	0.202196724	-4.94567855	3.15334956	15.59545328	-2.306168
Tmem208	Tmem208-001	856.68581	0.000728999	0.003568814	0.202197265	-4.945665324	1.732189464	8.566829365	-2.306165
Rnf115	Rnf115-002	1394.0771	0.000324331	0.001916883	0.202239509	-4.944632261	2.052569173	10.14919975	-2.305863
Ercc8	Ercc8-004	321.05543	0.001255845	0.005449144	0.20227383	-4.943793275	0.690283895	3.412620878	-2.305618
Mx2	Mx2-001	140.3595	0.042220222	0.085798185	0.202329066	-4.942443603	0.303904229	1.502029512	-2.305225
Hivep2	Hivep2-003	933.8659	0.000386671	0.002194446	0.202387372	-4.941019738	1.95889869	9.678957091	-2.304809
Slc10a1	Slc10a1-201	239	0.032743009	0.070072269	0.202465443	-4.939114473	0.392400126	1.938109143	-2.304252
Slc7a2	Slc7a2-002	10762.992	2.92773E-06	6.94075E-05	0.202486132	-4.938609833	19.16339645	94.64053812	-2.304105
Tirap	Tirap-001	4613.7066	1.15152E-07	1.03788E-05	0.202560738	-4.936790854	8.928510046	44.07818674	-2.303574
Atp1b1	Atp1b1-001	12218.22	0.01172838	0.031270201	0.202618011	-4.93539541	11.37500278	56.1401365	-2.303166
Smg1	Smg1-004	1629.5633	1.16088E-05	0.000171305	0.202652914	-4.934545368	3.505582371	17.29845525	-2.302917
P23-97M20	23-97M20.1-(	1825	1.797E-05	0.000233488	0.202708246	-4.933198422	3.692866571	18.21764354	-2.302523
BC027231	3C027231-00:	901.16327	0.000398853	0.002245352	0.20270903	-4.933179356	1.866525312	9.207904137	-2.302518
Rbm11	Rbm11-202	282.21694	0.003845014	0.013009465	0.202722974	-4.93284002	0.66861505	3.298171077	-2.302418
Tpcn1	Tpcn1-004	807.52489	0.001118516	0.004976122	0.202793279	-4.931129882	1.781836835	8.78646886	-2.301918
Prpf39	Prpf39-002	2769.1607	4.7863E-05	0.000466508	0.20280006	-4.930965019	6.225496764	30.69770677	-2.30187
Gm37314	Gm37314-001	640	0.000142339	0.001040581	0.202863578	-4.92942109	1.445470349	7.125332022	-2.301418
Irak1	Irak1-001	4335.183	0.00014691	0.00106355	0.202893474	-4.928694748	7.575560762	37.33762654	-2.301206
Cux1	Cux1-007	880.94009	0.001774646	0.007132318	0.202921803	-4.928006675	1.761034821	8.678391352	-2.301004
Uimc1	Uimc1-006	2345.7274	0.009625679	0.026740165	0.202945555	-4.927429923	4.908861657	24.18807181	-2.300835
Specc1	Specc1-003	4799.3356	2.5781E-05	0.000302008	0.202962477	-4.9270191	4.948136916	24.37956509	-2.300715
Tfdp2	Tfdp2-016	751	0.002037508	0.007945421	0.20296923	-4.926855163	1.575790179	7.763689979	-2.300667
Gm37959	Gm37959-001	310	0.00715201	0.021184498	0.202998558	-4.926143372	0.528517101	2.603551016	-2.300459
Arcn1	Arcn1-002	1234.8937	0.000212387	0.001394336	0.203002323	-4.926051995	2.575649722	12.68778445	-2.300432
Gm2986	Gm2986-001	211	0.015212133	0.038284631	0.203011416	-4.925831369	0.40816741	2.010563834	-2.300367
Platr28	Platr28-001	783	0.000364919	0.002093547	0.203011829	-4.925821352	1.664751341	8.200267703	-2.300364
Pign	Pign-007	6915.4861	1.72299E-08	3.25493E-06	0.203069593	-4.924420166	14.39504986	70.88727385	-2.299954
Gm37234	Gm37234-001	670	8.76461E-05	0.000723735	0.203089299	-4.923942348	1.324055007	6.519570522	-2.299814
Arhgef5	Arhgef5-004	238.26739	0.021768841	0.050757196	0.203098406	-4.923721547	0.2132522	1.04999445	-2.299749
Psph	Psph-005	178.4151	0.01514732	0.038159491	0.203106305	-4.923530074	0.400071069	1.969761941	-2.299693
)30443J06Ri	)0443J06Rik-(	761.8476	0.000113338	0.000876258	0.203142029	-4.922664225	1.494406003	7.356458971	-2.299439
Orc6	Orc6-201	1213.3181	0.043676285	0.088100994	0.20314676	-4.922549599	2.508978472	12.35057097	-2.299406
Xpr1	Xpr1-001	13382.969	1.21722E-07	1.06644E-05	0.203159324	-4.922245157	28.03516242	137.9959425	-2.299317
Ly6e	Ly6e-014	843	0.002357666	0.008903964	0.203242487	-4.920231076	1.770399399	8.710774139	-2.298726
00029M09R	)0029M09Rik-	306.55767	0.011227769	0.030179807	0.203259344	-4.919823014	0.605776051	2.980310956	-2.298606
Gm10125	Gm10125-004	220.40225	0.008688083	0.024657349	0.203271343	-4.919532608	0.429961766	2.11521093	-2.298521
Cyb5a	Cyb5a-003	304.06803	0.003816444	0.012943185	0.203290018	-4.91908068	0.648408444	3.18957345	-2.298389
Srpk2	Srpk2-009	803.90189	0.000389832	0.002207628	0.203293423	-4.918998291	1.608482039	7.9121204	-2.298365
23-316M11	3-316M11.10	679	0.005093397	0.016229309	0.203319708	-4.918362361	1.702071078	8.371402328	-2.298178
Sar1a	Sar1a-201	22103	1.29204E-08	2.99002E-06	0.203333441	-4.918030177	42.59184918	209.4679995	-2.298081
Smchd1	Smchd1-009	371.04932	0.003432459	0.011918261	0.203390996	-4.916638502	0.919320515	4.519968312	-2.297672
Tbce	Tbce-007	364.12036	0.007488899	0.02198087	0.203428337	-4.915735992	0.680582057	3.345561712	-2.297407
Vezt	Vezt-006	1694.4163	5.54191E-05	0.000519317	0.2034317	-4.915654744	3.058252494	15.03331338	-2.297384
Eprs	Eprs-003	375.72721	0.040475596	0.082891786	0.203501106	-4.913978206	0.817390402	4.016638622	-2.296891
Ppia	Ppia-005	24606.31	1.03201E-07	9.64322E-06	0.203516987	-4.91359476	50.27312696	247.0217732	-2.296779
Spen	Spen-003	860.24796	0.00157996	0.006510301	0.203517706	-4.913577387	1.737918741	8.539398224	-2.296774
Pnisr	Pnisr-003	3368.3096	3.9413E-05	0.000405861	0.203531427	-4.91324614	5.850492666	28.74491051	-2.296677
Txndc5	Txndc5-004	6858.9373	1.26899E-05	0.000182771	0.203536245	-4.913129845	18.56618058	91.21805591	-2.296642
Anapc1	Anapc1-002	1376.0318	1.86514E-05	0.000239884	0.203556181	-4.912648651	2.752025934	13.51973649	-2.296501
Mta3	Mta3-004	627.06071	0.016755508	0.041317511	0.203564888	-4.912438533	1.31263929	6.448259828	-2.296439
Hps3	Hps3-001	452.83926	0.039533771	0.081320144	0.203581689	-4.912033124	0.475526272	2.335800799	-2.29632
Strn	Strn-001	12673.467	6.14757E-08	7.23555E-06	0.203586146	-4.911925594	25.40801818	124.8022948	-2.296289
Sec61a2	Sec61a2-009	454.04131	0.006137473	0.018800504	0.20359118	-4.911804129	0.843854482	4.144847927	-2.296253
Polr3k	Polr3k-001	2959.0676	1.7401E-07	1.30698E-05	0.203597388	-4.911654363	6.208353069	30.49328444	-2.296209
Kctd6	Kctd6-202	1342.5831	0.000254462	0.001595337	0.203608121	-4.911395443	2.175597897	10.6852216	-2.296133
Atp8b2	Atp8b2-012	435.71609	0.048954227	0.096471937	0.203681936	-4.909615557	0.807643076	3.96521701	-2.29561
Rfx7	Rfx7-201	5216.3727	1.63136E-07	1.26693E-05	0.203731694	-4.908416469	10.9735032	53.86252385	-2.295258
Ythdf3	Ythdf3-001	3599.5306	1.24981E-05	0.000180692	0.203733891	-4.908363522	7.568105403	37.14701249	-2.295242
Ift57	Ift57-001	820.90601	0.019277523	0.046149099	0.20376098	-4.907710978	1.537040063	7.543348393	-2.29505
Thumpd3	Thumpd3-002	1611.2422	0.014510101	0.036916577	0.203817857	-4.906341448	2.882588306	14.14296248	-2.294648
Dab2	Dab2-008	5722.4905	0.000215823	0.001411751	0.203866943	-4.905160123	11.1598371	54.7407879	-2.2943
Mrps18c	Mrps18c-001	1031.9267	0.022024568	0.05120591	0.203898515	-4.904400601	2.427484624	11.90535705	-2.294077
Tbp	Tbp-002	706.70716	0.011040723	0.029786904	0.203913176	-4.904047991	1.002830369	4.917928254	-2.293973
Mre11a	Mre11a-002	2905.0077	5.78027E-06	0.000106955	0.203930206	-4.903638442	6.410596679	31.43524831	-2.293853
Gm26672	Gm26672-004	1412.3857	1.69641E-05	0.000224506	0.203958295	-4.902963117	3.072171359	15.06274286	-2.293654
Pign	Pign-011	591.72855	0.000283341	0.001729843	0.203958561	-4.90295674	1.245473928	6.10650479	-2.293652
Plekhg5	Plekhg5-005	835.89392	0.004683144	0.015192053	0.203964268	-4.902819538	1.466840981	7.19165662	-2.293612
Csnk1g1	Csnk1g1-003	1547.9298	4.61664E-05	0.00045481	0.203979912	-4.902443542	2.829497787	13.87145315	-2.293501
Pds5a	Pds5a-006	739.47809	0.000171133	0.001188427	0.204047264	-4.900825333	1.44469909	7.080217897	-2.293025
Raf1	Raf1-006	3465.6429	3.3148E-06	7.51368E-05	0.204051358	-4.900726998	7.721779851	37.84233499	-2.292996
Spryd7	Spryd7-001	777.47723	0.000826734	0.003929997	0.204071655	-4.900239569	1.401544488	6.867903758	-2.292852
Gpbp1	Gpbp1-002	7661.1994	1.68213E-06	4.78909E-05	0.204202621	-4.89709678	17.79643033	87.15084167	-2.291927
Akirin1	Akirin1-001	4628.6135	1.25905E-05	0.000181721	0.204234006	-4.89634425	10.28468703	50.35736821	-2.291705
Hyal3	Hyal3-001	1315	0.000440696	0.002427054	0.204246355	-4.896048195	2.517514746	12.32587353	-2.291618
Fxyd5	Fxyd5-007	30497.806	7.23635E-07	2.87857E-05	0.204259099	-4.895742746	47.75166547	233.7798699	-2.291528
130019J16R	30019J16Rik-	550	0.003894472	0.01314533	0.204260341	-4.895712975	1.334238218	6.532047354	-2.291519
Hdac7	Hdac7-001	768.77529	0.014438173	0.036781084	0.204263971	-4.895625964	1.38093474	6.760539968	-2.291493
Far1	Far1-001	7837.0987	4.84766E-06	9.58064E-05	0.204285171	-4.89511792	17.11423412	83.77619413	-2.291344
Prrc2b	Prrc2b-001	22391.903	8.89387E-06	0.000142945	0.204391306	-4.892576012	48.18618745	235.7545849	-2.290594
Kdm2b	Kdm2b-009	1178.2439	0.0082322	0.023614142	0.204422846	-4.891821137	2.633512546	12.88267234	-2.290372
Myom2	Myom2-002	897.21759	1.85001E-05	0.000238386	0.204500709	-4.889958587	1.891623605	9.249961088	-2.289822
Rpain	Rpain-006	392.37439	0.01921	0.046020918	0.204513638	-4.889649454	0.843357596	4.123723008	-2.289731
Gcc2	Gcc2-004	540.60785	0.000479876	0.00258953	0.204514519	-4.889628395	0.972363033	4.754493895	-2.289725
Btbd3	Btbd3-001	8447.7147	0.009342176	0.026130627	0.204553356	-4.888700035	9.569379622	46.7818265	-2.289451
Nupl2	Nupl2-001	643.52279	0.001681565	0.006824736	0.204563806	-4.888450302	1.14148163	5.580076219	-2.289377
P23-407F12	23-407F12.1-(	681	0.001785042	0.007162333	0.204603578	-4.887500053	1.331256373	6.506515594	-2.289097
Tmcc1	Tmcc1-001	3116.6019	0.000924314	0.004287927	0.204679161	-4.885695221	6.677736425	32.62538493	-2.288564
Sfxn5	Sfxn5-001	488.78229	0.004490862	0.014711815	0.204694551	-4.885327903	1.353166338	6.610661267	-2.288455
Ddx58	Ddx58-008	822.36314	0.000399082	0.002245589	0.204755435	-4.883875252	1.647110138	8.044280442	-2.288026
Gm13623	Gm13623-001	333	0.000554798	0.002900159	0.204755711	-4.883868671	0.764225426	3.732376615	-2.288024
Rbmx	Rbmx-004	4573.9161	0.005541174	0.017357345	0.204769045	-4.883550629	9.251598137	45.18064789	-2.28793
Gmps	Gmps-001	8170	9.28376E-09	2.48413E-06	0.204782933	-4.883219443	18.78872168	91.74945101	-2.287833

Ikbkg	Ikbkg-001	1389.2098	0.006748628	0.020218729	0.204805524	-4.8826808	2.945169927	14.38032465	-2.287673
Pphln1	Pphln1-203	3488.6685	0.003735598	0.012729502	0.204839764	-4.881864631	8.967459599	43.77792385	-2.287432
Cdk14	Cdk14-005	659.18479	0.002967809	0.010660456	0.204850539	-4.881607864	1.255658302	6.129631444	-2.287356
Atf2	Atf2-001	1859.4961	0.038418582	0.079564423	0.204850828	-4.881600974	5.240329066	25.58119547	-2.287354
Mtf2	Mtf2-014	231.48698	0.01126904	0.030269362	0.204860114	-4.881379685	0.563249098	2.749432706	-2.287289
Tor1aip2	Tor1aip2-001	6742.0829	1.23967E-06	4.00553E-05	0.204931772	-4.879672833	13.91897721	67.92005496	-2.286784
Gm15953	Gm15953-001	404	0.000845215	0.003995669	0.204945969	-4.879334808	0.93598979	4.567007561	-2.286684
Gsr	Gsr-001	7539.2791	0.007633638	0.022309729	0.205006354	-4.877897588	22.27170448	108.6390936	-2.286259
Ubn2	Ubn2-001	16406.47	2.90486E-08	4.4816E-06	0.205108595	-4.875466089	32.29466137	157.4515263	-2.28554
Gtf2h5	Gtf2h5-003	308.10869	0.0065734	0.019792434	0.205161747	-4.874202991	0.622561909	3.034493117	-2.285166
Yipf1	Yipf1-003	615.63644	4.492E-05	0.000446851	0.20516851	-4.874042326	1.584163711	7.721280979	-2.285119
Mbd5	Mbd5-006	866.56904	7.63478E-05	0.000658311	0.205202978	-4.873223616	1.705994637	8.313693354	-2.284876
Arhgap21	Arhgap21-01	13055.226	1.35766E-05	0.000191594	0.205217198	-4.872885938	25.25488353	123.0641668	-2.284776
Gm19024	Gm19024-001	289	0.000654181	0.003288187	0.205238052	-4.872390819	0.582561366	2.838466654	-2.28463
110082I17R	0082I17Rik-	1563.9384	0.00196556	0.007733477	0.205251086	-4.872081399	4.018898048	19.58039842	-2.284538
Asun	Asun-005	203.07631	0.008223638	0.023603419	0.20527441	-4.871527821	0.226218779	1.102031076	-2.284374
Ppp1r13b	Ppp1r13b-202	411.2697	0.001406127	0.005950803	0.205306552	-4.870765159	0.815273385	3.971005197	-2.284148
Zbtb40	Zbtb40-002	839.5874	0.000882839	0.004139251	0.205314577	-4.870574779	1.957860117	9.535904108	-2.284092
BC030499	3C030499-00	369.26044	0.00197547	0.007761577	0.205320884	-4.870425153	0.780582334	3.801767835	-2.284048
R3hdm1	R3hdm1-008	944.29051	0.00050825	0.002705011	0.205342737	-4.869906851	1.82973465	8.910637306	-2.283894
Ttc14	Ttc14-005	813.95587	0.021071521	0.049475532	0.205355566	-4.869602607	1.542808332	7.512863477	-2.283804
Gm26686	Gm26686-001	6686	1.47747E-06	4.44277E-05	0.205425687	-4.86794039	13.42131243	65.33414885	-2.283312
Prpf38b	Prpf38b-003	9468.473	1.15855E-06	3.8523E-05	0.205437341	-4.86766424	19.55329602	95.17887981	-2.28323
Msantd2	Msantd2-201	4448	1.87281E-07	1.37059E-05	0.205439029	-4.867624261	8.30322078	40.41695892	-2.283218
Pum2	Pum2-004	1913.8295	2.21176E-05	0.000271127	0.205509235	-4.865961375	3.707382636	18.03998071	-2.282725
Mapk6	Mapk6-002	282.0612	0.014335972	0.036574082	0.205514073	-4.86584682	0.718789246	3.497518369	-2.282691
Huwe1	Huwe1-008	1172.4163	0.002964799	0.010650761	0.205537684	-4.865287857	2.71276417	13.19837857	-2.282525
Dhx15	Dhx15-006	645.97188	0.003261344	0.011468278	0.205546424	-4.865080983	1.615988777	7.861916269	-2.282464
30034N14R	0034N14Rik-	3086	7.54319E-06	0.000127772	0.205608636	-4.863608945	6.779720552	32.97390952	-2.282027
Rnf6	Rnf6-201	2253.806	0.002059565	0.008009548	0.205657844	-4.862445217	4.611278462	22.4220889	-2.281682
Fam129b	Fam129b-003	400.95031	0.007653992	0.022352423	0.205680698	-4.861904931	0.746349252	3.628679107	-2.281522
Pex6	Pex6-001	619.89616	0.012050427	0.031915181	0.205692715	-4.861620897	1.252976844	6.09149841	-2.281437
Mcm6	Mcm6-004	1286.1436	0.009208905	0.025844396	0.205698226	-4.861490637	3.328741046	16.18264343	-2.281399
Phykpl	Phykpl-001	1474.0172	0.000543981	0.002855897	0.205783521	-4.859475607	3.136910515	15.24374013	-2.280801
Srsf11	Srsf11-016	291.41247	0.003181688	0.011257522	0.205795611	-4.859190129	0.425207268	2.066162961	-2.280716
Rbm4	Rbm4-003	3375.6973	1.98136E-07	1.41379E-05	0.205864602	-4.857561668	6.747510794	32.77644979	-2.280232
Zfp280d	Zfp280d-016	240.10293	0.004772779	0.015414209	0.205879862	-4.857201635	0.490063204	2.380335797	-2.280125
Rap1a	Rap1a-001	2757.4803	0.000345676	0.002011685	0.205912905	-4.856422187	5.111438142	24.8233016	-2.279894
Tbck	Tbck-003	832.05345	0.000135457	0.001003549	0.205935412	-4.855891424	1.689719932	8.205096528	-2.279736
Dst	Dst-201	39951.386	2.89239E-07	1.69341E-05	0.205999773	-4.854374275	85.50574679	415.0768976	-2.279285
Tmem60	Tmem60-001	1283.1889	7.66163E-05	0.000659821	0.206008063	-4.854178933	2.518439478	12.22495586	-2.279227
Yes1	Yes1-004	1893.3566	1.07366E-05	0.000162712	0.206019031	-4.853920512	3.229258344	15.67456331	-2.27915
Supv3l1	Supv3l1-004	583.61788	7.3083E-05	0.000638055	0.206024691	-4.853787156	1.136287429	5.51529733	-2.279111
Fam20b	Fam20b-002	612.06201	0.008233121	0.023614805	0.206028866	-4.853688801	1.388688753	6.740263046	-2.279082
Dvl1	Dvl1-005	1497.342	0.0364173	0.076305555	0.206074011	-4.852625506	3.295186733	15.99030719	-2.278766
Zfp184	Zfp184-001	224.35683	0.00959913	0.026683726	0.20616548	-4.850472546	0.632292632	3.066918053	-2.278125
Wasf2	Wasf2-004	283.70784	0.023402888	0.053665567	0.206183115	-4.850057683	0.582012118	2.822792346	-2.278002
Donson	Donson-002	602.34607	0.037148956	0.077506806	0.206188644	-4.849927612	1.501515836	7.282243114	-2.277963
Kif5b	Kif5b-003	5244.9499	0.000121076	0.000921932	0.20619574	-4.849760716	8.836287651	42.85388072	-2.277914
Micu3	Micu3-002	1298.9909	0.000115458	0.00088884	0.206215589	-4.84929392	2.924229831	14.18044994	-2.277775
Abhd13	Abhd13-002	2050.9691	0.004315498	0.014258712	0.206245469	-4.848591368	4.694226084	22.76038407	-2.277566
Ndufb8	Ndufb8-002	1563.1277	1.17339E-05	0.000172262	0.206250452	-4.848474224	2.83993063	13.76933046	-2.277531
Rpgrip1l	Rpgrip1l-001	1036.6078	0.002643355	0.009734937	0.206271983	-4.847968134	2.286188989	11.08337136	-2.27738
Ocln	Ocln-001	1431.3396	0.000117959	0.000904315	0.206296667	-4.847388049	3.136475645	15.20371456	-2.277208
Ndufaf4	Ndufaf4-002	1550.3306	0.000427807	0.002369887	0.206344988	-4.846252927	3.738879816	18.11955725	-2.27687
Trmt13	Trmt13-002	1164.5193	0.000188501	0.00127542	0.20647396	-4.843225753	2.319078589	11.23182115	-2.275968
Lcat	Lcat-001	480	0.002953461	0.010624518	0.206501444	-4.842581148	0.688412225	3.333692064	-2.275776
Chd6	Chd6-002	948.37415	0.000645463	0.003253918	0.206519584	-4.8421558	1.933367997	9.361669062	-2.275649
Ubxn6	Ubxn6-003	1408.1203	1.95256E-05	0.000247777	0.20652271	-4.8420825	2.89142853	14.00053548	-2.275628
Safb2	Safb2-003	184.71105	0.014574738	0.037053501	0.206525688	-4.842012683	0.418944305	2.028533639	-2.275607
Ppfibp1	Ppfibp1-005	1169.234	0.000350269	0.002032306	0.20659378	-4.840416787	2.722539531	13.17822605	-2.275131
Ankle2	Ankle2-001	3018.2475	0.001881919	0.007459341	0.206624955	-4.839686468	7.020189068	33.97551404	-2.274914
Zdhhc24	Zdhhc24-003	576.82583	0.000450886	0.002469668	0.206625298	-4.839678452	0.955302133	4.623355149	-2.274911
Rffl	Rffl-202	1568.5257	0.001858493	0.00738768	0.206661524	-4.838830089	4.098939345	19.83407104	-2.274658
Gm5138	Gm5138-001	967	0.000179781	0.001231009	0.206668965	-4.838655857	2.302525424	11.14112813	-2.274606
Gm9703	Gm9703-001	519	0.000830322	0.003941582	0.206682621	-4.838336168	1.251881993	6.057025926	-2.274511
Tgds	Tgds-201	1295.2353	0.000866137	0.00407713	0.206686861	-4.838236913	2.893782652	14.00080605	-2.274481
Zfp825	Zfp825-001	1616.6915	3.38089E-05	0.000364137	0.20668891	-4.838188956	3.469556252	16.78636874	-2.274467
Lman1	Lman1-001	12998.291	4.66849E-07	2.23944E-05	0.206787346	-4.835885858	27.60328121	133.4863172	-2.27378
Gm16091	Gm16091-001	619	0.003134037	0.011115332	0.20685295	-4.83435214	1.281190865	6.193727802	-2.273323
Ctage5	Ctage5-005	2257.9685	0.000221329	0.001438431	0.206906795	-4.833094046	5.105934777	24.67746297	-2.272947
30005N14R	0005N14Rik-	5574	8.0459E-07	3.07178E-05	0.206912337	-4.832964594	12.52155667	60.51624006	-2.272908
Cav1	Cav1-003	1283.3981	0.004730346	0.015306033	0.206919135	-4.832805821	2.178599998	10.52875075	-2.272861
St7	St7-006	535.14918	0.024664777	0.05589312	0.206930377	-4.832543258	1.291812226	6.242738462	-2.272783
Eri2	Eri2-003	556.26128	0.034698144	0.073426719	0.206945824	-4.832182544	1.351390472	6.530165449	-2.272675
Uhrf2	Uhrf2-003	4661.9663	2.79459E-08	4.38777E-06	0.206970104	-4.831615685	10.16733982	49.12467853	-2.272506
Eif4e2	Eif4e2-003	908.13849	0.000748463	0.0036377	0.206986579	-4.831231121	1.779613933	8.597726217	-2.272391
Zfp626	Zfp626-201	694	0.000252873	0.001588284	0.206997429	-4.830977876	1.625312609	7.851849256	-2.272315
10004P03R	0004P03Rik-	1514	2.14894E-05	0.000265984	0.207005249	-4.830795373	2.943346678	14.21870551	-2.272261
Trmt13	Trmt13-008	106.01328	0.03780754	0.078574529	0.207012806	-4.830619035	0.215559494	1.041285797	-2.272208
Ran	Ran-001	10370.689	0.000992544	0.004524171	0.207044817	-4.829872166	28.87324008	139.4540586	-2.271985
30403O15R	0403O15Rik-	651	0.000148655	0.001072787	0.207067528	-4.829342441	1.164360979	5.623097892	-2.271827
Diablo	Diablo-003	1136.1865	0.000231275	0.001486708	0.207070271	-4.829278462	2.479311862	11.97328738	-2.271808
Gm13034	Gm13034-001	561	0.000250288	0.001576675	0.207140818	-4.827633728	1.302689968	6.288910028	-2.271316
Fxr1	Fxr1-003	4165.1295	0.000480842	0.002592704	0.207151446	-4.82738604	9.674030279	46.70027872	-2.271242
Gm15708	Gm15708-002	676.26665	0.000160024	0.001132637	0.207170213	-4.826948741	1.550212321	7.482795411	-2.271112
Gapvd1	Gapvd1-014	1965.0324	0.000128057	0.000960781	0.207175772	-4.826819236	3.88145128	18.7350637	-2.271073
Park2	Park2-002	628.4104	0.000156389	0.001113813	0.207181534	-4.826684993	1.123521437	5.422884061	-2.271033
30064D06R	0064D06Rik-	342.75015	0.018335198	0.044333253	0.207225382	-4.825663673	0.648569904	3.129780226	-2.270727
Ctla2b	Ctla2b-202	1021.8519	0.00762723	0.022298988	0.207228916	-4.825581389	3.080032774	14.86294883	-2.270703
30043M19R	0043M19Rik-	254.33714	0.02347669	0.053795186	0.207256859	-4.824930788	0.611772951	2.951762146	-2.270508
Aplf	Aplf-202	955.64611	0.010930409	0.029556089	0.207266446	-4.824707623	1.816641554	8.764764354	-2.270442
Slc2a3	Slc2a3-002	1566.4506	5.66007E-05	0.000527503	0.20728173	-4.824351872	3.792482179	18.2962685	-2.270335
Rbbp4	Rbbp4-003	2300.9794	4.87251E-07	2.3114E-05	0.207290646	-4.824144357	5.541340357	26.73222581	-2.270273
Zdhhc13	Zdhhc13-001	1613.582	0.000275239	0.00169063	0.207296304	-4.824012684	3.693405801	17.81703643	-2.270234
Tbl2	Tbl2-001	3828.0242	4.15826E-06	8.66832E-05	0.207319278	-4.823478122	7.875297921	37.98632723	-2.270074
Zxdc	Zxdc-201	1800.9801	0.000159899	0.001132217	0.207332528	-4.823169869	3.315325756	15.99037929	-2.269982
Atp13a3	Atp13a3-201	8266.0868	0.000168342	0.001173562	0.207359792	-4.822535707	16.26871103	78.45643985	-2.269792
Klhdc2	Klhdc2-001	6852.0697	2.0825E-08	3.6337E-06	0.20738329	-4.821989275	15.52233322	74.84852431	-2.269628
R3hdm1	R3hdm1-010	3949.4817	8.95994E-07	3.29268E-05	0.207424678	-4.821027119	7.498400272	36.14999106	-2.269341
Sos2	Sos2-001	4179.7706	0.001048556	0.004724157	0.207446739	-4.82051443	9.097253073	43.85343971	-2.269187
Crat	Crat-001	861.07946	0.044462878	0.089364097	0.207464932	-4.820091715	1.846716825	8.901344467	-2.269061
Ly6e	Ly6e-201	416.44326	0.036986739	0.077248358	0.207542586	-4.818288241	0.674151109	3.248254362	-2.268521
Phactr4	Phactr4-201	3715.72	0.001833881	0.007313741	0.207559349	-4.817899088	8.083499454	38.94548465	-2.268404
Sppl2a	Sppl2a-001	13218.372	4.64291E-08	5.97818E-06	0.20759049	-4.81717636	22.01115664	106.0316234	-2.268188
Nkiras1	Nkiras1-004	488.78271	0.00239727	0.009025265	0.207644504	-4.815923267	0.987664127	4.756514647	-2.267812
Bud13	Bud13-004	161.22461	0.008806464	0.02490886	0.207644558	-4.815922017	0.360664005	1.736929724	-2.267812

Padi2	Padi2-001	1380	4.27653E-05	0.000431305	0.20767306	-4.815261074	3.252778277	15.66297662	-2.267614
Pwwp2a	Pwwp2a-002	1501.0726	3.00582E-05	0.000335767	0.207694066	-4.814774055	3.196265884	15.38929805	-2.267468
Slc25a40	Slc25a40-005	1530.3909	0.00036435	0.002091906	0.207697801	-4.814687466	3.40132358	16.37631001	-2.267442
Rpl11	Rpl11-006	432.18479	0.000957881	0.004402392	0.207701943	-4.814591452	1.104044807	5.315524689	-2.267413
AI606181	AI606181-001	900	0.00019055	0.001284206	0.207717302	-4.81423545	1.910806339	9.199071613	-2.267307
Xkr8	Xkr8-001	2205.9389	1.22762E-05	0.000178388	0.207718898	-4.814198467	4.130322405	19.88419179	-2.267296
Iqgap2	Iqgap2-201	1397	0.004430911	0.014548816	0.207800266	-4.812313369	4.546642276	21.87986741	-2.266731
Scyl2	Scyl2-006	545.37348	0.004289446	0.014186267	0.207850062	-4.811160451	1.224737349	5.892407896	-2.266385
Rnf214	Rnf214-005	288.60108	0.030815366	0.06678645	0.207854139	-4.811066085	0.809791836	3.89596204	-2.266357
Aimp1	Aimp1-002	555.36229	0.000421623	0.00234425	0.207865372	-4.810806092	1.109089637	5.335615182	-2.266279
Madd	Madd-026	1086.9822	1.70131E-05	0.000224761	0.207873467	-4.810618759	2.424296506	11.66236625	-2.266222
Tmem87a	Tmem87a-00(	3356.0487	1.82493E-06	5.05695E-05	0.207883719	-4.810381517	6.380804074	30.69410198	-2.266151
Vezt	Vezt-001	3394.848	4.31702E-06	8.8697E-05	0.20790363	-4.809920818	6.261942236	30.11944632	-2.266013
Bcl2l11	Bcl2l11-003	409.38007	0.001378685	0.005859284	0.20795006	-4.808846896	1.011269628	4.863040814	-2.265691
Gm13479	Gm13479-001	82	0.040361367	0.082707718	0.207964075	-4.808522826	0.181427195	0.872396807	-2.265594
Zwint	Zwint-002	4483.3645	3.2494E-05	0.000354475	0.207987473	-4.807981883	10.22416356	49.15759318	-2.265431
Nos1ap	Nos1ap-003	2890.1685	2.87901E-05	0.000325816	0.208018548	-4.807263639	3.832072458	18.42178259	-2.265216
Abcc5	Abcc5-006	539.89527	0.011001166	0.02970388	0.20813076	-4.804671822	0.94547431	4.542693777	-2.264438
Frmd4b	Frmd4b-002	3141.9572	9.17244E-06	0.000145848	0.208137096	-4.804525575	7.088146133	34.05517938	-2.264394
Taf1	Taf1-003	6312.3155	8.98549E-08	8.98218E-06	0.208202958	-4.803005728	12.53760496	60.21818842	-2.263938
Gbp3	Gbp3-001	2380.9676	0.00854635	0.024329397	0.208278378	-4.801266506	4.84337872	23.25435202	-2.263415
Gigyf2	Gigyf2-010	550.26973	0.006315509	0.019178827	0.208300604	-4.800754204	1.092811753	5.246320616	-2.263261
Ctdspl2	Ctdspl2-007	267.4347	0.046437123	0.092506918	0.208346483	-4.799697055	0.601663357	2.887801841	-2.262943
Mapk6	Mapk6-001	6152.5695	0.002788274	0.010144295	0.208363597	-4.799302834	16.62733833	79.79963198	-2.262825
Kansl1	Kansl1-001	3578.3113	0.000229768	0.001479793	0.208397061	-4.798532172	7.274885501	34.90877212	-2.262593
Ccnl2	Ccnl2-002	7606.8288	9.27138E-07	3.35538E-05	0.208498318	-4.796201757	16.3869117	78.59493467	-2.261892
Usp36	Usp36-001	708.36062	0.027838357	0.061518521	0.208507472	-4.795991188	1.665462487	7.987543413	-2.261829
Gm37321	Gm37321-001	519	0.008096771	0.023335599	0.208520134	-4.795699965	1.019342927	4.888462839	-2.261741
Scai	Scai-001	6517.6068	6.57366E-05	0.000589775	0.208605926	-4.793727676	15.25051452	73.10681353	-2.261148
Exoc2	Exoc2-001	1888.0175	0.035623791	0.074895024	0.208626563	-4.793253477	3.645024817	17.47152788	-2.261005
Rap1gds1	Rap1gds1-002	1152.5075	2.24635E-05	0.000273896	0.208631961	-4.793129461	2.642853957	12.66754116	-2.260968
Golim4	Golim4-003	2453.1241	0.000137601	0.001016283	0.208640768	-4.79292715	4.357689961	20.88609053	-2.260907
Stag2	Stag2-002	14184.136	2.44515E-06	6.18395E-05	0.208653004	-4.792646076	30.09503708	144.2348613	-2.260822
Dazap2	Dazap2-201	34920	1.04446E-09	9.17254E-07	0.208684884	-4.791913919	73.44793713	351.9561923	-2.260602
Atad2	Atad2-201	9386	0.000221017	0.001436813	0.208717802	-4.791158153	24.11670007	115.5469241	-2.260374
Fer	Fer-202	345.10914	0.005511792	0.017280982	0.208734911	-4.790765445	0.547740437	2.624095959	-2.260256
Macf1	Macf1-007	516.41206	0.006340721	0.019237283	0.208738476	-4.790683632	1.161235173	5.563110338	-2.260232
Tubb6	Tubb6-201	4073	0.038013466	0.078912568	0.208742208	-4.790597978	14.48966191	69.41414506	-2.260206
Rpl39	Rpl39-001	10657.534	6.07634E-07	2.58528E-05	0.20875707	-4.790256922	25.82398612	123.7035283	-2.260103
Nt5c3	Nt5c3-004	596.92867	0.000824173	0.003920546	0.208762811	-4.790125202	1.121486894	5.372062635	-2.260063
Rpl10	Rpl10-003	8957.7631	0.000841332	0.003982661	0.208773248	-4.789885728	21.66766631	103.7866036	-2.259991
Epb4.1l5	Epb4.1l5-003	246.71979	0.004883152	0.015704033	0.208831921	-4.788539964	0.505436516	2.420302957	-2.259586
10032F04R	0032F04Rik-	572.05245	0.008318378	0.023825427	0.208859323	-4.78791172	1.015106367	4.860239673	-2.259397
Pign	Pign-008	130.59575	0.016385453	0.040560984	0.208885327	-4.787315674	0.278453543	1.333045012	-2.259217
Mycbp	Mycbp-001	1329.4908	4.77558E-06	9.47836E-05	0.208909764	-4.786755676	3.153966458	15.09726685	-2.259048
RP23-4F17.	P23-4F17.1-0	1255	7.78048E-05	0.000666537	0.209051904	-4.783501046	3.11678339	14.90913661	-2.258067
930004J17R	30004J17Rik-	3702	3.58746E-06	7.89149E-05	0.209081044	-4.782834362	8.498046813	40.64475031	-2.257866
Gtf2i	Gtf2i-005	1246.0444	0.001160446	0.005120495	0.209116826	-4.782015954	2.902105611	13.87791533	-2.257619
Gm38248	Gm38248-001	985	0.000872918	0.004103974	0.209137533	-4.781542494	2.02605486	9.687667408	-2.257476
Agap1	Agap1-003	1860.149	1.52139E-05	0.000207573	0.209171717	-4.780761054	3.580353691	17.11681548	-2.25724
Tmem86b	Tmem86b-201	264	0.023142557	0.053211103	0.209180087	-4.78056977	0.693470314	3.315183221	-2.257183
Lgr5	Lgr5-001	142.01297	0.012421435	0.032660245	0.209201679	-4.780076359	0.283447041	1.354898501	-2.257034
Tgtp1	Tgtp1-001	2956.1764	0.008443376	0.024098802	0.209208932	-4.779910638	6.624530083	31.66466182	-2.256984
Gm4425	Gm4425-001	795	0.00023264	0.001492398	0.209209908	-4.779888344	1.369064132	6.543973686	-2.256977
Carf	Carf-006	569.8514	0.00018661	0.001266627	0.209255261	-4.778852367	1.14520966	5.472787893	-2.256664
Brd2	Brd2-002	1285.3045	0.002094595	0.008114454	0.209258841	-4.778770616	3.251913777	15.54015	-2.25664
Hspd1-ps3	Hspd1-ps3-00	2624	0.000326356	0.001925863	0.209262098	-4.778696246	7.099355901	33.92566539	-2.256617
Gpsm1	Gpsm1-009	236.9677	0.016180912	0.040152815	0.209275824	-4.7783828	0.406344336	1.941668784	-2.256522
Arhgef6	Arhgef6-001	1343.582	0.009362517	0.026178969	0.209313554	-4.777521471	2.979782165	14.23597327	-2.256262
Wdfy1	Wdfy1-002	72.312062	0.023423929	0.053688655	0.209451096	-4.774384175	0.167710275	0.800713282	-2.255315
Opa1	Opa1-001	6953.1975	1.46172E-07	1.18635E-05	0.209455705	-4.774279132	12.89479393	61.56334556	-2.255283
Metap1	Metap1-002	1635.7674	1.15544E-05	0.000170994	0.20951299	-4.772973739	3.322216849	15.85685377	-2.254888
Jagn1	Jagn1-201	1645	0.000135569	0.001003942	0.20952147	-4.772780568	4.001878886	19.10008978	-2.25483
Wnk1	Wnk1-203	19928.113	3.64375E-06	7.97944E-05	0.209548082	-4.772174426	40.82973228	194.8466042	-2.254647
Dnm3	Dnm3-011	245.25981	0.014422482	0.036748253	0.209586956	-4.771289306	0.435546077	2.07811634	-2.254379
Tek	Tek-001	19229.896	9.88401E-05	0.000790767	0.209639398	-4.77009574	22.1843662	105.8215507	-2.254018
Mtx2	Mtx2-001	1536.0625	0.000254006	0.001593062	0.209688163	-4.768986405	3.333934165	15.89948671	-2.253683
Blmh	Blmh-008	188.03286	0.006569349	0.019785758	0.209698602	-4.768749002	0.392913834	1.873707455	-2.253611
00066M21F	0066M21Rik-	504.40712	0.002744816	0.010030936	0.209717732	-4.768314021	1.106258949	5.274990059	-2.253479
Pus7	Pus7-009	433	0.001688351	0.006843774	0.209727248	-4.76809766	0.940967166	4.486623341	-2.253414
Gm37880	Gm37880-001	798	0.000182564	0.001245744	0.209734751	-4.767927088	1.534232122	7.315106891	-2.253362
Ccr2	Ccr2-001	2788.5396	0.047315694	0.093936653	0.209738008	-4.767853053	10.0833057	48.07571987	-2.25334
Ifi204	Ifi204-002	566.11316	0.002224696	0.008516356	0.209771215	-4.767098283	1.519739275	7.244746489	-2.253111
Frmd4a	Frmd4a-013	1448.3576	0.019128997	0.045860854	0.209873127	-4.764783442	3.52583808	16.7998549	-2.252411
Dnajc11	Dnajc11-002	713.71256	0.003063992	0.010926878	0.209922031	-4.763673428	1.696854223	8.083259372	-2.252075
Arid4a	Arid4a-004	5153.6366	4.87501E-05	0.000472065	0.209937905	-4.763313231	11.87452722	56.56209264	-2.251965
Atp5j2	Atp5j2-001	558.04727	0.000134661	0.000998518	0.209937972	-4.763311709	1.015837672	4.83875148	-2.251965
Clcn5	Clcn5-001	1917.5118	0.000268364	0.001660311	0.209949923	-4.763040576	4.797091563	22.84874176	-2.251883
Zcchc2	Zcchc2-001	5757.4562	3.62111E-06	7.94511E-05	0.209953391	-4.762961894	12.72938454	60.62957349	-2.251859
Rbm5	Rbm5-019	365.20707	0.004899433	0.015750476	0.209958191	-4.762853008	0.809773419	3.856831762	-2.251826
Amot	Amot-003	682.47747	0.017594592	0.042927396	0.209975341	-4.762463979	1.603400232	7.636135847	-2.251708
Ralgapa2	Ralgapa2-006	1383.8333	0.000311511	0.001857784	0.210023439	-4.761373331	2.580965706	12.28894128	-2.251378
Clcn3	Clcn3-007	281.2548	0.005049449	0.016126912	0.210032841	-4.761160177	0.54594616	2.599337114	-2.251313
Pdss1	Pdss1-002	309.14741	0.007500472	0.022007297	0.210087043	-4.759931821	0.663924817	3.160236861	-2.250941
Arhgap30	Arhgap30-004	206.14809	0.0288786	0.063314046	0.210094004	-4.759774106	0.686770279	3.268871389	-2.250893
Dleu2	Dleu2-008	5501.051	7.96139E-09	2.31621E-06	0.210100203	-4.759633661	12.36794108	58.8668687	-2.250851
Necab3	Necab3-002	134.98265	0.026090205	0.058423636	0.210129153	-4.758977924	0.254537924	1.211340362	-2.250652
Ints10	Ints10-002	1694.2848	3.25146E-05	0.000354544	0.210137913	-4.758779539	3.788988585	18.03096135	-2.250592
Rabggta	Rabggta-201	906.1612	0.034643086	0.07333286	0.210197798	-4.75742377	2.230099936	10.60953045	-2.250181
Decr2	Decr2-001	3883.1729	1.44363E-06	4.39114E-05	0.210205807	-4.757242512	7.642640034	36.35789207	-2.250126
Golt1b	Golt1b-001	3296.0941	0.00091317	0.004250622	0.210239133	-4.756488405	8.773190718	41.72957993	-2.249897
Fam45a	Fam45a-007	783.96566	0.000430757	0.002383153	0.210244157	-4.756374753	1.697400395	8.073472384	-2.249862
030005K06F	0005K06Rik-	733.03651	0.004364683	0.014382372	0.210253878	-4.756154842	1.19513329	5.684238985	-2.249796
Prpf38a	Prpf38a-003	635.34288	0.002006551	0.007851335	0.21030885	-4.754911649	1.411367464	6.710927596	-2.249419
Apex2	Apex2-001	1405.7401	0.002963872	0.010648548	0.210313515	-4.754806174	2.542185923	12.08760132	-2.249387
Rsrc2	Rsrc2-005	2085.3969	8.83129E-06	0.00014234	0.210334913	-4.754322451	4.577279375	21.7618621	-2.24924
Pbrm1	Pbrm1-001	13011.512	1.19623E-07	1.06142E-05	0.210347543	-4.754036999	28.12799774	133.721542	-2.249153
Bclaf1	Bclaf1-002	5683.5927	0.000981286	0.004482674	0.210402595	-4.752793092	13.17703933	62.6277415	-2.248776
Lamp2	Lamp2-001	8140.5327	1.56411E-06	4.59833E-05	0.210500698	-4.750578081	17.94124451	85.23128292	-2.248103
Brpf3	Brpf3-006	1064.2004	0.023687382	0.054154784	0.210511209	-4.750340881	2.113063473	10.0377718	-2.248031
Ubl3	Ubl3-002	3818.6197	3.45298E-06	7.69951E-05	0.210525694	-4.750014028	7.170216172	34.0586274	-2.247932
Glrx2	Glrx2-002	5868.1302	9.81529E-06	0.000152737	0.210527424	-4.749975007	12.23431308	58.11268137	-2.24792
Sirt7	Sirt7-007	454.5274	0.000598592	0.003070047	0.21053936	-4.7497057	0.909059652	4.317765812	-2.247838
Ift80	Ift80-010	158.27842	0.030429258	0.066096023	0.210548726	-4.74949442	0.311002852	1.47710631	-2.247774
Ogt	Ogt-001	18084.795	1.78526E-05	0.000232423	0.210548804	-4.74949266	36.50362091	173.3736796	-2.247773
Gm37864	Gm37864-001	1416	3.87822E-05	0.000400933	0.21055097	-4.749443809	2.844147249	13.50811755	-2.247759
Gprasp1	Gprasp1-003	1938.4042	0.000265448	0.001645834	0.210553947	-4.749376661	3.20739749	15.23313878	-2.247738

Gtf2b	Gtf2b-002	365	0.012235539	0.03227815	0.210569651	-4.749022445	0.832553746	3.953816428	-2.247631
Gm17083	Gm17083-001	554	0.002354712	0.008895278	0.210676135	-4.746622113	1.253231158	5.948614728	-2.246901
Med6	Med6-005	166.19604	0.041331091	0.084326553	0.210683515	-4.746455839	0.345080297	1.63790839	-2.246851
Zbed4	Zbed4-001	2692.6199	9.0162E-05	0.000739652	0.210709489	-4.745870738	6.518655585	30.93669679	-2.246673
Pdxdc1	Pdxdc1-006	196.33782	0.008858082	0.025032109	0.210806964	-4.743676298	0.366293548	1.737578024	-2.246006
Gm6877	Gm6877-001	164	0.011244556	0.030215445	0.210814094	-4.743515866	0.355754119	1.687525308	-2.245957
Umad1	Umad1-004	411.33465	0.020163178	0.047798233	0.210857143	-4.742547412	1.013080969	4.804584528	-2.245662
Api5	Api5-003	125.94177	0.024558011	0.055702774	0.210872642	-4.742198857	0.300117665	1.423217649	-2.245556
Osbpl9	Osbpl9-012	3324.6976	5.71282E-05	0.000531484	0.210953214	-4.740387604	6.283088412	29.78427442	-2.245005
Hcfc2	Hcfc2-002	1363.1232	8.81635E-05	0.000726739	0.210977152	-4.739849752	2.740558668	12.98983632	-2.244841
Zdhhc21	Zdhhc21-001	5694.1063	1.41206E-05	0.000196918	0.210982492	-4.739729789	13.53484182	64.15149296	-2.244805
Mfap2	Mfap2-001	192.35251	0.016043948	0.039892527	0.211018195	-4.738927847	0.319470586	1.513948057	-2.244561
Zfp719	Zfp719-001	3937.1781	5.80254E-06	0.000107215	0.211045457	-4.738315683	8.378240977	39.69875061	-2.244374
Gm37472	Gm37472-001	4033	2.34209E-05	0.000281944	0.211066375	-4.737846094	9.219907607	43.68250324	-2.244231
Capza1	Capza1-001	9868.9029	3.08805E-06	7.20303E-05	0.211072248	-4.737714253	23.31945665	110.4809222	-2.244191
Tgfbr1	Tgfbr1-001	7798.5398	1.08998E-05	0.000164538	0.211089231	-4.7373331	17.86158276	84.61626724	-2.244075
Csnk1a1	Csnk1a1-005	3751.0319	3.71793E-06	8.05433E-05	0.211092092	-4.737268883	8.242468	39.04678717	-2.244056
Nktr	Nktr-005	8223.545	1.11988E-07	1.01203E-05	0.211093669	-4.7372335	17.36270378	82.25118199	-2.244045
30617O03R	0617O03Rik-	1132.5939	0.00114137	0.005057824	0.2111221	-4.736595553	1.97633179	9.361084369	-2.24385
Gm18301	Gm18301-001	236	0.029815475	0.064999716	0.211171395	-4.73548987	0.569977022	2.699120415	-2.243514
Zfp384	Zfp384-004	891.04532	0.011767656	0.031340762	0.211201609	-4.73481242	1.846413604	8.742422065	-2.243307
Gm38026	Gm38026-001	296	0.007456326	0.021902158	0.211203315	-4.734774179	0.554601659	2.625913616	-2.243296
Bbip1	Bbip1-201	4451.6773	2.96195E-07	1.71608E-05	0.211279832	-4.733059419	7.539014848	35.68260524	-2.242773
Dus2	Dus2-005	157.45489	0.010361484	0.028388196	0.211297708	-4.732658993	0.265338468	1.255756487	-2.242651
Rapgef6	Rapgef6-001	16283.322	2.36668E-07	1.55689E-05	0.211320281	-4.732153467	34.25209582	162.086174	-2.242497
Kmt2e	Kmt2e-004	6371.7571	3.72963E-05	0.000390045	0.211336912	-4.731781067	13.22786736	62.59137232	-2.242383
Mbnl1	Mbnl1-002	5654.045	5.0279E-05	0.000482639	0.211343648	-4.731630257	8.457162148	40.01616431	-2.242337
Glud1	Glud1-001	58789.86	1.43519E-09	1.07321E-06	0.211362751	-4.731202616	109.2962545	517.102725	-2.242207
Zcchc7	Zcchc7-004	3216.8288	0.0001625	0.00114492	0.211396772	-4.730441206	6.765547419	32.00402429	-2.241975
Fnbp1	Fnbp1-003	9930.2025	2.51929E-07	1.58908E-05	0.211403853	-4.730282753	24.65743892	116.6366581	-2.241926
Ranbp17	Ranbp17-003	513.08371	0.027220496	0.060468414	0.21140597	-4.730235385	1.510109845	7.143175025	-2.241912
Ubl5	Ubl5-006	1157.2534	2.25582E-05	0.00027466	0.211417126	-4.729985786	2.324979872	10.99712175	-2.241836
Rcn1	Rcn1-002	5237.9619	2.01466E-05	0.000253871	0.211436123	-4.729560794	11.99923279	56.75110097	-2.241706
Tnfaip8	Tnfaip8-001	4949.8471	3.5263E-06	7.79701E-05	0.211455925	-4.729117899	10.64152969	50.32504851	-2.241571
Zfp606	Zfp606-004	1067.9981	0.000567354	0.002951117	0.211466754	-4.728875732	2.111060545	9.98294298	-2.241497
Gm38009	Gm38009-001	588	0.020792468	0.048951198	0.211515224	-4.727792071	1.287261255	6.085903554	-2.241167
Polk	Polk-201	4232.0887	8.51469E-05	0.0007101	0.211576716	-4.726418009	8.059684513	38.09343802	-2.240747
Fhl1	Fhl1-006	14907.64	0.013586453	0.035058843	0.211606191	-4.725759663	20.18630523	95.39562699	-2.240546
Atrx	Atrx-019	133.25564	0.024664174	0.05589312	0.21161776	-4.725501298	0.270946332	1.280357245	-2.240467
00012F01R	0012F01Rik-	1605.8193	0.000313754	0.001868885	0.211651177	-4.724755197	4.014524484	18.96764542	-2.24024
Ocln	Ocln-004	2579.2883	0.004171078	0.013883324	0.211671027	-4.724312127	5.806606978	27.43222376	-2.240104
Tial1	Tial1-007	2065.0707	0.004860631	0.015643359	0.211747624	-4.722603177	5.141109758	24.27942127	-2.239582
Wapal	Wapal-004	3981.2708	0.000353366	0.002046495	0.211822036	-4.720944141	7.514935745	35.47759188	-2.239075
Alg6	Alg6-007	421.55927	0.002122714	0.008194653	0.211832961	-4.720700662	0.970958518	4.58360452	-2.239001
Serac1	Serac1-001	3530.4445	3.72756E-06	8.05481E-05	0.21188005	-4.719651519	7.953207384	37.53636731	-2.23868
Gm20257	Gm20257-002	1036.4817	0.00065829	0.003303021	0.211882198	-4.719603683	2.056673573	9.70668417	-2.238666
Tamm41	Tamm41-002	180.81934	0.042281627	0.085882205	0.211889282	-4.719445881	0.375907946	1.77407721	-2.238617
Cox7a2	Cox7a2-201	2652	1.35355E-05	0.000191172	0.21189867	-4.719236793	5.599022006	26.42311066	-2.238554
Dbi	Dbi-001	3770.6562	0.000353387	0.002046495	0.211937894	-4.7183634	7.967381939	37.59300334	-2.238287
Gm38393	Gm38393-004	1625.6307	1.69864E-06	4.81608E-05	0.211975085	-4.717535544	3.337339553	15.74401796	-2.238033
Uprt	Uprt-001	1888	1.66362E-05	0.000221105	0.212115112	-4.714421279	4.329910163	20.41302061	-2.237081
Rbm25	Rbm25-017	5534.5509	4.92555E-07	2.31674E-05	0.212137599	-4.713921543	11.91548079	56.16864161	-2.236928
Eef1a1	Eef1a1-004	2359.1251	0.000233218	0.001495264	0.212141457	-4.713835821	5.678940376	26.76959257	-2.236902
Tssc4	Tssc4-001	675.46639	0.017998456	0.043688668	0.212162	-4.713379398	1.028807998	4.849162423	-2.236762
Gm37975	Gm37975-001	751	4.84427E-05	0.000470284	0.21218783	-4.712805636	1.818778877	8.571551342	-2.236586
RP23-1A12.	P23-1A12.2-0	955.29676	0.00160691	0.006593587	0.212239349	-4.711661651	1.831803552	8.630838548	-2.236236
Gm37024	Gm37024-001	2425	0.000169638	0.001180675	0.212324003	-4.709783097	4.920126242	23.17272741	-2.235661
Ddx27	Ddx27-002	904.63153	0.003395465	0.011817364	0.21232668	-4.709723707	2.037259995	9.594931696	-2.235642
P3h1	P3h1-007	1642.6707	0.000665175	0.003331223	0.212378636	-4.708571532	3.38305463	15.92935472	-2.235289
Chuk	Chuk-008	295.33068	0.004997426	0.015999845	0.212437067	-4.707276427	0.629434249	2.962921004	-2.234893
Sumf1	Sumf1-001	6773.0272	2.75458E-07	1.65516E-05	0.212449043	-4.707011072	13.22745118	62.26175919	-2.234811
Gm6776	Gm6776-201	174	0.006357304	0.019279057	0.212467222	-4.706608334	0.365653815	1.720989292	-2.234688
Gdap2	Gdap2-005	260.32609	0.007642569	0.022328584	0.212516182	-4.705524015	0.622030235	2.926978211	-2.234355
Cep128	Cep128-001	2639.5261	0.000474884	0.002570695	0.212520203	-4.705434989	6.630642034	31.20005503	-2.234328
H60c	H60c-201	209	0.027821776	0.061494335	0.212534629	-4.705115599	0.388029448	1.82572341	-2.23423
00029M09R	0029M09Rik-	1531.5092	0.000170327	0.001184267	0.212609394	-4.703461031	3.596755597	16.91719979	-2.233723
Cct3	Cct3-002	737.05972	0.004067084	0.013592663	0.212624398	-4.703129135	1.355615994	6.375637077	-2.233621
Macrod2os1	Macrod2os1-0	3123	1.09652E-05	0.000164816	0.21268288	-4.701835908	6.317751504	29.70503088	-2.233224
Gm2026	Gm2026-001	725.83504	0.000761523	0.003688609	0.212697645	-4.701509499	1.50836591	7.091596653	-2.233124
330007P06R	0007P06Rik-	1084.2984	4.70815E-05	0.000460726	0.212700472	-4.701447027	2.124336186	9.987454048	-2.233105
Hspe1	Hspe1-001	4136.9264	0.000426604	0.002365419	0.212799776	-4.699253069	11.92864803	56.05573589	-2.232431
Orc3	Orc3-004	940.63936	0.008413233	0.024030793	0.212838544	-4.69839712	1.970571534	9.25852762	-2.232169
Stox2	Stox2-202	3772.511	8.98432E-05	0.000738098	0.212846664	-4.698217873	6.273835407	29.47584564	-2.232114
Ubr1	Ubr1-004	240.14198	0.006388867	0.019361061	0.212853227	-4.698073012	0.482225585	2.265531005	-2.232069
Klhl12	Klhl12-003	1230.0089	0.000101987	0.000809702	0.212880922	-4.697461801	2.196475596	10.31786021	-2.231881
P23-113M2(	3-113M20.4-	1864	1.93115E-05	0.000245973	0.21289058	-4.697248694	3.916748736	18.39794289	-2.231816
Rpl30-ps3	Rpl30-ps3-001	178	0.019934068	0.04738959	0.213009719	-4.694621464	0.500421625	2.3492901	-2.231009
Kif1c	Kif1c-005	3664.5836	0.001311376	0.005635463	0.213043732	-4.693871961	5.437884467	25.52473343	-2.230778
Tmem88b	Tmem88b-001	950	6.76816E-05	0.000603043	0.21307488	-4.6931858	2.046287217	9.603606108	-2.230568
Rbm4b	Rbm4b-007	9515.9484	1.76869E-06	4.94915E-05	0.213224162	-4.68990002	20.48940472	96.09325962	-2.229557
Pdcd6ip	Pdcd6ip-001	23080.946	2.68659E-07	1.62936E-05	0.21332505	-4.68768202	42.11629543	197.4278008	-2.228875
Rabl3	Rabl3-001	686.31901	0.029258443	0.06398708	0.213345235	-4.687238509	1.316186694	6.169280959	-2.228738
Mbtd1	Mbtd1-004	1113.7635	8.18764E-05	0.000692582	0.213348865	-4.68715876	2.36672804	11.09323007	-2.228714
Fbxo30	Fbxo30-001	4306.2036	1.383E-06	4.27508E-05	0.213414987	-4.685706539	9.291919642	43.53920863	-2.228267
Gm20548	Gm20548-002	330.60717	0.005572587	0.017428645	0.213440795	-4.68513996	0.769248206	3.604035511	-2.228092
Gm27177	Gm27177-004	330.5809	0.014895588	0.037692856	0.213443555	-4.685079384	0.704540716	3.300829182	-2.228073
P23-403P24	23-403P24.5-	951.15068	3.62242E-05	0.000381748	0.213472643	-4.684440986	1.992048414	9.331633236	-2.227877
Tmem68	Tmem68-001	1569.9941	0.003273311	0.011500914	0.21347563	-4.684375459	3.872942444	18.14231654	-2.227857
Fam110a	Fam110a-002	310.93314	0.049923076	0.097846373	0.213498963	-4.683863506	0.677574413	3.173666064	-2.227699
Ing4	Ing4-003	949.50601	0.001914718	0.007565654	0.213562054	-4.682479773	2.286372295	10.70589203	-2.227273
Gm14443	Gm14443-002	262.42503	0.005733703	0.017800883	0.213596987	-4.681713985	0.556293464	2.604406888	-2.227037
Arrb2	Arrb2-007	1495.2635	0.039329134	0.080980092	0.213656498	-4.680409952	4.767531896	22.31400373	-2.226635
Ubap2l	Ubap2l-015	2270.774	6.18165E-06	0.000112141	0.213681074	-4.679871639	5.614405108	26.27469523	-2.226469
Ccdc88a	Ccdc88a-001	18896.901	6.87192E-09	2.13967E-06	0.213701753	-4.67941879	39.37240524	184.2399729	-2.226329
Gm11924	Gm11924-001	499	0.000789572	0.003789106	0.213711898	-4.67919667	0.983898339	4.603853833	-2.226261
Homer1	Homer1-002	403.73778	0.014529987	0.036958935	0.213725339	-4.678902383	1.271485864	5.949158237	-2.22617
Stk16	Stk16-002	700.31906	0.007709293	0.022454569	0.213727869	-4.678847006	1.430863033	6.694789219	-2.226153
Gm11221	Gm11221-001	183	0.005999098	0.018449233	0.213801153	-4.677243257	0.394962687	1.847336564	-2.225658
Krit1	Krit1-010	1400.6641	0.00067557	0.003367528	0.213872688	-4.675678842	3.173148301	14.83662238	-2.225176
Gnl3l	Gnl3l-002	3683.7045	2.05063E-05	0.000257362	0.213919523	-4.67465515	5.85863243	27.38708626	-2.22486
Phka2	Phka2-003	1041.0557	1.47202E-05	0.00020264	0.213961512	-4.673737775	2.213478667	10.34521886	-2.224577
Nmrk1	Nmrk1-001	1739.6379	7.98028E-07	3.06762E-05	0.213990271	-4.673109657	3.887421681	18.1663478	-2.224383
Nrep	Nrep-203	1186.2455	7.03922E-05	0.000619937	0.213990339	-4.673108154	2.471931899	11.55160511	-2.224382
Tmed5	Tmed5-001	9113.6734	2.1438E-07	1.48034E-05	0.213997269	-4.672956834	20.49105062	95.75379503	-2.224336
Matr3	Matr3-014	1923	7.52607E-07	2.94943E-05	0.214005659	-4.672773623	4.242686675	19.82511438	-2.224279
Kctd10	Kctd10-001	51624.326	2.11222E-09	1.31534E-06	0.21407688	-4.67121905	79.39859358	370.8882228	-2.223799
Tceanc	Tceanc-001	319.93605	0.038077439	0.078996443	0.214098286	-4.670752018	0.656448833	3.06610971	-2.223655

Chsy1	Chsy1-001	11682.397	8.80344E-05	0.00072585	0.214214023	-4.668228463	25.73859014	120.1536191	-2.222875
Rfwd2	Rfwd2-003	864.12417	0.000415243	0.00231479	0.214215833	-4.668189028	2.27467764	10.6186252	-2.222863
Myc	Myc-005	2502.0705	0.018939167	0.045525035	0.214220842	-4.668079859	8.961985164	41.83526244	-2.222829
230077H06I	0077H06Rik-	1780	3.70475E-06	8.05433E-05	0.214285254	-4.666676694	3.588067681	16.74435183	-2.222396
Ap2s1	Ap2s1-002	472.04854	0.006477746	0.019573251	0.214371208	-4.664805549	0.996721352	4.649511295	-2.221817
Cnot6l	Cnot6l-001	9199.3345	4.50639E-08	5.86858E-06	0.214392009	-4.664352958	21.01493764	98.02108656	-2.221677
Ncoa2	Ncoa2-001	4528.5014	9.03861E-05	0.000740959	0.214441423	-4.663278142	9.689851269	45.18647162	-2.221344
Gm12396	Gm12396-001	584	0.000732242	0.003579688	0.214467887	-4.662702719	1.319335489	6.151669171	-2.221166
Birc6	Birc6-002	3354.3529	0.002261575	0.008623796	0.214478044	-4.6624819	6.865705001	32.0112253	-2.221098
Usp40	Usp40-007	3905.3342	5.66237E-06	0.00010586	0.214481419	-4.662408538	8.460270375	39.44523683	-2.221075
Naa15	Naa15-001	1686.0942	0.000253126	0.001589223	0.214531175	-4.661327198	3.52804374	16.44536624	-2.220741
Nat10	Nat10-006	159.58894	0.034174979	0.072555909	0.214532406	-4.661300449	0.445459164	2.076419001	-2.220733
Zfp932	Zfp932-003	835.64686	0.017093923	0.041947901	0.214533859	-4.661268867	1.840260993	8.577951272	-2.220723
Srsf1	Srsf1-003	6295.5426	0.000299788	0.001810249	0.214544858	-4.661029912	16.02210601	74.67951536	-2.220649
Procr	Procr-001	14691.569	0.002431449	0.009120265	0.214557368	-4.660758152	35.4903356	165.411871	-2.220565
Fbxl5	Fbxl5-005	640.51264	0.037530099	0.078125929	0.214558157	-4.660741014	1.857591062	8.657750849	-2.220559
Dctn6	Dctn6-010	688.11972	0.00101373	0.004604808	0.214638914	-4.658987411	1.457886337	6.792274093	-2.220016
Rex2	Rex2-002	100	0.016395303	0.040576568	0.214716322	-4.657307801	0.18176406	0.846531172	-2.219496
Ube2d3	Ube2d3-002	7116.4596	2.52508E-05	0.000297406	0.214729905	-4.657013185	15.66868025	72.96925052	-2.219405
Fem1c	Fem1c-001	7938	4.12386E-07	2.07404E-05	0.214737693	-4.656844297	18.65969047	86.89527317	-2.219353
Map3k1	Map3k1-004	642.78308	0.008956384	0.025270297	0.214770638	-4.656129945	1.246350848	5.803171505	-2.219131
Pkn2	Pkn2-013	728.54381	0.001438071	0.006059037	0.214773922	-4.656058749	1.590020697	7.40322978	-2.219109
Gm6483	Gm6483-001	7209.8881	1.45901E-05	0.000201414	0.214823092	-4.65499305	15.57947042	72.52232652	-2.218779
Chchd2	Chchd2-002	397.18602	0.006161233	0.018841272	0.214883441	-4.653685712	0.765806869	3.563824484	-2.218374
Nfxl1	Nfxl1-009	388.07198	0.004612713	0.015021909	0.214898149	-4.653367201	0.89325235	4.156631189	-2.218275
Zfp760	Zfp760-201	3329	8.10054E-06	0.000134292	0.214900423	-4.653317963	6.533579394	30.40282235	-2.21826
Dnajc19	Dnajc19-001	2598.0907	8.67422E-05	0.000719177	0.214932087	-4.65263244	4.839567288	22.51672776	-2.218047
Asxl2	Asxl2-005	1132.7673	6.85473E-05	0.000609013	0.214967365	-4.651868893	2.855337394	13.2826552	-2.21781
Ocrl	Ocrl-006	238.24462	0.007978281	0.023080286	0.215023226	-4.650660385	0.577979266	2.687985276	-2.217436
Fyn	Fyn-008	2207.1298	0.000186671	0.001266742	0.215034922	-4.650407437	2.314139022	10.76168932	-2.217357
Pfdn5	Pfdn5-003	3593.4707	0.000306676	0.001839199	0.215046842	-4.650149664	9.380746809	43.62187662	-2.217277
Atp2c1	Atp2c1-005	2485.6647	0.036167457	0.075851778	0.215149569	-4.647929367	5.232516517	24.32036718	-2.216588
Rac1	Rac1-001	3617.5351	0.000103739	0.000819427	0.215163776	-4.647622474	7.849138245	36.4798313	-2.216493
C1d	C1d-001	2073.0638	5.89104E-06	0.000108361	0.215174656	-4.647387463	4.411168763	20.5004104	-2.21642
Gm37010	Gm37010-001	2634	2.26785E-06	5.91126E-05	0.215182145	-4.64722573	5.288906077	24.57874041	-2.21637
Zbtb6	Zbtb6-201	2719.5278	0.005218862	0.016559211	0.21519604	-4.646925649	5.564327831	25.85701772	-2.216277
Cnot6	Cnot6-002	898.14013	0.029640954	0.06468319	0.215315009	-4.64435808	1.680918015	7.806785164	-2.215479
Cenpc1	Cenpc1-004	5969.2775	6.1619E-06	0.000111901	0.215369649	-4.643179777	12.87222275	59.76804435	-2.215113
Gtdc1	Gtdc1-001	2769.6002	2.84308E-05	0.000322659	0.215448063	-4.641489859	5.599046343	25.98791682	-2.214588
Tet2	Tet2-001	8627.2825	2.23987E-06	5.86452E-05	0.21547842	-4.640835963	17.32809328	80.41683847	-2.214385
Ankrd17	Ankrd17-005	26604.605	5.59037E-08	6.76573E-06	0.215495887	-4.6404598	57.42179814	266.4635459	-2.214268
700060J05R	00060J05Rik-(	212.48788	0.022946868	0.052839198	0.21559658	-4.638292496	0.366339967	1.699191919	-2.213594
Zfand2a	Zfand2a-001	1808.3863	0.001504453	0.006272368	0.215643079	-4.637292338	2.678912011	12.42289814	-2.213283
Lmo7	Lmo7-005	861.38899	0.015167802	0.038198324	0.215647239	-4.637202888	1.365920843	6.334052076	-2.213255
Gm23158	Gm23158-201	143	0.028336748	0.062385653	0.215686174	-4.63636579	0.336547744	1.560358446	-2.212994
Gm7507	Gm7507-001	230	0.017645544	0.043030252	0.215704849	-4.635964401	0.438297757	2.0319328	-2.212869
Slc18b1	Slc18b1-005	657.81209	0.001331537	0.005699932	0.215706857	-4.635921244	1.460146339	6.76912343	-2.212856
Acpp	Acpp-002	798.47468	4.5434E-05	0.000450004	0.215748131	-4.635034354	1.396315646	6.471970986	-2.21258
Pak1ip1	Pak1ip1-003	1627.0871	0.006192482	0.018916564	0.215779278	-4.6343653	3.904570108	18.09520422	-2.212372
Luc7l3	Luc7l3-003	6283.2517	1.35086E-06	4.21759E-05	0.215820163	-4.633487379	13.82252531	64.04649657	-2.212098
Slc16a9	Slc16a9-001	1294.2986	0.003048275	0.010890105	0.215850598	-4.632834042	3.946292927	18.28252021	-2.211895
Irf1	Irf1-002	2073.3137	0.005605016	0.017493328	0.215852678	-4.632789411	3.98208517	18.44816201	-2.211881
Lonp2	Lonp2-002	1845.8961	0.001031724	0.004666874	0.215861881	-4.63259189	3.859606148	17.87998014	-2.21182
Gm17066	Gm17066-001	1062.9634	0.00016435	0.001154904	0.215871387	-4.632387889	2.193668397	10.16192292	-2.211756
Pgap2	Pgap2-005	1614.432	0.000398096	0.002242194	0.215957641	-4.630537713	3.291202972	15.24003948	-2.21118
Clk4	Clk4-003	1406.1916	0.000332771	0.00195563	0.21598211	-4.630013112	2.742266905	12.69673173	-2.211016
Ang	Ang-201	530.49584	0.045479885	0.09095977	0.215986371	-4.629921765	0.716075915	3.315292124	-2.210988
Gm37697	Gm37697-001	8080	3.51002E-06	7.77607E-05	0.216006632	-4.629487492	17.79834202	82.39720177	-2.210852
Cnot7	Cnot7-009	911.13309	0.017303288	0.042334281	0.216007715	-4.629464277	1.979368275	9.163414722	-2.210845
30579G24R	0579G24Rik-	484.90559	0.001073383	0.004809439	0.216019574	-4.629210139	0.999935456	4.628911353	-2.210766
Twsg1	Twsg1-201	8330	5.12783E-07	2.35742E-05	0.216032423	-4.628934805	18.71604834	86.63536757	-2.21068
Klhl12	Klhl12-005	1764.0164	6.92437E-05	0.00061311	0.216065763	-4.628220521	3.505540039	16.22441235	-2.210458
Phc3	Phc3-001	23733.072	1.69242E-06	4.81028E-05	0.216183511	-4.625699685	48.67945434	225.1765366	-2.209672
Zscan20	Zscan20-006	369.50615	0.046507751	0.092615297	0.216190144	-4.625557773	0.730992715	3.381249035	-2.209627
Glrx	Glrx-001	2047	0.000190299	0.001283019	0.216217616	-4.624970051	6.083512727	28.13606416	-2.209444
Gm11362	Gm11362-001	203	0.018405029	0.044473842	0.21625243	-4.624225499	0.551566464	2.550567709	-2.209212
Cep57	Cep57-007	1105.0796	2.526E-05	0.000297406	0.216258814	-4.624088985	2.452772017	11.34183606	-2.209169
Med23	Med23-002	1510.3037	0.013783942	0.035458917	0.216265296	-4.623950386	3.302851069	15.27221948	-2.209126
32428N05R	2428N05Rik-	2184.4728	0.040817709	0.083468059	0.216324758	-4.622679384	3.690999173	17.06230578	-2.208729
Gga2	Gga2-002	480.75367	0.001476367	0.006182366	0.216325281	-4.622668218	0.981901885	4.539006637	-2.208726
P23-187D2	23-187D22.1-	3999	2.61762E-07	1.60957E-05	0.216326237	-4.622647783	8.488796565	39.24071662	-2.208719
Rab7b	Rab7b-001	396.51216	0.028418324	0.062525059	0.216335241	-4.622455381	1.203606535	5.563617505	-2.208659
Nop58	Nop58-008	140.78612	0.041388065	0.084412626	0.216367484	-4.621766554	0.203509836	0.940574952	-2.208444
Rpl10-ps2	Rpl10-ps2-201	486	0.000544576	0.002858064	0.216417825	-4.620691482	1.119272847	5.171814511	-2.208109
Rmi2	Rmi2-002	1188	4.34767E-06	8.89532E-05	0.21651858	-4.618541277	2.544154688	11.75028344	-2.207437
Gm16008	Gm16008-001	259.07216	0.047338453	0.093947271	0.21653878	-4.618110431	0.436786547	2.01712851	-2.207303
Efcab14	Efcab14-001	17209.578	2.66038E-06	6.53097E-05	0.216563085	-4.617592136	34.93021147	161.2934698	-2.207141
Gm10032	Gm10032-201	191	0.006856954	0.020475212	0.216568338	-4.617480141	0.328253364	1.515703391	-2.207106
33422M21R	3422M21Rik-	1526	2.81699E-05	0.000321071	0.216602051	-4.616761461	3.153996426	14.56124915	-2.206881
Gm4924	Gm4924-201	620	0.000741874	0.00361645	0.216654475	-4.615644337	1.252746548	5.782232512	-2.206532
Tpp2	Tpp2-001	6980.8076	0.000343463	0.002002282	0.216678051	-4.615142118	17.22450238	79.49352638	-2.206375
Sclt1	Sclt1-005	389.43349	0.008113104	0.02337283	0.216732964	-4.613972789	1.054841034	4.867007828	-2.206009
Dhrs3	Dhrs3-001	780.7718	0.0023937	0.009014208	0.216751647	-4.613575099	1.461567874	6.743053151	-2.205885
Lpcat1	Lpcat1-003	3781.8217	0.003336682	0.011672083	0.216756432	-4.613473241	8.826422083	40.7204621	-2.205853
Cr2	Cr2-002	278.71832	0.009736666	0.026980648	0.216813475	-4.612259453	0.551864305	2.545341357	-2.205474
Cr2	Cr2-202	267.97899	0.010894772	0.029472076	0.216817695	-4.612169677	0.484004818	2.232312347	-2.205446
P24-423L4.	24-423L4.4-0	517	0.001058856	0.004758671	0.216834648	-4.611809097	1.197581577	5.52301761	-2.205333
Vta1	Vta1-002	854.9587	0.033636671	0.071627456	0.216871564	-4.61102406	1.915037543	8.830284185	-2.205087
P24-544J6.	24-544J6.4-0	263	0.006152541	0.01882814	0.216911172	-4.610182085	0.603116907	2.780478758	-2.204824
Rfx7	Rfx7-001	7545.5751	7.42069E-08	8.04299E-06	0.217020524	-4.607859113	18.46625132	85.08988441	-2.204097
Atxn2	Atxn2-018	1080.1531	2.99029E-05	0.000334807	0.217028888	-4.607681538	1.994604871	9.190504037	-2.204041
P23-407I6.	23-407I6.4-0	1073.1081	0.001848393	0.007357053	0.217055464	-4.607117384	2.152014484	9.914583341	-2.203864
Abhd2	Abhd2-001	27115.217	4.19167E-07	2.09584E-05	0.217084454	-4.60650213	53.66077069	247.1884545	-2.203672
00010M07R	0010M07Rik-	785.93673	0.000167818	0.001172055	0.21713879	-4.60534942	1.791472477	8.250356733	-2.203311
10403D21R	0403D21Rik-	609.20966	0.000405335	0.002273248	0.217140108	-4.605321469	1.346767506	6.202297307	-2.203302
Dhx9	Dhx9-007	1862.1294	9.21921E-05	0.000752342	0.21718278	-4.604416615	4.802563774	22.11300443	-2.203018
Tsc1	Tsc1-008	907.50633	0.000572555	0.002970761	0.217183357	-4.604404385	1.752564078	8.069513727	-2.203015
Rpl30-ps9	Rpl30-ps9-001	1416	1.81731E-05	0.000235589	0.217238063	-4.603244869	3.584006505	16.49805955	-2.202651
Nasp	Nasp-002	2350.1933	0.000935421	0.004327145	0.217290347	-4.60213724	5.477346378	25.20749974	-2.202304
Dennd2a	Dennd2a-006	1541	2.12418E-06	5.65732E-05	0.217306021	-4.601805299	3.316454111	15.2616761	-2.2022
Ensa	Ensa-002	2571.7345	0.000613912	0.003132206	0.217314454	-4.601626943	4.708533218	21.66691331	-2.202144
Ppp3cb	Ppp3cb-004	2206.3846	1.14992E-05	0.000170571	0.217318237	-4.601546621	5.160526162	23.74640172	-2.202119
Emp1	Emp1-002	11311.098	0.002187412	0.008395211	0.217430438	-4.599172088	26.60184534	122.3464646	-2.201374
Fry	Fry-005	896.20845	0.001893153	0.007496879	0.217463078	-4.598481771	1.698618261	7.811065112	-2.201158
10012C04R	0012C04Rik-	312	0.002614557	0.009652751	0.217560951	-4.596413069	0.732353993	3.366201466	-2.200508
P24-122N2	24-122N24.2-	1278	6.53536E-06	0.000115985	0.217645478	-4.594627969	3.018135603	13.86721026	-2.199948
Dgkd	Dgkd-002	1427.1443	0.000258593	0.001615616	0.217672389	-4.594059922	3.240001181	14.88475957	-2.19977

Il1rap	Il1rap-004	1220.9486	0.00485926	0.015640415	0.217700396	-4.593468899	3.388513607	15.56503187	-2.199584
00004A13R	0004A13Rik-	619.73902	0.000379973	0.002164322	0.21774091	-4.592547136	1.208289799	5.549127856	-2.199295
Rnf24	Rnf24-201	2875.3424	1.42263E-05	0.000197748	0.21774844	-4.592455402	5.963133534	27.38542481	-2.199266
00049G17R	0049G17Rik-	1110.3686	0.000299462	0.001808601	0.217755664	-4.592303049	2.319639417	10.65248717	-2.199218
Slc22a15	Slc22a15-001	2497.5249	1.24159E-05	0.000179732	0.217783964	-4.591706312	4.183740019	19.21050545	-2.19903
Strbp	Strbp-001	3842.2567	1.92686E-05	0.000245603	0.217822635	-4.590891123	7.72248036	35.45306653	-2.198774
P24-247A2	24-247A21.1-	5158	1.62531E-06	4.69663E-05	0.217904148	-4.589173765	9.004909425	41.32509409	-2.198234
Pde6g	Pde6g-002	521.84901	0.000668058	0.003341172	0.217963758	-4.587918695	1.078648082	4.948749703	-2.19784
Nfya	Nfya-010	3124.1357	0.001023194	0.004637341	0.218012472	-4.586893532	6.518108418	29.89786935	-2.197517
Gm37385	Gm37385-001	1000	4.56476E-06	9.20921E-05	0.218114245	-4.584753278	2.100035992	9.628146898	-2.196844
P23-408A1	23-408A1.4-(	305	0.015060096	0.037980231	0.218136088	-4.584294185	0.58640606	2.688257892	-2.1967
Fyco1	Fyco1-003	468.94842	0.00490907	0.015773374	0.218146947	-4.584065981	0.849598385	3.894615055	-2.196628
Amn1	Amn1-005	734.30967	0.00051626	0.002738407	0.21822098	-4.582510803	1.640710808	7.518575004	-2.196138
Manea	Manea-001	3309.0417	1.87824E-06	5.15462E-05	0.218240975	-4.582090966	6.233520706	28.56255891	-2.196006
Trim36	Trim36-202	520.07481	0.000474156	0.002568224	0.218260299	-4.581685288	1.182894552	5.419650568	-2.195878
Clk1	Clk1-009	1201.2201	0.000173958	0.001201222	0.218263224	-4.58162389	2.716343559	12.44526454	-2.195859
R3hdm1	R3hdm1-006	1401.8818	0.000261636	0.001627501	0.218285588	-4.581154479	2.613489215	11.97279782	-2.195711
Prrc2c	Prrc2c-014	694	0.015509642	0.038884223	0.218291376	-4.581033003	1.42506426	6.528266405	-2.195673
Sdha	Sdha-008	434.51552	0.003315875	0.011614718	0.21842213	-4.578290668	1.011119142	4.629197333	-2.194809
Gm22993	Gm22993-201	773	2.4421E-05	0.00028975	0.218423429	-4.57826345	1.746203672	7.994580447	-2.1948
Gm37238	Gm37238-001	1948	0.000365359	0.002095722	0.21844405	-4.577831262	4.449128877	20.36736126	-2.194664
Ampd3	Ampd3-001	2892.3403	0.000309769	0.001851911	0.218458536	-4.5775277	5.051133157	23.12170194	-2.194569
Agpat5	Agpat5-002	1618.0984	0.014621572	0.037147752	0.218502509	-4.57660648	4.920830778	22.52070603	-2.194278
Tanc2	Tanc2-005	908.86642	0.001491161	0.006226804	0.218568177	-4.575231469	1.226556114	5.611778131	-2.193845
Tbc1d19	Tbc1d19-002	1900.2319	2.03176E-05	0.000255555	0.218651324	-4.573491626	4.27927203	19.57121479	-2.193296
Larp4b	Larp4b-002	9982.8699	1.30866E-06	4.13237E-05	0.218663875	-4.57322911	23.16020111	105.9169059	-2.193213
Ccdc167	Ccdc167-001	1207.2016	0.004360788	0.014370922	0.218687641	-4.572732112	2.857996056	13.06885034	-2.193056
Alms1	Alms1-002	484.75596	0.000992097	0.004523338	0.21870232	-4.572425204	1.202352822	5.497668349	-2.19296
Ankrd44	Ankrd44-006	122.96114	0.01849497	0.044650224	0.218826529	-4.569829833	0.261418026	1.194635893	-2.19214
Alg14	Alg14-001	3160.4447	3.43239E-07	1.88049E-05	0.218832586	-4.569703349	6.912787207	31.58938685	-2.192101
Erp29	Erp29-003	1146.0294	3.0919E-06	7.20303E-05	0.218860042	-4.569130082	2.465683548	11.26602887	-2.19192
Samd12	Samd12-002	1270.4494	0.037986076	0.078864225	0.218864763	-4.569031511	2.045494925	9.345930767	-2.191888
Usp9x	Usp9x-003	1192.6396	0.00120124	0.005259169	0.218879971	-4.568714064	2.3081279	10.5451764	-2.191788
Mettl16	Mettl16-001	3662.4539	2.73936E-06	6.63998E-05	0.218886459	-4.568578631	8.041961029	36.74033131	-2.191745
Ide	Ide-001	8648.7512	3.43596E-06	7.68412E-05	0.218888111	-4.568544163	19.65715867	89.80459748	-2.191735
Arfip2	Arfip2-005	1580.2519	5.99944E-05	0.000551149	0.218925221	-4.567769734	3.402915449	15.54373419	-2.19149
Pgd	Pgd-003	826.40803	0.003689468	0.012614983	0.218959078	-4.567063446	1.842064884	8.412827195	-2.191267
St6galnac2	t6galnac2-00	2526.3254	0.003438378	0.011932764	0.218996528	-4.566282442	3.158756436	14.42377405	-2.19102
Acsl3	Acsl3-002	1204.0541	0.02566497	0.057644779	0.219082777	-4.564484776	2.294909128	10.47507778	-2.190452
Tkt	Tkt-002	293.61082	0.036810623	0.076959318	0.219100993	-4.564105275	0.618596634	2.82334016	-2.190332
Gm27896	Gm27896-201	594	0.001014597	0.004605946	0.219172498	-4.562616236	1.190014277	5.429578462	-2.189861
Lrrc2	Lrrc2-001	405	0.000923015	0.004284223	0.219182129	-4.562415767	0.928032275	4.234069084	-2.189798
Parp11	Parp11-010	209.53978	0.012059525	0.031934337	0.219193612	-4.56217674	0.495065539	2.258576485	-2.189722
Mis12	Mis12-001	2844.9113	2.94066E-05	0.00033109	0.219217519	-4.56167922	7.39295142	33.72427286	-2.189565
230415J03R	0415J03Rik-(	5122	0.000732681	0.003581323	0.219225252	-4.561518309	9.771489336	44.57282751	-2.189514
Smarca5	Smarca5-001	6700.0611	7.17544E-07	2.86691E-05	0.219281008	-4.560358454	16.3491323	74.55790373	-2.189147
Gm12322	Gm12322-001	91	0.039127342	0.080683545	0.219305579	-4.559847528	0.180010998	0.820822704	-2.188986
Tcn2	Tcn2-006	1363.3627	0.002739402	0.010016496	0.219389176	-4.558110009	1.961018764	8.938539257	-2.188436
Prpf3	Prpf3-007	265.88529	0.006460913	0.019536177	0.21939278	-4.558035144	0.686369385	3.128495781	-2.188412
Gm15823	Gm15823-001	771	0.000233685	0.0014977	0.219418482	-4.557501219	1.77215246	8.076586999	-2.188243
Gm15675	Gm15675-001	2270.4518	2.49061E-05	0.000294554	0.219421019	-4.557448535	4.190168038	19.09647519	-2.188226
Tardbp	Tardbp-202	2348.4846	0.005682372	0.017688017	0.219443841	-4.556974547	5.271608453	24.02258554	-2.188076
Gm15863	Gm15863-001	689.24588	0.000505789	0.002695811	0.219474797	-4.556331813	1.203353001	5.482875562	-2.187873
Maged1	Maged1-002	10912.08	3.68292E-05	0.000386381	0.219477852	-4.556268384	23.69166218	107.9455714	-2.187853
Cfl2	Cfl2-201	7219	3.19102E-07	1.80053E-05	0.219507412	-4.555654832	16.19584736	73.78269028	-2.187658
Gm15912	Gm15912-001	532	0.002227478	0.008526052	0.219510174	-4.555597496	1.023983438	4.664856386	-2.18764
Gdf3	Gdf3-001	91	0.03824671	0.079299582	0.219582758	-4.554091626	0.232572034	1.059154355	-2.187163
Dmtf1	Dmtf1-003	1146.0152	7.6919E-05	0.000661246	0.219594152	-4.553855324	2.280486188	10.38500417	-2.187088
Clpx	Clpx-002	390.50553	0.00900269	0.025377953	0.219675719	-4.552164453	0.818945381	3.727974052	-2.186553
330082K12R	0082K12Rik-	191.68791	0.010421766	0.028518318	0.21971578	-4.551334464	0.430420956	1.958989731	-2.18629
Ccdc62	Ccdc62-201	280.72869	0.012105239	0.032033101	0.21972396	-4.551165024	0.681009557	3.099386878	-2.186236
Snapc3	Snapc3-007	2630.7927	1.22303E-06	3.97941E-05	0.219749081	-4.550644733	5.150447636	23.43785741	-2.186071
Stard7	Stard7-005	503.4942	0.010859307	0.029397034	0.219762624	-4.550364305	0.839663164	3.820773287	-2.185982
Psmf1	Psmf1-003	1514.1465	0.000457544	0.00249735	0.219781177	-4.549980184	2.830045823	12.87665241	-2.18586
Cchcr1	Cchcr1-001	609.03957	0.015818062	0.039470248	0.219823592	-4.549102261	1.545644723	7.031295903	-2.185582
Gm37276	Gm37276-001	1070	4.47962E-05	0.000446008	0.219860352	-4.548341676	2.489300924	11.32219113	-2.185341
Pvrl3	Pvrl3-001	5420.1236	0.001510839	0.006289041	0.219861306	-4.548321938	7.906639037	35.96193979	-2.185334
Paip2	Paip2-007	461.98494	0.000331846	0.001951869	0.219862752	-4.548292012	1.055186065	4.799294351	-2.185325
Katnbl1	Katnbl1-002	548.06738	0.002740641	0.010018888	0.219879922	-4.547936851	1.360612251	6.187978595	-2.185212
Fkbp15	Fkbp15-005	1246.5389	0.001451252	0.006102787	0.219885869	-4.547813852	2.590231229	11.77988947	-2.185173
Ell2	Ell2-201	3086	7.94119E-06	0.000132353	0.219900678	-4.547507588	7.784813487	35.4014984	-2.185076
Gm37720	Gm37720-001	1075	0.003443558	0.011948318	0.219921351	-4.547080097	2.037441196	9.264408311	-2.18494
Baz1a	Baz1a-001	6306.1218	0.001604887	0.006587652	0.219961196	-4.546256431	19.01012414	86.42489913	-2.184679
Krit1	Krit1-002	2407.3057	0.000524837	0.00277274	0.219976157	-4.545947219	5.38627786	24.48573486	-2.184581
Srr	Srr-006	1608.1006	0.041398201	0.084428271	0.219983724	-4.545790851	2.313012919	10.51447297	-2.184531
Nfx1	Nfx1-002	3611.9467	4.66449E-05	0.000458024	0.219985984	-4.545744147	9.571154716	43.50802053	-2.184516
Suz12	Suz12-005	134.73063	0.021355934	0.050013042	0.219986797	-4.545727352	0.289970547	1.318127046	-2.184511
Rbm3	Rbm3-001	6820.3899	0.001421308	0.006001701	0.219997804	-4.545499918	17.63776159	80.17244389	-2.184439
Yap1	Yap1-001	4602.6764	1.49145E-05	0.000204575	0.220015216	-4.545140187	9.243781505	42.01428279	-2.184325
Toporsos	Toporsos-001	1003.1602	0.001256633	0.005450821	0.220066801	-4.544074776	2.023410822	9.194530077	-2.183987
Pdzd11	Pdzd11-001	968.94101	0.020976428	0.049297071	0.220069972	-4.544009307	1.94626525	8.84384741	-2.183966
Gm6153	Gm6153-001	176	0.013811028	0.035509926	0.220107821	-4.543227939	0.49839873	2.264339036	-2.183718
Dusp11	Dusp11-001	17964.369	2.70238E-08	4.32507E-06	0.220108914	-4.543205372	36.29334362	164.8881137	-2.183711
P23-235E15	23-235E15.6-	1839	0.000761381	0.003688609	0.220173246	-4.541877903	4.33923223	19.70826298	-2.183289
Orc5	Orc5-007	454.14538	0.000773962	0.003733361	0.220211665	-4.541085496	0.878368893	3.988748241	-2.183037
Vdac1	Vdac1-001	5911.9968	5.5732E-05	0.000521678	0.220240525	-4.540490447	13.33333802	60.53989392	-2.182848
Neil1	Neil1-002	462.02156	0.03247036	0.069602569	0.220254905	-4.540194002	0.984959753	4.471908364	-2.182754
Arl4c	Arl4c-001	5573.8158	0.002047856	0.007973972	0.220318338	-4.538886813	13.31219376	60.42254072	-2.182339
P24-324L19	24-324L19.2-	232.01958	0.009812771	0.027149802	0.220337108	-4.538500154	0.514233	2.333846549	-2.182216
Dennd6a	Dennd6a-201	11692	6.76342E-07	2.75916E-05	0.220343635	-4.538365717	25.86459614	117.3829964	-2.182173
Wdr44	Wdr44-004	1630.9643	0.004905907	0.015766849	0.22043849	-4.536412849	3.176511364	14.40996697	-2.181552
Carf	Carf-001	1644.3435	9.78562E-06	0.000152552	0.220439292	-4.536396357	3.148163025	14.28131528	-2.181547
Ccnl1	Ccnl1-012	585.36234	0.000712807	0.003511745	0.220460815	-4.535953475	1.520155032	6.895352502	-2.181406
Pdss2	Pdss2-001	743.02344	0.007542686	0.022102755	0.220477437	-4.535611511	1.567878289	7.111286814	-2.181297
Egr1	Egr1-201	23204.129	0.002405084	0.00904415	0.22054235	-4.534276527	75.84811642	343.9163339	-2.180872
Sri	Sri-007	658	0.003990131	0.013391671	0.220567267	-4.533764305	1.305994996	5.921073495	-2.180709
Cryzl1	Cryzl1-009	379.59179	0.005504166	0.017266687	0.220575906	-4.533586733	0.874268063	3.963570092	-2.180653
933434E20R	3434E20Rik-	894.00817	0.002082129	0.008081623	0.220597383	-4.533145348	1.997074646	9.053029642	-2.180512
Qk	Qk-201	19084.77	4.3247E-08	5.71384E-06	0.220640236	-4.532264908	43.85756674	198.7741107	-2.180232
Qsox1	Qsox1-007	4210.7184	0.008755909	0.024808891	0.220656198	-4.531937058	6.147624824	27.86064876	-2.180128
Camsap2	Camsap2-002	7894.7439	0.000125173	0.000946607	0.22065981	-4.53186288	17.25283505	78.18748274	-2.180104
Dlat	Dlat-001	5910.0495	3.6247E-06	7.94788E-05	0.220733447	-4.530351042	13.77792149	62.41882096	-2.179623
Gm38300	Gm38300-001	332	0.004539383	0.014836085	0.220816658	-4.52864384	0.779925678	3.532005618	-2.179079
Pla2g16	Pla2g16-001	5794.6978	0.012398275	0.032612006	0.220832726	-4.528314338	11.71096366	53.03092464	-2.178974
Gm37320	Gm37320-001	1235	0.000208636	0.001375244	0.220834538	-4.528277193	3.06798804	13.89270027	-2.178962
Mmachc	Mmachc-001	1324.6021	3.70426E-05	0.000388103	0.220863627	-4.527680775	3.219762468	14.57805663	-2.178772
Gm15283	Gm15283-001	717.35391	0.003132928	0.011112549	0.220867224	-4.527607045	2.090108737	9.463191043	-2.178749

Selp	Selp-005	1010.3197	0.003381885	0.011785801	0.22088315	-4.527280602	2.695830083	12.20477924	-2.178645
Lztfl1	Lztfl1-002	2126.2794	0.001513239	0.006294562	0.220982205	-4.525251262	4.674538765	21.15346244	-2.177998
Eftud2	Eftud2-004	117.44803	0.044985633	0.090139702	0.221014976	-4.52458027	0.171480775	0.775878532	-2.177784
Eogt	Eogt-001	20780.033	6.6882E-09	2.13967E-06	0.221099203	-4.52285665	33.56715395	151.8194255	-2.177234
Tbrg4	Tbrg4-007	567.94912	0.016368538	0.040532269	0.221109117	-4.522653856	1.233909544	5.580545756	-2.17717
Dctn3	Dctn3-201	211.04377	0.048729108	0.096144476	0.221152384	-4.521769017	0.534537615	2.417055628	-2.176887
Ctdspl2	Ctdspl2-008	955.0169	0.000698878	0.003461041	0.221189972	-4.521000608	1.924375043	8.700100741	-2.176642
Anapc1	Anapc1-004	1168.5767	3.55164E-06	7.83281E-05	0.221200028	-4.520795086	2.678454128	12.10874226	-2.176577
Pvrl3	Pvrl3-003	695.11732	0.009320235	0.026079103	0.221306419	-4.518621765	1.239001167	5.598577638	-2.175883
Rps2-ps5	Rps2-ps5-001	98	0.041426316	0.08448058	0.221322597	-4.518291467	0.250632046	1.132428635	-2.175777
Spcs3	Spcs3-001	13446	2.903E-07	1.69672E-05	0.221358951	-4.517549422	34.38343739	155.3288777	-2.17554
Gm37691	Gm37691-001	246	0.014831244	0.037566196	0.221378207	-4.517156462	0.694413877	3.136776131	-2.175415
Snx10	Snx10-001	1515.0803	0.004634242	0.015076252	0.221444002	-4.51581434	3.593076317	16.22566556	-2.174986
P24-447I18	24-447I18.1-(	12992	7.89911E-07	3.04904E-05	0.221448175	-4.515729252	27.49938526	124.1797784	-2.174959
Zbtb20	Zbtb20-001	285300.43	3.58624E-05	0.000378984	0.221451065	-4.515670303	561.1416482	2533.930677	-2.17494
D3Ertd254e	3Ertd254e-20	4188.2442	0.000345716	0.002011685	0.221506271	-4.514544876	9.143640705	41.27937629	-2.174581
Usp14	Usp14-001	3195.3981	0.000567988	0.002953331	0.221535092	-4.513957538	8.167915921	36.86962564	-2.174393
Hip1	Hip1-005	8285.4693	0.001612572	0.006608563	0.221591673	-4.512804958	15.00123856	67.69766376	-2.174024
Gm37566	Gm37566-001	789	0.00037177	0.002124664	0.221603286	-4.512568459	1.755159805	7.920278778	-2.173949
Gm37726	Gm37726-001	1455	0.000405274	0.002273248	0.221644753	-4.51172422	2.406405033	10.85703587	-2.173679
Hipk1	Hipk1-001	35631.201	3.32932E-08	4.79114E-06	0.221651546	-4.511585943	89.67371855	404.5706881	-2.173635
Uba5	Uba5-005	2387.5963	4.04861E-05	0.000413678	0.221693418	-4.510733825	5.37156424	24.22969651	-2.173362
30019P16R	0019P16Rik-	587.42239	0.01916829	0.045947737	0.22172763	-4.510037842	1.015323901	4.579149216	-2.17314
Sptlc2	Sptlc2-003	6714.4424	8.91009E-08	8.98218E-06	0.221740514	-4.509775777	11.85381215	53.45803492	-2.173056
Ppp3ca	Ppp3ca-005	312.32947	0.022594202	0.052224363	0.22174187	-4.509748204	0.548441666	2.473333817	-2.173047
Dclre1c	Dclre1c-003	1093.9318	0.000138682	0.001022065	0.22174733	-4.509637167	2.477324696	11.17183552	-2.173011
Tomm6	Tomm6-002	313.62367	0.016095201	0.039985258	0.221757721	-4.509425853	0.96242565	4.339987106	-2.172944
Tmem194	Tmem194-001	3088.6374	1.22804E-06	3.98678E-05	0.22180766	-4.508410569	7.059691685	31.82798861	-2.172619
Taf1a	Taf1a-201	712.50866	0.025245568	0.056923023	0.221859678	-4.507353523	1.458346225	6.573281996	-2.172281
Dlc1	Dlc1-004	356.39984	0.003123491	0.011084817	0.2219702	-4.505109239	0.747911043	3.369420949	-2.171562
Mast4	Mast4-015	902.18097	0.005025204	0.016064332	0.221978469	-4.50494142	2.564696608	11.55380798	-2.171508
Gm37268	Gm37268-001	461	0.001440854	0.006067035	0.222083599	-4.502808877	0.951081638	4.282538844	-2.170825
Ktn1	Ktn1-020	1889.8462	2.2305E-05	0.00027266	0.222103056	-4.502414407	3.533574496	15.90961672	-2.170699
Ankrd16	Ankrd16-004	2684.6362	0.019646305	0.046858353	0.222116473	-4.502142439	6.537201285	29.43141134	-2.170612
P24-404P10	24-404P10.7-	483	0.000470929	0.002554126	0.222136046	-4.501745749	0.994282645	4.476007671	-2.170485
Ifnar2	Ifnar2-001	1430.9342	0.001223014	0.00533948	0.222142514	-4.501614673	3.855372703	17.35540233	-2.170443
Calu	Calu-003	1695.4272	4.35941E-06	8.90871E-05	0.222158255	-4.5012957	3.655734587	16.45554238	-2.17034
Cox6a1	Cox6a1-002	1410.3841	0.024517118	0.055621069	0.222234573	-4.499749918	2.53401712	11.40244333	-2.169845
Pacrgl	Pacrgl-006	4433.536	2.57976E-07	1.60262E-05	0.222238944	-4.49966141	9.607070411	43.228564	-2.169816
Gm38192	Gm38192-001	419	0.003705741	0.01265925	0.222276909	-4.498892859	0.887811407	3.994168399	-2.16957
Gm17096	Gm17096-001	212	0.004387473	0.014443576	0.222287624	-4.498676003	0.518211803	2.331267003	-2.1695
BC024978	BC024978-00	1274.0326	0.00022097	0.001436813	0.222299139	-4.498442978	2.926278297	13.16369606	-2.169426
Stap1	Stap1-001	386.53737	0.00148006	0.006194045	0.222316291	-4.498095915	0.958558752	4.311689205	-2.169314
P23-281K1	23-281K10.2-	2660	4.72678E-06	9.43685E-05	0.222347801	-4.497458468	5.912989121	26.593423	-2.16911
Cux1	Cux1-001	13726.688	3.27817E-06	7.46028E-05	0.222359897	-4.497213805	28.58896667	128.5706956	-2.169031
Rasal2	Rasal2-001	10504.854	3.70749E-05	0.000388204	0.222418534	-4.496028187	32.014872	143.9397669	-2.168651
P23-113O9	23-113O9.3-(	830	4.29282E-05	0.000432204	0.222441885	-4.495556217	1.834601217	8.247552906	-2.1685
Stau1	Stau1-001	5288.9798	0.000230072	0.001481366	0.222462365	-4.495142355	11.91706016	53.56888185	-2.168367
Lpcat4	Lpcat4-004	902.7805	0.001192672	0.00523102	0.222500832	-4.494365211	1.45643298	6.545741716	-2.168117
Dip2b	Dip2b-001	15839.428	4.41459E-08	5.77098E-06	0.222538368	-4.49360714	32.85708684	147.64684	-2.167874
Gm13695	Gm13695-001	83.213426	0.035762268	0.075148454	0.222578097	-4.492805052	0.186186923	0.83650155	-2.167616
930036N10F	0036N10Rik-	712.07846	0.000515713	0.002736356	0.222583481	-4.492696393	1.752129106	7.871784113	-2.167582
Usf2	Usf2-002	2840.5379	0.000543052	0.002852332	0.22264659	-4.491422935	6.740450321	30.27421269	-2.167173
Gnb4	Gnb4-001	3863.7994	0.000975552	0.004462468	0.222657909	-4.491194604	8.819406203	39.60966955	-2.167099
Gm16555	Gm16555-001	201	0.006872834	0.020513462	0.222710712	-4.490129772	0.382634494	1.718078534	-2.166757
Bmpr2	Bmpr2-002	2330.9709	5.8668E-06	0.000108083	0.222780094	-4.488731389	4.468731999	20.05893759	-2.166308
Degs1	Degs1-001	16760.758	2.93864E-06	6.96047E-05	0.222908587	-4.486143913	44.09394415	197.8117792	-2.165476
Asf1a	Asf1a-201	2182	7.76575E-07	3.01561E-05	0.222909844	-4.486118608	5.034854227	22.58695324	-2.165468
Pigc	Pigc-006	418.11461	0.011877944	0.031563316	0.222919695	-4.485920362	1.159157641	5.199888866	-2.165404
Ctgf	Ctgf-003	7509.1169	0.009063221	0.025517085	0.222972218	-4.484863677	31.00383238	139.0479617	-2.165064
Pebp1	Pebp1-001	10044.15	1.48351E-07	1.19607E-05	0.222989455	-4.484516993	23.45196963	105.1707563	-2.164953
Gabpa	Gabpa-002	3498.2219	4.50127E-05	0.000447384	0.223037437	-4.483552235	8.265880139	37.06050538	-2.164642
Med28	Med28-005	186.12913	0.008799168	0.024896448	0.223047227	-4.483355446	0.32853025	1.472917886	-2.164579
Rapgef3	Rapgef3-005	1612.0695	0.001345961	0.005746355	0.223070396	-4.482889793	2.898328411	12.99288685	-2.164429
Gm11944	Gm11944-001	243	0.040216636	0.082485165	0.22307915	-4.482713862	0.694413877	3.112858712	-2.164372
Lrrfip2	Lrrfip2-011	368.06095	0.010253212	0.02814559	0.223114402	-4.482005599	0.688617299	3.086386589	-2.164144
Abi1	Abi1-001	695.49109	0.005406624	0.01704343	0.223115596	-4.481981608	1.814261985	8.131488851	-2.164137
Dhx33	Dhx33-005	3957.9219	3.90722E-07	2.01715E-05	0.223118193	-4.481929453	8.413856107	37.7103095	-2.16412
Zar1	Zar1-002	202	0.019736566	0.047021244	0.223132746	-4.481637136	0.42071038	1.885471262	-2.164026
130007C19F	0007C19Rik-	635.06557	0.004157909	0.013848913	0.223211208	-4.480061763	1.193390319	5.346462336	-2.163519
Mmgt2	Mmgt2-001	1074	0.001001155	0.004557962	0.223212298	-4.480039903	1.633595613	7.318570843	-2.163512
Gm12266	Gm12266-001	496	0.000564522	0.002938957	0.223233391	-4.479616591	0.833902911	3.735565317	-2.163375
Tmem33	Tmem33-008	242.11291	0.029471884	0.064371662	0.223288661	-4.478507758	0.526889853	2.359680293	-2.163018
Gtf2h2	Gtf2h2-005	440.32813	0.013330256	0.034556559	0.223289307	-4.478494806	1.087660311	4.871081053	-2.163014
Gm37082	Gm37082-001	7180	8.17089E-08	8.50945E-06	0.223295183	-4.478376945	15.42946546	69.0989624	-2.162976
Dcaf17	Dcaf17-003	192.93352	0.042537447	0.086273913	0.223298401	-4.478312403	0.414899263	1.858048514	-2.162955
Slc15a4	Slc15a4-007	622.10703	0.000269629	0.001665124	0.223302877	-4.478222634	1.465351033	6.562168164	-2.162926
Zmym5	Zmym5-202	5551.9384	0.000855235	0.004035237	0.223344342	-4.477391237	11.54356578	51.68506028	-2.162658
Casp8ap2	Casp8ap2-201	8184.9157	2.31174E-06	5.97562E-05	0.223356608	-4.477145361	19.18905518	85.91218937	-2.162579
Ilkap	Ilkap-011	453.34509	0.022596889	0.052226428	0.22336752	-4.476926643	0.818812325	3.665762712	-2.162509
Hspa4	Hspa4-003	2447.2105	0.000387908	0.002199645	0.223506327	-4.474146268	6.13604635	27.45356887	-2.161612
00037C18R	0037C18Rik-	591.51793	0.000939432	0.004341009	0.223542779	-4.473416688	1.228438052	5.49531528	-2.161377
Rpp40	Rpp40-003	542.91333	0.000504411	0.002690139	0.223585546	-4.47256104	1.400356977	6.263182056	-2.161101
Tcaf1	Tcaf1-002	1436.9678	0.000191528	0.001289634	0.223604645	-4.472179009	2.853611717	12.76186242	-2.160978
Stk11	Stk11-003	4881.0168	0.016466815	0.040715305	0.223628943	-4.471693094	9.905437203	44.29407513	-2.160821
Mbnl3	Mbnl3-004	3222.7174	1.07642E-05	0.000163058	0.223697926	-4.470314127	6.684789923	29.88311083	-2.160376
Sf3b1	Sf3b1-005	1756.1971	1.15212E-05	0.000170749	0.223701565	-4.470241412	4.029447975	18.01260521	-2.160353
Ergic1	Ergic1-002	821.32062	0.01387936	0.035644588	0.223714616	-4.469980622	1.97308262	8.819641078	-2.160269
Rbm39	Rbm39-011	139.00787	0.02408583	0.05485336	0.22372275	-4.46981812	0.298474093	1.334124911	-2.160216
921522P10R	1522P10Rik-	287	0.027558438	0.061056762	0.223823303	-4.46781003	0.527370738	2.356192271	-2.159568
Cln5	Cln5-201	5747	1.16422E-05	0.000171615	0.223829077	-4.467694791	12.46342354	55.68277242	-2.159531
Adrbk2	Adrbk2-002	1461.8672	0.003058682	0.010914758	0.223863591	-4.467005989	4.325674124	19.32281222	-2.159308
Gm4294	Gm4294-201	3962	6.38359E-05	0.000577033	0.223867501	-4.466927963	10.49341375	46.8733233	-2.159283
Dip2b	Dip2b-201	1907.7247	0.005923838	0.018262058	0.223901372	-4.46625223	4.678939431	20.89732367	-2.159065
Gm9840	Gm9840-001	145	0.015052336	0.037971753	0.223926503	-4.465750977	0.343480982	1.533900533	-2.158903
Gm37664	Gm37664-001	235	0.028093906	0.061970521	0.223949589	-4.465290626	0.48922782	2.184544399	-2.158754
Zfp808	Zfp808-001	1296	3.6094E-05	0.000380493	0.223954825	-4.465186227	2.919286721	13.03515886	-2.15872
Bpgm	Bpgm-001	33046.431	5.67039E-06	0.000105896	0.224030803	-4.463671902	94.70307243	422.7234434	-2.158231
Gm10482	Gm10482-001	126.60681	0.023325107	0.05353739	0.224055556	-4.463178774	0.291735949	1.302069697	-2.158072
Fkbp3	Fkbp3-201	3387.2046	1.21925E-05	0.000177397	0.224079825	-4.46269538	8.248304803	36.80967174	-2.157915
Mkln1	Mkln1-013	2113.0004	7.25464E-06	0.000124422	0.224101401	-4.462265729	4.396542299	19.61854003	-2.157776
Dst	Dst-001	35465.959	7.85215E-08	8.35206E-06	0.224121575	-4.461864057	82.60089452	368.5539623	-2.157647
Wdr11	Wdr11-002	329.24754	0.040085498	0.082265462	0.224163509	-4.461029387	0.454074379	2.025639147	-2.157377
Inip	Inip-001	1659.5796	0.000303153	0.001823179	0.224167332	-4.460953294	3.967067514	17.69690289	-2.157352
AK157302	AK157302-00	669.73989	0.002214492	0.008483207	0.224209154	-4.460121189	1.568130975	6.994054189	-2.157083
Gtf3c2	Gtf3c2-009	1168.918	0.003217488	0.011354705	0.224211509	-4.460074358	2.681014348	11.95752334	-2.157068
Npepps	Npepps-017	2232.5118	3.76585E-07	1.98431E-05	0.224223851	-4.459828856	4.638404967	20.68649231	-2.156988

Nlrx1	Nlrx1-202	320.81203	0.020016837	0.047536198	0.224229943	-4.459707683	0.803934019	3.585310721	-2.156949
Nlrx1	Nlrx1-201	108.34423	0.022568856	0.052193337	0.224271822	-4.458874917	0.271570087	1.210897047	-2.15668
Gm10419	Gm10419-002	681.75027	0.045316272	0.090671879	0.224324442	-4.457828978	1.338671791	5.967569899	-2.156341
Haus1	Haus1-201	577	0.006106619	0.018724412	0.224348703	-4.457346927	1.535378485	6.843714573	-2.156185
Rpl22l1	Rpl22l1-008	234.93074	0.040292907	0.082597084	0.224372443	-4.456875303	0.721278721	3.21464932	-2.156033
Zfp142	Zfp142-002	1254.2634	0.016401506	0.040588986	0.224403515	-4.456258192	2.709296791	12.07332602	-2.155833
)30024N18F	0024N18Rik-	809.19998	0.018402106	0.044469917	0.224417021	-4.455989987	1.25218187	5.579709873	-2.155746
Lrrc58	Lrrc58-001	51057.083	1.37827E-07	1.14024E-05	0.224447753	-4.45537987	122.4505639	545.5637775	-2.155548
Scamp1	Scamp1-001	8022.3467	3.71371E-07	1.96288E-05	0.224536594	-4.45361703	17.40669447	77.52275092	-2.154978
Uchl4	Uchl4-201	295	0.002196682	0.008424179	0.224551848	-4.453314503	0.733366293	3.265910751	-2.15488
Zyg11b	Zyg11b-001	16957.686	3.30154E-08	4.77122E-06	0.224589339	-4.452571105	35.6668723	158.809285	-2.154639
Srsf5	Srsf5-005	29454.604	2.09119E-07	1.46469E-05	0.224625124	-4.451861752	66.33985904	295.3358811	-2.154409
Brap	Brap-003	668.00511	0.000236877	0.001513342	0.224681889	-4.450737018	1.384873565	6.16370804	-2.154044
Sec61g	Sec61g-001	463.51221	0.008635878	0.024531708	0.224786686	-4.448662049	1.197266408	5.326233631	-2.153372
Ibtk	Ibtk-001	5274.2561	0.001512745	0.006294562	0.224802025	-4.448358514	14.54203367	64.6881793	-2.153273
Prcp	Prcp-201	7111	1.54628E-05	0.000209909	0.224848938	-4.447430394	14.26735599	63.45307269	-2.152972
Sulf1	Sulf1-001	61674.242	2.27152E-06	5.91386E-05	0.224881024	-4.446795826	109.1839921	485.5189202	-2.152766
Stard13	Stard13-001	8119.4716	5.57575E-05	0.000521699	0.224905734	-4.44630726	12.58399777	55.95232063	-2.152608
B3glct	B3glct-001	3768.2477	0.000120314	0.000917555	0.224930691	-4.445813926	7.867157584	34.97591874	-2.152448
Smim6	Smim6-001	344	0.023728008	0.054222329	0.22498587	-4.444723578	0.810136738	3.600833862	-2.152094
Mapk1	Mapk1-001	9471.4909	5.00467E-08	6.32509E-06	0.22501674	-4.444113811	19.55446436	86.90226513	-2.151896
Ghr	Ghr-010	659.89565	0.012003896	0.031823936	0.225073948	-4.442984215	1.042268109	4.630780755	-2.151529
Gm37789	Gm37789-001	546	0.000817656	0.003894953	0.225105347	-4.442364489	1.123115835	4.989289902	-2.151328
Pxdn	Pxdn-002	597.15208	0.029563104	0.064529751	0.225177729	-4.440936516	1.619319201	7.191293772	-2.150864
Slfn8	Slfn8-201	995.36898	0.003622619	0.012425785	0.225200617	-4.440485173	3.023337014	13.42508318	-2.150717
Sap18	Sap18-001	5973.3829	2.26498E-06	5.90827E-05	0.225206994	-4.440359432	12.52897024	55.6331312	-2.150676
Mllt10	Mllt10-003	389.50757	0.021645448	0.050542446	0.225242678	-4.439655966	0.597111667	2.650970375	-2.150448
Chm	Chm-001	4151.9004	0.00031073	0.001854482	0.225284554	-4.438830722	7.652772614	33.96936219	-2.15018
230325K18F	0325K18Rik-	1051.0221	0.000236525	0.001512049	0.225333166	-4.437873121	2.321031051	10.30044131	-2.149868
BC026585	BC026585-00	122.68256	0.049035427	0.096593051	0.225360125	-4.437342229	0.210355222	0.93341811	-2.149696
Ilkap	Ilkap-005	376.17266	0.005236853	0.016607613	0.225385261	-4.436847366	0.869299842	3.856950715	-2.149535
Rpl10-ps6	Rpl10-ps6-00	4523	3.83586E-07	2.00727E-05	0.225393928	-4.436676751	11.38418804	50.50796242	-2.149479
Pikfyve	Pikfyve-001	7183.5321	1.95572E-07	1.40427E-05	0.225413487	-4.436291781	16.4627339	73.0334911	-2.149354
Nasp	Nasp-004	2515.6965	6.39265E-07	2.6636E-05	0.225418449	-4.436194125	5.651342681	25.0704532	-2.149322
Macf1	Macf1-202	51454.906	9.54555E-08	9.28793E-06	0.225466807	-4.435242651	100.6722276	446.5057576	-2.149013
Bclaf1	Bclaf1-009	2407.3147	0.044902796	0.089994779	0.225555858	-4.433491599	7.009229259	31.07535903	-2.148443
Sort1	Sort1-005	1320.9045	0.022887934	0.05271895	0.225556168	-4.433485497	3.172053219	14.06325194	-2.148441
Prg4	Prg4-202	2215.9747	0.02245305	0.051981835	0.225599773	-4.432628582	5.970621854	26.46554908	-2.148162
Gm15739	Gm15739-001	383	0.002592013	0.009586053	0.225668344	-4.431281692	0.817460192	3.622396383	-2.147724
Vps39	Vps39-005	4001.8413	2.42039E-07	1.55824E-05	0.225807008	-4.428560513	8.815567288	39.04027319	-2.146838
510001J05R	0001J05Rik-	1564.7293	0.005305677	0.016790116	0.225864488	-4.427433506	3.390685587	15.01203498	-2.146471
Fam160a2	Fam160a2-00	2310.9549	0.02170548	0.050634294	0.225915989	-4.426424187	5.655927605	25.03553475	-2.146142
Auts2	Auts2-017	376	0.013270611	0.034425391	0.225974653	-4.425275075	1.00880917	4.464258078	-2.145767
Cep170	Cep170-009	403.35196	0.038945076	0.080392288	0.226002009	-4.424739431	0.975935966	4.31826235	-2.145592
Mdn1	Mdn1-002	206.0508	0.023670559	0.054123548	0.226031704	-4.424158116	0.442203389	1.956377712	-2.145403
Psmb4	Psmb4-001	3320.9903	0.000909443	0.004236046	0.226032531	-4.424141929	7.566590092	33.47566849	-2.145398
Zfp26	Zfp26-002	4292.8914	1.15963E-06	3.8523E-05	0.226039901	-4.423997696	10.31640409	45.63974791	-2.145351
Pea15a	Pea15a-004	8373.4606	0.000576492	0.00298397	0.226060393	-4.423596662	12.28197415	54.33049983	-2.14522
Rrbp1	Rrbp1-004	2356.0747	5.08292E-05	0.000486421	0.226064426	-4.423517736	6.024958559	26.65151105	-2.145194
Gm8837	Gm8837-001	967	0.000356514	0.002059121	0.226072905	-4.423351829	2.078091618	9.192130359	-2.14514
Tmem181b-p	mem181b-ps-0	1393.8141	8.47591E-05	0.000707876	0.226102849	-4.422766023	2.829952508	12.5162178	-2.144949
Smad3	Smad3-003	354.10917	0.020026631	0.04754364	0.226116365	-4.422501654	0.706935422	3.126423074	-2.144863
Tex13	Tex13-001	202	0.02491314	0.056314352	0.226206293	-4.42074351	0.604533103	2.672485793	-2.144289
Sec31a	Sec31a-013	1182.1876	0.000431184	0.002385021	0.226266662	-4.419564034	2.665032688	11.77828261	-2.143904
Cpsf2	Cpsf2-201	8176	2.34211E-05	0.000281944	0.226293041	-4.419048844	19.72865759	87.1819015	-2.143736
Auh	Auh-010	113.19721	0.035775506	0.075167847	0.226334283	-4.418243605	0.29958853	1.323655108	-2.143473
Tnks2	Tnks2-001	31247.273	1.4414E-09	1.07321E-06	0.226388423	-4.417186997	72.82730302	321.691816	-2.143128
Cnot6	Cnot6-004	704.90704	0.000182588	0.001245744	0.226458029	-4.415829304	1.446698275	6.388372637	-2.142684
42619	Sept6-002	1093.4028	0.002176861	0.008361276	0.226496363	-4.415081929	2.767270082	12.21772413	-2.14244
Ikbkg	Ikbkg-201	816.53659	0.008119932	0.023386114	0.226525991	-4.414504475	1.772981093	7.826832969	-2.142252
Zmynd8	Zmynd8-013	3075.4103	3.82143E-05	0.000396258	0.226526053	-4.414503256	7.287953147	32.1726929	-2.142251
Tnfaip6	Tnfaip6-001	362	0.010199855	0.028016779	0.226549575	-4.414044912	1.10372034	4.871871151	-2.142101
Rsl1	Rsl1-001	724	0.000706011	0.003486287	0.226560211	-4.413837706	1.780149644	7.85729162	-2.142034
Arl13b	Arl13b-002	350.54159	0.010681153	0.029034086	0.226561759	-4.413807534	0.679844196	3.000701434	-2.142024
Clk1	Clk1-006	4027.7548	6.25111E-05	0.000568055	0.22658251	-4.413403315	9.293742502	41.01703397	-2.141892
Gm16195	Gm16195-001	147	0.010689594	0.029046416	0.226595645	-4.41314748	0.377982007	1.668090343	-2.141808
Crlf3	Crlf3-002	1887.5922	0.00233572	0.0088357	0.226671084	-4.411678723	4.788319887	21.12452897	-2.141328
Opa1	Opa1-002	1039.235	0.002496168	0.009307827	0.22669508	-4.411211747	2.796818829	12.33736007	-2.141175
Nbeal1	Nbeal1-006	2967.2451	0.000105941	0.000831456	0.226735652	-4.410422414	6.843587911	30.18311351	-2.140917
Gm28809	Gm28809-001	715.20882	0.002847437	0.010319804	0.226775681	-4.409643916	1.44092523	6.353967172	-2.140662
Atp1b3	Atp1b3-001	43689.968	3.87381E-07	2.01638E-05	0.226822577	-4.408732199	94.36720459	416.0397334	-2.140364
Ehbp1	Ehbp1-001	985.59065	0.009476443	0.026432785	0.226990039	-4.405479657	1.871391253	8.244376097	-2.139299
Eif1ax	Eif1ax-001	7931.8621	1.99669E-08	3.56435E-06	0.227013928	-4.405016058	19.19861676	84.57021513	-2.139147
Tbce	Tbce-011	420.5739	0.032192453	0.069106331	0.227037096	-4.404566551	0.860807502	3.79148393	-2.139
Gm26947	Gm26947-001	940	1.44587E-05	0.000200247	0.22703967	-4.404516628	2.023224172	8.911324508	-2.138984
P23-235E15	23-235E15.5-	876	1.5993E-05	0.00021464	0.227049856	-4.40431902	2.144976379	9.447160262	-2.138919
Gm28417	Gm28417-00	906.79989	0.000344639	0.002007803	0.227055645	-4.404206723	2.17423566	9.575783312	-2.138882
Rab10os	Rab10os-001	2774.4845	9.54699E-06	0.000149923	0.227132707	-4.402712466	6.590197324	29.01474391	-2.138393
Ptpn11	Ptpn11-002	3176.2121	0.000102112	0.00081032	0.227164252	-4.402101085	6.177702894	27.19487261	-2.138192
Bloc1s3	Bloc1s3-201	859	0.001669165	0.006784821	0.227207322	-4.401266606	1.764882357	7.767717781	-2.137919
Ankra2	Ankra2-003	584.80908	0.004280583	0.014168296	0.227211761	-4.401180625	1.306934072	5.752052917	-2.137891
Styk1	Styk1-004	550	0.000270333	0.001668872	0.227235437	-4.400722051	1.254633673	5.521294073	-2.13774
Pfkp	Pfkp-004	1770.0381	3.10812E-05	0.000343439	0.227269955	-4.400053673	4.819511697	21.20611014	-2.137521
Gm10233	Gm10233-201	524	0.001473651	0.006173255	0.227286048	-4.399742129	1.45759574	6.413045386	-2.137419
Nmd3	Nmd3-001	3505.358	7.35309E-06	0.000125545	0.227299818	-4.399475591	8.757829376	38.52985657	-2.137332
Lrba	Lrba-003	5824.657	0.00025015	0.001576675	0.227348014	-4.398542925	9.484551367	41.71820631	-2.137026
Ppp2r1b	Ppp2r1b-001	564.89325	0.008589565	0.024434601	0.227395751	-4.397619551	1.352233646	5.946609122	-2.136723
Tgtp2	Tgtp2-001	3593.7951	0.01281341	0.033467496	0.227448005	-4.39660923	8.176627716	35.94943689	-2.136391
Nup88	Nup88-002	3060.2083	9.50955E-05	0.000769435	0.227480127	-4.39598841	6.143961174	27.00878211	-2.136188
Trim2	Trim2-009	649.42842	0.004283114	0.014171511	0.227529946	-4.39502587	1.405226574	6.176007145	-2.135872
Mtif2	Mtif2-002	3142.3443	8.21433E-05	0.000693325	0.227532979	-4.394967283	6.925433133	30.43705204	-2.135852
RP23-2C16.	P23-2C16.2-0	8346	0.003953019	0.013301003	0.227537171	-4.394886326	14.27583467	62.74067059	-2.135826
Ybx1	Ybx1-006	747.91651	0.000328602	0.001937778	0.227553604	-4.39456894	1.361568036	5.9835046	-2.135722
Tram1l1	Tram1l1-001	495	0.000675066	0.003366973	0.227648273	-4.392741423	1.167248429	5.127420524	-2.135122
Rnf24	Rnf24-003	1186.0039	4.56901E-05	0.000451757	0.227655747	-4.392597209	2.468758732	10.84426271	-2.135074
Prdm5	Prdm5-004	652.70963	0.001355004	0.005778001	0.227656624	-4.392580296	1.686513252	7.408144881	-2.135069
Rab3gap2	Rab3gap2-00	1836.9095	0.000286816	0.001746698	0.227661606	-4.392484175	4.181128881	18.36554244	-2.135037
Stxbp6	Stxbp6-002	22215.937	0.001073562	0.004809593	0.2277179	-4.391398309	31.97312654	140.4067338	-2.13468
Nae1	Nae1-002	2786.464	2.09407E-05	0.000261282	0.227726575	-4.391231011	5.969780002	26.21468308	-2.134625
Guf1	Guf1-009	347	0.007643934	0.022329375	0.227766898	-4.390453614	0.816260478	3.583753765	-2.13437
Zfp160	Zfp160-201	2630	3.31002E-05	0.000358193	0.227767688	-4.390438385	6.122048941	26.87847866	-2.134365
P23-363M4	23-363M4.2-	175	0.03103001	0.06718363	0.22781161	-4.389591902	0.467269765	2.051123575	-2.134087
Sike1	Sike1-001	13806.436	9.84894E-07	3.45267E-05	0.227889257	-4.388096274	25.27659156	110.9161172	-2.133595
Cep76	Cep76-201	1083	0.004339965	0.014314695	0.227894557	-4.38799423	2.637880155	11.5750029	-2.133562
Fn1	Fn1-001	11017.862	0.005448029	0.017136099	0.227896567	-4.387955522	33.82799172	148.435723	-2.133549
Acox3	Acox3-002	510.40344	0.000934791	0.004324815	0.227906769	-4.387759094	1.36342695	5.982388997	-2.133484
P23-293K21	23-293K21.2-	2149	9.0338E-06	0.000144389	0.227923238	-4.387442053	5.032618261	22.080321	-2.13338

Tmem243	Tmem243-004	2788.1741	1.47674E-06	4.44277E-05	0.227928692	-4.387337079	6.488446874	28.46700356	-2.133346
Ttf2	Ttf2-001	831.59665	0.015423414	0.038701421	0.227971743	-4.386508544	2.571800472	11.28122475	-2.133073
Mkln1	Mkln1-004	1444.8675	0.000189105	0.001277485	0.227998841	-4.385987217	2.904758544	12.74023384	-2.132902
Gm5806	Gm5806-201	218	0.005086599	0.016212172	0.228056818	-4.384872203	0.546981009	2.398441824	-2.132535
030013D06Rik	0013D06Rik-	807	0.000604527	0.003094003	0.228092846	-4.384179581	2.018099051	8.847708653	-2.132307
Gm15965	Gm15965-001	1219	1.09587E-05	0.000164816	0.228129942	-4.383466678	2.59227972	11.36317177	-2.132072
Pard3	Pard3-010	637.14816	0.000755684	0.003665137	0.228148862	-4.383103161	1.274727535	5.587262288	-2.131953
Polr1c	Polr1c-001	2908.5508	2.15295E-05	0.000266108	0.228191423	-4.382285654	6.864863955	30.08379483	-2.131684
Gm37349	Gm37349-001	327	0.004776833	0.015424394	0.228195165	-4.3822138	0.703653525	3.083560186	-2.13166
Gm5582	Gm5582-201	157	0.037546743	0.078145738	0.228226234	-4.381617233	0.389445645	1.70640175	-2.131463
Gm26982	Gm26982-001	646	0.003769511	0.012814845	0.228227816	-4.381586859	1.267515214	5.553728003	-2.131453
Hsp90b1	Hsp90b1-001	165486.73	3.27331E-06	7.45418E-05	0.2282395	-4.381362562	343.7138444	1505.93497	-2.13138
Xpr1	Xpr1-004	1252.8811	0.000171995	0.001192233	0.22824827	-4.381194208	3.079067068	13.4899908	-2.131324
42431	Marc2-002	1487.3407	1.51342E-05	0.000206759	0.228305151	-4.380102671	2.856779287	12.51298659	-2.130965
Lrch2	Lrch2-001	521.77638	0.019561443	0.046702414	0.228334481	-4.379540027	1.106006212	4.843798477	-2.130779
Amot	Amot-201	1654.4997	0.002749041	0.010044236	0.228378007	-4.37870535	2.667629975	11.68076564	-2.130504
Msi2	Msi2-001	15138.66	1.66608E-08	3.20336E-06	0.228382278	-4.378623458	32.29124391	141.3911981	-2.130477
Il13ra1	Il13ra1-001	7881	0.000790345	0.003791223	0.228462907	-4.377078162	24.2699581	106.2315036	-2.129968
Chpt1	Chpt1-002	753.06044	0.001006683	0.004578262	0.228590701	-4.374631138	1.627945665	7.121661797	-2.129161
Fkbp15	Fkbp15-006	1349.4577	6.14536E-05	0.000560081	0.228603413	-4.374387887	2.994439395	13.09883942	-2.129081
Mkln1	Mkln1-001	10707.767	4.80516E-08	6.16392E-06	0.228614868	-4.374168695	22.54732209	98.62579044	-2.129009
Ccdc60	Ccdc60-002	1375.7524	1.48091E-05	0.000203537	0.228668685	-4.373139241	2.891394045	12.64446876	-2.128669
Ifih1	Ifih1-002	1439.3958	0.000401216	0.002254948	0.228683623	-4.37285358	2.92560247	12.79323124	-2.128575
Brwd1	Brwd1-003	1523.3722	8.59878E-05	0.000714849	0.228689984	-4.37273196	3.829850638	16.74691028	-2.128535
Nkrf	Nkrf-001	1382	0.002751509	0.010046384	0.228755568	-4.371478293	3.773846268	16.49728704	-2.128121
Chid1	Chid1-003	304.37296	0.011483131	0.030752775	0.228791142	-4.370798592	0.672210097	2.938094946	-2.127897
Gapvd1	Gapvd1-001	1307.9347	0.020650648	0.048702256	0.228840799	-4.369850147	3.754907719	16.40838405	-2.127584
Arpc1b	Arpc1b-005	588.38117	0.024139591	0.054953871	0.228876785	-4.369163088	1.448682553	6.329530335	-2.127357
P23-70G12	23-70G12.3-(	1040	5.54426E-05	0.000519395	0.228913696	-4.368458587	2.144438418	9.367890423	-2.127124
Trmt1l	Trmt1l-006	1496.1997	2.60831E-05	0.00030448	0.228922087	-4.368298465	3.276456237	14.31253875	-2.127071
Nsf	Nsf-007	296.45321	0.032680343	0.069973853	0.228923475	-4.368271965	0.596120082	2.604014642	-2.127063
P23-209N1	23-209N19.1-	1054	4.87265E-05	0.000472065	0.2289488	-4.36778879	2.578184784	11.2609666	-2.126903
RP23-333I5	23-333I5.3-0	792	0.00087836	0.004122204	0.228971342	-4.367358782	1.715963213	7.494227006	-2.126761
Csgalnact2	Csgalnact2-00	2583.0169	0.000964363	0.004423978	0.229008199	-4.366655888	5.816241039	25.39752318	-2.126529
Slc35b4	Slc35b4-004	2070.5488	7.06547E-05	0.000621687	0.229012524	-4.366573413	5.145576653	22.4685382	-2.126502
Zfp709	Zfp709-001	735.04892	0.001583229	0.006519277	0.229016751	-4.366492827	1.423621259	6.216232017	-2.126475
Cdc23	Cdc23-002	3114.5347	5.33143E-05	0.000504703	0.229022691	-4.366379569	7.993437301	34.90238131	-2.126438
Slc12a6	Slc12a6-007	220.28052	0.037673965	0.07834922	0.229028467	-4.366269451	0.558625262	2.439108418	-2.126401
BC027231	BC027231-00	2227.0157	1.32672E-06	4.17265E-05	0.22904894	-4.365879194	5.31287862	23.19538623	-2.126272
Sephs1	Sephs1-001	3226.5448	4.13625E-05	0.000420251	0.229065476	-4.365564028	7.487330709	32.68642161	-2.126168
Myg1	Myg1-009	637.25151	0.007814842	0.022713707	0.229067791	-4.365519892	1.498823259	6.54314275	-2.126153
Ppfia1	Ppfia1-005	439.55156	0.004159475	0.013851435	0.229085795	-4.365176812	0.612502766	2.673682873	-2.12604
Dnaja1	Dnaja1-201	760.53938	0.010111367	0.027829823	0.229142516	-4.364096278	1.567230942	6.839546723	-2.125683
Ppp2r2d	Ppp2r2d-003	642.86118	0.012742193	0.033309428	0.229142918	-4.364088615	1.283468166	5.601168811	-2.12568
Gm15879	Gm15879-002	667.41843	0.000510387	0.002713559	0.229155863	-4.363842094	1.486229177	6.485669444	-2.125599
Celf2	Celf2-020	703.19878	0.023945098	0.054610291	0.229181061	-4.363362297	1.963894185	8.569181842	-2.12544
Zfp329	Zfp329-001	6077.3727	3.21397E-07	1.80753E-05	0.229194694	-4.363102748	13.57205241	59.21625917	-2.125354
Gm37410	Gm37410-001	360	0.002008593	0.007855922	0.229203455	-4.362935984	0.826686158	3.606778787	-2.125299
Trove2	Trove2-001	10398.099	1.98789E-06	5.37798E-05	0.22925917	-4.361875683	20.29760344	88.53562289	-2.124949
Trim13	Trim13-202	247.83228	0.019706423	0.046965764	0.229270455	-4.361660991	0.534433407	2.331017345	-2.124878
Hspe1-ps2	Hspe1-ps2-00	100	0.035527761	0.074734419	0.229316122	-4.36079239	0.163906849	0.714763741	-2.12459
Rhot1	Rhot1-004	1132.3959	0.007934829	0.02298443	0.229340513	-4.360328613	2.442889605	10.65180144	-2.124437
Arpp19	Arpp19-001	6368.2036	8.32864E-07	3.14852E-05	0.22935831	-4.359990273	13.78854404	60.11791791	-2.124325
Rbm33	Rbm33-001	2958.1735	5.48689E-06	0.000103655	0.229382087	-4.359538319	6.459917294	28.16225698	-2.124175
Gm26808	Gm26808-201	164	0.031813366	0.068476301	0.229400179	-4.359194511	0.348066437	1.517289303	-2.124062
Plec	Plec-014	2526.0156	0.005592468	0.017465984	0.22940796	-4.359046651	2.84783424	12.41384231	-2.124013
Gm37755	Gm37755-001	583	0.000538989	0.002832799	0.229423045	-4.358760039	1.069814037	4.663062674	-2.123918
10013O21Rik	0013O21Rik-	324.30379	0.007608366	0.022257135	0.229438929	-4.358458282	0.695351545	3.030660702	-2.123818
Rpl15-ps3	Rpl15-ps3-001	1772	0.000305257	0.001832935	0.229440536	-4.358427761	4.819227103	21.00425319	-2.123808
Gm14322	Gm14322-002	496.5856	0.010557838	0.02877121	0.229453606	-4.358179496	1.169725005	5.097871534	-2.123726
Gm29123	Gm29123-001	409	0.002960821	0.010644287	0.229460527	-4.358048041	0.820550443	3.57599825	-2.123682
Gm15883	Gm15883-001	88.062494	0.045903688	0.091653703	0.229464109	-4.357980003	0.13258903	0.577820339	-2.12366
Esd	Esd-003	1739.1767	0.001416062	0.005985556	0.229485348	-4.357576679	3.993429248	17.40167416	-2.123526
Ctsa	Ctsa-005	293.93602	0.017933657	0.043564474	0.229516879	-4.356978035	0.723877725	3.153919346	-2.123328
Pqlc1	Pqlc1-004	2115.8049	1.53271E-05	0.000208646	0.229566389	-4.35603837	4.232916478	18.4387466	-2.123017
Hiat1	Hiat1-006	1549.2359	6.52908E-06	0.000115985	0.229583264	-4.355718185	3.410884493	14.85685161	-2.122911
Dnttip1	Dnttip1-004	190.66675	0.027490226	0.060949005	0.229592613	-4.355540836	0.49611892	2.160866216	-2.122852
Lman2l	Lman2l-007	445.92709	0.0025115	0.009349878	0.229666033	-4.354148444	0.932333792	4.059519732	-2.122391
Rpl6	Rpl6-008	3359.8847	0.00071961	0.003534583	0.229757719	-4.352410896	8.09802689	35.24594048	-2.121815
Gm26518	Gm26518-201	2592.3524	2.91646E-06	6.9223E-05	0.229770179	-4.352174884	5.73763097	24.9711734	-2.121737
St7l	St7l-004	678.96362	0.002629457	0.009698351	0.229773508	-4.352111812	1.416766679	6.165927	-2.121716
Gm5891	Gm5891-001	252	0.005328405	0.016846475	0.229890966	-4.349888202	0.549476538	2.390161511	-2.120978
Cdan1	Cdan1-201	3886.2532	0.000447658	0.002454341	0.22989291	-4.349851417	8.471408071	36.8493664	-2.120966
810429I04Rik	0429I04Rik-(	1834.344	8.13478E-05	0.000689815	0.229898486	-4.349745906	4.601061671	20.01344917	-2.120931
Fam198b	Fam198b-002	680.69171	0.017040585	0.041852996	0.22995274	-4.348719658	0.994404223	4.32438519	-2.120591
Pvrl3	Pvrl3-002	2642.0665	1.99378E-05	0.000251702	0.230035531	-4.347154531	4.864256925	21.14567653	-2.120071
Rsbn1l	Rsbn1l-002	631.48582	0.001671647	0.006792492	0.230072921	-4.346448057	1.433028539	6.228584112	-2.119837
Rsrc2	Rsrc2-006	193.75716	0.041609252	0.08476773	0.230092072	-4.346086301	0.477848089	2.076769036	-2.119717
Mdn1	Mdn1-201	5367.8911	0.000725088	0.003554355	0.230156969	-4.344860829	15.66608226	68.06694714	-2.11931
Dars2	Dars2-003	412.00102	0.009264919	0.025954807	0.230234699	-4.343393947	1.051014647	4.564970655	-2.118823
Setdb1	Setdb1-004	1249.7446	0.000964082	0.004423284	0.230241636	-4.343263101	2.672908925	11.60914671	-2.118779
Ttc21b	Ttc21b-004	685.00532	0.006594473	0.019845418	0.230262096	-4.342877174	1.858952979	8.073204462	-2.118651
Sulf1	Sulf1-005	212.73473	0.040328921	0.08265553	0.23030893	-4.341994035	0.461007084	2.001690011	-2.118358
Ift140	Ift140-003	331.88494	0.015383168	0.038632157	0.230321689	-4.341753496	0.64054647	2.781094875	-2.118278
Mff	Mff-016	1380	2.96964E-05	0.000333695	0.230355285	-4.341120291	2.965830718	12.87502791	-2.118067
030032P07Rik	0032P07Rik-	291.19969	0.006253344	0.019054897	0.230400896	-4.340260896	0.622966721	2.703838099	-2.117782
Cdh13	Cdh13-002	4527.88	0.00011627	0.000893766	0.23040648	-4.340155706	9.55805654	41.48345364	-2.117747
Pigt	Pigt-005	521.48421	0.045355568	0.090735411	0.230428005	-4.339750276	1.057104345	4.587568875	-2.117612
Gm16675	Gm16675-201	558	0.002578083	0.009540719	0.230452713	-4.339285005	1.522431619	6.606264696	-2.117457
Cecr2	Cecr2-001	683.10248	0.019379818	0.046342314	0.230468095	-4.338995387	1.971471753	8.554206841	-2.117361
Taf3	Taf3-002	1858.9795	3.16835E-06	7.32226E-05	0.230527155	-4.337883755	4.135831966	17.9407583	-2.116991
Cux1	Cux1-002	2583.238	0.000209918	0.001381836	0.230555318	-4.337353862	5.341089245	23.16619407	-2.116815
Mtch2	Mtch2-001	1281.0371	0.010374599	0.02841115	0.230572927	-4.337022622	3.407316903	14.77761049	-2.116705
Fgfr1op	Fgfr1op-003	495.41289	0.016942793	0.041669831	0.230620516	-4.33612766	1.239435783	5.37435178	-2.116407
Mkln1	Mkln1-014	3641.3951	4.21851E-07	2.10311E-05	0.230692895	-4.334767224	7.937002104	34.40505658	-2.115955
Vps53	Vps53-002	427.66244	0.016181878	0.040152815	0.230700563	-4.334623139	0.792006095	3.433047944	-2.115907
Zfp280d	Zfp280d-001	382.34204	0.007872065	0.022845131	0.230789691	-4.332949175	0.87634606	3.797162938	-2.115349
Rsl24d1	Rsl24d1-001	2962.8484	4.54835E-05	0.000450234	0.230795064	-4.332848289	7.682739439	33.28814443	-2.115316
Snap23	Snap23-001	14418.478	2.59147E-07	1.60262E-05	0.230863275	-4.331568118	32.02715071	138.7277849	-2.114889
Max	Max-201	5086.9814	0.000411743	0.002298649	0.230889374	-4.331078485	12.12798437	52.52725216	-2.114726
Mast1	Mast1-003	548.44908	0.00021	0.001382107	0.230904437	-4.330795951	1.092633405	4.731972325	-2.114632
Cse1l	Cse1l-002	504.38494	0.027390435	0.060751354	0.23094772	-4.329984288	1.20273876	5.207839936	-2.114362
P24-245O2	24-245O20.1-	359	0.000687302	0.003415071	0.230963027	-4.329697319	0.79953595	3.461748659	-2.114266
Xiap	Xiap-002	1028.9656	0.017844152	0.043394079	0.231056957	-4.327937201	2.434023278	10.53429989	-2.11368
Pid1	Pid1-001	805.03115	0.008386386	0.023974102	0.231112903	-4.326889533	2.282513026	9.876181719	-2.11333
Nufip2	Nufip2-001	16089.272	6.31259E-08	7.32903E-06	0.23127601	-4.323837997	37.87225884	163.7535118	-2.112312
Mmp12	Mmp12-005	4899.8876	3.32495E-06	7.53092E-05	0.231306701	-4.323264284	11.59053074	50.10892757	-2.112121

P23-446J15	23-446J15.1-(	1961	2.81939E-06	6.78119E-05	0.231360302	-4.322262688	4.006196214	17.31583242	-2.111787
Zfp420	Zfp420-003	977.28501	0.000394194	0.002226807	0.231360401	-4.322260844	2.100175863	9.077507898	-2.111786
Gm16938	Gm16938-201	585.44034	0.006251974	0.019054111	0.231421317	-4.321123108	1.523137014	6.58166255	-2.111406
Stk38	Stk38-004	1656.7405	0.000296845	0.001794696	0.231423394	-4.321084332	3.352630327	14.48699837	-2.111393
Rmnd1	Rmnd1-001	1327.0502	9.47656E-05	0.000768326	0.23143819	-4.320808075	2.734660145	11.81594163	-2.111301
Mrpl33	Mrpl33-001	2021.9356	0.000178254	0.001223975	0.231494141	-4.319763761	5.837308794	25.21579499	-2.110952
Trpm7	Trpm7-007	444.1872	0.001006349	0.004577958	0.231509604	-4.319475225	1.169939542	5.053524865	-2.110856
Anapc10	Anapc10-201	2213	6.49329E-06	0.000115754	0.231547187	-4.318774131	4.90942079	21.2026795	-2.110622
Pknox2	Pknox2-004	511.0559	0.013874677	0.035638795	0.231562119	-4.31849564	0.927056789	4.003490702	-2.110529
Ddx26b	Ddx26b-005	2815.2998	0.000280683	0.001717595	0.231574895	-4.318257377	6.536521336	28.22638148	-2.110449
Nfatc3	Nfatc3-201	13380.142	2.32487E-08	3.84182E-06	0.231613067	-4.317545696	29.39036147	126.8942287	-2.110211
S100pbp	S100pbp-011	584.82789	0.003823246	0.012958553	0.231614262	-4.317523419	1.297651173	5.60263933	-2.110204
P24-500I24	24-500I24.6-(	268	0.0048396	0.015590323	0.23164779	-4.316898508	0.585596561	2.527960921	-2.109995
Skint8	Skint8-002	1461	2.87781E-05	0.000325816	0.231739725	-4.315185921	2.966464779	12.80084705	-2.109423
30307A14F	0307A14Rik-	421.49488	0.002406788	0.009046588	0.231752276	-4.314952221	0.982729215	4.24042961	-2.109345
Cdc73	Cdc73-004	919.8924	0.0001816	0.001240486	0.231796934	-4.314120908	1.811891991	7.81672112	-2.109067
Peli1	Peli1-004	2061.2109	3.19875E-05	0.000350583	0.231821502	-4.313663712	3.718999973	16.04251523	-2.108914
Stam2	Stam2-001	3954.5294	1.86246E-06	5.12362E-05	0.231970278	-4.310897098	8.430037841	36.34102566	-2.107988
Asb8	Asb8-001	2575.5769	3.05872E-05	0.000339583	0.231993892	-4.310458314	5.594203079	24.11357917	-2.107841
Lrrc8c	Lrrc8c-001	63214.617	3.62431E-08	5.0874E-06	0.232007117	-4.310212608	159.8908039	689.1633588	-2.107759
Zfp120	Zfp120-003	974.32284	0.029132952	0.06376791	0.232096912	-4.308545039	2.086031215	8.987759442	-2.107201
Gm9978	Gm9978-201	1093	1.51267E-05	0.00020674	0.232120345	-4.308110088	2.802374415	12.07293749	-2.107055
30111J21Ri	0111J21Rik2-	1141.7882	0.00071536	0.00352128	0.232148534	-4.307586973	2.032047152	8.753219842	-2.10688
Bgn	Bgn-006	801.57322	0.010933955	0.029559394	0.232198962	-4.306651457	1.550725217	6.678433014	-2.106567
Trim6	Trim6-001	920.15799	0.006441757	0.019489393	0.232231008	-4.306057179	2.271332027	9.780485581	-2.106367
Chd9	Chd9-201	11802.294	0.000235021	0.001504576	0.232254519	-4.305621277	24.38196771	104.979519	-2.106221
Hmgn1	Hmgn1-001	12103.281	7.05578E-07	2.82937E-05	0.232346835	-4.303910582	31.28144759	134.6325533	-2.105648
030423F21R	0423F21Rik-	350	0.000458435	0.00250022	0.232370292	-4.303476105	0.737681915	3.174596496	-2.105502
Dcun1d3	Dcun1d3-001	4268.1495	0.000178805	0.001226242	0.232382586	-4.303248441	10.20914029	43.93246705	-2.105426
Capns1	Capns1-002	23883.026	2.16243E-07	1.48423E-05	0.232416248	-4.302625183	41.83474783	179.9992395	-2.105217
Vps8	Vps8-011	1275.0454	0.024874228	0.05624124	0.232492111	-4.301221218	2.976207865	12.80132842	-2.104746
Gm12537	Gm12537-001	1216	0.000290487	0.001764977	0.232519168	-4.300720708	3.293403042	14.16400666	-2.104578
Gm13363	Gm13363-001	212	0.004206253	0.013970537	0.232537277	-4.300385777	0.564771188	2.428733984	-2.104466
Helq	Helq-001	404.44908	0.019383553	0.046347857	0.232625469	-4.298755435	0.688748746	2.960762416	-2.103919
RP23-7M16.	P23-7M16.1-0	1050.3103	4.68533E-05	0.000459806	0.232696752	-4.297438584	2.477303031	10.64605763	-2.103477
Bclaf1	Bclaf1-201	3135.989	9.23647E-05	0.000752854	0.232849357	-4.294622119	7.089495982	30.44670625	-2.102531
Gm26623	Gm26623-201	932	0.012655414	0.033135687	0.232900567	-4.293677832	2.5067816	10.76331259	-2.102214
P23-189N6	23-189N6.3-(	921	0.000481922	0.002598114	0.232966693	-4.292459099	1.960938878	8.41724993	-2.101804
Rprd2	Rprd2-007	684.59914	0.00055346	0.002894933	0.233035914	-4.291184059	1.677479375	7.198372751	-2.101376
Epha4	Epha4-201	4773.9718	0.000491306	0.002637497	0.233068369	-4.290586503	9.076331394	38.94278497	-2.101175
230216N24F	0216N24Rik-	1212	0.000650533	0.003274657	0.233092562	-4.29014119	2.545448796	10.92033473	-2.101025
Rnf170	Rnf170-001	3059.1246	1.09717E-05	0.000164816	0.233158269	-4.288932164	7.120326275	30.53859638	-2.100618
Gk5	Gk5-001	3035.2447	0.003641363	0.012479154	0.233161322	-4.288876014	6.682280117	28.65947091	-2.1006
Ap4e1	Ap4e1-001	2374.4677	1.77276E-05	0.000231656	0.233234912	-4.287522794	5.137796604	22.02842005	-2.100144
Srp9	Srp9-001	5395.2725	1.55251E-06	4.57604E-05	0.233255021	-4.287153165	12.85068311	55.09284679	-2.10002
Gm12013	Gm12013-001	143	0.014884273	0.037678223	0.233267857	-4.286917253	0.381151266	1.633963937	-2.099941
32419C18R	2419C18Rik-	382	0.000915393	0.004256357	0.233311744	-4.286110858	0.887610312	3.804396194	-2.099669
Shroom3	Shroom3-010	2053.4842	0.00025899	0.001617209	0.233324678	-4.285873261	4.924200728	21.10450023	-2.099589
Taf1	Taf1-002	2397.8533	0.039254753	0.080875559	0.233351457	-4.285381423	4.885924384	20.93804959	-2.099424
Map4k2	Map4k2-001	6940.4565	0.000207693	0.001370542	0.23335837	-4.285254481	13.62590264	58.39046035	-2.099381
Gm37653	Gm37653-001	1762	0.000546658	0.002866358	0.23340982	-4.284309884	3.704772068	15.87239159	-2.099063
Dhrs1	Dhrs1-002	772.98662	0.017485551	0.04270699	0.233439862	-4.283758538	1.595039937	6.83276595	-2.098877
Gm26604	Gm26604-201	154.68437	0.025241428	0.056921181	0.233465086	-4.283295706	0.319634096	1.369087351	-2.098721
Sfrp1	Sfrp1-001	194745	0.00017291	0.001195917	0.233621566	-4.280426752	425.4359197	1821.047292	-2.097755
Mast4	Mast4-004	670.69567	0.00211115	0.008162892	0.233677273	-4.27940632	1.597928401	6.838184899	-2.097411
700110I01R	0110I01Rik-(	1967.9743	1.16448E-05	0.000171615	0.233684723	-4.279269897	4.476669301	19.15687618	-2.097365
Lims1	Lims1-003	1668.9378	0.014419674	0.036746565	0.233724283	-4.27854559	4.290976139	18.35913703	-2.09712
Asph	Asph-004	1023.9331	0.000409755	0.00229231	0.233728288	-4.27847227	2.343565422	10.02687967	-2.097096
Fxyd5	Fxyd5-010	2689.9935	0.000507841	0.002703803	0.233748159	-4.278108557	4.101850293	17.54816084	-2.096973
Slbp	Slbp-004	756.23664	5.71682E-05	0.000531547	0.233775632	-4.277605806	1.538727979	6.582071738	-2.096804
630035G10F	0035G10Rik-	249	0.006498309	0.019611209	0.233867793	-4.275920119	0.496982533	2.125057614	-2.096235
Mettl21a	Mettl21a-001	938.03156	0.001282033	0.005539252	0.233886964	-4.275569634	1.927622566	8.241684509	-2.096117
Zfand5	Zfand5-001	6311.5253	1.18916E-06	3.90495E-05	0.233897207	-4.275382397	15.66813496	66.98726838	-2.096053
Tpd52l2	Tpd52l2-008	498.51385	0.041443152	0.084499445	0.233908277	-4.27518005	1.098208949	4.695040991	-2.095985
10001H17R	0001H17Rik-	407.6394	0.016867225	0.041528464	0.233935841	-4.274676312	1.036587229	4.431074875	-2.095815
Slc9a3r2	Slc9a3r2-202	45308.733	0.000174847	0.001206388	0.233957187	-4.274286312	28.4861857	121.7581136	-2.095684
Itsn1	Itsn1-002	1217.0065	8.14323E-05	0.00069019	0.233977102	-4.273922492	2.85519694	12.20289042	-2.095561
Son	Son-004	21025.738	6.16384E-07	2.5999E-05	0.233988788	-4.27370905	46.96710985	200.7237624	-2.095489
42436	March7-005	1777.178	9.9384E-06	0.000153814	0.234050096	-4.27258957	3.851885212	16.45752458	-2.095111
Odc1	Odc1-201	10699	0.000536549	0.002824157	0.234063597	-4.272343123	34.26167292	146.3776227	-2.095028
Atr	Atr-003	258.04246	0.006872539	0.020513462	0.234101173	-4.271657359	0.538613551	2.30077254	-2.094796
Pam	Pam-010	498.91395	0.039655243	0.081504565	0.234106902	-4.271552836	1.042278585	4.452148047	-2.094761
Ube3a	Ube3a-001	5762.6085	3.10407E-07	1.76309E-05	0.234114331	-4.271417281	13.15361394	56.18457388	-2.094715
10030O07F	0030O07Rik-	5996	1.46762E-06	4.42482E-05	0.234167038	-4.270455854	13.91297203	59.41473287	-2.09439
Ddx5	Ddx5-001	71637.89	3.4685E-08	4.91224E-06	0.234196045	-4.269926937	158.1921382	675.4688719	-2.094211
Gm14340	Gm14340-001	7611	9.60906E-08	9.2969E-06	0.234213623	-4.269606461	16.77994826	71.64377552	-2.094103
Zhx1	Zhx1-006	3646.3472	0.016890845	0.041571676	0.234283955	-4.268324741	6.42030034	27.40392678	-2.09367
Kazn	Kazn-003	568.78958	0.003372997	0.011765658	0.234297752	-4.268073378	1.222664175	5.218420414	-2.093585
Phf11d	Phf11d-001	1292.4973	0.000309647	0.001851502	0.234302282	-4.267990863	2.392247768	10.21009162	-2.093557
P23-324D2	23-324D2.1-(	230	0.017180568	0.042109236	0.234309215	-4.267864581	0.483373913	2.062974404	-2.093514
30433N12F	0433N12Rik-	276.77122	0.005241063	0.016616349	0.234385643	-4.266472925	0.593607426	2.532610011	-2.093044
Naa15	Naa15-004	655.76456	0.001543322	0.006388542	0.234426195	-4.265734895	1.773864317	7.566834917	-2.092794
Hfe	Hfe-002	1863.8772	6.13929E-05	0.000559677	0.234435349	-4.265568329	3.803043318	16.22214113	-2.092738
Bloc1s4	Bloc1s4-001	1102	0.002414922	0.009074172	0.2344359	-4.265558305	2.835268418	12.09400274	-2.092735
30068E07F	0068E07Rik-	10704.633	7.32325E-07	2.89967E-05	0.234489209	-4.264588563	24.08740178	102.7228581	-2.092407
Galnt7	Galnt7-001	1032.9407	0.00241903	0.009085623	0.23450563	-4.26428994	2.764671848	11.78936235	-2.092306
Ttc25	Ttc25-002	1421	7.22857E-06	0.000124099	0.23451707	-4.264081934	3.110701025	13.26428404	-2.092235
Dpf2	Dpf2-002	522.2703	0.04797	0.094953043	0.234582026	-4.2629012	1.311811033	5.592120826	-2.091836
P23-477N9	23-477N9.4-(	2667	7.8656E-07	3.04403E-05	0.234601972	-4.262538771	6.031168173	25.70808817	-2.091713
Suz12	Suz12-001	6379.4686	5.33448E-05	0.000504852	0.234681015	-4.261103092	15.20746851	64.8005911	-2.091227
Bet1	Bet1-001	1523.0655	0.00075807	0.003674482	0.234741074	-4.260012882	3.067935624	13.06944528	-2.090858
N4bp1	N4bp1-201	15690	2.13261E-06	5.67535E-05	0.234784489	-4.259225157	33.71450839	143.5976823	-2.090591
Wdr11	Wdr11-003	1121.9859	0.00026933	0.001664351	0.234788035	-4.259160829	2.615683032	11.14061471	-2.090569
Mib1	Mib1-001	26482.58	7.19356E-08	7.87397E-06	0.234801504	-4.258916508	60.6125036	258.1435922	-2.090486
Dleu2	Dleu2-002	916.28445	0.000238093	0.001518283	0.234817163	-4.258632493	2.249800341	9.581072835	-2.09039
931406P16R	1406P16Rik-	4702.6039	0.000348938	0.002027332	0.234905459	-4.257031764	9.753248451	41.51988846	-2.089848
Lin7c	Lin7c-001	15452.723	5.67252E-09	1.94284E-06	0.234930034	-4.256586456	33.9326102	144.437089	-2.089697
Hnrnpab	Hnrnpab-001	9148.8158	0.000478211	0.002583195	0.234930194	-4.256583562	26.70376463	113.6668056	-2.089696
Gm20342	Gm20342-001	7638	1.73751E-05	0.000228093	0.234972495	-4.255817254	16.17790911	68.8502247	-2.089436
Ptpn12	Ptpn12-004	4864.843	7.70095E-05	0.000661378	0.235024345	-4.254878365	9.886928719	42.0676791	-2.089118
Ints3	Ints3-003	2975.1412	0.038712095	0.080036778	0.235047831	-4.254453222	6.60449221	28.09850316	-2.088974
Rcbtb2	Rcbtb2-003	633.70418	0.008858441	0.025032109	0.235054027	-4.254341059	1.758165632	7.479836238	-2.088936
Akap17b	Akap17b-002	587.48195	0.034437969	0.072993405	0.235063541	-4.254168884	1.288057343	5.479613471	-2.088877
Dbhos	Dbhos-001	1702	0.000101108	0.000803285	0.235090025	-4.253689624	3.997405249	17.00372123	-2.088715
Ptbp1	Ptbp1-018	870.74021	0.000288142	0.001754155	0.235127289	-4.253015488	2.270309736	9.65566247	-2.088486
Phkb	Phkb-002	677.88774	0.013446734	0.034777035	0.235202777	-4.251650489	0.675480413	2.871906628	-2.088023
Mcm4	Mcm4-201	4223	0.001490698	0.006226392	0.235223161	-4.251282046	12.68893464	53.94424002	-2.087898

Gm38394	Gm38394-001	20397.121	1.91981E-07	1.39309E-05	0.235224526	-4.251257373	42.31326653	179.8845863	-2.08789
Gm38394	Gm38394-201	20397.121	1.91981E-07	1.39309E-05	0.235224526	-4.251257373	42.31326653	179.8845863	-2.08789
Il17f	Il17f-004	80.083915	0.043221319	0.087376354	0.235279937	-4.25025615	0.162198809	0.689386484	-2.08755
Galk2	Galk2-008	182.31623	0.023575337	0.053942402	0.235350656	-4.248979027	0.505540015	2.148028921	-2.087116
Phf8	Phf8-001	5342.3323	1.2398E-05	0.000179624	0.235361344	-4.248786071	12.03308222	51.12599212	-2.087051
Ppih	Ppih-003	338.00517	0.007143509	0.021167722	0.235383184	-4.248391853	1.040133853	4.418896187	-2.086917
Snrnp48	Snrnp48-201	2129.7662	0.000382236	0.002175767	0.235383956	-4.248377905	4.937296311	20.97550056	-2.086912
Xaf1	Xaf1-004	2090.937	6.88717E-06	0.000120227	0.235438921	-4.247386089	4.258463528	18.08733875	-2.086575
Crebrf	Crebrf-001	13632.609	0.004233108	0.014042028	0.235465304	-4.246910188	31.65946647	134.4549107	-2.086414
Cpeb4	Cpeb4-001	5062.1014	0.000189262	0.001278042	0.235465643	-4.246904073	10.60812067	45.05167087	-2.086412
Gm13292	Gm13292-001	966	0.001503859	0.006270654	0.235479229	-4.246659046	2.670830919	11.34210829	-2.086328
Gm10358	Gm10358-201	735	0.001246926	0.005421761	0.235522847	-4.245872592	1.948691399	8.2738954	-2.086061
Phykpl	Phykpl-008	388.03974	0.049780072	0.097621957	0.23553405	-4.245670644	1.112930372	4.725135808	-2.085992
Uvssa	Uvssa-004	2885.3851	0.001064121	0.004775437	0.235553101	-4.245327254	6.369020991	27.0385784	-2.085876
Mospd2	Mospd2-002	2994.3235	0.000207635	0.001370494	0.235605425	-4.244384434	7.144818443	30.32535618	-2.085555
Slc12a5	Slc12a5-002	305.85013	0.011900419	0.031607591	0.235621372	-4.244097187	0.540897281	2.295620627	-2.085458
Ifnar1	Ifnar1-002	1708.5769	7.44026E-05	0.000646282	0.235624687	-4.244037472	4.586286119	19.46437014	-2.085437
Lrrc59	Lrrc59-001	16526.358	0.000220526	0.001435662	0.235641847	-4.243728413	46.18593255	196.0005542	-2.085332
Rnf144a	Rnf144a-202	2538.2652	0.001623864	0.006645637	0.235663953	-4.243330328	5.272919009	22.37473714	-2.085197
Cubn	Cubn-001	385.52243	0.010057415	0.027717403	0.235705844	-4.242576181	1.016646303	4.313199389	-2.084941
Gm4724	Gm4724-003	339.09877	0.012117267	0.032054921	0.235730159	-4.242138565	0.664685202	2.819686727	-2.084792
Rbm26	Rbm26-002	3842.3003	0.000317801	0.001888061	0.23579793	-4.240919331	9.716239837	41.20578935	-2.084377
Exosc3	Exosc3-001	1292.6819	0.000515191	0.002734434	0.235808569	-4.240727992	3.781630491	16.03686628	-2.084312
Hnrnpk	Hnrnpk-007	2190.641	5.29847E-05	0.000502416	0.235812042	-4.240665536	4.832564144	20.49328822	-2.084291
Mto1	Mto1-002	530.61166	0.00217431	0.008355226	0.235862929	-4.239750622	1.197781061	5.078293	-2.083979
Erh	Erh-001	1918.033	0.004660208	0.01513198	0.235913478	-4.238842176	5.470567902	23.18887395	-2.08367
Gm38156	Gm38156-001	963	0.001245822	0.005419026	0.235918585	-4.23875042	1.568112767	6.646838651	-2.083639
Mzt1	Mzt1-201	2747	1.01145E-05	0.000155695	0.235918882	-4.238745087	6.680316616	28.31615924	-2.083637
Foxn2	Foxn2-001	6006.7188	1.65243E-06	4.74002E-05	0.235926266	-4.238612419	13.90893353	58.95457839	-2.083592
Gin1	Gin1-004	453.97279	0.048425213	0.095660604	0.235941827	-4.238332858	0.913315855	3.8709366	-2.083497
Peg3	Peg3-003	3259.0513	0.008591576	0.024435912	0.235944608	-4.238282912	7.72463506	32.73918878	-2.08348
Pgk1-rs7	Pgk1-rs7-201	2538	0.005738328	0.017812012	0.235947273	-4.238235036	7.585462208	32.14897169	-2.083464
Alas2	Alas2-003	89.163608	0.028889417	0.063329654	0.235994618	-4.237384778	0.209283838	0.88681615	-2.083174
Ipmk	Ipmk-001	3159.2577	8.19292E-06	0.000135167	0.235999179	-4.237302884	7.8535534	33.27788447	-2.083146
Mrpl36	Mrpl36-201	856	0.002562473	0.009501428	0.236011336	-4.237084604	2.412219271	10.22077713	-2.083072
Mir22hg	Mir22hg-002	1185.9103	0.012219851	0.032254152	0.236014154	-4.237034023	2.683866752	11.37163474	-2.083055
Rab36	Rab36-001	201.05288	0.029213962	0.06391784	0.236022452	-4.236885059	0.425775852	1.803963348	-2.083004
Braf	Braf-003	792.6218	0.000188013	0.001273374	0.236064229	-4.236135246	1.759075814	7.451683058	-2.082749
Abhd5	Abhd5-005	643.45007	0.000844705	0.00399381	0.236087148	-4.235724013	1.299444413	5.504087901	-2.082609
P24-246N2	24-246N21.1-	6210	1.65344E-05	0.000220009	0.236106713	-4.235373004	17.21126443	72.89612472	-2.082489
Gm1818	Gm1818-201	977	2.56876E-05	0.000301198	0.236115606	-4.235213488	2.362894525	10.00736276	-2.082435
Ap1g2	Ap1g2-003	844.92677	0.000876438	0.004117132	0.236151813	-4.234564139	1.625275702	6.882334205	-2.082213
Raph1	Raph1-007	10077	9.96916E-05	0.000795906	0.236202214	-4.233660577	28.31228753	119.8646156	-2.081906
Zc3hav1	Zc3hav1-004	13583.554	4.0164E-08	5.43723E-06	0.236213975	-4.233449777	29.26596956	123.8960123	-2.081834
Gm12543	Gm12543-001	1193.2547	0.002020345	0.007891975	0.23624646	-4.232867659	2.774737228	11.74509548	-2.081635
Zcchc11	Zcchc11-003	412.42583	0.020019578	0.047536198	0.236284909	-4.232178866	0.861619974	3.646529844	-2.081401
Ankrd13c	Ankrd13c-003	1058.1441	0.001629614	0.006663595	0.236315189	-4.231636591	2.326234349	9.843778389	-2.081216
Chst2	Chst2-001	1311	0.000841758	0.003982724	0.236318884	-4.231570421	3.769853829	15.95240195	-2.081193
H2-Bl	H2-Bl-006	302.17024	0.007892444	0.022894572	0.236322572	-4.231504387	0.712622336	3.01546454	-2.081171
22401L13R	2401L13Rik-	739.73299	0.003355525	0.011722039	0.236344787	-4.231106654	1.946651665	8.236490814	-2.081035
Casp2	Casp2-001	3359.4448	8.35159E-07	3.15371E-05	0.236346274	-4.231080034	7.902582928	33.43646084	-2.081026
Pomgnt1	Pomgnt1-008	549.42628	0.005846987	0.018073945	0.236415859	-4.229834676	1.329145353	5.622065103	-2.080601
B3gat2	B3gat2-001	1020.3502	0.001345182	0.005744413	0.236440534	-4.229393257	2.442357645	10.32969096	-2.080451
Pigq	Pigq-003	4249.2634	0.000106742	0.000835636	0.23645914	-4.229060454	9.075269752	38.37986442	-2.080337
Mpp7	Mpp7-201	2777	1.36242E-05	0.000191949	0.236478623	-4.228712037	6.309685583	26.68184337	-2.080218
Hibch	Hibch-007	1284.0078	6.64831E-05	0.000594219	0.236518963	-4.227990809	2.241714391	9.477947842	-2.079972
Stau1	Stau1-006	1205.5956	0.000879428	0.00412665	0.236620878	-4.226169759	3.441056112	14.54248728	-2.079351
AC107830.1	C107830.1-2(	825.35184	0.011738512	0.031283466	0.236630728	-4.225993846	1.526579949	6.45131747	-2.079291
Dtnb	Dtnb-006	1245.0713	0.000312443	0.00186172	0.23670349	-4.224694785	2.596550109	10.96963171	-2.078847
32438A13R	2438A13Rik-	8324.8096	0.00017556	0.001209603	0.23672778	-4.224261297	19.11673893	80.75410038	-2.078699
Ero1l	Ero1l-201	8060	1.21997E-06	3.97565E-05	0.236740988	-4.224025621	18.61768236	78.64156729	-2.078619
Cstf3	Cstf3-003	1569.2471	2.73788E-05	0.000315193	0.236742849	-4.223992419	3.961784293	16.73454682	-2.078607
P23-370G17	23-370G17.1-	956.95872	0.000300341	0.001812312	0.236808174	-4.222827212	2.469807259	10.4295693	-2.078209
Zfp292	Zfp292-002	11357.369	4.73292E-05	0.000462622	0.236831483	-4.222411604	24.89204314	105.1044518	-2.078067
Dab2	Dab2-001	8676.3994	1.15599E-05	0.000170994	0.236871307	-4.221701694	17.81356638	75.20356337	-2.077825
Zfp322a	Zfp322a-003	3963.9161	6.34814E-08	7.3454E-06	0.23691313	-4.22095644	9.397155345	39.66498337	-2.07757
Pik3cb	Pik3cb-002	2897.1227	6.33802E-05	0.000574112	0.236927865	-4.220693923	7.282251403	30.73615424	-2.07748
Sptbn1	Sptbn1-201	278963.52	8.99065E-05	0.000738441	0.236928013	-4.220691284	451.809979	1906.95044	-2.077479
Ptpn4	Ptpn4-001	4318.8829	0.000139651	0.001027508	0.236969228	-4.219957201	11.03823385	46.58087442	-2.077228
Coq10b	Coq10b-003	547.90516	0.013403532	0.034688754	0.236999611	-4.219416211	1.359250376	5.735243072	-2.077043
Mfsd4	Mfsd4-010	3953.8671	1.12904E-06	3.78374E-05	0.237025657	-4.218952547	7.949599057	33.53898119	-2.076885
Rbm4b	Rbm4b-003	3759.4886	0.000125141	0.000946607	0.237025888	-4.218948445	8.870998905	37.42628703	-2.076883
Celf1	Celf1-005	2127.5077	0.005188319	0.016472	0.237107763	-4.217491611	5.528054519	23.31452356	-2.076385
Rbm12b1	Rbm12b1-002	2682.151	0.007536001	0.022086063	0.237120209	-4.21727023	6.466762423	27.27208465	-2.076309
Rpf2	Rpf2-006	355.49869	0.011235563	0.030198386	0.237121357	-4.217249818	1.195161227	5.040293465	-2.076302
Zc3h11a	Zc3h11a-002	2925.7472	6.16047E-07	2.5999E-05	0.237154644	-4.216657879	6.515182113	27.47229399	-2.0761
Ctsd	Ctsd-003	9889.0733	0.001120683	0.00498162	0.237259745	-4.214789993	15.74153626	66.34726952	-2.075461
Gufl	Gufl-007	1909.3191	0.000128624	0.000964395	0.23727529	-4.214513861	4.034203379	17.00220606	-2.075366
Gm14403	Gm14403-002	743.18312	0.001206916	0.005278681	0.23734277	-4.213315612	1.519897206	6.403806625	-2.074956
Slc11a2	Slc11a2-001	798.28508	0.01008323	0.027772466	0.237357018	-4.213062702	1.840343834	7.753483964	-2.074869
42624	Sept11-001	20729.674	4.61669E-07	2.22081E-05	0.237413008	-4.212069125	35.36304833	148.951604	-2.074529
Pa2g4	Pa2g4-001	12852.581	1.40271E-06	4.31263E-05	0.237470482	-4.211049699	35.23447715	148.3741344	-2.07418
Phactr2	Phactr2-001	421.85561	0.007035051	0.020910397	0.237479149	-4.21089601	0.962008609	4.050918215	-2.074127
Klf7	Klf7-003	3628.0797	8.76385E-05	0.000723735	0.237499054	-4.210543087	7.03551248	29.62332844	-2.074006
Gm12666	Gm12666-001	212	0.008870618	0.025057211	0.237504566	-4.210445362	0.514166014	2.16486791	-2.073973
Uqcc1	Uqcc1-005	966.1251	0.001761573	0.007087263	0.237540959	-4.209800299	2.200938819	9.2655129	-2.073752
Rab34	Rab34-005	824.87299	8.21666E-05	0.000693325	0.237551513	-4.209613261	2.119298142	8.921425562	-2.073688
Gm17690	Gm17690-001	1690.4105	0.000592307	0.003044652	0.237601229	-4.208732444	3.69769694	15.56261708	-2.073386
Ranbp6	Ranbp6-001	3308	1.3896E-06	4.28773E-05	0.237624775	-4.208315399	7.093948971	29.8535747	-2.073243
Zfp266	Zfp266-001	12895.781	2.1387E-07	1.4798E-05	0.237634893	-4.20813622	27.08513418	113.9779342	-2.073181
Il6ra	Il6ra-001	1885.6532	0.001318159	0.005655385	0.237661813	-4.207659559	4.686301631	19.71836185	-2.073018
Slc35g1	Slc35g1-201	397	0.049648093	0.097413335	0.237676734	-4.207395413	1.161717705	4.887805744	-2.072927
Sorbs2	Sorbs2-008	6498.8176	0.000519623	0.002752409	0.237743878	-4.20620716	14.94290476	62.85295297	-2.07252
Ubxn2b	Ubxn2b-001	3079	1.9099E-05	0.000244174	0.237787031	-4.205443826	7.731116027	32.51277416	-2.072258
Ksr2	Ksr2-001	441.9688	0.000653427	0.003285841	0.237833271	-4.204626189	1.015297138	4.268944936	-2.071978
Elk4	Elk4-002	14437.962	2.56813E-07	1.60262E-05	0.23787138	-4.203952565	29.9374372	125.8555659	-2.071746
Atxn2	Atxn2-019	2318.4566	2.96352E-06	6.99522E-05	0.23789058	-4.203613266	4.8526366	20.39860759	-2.07163
Nlk	Nlk-001	7411.2513	1.64366E-07	1.26693E-05	0.237962717	-4.202338977	15.23101401	64.00588382	-2.071193
Kif2a	Kif2a-002	2386.1256	0.000107577	0.000840452	0.238021962	-4.201292981	5.765650982	24.223189	-2.070833
Pam	Pam-003	1013.5431	0.009573232	0.02662254	0.238062496	-4.200577651	2.355131853	9.892914225	-2.070588
Dok6	Dok6-002	359	0.001372871	0.005836882	0.238065139	-4.200531008	0.856345577	3.597106148	-2.070572
Dapp1	Dapp1-001	1244.9527	0.008368335	0.023930491	0.238133309	-4.199328541	3.511996465	14.74802699	-2.070159
Supt4a	Supt4a-001	1934.0069	0.00626996	0.019090929	0.238179511	-4.198513944	5.802455815	24.36169165	-2.069879
Vps9d1	Vps9d1-002	713.18652	0.006151895	0.01882814	0.238191326	-4.198305686	1.63370392	6.858788455	-2.069807
Dnajc5	Dnajc5-003	1725.1277	0.005218456	0.016559211	0.238243313	-4.197389577	3.661709374	15.36962076	-2.069492
Pot1b	Pot1b-201	11041	2.46269E-07	1.57364E-05	0.238293024	-4.196513956	25.59729478	107.4194048	-2.069191
Ablim1	Ablim1-007	1786.628	0.020688298	0.048766292	0.238310428	-4.196207471	3.542056711	14.86320483	-2.069086

Anp32-ps	Anp32-ps-001	216	0.048670696	0.096040292	0.238324942	-4.195951929	0.542273466	2.275353398	-2.068998
Bzw1	Bzw1-001	15835.946	5.55501E-06	0.000104538	0.238336961	-4.19574033	36.59541181	153.5448452	-2.068925
Tnfaip8	Tnfaip8-010	1434.3308	0.000304526	0.001829185	0.238360327	-4.195329026	3.558918637	14.93083466	-2.068784
Sfmbt1	Sfmbt1-001	2512.1974	8.23533E-05	0.000694045	0.238398413	-4.194658791	6.380003163	26.76193635	-2.068553
Ccdc47	Ccdc47-005	690.80754	0.010555219	0.028769397	0.238399035	-4.194647847	1.492264558	6.259524315	-2.06855
Ncor1	Ncor1-022	4153.4171	7.745E-05	0.000664358	0.238404429	-4.194552952	8.931989475	37.46570282	-2.068517
Trdmt1	Trdmt1-001	1270.7894	7.8511E-05	0.000670741	0.238413952	-4.194385408	2.837672455	11.90229194	-2.068459
Osbpl9	Osbpl9-016	881.8753	8.96913E-05	0.000737027	0.238526869	-4.192399801	1.922893572	8.061538627	-2.067776
Slc35a5	Slc35a5-001	2701.5695	3.68016E-05	0.000386288	0.238628664	-4.190611402	5.008688443	20.9894669	-2.067161
Agtpbp1	Agtpbp1-011	793.63642	0.045030803	0.090209102	0.238635603	-4.190489549	1.869683097	7.834887476	-2.067119
Lsm4	Lsm4-001	968.49051	0.019197272	0.046005217	0.238701279	-4.189336586	2.609006528	10.9300065	-2.066722
Fbxl3	Fbxl3-001	8276.0578	2.56054E-06	6.36908E-05	0.238801779	-4.187573495	19.26479009	80.67272437	-2.066115
Pdpk1	Pdpk1-002	1927.1477	7.51316E-05	0.000649826	0.238834782	-4.186994842	4.207644028	17.61738384	-2.065915
Etl4	Etl4-015	1880.7742	0.010997363	0.029697997	0.238870193	-4.186374149	4.696434798	19.66103323	-2.065701
Gm37323	Gm37323-001	129	0.045840708	0.09155101	0.238921347	-4.185477834	0.299011524	1.251506106	-2.065392
Rsl1d1	Rsl1d1-001	7887.1157	3.00487E-05	0.000335767	0.238930479	-4.185317848	23.05242328	96.48171859	-2.065337
Gm13157	Gm13157-001	183.42078	0.024088783	0.054856437	0.238968938	-4.184644283	0.378530432	1.584015208	-2.065105
Soat2	Soat2-001	1124.5155	9.99575E-05	0.000797286	0.238982617	-4.184404759	2.799719166	11.7151582	-2.065022
Lrig2	Lrig2-008	635.95261	0.010031194	0.027658058	0.23908986	-4.182527854	1.636080389	6.842951799	-2.064375
Pde4c	Pde4c-002	455.75217	0.00064251	0.003243326	0.239094042	-4.182454699	0.93768465	3.921823571	-2.06435
Prkab2	Prkab2-001	11021.095	7.89481E-05	0.000673705	0.239119543	-4.182008665	21.92754575	91.70118632	-2.064196
Orai3	Orai3-002	2640.1238	2.80682E-05	0.000320339	0.239145362	-4.181557154	6.190620923	25.88643521	-2.06404
Zfp207	Zfp207-004	4222.2016	3.34275E-06	7.54707E-05	0.239203624	-4.180538673	9.958911557	41.63361491	-2.063689
Zfp207	Zfp207-202	4222.2016	3.34275E-06	7.54707E-05	0.239203624	-4.180538673	9.958911557	41.63361491	-2.063689
Ptcra	Ptcra-001	163	0.00730373	0.021533334	0.239222337	-4.180211658	0.368419176	1.540070136	-2.063576
Lmf1	Lmf1-006	326.77155	0.011037019	0.029781603	0.239232349	-4.180036709	0.670780423	2.803886791	-2.063516
Ccdc186	Ccdc186-001	11334.405	1.70043E-05	0.000224761	0.239306339	-4.1787443	28.55420893	119.3207378	-2.063069
Gm37868	Gm37868-001	1069	1.97859E-05	0.000250338	0.239321261	-4.17848375	2.358121656	9.853373018	-2.06298
30111J21Ri	0111J21Rik1-	1720.5906	0.000486481	0.002618162	0.239328396	-4.178359177	3.054390114	12.76233896	-2.062937
Elk3	Elk3-001	26112.725	1.59749E-07	1.25679E-05	0.239340251	-4.178152208	55.53525653	232.0347547	-2.062865
Prrc2c	Prrc2c-011	163.10887	0.047664546	0.094484935	0.23937405	-4.177562274	0.326296471	1.363123829	-2.062661
Qsox2	Qsox2-002	3088.8687	0.000162028	0.00114247	0.239397878	-4.177146462	7.386062692	30.85266564	-2.062518
Ppp1r18	Ppp1r18-003	1553.7887	0.002329992	0.008825728	0.239405416	-4.177014939	4.567413307	19.07815362	-2.062472
42614	Sept1-007	94.616767	0.038581765	0.079833168	0.239427282	-4.17663347	0.260867262	1.089546936	-2.062341
Abl1	Abl1-011	345.40557	0.006967984	0.020745259	0.239467459	-4.175932738	0.531921431	2.221268116	-2.062098
Paqr8	Paqr8-001	3902.0955	0.000321295	0.001903869	0.239469348	-4.175899791	5.466396839	22.82712542	-2.062087
Asb3	Asb3-001	386.76824	0.039647016	0.081497437	0.239582618	-4.173925507	1.036998525	4.328354593	-2.061405
Mapk9	Mapk9-202	858.33667	0.000485269	0.002613279	0.239596387	-4.173685642	2.286290475	9.542257728	-2.061322
Vps39	Vps39-002	3134.4774	0.010781502	0.029214531	0.239600062	-4.173621629	6.120826628	25.5460144	-2.0613
C1galt1c1	C1galt1c1-001	3809	2.24136E-06	5.86452E-05	0.239624996	-4.173187336	8.50101294	35.47631954	-2.06115
Srrm2	Srrm2-003	14626.527	4.71075E-07	2.25655E-05	0.239660584	-4.172567651	34.07798237	142.1926868	-2.060935
Sema6a	Sema6a-001	9766.1566	0.00076351	0.0036951	0.239741434	-4.171160504	27.81905678	116.0377509	-2.060449
Fbxw2	Fbxw2-008	936.04058	0.00014024	0.001030957	0.239744713	-4.171103453	1.885343039	7.863960858	-2.060429
Gm20939	Gm20939-201	379	0.005413262	0.017053369	0.239749041	-4.171028156	0.979043051	4.083616133	-2.060403
Tfb1m	Tfb1m-201	260.68	0.016792876	0.041384409	0.239759198	-4.170851454	0.627465766	2.617066504	-2.060342
Spag9	Spag9-002	5495.8428	0.000210448	0.00138426	0.239767073	-4.170714472	13.27801938	55.37882756	-2.060295
Srp72	Srp72-003	995.99675	0.000186809	0.001267221	0.239775612	-4.170565935	2.505033863	10.44740889	-2.060243
Exosc8	Exosc8-001	1285.1571	0.001055946	0.004750579	0.239789508	-4.170324245	3.60154747	15.01962073	-2.06016
Las1l	Las1l-002	844.53715	0.004073623	0.013605713	0.239815245	-4.169876685	2.202024066	9.182168812	-2.060005
Mir99ahg	Mir99ahg-010	651.86859	0.00018405	0.001253724	0.239840192	-4.169442966	1.201447525	5.009366931	-2.059855
Asb3	Asb3-002	634.66787	0.002938575	0.010584308	0.239861115	-4.169079255	1.737361213	7.243196589	-2.059729
Srcap	Srcap-001	5572.1984	4.12133E-06	8.60181E-05	0.239894485	-4.168499332	13.79286135	57.49553333	-2.059528
Phip	Phip-004	726.14729	0.0081659	0.023473108	0.23989451	-4.168498893	1.349181078	5.624059832	-2.059528
Gm38010	Gm38010-001	255	0.022186851	0.051492739	0.239933974	-4.167813269	0.458435719	1.910674474	-2.059291
Gm38312	Gm38312-001	6768	2.33675E-06	5.99827E-05	0.239943218	-4.167652694	13.56984891	56.55441736	-2.059235
Ewsr1	Ewsr1-010	531.87782	0.000375535	0.002143322	0.239950521	-4.167525855	1.36208537	5.676525997	-2.059191
Hnrnpr	Hnrnpr-001	12541.64	2.64207E-07	1.61538E-05	0.239962541	-4.167317094	28.77298755	119.9061629	-2.059119
Psmg2	Psmg2-201	944	0.002951473	0.010619598	0.239974757	-4.167104964	2.409345503	10.0399956	-2.059045
Serp1	Serp1-001	9434.3255	5.52505E-05	0.000518021	0.239980315	-4.167008453	26.92620774	112.2017353	-2.059012
Oasl2	Oasl2-001	1136.0838	0.005701543	0.01772353	0.240003947	-4.166598135	3.237868943	13.4908987	-2.05887
Kctd4	Kctd4-201	239	0.048664594	0.096033783	0.240028115	-4.166178613	0.527774634	2.198803394	-2.058725
Pola1	Pola1-002	364.04734	0.001069316	0.004795609	0.240038182	-4.166003898	0.95404035	3.974535818	-2.058664
00021K19R	0021K19Rik-	790.97239	0.019973841	0.047470963	0.2401126	-4.164712719	1.11365154	4.638038732	-2.058217
Uggt1	Uggt1-001	15988.86	1.73562E-06	4.87653E-05	0.240144991	-4.164150981	40.22446068	167.5007274	-2.058022
Hspa5	Hspa5-001	59969.863	5.90371E-05	0.000544139	0.240201092	-4.16317841	169.2395018	704.5742401	-2.057685
Gbp6	Gbp6-002	1650.236	0.013221911	0.034319853	0.240295222	-4.161547577	2.598068386	10.8119852	-2.05712
Ralgps1	Ralgps1-003	224.21617	0.025613905	0.057564058	0.240311422	-4.161267047	0.582232753	2.422825969	-2.057023
Ccdc132	Ccdc132-005	1493.2266	9.09307E-06	0.00014499	0.240444061	-4.158971516	3.0801215	12.81013759	-2.056227
Ankhd1	Ankhd1-008	436.22778	0.008475118	0.024175298	0.240486715	-4.15823386	0.854390133	3.55275398	-2.055971
Glmp	Glmp-010	689.70564	0.035780405	0.075173529	0.240498066	-4.158037588	1.54080912	6.406742235	-2.055903
Ttll4	Ttll4-001	1920.211	0.005916656	0.01824813	0.240501487	-4.157978454	4.379967111	18.21180888	-2.055882
Anxa4	Anxa4-003	3059.9784	1.16519E-05	0.000171646	0.240502195	-4.157966203	7.12186491	29.6124736	-2.055878
Ppp2r5a	Ppp2r5a-006	813.786	0.006090295	0.018684396	0.240518519	-4.157684006	1.719446819	7.148916537	-2.05578
Max	Max-202	4375.0107	4.61621E-05	0.000454881	0.240521099	-4.157639413	11.25047411	46.77541458	-2.055765
Rtn3	Rtn3-201	14689.26	8.45755E-07	3.1832E-05	0.240553347	-4.157082046	27.99482334	116.3767775	-2.055571
Pik3ca	Pik3ca-201	1892.9667	0.000138743	0.001022145	0.240557033	-4.157018347	4.603382234	19.1363444	-2.055549
Tra2b	Tra2b-006	2398.9358	0.007613536	0.02226922	0.240621518	-4.155904295	6.536660972	27.16573741	-2.055162
Emg1	Emg1-001	1654.7295	0.00630058	0.019149424	0.240645918	-4.155482908	4.520689402	18.78564754	-2.055016
Sgce	Sgce-006	354.82389	0.036226064	0.075965389	0.240677823	-4.154932051	0.740490651	3.07668834	-2.054825
Trmt1l	Trmt1l-003	946.44715	0.01093187	0.029556089	0.240686142	-4.154788431	1.935065523	8.039787849	-2.054775
Chst10	Chst10-006	1442.6906	5.67749E-06	0.00010597	0.240693181	-4.154666927	3.440180529	14.29280427	-2.054733
Cxadr	Cxadr-001	917.35644	0.001860659	0.007392133	0.24071791	-4.154240121	2.388334782	9.921716172	-2.054585
Gnas	Gnas-012	429.0902	0.022001751	0.051168147	0.240751193	-4.153665812	1.244001026	5.167164532	-2.054385
Gm8783	Gm8783-001	441	0.006800532	0.020342204	0.240787578	-4.153038163	1.044537272	4.338003155	-2.054167
Gnptab	Gnptab-002	3493.5323	0.000383876	0.002182874	0.240791497	-4.152970572	8.389247041	34.84029608	-2.054144
Mtg2	Mtg2-004	1330.9474	2.30963E-06	5.97469E-05	0.240796195	-4.152889546	2.830711155	11.75563077	-2.054116
Ubp1	Ubp1-202	6064.3498	7.92775E-06	0.000132258	0.24081165	-4.152623022	16.61384472	68.99103408	-2.054023
Il17ra	Il17ra-201	5685	0.008174628	0.023492283	0.240870378	-4.151610538	20.38446366	84.62835414	-2.053671
Acbd5	Acbd5-002	2495.4965	0.010467081	0.028608852	0.240929888	-4.150585088	5.312465678	22.04984082	-2.053315
Auts2	Auts2-001	3661.3549	0.002640862	0.00972994	0.240943996	-4.150342063	9.327429024	38.71202102	-2.05323
Dcaf6	Dcaf6-002	1547.0522	0.004999513	0.016001547	0.240972032	-4.149859191	3.090039815	12.82323013	-2.053062
Pnisr	Pnisr-012	1883.2024	0.001964797	0.007731422	0.240975542	-4.149798739	4.436931293	18.41237189	-2.053041
Cfh	Cfh-002	264056.48	0.009154715	0.025728153	0.241067602	-4.148213988	825.498247	3424.343375	-2.05249
Ccdc9	Ccdc9-003	830.76579	0.000778943	0.003749128	0.241095925	-4.14772667	2.074193232	8.603186587	-2.052321
Mga	Mga-012	509.68023	0.000211072	0.001387565	0.241162078	-4.146588924	1.294882196	5.36934417	-2.051925
Nelfa	Nelfa-002	671.94862	0.003096003	0.011010081	0.241225691	-4.145495433	1.307945346	5.422081458	-2.051545
Sema6d	Sema6d-006	68.807668	0.035005132	0.073925624	0.241235496	-4.14532693	0.152527647	0.632276964	-2.051486
Dynlt1a	Dynlt1a-201	463	0.003275524	0.011506327	0.241320753	-4.143862419	1.355251005	5.615973707	-2.050976
Vps29	Vps29-001	4267.8835	2.86483E-06	6.85678E-05	0.24134374	-4.143467742	8.709999707	36.08960282	-2.050839
Gm27316	Gm27316-201	259	0.003559892	0.012269722	0.24135676	-4.143244212	0.593944291	2.460856245	-2.050761
Spaca6	Spaca6-014	1043.2849	8.29329E-05	0.000696872	0.241367537	-4.143059215	2.031035091	8.414698649	-2.050696
Socs2	Socs2-011	552.24335	0.007201412	0.021302761	0.241382143	-4.142808521	1.349132833	5.589198998	-2.050609
Rps4x	Rps4x-001	67067.539	1.21216E-08	2.8812E-06	0.241407491	-4.142373532	177.3404795	734.6105086	-2.050458
Camk1d	Camk1d-001	4778.5692	0.000177413	0.001219915	0.241410795	-4.142316835	11.86905728	49.16539578	-2.050438
Ccng1	Ccng1-001	7897.4192	2.32572E-07	1.54972E-05	0.241432445	-4.141945376	20.64764708	85.52142635	-2.050309
Ptpn14	Ptpn14-006	4741.7564	0.002481333	0.009268803	0.241434626	-4.141907965	10.31050036	42.70514358	-2.050295
P23-157G2	23-157G2.2-(	1937	2.5146E-05	0.000296779	0.241491591	-4.140930929	3.588040318	14.85782713	-2.049955

Txndc17	Txndc17-001	6328.2928	6.96435E-07	2.81616E-05	0.241504679	-4.14070652	15.11421588	62.58353225	-2.049877
Setd5	Setd5-006	3609.7675	0.000315064	0.001874077	0.241505342	-4.140695153	7.681950543	31.80861538	-2.049873
Rpe	Rpe-003	1300.1767	0.016813366	0.04141785	0.241540503	-4.140092395	3.163455317	13.0969973	-2.049663
Thoc2	Thoc2-008	356.69317	0.005053388	0.016131843	0.241597765	-4.139111134	0.86095708	3.563597035	-2.049321
Kctd18	Kctd18-002	2015.7971	0.000137255	0.001014453	0.241604092	-4.139002748	4.688132896	19.40419494	-2.049283
Atp5j2	Atp5j2-005	425	0.01258194	0.032988941	0.241606988	-4.138953132	1.056138385	4.371307277	-2.049266
Dcst1	Dcst1-004	1224.228	5.38466E-06	0.000102231	0.241668093	-4.137906608	2.757164195	11.40888794	-2.048901
Ccl9	Ccl9-001	3123.2636	0.000936748	0.00433036	0.241678772	-4.137723777	12.14464988	50.25120656	-2.048837
Kmt2e	Kmt2e-003	3259.3413	0.000399092	0.002245589	0.24169823	-4.137390665	7.735769522	32.00590061	-2.048721
'00007P06R	0007P06Rik-	459	0.013490287	0.034860596	0.241748208	-4.13653531	1.079997247	4.467446749	-2.048423
Mrpl3	Mrpl3-001	515.80634	0.010780088	0.029214531	0.241775519	-4.13606805	1.02738189	4.24932141	-2.04826
Gm14150	Gm14150-001	3114	0.005685821	0.017691512	0.241781226	-4.135970424	8.850299474	36.60457687	-2.048226
Fbxl2	Fbxl2-001	1038.8959	0.000360034	0.002075955	0.241800945	-4.135633128	1.977395736	8.177783312	-2.048108
Gm12176	Gm12176-001	106	0.033407203	0.071227384	0.241811163	-4.135458373	0.332232122	1.373932109	-2.048047
Atrip	Atrip-007	1470.9281	8.76947E-06	0.000141677	0.241821816	-4.135276207	3.127618009	12.93356434	-2.047984
Zdhhc9	Zdhhc9-004	331.22964	0.002134142	0.00823044	0.241833627	-4.135074231	0.941899123	3.89482279	-2.047913
Gm28048	Gm28048-001	3215.9778	5.48401E-05	0.000515302	0.241849332	-4.134805716	7.748867547	32.04006183	-2.04782
Kif2a	Kif2a-005	1849.7447	0.000385491	0.00218884	0.241871978	-4.134418574	4.043349868	16.7169008	-2.047684
Gm37932	Gm37932-001	453	0.001877833	0.007448265	0.241878641	-4.134304685	0.992179037	4.101970442	-2.047645
Ktn1	Ktn1-016	242.5816	0.006329206	0.019212446	0.241882756	-4.134234358	0.591311157	2.444618902	-2.04762
Casd1	Casd1-001	5715.8656	7.3168E-06	0.000125113	0.241927845	-4.133463839	12.34778865	51.03913788	-2.047351
Ckap4	Ckap4-201	11284.084	0.006102976	0.018714918	0.241933166	-4.133372929	34.34996784	141.9812272	-2.04732
Zbtb44	Zbtb44-201	12682.984	2.24321E-06	5.86487E-05	0.241940063	-4.133255108	30.93478141	127.8613433	-2.047278
Actr2	Actr2-003	24520.126	1.54579E-05	0.000209909	0.241994783	-4.132320489	61.710438	255.0073073	-2.046952
Fus	Fus-002	12701.055	9.30987E-05	0.000757574	0.242008147	-4.132092298	30.81090931	127.313521	-2.046872
Slc25a25	Slc25a25-004	290.58345	0.03613295	0.075797975	0.242028419	-4.131746206	0.842627603	3.4815234	-2.046752
Ftx	Ftx-002	685.73488	0.003563556	0.012279197	0.24206123	-4.131186144	1.878212861	7.759246948	-2.046556
Zfp953	Zfp953-001	1620.1415	1.94104E-05	0.000246957	0.242066384	-4.131098192	4.009634026	16.56419187	-2.046525
Hnrnpk	Hnrnpk-003	619.3842	0.030264467	0.06580485	0.24211415	-4.130283178	1.433111683	5.919157076	-2.046241
Vkorc1l1	Vkorc1l1-002	1881.5441	0.006596443	0.019849602	0.242121617	-4.130155805	4.75717948	19.64789244	-2.046196
Msn	Msn-001	297130.46	5.38889E-05	0.000509157	0.242159296	-4.129513165	780.072099	3221.318003	-2.045972
Ugt3a1	Ugt3a1-005	455	0.01036081	0.028388196	0.242187813	-4.129026915	1.065173526	4.398130157	-2.045802
Poglut1	Poglut1-002	2755.1028	7.38231E-05	0.000642878	0.242192376	-4.128949129	6.909092504	28.52729147	-2.045775
Acly	Acly-201	3840.6426	0.000102085	0.000810295	0.242192803	-4.12894185	9.821252169	40.5513791	-2.045772
Gm14295	Gm14295-001	246.91605	0.009809622	0.027147669	0.242204539	-4.128741777	0.574228605	2.370841631	-2.045702
Mxd1	Mxd1-201	4533	0.044384404	0.089243034	0.24220701	-4.128699658	15.46395963	63.84604484	-2.045687
Pex13	Pex13-001	2945.8018	2.78316E-07	1.66125E-05	0.242223663	-4.128415814	6.951975566	28.70064586	-2.045588
Psme3	Psme3-001	5956.668	6.8289E-05	0.000607002	0.24227501	-4.127540848	17.33968583	71.57026155	-2.045282
Meg3	Meg3-201	502.10785	0.021150075	0.049625955	0.242294501	-4.127208818	0.651473518	2.688767247	-2.045166
Tmbim1	Tmbim1-003	14859.04	6.60344E-06	0.000116702	0.242295738	-4.127187738	26.10642559	107.7461196	-2.045159
Usp33	Usp33-007	1213.1577	8.06752E-05	0.000685639	0.242353556	-4.12620312	2.849646855	11.75822174	-2.044815
Gm26737	Gm26737-201	500.48609	0.001127709	0.005007653	0.242353831	-4.126198438	1.143756704	4.719367126	-2.044813
Uchl5	Uchl5-002	838.03061	0.002762326	0.010068072	0.242354342	-4.126189738	2.172873397	8.96568791	-2.04481
Phtf1os	Phtf1os-001	474.98989	0.012274517	0.032359585	0.242447703	-4.124600836	0.997032279	4.112360173	-2.044255
Itpr2	Itpr2-005	8605.4029	6.40613E-05	0.000578155	0.242533	-4.123150254	12.44974732	51.33217883	-2.043747
Zscan29	Zscan29-001	4304.848	1.90409E-05	0.000243706	0.242534944	-4.123117198	9.447229947	38.95203627	-2.043735
Dexi	Dexi-003	795.19727	0.023410775	0.053676466	0.242543545	-4.122970997	1.772224254	7.306829201	-2.043684
Coq4	Coq4-003	1217.511	9.94839E-06	0.000153899	0.242544696	-4.122951422	2.74633896	11.32302212	-2.043677
Gm37381	Gm37381-001	665.86882	0.00203457	0.007937033	0.242631234	-4.121480912	1.828963793	7.538039361	-2.043163
Rnf138	Rnf138-201	704.52262	0.029768251	0.064923751	0.242802707	-4.118570223	1.127170615	4.642331331	-2.042144
Fam188a	Fam188a-018	296.35751	0.027535381	0.061026761	0.242805647	-4.118520354	0.811374083	3.341660676	-2.042126
Cd40	Cd40-001	4149.3501	1.84689E-06	5.09307E-05	0.242860896	-4.117583425	8.170969536	33.64464873	-2.041798
Ly6h	Ly6h-202	467	0.001574364	0.006495057	0.242870381	-4.117422625	1.054113785	4.340231948	-2.041742
Ppp3cb	Ppp3cb-003	2121.2425	0.000245715	0.001555302	0.242895376	-4.116998918	4.190273063	17.25134966	-2.041593
Slmap	Slmap-012	282.66606	0.047142639	0.093634621	0.242895449	-4.116997685	0.772426378	3.180077609	-2.041593
Dpysl3	Dpysl3-001	13606.683	0.003716471	0.01268578	0.24289829	-4.116949531	48.45557161	199.4891428	-2.041576
Ube2r2	Ube2r2-001	7401.2188	4.57831E-08	5.93966E-06	0.242903614	-4.116859294	17.37644939	71.53639717	-2.041544
Gm37728	Gm37728-001	2539	0.036992382	0.077253233	0.24291049	-4.11674276	5.909225141	24.32675981	-2.041503
Rest	Rest-002	4732.9098	0.001876441	0.007443607	0.242926344	-4.116474087	13.31514085	54.81143227	-2.041409
Hnrnph1	Hnrnph1-014	1909.0784	0.000486959	0.002620321	0.24293723	-4.116289624	4.333577932	17.83826188	-2.041344
Ubap2l	Ubap2l-010	449.00139	0.00382616	0.012960735	0.24296042	-4.115896739	1.109774234	4.567716151	-2.041207
Pds5a	Pds5a-201	13703.681	3.47529E-07	1.88161E-05	0.243003235	-4.115171546	32.44124895	133.5013046	-2.040953
Prdm10	Prdm10-002	3326.0774	5.63871E-07	2.47597E-05	0.243020956	-4.114871482	7.276727696	29.94279928	-2.040847
Gm4788	Gm4788-002	6931.7753	0.010238692	0.028112491	0.243024719	-4.114807767	21.03874226	86.57038006	-2.040825
Gm13134	Gm13134-001	628.20124	0.000290718	0.001766068	0.243049573	-4.114386983	1.000879019	4.118003607	-2.040677
Eif4g2	Eif4g2-013	404.97485	0.001457396	0.006124641	0.243090456	-4.113695033	1.005057802	4.134501289	-2.040435
RP24-237I4.	24-237I4.3-0	456	0.011732988	0.031275184	0.243265917	-4.11072793	0.973646391	4.002395415	-2.039394
10310C07R	0310C07Rik-	424.36549	0.003996614	0.013409487	0.243270074	-4.110657696	1.320371461	5.427595108	-2.039369
Ccdc28b	Ccdc28b-003	765.00315	0.048698227	0.09608908	0.243270628	-4.110648333	1.585034992	6.515521448	-2.039366
Mpc2	Mpc2-005	567.33068	0.001491062	0.006226804	0.2432823	-4.110451102	1.644017557	6.757653777	-2.039297
Herc1	Herc1-005	951.13446	0.000121432	0.000923825	0.243348378	-4.109334973	2.270347879	9.32961994	-2.038905
Ptma	Ptma-001	48439.446	0.000116756	0.000896498	0.243417122	-4.10817445	130.3842014	535.6410448	-2.038497
Cnot1	Cnot1-201	12448.58	3.89097E-06	8.2671E-05	0.243439579	-4.107795478	24.0006769	98.58987202	-2.038364
St6galnac4	St6galnac4-00	1141.1245	0.000604524	0.003094003	0.243446923	-4.107671558	1.894053239	7.780148618	-2.038321
Slc25a16	Slc25a16-001	2493.7549	5.01254E-05	0.000481434	0.243499447	-4.106785501	5.637363582	23.15144303	-2.03801
Atp2c1	Atp2c1-010	487.14983	0.01012528	0.027856924	0.243532772	-4.10622353	1.006016632	4.130929166	-2.037812
Sec24a	Sec24a-002	1827.8211	8.28135E-05	0.000696574	0.243556882	-4.105817049	4.215332622	17.30738455	-2.037669
Nsun4	Nsun4-006	207.31683	0.015989227	0.039784309	0.243589651	-4.105264712	0.523817244	2.150408449	-2.037475
Paics	Paics-001	15249.194	1.04774E-05	0.000159702	0.243629762	-4.104588828	26.93369418	110.5517402	-2.037238
RP23-31J24.	23-31J24.2-0	1220	7.21431E-05	0.000631944	0.243633226	-4.104530473	2.754008618	11.3039123	-2.037217
Med13	Med13-001	42203.82	2.1528E-09	1.31667E-06	0.243639945	-4.104417285	97.41303678	399.8237519	-2.037177
Rpl35a	Rpl35a-003	710.21343	0.003217704	0.011354705	0.243667822	-4.103947716	1.640111837	6.730933227	-2.037012
Ptbp3	Ptbp3-009	12309.126	1.32475E-05	0.000188446	0.243737429	-4.102775688	32.00046224	131.2907185	-2.0366
Zfp429	Zfp429-002	566.04143	0.000629456	0.003192961	0.243762992	-4.102345443	1.272874618	5.221771387	-2.036449
Tmem201	Tmem201-003	963.12838	0.000235653	0.001507864	0.243828448	-4.101244164	2.363118323	9.691725229	-2.036062
Eif4a2	Eif4a2-015	508.91536	0.003337461	0.011673614	0.243841155	-4.101030448	1.630451082	6.686529533	-2.035986
Gm20045	Gm20045-001	2372	1.60017E-05	0.000214672	0.243988956	-4.098546168	4.95569633	20.3111502	-2.035112
Znfx1	Znfx1-005	768.60135	0.024659504	0.055888565	0.244005633	-4.098266044	1.805575338	7.3997281	-2.035014
RP24-188E19	24-188E19.4-	1201	0.000169375	0.001179566	0.244008976	-4.09820989	2.115330642	8.669068956	-2.034994
Arih1	Arih1-006	459.82605	0.001491652	0.006228095	0.244057621	-4.097393044	1.324047014	5.425141024	-2.034706
Egfl7	Egfl7-019	5934.877	2.1236E-05	0.000263948	0.244094296	-4.09677742	12.021038	49.24751704	-2.03449
Stag1	Stag1-005	1299.0834	0.004039223	0.013521194	0.244099762	-4.096685686	2.943249285	12.05756722	-2.034457
Exph5	Exph5-002	132	0.020715067	0.04880999	0.244172036	-4.095473075	0.322129625	1.319273205	-2.03403
Supt7l	Supt7l-002	1171.4485	0.001368126	0.005823802	0.244175037	-4.095422744	2.16564432	8.869229006	-2.034012
Derl2	Derl2-003	2021.7078	9.70607E-06	0.000151588	0.244183235	-4.095285242	4.693691957	19.2220074	-2.033964
Gbp7	Gbp7-001	15199.714	0.000129234	0.000967914	0.244197628	-4.095043878	28.93468895	118.4888208	-2.033879
Tigd3	Tigd3-201	555	0.002620761	0.00967357	0.244288782	-4.093515842	1.241092085	5.080430112	-2.03334
Gm16973	Gm16973-202	1525.2205	2.18756E-05	0.000269123	0.244290531	-4.093486534	3.09337665	12.66269566	-2.03333
R3hcc1l	R3hcc1l-002	726.26799	0.000711427	0.003506457	0.244297103	-4.093376423	1.827204514	7.47943588	-2.033291
Smarcc1	Smarcc1-003	2185.9449	0.021478819	0.050244414	0.244320598	-4.092982783	6.033037963	24.69312051	-2.033153
Krit1	Krit1-009	18829.296	3.9648E-07	2.0359E-05	0.244332369	-4.092785597	42.26600546	172.9856984	-2.033083
930526L06R	0526L06Rik-	215	0.030032539	0.065388811	0.244359145	-4.092337119	0.480204655	1.965159335	-2.032925
Bclaf1	Bclaf1-001	3405.7868	0.000517943	0.002744788	0.244362535	-4.092280347	8.583693072	35.12687847	-2.032905
Gimap6	Gimap6-002	2409.863	1.0322E-05	0.000157967	0.244411607	-4.091458722	6.718861711	27.48994535	-2.032615
Mier3	Mier3-003	234.22202	0.038894882	0.080306009	0.244413421	-4.091428344	0.5816563	2.37980507	-2.032605
Etl4	Etl4-013	4930.9704	0.00873532	0.024760796	0.244443962	-4.090917157	12.0682437	49.37018521	-2.032424
Ctnnd1	Ctnnd1-020	558.7462	0.025111036	0.05668312	0.244451528	-4.090790545	1.24153635	5.078865162	-2.03238

Zfp799	Zfp799-001	1397.7739	0.002400285	0.009032223	0.244486684	-4.090202304	3.781045975	15.46524296	-2.032172
Gm11345	Gm11345-001	204	0.016218402	0.04022596	0.244512872	-4.089764239	0.421519879	1.723916927	-2.032018
Rps15a-ps3	ps15a-ps3-00	491	0.012253602	0.032310868	0.244613835	-4.088076217	1.415351978	5.786066758	-2.031422
Fbxo11	Fbxo11-004	9008.8161	1.08714E-05	0.000164318	0.24462184	-4.087942433	19.29360699	78.87115469	-2.031375
Antxr2	Antxr2-001	3187.547	0.000975619	0.004462468	0.244676109	-4.087035729	9.210488914	37.64359727	-2.031055
Gm37078	Gm37078-001	514	0.00262787	0.009693544	0.244773982	-4.085401533	1.230869206	5.028594942	-2.030478
Znrf2	Znrf2-001	7860.0406	5.74075E-08	6.89897E-06	0.24477964	-4.085307095	19.13746692	78.18242938	-2.030445
Lrp5	Lrp5-003	2904.2396	0.002637857	0.009720958	0.244789664	-4.085139802	4.401258518	17.97975635	-2.030385
Plp2	Plp2-001	5832.9304	0.000107621	0.000840598	0.244816696	-4.084688734	12.9000494	52.69268643	-2.030226
Golm1	Golm1-202	2413.7961	0.000252965	0.001588566	0.244895683	-4.083371285	2.930062963	11.96453497	-2.029761
Tlk1	Tlk1-002	6916.9875	0.000402108	0.002258846	0.244905556	-4.08320667	16.67183611	68.0745524	-2.029703
Zzz3	Zzz3-008	2857.494	3.38543E-06	7.59992E-05	0.244906067	-4.083198155	6.627760968	27.06246136	-2.0297
Adgrl2	Adgrl2-011	463.70995	0.015026511	0.037914984	0.244912268	-4.083094763	1.223062734	4.993881043	-2.029663
Klf3	Klf3-004	8961.9935	9.4064E-07	3.36963E-05	0.244995265	-4.081711538	14.90567394	60.84066128	-2.029174
Mtmr2	Mtmr2-003	4842.5886	0.004195435	0.013942717	0.245018096	-4.081331194	11.46850169	46.8067537	-2.02904
Swt1	Swt1-009	1629.8615	0.000217049	0.001417066	0.245050039	-4.08079919	3.899144967	15.91162762	-2.028852
Gm26720	Gm26720-201	478.96147	0.018158497	0.043983631	0.245062582	-4.080590318	1.366058534	5.574325228	-2.028778
Gapvd1	Gapvd1-002	8472.5967	1.71521E-06	4.82711E-05	0.245085253	-4.080212864	18.39364957	75.05000559	-2.028644
Rtn3	Rtn3-202	3299.8354	5.43963E-06	0.000103046	0.24509382	-4.08007024	7.798694138	31.81921986	-2.028594
Hmmr	Hmmr-001	3386.5264	0.000493344	0.002645992	0.245102476	-4.07992615	8.819600399	35.9833183	-2.028543
Mir17hg	Mir17hg-001	1103.2963	0.017073695	0.041907272	0.245126975	-4.079518381	3.59358173	14.66008272	-2.028399
Ganc	Ganc-001	1819.1891	0.001692999	0.006858096	0.245181027	-4.078619014	3.205037207	13.07212569	-2.028081
Gm26562	Gm26562-201	471	0.000155522	0.001109714	0.245186565	-4.078526902	1.149525282	4.688369786	-2.028048
Elp5	Elp5-002	972.3611	0.012348932	0.032504683	0.24518663	-4.078525823	2.008387606	8.191260713	-2.028048
Pld4	Pld4-201	912	0.049013017	0.096565569	0.245209241	-4.078149733	3.858587908	15.73589925	-2.027915
P23-371A16	23-371A16.4-	261	0.009815515	0.027154101	0.245215949	-4.078038175	0.669286563	2.729376155	-2.027875
21523L03R	1523L03Rik-	380.91344	0.003824791	0.012960735	0.245270875	-4.077124929	0.902712418	3.680471301	-2.027552
Mga	Mga-009	386.7794	0.00163828	0.00668865	0.245278689	-4.076995048	0.904163497	3.686270099	-2.027506
Gm14963	Gm14963-001	1096	2.38391E-05	0.000284888	0.245301023	-4.076623854	2.414391616	9.842566456	-2.027375
AC137871.	C137871.1-2	238	0.003435071	0.011924912	0.245302062	-4.076606577	0.499275261	2.035348813	-2.027369
Tnfrsf23	Tnfrsf23-002	2397.2819	0.005025783	0.016064681	0.245314849	-4.076394095	8.090434207	32.97979823	-2.027294
Pomp	Pomp-002	1788.09	3.40878E-05	0.000365646	0.245336439	-4.076035362	4.028940905	16.4221056	-2.027167
Ifngr2	Ifngr2-001	3017.5148	0.000505972	0.002696036	0.245346635	-4.075865972	9.565899655	38.98932489	-2.027107
Dpm1	Dpm1-009	2032.4244	0.000105466	0.000828527	0.245457962	-4.074017362	4.707085871	19.17674956	-2.026452
Snx7	Snx7-004	771	0.000887248	0.004157078	0.245573072	-4.072107707	1.633204798	6.650585844	-2.025776
Strbp	Strbp-201	13977.253	2.82878E-05	0.000321769	0.245622143	-4.07129417	24.54744505	99.93986991	-2.025487
Aspscr1	Aspscr1-015	679.7726	0.001466415	0.006148208	0.245624108	-4.071261609	1.708545364	6.955935149	-2.025476
Gm37163	Gm37163-001	379	0.004128432	0.013753498	0.245672695	-4.070456425	0.825068866	3.358406867	-2.025191
Chrnb4	Chrnb4-201	367	0.004026928	0.013490052	0.245733239	-4.069453537	0.87379889	3.555883985	-2.024835
Fam20a	Fam20a-004	1383.7865	0.028967323	0.06345982	0.245823073	-4.067966393	1.041061086	4.235001509	-2.024308
Phf5a	Phf5a-001	1819.5784	0.001214802	0.00530769	0.245852886	-4.06747309	4.63426492	18.84974785	-2.024133
Ap5z1	Ap5z1-001	833.6498	0.010686023	0.02904502	0.245884595	-4.066948569	1.985824341	8.076245459	-2.023947
Atg2a	Atg2a-004	375.65071	0.002936067	0.010576387	0.245897312	-4.066738225	0.806228882	3.278721812	-2.023872
Eif4h	Eif4h-001	1966.0242	0.014031719	0.035961267	0.245913263	-4.066474444	3.581294344	14.56324193	-2.023779
Gm15417	Gm15417-001	187.91066	0.014639988	0.037183506	0.245913842	-4.066464873	0.303358998	1.233598711	-2.023775
Dhrs7b	Dhrs7b-001	1691.237	2.97211E-05	0.000333753	0.24592939	-4.066207786	3.354977389	13.64203518	-2.023684
Zrsr2	Zrsr2-002	2838.4719	2.20151E-05	0.00027016	0.246008201	-4.064905134	7.578270786	30.80495183	-2.023222
Stxbp1	Stxbp1-004	363.82599	0.019376859	0.046342314	0.246023225	-4.064656905	0.605971178	2.463064931	-2.023134
Mrpl1	Mrpl1-201	1049.0891	0.000655769	0.003292787	0.246131279	-4.062872477	2.499042429	10.1532907	-2.0225
Psmd14	Psmd14-001	4390.995	0.000496706	0.002658155	0.246149258	-4.062575721	12.18580189	49.5057429	-2.022395
Mrpl1	Mrpl1-001	196.63241	0.007921779	0.022956682	0.246162009	-4.06236528	0.468478003	1.903128773	-2.02232
Plcb4	Plcb4-013	847.88788	0.004875513	0.015682412	0.246178613	-4.062091299	1.574165721	6.394404878	-2.022223
Idi1	Idi1-001	946.03112	0.001081032	0.004837385	0.246201659	-4.061711055	2.259257364	9.176450611	-2.022088
Trpm2	Trpm2-004	1253.4663	0.000160952	0.001137326	0.246216452	-4.061467024	3.469713524	14.09212706	-2.022001
Gm37983	Gm37983-001	571	0.002953102	0.010624342	0.246219819	-4.061411485	1.400731057	5.688945204	-2.021981
00007E05R	0007E05Rik-	280.32263	0.009247676	0.025920967	0.24627549	-4.060493393	0.693336463	2.815288127	-2.021655
Rps15a-ps7	ps15a-ps7-00	1226	0.000132677	0.000987719	0.246276824	-4.060471394	3.474125245	14.10658618	-2.021647
Chrac1	Chrac1-002	409.82694	0.022454106	0.051981835	0.246283582	-4.06035998	1.202087416	4.880907638	-2.021608
Zfp850	Zfp850-002	610.07664	0.010190751	0.028001061	0.246283722	-4.060357679	1.886671393	7.660560679	-2.021607
Zbtb37	Zbtb37-001	10263.298	1.11054E-06	3.74371E-05	0.246293797	-4.06019157	22.48316038	91.28593825	-2.021548
31406P16R	1406P16Rik-	1046.0794	0.002521664	0.009374469	0.24630414	-4.060021072	2.522274881	10.24048917	-2.021487
Rhou	Rhou-001	1506.6505	0.01715878	0.042067875	0.246311927	-4.059892727	3.519481504	14.28871736	-2.021442
Kdm5b	Kdm5b-006	569.06222	0.00727329	0.021469421	0.246318851	-4.059778601	1.484522452	6.026832484	-2.021401
Mecom	Mecom-001	6403.413	0.00328554	0.011532049	0.246337844	-4.059465581	16.25711285	65.99519005	-2.02129
Postn	Postn-002	1058.2881	0.020679798	0.048749611	0.246343125	-4.059378556	3.551344459	14.41625154	-2.021259
Spast	Spast-201	5924	8.10154E-07	3.08652E-05	0.246362087	-4.059066113	14.36568605	58.31126942	-2.021148
Lgals8	Lgals8-003	1114.1873	0.008031176	0.023196809	0.246412214	-4.058240388	2.43705283	9.890146222	-2.020854
Gm29488	Gm29488-001	469	0.003733567	0.01272648	0.246437428	-4.05782517	1.236520312	5.017583244	-2.020707
Taf2	Taf2-201	9008	1.66867E-07	1.26693E-05	0.246472457	-4.057248474	23.21431643	94.18624988	-2.020502
30581F22R	0581F22Rik-	424.64068	0.004884322	0.015706134	0.246490306	-4.056954681	1.410295611	5.721505378	-2.020397
Slc39a13	Slc39a13-002	4122.8608	9.73224E-06	0.000151928	0.246502023	-4.056761831	9.360885994	37.97488501	-2.020329
Phykpl	Phykpl-003	4855.7624	5.51966E-07	2.44881E-05	0.246505515	-4.056704367	12.49519286	50.68930342	-2.020308
Slc18b1	Slc18b1-001	1182.0063	0.017671394	0.043070149	0.246527918	-4.056335727	2.700519899	10.95421535	-2.020177
Ap1b1	Ap1b1-001	745.53291	0.04099132	0.083768062	0.246704278	-4.053435985	1.083430613	4.391616635	-2.019145
Wdr91	Wdr91-008	512.47026	0.001470123	0.00616149	0.246758421	-4.052546606	1.214100549	4.92019906	-2.018829
Gm13075	Gm13075-001	1157	2.40002E-05	0.000286214	0.246773789	-4.052294226	2.685924474	10.88415624	-2.018739
Phc3	Phc3-007	1543.759	0.000744424	0.003624755	0.246783747	-4.052130717	3.688564492	14.94654548	-2.018681
Cpsf6	Cpsf6-004	882.59273	0.001227949	0.005358296	0.246915109	-4.049974927	2.314785391	9.374822793	-2.017913
Ppil4	Ppil4-003	2267.6641	2.57831E-05	0.000302008	0.246940909	-4.049551783	5.229287053	21.17626871	-2.017762
Otud7b	Otud7b-002	4371.9795	0.001841421	0.007336977	0.246961588	-4.049212714	10.1985084	41.29592987	-2.017641
Gm17130	Gm17130-001	177.04671	0.015857248	0.039537523	0.246991549	-4.048721514	0.597586183	2.419460037	-2.017466
Jakmip1	Jakmip1-002	634.32416	0.001946472	0.007674304	0.246994288	-4.048676625	1.378877137	5.582627634	-2.01745
Dnajc24	Dnajc24-002	302.87069	0.006516948	0.019657021	0.247017387	-4.048298019	0.626892719	2.537848554	-2.017315
Wdyhv1	Wdyhv1-201	757	0.001150038	0.005087677	0.247036623	-4.047982798	1.89007536	7.650992545	-2.017203
Gm14295	Gm14295-002	1197.1252	0.000338191	0.001980124	0.247054157	-4.047695507	2.655109762	10.74707586	-2.017101
Tsfm	Tsfm-006	217.72731	0.044100127	0.088780893	0.247074088	-4.047368981	0.526519589	2.131019051	-2.016984
Rbfox2	Rbfox2-202	3514.3481	0.004263359	0.014121299	0.247092562	-4.047066383	6.48380745	26.24039917	-2.016877
Gm37916	Gm37916-001	89	0.031379723	0.067729996	0.247098072	-4.046976131	0.255487334	1.033951142	-2.016844
Ttf1	Ttf1-002	469.19153	0.00366077	0.012531871	0.247108947	-4.046798028	1.040561968	4.21094412	-2.016781
Slc38a9	Slc38a9-201	3859	1.27526E-05	0.000183211	0.247135875	-4.046357097	10.04953752	40.66401748	-2.016624
Rnf149	Rnf149-001	2864.0088	0.0452211	0.090532072	0.247149484	-4.046134281	10.68938744	43.25069694	-2.016544
P23-382L1	23-382L1.3-0	241	0.005547159	0.017366685	0.247150077	-4.04612457	0.622390314	2.51826874	-2.016541
Xrn1	Xrn1-002	3039.6765	0.004041561	0.013525841	0.247164887	-4.045882135	7.206029149	29.1547446	-2.016454
Rab10	Rab10-201	19667.728	6.7013E-08	7.59514E-06	0.247169447	-4.045807487	45.94248856	185.8744642	-2.016428
Ubtf	Ubtf-005	592.03355	0.011513637	0.030817519	0.247226048	-4.04488123	0.88605512	3.583987725	-2.016097
Cd9	Cd9-003	2380.1307	1.26425E-05	0.000182241	0.247235865	-4.044720618	5.631869811	22.77933994	-2.01604
Gatad2b	Gatad2b-007	3769	3.02662E-05	0.000337059	0.247261305	-4.044304467	8.840982194	35.75562378	-2.015892
Gm7488	Gm7488-001	772	0.001784489	0.007161347	0.247264897	-4.044245718	1.971539667	7.973390854	-2.015871
Trim23	Trim23-004	473.90225	0.016934203	0.041657433	0.247273683	-4.044102017	1.027519122	4.155392151	-2.015819
Fam118b	Fam118b-007	5516.9719	3.71291E-06	8.05433E-05	0.247285915	-4.043901974	8.904045089	36.00708551	-2.015748
Sc5d	Sc5d-201	1647.5705	3.46154E-05	0.000369339	0.247304638	-4.043595816	3.28600734	13.28728553	-2.015639
Gm38020	Gm38020-001	1769	0.00162216	0.006639457	0.247341402	-4.042994788	4.620327918	18.67996169	-2.015424
42435	March6-201	29930	1.10448E-08	2.78999E-06	0.247350172	-4.04285144	64.92900522	262.4983223	-2.015373
Dcaf10	Dcaf10-001	7321.9681	5.13277E-08	6.39263E-06	0.247358509	-4.042715176	16.82620909	68.02357086	-2.015325
Gnpda2	Gnpda2-001	856.58472	0.035460284	0.074628696	0.247385057	-4.04228134	1.757467714	7.104178948	-2.01517
30017A19F	0017A19Rik-	380	0.018418305	0.044499644	0.247404559	-4.04196271	1.017173993	4.11137935	-2.015056
Txndc9	Txndc9-005	2342.8988	2.04124E-05	0.000256559	0.247473218	-4.040841302	6.095004769	24.628947	-2.014656

Xpr1	Xpr1-006	1075.7865	0.003609939	0.012395268	0.247481615	-4.040704201	2.659671834	10.74694715	-2.014607
Rpl31-ps11	.pl31-ps11-00	1091	2.87954E-05	0.000325816	0.247568208	-4.039290863	2.843201981	11.48451978	-2.014102
Dennd1b	Dennd1b-004	747.93719	0.019177896	0.045965217	0.247608309	-4.038636679	2.258977644	9.123189972	-2.013868
Srsf11	Srsf11-005	8518.4336	4.27468E-05	0.000431246	0.247608395	-4.038635278	20.06924594	81.05236467	-2.013868
Gpx1	Gpx1-001	11036.803	7.16385E-05	0.000628328	0.24765296	-4.037908539	26.12348188	105.4842305	-2.013608
Slc29a3	Slc29a3-003	1505.6291	0.004954126	0.015889018	0.247714815	-4.036900251	4.402333183	17.77177994	-2.013248
Zmat1	Zmat1-003	1365.0118	0.002482495	0.009271121	0.247739768	-4.036493654	3.14550957	12.69682942	-2.013103
Pex1	Pex1-014	302	0.007580551	0.022183346	0.247744914	-4.036409802	0.715103481	2.886450699	-2.013073
Gm37031	3m37031-001	501	0.007575667	0.022170947	0.247812215	-4.035313599	1.104651927	4.457616943	-2.012681
Nrd1	Nrd1-008	3578.2335	0.001836772	0.007321861	0.247843477	-4.034804598	8.486366532	34.24083071	-2.012499
33434E20R	3434E20Rik-	425.41774	0.033867648	0.072034213	0.247864381	-4.034464315	1.070484193	4.318830275	-2.012377
Pkn2	Pkn2-001	7968.1684	1.16201E-06	3.85647E-05	0.247875082	-4.03429014	18.99956507	76.64975804	-2.012315
Zfp111	Zfp111-001	3942.7316	0.00023849	0.001520248	0.247882493	-4.034169521	9.050779762	36.51237986	-2.012272
Cr1l	Cr1l-005	8167.516	8.29144E-05	0.000696872	0.247960878	-4.032894259	18.42981115	74.32547957	-2.011816
Dfna5	Dfna5-003	442.00917	0.008728132	0.024748614	0.247984872	-4.03250404	1.005186694	4.053419402	-2.011676
Gm8979	Gm8979-001	3930	3.66702E-05	0.000385144	0.248001567	-4.03223258	8.438449249	34.02578998	-2.011579
Bcl7a	Bcl7a-002	1288.8515	0.000395294	0.002231186	0.248049421	-4.031454682	3.665873027	14.77880098	-2.011301
Stx16	Stx16-201	5317.3219	3.29881E-05	0.000357658	0.248068161	-4.031150129	11.87687583	47.87746952	-2.011192
Pcgf6	Pcgf6-201	623	0.015147925	0.038159491	0.248156582	-4.029713781	2.241707972	9.033441506	-2.010677
Ahsa2	Ahsa2-007	455.56753	0.001033652	0.004672864	0.248182662	-4.029290332	1.176056211	4.738671921	-2.010526
Dnm2	Dnm2-011	1131.0081	0.005333708	0.016860128	0.248191005	-4.029154885	2.109530474	8.499625015	-2.010477
Gm10020	3m10020-201	2527	5.40468E-05	0.000510086	0.248200499	-4.029000759	7.198802917	29.00398242	-2.010422
Gm17613	3m17613-001	223	0.017939904	0.043571246	0.248295423	-4.027460464	0.673468122	2.712366234	-2.00987
P23-397C5.	23-397C5.5-(	183	0.038892581	0.080306009	0.248304306	-4.027316389	0.483980611	1.949143047	-2.009819
P23-153H17	23-153H17.5-	2224	3.22607E-06	7.39401E-05	0.248337407	-4.02677959	5.624678386	22.64934012	-2.009627
Rapgef2	Rapgef2-003	1127.0884	0.000266715	0.001652193	0.248385101	-4.026006373	2.342718657	9.431800241	-2.009349
Gmfb	Gmfb-001	7915.0966	8.72155E-07	3.22933E-05	0.24839934	-4.025775585	19.59746117	78.89498073	-2.009267
Gnai3	Gnai3-001	20101	1.20662E-08	2.8812E-06	0.248404224	-4.025696433	42.64912354	171.6924245	-2.009238
Bbs12	Bbs12-001	247.38397	0.026987557	0.060040522	0.248493785	-4.024245518	0.868028532	3.493159931	-2.008718
Gas2l3	Gas2l3-201	1324.9263	0.000422457	0.002348126	0.248526407	-4.023717284	3.564957969	14.344383	-2.008529
Ncoa4	Ncoa4-003	6892.2635	0.000574153	0.002976721	0.248538898	-4.02351506	12.52833043	50.40792617	-2.008456
Gm12798	3m12798-001	540	0.00487865	0.015691029	0.248568665	-4.023033225	1.258208534	5.061814737	-2.008284
Gas2l3	Gas2l3-202	1349.0737	0.000415072	0.002314592	0.24859838	-4.022552357	3.63057291	14.60416962	-2.008111
Utp14a	Utp14a-002	1806.3195	3.22914E-06	7.39401E-05	0.248638169	-4.021908641	4.495590987	18.08085624	-2.00788
P24-428N8	24-428N8.5-(	1154	0.000330329	0.001945825	0.248662819	-4.02150995	2.575553486	10.35761397	-2.007737
Epb4.1l2	Epb4.1l2-202	12423.768	1.2887E-05	0.000184216	0.248667964	-4.021426743	26.86546062	108.0374818	-2.007707
Gmip	Gmip-009	818.30759	2.88904E-05	0.000326458	0.248692278	-4.02103357	1.836381953	7.384153482	-2.007566
RP23-23C4.	23-23C4.5-0	2175	1.02909E-05	0.000157596	0.248706957	-4.020796245	4.90851486	19.73613812	-2.007481
Fam78b	Fam78b-001	526.58431	0.008501081	0.024230941	0.248722483	-4.020545263	0.816441419	3.282539681	-2.007391
Pex11g	Pex11g-001	1459.1714	0.006663781	0.020010038	0.248726815	-4.020475232	2.694222513	10.83205488	-2.007366
Phactr2	Phactr2-201	42979.439	4.6378E-07	2.22784E-05	0.248745773	-4.020168811	105.1988534	422.9171495	-2.007256
Ankrd49	Ankrd49-201	1681	0.003442614	0.011946254	0.248795794	-4.019360547	4.429003221	17.80176081	-2.006966
Ifi27	Ifi27-009	3790.7036	0.000529518	0.002791871	0.248821801	-4.01894045	6.831402792	27.45500101	-2.006815
Gm14391	3m14391-201	472.37959	0.020181883	0.04782933	0.248826964	-4.018857055	1.130309009	4.542550335	-2.006785
Ankrd17	Ankrd17-011	3594.7893	3.36769E-06	7.57344E-05	0.248832854	-4.01876192	8.65435355	34.77978649	-2.006751
Gxylt1	Gxylt1-001	11408.277	3.91208E-07	2.01715E-05	0.24885459	-4.018410912	27.9651515	112.3754699	-2.006625
Gnpda1	Gnpda1-001	1385.9015	9.62317E-05	0.000775187	0.248870159	-4.018159525	3.236169219	13.00344417	-2.006535
Baz1b	Baz1b-005	1425.5081	3.41475E-05	0.000365779	0.248878972	-4.018017245	3.592787576	14.43588244	-2.006484
Zmat3	Zmat3-003	197.36339	0.014021286	0.035939907	0.248883919	-4.017937366	0.40949108	1.64530951	-2.006455
P23-267O21	23-267O21.2-	522	0.006888725	0.020552133	0.248916005	-4.017419457	1.191891133	4.788326626	-2.006269
Cisd2	Cisd2-002	470.17369	0.04203264	0.085487999	0.248919045	-4.017370389	1.224453679	4.919083953	-2.006251
Fnbp4	Fnbp4-005	1298.3097	7.83209E-05	0.000669786	0.248936369	-4.017090803	3.63385329	14.59751863	-2.006151
Use1	Use1-003	758.39914	0.003742846	0.012746592	0.24903652	-4.015475317	1.340618395	5.383220075	-2.005571
Cd93	Cd93-001	282688	1.76594E-07	1.3206E-05	0.249060434	-4.015089767	566.5087194	2274.583362	-2.005432
Blcap	Blcap-004	122.03871	0.031889315	0.068597477	0.249080892	-4.014759985	0.363764642	1.46042773	-2.005314
Itsn1	Itsn1-001	1051.0424	0.000825188	0.003924827	0.249167408	-4.013365981	2.344046734	9.40751742	-2.004813
Hykk	Hykk-001	3352.1107	0.000765259	0.00370128	0.249205154	-4.012758103	5.46325216	21.92270937	-2.004594
Gm26690	3m26690-201	581.97793	0.003691785	0.012621645	0.24920765	-4.012717901	1.381430744	5.543291874	-2.00458
Timm17b	Timm17b-001	1616.1764	4.39793E-05	0.00044005	0.249274342	-4.011644324	3.766529425	15.10997639	-2.004194
Epha4	Epha4-002	5104.1425	0.002391198	0.009005776	0.249328459	-4.010773589	10.00716007	40.13645332	-2.003881
Ciao1	Ciao1-001	2140.8996	0.000271488	0.001673696	0.249332317	-4.01071154	4.899678547	19.65119729	-2.003858
Fam96a	Fam96a-001	3588	0.000123397	0.000936481	0.249357638	-4.010304269	10.5706671	42.39159139	-2.003712
Eif4e	Eif4e-001	3727.1511	1.37607E-05	0.000193451	0.249572205	-4.00685645	10.05943566	40.30671466	-2.002471
Gm37759	3m37759-001	326.91328	0.003852192	0.013029586	0.249603114	-4.006360275	0.693263688	2.7774641	-2.002292
Gm13394	3m13394-001	1222	0.000713174	0.003513048	0.2496554	-4.005521214	3.467852054	13.89055497	-2.00199
Kctd2	Kctd2-001	4365.4341	1.38652E-05	0.000194464	0.249667315	-4.005330046	9.372520954	37.54003978	-2.001921
Gpcpd1	Gpcpd1-006	1599.1647	0.001527168	0.006338522	0.24967534	-4.005201313	4.365908952	17.48634427	-2.001875
Il5ra	Il5ra-201	87	0.03832025	0.079428018	0.249678039	-4.005158016	0.237966988	0.953095391	-2.001859
30062P05R	0062P05Rik-	1041	3.14834E-05	0.000346945	0.249718807	-4.004504162	2.517193599	10.08011224	-2.001624
Gm26710	3m26710-201	215	0.040624759	0.083140058	0.249722962	-4.004437525	0.595833122	2.385976511	-2.0016
Ahsa2	Ahsa2-003	1700.8541	0.000151168	0.001086342	0.249788732	-4.003383142	4.222834691	16.90562521	-2.00122
Gm26699	3m26699-201	1525.2712	0.000110201	0.000857818	0.249792932	-4.00331584	3.497304456	14.00081433	-2.001195
Fcf1	Fcf1-001	2882.8605	0.000425627	0.002361529	0.249869025	-4.002096705	7.333781725	29.35050368	-2.000756
Gsk3b	Gsk3b-002	1139.2052	0.000521918	0.002760299	0.249891232	-4.001741053	2.93820839	11.75794914	-2.000628
Ndrg3	Ndrg3-003	487.5444	0.00563624	0.017574766	0.249907223	-4.001484989	0.933285894	3.734529494	-2.000535
Gm6505	Gm6505-001	410	0.0030445	0.010881156	0.249973051	-4.000431236	1.128579527	4.514804791	-2.000156
Gm37678	3m37678-001	863.30329	0.000333905	0.001960273	0.249978308	-4.000347109	2.133157278	8.533369551	-2.000125
Slc9a8	Slc9a8-002	761.19642	0.002545416	0.009450459	0.249985095	-4.000238498	1.658887236	6.635944587	-2.000086
Gtf2h5	Gtf2h5-002	1259.9606	0.012551349	0.032923618	0.249985488	-4.00023221	3.336342853	13.34614615	-2.000084
Tm9sf4	Tm9sf4-007	126.1915	0.046254031	0.092225931	0.250007807	-3.99987509	0.324860289	1.299400578	-1.999955
Ift22	Ift22-001	1249.3811	0.000224517	0.001453908	0.250043549	-3.999303335	3.2328688	12.92922298	-1.999749
Tfg	Tfg-001	4523.1615	2.79214E-05	0.000319408	0.250087744	-3.998596593	12.47207667	49.87080328	-1.999494
Ercc6l	Ercc6l-001	1918	0.002541	0.009437132	0.250094255	-3.998492488	5.382215672	21.52074893	-1.999456
Gm12840	3m12840-001	30009	0.002350378	0.008883814	0.25013511	-3.997839407	90.63483148	362.3435009	-1.999221
Dapk1	Dapk1-201	4227.0199	0.005793715	0.017946525	0.250206972	-3.996691195	8.672712678	34.66215439	-1.998806
Kmt2e	Kmt2e-010	3234.2215	0.000169627	0.001180675	0.250244806	-3.996086943	7.659630036	30.60854757	-1.998588
Gm25960	3m25960-201	131	0.018967811	0.045566917	0.250265505	-3.995756422	0.298674659	1.193431188	-1.998469
Tpp2	Tpp2-003	1917.4199	6.89763E-05	0.000611397	0.250278704	-3.995545707	5.121569083	20.46346336	-1.998393
Uggt1	Uggt1-003	2500.6744	0.003617592	0.012412594	0.250303303	-3.995153029	5.999500649	23.96892319	-1.998251
Zc2hc1a	Zc2hc1a-001	2402.9695	0.003646892	0.012495603	0.250314681	-3.994971432	5.398966779	21.56871805	-1.998185
Tdrd3	Tdrd3-002	2131.4005	0.000151233	0.001086583	0.25031981	-3.994889582	4.686317918	18.72132262	-1.998156
Ap1ar	Ap1ar-006	579.91277	0.001620889	0.006635049	0.250330082	-3.994725657	1.634990274	6.531337596	-1.998096
Capn7	Capn7-002	2534.285	0.008592166	0.024435912	0.250337698	-3.994604128	6.071504327	24.25325625	-1.998053
Fbxo28	Fbxo28-001	4213.3693	1.17061E-05	0.000172001	0.250346777	-3.99445926	10.52531565	42.04294455	-1.998
32433K11F	2433K11Rik-	530.74118	0.009869592	0.027278368	0.250389766	-3.993773457	1.016809177	4.060905502	-1.997752
Stx19	Stx19-001	727	9.60996E-05	0.00077445	0.250393988	-3.993706104	1.44041226	5.752583233	-1.997728
Gm37819	3m37819-001	267	0.033224162	0.070881247	0.250401282	-3.993589772	0.603601516	2.410536842	-1.997686
Gm10182	3m10182-201	879	0.000677202	0.003373208	0.250421872	-3.993261425	2.128129763	8.498178489	-1.997568
Sgk3	Sgk3-010	1153.9843	0.000353733	0.002047202	0.250449077	-3.992827645	3.008469861	12.01230163	-1.997411
BC025920	3C025920-20	1119.9187	0.000114371	0.000882359	0.250480245	-3.99233082	2.735745806	10.9220023	-1.997231
Camk2d	Camk2d-004	1367.4245	0.000289686	0.00176105	0.250491763	-3.992147245	3.339546784	13.33196249	-1.997165
Gm38111	3m38111-001	620	0.000724957	0.003554217	0.250501223	-3.991996477	1.590555433	6.349491684	-1.99711
Cdk2ap2	Cdk2ap2-004	523.273	0.03869991	0.080021245	0.250545656	-3.991288519	1.339525219	5.346431629	-1.996855
Actr10	Actr10-201	6083	4.76993E-06	9.47836E-05	0.250567778	-3.990936142	14.94193784	59.63231976	-1.996727
Ap2a2	Ap2a2-002	593.45889	0.003217648	0.011354705	0.2505848	-3.99066503	1.378681739	5.501857004	-1.996629
Mbnl1	Mbnl1-004	10798.564	5.5512E-07	2.45962E-05	0.250741615	-3.98816925	22.36891078	89.21100211	-1.995727
Kif24	Kif24-001	709.53717	0.006828111	0.02041044	0.250762243	-3.98784119	1.978323274	7.889239041	-1.995608

Ict1	Ict1-001	592.80395	0.001633102	0.006673873	0.250770123	-3.987715871	1.192838152	4.75669963	-1.995563
10002M06F	0002M06Rik-	3434.0426	8.73728E-06	0.000141357	0.250798167	-3.987269971	7.526340669	30.00955214	-1.995401
Gm36988	Gm36988-001	651	0.002298832	0.008737372	0.250828467	-3.98678831	1.589625552	6.337500566	-1.995227
Gpn1	Gpn1-002	847.90574	0.00013222	0.000985106	0.250829543	-3.986771209	1.754541277	6.994954649	-1.995221
Sin3a	Sin3a-004	2971.1515	0.005502779	0.017263918	0.250849151	-3.986459577	6.553428927	26.12497951	-1.995108
Fry	Fry-011	2279	2.87898E-05	0.000325816	0.250912043	-3.985460352	5.16516894	20.58557602	-1.994746
Fip1l1	Fip1l1-010	1240.6737	0.016695666	0.041188975	0.25093658	-3.985070658	3.122570423	12.44366377	-1.994605
Entpd7	Entpd7-001	1295.9752	0.002355106	0.008895278	0.250943112	-3.984966919	4.222762529	16.82756899	-1.994568
Cobll1	Cobll1-003	427.62029	0.039345552	0.081009027	0.250952965	-3.98481046	0.885067326	3.526825538	-1.994511
Gabpb2	Gabpb2-201	7831.1459	3.99661E-06	8.40811E-05	0.2510008	-3.984051044	17.53371122	69.85520051	-1.994236
Trim26	Trim26-006	4269.0511	2.98248E-05	0.000334243	0.251058995	-3.983127553	9.876072219	39.33765537	-1.993902
Mndal	Mndal-002	559.10233	0.027494143	0.060953744	0.25106979	-3.98295629	1.292078781	5.146293309	-1.99384
Pcid2	Pcid2-002	668.15765	0.046797971	0.093095807	0.251099427	-3.982486182	1.719998488	6.849870214	-1.993669
Eif3e	Eif3e-201	18272	1.42954E-08	3.11956E-06	0.251116122	-3.982221416	51.20469426	203.9084301	-1.993573
Dgcr2	Dgcr2-006	219.133	0.018629492	0.044936974	0.251122916	-3.982113683	0.35497398	1.413546744	-1.993534
Cdc16	Cdc16-005	217.98282	0.006182248	0.018888669	0.251183673	-3.981150473	0.56049849	2.231428828	-1.993185
Rps18	Rps18-002	1219.072	0.001199404	0.005253276	0.251202798	-3.980847382	3.477692833	13.84416441	-1.993076
Nudcd2	Nudcd2-001	644.35436	0.003198878	0.011305497	0.251204412	-3.980821805	2.105462268	8.381470104	-1.993066
Hnrnpa1	Hnrnpa1-201	4294.6808	0.000777785	0.003745661	0.251224811	-3.980498568	12.23648909	48.70732731	-1.992949
Papss2	Papss2-201	7628	3.23825E-06	7.39401E-05	0.25127967	-3.979629547	21.45786916	85.39437014	-1.992634
Dynlt1c	Dynlt1c-003	379.87502	0.021828068	0.050871757	0.251281838	-3.979595221	0.874752638	3.481161418	-1.992622
Rpl27	Rpl27-001	5735.0164	2.05183E-05	0.000257391	0.251301181	-3.979288905	16.25858633	64.69761217	-1.992511
Gm13936	Gm13936-001	2350	6.97042E-06	0.000121002	0.251384742	-3.977966172	5.410806029	21.52400335	-1.992031
Map3k12	Map3k12-004	1484.5719	0.000156791	0.001115749	0.251396529	-3.977779667	3.675117767	14.61880873	-1.991963
Otud7b	Otud7b-001	3238.9263	0.000213592	0.001400331	0.251419362	-3.977418418	7.363534116	29.28785621	-1.991832
Gnal	Gnal-202	1144.8743	0.037883472	0.078689363	0.251447387	-3.976975118	2.255489094	8.970024005	-1.991672
Ptges3l	Ptges3l-003	407.4679	0.000478162	0.002583195	0.251469941	-3.976618426	0.903812497	3.594117428	-1.991542
Abcd2	Abcd2-201	352	0.024505216	0.055601433	0.251494377	-3.976232035	1.166856508	4.639692226	-1.991402
Cuta	Cuta-201	1683.2779	0.000529006	0.002789598	0.251497328	-3.976185385	3.900821445	15.51038922	-1.991385
Vangl1	Vangl1-001	3455.7958	0.000148276	0.001070952	0.251508206	-3.976013412	6.593493773	26.21581967	-1.991323
Cnot6	Cnot6-001	7029.0038	6.80997E-06	0.000119366	0.251532865	-3.975623621	18.77285371	74.63380063	-1.991181
Ccdc77	Ccdc77-004	2980.1761	1.60253E-06	4.65309E-05	0.251579972	-3.974879203	7.128066952	28.33320508	-1.990911
Gm30948	Gm30948-001	1041	0.000206873	0.001367049	0.251602538	-3.974522703	2.524018431	10.03176856	-1.990782
42615	Sept2-010	500.48401	0.002814483	0.010225315	0.25167688	-3.973348684	1.252217745	4.975497731	-1.990355
Fubp1	Fubp1-003	6154.6223	2.22272E-06	5.83932E-05	0.251698139	-3.97301309	15.93177861	63.29716497	-1.990234
Senp1	Senp1-005	1650.0169	0.000563357	0.002934599	0.251722053	-3.97263565	3.739488807	14.85562655	-1.990096
Kcmf1	Kcmf1-201	11875	2.20329E-07	1.49728E-05	0.251744313	-3.972284375	29.24949949	116.1873298	-1.989969
P23-403P24	23-403P24.1-	1695	4.44263E-05	0.000443098	0.251791758	-3.971535881	4.112478663	16.33285657	-1.989697
Dld	Dld-201	8512	4.44355E-06	9.02678E-05	0.251809597	-3.971254518	20.7003179	82.20623099	-1.989595
Cbx5	Cbx5-001	24624.608	3.91223E-07	2.01715E-05	0.251899701	-3.969834011	58.96728426	234.0903306	-1.989079
Gm5851	Gm5851-001	481	0.000869809	0.00409104	0.25191558	-3.969583779	1.331931809	5.287214902	-1.988988
Gm28085	Gm28085-001	468	0.0068498	0.020457416	0.25193818	-3.969227694	1.031940953	4.096008607	-1.988858
P23-298M2	23-298M23.7-	684	0.000232344	0.001491616	0.251977115	-3.968614365	1.656713114	6.57485546	-1.988635
Gm26748	Gm26748-201	859	6.60825E-05	0.000591874	0.251999217	-3.968266306	2.072346118	8.223621273	-1.988509
Gapdh	Gapdh-006	12144.943	2.15355E-06	5.71776E-05	0.252000805	-3.968241295	31.50921377	125.0361632	-1.9885
Cox7c	Cox7c-004	661.93418	0.005549127	0.017366685	0.252039391	-3.967633766	1.565596157	6.211712175	-1.988279
Tpd52	Tpd52-007	317.96318	0.021766329	0.050757196	0.252091992	-3.966805902	0.843677222	3.346703781	-1.987978
Ptbp1	Ptbp1-002	1415.7058	0.010445918	0.028564096	0.252121265	-3.966345316	4.039623498	16.02254174	-1.98781
Mr1	Mr1-002	2750.6101	0.004615799	0.015030531	0.252146603	-3.96594675	6.121405252	24.27716727	-1.987665
Gm37167	Gm37167-001	910	9.01013E-05	0.000739331	0.25218298	-3.96537467	2.174824917	8.623995637	-1.987457
Npm1	Npm1-003	26367.27	1.17391E-06	3.87268E-05	0.25222905	-3.964650383	82.0077518	325.1320646	-1.987194
Brd8	Brd8-004	2224.7448	0.000461345	0.00251329	0.252329629	-3.96307007	6.24700916	24.75733503	-1.986618
Gm37366	Gm37366-001	1190	0.001638222	0.00668865	0.252477236	-3.960753118	2.951575731	11.69046278	-1.985775
Ergic2	Ergic2-001	4309.4333	5.44569E-07	2.44129E-05	0.252667333	-3.957773199	9.630756173	38.11634867	-1.984689
BC017158	BC017158-00	2327.3399	3.28814E-06	7.47304E-05	0.252704067	-3.957197894	5.562200745	22.01072907	-1.984479
Ints2	Ints2-001	2061.1148	4.0682E-05	0.000415279	0.25272673	-3.956843023	5.919895294	23.42409639	-1.98435
Atp11a	Atp11a-005	1963.5748	1.12066E-06	3.77411E-05	0.252732813	-3.956747786	4.344579366	17.19040479	-1.984315
Gm37091	Gm37091-001	1356	3.4018E-05	0.000365452	0.252746259	-3.95653729	3.150354534	12.46449519	-1.984238
Pydc4	Pydc4-005	260.50583	0.021065042	0.04946891	0.252779417	-3.956018299	0.595803318	2.357008827	-1.984049
Cdc25a	Cdc25a-003	699.33005	0.013880055	0.035644588	0.252815099	-3.955459947	2.164693532	8.562358563	-1.983845
Degs2	Degs2-202	1376.0251	0.01266084	0.033144828	0.252816321	-3.955440831	3.893552093	15.40071493	-1.983838
Cdc26	Cdc26-003	2315.2697	6.57247E-05	0.000589775	0.252826605	-3.955279939	5.738780334	22.69848273	-1.98378
Gm38198	Gm38198-001	1142	1.15655E-05	0.000170994	0.252838986	-3.955086257	2.498958149	9.883595034	-1.983709
Hn1l	Hn1l-201	4240	0.000150474	0.001082036	0.252879623	-3.954450699	12.82885559	50.73107697	-1.983477
30034C11F	0034C11Rik-	1033.3242	4.85737E-05	0.000471156	0.25288902	-3.954303749	2.598888069	10.27679284	-1.983424
Dus4l	Dus4l-201	632	0.00571805	0.017763557	0.252890238	-3.954284708	1.872082381	7.402746729	-1.983417
Prickle2	Prickle2-001	1599.5623	0.001403595	0.005944495	0.252933768	-3.95360417	2.366673835	9.356891545	-1.983168
Trmt1l	Trmt1l-007	2777.2766	6.81117E-05	0.000606052	0.252938193	-3.953534999	6.998063332	27.66708831	-1.983143
Ahdc1	Ahdc1-201	2396.5262	0.006337532	0.019230984	0.25298125	-3.952862118	4.503272282	17.80081441	-1.982898
Casp8ap2	Casp8ap2-001	1622.8942	0.001336337	0.005714765	0.252988552	-3.952748034	3.975496808	15.71413719	-1.982856
Morf4l1	Morf4l1-006	752.29697	0.000509735	0.002710933	0.253002722	-3.95252665	2.083240144	8.234062185	-1.982775
Kif1c	Kif1c-001	1827.4012	0.012124081	0.032060667	0.253084071	-3.951256179	3.413716423	13.48846811	-1.982311
Pde5a	Pde5a-001	1541.9133	0.003538783	0.012205773	0.253103251	-3.950956761	3.570270095	14.10598277	-1.982202
10037D02F	0037D02Rik-	3631.212	1.62779E-06	4.69688E-05	0.253110375	-3.950845554	8.007167129	31.63508065	-1.982161
Rbpms2	Rbpms2-201	217.72432	0.017206513	0.042158056	0.253119064	-3.950709933	0.657704364	2.598399165	-1.982112
Nsun5	Nsun5-008	419.22674	0.022129865	0.051382814	0.253142045	-3.950351269	0.991410774	3.916420811	-1.981981
Vopp1	Vopp1-003	551.55019	0.002244523	0.00857596	0.253168966	-3.949931211	1.08999651	4.305411237	-1.981828
Fem1b	Fem1b-201	11850	1.21044E-08	2.8812E-06	0.253205141	-3.949366891	28.84888549	113.9348332	-1.981621
P23-297M4	23-297M4.7-	724	0.00018446	0.001255533	0.253234139	-3.948914647	1.563594344	6.174500607	-1.981456
Lpcat2	Lpcat2-001	3191.3293	0.000482909	0.00260243	0.253269538	-3.948362718	9.236400241	36.46865837	-1.981255
Tle1	Tle1-005	2226.2371	0.004072999	0.013605713	0.253302599	-3.947847366	5.426054081	21.42123331	-1.981066
Nhlrc3	Nhlrc3-002	2867.8834	0.000992297	0.004523648	0.253307171	-3.94777612	6.338411995	25.02263151	-1.98104
Lca5	Lca5-001	646.88406	0.003733319	0.01272648	0.253491818	-3.944900495	1.629640979	6.428771503	-1.979989
Arhgef1	Arhgef1-006	4263.0979	0.001431757	0.006036892	0.25349337	-3.944876354	9.685617112	38.20856192	-1.97998
Map3k5	Map3k5-001	2348.6427	0.002302996	0.008748625	0.253498739	-3.944792804	5.154723172	20.33431487	-1.97995
Psmd11	Psmd11-001	5156.9003	3.21181E-05	0.00035134	0.253508251	-3.944644777	12.65629344	49.92458182	-1.979895
Poli	Poli-004	992.05259	0.000362741	0.002085684	0.253518021	-3.944492764	2.247249279	8.864258519	-1.97984
30089K02F	0089K02Rik-	1693	0.003536066	0.012198859	0.253521604	-3.944437014	6.429318464	25.36004173	-1.979819
Tmem18	Tmem18-201	3290	1.34586E-05	0.000190243	0.253541895	-3.944121351	7.168560752	28.27367352	-1.979704
Gm11714	Gm11714-001	1124	3.63709E-05	0.000382895	0.253558465	-3.943863598	2.429741322	9.582568354	-1.97961
Ifrd1	Ifrd1-001	2106.6932	0.001061121	0.00476698	0.253606157	-3.943121938	6.272700923	24.73402462	-1.979338
Mrpl20	Mrpl20-004	256.71666	0.011552999	0.030901227	0.253611815	-3.943033969	0.578231352	2.279985863	-1.979306
Hnrnpa1	Hnrnpa1-202	22539.238	0.000952822	0.00438572	0.253625915	-3.942814758	67.92414834	267.8123345	-1.979226
Myh9	Myh9-004	2124.0635	0.008022636	0.023179964	0.253658386	-3.942310026	6.333545437	24.96879968	-1.979041
Vdac2	Vdac2-001	7654.9677	0.000126649	0.000953977	0.253671523	-3.942105877	18.59919275	73.31998705	-1.978967
Sulf1	Sulf1-009	1582.5473	4.64961E-05	0.000456797	0.253795663	-3.94017766	3.007408384	11.84972333	-1.978261
Calm1	Calm1-001	90582.186	7.69178E-07	2.99411E-05	0.25379931	-3.940121032	169.4747131	667.7508816	-1.97824
Dusp16	Dusp16-002	1800.9432	0.006341736	0.019238658	0.253801276	-3.940090513	4.49736768	17.72003573	-1.978229
Cndp1	Cndp1-003	505	0.000545298	0.002860978	0.253812057	-3.939923148	1.253959944	4.940505809	-1.978167
Ylpm1	Ylpm1-005	374.8575	0.013794183	0.035477266	0.25382537	-3.939716511	1.013273132	3.992008887	-1.978092
Gm20707	Gm20707-001	792.87486	0.003598206	0.012368383	0.253834889	-3.939568761	2.050590872	8.078443742	-1.978038
Khdrbs1	Khdrbs1-002	7681.363	1.7394E-06	4.88315E-05	0.253902238	-3.938523765	21.47563768	84.58230936	-1.977655
Gm5113	Gm5113-001	2030	2.32183E-06	5.97816E-05	0.253929914	-3.938094502	4.681100915	18.43461778	-1.977498
Dpy19l4	Dpy19l4-001	4639.2463	6.76698E-07	2.75916E-05	0.253967177	-3.937516692	10.95394685	43.13134857	-1.977286
Pgm2l1	Pgm2l1-002	2443.4811	0.000333382	0.001958213	0.253967515	-3.937511453	7.088953496	27.91283558	-1.977284
Pak4	Pak4-201	7837.2708	1.10705E-05	0.000165791	0.253969566	-3.937479659	8.152780665	32.10140804	-1.977272
Snx14	Snx14-008	875.88643	0.000141792	0.001038724	0.253998015	-3.937038636	2.152874284	8.475949234	-1.977111

Cct8	Cct8-001	4934.4764	9.41723E-05	0.000764313	0.25400398	-3.936946186	12.83032826	50.51231192	-1.977077
Ggps1	Ggps1-201	5514.6333	4.60495E-07	2.21869E-05	0.254008856	-3.936870609	14.24550116	56.08269481	-1.977049
Armc10	Armc10-001	3489.2986	5.94026E-07	2.54955E-05	0.254009094	-3.936866929	7.748394413	30.50439772	-1.977048
Gabpb2	Gabpb2-004	6264.0934	1.52253E-05	0.00020759	0.254032511	-3.936504017	13.97932836	55.02968225	-1.976915
Gm15527	Gm15527-003	393.06923	0.022117431	0.051360906	0.254048465	-3.936256808	0.670864033	2.640693118	-1.976824
Map4k4	Map4k4-021	2767.1399	3.16767E-05	0.000347956	0.254060251	-3.936074198	8.130211093	32.00111411	-1.976757
Ankdd1b	Ankdd1b-003	440.62067	0.003149072	0.011164034	0.254079704	-3.935772854	0.875861174	3.447190634	-1.976647
Slc11a2	Slc11a2-002	1242.6194	0.000474178	0.002568224	0.254103653	-3.935401897	3.709890896	14.59991167	-1.976511
Slc25a14	Slc25a14-201	355.84278	0.011236746	0.030199195	0.254230388	-3.933440085	0.946896316	3.724559925	-1.975792
Dnajc9	Dnajc9-201	2264	9.18955E-05	0.000750406	0.254233179	-3.933396914	6.159961694	24.22957431	-1.975776
Psmd11	Psmd11-002	2241.5509	0.002630519	0.00970018	0.254286079	-3.932578636	6.412874209	25.21913211	-1.975476
Gm15910	Gm15910-001	4573	0.001168524	0.005149929	0.254291831	-3.932489676	10.67354871	41.97362012	-1.975443
P23-293K21	23-293K21.3-	2826	6.30759E-06	0.000113636	0.254323351	-3.93200229	7.036212875	27.66640514	-1.975264
Cdv3	Cdv3-006	665.14824	0.000311838	0.001859085	0.254342581	-3.931705013	1.629396574	6.406306677	-1.975155
Tmem41b	Tmem41b-007	411.12396	0.045814487	0.09151675	0.254388878	-3.930989471	1.098176656	4.316920873	-1.974892
Ncaph2	Ncaph2-012	181.12847	0.040116689	0.082314678	0.254389744	-3.930976081	0.359484307	1.413124211	-1.974888
Sfpq	Sfpq-003	1499.0595	8.35724E-05	0.000701168	0.254398319	-3.930843583	3.901227004	15.33511313	-1.974839
Pomp	Pomp-001	6942.9473	2.28661E-05	0.000277423	0.254417897	-3.930541104	17.70678042	69.59722827	-1.974728
Nt5dc1	Nt5dc1-001	647.12632	0.003482604	0.012053271	0.25447006	-3.929735388	1.762729129	6.927059038	-1.974432
Tnpo3	Tnpo3-002	816.93398	0.040256382	0.082537031	0.254482267	-3.929546891	0.769889866	3.025318329	-1.974363
Smim13	Smim13-001	3126	8.84822E-06	0.000142412	0.254496596	-3.929325647	8.553309591	33.60873874	-1.974282
Ndufaf7	Ndufaf7-201	1981	1.88961E-06	5.17341E-05	0.254555421	-3.92841762	4.361080503	17.13214549	-1.973948
Nmrk2	Nmrk2-201	308	0.002238819	0.008559897	0.254676389	-3.926551673	0.736535552	2.892044902	-1.973263
Emilin1	Emilin1-004	1530.615	0.00275515	0.010050474	0.254722814	-3.925836027	3.450474454	13.54599692	-1.973
Timeless	Timeless-012	329	0.006345324	0.019247679	0.254727289	-3.925767056	0.861795587	3.383208723	-1.972975
Gm37315	Gm37315-001	1928	6.5358E-06	0.000115985	0.254744821	-3.925496886	4.022881403	15.79180842	-1.972875
Rttn	Rttn-201	2509	6.07811E-06	0.000110555	0.254770012	-3.92510874	6.073502919	23.83915939	-1.972733
Bpnt1	Bpnt1-001	2856.5351	3.03896E-05	0.000337826	0.254770812	-3.925096413	7.33766791	28.80105399	-1.972728
Gm6410	Gm6410-201	1379	4.90115E-05	0.000473658	0.254777944	-3.924986539	3.08208127	12.09712749	-1.972688
Chic2	Chic2-001	6214.6833	7.97564E-07	3.06762E-05	0.254790457	-3.92479378	16.79296878	65.90893943	-1.972617
Rps12-ps10	ps12-ps10-00	313	0.004289844	0.014186267	0.254835315	-3.924102911	0.882282389	3.462166893	-1.972363
Tek	Tek-004	656.69052	0.009500945	0.02648172	0.254859725	-3.923727063	1.134590171	4.45182216	-1.972225
Pola2	Pola2-201	514.14079	0.026099879	0.058441477	0.25490343	-3.923054313	1.233933138	4.840786718	-1.971977
Rc3h1	Rc3h1-001	21876.203	1.53433E-08	3.19391E-06	0.254942566	-3.922452089	53.88615151	211.3658475	-1.971756
Ankhd1	Ankhd1-001	1869.8557	0.00899534	0.025365585	0.254996504	-3.921622397	4.084778475	16.01895875	-1.971451
Ywhab	Ywhab-001	45379.553	4.27675E-08	5.69956E-06	0.25503676	-3.921003393	97.91926017	383.9417514	-1.971223
Wipi2	Wipi2-004	712.40084	0.001634131	0.006676489	0.255059171	-3.920658865	1.691595194	6.632167691	-1.971096
Mrpl48	Mrpl48-001	1713.8952	0.000127959	0.000960255	0.255067908	-3.920524571	4.3154787	16.91894028	-1.971047
Akap13	Akap13-201	23144.801	4.36881E-07	2.14372E-05	0.255068614	-3.920513714	52.65880343	206.449561	-1.971043
Mat2a	Mat2a-201	25134	7.00968E-07	2.81865E-05	0.255092646	-3.920144375	71.41099471	279.9414092	-1.970907
P24-276A6	24-276A6.1-(	310	0.005834548	0.018043636	0.255117986	-3.919754999	0.556084888	2.179716519	-1.970763
Prmt1	Prmt1-002	502.76168	0.002133649	0.008230374	0.255126952	-3.919617246	1.412000967	5.534503341	-1.970713
30438D12R	0438D12Rik-	470.21609	0.007789385	0.022648794	0.255139657	-3.919422059	1.155703499	4.529689789	-1.970641
30031M04R	0031M04Rik	269	0.003751776	0.012769386	0.255208566	-3.918363779	0.690368088	2.70511331	-1.970251
Trib1	Trib1-001	4326.0273	0.000735101	0.003590078	0.255267966	-3.917451986	13.92415177	54.54719601	-1.969916
Cyb5r1	Cyb5r1-001	3523.442	6.57743E-05	0.000589775	0.255325695	-3.916566254	7.410648366	29.02429531	-1.969589
Eprs	Eprs-002	1911.7683	3.08883E-05	0.000341927	0.255330938	-3.916485829	5.102726487	19.98475598	-1.96956
Zdhhc2	Zdhhc2-001	2129.14	1.16985E-05	0.000172001	0.255331447	-3.916478015	5.83660446	22.85893305	-1.969557
Nktr	Nktr-003	2253.3772	0.000288667	0.001756722	0.255335077	-3.916422341	6.80297923	26.64333985	-1.969536
Mecp2	Mecp2-201	14785.794	2.12391E-05	0.000263948	0.255356251	-3.916097592	33.61135395	131.6253423	-1.969417
Fmr1	Fmr1-007	1872.7939	1.02819E-05	0.000157564	0.255365554	-3.91595493	4.36330571	17.08650851	-1.969364
Slc35a3	Slc35a3-007	276.01372	0.017061077	0.041882303	0.255387617	-3.915616636	0.572326566	2.241011423	-1.96924
33412K13R	3412K13Rik-	367	0.011489284	0.030764443	0.2553894	-3.915589286	1.1465708	4.489500342	-1.969229
Trnt1	Trnt1-002	355.45544	0.013611085	0.035103891	0.255396836	-3.915475282	1.088621405	4.262470201	-1.969187
Morc3	Morc3-201	2337.6762	2.33E-05	0.000281193	0.255440949	-3.914799104	5.995218938	23.47007773	-1.968938
Mall	Mall-001	30252	1.12129E-05	0.000167557	0.255463983	-3.914446137	55.34527002	216.6460785	-1.968808
Atr	Atr-010	272.71951	0.010841297	0.029355239	0.255472452	-3.914316363	0.694279403	2.717629226	-1.96876
Entpd6	Entpd6-003	223.32206	0.012399537	0.032612819	0.255515372	-3.913658858	0.631447807	2.471271302	-1.968518
Tbrg4	Tbrg4-002	1301.9541	6.00986E-06	0.000109663	0.255579961	-3.912669821	3.008464248	11.77112727	-1.968153
Rrp1b	Rrp1b-009	286.8563	0.04389337	0.088460837	0.255584648	-3.912598068	0.956721169	3.743265399	-1.968127
Soat1	Soat1-001	5378.2136	9.31384E-05	0.000757718	0.255656445	-3.911499279	16.74933196	65.51499989	-1.967722
Gm11033	Gm11033-001	3019	7.24588E-06	0.000124334	0.255669727	-3.911296083	7.410061069	28.98294283	-1.967647
P23-267O21	23-267O21.3-	1066	0.000143536	0.001046904	0.255733609	-3.910319035	2.578523355	10.08284896	-1.967286
Bscl2	Bscl2-007	526.49931	0.025895014	0.058073877	0.255756325	-3.909971729	1.445547551	5.652050057	-1.967158
Ccdc163	Ccdc163-007	308.92125	0.049765324	0.0976098	0.255758127	-3.909944183	0.755100054	2.952399066	-1.967148
Gm13982	Gm13982-001	117	0.030480575	0.066182305	0.255771999	-3.909732122	0.216198053	0.845276471	-1.96707
Dnajb14	Dnajb14-003	420.82626	0.01193015	0.031662738	0.255782679	-3.909568871	0.986368516	3.856275646	-1.96701
P23-171F16	23-171F16.5-(	675.77303	0.000966938	0.004431344	0.255832986	-3.908800089	1.328949277	5.194596858	-1.966726
Ctnnd1	Ctnnd1-018	2417.6431	0.003184769	0.011264933	0.255837987	-3.908723694	5.575100629	21.79152793	-1.966698
Mapkapk3	Mapkapk3-00	1505.3505	0.011738835	0.031283466	0.255838045	-3.908722809	3.862619727	15.09790983	-1.966697
00028B04R	0028B04Rik-	152	0.047906305	0.094848891	0.25586315	-3.908339284	0.449006952	1.754871509	-1.966556
Zfp442	Zfp442-001	755.47403	0.002237973	0.008558572	0.255931444	-3.907296359	1.672796724	6.536112549	-1.966171
Gm26610	Gm26610-201	250	0.008094017	0.023331095	0.255931633	-3.907293469	0.646641097	2.526616534	-1.96617
Ebag9	Ebag9-201	2116	1.13809E-05	0.000169255	0.255954756	-3.906940484	5.560061026	21.72282752	-1.966039
RP23-407I6.	23-407I6.6-0	854	0.006438061	0.019482556	0.255968572	-3.906729612	2.394362061	9.354125165	-1.965961
Hmgcr	Hmgcr-001	1798.7463	0.000208519	0.001375003	0.25597249	-3.906669809	4.496050775	17.56458582	-1.965939
Vps9d1	Vps9d1-008	1062.365	0.00073971	0.003607955	0.256000372	-3.906244331	2.377011669	9.285188356	-1.965782
Inip	Inip-002	593.25579	0.007844112	0.022775588	0.256013842	-3.906038793	1.716381134	6.704251292	-1.965706
Sf3a1	Sf3a1-004	344.87651	0.008334483	0.0238536	0.256022907	-3.905900501	0.663993357	2.593491985	-1.965655
Yae1d1	Yae1d1-201	3977	3.41992E-06	7.66571E-05	0.256024864	-3.905870636	10.82727124	42.2899208	-1.965644
Ttc17	Ttc17-004	814.34351	0.000226941	0.001466275	0.256036099	-3.905699248	2.215655316	8.653683302	-1.965581
Rpl15	Rpl15-002	5908.2793	0.001898314	0.007513917	0.256066564	-3.905234581	17.93060514	70.02321926	-1.965409
30550D23R	0550D23Rik-	237.68902	0.011914254	0.031635258	0.256142739	-3.904073182	0.380069348	1.483818548	-1.96498
Uba5	Uba5-001	4503.7962	1.5656E-05	0.000211276	0.256165767	-3.903722228	11.36163946	44.35268451	-1.96485
Gas5	Gas5-012	1244.2257	0.017867123	0.043437605	0.256179341	-3.903515392	4.097323847	15.9939667	-1.964774
Gm38356	Gm38356-001	11202	3.38612E-06	7.59992E-05	0.256182629	-3.903465282	27.0122598	105.4414183	-1.964755
Wdr11	Wdr11-006	1521.6834	0.000207722	0.001370542	0.256276108	-3.90204147	2.809425607	10.96249523	-1.964229
Mynn	Mynn-004	1309.5359	0.043979256	0.088589632	0.256303969	-3.901617307	2.669959359	10.41715964	-1.964072
Gm17491	Gm17491-201	1524	0.000723307	0.003548671	0.256326482	-3.901274618	3.698218775	14.42776704	-1.963946
Map1b	Map1b-201	34336	0.00077317	0.00373078	0.256326703	-3.901271264	99.5906457	388.5301243	-1.963944
Car8	Car8-001	56973	2.23398E-06	5.85865E-05	0.256368938	-3.900628549	105.03473	409.7014664	-1.963707
Ints8	Ints8-006	992.05043	0.005505596	0.017269592	0.256376135	-3.900519061	2.837021465	11.0658563	-1.963666
Ccser2	Ccser2-001	20150.955	7.42673E-07	2.92374E-05	0.256401056	-3.900139949	40.89596314	159.4999796	-1.963526
Psmd10	Psmd10-001	933.59068	0.008419954	0.024045979	0.256406214	-3.900061486	2.388007021	9.31337421	-1.963497
Tardbp	Tardbp-006	5963.1183	0.034924235	0.073799367	0.256441633	-3.899522825	18.10307352	70.59334838	-1.963298
Taf9b	Taf9b-002	4009.6848	0.00074926	0.003641056	0.256447896	-3.899427578	7.149718121	27.87980802	-1.963262
Kantr	Kantr-004	255.03086	0.018042611	0.043758635	0.256454481	-3.899327466	0.565783488	2.206175095	-1.963225
Cep350	Cep350-004	1951.6781	5.16969E-05	0.000493072	0.256470378	-3.89908577	5.083216816	19.81989835	-1.963136
Sp100	Sp100-004	3077.5773	0.00021594	0.00141225	0.256475284	-3.899011177	6.727498627	26.23059234	-1.963108
Ppa2	Ppa2-007	680	0.001845061	0.007346354	0.256556391	-3.897778563	1.79284377	6.988108013	-1.962652
Mcc	Mcc-003	1456.1425	0.001408868	0.005960196	0.256612064	-3.896932928	3.699505752	14.41672578	-1.962339
Rap2b	Rap2b-001	3576.3148	9.05593E-05	0.0007422	0.256766336	-3.894591542	9.338587841	36.36998522	-1.961472
Suv420h1	Suv420h1-007	2095.1376	0.000875465	0.004114255	0.256809161	-3.893942091	4.732571639	18.4283599	-1.961231
Ptpra	Ptpra-002	4391.1581	0.007392909	0.021756928	0.256827607	-3.893662407	10.73829438	41.81129315	-1.961128
Gm21769	Gm21769-201	11276	3.00053E-07	1.72719E-05	0.256848109	-3.893351609	27.48701432	107.0166114	-1.961013
Pik3cb	Pik3cb-001	1478.3956	0.015538962	0.038924117	0.256853536	-3.893269353	3.664402338	14.26650532	-1.960982
Gm9431	Gm9431-001	137	0.019454612	0.046478827	0.25687948	-3.892876147	0.372464965	1.44995998	-1.960836

Ttyh3	Ttyh3-005	590.66863	0.048928561	0.096426907	0.256913626	-3.892358752	1.360174825	5.294288385	-1.960645
Dctn3	Dctn3-001	2645.9501	0.000307507	0.001843214	0.256967019	-3.891549989	6.005255368	23.36975146	-1.960345
Stat2	Stat2-202	2212.6299	0.002129661	0.008216839	0.257015068	-3.890822465	4.662936903	18.14265966	-1.960075
RP24-333I1.	P24-333I1.5-0	476	0.001631226	0.006667801	0.25704438	-3.890378779	1.148109085	4.46657922	-1.959911
Pigc	Pigc-002	1015.7181	0.03780035	0.078566964	0.257052187	-3.890260618	2.228700814	8.670227007	-1.959867
Tmem131	Tmem131-201	16275.746	6.29873E-07	2.64053E-05	0.257111501	-3.889363159	41.12325927	159.9432896	-1.959534
Mtcp1	Mtcp1-003	2248.8929	0.00286552	0.010377927	0.257136655	-3.888982691	6.498739256	25.27348448	-1.959393
Ttc19	Ttc19-003	3100.3023	7.42994E-06	0.000126416	0.257225304	-3.887642414	7.164633293	27.85353227	-1.958896
Crot	Crot-001	3251.2911	0.000477658	0.002582038	0.25736124	-3.885588988	7.451339347	28.95284211	-1.958133
Pik3c2b	Pik3c2b-004	1946.9362	0.01374596	0.035390449	0.257361879	-3.88557934	2.475075133	9.617100802	-1.95813
Gon4l	Gon4l-002	2692.6866	1.76889E-05	0.000231239	0.257371357	-3.88543625	7.986142062	31.02964587	-1.958077
Amd-ps4	Amd-ps4-001	167	0.008639092	0.024538804	0.257383737	-3.885249368	0.436274863	1.695036635	-1.958007
Gm29480	Gm29480-001	604	0.000407185	0.002281008	0.257384132	-3.885243398	1.417185752	5.506111589	-1.958005
Igtp	Igtp-003	2286.7365	0.014752257	0.037407426	0.257392868	-3.885111537	3.697386447	14.36475874	-1.957956
Pafah1b1	Pafah1b1-004	4684.5135	0.000109312	0.000852444	0.257489917	-3.883647221	10.73918585	41.70720929	-1.957412
Odf2	Odf2-002	1531.9231	0.006797367	0.020336288	0.257498364	-3.883519818	4.220145595	16.38901905	-1.957365
P24-567O8	24-567O8.3-(	1952	0.000378327	0.002156739	0.257521503	-3.883170879	4.747153601	18.43400862	-1.957235
D6Wsu163e	6Wsu163e-0(	884.48322	0.000303116	0.001823179	0.257531948	-3.883013387	2.028611597	7.877125989	-1.957177
Gm37733	Gm37733-001	1408	7.87315E-06	0.000131475	0.257547814	-3.882774173	3.25087747	12.62242308	-1.957088
Paxbp1	Paxbp1-005	314.75309	0.002452137	0.009184787	0.257559241	-3.882601902	0.828340022	3.216114543	-1.957024
Ets2	Ets2-001	4844.8765	1.51261E-05	0.00020674	0.257627228	-3.881577305	11.49148367	44.6050822	-1.956643
Gm11033	Gm11033-003	247	0.006939727	0.020684506	0.257628153	-3.881563357	0.57608708	2.236118502	-1.956638
Xrn1	Xrn1-006	1160.8044	0.003827916	0.012965399	0.257650561	-3.881225776	2.943891199	11.4259064	-1.956512
Hadhb	Hadhb-002	1542.1912	0.012297675	0.03240463	0.257718001	-3.880210138	3.799303549	14.74209615	-1.956135
Adprm	Adprm-005	885.37659	0.000840872	0.00398173	0.257743612	-3.879824575	2.072791366	8.04206688	-1.955991
Rabgap1l	Rabgap1l-014	1099.7287	0.001300342	0.005599601	0.257758928	-3.879594035	2.784855775	10.80410985	-1.955906
Sestd1	Sestd1-001	9149.1514	4.65251E-06	9.31863E-05	0.257812733	-3.878784367	24.77273513	96.08809774	-1.955605
Jade1	Jade1-002	2356.4241	0.001983016	0.007783439	0.257830279	-3.878520413	4.801566105	18.62297215	-1.955506
Zfp799	Zfp799-201	2092.4978	4.14012E-05	0.00042052	0.257948834	-3.876737816	4.640423405	17.98970489	-1.954843
Plaa	Plaa-002	738.09142	0.003669215	0.012554037	0.257966187	-3.87647704	1.778838941	6.895628312	-1.954746
Stau2	Stau2-004	382.57253	0.04232487	0.085954746	0.257977136	-3.876312513	1.355353092	5.25377215	-1.954685
Prkdc	Prkdc-001	4151.392	1.80301E-05	0.000234002	0.257986748	-3.876168083	10.22108996	39.61866268	-1.954631
Zfp532	Zfp532-014	3047.7341	0.000253922	0.001592827	0.258015994	-3.875728722	7.186202826	27.85177269	-1.954468
Tpp2	Tpp2-002	2657.7243	0.017345687	0.042416805	0.258030204	-3.875515289	6.69885684	25.9615221	-1.954388
Pabpc4	Pabpc4-007	425.38554	0.018850411	0.045358057	0.258034858	-3.875445394	1.435703889	5.563992025	-1.954362
Tbl1x	Tbl1x-001	19687.024	2.26081E-08	3.80321E-06	0.258111016	-3.874301895	42.76973138	165.7028513	-1.953936
Mbd5	Mbd5-005	2328.7787	7.19283E-05	0.000630385	0.258157474	-3.873604689	6.35331649	24.61023655	-1.953677
Ier5	Ier5-001	4792	0.032168369	0.069067618	0.258157547	-3.873603585	13.45098071	52.10376708	-1.953676
Bcl2l2	Bcl2l2-001	4235.645	0.000599855	0.003075604	0.258189976	-3.87311706	9.248708773	35.82133173	-1.953495
Lsm14a	Lsm14a-001	18062.42	1.73913E-08	3.25493E-06	0.258250107	-3.872215241	44.03683393	170.5200995	-1.953159
Zmynd11	Zmynd11-008	4292.2641	0.000467736	0.002542022	0.258337788	-3.870901058	9.325279448	36.09723408	-1.952669
Ap1g1	Ap1g1-001	10353.188	7.41194E-07	2.92128E-05	0.25833841	-3.870891678	26.56483482	102.829598	-1.952666
Setd4	Setd4-003	1845.0277	4.01915E-05	0.00041165	0.258343461	-3.870815987	4.29577214	16.62814348	-1.952638
P24-500I24	24-500I24.4-(	1094	0.00016841	0.001173562	0.258345055	-3.870792105	2.682038406	10.38161309	-1.952629
Rab7	Rab7-005	1160.5914	0.004324821	0.014279874	0.258393438	-3.870067314	1.981629293	7.669038757	-1.952359
Cenph	Cenph-002	585.63439	0.002284946	0.008695487	0.258402622	-3.869929765	1.429703065	5.532850446	-1.952307
Rbm25	Rbm25-004	5989.7177	6.49818E-06	0.000115754	0.258420749	-3.869658312	15.05577671	58.26071147	-1.952206
P23-200E11	23-200E11.2-(	583	0.003557428	0.012263906	0.258438928	-3.869386117	1.442772019	5.58264202	-1.952105
Rbx1	Rbx1-201	4975	1.4986E-06	4.4805E-05	0.258465572	-3.868987247	12.46121185	48.21226974	-1.951956
P24-351O18	24-351O18.5-	2173	2.21391E-06	5.82686E-05	0.258472662	-3.868881117	5.548433925	21.46623124	-1.951916
Traf6	Traf6-001	7821.6768	1.21418E-07	1.06644E-05	0.258475169	-3.868843591	18.94079393	73.2789692	-1.951902
Slc25a20	Slc25a20-001	2732.8158	2.06724E-06	5.5436E-05	0.258500306	-3.868467373	7.374843971	28.52934328	-1.951762
Armcx2	Armcx2-006	1108.3139	0.031639508	0.068180014	0.258512049	-3.868291652	2.908321994	11.25023769	-1.951697
Rnf13	Rnf13-001	4630.112	2.02164E-05	0.000254469	0.258514553	-3.868254185	10.08400452	39.00749267	-1.951683
Ninl	Ninl-007	139.81849	0.023584579	0.053959375	0.258525373	-3.868092283	0.357974777	1.384679473	-1.951622
Fbxo9	Fbxo9-002	4193.0341	0.000574871	0.002978721	0.258557922	-3.867605342	9.360115462	36.20123256	-1.951441
Metap1d	Metap1d-002	1584.8429	0.000130957	0.000978121	0.258564425	-3.867508074	4.397658855	17.00798113	-1.951404
Rabif	Rabif-001	2849	5.67044E-06	0.000105896	0.258570684	-3.867414459	7.08727359	27.40942435	-1.951369
Zc3h7a	Zc3h7a-001	14319.375	3.04713E-08	4.57738E-06	0.258580916	-3.867261413	29.6642604	114.7194496	-1.951312
Il1r1	Il1r1-001	1249.2543	0.000915894	0.004256357	0.258581032	-3.867259691	3.838899969	14.84602311	-1.951312
P23-189N6	23-189N6.2-(	1583	3.33077E-05	0.000359983	0.258648799	-3.866246452	3.345276667	12.93366404	-1.950934
Dtymk	Dtymk-001	888.42585	0.01367984	0.035246171	0.258665332	-3.865999324	2.455975878	9.494801082	-1.950841
Suv39h1	Suv39h1-004	365.30528	0.045734387	0.091404724	0.25872027	-3.865178409	1.032714317	3.99162508	-1.950535
30408A02F	0408A02Rik-	768.96139	0.001136347	0.005037522	0.258743522	-3.864831053	1.801564272	6.962741541	-1.950405
Mier1	Mier1-005	1282.5527	0.00044348	0.00243651	0.258779587	-3.864292431	3.235037536	12.50113106	-1.950204
Trip11	Trip11-001	18167.284	2.13269E-07	1.47864E-05	0.258847662	-3.863276149	41.45868134	160.1663348	-1.949825
Il1r1	Il1r1-002	6706.344	0.000147475	0.001066294	0.258910797	-3.862334098	15.47957421	59.78728728	-1.949473
Malat1	Malat1-002	608.9686	0.010506838	0.028676324	0.25891184	-3.862318547	1.657386089	6.40135303	-1.949467
Ilf2	Ilf2-003	667.17102	0.009147381	0.025716074	0.258913054	-3.862300438	1.825165595	7.049337877	-1.94946
Gm26703	Gm26703-001	704	0.003598018	0.012368383	0.258921155	-3.862179586	1.766298554	6.821762216	-1.949415
Klhdc3	Klhdc3-202	788.83268	0.002593554	0.009590718	0.258926722	-3.862096557	1.666358662	6.435638053	-1.949384
Ubp1	Ubp1-201	3693.2751	0.000162785	0.001146494	0.258943182	-3.861851059	7.411945298	28.6238288	-1.949293
Pcdhgb1	Pcdhgb1-002	3466.5993	0.000144478	0.001051279	0.258948667	-3.861769259	8.247633489	31.85045747	-1.949262
Atr	Atr-001	3964.7868	0.000153828	0.001101301	0.258984774	-3.861230859	11.27066667	43.51864593	-1.949061
Birc3	Birc3-007	300	0.012712705	0.033247568	0.259010455	-3.860848008	1.040774998	4.018274078	-1.948918
Ttc19	Ttc19-002	536.78047	0.006493773	0.019600926	0.259092614	-3.85962373	1.422325613	5.489641689	-1.94846
Hdac7	Hdac7-006	2004.1107	0.039578794	0.081386509	0.259109896	-3.859366292	4.499050767	17.36348488	-1.948364
Gm32496	Gm32496-001	424	0.005825554	0.018023958	0.259114701	-3.859294737	1.137805493	4.39112675	-1.948337
Thap2	Thap2-201	1354	0.000349665	0.002030177	0.259161634	-3.858595833	3.859785917	14.89335385	-1.948076
AI662270	AI662270-001	309.66878	0.028674127	0.062950378	0.259187754	-3.858206968	1.125904057	4.343970878	-1.947931
P23-333I24	23-333I24.3-(	1469	0.00039787	0.002241288	0.259193333	-3.858123924	2.887277813	11.1394756	-1.947899
Klhdc7a	Klhdc7a-001	783	0.002880709	0.010423017	0.259253186	-3.857233213	1.654837964	6.383095957	-1.947566
Sec24d	Sec24d-008	514.39604	0.00423681	0.014051589	0.259254928	-3.857207291	1.288829003	4.971280628	-1.947557
Pptc7	Pptc7-001	6352.063	1.3249E-05	0.000188446	0.259364656	-3.855575444	14.09350447	54.33856974	-1.946946
Acaa1b	Acaa1b-001	221.71607	0.013382246	0.034644136	0.259378067	-3.855376102	0.490500905	1.891065466	-1.946872
Idnk	Idnk-202	1738.9837	0.002420259	0.009088071	0.259386918	-3.855244547	4.009794049	15.45873664	-1.946822
Psmb11	Psmb11-201	1352	2.7809E-05	0.000318888	0.25942957	-3.854610718	3.26192353	12.5734454	-1.946585
Sepsecs	Sepsecs-005	281.39501	0.025403574	0.057203971	0.259430415	-3.854598154	0.44570363	1.71800839	-1.94658
Adora3	Adora3-002	242.38733	0.004180688	0.013905853	0.25943284	-3.85456215	0.687346575	2.649420091	-1.946567
Gm20637	Gm20637-001	137	0.019619166	0.046816444	0.259496039	-3.853623362	0.350898831	1.352231933	-1.946216
Gm37874	Gm37874-001	569	0.028183981	0.062101218	0.259497703	-3.853598653	1.145357405	4.413747753	-1.946206
Gm16755	Gm16755-201	156.75897	0.022029192	0.051208079	0.259540786	-3.852958974	0.380115208	1.464568302	-1.945967
Cd84	Cd84-001	2132.2114	0.000592447	0.003044915	0.259582781	-3.852335639	6.512073922	25.08669445	-1.945733
Gm5834	Gm5834-001	2452	0.014866558	0.037644507	0.25959966	-3.852085165	7.420254879	28.58345374	-1.94564
32438A13F	2438A13Rik-	683.2224	0.002382312	0.008978232	0.259639672	-3.851491546	1.816448043	6.996034282	-1.945417
Zfp639	Zfp639-201	1291.9023	0.011557586	0.030905385	0.259666069	-3.851100013	2.81198405	10.82923181	-1.945271
Pdzd2	Pdzd2-006	1024.6639	0.000745587	0.003629384	0.259745165	-3.849927289	2.543671986	9.792952192	-1.944831
Arhgap44	Arhgap44-002	749.88482	0.003246898	0.011429216	0.259756412	-3.849760593	1.870424618	7.200686987	-1.944769
Gm12276	Gm12276-001	256	0.014492556	0.036885639	0.259762554	-3.849669571	0.621567133	2.392828079	-1.944735
Rpl22l1	Rpl22l1-002	560.30314	0.002236794	0.008555973	0.259791502	-3.849240605	1.545523618	5.949092266	-1.944574
Pkia	Pkia-001	4231.2783	0.000258533	0.001615616	0.259798804	-3.849132425	9.207882758	35.44236009	-1.944533
Gm28555	Gm28555-001	509.87826	0.009153137	0.02572794	0.259819536	-3.84882528	1.178515645	4.535900808	-1.944418
Pcdh7	Pcdh7-006	577.50753	0.016657036	0.041103068	0.25983463	-3.848601707	1.419426263	5.462806338	-1.944334
Scyl3	Scyl3-006	2361.5992	2.20595E-06	5.82079E-05	0.259843952	-3.84846363	5.797824994	22.31271862	-1.944283
Gm37375	Gm37375-001	975	0.000116796	0.000896517	0.259850979	-3.84835957	2.332440994	8.976071622	-1.944244
Ncoa2	Ncoa2-201	6190.1516	0.00016478	0.001157208	0.259899485	-3.847641336	15.10879432	58.13322156	-1.943974
Rassf4	Rassf4-201	1660	0.034345197	0.072841848	0.259918669	-3.847357347	7.356972342	28.30490159	-1.943868

Gm38235	Gm38235-001	1542	0.000352254	0.00204175	0.259950872	-3.846880735	3.912216631	15.04983079	-1.943689
Pigm	Pigm-001	6932	7.19839E-07	2.87014E-05	0.259993388	-3.846251652	17.20793748	66.18605797	-1.943453
Srpk2	Srpk2-002	620.14612	0.000834291	0.003955491	0.259998088	-3.846182127	1.23699861	4.757721944	-1.943427
Nbr1	Nbr1-006	4152.2449	0.00946051	0.026402283	0.260033145	-3.845663599	10.76881204	41.41322848	-1.943233
Impad1	Impad1-001	16929	1.53236E-07	1.21842E-05	0.260077379	-3.845009522	43.01111315	165.3781396	-1.942987
P23-344O1	23-344O11.3-	264	0.021201787	0.049720041	0.260154865	-3.843864304	0.635931902	2.444435938	-1.942557
Yars2	Yars2-001	461.05726	0.006242317	0.019036537	0.26016256	-3.84375061	1.046175127	4.021236282	-1.942515
Gm16287	Gm16287-001	447	0.001748945	0.007050537	0.260184045	-3.843433218	1.135512765	4.364267482	-1.942396
Ube2g1	Ube2g1-002	1472.5679	0.014725891	0.037367194	0.260186048	-3.84340362	3.132482684	12.03939529	-1.942384
Kdm2a	Kdm2a-003	630.00825	0.000953208	0.004386317	0.260188043	-3.843374156	1.531813253	5.887331467	-1.942373
Rnpc3	Rnpc3-005	736.44151	0.028221717	0.062172369	0.260198295	-3.843222722	1.859349911	7.145895827	-1.942317
Supt6	Supt6-002	1110.4641	0.000355428	0.002054235	0.260199269	-3.843208342	2.169377545	8.33736988	-1.942311
Gm8623	Gm8623-001	879	0.000175604	0.001209665	0.260247099	-3.842502003	2.570631166	9.877655405	-1.942046
Ddhd1	Ddhd1-011	422.04006	0.035764082	0.075148454	0.260291679	-3.841843899	1.618823561	6.219267423	-1.941799
Focad	Focad-001	923.00508	0.009124154	0.025665431	0.260292179	-3.841836529	2.246235619	8.629670051	-1.941796
Gm16249	Gm16249-001	1624	0.000345605	0.002011685	0.260292749	-3.841828114	3.698961242	14.21077329	-1.941793
Sri	Sri-006	3418.0838	0.000106142	0.000832425	0.260394204	-3.840331257	7.080972799	27.19328117	-1.941231
Setd2	Setd2-003	632.35441	0.004597104	0.014978199	0.260405678	-3.840162048	1.245087067	4.781336101	-1.941167
P23-44L13	23-44L13.1-0	567	0.007073641	0.021003226	0.260448141	-3.839535955	1.380730571	5.301364669	-1.940932
Hbp1	Hbp1-003	804.7892	0.006873357	0.020513462	0.260465017	-3.839287185	1.85202738	7.110464988	-1.940838
Tbcd	Tbcd-005	527.8624	0.032250382	0.069200325	0.260548797	-3.838052646	1.402756021	5.383851456	-1.940375
Gm12743	Gm12743-001	792.32523	0.005508408	0.017276828	0.260572415	-3.837704767	1.767774605	6.784197027	-1.940244
700020I14R	00020I14Rik-0	43306.53	1.59724E-08	3.19391E-06	0.260578346	-3.837617423	95.63533847	367.0118411	-1.940211
42621	Sept8-004	635.35303	0.020558086	0.048549573	0.260641275	-3.836690866	1.064210753	4.083047676	-1.939863
Dut	Dut-001	992.85486	0.020467768	0.048372968	0.26067451	-3.836201704	3.240916271	12.43280852	-1.939679
Gemin5	Gemin5-201	2094.814	0.00287385	0.010403695	0.260772093	-3.834766162	6.002680841	23.01887737	-1.939139
Synj1	Synj1-002	753.00957	0.008330896	0.023845329	0.260816258	-3.834116819	1.476931813	5.662729103	-1.938894
Srsf3	Srsf3-001	5232.1718	0.000606567	0.003103512	0.260844894	-3.833695897	14.21372719	54.49110762	-1.938736
Pcnp	Pcnp-007	10337.639	1.60241E-07	1.25679E-05	0.260861	-3.833459201	24.95274183	95.65531775	-1.938647
Cisd2	Cisd2-001	4302.3184	0.000130781	0.000977146	0.26086661	-3.833376753	12.0693904	46.26652057	-1.938616
Azin1	Azin1-001	7862.2885	0.00012628	0.000952451	0.260923248	-3.832544661	12.66437224	48.53677222	-1.938303
Rps25	Rps25-201	23137.152	1.811E-07	1.33171E-05	0.260936616	-3.832348314	65.99555467	252.9179527	-1.938229
Dsel	Dsel-002	1640.6031	0.005548928	0.017366685	0.260996035	-3.831475829	3.310419526	12.6837924	-1.9379
Cops7b	Cops7b-001	1519.1848	0.000719391	0.003534015	0.261038363	-3.830854548	3.713682876	14.22657894	-1.937666
Prr14	Prr14-009	380.80434	0.031839587	0.068524938	0.261044828	-3.830759674	0.983386419	3.767117037	-1.937631
Kcnn2	Kcnn2-005	138	0.03173721	0.068347549	0.261060233	-3.830533628	0.28425654	1.088854236	-1.937545
Selplg	Selplg-002	341.12297	0.011004462	0.029706361	0.261075415	-3.830310874	0.862363455	3.303120118	-1.937461
Rasgrp2	Rasgrp2-016	1054.277	6.25276E-05	0.000568055	0.261114449	-3.82973828	2.077495294	7.956263254	-1.937246
030533I22R	0533I22Rik-0	419	0.00108899	0.004865367	0.261187177	-3.828671878	1.117047151	4.276807015	-1.936844
Clk1	Clk1-010	1196.0038	0.001626249	0.00665222	0.261187415	-3.828668389	2.999344613	11.48349591	-1.936843
Nnat	Nnat-003	223.43422	0.014291103	0.036486788	0.261190832	-3.828618305	0.37556712	1.437903152	-1.936824
Prps2	Prps2-001	2172.9297	0.000154275	0.001103054	0.261204636	-3.828415972	5.730450524	21.93854831	-1.936748
Gje1	Gje1-001	399	0.003387926	0.01180072	0.261247806	-3.827783338	1.05497834	4.038228512	-1.936509
Casp4	Casp4-001	5444.3859	1.2817E-05	0.000183812	0.261319467	-3.826733658	14.25048012	54.53279192	-1.936113
Smap1	Smap1-002	366.80766	0.03671026	0.076773915	0.261339045	-3.826446977	0.811980402	3.106999953	-1.936005
Rpp30	Rpp30-201	958	0.001229568	0.005363263	0.261370896	-3.825980687	2.946356216	11.27270198	-1.93583
Gm16586	Gm16586-001	5980.1863	2.08728E-07	1.46469E-05	0.261374909	-3.825921944	15.80019531	60.45031397	-1.935807
Lzic	Lzic-001	1562.8798	0.000283169	0.001729413	0.26141144	-3.825387296	4.192706417	16.03872587	-1.935606
Dgat1	Dgat1-003	357.53785	0.035204846	0.074246673	0.261421407	-3.825241444	0.856052447	3.274607298	-1.935551
Chordc1	Chordc1-201	4935	8.60084E-07	3.20892E-05	0.261475293	-3.824453116	13.32549736	50.96273992	-1.935253
Fto	Fto-002	650.60591	0.016202206	0.040197433	0.261477901	-3.824414975	1.823090853	6.972255958	-1.935239
Cryzl1	Cryzl1-003	654.81612	0.008743641	0.02478028	0.261508206	-3.823971778	1.327630656	5.076822161	-1.935072
Parg	Parg-005	1214.2792	0.00573334	0.017800883	0.261532484	-3.823616808	3.232732583	12.36073064	-1.934938
Itgav	Itgav-007	585.26667	0.014782879	0.037463086	0.261549866	-3.82336269	1.712396495	6.547112868	-1.934842
Csnk2a1	Csnk2a1-002	1885.252	0.002686469	0.009856621	0.261577456	-3.822959427	4.212663309	16.10484091	-1.93469
Mterf1b	Mterf1b-001	271	0.025386025	0.057171971	0.261612359	-3.822449381	0.65372208	2.498819562	-1.934497
Fxyd5	Fxyd5-006	866.20603	0.005751615	0.01784032	0.2616525	-3.821862972	1.493753629	5.708921683	-1.934276
Rhobtb3	Rhobtb3-001	1391.1009	0.000168231	0.001173265	0.261660664	-3.821743728	4.027707292	15.39286508	-1.934231
D11Wsu47e	11Wsu47e-00	393.62099	0.039053426	0.080573953	0.261712983	-3.820979724	0.944791571	3.610029438	-1.933943
Gm37022	Gm37022-001	953	0.000138493	0.001021182	0.26174552	-3.820504734	2.264126355	8.650105461	-1.933763
Coa5	Coa5-001	8610.8764	2.4061E-07	1.55782E-05	0.261805727	-3.819626145	21.57875146	82.42276326	-1.933431
Kpna6	Kpna6-001	8698.2603	2.5495E-07	1.59928E-05	0.261886412	-3.81844935	21.64795168	82.66160702	-1.932987
Adam17	Adam17-007	6224.8168	0.00119	0.005223982	0.261903761	-3.818196417	13.81642555	52.75382652	-1.932891
Ube4b	Ube4b-001	11610.862	6.59463E-06	0.000116702	0.2619757	-3.817147928	29.54522995	112.7785133	-1.932495
Fgd4	Fgd4-003	1024.1251	0.000209031	0.001377058	0.262033815	-3.816301341	2.706948783	10.33053227	-1.932175
Fam120c	Fam120c-002	3443.9889	0.000133509	0.000992332	0.262038427	-3.816234175	6.686783325	25.51833105	-1.93215
Atrx	Atrx-022	2359.4137	0.001507197	0.006279225	0.262081534	-3.81560648	6.45086855	24.61397584	-1.931912
Sox6	Sox6-202	1349.4121	0.03086951	0.066882636	0.26209354	-3.8154317	2.817762418	10.75098005	-1.931846
Vkorc1	Vkorc1-001	3097.0614	6.88388E-05	0.00061071	0.262130254	-3.814897309	8.149941867	31.0911913	-1.931644
Six4	Six4-002	645.38632	0.000909544	0.004236046	0.262142359	-3.814721143	1.94784596	7.430489169	-1.931578
Memo1	Memo1-201	2324	3.58597E-05	0.000378984	0.262175437	-3.814239846	6.556751175	25.00902159	-1.931396
Psma1	Psma1-001	4648.9497	0.000190026	0.001281685	0.262177215	-3.81421399	12.49258367	47.64938742	-1.931386
Fam193a	Fam193a-002	2712.469	3.8988E-06	8.27862E-05	0.262225839	-3.813506729	7.018948407	26.76680698	-1.931118
Phldb2	Phldb2-004	10001.045	0.000160659	0.001135723	0.262260108	-3.813008423	25.67819091	97.91115824	-1.93093
230307C23F	0307C23Rik-	230.16688	0.019859249	0.047267498	0.262322446	-3.812102302	0.695120596	2.649870824	-1.930587
Rer1	Rer1-001	3401.586	0.001702598	0.006886534	0.262346927	-3.81174658	6.906878888	26.32727198	-1.930452
Plagl1	Plagl1-011	132.84252	0.037681424	0.078355217	0.262347675	-3.811735706	0.257919834	0.983122241	-1.930448
P23-477N9	23-477N9.1-0	352	0.00513705	0.016348632	0.262415363	-3.810752496	0.817649312	3.115859155	-1.930076
Rars	Rars-001	7742.9194	2.00647E-05	0.000253025	0.262430215	-3.810536833	23.19732397	88.39425741	-1.929994
Gm12070	Gm12070-001	1708	0.00088495	0.004148437	0.262461017	-3.81008964	4.810540802	18.32859167	-1.929825
Srprb	Srprb-002	1105.654	0.005517193	0.017293297	0.262558576	-3.80867391	2.593424505	9.877508249	-1.929289
Hnrnph3	Hnrnph3-002	2418.0542	0.00020349	0.0013499	0.262559602	-3.808659035	6.517138735	24.82155933	-1.929283
Gm26538	Gm26538-201	1203	7.03662E-05	0.000619867	0.262605328	-3.807995856	2.77944714	10.58412319	-1.929032
Plcb1	Plcb1-001	1836.3218	0.004056598	0.013566401	0.262611291	-3.807909383	4.427048691	16.85780025	-1.928999
Casp4	Casp4-006	596.35348	0.03896337	0.080420358	0.262659547	-3.807209796	1.43700805	5.470991126	-1.928734
Gm37581	Gm37581-001	328	0.022157414	0.051439804	0.262678117	-3.806940644	0.673535153	2.564108351	-1.928632
Phf3	Phf3-002	4700.5808	0.00051222	0.002722037	0.262691593	-3.806745352	10.75122802	40.9271873	-1.928558
Nfkb1	Nfkb1-008	925.75252	0.00085393	0.004030192	0.26278199	-3.805435832	2.788317119	10.61076188	-1.928062
Kctd11	Kctd11-001	3534	6.90729E-06	0.000120432	0.26280943	-3.805038497	9.303123269	35.39874218	-1.927911
Pclo	Pclo-001	1192.7434	0.013816934	0.035519778	0.262824433	-3.804821302	2.141316703	8.147327405	-1.927829
Arl5b	Arl5b-001	6002.9262	3.58465E-06	7.89037E-05	0.262831722	-3.804715776	14.42322824	54.87628405	-1.927789
Ep400	Ep400-002	11403.385	0.000145002	0.001054195	0.262849293	-3.804461439	28.97668733	110.2406896	-1.927692
Gm4070	Gm4070-001	25747.04	0.000120371	0.000917789	0.262914786	-3.803513742	60.43199192	229.8539117	-1.927333
Pnrc2	Pnrc2-001	9466.8489	4.97469E-07	2.3248E-05	0.26296846	-3.802737413	24.25594397	92.23898564	-1.927038
Gm17195	Gm17195-001	389	0.01047944	0.028631208	0.263046152	-3.801614245	1.052616875	4.001643307	-1.926612
Usp48	Usp48-008	257.19701	0.01298755	0.033824994	0.263073886	-3.80121347	0.533786259	2.029035516	-1.92646
RP23-23C4.	P23-23C4.4-0	1013	0.000345652	0.002011685	0.263124555	-3.800481482	2.000915571	7.604442572	-1.926182
Rbm18	Rbm18-001	3127.1197	9.75077E-06	0.000152078	0.263137997	-3.800287343	9.073340381	34.48130061	-1.926109
Nck1	Nck1-001	11229.596	1.36102E-05	0.000191949	0.263217066	-3.799145754	18.9698454	72.06920761	-1.925675
Lrch4	Lrch4-001	898.0123	0.022680039	0.052372664	0.263231103	-3.798943173	2.047727574	7.779200686	-1.925598
Plgrkt	Plgrkt-001	1416.9366	0.000173567	0.001199975	0.263270233	-3.798378532	3.817728004	14.50117609	-1.925384
Gm21710	Gm21710-001	2042	3.22785E-06	7.39401E-05	0.263279253	-3.798248397	4.36781094	16.5900309	-1.925334
Fgfr1	Fgfr1-015	3514.6774	0.000159979	0.001132548	0.26329091	-3.798080223	8.754800351	33.25143407	-1.92527
Ptk2b	Ptk2b-006	4728.1813	7.29867E-05	0.000637377	0.263354855	-3.797158014	11.46669162	43.54083997	-1.92492
Smim20	Smim20-201	688	0.004506131	0.01475055	0.26336543	-3.797005558	1.970379621	7.481542374	-1.924862
Naa25	Naa25-002	1420.8667	7.00292E-05	0.00061817	0.263379952	-3.79679619	3.375040102	12.8143394	-1.924783
Napg	Napg-001	3231.2238	1.132E-06	3.78994E-05	0.263389185	-3.796663102	8.068529664	30.63348886	-1.924732

Denr	Denr-005	444.13498	0.004219149	0.014005219	0.263398009	-3.796535918	0.945329801	3.588978544	-1.924684
Rb1cc1	Rb1cc1-003	7687.7531	0.000216649	0.001415806	0.263414807	-3.796293809	16.5969333	63.00683515	-1.924592
Rpl13a	Rpl13a-009	3041.7142	0.00188215	0.007459341	0.263415763	-3.796280022	8.307277889	31.53675309	-1.924586
P23-372C7.	23-372C7.5-(	2286	0.003495984	0.012091027	0.26343468	-3.796007417	4.806256285	18.24458451	-1.924483
Ankrd17	Ankrd17-008	1095.6787	0.000280351	0.001715871	0.263446404	-3.795838487	3.059897136	11.61487532	-1.924419
Pik3r3	Pik3r3-001	4590.0007	2.38984E-05	0.000285298	0.263481485	-3.795333094	8.313465098	31.55236921	-1.924227
Gm37371	Gm37371-001	1004	3.24978E-05	0.000354475	0.263534623	-3.794567817	2.455272533	9.316698134	-1.923936
Fryl	Fryl-003	1257.8286	0.000485944	0.002616091	0.26356522	-3.794127319	3.672130373	13.93253017	-1.923768
Nptx1	Nptx1-001	518	0.041190798	0.0840854	0.26358587	-3.793830066	1.635552581	6.205008558	-1.923655
Gm20712	Gm20712-001	837	0.021624312	0.050496592	0.263616185	-3.793393796	1.937119355	7.348256542	-1.923489
Nif3l1	Nif3l1-001	702.43994	0.024018244	0.054732194	0.263642575	-3.793014081	1.905379346	7.227130691	-1.923345
Bcl6	Bcl6-001	6742.8345	0.000128877	0.00096566	0.263685685	-3.792393965	18.18288831	68.95667588	-1.923109
P23-257E17	23-257E17.2-(	1042	0.000187863	0.001272609	0.263693301	-3.792284434	2.336136237	8.859293088	-1.923067
Trim12a	Trim12a-004	3762.7514	1.09606E-05	0.000164816	0.263778912	-3.79105362	9.398946718	35.63191098	-1.922599
Ube2d3	Ube2d3-001	3768.6103	6.74809E-05	0.000601567	0.263803254	-3.790703805	8.351224723	31.65701933	-1.922466
Gm26788	Gm26788-002	2078.9378	6.5049E-06	0.000115754	0.263850658	-3.790022757	5.370395221	20.3539201	-1.922207
Rpl31-ps12	pl31-ps12-00	3818	5.41669E-07	2.43727E-05	0.263866415	-3.789796433	10.77256384	40.82582403	-1.92212
Atp6v0e	Atp6v0e-202	1048.9478	0.000199318	0.001328913	0.263902758	-3.789274539	2.636723999	9.991271116	-1.921922
Bvht	Bvht-001	1156.1688	0.00105844	0.004758048	0.263945746	-3.788657386	2.404832769	9.111087431	-1.921687
Cdh11	Cdh11-201	570	0.003667093	0.012549681	0.263970588	-3.788300846	1.768861114	6.700978055	-1.921551
Gm9924	Gm9924-201	701.80188	0.000213657	0.001400331	0.264025544	-3.78751232	1.652823666	6.260089999	-1.921251
Gm9924	Gm9924-001	732.19805	0.000192873	0.001296564	0.264034372	-3.787385687	1.724451796	6.531164053	-1.921202
AI314180	AI314180-007	238.28342	0.029945146	0.065226485	0.264101548	-3.786422338	0.586959722	2.222477403	-1.920835
Dleu2	Dleu2-005	2576.1642	0.006544412	0.019725963	0.264135797	-3.785931375	7.077132811	26.79353915	-1.920648
Rnf150	Rnf150-201	814	0.014120421	0.036145323	0.264138998	-3.785885495	3.051436914	11.55239075	-1.920631
Scd3	Scd3-201	995	0.000101544	0.000806561	0.264183518	-3.785247489	2.562821397	9.700913258	-1.920388
Snx27	Snx27-001	5119.3566	1.29261E-06	4.11068E-05	0.264289165	-3.783734381	11.5947484	43.87144815	-1.919811
Gm6493	Gm6493-001	313	0.004029173	0.013496252	0.264325483	-3.783214498	0.831474414	3.145646059	-1.919613
Anapc4	Anapc4-001	1501.542	5.94197E-05	0.000546951	0.26432882	-3.783166738	3.610539011	13.65927109	-1.919594
Apbb2	Apbb2-010	4972.9127	1.50842E-06	4.49562E-05	0.264468969	-3.781161941	11.55893403	43.70620144	-1.91883
Gm38292	Gm38292-001	1580	0.000178212	0.001223933	0.264483596	-3.780952822	4.096926155	15.49028451	-1.91875
Gm6158	Gm6158-201	750	0.000154637	0.001104778	0.264538066	-3.780174312	1.969571828	7.44532483	-1.918453
Vps53	Vps53-006	644.64415	0.003047737	0.010889843	0.264550797	-3.779992395	1.480866619	5.597664557	-1.918383
Kcnj2	Kcnj2-001	4074	2.73903E-06	6.63998E-05	0.264590906	-3.779419387	7.309802848	27.6268106	-1.918165
Samhd1	Samhd1-002	2713.29	4.85449E-05	0.0004711	0.264624052	-3.778945983	7.821641384	29.55756029	-1.917984
700007J10R	00007J10Rik-(	206.14021	0.033542862	0.071467685	0.264701194	-3.777844692	0.551190966	2.082313866	-1.917563
Sirt1	Sirt1-003	2085.8275	2.88456E-05	0.000326079	0.264724989	-3.777505116	6.011301163	22.7077209	-1.917434
Setd5	Setd5-005	1347.8265	0.005066733	0.016163897	0.264747445	-3.777184705	3.18248706	12.02084144	-1.917311
Agk	Agk-003	547.03903	0.0102691	0.028168884	0.2647656	-3.776925707	1.400483696	5.289522873	-1.917212
Ehbp1l1	Ehbp1l1-202	2872.0733	0.017907448	0.043520193	0.26476932	-3.776872638	5.514890707	20.82903981	-1.917192
Gm36527	Gm36527-001	627	0.007755285	0.022566379	0.264795234	-3.776503018	1.433735172	5.414505206	-1.917051
Bdp1	Bdp1-005	398.40875	0.013711801	0.03531312	0.264940249	-3.774435943	0.831864268	3.139818394	-1.916261
P23-218L17	23-218L17.2-(	1308	0.000108341	0.000845639	0.264941438	-3.774419005	2.95958489	11.17071346	-1.916255
Ptpre	Ptpre-201	7151	0.021534283	0.050345856	0.264955816	-3.774214327	23.87744703	90.11860267	-1.916176
Ift80	Ift80-004	1927.7344	4.60536E-05	0.000454171	0.264955838	-3.77421387	4.703123333	17.75059332	-1.916176
Fam129a	Fam129a-001	16907.74	8.05229E-07	3.07178E-05	0.265022759	-3.773260843	39.85645392	150.3887969	-1.915812
Naip6	Naip6-001	471.34175	0.006130246	0.018781727	0.265038292	-3.773039713	1.647412434	6.215752537	-1.915727
Raly	Raly-005	832.654	0.00090428	0.004220137	0.26513775	-3.771624366	2.203908005	8.312313133	-1.915186
Immt	Immt-005	2743.3063	0.006112394	0.018738765	0.265148296	-3.771474364	6.726910197	25.37036936	-1.915129
Sorbs2	Sorbs2-203	319.02043	0.016106969	0.04000462	0.265153401	-3.771401745	0.98431961	3.712264696	-1.915101
Vamp2	Vamp2-002	2202.4464	0.000111651	0.000866276	0.265167059	-3.771207495	4.944683705	18.64742825	-1.915027
720464F23R	0464F23Rik-	871	0.001154556	0.005101735	0.265246783	-3.770073997	2.482828343	9.360446577	-1.914593
Mdm2	Mdm2-003	7861.6619	8.25115E-07	3.12267E-05	0.265286882	-3.769504142	21.58018507	81.346597	-1.914375
P23-453M1	23-453M11.4-	406	0.007780616	0.022633437	0.265309457	-3.769183396	0.916797096	3.455576392	-1.914252
Racgap1	Racgap1-003	1195.1066	0.000195519	0.001310222	0.265340826	-3.768737792	2.876784947	10.84184815	-1.914081
Gkn3	Gkn3-001	5219	0.021974459	0.051131175	0.26535399	-3.768550835	11.22340783	42.29598293	-1.91401
Arhgap20	Arhgap20-005	1966.1439	0.000687511	0.003415617	0.265366175	-3.768377786	4.633571926	17.46104952	-1.913944
Prosc	Prosc-202	1405.2917	5.62186E-05	0.000524513	0.265382062	-3.768152191	3.571760612	13.45893758	-1.913857
30402H24R	0402H24Rik-	9792.7126	1.33958E-06	4.19154E-05	0.265400652	-3.767888264	22.69227939	85.5019732	-1.913756
Abcb9	Abcb9-002	678.66378	0.000157683	0.001120464	0.265416065	-3.767669448	1.660353497	6.255663143	-1.913672
Slc18a2	Slc18a2-201	1345	7.40447E-05	0.000644317	0.265416835	-3.767658519	3.683341703	13.87757375	-1.913668
Gtpbp2	Gtpbp2-002	3047.4503	0.030358491	0.065971595	0.265543544	-3.765860714	6.725641141	25.32782775	-1.91298
Klhdc8b	Klhdc8b-007	915.10244	6.92938E-05	0.000613123	0.265581241	-3.765326176	2.204577019	8.300951556	-1.912775
Fam204a	Fam204a-201	2792.7713	3.27278E-05	0.000356173	0.265592417	-3.765167733	6.607302998	24.87760405	-1.912714
Gm14305	Gm14305-001	385.13066	0.001460611	0.006132319	0.265606155	-3.764972991	0.970125736	3.652497196	-1.91264
Col4a3bp	Col4a3bp-201	8813.5967	0.000262854	0.001634192	0.26560664	-3.764966119	17.41812622	65.57865505	-1.912637
Cep290	Cep290-201	3917.2677	4.05472E-05	0.000414179	0.265612199	-3.764887316	10.3606444	39.00665869	-1.912607
Tuba1c	Tuba1c-201	13786	0.00490741	0.015770201	0.26564751	-3.764386869	44.14278687	166.1705273	-1.912415
Eprs	Eprs-004	2954.5903	4.88326E-07	2.3133E-05	0.265677478	-3.76396226	8.048489274	30.29420988	-1.912252
Exosc1	Exosc1-201	1621.4878	0.000309419	0.001851502	0.265689019	-3.763798759	4.91685071	18.5060366	-1.912189
Hadhb	Hadhb-001	3127.9285	0.000780315	0.003753894	0.265701305	-3.763624722	7.142221823	26.88064263	-1.912123
Psip1	Psip1-001	7295.7626	2.49945E-06	6.27005E-05	0.265748841	-3.762951495	18.49137012	69.58212885	-1.911865
30014A01F	0014A01Rik-	712	0.003905448	0.01317459	0.265754666	-3.762869016	1.686925879	6.347681124	-1.911833
Tmem175	Tmem175-00(	237.64273	0.018018846	0.043719572	0.26578264	-3.76247298	0.44399801	1.670530514	-1.911681
Vps37a	Vps37a-201	9059	4.37536E-07	2.14386E-05	0.265895642	-3.760873972	23.35200624	87.82395245	-1.911068
Ephb4	Ephb4-201	3944.1551	0.007658117	0.022360656	0.266007175	-3.759297094	6.912648201	25.9866983	-1.910463
Ddhd1	Ddhd1-007	194.58148	0.015510547	0.038884223	0.266015434	-3.759180373	0.578782974	2.175749596	-1.910418
Usp38	Usp38-201	4405.8109	1.64035E-06	4.7172E-05	0.266028845	-3.758990877	11.07980451	41.64888408	-1.910345
Txndc11	Txndc11-002	1549.3056	0.001346073	0.005746355	0.266038652	-3.758852301	4.298303506	16.15668803	-1.910292
Rps13-ps2	Rps13-ps2-00	3254	2.33718E-06	5.9827E-05	0.266059474	-3.75855813	9.572090154	35.97725727	-1.910179
Rcn2	Rcn2-001	11287.445	8.21303E-08	8.50945E-06	0.266109483	-3.757851805	25.98253426	97.63851324	-1.909908
Gm37063	Gm37063-001	197	0.027909492	0.061639161	0.266116065	-3.757758857	0.388232249	1.458883174	-1.909872
30116N13R	0116N13Rik-	365.85667	0.001663946	0.00677092	0.266118235	-3.75772822	0.879722116	3.305756621	-1.909861
Gm38340	Gm38340-001	876	0.000601551	0.003081532	0.266246945	-3.755911638	2.208234634	8.293934162	-1.909163
Gm16222	Gm16222-001	1002	0.000110366	0.00085851	0.266277693	-3.755477939	2.492686664	9.361229777	-1.908997
31406C07R	1406C07Rik-	1536.3825	0.003189408	0.011279013	0.266312089	-3.754992886	3.96174509	14.87632463	-1.90881
Zfp827	Zfp827-002	271.5845	0.012400942	0.032614012	0.266358457	-3.754339219	0.724010712	2.718181809	-1.908559
Lyrm4	Lyrm4-201	1287	0.000154321	0.001103054	0.26637718	-3.754075332	3.322038184	12.4711816	-1.908458
Gm8388	Gm8388-001	124	0.020000368	0.047507636	0.26640738	-3.753649773	0.267006028	1.002247115	-1.908294
Slc4a7	Slc4a7-201	4605	4.98409E-05	0.000479643	0.266418705	-3.753490202	13.46966795	50.55826668	-1.908233
Dnase1	Dnase1-002	482.84607	0.004276643	0.014159015	0.266430892	-3.753318517	1.253410674	4.704449493	-1.908167
Usp33	Usp33-002	3208.7295	0.004748876	0.015354386	0.266509561	-3.752210598	7.615609171	28.57536944	-1.907741
Ctf2	Ctf2-001	762	0.002636038	0.009716349	0.266601635	-3.750914725	1.767499974	6.629741676	-1.907242
Gm3608	Gm3608-201	4680	1.36151E-05	0.000191949	0.266621801	-3.750631035	12.71027265	47.67154306	-1.907133
Sparc	Sparc-005	492.24979	0.040238689	0.082515574	0.266633826	-3.750461878	0.730814012	2.740890093	-1.907068
Papolb	Papolb-001	266	0.040103553	0.082292653	0.2667464	-3.748879083	0.582561366	2.183952121	-1.906459
Pafah1b2	Pafah1b2-201	19976	2.5334E-07	1.59209E-05	0.266760923	-3.748674982	46.24139316	173.3439537	-1.906381
Huwe1	Huwe1-013	305.71678	0.040999873	0.083775543	0.266773111	-3.748503721	0.587582971	2.202556951	-1.906315
Phax	Phax-001	2582.7608	8.22486E-05	0.000693675	0.266776999	-3.748449089	6.546479296	24.53914436	-1.906294
Kin	Kin-001	2416.6063	7.3745E-05	0.000642525	0.266876884	-3.747046142	6.189285803	23.19153949	-1.905754
Gm8989	Gm8989-001	2334	0.000102341	0.000811195	0.266887032	-3.746903675	5.280262858	19.78463631	-1.905699
Mpv17	Mpv17-001	4341.1128	9.54996E-06	0.000149923	0.266924744	-3.746374303	10.22658032	38.3125977	-1.905495
P23-180G16	23-180G16.2-	324	0.001411174	0.005969213	0.266925437	-3.746364567	0.891238523	3.338904422	-1.905491
Phf14	Phf14-002	1866.6117	0.021369293	0.050030643	0.266981462	-3.745578416	4.593626479	17.20578819	-1.905189
Bsdc1	Bsdc1-008	1543.93	0.005634217	0.017570055	0.266999735	-3.745322066	3.231757475	12.10397258	-1.90509
Usp24	Usp24-001	31718.071	9.80624E-07	3.44358E-05	0.26701798	-3.745066149	72.21597593	270.4536069	-1.904991
Zkscan3	Zkscan3-004	7995.4738	4.97137E-07	2.3248E-05	0.267039873	-3.744759117	17.63858031	66.05223441	-1.904873

Trerf1	Trerf1-001	6427.4465	0.000847944	0.00400691	0.267128006	-3.743523615	11.57242988	43.32166453	-1.904397
Zfp664	Zfp664-006	636	0.001126804	0.005004705	0.267150768	-3.743204659	1.575114744	5.895976846	-1.904274
Appl1	Appl1-002	1207.8563	1.94478E-05	0.000247248	0.267167207	-3.742974338	2.835614911	10.61363384	-1.904185
Dad1	Dad1-002	824.43185	0.036545594	0.076499609	0.267182581	-3.742758964	2.246151235	8.406802669	-1.904102
Mfsd2b	Mfsd2b-006	282.20868	0.006275919	0.019103291	0.267190434	-3.742648961	0.63470125	2.375463973	-1.90406
Cenpo	Cenpo-001	854.1478	0.005624777	0.017545411	0.267205631	-3.742436106	2.142261025	8.017275009	-1.903978
Vamp1	Vamp1-005	818.16764	6.55015E-05	0.000588671	0.267206348	-3.742426054	2.207748106	8.262334035	-1.903974
Trim34a	Trim34a-001	1136.9698	0.046906404	0.093262761	0.267241835	-3.741929104	2.53397636	9.48195989	-1.903782
Kalrn	Kalrn-010	2624.5531	0.001153567	0.005098681	0.267312093	-3.740945604	5.016006054	18.76460579	-1.903403
Uspl1	Uspl1-011	657.84216	0.000365656	0.002096721	0.267316132	-3.740889081	1.643298536	6.14739755	-1.903381
Slc7a7	Slc7a7-002	569.89267	0.00667827	0.020046517	0.26732963	-3.740700202	1.228271647	4.594595997	-1.903308
Gm37701	Gm37701-001	17847	1.28486E-07	1.09569E-05	0.267339664	-3.740559804	44.3145974	165.7614018	-1.903254
Zfp654	Zfp654-201	3442	1.51834E-06	4.50505E-05	0.267376949	-3.740038183	8.641461255	32.31939505	-1.903053
Gpr107	Gpr107-009	1106.9371	0.000136064	0.001006739	0.267470718	-3.738727019	2.168587943	8.107758336	-1.902547
Schip1	Schip1-006	1080.0246	0.001790743	0.007177643	0.267481461	-3.738576865	2.972516451	11.11298123	-1.902489
Slc2a1	Slc2a1-004	2966.4874	0.005161848	0.016413823	0.267504549	-3.738254189	8.8176194	32.96250266	-1.902365
Casc3	Casc3-001	2224.9292	0.010940815	0.029570937	0.267556543	-3.737527738	4.033973403	15.07708749	-1.902084
Nup35	Nup35-005	225.22379	0.041541478	0.084670055	0.267589449	-3.737068117	0.634233177	2.370172586	-1.901907
Acat2	Acat2-001	1438.1043	0.000601154	0.003080396	0.267680486	-3.735797164	4.914997856	18.36143506	-1.901416
Trmt2a	Trmt2a-007	364.61954	0.009355257	0.026160806	0.267708937	-3.73540013	1.144716828	4.275975388	-1.901263
Frrs1	Frrs1-001	14095.974	3.53876E-06	7.81447E-05	0.267775588	-3.734470373	35.30627845	131.8502508	-1.900904
Arhgap12	Arhgap12-005	1559.6422	0.017807806	0.043314918	0.26778269	-3.734371326	3.894973709	14.54527813	-1.900865
20431F20R	0431F20Rik-	22108.5	4.81803E-07	2.2951E-05	0.267787882	-3.73429892	55.3127673	206.5544072	-1.900837
Clip3	Clip3-007	1441.2378	0.000273625	0.001682829	0.267840606	-3.733563833	3.430338203	12.80738665	-1.900553
Zdhhc17	Zdhhc17-201	8063.4546	3.33099E-06	7.53543E-05	0.267861587	-3.733271389	21.12918092	78.88096663	-1.90044
Zbed5	Zbed5-001	393.63058	0.022560672	0.052177934	0.267899933	-3.732737033	1.431776482	5.344445097	-1.900234
Dgkz	Dgkz-013	1001.9823	0.003390234	0.011803955	0.267996678	-3.731389534	2.278115324	8.500535677	-1.899713
Stam	Stam-002	1025.9667	0.002250169	0.00859274	0.267999834	-3.731345592	2.867061992	10.69799913	-1.899696
Xrn1	Xrn1-001	10761.975	3.77774E-05	0.000393567	0.268001423	-3.731323475	26.91617496	100.4329555	-1.899687
P24-470E5.	24-470E5.9-0	1719	0.00117491	0.005171659	0.268018671	-3.731083344	3.290086038	12.27558522	-1.899595
Trim6	Trim6-002	781.92352	0.000140449	0.001031801	0.268077812	-3.730260234	1.946939811	7.262592157	-1.899276
Acer1	Acer1-201	512	0.00074646	0.003631616	0.268144708	-3.729329613	1.210569817	4.514613869	-1.898916
Snrpd1	Snrpd1-201	2376	0.005121968	0.01631209	0.26818676	-3.728744847	7.032619215	26.22284266	-1.89869
Arhgap21	Arhgap21-010	179.23226	0.03387924	0.072049829	0.268245833	-3.727923703	0.277223172	1.033466833	-1.898372
Ldah	Ldah-202	3517.6827	0.001910182	0.007552958	0.268258276	-3.727750794	8.603993294	32.07354284	-1.898305
Eif3j1	Eif3j1-001	6699.0212	1.59433E-06	4.6473E-05	0.268318813	-3.726909745	18.20306425	67.84117755	-1.89798
Ybey	Ybey-201	1654.6759	6.01222E-05	0.000551469	0.268327542	-3.72678851	3.824690864	14.25381396	-1.897933
Blnk	Blnk-003	305.3266	0.00257385	0.009528142	0.268373833	-3.72614569	0.678309728	2.52748087	-1.897684
Gm14270	Gm14270-001	287	0.007560685	0.022142238	0.268374254	-3.726139842	0.908219202	3.384151755	-1.897682
Dmd	Dmd-003	12677.259	3.87586E-05	0.000400827	0.268566046	-3.723478881	25.47837001	94.86817265	-1.896651
Senp6	Senp6-004	1663.5635	0.003734294	0.01272648	0.268573119	-3.723380825	4.825055475	17.96551903	-1.896613
Tbl1xr1	Tbl1xr1-001	7844.0605	4.37932E-05	0.000438619	0.268574621	-3.72336	21.77004286	81.05770677	-1.896605
B3galnt2	B3galnt2-201	3323.5914	2.41696E-06	6.15014E-05	0.268575137	-3.72335285	9.600645328	35.74659014	-1.896602
Brwd1	Brwd1-006	1563.0702	0.000548568	0.002873291	0.26857946	-3.723292918	4.066564069	15.1410092	-1.896579
Mnat1	Mnat1-001	708.82434	0.038341098	0.079451997	0.268591164	-3.723130666	1.502162063	5.592745641	-1.896516
Pnp	Pnp-201	8099.2085	2.62101E-05	0.000305547	0.268666119	-3.722091958	21.7948404	81.12240019	-1.896114
Rgag4	Rgag4-002	489.87698	0.01496235	0.037803222	0.268750013	-3.72093005	0.90133535	3.353805789	-1.895663
Rgag4	Rgag4-201	489.87698	0.01496235	0.037803222	0.268750013	-3.72093005	0.90133535	3.353805789	-1.895663
Clasp1	Clasp1-013	4139.6233	9.0351E-07	3.30258E-05	0.26877278	-3.720614864	10.07806487	37.49659794	-1.895541
Prdm9	Prdm9-003	285.61285	0.009249055	0.025920967	0.268835089	-3.719752516	0.484364872	1.801717451	-1.895207
030030I06R	0030I06Rik-	3086.942	2.47718E-06	6.23966E-05	0.268859905	-3.719409178	7.864468338	29.25117571	-1.895073
Nifk	Nifk-001	2587.1761	0.000116076	0.000892794	0.268912232	-3.718685434	9.211703412	34.2554273	-1.894793
Zfp560	Zfp560-001	964.21824	0.003098276	0.011013936	0.268949003	-3.718177007	2.102514863	7.81752242	-1.894595
Gm10171	Gm10171-001	171	0.037643589	0.078305066	0.268959166	-3.718036508	0.544688282	2.025170918	-1.894541
Fbxl12	Fbxl12-004	567.05766	0.04789239	0.094832303	0.268979201	-3.717759577	1.299436025	4.830990726	-1.894433
Ctsc	Ctsc-001	3678.0116	0.001257727	0.005454877	0.269039078	-3.716932151	15.77424859	58.63181177	-1.894112
Ptbp1	Ptbp1-006	1082.774	0.010263313	0.028164286	0.269054369	-3.716720916	2.980661482	11.07828687	-1.89403
RP23-1B20.	23-1B20.4-0	12541	1.71468E-07	1.29357E-05	0.269073472	-3.716457042	31.63934572	117.5862692	-1.893928
Rab14	Rab14-010	1984.9777	7.69848E-05	0.000661331	0.269080338	-3.716362218	5.57015231	20.70070359	-1.893891
30402H24R	0402H24Rik-	229.37023	0.042359133	0.085993736	0.269103038	-3.716048717	0.720993193	2.679245831	-1.893769
00012H06R	0012H06Rik-	2746.8805	0.007281225	0.021483628	0.269148939	-3.715414977	7.425084003	27.58726831	-1.893523
Hadha	Hadha-005	252.95636	0.029554442	0.064514954	0.2691847	-3.714921387	0.655616451	2.435563575	-1.893332
Ano4	Ano4-004	556	0.000798483	0.003823297	0.269210986	-3.714558662	1.457272557	5.4131244	-1.893191
Enah	Enah-008	1136	2.34429E-05	0.000281944	0.269238843	-3.714174338	2.690846794	9.994274108	-1.893042
Psmd6	Psmd6-001	4961.691	6.14341E-06	0.000111624	0.269240132	-3.714156557	13.55295378	50.33779214	-1.893035
Ipo11	Ipo11-007	377.88927	0.026396591	0.058967078	0.269266389	-3.713794376	1.023856463	3.802392374	-1.892894
30133O14R	0133O14Rik-	1110	0.017023022	0.041815857	0.269272021	-3.713716701	4.23700617	15.73504058	-1.892864
Pola1	Pola1-001	3389.9864	0.000569986	0.002960639	0.269334432	-3.712856135	9.012506117	33.46213863	-1.892529
Smg7	Smg7-003	1539.4839	0.002305855	0.008758514	0.269358764	-3.712520749	2.932636042	10.88747215	-1.892399
20014N23R	0014N23Rik-	581.39169	0.004547801	0.014859968	0.269386086	-3.712144215	1.698119621	6.303664925	-1.892253
Fam53b	Fam53b-003	214.74891	0.037069021	0.07736831	0.269455616	-3.711186329	0.658850447	2.445116773	-1.89188
Eri3	Eri3-006	701.66677	0.010896269	0.029473796	0.269522491	-3.710265498	1.643346865	6.097253172	-1.891522
Dguok	Dguok-003	485.02166	0.019951071	0.047426719	0.269542168	-3.709994652	1.221035072	4.530033585	-1.891417
Abcc4	Abcc4-202	3254.3637	0.000134504	0.000998105	0.269554673	-3.70982253	7.464323731	27.69131635	-1.89135
Eda2r	Eda2r-001	584.60507	0.015737833	0.039307284	0.269595955	-3.709254464	1.690118284	6.26907879	-1.891129
Setx	Setx-001	25288.163	1.22749E-07	1.07249E-05	0.269664199	-3.708315759	66.81772666	247.7812288	-1.890764
Phykpl	Phykpl-005	741.14776	0.037441816	0.077965845	0.269682973	-3.708057609	1.997093499	7.405337742	-1.890664
Trim16	Trim16-002	1121.3427	0.026621632	0.059361012	0.269799742	-3.706452768	1.791020086	6.638331355	-1.890039
Strap	Strap-001	7283.872	3.72739E-05	0.00038993	0.269802243	-3.706418413	19.72738181	73.11793116	-1.890026
P24-294K1	24-294K15.4-	573.70387	0.001262273	0.005470437	0.269812043	-3.706283782	1.494585167	5.539356767	-1.889973
Gm21893	Gm21893-201	117	0.047890022	0.094832303	0.269814289	-3.70625293	0.276905723	1.026282649	-1.889961
Stx4a	Stx4a-001	4297.0965	0.000219186	0.001429108	0.269837028	-3.705940605	10.7313331	39.76968309	-1.88984
230307C23R	0307C23Rik-	489.37603	0.005920961	0.018256475	0.269839787	-3.705902718	1.321998173	4.899196623	-1.889825
30503L19R	0503L19Rik-	3413	1.70283E-06	4.82397E-05	0.269842071	-3.70587135	9.020515283	33.42886915	-1.889813
Gm4609	Gm4609-001	762	0.001002426	0.004563146	0.269851745	-3.705738503	2.150641166	7.969713774	-1.889761
Gm10053	Gm10053-201	1700	0.029646456	0.064691074	0.269902771	-3.705037914	5.293787353	19.61368285	-1.889488
Rhobtb1	Rhobtb1-001	1079.7834	0.008853061	0.025024127	0.269906355	-3.704988721	2.711752137	10.04701108	-1.889469
Cyfip1	Cyfip1-009	8123.2614	8.04563E-07	3.07178E-05	0.269961639	-3.704229986	19.24701206	71.29535922	-1.889174
Zfp141	Zfp141-001	1754.3366	2.99466E-05	0.000335163	0.270001718	-3.703680135	4.887682482	18.10241251	-1.88896
Srsf1	Srsf1-001	7071.3456	2.61105E-05	0.000304593	0.270053155	-3.702974694	19.73493086	73.07794958	-1.888685
G3bp2	G3bp2-001	11598.187	9.78462E-07	3.44358E-05	0.270094933	-3.702401932	27.19156428	100.6741001	-1.888462
00004C02R	0004C02Rik-	888.86258	0.000177795	0.001221808	0.270112528	-3.702160759	2.128160906	7.878793797	-1.888368
Gm15601	Gm15601-001	492.20056	0.020397091	0.048225899	0.27011723	-3.702096305	1.393932647	5.160472902	-1.888342
Zufsp	Zufsp-201	3450	7.06635E-05	0.000621687	0.270118116	-3.702084171	8.914045751	33.00054767	-1.888338
Drr1	Drr1-001	224	0.009219582	0.025864225	0.2701331	-3.701878808	0.579594909	2.145590112	-1.888258
Ube2d1	Ube2d1-003	229.10801	0.007252622	0.021430618	0.270151464	-3.701627173	0.52108692	1.928869503	-1.88816
Gm37471	Gm37471-001	1044	4.10612E-05	0.000417809	0.270212535	-3.700790561	2.48197747	9.185278792	-1.887833
Xcr1	Xcr1-001	1146	1.51562E-05	0.000206978	0.27029678	-3.699637124	2.89328117	10.70409043	-1.887384
Gm38215	Gm38215-001	1126	2.73965E-05	0.000315193	0.270300155	-3.69959093	2.924679969	10.82011948	-1.887366
Ppp2r5c	Ppp2r5c-202	5637.3934	0.033830317	0.071963752	0.270320774	-3.699308728	13.7665037	50.92654728	-1.887256
Ssh2	Ssh2-006	247.29258	0.016476417	0.040736106	0.270337965	-3.699073495	0.811085189	3.000263724	-1.887164
Ebf1	Ebf1-001	2388.1853	0.013071665	0.034009762	0.270368132	-3.698660753	5.49476485	20.3232711	-1.887003
Mrfap1	Mrfap1-001	31873	1.65058E-08	3.19391E-06	0.270372764	-3.698597392	82.56882511	305.3888412	-1.886978
Hnrnph1	Hnrnph1-006	6428.15	0.000640645	0.003237251	0.270383178	-3.698454941	15.41386267	57.00747655	-1.886923
Tsga10	Tsga10-007	416.41663	0.020214932	0.047884461	0.270421983	-3.697924213	0.878798122	3.249728852	-1.886716
Slfn9	Slfn9-001	9040.4357	9.50163E-05	0.000769169	0.270455631	-3.697464144	25.91769183	95.82973622	-1.886536
Ccdc132	Ccdc132-001	2097.5544	0.004467134	0.014652302	0.270468416	-3.697289374	5.078967141	18.77841124	-1.886468

Rnpep	Rnpep-008	1169	7.90545E-05	0.000674207	0.270516441	-3.696632988	3.280591945	12.1271444	-1.886212
Lsm3	Lsm3-001	1129.3309	0.019202067	0.046007052	0.270517979	-3.696611976	3.153980369	11.6590416	-1.886204
Apc	Apc-004	2211.6332	0.000103834	0.000819427	0.270554589	-3.696111769	5.194064462	19.19784279	-1.886008
23-298M2	33-298M23.11	379	0.008346143	0.023880986	0.270563346	-3.695992135	0.842589212	3.114203099	-1.885962
Topbp1	Topbp1-001	8988.977	0.00044117	0.002428098	0.270667951	-3.694563748	24.1865855	89.35888199	-1.885404
Cox7b	Cox7b-001	2427.3501	0.000489921	0.002632123	0.270704335	-3.694067188	5.97629083	22.07681986	-1.88521
Svil	Svil-008	4395.8196	0.003237925	0.011405836	0.27073413	-3.693660646	10.26529065	37.91650009	-1.885051
Rnf103	Rnf103-002	5258.0132	2.95731E-05	0.000332527	0.270774111	-3.69311526	12.32488435	45.51721846	-1.884838
Palmd	Palmd-004	5739.9286	0.000303089	0.001823179	0.270775101	-3.69310175	9.122035315	33.68860458	-1.884833
Car5a	Car5a-001	253	0.009183849	0.02578464	0.270843201	-3.692173165	0.67938906	2.508422056	-1.88447
10315B03R	0315B03Rik-	1401.7281	0.000590131	0.003036199	0.270850621	-3.692072027	3.702441884	13.66968211	-1.884431
Prpf18	Prpf18-001	3928.3541	2.16936E-05	0.000267268	0.270882141	-3.691642408	9.6471043	35.61365935	-1.884263
Dcun1d1	Dcun1d1-009	1042.7182	0.001332052	0.005700711	0.270889066	-3.691548044	2.751790581	10.15836714	-1.884226
Ireb2	Ireb2-201	10494	5.35097E-07	2.41782E-05	0.270900848	-3.691387485	27.18119351	100.3363175	-1.884163
Taok1	Taok1-001	45075.285	1.90123E-06	5.1969E-05	0.270922299	-3.691095215	108.0462999	398.8091807	-1.884049
Prrc1	Prrc1-201	8613	7.04412E-07	2.82838E-05	0.270922874	-3.691087381	22.50198188	83.05678135	-1.884046
Frmd4a	Frmd4a-001	1713.3933	0.027838199	0.061518521	0.270941248	-3.690837065	6.309149529	23.28604293	-1.883948
Gm22692	Gm22692-201	332	0.002458699	0.009202391	0.270950652	-3.690708969	0.855658166	3.157985267	-1.883898
Aasdhppt	Aasdhppt-201	1746	4.36953E-05	0.000437976	0.270969774	-3.69044851	4.580498971	16.9040956	-1.883796
Mgea5	Mgea5-201	26806.382	1.921E-08	3.51841E-06	0.271004006	-3.689982357	61.19324435	225.8019776	-1.883614
Nova1	Nova1-201	8679	0.001790099	0.007175898	0.271017603	-3.689797222	20.54230849	75.79695279	-1.883542
Mrps36	Mrps36-001	750.1641	0.003857047	0.013042143	0.271017886	-3.689793374	1.833594869	6.765586199	-1.88354
Samhd1	Samhd1-005	5668.8446	0.002284511	0.008695487	0.271032186	-3.689598701	20.39775906	75.25954532	-1.883464
Ankrd17	Ankrd17-010	1839.9109	0.000135688	0.001004605	0.271045294	-3.68942027	4.999726306	18.44609158	-1.883394
Psmd12	Psmd12-002	3313.5563	0.006236	0.019022354	0.271135088	-3.688198407	9.783676426	36.08413981	-1.882916
Tpp1	Tpp1-201	16695	6.79347E-08	7.6038E-06	0.271145824	-3.688052375	35.44366761	130.7181025	-1.882859
Gm26674	Gm26674-201	153.14136	0.024932252	0.056347388	0.271150452	-3.687989427	0.349351478	1.288404555	-1.882835
RP23-407I6.	P23-407I6.5-0	629	0.000555299	0.002901449	0.271209099	-3.687191931	1.707735865	6.296749902	-1.882523
Oard1	Oard1-201	1791.6588	0.011651537	0.031106488	0.271231858	-3.686882537	5.023558956	18.52127179	-1.882401
Casp3	Casp3-201	1045	0.034652138	0.073347489	0.271270826	-3.68635291	3.548453511	13.08085193	-1.882194
Glg1	Glg1-002	4463.9324	0.018414707	0.04449409	0.271299458	-3.685963869	13.03847661	48.05935368	-1.882042
Trpm7	Trpm7-002	5339.0371	0.002924934	0.010546688	0.271302395	-3.685923973	13.31256788	49.06911311	-1.882026
Zfp157	Zfp157-001	1584.6779	1.71441E-05	0.000225889	0.27131368	-3.685770655	4.237174796	15.61725452	-1.881966
Aebp2	Aebp2-008	1230.0072	0.000631802	0.003201543	0.271330486	-3.685542355	2.458363844	9.060404071	-1.881877
P23-313F7.	23-313F7.2-0	385	0.001388277	0.005889814	0.271347287	-3.685314163	1.153840904	4.252266226	-1.881788
Bcl9	Bcl9-001	4036.5177	0.00020857	0.001375075	0.271359917	-3.685142633	11.40094957	42.0141253	-1.88172
Npdc1	Npdc1-002	1033.7524	0.014258496	0.036419861	0.271428475	-3.684211841	2.596183792	9.564891069	-1.881356
Rint1	Rint1-001	463.05626	0.008815426	0.024930092	0.271471957	-3.683621728	1.366288611	5.032890413	-1.881125
Akap2	Akap2-005	37693.325	9.35774E-08	9.18346E-06	0.271555253	-3.682491832	84.34391811	310.5957895	-1.880682
Gm34267	Gm34267-001	456	0.000437735	0.002413851	0.271581843	-3.682131277	1.19891706	4.414570006	-1.880541
Mboat1	Mboat1-001	314.7901	0.014473703	0.036854086	0.271796909	-3.679217705	0.66746647	2.455754453	-1.879399
Spaca6	Spaca6-007	290.49448	0.013325132	0.034551121	0.271852394	-3.678466774	0.574874277	2.114655927	-1.879105
Ginm1	Ginm1-005	148.34221	0.039618096	0.081451243	0.27186926	-3.678238583	0.383916627	1.412136952	-1.879015
Znhit3	Znhit3-002	416.27898	0.001902838	0.007527395	0.271876181	-3.678144942	0.987041883	3.63048311	-1.878978
Sptbn1	Sptbn1-002	22218.185	4.5315E-05	0.000449216	0.271896446	-3.677870804	44.02669782	161.9245065	-1.878871
Fam214b	Fam214b-006	627.61661	0.020482427	0.048397595	0.271900404	-3.677817263	1.389796723	5.111418381	-1.87885
Mark2	Mark2-009	1006.2126	0.000367976	0.002106499	0.271980934	-3.676728314	2.82131706	10.37321632	-1.878423
Gm7206	Gm7206-001	3243	3.4371E-06	7.68412E-05	0.272009472	-3.676342569	9.387942541	34.5132928	-1.878271
Pacsin2	Pacsin2-202	10557.987	2.09014E-05	0.000260886	0.272067196	-3.675562559	25.9620633	95.42518784	-1.877965
Ogfod1	Ogfod1-004	1741.6032	0.013787954	0.035466574	0.272116856	-3.674891783	5.180678812	19.038434	-1.877702
Fblim1	Fblim1-002	12349.687	1.14757E-05	0.000170296	0.272142091	-3.67455103	38.55121883	141.6584208	-1.877568
Slc9a9	Slc9a9-001	988.06796	0.007570414	0.022163149	0.272145347	-3.674507067	1.942782232	7.138767043	-1.877551
Ankrd13a	Ankrd13a-003	1817.0491	0.009858859	0.027257501	0.272153029	-3.674403339	5.303434452	19.48695726	-1.87751
Tet2	Tet2-004	1860.1347	0.000111946	0.000868044	0.272168472	-3.674194857	5.182859251	19.04283481	-1.877428
Mdm4-ps	Mdm4-ps-001	752	0.000269016	0.00166314	0.272216153	-3.673551288	1.961356456	7.205143537	-1.877175
Foxp1	Foxp1-002	571.40437	0.024741698	0.056018754	0.272236542	-3.673276156	1.199280971	4.405290196	-1.877067
Usp8	Usp8-002	3112.1816	0.00015753	0.001120075	0.272287612	-3.672587202	7.239070618	26.5861181	-1.876797
Gm38065	Gm38065-001	656	0.004190823	0.013929308	0.27232903	-3.672028648	1.472945446	5.408697875	-1.876577
Fundc1	Fundc1-001	1337.0662	0.007128115	0.021131996	0.272336982	-3.671921431	3.614103883	13.2707055	-1.876535
Srp72	Srp72-001	8894.2316	6.73401E-06	0.000118559	0.272343315	-3.671836038	24.59573951	90.31152272	-1.876502
Tpr	Tpr-001	33703.59	6.65369E-07	2.73299E-05	0.272431591	-3.670646256	83.56846916	306.7502884	-1.876034
Zfp422	Zfp422-002	231.9223	0.004730159	0.015306033	0.272471076	-3.670114326	0.566417916	2.078818507	-1.875825
Gm25219	Gm25219-201	744	0.006548474	0.019732997	0.272479179	-3.67000519	1.872137437	6.870754109	-1.875782
Sat1	Sat1-002	4661.4377	0.001607845	0.006596633	0.272509694	-3.669594233	13.82550359	50.73398824	-1.875621
Lox	Lox-202	38340.168	0.000163855	0.001152608	0.272532792	-3.669283216	62.43041629	229.0748786	-1.875498
Rassf1	Rassf1-007	1090.5159	0.004349296	0.014342712	0.27253898	-3.669199909	2.139708774	7.851019237	-1.875466
Chic1	Chic1-001	3427	0.000110241	0.000857935	0.272622805	-3.66807172	8.784645202	32.22270864	-1.875022
Gm6793	Gm6793-201	10648	6.31308E-05	0.000572322	0.272677149	-3.667340674	29.69278952	108.8935747	-1.874734
Rab32	Rab32-001	998	0.015294824	0.038458829	0.272687348	-3.667203514	3.60123103	13.20644709	-1.87468
Rpl31	Rpl31-005	6745.321	1.00629E-05	0.000155177	0.272711305	-3.666881353	20.14788931	73.87991962	-1.874554
Lnp	Lnp-005	350.12997	0.015073514	0.037999989	0.272713083	-3.666857455	0.997210458	3.656628603	-1.874544
Tmem234	Tmem234-001	8836.347	6.37146E-07	2.65801E-05	0.27271921	-3.666775071	21.5753794	79.11206332	-1.874512
Btbd19	Btbd19-003	2526.6277	5.23727E-05	0.000497853	0.272726255	-3.666680343	6.110324411	22.40460641	-1.874474
Ints7	Ints7-001	541.68804	0.02713938	0.060331263	0.272735974	-3.666549691	1.630223798	5.977296561	-1.874423
Invs	Invs-005	1646.6608	0.000644532	0.003251136	0.272739199	-3.66650633	3.980925145	14.59608724	-1.874406
Bnip2	Bnip2-001	26214.105	9.29709E-07	3.35538E-05	0.272812757	-3.66551774	55.19804484	202.3294125	-1.874017
Arhgap21	Arhgap21-008	455.29859	0.039134664	0.080686386	0.272882115	-3.664586083	1.343948287	4.92501419	-1.87365
30046D13R	0046D13Rik-	1367	0.000166496	0.001166627	0.272909235	-3.664221921	3.464696477	12.69541678	-1.873507
Aste1	Aste1-009	408.55778	0.034884573	0.073730123	0.272917496	-3.66411101	1.139902677	4.17672995	-1.873463
Gm11672	Gm11672-001	239	0.031937603	0.068684095	0.272933619	-3.66389456	0.555748023	2.036202158	-1.873378
Wdr73	Wdr73-003	730.98659	0.002245686	0.008578491	0.272945545	-3.663734463	1.932585815	7.080481253	-1.873315
Nbeal1	Nbeal1-008	876.70131	0.004596717	0.014978199	0.272959465	-3.66354763	2.531145329	9.272971468	-1.873241
Prpf4	Prpf4-001	2903.3475	1.61177E-05	0.000215468	0.273016694	-3.662779687	8.422236272	30.84879594	-1.872939
Dlg1	Dlg1-012	907.78113	0.000482222	0.002599323	0.273020872	-3.662723637	1.909765815	6.994944392	-1.872917
Gm4793	Gm4793-001	709.75494	0.00027667	0.00169742	0.273037287	-3.662503432	2.024571438	7.414999841	-1.87283
P24-187P11	24-187P11.3-	297	0.017666433	0.043068022	0.273055121	-3.662264222	0.795974771	2.915069924	-1.872736
Rgs9	Rgs9-005	442.72497	0.00275446	0.010050097	0.27312354	-3.661346803	1.091720177	3.997166179	-1.872374
Gm11539	Gm11539-001	221	0.017231949	0.042200947	0.273164651	-3.660795779	0.683165016	2.500927608	-1.872157
Lipo2	Lipo2-001	179.39589	0.018001469	0.043692887	0.273221236	-3.660037606	0.41841319	1.531408009	-1.871858
Erc1	Erc1-005	854.78249	0.007670926	0.022386608	0.273224813	-3.659989698	2.013422332	7.369104991	-1.87184
Gm11525	Gm11525-001	964	0.000242724	0.001540433	0.273331209	-3.658565025	2.422998909	8.864699064	-1.871278
Mlh3	Mlh3-202	1735.6306	0.000611423	0.003122883	0.273359692	-3.658183809	4.310616431	15.76902723	-1.871128
Gm13231	Gm13231-001	414	0.004037909	0.013518905	0.273426264	-3.657293148	1.132881467	4.143279627	-1.870776
Fam175b	Fam175b-005	343.07384	0.007106475	0.021087852	0.273435391	-3.657171061	0.923661467	3.377987988	-1.870728
30306N03R	0306N03Rik-	1040.8743	0.001311772	0.005636455	0.273459296	-3.656851364	3.573532648	13.06787774	-1.870602
Pvr	Pvr-002	796.88023	0.01162435	0.031043587	0.273500022	-3.656306841	1.929400342	7.054479668	-1.870387
Cd8b1	Cd8b1-201	357	0.003737877	0.012732201	0.273562933	-3.655466	0.994471765	3.635257724	-1.870055
Pmepa1	Pmepa1-001	16408.33	0.000823276	0.003916821	0.273612424	-3.6548048	46.9298045	171.5192748	-1.869794
Mdh1	Mdh1-001	3656.2844	6.12563E-07	2.5948E-05	0.27367881	-3.653918262	9.537811344	34.85038305	-1.869444
Phc3	Phc3-006	1075.1092	0.000584294	0.003013867	0.273710835	-3.653490737	2.375169998	8.677661588	-1.869276
Rnmt	Rnmt-001	1495.557	9.98962E-05	0.000796952	0.273753954	-3.65291528	4.104553607	14.9935867	-1.869048
Slc7a1	Slc7a1-005	1313.4884	0.001523865	0.006327881	0.273771848	-3.652676521	3.670376268	13.40669722	-1.868954
Dnttip2	Dnttip2-001	7887.1297	3.78505E-07	1.99137E-05	0.273840413	-3.651761954	22.18016644	80.99668794	-1.868593
Gm38077	Gm38077-001	1035	0.000283407	0.001729938	0.273873318	-3.651323205	2.652368717	9.684655445	-1.868419
Kansl1l	Kansl1l-005	961.51084	0.006975846	0.02076325	0.273874407	-3.651308683	2.387811697	8.718637582	-1.868414
Gm26547	Gm26547-201	10662	3.70308E-06	8.05433E-05	0.273882408	-3.651202013	26.20304112	95.67259649	-1.868371
Ankrd11	Ankrd11-201	17931.855	4.31654E-05	0.000434317	0.273918634	-3.650719135	42.96740605	156.8619314	-1.868181

Spag5	Spag5-007	474.27388	0.003113207	0.011059778	0.273950464	-3.650294962	1.212292647	4.425225741	-1.868013
Ino80d	Ino80d-201	18036.756	3.47084E-08	4.91224E-06	0.273960053	-3.650167201	43.30451526	158.0687213	-1.867963
Csnk2a1	Csnk2a1-003	601.19849	0.003760195	0.012790413	0.274004844	-3.649570522	1.207606538	4.407245224	-1.867727
Slc35f5	Slc35f5-001	3773.3457	3.4129E-05	0.000365779	0.274117703	-3.64806792	8.642071422	31.52686352	-1.867133
Sh3bp5l	Sh3bp5l-002	1276.2829	0.01074383	0.029146629	0.274125223	-3.647967854	3.173714388	11.57760807	-1.867093
Slc26a2	Slc26a2-001	7997.8506	5.34067E-06	0.000101621	0.274220325	-3.646702702	20.57691871	75.03790507	-1.866593
Spaca6	Spaca6-002	379.131	0.017066522	0.041892667	0.274252012	-3.646281369	1.027801811	3.747654593	-1.866426
Kcnn3	Kcnn3-002	16170.06	0.011574175	0.030936125	0.274261905	-3.646149835	22.65829031	82.61552147	-1.866374
Grsf1	Grsf1-007	398.93129	0.036852176	0.077037782	0.274271868	-3.646017389	1.036547365	3.779269716	-1.866321
Tbc1d24	Tbc1d24-003	2404.9623	0.004102	0.013682655	0.274309153	-3.645521804	4.630221722	16.87957425	-1.866125
Gm15541	Gm15541-001	1133	0.000141432	0.001037044	0.27431791	-3.645405437	3.128654336	11.40521353	-1.866079
Srsf7	Srsf7-201	8561	0.000266659	0.001652145	0.274328819	-3.645260477	26.80940788	97.72727495	-1.866022
Mbtps2	Mbtps2-003	719.16466	0.011070189	0.029845244	0.27433149	-3.645224979	2.188053818	7.975948433	-1.866008
Mroh2a	Mroh2a-001	771.30578	0.00142074	0.006000042	0.274340515	-3.645105067	1.908929531	6.958248707	-1.86596
Mroh2a	Mroh2a-201	581.4364	0.001345283	0.005744413	0.274342014	-3.64508514	1.528082985	5.569992581	-1.865953
Tcp11l1	Tcp11l1-001	4880.2508	5.26369E-05	0.000499533	0.274387525	-3.644480554	14.96034528	54.52268747	-1.865713
Pdap1	Pdap1-005	1080.0668	0.003398339	0.011824964	0.274413702	-3.644132896	2.928269642	10.67100373	-1.865576
Atl1	Atl1-201	775.63904	0.000827118	0.003930733	0.274458903	-3.643532738	1.868844385	6.809195698	-1.865338
Mycbp2	Mycbp2-005	644.97319	0.016050047	0.039892527	0.274519825	-3.642724166	1.335593811	4.865199851	-1.865018
Parp10	Parp10-002	449.87115	0.006990245	0.020798879	0.274521185	-3.642706123	1.060345282	3.862526253	-1.865011
Gm28112	Gm28112-001	365	0.014751961	0.037407426	0.274539277	-3.642466066	0.933159102	3.399000363	-1.864916
Ago3	Ago3-001	19168.253	5.30914E-06	0.000101303	0.274559776	-3.642194115	44.80520275	163.1892458	-1.864808
Rpl10	Rpl10-002	3406.2465	2.16022E-05	0.000266911	0.274656575	-3.640910477	9.314782589	33.91428952	-1.864299
Nup160	Nup160-001	8700.4662	2.27697E-07	1.5202E-05	0.274669233	-3.640742678	19.77699861	72.00296287	-1.864233
10052M02R	0052M02Rik-	437	0.004337038	0.014307799	0.274756366	-3.639588101	0.948182213	3.4509927	-1.863775
Gas5	Gas5-003	3927.4536	0.000689856	0.003425278	0.274764692	-3.639477811	12.89265834	46.92254395	-1.863731
Gm36963	Gm36963-001	817	0.001478727	0.006189979	0.274839764	-3.638483694	1.662310869	6.04829099	-1.863337
Ankrd13c	Ankrd13c-00	647.03646	0.01835807	0.044376025	0.274869703	-3.638087383	1.840473297	6.695802682	-1.86318
30403G16R	0403G16Rik-	1116	0.003505638	0.012120746	0.274878479	-3.637971229	2.768371681	10.07125653	-1.863134
Pcdhb19	Pcdhb19-001	653	0.024998068	0.056480231	0.274938173	-3.637181361	1.809932856	6.583054048	-1.862821
Ube2e1	Ube2e1-002	881.78615	0.000178088	0.001223571	0.274943617	-3.637109345	2.375846671	8.641214132	-1.862792
Dpy19l3	Dpy19l3-001	1215.388	0.003389764	0.011803955	0.274984328	-3.636570884	3.700171488	13.4559359	-1.862579
Fam160a2	Fam160a2-20	2638.4071	0.030177536	0.065653346	0.275038344	-3.635856684	6.784766753	24.66843955	-1.862295
Arhgdib	Arhgdib-002	8830.921	1.96621E-05	0.000249196	0.27504167	-3.635812716	19.89610434	72.33850916	-1.862278
Tmem5	Tmem5-002	1338.6686	9.18619E-05	0.000750363	0.275090533	-3.635166905	3.226545338	11.72903083	-1.862022
Morf4l1	Morf4l1-002	1869.1454	0.005178809	0.016451265	0.275101787	-3.635018197	4.069274148	14.79188558	-1.861963
Eif4ebp2	Eif4ebp2-201	6109.9363	1.66824E-06	4.76145E-05	0.275128395	-3.634666637	15.23308378	55.36718139	-1.861823
Rabgap1l	Rabgap1l-015	341	0.028034	0.061876231	0.275129639	-3.634650211	0.570975641	2.075296734	-1.861817
Cspp1	Cspp1-018	1790.6951	0.00048295	0.00260243	0.27516763	-3.634148386	5.382981621	19.56255397	-1.861617
Pigu	Pigu-001	1879.393	0.000343102	0.002000895	0.2751768	-3.634027292	4.521522715	16.43133695	-1.861569
Jup	Jup-004	217.7156	0.00726007	0.021443376	0.275274149	-3.63274214	0.506911379	1.841478326	-1.861059
Acbd3	Acbd3-002	1539.9472	5.65932E-05	0.000527503	0.275305085	-3.632333922	3.821756599	13.88189614	-1.860897
Macf1	Macf1-201	36909.196	4.92608E-08	6.25603E-06	0.27531247	-3.632236496	83.15568706	302.0411214	-1.860858
Fubp1	Fubp1-006	493.91661	0.003685074	0.012602476	0.275343736	-3.63182404	1.470852839	5.341878701	-1.860694
Slc25a36	Slc25a36-003	3966.3635	0.024469031	0.055537728	0.275359004	-3.631622669	10.7924993	39.1942851	-1.860614
Stat5b	Stat5b-002	3716.3165	0.022371055	0.051837401	0.275361271	-3.631592771	9.853857554	35.78519786	-1.860602
Tmed4	Tmed4-001	7185.059	3.61583E-06	7.9386E-05	0.275420651	-3.630809802	17.81099492	64.66833494	-1.860291
Arih1	Arih1-003	260.31406	0.021092955	0.049522463	0.275422858	-3.630780704	1.024382312	3.719307534	-1.86028
30024N20R	0024N20Rik-	1226	0.000671942	0.0033553	0.275429985	-3.630686754	2.709647567	9.837881528	-1.860242
Pla2g2d	Pla2g2d-001	238	0.005690022	0.017698959	0.275444636	-3.630493649	0.591058547	2.145834301	-1.860166
Cttnbp2nl	Cttnbp2nl-00	4234.0584	2.36093E-05	0.000283427	0.275465905	-3.630213323	13.81337843	50.14551042	-1.860054
Abca3	Abca3-005	2490.3277	0.002425596	0.009101298	0.275526775	-3.629411333	5.335414262	19.36441299	-1.859736
Isoc1	Isoc1-201	1854	0.001641623	0.006698307	0.275530978	-3.62935597	5.320452621	19.30981649	-1.859714
Nfib	Nfib-201	21649.061	1.84049E-05	0.000237849	0.275562306	-3.628943352	50.14374654	181.9688156	-1.85955
Rchy1	Rchy1-002	1339.1107	0.011745945	0.031299978	0.275563605	-3.628926252	3.343765037	12.13427672	-1.859543
Pank3	Pank3-001	11587	3.9438E-07	2.02816E-05	0.275581397	-3.628691963	28.48012	103.3455826	-1.85945
Jpx	Jpx-001	858.64628	0.000783205	0.003765831	0.275584941	-3.628645299	2.638926961	9.575729913	-1.859431
Galnt1	Galnt1-001	3423.6479	0.011060991	0.029832188	0.275647328	-3.627824035	11.74972028	42.62591763	-1.859104
Gria3	Gria3-003	1713.9608	0.016095903	0.039985258	0.275693888	-3.627211354	2.859088318	10.37051761	-1.858861
P24-274B10	24-274B10.1-	796	0.000111346	0.000864369	0.275798588	-3.625834376	2.041406272	7.401801035	-1.858313
Fam214a	Fam214a-201	887.1592	0.040554069	0.083023125	0.275845714	-3.625214929	2.456709282	8.906099164	-1.858067
Golph3	Golph3-201	13288.744	3.55917E-07	1.9041E-05	0.275852257	-3.625128936	31.84518837	115.4429138	-1.858032
Stk11ip	Stk11ip-003	944.50373	0.010050434	0.027706477	0.275861802	-3.625003509	2.314459968	8.389925505	-1.857982
00109H08R	0109H08Rik-	1740	0.000156191	0.00111356	0.275932269	-3.624077762	4.768883198	17.28280354	-1.857614
Micu1	Micu1-003	611.74719	0.009843541	0.027219545	0.275940945	-3.62396381	1.497233504	5.425920035	-1.857569
Spata6	Spata6-003	937.37159	0.005068359	0.01616758	0.276032369	-3.622763528	2.475972751	8.96986378	-1.857091
Gm5850	Gm5850-001	520	0.001291953	0.005573121	0.276038775	-3.622679457	1.599781399	5.795495209	-1.857057
Cyld	Cyld-203	8827.8091	4.97208E-05	0.000478891	0.276039943	-3.622664135	25.23853097	91.43072099	-1.857051
Gm26853	Gm26853-201	10680	1.68401E-06	4.79048E-05	0.27605468	-3.62247074	26.4463718	95.80120802	-1.856974
Gm26765	Gm26765-201	456.94074	0.011050077	0.029807446	0.276111448	-3.621725967	1.005884625	3.643038465	-1.856677
Mbd5	Mbd5-003	5613.899	0.000316249	0.001880476	0.276113974	-3.62169283	12.61183757	45.67620169	-1.856664
Afap1	Afap1-004	2503.7045	1.49238E-06	4.4719E-05	0.276125282	-3.621544515	5.890923625	21.33424214	-1.856605
Peg3	Peg3-001	10983.876	0.007901744	0.022914148	0.276178102	-3.620851882	29.86282676	108.1288725	-1.856329
Hnrnpf	Hnrnpf-009	14238.047	4.36282E-06	8.91035E-05	0.27622493	-3.620238042	39.47177424	142.8972187	-1.856085
Parp2	Parp2-201	4174	4.42726E-05	0.000441951	0.276236332	-3.620088612	11.44057153	41.41588271	-1.856025
Gm10025	Gm10025-201	2907	3.1421E-06	7.27812E-05	0.276242497	-3.620007818	7.290651529	26.39221553	-1.855993
Kalrn	Kalrn-004	323.92054	0.036620173	0.076608901	0.276288266	-3.61940815	0.698204681	2.527087714	-1.855754
Ppp3cb	Ppp3cb-008	242.81984	0.044237084	0.089025214	0.276294252	-3.619329734	0.618895258	2.239986008	-1.855723
Ppap2a	Ppap2a-201	9122.5554	7.95878E-06	0.000132582	0.276311391	-3.619105225	15.45060011	55.91734759	-1.855633
Ahr	Ahr-001	4006.7023	0.001808986	0.00723249	0.276339565	-3.618736249	15.42692599	55.8259763	-1.855486
P24-559G13	24-559G13.2-	272	0.022969899	0.052872481	0.276425095	-3.617616557	0.980998914	3.548877914	-1.85504
Gm10286	Gm10286-201	452	0.000691386	0.00343188	0.276432599	-3.617518351	1.048314934	3.792298512	-1.855
Dars	Dars-002	370.99614	0.009043887	0.025475213	0.27647642	-3.616944988	0.914090046	3.306213412	-1.854772
Gabbr1	Gabbr1-004	1840.904	0.000119991	0.000915911	0.276506675	-3.616549223	3.971697923	14.36384103	-1.854614
Stxbp1	Stxbp1-003	8929.3645	2.51892E-05	0.000297083	0.27651803	-3.616400711	17.53141361	63.40061666	-1.854555
Zfp422	Zfp422-003	2467.7971	0.000194567	0.001305371	0.276525785	-3.616299291	6.525831078	23.5993583	-1.854514
Foxn3	Foxn3-201	5170.745	0.020504043	0.048445327	0.276552091	-3.615955301	12.14745643	43.92465945	-1.854377
Sulf1	Sulf1-007	5117.3531	1.41639E-05	0.000197137	0.276588542	-3.615478762	11.39129696	41.18499223	-1.854187
Gm6483	Gm6483-004	12001.081	2.29663E-06	5.95363E-05	0.276594177	-3.615405102	30.22288611	109.2679767	-1.854157
Atl3	Atl3-201	30133.828	3.97559E-08	5.40333E-06	0.276625191	-3.614999758	67.93125546	245.5714721	-1.853996
P2ry6	P2ry6-201	2887	0.014728176	0.037368543	0.276660675	-3.614536113	7.023666494	25.38729619	-1.853811
Irak4	Irak4-001	3786.9425	1.15131E-05	0.000170704	0.276669158	-3.614425278	8.104303796	29.2924005	-1.853766
P23-342M4	23-342M4.7-	9516	9.34979E-07	3.36377E-05	0.276794873	-3.612783679	23.31710065	84.23964068	-1.853111
Fam208b	Fam208b-201	13048.822	5.22049E-07	2.37768E-05	0.276800631	-3.612708521	39.29141539	141.9484312	-1.853081
Gmfb	Gmfb-201	2711.0227	0.00047732	0.002581434	0.276849565	-3.612069973	6.278394367	22.67799977	-1.852826
Rpsa-ps5	Rpsa-ps5-001	1053	0.000161891	0.001142071	0.27686042	-3.611928347	3.289883237	11.88282252	-1.852769
Fundc2	Fundc2-001	7543.9378	1.39102E-05	0.000194856	0.276882053	-3.611646152	18.76612729	67.77661139	-1.852657
Gtf2a1	Gtf2a1-001	7880.1956	6.96308E-06	0.000120936	0.276977354	-3.610403473	20.86419026	75.32814498	-1.85216
Mast4	Mast4-003	6263.7711	2.14016E-05	0.000265485	0.277021604	-3.609826765	20.67532787	74.63435193	-1.85193
Cgref1	Cgref1-002	1790	1.80099E-05	0.000233828	0.277027415	-3.609751041	4.389254987	15.84411776	-1.851899
230072E10R	0072E10Rik-	705.07486	0.000329608	0.001942707	0.277051583	-3.609436149	1.778506513	6.4194057	-1.851773
Dynlrb1	Dynlrb1-001	1409.9488	6.19959E-05	0.000564023	0.277070547	-3.609189103	3.28650875	11.86163157	-1.851675
Nol4l	Nol4l-006	3368.4277	0.000143695	0.00104736	0.277071685	-3.609174284	9.091205404	32.81174476	-1.851669
P24-448C16	24-448C16.4-	441	0.000741986	0.003616482	0.277075273	-3.60912755	1.122105241	4.049820938	-1.85165
Mydgf	Mydgf-001	5689.2896	8.68225E-05	0.000719383	0.277076915	-3.609106157	15.53362806	56.06251267	-1.851642
Sbno1	Sbno1-006	347.91719	0.005372667	0.016958239	0.277096951	-3.608845189	0.807245494	2.913224016	-1.851537
Coq10b	Coq10b-002	224.72409	0.017402473	0.042540482	0.277121409	-3.608526688	0.45349712	1.636456461	-1.85141

Ric3	Ric3-003	613.62003	0.01696056	0.041707292	0.277183862	-3.607713636	1.813351333	6.54205233	-1.851085
Tnni3	Tnni3-001	291.90102	0.040667245	0.083190177	0.277201812	-3.607480027	0.856885355	3.091196804	-1.850991
Zfp874b	Zfp874b-001	2394	0.000427963	0.002369887	0.277230869	-3.607101926	6.040407821	21.78836669	-1.85084
Arhgap20os	rhgap20os-0(	217.24823	0.02194384	0.051068594	0.277249002	-3.606866	0.501512284	1.808887604	-1.850746
Tmem127	Tmem127-00	11259.823	3.35556E-07	1.84549E-05	0.277257425	-3.606756431	27.59631701	99.53319385	-1.850702
Mtfr1	Mtfr1-001	1747.6399	0.000146356	0.001061116	0.277262508	-3.606690311	4.639238298	16.73229582	-1.850676
Foxp1	Foxp1-004	14081.424	0.000429341	0.002376744	0.277366368	-3.605339779	23.9469576	86.33691881	-1.850135
Gm12033	Gm12033-001	1192	0.000641829	0.003241173	0.277372029	-3.605266192	3.487732159	12.57420284	-1.850106
Zzef1	Zzef1-006	828.38539	0.025118956	0.056691669	0.277372876	-3.605255182	2.173046315	7.834386486	-1.850101
Ufd1l	Ufd1l-007	429.23104	0.000728906	0.003568814	0.277411501	-3.60475322	1.125176545	4.055983772	-1.8499
Eif4g3	Eif4g3-201	8327.5553	0.000222626	0.001444611	0.277442655	-3.604348443	19.86428022	71.59778748	-1.849738
Map4k3	Map4k3-202	3672.2618	0.000829	0.003937442	0.2774574	-3.604156895	8.00069692	28.83576697	-1.849662
Atxn3	Atxn3-001	7917.8534	6.98272E-07	2.81865E-05	0.277465368	-3.604053389	20.46656352	73.76258762	-1.84962
Pcdhga8	Pcdhga8-002	900.05637	0.001434088	0.006044489	0.277500498	-3.603597142	2.170071095	7.820061997	-1.849438
Glb1l	Glb1l-010	430.90434	0.005321344	0.016830365	0.277505643	-3.603530323	1.114459653	4.015989151	-1.849411
Vps54	Vps54-002	4937.1961	0.000206053	0.001362414	0.277512483	-3.603441506	10.68816216	38.51416714	-1.849375
Gm10069	Gm10069-001	587	0.000401778	0.002257364	0.27755563	-3.602881346	1.513676581	5.453597118	-1.849151
Mettl14	Mettl14-001	4800.779	5.60331E-07	2.46675E-05	0.277567185	-3.602731358	12.10300023	43.60385844	-1.849091
Dqx1	Dqx1-201	428	0.001842712	0.007339559	0.277578543	-3.602583935	1.19129641	4.29174531	-1.849032
Alg9	Alg9-001	360.05876	0.031564634	0.068044355	0.277600117	-3.602303962	0.926638265	3.338032694	-1.84892
Gm20442	Gm20442-001	147	0.013763018	0.03541593	0.277657211	-3.601563218	0.463897705	1.670756912	-1.848623
Me3	Me3-003	1221.8223	0.000588282	0.003028509	0.277660083	-3.601525974	3.225605358	11.61710148	-1.848608
Dynll1	Dynll1-003	2095.7274	0.024315911	0.055278107	0.277691173	-3.60112275	6.091374784	21.93578831	-1.848447
Jmjd1c	Jmjd1c-011	320.37849	0.026985091	0.060038934	0.277699452	-3.60101539	0.921209802	3.317290676	-1.848404
P24-511F2.	24-511F2.1-(	781.07515	0.000439304	0.002420556	0.277765005	-3.600165542	1.734905755	6.24594792	-1.848063
Ccdc47	Ccdc47-001	22842.993	7.00755E-08	7.79249E-06	0.277797985	-3.599738129	56.18972122	202.2682819	-1.847892
Il18bp	Il18bp-201	1546	0.001047589	0.004720421	0.277826546	-3.599368079	3.026686134	10.89415745	-1.847744
Rbm3	Rbm3-004	13719.809	5.71929E-05	0.000531576	0.277864301	-3.598879005	39.74398477	143.0337924	-1.847548
Plbd2	Plbd2-003	981.74711	0.016709334	0.041216758	0.277931951	-3.598003026	2.350950266	8.458726169	-1.847196
Idua	Idua-002	1638.4587	0.013122152	0.034115206	0.278038731	-3.596621215	3.51736651	12.65063501	-1.846642
Etl4	Etl4-010	5173.0432	0.023760081	0.054277515	0.278049927	-3.596476404	13.30004823	47.83330963	-1.846584
Gm23638	Gm23638-201	174	0.031373146	0.067721072	0.278072864	-3.596179746	0.419900881	1.510039044	-1.846465
Tab2	Tab2-001	14633.801	2.79384E-07	1.66415E-05	0.278086848	-3.595998907	41.43166615	148.9882262	-1.846393
Pbdc1	Pbdc1-002	633.25808	0.004392569	0.014454792	0.278095479	-3.595887296	1.772941514	6.375297865	-1.846348
Gm10359	Gm10359-001	1294	0.002932498	0.010567745	0.27809771	-3.59585845	3.889203195	13.98502417	-1.846336
Rlim	Rlim-001	9046.3566	1.73012E-05	0.000227473	0.278129518	-3.595447212	24.78341095	89.1074458	-1.846171
Ogt	Ogt-007	6846.9791	3.21117E-05	0.00035134	0.278156241	-3.595101787	17.40700928	62.57997016	-1.846033
Ndufc1	Ndufc1-001	760.17206	0.000723267	0.003548671	0.278175215	-3.594856574	2.025146134	7.280109896	-1.845934
Aig1	Aig1-003	536.5885	0.002041012	0.007955462	0.278180939	-3.594782607	1.703339568	6.123135452	-1.845905
Utp11l	Utp11l-002	2958.9715	0.000110356	0.00085851	0.278182976	-3.594756279	8.521485035	30.63266183	-1.845894
Ick	Ick-001	11183.489	0.000105716	0.000830069	0.278219366	-3.594286097	18.01992669	64.76877195	-1.845705
Zfhx2	Zfhx2-003	564	0.01223942	0.0322859	0.278230316	-3.594144642	1.401958134	5.038840318	-1.845648
Sra1	Sra1-002	2013.6841	0.000839296	0.00397592	0.278250534	-3.593883484	4.760981413	17.11041247	-1.845544
Tsc22d2	Tsc22d2-004	3957.7402	1.78387E-05	0.000232423	0.278264104	-3.593708232	10.3573989	37.22146968	-1.845473
P23-162P5.	23-162P5.1-(	2242.2128	7.08061E-06	0.000122357	0.278269678	-3.593636247	5.658012507	20.33283883	-1.845444
Pcgf2	Pcgf2-004	364.51056	0.023182029	0.053276822	0.278347568	-3.592630636	0.967945997	3.477472441	-1.845041
Zfp229	Zfp229-005	878.08018	0.00428302	0.014171511	0.278347914	-3.592626168	2.048486977	7.35944792	-1.845039
Gpr107	Gpr107-012	1089.2389	4.74933E-05	0.000463828	0.278374732	-3.592280058	2.474075809	8.887573193	-1.8449
Ube2q1	Ube2q1-001	3116.0206	8.35874E-05	0.000701168	0.27839336	-3.592039696	8.552828804	30.72210058	-1.844803
Depdc5	Depdc5-004	504.74324	0.038046736	0.078966229	0.278435351	-3.591497981	1.6595881	5.96040731	-1.844586
Map4	Map4-002	60162.832	6.07145E-07	2.58528E-05	0.278757682	-3.587345083	126.3437467	453.2386183	-1.842917
Hnrnpr	Hnrnpr-003	3675.7898	0.000444784	0.002441711	0.278847814	-3.58618554	11.32137913	40.60056612	-1.84245
Araf	Araf-005	3303.0197	0.004252776	0.014093621	0.278853571	-3.586111509	7.866839851	28.21136493	-1.84242
Ormdl1	Ormdl1-001	493.49544	0.011750592	0.031306765	0.278891452	-3.585624414	1.493185069	5.354000839	-1.842224
Fbxl5	Fbxl5-007	1475.0655	0.001073979	0.004809593	0.278906895	-3.585425876	4.485571759	16.08268505	-1.842144
Pign	Pign-001	1029.1649	0.002699378	0.009895503	0.278967171	-3.584651191	2.827260664	10.13474331	-1.841833
Kras	Kras-001	11611.723	6.92988E-06	0.000120646	0.278978581	-3.584504574	18.64802496	66.84393075	-1.841774
Suz12	Suz12-201	2642.3868	0.009409995	0.026288117	0.278980785	-3.584476256	8.482146193	30.40405163	-1.841762
Gfm2	Gfm2-009	435.95735	0.040825053	0.083478093	0.278981502	-3.58446704	0.892295905	3.198405262	-1.841759
Gemin6	Gemin6-001	378.14447	0.049280601	0.096942196	0.279049825	-3.583589419	1.392197673	4.98906485	-1.841405
Cdk12	Cdk12-003	765.25853	0.000438202	0.002415647	0.279086765	-3.583115097	1.642709358	5.886016699	-1.841214
Acyp1	Acyp1-001	923.61423	8.32159E-05	0.000698908	0.279145732	-3.582358195	2.251544254	8.065838009	-1.84091
Clasp1	Clasp1-014	414	0.001575595	0.006498079	0.279177827	-3.58194636	0.970963449	3.477938991	-1.840744
Lamtor3	Lamtor3-002	777.40137	0.000621086	0.003161272	0.279191157	-3.58177534	1.914278544	6.856515683	-1.840675
00028K03R	0028K03Rik-	195.23558	0.026519376	0.059178793	0.279199935	-3.58166272	0.296628214	1.062422214	-1.840629
Cdc42	Cdc42-001	28779.146	1.19285E-06	3.90957E-05	0.279207723	-3.581562817	74.02607907	265.1290523	-1.840589
Htatsf1	Htatsf1-001	12632.329	6.02894E-07	2.58089E-05	0.279229556	-3.581282777	31.50715617	112.8360357	-1.840476
Nup160	Nup160-003	280.74806	0.042883393	0.086846984	0.279235851	-3.58120204	0.521278743	1.866804497	-1.840444
Dnajc21	Dnajc21-004	1763.8621	0.004321202	0.01427181	0.279277263	-3.580671016	4.659695993	16.68483839	-1.84023
Mtmr1	Mtmr1-201	3146.9142	0.000337348	0.001976762	0.279282433	-3.580604727	6.609888213	23.66739698	-1.840203
Psd3	Psd3-004	4938.7871	0.008237779	0.023626187	0.279291974	-3.580482409	9.138996755	32.72201712	-1.840154
Ulk2	Ulk2-001	5731.9625	3.80719E-06	8.16753E-05	0.279318208	-3.580146126	13.72982725	49.15478784	-1.840018
)15Ertd621	15Ertd621e-2(	13167	9.6244E-06	0.000150558	0.27933632	-3.579913994	38.55634937	138.0284147	-1.839925
Rcn2	Rcn2-002	989.7151	0.006269666	0.019090929	0.279380006	-3.579354202	3.011550902	10.77940759	-1.839699
Gm37775	Gm37775-001	376	0.002485641	0.009279833	0.279487029	-3.577983574	0.977032132	3.495804921	-1.839147
Klhl15	Klhl15-001	1002.8378	0.005245196	0.016626375	0.279492319	-3.577915856	2.728831619	9.76352992	-1.839119
Slc9a7	Slc9a7-002	1479.0549	2.23464E-05	0.000272894	0.279625266	-3.576214741	4.022868407	14.3866413	-1.838433
Atrx	Atrx-026	447.87438	0.002105861	0.008145016	0.279636549	-3.576070457	1.203943547	4.305386949	-1.838375
10013L24R	0013L24Rik-	11706	3.45273E-07	1.88049E-05	0.279697613	-3.575289722	30.46905743	108.9357079	-1.83806
Pdhb	Pdhb-201	4254	1.34022E-05	0.000189713	0.27970224	-3.575230569	9.837449333	35.17114958	-1.838036
Sgk1	Sgk1-001	5630.7056	0.000690893	0.003429931	0.279736991	-3.574786437	17.95681256	64.19177002	-1.837857
Nampt	Nampt-201	10745	3.81794E-08	5.27276E-06	0.279739358	-3.57475619	22.18632361	79.31069767	-1.837845
Cd81	Cd81-001	39633.077	8.78638E-06	0.000141883	0.279756317	-3.57453948	77.35896164	276.5226625	-1.837757
Sugp2	Sugp2-004	1119.5996	0.004258541	0.014109996	0.279756867	-3.57453245	3.085219303	11.02821652	-1.837755
Gm16503	Gm16503-201	1040	0.002146468	0.008268644	0.279846857	-3.573382991	2.967274278	10.60320743	-1.837291
Xpo6	Xpo6-003	2303.2897	0.000253641	0.001591489	0.279893589	-3.572786376	5.904720351	21.09630442	-1.83705
Wrn	Wrn-202	5549.7879	0.001169899	0.005152248	0.279897234	-3.572739845	12.83028648	45.83927571	-1.837031
Ube2f	Ube2f-002	2128.2896	0.002947325	0.010609132	0.279912257	-3.572548099	5.309966193	18.97010963	-1.836953
Tspan13	Tspan13-001	8597.5577	9.37811E-05	0.00076168	0.279944313	-3.572139006	23.11767636	82.57955348	-1.836788
Wdhd1	Wdhd1-002	569.95275	0.00128822	0.005561772	0.279944551	-3.572135972	1.784627749	6.374932978	-1.836787
Stx11	Stx11-202	1027.9641	0.006651956	0.019986796	0.280050858	-3.57077999	2.961329852	10.57425738	-1.836239
30004L10R	0004L10Rik-	277.78579	0.028604495	0.062835366	0.280073896	-3.570486263	0.61728367	2.204002866	-1.836121
P23-372C7.	23-372C7.4-(	1091	0.001833326	0.00731238	0.280120302	-3.569894765	2.629652807	9.38758379	-1.835882
Rab23	Rab23-001	1352.301	0.003011643	0.010789645	0.280142075	-3.569617307	3.126103274	11.15899235	-1.835769
Cul1	Cul1-001	11163.486	5.67586E-08	6.84501E-06	0.280168632	-3.56927895	27.24073696	97.22978903	-1.835633
Ikbkb	Ikbkb-013	851.12714	0.008560443	0.024361875	0.280218441	-3.568644508	2.253939793	8.043509861	-1.835376
Cpsf6	Cpsf6-003	1413.3416	0.000173674	0.001199986	0.280236452	-3.568415152	3.663235005	13.0719433	-1.835283
Prpf40a	Prpf40a-001	9677.9809	2.42808E-06	6.16537E-05	0.280248533	-3.568261317	27.79689464	99.18658386	-1.835221
30430E12R	0430E12Rik-	961.13788	0.000820359	0.003905657	0.280248832	-3.568257509	2.39797946	8.556608216	-1.83522
Gm17477	Gm17477-001	1011	0.000125715	0.00094949	0.280312965	-3.567441122	2.754479547	9.826443605	-1.83489
Git2	Git2-013	376.67878	0.004030328	0.013498802	0.280370008	-3.566715314	0.933290208	3.328780476	-1.834596
Nuf2	Nuf2-004	288.12748	0.023019172	0.052970079	0.280401359	-3.566316522	0.662229432	2.361719766	-1.834435
Rbm12b1	Rbm12b1-001	2623.6537	0.001178765	0.00518596	0.28041914	-3.566090392	6.000566148	21.39856129	-1.834343
Ict1os	Ict1os-001	405	0.003565358	0.012283827	0.280425405	-3.566010716	0.9001396	3.209907459	-1.834311
Hjurp	Hjurp-005	2769.3675	2.17891E-06	5.77613E-05	0.280506361	-3.56498155	7.495507245	26.72134504	-1.833895
Psmb4	Psmb4-003	529.98066	0.00066774	0.003341141	0.280513325	-3.564893035	1.471357778	5.245233094	-1.833859
Cab39	Cab39-005	526	0.024913108	0.056314352	0.280544092	-3.564502087	1.289486951	4.596378926	-1.833701

Glce	Glce-001	7966.6212	0.000296805	0.001794696	0.280637594	-3.563314466	17.87932807	63.70966837	-1.83322
Tprkb	Tprkb-003	1730.0825	0.003948807	0.013290577	0.280654894	-3.563094825	3.64951203	13.00355743	-1.833131
Gm38375	Gm38375-001	1328	0.000421363	0.002343565	0.280666813	-3.562943514	3.472652286	12.37286394	-1.83307
Prrg4	Prrg4-001	550.91768	0.040733843	0.083321436	0.280705124	-3.56245723	1.195828515	4.260087939	-1.832873
Casp1	Casp1-201	3622	6.31054E-06	0.000113636	0.280733877	-3.56209236	7.591347219	27.04107993	-1.832725
Zc4h2	Zc4h2-001	575.09242	0.021981153	0.051140402	0.280762212	-3.561732873	1.285202664	4.577548577	-1.832579
Psen1	Psen1-202	4428.389	6.82811E-05	0.000607002	0.280787944	-3.56140647	12.51057759	44.55525198	-1.832447
Tet3	Tet3-001	14262.072	2.35363E-07	1.55411E-05	0.280807104	-3.561163471	34.64621831	123.3808471	-1.832349
Prelid2	Prelid2-201	235	0.010558404	0.02877121	0.280874624	-3.560307393	0.429193879	1.52806214	-1.832002
Cops8	Cops8-002	1031.2739	0.007420109	0.021820103	0.280913279	-3.559817473	2.728471693	9.712861205	-1.831803
P23-114L12	23-114L12.4-	478	0.008360787	0.023910901	0.280997022	-3.55875657	0.851342544	3.029720871	-1.831373
Leo1	Leo1-201	3677.9758	0.00010364	0.000819077	0.281001037	-3.558705724	10.28743864	36.60996677	-1.831353
Eif2s3x	Eif2s3x-001	12626.844	7.58308E-07	2.96146E-05	0.281018029	-3.558490551	33.38606246	118.8039878	-1.831265
Gm14308	Gm14308-001	406.05615	0.006010491	0.018479294	0.281064237	-3.557905519	1.170982911	4.166246562	-1.831028
Atxn7l1	Atxn7l1-001	1618.6369	0.003115014	0.011063905	0.281103341	-3.55741058	4.252116319	15.12652358	-1.830827
Gm37513	Gm37513-001	684	0.01270452	0.033235389	0.281110132	-3.557324641	1.658722327	5.900613804	-1.830793
Cables2	Cables2-001	1984.7894	0.000247648	0.001564936	0.281125152	-3.557134576	5.943475547	21.14174237	-1.830716
Spata22	Spata22-002	201	0.01651178	0.040802919	0.281162121	-3.556666872	0.482024748	1.714401454	-1.830526
Il2ra	Il2ra-001	905	0.000257411	0.001609613	0.281188597	-3.556331974	2.234061335	7.945063756	-1.83039
Tulp1	Tulp1-005	151	0.012264889	0.032338142	0.281207658	-3.556090927	0.335131547	1.191758253	-1.830292
Arfgap1	Arfgap1-011	556.19217	0.011583862	0.030954768	0.281264262	-3.555375263	1.766916991	6.28205296	-1.830002
Pomk	Pomk-201	1844	0.00074125	0.003613924	0.281296372	-3.554969415	5.090553417	18.0967617	-1.829837
Scpep1os	Scpep1os-001	415	0.002316857	0.008793478	0.281306677	-3.554839194	1.111652197	3.951744802	-1.829784
Ei24	Ei24-001	6935.3375	8.65724E-06	0.000140327	0.281308443	-3.554816868	16.2516643	57.77169038	-1.829775
Cspp1	Cspp1-004	2610.5356	0.000340131	0.001988103	0.281321168	-3.554656075	7.652920292	27.20349961	-1.82971
Nelfa	Nelfa-001	5287.0523	2.63791E-06	6.49989E-05	0.281355696	-3.554219854	14.43642906	51.31024278	-1.829533
P23-465A17	23-465A17.7-	792	0.003334071	0.011666523	0.281400492	-3.553654057	2.430334339	8.636567483	-1.829303
Heg1	Heg1-002	693.82343	0.010231383	0.028099179	0.281434814	-3.553220677	1.714407719	6.091668955	-1.829127
Tm9sf4	Tm9sf4-003	1072.0351	0.003235138	0.01139719	0.281463541	-3.552858019	3.381474665	12.01389938	-1.82898
Pi4ka	Pi4ka-002	1599.1958	0.000125721	0.00094949	0.281473832	-3.552728126	4.402252761	15.6400072	-1.828927
Zfp260	Zfp260-002	5730.2977	1.79118E-05	0.000232908	0.281483654	-3.552604162	13.74861543	48.84338841	-1.828877
Lpgat1	Lpgat1-001	5995.3294	4.40392E-06	8.9569E-05	0.281497343	-3.5524314	19.2880919	68.51962333	-1.828807
Nxt2	Nxt2-004	1150.269	0.000579788	0.002996941	0.281499217	-3.552407749	3.166207421	11.24765978	-1.828797
Omg	Omg-001	463	0.022842292	0.052658168	0.281520539	-3.552138701	1.17950959	4.189781661	-1.828688
Tasp1	Tasp1-001	316.81031	0.027632935	0.061174329	0.281522448	-3.552114608	0.896347104	3.183927641	-1.828678
Twf1	Twf1-001	9846.191	8.15467E-07	3.10331E-05	0.28153616	-3.551941602	27.71321045	98.43570514	-1.828608
Zc3h7a	Zc3h7a-003	5956.8563	9.69863E-07	3.43159E-05	0.281539497	-3.551899504	14.49883027	51.49838804	-1.828591
Cyp2u1	Cyp2u1-001	2114.1217	6.12207E-05	0.000558554	0.281585959	-3.55131344	5.550572143	19.71182145	-1.828353
Gm38218	Gm38218-001	741	0.015905802	0.039629373	0.281611597	-3.550990122	1.438442715	5.107895873	-1.828221
Ctsh	Ctsh-002	7556.4603	0.000135956	0.001006159	0.281626787	-3.550798594	14.76717903	52.43527854	-1.828144
Hif1a	Hif1a-002	817.04918	0.04625568	0.092225931	0.281644245	-3.550578501	2.780661976	9.872958631	-1.828054
Gm38104	Gm38104-001	206	0.040844783	0.083507685	0.2816741	-3.550202166	0.487959368	1.732354407	-1.827901
Gm15950	Gm15950-001	398	0.008501839	0.024230941	0.281686427	-3.550046807	1.139017183	4.043564312	-1.827838
Csde1	Csde1-008	4169.8806	1.43383E-05	0.00019874	0.281686668	-3.550043772	11.91871888	42.31197375	-1.827837
Klhl28	Klhl28-201	1944	0.000260855	0.001624711	0.281863309	-3.547818994	4.723626486	16.75857177	-1.826932
Tmem39a	Tmem39a-002	1036.747	0.020030139	0.047548673	0.281994424	-3.546169407	3.087856791	10.95006329	-1.826261
Mocs2	Mocs2-002	736.00232	0.023882845	0.054507059	0.282003278	-3.546058072	1.661116868	5.890416877	-1.826216
Ugp2	Ugp2-001	1866.2554	0.002278985	0.008680521	0.28200349	-3.546055411	4.512212779	16.00055654	-1.826215
Rnf145	Rnf145-001	474.96653	0.021065812	0.04946891	0.282011334	-3.545956771	1.435916869	5.091699143	-1.826175
Ap4e1	Ap4e1-003	1928.0199	4.18654E-05	0.000424228	0.282019947	-3.545848479	4.373124349	15.50643632	-1.826131
Ube2l3	Ube2l3-201	8448.8167	1.76734E-05	0.000231124	0.282037063	-3.545633293	21.96267657	77.87159723	-1.826043
Wdhd1	Wdhd1-003	484.05842	0.003219793	0.011357148	0.282040308	-3.545592492	1.300954131	4.6126532	-1.826027
Zfp423	Zfp423-002	7821.4802	0.002898397	0.010477054	0.282076664	-3.545135516	12.3512233	43.78676039	-1.825841
Vcp	Vcp-003	363.87151	0.022606641	0.052245442	0.282139746	-3.544342875	0.95684845	3.391398986	-1.825518
Gtf3c6	Gtf3c6-201	2410	2.34372E-05	0.000281944	0.282151535	-3.54419479	6.8002296	24.10133832	-1.825458
Slc25a23	Slc25a23-002	1985.3019	3.37536E-05	0.00036377	0.282171918	-3.543938794	4.871112903	17.26292599	-1.825354
Surf2	Surf2-006	407.07947	0.030356828	0.065971595	0.282173281	-3.543921645	1.011764092	3.585612667	-1.825347
P24-122N23	24-122N23.3-	696.23135	0.000478693	0.002584371	0.282233762	-3.543162212	1.823768361	6.46190714	-1.825038
Gm15396	Gm15396-001	319.28562	0.002827426	0.010260578	0.282269615	-3.542713462	0.8245292	2.921070697	-1.824855
Gch1	Gch1-201	1726	8.73016E-05	0.000721814	0.282310472	-3.542199449	3.344614913	11.8472931	-1.824645
Ints4	Ints4-201	5635	2.8366E-06	6.80824E-05	0.282310712	-3.542196444	16.22776328	57.4819254	-1.824644
P24-68G17	24-68G17.3-(	894	0.003258636	0.011459933	0.282376795	-3.541367483	2.512635507	8.898165681	-1.824307
Mcl1	Mcl1-001	52130.773	1.57277E-07	1.24119E-05	0.282386701	-3.541243248	139.8325797	495.1811788	-1.824256
Nsfl1c	Nsfl1c-006	639.56776	0.005179546	0.01645177	0.282438385	-3.540595238	1.794494345	6.353578132	-1.823992
Gm24979	Gm24979-201	252	0.014845715	0.037597289	0.282438806	-3.540589963	0.650686886	2.303815457	-1.82399
Nol8	Nol8-201	6040	6.99013E-07	2.81865E-05	0.282447805	-3.540477148	19.63256006	69.50863022	-1.823944
Gm27017	Gm27017-001	259.64504	0.024691616	0.055931737	0.282451389	-3.540432223	0.613028405	2.170385517	-1.823925
Ccpg1	Ccpg1-010	300.39596	0.007615557	0.022272463	0.28247965	-3.540078024	0.971768272	3.440135505	-1.823781
Shprh	Shprh-003	1260.4876	0.00022338	0.001448183	0.282485164	-3.540008922	2.876976571	10.18452273	-1.823753
10001H17F	0001H17Rik-	435.64113	0.004617586	0.015034921	0.282512475	-3.539666702	1.410970429	4.994365045	-1.823614
Fosl2	Fosl2-001	17248.884	0.00144418	0.006078797	0.282575964	-3.538871408	66.06534246	233.7967515	-1.823289
Gstm2-ps1	Gstm2-ps1-00	630	0.016973129	0.041726218	0.282589056	-3.538707456	1.322516723	4.679999787	-1.823222
430049J08R	0049J08Rik-	701	0.003709904	0.012668416	0.282622456	-3.538289252	1.849868174	6.545368676	-1.823052
Rorc	Rorc-002	983.822	0.049330007	0.097000387	0.282639216	-3.538079439	2.959340209	10.47038075	-1.822966
Bptf	Bptf-001	16054.351	0.001217428	0.005318482	0.282653503	-3.537900614	40.39393585	142.9097304	-1.822894
Cenpc1	Cenpc1-001	3395.8757	2.04631E-06	5.49695E-05	0.282656624	-3.537861548	9.520140464	33.68093888	-1.822878
Tmem128	Tmem128-001	1446.6315	4.72063E-05	0.000461683	0.2826649	-3.537757958	3.780734509	13.3753236	-1.822835
31406C07R	1406C07Rik-	1858.3654	0.00038755	0.002197977	0.282688002	-3.537468843	4.442893783	15.71659833	-1.822717
Pitpnm2	Pitpnm2-006	679.32935	0.007173628	0.02123384	0.282688381	-3.5374641	1.8782053	6.64408382	-1.822716
Txn2	Txn2-003	548.86636	0.013095161	0.03405796	0.28274168	-3.536797264	1.565845832	5.538079255	-1.822444
Rnf219	Rnf219-201	3036	5.69289E-06	0.000106199	0.282763356	-3.53652614	8.791683435	31.09201829	-1.822333
Cspp1	Cspp1-002	1278.6031	6.57454E-05	0.000589775	0.282773342	-3.536401246	3.896039149	13.7779577	-1.822282
Mllt3	Mllt3-002	2153.1548	0.000734137	0.003586836	0.282781642	-3.536297452	5.640354359	19.94597075	-1.82224
Commd7	Commd7-001	3218.914	0.000257173	0.001608796	0.282792113	-3.536166507	9.592318398	33.92003504	-1.822186
Ppm1b	Ppm1b-202	4186.3045	6.61366E-06	0.000116822	0.282854441	-3.53538731	11.65428222	41.20240146	-1.821868
Zmym2	Zmym2-201	16270	1.8246E-08	3.35982E-06	0.282892981	-3.534905658	41.95293589	148.2996704	-1.821672
Ikbkb	Ikbkb-010	8805.8374	3.91652E-07	2.01715E-05	0.282921847	-3.534544998	21.84723456	77.22003362	-1.821525
Tcea1	Tcea1-001	8762.2838	1.02461E-06	3.5458E-05	0.282993891	-3.533645178	24.19018291	85.4795232	-1.821157
Strbp	Strbp-003	2047.1516	0.00103124	0.004666874	0.283014033	-3.533393697	4.48440514	15.84516886	-1.821055
Eif2d	Eif2d-004	988.02317	0.007138411	0.021155195	0.28302767	-3.533223444	1.670377002	5.901815186	-1.820985
Prrc2c	Prrc2c-006	81695.308	2.29975E-06	5.95363E-05	0.283069536	-3.532700887	220.2253684	777.9903544	-1.820772
Rcor1	Rcor1-001	6463.249	1.14479E-06	3.81412E-05	0.283076622	-3.532612458	16.80428941	59.36304211	-1.820735
P23-393A24	23-393A24.1-	5677	2.94292E-06	6.96579E-05	0.283184358	-3.531268493	14.22011943	50.21505972	-1.820187
Sppl2a	Sppl2a-002	1091.0047	0.001182744	0.005199458	0.283242821	-3.530539616	2.350665529	8.299117776	-1.819889
730081D07F	0081D07Rik-	625.52588	0.004581527	0.014945927	0.283252222	-3.53042244	1.8605765	6.568621026	-1.819841
Clk4	Clk4-014	5476.6749	7.87158E-06	0.000131475	0.283254614	-3.530392627	13.30738279	46.98028608	-1.819829
530075F10F	0075F10Rik-	282	0.008836425	0.024983287	0.283298524	-3.529845428	0.60918559	2.15033097	-1.819605
Gstcd	Gstcd-001	582.05021	0.014010292	0.035917105	0.283299213	-3.529836842	1.908092981	6.735256904	-1.819601
Mysm1	Mysm1-002	3222.4239	9.52365E-05	0.000770031	0.283417836	-3.528359446	8.317020012	29.34543612	-1.818998
Tpgs2	Tpgs2-001	1707.938	0.000188082	0.001273585	0.28346118	-3.52781993	4.069437912	14.35624417	-1.818777
10417H13F	0417H13Rik-	915.31632	0.000228642	0.001474211	0.283475956	-3.527636047	2.348747944	8.285527913	-1.818702
Gm15459	Gm15459-001	31423.779	0.000478604	0.002584295	0.283479324	-3.527594136	105.8863769	373.5241624	-1.818685
Tbce	Tbce-005	435.41907	0.013363345	0.034610901	0.283493836	-3.527413553	1.323725543	4.66932742	-1.818611
Senp7	Senp7-004	2432.5146	5.83169E-05	0.000539534	0.283552797	-3.526680071	6.746378907	23.79232004	-1.818311
Nploc4	Nploc4-002	2320.1089	0.000868552	0.004086456	0.283595661	-3.526147032	6.416144547	22.62426906	-1.818093
BC052040	BC052040-00	433.67529	0.012674415	0.033174097	0.283658994	-3.52535975	1.010758226	3.563286367	-1.81777
Zfp330	Zfp330-201	5280	6.90947E-06	0.000120432	0.283663896	-3.525298823	14.87715357	52.44641197	-1.817746

Hipk2	Hipk2-004	5767.5293	3.42395E-06	7.66974E-05	0.283677178	-3.525133775	16.13013734	56.86089191	-1.817678
Kcnj13	Kcnj13-001	470.24476	0.010290648	0.02821669	0.2837742	-3.52392853	1.299673153	4.579955302	-1.817185
Edem1	Edem1-201	9279	0.000189496	0.001279113	0.283784226	-3.523804039	27.38817604	96.51056533	-1.817134
Cald1	Cald1-003	1569.6637	0.011295371	0.03032344	0.283841003	-3.523099159	4.955996686	17.46046776	-1.816845
Gm12671	Gm12671-001	1322	0.001307275	0.005622778	0.283899483	-3.522373447	4.078084008	14.36453483	-1.816548
Ercc3	Ercc3-002	2057.9716	0.000334149	0.00196137	0.283914886	-3.522182347	5.279520054	18.59543234	-1.81647
Ankrd13a	Ankrd13a-00(	1260.7338	0.009558777	0.026597457	0.28393085	-3.521984308	3.666134431	12.91206794	-1.816388
Lhfp	Lhfp-004	452	0.003745082	0.012752937	0.283948644	-3.521763609	1.141028101	4.018431244	-1.816298
Ggnbp2	Ggnbp2-001	807.61172	0.030239083	0.065758007	0.283948957	-3.521759718	2.172956444	7.652630474	-1.816296
Wwtr1	Wwtr1-002	18922.692	8.49712E-07	3.18758E-05	0.283997847	-3.52115346	53.85435896	189.6294624	-1.816048
210408I21R	0408I21Rik-(	1295	0.000361407	0.002081386	0.284041004	-3.520618453	3.334218632	11.73851164	-1.815829
10040B10R	0040B10Rik-	181.88326	0.028249914	0.062210478	0.284051654	-3.520486456	0.452109178	1.591644239	-1.815775
Cdc26	Cdc26-002	737.13972	0.007044273	0.020934173	0.284113834	-3.519715975	1.877132477	6.606973168	-1.815459
Kif1b	Kif1b-002	1612.2437	8.94627E-05	0.000735325	0.284117902	-3.51966558	4.177632023	14.70386763	-1.815438
Clcn3	Clcn3-010	5438.0644	2.74501E-05	0.000315598	0.284149055	-3.51927969	13.08559241	46.05185961	-1.81528
Ndufs1	Ndufs1-001	3230.288	0.002046821	0.007970902	0.284154677	-3.519210065	9.691734233	34.10724866	-1.815252
Shc1	Shc1-201	3712.0683	0.000274985	0.001689408	0.284168344	-3.519040806	9.29251116	32.70072596	-1.815182
Gm13298	Gm13298-001	207	0.009558268	0.026597457	0.284183462	-3.518853602	0.671849124	2.364138709	-1.815105
Rgs19	Rgs19-001	1764.0336	0.001615722	0.006618642	0.284202057	-3.518623367	5.744338409	20.21216335	-1.815011
Gm13961	Gm13961-001	177	0.036405626	0.076285878	0.284208326	-3.518545761	0.393276657	1.383761915	-1.814979
Snx13	Snx13-201	10501.318	1.49694E-07	1.20353E-05	0.284288592	-3.517552332	24.96259142	87.80722167	-1.814572
Tlr2	Tlr2-001	1962	0.000173642	0.001199986	0.28431258	-3.517255545	6.339856975	22.2988971	-1.81445
Naca	Naca-202	5599.9772	1.21495E-05	0.000177148	0.284398414	-3.516194017	14.07149797	49.47811696	-1.814015
Drg1	Drg1-002	1164.9262	0.009136802	0.025692568	0.284419795	-3.515929679	2.918567329	10.26147749	-1.813906
Hltf	Hltf-001	2032.281	0.008075358	0.023294956	0.284457853	-3.515459283	4.95222309	17.40933863	-1.813713
Smim14	Smim14-002	1689.7333	0.000446176	0.002448179	0.284483592	-3.515141219	3.683467203	12.94790739	-1.813583
S100a7a	S100a7a-001	417	0.010766926	0.029186166	0.284552817	-3.514286066	1.118260547	3.929887458	-1.813232
Eif2d	Eif2d-002	587.66771	0.015669771	0.039174427	0.284564057	-3.514147252	1.844053717	6.480276304	-1.813175
Akap7	Akap7-003	433.34021	0.00184221	0.00733859	0.28464438	-3.513155605	1.009889171	3.547897803	-1.812767
Nktr	Nktr-004	1971.783	7.66067E-05	0.000659821	0.28469398	-3.512543542	4.375495914	15.36911991	-1.812516
Lats2	Lats2-001	21392.022	1.65143E-06	4.74002E-05	0.284732045	-3.512073959	53.48168878	187.8316464	-1.812323
Ghr	Ghr-004	599.74704	0.009084724	0.02556751	0.284755414	-3.511785736	1.152954443	4.048928968	-1.812205
Gm10273	Gm10273-201	1088	0.001721911	0.006952193	0.284759374	-3.511736899	3.657731485	12.84499062	-1.812185
Cep95	Cep95-001	1100.2642	0.033724676	0.071779155	0.284781438	-3.511464813	3.300731443	11.59040232	-1.812073
Tmem154	Tmem154-001	3485.6533	1.90084E-05	0.000243561	0.284782638	-3.511450017	10.44923756	36.6919754	-1.812067
Vmp1	Vmp1-001	4789.1955	0.005695523	0.017712854	0.28479664	-3.511277374	18.06323954	63.42504431	-1.811996
Pfdn2	Pfdn2-001	1169.961	0.000791828	0.003797609	0.284807916	-3.511138369	3.186495849	11.18822784	-1.811939
Oxct1	Oxct1-001	5394.9969	4.45035E-05	0.00044361	0.284848538	-3.510637643	15.98168156	56.10589288	-1.811733
P23-57A17	23-57A17.2-(	256	0.034443227	0.073000032	0.284918307	-3.50977798	0.948991712	3.330750214	-1.81138
Frmd4a	Frmd4a-007	3590.1668	2.16635E-05	0.000266994	0.284930863	-3.50962332	11.60013597	40.71210771	-1.811316
Heatr6	Heatr6-001	2242.2287	0.000603673	0.003091014	0.284938315	-3.509531527	5.721610076	20.08017095	-1.811278
Tnks2	Tnks2-005	358.82201	0.024630555	0.055834039	0.284948432	-3.509406917	1.020215441	3.580351125	-1.811227
Hilpda	Hilpda-001	646.26877	0.011721212	0.031257743	0.284985929	-3.508945171	2.097636609	7.360491849	-1.811037
Chp1	Chp1-001	9395.3143	2.48513E-08	4.03392E-06	0.285002508	-3.508741054	24.67940964	86.59365777	-1.810953
Ppa1	Ppa1-001	2008.6687	0.043567949	0.087921415	0.285004993	-3.508710457	7.861569305	27.58397043	-1.810941
Nsmce2	Nsmce2-201	3250.7611	5.0421E-05	0.000483731	0.285005904	-3.508699241	8.986481999	31.53086257	-1.810936
Fam133b	Fam133b-002	1462.9975	0.001152794	0.005096578	0.285022446	-3.508495605	4.485328518	15.73675539	-1.810853
P24-347B12	24-347B12.1-	745	0.003065337	0.010930537	0.285024918	-3.508465181	1.866969235	6.550196556	-1.81084
Rabep1	Rabep1-001	3918.7184	7.44368E-06	0.000126423	0.285043043	-3.508242092	9.746486567	34.19303442	-1.810748
Rars2	Rars2-001	888.65438	0.015807591	0.039452743	0.285047661	-3.508185253	2.453287227	8.606586071	-1.810725
Nek6	Nek6-001	2251.1021	0.00018975	0.001280327	0.285128436	-3.507191402	7.2530412	25.43780373	-1.810316
Nin	Nin-001	10920.529	1.01375E-05	0.000155839	0.285227902	-3.505968362	32.65592175	114.4906285	-1.809813
Cstf2	Cstf2-001	1779.912	0.002652156	0.009763151	0.285274724	-3.505392928	4.234140346	14.84232563	-1.809576
Rbpms	Rbpms-002	8414.191	0.000983333	0.004489954	0.285280749	-3.505318891	25.73401406	90.20592565	-1.809546
Psd3	Psd3-002	2135.2099	0.011046944	0.029801343	0.285284572	-3.505271918	3.236176896	11.34368	-1.809526
Chuk	Chuk-001	1870.3195	0.001013063	0.004603	0.285290793	-3.505195485	5.188956584	18.18830719	-1.809495
Sema6a	Sema6a-006	1876.8434	0.018833368	0.045320231	0.285310106	-3.504958212	5.442041867	19.07412933	-1.809397
Rnf44	Rnf44-004	1769.7986	0.003686527	0.012606185	0.285323951	-3.504788147	5.175577859	18.13930393	-1.809327
Zfp459	Zfp459-001	454	0.002370149	0.00894125	0.285330532	-3.504707304	1.10101174	3.858723888	-1.809294
Vldlr	Vldlr-001	275.92077	0.039612701	0.081451243	0.285419985	-3.503608896	0.555483849	1.946198156	-1.808842
Zfp418	Zfp418-001	890	0.009707708	0.026917826	0.285436407	-3.503407324	2.054096986	7.196338426	-1.808759
Cdc16	Cdc16-003	883.37448	0.029600313	0.064606852	0.285438126	-3.503386224	2.508729828	8.789049518	-1.80875
Xpr1	Xpr1-003	3217.3918	0.002505455	0.009335104	0.285444832	-3.50330392	7.254316896	25.41407682	-1.808716
Pbx2	Pbx2-001	1979.4897	2.25218E-05	0.000274314	0.285466723	-3.503035269	5.597532442	19.60835356	-1.808606
Cbln3	Cbln3-201	648	0.000705659	0.003486287	0.285500305	-3.502623231	1.815528905	6.35911372	-1.808436
Pecam1	Pecam1-009	39863.615	0.000321102	0.001903199	0.285570991	-3.501756241	82.17785895	287.7668305	-1.808079
Gm38224	Gm38224-001	1655	3.2359E-05	0.000353636	0.285576552	-3.501688053	4.141273526	14.50144803	-1.808051
Stxbp5	Stxbp5-005	4179.1789	0.005278857	0.016719145	0.285603931	-3.501352364	9.526114964	33.35428515	-1.807912
Rps15a	Rps15a-001	4475.9502	6.71507E-06	0.000118369	0.285618583	-3.501172752	13.12615731	45.95694431	-1.807838
Qrich1	Qrich1-005	1539.3374	0.000676287	0.003370122	0.285671958	-3.500518592	3.889004739	13.61353339	-1.807569
Gm6733	Gm6733-001	961	0.000730927	0.00357428	0.285700572	-3.500168007	2.214167548	7.749958415	-1.807424
Tmem252	Tmem252-201	27080	8.72395E-05	0.000721553	0.285762537	-3.499409027	67.72771202	237.0069668	-1.807111
Nup214	Nup214-008	255.34862	0.032852009	0.07026674	0.285762611	-3.499408111	0.74767747	2.616428602	-1.807111
Atp13a2	Atp13a2-003	1582.8363	0.000116233	0.000893766	0.285770754	-3.499308398	4.048927122	14.16844468	-1.80707
Gstm5	Gstm5-001	307.26418	0.015419043	0.038694478	0.285793183	-3.499033773	0.593136861	2.075405909	-1.806957
Abi3bp	Abi3bp-201	2169.0757	0.042751177	0.086620196	0.285816043	-3.498753912	6.391539815	22.36242494	-1.806841
Whsc1l1	Whsc1l1-008	1261.4258	0.000604429	0.003094003	0.285851833	-3.498315855	3.636432609	12.72138985	-1.806661
Taf5	Taf5-201	1694	2.78294E-05	0.000318888	0.285873792	-3.498047139	4.82691649	16.88478142	-1.80655
Gm18821	Gm18821-001	678	0.025493263	0.057353144	0.285884242	-3.49791927	1.728198717	6.045099594	-1.806497
Gm26563	Gm26563-202	213	0.019080235	0.04576349	0.285886836	-3.497887538	0.541991658	1.895825866	-1.806484
Fgf2	Fgf2-007	10006.327	5.25604E-05	0.000499118	0.285944585	-3.497181106	20.71975941	72.46075114	-1.806193
Gm5559	Gm5559-001	1776	0.002481153	0.009268803	0.285965055	-3.496930771	5.688204769	19.89125829	-1.806089
Gm8186	Gm8186-201	2389	0.000204171	0.001352583	0.285979871	-3.496749604	7.225360109	25.2652751	-1.806014
Mbnl1	Mbnl1-008	5243.8751	0.000278882	0.001708104	0.285994954	-3.496565182	13.63220636	47.66589811	-1.805938
Npy1r	Npy1r-201	687	0.000583196	0.00301047	0.286012225	-3.496354043	1.864327667	6.518349577	-1.805851
Suv420h1	Suv420h1-00(	275.43796	0.015372103	0.038616293	0.286039678	-3.496018483	0.62771126	2.194490167	-1.805713
Meaf6	Meaf6-001	3067.4776	3.38783E-05	0.000364481	0.286154157	-3.494619865	8.124677103	28.392658	-1.805136
Cluh	Cluh-001	3355.7942	0.001055169	0.004748332	0.286190098	-3.494180992	9.131568774	31.90735404	-1.804954
Srpk1	Srpk1-004	4381.6377	4.54119E-07	2.20013E-05	0.286199785	-3.494062719	11.7311966	40.9895367	-1.804906
Sp2	Sp2-201	1712.5107	0.003390161	0.011803955	0.286200078	-3.49405914	4.969754572	17.36461638	-1.804904
Gm16892	Gm16892-201	2388.2504	1.00549E-05	0.000155177	0.286201603	-3.494040531	6.48272563	22.6509061	-1.804896
Cdc23	Cdc23-003	501.29861	0.001130611	0.005016637	0.286213675	-3.493893158	1.629863432	5.694568695	-1.804835
H25-194I22	25-194I22.2-(	494	0.007575274	0.022170947	0.286229682	-3.493697758	1.207211439	4.2176319	-1.804755
Bloc1s6	Bloc1s6-001	338.04885	0.005057542	0.016142093	0.286277802	-3.493110517	0.840961506	2.937571483	-1.804512
Rad23a	Rad23a-001	2209.8124	0.01038015	0.028423421	0.286310862	-3.492707172	5.521146926	19.28374947	-1.804346
Cfhr2	Cfhr2-002	9042.5449	0.031947669	0.06870143	0.28636725	-3.492019425	31.64287662	110.4975398	-1.804062
Sec61a2	Sec61a2-007	162.63525	0.013881274	0.035645048	0.28638978	-3.491744711	0.352851586	1.23206766	-1.803948
Ldha	Ldha-001	21738.036	0.000126542	0.000953383	0.286398667	-3.49163636	63.23428494	220.7911285	-1.803903
Sypl	Sypl-003	1736.1746	0.005492558	0.017241325	0.286400061	-3.491619373	4.147828465	14.48263823	-1.803896
Gm29216	Gm29216-001	268708	3.92804E-08	5.35998E-06	0.286420339	-3.491372169	688.5784411	2404.083606	-1.803794
Gm14827	Gm14827-001	198	0.012087561	0.031996211	0.286436438	-3.491175938	0.506962942	1.769896826	-1.803713
Ppp3ca	Ppp3ca-002	11878.651	0.000129744	0.000970674	0.286442453	-3.491102629	25.65591221	89.56742256	-1.803683
Cdc123	Cdc123-001	7250.33	4.94945E-07	2.32217E-05	0.286446883	-3.491048629	18.12954808	63.29113398	-1.80366
Tmem64	Tmem64-001	10584	7.49054E-05	0.000648511	0.286507657	-3.490308112	24.83954072	86.69765046	-1.803354
U2surp	U2surp-007	1565.4757	0.000152955	0.001096918	0.286517012	-3.490194156	3.944247406	13.76618924	-1.803307
Iqcb1	Iqcb1-004	948.68708	0.000198281	0.001324324	0.286593117	-3.489267335	2.553025164	8.908187311	-1.802924
Mkrn1	Mkrn1-002	494.02362	0.023495211	0.053819621	0.286607502	-3.489092201	1.445961776	5.045093957	-1.802852

Ppp6c	Ppp6c-001	6764.018	1.8119E-07	1.33171E-05	0.286613838	-3.48901507	17.50989851	61.09229978	-1.80282
Slc12a6	Slc12a6-005	1269.2595	0.028464728	0.062603027	0.286688285	-3.48810904	3.359369069	11.71784562	-1.802445
30114K14R	0114K14Rik-	684.31152	0.000469716	0.002549566	0.286732057	-3.48757656	1.564728791	5.457111453	-1.802225
Dpp8	Dpp8-201	25514.324	4.24188E-07	2.11169E-05	0.286739821	-3.48748212	62.84770915	219.1802619	-1.802186
Brcc3	Brcc3-005	979.48733	0.002058835	0.008007618	0.286760816	-3.487226796	3.229831079	11.26315348	-1.80208
Zbtb34	Zbtb34-001	2302.3448	0.000324518	0.001917656	0.286765495	-3.487169888	6.55491511	22.85810259	-1.802057
Fam63b	Fam63b-201	38117.577	0.001416681	0.00598733	0.286840761	-3.486254872	78.16691709	272.5097955	-1.801678
Zfp780b	Zfp780b-201	1277	0.000746193	0.003631616	0.286882087	-3.485752669	3.468688916	12.09099165	-1.80147
Fam103a1	⁼am103a1-00	3906.4765	1.00055E-05	0.000154609	0.286951098	-3.484914357	10.20760157	35.57261727	-1.801123
Ric1	Ric1-003	1008.5742	0.026210701	0.058632129	0.287010057	-3.484198465	2.725189536	9.495101199	-1.800827
Irf9	Irf9-004	1088.7412	0.003047884	0.010889843	0.287010685	-3.484190846	2.588881709	9.020157951	-1.800824
Pon2	Pon2-002	1008.1635	0.000981346	0.004482674	0.287061989	-3.483568144	2.164123995	7.538873409	-1.800566
Atp5b	Atp5b-003	1178.233	0.000213411	0.001399954	0.28707055	-3.483464261	3.186622544	11.10048574	-1.800523
Gm17644	Gm17644-001	7666	7.09783E-06	0.000122592	0.287106137	-3.483032479	18.40486538	64.10474391	-1.800344
R3hdm4	R3hdm4-201	4207.814	1.12697E-05	0.00016826	0.287121645	-3.482844351	11.0003704	38.31257791	-1.800266
Nckap1	Nckap1-002	5210.0821	0.000111973	0.000868059	0.287151401	-3.482483446	13.94160435	48.55140637	-1.800116
33407K13R	3407K13Rik-	1601.3242	0.001483339	0.006203978	0.287160829	-3.482369108	3.472777848	12.0934943	-1.800069
Ddx3x	Ddx3x-004	1270.9959	2.11486E-05	0.000263109	0.287183864	-3.48208979	3.610347363	12.57155369	-1.799953
Ugdh	Ugdh-004	2042.3713	0.000783551	0.003766542	0.287241544	-3.481390555	5.957095371	20.73897556	-1.799664
Ubap2l	Ubap2l-014	661.40006	0.002870547	0.010393936	0.287258237	-3.481188256	1.783968412	6.210329884	-1.79958
Rab24	Rab24-201	3775	5.26525E-06	0.000100729	0.287275679	-3.480976895	10.39973993	36.20125439	-1.799492
Dtymk	Dtymk-002	722.6598	0.004706143	0.015249329	0.287303254	-3.480642788	1.881960552	6.550432421	-1.799354
Hypk	Hypk-003	701.18904	0.003714661	0.012682129	0.287320425	-3.480434774	2.115048168	7.361287194	-1.799268
Gorasp2	Gorasp2-001	6185.5326	0.001531495	0.006351657	0.287340587	-3.480190564	17.62345611	61.33298565	-1.799166
Arhgap1	Arhgap1-001	2211.6492	0.011549382	0.030893966	0.287364796	-3.479897379	6.304757908	21.93991052	-1.799045
Wls	Wls-007	393.96077	0.025438207	0.057260136	0.287458998	-3.478756998	1.050449925	3.654260029	-1.798572
Ppwd1	Ppwd1-201	2564	7.3913E-05	0.000643334	0.287475843	-3.478553157	6.692299085	23.27951811	-1.798487
Mbd4	Mbd4-001	1347.1006	0.004311992	0.014249878	0.287557188	-3.477569132	3.295961271	11.46193318	-1.798079
Nfat5	Nfat5-001	30595.323	3.37316E-05	0.000363648	0.287560068	-3.477534304	75.01735392	260.8754216	-1.798065
Spop	Spop-002	499.935	0.025512662	0.057393015	0.287564385	-3.477482099	1.80379623	6.272669102	-1.798043
Vrk2	Vrk2-003	245.41996	0.019786475	0.047117206	0.287615729	-3.476861312	0.481489261	1.674071383	-1.797786
Alg2	Alg2-001	3711.0821	1.78974E-05	0.000232809	0.287655268	-3.476383401	9.32342555	32.41180182	-1.797587
Txnl1	Txnl1-201	7473	3.87365E-06	8.24052E-05	0.287658836	-3.47634028	22.07206278	76.73000092	-1.797569
90002N15R	0002N15Rik-	14229	0.018454978	0.044572526	0.287694883	-3.475904715	23.19231558	80.61427909	-1.797389
Copg2	Copg2-201	1327.7333	0.000520762	0.002756312	0.287715386	-3.475657018	2.935905927	10.20420204	-1.797286
Med28	Med28-001	1433.7653	0.000672313	0.003355927	0.287719732	-3.475604518	2.974067086	10.336681	-1.797264
Rbpj	Rbpj-004	5219.3383	1.96876E-06	5.33805E-05	0.287798246	-3.474656334	13.88826178	48.25693678	-1.79687
Ppt1	Ppt1-001	9542.0793	2.38598E-07	1.55689E-05	0.287819916	-3.474394736	25.64464742	89.09962802	-1.796762
Apbb2	Apbb2-004	9741.7338	0.000635008	0.003214457	0.28783758	-3.474181519	20.29526321	70.50942839	-1.796673
Gm4070	Gm4070-003	496.33495	0.018786147	0.045219308	0.287860031	-3.47391055	1.290291787	4.482358251	-1.796561
00081O15R	0081O15Rik-	1784.7074	0.031111474	0.06729891	0.287880605	-3.473662282	4.637568383	16.10934637	-1.796457
Mrps10	Mrps10-004	730.44891	0.004640552	0.015093915	0.287885957	-3.473597703	2.380379559	8.268480967	-1.796431
Aida	Aida-001	9741.5706	1.38594E-06	4.2803E-05	0.287902771	-3.473394848	21.13612574	73.41411025	-1.796346
Gas5	Gas5-001	5256.4817	0.000697715	0.003457781	0.287923376	-3.473146272	17.19329149	59.71481624	-1.796243
Myo9a	Myo9a-003	7017.8872	6.63515E-05	0.00059345	0.288016002	-3.472029303	14.77071819	51.28436638	-1.795779
Zfp60	Zfp60-001	3687.0441	1.15757E-05	0.000171039	0.288076236	-3.471303336	9.421822387	32.70600349	-1.795477
Rab14	Rab14-001	15086.22	2.98523E-07	1.72269E-05	0.288093288	-3.471097875	38.47980027	133.567153	-1.795392
Cyb5r1	Cyb5r1-002	916.66231	0.023958037	0.05462407	0.28809666	-3.471057254	1.67950723	5.829665753	-1.795375
Lin37	Lin37-001	772.1233	0.019397095	0.046377	0.288106142	-3.470943005	2.085422706	7.238383353	-1.795328
Vwa9	Vwa9-202	3324.3891	0.00027238	0.00167818	0.288208276	-3.469712991	9.194516104	31.90233197	-1.794816
Ino80d	Ino80d-002	1491.5812	0.015722451	0.039274593	0.288210079	-3.469691291	5.200429164	18.04388378	-1.794807
S100a11	S100a11-001	38113	0.000238678	0.001520672	0.288235425	-3.469386177	106.1098742	368.1361307	-1.79468
Lgals8	Lgals8-008	1168.1754	0.00951053	0.026504121	0.288246598	-3.469251699	2.567860873	8.908555697	-1.794625
Ccdc14	Ccdc14-201	639	0.012858274	0.033556528	0.288256622	-3.469131061	1.960249431	6.800362189	-1.794574
Med1	Med1-001	16928.925	3.82999E-06	8.17302E-05	0.288278588	-3.468866724	48.1824371	167.1384527	-1.794464
'00030L20R	0030L20Rik-	140	0.0207551	0.048880022	0.288279353	-3.468857511	0.267612725	0.928310413	-1.794461
Purb	Purb-001	22060.426	2.4873E-06	6.24561E-05	0.288349952	-3.468008207	60.68402514	210.4526972	-1.794107
Eef1a1	Eef1a1-005	1063.7432	0.009985603	0.027547879	0.2883556	-3.467940278	3.271153227	11.34416403	-1.794079
Pdlim1	Pdlim1-002	3989.0709	3.91784E-06	8.30358E-05	0.288374552	-3.467712368	10.60913493	36.78942841	-1.793984
Gm6206	Gm6206-001	1855	1.3376E-05	0.000189544	0.2883892	-3.467536233	4.973605184	17.24615619	-1.793911
Nhlrc3	Nhlrc3-005	953.65708	0.003119472	0.01107285	0.288391418	-3.467509568	2.232021278	7.739555136	-1.7939
Ntan1	Ntan1-008	1922.4754	0.001031354	0.004666874	0.288414039	-3.467237603	4.066911718	14.10094924	-1.793787
Cage1	Cage1-003	408.79732	0.012486108	0.032795184	0.28844364	-3.466881781	0.963848032	3.341547182	-1.793639
Pigl	Pigl-001	1963.9766	0.000112622	0.000871924	0.28844453	-3.466871079	5.308581675	18.40416828	-1.793634
Mrpl11	Mrpl11-201	1513	0.000123672	0.000937761	0.288470765	-3.466555792	3.927359794	13.61441184	-1.793503
Mrps9	Mrps9-010	400.86908	0.032710787	0.070021529	0.288477524	-3.466474572	1.167736417	4.047928595	-1.793469
Stard4	Stard4-001	6286.0889	5.86778E-06	0.000108083	0.288492317	-3.466296812	15.48088724	53.6613501	-1.793395
Bclaf1	Bclaf1-008	18999.4	2.16893E-07	1.48571E-05	0.288534708	-3.46578755	50.32352456	174.4106449	-1.793183
Ppp1r2	Ppp1r2-001	11340.337	5.51247E-07	2.4488E-05	0.288555784	-3.465534416	23.93629803	82.95206461	-1.793078
Msh3	Msh3-003	599.20161	0.019884057	0.047313572	0.28855843	-3.465502639	2.015682929	6.985354508	-1.793065
Usp36	Usp36-002	882.16291	0.00142809	0.006024396	0.288572036	-3.46533924	2.337876942	8.101536706	-1.792997
Galnt4	Galnt4-001	4283	3.7605E-05	0.000392434	0.288616848	-3.4648012	10.58396825	36.6713459	-1.792773
Fyb	Fyb-006	431.53137	0.024472288	0.055541439	0.288666629	-3.464203687	1.636821955	5.670284651	-1.792524
Prmt6	Prmt6-001	408.7593	0.048793901	0.096233509	0.288669789	-3.464165761	1.135219302	3.932587837	-1.792508
Ncam1	Ncam1-006	1814.8137	0.018127951	0.043925173	0.288669968	-3.464163615	1.13563977	3.934041972	-1.792507
Gm38162	Gm38162-001	894.25578	0.005177435	0.016450041	0.288689861	-3.463924902	2.368155577	8.203113076	-1.792408
Cox7a2l	Cox7a2l-201	16959.062	9.92708E-06	0.000153708	0.288697506	-3.463833181	48.32044814	167.3739716	-1.792369
Sox7	Sox7-201	17757	1.28798E-05	0.000184189	0.288729449	-3.463449961	47.50334815	164.5254693	-1.79221
Zeb2	Zeb2-005	1049.729	0.031275606	0.067572112	0.288819054	-3.46237544	2.6754391	9.263374631	-1.791762
Chpt1	Chpt1-004	1627.3583	0.000220183	0.001433972	0.288853781	-3.461959181	4.104793042	14.21062596	-1.791589
Pmf1	Pmf1-006	974.53266	0.000219864	0.001432887	0.288950583	-3.460799389	2.697805689	9.336564281	-1.791105
Gm10224	Gm10224-001	191	0.017260114	0.042246759	0.289013225	-3.460049278	0.595360487	2.059976625	-1.790793
Pcmt1	Pcmt1-003	417.99936	0.020803167	0.048969654	0.289017812	-3.459994366	1.033077533	3.574442443	-1.79077
Gm16279	Gm16279-001	756.92492	0.001990304	0.007806491	0.289062901	-3.459454655	2.298037633	7.949956984	-1.790545
Rps13	Rps13-201	3284	1.01434E-05	0.00015586	0.289100772	-3.459001489	10.2190779	35.34780566	-1.790356
Vcpip1	Vcpip1-001	12886.945	1.30671E-07	1.09963E-05	0.289104704	-3.458954439	34.65588857	119.8731396	-1.790336
Tcf7l2	Tcf7l2-011	1637.9934	0.004652179	0.015114507	0.289142804	-3.458498655	3.590778166	12.41870146	-1.790146
Gm37531	Gm37531-001	1291	0.000846961	0.004002815	0.289157471	-3.458323236	3.737239929	12.92458368	-1.790073
Mrpl37	Mrpl37-003	462.21471	0.008723757	0.024740305	0.289192159	-3.457908414	1.012498216	3.5011261	-1.7899
Gm15801	Gm15801-001	295	0.006155036	0.018832409	0.289255066	-3.457156385	0.777497181	2.687929344	-1.789586
Gm37699	Gm37699-001	4762	0.001299994	0.005599196	0.28931624	-3.456425396	11.61348675	40.14115053	-1.789281
Arfgef1	Arfgef1-003	1315.379	0.001462369	0.006136557	0.289356259	-3.455947365	3.863125356	13.3507579	-1.789081
Srpk2	Srpk2-004	1054.2766	7.01615E-05	0.000618618	0.289378416	-3.455682754	2.307055016	7.972450231	-1.788971
Zeb2	Zeb2-014	789.62863	0.027177692	0.060392912	0.289459529	-3.454714392	2.574070195	8.892677349	-1.788566
Zeb2	Zeb2-010	167.31213	0.046278386	0.09226046	0.289489226	-3.454359989	0.564791356	1.950992662	-1.788418
Mrps17	Mrps17-001	1270.3649	0.00032221	0.001908303	0.289547383	-3.453666162	3.935013785	13.59022395	-1.788129
F11r	F11r-001	34141.048	1.03473E-08	2.66462E-06	0.289598895	-3.453051845	78.38057232	270.6521798	-1.787872
Ets1	Ets1-001	9136.1254	0.001882127	0.007459341	0.289614147	-3.452870004	30.78137335	106.2840807	-1.787796
Tmem173	Tmem173-201	4217	0.005673694	0.017664215	0.289634521	-3.452627114	14.5560241	50.25652348	-1.787695
Lypla1	Lypla1-001	3301.6037	0.000309315	0.001851458	0.289681644	-3.452065467	10.03442935	34.63950705	-1.78746
Tns3	Tns3-001	6791.4803	0.00260662	0.009629678	0.289684065	-3.452036619	22.14991605	76.4623213	-1.787448
'00062C07R	0062C07Rik-	1147	3.3587E-05	0.000362316	0.289711862	-3.451705407	3.333947093	11.50780321	-1.787309
Sco1	Sco1-004	666.43605	0.00277236	0.01009712	0.289714298	-3.451676381	1.419726471	4.900436328	-1.787297
P24-257C17	24-257C17.2-	271	0.006964594	0.020736968	0.289766112	-3.45105918	0.939698714	3.242955873	-1.787039
Crl1	Crl1-002	1616.89	0.000964956	0.00442473	0.28981297	-3.450501194	3.81175296	13.15245814	-1.786806
RP23-21D6.	P23-21D6.1-0	2855	0.000291681	0.00177035	0.289956096	-3.448797988	6.625321635	22.84939592	-1.786094
Zfp191	Zfp191-001	2773.6576	6.58059E-05	0.000589859	0.289992147	-3.448369236	8.027794331	27.68279901	-1.785914

Dnajc5	Dnajc5-001	6026.2144	2.90992E-05	0.000328601	0.28999398	-3.44834745	15.48680489	53.40388415	-1.785905
Cnot4	Cnot4-005	5319.1821	1.60192E-05	0.000214823	0.290004086	-3.448227274	16.17231569	55.76582007	-1.785855
Lepr	Lepr-004	653.76411	0.000645831	0.003255292	0.290040109	-3.447799009	1.808059862	6.233827	-1.785676
Ncoa6	Ncoa6-201	16806.217	7.84727E-08	8.35206E-06	0.290041143	-3.447786713	43.35046496	149.4631571	-1.785671
Mtmr3	Mtmr3-005	2852.5832	0.00129259	0.005574313	0.2900859	-3.447254761	7.272578953	25.07043242	-1.785448
Ppp1r2-ps1	pp1r2-ps1-20	128	0.032556809	0.069744227	0.290128851	-3.446744423	0.214781856	0.740298164	-1.785234
Pigv	Pigv-001	2615.6689	6.43404E-06	0.000115254	0.290157142	-3.446408366	6.481891376	22.33924467	-1.785094
Adam17	Adam17-004	2160.3786	7.07907E-06	0.000122357	0.290212838	-3.445746948	5.439576308	18.74340346	-1.784817
Rpl24	Rpl24-004	234.01044	0.040424116	0.082816483	0.290219205	-3.445671353	0.611513713	2.107075282	-1.784785
Slamf1	Slamf1-002	1273.3668	7.73255E-05	0.000663759	0.290432913	-3.443135932	3.464581313	11.92902441	-1.783723
Slc2a10	Slc2a10-001	2325.7383	4.07193E-06	8.53831E-05	0.290436884	-3.44308886	4.957050996	17.06756706	-1.783703
Srrm2	Srrm2-004	4885.5989	3.59187E-05	0.000379345	0.290464556	-3.442760847	12.27259023	42.25159315	-1.783566
Strada	Strada-003	1661.0995	0.001749683	0.007052682	0.290509315	-3.442230419	4.410358176	15.18146907	-1.783344
Cep83os	Cep83os-201	1156	0.001547057	0.00639936	0.290521332	-3.442088028	2.755816736	9.485763796	-1.783284
Eif2s3y	Eif2s3y-001	4031.0029	5.96153E-06	0.00010913	0.290538613	-3.4418833	11.04615804	38.01958689	-1.783198
Nbeal2	Nbeal2-005	539.48789	0.023877546	0.054498597	0.290548563	-3.441765433	1.143547901	3.935823637	-1.783149
Pitpna	Pitpna-001	12911.385	1.4941E-05	0.000204609	0.290555021	-3.441688931	34.87674138	120.0348948	-1.783117
Tbc1d20	Tbc1d20-002	4164.3423	0.002327613	0.008821593	0.290577159	-3.441426717	11.17025059	38.44159884	-1.783007
Dcaf8	Dcaf8-008	2196.7362	8.75307E-05	0.000723263	0.290645123	-3.440621984	5.949962816	20.47157287	-1.782669
030013C21R	0013C21Rik-	514	0.033067373	0.070612726	0.290678941	-3.440221697	1.197837728	4.120827342	-1.782502
Gm38042	Gm38042-001	2399	1.65035E-05	0.00021975	0.290680784	-3.440199881	5.5109921	18.95891437	-1.782492
Atxn1	Atxn1-203	9392.6443	0.000353041	0.002045617	0.290693002	-3.440055288	27.89420959	95.95762321	-1.782432
Glo1	Glo1-201	3366.0185	2.15107E-05	0.000266068	0.290695767	-3.440022566	8.627534993	29.67891506	-1.782418
Rnf14	Rnf14-202	1290.6067	0.012883823	0.033607839	0.290714655	-3.439799076	2.829610975	9.733293219	-1.782324
Mpdu1	Mpdu1-004	239.81898	0.040858742	0.083527032	0.290717287	-3.439767922	0.65118124	2.239912341	-1.782311
Trp53	Trp53-201	4081.6062	0.012960671	0.033765366	0.290726029	-3.439664495	12.88865754	44.33265773	-1.782268
Gm36955	Gm36955-001	2769	4.4575E-05	0.000444194	0.290765384	-3.439198935	6.037762511	20.7650664	-1.782073
Helb	Helb-001	1648.195	0.00148077	0.006195503	0.290780634	-3.439018564	5.039326226	17.33033644	-1.781997
Slain2	Slain2-003	2653.4057	0.00062811	0.003187551	0.290844525	-3.438263104	7.541036874	25.92806885	-1.78168
Ap4m1	Ap4m1-003	613.64992	0.000346109	0.00201363	0.290895384	-3.437661977	1.559710576	5.361757743	-1.781428
Atp11b	Atp11b-003	1402.094	0.029133768	0.06376791	0.290976673	-3.436701602	4.864896929	16.71919907	-1.781025
Dcun1d4	Dcun1d4-001	800.81157	0.032985699	0.070495313	0.290986973	-3.436579955	1.520434649	5.225095238	-1.780974
Hscb	Hscb-001	2033.3631	8.5332E-05	0.0007111	0.290996867	-3.436463116	5.777546684	19.85432608	-1.780924
Dmxl2	Dmxl2-005	1016.8111	0.00070866	0.003496872	0.291028087	-3.436094468	2.969005807	10.20178443	-1.78077
Dnajc1	Dnajc1-001	6415.5733	1.00624E-05	0.000155177	0.291056342	-3.4357609	15.29652976	52.55521886	-1.78063
Inpp4a	Inpp4a-001	5048.4401	1.49121E-06	4.4719E-05	0.291081572	-3.435463098	11.80260827	40.54742517	-1.780505
Pknox1	Pknox1-001	1311.7709	0.000745201	0.003628021	0.291132552	-3.434861522	3.838056823	13.1831937	-1.780252
430013B06R	0013B06Rik-	880	0.006055663	0.01859314	0.291133253	-3.434853247	2.518556445	8.650871783	-1.780248
Tctn2	Tctn2-004	432.95068	0.002717044	0.009948551	0.291222155	-3.43380468	0.778717016	2.673962136	-1.779808
Alms1	Alms1-001	4682.6444	4.21994E-06	8.75427E-05	0.291244501	-3.433541226	14.01656624	48.12645802	-1.779697
Letm1	Letm1-003	2112.0994	0.000432848	0.00239268	0.291254575	-3.43342246	4.673781587	16.04706668	-1.779647
Csnk1g1	Csnk1g1-004	528.17585	0.008227714	0.023607844	0.291274997	-3.433181737	1.164475568	3.997856251	-1.779546
Ttpal	Ttpal-001	3724.7526	0.045115596	0.090347258	0.291317925	-3.432675835	9.843616325	33.78994389	-1.779334
Arnt	Arnt-004	927.19832	0.027727235	0.061351367	0.291346705	-3.432336738	2.39477778	8.219683755	-1.779191
Zak	Zak-002	12702.44	7.84344E-08	8.35206E-06	0.291361044	-3.432167821	37.82483361	129.8211767	-1.77912
Lmbrd2	Lmbrd2-201	4677	1.09051E-05	0.000164538	0.291416621	-3.431513263	11.61038892	39.84120358	-1.778845
Tjp1	Tjp1-001	20142.471	0.000133761	0.000993774	0.291418265	-3.431493906	51.1390447	175.4833202	-1.778837
10039L15R	0039L15Rik-	472	0.003282564	0.011526331	0.291468453	-3.430903041	0.960591119	3.295694991	-1.778588
Fadd	Fadd-001	2861.7719	6.47885E-06	0.000115754	0.291483426	-3.430726793	7.047202939	24.17702794	-1.778514
Gm14698	Gm14698-001	2122.4667	0.000115754	0.000890713	0.291488884	-3.430662553	5.781939031	19.83588171	-1.778487
Ypel1	Ypel1-001	340.16935	0.010537792	0.028742485	0.2915395	-3.430066944	0.79450171	2.725194053	-1.778237
Irgq	Irgq-201	5848	3.96074E-06	8.36863E-05	0.291575633	-3.429641875	16.88040246	57.89373515	-1.778058
Psmd5	Psmd5-001	3358.8743	4.77551E-06	9.47836E-05	0.291610392	-3.429233073	9.139331716	31.34089858	-1.777886
Pmp22	Pmp22-001	9663.5585	2.61995E-06	6.46492E-05	0.291636137	-3.428930343	15.72520125	53.92061973	-1.777759
Sik2	Sik2-006	2751.4313	0.005587479	0.017457196	0.291639383	-3.428892179	6.272120012	21.50642325	-1.777743
Uba7	Uba7-005	526.09979	0.011700814	0.031215419	0.291714421	-3.428010165	1.443218364	4.947367223	-1.777371
Cblb	Cblb-201	3346	0.000132737	0.000987886	0.291776392	-3.427282076	9.577663958	32.82535601	-1.777065
Socs2	Socs2-002	672.36794	0.019169262	0.045947737	0.291793122	-3.427085579	1.921073194	6.583682238	-1.776982
Hdlbp	Hdlbp-011	519.18189	0.024861773	0.056216791	0.29188358	-3.426023486	1.131200417	3.875519197	-1.776535
Gng12	Gng12-001	10755.084	4.27555E-07	2.11533E-05	0.291905125	-3.425770611	30.40526256	104.1614549	-1.776429
Sin3a	Sin3a-003	4624.6047	0.000281501	0.001721372	0.291923501	-3.425554974	11.02274342	37.75901355	-1.776338
Gm23563	Gm23563-201	255	0.011775067	0.031357285	0.292025813	-3.424354818	0.631561224	2.162689719	-1.775832
Lcp1	Lcp1-011	2243.5722	5.63496E-06	0.000105463	0.292044636	-3.424134108	6.277331246	21.49442403	-1.775739
Sema4d	Sema4d-012	1228.0516	4.70083E-05	0.000460726	0.29211658	-3.423290797	3.156432512	10.80538637	-1.775384
Slc36a1os	Slc36a1os-001	326	0.003843019	0.013006266	0.29212945	-3.423139985	0.881878493	3.018793531	-1.77532
Rsu1	Rsu1-004	2644.2585	0.000372085	0.00212587	0.292167539	-3.422693718	6.217477821	21.28052228	-1.775132
Polr3f	Polr3f-001	3160.4354	2.05913E-05	0.00025805	0.292242687	-3.421813592	7.754458072	26.53431003	-1.774761
Nudcd2	Nudcd2-002	697.67471	0.001147158	0.005076905	0.292288537	-3.421276828	1.969931632	6.739681446	-1.774535
Nop16	Nop16-001	885.80293	0.002039737	0.007953209	0.2923667	-3.420362172	3.365037617	11.50964737	-1.774149
Ikbkb	Ikbkb-009	1146.6766	0.00031734	0.001886304	0.292368241	-3.420344142	3.003074775	10.27154922	-1.774141
Ppcs	Ppcs-002	199.23109	0.010633008	0.028933069	0.292389584	-3.420094471	0.625982714	2.14092002	-1.774036
30010A21R	0010A21Rik-	1592	0.000308063	0.001845577	0.292391837	-3.420068119	3.745033981	12.80827132	-1.774025
Lztfl1	Lztfl1-001	1093.7086	0.021769518	0.050757196	0.292404072	-3.419925017	2.967139765	10.14739551	-1.773965
Gcfc2	Gcfc2-004	399.7441	0.010080825	0.027770664	0.292440333	-3.419500963	0.820451074	2.805533239	-1.773786
Gm26930	Gm26930-001	259	0.039693265	0.081572922	0.292451108	-3.419374969	0.833038356	2.848470504	-1.773733
Atp11b	Atp11b-001	5619.3301	0.001718523	0.006942606	0.292497734	-3.418829909	13.11845176	44.84975523	-1.773503
Tpbg	Tpbg-002	522.16983	0.002413395	0.009069428	0.292499713	-3.418806775	1.422958421	4.864819892	-1.773493
Kdm2a	Kdm2a-006	320.26725	0.01222394	0.032257364	0.292550242	-3.418216283	0.796616898	2.720957922	-1.773244
Cflar	Cflar-012	1999.7649	2.78979E-05	0.000319352	0.292656401	-3.416976342	4.302468311	14.70143243	-1.77272
Parg	Parg-001	2965.2074	8.6219E-06	0.000140019	0.292659012	-3.416945857	7.47393914	25.53804538	-1.772707
Csrnp2	Csrnp2-201	1647	6.63128E-05	0.000593316	0.292686808	-3.416621356	4.943705002	16.89076809	-1.77257
Kif3a	Kif3a-001	1387.0098	0.000486149	0.002616784	0.292691847	-3.416562534	3.936064638	13.44781097	-1.772546
Rara	Rara-201	4947.8797	0.00240033	0.009032223	0.292714928	-3.416293135	17.29533437	59.08593208	-1.772432
Dhx33	Dhx33-001	2504.2712	0.000877608	0.004120103	0.292725766	-3.416166649	7.880057575	26.91958988	-1.772378
Ube2q2	Ube2q2-001	1877.7172	0.005470883	0.017193772	0.292728202	-3.416138227	4.503099615	15.38321073	-1.772366
Chchd4	Chchd4-201	805	0.005578309	0.01744004	0.292729973	-3.416117556	2.531448255	8.647724828	-1.772358
Tfam	Tfam-001	1923.4904	6.05084E-05	0.000553823	0.29273119	-3.416103356	5.545713129	18.94472923	-1.772352
10007H06R	0007H06Rik-	277.26693	0.034203961	0.072605248	0.292755508	-3.415819588	0.574467208	1.962276342	-1.772232
Gm14410	Gm14410-003	164.73052	0.02458236	0.055750619	0.292804549	-3.41524749	0.460796623	1.573734509	-1.77199
23-458A10	23-458A10.4-	293	0.008478649	0.024183355	0.292914435	-3.41396627	1.032155729	3.523744846	-1.771449
Amz2	Amz2-004	1412.3005	0.004023178	0.013481134	0.292939079	-3.413679064	3.433117787	11.71956231	-1.771327
Ppox	Ppox-002	1201.993	0.00158869	0.006536051	0.29300725	-3.412884831	2.689089546	9.177552922	-1.770992
Tmtc4	Tmtc4-001	955.58613	0.008595209	0.024440508	0.293033479	-3.412579351	2.688066247	9.17323937	-1.770863
30406C13R	0406C13Rik-	1911.0651	0.00434974	0.014342794	0.293046385	-3.412429064	4.474544068	15.26906423	-1.770799
Ctnnd1	Ctnnd1-002	144.50618	0.02944347	0.064317804	0.293065836	-3.412202574	0.329588034	1.124621137	-1.770703
Nrd1	Nrd1-011	830.40686	0.001291988	0.005573121	0.293148317	-3.411242504	2.224317009	7.587684724	-1.770297
Apip	Apip-001	867.61202	0.003984612	0.013381015	0.293166517	-3.411030733	2.643558477	9.01725921	-1.770208
Gm20938	Gm20938-001	177	0.035881876	0.075349739	0.293189519	-3.410763127	0.53748521	1.833234737	-1.770095
Pagr1a	Pagr1a-003	824.91695	0.009337069	0.02612012	0.293207455	-3.41055448	1.87215142	6.385074412	-1.770006
Nxpe4	Nxpe4-201	1791	0.001226637	0.005353932	0.293221088	-3.41039591	3.035441172	10.35205616	-1.769939
Hlcs	Hlcs-202	1948.5284	0.030940743	0.067015774	0.29332602	-3.409175904	4.402900827	15.01026341	-1.769423
Gm37780	Gm37780-001	1440	0.003603035	0.012378768	0.293353101	-3.408861189	3.995260267	13.6192876	-1.76929
Ash1l	Ash1l-005	970.22563	0.000103758	0.000819427	0.293451011	-3.407723821	2.61895539	8.92467667	-1.768808
10474O19R	0474O19Rik-	16845.502	2.81982E-08	4.38777E-06	0.293485766	-3.407320267	46.42172958	158.1737	-1.768638
H2-Ke6	H2-Ke6-007	591.35299	0.008101973	0.023344206	0.293488254	-3.407291385	1.678762412	5.720032703	-1.768625
Morf4l1	Morf4l1-004	3050.8833	0.000161727	0.001141491	0.293534291	-3.406757002	7.98007963	27.18619216	-1.768399
Med14	Med14-001	4392.503	0.005345187	0.016885507	0.293567369	-3.406373136	12.67456264	43.1742897	-1.768236

Nprl3	Nprl3-014	514.80315	0.009577488	0.026632213	0.293580504	-3.406220732	1.165659779	3.970494507	-1.768172
Gm8250	Gm8250-001	447	0.011153621	0.030011117	0.293618661	-3.405778085	1.306062028	4.448157432	-1.767984
Ercc5	Ercc5-007	337.49718	0.012740945	0.033308707	0.29364851	-3.405431893	0.884366878	3.011651173	-1.767838
Tcf7l2	Tcf7l2-013	452.92619	0.031651575	0.068193145	0.293711788	-3.40469821	0.985242334	3.354452813	-1.767527
10009A05R	0009A05Rik-	742.42144	0.000718732	0.003532883	0.293722522	-3.404573784	1.917463526	6.528146053	-1.767474
Mansc1	Mansc1-001	4222	0.000985926	0.004498795	0.293738188	-3.404392212	9.673788523	32.93337031	-1.767397
P23-300K5	23-300K5.1-(	2742	0.00023836	0.001519699	0.293750066	-3.40425456	8.501463329	28.9411453	-1.767339
Atp2c1	Atp2c1-008	2913.1492	1.95025E-05	0.000247621	0.293874837	-3.402809204	7.35620055	25.03174694	-1.766726
Rin2	Rin2-001	10470.97	0.001784397	0.007161347	0.293933605	-3.402128859	27.5149623	93.6094473	-1.766438
Rptor	Rptor-001	4003.9157	0.00564343	0.01759078	0.294008233	-3.401265296	8.100676183	27.55254877	-1.766072
Msto1	Msto1-005	1115.8008	0.001098783	0.004902726	0.294085468	-3.400372036	3.263773004	11.09804245	-1.765693
Hax1	Hax1-002	646.96729	0.04831275	0.095500385	0.294141759	-3.399721293	2.660518565	9.045021617	-1.765416
Cbx1	Cbx1-003	4957.8774	3.91003E-05	0.00040337	0.29418155	-3.39926144	13.72085603	46.64077683	-1.765221
Ypel5	Ypel5-201	6921	1.37663E-06	4.27276E-05	0.294189341	-3.399171422	17.99673435	61.1739851	-1.765183
Dnajc3	Dnajc3-201	28212	6.77033E-08	7.60275E-06	0.294246545	-3.398510598	73.11580537	248.4848394	-1.764903
32487G21R	2487G21Rik-	1143.6785	0.001562268	0.006453708	0.294277558	-3.398152435	2.635337811	8.955279599	-1.764751
Twf2	Twf2-003	170.10563	0.043984832	0.088595654	0.294279752	-3.398127095	0.4244607	1.442371404	-1.76474
P23-441I24	23-441I24.3-(	462	0.003532756	0.012189896	0.294307182	-3.397810385	1.281582786	4.354575301	-1.764605
Inpp5b	Inpp5b-001	3857.5228	0.000660011	0.003310686	0.294323906	-3.397617323	10.6535893	36.19681955	-1.764523
Arhgef1	Arhgef1-009	319	0.015229625	0.038311763	0.294414885	-3.396567403	0.686996028	2.333428316	-1.764077
Tmprss11b	mprss11b-00	883	0.002495285	0.009305697	0.294420358	-3.39650426	2.523803654	8.572109864	-1.764051
Ociad1	Ociad1-001	1783.224	0.000285936	0.00174196	0.294445272	-3.396216875	4.500853745	15.28587544	-1.763929
)10Wsu102	10Wsu102e-2	3437	0.000102192	0.000810397	0.294460064	-3.396046267	11.36151351	38.58422554	-1.763856
Gm26621	Gm26621-001	2478.1084	8.18559E-06	0.000135117	0.294492315	-3.395674351	6.590918747	22.38061374	-1.763698
Rogdi	Rogdi-001	631.43768	0.044582776	0.089536771	0.294495455	-3.395638144	1.788935353	6.074577121	-1.763683
Cirh1a	Cirh1a-201	2830	2.52425E-05	0.000297406	0.294510689	-3.395462495	8.429836839	28.62319483	-1.763608
Dab2	Dab2-013	365.97682	0.010072623	0.02775218	0.294512721	-3.395439075	1.115998383	3.789304518	-1.763598
10046J04R	0046J04Rik-(	288	0.004981084	0.015960531	0.294544108	-3.395077248	0.740514309	2.514103282	-1.763444
Gm37510	Gm37510-001	1421	2.7932E-05	0.000319423	0.294562438	-3.394865979	4.156487462	14.11071788	-1.763355
Sf3b1	Sf3b1-001	42080.642	1.18454E-07	1.05378E-05	0.294619286	-3.394210924	116.235092	394.5264189	-1.763076
Cmah	Cmah-202	4123.4147	0.002046645	0.007970902	0.294671729	-3.393606861	7.708399166	26.1592763	-1.762819
Cmah	Cmah-201	16521.622	2.20525E-06	5.82079E-05	0.294675917	-3.393558621	41.21499125	139.8654888	-1.762799
Pias1	Pias1-201	8950	5.2673E-06	0.000100729	0.294844559	-3.391617609	23.2397974	78.82050611	-1.761974
Spag9	Spag9-006	451.4847	0.036910427	0.077126601	0.294874162	-3.391277121	1.266379744	4.294644652	-1.761829
Sympk	Sympk-002	1772.784	5.80371E-06	0.000107215	0.294899523	-3.390985479	4.885393772	16.56629934	-1.761705
Dedd	Dedd-005	697.56216	0.001307997	0.005625177	0.294903507	-3.390939666	1.759585662	5.966648815	-1.761685
Mbtd1	Mbtd1-009	2846.4354	0.001036327	0.004680762	0.294903621	-3.390938362	10.40139665	35.27049493	-1.761685
Rnf32	Rnf32-011	595.36325	0.049181061	0.096801984	0.295001118	-3.389817658	1.431402096	4.852192099	-1.761208
Nek1	Nek1-001	1133.7436	0.011099677	0.02990316	0.295017813	-3.389625838	2.889331927	9.793754153	-1.761126
Tmem47	Tmem47-002	4140.8215	1.59535E-05	0.000214216	0.295049793	-3.389258438	11.67153526	39.55784936	-1.76097
Gm20678	Gm20678-001	2009	5.04751E-06	9.84098E-05	0.295050955	-3.389245084	5.671022994	19.2204868	-1.760964
Mrpl13	Mrpl13-002	2576.7078	6.96041E-06	0.000120936	0.295056745	-3.38917858	7.167176901	24.29084244	-1.760936
Tmem57	Tmem57-002	628.439	0.000343601	0.002002437	0.295084935	-3.38885481	1.285958696	4.357927312	-1.760798
Stx3	Stx3-203	846.19192	0.020195437	0.047851521	0.295164988	-3.387935702	2.323318801	7.871254712	-1.760406
Naa15	Naa15-201	8070.0246	8.90629E-06	0.00014301	0.29516708	-3.387911683	24.70418986	83.69561345	-1.760396
Tmem135	Tmem135-001	3173.7596	3.34686E-05	0.000361266	0.295179855	-3.387765067	7.548471897	25.5724494	-1.760334
Birc6	Birc6-016	1072.3163	0.001275165	0.00551478	0.295201068	-3.387521616	3.160484904	10.70621093	-1.76023
Raf1	Raf1-002	2868.9153	0.001624148	0.006646005	0.295234686	-3.387135889	7.319755683	24.79300717	-1.760066
32427E13R	2427E13Rik-	889.82052	9.75537E-05	0.000783586	0.295275118	-3.38667209	2.201110861	7.454440719	-1.759868
Xpnpep2	Xpnpep2-002	348.61887	0.00484581	0.015604457	0.295351423	-3.385797125	0.953969088	3.229945795	-1.759496
Arhgap29	Arhgap29-001	13259.409	0.000906874	0.004225911	0.295359666	-3.385702642	32.25398608	109.2024059	-1.759455
Timeless	Timeless-008	3258.203	0.006030585	0.018527766	0.295396719	-3.385277957	8.900259198	30.12985128	-1.759274
Gm10825	Gm10825-001	1141	0.01494106	0.037774513	0.295483853	-3.384279685	3.868958532	13.09363776	-1.758849
Atf6	Atf6-001	15837.397	5.94274E-06	0.000108927	0.295518033	-3.383888253	45.34840889	153.4539481	-1.758682
Mcts2	Mcts2-001	815	0.001384497	0.005878878	0.295530125	-3.38374979	2.081788566	7.044251624	-1.758623
10056K07R	0056K07Rik-	805.38548	0.002542822	0.009442875	0.295568295	-3.383312812	1.988137918	6.726492488	-1.758437
Ybx3	Ybx3-202	26366.011	2.02126E-05	0.000254469	0.295576405	-3.383219979	66.13543723	223.7507326	-1.758397
Gm14296	Gm14296-001	2068.849	0.001090232	0.004869644	0.295577224	-3.383210603	5.585505091	18.89694005	-1.758393
Zcchc4	Zcchc4-003	1006.2365	0.001542512	0.006386729	0.295592187	-3.383039347	2.736136744	9.256458264	-1.75832
Ywhag	Ywhag-002	743.81608	0.008028441	0.023192823	0.295653507	-3.382337689	2.492999697	8.432166834	-1.758021
Eif4g2	Eif4g2-001	58575.977	2.0169E-08	3.56435E-06	0.295700871	-3.38179592	154.6617623	523.0345166	-1.75779
Igf1	Igf1-001	674.64306	0.00849386	0.024214642	0.295714097	-3.381644668	3.034770821	10.26251657	-1.757725
Fgd4	Fgd4-005	365.00866	0.006897246	0.020574001	0.295758944	-3.381131905	1.147618445	3.880249339	-1.757506
Znrd1	Znrd1-002	1273.1455	0.008141326	0.023420159	0.295772351	-3.380978639	3.577602339	12.09579709	-1.757441
Rc3h2	Rc3h2-003	3041.3245	0.026229925	0.058660327	0.295781841	-3.380870162	6.588012801	22.27321591	-1.757395
Lig3	Lig3-009	2276.6743	0.006210221	0.018960605	0.295802402	-3.380635156	6.012893654	20.32739967	-1.757294
Cdk2ap2	Cdk2ap2-005	520.64196	0.022716852	0.052432926	0.295816585	-3.380473067	1.456229851	4.922745791	-1.757225
Ccng2	Ccng2-002	2041.7226	0.004592544	0.014970461	0.295845217	-3.380145911	6.093105028	20.59558404	-1.757086
Nox4	Nox4-004	2800.6385	8.34236E-06	0.000136907	0.29586905	-3.379873634	6.960225171	23.52468154	-1.756969
Trmt2a	Trmt2a-005	1333.4698	0.000810864	0.003870675	0.29588241	-3.379721021	4.236785537	14.31915314	-1.756904
D17H6S53E	17H6S53E-0(	2228.9315	9.15979E-06	0.000145714	0.295895768	-3.379568441	5.808647586	19.63072207	-1.756839
Exoc5	Exoc5-001	6102.7071	7.80197E-06	0.000130715	0.295914824	-3.379350814	16.69948008	56.43340159	-1.756746
B4galt3	B4galt3-005	1095.541	0.007414303	0.021806774	0.29594403	-3.379017314	3.028244975	10.2324922	-1.756604
Nup88	Nup88-005	424.5302	0.003262295	0.011469106	0.296022517	-3.378121405	1.11048912	3.751367068	-1.756221
Sacs	Sacs-003	647.82578	0.024832558	0.056170933	0.29612107	-3.376997121	1.492990962	5.041826181	-1.755741
Cfap45	Cfap45-008	719	0.000938869	0.004338993	0.296228349	-3.375774135	1.641444121	5.541144607	-1.755218
Pafah1b1	Pafah1b1-005	11479.823	9.00691E-08	8.98218E-06	0.296304858	-3.374902485	28.51676581	96.24128794	-1.754846
Met	Met-001	4001.2469	0.000410875	0.00229455	0.296318339	-3.374748939	11.51784157	38.86982362	-1.75478
Lrch3	Lrch3-008	1790.453	0.003729639	0.012719319	0.296322783	-3.37469833	4.162599897	14.04751892	-1.754759
Thrap3	Thrap3-002	1391.0121	0.003091697	0.010997052	0.296397856	-3.373843566	4.10076498	13.83533954	-1.754393
Enpp4	Enpp4-002	10664.772	0.000695806	0.003449318	0.296419551	-3.373596637	22.51568076	75.95882489	-1.754287
Gm28826	Gm28826-001	377	0.002459977	0.0092061	0.296427063	-3.373511141	1.043944256	3.521757578	-1.754251
Smc6	Smc6-201	18025	4.259E-07	2.11533E-05	0.296445686	-3.373299211	48.67293666	164.1883788	-1.75416
00002H07R	0002H07Rik-	617.90938	0.002699716	0.009895684	0.296479897	-3.372909965	1.562787678	5.271142131	-1.753994
Dus1l	Dus1l-007	283.43939	0.03853681	0.079748572	0.296537573	-3.372253938	0.889470294	2.999519702	-1.753713
Fubp1	Fubp1-001	3409.8839	0.000584079	0.003013211	0.296550996	-3.372101298	10.40489929	35.08637441	-1.753648
Ap1ar	Ap1ar-001	4217.1885	1.16925E-05	0.000172001	0.296564274	-3.37195033	14.28392982	48.16470186	-1.753583
Psmb7	Psmb7-001	5859.7675	0.000332686	0.00195563	0.296709338	-3.370301746	16.90669895	56.980677	-1.752878
Luc7l3	Luc7l3-004	5459.8927	7.68908E-05	0.000661246	0.296745259	-3.369893766	16.37049294	55.16682212	-1.752703
Zfp937	Zfp937-001	1439	0.0002113	0.0013888	0.296770234	-3.36961017	3.921872151	13.21518029	-1.752582
Lage3	Lage3-001	738.81442	0.001690926	0.006852132	0.296772286	-3.369586875	1.980144182	6.672267845	-1.752572
32415L05R	2415L05Rik-	4364.0981	4.62025E-05	0.000454881	0.296911283	-3.368009422	10.63296238	35.81191746	-1.751896
00120C14R	0120C14Rik-	626.46278	0.002669045	0.009807403	0.296920966	-3.367899592	1.598824155	5.384679219	-1.751849
Ubxn7	Ubxn7-201	17272	6.54372E-08	7.52089E-06	0.296936231	-3.367726458	48.51581747	163.3880021	-1.751775
00097O09R	0097O09Rik-	1382.8403	0.002824083	0.010251706	0.296960973	-3.367445868	3.63377531	12.23654165	-1.751655
Apold1	Apold1-201	271	0.025106993	0.05668312	0.296961401	-3.367441009	0.875203111	2.947194849	-1.751653
Supt7l	Supt7l-003	1481.1136	2.60191E-05	0.000304045	0.296966786	-3.367379943	3.909472152	13.16467811	-1.751627
Akr1b3	Akr1b3-007	490.36706	0.003822918	0.012958553	0.296999673	-3.367007074	1.222618643	4.116565621	-1.751467
Cox5a	Cox5a-201	2657.5713	0.003836335	0.012988778	0.297005525	-3.366940737	7.414639641	24.96465226	-1.751438
P24-231P10	24-231P10.4-(	863	0.006406792	0.019403362	0.297008187	-3.366910555	2.117286504	7.12871428	-1.751425
Wdr55	Wdr55-201	1636	0.001062114	0.004770181	0.297025681	-3.36671225	5.223626963	17.58644888	-1.75134
Gramd1a	Gramd1a-004	512.32878	0.033422408	0.071246498	0.297039636	-3.366554081	1.600189468	5.387124384	-1.751273
Gm37589	Gm37589-001	6242	5.32028E-05	0.000503926	0.29706666	-3.366247828	14.0223321	47.20264496	-1.751141
Cep89	Cep89-008	1270.3826	0.000261036	0.001625246	0.297074103	-3.366163495	3.380377637	11.3789038	-1.751105
Ip6k2	Ip6k2-005	1636.5053	0.001209876	0.005289541	0.29710657	-3.365795652	4.098654184	13.79523243	-1.750948
Rad54l	Rad54l-004	796.60735	0.005471867	0.017195289	0.297109364	-3.365763994	2.115481251	7.120210624	-1.750934
Emp1	Emp1-001	18057.602	0.01033451	0.028323978	0.297120835	-3.365634048	46.09842238	155.1504199	-1.750878

Gm4419	Gm4419-201	1870	0.000107052	0.000837683	0.297171874	-3.365056004	4.799767987	16.15148809	-1.750631
Sbno1	Sbno1-201	9082.7355	2.73946E-05	0.000315193	0.297174849	-3.365022316	26.50742106	89.19806341	-1.750616
Emb	Emb-001	5091.7838	0.000451895	0.002473612	0.297185668	-3.364899823	19.99986145	67.29753024	-1.750564
Cdk2	Cdk2-201	1111.1722	0.016970673	0.041723174	0.297223674	-3.36446955	3.290982989	11.07241206	-1.750379
D1Ertd622e	1Ertd622e-00	5055.2779	0.002754247	0.010050097	0.297264738	-3.364004785	15.61532411	52.53002503	-1.75018
Yaf2	Yaf2-002	1863.5028	0.000704515	0.003482199	0.29728705	-3.363752312	5.469066813	18.39658614	-1.750071
Smarcad1	Smarcad1-201	5244	4.45179E-06	9.03816E-05	0.29735055	-3.363033968	15.26013475	51.32035152	-1.749763
Spg20	Spg20-008	884.92395	0.01200113	0.031819066	0.297381474	-3.362684257	2.228204924	7.492749621	-1.749613
Rpa1	Rpa1-001	721.27251	0.033017828	0.070542113	0.297444885	-3.361967381	1.721519042	5.787690863	-1.749306
AI837181	AI837181-002	1350.589	0.000443346	0.002436161	0.297572959	-3.360520398	3.3414703	11.2290791	-1.748685
Gm27219	Gm27219-001	148	0.034479778	0.073068457	0.297672868	-3.359392495	0.533979087	1.793845338	-1.7482
P24-411A22	24-411A22.2-	836	0.000436749	0.002409191	0.297744003	-3.358589894	2.30379217	7.737493102	-1.747856
Pex26	Pex26-005	445.22735	0.023745529	0.054251509	0.297744478	-3.358584541	0.975834018	3.277421047	-1.747853
Atic	Atic-006	168.75267	0.026625066	0.059361314	0.297776179	-3.358226989	0.569553812	1.912690983	-1.7477
Ctnnb1	Ctnnb1-201	47882.135	4.05813E-07	2.05608E-05	0.297895255	-3.356884625	119.5434539	401.2935824	-1.747123
Gm3200	Gm3200-001	480	0.010765118	0.029183574	0.297931106	-3.35648068	1.446023696	4.853550599	-1.746949
Dnajb1	Dnajb1-201	7469	6.47892E-07	2.68973E-05	0.297945031	-3.356323801	18.80272222	63.10802411	-1.746882
Psmc3	Psmc3-008	419.36401	0.016404879	0.040594402	0.297965828	-3.356089548	1.017338454	3.414278951	-1.746781
Gm13835	Gm13835-001	2349	0.000141185	0.001035871	0.298031456	-3.355350516	6.931479311	23.25754268	-1.746463
Api5	Api5-001	11787.997	4.09635E-07	2.06628E-05	0.298079027	-3.354815033	31.21446442	104.7187545	-1.746233
Mmgt1	Mmgt1-001	2809	0.001062968	0.004772381	0.298096159	-3.354622219	8.07763067	27.09739932	-1.74615
Mrpl16	Mrpl16-201	1637	0.000291127	0.001768236	0.298228921	-3.353128856	4.643863927	15.57147414	-1.745508
Mtfmt	Mtfmt-001	1307.6446	0.000373466	0.002132581	0.298238318	-3.353023202	3.671441862	12.31042975	-1.745462
Rufy2	Rufy2-007	779.98187	0.012164439	0.032145053	0.298244654	-3.352951967	1.849325693	6.200700221	-1.745432
Plekha1	Plekha1-002	7523.4453	4.35847E-06	8.90871E-05	0.298302435	-3.352302503	19.99731916	67.03706307	-1.745152
Dnpep	Dnpep-003	658.10151	0.014209931	0.036328292	0.298499267	-3.350091975	1.493779649	5.004299214	-1.744201
Pkn2	Pkn2-012	279.03502	0.035249221	0.074317379	0.298505486	-3.350022183	0.828666425	2.776050905	-1.744171
Rpl10a	Rpl10a-007	798.18599	0.040195903	0.082457456	0.298544622	-3.349583037	2.602288086	8.716580031	-1.743982
Stk19	Stk19-002	1253.6909	0.000631615	0.003201067	0.298559394	-3.349417299	3.591572283	12.02967433	-1.74391
Cstf3	Cstf3-002	404.00601	0.041187363	0.084083399	0.298565152	-3.349352703	1.064132767	3.56415596	-1.743882
Rftn2	Rftn2-001	1821.9677	0.006433347	0.019470013	0.298593501	-3.349034714	4.831896053	16.18218762	-1.743745
Acmsd	Acmsd-002	442.23134	0.000755373	0.003665042	0.298600695	-3.348954031	0.980256318	3.282833346	-1.743711
P23-204O17	23-204O17.4-	416	0.003061895	0.010921674	0.29861641	-3.348777781	1.219809465	4.084870834	-1.743635
Sfswap	Sfswap-012	459.95926	0.031191998	0.067423536	0.298650843	-3.348391688	1.415952515	4.741163633	-1.743468
Gm6169	Gm6169-201	2352	5.73137E-06	0.000106453	0.298658865	-3.348301749	6.166676744	20.64789453	-1.74343
Ptbp2	Ptbp2-010	1553.4723	2.70783E-05	0.000312583	0.298680083	-3.348063891	4.650294706	15.56948379	-1.743327
Gm37962	Gm37962-001	758	0.000914411	0.004254663	0.298705707	-3.347776678	1.937186387	6.485267407	-1.743203
Hmbox1	Hmbox1-003	733.36926	0.000441591	0.002430026	0.298758621	-3.347183741	2.124601141	7.111430393	-1.742948
Utp14b	Utp14b-001	3645.5127	2.17355E-05	0.000267688	0.298807493	-3.346636294	9.018779989	30.18257644	-1.742712
Ndrg3	Ndrg3-004	3171.9304	2.31405E-06	5.97571E-05	0.298808702	-3.346622749	7.9022158	26.44573516	-1.742706
Copz2	Copz2-005	2007.9792	0.023827741	0.054415297	0.298822734	-3.346465557	4.135359544	13.83883828	-1.742638
Gm4076	Gm4076-201	55016	2.60259E-06	6.436E-05	0.298829213	-3.346393043	151.9048437	508.3333121	-1.742607
Ppp1cc	Ppp1cc-001	7455.6517	3.42123E-05	0.000366179	0.298840723	-3.346264161	21.65821659	72.47411395	-1.742551
Aldh5a1	Aldh5a1-001	1247.7554	0.025207982	0.056864102	0.298873147	-3.345901126	3.480042392	11.64387776	-1.742395
Ppapdc2	Ppapdc2-001	2000.6898	8.26501E-05	0.00069552	0.298905907	-3.345534422	5.480312432	18.33457388	-1.742237
Tstd2	Tstd2-002	3848.5863	5.57799E-05	0.000521699	0.298934805	-3.345211004	10.73516754	35.91140059	-1.742097
Nsun2	Nsun2-002	692.46773	0.019348016	0.046291971	0.298942642	-3.345123309	2.558846952	8.559658583	-1.742059
Prkd3	Prkd3-006	1004.9634	0.000574225	0.002976721	0.298966799	-3.344853017	2.400832362	8.030431369	-1.741943
Hiat1	Hiat1-001	6488.938	3.38041E-06	7.59705E-05	0.299029693	-3.34414951	17.30976723	57.88644961	-1.741639
Impdh1	Impdh1-010	412.62422	0.013636224	0.035155489	0.299093846	-3.343432211	1.248926012	4.175699457	-1.74133
Luc7l2	Luc7l2-008	10175.576	7.06123E-06	0.000122207	0.299148599	-3.342820269	25.17118719	84.14275472	-1.741066
Vkorc1l1	Vkorc1l1-001	6427.9548	9.83967E-06	0.000152938	0.29916959	-3.342585723	17.42686895	58.25080334	-1.740965
Dennd4b	Dennd4b-001	1411.5413	0.005686594	0.017691512	0.299177707	-3.34249503	4.056313643	13.55820819	-1.740925
Rab11a	Rab11a-007	791.34562	0.047309927	0.093934202	0.29919914	-3.342255594	1.638315231	5.475668245	-1.740822
Ube2k	Ube2k-004	4170.8832	1.38449E-05	0.00019426	0.299240835	-3.341789903	12.98219139	43.38375609	-1.740621
Gm37519	Gm37519-001	530	0.012135636	0.032081317	0.299252209	-3.341662882	1.105609171	3.694573129	-1.740566
Rpl10-ps1	Rpl10-ps1-001	7049	1.71483E-06	4.82711E-05	0.299334435	-3.340744946	21.15608079	70.67706996	-1.74017
Ncf2	Ncf2-002	1740.3511	0.000857691	0.004045156	0.299398958	-3.34002499	5.400429429	18.03756925	-1.739859
Clasp1	Clasp1-203	4218.3256	0.003432346	0.011918261	0.2994856	-3.339058704	9.923694328	33.13579792	-1.739441
Cd24a	Cd24a-201	152046	2.41979E-05	0.000287844	0.299491243	-3.338995792	412.4332884	1377.113014	-1.739414
Cep170	Cep170-010	2238.4601	0.000717415	0.003528356	0.299495491	-3.33894843	6.865767236	22.92444273	-1.739394
Limch1	Limch1-001	14154.974	0.007118179	0.021108557	0.299511727	-3.338767435	34.35048969	114.6882873	-1.739316
Tgfbrap1	Tgfbrap1-006	589.48715	0.024489807	0.055573835	0.299513761	-3.338744757	1.095921369	3.659001726	-1.739306
Psmb2	Psmb2-001	3735.1034	0.003270423	0.011491944	0.299603966	-3.33773953	11.37309132	37.96041649	-1.738871
Slc22a15	Slc22a15-005	1107.5913	0.000705929	0.003486287	0.299623225	-3.337524986	2.714679999	9.060312325	-1.738779
Haus2	Haus2-001	2245.369	0.001540735	0.006383232	0.299643714	-3.337296774	6.562164207	21.89988944	-1.73868
Hyi	Hyi-004	614.10668	0.004732814	0.015311126	0.299707658	-3.336584741	1.650108093	5.505725483	-1.738372
Pias4	Pias4-004	1011.0879	0.00253494	0.009417692	0.299708084	-3.336580004	2.366537556	7.89614189	-1.73837
Arhgef15	Arhgef15-201	23248.269	0.000586174	0.00302038	0.299712354	-3.336532465	49.85632751	166.3472553	-1.73835
Fxyd5	Fxyd5-003	5447.9207	0.000199726	0.001331117	0.299719647	-3.336451284	12.26085682	40.90775147	-1.738314
Ak9	Ak9-001	280	0.004648509	0.015107357	0.299720513	-3.336441639	0.785117831	2.619499823	-1.73831
Gm10557	Gm10557-201	2702	1.13868E-05	0.00016927	0.299764672	-3.335950147	7.155330372	23.8698254	-1.738098
Slc12a9	Slc12a9-001	845.31903	0.006400296	0.019387118	0.299820611	-3.335327734	3.071142097	10.24326541	-1.737829
Ccnt2	Ccnt2-004	2079.8185	1.70173E-05	0.000224761	0.299844352	-3.335063649	5.429304741	18.10707688	-1.737714
Rnpc3	Rnpc3-004	362.43605	0.008359058	0.023907953	0.299858451	-3.334906844	0.714102932	2.381466755	-1.737646
Mpp5	Mpp5-201	10317	3.39797E-06	7.62151E-05	0.299882154	-3.334643246	29.50900577	98.4020068	-1.737532
530017D24R	0017D24Rik-	762.28438	0.002158989	0.008310337	0.299883764	-3.33462534	1.973268778	6.580112068	-1.737525
Erlin1	Erlin1-201	1086.5397	0.048894023	0.096375477	0.299936722	-3.334036568	3.579374921	11.93376688	-1.73727
Dnajc1	Dnajc1-004	4230.949	5.61813E-05	0.000524308	0.299963054	-3.333743896	11.61385481	38.7176176	-1.737143
Tmx4	Tmx4-002	403.17783	0.020798295	0.04896155	0.300134152	-3.331843425	1.321731124	4.403801156	-1.736321
Ddx17	Ddx17-201	61439	6.8055E-07	2.77156E-05	0.300135319	-3.331830467	162.0332573	539.8673435	-1.736315
Lrrc40	Lrrc40-003	616.00576	0.026163338	0.058556774	0.300146667	-3.331704498	1.707135171	5.687669927	-1.73626
Evi5	Evi5-001	8701.8753	4.32243E-06	8.87018E-05	0.30015552	-3.331606233	21.6782397	72.2233585	-1.736218
Eif2ak2	Eif2ak2-001	11042.77	3.15531E-07	1.78627E-05	0.300201144	-3.331099895	27.98427227	93.21840642	-1.735999
Synj1	Synj1-201	8687.1738	2.19993E-05	0.000270113	0.300207299	-3.331031599	21.4892782	71.58146474	-1.735969
Oxr1	Oxr1-207	587.0689	0.039402614	0.08110701	0.300253162	-3.330522794	1.32057792	4.398214865	-1.735749
32404H12R	2404H12Rik-	2058.7142	0.000317603	0.001887216	0.300258859	-3.330459597	5.937955425	19.77612063	-1.735721
Klhl6	Klhl6-003	766.01096	0.006240169	0.019031681	0.300372799	-3.32919626	2.757394578	9.179907716	-1.735174
Myzap	Myzap-003	9185.0696	0.000227885	0.001470713	0.300393827	-3.328963219	24.12467601	80.3101591	-1.735073
Churc1	Churc1-001	1764.6607	0.001801668	0.007211443	0.300473293	-3.328082809	3.893240389	12.95702641	-1.734691
Agl	Agl-011	875.57669	0.002326311	0.008819793	0.30048392	-3.327965105	2.32061083	7.722911863	-1.73464
Birc3	Birc3-002	2410.6892	4.07692E-06	8.53944E-05	0.300490333	-3.327894078	6.762524942	22.50496671	-1.73461
Ppp2r3a	Ppp2r3a-007	252.7196	0.008433093	0.024075472	0.300528445	-3.327472047	0.590441344	1.964677069	-1.734427
Bpifc	Bpifc-001	245	0.005405596	0.017043211	0.300552554	-3.32720513	0.581482034	1.934710008	-1.734311
Ermard	Ermard-202	1098.9476	0.002664663	0.009796554	0.300578928	-3.326913193	3.14295136	10.45632634	-1.734184
Fam132b	Fam132b-001	242.85353	0.035470187	0.074628696	0.300603233	-3.3266442	0.600126081	1.996405947	-1.734068
Zfp51	Zfp51-201	2176	2.51832E-05	0.000297083	0.300662508	-3.325988349	5.763127757	19.16809578	-1.733783
Tbc1d25	Tbc1d25-002	1301.6467	0.000662097	0.003318236	0.300669659	-3.325909249	3.711257134	12.34330443	-1.733749
Gm19331	Gm19331-001	413	0.009493911	0.026468576	0.30067345	-3.325867315	1.127419481	3.749647603	-1.733731
Phkb	Phkb-001	4028.8829	0.001177297	0.005180168	0.300725312	-3.325293753	9.115559433	30.31191284	-1.733482
Thrap3	Thrap3-004	3354.2884	0.002513057	0.009351667	0.300808202	-3.324377441	8.124941262	27.01037144	-1.733084
330069E16R	0069E16Rik-	289	0.007455462	0.021901499	0.300810968	-3.324346874	0.764024331	2.539881895	-1.733071
Bgn	Bgn-002	988.27184	0.003097222	0.011012131	0.300815272	-3.324299303	2.391046075	7.948552802	-1.73305
Atp5c1	Atp5c1-001	6313.96	0.00358234	0.012325992	0.300816174	-3.324289334	18.31985589	60.90050153	-1.733046
Sf1	Sf1-005	971.45912	0.006099706	0.018706565	0.300819571	-3.324251799	3.027822154	10.06524324	-1.73303
Dnaja3	Dnaja3-001	449.55548	0.03525702	0.074329247	0.300933188	-3.322996728	0.944462982	3.1384474	-1.732485
Arrdc3	Arrdc3-004	937.16589	0.008011692	0.023154203	0.300935467	-3.322971565	2.905094418	9.653546146	-1.732474

Etl4	Etl4-005	1086.3363	0.001322741	0.00566897	0.300981755	-3.322460522	3.07002245	10.20002839	-1.732252
Phf3	Phf3-201	9072.8083	9.29562E-07	3.35538E-05	0.301050354	-3.321703451	25.63439532	85.1498594	-1.731923
Parl	Parl-001	1465.3403	0.010890524	0.029465237	0.301138231	-3.32073412	4.129669189	13.71353338	-1.731502
Atp5l	Atp5l-201	3010	0.000173178	0.001197527	0.301153417	-3.320566671	8.552929646	28.40057312	-1.731429
Gm37411	Gm37411-001	865	0.002800006	0.010179409	0.301199537	-3.320058228	2.215679846	7.356186102	-1.731209
Tpm3	Tpm3-014	1482.5728	0.002513487	0.009352191	0.301215395	-3.319883434	3.88269779	12.89010407	-1.731133
Harbi1	Harbi1-003	1218.4242	2.40501E-05	0.00028671	0.301284927	-3.319117255	3.325087999	11.03635695	-1.7308
Zfp516	Zfp516-202	2242.6724	0.005931598	0.018277753	0.301446807	-3.317334853	7.311582069	24.25496603	-1.730025
Rraga	Rraga-001	4807	2.16297E-05	0.000266961	0.301490775	-3.316851072	11.99481225	39.78500587	-1.729814
Wnk1	Wnk1-004	7438.7951	1.21552E-05	0.000177155	0.301539279	-3.316317541	17.46974283	57.93521457	-1.729582
Npc1	Npc1-001	6621.0722	3.25299E-06	7.42271E-05	0.301540546	-3.316303608	18.1186482	60.0869384	-1.729576
Cep128	Cep128-004	554.2597	0.000572507	0.002970761	0.301547647	-3.316225512	1.309979001	4.344185783	-1.729542
Tmem71	Tmem71-201	718	0.00146418	0.006142105	0.301597422	-3.315678205	2.005648771	6.650085916	-1.729304
Eif4g2	Eif4g2-002	49646.449	8.38982E-07	3.16466E-05	0.301610093	-3.315538915	137.0168343	454.2846463	-1.729243
Zbtb2	Zbtb2-202	1879.8017	0.002614239	0.009652618	0.301610582	-3.31553354	5.089997013	16.87605582	-1.729241
730013B05R	0013B05Rik-	351.67556	0.015657198	0.039159587	0.301679045	-3.314781114	0.901085709	2.986901891	-1.728914
Tmx1	Tmx1-001	21201.178	0.000203018	0.001348337	0.301717603	-3.3143575	57.35269681	190.0873408	-1.728729
Malt1	Malt1-001	6599	3.79558E-06	8.16753E-05	0.301733855	-3.314178982	20.11699743	66.67133004	-1.728652
Macf1	Macf1-013	44553.297	9.77441E-07	3.44358E-05	0.301806115	-3.313385483	105.4914223	349.5337472	-1.728306
Gm2606	Gm2606-001	1573	0.000371234	0.002121956	0.301814535	-3.313293044	4.916472374	16.28971372	-1.728266
P23-347G20	23-347G20.5-	374	0.011666268	0.031131258	0.301815143	-3.313286377	1.080873778	3.581244364	-1.728263
Mtmr4	Mtmr4-001	3075.7543	0.000316332	0.001880639	0.301820126	-3.313231674	8.908568257	29.51615052	-1.728239
Pgs1	Pgs1-002	1130.4368	0.047794453	0.094968601	0.301836959	-3.313046892	4.507434661	14.9333424	-1.728159
Atp6v1b2	Atp6v1b2-003	524.18988	0.003845041	0.013009465	0.30188316	-3.312539859	1.429147005	4.734106418	-1.727938
30406C13R	0406C13Rik-	498.93686	0.034610503	0.073287719	0.301937978	-3.311938457	1.205613913	3.992919083	-1.727676
Zbtb46	Zbtb46-001	6330.9415	0.000705686	0.003486287	0.301957546	-3.311723825	11.23446594	37.2054485	-1.727582
Ccdc43	Ccdc43-201	321.52348	0.025071517	0.056608839	0.301969332	-3.311594571	0.844213302	2.795692187	-1.727526
P23-381C14	23-381C14.5-	486	0.000835062	0.003958596	0.302003096	-3.311224329	1.275122182	4.222215591	-1.727365
P23-442L7.	23-442L7.3-0	2176	0.000186456	0.001266333	0.30205286	-3.310678797	5.775081122	19.11943862	-1.727127
Gla	Gla-001	1127.6116	0.000984507	0.004494717	0.302067634	-3.310516875	3.062067101	10.13702481	-1.727056
Srsf10	Srsf10-001	3501.0116	0.002573502	0.009527882	0.30208884	-3.310284488	11.68077009	38.66667202	-1.726955
Tmem140	Tmem140-001	4505.8125	0.003140478	0.011134718	0.302114744	-3.310000655	8.979203525	29.72116955	-1.726832
Ankrd44	Ankrd44-005	455.99429	0.006421992	0.019440802	0.302192944	-3.309144107	1.217485855	4.028836142	-1.726458
Plag1	Plag1-002	576.7512	0.017288637	0.042304481	0.302221524	-3.308831176	1.133295765	3.749884358	-1.726322
Cyb5d2	Cyb5d2-004	564.87886	0.002643279	0.009734937	0.302223463	-3.308809946	1.444474156	4.779490455	-1.726312
Timmdc1	Timmdc1-001	2062.0854	2.19561E-06	5.81141E-05	0.302235784	-3.308675056	5.375873744	17.78701936	-1.726254
C1qtnf6	C1qtnf6-001	454.71151	0.047054229	0.093476905	0.302246067	-3.308562491	1.236324945	4.09045834	-1.726205
Rbm22	Rbm22-002	946.75229	0.002031005	0.007928189	0.302298807	-3.307985264	2.191647324	7.249937051	-1.725953
Rb1	Rb1-006	479.21803	0.022292341	0.051693478	0.302335458	-3.307584249	1.349563028	4.463793415	-1.725778
Fblim1	Fblim1-201	13510.742	4.61195E-05	0.00045469	0.302362845	-3.307284658	46.26697505	153.0180568	-1.725647
30428H23R	0428H23Rik-	894.90224	0.001265833	0.005479394	0.302389237	-3.306996004	2.568666536	8.49456997	-1.725521
Traf6	Traf6-002	316.76606	0.0221842	0.051491518	0.302389313	-3.306995175	0.914961844	3.025774403	-1.725521
Naa35	Naa35-001	2863.282	0.000672088	0.00335554	0.30244271	-3.306411324	8.591928798	28.40845068	-1.725266
Smc3	Smc3-001	27096.445	1.6332E-08	3.19391E-06	0.302455267	-3.306274051	76.58415061	253.2081899	-1.725206
Tia1	Tia1-006	1735.4533	0.000130997	0.000978123	0.3025397	-3.305351328	5.041341506	16.66340484	-1.724804
Rrm1	Rrm1-201	6018	0.007936304	0.022986702	0.302539976	-3.305348313	19.17559916	63.38203433	-1.724802
Arfgef1	Arfgef1-002	14810.289	1.4264E-07	1.16597E-05	0.302565648	-3.305067867	41.21757772	136.2268917	-1.72468
Ppp1r12b	Ppp1r12b-004	1082.6067	0.006414288	0.019424346	0.302570153	-3.305018655	1.960713351	6.480194204	-1.724658
Phf10	Phf10-002	2210.5632	0.000808096	0.003861231	0.302571909	-3.304999474	6.420978449	21.2213304	-1.72465
Nupl1	Nupl1-201	4614.5338	0.0002825	0.001726868	0.302581591	-3.304893722	13.52596044	44.70186176	-1.724604
430010I23R	0010I23Rik-	1201.9764	0.002331347	0.008828907	0.302616306	-3.304514597	3.338965415	11.03365995	-1.724438
Rora	Rora-001	13589.436	0.000828122	0.003934414	0.302697452	-3.303628731	29.38418786	97.07444725	-1.724052
Arap1	Arap1-004	1897.5937	0.039183061	0.080752142	0.302698843	-3.303613555	4.94312746	16.33018288	-1.724045
Tshb	Tshb-004	1499.8091	0.000118329	0.000906504	0.302787625	-3.302644877	3.500910433	11.56226391	-1.723622
Chrna1	Chrna1-001	582	0.001312341	0.005637488	0.30279624	-3.302550912	1.794246285	5.925589706	-1.723581
Npepl1	Npepl1-002	1970.0961	0.000764427	0.00369849	0.302797049	-3.302542098	4.463238367	14.7400326	-1.723577
B3galt2	B3galt2-001	2486.1077	2.14962E-05	0.000265984	0.302832283	-3.302157844	6.66897649	22.02201303	-1.723409
Rplp2	Rplp2-006	409.00217	0.009703354	0.026910112	0.302860076	-3.301854809	1.242475332	4.102473151	-1.723277
Ptprg	Ptprg-003	470.63961	0.02414255	0.054956955	0.302865035	-3.301800753	0.927150752	3.061267051	-1.723253
Cltc	Cltc-006	23236.498	5.22556E-05	0.000496878	0.3028886	-3.30154387	64.49600799	212.9363998	-1.723141
Rpl23a	Rpl23a-002	9281.4567	8.79917E-05	0.000725711	0.302903515	-3.3013813	29.08038152	96.00542776	-1.72307
Mir99ahg	Mir99ahg-007	1243.6713	0.001119164	0.004977254	0.302923432	-3.301164237	2.766779214	9.133592593	-1.722975
Appbp2	Appbp2-001	13861	3.81362E-07	2.00025E-05	0.302952853	-3.300843647	39.49869665	130.3790219	-1.722835
42628	Sep15-006	352.25079	0.012201627	0.032220951	0.303045596	-3.299833468	1.141427268	3.7665199	-1.722393
Hspa8	Hspa8-003	32429.71	0.000423816	0.002353387	0.303063036	-3.299643572	113.7879011	375.4595165	-1.72231
Gdf11	Gdf11-001	421	0.039296976	0.080933339	0.303099945	-3.29924177	1.380661833	4.55513719	-1.722135
Traf1	Traf1-004	928.28553	0.007672268	0.022387488	0.303117113	-3.29905491	3.448268454	11.37602697	-1.722053
Aga	Aga-201	2126	7.95969E-05	0.000677821	0.30315545	-3.298637708	4.08156618	13.46360811	-1.72187
Mier1	Mier1-012	775.76361	0.003970435	0.013344703	0.303159081	-3.298598201	2.144654557	7.074353664	-1.721853
Rock2	Rock2-201	121854	2.81819E-07	1.66746E-05	0.303165603	-3.298527245	274.114729	904.1749018	-1.721822
Rpl9	Rpl9-001	14392.969	3.89545E-07	2.01715E-05	0.303180686	-3.298363139	46.05874409	151.9184637	-1.72175
Rpl36al	Rpl36al-001	12180.308	3.04036E-05	0.000337873	0.303194207	-3.298216047	40.19013369	132.5557439	-1.721686
Fat1	Fat1-201	2622.1034	0.025623819	0.05758256	0.303218125	-3.297955888	6.856589632	22.61273015	-1.721572
Tpm3	Tpm3-006	3634.8519	0.000623493	0.003169286	0.303221008	-3.297924533	11.34789786	37.42451074	-1.721558
Usp31	Usp31-201	9149.9814	4.81358E-06	9.53238E-05	0.303259159	-3.297509642	27.43014813	90.45117793	-1.721377
Gm37879	Gm37879-001	919	0.000403443	0.002265235	0.303265698	-3.297438539	2.920890331	9.631456347	-1.721346
Cyp2j13	Cyp2j13-201	187	0.022509975	0.052088821	0.30334389	-3.296588566	0.476158866	1.569699874	-1.720974
Gosr1	Gosr1-001	4922.1127	4.21126E-07	2.10256E-05	0.303364436	-3.296365302	13.40743938	44.19581796	-1.720876
Pigu	Pigu-003	641.24448	0.015344677	0.038561536	0.303389244	-3.296095755	1.970909682	6.496307039	-1.720758
Pten	Pten-001	29401.693	2.64538E-07	1.61538E-05	0.303438694	-3.295558606	78.73221835	259.4666397	-1.720523
Apobec3	Apobec3-006	2493.332	0.001158101	0.005112543	0.303450759	-3.295427582	8.108053188	26.71950211	-1.720466
Phip	Phip-013	813.90179	0.000961542	0.004413994	0.303463554	-3.295288634	2.191618411	7.222015239	-1.720405
Gm15774	Gm15774-001	921.3629	0.021678048	0.05057719	0.303466651	-3.295255002	2.167489694	7.142431257	-1.72039
Add1	Add1-005	3140.1711	0.004666565	0.015145441	0.303479161	-3.295119168	9.007041727	29.67927584	-1.720331
Cacfd1	Cacfd1-002	2645.9123	0.001846624	0.007351724	0.303485844	-3.29504661	7.21804488	23.78379431	-1.720299
Slc25a5	Slc25a5-001	8353.9753	0.040251346	0.082531645	0.303502781	-3.29486273	28.85709372	95.08016257	-1.720218
33431E20R	3431E20Rik-	1419.7652	0.000215756	0.001411583	0.30354926	-3.29435822	3.811743665	12.55724907	-1.719997
Efr3b	Efr3b-201	1796	0.002432531	0.009122322	0.303555193	-3.294293832	5.114871232	16.84988875	-1.719969
Mgat4a	Mgat4a-003	667.83993	0.006158274	0.018838824	0.303589846	-3.293917812	1.709872936	5.63218092	-1.719805
Pir	Pir-002	458.96126	0.01225221	0.032309685	0.303603191	-3.293773019	0.481102678	1.58464302	-1.719741
Fbxl3	Fbxl3-002	8696.6039	3.90607E-05	0.000403081	0.303670909	-3.293038522	22.2316095	73.2095465	-1.719419
Tor1b	Tor1b-002	3365.3413	0.00031078	0.001854482	0.303673757	-3.293007631	9.20846086	30.32353188	-1.719406
Chpt1	Chpt1-006	526.52664	0.000655414	0.003291947	0.303675542	-3.292988282	1.454558167	4.789843	-1.719397
Zfp605	Zfp605-001	1344.4729	0.000138783	0.0010222	0.303698209	-3.2927425	3.611038037	11.89021841	-1.71929
Papola	Papola-001	4044.2733	0.009761594	0.027038788	0.303715319	-3.292556996	11.9313184	39.28454586	-1.719208
Bloc1s2	Bloc1s2-201	794	0.01203879	0.031897077	0.303733684	-3.292357915	2.667675343	8.782942029	-1.719121
Psg16	Psg16-001	18080	1.01497E-06	3.53328E-05	0.303872976	-3.290848736	48.36346881	159.1568602	-1.71846
Dcp2	Dcp2-001	8388.6726	1.00266E-06	3.5006E-05	0.30391572	-3.290431372	24.63548539	81.06137397	-1.718277
Klf7	Klf7-001	48938.584	1.81365E-05	0.000235204	0.303949575	-3.290019405	113.9626355	374.9392822	-1.718096
Ppp3ca	Ppp3ca-008	2129.7004	0.004903033	0.015759092	0.303970176	-3.289796425	5.455086476	17.94612399	-1.717998
Atp5g3	Atp5g3-002	1818.5226	0.01220074	0.032220951	0.303988148	-3.289601937	5.147084894	16.93186043	-1.717913
Rpl7a	Rpl7a-001	11476.579	0.000458144	0.002499032	0.303996291	-3.28951382	38.27689342	125.9123699	-1.717874
Ripply3	Ripply3-001	1207	0.000478249	0.002583195	0.304064387	-3.288777123	3.042426055	10.00586121	-1.717551
Ccdc25	Ccdc25-201	3528	7.50531E-05	0.000649625	0.304068829	-3.288729081	10.14287107	33.35715505	-1.71753
Nfkb1	Nfkb1-001	5577.6218	0.028718913	0.063020423	0.304091271	-3.288486366	17.53276579	57.65626125	-1.717424
Gm37204	Gm37204-001	8936	3.82496E-06	8.16753E-05	0.304112563	-3.28825613	22.71032318	74.6773594	-1.717323
Mlh3	Mlh3-201	2360.3704	0.000121292	0.000923371	0.304116495	-3.288213612	7.006812991	23.03989786	-1.717304

Abhd11	Abhd11-006	510.31785	0.039896519	0.081936546	0.304152794	-3.287821184	1.510045644	4.964760057	-1.717132
Gimap5	Gimap5-001	3882.3997	3.47791E-05	0.000370611	0.304188025	-3.287440389	11.06066878	36.36128929	-1.716965
Nxf1	Nxf1-001	6311.4073	9.51608E-05	0.000769782	0.304293926	-3.286296291	18.73976941	61.58443469	-1.716463
Ptp4a1	Ptp4a1-005	5650.4661	0.002948118	0.010610871	0.304304769	-3.286179194	17.48517507	57.45941851	-1.716411
Vps54	Vps54-001	7356.0992	3.21292E-05	0.000351349	0.304338427	-3.285815761	20.16672683	66.26414885	-1.716252
Slc25a32	Slc25a32-201	3235	7.43602E-06	0.000126423	0.304362278	-3.285558269	8.846093964	29.06435717	-1.716139
Ints1	Ints1-005	457.22836	0.023114139	0.053149328	0.30437082	-3.285466063	1.273119448	4.18279074	-1.716098
Gm12430	Gm12430-001	754	0.001113742	0.004959134	0.304374143	-3.285430196	1.839659306	6.044072235	-1.716082
Txndc9	Txndc9-004	1943.6257	0.000147436	0.001066235	0.304409961	-3.285043615	4.984344936	16.37379051	-1.715913
Dnhd1	Dnhd1-001	869.16702	0.012450619	0.032722352	0.304411855	-3.285023185	1.961002167	6.441937586	-1.715904
Rpa2	Rpa2-001	1173.8501	0.018338134	0.044337223	0.304439735	-3.284722345	3.821451571	12.55240736	-1.715771
Ankrd28	Ankrd28-201	7045	2.65681E-05	0.000308006	0.30443979	-3.284721747	25.4623912	83.63687009	-1.715771
Etl4	Etl4-004	781.03016	0.049207099	0.096847669	0.30446848	-3.284412231	1.62618242	5.341053432	-1.715635
St7l	St7l-001	3317.2855	2.14577E-06	5.70153E-05	0.304474557	-3.284346682	8.822140929	28.97496929	-1.715606
Rtn4	Rtn4-007	1680.6423	0.002927126	0.010553059	0.304493207	-3.284145519	4.277800705	14.04892002	-1.715518
Cnr2	Cnr2-002	529.60016	0.001698772	0.006873969	0.304518739	-3.283870156	1.550461482	5.091514188	-1.715397
Cerkl	Cerkl-002	1006.5684	0.025312932	0.057048623	0.304552516	-3.283505957	2.081584423	6.834894852	-1.715237
Tipin	Tipin-201	2214	0.003427367	0.011907824	0.304593919	-3.283059633	7.344290192	24.11174266	-1.715041
Usp37	Usp37-003	2408.3473	8.15201E-06	0.000134777	0.304619118	-3.282788051	6.477061141	21.26281892	-1.714922
Rnf111	Rnf111-202	6918.9947	7.23758E-05	0.000633175	0.304626369	-3.282709904	20.71183577	67.99094839	-1.714887
Ptgs1	Ptgs1-003	30169.913	0.019983045	0.047482953	0.30465304	-3.282422528	41.89494888	137.516924	-1.714761
Cwf19l1	Cwf19l1-201	1409	0.000188439	0.001275249	0.30465403	-3.282411853	4.335238085	14.23003687	-1.714756
Lrp5	Lrp5-002	3449.2386	0.00197306	0.007755025	0.304663071	-3.282314448	6.676415629	21.91409351	-1.714713
Zfp398	Zfp398-002	1274.5579	0.000331527	0.001950998	0.304742669	-3.281457118	3.558086123	11.67570703	-1.714337
Rab11b	Rab11b-001	10863.039	5.79636E-08	6.91725E-06	0.304803296	-3.280804419	27.79334966	91.18454438	-1.71405
Rbbp7	Rbbp7-001	4402.9344	0.000214279	0.00140326	0.304839368	-3.2804162	12.24885945	40.18135696	-1.713879
Wdr45b	Wdr45b-003	1379.7153	0.00039374	0.002224979	0.304864266	-3.280148289	3.892351622	12.76749051	-1.713761
Zfat	Zfat-005	176.43288	0.045785032	0.091479252	0.304873891	-3.280044727	0.595645419	1.953743617	-1.713715
Gm17203	Gm17203-001	318	0.041666884	0.084865055	0.30487523	-3.280030325	0.869698045	2.852635962	-1.713709
Tmem254a	Tmem254a-00	1776.0377	5.17519E-05	0.000493321	0.304903698	-3.27972408	4.417707542	14.4888618	-1.713574
Ikzf2	Ikzf2-001	2100.3841	0.000171525	0.001189937	0.304953221	-3.279191464	6.488207932	21.27607607	-1.71334
Tmem209	Tmem209-00	774.06898	0.000203285	0.001349848	0.304958165	-3.279138302	2.022619236	6.632448207	-1.713317
P24-303G1	24-303G10.1-	1218	0.016254064	0.040302737	0.304968486	-3.279027325	3.755136478	12.31319512	-1.713268
Rufy2	Rufy2-002	2031.2479	0.000977952	0.004471345	0.304987975	-3.278817795	4.717573268	15.46806318	-1.713176
Rab27b	Rab27b-001	2739.1752	0.011262166	0.03025327	0.305120332	-3.277395492	5.961659954	19.53871746	-1.71255
Asxl2	Asxl2-007	9690.1572	2.53797E-06	6.33106E-05	0.305137101	-3.277215376	25.57355462	83.81004642	-1.71247
42615	Sept2-011	475.03597	0.00144228	0.00607229	0.305257462	-3.275923195	1.432805931	4.693762182	-1.711902
Odf2	Odf2-013	1465.1279	0.000242501	0.001540083	0.305269998	-3.275788672	4.06748865	13.32423324	-1.711842
Heg1	Heg1-007	1120.5418	0.010686978	0.029045056	0.305406993	-3.274319259	2.245053486	7.351021867	-1.711195
P23-155F13	23-155F13.3-	671	0.002457276	0.009199006	0.30542284	-3.274149374	1.701990365	5.572570689	-1.71112
Rsrc2	Rsrc2-202	1917.7554	0.000150422	0.001082036	0.30548413	-3.273492474	5.883378181	19.2591942	-1.710831
30419L09R	0419L09Rik-	1331.6218	0.001180898	0.00519401	0.305485562	-3.27347713	3.771182227	12.34487877	-1.710824
Map9	Map9-001	589.61858	0.008543762	0.02432451	0.305599622	-3.272255361	1.920668303	6.284917152	-1.710285
Emcn	Emcn-002	1520.2183	0.019484602	0.046537491	0.305604134	-3.272207045	6.356886715	20.80104949	-1.710264
Glmp	Glmp-001	2876.5463	0.00012759	0.000958875	0.305621684	-3.272019138	6.846637531	22.40232903	-1.710181
Chka	Chka-002	1098.9106	0.00860094	0.024452744	0.30575133	-3.270631728	2.339327132	7.65107754	-1.709569
P24-230J1	24-230J14.3-	995	0.000301572	0.001817178	0.305766035	-3.270474436	2.687340671	8.788878963	-1.7095
Rasgrf2	Rasgrf2-002	1462.1098	0.001501742	0.006264881	0.305812818	-3.26997412	2.907833549	9.508540448	-1.709279
530001G21	0001G21Rik-	916.06862	0.000455005	0.002487456	0.3058283	-3.269808585	2.886442054	9.438113008	-1.709206
Gm16121	Gm16121-001	317	0.04477601	0.089827878	0.30583322	-3.269755984	1.102481287	3.604844786	-1.709183
Lrpprc	Lrpprc-003	388.15026	0.033668148	0.07168557	0.305882399	-3.269230276	0.9914053	3.241132222	-1.708951
Zfp68	Zfp68-002	2979.1736	0.00408806	0.013640141	0.305948875	-3.26851994	7.974795049	26.06577664	-1.708637
Trmt1	Trmt1-009	1242.7921	0.005303863	0.016785929	0.305985379	-3.268130011	3.464015715	11.32085371	-1.708465
Synb	Synb-001	633	0.0064912	0.019599341	0.305999422	-3.267980029	1.742707819	5.695134349	-1.708399
Zc3h7a	Zc3h7a-018	867.4926	0.006080766	0.018661848	0.306002567	-3.267946447	2.404374886	7.857368366	-1.708384
Dynlt1c	Dynlt1c-002	11772.644	5.72484E-07	2.49778E-05	0.306068121	-3.267246506	32.19632483	105.1933298	-1.708075
Crtc2	Crtc2-003	964.86779	0.017512772	0.042761278	0.306146643	-3.266408506	3.241772834	10.58895436	-1.707705
Ap4s1	Ap4s1-201	2004	0.000440951	0.002427675	0.306168103	-3.266179558	5.135310132	16.77284498	-1.707604
Rpl9-ps7	Rpl9-ps7-001	2721	2.22055E-05	0.000272009	0.306187175	-3.265976112	8.775404178	28.66026042	-1.707514
10055G02	0055G02Rik-	3347	8.70005E-05	0.000720171	0.306198158	-3.265858964	9.060210167	29.58936859	-1.707462
Kpnb1	Kpnb1-001	38502.888	2.97159E-05	0.000333753	0.306345761	-3.264285416	116.3321331	379.7412854	-1.706767
Eif4e	Eif4e-004	1717.2477	0.000658516	0.00330367	0.30642888	-3.263399973	5.174691269	16.88708735	-1.706376
Usp3	Usp3-001	5735.4899	0.00047019	0.002551333	0.306452602	-3.263147365	15.77605731	51.47959985	-1.706264
Tnfrsf18	Tnfrsf18-005	609.78287	0.028731216	0.063033179	0.306571057	-3.26188652	1.57664313	5.142830973	-1.705707
Yeats4	Yeats4-201	3198	8.15007E-05	0.000690303	0.306583697	-3.26175204	8.607996481	28.07715008	-1.705647
Cep97	Cep97-001	2302.2797	8.11326E-05	0.000688331	0.306665355	-3.260883514	6.22248456	20.29079732	-1.705263
Psap	Psap-202	33574.412	0.000449268	0.002461991	0.306677391	-3.260755531	94.39205013	307.7893995	-1.705206
Srsf2	Srsf2-004	5294.6289	1.60389E-05	0.000214919	0.306739903	-3.260091011	14.75389701	48.099047	-1.704912
Mrpl1	Mrpl1-002	827.27836	0.000666124	0.003334725	0.306777142	-3.259695279	2.13221458	6.950369801	-1.704737
Acaca	Acaca-001	1120.1377	0.002826289	0.010257538	0.306844268	-3.25898218	2.964940786	9.662689187	-1.704421
Mapk9	Mapk9-002	870.16614	0.006845254	0.020450974	0.306874538	-3.258660709	2.571114204	8.378388834	-1.704279
Ddx58	Ddx58-006	1651.9703	0.011990791	0.031794117	0.306920125	-3.258176697	4.521109745	14.73057442	-1.704065
Fam210b	Fam210b-001	3580	2.21797E-05	0.00027179	0.306949694	-3.257862831	10.44446587	34.02663714	-1.703926
Txlng	Txlng-003	862.15861	0.009189476	0.025798323	0.30709001	-3.256374242	2.572819093	8.378061825	-1.703267
Gpkow	Gpkow-001	8186.2767	4.15167E-07	2.08191E-05	0.307116468	-3.256093716	23.4208045	76.26033437	-1.703142
Fam69a	Fam69a-001	2428.7982	6.41423E-05	0.000578734	0.307118165	-3.25607572	6.409633627	20.87025242	-1.703134
Wdr12	Wdr12-001	4721.7561	6.19628E-06	0.000112228	0.307182209	-3.255396867	13.73555118	44.71467029	-1.702833
Rbmx2	Rbmx2-001	1082.8635	0.0043505	0.014343919	0.307215691	-3.255042069	3.510622509	11.42722396	-1.702676
Ncor1	Ncor1-202	7188.2588	0.000256772	0.001606876	0.307242774	-3.254755147	17.86953238	58.16095248	-1.702549
Tyw1	Tyw1-002	463.88649	0.046340545	0.092357502	0.307262929	-3.254541653	1.032769973	3.361192896	-1.702454
Gprasp1	Gprasp1-005	1068.3967	0.001186288	0.005210356	0.307328639	-3.253845795	2.985045454	9.712877599	-1.702146
Zfyve28	Zfyve28-002	272.92422	0.006506539	0.019629082	0.307359504	-3.253519046	0.731644319	2.380418727	-1.702001
Brip1os	Brip1os-001	7258.3716	1.49304E-05	0.000204609	0.307418191	-3.252897942	20.26743199	65.9278878	-1.701726
Gm38115	Gm38115-001	506	0.021497749	0.050276402	0.307418215	-3.252897685	1.827963468	5.946178134	-1.701725
Gm37601	Gm37601-001	2447	3.87328E-05	0.000400785	0.307482845	-3.252213957	6.975909101	22.68714894	-1.701422
Atp11a	Atp11a-009	677.15457	0.00319985	0.011307766	0.307578753	-3.251199863	2.045872296	6.651539728	-1.700972
Dolpp1	Dolpp1-001	254.27454	0.025660813	0.057643001	0.307597867	-3.250997838	0.527658541	1.715416777	-1.700883
Csde1	Csde1-014	240.15476	0.022549368	0.052155311	0.307674269	-3.250190547	0.951745732	3.09335498	-1.700524
Zfp935	Zfp935-201	998	0.001290995	0.005570758	0.307691183	-3.250011883	2.604107915	8.463381669	-1.700445
Rbck1	Rbck1-007	2247.1094	0.000279479	0.001711451	0.307712714	-3.249784476	6.304773735	20.48915581	-1.700344
Gng5	Gng5-002	525.24511	0.003985321	0.013382083	0.307756779	-3.249319169	1.462266514	4.751370613	-1.700137
P23-385G2	23-385G21.1-	164	0.035820556	0.075248654	0.307779067	-3.249083866	0.456357769	1.482744663	-1.700033
Tufm	Tufm-201	792.69693	0.009058504	0.025510094	0.307879011	-3.248029142	2.677208397	8.695650892	-1.699565
Ascc3	Ascc3-201	16271	5.99824E-08	7.08413E-06	0.307889246	-3.247921172	46.87334582	152.2409323	-1.699517
Nfe2l2	Nfe2l2-001	23204.666	3.54324E-07	1.89915E-05	0.307953814	-3.247240181	60.80046742	197.4337208	-1.699214
Tcf20	Tcf20-202	4790.6124	0.003575037	0.012307267	0.307987862	-3.246881203	14.49919527	47.07716458	-1.699055
Cyb5r4	Cyb5r4-005	11500.091	1.28873E-06	4.10213E-05	0.307989357	-3.246865441	31.29594264	101.6137146	-1.699048
Apobec3	Apobec3-007	2925.2363	0.000146711	0.001063008	0.308099979	-3.245699667	9.294126933	30.16594469	-1.69853
Syncrip	Syncrip-004	3620.4582	0.000769336	0.003715422	0.308118293	-3.245506754	11.50521112	37.34024039	-1.698444
Nfu1	Nfu1-002	659.53977	0.009390083	0.026247478	0.308127453	-3.245410265	1.967572393	6.385579639	-1.698401
Wdhd1	Wdhd1-201	327.63522	0.049794529	0.097644717	0.308143932	-3.245236713	1.18926942	3.859460784	-1.698324
Gm5595	Gm5595-001	1722	0.00018899	0.001276958	0.308208987	-3.244551726	4.609457297	14.95562263	-1.698019
Chchd2	Chchd2-001	7327.8008	4.30814E-06	8.85678E-05	0.308314638	-3.2434399	21.15792933	68.62447219	-1.697525
Gm16153	Gm16153-001	999.02716	0.001106473	0.004930616	0.308408502	-3.242452769	2.648951871	8.589101332	-1.697086
Sp100	Sp100-002	3724.0547	0.002715643	0.009944484	0.308454837	-3.241965697	6.954475911	22.54617234	-1.696869
Ocrl	Ocrl-007	1530.0821	0.000459968	0.002507785	0.30848197	-3.241680541	4.405035446	14.27971769	-1.696742
Araf	Araf-003	3173.3231	0.000105368	0.000828288	0.308502151	-3.241468481	8.180087027	26.51549427	-1.696648

Edem3	Edem3-001	15480.226	6.16374E-07	2.5999E-05	0.308532252	-3.241152238	42.18752284	136.7361841	-1.696507
Marf1	Marf1-201	21824	9.48987E-06	0.000149507	0.308631462	-3.240110373	60.21746944	195.1112474	-1.696043
Gm20404	Gm20404-001	435	0.017770386	0.043239253	0.308683582	-3.239563286	1.200211169	3.888160039	-1.695799
Gm2223	Gm2223-001	4083	6.724E-06	0.000118466	0.308716904	-3.239213623	12.07944174	39.12789225	-1.695644
Oas1c	Oas1c-001	539.02629	0.039759096	0.08169222	0.308767255	-3.238685393	1.352568062	4.380542425	-1.695408
Golga7	Golga7-002	1184.0055	0.014568778	0.037043848	0.308786528	-3.238483252	4.289656982	13.89198229	-1.695318
Tbce	Tbce-006	293.94488	0.027369222	0.060712168	0.308787149	-3.238476743	0.977408501	3.165314697	-1.695315
Elf2	Elf2-008	724.18811	0.023938173	0.054604224	0.308796835	-3.238375158	2.2081063	7.150676589	-1.69527
00004A13R	0004A13Rik-	4156.7594	4.50657E-05	0.000447781	0.308798881	-3.238353707	10.64351868	34.46747818	-1.695261
Ncor2	Ncor2-010	764.80581	0.004685621	0.015198647	0.308813503	-3.238200367	1.747521125	5.658823547	-1.695192
Gm37696	Gm37696-001	668.37696	0.000695361	0.003448111	0.308823949	-3.238090839	1.564484556	5.065943109	-1.695143
Snrpe	Snrpe-001	1772.3332	0.00086768	0.00408327	0.308847025	-3.237848904	5.960034744	19.29769196	-1.695036
Lix1	Lix1-002	1709.1969	4.73752E-06	9.4392E-05	0.308866389	-3.237645905	4.21404991	13.64360144	-1.694945
Zmat3	Zmat3-001	8074.6355	1.58962E-06	4.64544E-05	0.308876942	-3.237535291	19.72163104	63.84947649	-1.694896
Zfp236	Zfp236-002	3882.1266	5.2564E-05	0.000499118	0.308950227	-3.236767326	10.96068856	35.47719859	-1.694554
Gm10222	Gm10222-001	21895	1.17379E-06	3.87268E-05	0.308970591	-3.236553991	60.32645983	195.2498443	-1.694459
Gm17800	Gm17800-001	619	0.00304498	0.010881736	0.309045351	-3.235771049	1.825333876	5.906362511	-1.69411
Rdh16	Rdh16-003	1054.67	0.000969685	0.004439476	0.309055449	-3.235665326	3.531675633	11.42732039	-1.694062
Nqo2	Nqo2-202	2423.8536	0.007330074	0.021601705	0.309106869	-3.235127071	7.558761545	24.4535541	-1.693822
Trerf1	Trerf1-003	1298.7976	0.021412074	0.0501171	0.30910711	-3.235124548	1.594455107	5.158260857	-1.693821
10203C20R	0203C20Rik-	2765.0387	0.000467974	0.002542128	0.309159385	-3.23457753	9.128376549	29.52644167	-1.693577
Echdc1	Echdc1-001	1751.3913	0.005928736	0.018274061	0.309171885	-3.234446751	5.558662394	17.97919752	-1.693519
Gm26884	Gm26884-201	433.94432	0.001801703	0.007211443	0.309187474	-3.234283672	1.108753961	3.586024833	-1.693446
Was	Was-001	781.73514	0.035029411	0.073958656	0.309303518	-3.233070243	2.938171256	9.499314058	-1.692905
Kmt2c	Kmt2c-006	2783.959	0.000322453	0.001908746	0.309546608	-3.230531279	6.891917034	22.26455355	-1.691771
Cgn	Cgn-004	409.80892	0.001013318	0.004603549	0.309559722	-3.230394421	1.239647561	4.004550565	-1.69171
Eif4e2	Eif4e2-002	689.57894	0.026057805	0.05838544	0.309569182	-3.230295705	1.808514652	5.842037111	-1.691666
Gm21092	Gm21092-202	30228.605	4.14525E-06	8.64646E-05	0.309589741	-3.230081192	82.76995858	267.3536865	-1.69157
RP24-79P8.	P24-79P8.1-0	1326	0.001126899	0.005004705	0.309631065	-3.229650099	3.648936779	11.78478903	-1.691378
Fam65b	Fam65b-003	1777.5127	0.013349786	0.03458363	0.309631254	-3.229648132	4.856812756	15.68579625	-1.691377
Rcor3	Rcor3-010	1395.1866	0.006446403	0.019499188	0.309633351	-3.229626257	2.922537124	9.438702633	-1.691367
Zfp606	Zfp606-002	1459.6396	0.003645222	0.012491129	0.309633616	-3.229623492	3.823942372	12.34989412	-1.691366
Zfp692	Zfp692-001	707.97522	0.021214741	0.049739889	0.309653849	-3.229412462	1.817208252	5.868514975	-1.691272
30405H02R	0405H02Rik-	1674.3803	6.78981E-06	0.000119106	0.309737881	-3.228536326	4.675234439	15.09416422	-1.69088
Tes3-ps	Tes3-ps-201	293	0.008884605	0.02509258	0.30974541	-3.228457844	0.883024857	2.850808525	-1.690845
Kdelr3	Kdelr3-201	1134	0.008222426	0.023601918	0.30976388	-3.228265352	3.919209748	12.65224904	-1.690759
Rab14	Rab14-009	4718.5567	6.35119E-06	0.000114131	0.309765467	-3.228248807	12.20750896	39.40887624	-1.690752
Mcf2l	Mcf2l-017	173.67873	0.025114725	0.056687712	0.309781763	-3.228078993	0.236877975	0.764660814	-1.690676
Sypl	Sypl-005	161.05823	0.048041154	0.095072829	0.309784257	-3.228053	0.423083643	1.365736423	-1.690664
Wdr20	Wdr20-003	822.88226	0.001668399	0.006784431	0.309793884	-3.227952689	2.387533043	7.706843707	-1.690619
Tbc1d4	Tbc1d4-008	1144.933	0.045725994	0.091393283	0.309861064	-3.227252842	2.741177125	8.846471668	-1.690307
Rbm5	Rbm5-020	3301.7322	3.1085E-05	0.000343439	0.30993609	-3.226471629	8.88171858	28.65661302	-1.689957
Gls	Gls-002	26363.125	0.000288376	0.001755268	0.309947612	-3.226351679	70.90108691	228.7518408	-1.689904
Zmym4	Zmym4-001	9386.3938	9.62364E-05	0.000775187	0.309952728	-3.226298435	26.22888677	84.62221632	-1.68988
Sfrp2	Sfrp2-001	2205.1625	0.010505199	0.028674137	0.30995739	-3.226249908	6.330229155	20.42290123	-1.689858
Ppm1h	Ppm1h-001	2725.4111	0.000238555	0.001520376	0.309979628	-3.226018457	9.634863441	31.08224729	-1.689755
Klf13	Klf13-003	813.94194	0.043588726	0.087945613	0.310099961	-3.22476661	2.087514313	6.731746454	-1.689195
Gdpgp1	Gdpgp1-001	1527	0.000818071	0.003895845	0.310134773	-3.224404634	4.254987174	13.71980036	-1.689033
10002H16R	0002H16Rik-	2712.183	4.48992E-05	0.000446775	0.310162285	-3.22411863	7.794981408	25.13194478	-1.688905
Zfp652	Zfp652-001	14187.041	1.26993E-07	1.09317E-05	0.310228654	-3.223428876	41.23468737	132.917082	-1.688596
Serinc5	Serinc5-201	2495	0.000214217	0.001403126	0.310234671	-3.223366351	8.392888483	27.05335432	-1.688568
Oas1b	Oas1b-001	1004.2762	0.002490949	0.009292389	0.31024139	-3.22329654	2.582990277	8.325743621	-1.688537
Gm28941	Gm28941-001	504	0.032023009	0.068807282	0.310375673	-3.221901995	1.454587909	4.686539685	-1.687913
Olfm1	Olfm1-201	3134.2418	0.003079559	0.010967547	0.310397995	-3.221670294	6.047742646	19.48383283	-1.687809
Impa1	Impa1-001	1465.1078	0.000636087	0.003218493	0.310553036	-3.220061898	4.390043503	14.13621182	-1.687088
Kremen2	Kremen2-201	219	0.028460735	0.062598264	0.310554623	-3.22004545	0.606690061	1.953569572	-1.687081
Cops5	Cops5-001	1941.89	0.015978504	0.039763407	0.310557874	-3.220011738	5.236212304	16.86066508	-1.687066
Hax1	Hax1-001	1096.3834	0.014233627	0.036369869	0.310562769	-3.219960989	3.431901363	11.05058851	-1.687043
Gm11964	Gm11964-001	233	0.030636254	0.066461343	0.310611454	-3.219456287	0.621580815	2.001152262	-1.686817
Tle3	Tle3-012	2116.2871	0.000680728	0.003388308	0.310645287	-3.219105651	6.119572205	19.69954946	-1.68666
Gm13236	Gm13236-001	987	0.000261241	0.00162593	0.310689871	-3.218643717	2.761277016	8.887566919	-1.686453
Fchsd2	Fchsd2-005	1403.7758	0.000198689	0.001326269	0.310693189	-3.218609337	2.927568094	9.422698004	-1.686437
Pold3	Pold3-001	2498.6962	6.88453E-05	0.00061071	0.310709238	-3.218443085	7.916874906	25.48001129	-1.686363
Aff4	Aff4-005	2479.3223	8.83031E-06	0.00014234	0.310755074	-3.217968378	6.986538941	22.48246138	-1.68615
Gm36936	Gm36936-001	3522	2.64754E-05	0.000307383	0.310947873	-3.215973114	9.910213657	31.87098068	-1.685255
Asap1	Asap1-201	347.06433	0.025736351	0.057763436	0.310964309	-3.215803132	1.328234518	4.271340724	-1.685179
Sf1	Sf1-002	3146.5542	5.44024E-05	0.000512683	0.310982371	-3.215616356	9.558808535	30.73746107	-1.685095
Ythdc1	Ythdc1-009	2902	2.23064E-05	0.00027266	0.311020987	-3.215217111	8.327540301	26.77485007	-1.684916
Gtpbp8	Gtpbp8-001	2743.4676	9.62691E-06	0.000150558	0.311075842	-3.214650138	7.516341452	24.16240809	-1.684662
Xrcc5	Xrcc5-201	2395	5.43781E-05	0.000512648	0.311222967	-3.213130474	6.837428955	21.96955134	-1.68398
Zfp101	Zfp101-001	1323.7187	0.001632806	0.006673461	0.311252787	-3.212822637	3.685996718	11.8424537	-1.683841
AW549877	W549877-20	9819	5.07217E-05	0.000485754	0.311255793	-3.212791614	27.73917904	89.1202018	-1.683827
Schip1	Schip1-005	647.12037	0.005411824	0.017053369	0.311343661	-3.211884888	1.471764824	4.727139196	-1.68342
P24-189M4	24-189M4.1-0	694	0.000389245	0.002205767	0.311452396	-3.210763548	1.916955736	6.154891598	-1.682916
Msl2	Msl2-001	8240.9891	6.12903E-07	2.5948E-05	0.311510046	-3.210169349	23.09718778	74.14588425	-1.682649
Sirt6	Sirt6-001	873.89007	0.017379902	0.042494407	0.311533493	-3.209927739	2.740406248	8.796506031	-1.682541
Iscu	Iscu-002	663.15071	0.012771555	0.033372702	0.311536919	-3.209892441	1.758230922	5.643732147	-1.682525
Slc9a8	Slc9a8-003	448.41264	0.003039326	0.010868307	0.311675555	-3.208464651	1.554697025	4.988190447	-1.681883
Smim14	Smim14-008	2089.6358	0.00026083	0.001624711	0.311719901	-3.208008208	4.986325301	15.99617249	-1.681678
Ccdc127	Ccdc127-001	5051.2093	2.03858E-06	5.48048E-05	0.311761616	-3.207578961	13.76472874	44.15145433	-1.681485
Gm27818	Gm27818-201	287	0.016357289	0.040517621	0.311783005	-3.207358918	0.819214959	2.627516405	-1.681386
Ensa	Ensa-003	952.80858	0.000762811	0.003693265	0.311790843	-3.207278287	2.439746406	7.824945675	-1.68135
Gm38125	Gm38125-001	2948	0.000476889	0.002579916	0.311970663	-3.205429611	7.503355277	24.05147719	-1.680518
10004F10R	0004F10Rik-	2554.9509	0.011487233	0.030761354	0.311972077	-3.20541508	7.901178408	25.32655642	-1.680511
Cdc40	Cdc40-201	7881	1.78997E-06	4.98835E-05	0.311997264	-3.205156312	22.10787193	70.85918526	-1.680395
Snx10	Snx10-201	1285.0044	0.015999749	0.03979879	0.312035633	-3.204762195	4.316533192	13.83346239	-1.680217
Myo1c	Myo1c-004	10072.2	5.17465E-05	0.000493321	0.312067684	-3.204433049	25.96979653	83.21847426	-1.680069
Sdf4	Sdf4-001	25842.66	0.000764093	0.003697399	0.312108393	-3.204015091	68.45737762	219.338471	-1.679881
Tmem44	Tmem44-005	1829.5552	0.025911867	0.058096454	0.312131332	-3.203779618	3.991774328	12.78876523	-1.679775
Pcdhb22	Pcdhb22-001	1851.591	5.3267E-05	0.000504395	0.312137975	-3.203711439	4.488707423	14.38052332	-1.679744
Thap4	Thap4-001	403.59886	0.010451294	0.028574823	0.312142446	-3.203665543	0.851022671	2.726392009	-1.679724
Gosr2	Gosr2-001	8944.7867	0.000103521	0.000818792	0.312154578	-3.203541039	19.89517452	63.73500806	-1.679667
Osmr	Osmr-002	7705.4513	0.0012537	0.005444431	0.312285186	-3.202201207	22.5957327	72.35608253	-1.679064
Apc	Apc-007	564.4571	0.006156064	0.018833871	0.312318095	-3.201863789	1.148287691	3.676660777	-1.678912
Ilf3	Ilf3-202	2535.2186	0.006864585	0.020496212	0.312371752	-3.201313802	8.04861479	25.76614161	-1.678664
Cdk11b	Cdk11b-003	6671.9235	0.000236542	0.001512049	0.312390296	-3.201123758	17.4015897	55.70464221	-1.678578
Gm17538	Gm17538-201	577	0.000647709	0.003262836	0.312433745	-3.2006786	1.688111912	5.403103671	-1.678378
Skp1a	Skp1a-001	8184.2628	1.86866E-05	0.000240246	0.31244084	-3.200605916	26.26929143	84.07764958	-1.678345
Ube2f	Ube2f-003	332.06749	0.016624893	0.041040982	0.312566595	-3.199318216	1.353185164	4.329269945	-1.677764
Mrps5	Mrps5-001	2242.311	0.006077151	0.018652426	0.312577695	-3.199204606	7.071439619	22.6229822	-1.677713
Adam4	Adam4-001	544	0.004767285	0.015402275	0.31262763	-3.198693601	1.190770426	3.808909742	-1.677483
Snrpa1	Snrpa1-003	536.39404	0.00998925	0.027555719	0.312648911	-3.198475878	1.685959597	5.392501102	-1.677385
Slirp	Slirp-003	941.818	0.009827166	0.027178652	0.312705529	-3.19789677	2.706403545	8.654799153	-1.677123
Lima1	Lima1-003	30368.334	0.00011918	0.000911348	0.312726953	-3.197677692	81.75058868	261.4113942	-1.677025
Zxdc	Zxdc-001	2775.1108	2.28842E-05	0.000277544	0.312747903	-3.197463483	7.91020082	25.29257827	-1.676928
Tmem47	Tmem47-001	5040.8213	7.21774E-06	0.000123976	0.312840053	-3.196521644	15.00967688	47.978757	-1.676503
Nav2	Nav2-009	5493.7487	0.00152986	0.006346621	0.312971003	-3.195184184	20.20491774	64.55843361	-1.675899

Hdac4	Hdac4-001	5044.4052	5.17802E-07	2.36686E-05	0.312988816	-3.195002345	14.15791643	45.23457618	-1.675817
St13	St13-001	4534.5819	3.9308E-05	0.000404902	0.312995766	-3.194931397	13.47990437	43.06736969	-1.675785
00094K13R	0094K13Rik-	624.26419	0.020400742	0.048231202	0.313005161	-3.194835501	2.386978141	7.626002506	-1.675742
Gm6395	Gm6395-201	2137	6.11263E-05	0.000558286	0.313007718	-3.194809397	6.577143253	21.01271907	-1.67573
Larp7	Larp7-001	5871.2797	4.70459E-05	0.000460726	0.313045615	-3.194422639	16.96018182	54.17798878	-1.675555
Emc4	Emc4-001	3753.9203	1.90575E-05	0.000243734	0.313115057	-3.193714189	9.981561263	31.87825384	-1.675235
Fxr1	Fxr1-009	702.137	0.032373988	0.069417709	0.313144917	-3.193409651	2.363565813	7.547833877	-1.675098
Wdr25	Wdr25-202	420.74296	0.043032892	0.087067377	0.313172958	-3.193123723	1.122787488	3.585199365	-1.674968
10474O19R	0474O19Rik-	520.21096	0.007243735	0.0214152	0.313236773	-3.192473185	1.204607178	3.845676115	-1.674675
Otud6b	Otud6b-002	1216.0731	0.005870375	0.018128243	0.313258033	-3.192256527	3.476669856	11.09842204	-1.674577
Sec63	Sec63-001	24794.837	8.5226E-07	3.19365E-05	0.313288955	-3.191941441	68.04005967	217.1798861	-1.674434
Huwe1	Huwe1-016	678.43431	0.048855689	0.096338727	0.313369219	-3.191123888	2.24137022	7.152490051	-1.674065
Wtap	Wtap-003	1194.9842	0.012064912	0.031943959	0.313382251	-3.190991183	2.991043488	9.544393398	-1.674005
Cybrd1	Cybrd1-001	1238	0.000221	0.001436813	0.31344643	-3.190337819	3.534020005	11.27471767	-1.673709
Mak16	Mak16-201	4085.3332	0.000396991	0.002237444	0.313458316	-3.190216842	14.05264201	44.83097522	-1.673654
Tra2b	Tra2b-001	5418.1389	0.000225976	0.00146197	0.313471253	-3.190085182	17.61783064	56.20238045	-1.673595
Gm4963	Gm4963-001	4129	9.92378E-06	0.000153708	0.313521765	-3.189571221	13.33843258	42.5438807	-1.673362
P23-167C5.	23-167C5.1-(	2065	7.70387E-06	0.000129694	0.313571182	-3.189068569	5.819724313	18.55949988	-1.673135
Sft2d2	Sft2d2-001	25599.485	1.08667E-06	3.68866E-05	0.313649622	-3.188271019	73.04346202	232.8823531	-1.672774
Blmh	Blmh-001	4327.6997	8.09855E-06	0.000134292	0.313650123	-3.188265927	12.06094086	38.4534868	-1.672772
Usp37	Usp37-002	3876.335	0.001050188	0.004729644	0.313657852	-3.188187362	11.79192492	37.59486599	-1.672736
Fndc3a	Fndc3a-004	892.69822	0.001987076	0.007795803	0.31365874	-3.188178336	2.775136822	8.847631098	-1.672732
Zeb1	Zeb1-005	2828.1999	0.001291086	0.005570758	0.3136735	-3.18802832	8.004542161	25.5187071	-1.672664
Xrcc2	Xrcc2-001	677.34446	0.004884722	0.015706134	0.313681142	-3.187950652	2.016915271	6.429826353	-1.672629
Gramd4	Gramd4-001	3530.4679	0.019789715	0.047121645	0.313685722	-3.187904107	10.01600453	31.93006197	-1.672608
Itfg1	Itfg1-201	9275	1.26035E-06	4.03052E-05	0.313896226	-3.185766244	23.01960021	73.3350653	-1.67164
30400D17R	0400D17Rik-	684.86648	0.005646241	0.017596337	0.313897281	-3.185755532	1.888811584	6.017291952	-1.671636
Arl5a	Arl5a-001	10749.244	2.22369E-06	5.83932E-05	0.313899827	-3.185729692	32.5379372	103.6570727	-1.671624
Sec31a	Sec31a-004	1188.3203	0.025343679	0.057106645	0.313934835	-3.18537444	3.15749284	10.05779699	-1.671463
Gm37813	Gm37813-001	648	0.000431508	0.002386427	0.31394723	-3.185248682	1.824376632	5.811093262	-1.671406
Adss	Adss-001	14300.104	1.91226E-06	5.21456E-05	0.314036381	-3.184344429	44.37660811	141.3104048	-1.670996
Tnpo1	Tnpo1-001	13294.733	1.72425E-06	4.84857E-05	0.314039593	-3.184311855	39.40153214	125.4667659	-1.670982
Tnks2	Tnks2-009	688.204	0.004508279	0.014751987	0.314043488	-3.184272369	1.65494401	5.269792482	-1.670964
Proser3	Proser3-002	359.49084	0.006141388	0.01880577	0.314103126	-3.183667774	0.951350859	3.028785072	-1.67069
Smek2	Smek2-003	10442.682	1.05058E-06	3.60544E-05	0.314212777	-3.182556767	28.61214318	91.0597699	-1.670186
Hsbp1	Hsbp1-201	8609	1.17102E-06	3.87268E-05	0.314246339	-3.182216863	21.66713622	68.94952625	-1.670032
Gm13251	Gm13251-001	1568	0.000754834	0.003664501	0.31426125	-3.18206588	5.029876851	16.00539951	-1.669964
10018L13R	0018L13Rik-	672.62756	0.006634328	0.019937328	0.314269909	-3.181978198	1.688985158	5.374313948	-1.669924
Gm5776	Gm5776-001	157	0.042214494	0.085791635	0.314304623	-3.181626766	0.413629396	1.316014358	-1.669765
Gid4	Gid4-001	5759.1172	2.33157E-05	0.000281284	0.314343813	-3.181230105	17.3056659	55.05330534	-1.669585
Msrb1	Msrb1-001	1488.3204	0.029361691	0.064168004	0.314371516	-3.180949768	6.028925145	19.17770804	-1.669458
Pcdh19	Pcdh19-004	5693.6831	0.001968185	0.007741195	0.314372154	-3.180943305	11.37242933	36.17505295	-1.669455
Ccnd2	Ccnd2-001	33233.703	7.04887E-06	0.000122054	0.314497192	-3.179678629	70.96688487	225.6518872	-1.668881
Gm26668	Gm26668-201	710.71984	0.008389586	0.023977246	0.314502106	-3.17962895	2.360720363	7.506214809	-1.668858
Gm38378	Gm38378-001	687	0.02665393	0.059410197	0.314516865	-3.179479741	1.892137924	6.016014196	-1.668791
Asap1	Asap1-001	179.82943	0.034040469	0.072320953	0.314653671	-3.178097354	0.61847468	1.965572743	-1.668163
Xpnpep1	Xpnpep1-001	607.07027	0.002880579	0.010423017	0.314686244	-3.177768393	1.642286393	5.218805792	-1.668014
Nudt5	Nudt5-002	707.41694	0.001657804	0.006753919	0.314695281	-3.177677137	2.20089898	6.993746371	-1.667973
Fndc1	Fndc1-005	2427.7601	0.042169488	0.085715428	0.314704517	-3.177583876	2.416196599	7.677667354	-1.66793
10037F02R	0037F02Rik-	2741.5395	0.00942438	0.026321863	0.314751778	-3.17710675	8.313003571	26.41129976	-1.667714
Rcor3	Rcor3-001	2217.2883	0.00102085	0.004627945	0.314791591	-3.176704935	6.031541487	19.16042761	-1.667531
Gpd2	Gpd2-201	6506.1202	0.000639096	0.003230376	0.314809709	-3.176522108	14.90496069	47.34593716	-1.667448
Brcc3	Brcc3-003	1279.0466	0.00338882	0.011802631	0.314875191	-3.175861514	4.119682716	13.08354179	-1.667148
Fam210a	Fam210a-001	10327.01	4.70002E-05	0.000460726	0.314889443	-3.175717773	31.73819469	100.791549	-1.667083
Mettl17	Mettl17-006	1918.0917	5.1577E-05	0.000492476	0.314918114	-3.175428641	5.393116667	17.12545713	-1.666951
Lmbrd1	Lmbrd1-002	7321.6594	0.000461701	0.002514429	0.314966087	-3.174944992	18.70428857	59.38508731	-1.666732
Gm7102	Gm7102-001	2125	3.94423E-05	0.000406041	0.314998347	-3.174619837	6.047583529	19.19877864	-1.666584
Gpx1	Gpx1-003	6469.4481	0.006815054	0.020378007	0.315073053	-3.173867113	18.52595888	58.79893163	-1.666242
Man1a2	Man1a2-001	10097.586	2.59135E-07	1.60262E-05	0.315074652	-3.173851006	28.11758095	89.24101258	-1.666234
Car5b	Car5b-002	600.52429	0.012117421	0.032054921	0.315095389	-3.173642121	1.821255675	5.780013724	-1.666139
Ciz1	Ciz1-201	5344.6558	0.003665773	0.012547744	0.315098373	-3.173612073	12.49551056	39.65590317	-1.666126
Rab2a	Rab2a-001	25975	9.79492E-08	9.39677E-06	0.315168064	-3.17291031	69.85485493	221.6431894	-1.665807
Clic4	Clic4-002	2015.4245	0.001613347	0.006610495	0.315207964	-3.172508671	5.949084779	18.87352304	-1.665624
Ackr4	Ackr4-001	1682.4007	0.027303914	0.060622265	0.315297601	-3.171606748	3.957116853	12.55041851	-1.665214
Gm37023	Gm37023-001	511	0.014096809	0.036100301	0.315383436	-3.170743561	1.736964024	5.507467495	-1.664821
Platr17	Platr17-001	409	0.001306959	0.005622122	0.315384188	-3.170736007	1.112124832	3.526254248	-1.664818
Nanos1	Nanos1-201	462	0.013972726	0.035836892	0.31543053	-3.170270172	1.471757708	4.665869562	-1.664606
Phf14	Phf14-201	6773.4483	0.000384356	0.002184098	0.315454648	-3.17002779	18.94943618	60.07023931	-1.664495
Cryzl1	Cryzl1-002	364.32153	0.021124737	0.049586886	0.315461409	-3.169959851	0.711783011	2.256323568	-1.664465
Gm2824	Gm2824-201	673	0.001359661	0.005794251	0.315461977	-3.169954143	1.946532735	6.170419508	-1.664462
'00128F08R	0128F08Rik-	1758	4.8127E-05	0.000468414	0.315529671	-3.16927406	4.843491567	15.35035218	-1.664152
Syncrip	Syncrip-005	3738.5511	0.002074193	0.008055461	0.315606275	-3.168504806	11.81740099	37.44349185	-1.663802
Cand2	Cand2-001	1272	0.019064699	0.045741923	0.315611106	-3.168456307	4.590329599	14.54425877	-1.66378
Utp6	Utp6-001	4684.205	1.54008E-05	0.000209399	0.315628393	-3.168282769	13.7839793	43.67153779	-1.663701
Dnm1l	Dnm1l-201	5874.9653	0.000267755	0.001657142	0.31564662	-3.168099824	14.50948766	45.9675053	-1.663618
Gfm2	Gfm2-007	638.78609	0.012422336	0.032660245	0.315658722	-3.167978357	1.646812903	5.217067635	-1.663562
Trim44	Trim44-001	30111.307	2.00572E-08	3.56435E-06	0.315672453	-3.167840559	85.59446552	271.1496195	-1.6635
Rassf5	Rassf5-003	889.65134	0.04860953	0.095947233	0.315696601	-3.167598249	3.697798844	11.71314114	-1.663389
Ccdc55	Ccdc55-001	10810.268	6.9414E-06	0.000120743	0.315740688	-3.167155955	30.68893925	97.19665672	-1.663188
Jam2	Jam2-001	56371.065	0.005576791	0.017437019	0.315745023	-3.167112466	96.03813768	304.1635831	-1.663168
Cdk14	Cdk14-015	376.5206	0.019627762	0.046830433	0.315750116	-3.16706139	0.891189029	2.822450366	-1.663145
Rnf135	Rnf135-001	899.60171	0.00470567	0.015249239	0.31584235	-3.166136521	2.567401261	8.128742898	-1.662723
Gm13632	Gm13632-001	875	0.003285364	0.011532049	0.31587913	-3.16576787	2.377664896	7.527135132	-1.662555
Setd7	Setd7-002	721.8966	0.010781339	0.029214531	0.31592606	-3.165297598	2.106534554	6.667808763	-1.662341
P24-324M1(	24-324M10.2-	1007	0.000766928	0.003706718	0.315927577	-3.165282403	2.599439711	8.227960775	-1.662334
Slc30a1	Slc30a1-001	5726.729	4.25631E-05	0.000430026	0.315927581	-3.165282361	13.66323708	43.24800331	-1.662334
Ercc6	Ercc6-201	5482	6.12105E-05	0.000558554	0.315953231	-3.165025394	16.80095419	53.17544666	-1.662217
Cers5	Cers5-001	6650.1376	2.46825E-05	0.000292518	0.315972007	-3.164837319	17.91156342	56.68718434	-1.662131
Ppp1r10	Ppp1r10-001	2535.0135	0.000119173	0.000911348	0.31597468	-3.164810546	7.063230275	22.35378567	-1.662119
Gm7336	Gm7336-201	1893	0.003202987	0.011315349	0.31598244	-3.164732822	6.253807543	19.79162999	-1.662084
Hnrnpa0	Hnrnpa0-201	13623	2.63371E-05	0.000306298	0.316106455	-3.163491241	42.43335483	134.2375463	-1.661518
Gm38043	Gm38043-001	1750	0.000104779	0.000824384	0.316116787	-3.163387838	4.31303789	13.6438116	-1.66147
Prepl	Prepl-202	1654.5814	0.002956599	0.010633531	0.316167481	-3.162880621	3.555535799	11.24573527	-1.661239
Acp6	Acp6-009	1892.0908	4.88666E-05	0.000472791	0.316212158	-3.162433752	5.692745466	18.0029304	-1.661035
Ogt	Ogt-004	11989.22	1.83345E-05	0.000237233	0.316216278	-3.162392549	31.08733892	98.31036897	-1.661016
Rnf207	Rnf207-002	535.70061	0.010268393	0.028168884	0.316315243	-3.161403134	1.385546052	4.38026963	-1.660565
Lactb2	Lactb2-001	3543.3153	2.35617E-06	6.0223E-05	0.31631884	-3.161367184	9.820218825	31.04531753	-1.660549
Dpm2	Dpm2-001	1210.0116	0.001523523	0.006327229	0.316374482	-3.160811182	3.042828507	9.617806369	-1.660295
P24-529E15	24-529E15.3-(	354.90814	0.01111475	0.029925334	0.316384547	-3.160710627	0.971714245	3.07130754	-1.660249
Bnip3l	Bnip3l-002	16412.379	0.000149043	0.001074903	0.316447716	-3.160079691	44.00780006	139.0681552	-1.659961
Slc22a15	Slc22a15-004	494.03347	0.00648022	0.01957681	0.316460833	-3.159948712	1.207806643	3.816607046	-1.659901
Rpl13-ps3	Rpl13-ps3-201	2654	3.09829E-06	7.20899E-05	0.31651215	-3.15943638	8.642824101	27.30645289	-1.659667
Utp15	Utp15-201	3250	0.000249497	0.001573251	0.316572711	-3.158831967	11.43757226	36.12936888	-1.659391
Tmem41b	Tmem41b-00	430.16093	0.045263055	0.090600177	0.316577521	-3.158783978	1.26197429	3.986304169	-1.659369
Spaca6	Spaca6-003	170.8651	0.04243684	0.086100453	0.316624318	-3.158317104	0.385225545	1.216664427	-1.659156
Usp32	Usp32-001	12383.908	1.07282E-06	3.65613E-05	0.316626428	-3.158296063	32.2039773	101.7096947	-1.659146
Gna13	Gna13-001	13693.023	2.4435E-07	1.56722E-05	0.316632399	-3.158236505	40.63943733	128.3489545	-1.659119

Dock8	Dock8-201	3654	0.01585383	0.039537523	0.316642385	-3.158136899	14.72411717	46.50077775	-1.659074
Tmem168	Tmem168-001	2118.2143	0.003603419	0.012378846	0.31676103	-3.156953993	6.172547603	19.4864488	-1.658533
Wrb	Wrb-001	2232.5778	3.77825E-05	0.000393567	0.316789064	-3.156674629	6.395939341	20.18989945	-1.658406
Irak2	Irak2-004	1368.1757	0.019525618	0.046622448	0.316817979	-3.156386529	4.489405633	14.17029947	-1.658274
Rnf122	Rnf122-002	242.10869	0.025902326	0.058086471	0.31683009	-3.156265874	0.777793231	2.454922233	-1.658219
Hps5	Hps5-004	2562.1142	0.00076648	0.003705808	0.316852814	-3.156039511	6.374703026	20.11881462	-1.658115
Trnau1ap	Trnau1ap-002	865.55047	0.028142887	0.062034977	0.316861449	-3.155953506	1.800344028	5.681802046	-1.658076
BC030336	BC030336-20	4702	8.14808E-06	0.000134777	0.316958619	-3.154985981	13.11024162	41.36262852	-1.657634
Bmpr2	Bmpr2-001	212827.84	6.60058E-05	0.000591342	0.317011003	-3.154464645	531.3179222	1676.023601	-1.657395
Cables2	Cables2-002	1707.341	0.000343236	0.002001335	0.317019942	-3.154375694	4.20580342	13.26668408	-1.657354
30458C11R	0458C11Rik-	3677	1.34459E-05	0.00019016	0.317031051	-3.154265159	10.21959191	32.23530269	-1.657304
Sp100	Sp100-005	4809.0834	0.000553637	0.002895413	0.317097181	-3.153607346	11.41203144	35.98906618	-1.657003
Tbc1d8b	Tbc1d8b-001	5858.3268	2.38589E-06	6.08012E-05	0.317115259	-3.153427571	15.79436161	49.80637536	-1.656921
Gm10778	Gm10778-001	836.96241	0.002010454	0.007857817	0.317217216	-3.152414026	2.676708879	8.438094614	-1.656457
Trrap	Trrap-013	1494.3788	0.000337732	0.001978319	0.317241833	-3.152169401	4.370687119	13.7771462	-1.656345
Klhl42	Klhl42-001	4565	5.28403E-05	0.000501185	0.317249258	-3.152095629	10.92840087	34.44736463	-1.656311
Mrpl50	Mrpl50-001	2398	0.000127489	0.00095883	0.317308929	-3.151502873	6.981099548	22.00095528	-1.65604
Mplkip	Mplkip-201	2204	6.57793E-05	0.000589775	0.317336404	-3.151230008	5.819842659	18.33966283	-1.655915
Gnai2	Gnai2-006	480.61825	0.012111884	0.032045735	0.317373849	-3.150858214	1.627065044	5.126651257	-1.655745
Dag1	Dag1-001	19760.036	5.10499E-06	9.92315E-05	0.317381068	-3.150786552	46.23805424	145.6862395	-1.655712
Hdgfrp3	Hdgfrp3-001	10977.991	6.89372E-06	0.00012028	0.317401107	-3.150587627	29.48551873	92.8967105	-1.655621
Gm9844	Gm9844-001	1943	0.002684611	0.009850861	0.317412383	-3.150475701	6.933405775	21.84352642	-1.65557
Adarb1	Adarb1-009	833.22335	0.005582975	0.017446797	0.317419408	-3.150405972	2.566689356	8.086113476	-1.655538
Tbpl1	Tbpl1-001	3953.457	1.00078E-05	0.000154609	0.317425572	-3.150344803	12.64059899	39.82224533	-1.65551
Ppig	Ppig-011	3645.3551	0.000202824	0.001347306	0.317480877	-3.149796014	10.28303998	32.38947835	-1.655258
42437	March8-001	5346.5764	6.74659E-05	0.000601567	0.317481155	-3.149793253	13.9653987	43.98811861	-1.655257
P23-34P11.	23-34P11.2-0	1092.8792	0.000827453	0.003931781	0.317486865	-3.149736608	2.707337979	8.527401543	-1.655231
Mapkap1	Mapkap1-007	985.15026	0.009659291	0.026813967	0.317496494	-3.149641081	2.703224793	8.514187858	-1.655187
Abcg1	Abcg1-002	391.81012	0.04496245	0.090098521	0.317558424	-3.149026839	1.185447829	3.733007031	-1.654906
Slc26a8	Slc26a8-001	159.89922	0.037114372	0.077448806	0.317576575	-3.14884686	0.428667894	1.349809553	-1.654824
Slc39a14	Slc39a14-009	2087.7347	0.000142742	0.001042415	0.317578633	-3.148826452	5.905356727	18.59494347	-1.654814
P23-422D12	23-422D12.3-	2272	0.000111775	0.000866911	0.317621817	-3.148398333	6.003354505	18.90095131	-1.654618
Gm13669	Gm13669-001	241	0.013527577	0.034935871	0.317626554	-3.148351382	0.738828279	2.326091034	-1.654597
Zfp672	Zfp672-007	1525.7281	0.012474152	0.032773834	0.317634476	-3.148272863	4.605868268	14.50053008	-1.654561
Parvg	Parvg-001	720.60378	0.026809961	0.059695823	0.317652219	-3.148097012	3.094103597	9.740538288	-1.65448
Dnajb4	Dnajb4-002	8054.2264	0.002480647	0.009268803	0.317665351	-3.147966863	25.73829467	81.02329874	-1.65442
Tead4	Tead4-002	1074.425	0.021169441	0.04966459	0.317740596	-3.147221386	2.521598805	7.936029685	-1.654079
Tmem167	Tmem167-003	449.87693	0.039096524	0.08063202	0.317757035	-3.147058574	1.383322945	4.353398335	-1.654004
Tmem80	Tmem80-009	1025.497	6.93573E-05	0.000613345	0.31778419	-3.14678965	3.152923531	9.921587134	-1.653881
Zfp207	Zfp207-002	8596.9727	0.000148106	0.001070173	0.31780346	-3.146598849	25.8781471	81.42814787	-1.653793
Asnsd1	Asnsd1-001	2077.3951	0.000959025	0.004404209	0.317835456	-3.146282085	6.955010626	21.88242533	-1.653648
Gm38220	Gm38220-001	506.9439	0.03670719	0.076772182	0.31790486	-3.145595199	1.588034305	4.995313085	-1.653333
Phf3	Phf3-003	9007.9891	9.50124E-06	0.000149548	0.317924607	-3.145399818	26.27925142	82.65875263	-1.653243
Pdss1	Pdss1-003	1254.158	0.004922072	0.015804816	0.317986899	-3.144783646	3.048698949	9.587498595	-1.652961
730003C15F	0003C15Rik-	114.10066	0.046961475	0.093339747	0.318086218	-3.143801726	0.323615302	1.017382344	-1.65251
730003C15F	0003C15Rik-	119.04844	0.044322797	0.089145329	0.318086708	-3.143796876	0.337644924	1.061487059	-1.652508
Taf1a	Taf1a-002	1247.144	0.000859131	0.004050832	0.318088982	-3.143774405	3.405481187	10.70606459	-1.652498
Tbck	Tbck-001	6356.8957	9.05195E-06	0.000144536	0.31813991	-3.143271149	16.21520314	50.9687802	-1.652267
Cdkn1b	Cdkn1b-001	7158.4897	3.07889E-05	0.000341158	0.318219547	-3.142484523	20.45702725	64.28589151	-1.651906
Frs2	Frs2-201	9285	9.53389E-07	3.40141E-05	0.318277162	-3.141915665	22.62620225	71.08961927	-1.651644
Atg9a	Atg9a-005	1056.3725	0.010805154	0.029268944	0.318287656	-3.14181207	3.453873703	10.85142209	-1.651597
Uhrf1bp1l	Uhrf1bp1l-201	45642	1.55112E-05	0.000210456	0.318297812	-3.141711823	122.7343484	385.5959534	-1.651551
Siae	Siae-001	2648	0.001395267	0.005916539	0.318314612	-3.141546009	6.523128056	20.49270691	-1.651475
Rab6b	Rab6b-001	13623.091	0.003100844	0.011020434	0.318368178	-3.141017442	37.3870781	117.4334644	-1.651232
Rbm25	Rbm25-002	9824.5652	0.000202579	0.001346462	0.318373139	-3.140968494	27.09563696	85.106542	-1.651209
Nphs1	Nphs1-001	318	0.003949144	0.013290577	0.318417731	-3.140528628	0.900949099	2.829456437	-1.651007
Fahd1	Fahd1-201	511	0.003750065	0.0127661	0.318423894	-3.140467847	1.594453477	5.007329877	-1.650979
Nars	Nars-201	11818	7.30753E-06	0.000125079	0.318456852	-3.140142832	36.87339929	115.7877456	-1.65083
Kdm2a	Kdm2a-201	20322.232	4.28069E-07	2.11533E-05	0.318543038	-3.139293222	57.25997314	179.7558456	-1.65044
Vps4b	Vps4b-006	816.12272	0.005470083	0.017193772	0.318591022	-3.138820407	2.135505395	6.702967914	-1.650222
Eif5b	Eif5b-001	29004.749	7.37428E-08	8.01806E-06	0.318798171	-3.136780855	86.2830409	270.6509908	-1.649285
Hoxa2	Hoxa2-001	1966.8395	0.017136142	0.042024406	0.318811242	-3.136652254	4.366411533	13.69591401	-1.649226
Wdr66	Wdr66-006	142	0.026320175	0.05882323	0.318855045	-3.136221352	0.396176082	1.242495889	-1.649027
Fbxl4	Fbxl4-001	727.06614	0.022784482	0.052553608	0.318872628	-3.136048421	2.104241455	6.599003093	-1.648948
Cct4	Cct4-001	3450.1081	0.000882715	0.004139251	0.318933337	-3.135451469	11.10591377	34.82205364	-1.648673
Lrrc49	Lrrc49-002	1091.7016	0.029166921	0.06383176	0.318955515	-3.135233448	3.328764471	10.43645371	-1.648573
Glg1	Glg1-001	39777.031	3.1216E-06	7.25338E-05	0.318993432	-3.134860788	105.9967334	332.2850031	-1.648401
Vrk1	Vrk1-202	1923.8787	0.000367836	0.002106049	0.319000184	-3.134794428	5.803754583	18.19357752	-1.648371
Dtnbos	Dtnbos-001	721	0.00158328	0.006519277	0.319013197	-3.134666559	2.09296869	6.56075896	-1.648312
Kdm5b	Kdm5b-001	3676.1798	0.002921347	0.010539991	0.31921192	-3.132715091	11.35747267	35.57972603	-1.647414
Nup93	Nup93-202	977.58192	0.011428716	0.03063819	0.319272026	-3.132125331	3.510511323	10.99536144	-1.647142
Ppp4r2	Ppp4r2-201	25261	2.59227E-07	1.60262E-05	0.319284142	-3.132006474	73.40346767	229.900136	-1.647087
Vps13d	Vps13d-001	21277.907	0.000218871	0.001427599	0.31942086	-3.130665914	50.5397514	158.223077	-1.64647
Mier3	Mier3-005	3604.3603	1.78623E-05	0.00023244	0.319433242	-3.13054457	10.91840479	34.18055284	-1.646414
Tanc2	Tanc2-001	26669.829	0.002280518	0.008685394	0.319531345	-3.129583424	53.49382175	167.4133778	-1.645971
Ormdl2	Ormdl2-201	1275.6171	0.001536678	0.006368734	0.319576332	-3.129142872	3.381130209	10.58003949	-1.645768
B3gnt3	B3gnt3-201	12629	6.86598E-06	0.000119918	0.319600289	-3.128908308	27.04642636	84.62578814	-1.645659
Nrip1	Nrip1-001	15468.783	1.30082E-06	4.12529E-05	0.319610761	-3.128805787	41.09011229	128.5629811	-1.645612
Pknox1	Pknox1-004	702.72252	0.031960886	0.068721065	0.319628193	-3.128635155	1.63836547	5.125847807	-1.645533
AI314180	AI314180-005	2008.3085	0.00195826	0.0077119	0.319692296	-3.128007818	5.299797763	16.57780884	-1.645244
Elk4	Elk4-004	915.44065	0.031049092	0.067209816	0.319735312	-3.127586981	2.217260548	6.934675225	-1.64505
Gm38253	Gm38253-001	2346.4398	2.82668E-05	0.000321769	0.319736374	-3.127576595	6.685530337	20.90950821	-1.645045
230062I16R	0062I16Rik-	291.71368	0.018040457	0.043756507	0.319742979	-3.127511986	0.81758228	2.55699838	-1.645015
Sgsm1	Sgsm1-005	827.84426	0.000496106	0.002655774	0.319750692	-3.127436551	2.260296501	7.068933894	-1.644981
Rad51c	Rad51c-002	1167.6764	0.000331205	0.001949438	0.319758364	-3.127361509	3.280658977	10.25980661	-1.644946
Gm26901	Gm26901-001	435	0.005253403	0.016650846	0.319804736	-3.126908042	1.22728237	3.837599111	-1.644737
Ube2d3	Ube2d3-016	1323.7473	0.000810521	0.003869578	0.319807008	-3.12688583	3.979309647	12.44284695	-1.644727
30401M01F	0401M01Rik-	1555.1075	0.00024068	0.001530216	0.319869008	-3.126279741	4.500282328	14.06914147	-1.644447
Pdcd10	Pdcd10-003	381.70918	0.023998867	0.054698954	0.31990838	-3.125894978	1.414253688	4.420808501	-1.644269
Npat	Npat-001	5323.569	3.30715E-06	7.503E-05	0.32002872	-3.124719552	15.66741316	48.95627225	-1.643727
Tsr1	Tsr1-001	3442.2156	0.000271021	0.001671917	0.320039082	-3.124618386	13.69356507	42.7871652	-1.64368
Tox4	Tox4-001	10418.153	1.51144E-06	4.49754E-05	0.320095377	-3.12406886	30.97285449	96.76133022	-1.643426
Mtf1	Mtf1-201	3857.3016	1.57855E-05	0.000212688	0.320130746	-3.123723707	10.89787437	34.04194852	-1.643267
Ctbs	Ctbs-003	393.32435	0.011620177	0.031034863	0.320142254	-3.123611414	1.308864008	4.088382556	-1.643215
Smchd1	Smchd1-007	1357.121	5.665E-05	0.000527675	0.320178702	-3.123255834	3.77481975	11.7897278	-1.643051
Etfdh	Etfdh-005	3914.5786	2.34129E-05	0.000281944	0.320179085	-3.123252105	10.71642698	33.47010314	-1.643049
330008E24F	0008E24Rik-	859	0.002354877	0.008895278	0.320255559	-3.122506294	2.638311414	8.238143998	-1.642704
Xaf1	Xaf1-001	1232.9516	0.000181578	0.001240486	0.320286542	-3.122204238	3.372645619	10.53008844	-1.642565
Atp5g3	Atp5g3-001	5444.7307	8.75077E-05	0.000723248	0.320287256	-3.122197282	15.56759509	48.60510308	-1.642562
RP23-7D3.3	P23-7D3.3-00	1013.434	0.000977103	0.004468659	0.320289806	-3.122172423	2.454522199	7.663441522	-1.64255
Polh	Polh-201	1698	0.003230008	0.0113838	0.320391126	-3.121185068	4.80616156	15.0009197	-1.642094
00029M09F	0029M09Rik-	662.46803	0.010581607	0.02882167	0.320534534	-3.119788642	2.13045208	6.646560204	-1.641448
Gm37607	Gm37607-001	11137	4.62967E-06	9.29166E-05	0.320569996	-3.119443532	30.58927477	95.42151532	-1.641289
Ttc39b	Ttc39b-001	4184.9872	0.000158066	0.001122413	0.320621654	-3.118940931	13.08153063	40.80052131	-1.641056
Tmpo	Tmpo-203	5136.3173	0.006294209	0.019137785	0.320627899	-3.11888018	10.4441493	32.57405025	-1.641028
Spaca6	Spaca6-011	11501.567	8.40394E-05	0.000703925	0.32068916	-3.118284387	25.60579992	79.84616612	-1.640753
Pcdhb21	Pcdhb21-001	544.56311	0.049295488	0.096960343	0.32072997	-3.117887607	1.519235591	4.736805821	-1.640569

Gm5108	Gm5108-001	794.8694	0.003740312	0.01273923	0.320737686	-3.117812601	2.25985884	7.045816368	-1.640534
Nf1	Nf1-001	7310.8873	5.25818E-06	0.000100729	0.320767428	-3.117523515	20.63708912	64.33661061	-1.6404
Pi4k2b	Pi4k2b-001	1180.4465	0.003154054	0.011179892	0.32078626	-3.117340495	4.035359323	12.57958903	-1.640316
Tmem45a	Tmem45a-001	2946.2946	0.001353877	0.005774632	0.320871088	-3.116516376	6.057068267	18.87695245	-1.639934
Ints8	Ints8-001	3309.0936	2.32447E-05	0.000280823	0.320941282	-3.115834755	11.63338709	36.24771181	-1.639619
Gm37931	Gm37931-001	767	0.005378746	0.016975861	0.321109106	-3.114206294	2.637287138	8.213056205	-1.638865
Brcc3	Brcc3-001	1757.9553	8.62018E-05	0.000715869	0.321145707	-3.113851366	5.5072966	17.14890304	-1.6387
Rprd1b	Rprd1b-003	4069.8547	0.012517486	0.032854922	0.321149366	-3.113815894	10.04667437	31.28349435	-1.638684
Ralgapb	Ralgapb-201	4834.1994	0.008795307	0.02488758	0.321174882	-3.113568516	13.35155541	41.57098256	-1.638569
Pcf11	Pcf11-001	8280.9834	4.80751E-05	0.000468042	0.321213261	-3.113196503	25.64757447	79.84593912	-1.638397
Hyou1	Hyou1-002	3104.7425	0.047317834	0.093936653	0.321225436	-3.113078505	10.29818585	32.05906101	-1.638342
Gm11410	Gm11410-001	145402	3.99529E-07	2.03932E-05	0.32132607	-3.11210354	392.7784789	1222.367295	-1.63789
Fam83a	Fam83a-001	179.70844	0.028071462	0.061928989	0.321376345	-3.111616697	0.611631616	1.903163148	-1.637664
Ndufb3	Ndufb3-001	2342	0.000296317	0.001792448	0.321383292	-3.111549431	5.98270457	18.615481	-1.637633
Inafm1	Inafm1-201	2612	0.00734293	0.021632785	0.321403383	-3.111354934	7.005049723	21.79519602	-1.637543
Gm12166	Gm12166-001	177.32267	0.029131731	0.06376791	0.321453249	-3.110872276	0.562424805	1.749631732	-1.637319
Rabgap1l	Rabgap1l-003	2685.6934	0.001091142	0.004872438	0.321460395	-3.11080312	6.796668913	21.14309886	-1.637287
Kctd3	Kctd3-003	2016.0948	9.99982E-05	0.000797352	0.321600368	-3.109449175	5.851313384	18.19436157	-1.636659
Senp1	Senp1-003	839.83989	0.046588572	0.092738497	0.321677904	-3.108699687	2.747396986	8.540832151	-1.636311
Ankhd1	Ankhd1-014	8872.774	0.008874473	0.025066031	0.321790896	-3.107608118	22.68168696	70.48579454	-1.635805
Cyc1	Cyc1-201	4059	4.54481E-05	0.000450014	0.321833145	-3.107200159	11.63400157	36.14917154	-1.635615
Cir1	Cir1-002	364.57275	0.009784356	0.027090881	0.321870041	-3.106843978	0.521088103	1.618939434	-1.63545
P24-155C13	24-155C13.7-	493.32508	0.001321906	0.005666495	0.321902917	-3.106526684	1.338958567	4.159510517	-1.635302
Dpp10	Dpp10-001	9378	9.33038E-06	0.000147333	0.321959694	-3.105978848	25.83902014	80.25545003	-1.635048
Fam58b	Fam58b-001	862	0.002119475	0.008186762	0.321987984	-3.105705956	2.69766992	8.378169537	-1.634921
Pdlim1	Pdlim1-001	9331.8215	0.000611447	0.003122883	0.321994439	-3.105643695	26.48023957	82.23818906	-1.634892
Snhg14	Snhg14-011	2050	0.000144297	0.001050409	0.321995983	-3.105628806	5.258799682	16.33187978	-1.634885
Pdpk1	Pdpk1-001	7332.6182	0.000116281	0.000893766	0.322055396	-3.105055876	19.72790334	61.25624218	-1.634619
Mboat7	Mboat7-003	988.9031	0.001466404	0.006148208	0.322108443	-3.104544513	3.231137439	10.03121001	-1.634382
Endov	Endov-003	2551.4703	0.001292544	0.005574313	0.322113215	-3.104498518	7.041219881	21.85945669	-1.63436
Sulf1	Sulf1-004	30679.437	0.000116486	0.000894944	0.322127041	-3.104365277	69.66789148	216.2745832	-1.634298
Dynlt1b	Dynlt1b-201	573	0.002882632	0.010428875	0.322305317	-3.10264816	1.895322569	5.880519081	-1.6335
Tmed8	Tmed8-201	5732	4.86202E-07	2.30963E-05	0.322318638	-3.102519937	16.67097293	51.72202589	-1.63344
Cdc42	Cdc42-002	1506.7799	0.001531487	0.006351657	0.322354386	-3.102175876	3.947209718	12.24493877	-1.63328
Emc7	Emc7-001	9369	1.63304E-06	4.70132E-05	0.322357564	-3.102145289	26.3738862	81.81562684	-1.633266
Yipf5	Yipf5-201	4397	3.53276E-05	0.000375068	0.322392552	-3.101808627	13.44088028	41.6910384	-1.63311
Mtrf1	Mtrf1-201	728	0.007266366	0.021456433	0.322436824	-3.101382736	2.344121115	7.270016756	-1.632912
Dennd4c	Dennd4c-001	13366.797	2.01134E-06	5.42665E-05	0.322454227	-3.101215354	34.98869674	108.5074835	-1.632834
Ap1s2	Ap1s2-003	1177.514	0.003060352	0.010918444	0.322464732	-3.101114325	3.380048514	10.48191686	-1.632787
Dtwd2	Dtwd2-201	501.50821	0.017926348	0.043551107	0.322510569	-3.100673577	1.626495835	5.043232658	-1.632582
Gm10417	Gm10417-001	374	0.007637094	0.022316395	0.322674524	-3.099098088	1.146369705	3.552712161	-1.631848
Ttll7	Ttll7-004	3852.4248	0.00011414	0.000881066	0.322728809	-3.0985768	7.719128354	23.91831203	-1.631606
P24-285A14	24-285A14.1-	334	0.01463901	0.037183506	0.322757646	-3.098299958	0.828710759	2.567594508	-1.631477
Zfp26	Zfp26-003	991.34179	0.005785785	0.017922251	0.322835233	-3.09755534	2.491377196	7.717178738	-1.63113
Cpe	Cpe-201	217673	0.01109337	0.029893601	0.322872882	-3.097194146	640.8399998	1984.805895	-1.630962
Cdr2	Cdr2-002	2347.8032	0.02280848	0.052601874	0.322876366	-3.097160729	8.254278225	25.56482637	-1.630946
Vopp1	Vopp1-002	4345.9452	0.000132037	0.000984174	0.322891503	-3.097015534	10.53436533	32.62509305	-1.630879
Trpc4ap	Trpc4ap-005	1738.0226	7.51806E-05	0.000649908	0.322919583	-3.096746224	4.709005448	14.58259484	-1.630753
Ppap2c	Ppap2c-003	1807.5602	0.01144103	0.030666402	0.322976042	-3.096204889	6.356606001	19.68135458	-1.630501
Spata1	Spata1-002	878.86121	0.000241098	0.001532308	0.322991226	-3.096059334	2.377251372	7.360111302	-1.630433
30057M04R	0057M04Rik-	234.30474	0.047576399	0.094342964	0.322999604	-3.095979028	0.836907419	2.591047817	-1.630396
30005N03R	0005N03Rik-	1181.1842	0.028414851	0.062525059	0.322999856	-3.095976616	3.651389049	11.30461511	-1.630395
Zzef1	Zzef1-005	2048.6847	0.000213532	0.001400245	0.323023871	-3.095746449	5.874455901	18.18582599	-1.630287
Efcab2	Efcab2-001	1337.5723	0.002147116	0.00827021	0.323025148	-3.095734201	3.491882127	10.80993893	-1.630282
Dcaf17	Dcaf17-001	4306.1282	2.4805E-05	0.000293766	0.32303859	-3.095605387	12.16496172	37.65792103	-1.630222
Sgk1	Sgk1-009	649.82696	0.021851896	0.050901485	0.323046673	-3.095527936	1.933743484	5.985956974	-1.630185
Pcmt1	Pcmt1-005	548.818	0.021305004	0.049907421	0.323072298	-3.095282409	1.502466135	4.650556997	-1.630071
Mtch2	Mtch2-010	1293.3649	0.000432121	0.002389434	0.323093642	-3.095077931	3.277191264	10.14316236	-1.629976
BC051077	BC051077-00	1894	5.7258E-06	0.000106453	0.323116256	-3.094861316	5.409094342	16.74039683	-1.629875
Ghitm	Ghitm-202	10634.058	2.31037E-05	0.000279415	0.323146045	-3.094576017	28.98179903	89.6863802	-1.629742
Fam122b	Fam122b-002	644.88215	0.020115664	0.047718377	0.323189386	-3.094161015	1.49175506	4.615730351	-1.629548
Phf21a	Phf21a-016	232.29119	0.039483862	0.081249835	0.323254422	-3.093538502	0.516456366	1.597677653	-1.629258
Noc3l	Noc3l-201	3297	0.000802236	0.003838026	0.323274695	-3.093344499	10.89462726	33.7008353	-1.629168
Kpna1	Kpna1-002	675.35224	0.002360487	0.00891048	0.323306089	-3.093044123	2.100161855	6.495893282	-1.629027
Irf2bp2	Irf2bp2-001	28680.356	5.94224E-05	0.000546951	0.323357548	-3.092551902	75.41254122	233.2171978	-1.628798
Ngly1	Ngly1-001	4653.7722	0.000314772	0.001873314	0.323369284	-3.09243966	11.67844343	36.11488163	-1.628745
Cul3	Cul3-001	5489.8208	8.38296E-06	0.000137376	0.323392167	-3.092220843	17.24068234	53.31199729	-1.628643
Wdr82	Wdr82-003	801.54401	0.001674432	0.006802194	0.323399221	-3.092153397	2.362651303	7.305680252	-1.628612
Mien1	Mien1-001	1470.1236	0.000600172	0.003076307	0.323399556	-3.09215019	4.581152826	14.16561258	-1.62861
Crot	Crot-002	7927.6595	8.62661E-06	0.000140029	0.323409261	-3.092057404	21.80278016	67.41544784	-1.628567
Kmt2e	Kmt2e-006	10407.21	1.05061E-05	0.000159866	0.323438892	-3.091774135	29.07672002	89.8986509	-1.628435
Rpl30	Rpl30-002	7174.073	3.53957E-05	0.000375606	0.323449933	-3.0916686	23.27702942	71.96486094	-1.628386
Fam177a	Fam177a-201	11795	5.21003E-05	0.00049609	0.32350032	-3.091187054	31.00245224	95.834379	-1.628161
RP24-488I7.	P24-488I7.1-0	1740	0.043760921	0.088217278	0.323530991	-3.090893999	4.545580368	14.04990708	-1.628024
Knop1	Knop1-202	4119.7773	0.002006509	0.007851335	0.323561051	-3.090606846	12.64649203	39.08533486	-1.62789
Psma6	Psma6-001	4346.6352	0.000471442	0.002555694	0.323579911	-3.090426711	13.45810322	41.59128168	-1.627806
Aoc2	Aoc2-001	650.99658	0.001276615	0.005520017	0.323586954	-3.090359447	2.027000067	6.264158805	-1.627775
Prmt3	Prmt3-001	1608.7384	0.00459475	0.014973377	0.323590416	-3.090326382	5.937219317	18.34794549	-1.627759
Kat6b	Kat6b-009	1453.9276	0.001166129	0.005141018	0.323600662	-3.090228537	3.562630839	11.00934349	-1.627714
Slc35e2	Slc35e2-001	5058.394	5.24301E-06	0.000100601	0.323603233	-3.090203983	14.23952033	44.00302242	-1.627702
Ccdc39	Ccdc39-001	1190.589	0.01556023	0.038965991	0.323650161	-3.089755915	2.003475122	6.190249109	-1.627493
Ddc	Ddc-008	128	0.038427524	0.07957813	0.323784684	-3.088472216	0.343615046	1.061245523	-1.626893
Phkg2	Phkg2-003	917.7044	0.003989183	0.013391112	0.323826153	-3.088076702	2.602390043	8.03638006	-1.626709
Gm15975	Gm15975-001	841	0.00020256	0.001346462	0.323852258	-3.087827781	2.515791084	7.768329601	-1.626592
Spopl	Spopl-001	5542.055	1.22932E-05	0.000178483	0.323852799	-3.087822623	15.64549349	48.31050876	-1.62659
Nudt16	Nudt16-001	590.7411	0.008258958	0.023680979	0.323878245	-3.087580021	1.659316765	5.123273293	-1.626477
Pcmtd2	Pcmtd2-007	556.14064	0.030901321	0.066943089	0.323891933	-3.087449542	1.201264633	3.70884394	-1.626416
Hnrnpa1l2-p	rnpa1l2-ps-0	433	0.004643187	0.015095317	0.323934406	-3.087044728	1.246433689	3.847796548	-1.626226
Wdr45	Wdr45-002	1545.2561	0.008651959	0.024567204	0.32395979	-3.086802839	4.072787247	12.57189124	-1.626113
Ercc4	Ercc4-004	1547.7846	0.001019478	0.004622336	0.323976545	-3.086643197	4.731053042	14.60307269	-1.626039
Specc1	Specc1-002	1469.3855	0.002481605	0.009268809	0.323988999	-3.086524554	3.08012778	9.50689002	-1.625983
Kifap3	Kifap3-002	3854.7327	0.013837417	0.035561757	0.324013432	-3.086291805	10.28224725	31.73401544	-1.625874
Tmem181b-p	mem181b-ps-0	768.17592	0.002720104	0.009956728	0.324116818	-3.085307351	2.089024205	6.445281736	-1.625414
Gtdc1	Gtdc1-202	554.85052	0.001176816	0.005178717	0.324158393	-3.084911643	1.494668819	4.610921242	-1.625229
810037I17R	0037I17Rik-0	3256	4.1242E-05	0.000419151	0.324179397	-3.084711762	9.783927641	30.18059668	-1.625136
Nudcd3	Nudcd3-002	1177.2987	0.00740883	0.021794419	0.32421621	-3.084361515	3.46503657	10.68742544	-1.624972
Mapk3	Mapk3-002	6772.237	0.00436006	0.014369907	0.324223298	-3.084294087	17.41053036	53.69919582	-1.62494
Gm10143	Gm10143-201	742.15053	0.004691234	0.015211092	0.324239663	-3.084138417	2.447433722	7.548224366	-1.624868
Gm6645	Gm6645-001	241	0.008206864	0.023565148	0.324247187	-3.084066845	0.712204055	2.196484915	-1.624834
Trub1	Trub1-201	1512.3805	0.001083431	0.004844957	0.324278711	-3.08376704	4.85710295	14.97817398	-1.624694
Gm18294	Gm18294-001	2510	5.98297E-05	0.000549965	0.324313395	-3.083437245	6.455109238	19.90392424	-1.624539
30601H04R	0601H04Rik-	817.57696	0.02031392	0.048049156	0.324327586	-3.083302323	2.453575782	7.565115906	-1.624476
Pigt	Pigt-002	5923.8574	0.00066134	0.003314927	0.324328597	-3.083292713	10.6669578	32.88935325	-1.624472
Gm38310	Gm38310-001	1327	0.00026139	0.001626336	0.324346638	-3.08312121	3.949426256	12.17655986	-1.624392
Rab33b	Rab33b-001	6139.479	3.71801E-05	0.000389067	0.324379972	-3.08280438	16.99364866	52.38809452	-1.624243
Dpf2	Dpf2-009	2543.2494	0.003883521	0.013117417	0.324406004	-3.082557002	8.389099311	25.85987682	-1.624128
Tsen2	Tsen2-002	389.82437	0.008479982	0.024185142	0.324430627	-3.082323055	1.109582468	3.420091623	-1.624018

Tead2	Tead2-003	847.07207	0.026369477	0.058918038	0.324472651	-3.081923846	2.658252927	8.192533085	-1.623831
Chaf1b	Chaf1b-002	480.64891	0.006178493	0.018880566	0.324488925	-3.081769274	1.462148867	4.506005453	-1.623759
Gm10548	Gm10548-001	530.26984	0.00115095	0.005090396	0.324489865	-3.081760351	1.427613139	4.399561567	-1.623755
Gm14853	Gm14853-001	325	0.022087489	0.05131225	0.324527616	-3.081401863	1.025074746	3.158667231	-1.623587
Gm2004	Gm2004-001	632.29567	0.015005083	0.037880451	0.324550631	-3.081183347	1.961779308	6.044601736	-1.623485
Atrx	Atrx-014	12068.932	1.69474E-05	0.000224372	0.324593645	-3.080775045	36.07062741	111.1254888	-1.623293
Rin2	Rin2-201	1270.8869	0.004070327	0.01359978	0.324595506	-3.080757381	3.998824894	12.31940931	-1.623285
Mbnl1	Mbnl1-009	2333.9226	0.000729991	0.003570982	0.32459646	-3.080748327	5.738284726	17.67821107	-1.623281
Ufd1l	Ufd1l-201	6414.3323	6.68649E-06	0.000117987	0.324614788	-3.080574378	17.91518599	55.18906292	-1.623199
Ccdc50	Ccdc50-001	21588.049	2.06978E-07	1.45864E-05	0.324629225	-3.080437378	58.71709599	180.8743372	-1.623135
Xpnpep3	Xpnpep3-001	1487.2984	0.00695491	0.020722547	0.324673808	-3.080014391	3.97823522	12.25302173	-1.622937
Ppp1r2-ps4	pp1r2-ps4-00	685	0.007682607	0.022411147	0.324724167	-3.079536735	1.806504034	5.563195537	-1.622713
Tax1bp3	Tax1bp3-002	2188.2499	8.51869E-05	0.0007101	0.324770242	-3.07909984	6.435165792	19.81451796	-1.622509
Pak1ip1	Pak1ip1-004	561.25519	0.022224599	0.051570179	0.32482187	-3.078610442	1.542997244	4.750287427	-1.622279
Gm17056	Gm17056-001	335	0.033580829	0.071535228	0.324966658	-3.077238769	1.067643398	3.285393654	-1.621636
Anapc16	Anapc16-003	2476.963	3.8846E-06	8.25869E-05	0.325046484	-3.076483053	6.676782159	20.54100716	-1.621282
Ralgapa2	Ralgapa2-001	18193.137	8.41124E-06	0.000137774	0.325071597	-3.07624538	52.12222032	160.3407395	-1.621171
Plcg1	Plcg1-012	378.66009	0.040246015	0.082525655	0.32510187	-3.075958931	0.902223364	2.775202016	-1.621036
Tor2a	Tor2a-009	331.23758	0.034738845	0.073499223	0.325102023	-3.075957479	0.93493715	2.87582692	-1.621036
42622	Sept9-010	717.69092	0.011199413	0.030108312	0.325102537	-3.075952618	0.971489161	2.988254629	-1.621033
Gtf2a2	Gtf2a2-006	822.85492	0.0062444	0.019041196	0.325147135	-3.075530709	2.143706807	6.593036115	-1.620835
P24-473H2(	24-473H20.4-	165	0.049366736	0.097050322	0.32539968	-3.073143768	0.492477792	1.513455057	-1.619715
Rps13-ps4	Rps13-ps4-00	1617	0.000538142	0.002829502	0.325419097	-3.072960404	5.438509585	16.71232461	-1.619629
Plekhb1	Plekhb1-006	588.81219	0.049095854	0.096684281	0.325419655	-3.07295514	1.601250313	4.92057038	-1.619627
Cgnl1	Cgnl1-003	20546.256	0.004471671	0.014660163	0.325432625	-3.072832666	34.75292306	106.7899172	-1.619569
Fgd6	Fgd6-001	5078.9106	5.26058E-06	0.000100729	0.325472618	-3.072455084	15.1348065	46.50101317	-1.619392
Gm37655	Gm37655-001	746	0.002732127	0.009996298	0.325475601	-3.072426923	1.568853529	4.82018782	-1.619379
Dnajc13	Dnajc13-003	498.90057	0.006330434	0.019212882	0.325484767	-3.072340401	1.899321129	5.835361039	-1.619338
Papd5	Papd5-002	3500.4429	5.07311E-05	0.000485754	0.325486579	-3.072323299	10.56736033	32.46634734	-1.61933
Papd5	Papd5-001	2801.51	4.96136E-05	0.000478194	0.325493487	-3.072258097	8.242180652	25.32210624	-1.619299
Large	Large-201	2283.8672	0.001260916	0.005465247	0.325608161	-3.071176097	7.103392489	21.81576922	-1.618791
Psma2	Psma2-201	6648	1.97847E-05	0.000250338	0.325697328	-3.070335289	20.56802233	63.15072479	-1.618396
Abhd10	Abhd10-001	960.68681	0.000631462	0.003200764	0.325715931	-3.07015993	2.48481161	7.628769037	-1.618314
Ppat	Ppat-002	543.03609	0.034170239	0.072551651	0.325750263	-3.069836352	1.801654376	5.530784097	-1.618162
Nipa1	Nipa1-001	1488.5901	0.000127714	0.000959046	0.325751481	-3.069824877	4.273487119	13.11885707	-1.618156
Cct4	Cct4-004	4666.3033	0.000212146	0.001393021	0.32582329	-3.069148312	15.40937017	47.29364244	-1.617838
Wdr1	Wdr1-004	1945.1784	0.000229579	0.001478855	0.325823823	-3.069143288	5.741179387	17.62050218	-1.617836
Sema6d	Sema6d-203	3340.131	0.001760765	0.007085677	0.325829372	-3.069091023	10.89493841	33.43755766	-1.617811
Ghr	Ghr-003	1970.9761	0.022863242	0.052692689	0.325841848	-3.06897351	4.593489542	14.09729772	-1.617756
Mtf2	Mtf2-001	649.62643	0.005487344	0.017232856	0.325915925	-3.068275964	1.925880872	5.909133989	-1.617428
Tulp4	Tulp4-002	17371.324	0.000280147	0.001714929	0.325919315	-3.068244049	35.14003602	107.8182064	-1.617413
Derl2	Derl2-001	2328.1992	0.000127635	0.000958875	0.325928938	-3.068153466	5.386306377	16.52601458	-1.617371
Ppfibp1	Ppfibp1-004	1868.8747	0.011082517	0.029873777	0.325962114	-3.067841188	6.194068422	19.00241823	-1.617224
730455P16R	0455P16Rik-	1485.7318	0.016271218	0.04033651	0.32597565	-3.067713798	4.161455986	12.76615595	-1.617164
Pcgf2	Pcgf2-003	324.93669	0.018663919	0.044994666	0.32597958	-3.067676818	0.862067951	2.644545868	-1.617147
Man1a	Man1a-001	24753.893	6.87265E-05	0.000610171	0.326031232	-3.067190814	60.94995218	186.9451335	-1.616918
Clasp1	Clasp1-006	592.87384	0.006081872	0.018663571	0.326065702	-3.066866567	1.793977697	5.501890221	-1.616765
Gm8394	Gm8394-201	1079	0.004726286	0.015300123	0.326088131	-3.066655625	3.436303805	10.53796039	-1.616666
Gm5316	Gm5316-002	491	0.001269466	0.005492575	0.326102105	-3.066524213	1.520259274	4.661911873	-1.616604
Kif5b	Kif5b-001	33319.916	6.05554E-06	0.00011032	0.326139216	-3.066175271	87.42997223	268.0756188	-1.61644
Tma7	Tma7-001	2445.3187	0.000113547	0.000877638	0.326152459	-3.066050777	7.01694539	21.51431086	-1.616382
Itpripl2	Itpripl2-201	35900	7.74074E-06	0.000130152	0.326179937	-3.065792486	122.301704	374.9516452	-1.61626
Tpr	Tpr-002	46055.86	3.71271E-06	8.05433E-05	0.326204504	-3.065561597	136.8278995	419.4543539	-1.616151
Ptges3	Ptges3-001	11994.446	0.000346715	0.002016467	0.326348011	-3.064213555	40.41259938	123.8328348	-1.615517
Prdx6	Prdx6-009	599.49261	0.001695125	0.00686265	0.326374276	-3.063966968	1.95125373	5.978576974	-1.615401
Emc10	Emc10-001	1388.8233	0.002733031	0.009998513	0.32641018	-3.063629943	3.525457528	10.80069724	-1.615242
Emc10	Emc10-201	1023.7235	0.003304889	0.011586902	0.326412218	-3.063610816	2.598662983	7.961292021	-1.615233
Phlpp2	Phlpp2-001	837.09257	0.0026789	0.00983412	0.326419223	-3.063545068	2.008986556	6.154620855	-1.615202
Tbl3	Tbl3-003	297.99342	0.008140996	0.023420159	0.326436603	-3.063381954	0.931167416	2.85252146	-1.615125
Cbfa2t2	Cbfa2t2-003	952.88738	0.008509494	0.024247102	0.326445467	-3.06329878	3.119384789	9.555607619	-1.615086
Thsd4	Thsd4-002	381.74366	0.011198467	0.030108133	0.326480456	-3.062970483	0.984791739	3.016388029	-1.614931
10006K23R	0006K23Rik-	1395.404	0.000814566	0.003883819	0.326505521	-3.062735346	3.587128395	10.98642493	-1.614821
Setd2	Setd2-005	1056.0523	0.000948187	0.00437026	0.326568047	-3.062148944	2.602766975	7.970060144	-1.614544
Phf3	Phf3-011	1384.4977	0.004658009	0.015127708	0.32657128	-3.062118632	4.075815389	12.48063024	-1.61453
Gm14326	Gm14326-002	788.02746	0.010714269	0.029095294	0.326576356	-3.062071034	2.228902736	6.825058506	-1.614508
Prr14l	Prr14l-001	12396.987	1.10215E-05	0.000165274	0.326609954	-3.061756045	35.10588158	107.4856452	-1.614359
Hnrnpu	Hnrnpu-001	26426.045	6.92491E-06	0.00012064	0.326626199	-3.061603764	79.65731104	243.8791233	-1.614288
Gm37660	Gm37660-001	676	0.027989781	0.061796557	0.32665872	-3.061298961	2.142602938	6.559148148	-1.614144
Canx	Canx-001	50629.186	3.16359E-05	0.000347651	0.326676286	-3.061134348	147.5101953	451.5485255	-1.614066
Chkb	Chkb-004	1184.5894	0.030712145	0.066604885	0.326683224	-3.061069337	3.456976885	10.58204594	-1.614036
Gm5931	Gm5931-001	1280	0.000537269	0.002826212	0.326815152	-3.059833649	4.172605293	12.76747808	-1.613453
Dzip3	Dzip3-001	929.93526	0.014731389	0.037368543	0.326868476	-3.059334485	2.733274079	8.361999648	-1.613218
Atxn2	Atxn2-010	640.46618	0.007101229	0.021074178	0.326914797	-3.058901005	1.724376015	5.274695526	-1.613013
B4galt1	B4galt1-001	41541.191	4.83971E-06	9.57309E-05	0.32692011	-3.058851287	102.573225	313.7562413	-1.61299
Inpp5k	Inpp5k-004	1064.2852	0.004588684	0.014964999	0.32707426	-3.057409658	2.395123496	7.32287371	-1.61231
Grpel2	Grpel2-201	2082	0.000306365	0.001837651	0.327149721	-3.056704429	6.054593739	18.5071035	-1.611977
Ifih1	Ifih1-001	4519.889	0.002776649	0.010109517	0.327156653	-3.056639662	12.7170711	38.8715039	-1.611946
Usp36	Usp36-004	1405.0396	0.038453512	0.079617507	0.327242103	-3.055841503	4.66430002	14.25336158	-1.61157
Glis3	Glis3-001	3227.972	0.000273481	0.001682543	0.327259359	-3.055680377	14.84930082	45.37471714	-1.611494
Nhp2l1	Nhp2l1-001	4409.5506	4.82543E-05	0.000469105	0.327280646	-3.05548162	13.83782128	42.28120859	-1.6114
230219D22F	0219D22Rik-	18144	4.93176E-08	6.25603E-06	0.327286381	-3.055428084	47.9772979	146.5911834	-1.611375
Hook3	Hook3-001	50657.162	1.53325E-07	1.21842E-05	0.327308179	-3.055224598	137.0557697	418.7361588	-1.611278
Asap2	Asap2-002	20653.738	0.002777946	0.010113165	0.327388472	-3.054475299	54.83693069	167.4980503	-1.610925
Lrrc16a	Lrrc16a-002	674.81263	0.031349311	0.06768242	0.327394517	-3.054418898	1.158756899	3.539328972	-1.610898
Zfp217	Zfp217-001	1347.6427	0.030414562	0.066072482	0.327467444	-3.053738684	4.850375761	14.81178009	-1.610577
Atg10	Atg10-201	747	0.010425631	0.028518318	0.327497508	-3.05345835	2.508251147	7.65884041	-1.610444
Fam120b	Fam120b-201	7476	2.67583E-05	0.000309416	0.32752329	-3.053217987	20.45845985	62.4641376	-1.610331
Gm10699	Gm10699-201	1178	0.000556912	0.002907657	0.32754235	-3.053040321	3.475539735	10.61096295	-1.610247
P23-314N3	23-314N3.1-(	5700	2.30502E-05	0.000279161	0.327624144	-3.052278098	15.85169655	48.38378618	-1.609886
Ssr1	Ssr1-201	27875	3.27122E-06	7.45418E-05	0.327626926	-3.05225218	83.28643016	254.211188	-1.609874
Insig2	Insig2-002	5839.007	0.000881536	0.004135409	0.327703727	-3.051536855	16.11262451	49.16826753	-1.609536
Ralgapa2	Ralgapa2-003	1319.8818	0.000474454	0.002569178	0.327754702	-3.051062257	4.046878338	12.34727776	-1.609312
Usp6nl	Usp6nl-001	6424.869	1.19867E-06	3.92117E-05	0.3277655	-3.050961737	19.8684375	60.61784258	-1.609264
Adh5	Adh5-001	4736.6902	5.1621E-06	9.96068E-05	0.327815422	-3.050497117	13.76915552	42.00276922	-1.609044
Srgap2	Srgap2-006	999.94581	0.002010193	0.007857817	0.327837646	-3.050290325	3.017985788	9.205732849	-1.608947
Cntnap2	Cntnap2-002	440.87692	0.001191943	0.005229828	0.327882471	-3.049873316	1.265829184	3.86061865	-1.608749
Ndufa9	Ndufa9-001	4418.727	0.000133403	0.000991764	0.327896393	-3.049743824	12.55306757	38.28364029	-1.608688
Wdr41	Wdr41-201	1236.3602	0.001082426	0.004842989	0.328009101	-3.048695896	4.581334545	13.96709582	-1.608192
Gm12630	Gm12630-001	254	0.049399089	0.097097206	0.328013937	-3.048650946	0.718339771	2.189967222	-1.608171
Ikbkg	Ikbkg-203	390.25301	0.015829825	0.039493846	0.328019341	-3.048600721	0.943086669	2.8750947	-1.608147
Gli3	Gli3-001	1996.355	0.00261049	0.009641895	0.328086905	-3.047972916	6.741827564	20.54890782	-1.60785
Gm37485	Gm37485-001	1747	0.000336625	0.001973537	0.328098682	-3.047863508	4.803540532	14.6405359	-1.607798
E2f5	E2f5-001	1791.7457	0.015745899	0.039318827	0.328121146	-3.047654841	4.829099857	14.71742956	-1.6077
Cnnm4	Cnnm4-001	1033.3927	0.004830315	0.015566266	0.328306063	-3.045938265	3.815937655	11.62311052	-1.606887
Wls	Wls-001	3707.0678	5.50152E-05	0.00051638	0.328384673	-3.045209117	9.384072464	28.57646302	-1.606541
Vsig10	Vsig10-004	1067.6428	0.034842693	0.07365979	0.328386221	-3.045194761	3.191197506	9.717817926	-1.606535
Lias	Lias-201	1663.3985	5.36529E-05	0.000507487	0.328433593	-3.044755535	4.449565692	13.54783977	-1.606326

Gm37140	Gm37140-001	2966	0.004001868	0.013422367	0.328455895	-3.044548793	9.110596822	27.73765656	-1.606228
Uso1	Uso1-002	1110.6153	0.023733638	0.054231576	0.328461146	-3.044500127	3.571597104	10.87372784	-1.606205
Teddm1b	Teddm1b-001	389	0.029379058	0.064197573	0.328531343	-3.043849613	1.209882406	3.682700094	-1.605897
Ripk1	Ripk1-002	3311.6373	0.002925051	0.010546688	0.328597058	-3.043240882	10.07665216	30.66567981	-1.605609
Eif3j2	Eif3j2-001	5015	4.67244E-05	0.000458673	0.32861569	-3.043068333	16.10145535	48.9978289	-1.605527
Adamts10	Adamts10-01(	18608.062	0.002543651	0.00944493	0.328634695	-3.042892352	36.06511471	109.7422617	-1.605443
Gm26510	Gm26510-002	1395.289	0.000182443	0.001245249	0.328686912	-3.042408942	4.24892129	12.92695613	-1.605214
Commd2	Commd2-001	785.46933	0.004955069	0.015890552	0.328713286	-3.042164835	2.266784477	6.895932025	-1.605098
Rlim	Rlim-002	13418.804	4.94738E-06	9.70491E-05	0.328729318	-3.04201647	41.1306348	125.1200685	-1.605028
Synpo	Synpo-003	1507.6665	0.00676667	0.020262148	0.328797133	-3.041389051	4.625755006	14.06872063	-1.60473
Anxa3	Anxa3-006	1680.0485	0.022783249	0.052553608	0.328839554	-3.040996704	4.120146177	12.52935094	-1.604544
Gm9761	Gm9761-001	1455	0.004536732	0.014830873	0.328851634	-3.040885	4.677051714	14.2223764	-1.604491
Smad4	Smad4-001	6804.1393	4.1929E-06	8.7193E-05	0.328856481	-3.040840182	18.05569338	54.90447794	-1.60447
Nemf	Nemf-201	14695	7.28748E-07	2.89219E-05	0.328958157	-3.039900303	41.85323098	127.2296495	-1.604024
Golga3	Golga3-001	7549.3264	9.27919E-06	0.000146795	0.328963751	-3.03984861	24.01441455	73.00018471	-1.603999
Tial1	Tial1-009	527.97108	0.007147139	0.021175565	0.328966663	-3.039821694	1.826092057	5.550994249	-1.603987
Klhl15	Klhl15-004	301.83912	0.019244049	0.046078092	0.328975356	-3.039741373	0.855926277	2.601794515	-1.603949
Scmh1	Scmh1-007	1370.3565	0.011492386	0.030770342	0.328985369	-3.039648856	3.252966694	9.887876491	-1.603905
Aqr	Aqr-006	516.34606	0.004934326	0.015840349	0.329048719	-3.039063652	1.551372138	4.714718673	-1.603627
Zfp207	Zfp207-003	4240.4859	0.000207358	0.001369196	0.32905421	-3.039012935	13.57103657	41.24255568	-1.603603
Hbp1	Hbp1-001	11163.378	1.41068E-06	4.3255E-05	0.32907671	-3.038805145	32.98355065	100.2305834	-1.603504
Ube4a	Ube4a-003	5674.4909	7.79454E-06	0.000130672	0.329079857	-3.038776089	15.00034202	45.58268068	-1.60349
Slc28a3	Slc28a3-003	394	0.00435972	0.014369907	0.329128025	-3.038331359	1.203435483	3.656435767	-1.603279
Tmem254b	mem254b-00	1867.0606	6.62681E-05	0.000593165	0.329145702	-3.038168182	4.978390239	15.12518682	-1.603202
Gm28370	Gm28370-001	6899	6.74061E-06	0.000118575	0.329173321	-3.037913272	19.53704015	59.35183357	-1.603081
Mars2	Mars2-001	1592	0.000609412	0.003115279	0.329187269	-3.037784552	5.262187459	15.98539177	-1.60302
Brwd3	Brwd3-001	1316.3074	0.009655432	0.026805427	0.329253503	-3.037173458	4.016349824	12.19835108	-1.602729
Eed	Eed-001	3703.4327	3.28865E-05	0.000357122	0.329314527	-3.036610648	11.35678209	34.48612544	-1.602462
Cxcl16	Cxcl16-001	3121.6671	2.91187E-05	0.000328659	0.329370387	-3.036095648	12.28108181	37.28653902	-1.602217
Arhgap18	Arhgap18-001	1080.9494	0.030314074	0.065891796	0.32943102	-3.03553685	3.8461863	11.67524025	-1.601952
Lrrtm2	Lrrtm2-201	2414	0.000234191	0.00150066	0.329542047	-3.034514137	6.973415278	21.16092724	-1.601466
Ccdc173	Ccdc173-001	229.37467	0.030842487	0.066878771	0.329638041	-3.033630455	0.877866376	2.663122173	-1.601045
Mrps25	Mrps25-001	3943.4285	1.95061E-05	0.000247621	0.329646907	-3.033548859	11.54728835	35.02926341	-1.601007
Maged2	Maged2-004	1342.4328	0.04183787	0.085132311	0.329654108	-3.033482596	3.415454955	10.36072316	-1.600975
Prpf38b	Prpf38b-006	11035.791	6.95607E-06	0.000120936	0.329682429	-3.033222009	32.94310539	99.92375231	-1.600851
Gmeb1	Gmeb1-201	4850.7933	7.58069E-05	0.000654496	0.329696453	-3.033092992	13.86964997	42.06793812	-1.60079
Reep3	Reep3-201	21929	2.21027E-07	1.49904E-05	0.329747341	-3.032624913	54.40147708	164.9792747	-1.600567
Fam126a	Fam126a-001	5454.7159	7.70935E-06	0.000129694	0.329770015	-3.032416391	16.88314754	51.19673334	-1.600468
Rb1	Rb1-001	10929.862	6.72055E-07	2.75005E-05	0.32983544	-3.031814898	31.14025084	94.41147642	-1.600182
Emcn	Emcn-003	10061.794	0.001825489	0.007286214	0.329926988	-3.030973625	37.96140056	115.0600039	-1.599781
930430F08Rik	0430F08Rik-	634.73258	0.001503754	0.006270654	0.32999673	-3.030333055	1.883813036	5.708580913	-1.599476
Myg1	Myg1-007	267.6516	0.018279835	0.044221242	0.330020014	-3.03011926	0.870373116	2.637334341	-1.599375
632411P08Rik	2411P08Rik-	272	0.018983441	0.045594869	0.33003261	-3.030003613	0.905333458	2.743163649	-1.59932
Gm38056	Gm38056-001	997.75223	0.005762976	0.017870069	0.330059032	-3.02976105	3.400352137	10.30225446	-1.599204
Gtpbp4	Gtpbp4-201	8129	1.61994E-05	0.000216392	0.330092085	-3.029457671	28.45563839	86.20515202	-1.59906
333407K13Rik	3407K13Rik-	403.52627	0.040511788	0.0829564	0.330095636	-3.029425089	0.963405564	2.918564987	-1.599044
Zfp931	Zfp931-002	1007.4055	0.000349819	0.002030727	0.330095774	-3.029423817	2.869332969	8.692425634	-1.599043
Jmjd1c	Jmjd1c-001	8657.0719	0.004120727	0.013734422	0.330104086	-3.029347533	25.78191328	78.1023754	-1.599007
Lamc1	Lamc1-003	1185.4439	0.02346908	0.053781345	0.330114607	-3.029250991	3.175169167	9.618384344	-1.598961
Dbr1	Dbr1-001	1271.9988	0.00214752	0.008270836	0.330123379	-3.029170501	3.860350648	11.69366031	-1.598923
Arhgap29	Arhgap29-00(	22145.449	2.84767E-06	6.83003E-05	0.330146175	-3.028961338	64.78208407	196.222428	-1.598823
Spats2l	Spats2l-001	2167.532	0.014903204	0.037706637	0.330171769	-3.028726541	4.630886771	14.02568967	-1.598711
Zfp397	Zfp397-201	7170	6.50271E-06	0.000115754	0.330209718	-3.028378469	19.37790118	58.68361869	-1.598546
Gm20594	Gm20594-201	623661	2.75679E-06	6.66337E-05	0.330211072	-3.02836605	1807.797803	5474.673492	-1.59854
Tmtc3	Tmtc3-003	520.06108	0.003314458	0.011612129	0.330239933	-3.028101387	1.3798546	4.178339627	-1.598414
Gm15781	Gm15781-001	184	0.036665912	0.076699901	0.330280831	-3.02772643	0.474754645	1.437427186	-1.598235
Stx18	Stx18-004	337.03989	0.022380195	0.051844551	0.330293785	-3.027607679	0.528203244	1.599192196	-1.598178
Phb2	Phb2-002	913.38075	0.009972692	0.027521128	0.330305334	-3.027501824	2.943474743	8.911375151	-1.598128
Nmt2	Nmt2-001	18150.212	0.000112133	0.0008691	0.330342688	-3.027159477	50.46374066	152.7617908	-1.597965
Chn2	Chn2-003	964.35367	0.013296493	0.034482084	0.330364819	-3.02695669	1.598031192	4.837171209	-1.597868
Eef1a1	Eef1a1-007	1011.8703	0.044686097	0.089675915	0.330382229	-3.026797184	3.703801605	11.21065627	-1.597792
Maff	Maff-202	2380.5644	0.002427859	0.009107795	0.330391911	-3.026708488	10.49230514	31.75714902	-1.59775
RP23-4P1.5	P23-4P1.5-00	1284	0.03209273	0.068931143	0.330538427	-3.025366853	2.938221557	8.889198107	-1.59711
Gm12254	Gm12254-001	2687	6.54566E-05	0.000588422	0.330554027	-3.025224069	9.186559803	27.79140183	-1.597042
Ktn1	Ktn1-007	2052.2029	0.001950197	0.007687219	0.330565417	-3.025119834	4.46523885	13.50788261	-1.596992
Spata5	Spata5-009	1386.3916	0.017190158	0.042126712	0.330887502	-3.02217519	4.741402815	14.32934995	-1.595587
Rbm12b2	Rbm12b2-001	4741.1691	0.000301406	0.0018165	0.3309566	-3.021544215	13.81785841	41.75127014	-1.595286
Mrpl51	Mrpl51-001	1934.9106	9.50911E-05	0.000769435	0.330959049	-3.021521852	5.243136035	15.84225011	-1.595275
Chtop	Chtop-001	4712.3434	7.00942E-05	0.000618585	0.330982898	-3.021304144	15.27958938	46.1642867	-1.595171
Rbm34	Rbm34-201	2738	0.000353566	0.002046928	0.331088571	-3.020339839	8.655717617	26.14320875	-1.594711
Pon3	Pon3-001	4240.705	0.00188199	0.007459341	0.331190054	-3.019414349	9.66243218	29.17488637	-1.594269
Rpl10a	Rpl10a-001	22300.368	0.000131198	0.000979413	0.331301188	-3.018401489	76.76163414	231.6974308	-1.593785
Plekhg2	Plekhg2-006	1497.056	0.031339079	0.067668859	0.331324976	-3.018184784	4.157679011	12.54864353	-1.593681
Gm33280	Gm33280-001	1247	0.016353107	0.040510192	0.331337016	-3.018075105	3.795546466	11.4552443	-1.593629
Gm5641	Gm5641-002	8361.9905	0.000608318	0.003111079	0.331362572	-3.017842345	26.34927935	79.51797097	-1.593517
Cbl	Cbl-201	14201	1.47127E-05	0.000202618	0.331369022	-3.017783603	44.38522527	133.9450051	-1.593489
Ssbp2	Ssbp2-001	1199.2694	0.042375974	0.086022826	0.331439673	-3.017140318	4.048285402	12.2142451	-1.593182
Nlgn2	Nlgn2-002	1969.7299	0.02156614	0.050391616	0.331486095	-3.016717788	5.086162685	15.34351744	-1.59298
Plekhm3	Plekhm3-001	2474.7528	0.002102166	0.008133927	0.331538453	-3.016241378	8.125429351	24.50825623	-1.592752
Nxpe2	Nxpe2-001	4284.234	0.001517661	0.006308072	0.331542471	-3.01620482	6.433124881	19.40362228	-1.592734
Selt	Selt-001	16806.712	1.99103E-07	1.41479E-05	0.331569418	-3.015959694	48.27435212	145.5935002	-1.592617
Rint1	Rint1-006	700.57277	0.004690458	0.015210017	0.331733697	-3.014466145	1.97631184	5.957525134	-1.591903
Ube2d1	Ube2d1-001	3311.9657	7.4904E-05	0.000648511	0.331745935	-3.014354949	9.356397141	28.20350203	-1.591849
Usp4	Usp4-002	2612.1583	0.00408164	0.013626684	0.331830096	-3.013590421	7.945791779	23.94536199	-1.591483
Mcph1	Mcph1-001	1966.4299	0.000106354	0.000833554	0.33186936	-3.013233883	6.411930407	19.32064596	-1.591313
Apc-ps1	Apc-ps1-001	592	0.003527654	0.012177197	0.331894278	-3.013007657	1.511425229	4.553935786	-1.591204
Pde6d	Pde6d-001	1275.6251	0.000384465	0.002184098	0.331980162	-3.012228185	3.404785169	10.25598985	-1.590831
Bscl2	Bscl2-005	1554.5885	7.94993E-05	0.000677326	0.332004746	-3.012005133	4.463272378	13.44339931	-1.590724
Rc3h2	Rc3h2-002	6527.1152	0.000517862	0.002744778	0.332072241	-3.011392932	19.23151624	57.91365207	-1.590431
Uty	Uty-003	334.26706	0.027012859	0.060089005	0.332084272	-3.011283832	0.918571535	2.766079613	-1.590379
Tsc22d1	Tsc22d1-010	936.37411	0.000841417	0.003982661	0.33210218	-3.011121461	3.187592719	9.598228844	-1.590301
Arpc1a	Arpc1a-002	1321.7088	0.002254779	0.008605474	0.332177313	-3.010440391	3.258015138	9.808060365	-1.589975
Ccnd3	Ccnd3-004	348.99641	0.031169747	0.067392452	0.332230425	-3.009959126	1.053385497	3.170647289	-1.589744
Parl	Parl-006	422.4837	0.006501535	0.019615714	0.332264292	-3.009652333	1.283591701	3.863164756	-1.589597
Slc45a4	Slc45a4-001	1397.9942	0.01365023	0.035187323	0.33227077	-3.009593655	5.37627775	16.1804114	-1.589569
Zcrb1	Zcrb1-008	441.51587	0.043289666	0.087479823	0.33240943	-3.008338245	0.942491393	2.835332905	-1.588967
Msh6	Msh6-201	5247	0.00015381	0.001101301	0.33241068	-3.008326927	18.84064865	56.67883067	-1.588961
P23-354G14	23-354G14.3-	1209	0.000107441	0.000839959	0.332414544	-3.008291957	3.179933239	9.566167589	-1.588945
Swt1	Swt1-002	4336.9973	0.000121677	0.00092507	0.332417443	-3.008265726	12.29231633	36.9785539	-1.588932
Sdc4	Sdc4-001	5449.0455	0.002751757	0.010046384	0.332485152	-3.007653107	20.20937001	60.78277451	-1.588638
Rab11a	Rab11a-001	8382.4398	6.35367E-06	0.000114131	0.332508768	-3.00743949	19.53916962	58.76287031	-1.588536
P23-256A5	23-256A5.2-(	718	0.00306024	0.010918444	0.332509666	-3.007431371	2.038371077	6.130261122	-1.588532
Mkks	Mkks-003	645.58293	0.041312046	0.08429774	0.332512077	-3.007409563	2.038055647	6.129268044	-1.588521
Gm15446	Gm15446-002	396.28569	0.004026323	0.013489345	0.332528366	-3.00726224	1.076407115	3.237038472	-1.588451
Fam193a	Fam193a-201	10609.379	0.000222315	0.001443191	0.33258853	-3.006718245	29.30292372	88.10563538	-1.58819
Slc23a1	Slc23a1-001	653.93235	0.000921963	0.004280499	0.332637042	-3.006279735	1.975505489	5.93892212	-1.587979
Rnh1	Rnh1-202	4924.1462	0.00036913	0.002112401	0.332646674	-3.006192692	14.36318685	43.17850736	-1.587937

Slmap	Slmap-005	935.23669	0.016361176	0.040524194	0.332709478	-3.005625225	2.655927891	7.982723865	-1.587665
Klhdc10	Klhdc10-001	4580.3757	4.39095E-06	8.94115E-05	0.332730803	-3.00543259	13.65020898	41.02478293	-1.587573
Gm28438	im28438-001	7778	6.26985E-06	0.000113261	0.332742336	-3.005328423	24.59658279	73.92080938	-1.587523
Cdk7	Cdk7-201	5364.2495	2.28788E-06	5.93809E-05	0.332747584	-3.005281022	14.96845143	44.98440301	-1.5875
Rpl22	Rpl22-002	2534.4278	0.000641989	0.003241173	0.332798752	-3.004818961	7.864693552	23.63198031	-1.587278
Ggnbp2	Ggnbp2-003	2730.5921	0.001816927	0.007259654	0.332865969	-3.004212189	8.215539081	24.68122265	-1.586987
Tmem50a	mem50a-001	9898.7734	3.07542E-05	0.000340884	0.332934865	-3.003590503	25.9395264	77.91171513	-1.586688
Ipo9	Ipo9-001	8099.3813	2.59398E-05	0.000303532	0.333070612	-3.002366358	25.45699335	76.4312204	-1.5861
Erp29	Erp29-001	2033.2412	9.72486E-05	0.000781686	0.333234674	-3.000888196	6.153170866	18.46497782	-1.58539
Nedd4	Nedd4-003	243.13106	0.020690068	0.048767106	0.333263942	-3.000624655	0.73122318	2.194126301	-1.585263
Gm17039	im17039-001	617.99346	0.010753633	0.029161674	0.333281387	-3.000467586	1.53043976	4.592034894	-1.585187
Mllt11	Mllt11-001	999.15372	0.008405234	0.024015955	0.333285487	-3.000430683	3.087736729	9.264540023	-1.58517
Dram2	Dram2-005	529.10576	0.011385992	0.030530826	0.333314108	-3.000173034	0.591922711	1.775870555	-1.585046
Brip1	Brip1-001	3700.7132	0.000395042	0.00223013	0.333327269	-3.000054578	11.04921333	33.14824303	-1.584989
Fnbp1l	Fnbp1l-004	5542.6191	0.000911235	0.004242769	0.333338973	-2.999949245	14.84355611	44.52991494	-1.584938
Gm37855	im37855-001	2139	0.001014435	0.004605946	0.333355326	-2.999802076	4.739003555	14.2160727	-1.584867
P23-151C5.	23-151C5.3-(	6308	0.001404341	0.005946159	0.333447609	-2.99897187	19.8154385	59.42594265	-1.584468
Zfp292	Zfp292-001	10844.671	0.000134737	0.000998861	0.333459098	-2.998868543	34.9587353	104.8366516	-1.584418
Sh3glb1	Sh3glb1-003	25949.074	4.56562E-06	9.20921E-05	0.333513828	-2.998376425	68.24052199	204.6107724	-1.584182
Cd2ap	Cd2ap-201	121497	0.005087774	0.016214411	0.333565503	-2.997911924	306.5137611	918.9012594	-1.583958
Dpy30	Dpy30-201	1017.3786	0.012395458	0.032607099	0.333630239	-2.997330223	3.121988001	9.35762899	-1.583678
Adamts13	Adamts13-002	663	0.001786239	0.007165775	0.333646893	-2.997180618	2.192831578	6.572312303	-1.583606
Gm12335	im12335-001	527	0.022454635	0.051981835	0.333681122	-2.996873162	1.435515597	4.302058167	-1.583458
330032B11F	0032B11Rik-	852	0.0004575	0.00249735	0.333775864	-2.996022507	2.466737876	7.390402195	-1.583048
Atrx	Atrx-001	44676.041	3.11311E-06	7.23855E-05	0.333802069	-2.995787304	130.5315747	391.0448344	-1.582935
Gjc1	Gjc1-003	980.61153	0.00022266	0.001444611	0.33382502	-2.995581336	2.055483836	6.157369017	-1.582836
Acot2	Acot2-001	1926	0.005689771	0.017698959	0.333868169	-2.995194194	5.324782255	15.94875689	-1.58265
10016O06F	0016O06Rik-	802.81238	0.010173223	0.02796636	0.333884911	-2.995044006	2.501711535	7.492736139	-1.582577
Ctdspl	Ctdspl-001	5549.2379	0.000527325	0.002782452	0.33389587	-2.994945699	16.00213713	47.92553178	-1.58253
Arsk	Arsk-001	1724.4514	0.00016358	0.001151379	0.333898478	-2.994922309	5.17879087	15.51007631	-1.582519
Gm17281	im17281-201	300	0.007289109	0.021501332	0.333926134	-2.994674266	0.778913378	2.332591849	-1.582399
Gm7357	Gm7357-001	1713	0.025928245	0.058129371	0.333931213	-2.994628713	4.918321536	14.72854689	-1.582377
Tm4sf1	Tm4sf1-201	76324.553	4.28625E-07	2.11533E-05	0.333961649	-2.994355797	261.4869795	782.9850529	-1.582246
Dcun1d4	Dcun1d4-003	218.95623	0.033676385	0.07169865	0.33396626	-2.994314456	0.664486703	1.98968214	-1.582226
Ncor1	Ncor1-006	262.66902	0.020918015	0.049172411	0.333987266	-2.994126129	0.876636028	2.624758836	-1.582135
Pmpca	Pmpca-001	7281.0115	5.91174E-07	2.54485E-05	0.333997292	-2.994036249	22.31163577	66.80184628	-1.582092
Gm7658	Gm7658-001	259	0.018146344	0.043960411	0.334050936	-2.993555451	0.898656371	2.690177679	-1.58186
Gm17509	im17509-201	432.21643	0.003037677	0.010864562	0.334104468	-2.993075809	1.396403286	4.179540896	-1.581629
Gm14434	im14434-001	375.69927	0.023273247	0.053450595	0.33420754	-2.992152719	1.199393023	3.588767094	-1.581184
Antxr1	Antxr1-201	16430	0.006043398	0.018563801	0.334254633	-2.991731158	47.85544866	143.1706368	-1.580981
Gm10775	im10775-201	266	0.013762615	0.03541593	0.334318776	-2.991157154	0.858896162	2.569093398	-1.580704
Acad11	Acad11-008	332	0.031842269	0.068526403	0.334421712	-2.990236474	1.144132034	3.421225338	-1.58026
Arl8b	Arl8b-201	8643	8.87982E-07	3.27378E-05	0.334461921	-2.989876981	25.04906664	74.89362774	-1.580086
Sec31a	Sec31a-003	929.57041	0.016173874	0.040144588	0.334482222	-2.989695518	2.362563552	7.063345662	-1.579999
Myo1d	Myo1d-003	1790.7867	0.005801261	0.017965027	0.334519842	-2.989359293	4.261558582	12.73932975	-1.579836
Trim24	Trim24-003	2339.2674	0.000160729	0.001135981	0.334656092	-2.988142223	5.779802353	17.27087145	-1.579249
Gm16907	im16907-201	7633.8676	6.16214E-05	0.000561163	0.33470149	-2.987736922	17.54656024	52.42450588	-1.579053
Ap1g1	Ap1g1-002	6763.8029	6.76163E-05	0.000602617	0.334706756	-2.987689915	20.50937779	61.27566119	-1.57903
Mrpl35	Mrpl35-001	3710	4.30211E-05	0.000432993	0.33483314	-2.986562205	10.48101852	31.30221379	-1.578486
Cul4b	Cul4b-003	3868.5923	0.003305789	0.011587685	0.334882961	-2.986117886	10.79921198	32.24772005	-1.578271
Rpl31	Rpl31-003	24099.81	2.51933E-07	1.58908E-05	0.335084499	-2.984321874	76.85863748	229.370913	-1.577403
Bhlhe40	Bhlhe40-001	17333.615	0.00252259	0.009376894	0.335165397	-2.98360156	37.94969129	113.2267581	-1.577055
Slc25a37	Slc25a37-001	2177.7139	0.024405056	0.055436608	0.335262784	-2.982734883	6.087276758	18.15673273	-1.576636
Psma7	Psma7-001	9794.1914	3.81252E-05	0.000395693	0.335296155	-2.982438017	30.30058375	90.36961291	-1.576492
Stk25	Stk25-001	1771.4951	0.00046088	0.002511558	0.335321872	-2.982209281	5.10764734	15.2320733	-1.576382
Srgap2	Srgap2-001	4475.7174	5.8158E-05	0.000538209	0.335374025	-2.981745529	12.83630439	38.27459323	-1.576157
Gm15728	im15728-001	325	0.014598812	0.037104814	0.335418457	-2.981350542	0.924053518	2.754927456	-1.575966
Spred2	Spred2-001	3834.3916	0.000617788	0.003147292	0.3355014	-2.980613494	10.77077096	32.10350525	-1.575609
Ints7	Ints7-004	815.91502	0.012528539	0.032881415	0.335618984	-2.97956924	2.566589892	7.647332294	-1.575104
Mrpl33	Mrpl33-006	431.80126	0.017045995	0.041860878	0.335717384	-2.978695915	1.353590715	4.031935134	-1.574681
Upb1	Upb1-002	399	0.004338974	0.014312806	0.335899912	-2.977077288	1.022647955	3.044501999	-1.573897
P24-496C22	24-496C22.6-	2159	0.010781655	0.029214531	0.335912624	-2.976964633	5.675285596	16.8951245	-1.573842
Dnah7b	Dnah7b-006	996.53872	0.001762727	0.007091073	0.33598434	-2.97632919	2.831590598	8.427745753	-1.573534
Gm37567	im37567-001	16556	8.84659E-06	0.000142412	0.335987766	-2.976298849	47.29271819	140.7572627	-1.573519
Psmd12	Psmd12-001	4633.1246	0.000166327	0.001165687	0.336068512	-2.975583739	14.40399775	42.86030149	-1.57317
Fam220a	Fam220a-006	276.45345	0.048413582	0.095648661	0.336080624	-2.975476506	0.7990973	2.377695242	-1.573121
Zfp106	Zfp106-002	2176.0304	0.029287347	0.064038027	0.336120791	-2.975120929	6.362033037	18.92781764	-1.572948
Rb1cc1	Rb1cc1-015	533.35479	0.011496995	0.030780277	0.336123636	-2.975095749	1.094791711	3.257110166	-1.572936
Utp3	Utp3-001	8014	2.53615E-06	6.33106E-05	0.336127406	-2.975062381	25.12050916	74.73508181	-1.57292
Tsc22d2	Tsc22d2-001	3799.9636	2.90494E-06	6.91411E-05	0.336128125	-2.975056017	11.80987255	35.1350324	-1.572917
Lrwd1	Lrwd1-006	611.12971	0.002798323	0.01017653	0.336261464	-2.973876301	1.840944576	5.474741447	-1.572345
Rnf139	Rnf139-201	5833	3.19303E-06	7.36936E-05	0.336268664	-2.973812626	17.07075998	50.76524155	-1.572314
Cacybp	Cacybp-001	2702.56	0.00017165	0.001190565	0.336283414	-2.973682191	9.240519656	27.47836874	-1.57225
Gdi1	Gdi1-001	11927.671	1.12538E-06	3.78254E-05	0.336310778	-2.973440237	29.94103368	89.02787427	-1.572133
42615	Sept2-203	14320.228	0.000194394	0.001304465	0.336313675	-2.973414626	39.37300047	117.0722555	-1.572121
Ppid	Ppid-002	4449.2291	0.000420858	0.002341136	0.336364609	-2.97296438	15.55877729	46.2556907	-1.571902
Gm37899	im37899-001	2902	0.001800871	0.007211167	0.336421803	-2.972458955	6.815173704	20.2578241	-1.571657
Cipc	Cipc-001	1801.0875	0.000915571	0.004256357	0.336475171	-2.971987492	3.898941504	11.58760538	-1.571428
Fam126b	Fam126b-001	2439.7267	1.03798E-05	0.000158633	0.336475856	-2.971981444	7.490207939	22.26075901	-1.571425
Rps3a1	Rps3a1-001	41953	1.56109E-07	1.23481E-05	0.336527139	-2.971528545	142.8924644	424.6090368	-1.571205
Brk1	Brk1-201	4440	0.00013707	0.001013304	0.336580068	-2.971061256	13.468314	40.01518591	-1.570978
Zfp280b	Zfp280b-201	2432	0.000538044	0.002829418	0.336594814	-2.970931099	7.127559784	21.17548902	-1.570915
Slc25a37	Slc25a37-002	5152.8537	0.000337787	0.001978319	0.336686411	-2.970122843	15.34801959	45.58550358	-1.570523
Nisch	Nisch-011	187.02495	0.042357911	0.085993736	0.336711964	-2.969897441	0.511287679	1.51847197	-1.570413
Tmem29	Tmem29-006	508.49399	0.006462703	0.019539863	0.33672507	-2.96978185	1.186193321	3.522735395	-1.570357
Gm37296	im37296-001	890.92724	0.000894993	0.004188744	0.336792117	-2.969190632	2.511053833	7.455797517	-1.57007
Pdp1	Pdp1-001	786.73618	0.023748331	0.054254292	0.336819444	-2.96894974	2.026596	6.016861668	-1.569953
Rpl10	Rpl10-001	22866.271	2.38784E-05	0.000285255	0.336831486	-2.968843598	72.25178219	214.504241	-1.569901
Gatad1	Gatad1-001	5621.7467	1.69753E-05	0.000224568	0.336869987	-2.968504286	14.89889187	44.22742436	-1.569736
Rnd3	Rnd3-001	2526.1796	0.005930797	0.018276931	0.336893282	-2.96829902	10.90066433	32.35643126	-1.569636
Tgfa	Tgfa-002	555.26326	0.018398294	0.044463843	0.33689534	-2.968280889	1.689940016	5.016216652	-1.569628
Prkca	Prkca-001	2218.5272	0.006581498	0.019813334	0.336898523	-2.968252844	6.996210517	20.76652177	-1.569614
30581F22R	0581F22Rik-	822.42975	0.005650919	0.01760611	0.3369269	-2.968002849	2.798852136	8.307001112	-1.569492
Cd34	Cd34-003	602.19704	0.04438967	0.089248382	0.33699999	-2.967359136	1.15171852	3.417562472	-1.56918
Gm38169	im38169-001	1059	0.011023458	0.029749698	0.337022498	-2.967160961	3.741636264	11.10203705	-1.569083
Ewsr1	Ewsr1-012	2301.146	0.000103324	0.000817474	0.337023343	-2.967153524	7.534106363	22.35485024	-1.56908
Cox10	Cox10-001	1051.9983	0.012639361	0.03310631	0.337087888	-2.966585377	3.595425199	10.66613582	-1.568803
Mtch2	Mtch2-007	789.68115	0.0059238	0.018262058	0.337097697	-2.96649906	2.640146606	7.831992426	-1.568761
Nfe2l1	Nfe2l1-008	10871.046	1.87717E-05	0.000241068	0.337175955	-2.965810536	28.22970988	83.72397097	-1.568426
Ccdc117	Ccdc117-001	5412	3.64852E-05	0.000383789	0.33722441	-2.965384387	17.38543911	51.55450972	-1.568219
Socs4	Socs4-001	7910	1.48852E-06	4.46816E-05	0.337226965	-2.965361917	21.19724181	62.8574936	-1.568208
Slc30a6	Slc30a6-201	766.35602	0.01479136	0.037479033	0.337264677	-2.965030338	2.048584785	6.074116037	-1.568047
Tango2	Tango2-009	934.29554	0.000219895	0.001432887	0.33726501	-2.965027414	2.764707661	8.197434007	-1.568045
Smarcd2	Smarcd2-002	1498.5444	0.004581364	0.014945927	0.33728704	-2.964833755	5.064195302	15.01449717	-1.567951
Zdhhc20	Zdhhc20-201	10958	5.63675E-07	2.47597E-05	0.337376365	-2.964048769	30.5598777	90.58096788	-1.567569
10057M21F	0057M21Rik-	1316	0.000915003	0.004256263	0.337405768	-2.963790472	4.063246605	12.04261157	-1.567443
Sugp2	Sugp2-005	827.56656	0.007004473	0.020832163	0.337411436	-2.963740687	2.67151964	7.917691453	-1.567419

Elmo1	Elmo1-004	5952.8075	3.31997E-05	0.00035903	0.337417092	-2.963691	14.03437631	41.59355475	-1.567395
Eif4g3	Eif4g3-002	611.98736	0.015302935	0.038475039	0.337424502	-2.963625916	1.177831589	3.490652222	-1.567363
Itpripl1	Itpripl1-001	1306.9102	0.006388109	0.019360475	0.337442554	-2.963467374	5.355847589	15.87187959	-1.567286
Map4	Map4-008	966.53539	0.002919126	0.010533638	0.33746543	-2.963266487	2.759542596	8.177260095	-1.567188
Hibch	Hibch-001	1547.8596	0.002379935	0.008972959	0.337507058	-2.962901001	3.889267153	11.52351354	-1.56701
Ppat	Ppat-001	1739.136	0.002752458	0.010047072	0.33757977	-2.962262814	6.260895234	18.54641713	-1.5667
Ewsr1	Ewsr1-007	12314.715	0.003222602	0.011364716	0.337688694	-2.961307318	37.89382076	112.2152487	-1.566234
Ces5a	Ces5a-202	1405	0.000249088	0.001571424	0.33771596	-2.961068233	3.91447996	11.59104226	-1.566118
Gys1	Gys1-003	626.61149	0.002082345	0.008081623	0.337746653	-2.960799143	1.917561673	5.67751496	-1.565987
42431	March2-005	1080.8495	0.005913098	0.018240441	0.337759526	-2.960686294	3.260612966	9.653652117	-1.565932
Ago2	Ago2-001	25106.729	1.25558E-06	4.02278E-05	0.337785182	-2.960461424	78.8446715	233.4166085	-1.565822
Csnk1g3	Csnk1g3-201	6484	3.97862E-06	8.38756E-05	0.337785935	-2.960454818	18.92825038	56.03623005	-1.565819
Gm37968	Gm37968-001	384	0.006177331	0.018878699	0.337807179	-2.960268646	1.052023859	3.114273243	-1.565728
30104L06R	0104L06Rik-	870.66564	0.033154207	0.070762858	0.337821447	-2.960143613	2.543563472	7.529313164	-1.565667
Zfp748	Zfp748-001	2965.0369	6.38933E-05	0.000577088	0.337915557	-2.959319216	8.197266194	24.25832737	-1.565265
Cdip1	Cdip1-001	3584.2095	0.005954227	0.018335936	0.33793255	-2.959170403	10.18285941	30.13281618	-1.565193
Gm13141	Gm13141-001	164	0.025889276	0.058064809	0.337936282	-2.959137721	0.500961291	1.482413452	-1.565177
Hnrnpf	Hnrnpf-001	6013.2815	0.023363075	0.05359883	0.337971456	-2.958829756	18.92859223	56.00648193	-1.565027
Psme4	Psme4-001	13183.408	6.29173E-08	7.32903E-06	0.338050092	-2.958141481	40.11855928	118.6763744	-1.564691
Gm17029	Gm17029-001	762.64818	0.00250091	0.009319571	0.338052982	-2.958116194	2.255523864	6.672101667	-1.564679
Mphosph8	Mphosph8-00	7574.4061	9.56759E-07	3.40783E-05	0.338082662	-2.957856502	22.30616331	65.97843019	-1.564552
Usp53	Usp53-001	10508.287	0.018262706	0.044201659	0.33810201	-2.957687241	34.1495215	101.003604	-1.56447
Dynlt1-ps1	Dynlt1-ps1-00	208	0.025562971	0.057485545	0.338179536	-2.957009206	0.740973262	2.191064756	-1.564139
Gm14760	Gm14760-001	1119	0.006180134	0.018883897	0.338249062	-2.956401399	3.855647439	11.39884148	-1.563842
Tra2a	Tra2a-201	8615	1.06834E-05	0.000162121	0.338272255	-2.956198696	26.84612196	79.36247074	-1.563743
Synj2bp	Synj2bp-004	7163.625	0.000123711	0.000937827	0.338318252	-2.955796778	20.35105131	60.1535719	-1.563547
Drosha	Drosha-201	3877.9345	0.010586522	0.028829481	0.338397467	-2.955104861	11.31281194	33.43054557	-1.563209
Gm26617	Gm26617-201	1905.4056	0.00063539	0.003215915	0.338451756	-2.954630852	5.724411022	16.91352142	-1.562978
Ypel2	Ypel2-001	6175	0.000130341	0.000974283	0.338470387	-2.954468212	20.62151225	60.92560244	-1.562898
Gm38155	Gm38155-001	758	0.004648824	0.015107357	0.338557892	-2.953704593	1.904830344	5.626306136	-1.562526
Rab7	Rab7-003	26863.935	1.23454E-05	0.000178938	0.338591318	-2.953413	74.92296886	221.2784702	-1.562383
Kmt2e	Kmt2e-002	18967.881	0.000339458	0.001985051	0.33859817	-2.953353234	56.19066488	165.9508819	-1.562354
Zbed5	Zbed5-003	486	0.002362645	0.00891687	0.33873812	-2.952133056	1.36852276	4.040061277	-1.561758
Rbfox2	Rbfox2-203	4628.9975	0.00046104	0.002512029	0.338846816	-2.951186059	13.0213024	38.42828612	-1.561295
Osbpl10	Osbpl10-002	319.92557	0.031698137	0.068276283	0.338858222	-2.951086727	0.901187314	2.659481921	-1.561246
Cep83	Cep83-201	4570	1.96267E-05	0.000248876	0.338868476	-2.950997428	14.04559827	41.44852438	-1.561203
Tcp1	Tcp1-002	1733.3389	0.018073189	0.043818969	0.338891294	-2.950798731	5.540573542	16.34911738	-1.561106
Zbtb25	Zbtb25-003	357.10323	0.020639976	0.048682541	0.338913614	-2.9506044	1.255803074	3.705378074	-1.561011
Plrg1	Plrg1-001	2667.3572	0.004057943	0.013566401	0.338945483	-2.950326973	8.255541536	24.35654687	-1.560875
Gm13977	Gm13977-001	3398	9.57855E-06	0.000150213	0.33896092	-2.950192609	9.955691674	29.37120799	-1.560809
Spin4	Spin4-001	1058	0.002895788	0.010469834	0.338964863	-2.950158288	2.495910979	7.363332462	-1.560792
Meis2	Meis2-014	1799.6263	0.003100335	0.011019766	0.338967898	-2.950131877	5.438148571	16.04325545	-1.560779
Bod1	Bod1-001	3154.3787	0.000273061	0.001680875	0.338987416	-2.949962015	9.649411933	28.46539867	-1.560696
Mphosph10	Mphosph10-00	3973.7126	6.00653E-05	0.000551243	0.339031634	-2.949577268	15.126301	44.61619358	-1.560508
230066G23F	0066G23Rik-	1151	0.007902538	0.022914148	0.339078799	-2.949166988	3.444264731	10.15771184	-1.560308
Mdh2	Mdh2-007	329.58592	0.033819382	0.071944958	0.339143038	-2.948608368	0.798834022	2.355448683	-1.560034
Pigb	Pigb-007	596.00432	0.002155227	0.008296788	0.339145001	-2.948591302	1.571496953	4.633702248	-1.560026
Ubtf	Ubtf-008	2265.9867	0.004177874	0.013900543	0.339192777	-2.948175989	6.751075533	19.90335879	-1.559823
Gm38245	Gm38245-001	584	0.017434821	0.042606044	0.33922756	-2.947873696	1.671468097	4.927276837	-1.559675
Gpn1	Gpn1-001	2040.8493	0.000580367	0.002998578	0.339231097	-2.947842957	6.494429059	19.14455696	-1.55966
Secisbp2l	Secisbp2l-002	1797.0469	0.001602402	0.006580607	0.339240479	-2.947761436	4.081529664	12.03137574	-1.55962
Camk2d	Camk2d-009	1192.8366	0.004997557	0.015999845	0.339269793	-2.947506739	2.708748488	7.984054425	-1.559495
Bnip3l	Bnip3l-001	1600.9049	0.004646688	0.01510383	0.339333674	-2.946951857	5.013540809	14.7746634	-1.559223
Gorab	Gorab-002	510.09186	0.028417525	0.062525059	0.339348338	-2.946824514	1.227770851	3.61802524	-1.559161
L3mbtl3	L3mbtl3-201	1522.3369	0.002247526	0.008583605	0.33936066	-2.946717512	5.118708775	15.08338879	-1.559109
Ahctf1	Ahctf1-001	31090.962	1.29408E-05	0.000184676	0.339510185	-2.945419739	97.42373791	286.9538007	-1.558473
Map2k4	Map2k4-005	6028.8421	0.000223722	0.001449306	0.339527322	-2.945271072	20.83970336	61.37857545	-1.5584
Pan3	Pan3-002	4450.2601	0.000964743	0.004424537	0.339562759	-2.944963698	12.53914301	36.92732097	-1.55825
P24-164P24	24-164P24.2-	2323	0.001838161	0.007326548	0.33963404	-2.944345626	6.001864418	17.67156325	-1.557947
Vmac	Vmac-202	1053.4252	0.020317281	0.048053786	0.339691695	-2.943845891	3.36223394	9.897898569	-1.557702
Sirpa	Sirpa-001	8305.7978	0.002516376	0.009359893	0.339719807	-2.943602284	25.96358377	76.4264645	-1.557583
230104I21R	0104I21Rik-	354.80026	0.033717316	0.071767949	0.339726987	-2.943540071	1.109678085	3.266381909	-1.557552
Pxdc1	Pxdc1-001	1063.3502	0.037545785	0.078145738	0.33979001	-2.94299412	2.448751309	7.206660704	-1.557285
Bicd1	Bicd1-001	1809.1685	0.000175212	0.001207689	0.339811594	-2.94280718	4.156033245	12.23040447	-1.557193
Kdm5d	Kdm5d-002	2428.1888	5.88853E-05	0.000543216	0.339817189	-2.942758729	7.596782332	22.35549752	-1.557169
Gm3839	Gm3839-001	3955.695	6.91536E-05	0.000612493	0.33984173	-2.942546229	12.51017291	36.81176212	-1.557065
Sft2d1	Sft2d1-003	1599.6988	0.000425619	0.002361529	0.339885502	-2.942167275	5.370582556	15.80115225	-1.556879
Ikbip	Ikbip-001	4090.5705	4.74495E-05	0.000463532	0.339908849	-2.941965184	11.25595898	33.11463942	-1.55678
Ufc1	Ufc1-001	2258.6579	0.002627732	0.009693544	0.339912721	-2.941931675	6.438853273	18.94266639	-1.556764
Gm28818	Gm28818-001	590	0.001816057	0.007257873	0.33991501	-2.941911864	1.873701379	5.512264314	-1.556754
Ly75	Ly75-001	2060.5238	0.001233122	0.005376758	0.339921198	-2.941858308	6.920834751	20.36011521	-1.556728
Ttc13	Ttc13-003	1610.271	0.003625087	0.012430171	0.339941421	-2.941683293	4.907974929	14.43770785	-1.556642
Paip2b	Paip2b-002	1060.6605	0.000796257	0.00381584	0.339942315	-2.941675558	3.097840893	9.112842837	-1.556638
Appl1	Appl1-001	11914.78	4.92247E-07	2.31674E-05	0.339976235	-2.941382069	31.99540895	94.11072482	-1.556494
Lrrc47	Lrrc47-001	695.07318	0.000908654	0.00423363	0.339985415	-2.941302646	2.334500373	6.866472123	-1.556455
Nfya	Nfya-003	669.34675	0.006469177	0.019550808	0.340081878	-2.940468358	1.983699263	5.833004916	-1.556046
Snx11	Snx11-004	1375.4416	0.000592904	0.003046804	0.340117426	-2.940161027	3.901157226	11.47003044	-1.555895
Mfsd6	Mfsd6-001	17748.852	0.00010424	0.000821311	0.340119142	-2.940146194	30.04741037	88.34377924	-1.555888
Dab2	Dab2-006	1461.0467	0.024781834	0.056087876	0.340146389	-2.939910678	5.472764322	16.08943827	-1.555772
Gin1	Gin1-006	3114.2391	2.94299E-05	0.000331243	0.340164495	-2.939754192	9.164470111	26.94128942	-1.555696
30159M07F	0159M07Rik-	336.94874	0.013681438	0.035248177	0.340227542	-2.939209433	1.153056324	3.389074023	-1.555428
Gm6710	Gm6710-001	456.78621	0.009031165	0.025443565	0.340267801	-2.93886168	1.255051926	3.688424013	-1.555257
Wbp2	Wbp2-003	443.42838	0.025145789	0.056739131	0.3404211	-2.937538243	1.596276429	4.689123058	-1.554608
Socs6	Socs6-001	2117.3585	0.000172476	0.001194121	0.340421298	-2.937536535	6.260960028	18.39179882	-1.554607
P23-412L13	23-412L13.5-	326	0.01547419	0.03880728	0.340449885	-2.937289877	0.976413459	2.868009369	-1.554486
Gm6498	Gm6498-001	334.83296	0.016983377	0.041739152	0.340522605	-2.9366626	0.741113604	2.176400604	-1.554178
Zfp74	Zfp74-002	296.5394	0.026171533	0.058564741	0.340564082	-2.936304954	0.936054259	2.748540756	-1.554002
Gls	Gls-009	1196.7085	0.002010227	0.007857817	0.340605923	-2.935944244	3.135278838	9.205003857	-1.553825
530045J12R	30045J12Rik-	373.03866	0.008529525	0.024292054	0.340618584	-2.935835118	0.943831395	2.770933354	-1.553771
Atrx	Atrx-021	16023.948	3.03364E-05	0.000337564	0.340634743	-2.935695847	46.65767799	136.9727515	-1.553703
Crbn	Crbn-002	737.36507	0.0030925	0.010998767	0.340637584	-2.935671362	2.188543455	6.424844345	-1.55369
Srsf11	Srsf11-003	971.21399	0.007555882	0.022130064	0.340681532	-2.935292661	3.203966771	9.404580149	-1.553504
Pafah2	Pafah2-001	1952.6099	0.02042313	0.048277466	0.340719674	-2.934964065	5.057812703	14.84449853	-1.553343
Sptan1	Sptan1-015	814.98699	0.024731127	0.056002717	0.340740093	-2.934788184	1.933494778	5.674397628	-1.553256
Irf3	Irf3-003	876.67569	0.031608378	0.068130079	0.341074918	-2.931907178	2.415155102	7.081010577	-1.551839
P24-374B14	24-374B14.6-	291	0.010403481	0.028478198	0.341076423	-2.931894242	0.93975377	2.755258668	-1.551833
Tpm4	Tpm4-001	86860.208	3.9643E-05	0.000407716	0.341152504	-2.931240395	240.316644	704.4258544	-1.551511
Neo1	Neo1-201	749	0.044078833	0.088743243	0.341338443	-2.929643641	1.947436629	5.705295335	-1.550725
Gm21761	Gm21761-001	316	0.005500836	0.017260984	0.341414926	-2.928987352	0.830800685	2.433404698	-1.550402
Gm16702	Gm16702-201	3253	6.63447E-06	0.000117129	0.341471601	-2.928501221	9.2447497	27.07326079	-1.550162
Arpc1b	Arpc1b-007	451.00761	0.013780075	0.035451632	0.34147185	-2.92849908	1.421684813	4.163402667	-1.550161
Ccdc90b	Ccdc90b-001	1806.7687	0.000294441	0.001782995	0.341477957	-2.928446712	4.678768024	13.70152283	-1.550136
Abcd3	Abcd3-005	788.9872	0.011789335	0.031383858	0.341504299	-2.928220829	2.041675626	5.978477094	-1.550024
Gm37169	Gm37169-001	829	0.000464344	0.002526011	0.34155576	-2.927779642	2.532771763	7.415397607	-1.549807
Akap11	Akap11-001	11019.549	0.004158913	0.01385091	0.341574527	-2.927618776	37.49989232	109.7853888	-1.549728
Gab1	Gab1-201	15250	0.000902082	0.004212506	0.341580562	-2.927567052	51.10896404	149.6249192	-1.549702
Lama4	Lama4-005	879.33472	0.031846752	0.068531745	0.341616184	-2.927261787	2.268375872	6.640130009	-1.549552

Apbb2	Apbb2-007	2619.904	0.025711567	0.057722442	0.341682914	-2.926690095	6.419137277	18.78682549	-1.54927
Sec11c	Sec11c-003	1248.5645	7.32434E-05	0.000639293	0.341758301	-2.926044508	3.825195156	11.19269128	-1.548952
Mipol1	Mipol1-007	261.34729	0.034029168	0.072305913	0.341795726	-2.92572412	1.062294347	3.107980192	-1.548794
Ubiad1	Ubiad1-001	669.82018	0.026217302	0.058639234	0.341824915	-2.925474291	2.281094418	6.673283074	-1.548671
Tcp1	Tcp1-003	13567.291	0.000109777	0.000855224	0.341832166	-2.92541223	51.07267458	149.4086269	-1.54864
Ndrg1	Ndrg1-005	316.1029	0.034310187	0.072784163	0.34185352	-2.925229494	0.870043731	2.545077582	-1.54855
Kdelr2	Kdelr2-001	12248	0.000501817	0.002678806	0.341892004	-2.924900229	37.46461054	109.5802479	-1.548387
Furin	Furin-003	1808.7587	0.000180877	0.001236533	0.341928081	-2.92459162	5.712171932	16.70577017	-1.548235
Rps11-ps1	Rps11-ps1-00	951	0.000500016	0.002670813	0.341976884	-2.924174253	2.958964511	8.65252784	-1.548029
Hnrnpd	Hnrnpd-001	5830.5527	5.58088E-06	0.000104794	0.342019433	-2.923810468	17.14762463	50.1364044	-1.54785
Aen	Aen-006	320	0.011939441	0.031677578	0.34203215	-2.92370176	1.146692888	3.352588016	-1.547796
Gm37033	Gm37033-001	7097	0.000188698	0.001276288	0.342032604	-2.923697885	19.88558934	58.13945549	-1.547794
Ptpn2	Ptpn2-002	620.70356	0.005545742	0.0173653	0.342037205	-2.923658554	1.763411622	5.155613472	-1.547775
Hgh1	Hgh1-201	480	0.015524081	0.038895375	0.342039006	-2.923643162	1.637640802	4.787877332	-1.547767
H3f3b	H3f3b-201	17918.747	0.000100241	0.000798674	0.342046274	-2.923581034	59.28545112	173.3258205	-1.547737
Nedd4	Nedd4-005	2518.2963	0.000227804	0.001470596	0.34210032	-2.923119157	6.900046659	20.16965858	-1.547509
Pdcd6	Pdcd6-201	3944	1.97286E-06	5.34153E-05	0.34213074	-2.922859258	12.64566693	36.96150467	-1.54738
Cmtm3	Cmtm3-201	1459	0.007098444	0.021070156	0.342183607	-2.922407679	5.569284956	16.27572112	-1.547157
Eif5a2	Eif5a2-001	2723	8.16882E-05	0.000691503	0.342261422	-2.921743253	8.705827911	25.43619396	-1.546829
Serpine2	Serpine2-003	2158	0.000113568	0.000877638	0.342284577	-2.921545603	5.771764119	16.86247208	-1.546732
Mrpl39	Mrpl39-001	2713.8678	0.000208184	0.001373326	0.342295691	-2.921450741	8.497901282	24.8262	-1.546685
Setd5	Setd5-008	4302.1063	0.049952373	0.097886988	0.342341811	-2.921057167	12.46758814	36.41853769	-1.546491
931440P22R	1440P22Rik-	435.11596	0.003041945	0.010873161	0.342375227	-2.920772069	1.38419468	4.042917159	-1.54635
Serbp1	Serbp1-201	45580	6.53481E-06	0.000115985	0.342385543	-2.920684065	149.6461163	437.0690271	-1.546306
Dram2	Dram2-001	4585.437	0.003332703	0.011662928	0.342444268	-2.920183203	10.80936729	31.5653328	-1.546059
Bcl2	Bcl2-001	4574.7687	0.001643906	0.006706822	0.342469655	-2.919966738	15.14394472	44.21981486	-1.545952
Necap1	Necap1-201	2866	0.000516903	0.002740973	0.34247746	-2.919900186	9.477178984	27.67241668	-1.545919
H3f3a-ps2	H3f3a-ps2-00	230	0.048579416	0.095904374	0.342533203	-2.919425012	0.819349023	2.392028031	-1.545684
Smchd1	Smchd1-001	16035.511	5.74509E-06	0.000106519	0.342594272	-2.918904609	56.03302401	163.555052	-1.545427
Tmem203	Tmem203-001	322.13359	0.041820712	0.085102453	0.342629549	-2.918604078	0.921310251	2.688939856	-1.545279
Ablim1	Ablim1-004	1418.2695	0.017765434	0.043233347	0.342670284	-2.918257133	3.984972585	11.62917467	-1.545107
Acbd5	Acbd5-202	1649.8461	0.020717013	0.048810463	0.34269899	-2.918012681	3.971982928	11.59029655	-1.544986
Gm16581	Gm16581-001	281	0.023629095	0.054039224	0.342703455	-2.917974665	0.92681888	2.70443401	-1.544967
Frmd4b	Frmd4b-008	561.97359	0.026766773	0.059615163	0.342739939	-2.917664058	1.831247231	5.342964228	-1.544814
Cldnd1	Cldnd1-001	1931.5761	0.004623305	0.015050254	0.342830076	-2.91689694	6.164604229	17.98151521	-1.544434
Rgs3	Rgs3-012	797.01857	0.015914327	0.039646536	0.342847244	-2.91675088	2.655116094	7.744312203	-1.544362
Mga	Mga-002	12031.395	2.78291E-05	0.000318888	0.342914695	-2.916177154	35.748467	104.2488627	-1.544078
Cers4	Cers4-001	12168.113	0.008859539	0.025032109	0.342996987	-2.915477503	32.01863156	93.3496	-1.543732
Nsmce2	Nsmce2-202	563.23971	0.017970534	0.043630168	0.343009758	-2.915368958	1.415040159	4.125364152	-1.543678
Dennd5a	Dennd5a-003	2410.6578	0.000955393	0.004394012	0.343064234	-2.914906014	6.642432367	19.36206606	-1.543449
Dclre1a	Dclre1a-003	906.17315	0.000943184	0.004354819	0.34307417	-2.914821598	2.837786182	8.271640454	-1.543408
Trabd	Trabd-005	544.11935	0.001451305	0.006102787	0.343078295	-2.914786552	1.531356844	4.463578335	-1.54339
Usmg5	Usmg5-201	1474	0.000980826	0.004481493	0.343123053	-2.914406338	4.327266889	12.61141405	-1.543202
Akap9	Akap9-003	7184.6765	0.00112887	0.005010861	0.343127044	-2.914372442	21.2367603	61.89182897	-1.543185
Zfp652	Zfp652-003	2230.9529	0.000142096	0.001039468	0.343170625	-2.914002326	5.903669255	17.20330594	-1.543002
Rhoa	Rhoa-001	35168.404	2.74679E-05	0.000315697	0.343232066	-2.913480703	87.17595327	253.9854576	-1.542744
Gm21811	Gm21811-001	3371	2.93574E-05	0.000330753	0.343244237	-2.913377393	9.997584891	29.1267378	-1.542693
Abca9	Abca9-001	1645.2898	0.005745287	0.01782715	0.343262285	-2.913224217	5.271178859	15.3561259	-1.542617
Ing4	Ing4-005	1894.6677	7.46287E-05	0.000647589	0.343326583	-2.912678624	5.652360757	16.46351035	-1.542347
Cand1	Cand1-001	13012.302	9.68599E-07	3.43066E-05	0.343351368	-2.912468375	38.98899657	113.5542195	-1.542242
Yap1	Yap1-003	2991.2436	0.000100608	0.000800234	0.343375711	-2.9122619	8.848473504	25.76907226	-1.54214
Ube2d2a	Ube2d2a-202	10337.153	3.82477E-06	8.16753E-05	0.343426084	-2.911834731	31.13453066	90.65860771	-1.541928
Ankfy1	Ankfy1-006	1564.5585	0.000272327	0.001678158	0.343440265	-2.911714504	4.759627345	13.85867597	-1.541869
Gfod2	Gfod2-001	1679.3589	0.000227824	0.001470596	0.343515309	-2.911078408	4.480417236	13.04284587	-1.541554
930016H08R	0016H08Rik-	1006.581	0.00067827	0.003376959	0.343587783	-2.910464372	3.273291358	9.526797876	-1.541249
Rab28	Rab28-001	1422.9748	0.006513065	0.01964704	0.343588411	-2.91045905	4.304514162	12.5281122	-1.541247
Zfp772	Zfp772-201	1564	0.000516657	0.002740092	0.343633464	-2.910077468	4.309533472	12.54107626	-1.541058
Chd6	Chd6-006	741.63186	0.037900955	0.078714597	0.343653066	-2.909911473	2.246070343	6.535865861	-1.540975
Lix1l	Lix1l-001	7544.8645	2.31351E-05	0.000279596	0.343710661	-2.909423865	24.07500246	70.04438671	-1.540733
Maml1	Maml1-002	4572.4324	1.93339E-05	0.000246075	0.343739406	-2.909180568	14.86047217	43.23179687	-1.540613
E2f6	E2f6-201	3429	2.65794E-06	6.53097E-05	0.343805747	-2.90861921	9.912922257	28.83291611	-1.540334
Gm37152	Gm37152-001	553	0.004655549	0.015122588	0.343832708	-2.908391133	1.495807395	4.350392965	-1.540221
Pprc1	Pprc1-003	1440.3361	0.041611674	0.08476773	0.343835038	-2.908371423	5.046051845	14.67579298	-1.540212
Zfp65	Zfp65-002	996.29206	0.013766373	0.035421701	0.343874584	-2.908036964	3.507313061	10.19939603	-1.540046
RP24-80F7.	P24-80F7.5-0	407.83219	0.049820659	0.097690363	0.343936737	-2.907511445	1.582338671	4.600667795	-1.539785
Adgrl4	Adgrl4-004	1041.8235	0.010679732	0.029032528	0.343959385	-2.907320007	3.47396199	10.0999192	-1.53969
Pigb	Pigb-001	1251.4107	0.016305417	0.040418365	0.343977381	-2.907167898	3.916165637	11.38495102	-1.539614
Ranbp9	Ranbp9-201	14528.309	3.92317E-05	0.000404238	0.343987689	-2.907080786	45.12578601	131.1843054	-1.539571
Tbc1d32	Tbc1d32-201	2898	3.75135E-05	0.000391718	0.344029486	-2.906727596	8.600231498	24.99853023	-1.539396
Arhgef12	Arhgef12-201	48109.578	0.000397433	0.002239567	0.344038288	-2.906653225	132.2378288	384.3695115	-1.539359
Ptcd3	Ptcd3-201	3881	1.06081E-05	0.000161192	0.344039309	-2.906644604	12.37384598	35.9692886	-1.539355
830082K12R	0082K12Rik-	415.31209	0.003927227	0.013229815	0.344057548	-2.906490512	1.28610121	3.738040963	-1.539278
Pwp1	Pwp1-001	2494.1058	0.000771904	0.003725194	0.344091337	-2.906205108	8.54608463	24.83667481	-1.539137
Gm11878	Gm11878-001	2008	9.00036E-05	0.000738707	0.344127476	-2.905899907	7.073743977	20.55559197	-1.538985
Commd1	Commd1-001	543.4046	0.028324185	0.062362004	0.34426153	-2.90476836	1.691114836	4.912296869	-1.538423
Orc3	Orc3-009	4147.7642	7.01851E-05	0.000618618	0.344276741	-2.904640022	11.13800363	32.35189109	-1.538359
Gm13341	Gm13341-001	9503	2.06818E-06	5.5436E-05	0.344316656	-2.904303302	27.86304716	80.92273988	-1.538192
810013P06R	0013P06Rik-	849	0.026470046	0.059108037	0.344418656	-2.903443183	2.770313863	8.043448899	-1.537765
Dtd2	Dtd2-001	1091.2114	0.001870391	0.007426488	0.344521138	-2.90257952	2.856400387	8.290929264	-1.537336
Gm12116	Gm12116-001	397.14566	0.013427698	0.034740795	0.344521925	-2.902572891	1.199836318	3.482612369	-1.537332
Gm13988	Gm13988-001	553	0.007053882	0.020959092	0.344548355	-2.902350233	1.612661233	4.680507707	-1.537222
Sfpq	Sfpq-005	336.91001	0.03853777	0.079748572	0.344624608	-2.901708053	1.047325188	3.039031933	-1.536902
Prkcq	Prkcq-003	3776.8481	9.86772E-05	0.000789833	0.344630933	-2.901654799	11.11178457	32.24256304	-1.536876
Tmem189	Tmem189-001	3948.3666	1.24072E-05	0.000179682	0.344690109	-2.901156643	9.068145311	26.30811001	-1.536628
RP23-370G17	23-370G17.2-	1886.5311	0.004256716	0.014105315	0.344720508	-2.900900806	6.647809921	19.28463716	-1.536501
RP23-151D16	23-151D16.2-	927	0.000677113	0.003373208	0.344762338	-2.900548843	2.374696733	6.887923861	-1.536326
Atxn7	Atxn7-201	12673	1.07744E-06	3.66824E-05	0.344766995	-2.90050966	37.15136502	107.7578931	-1.536306
Akap11	Akap11-201	10168.47	0.001309262	0.00562951	0.344797832	-2.900250257	28.98269041	84.0570553	-1.536177
Gm37404	Gm37404-001	6317	8.48369E-06	0.000138299	0.344816358	-2.900094431	18.39058644	53.33443733	-1.5361
Fpgt	Fpgt-001	1409.2535	0.005171612	0.016437403	0.344912243	-2.899288209	4.161553717	12.06554362	-1.535699
Sgms2	Sgms2-001	12481.487	1.12672E-06	3.78334E-05	0.344943933	-2.89902185	37.45775528	108.590851	-1.535566
Amd2	Amd2-201	1849	0.000555467	0.002901883	0.344987097	-2.898659133	6.565501095	19.03114971	-1.535386
Dnajb4	Dnajb4-006	1209.6542	0.012123438	0.032060667	0.345055177	-2.898087227	3.281166784	9.509107545	-1.535101
RP24-102B13	24-102B13.5-	7203	1.85E-05	0.000238386	0.345063164	-2.89802014	21.40167683	62.0224905	-1.535068
Airn	Airn-006	1432.9003	0.001586067	0.006529181	0.345117291	-2.897565627	4.547694245	13.17724253	-1.534841
Hectd2	Hectd2-003	1459.0123	0.006737995	0.020190404	0.345269731	-2.89628632	4.4164475	12.79129648	-1.534204
Taf1b	Taf1b-201	2715	7.23213E-05	0.00063302	0.345281755	-2.896185468	7.596128322	21.99979646	-1.534154
Pbrm1	Pbrm1-008	939.71519	0.022161184	0.051445069	0.345322105	-2.895847055	2.990376022	8.659671596	-1.533985
Abracl	Abracl-201	2122.0843	0.00654788	0.019732944	0.345369519	-2.895449498	8.756756268	25.35474554	-1.533787
Tfe3	Tfe3-009	2041.4521	0.001237543	0.0053926	0.345426609	-2.894970951	6.461389193	18.70553402	-1.533549
AI597479	AI597479-001	2386	3.87603E-05	0.000400827	0.34544356	-2.8948289	6.96282476	20.15618634	-1.533478
Actr3	Actr3-001	13661.712	0.008327042	0.023836287	0.345499985	-2.894356128	53.08338392	153.6422176	-1.533242
Myo9b	Myo9b-201	5395.874	0.003898795	0.013156031	0.345529981	-2.89410487	18.64367467	53.95674965	-1.533117
Klhl13	Klhl13-201	1183.0864	0.027328782	0.060654626	0.345608461	-2.893447682	2.863268444	8.284717441	-1.53279
Drr1	Drr1-002	211	0.046555953	0.092689729	0.345627891	-2.893285021	0.57878541	1.674591158	-1.532708
Ktn1	Ktn1-008	2081.3742	0.010240281	0.028114598	0.345690102	-2.892764339	6.269068416	18.13493755	-1.532449
Ube2e3	Ube2e3-001	4072.0397	3.19379E-05	0.000350151	0.345828466	-2.891606959	11.89499865	34.39566088	-1.531871

Hdhd2	Hdhd2-001	829.13307	0.037210616	0.077607089	0.345923675	-2.890811106	2.59011344	7.487528696	-1.531474
Kpna1	Kpna1-001	5576.5668	0.000129672	0.000970342	0.345934665	-2.890719267	18.39496713	53.17468589	-1.531429
Ttn	Ttn-001	795.81808	0.019575441	0.046725127	0.345956651	-2.890535554	2.362772957	6.829679238	-1.531337
Rtf1	Rtf1-001	14721.404	1.02285E-06	3.5458E-05	0.346031954	-2.88990652	45.50165052	131.4955165	-1.531023
230088H06R	0088H06Rik-	690.02626	0.011915231	0.0316354	0.346039184	-2.889846138	1.78768854	5.166144824	-1.530993
Por	Por-009	285.98085	0.013394372	0.034670287	0.346084502	-2.889467727	0.517469896	1.495212565	-1.530804
Senp7	Senp7-202	2117.6955	0.003925336	0.013227104	0.346104361	-2.889301936	5.927077254	17.12511578	-1.530721
Fbxl20	Fbxl20-001	11941.4	1.32879E-05	0.000188712	0.346162322	-2.888818152	33.84022379	97.75825273	-1.530479
310058I24R	0058I24Rik-(	726.23485	0.047847074	0.094780931	0.346210553	-2.888415708	2.554701419	7.379039707	-1.530278
Psmc5	Psmc5-002	721.40775	0.032096678	0.068935299	0.346275136	-2.887877001	2.425844676	7.005541047	-1.530009
Gm3355	Gm3355-001	483	0.011901988	0.031607591	0.346438852	-2.886512281	1.27722579	3.686727927	-1.529327
Atf7	Atf7-008	421.30712	0.016365377	0.040528862	0.346442639	-2.886480726	0.901625669	2.602525116	-1.529312
Eif2b1	Eif2b1-001	1949.5748	0.00017736	0.001219797	0.346496393	-2.886032927	6.378253085	18.40784842	-1.529088
I1-1749A12	1-1749A12.1-	3187	2.38865E-05	0.000285255	0.346510503	-2.88591541	9.568274529	27.61323091	-1.529029
00002B13R	0002B13Rik-	542	0.002910015	0.010507961	0.346584298	-2.885300936	1.657197723	4.781514142	-1.528722
Gm10247	Gm10247-201	255	0.018698977	0.045047476	0.346661748	-2.884656312	0.907760249	2.618576333	-1.528399
30016H08R	0016H08Rik-	4491.5301	7.16628E-06	0.000123215	0.346687744	-2.88444001	13.27787676	38.29923898	-1.528291
Ipo8	Ipo8-004	1237.765	0.01219381	0.032207757	0.346832064	-2.883239767	2.737475803	7.892799095	-1.527691
Fbrsl1	Fbrsl1-201	2282.6428	0.002470975	0.0092412	0.346877748	-2.882860042	6.042334829	17.41920564	-1.527501
Gm14401	Gm14401-004	649.41638	0.022103397	0.051340782	0.34696826	-2.882108003	1.827399851	5.266763736	-1.527124
Tspan5	Tspan5-003	2119.7428	0.014564389	0.037035437	0.347133917	-2.880732628	6.131371986	17.66284334	-1.526436
Snx24	Snx24-202	5043.7045	0.00044089	0.002427675	0.347238003	-2.87986911	16.7894043	48.35128681	-1.526003
Dopey1	Dopey1-012	1247.4098	0.003470603	0.012020241	0.347302304	-2.879335925	3.142500315	9.048314053	-1.525736
P23-381G13	23-381G13.3-	9993	9.21678E-06	0.000146108	0.347305193	-2.879311969	28.23098262	81.28580616	-1.525724
Eci2	Eci2-002	1178.8867	0.021095913	0.049526015	0.347324061	-2.879155553	3.398640193	9.785213786	-1.525646
10003H01R	0003H01Rik-	529.36189	0.026117532	0.058471918	0.347337512	-2.879044056	1.013345451	2.917466197	-1.52559
Mknk1	Mknk1-013	251.12669	0.031932953	0.068678407	0.347407929	-2.878460495	0.791267673	2.277632738	-1.525297
Chpf2	Chpf2-001	3471.8878	0.004108312	0.013702374	0.347423831	-2.878328746	9.567478389	27.53834808	-1.525231
H2-T23	H2-T23-003	1017.6676	0.022419407	0.051928362	0.347532328	-2.877430154	2.58591106	7.440778461	-1.524781
Zfp169	Zfp169-003	712.50689	0.00778634	0.022644318	0.347555648	-2.877237085	1.83031174	5.266240814	-1.524684
Pecam1	Pecam1-004	191951.78	0.000309463	0.001851502	0.347640955	-2.876531046	425.5553468	1224.123167	-1.52433
Gm9803	Gm9803-201	444	0.016201624	0.040197433	0.347643781	-2.876507663	1.318727085	3.793328566	-1.524318
Rab14	Rab14-006	284.54002	0.028379878	0.062464548	0.347645915	-2.876490008	0.818008661	2.352993739	-1.524309
Mrps35	Mrps35-005	482.1524	0.038352615	0.079461441	0.347688722	-2.876135858	1.475890822	4.244862516	-1.524132
Rtp4	Rtp4-201	1720	0.000628222	0.003187646	0.347862991	-2.874694999	5.182892086	14.89923396	-1.523409
Kitl	Kitl-001	4491.2165	0.001584754	0.006524563	0.347874985	-2.874595885	11.35004061	32.62678003	-1.523359
Rasa1	Rasa1-002	276.18093	0.044026515	0.088658756	0.347893653	-2.874441629	0.707779658	2.034471313	-1.523282
Zfp148	Zfp148-202	11627.697	9.07622E-06	0.000144788	0.347895248	-2.874428452	33.22271094	95.49630561	-1.523275
Bnip2	Bnip2-007	768.36703	0.002322731	0.008808941	0.34792461	-2.87418588	1.747328881	5.022147997	-1.523153
Gm12879	Gm12879-001	1600	0.000490584	0.002634777	0.347950232	-2.873974233	4.637942988	13.32932864	-1.523047
Pou2f1	Pou2f1-001	5114.1951	3.65499E-05	0.000384234	0.347962804	-2.873870393	14.89561066	42.80805445	-1.522995
Zfp518b	Zfp518b-201	1717.3045	0.005298831	0.016776868	0.348037141	-2.873256563	5.504914762	15.81703247	-1.522687
Emc3	Emc3-001	8676.2125	2.53758E-06	6.33106E-05	0.348038188	-2.873247922	22.76576768	65.41169468	-1.522682
Gbf1	Gbf1-002	4969.2562	0.001156578	0.005108031	0.348147686	-2.872344237	16.69981723	47.96762378	-1.522229
Rhoa	Rhoa-005	1773.2677	0.000667423	0.003340039	0.348181863	-2.872062291	3.882255036	11.15007829	-1.522087
Nsun6	Nsun6-001	822.26991	0.047786292	0.094687909	0.348243739	-2.871551987	1.988369817	5.709707298	-1.521831
Timp3	Timp3-002	5357.7728	0.001658816	0.006755582	0.348292308	-2.871151551	8.463409176	24.29973039	-1.521629
Rpl36-ps2	Rpl36-ps2-001	950	0.001542509	0.006386729	0.348312339	-2.870986435	3.47119675	9.965758783	-1.521547
230319C09R	0319C09Rik-	447.31211	0.012291001	0.032389536	0.348353985	-2.870643208	0.973916946	2.795768066	-1.521374
Cers2	Cers2-001	13259.953	3.48014E-07	1.88161E-05	0.348401891	-2.870248486	35.33345292	101.4157898	-1.521176
Pofut1	Pofut1-005	1716.0632	0.017666152	0.043068022	0.348413774	-2.870150597	4.544672166	13.04389353	-1.521126
Zfp9	Zfp9-001	5723	2.34766E-06	6.01687E-05	0.348431847	-2.870001716	19.26367897	55.28679169	-1.521052
Fn1	Fn1-015	516.39102	0.044245047	0.089031245	0.348470858	-2.869680421	2.337315058	6.707347261	-1.52089
Naip5	Naip5-001	2157	3.01198E-05	0.000335809	0.348510651	-2.869352768	7.22348496	20.72672656	-1.520725
Cizl	Cizl-011	525.22288	0.008319796	0.023825983	0.348547394	-2.869050288	1.594866189	4.5757513	-1.520573
Fndc3a	Fndc3a-005	4997.9573	4.92986E-06	9.68163E-05	0.348592846	-2.868676196	14.24117512	40.85332007	-1.520385
Tspan3	Tspan3-201	3873	1.08043E-05	0.000163376	0.348596903	-2.868642811	11.64985502	33.41927285	-1.520368
Kmt2c	Kmt2c-005	1568.9092	0.000705823	0.003486287	0.34861755	-2.868472911	4.778319608	13.70648036	-1.520283
Ehmt1	Ehmt1-201	579.50805	0.048395652	0.09562427	0.348617646	-2.868472126	1.462205525	4.194295792	-1.520282
Wars2	Wars2-001	2731.8465	0.000400497	0.002252016	0.34863407	-2.868336989	7.405604854	21.24177033	-1.520215
Gm38387	Gm38387-001	821	0.000259882	0.001621302	0.348674847	-2.868001545	2.305748033	6.612888922	-1.520046
Rps6kc1	Rps6kc1-004	850.94967	0.013062619	0.033996558	0.348707017	-2.867736954	2.559467766	7.339880295	-1.519913
32438A13R	2438A13Rik-	826.84792	0.006853384	0.020466337	0.348741088	-2.86745679	2.404146926	6.893787429	-1.519772
Rnf38	Rnf38-001	1595.01	0.008225928	0.023607844	0.348760469	-2.867297441	5.695646682	16.33111316	-1.519692
Enpp6	Enpp6-001	26019.084	0.000158121	0.001122413	0.348849584	-2.866564977	63.70990133	182.6285718	-1.519323
Thnsl1	Thnsl1-001	699.11379	0.009949659	0.027464202	0.348915358	-2.866024601	2.504885508	7.17906349	-1.519051
Mtrf1l	Mtrf1l-003	645.56171	0.049299209	0.096962094	0.348931989	-2.865888001	1.882615727	5.395365824	-1.518982
Becn1	Becn1-002	3416.2043	0.007247027	0.021417781	0.349005243	-2.865286469	8.321735479	23.84415606	-1.518679
30309D02R	0309D02Rik-	634.37379	0.022527951	0.052123374	0.349025108	-2.86512339	1.61059767	4.614561056	-1.518597
Mmp17	Mmp17-001	758.96447	0.000796038	0.003815322	0.349047267	-2.864941496	2.209203854	6.329239794	-1.518506
Git2	Git2-015	11515.387	2.28601E-06	5.93809E-05	0.349080293	-2.86467045	37.63399795	107.8090019	-1.518369
00009A05R	0009A05Rik-	297.53566	0.047891188	0.094832303	0.349162219	-2.863998294	0.827895875	2.371092374	-1.518031
P23-167H23	3-167H23.2-	1811	5.93095E-05	0.000546503	0.349165943	-2.863967747	5.219622219	14.94882968	-1.518015
Osbpl8	Osbpl8-201	6458.251	0.00019606	0.001312669	0.349373842	-2.862263514	15.4720989	44.28522417	-1.517156
Psmb5	Psmb5-201	2884	0.000670303	0.003348094	0.349427377	-2.861824989	9.214586543	26.37053403	-1.516935
Eif4g1	Eif4g1-010	3488.8088	0.003485453	0.012059478	0.349564635	-2.860701281	8.414561283	24.07154624	-1.516369
Parp8	Parp8-201	10400	5.87258E-06	0.000108083	0.349574002	-2.860624633	33.95202618	97.12400243	-1.51633
Sptbn1	Sptbn1-003	6025.693	0.000946977	0.004365272	0.349681885	-2.859742079	11.00467282	31.47052593	-1.515885
920009B18R	0009B18Rik-	459.48163	0.008489596	0.024208531	0.349709367	-2.859517341	1.617435808	4.625085741	-1.515772
Abi2	Abi2-001	8259.9394	6.9259E-06	0.00061311	0.34971162	-2.859498921	27.4536291	78.50360563	-1.515762
B4galt6	B4galt6-201	3051	0.002006733	0.007851335	0.349822892	-2.858589367	11.2519845	32.16480325	-1.515303
930095G15R	0095G15Rik-	2216	0.001892571	0.007495862	0.349829379	-2.858536358	6.576971556	18.80051232	-1.515277
Ptch1	Ptch1-005	12031.516	0.035422786	0.074577724	0.349848613	-2.858379201	39.33494018	112.4341749	-1.515197
Cs	Cs-201	14004	0.000401282	0.00225495	0.349937547	-2.857652771	44.77500287	127.951411	-1.514831
Ifi203	Ifi203-003	852.12995	0.012480329	0.032785034	0.349995409	-2.857180337	2.506306417	7.160969413	-1.514592
Suclg2	Suclg2-201	2601	0.000331145	0.001949421	0.350031523	-2.856885548	9.077529417	25.9334626	-1.514443
Gm20655	Gm20655-002	3125.341	0.026582843	0.05928752	0.350091329	-2.856397512	8.731131063	24.93958104	-1.514197
Tpm1	Tpm1-005	2516.4282	0.006085949	0.018672737	0.350201814	-2.855496343	8.461813784	24.16267831	-1.513742
Mapkapk5	Mapkapk5-01	720.34558	0.018959537	0.045560838	0.350248721	-2.85511392	2.502188183	7.14403231	-1.513548
Gm6285	Gm6285-001	280	0.048878908	0.096368182	0.350288163	-2.854792439	1.042514378	2.976162164	-1.513386
Ap1s2	Ap1s2-002	1622.5315	0.00501247	0.016032601	0.350295275	-2.854734478	4.848114635	13.84008	-1.513357
P24-187P11	24-187P11.2-(	399	0.003996223	0.013409487	0.350311007	-2.854606276	1.178105368	3.363026978	-1.513292
Ifrd1	Ifrd1-007	1051.0354	0.002416229	0.009078085	0.350319134	-2.854540059	4.850641181	13.84634956	-1.513258
Cbfa2t3	Cbfa2t3-001	18855.037	0.002498213	0.009310532	0.35034994	-2.854289054	40.4283846	115.3942956	-1.513131
Prom1	Prom1-015	655.05087	0.005545407	0.0173653	0.350379961	-2.854044495	1.590955452	4.540657648	-1.513008
Pofut1	Pofut1-003	506.54626	0.013996117	0.035891509	0.350385603	-2.853998543	1.427830016	4.075024787	-1.512985
Gm26531	Gm26531-201	470	0.015476463	0.038810136	0.3503876	-2.853982279	1.346753824	3.843611548	-1.512976
Snrnp40	Snrnp40-001	1631.7078	0.00043333	0.002394183	0.350433428	-2.853609046	6.258849331	17.86030907	-1.512788
Dnm2	Dnm2-005	2531.3212	0.002327513	0.008821593	0.35044097	-2.853547634	9.000473057	25.6832786	-1.512757
Nbea	Nbea-009	737.36522	0.012195909	0.032210817	0.350462448	-2.853372753	1.688916249	4.819107609	-1.512668
Atxn1	Atxn1-202	9503.7181	0.000521911	0.002760299	0.35047046	-2.853307525	33.98108222	96.95847763	-1.512635
F2r	F2r-201	42563	6.45212E-05	0.000581539	0.350472749	-2.853288886	133.6832413	381.4369067	-1.512626
Gm4767	Gm4767-001	731.58087	0.0031886	0.011277319	0.350494861	-2.853108879	2.223839915	6.344857406	-1.512535
Tpm3	Tpm3-004	5147.7641	0.000106598	0.000834979	0.350553323	-2.852633063	13.18058683	37.59937777	-1.512294
Riok1	Riok1-201	3302	1.09677E-05	0.000164816	0.350574218	-2.852463045	10.91546802	31.13596914	-1.512208
Kank3	Kank3-005	990.77482	0.003194318	0.011294044	0.350606528	-2.85220017	1.403512224	4.003097804	-1.512075
Nceh1	Nceh1-001	3268.6655	0.000232143	0.001490817	0.350685117	-2.851560988	11.28138748	32.16956442	-1.511752

Ylpm1	Ylpm1-003	1001.3926	0.001183852	0.005202992	0.35068905	-2.851529015	2.783969341	7.93856935	-1.511736
Syncrip	Syncrip-001	1474.4572	0.037608186	0.078241029	0.350777757	-2.8508079	5.282092854	15.05823203	-1.511371
Tmem70	Tmem70-002	1571.5415	0.008227612	0.023607844	0.350859312	-2.850145248	4.382643853	12.49117155	-1.511035
Prl8a1	Prl8a1-001	6189	6.17038E-06	0.000111995	0.350881834	-2.849962302	18.11071816	51.61486403	-1.510943
Nek4	Nek4-201	630	0.001411893	0.005971518	0.350906786	-2.84975965	2.376788365	6.773275579	-1.51084
Vma21	Vma21-001	1607.2247	0.000201457	0.00134083	0.350944938	-2.849449847	4.706694746	13.41149062	-1.510683
Qars	Qars-005	1288.4722	0.000143674	0.00104736	0.350951448	-2.849396991	3.847030308	10.96171659	-1.510657
Zbtb14	Zbtb14-003	1146.7404	0.028781198	0.063124746	0.350973624	-2.849216956	3.421199437	9.747739445	-1.510565
Ash2l	Ash2l-004	1273.9837	0.006661288	0.020005151	0.350996423	-2.84903188	4.208505658	11.99016679	-1.510472
Whsc1l1	Whsc1l1-005	4837.1356	0.001053326	0.00474128	0.351002312	-2.848984086	13.41352984	38.21493306	-1.510448
Tnrc6b	Tnrc6b-201	34967	3.17556E-05	0.00034871	0.351009423	-2.848926363	106.9880326	304.8010267	-1.510418
Lpar6	Lpar6-001	6295.4946	9.50177E-05	0.000769169	0.351046319	-2.848626936	23.41446939	66.6990882	-1.510267
Plxna4	Plxna4-002	569.94235	0.032417953	0.069494579	0.351081201	-2.848343907	1.959463445	5.581225764	-1.510123
30112E08R	0112E08Rik-	774.05663	0.049968043	0.097903771	0.351093897	-2.848240908	2.466973915	7.026536025	-1.510071
Pggt1b	Pggt1b-201	5085	1.31674E-06	4.14888E-05	0.351161147	-2.847695448	13.28418519	37.82931369	-1.509795
Katnal1	Katnal1-004	477.41779	0.007201888	0.021302761	0.351170962	-2.847615857	1.637975786	4.664325821	-1.509755
Ncor1	Ncor1-010	2953.989	5.52437E-05	0.000518021	0.351177619	-2.847561876	8.721642661	24.83541714	-1.509727
Rreb1	Rreb1-001	2502.6046	0.015039242	0.037941618	0.351181642	-2.847529257	8.10030048	23.0658426	-1.509711
R3hdm2	R3hdm2-005	883.84261	0.011841374	0.031488126	0.351198314	-2.847394076	2.030022858	5.780275058	-1.509642
Zfp512	Zfp512-007	1680.4002	0.000495274	0.002652564	0.35133798	-2.846262162	4.817862221	13.71289894	-1.509069
Iffo1	Iffo1-008	4219.3646	1.46402E-05	0.000201862	0.351370176	-2.846001367	11.77827288	33.52098072	-1.508936
Rev3l	Rev3l-201	7111.3858	0.014417055	0.03674536	0.351466622	-2.845220395	20.34358302	57.88197732	-1.50854
Gusb	Gusb-006	2590.494	0.002838317	0.010294354	0.351527109	-2.844730821	9.941363863	28.28050418	-1.508292
Vdac3	Vdac3-201	4831.5041	0.000153658	0.001100773	0.351597027	-2.84416512	14.0690511	40.01470441	-1.508005
Mef2a	Mef2a-005	2424.719	0.000588042	0.003028133	0.351632509	-2.843878127	6.54023187	18.59962236	-1.50786
Rpl9-ps4	Rpl9-ps4-201	1880	2.83479E-05	0.000322029	0.351739461	-2.843013398	6.481572035	18.42719614	-1.507421
Phf3	Phf3-004	521.08646	0.004215489	0.013995784	0.351743285	-2.84298249	1.347828134	3.831851785	-1.507405
Gm26600	Gm26600-201	168	0.026061279	0.058389403	0.351769174	-2.842773259	0.522730226	1.48600351	-1.507299
Tada1	Tada1-001	1234.3406	0.014097563	0.036100301	0.351788523	-2.842616895	3.832555097	10.89448587	-1.50722
Snhg14	Snhg14-015	477	0.009851957	0.027240617	0.351835318	-2.842238823	1.397482792	3.971979845	-1.507028
Mllt3	Mllt3-007	1734.9523	0.030754257	0.066683547	0.351888126	-2.841812289	5.845761462	16.61255676	-1.506811
Rgs3	Rgs3-008	4815.4272	0.000119165	0.000911348	0.351920971	-2.841547055	13.50432845	38.37318474	-1.506677
30405O15R	0405O15Rik-	930.49809	0.000531043	0.002799049	0.351948705	-2.841323143	2.601200343	7.390850734	-1.506563
Aig1	Aig1-004	605.61631	0.025358311	0.057128341	0.352078715	-2.840273945	2.359602889	6.701918606	-1.50603
Gm37626	Gm37626-001	2593	0.035667593	0.074973307	0.352146838	-2.839724488	7.448870563	21.15274015	-1.505751
Snx12	Snx12-002	9014.2284	0.000105803	0.000830565	0.352263397	-2.838784866	26.70878859	75.82050484	-1.505274
Fancm	Fancm-201	1370	0.0199999	0.047507636	0.352314365	-2.838374191	4.418620621	12.54169873	-1.505065
Tomm70a	Tomm70a-201	6693	0.000110577	0.000859954	0.35232484	-2.838289802	23.72207132	67.3301131	-1.505022
Pcdhb18	Pcdhb18-001	825	0.015058817	0.037980231	0.35236457	-2.837969775	2.561513276	7.269497255	-1.504859
Carf	Carf-009	686.0188	0.00703886	0.020919903	0.352393603	-2.837735962	1.818913636	5.161596637	-1.50474
Nsmce1	Nsmce1-001	2100.9732	0.006692182	0.020081238	0.352411575	-2.837591243	6.414781855	18.20252881	-1.504667
Mir99ahg	Mir99ahg-002	828.157	0.025564245	0.057485545	0.352431374	-2.837431833	2.278623482	6.465438803	-1.504586
Yme1l1	Yme1l1-001	10991.412	7.25669E-07	2.88331E-05	0.35250318	-2.83685384	34.57117839	98.07338019	-1.504292
Gnb2l1	Gnb2l1-004	11287.906	8.33205E-05	0.000699405	0.352582593	-2.83621489	41.11818484	116.6200081	-1.503967
Capza2	Capza2-001	12627.87	5.15244E-05	0.000492111	0.352597701	-2.836093366	39.42737587	111.8197191	-1.503905
Tspan12	Tspan12-001	4446.083	0.000312368	0.00186172	0.35272207	-2.835093361	10.42985416	29.56961028	-1.503396
Rer1	Rer1-002	3625.308	0.01050103	0.028667326	0.352899379	-2.833668915	12.98662984	36.7998093	-1.502671
Atp8b2	Atp8b2-001	1951.6234	0.012189419	0.032198644	0.353032639	-2.83259928	3.226972414	9.140719738	-1.502127
Gja5	Gja5-001	140762.6	2.45195E-06	6.18861E-05	0.353090658	-2.832133836	245.9657686	696.6079756	-1.501889
Zfp113	Zfp113-001	4710.6455	3.29577E-05	0.000357658	0.353147035	-2.831681707	13.24962767	37.51872831	-1.501659
P23-286M3	23-286M3.5-0	629.79701	0.028037483	0.061876231	0.353203296	-2.831230658	2.152381483	6.093888442	-1.501429
P23-60E24.	23-60E24.3-0	2098	8.08829E-05	0.000687233	0.353223559	-2.83106824	6.378460881	18.05785802	-1.501347
Fbxl3	Fbxl3-006	1634.4619	4.42019E-05	0.000441503	0.353233889	-2.830985447	4.78466288	13.54474479	-1.501304
Harbi1	Harbi1-001	596.57224	0.01801232	0.043712223	0.35326533	-2.830733487	1.732923829	4.905445514	-1.501176
Ceacam1	Ceacam1-201	4585.3133	0.003181405	0.011257522	0.353277334	-2.830637302	11.38867788	32.23721643	-1.501127
Mllt4	Mllt4-003	1425.0496	0.001289218	0.005565116	0.353295848	-2.83048897	5.016547009	14.19928097	-1.501051
Wdr75	Wdr75-001	1445.9621	0.031162187	0.067384614	0.35337125	-2.829884998	5.396759393	15.27220844	-1.500743
Gm11407	Gm11407-001	66758	1.56326E-05	0.000211228	0.353482292	-2.828996031	203.6623913	576.1600966	-1.50029
Gm19557	Gm19557-001	1136.9857	0.011862639	0.031530608	0.353494725	-2.828896517	3.404429725	9.63077939	-1.500239
Gm15536	Gm15536-001	175	0.032264394	0.069226053	0.353509065	-2.828781775	0.587146821	1.660910228	-1.500181
P23-97A19	23-97A19.1-0	921	0.000632759	0.003205917	0.353529638	-2.828617161	1.558819769	4.40930435	-1.500097
Heatr1	Heatr1-201	5937	0.003803357	0.01290391	0.353558228	-2.828388426	24.29598727	68.71848921	-1.49998
Gm14419	Gm14419-001	569.87947	0.004326762	0.014282023	0.353576333	-2.828243594	1.823583587	5.157538598	-1.499906
Clcc1	Clcc1-002	4379.4217	1.94278E-05	0.000247085	0.353633591	-2.82778567	14.54219326	41.12220572	-1.499673
Chn1	Chn1-001	2563.8713	0.0009158	0.004256357	0.353637004	-2.827758375	5.274998976	14.91642253	-1.499659
Gm10030	Gm10030-001	692.52579	0.006164494	0.018846195	0.353643521	-2.827706264	2.160498633	6.109255518	-1.499632
Kif1b	Kif1b-007	8230.5114	9.33928E-05	0.000758885	0.353649685	-2.827656976	22.44669762	63.4715611	-1.499607
Ssr2	Ssr2-001	2528.2078	0.00742892	0.021835033	0.353724903	-2.827055689	8.461504062	23.9211432	-1.4993
Rab18	Rab18-201	11503	3.56359E-07	1.9041E-05	0.353837212	-2.826158374	31.66612625	89.49348788	-1.498842
32438A13R	2438A13Rik-	2596.4046	8.44822E-05	0.000706598	0.354012933	-2.824755556	7.260199275	20.50828824	-1.498126
Cntrl	Cntrl-008	562.27109	0.020602708	0.048628128	0.354020212	-2.824697479	1.730084356	4.886964918	-1.498096
Dnajc18	Dnajc18-201	8468	1.01249E-05	0.000155776	0.354052967	-2.824436155	23.56129963	66.54738654	-1.497963
Rab7	Rab7-001	18930.067	2.49027E-05	0.000294554	0.354080295	-2.824218159	55.64426969	157.1515569	-1.497852
Zfr	Zfr-005	1591.2776	0.016793848	0.041384409	0.354124708	-2.823863958	5.030441975	14.20528379	-1.497671
Adgre5	Adgre5-201	3425.941	0.000254993	0.001597803	0.354216451	-2.823132568	9.221670115	26.03399723	-1.497297
Arpc2	Arpc2-003	3998.3772	0.011294034	0.030322228	0.354276248	-2.822656067	12.80880298	36.15484543	-1.497053
Fam178a	Fam178a-201	18327	1.48775E-06	4.46816E-05	0.354308092	-2.822402375	54.98652288	155.1940928	-1.496924
Mtmr10	Mtmr10-002	1462.1604	0.001168896	0.005150479	0.354310506	-2.822383146	4.918080971	13.88070884	-1.496914
Ttbk2	Ttbk2-001	2056.6262	0.011094235	0.029893601	0.3543278	-2.822245388	5.582958818	15.75647978	-1.496843
Ikbkb	Ikbkb-005	951.44395	0.003855805	0.013039229	0.354453885	-2.821241469	3.19570139	9.015845283	-1.49633
Usp42	Usp42-002	1100.5562	0.017909631	0.043522411	0.354465717	-2.821147295	3.441977139	9.710324495	-1.496282
Ldb2	Ldb2-002	3884.3696	0.041033843	0.083834951	0.354671714	-2.819508748	8.841974702	24.93002502	-1.495444
Gm16897	Gm16897-001	1869.6618	0.002140291	0.008248562	0.35487097	-2.817925626	4.047045082	11.40427205	-1.494634
Smo	Smo-002	1073.0118	0.005828782	0.018032318	0.35488027	-2.817851778	3.288455863	9.266381202	-1.494596
Swi5	Swi5-002	3001.3264	4.88894E-05	0.000472878	0.354893747	-2.817744772	9.248864184	26.0609387	-1.494541
Gm37250	Gm37250-001	351	0.022073795	0.051293057	0.354927762	-2.817474727	1.026547704	2.892272213	-1.494403
Rnft1	Rnft1-001	3590.8303	0.000351662	0.002039354	0.354987137	-2.817003477	10.86280516	30.6005599	-1.494161
Mbtps2	Mbtps2-001	2412.7434	0.000167584	0.001171141	0.355044074	-2.816551725	7.367556454	20.75110384	-1.49393
Gm37886	Gm37886-001	529	0.002581061	0.009550707	0.355061217	-2.81641574	1.709017999	4.813305191	-1.49386
Fhl1	Fhl1-004	20083.794	0.03801397	0.078912568	0.355104067	-2.816075882	38.01983943	107.0667528	-1.493686
RP24-285I4.	24-285I4.2-0	16779	1.08464E-06	3.68542E-05	0.355106608	-2.816055738	49.40954358	139.1400287	-1.493676
Gm14410	Gm14410-002	780.90746	0.004234525	0.01404537	0.355141737	-2.815777182	2.212708803	6.230494958	-1.493533
Gm26786	Gm26786-201	2259.9099	0.003605854	0.012384727	0.35517406	-2.815520932	6.646852145	18.71435134	-1.493402
Uri1	Uri1-001	4592	7.52953E-05	0.000650506	0.355211216	-2.815226424	13.29467245	37.42751319	-1.493251
Alg5	Alg5-001	2436.9467	0.002042462	0.007959305	0.355217849	-2.815173848	8.208960256	23.10965023	-1.493224
Pard3b	Pard3b-007	714.63497	0.013189928	0.034267998	0.355233333	-2.815051144	2.169884337	6.108335385	-1.493161
Gm17546	Gm17546-201	538	0.014338399	0.036577549	0.355278979	-2.814689469	1.513595868	4.26030235	-1.492976
Cenpj	Cenpj-001	2981	0.000128827	0.00096555	0.355335607	-2.814240905	9.074115983	25.53674838	-1.492746
Cwc22	Cwc22-011	3447.4279	0.00069666	0.003453053	0.35535282	-2.814104585	11.21312573	31.55490852	-1.492676
Svil	Svil-001	6823.2906	0.002350918	0.00888436	0.355425136	-2.813532015	16.25547397	45.73529645	-1.492382
Zkscan3	Zkscan3-003	793.19544	0.003930811	0.013237983	0.35542967	-2.813496125	1.738240546	4.890533041	-1.492364
Gm37673	Gm37673-001	913	0.004816235	0.015531124	0.355432983	-2.8134699	2.868894618	8.071548654	-1.492351
Trim52	Trim52-002	315	0.019760248	0.047067842	0.355449813	-2.813336688	0.923945111	2.59936868	-1.492282
Zc3h14	Zc3h14-203	1060.3061	0.044194889	0.088950751	0.35549453	-2.812982804	2.367423014	6.659520228	-1.492101
Dnajc2	Dnajc2-004	867.09888	0.032614706	0.06985078	0.355530352	-2.812699382	3.205210002	9.01529219	-1.491955
RP23-30B3.	23-30B3.2-0	648	0.001978692	0.007769566	0.355538234	-2.812637025	2.013850461	5.664230369	-1.491923
Ubfd1	Ubfd1-002	903.21711	0.019744023	0.047035738	0.355660321	-2.811671532	2.877971641	8.091910934	-1.491428

Dstyk	Dstyk-001	4281.1216	1.55626E-05	0.000210782	0.355727074	-2.811143915	11.8908691	33.42694433	-1.491157
Llph	Llph-001	4688.53	2.37375E-05	0.000284181	0.355732199	-2.811103416	16.36591837	46.00628905	-1.491137
Glrx3	Glrx3-201	3834	1.83929E-05	0.000237849	0.355754265	-2.810929056	13.02635397	36.61615687	-1.491047
Commd4	Commd4-201	1183	0.005237837	0.016609196	0.355818411	-2.810422309	3.879910197	10.90418617	-1.490787
Fign	Fign-001	1809.7521	0.006842629	0.02044848	0.355841851	-2.810237181	5.058263777	14.21492093	-1.490692
Ndrg2	Ndrg2-202	3033.7567	0.008190248	0.023529274	0.356019241	-2.80883695	11.30680766	31.75897913	-1.489973
Psmc2	Psmc2-002	10345.353	5.97614E-07	2.56174E-05	0.356067935	-2.808452834	30.28668246	85.0587192	-1.489776
Gapvd1	Gapvd1-005	3249.6669	7.59462E-05	0.000655534	0.356081852	-2.808343065	9.601202407	26.96347019	-1.489719
Gm31258	Gm31258-001	6436	5.96582E-06	0.00010915	0.356108906	-2.808129713	18.52898417	52.031791	-1.48961
Slc7a1	Slc7a1-006	421	0.013612714	0.035105447	0.35613629	-2.807913792	1.384963773	3.888858881	-1.489499
Slc12a6	Slc12a6-006	10893.138	2.36561E-05	0.00028379	0.356204207	-2.807378407	31.17073211	87.50804024	-1.489224
Def8	Def8-004	332.17857	0.028115335	0.062001434	0.356245753	-2.807051004	1.163397459	3.265716005	-1.489055
Mgat5	Mgat5-001	13974.852	0.000621371	0.003161781	0.356276014	-2.806812587	43.27247759	121.4577347	-1.488933
Ctnnd1	Ctnnd1-201	13860.135	0.005834464	0.018043636	0.356288989	-2.806710366	38.4334749	107.8716324	-1.48888
Ltbp4	Ltbp4-201	29479.759	0.024621024	0.055823662	0.35630133	-2.806613152	69.80911387	195.9271771	-1.48883
Gm37534	Gm37534-001	618	0.008356567	0.023904822	0.356323152	-2.806441269	2.250192846	6.315034066	-1.488742
Tspan18	Tspan18-004	2789.2765	1.62461E-05	0.000216931	0.356350083	-2.806229174	9.253676679	25.96793746	-1.488633
Psip1	Psip1-005	742.31325	0.00917693	0.025773665	0.356361785	-2.806137028	2.201179938	6.176812531	-1.488585
Actr6	Actr6-201	838	0.006394952	0.019374358	0.356364404	-2.806116401	2.487400117	6.979934263	-1.488575
Mkln1	Mkln1-007	2372.1129	0.000139586	0.001027248	0.356404055	-2.805804217	6.704095416	18.81037919	-1.488414
Nktr	Nktr-016	277.09168	0.02247715	0.052026923	0.356496796	-2.805074298	1.002165506	2.811148702	-1.488039
Gas7	Gas7-201	1952.9679	0.038331028	0.079440743	0.356647405	-2.803889739	8.801029857	24.67711731	-1.48743
Fubp1	Fubp1-008	7557.5458	0.000821091	0.003908055	0.356660493	-2.803786849	26.04003397	73.01070481	-1.487377
Gnaq	Gnaq-002	3152.4979	0.004263081	0.014121299	0.356682315	-2.803615312	7.419573802	20.80163072	-1.487288
Mgl2	Mgl2-001	307.59192	0.038411763	0.079559923	0.356684613	-2.803597247	1.106211113	3.101370432	-1.487279
Slc50a1	Slc50a1-002	902.18807	0.026507405	0.059168314	0.356706553	-2.803424805	1.5175034	4.254206673	-1.48719
Arpc1b	Arpc1b-001	9722.6832	8.21256E-05	0.000693325	0.356714875	-2.803359409	26.44366614	74.13110029	-1.487157
Topbp1	Topbp1-002	3678.5285	4.5779E-05	0.000452506	0.356721493	-2.803307395	11.4191536	32.01139772	-1.48713
Atox1	Atox1-001	3845.8178	0.000393864	0.002225315	0.356805837	-2.802644732	10.2122762	28.62138211	-1.486789
Micu3	Micu3-003	489.34851	0.026564817	0.059253989	0.356887517	-2.802003299	1.415891644	3.967333057	-1.486459
Gm5570	Gm5570-001	237	0.038237146	0.079284552	0.356912974	-2.801803445	0.578056625	1.619601042	-1.486356
Gm26586	Gm26586-201	804.72549	0.003054427	0.010902983	0.356994216	-2.801165834	2.315772677	6.486863301	-1.486027
Pcdhga3	Pcdhga3-001	2732.3313	1.86E-05	0.000239402	0.357054916	-2.800689625	6.261364392	17.53613829	-1.485782
Prmt10	Prmt10-002	1112.6163	0.002639943	0.0097276	0.3570792	-2.800499162	3.580805847	10.02804378	-1.485684
Gpatch8	Gpatch8-001	3656.5117	0.007974111	0.023072847	0.35711903	-2.800186821	10.41741368	29.17070451	-1.485523
Myo9a	Myo9a-001	7389.4697	0.003973221	0.013349696	0.357171544	-2.799775116	18.10264463	50.68333398	-1.485311
Snupn	Snupn-001	1050	0.000255093	0.001598119	0.357313883	-2.798659796	3.074405564	8.604215247	-1.484736
Gm4518	Gm4518-001	347	0.023854399	0.054463915	0.357314735	-2.798653127	1.220685996	3.416276679	-1.484733
N6amt1	N6amt1-003	692.89534	0.010065504	0.027734795	0.357437288	-2.797693567	2.409917978	6.742212025	-1.484238
Fam227a	Fam227a-202	525.39391	0.006116356	0.018748881	0.357459564	-2.79751922	1.629922094	4.559738386	-1.484148
Zmpste24	Zmpste24-001	12324.933	1.49137E-05	0.000204575	0.35754045	-2.796886336	35.14373017	98.29301873	-1.483822
Psap	Psap-203	2586.2885	0.01734496	0.042416805	0.35761744	-2.796284212	7.456419834	20.85026906	-1.483511
31439G07R	31439G07Rik-	7970.9059	4.29009E-05	0.000432164	0.357643655	-2.796079246	26.28922642	73.5067604	-1.483405
P24-496C22	24-496C22.1-	1360.3847	0.000789683	0.003789106	0.357668979	-2.795881273	3.404480744	9.518523956	-1.483303
Vcam1	Vcam1-001	83779.373	1.62687E-05	0.000217064	0.357781183	-2.795004451	307.6970628	860.0146603	-1.482851
AU019823	AU019823-00	766.42637	0.003869188	0.013076022	0.357792661	-2.794914789	2.212511598	6.183781386	-1.482804
Eln	Eln-004	14930.029	0.003179278	0.01125132	0.357818661	-2.794711708	21.39103644	59.78177998	-1.482699
Patl1	Patl1-201	8777	4.16566E-06	8.67847E-05	0.357894254	-2.794121418	28.17051945	78.71185175	-1.482395
Gm16023	Gm16023-001	545.64998	0.04680908	0.093105717	0.357900995	-2.79406879	1.809167284	5.054937844	-1.482368
Gm12966	Gm12966-001	958	0.001513757	0.006294562	0.35793081	-2.793836048	3.157571287	8.821736486	-1.482247
Notch4	Notch4-006	972.85877	0.001033897	0.004672864	0.357943872	-2.793734094	2.540941637	7.098715282	-1.482195
Eif3i	Eif3i-001	10445.458	1.44926E-05	0.000200392	0.358038399	-2.792996516	36.97233152	103.2635931	-1.481814
Gm7299	Gm7299-001	590	0.001557766	0.006437437	0.358071481	-2.792738469	2.066626274	5.771546697	-1.48168
Acvr2a	Acvr2a-001	3465.4575	1.27579E-05	0.000183211	0.358099561	-2.792519483	10.85528701	30.31360047	-1.481567
Gm37060	Gm37060-001	617	0.015849344	0.039531025	0.35811284	-2.792415934	2.153595647	6.0137348	-1.481514
Cul4b	Cul4b-001	2241.5123	0.045009708	0.090172117	0.358141472	-2.79219269	8.612185392	24.0468811	-1.481399
Loxl2	Loxl2-001	2721.4383	0.002570601	0.00952126	0.358155109	-2.792086377	9.536938786	26.62795687	-1.481344
Gm37212	Gm37212-001	318	0.021197904	0.049717743	0.358168904	-2.79197884	1.04527974	2.918398915	-1.481288
Hspb6	Hspb6-003	1276.3673	0.004593386	0.014970509	0.358178081	-2.7919073	4.827205855	13.47711127	-1.481251
Pdcl3	Pdcl3-003	2343.6072	0.000100267	0.000798674	0.358202585	-2.791716312	7.822270247	21.83755944	-1.481152
Mynn	Mynn-003	2757.0769	0.007510646	0.022027712	0.358310229	-2.790877625	7.243341662	20.21528017	-1.480719
Adat1	Adat1-003	2328.6012	2.83276E-05	0.000322012	0.358326118	-2.790753871	7.447907009	20.78527532	-1.480655
Abcb7	Abcb7-001	4670.1687	8.33739E-06	0.000136891	0.358337455	-2.790665575	15.03071605	41.94570184	-1.480609
Arid4b	Arid4b-003	405.92828	0.026444452	0.059062437	0.358388747	-2.790266182	1.505312413	4.200222319	-1.480403
Gm26816	Gm26816-201	974.84274	0.005254841	0.016653865	0.358414943	-2.790062247	3.089604565	8.620189055	-1.480297
Nhsl2	Nhsl2-003	101745.34	3.05056E-05	0.000338787	0.358461303	-2.789701401	223.8563731	624.4924376	-1.480111
Gm26825	Gm26825-201	13178.673	0.001369039	0.00582688	0.358520097	-2.789243918	48.85664364	136.2730961	-1.479874
Idh3g	Idh3g-001	4019.9945	9.57375E-05	0.000773142	0.358538035	-2.789104372	11.89740205	33.18309609	-1.479802
Wdr37	Wdr37-002	3939.3626	1.99832E-05	0.000252183	0.358551024	-2.789003329	12.26761173	34.21440995	-1.47975
Utp14a	Utp14a-001	3070.6318	4.54014E-05	0.000449811	0.358632962	-2.788366118	11.72248326	32.68657512	-1.47942
Tmem201	Tmem201-001	1292.6183	0.006762461	0.020253087	0.358647623	-2.788252131	3.864033993	10.77390102	-1.479361
Entpd4	Entpd4-003	1003.89	0.004369906	0.014394044	0.358656015	-2.788186896	3.021232287	8.423760273	-1.479327
Xiap	Xiap-001	2070.7904	0.032017197	0.068799109	0.358683174	-2.787975776	5.822122697	16.23193705	-1.479218
Gls	Gls-005	3368.6694	0.000598217	0.003068582	0.358730959	-2.787604398	10.98975155	30.63507974	-1.479026
Egln1	Egln1-201	5853	2.76677E-06	6.68279E-05	0.358783615	-2.787195286	17.28693533	48.18206466	-1.478814
Mrps10	Mrps10-003	1505.7844	0.000295194	0.001786577	0.358807619	-2.78700882	4.965625897	13.83924317	-1.478718
430025E21R	0025E21Rik-	7357	5.0541E-06	9.84098E-05	0.358809825	-2.786991687	24.24788619	67.57865724	-1.478709
Mphosph6	Mphosph6-20	1702	0.000703095	0.0034769	0.358838312	-2.786770439	5.574331069	15.53438104	-1.478594
10013D15R	0013D15Rik-	1040.8933	0.002682995	0.009845984	0.358886618	-2.786395336	2.844689243	7.92642884	-1.4784
Minpp1	Minpp1-201	3133	0.00057449	0.002977644	0.35888917	-2.786375525	9.274498396	25.84223534	-1.47839
630011O06R	0011O06Rik-	3435.7865	8.1101E-05	0.000688233	0.358914179	-2.786181371	12.58774172	35.07173147	-1.478289
Atp13a3	Atp13a3-002	602.52399	0.012610447	0.033053326	0.35893439	-2.786024486	1.757590398	4.896689886	-1.478208
Gm38126	Gm38126-001	492	0.031421371	0.067795261	0.359031608	-2.785270095	1.645801118	4.584000635	-1.477817
Gjc3	Gjc3-001	1096	0.00039219	0.002218107	0.359041993	-2.785189534	2.931154255	8.163820154	-1.477776
Exosc7	Exosc7-001	3102.1715	0.000906719	0.004225911	0.359055656	-2.785083546	8.299103099	23.11369549	-1.477721
Ifitm3	Ifitm3-001	48457	2.37519E-05	0.000284181	0.359061291	-2.785039837	151.2576224	421.258504	-1.477698
Eya3	Eya3-001	3092.3991	0.000118335	0.000906504	0.359082462	-2.784875634	7.368217802	20.51957022	-1.477613
Ndufab1	Ndufab1-001	1461.2871	5.21269E-05	0.000496188	0.359130573	-2.784502557	4.447940858	12.38530269	-1.47742
Nln	Nln-201	2206	0.00331699	0.011617438	0.359135016	-2.784468114	7.605844346	21.17823106	-1.477402
Gm20625	Gm20625-001	2996	7.85825E-05	0.000671185	0.359260007	-2.78349936	8.675587452	24.14849212	-1.4769
Snx2	Snx2-201	9869	9.41679E-07	3.36963E-05	0.35927841	-2.783356783	31.32410471	87.1861593	-1.476826
Gm2810	Gm2810-001	2253	0.001016566	0.004611574	0.359299683	-2.783191988	7.106952599	19.78001353	-1.47674
Psph	Psph-001	526.79902	0.046262476	0.092234111	0.359363141	-2.78270052	1.63466542	4.548784315	-1.476486
Abcd4	Abcd4-201	2523	2.37545E-05	0.000284181	0.359461228	-2.781941199	6.610284843	18.38942374	-1.476092
Letm1	Letm1-002	281.94589	0.028773009	0.063110817	0.359551552	-2.781242341	0.922057552	2.564465504	-1.475729
Tmx2	Tmx2-201	2275.8348	0.004562941	0.014896649	0.35955527	-2.781213579	6.240834188	17.35709279	-1.475715
Lrpprc	Lrpprc-001	4868.102	8.06416E-05	0.000685524	0.35966174	-2.78039026	17.71304862	49.24918785	-1.475287
Itch	Itch-002	3661.5842	0.024059109	0.054799792	0.359700223	-2.780092801	12.35872014	34.35838889	-1.475133
Tm4sf1	Tm4sf1-001	764.50096	0.035825031	0.075253438	0.359736231	-2.779814522	2.45075548	6.812645671	-1.474989
Gm3076	Gm3076-001	252	0.015924901	0.039655966	0.359943315	-2.778215234	0.883698586	2.455104875	-1.474158
Ctdnep1	Ctdnep1-001	3977.5995	0.002490412	0.009291569	0.359965286	-2.778045661	12.21399892	33.93104671	-1.47407
10013O21R	0013O21Rik-	1037.1217	0.002885359	0.010437635	0.360200731	-2.776229789	2.912020437	8.084437882	-1.473127
Set	Set-002	14442.276	0.000158031	0.001122413	0.360245499	-2.775884785	49.87363934	138.4434766	-1.472948
Pcm1	Pcm1-201	34817	2.64488E-05	0.000307179	0.360340596	-2.775152206	100.1966122	278.0608494	-1.472567
Dad1	Dad1-001	3876.5662	0.002943079	0.010596074	0.360378657	-2.774859109	12.82577277	35.58971239	-1.472415
Prps1	Prps1-001	1890.7763	0.000246899	0.001561353	0.36038418	-2.774816587	6.169758863	17.11994923	-1.472392
Srpk2	Srpk2-010	570.40342	0.008741193	0.024775393	0.36039945	-2.774699019	1.149939343	3.190735568	-1.472331

Rab27a	Rab27a-001	3549.1277	0.040235192	0.082513343	0.360476574	-2.774105372	8.502349006	23.58641205	-1.472023
Matr3	Matr3-001	7737.9258	2.76361E-05	0.000317204	0.36047848	-2.774090703	24.06161522	66.74910308	-1.472015
Slc7a2	Slc7a2-001	2993.1925	0.019245171	0.046078092	0.360482992	-2.774055984	7.345744081	20.37750533	-1.471997
Zfp788	Zfp788-004	830.86244	0.00269933	0.009895503	0.360492172	-2.773985342	1.965408742	5.452015043	-1.47196
Tax1bp1	Tax1bp1-002	586.82867	0.014442966	0.036789497	0.360560115	-2.773462613	1.693981668	4.698194824	-1.471688
Ercc6l2	Ercc6l2-010	1211.4703	0.031488993	0.067902664	0.360678424	-2.772552873	4.260271244	11.81182728	-1.471215
Lman2l	Lman2l-004	2648.7628	3.38024E-05	0.000364137	0.360684651	-2.772505003	8.488508697	23.53443283	-1.47119
Rab28	Rab28-004	384.77635	0.039096	0.08063202	0.360690142	-2.772462801	1.021498301	2.832066042	-1.471168
P23-396H1(	23-396H16.2-	848	0.020925286	0.049186126	0.360690911	-2.772456884	2.913835005	8.078481917	-1.471165
Orc2	Orc2-001	2160.7247	0.003614121	0.012405658	0.360750024	-2.772002586	7.967742947	22.08660405	-1.470929
Abca5	Abca5-001	3355.1344	0.000362757	0.002085684	0.360757619	-2.771944227	10.34989566	28.68933352	-1.470898
Slc30a4	Slc30a4-001	6665.207	1.142E-05	0.000169616	0.360772814	-2.771827484	21.37930939	59.25975736	-1.470837
Zfp182	Zfp182-003	1380.2097	0.021013137	0.049365522	0.360814842	-2.771504616	4.556812415	12.62922664	-1.470669
Gm38142	Gm38142-001	2345	8.25544E-05	0.000695227	0.360824215	-2.771432619	7.00413523	19.41148884	-1.470632
Mecom	Mecom-011	3012.2268	0.002135774	0.008234858	0.360844134	-2.771279635	9.416452795	26.09562387	-1.470552
Tnks2	Tnks2-003	2433.3052	4.2167E-05	0.000426653	0.360915093	-2.770734775	6.751662924	18.70706725	-1.470269
Glipr1	Glipr1-003	347.58987	0.01998499	0.04748363	0.360939882	-2.770544488	1.073670706	2.974652457	-1.47017
Alkbh8	Alkbh8-201	3229.4067	0.000617163	0.003145042	0.360944607	-2.770508217	9.810961237	27.18134872	-1.470151
Gabarapl2	Gabarapl2-001	4757.7817	5.98148E-05	0.000549965	0.361022686	-2.769909036	13.01165045	36.04108815	-1.469839
AC114588.1	C114588.1-2(	4619.2709	1.96906E-05	0.00024941	0.361075292	-2.76950548	13.66599706	37.84805375	-1.469628
Sra1	Sra1-001	2229.6839	0.000160481	0.001134931	0.361118739	-2.769172279	6.436610948	17.82408461	-1.469455
Trappc11	Trappc11-001	3600.1466	0.001298658	0.005594143	0.361119019	-2.76917013	8.970439976	24.84067444	-1.469454
P24-378G4	24-378G4.6-(	710	0.010265277	0.02816742	0.361128358	-2.769098513	2.023937571	5.604482517	-1.469416
Mtdh	Mtdh-003	4868.6076	0.000186594	0.001266627	0.361198583	-2.768560138	16.00384783	44.30761516	-1.469136
Ppia	Ppia-003	1728.9281	0.036369024	0.076227836	0.361203342	-2.768523661	5.121712217	14.17958146	-1.469117
Rap1gds1	Rap1gds1-00(	278.20371	0.037633635	0.078289116	0.361227643	-2.768337416	0.540976215	1.497604696	-1.46902
Rbm27	Rbm27-202	9219.693	2.31351E-05	0.000279596	0.361315915	-2.76766109	28.81510674	79.75044973	-1.468667
Gm15878	Gm15878-001	224	0.017456102	0.042644187	0.361324625	-2.767594374	0.745491685	2.063218593	-1.468633
Klhl4	Klhl4-201	1787.5037	0.009179023	0.025776725	0.361368391	-2.76725919	4.474027459	12.3807936	-1.468458
30040H23F	0040H23Rik-	565	0.012652573	0.033130782	0.361430959	-2.766780139	1.751961148	4.847291309	-1.468208
Mgat4a	Mgat4a-006	486.98542	0.035406284	0.074552147	0.361520736	-2.766093064	1.380625699	3.818939168	-1.46785
Hp1bp3	Hp1bp3-011	4431.5933	0.000125158	0.000946607	0.361552032	-2.76585363	13.95219356	38.5897252	-1.467725
Slc23a2	Slc23a2-001	9329.1271	1.49235E-05	0.000204609	0.361573464	-2.765689683	28.0187751	77.49123722	-1.467639
Zdhhc7	Zdhhc7-001	3148.5272	0.000927385	0.004296352	0.361764643	-2.764228126	10.29733017	28.46416966	-1.466877
Ubxn8	Ubxn8-201	3236.9139	6.83576E-06	0.000119573	0.361828493	-2.763740333	9.049130951	25.00944819	-1.466622
Cox5b	Cox5b-005	315.9092	0.029875252	0.065107366	0.361839728	-2.76365452	0.871352615	2.408117593	-1.466577
Stat3	Stat3-002	2860.6077	0.004800146	0.015489448	0.3618685	-2.763434782	8.52673009	23.56306251	-1.466463
00006K01F	0006K01Rik-	166.64304	0.028040321	0.061876231	0.361890221	-2.763268917	0.519610306	1.435823007	-1.466376
Trim23	Trim23-001	2608.5428	0.002176667	0.008361276	0.361968248	-2.762673261	7.798719629	21.54531419	-1.466065
Cops2	Cops2-002	5282.3079	0.001175649	0.005174245	0.361998093	-2.76244549	17.18432035	47.47074824	-1.465946
Dcun1d5	Dcun1d5-201	3882	0.001011783	0.004598401	0.362054872	-2.762012276	14.27705803	39.42189775	-1.46572
30596B20F	0596B20Rik-	1727.3856	0.000623925	0.003170538	0.362063794	-2.761944208	5.346503711	14.76674496	-1.465684
Sidt2	Sidt2-002	3838.382	0.044248971	0.089033443	0.362140016	-2.761362883	9.684103825	26.74132486	-1.46538
10007P14F	0007P14Rik-	641.72663	0.009007664	0.025385706	0.362141524	-2.761351386	1.684702755	4.652056287	-1.465374
Tnpo3	Tnpo3-007	548.75253	0.049779503	0.097621957	0.362343355	-2.759813273	1.600103261	4.415986217	-1.464571
Ghitm	Ghitm-201	4269.891	0.001566267	0.006467104	0.36236346	-2.759660153	11.68861207	32.25659696	-1.464491
Ints9	Ints9-201	3303	3.7409E-05	0.000390961	0.362380081	-2.759533573	9.859443315	27.20746484	-1.464424
Ddi2	Ddi2-001	13711.359	2.85877E-05	0.000324	0.362382622	-2.759514224	48.36289906	133.4581079	-1.464414
Zfp518b	Zfp518b-001	1711.962	0.029512036	0.064438813	0.362394032	-2.759427342	6.07649811	16.76765503	-1.464369
Ap3s2	Ap3s2-201	4947.2136	2.69591E-05	0.000311416	0.362406175	-2.759334879	15.17608642	41.87590459	-1.464321
Usp8	Usp8-001	13127.934	6.58856E-06	0.000116691	0.362416894	-2.759253269	38.88134262	107.2834717	-1.464278
Zfp68	Zfp68-006	844.84633	0.007157845	0.021196283	0.362419327	-2.759234747	3.152816204	8.699360023	-1.464268
Mbnl1	Mbnl1-005	6984.1847	7.13275E-05	0.000626134	0.362426074	-2.759183378	17.59267373	48.54141293	-1.464241
Aig1	Aig1-002	795.89847	0.001646053	0.006714783	0.362452319	-2.758983588	2.823259307	7.789326094	-1.464137
Gng11	Gng11-001	6230	8.62596E-05	0.000715869	0.362480189	-2.758771462	15.71477178	43.35346392	-1.464026
Nit2	Nit2-201	592	0.019655499	0.046877018	0.362516856	-2.758492422	2.074451431	5.722358554	-1.46388
Rbm18	Rbm18-002	1771.6836	0.000591466	0.003041241	0.362544477	-2.758282261	5.743406607	15.84193656	-1.46377
Polr3f	Polr3f-004	465.99642	0.042550777	0.086295837	0.362597942	-2.757875552	1.423263474	3.925183538	-1.463557
Gm5431	Gm5431-002	1274.7288	0.011162971	0.030033309	0.362623455	-2.757681521	3.116144163	8.593333174	-1.463456
Gm12355	Gm12355-001	2669	0.0035148	0.012140168	0.362636062	-2.757585652	9.828840334	27.10386908	-1.463406
Gm28441	Gm28441-001	356	0.013239585	0.03436052	0.36270289	-2.75707756	1.02931136	2.837891253	-1.46314
Gm12992	Gm12992-002	1021.244	0.001480313	0.006194346	0.362738839	-2.756804325	3.286723991	9.060854914	-1.462997
Pogz	Pogz-001	4984.7913	0.000705859	0.003486287	0.362781419	-2.756480754	12.90998643	35.58612912	-1.462828
Phf21b	Phf21b-001	180.23911	0.037801606	0.078566964	0.3627919	-2.756401119	0.637733469	1.757849246	-1.462786
Klhl21	Klhl21-001	5176	1.65671E-06	4.7483E-05	0.362829236	-2.756117485	17.77705803	48.99566046	-1.462637
Fat4	Fat4-001	11649.368	0.002025331	0.007909644	0.362897371	-2.755600009	40.63094379	111.9626291	-1.462366
Kctd12b	Kctd12b-001	2338.3157	0.037762244	0.078499444	0.362954302	-2.755167787	5.97964023	16.47491214	-1.46214
Dph3	Dph3-001	2589.9835	0.002836319	0.010289079	0.363007067	-2.75476731	6.755815715	18.61070524	-1.46193
Ltn1	Ltn1-201	12532	3.0297E-07	1.73524E-05	0.363091832	-2.7541242	35.76784188	98.50907891	-1.461594
Stx17	Stx17-001	6095.6692	1.00693E-05	0.000155177	0.363138374	-2.753771215	19.45740207	53.58123374	-1.461409
Deb1	Deb1-201	547	0.009491767	0.026464753	0.363154877	-2.753646072	1.9575805	5.390483855	-1.461343
Plaa	Plaa-001	5384.5399	0.004316778	0.014260191	0.363207974	-2.75324352	18.11918413	49.88652629	-1.461132
Fbxl20	Fbxl20-002	1800.8115	0.003205108	0.011321673	0.363293453	-2.752595712	5.754244715	15.83910933	-1.460793
Ppig	Ppig-001	5578.5284	0.000504067	0.002689981	0.363309232	-2.752476162	15.80652881	43.50709387	-1.46073
Kmt2c	Kmt2c-007	9313.4578	0.002668351	0.009806957	0.363331916	-2.752304313	22.49815675	61.92177385	-1.46064
Rnf10	Rnf10-011	1028.7987	0.011824006	0.031449266	0.363363106	-2.752068068	2.554771558	7.030905225	-1.460516
Timm10b	Timm10b-001	892.28469	0.003239178	0.011407871	0.363369267	-2.752021404	2.690489384	7.404284374	-1.460492
Gm37086	Gm37086-001	20554	3.08244E-06	7.19655E-05	0.363511037	-2.750948109	61.77240127	169.9326705	-1.459929
Tmem50b	Tmem50b-001	3074.7725	0.000704573	0.003482199	0.36355519	-2.750614012	10.94572497	30.10746448	-1.459754
Gm12350	Gm12350-001	415	0.026627342	0.059362526	0.363575646	-2.750459258	1.187735231	3.266817362	-1.459673
30594C11F	0594C11Rik-	1795	4.2586E-05	0.00043013	0.363578968	-2.750434121	5.815771213	15.99589558	-1.459659
42620	Sept7-201	5274.4034	0.003586391	0.012336436	0.363610656	-2.750194427	13.71954148	37.73140651	-1.459534
Sphk1	Sphk1-016	1215.5998	0.003362911	0.011741052	0.363615569	-2.750157271	4.009161847	11.0258256	-1.459514
Flii	Flii-003	848.80423	0.004117048	0.013723492	0.363619932	-2.750124272	2.586541657	7.113310992	-1.459497
Ccar1	Ccar1-201	19736	1.4275E-06	4.3567E-05	0.36366059	-2.749816799	59.79789285	164.4332503	-1.459336
Gm17540	Gm17540-201	367	0.018083004	0.043834872	0.363713277	-2.749418465	1.247432308	3.429713421	-1.459127
Gabpb1	Gabpb1-009	1009.3817	0.002182706	0.008379029	0.36385282	-2.748364025	2.89859447	7.966392765	-1.458573
Rlf	Rlf-001	7215.5587	5.89558E-05	0.000543683	0.363909899	-2.747932941	23.01245277	63.23667701	-1.458347
Paip2b	Paip2b-001	4082.2019	0.00041471	0.002312947	0.363936151	-2.747734725	12.49605582	34.33584649	-1.458243
Ndufv3	Ndufv3-004	289.69376	0.034302401	0.072776526	0.363962528	-2.747535596	0.452754136	1.243958104	-1.458138
Dnajc30	Dnajc30-001	888.09277	0.000581694	0.003004074	0.363980889	-2.747396993	2.172713905	5.969307649	-1.458065
Tpt1	Tpt1-005	103524.23	6.55605E-06	0.000116224	0.363993091	-2.747304893	349.270081	959.5514024	-1.458017
Gm26951	Gm26951-001	3935.4933	1.4023E-05	0.000196115	0.364151696	-2.746108315	14.16326917	38.89387123	-1.457389
Smu1	Smu1-001	8120.9937	6.07045E-06	0.000110474	0.364208791	-2.74567782	26.61374078	73.07275776	-1.457162
Gm37368	Gm37368-001	2330	0.005483308	0.017224918	0.364255928	-2.745322514	5.820236286	15.97842571	-1.456976
Spag7	Spag7-001	5312.231	1.12802E-05	0.00016826	0.364292651	-2.745045767	14.78116957	40.57498695	-1.45683
00004A13F	0004A13Rik-	4209.6423	5.32554E-06	0.000101535	0.364305068	-2.744952207	12.93126401	35.49570169	-1.456781
Atp6v1c1	Atp6v1c1-201	4298	1.12672E-05	0.00016826	0.364336704	-2.744713857	12.68888366	34.8273548	-1.456656
Casc4	Casc4-001	5207.3447	8.53197E-06	0.000138756	0.364399015	-2.744244523	15.76034401	43.25023774	-1.456409
Lrif1	Lrif1-002	1621.3219	0.033071436	0.070617	0.364422143	-2.744070354	4.556262786	12.50270564	-1.456317
Ikbke	Ikbke-003	764.10458	0.005957097	0.018340945	0.364500493	-2.743480516	2.038541594	5.592698595	-1.456007
Trim30a	Trim30a-001	7958.7856	4.10273E-05	0.000417589	0.364507376	-2.743428706	22.96488838	63.00253402	-1.45598
Zfp26	Zfp26-001	1416.252	0.011652597	0.031106893	0.364529507	-2.743262152	4.226512354	11.59443137	-1.455892
Zswim6	Zswim6-201	10779	7.48946E-05	0.000648511	0.364559668	-2.743035193	33.76301882	92.61314884	-1.455773
Gm38001	Gm38001-001	1091	0.023041825	0.053018644	0.364582587	-2.742862756	2.830536923	7.763774306	-1.455682
Med27	Med27-002	705.23868	0.025733486	0.05776079	0.364670061	-2.742204822	1.905503725	5.225281503	-1.455336
Gm37621	Gm37621-001	3967	9.79673E-05	0.000785436	0.364685332	-2.742089993	12.11285658	33.2145428	-1.455276

Zfp72	Zfp72-201	350	0.016580768	0.040955668	0.364787372	-2.741322963	0.885640767	2.427827372	-1.454872
Gm37954	Gm37954-001	30932	3.09215E-06	7.20303E-05	0.36485965	-2.740779913	95.55878181	261.9055897	-1.454586
Klhl7	Klhl7-003	1742.5949	0.020251465	0.04794142	0.364900456	-2.740473419	4.212682847	11.54474537	-1.454425
Tm9sf3	Tm9sf3-201	37593	1.37419E-06	4.27276E-05	0.364997167	-2.739747294	121.7305578	333.5109665	-1.454043
Oxr1	Oxr1-203	490.40838	0.040976228	0.083742217	0.365067282	-2.739221094	1.022532244	2.800941892	-1.453766
Slc25a15	Slc25a15-201	1061	0.002034661	0.007937033	0.365231564	-2.737988985	3.495325115	9.570161666	-1.453117
Atl2	Atl2-201	21679.824	0.002299013	0.008737372	0.365232731	-2.737980242	50.66977415	138.7328405	-1.453112
Ankrd33b	Ankrd33b-001	3617.7484	0.000258075	0.001613265	0.365252576	-2.737831476	4.867027137	13.32510009	-1.453034
Gvin1	Gvin1-001	28207.097	0.002036469	0.007942276	0.365343984	-2.737146481	86.55341879	236.9093857	-1.452673
Gm4430	Gm4430-001	486.19234	0.009622076	0.026734494	0.365359624	-2.737029313	1.597853073	4.373370698	-1.452611
Gm18180	Gm18180-001	554	0.006553703	0.019745278	0.3653939	-2.736772564	1.821814072	4.985890767	-1.452476
Slc38a2	Slc38a2-201	40521	0.002168252	0.008335686	0.365394519	-2.736767927	147.1664458	402.7604088	-1.452473
Jarid2	Jarid2-005	1764.0356	0.031563509	0.068044355	0.365399149	-2.73673325	6.437578109	17.61793406	-1.452455
Synj2bp	Synj2bp-001	1221.8657	0.043580642	0.087936667	0.365425315	-2.736537284	3.571101976	9.772453704	-1.452352
Nop9	Nop9-001	2007.5164	0.000333466	0.001958367	0.365454702	-2.736317239	7.568388839	20.70951285	-1.452236
Nck1	Nck1-002	7090.3187	0.000198659	0.001326269	0.365481629	-2.736115636	12.89290385	35.27647581	-1.452129
Vdac3-ps1	Vdac3-ps1-20	2029	9.20853E-05	0.000751649	0.365499789	-2.735979689	5.879337264	16.08574734	-1.452058
Calhm2	Calhm2-001	3682.4256	0.000736593	0.003595311	0.365501931	-2.735963657	10.80469441	29.56125123	-1.452049
Gatad2b	Gatad2b-003	7565.7212	0.002781348	0.010124471	0.365555338	-2.73556394	24.55522723	67.17239415	-1.451838
Sema7a	Sema7a-201	4474	0.001541976	0.006386561	0.365573276	-2.735429711	17.40690816	47.61537374	-1.451767
Tanc2	Tanc2-003	1474.7892	0.033728856	0.07178359	0.365600138	-2.73522873	4.224438158	11.55480462	-1.451661
Mtmr7	Mtmr7-002	275.45053	0.025992698	0.058254804	0.365781738	-2.733870763	0.701211346	1.917021198	-1.450945
Trp53bp2	Trp53bp2-001	5603.9092	0.00010389	0.000819682	0.365819608	-2.733587756	19.9413038	54.51130391	-1.450796
Csnk1d	Csnk1d-002	8466.9923	1.37645E-06	4.27276E-05	0.36582046	-2.733581387	25.86164183	70.69490274	-1.450792
Spata6	Spata6-004	1257.7521	0.036399393	0.076277484	0.365891017	-2.733054251	3.855752652	10.53798118	-1.450514
Zfp277	Zfp277-202	5755.1756	5.5187E-06	0.000104083	0.365990211	-2.732313516	17.53969089	47.92393448	-1.450123
Senp1	Senp1-001	3938.6865	0.008310347	0.02381238	0.366044163	-2.731910796	13.05078734	35.65358682	-1.44991
Tubgcp3	Tubgcp3-002	2359.5499	0.000324764	0.001918451	0.366046401	-2.731894094	6.811298801	18.60774697	-1.449902
42432	March3-002	2369.9779	0.002097885	0.008124964	0.366114907	-2.731382907	7.871312597	21.49956869	-1.449632
Tmem185b	Tmem185b-00	681.40839	0.00221933	0.008498664	0.366230718	-2.730519179	2.260346536	6.171919566	-1.449175
Cox7b	Cox7b-003	3053.7354	0.000333756	0.001959735	0.366301014	-2.729995173	9.398381039	25.65753487	-1.448898
Lum	Lum-201	1618	0.036012506	0.075596245	0.36634856	-2.729640864	7.33761481	20.02905323	-1.448711
Dgcr8	Dgcr8-003	3682.0521	0.000175179	0.001207689	0.366350295	-2.729627934	10.79915898	29.47768601	-1.448704
Fxyd6	Fxyd6-201	7243	0.00015775	0.00112071	0.366398388	-2.729269651	15.83884066	43.22846714	-1.448515
Pcdhga11	Pcdhga11-001	3850.7525	0.000291256	0.001768711	0.366488589	-2.728597915	11.10703879	30.30664288	-1.44816
Akap1	Akap1-002	786.46485	0.011163622	0.030033309	0.366507898	-2.728454162	1.92379242	5.248979434	-1.448084
Gm26909	Gm26909-201	1222.3359	0.001417953	0.005990489	0.366601972	-2.727754012	3.846461141	10.49219981	-1.447714
Pou2f1	Pou2f1-015	1640.1615	0.009806917	0.027142375	0.366638264	-2.727484004	5.178704135	14.12483269	-1.447571
Herc6	Herc6-201	5641	0.000479526	0.002588049	0.366737403	-2.726746687	15.62572616	42.60739705	-1.447181
Krr1	Krr1-003	2054.4312	0.00240667	0.009046588	0.366810701	-2.726201819	6.526990778	17.79389413	-1.446892
Kdm5a	Kdm5a-201	6743.3765	0.000159658	0.00113153	0.366940183	-2.725239824	22.87132085	62.32983442	-1.446383
G3bp1	G3bp1-002	305.05928	0.049969513	0.097903771	0.367079178	-2.724207906	1.025698565	2.79421614	-1.445837
Mtf2	Mtf2-015	684.22123	0.006786359	0.020310451	0.367123582	-2.723878416	2.574608998	7.01292188	-1.445662
Atp5h	Atp5h-005	2453.3363	0.025355869	0.057126596	0.367187141	-2.723406921	6.337711168	17.26016646	-1.445413
Gm28661	Gm28661-001	123473	5.68819E-07	2.48495E-05	0.367273278	-2.722768195	368.6330215	1003.702266	-1.445074
Gm6123	Gm6123-001	277	0.030458648	0.066143082	0.367280299	-2.722716146	0.863763425	2.351782624	-1.445047
Esf1	Esf1-001	7814.9697	5.70094E-05	0.000530631	0.36729309	-2.722621328	27.8803061	75.90751603	-1.444996
Ammecr1l	Ammecr1l-20	6895.2649	1.50882E-05	0.000206378	0.367321311	-2.722412152	20.56453941	55.985152	-1.444885
Gm9917	Gm9917-004	3987.8735	0.000723559	0.003549399	0.367330076	-2.722347192	8.478499255	23.08141864	-1.444851
Arhgef1	Arhgef1-004	1513.663	0.001433932	0.006044489	0.367392002	-2.721888323	3.415238806	9.295898628	-1.444608
Hbs1l	Hbs1l-202	897.1902	0.015514598	0.038885838	0.367518896	-2.720948531	2.948011666	8.021388009	-1.44411
Gpr107	Gpr107-001	7519.8188	5.59484E-06	0.000104999	0.367527994	-2.720881172	20.51224039	55.81136868	-1.444074
Ralgps2	Ralgps2-006	536.64692	0.010651407	0.028973924	0.367572846	-2.720549165	1.753433751	4.770302729	-1.443898
Suv420h1	Suv420h1-00	3011.2935	0.000613654	0.003131355	0.367575657	-2.720528365	8.644513876	23.5176452	-1.443887
Stxbp3	Stxbp3-001	5266.1993	0.000100596	0.000800234	0.367642995	-2.720030068	13.68115665	37.21315746	-1.443623
Zdhhc3	Zdhhc3-003	5574.8613	0.000966433	0.004429916	0.367704866	-2.719572389	14.11577364	38.38886824	-1.44338
Gm26599	Gm26599-201	1265	0.000757617	0.003672807	0.367708968	-2.719542046	3.619615931	9.843697715	-1.443364
Aldh9a1	Aldh9a1-001	4217.328	0.00055019	0.002880464	0.367749829	-2.719239879	13.57177774	36.90491926	-1.443203
30480K23R	30480K23Rik-	263.02051	0.023950565	0.054614305	0.367811655	-2.718782794	0.641515272	1.744140683	-1.442961
Gm6768	Gm6768-201	3639	5.36763E-05	0.000507568	0.367827725	-2.718664016	9.52767201	25.90253905	-1.442898
Gm15462	Gm15462-001	4388	1.46482E-05	0.000201892	0.367847452	-2.71851822	12.96719964	35.25156848	-1.44282
Osbpl3	Osbpl3-002	2111.0985	0.035436402	0.074590672	0.367908706	-2.718065603	7.642348656	20.77240501	-1.44258
Thada	Thada-201	5378	9.07335E-05	0.000743095	0.367934468	-2.717875289	18.2412032	49.57731542	-1.442479
Gatc	Gatc-001	4514	7.779E-06	0.00013058	0.367947662	-2.717777838	13.91412726	37.81550669	-1.442428
Cdca4	Cdca4-201	1453	0.010468353	0.028610045	0.367954333	-2.717728559	5.584609005	15.17745138	-1.442401
Hspa14	Hspa14-007	241.72082	0.049497235	0.097234316	0.367968162	-2.71762642	0.768373212	2.08815134	-1.442347
Zfp846	Zfp846-001	987.82546	0.000948552	0.004371355	0.36797104	-2.717605171	3.04631498	8.278681343	-1.442336
Gm37364	Gm37364-001	751	0.009313173	0.026064443	0.367975722	-2.71757059	1.882038838	5.114573397	-1.442318
Dgkq	Dgkq-012	577.00183	0.014943021	0.03777668	0.368139993	-2.716357962	1.89344412	5.14327201	-1.441674
Ipo5	Ipo5-201	10952	0.000989679	0.004514118	0.368154435	-2.716251404	41.54689823	112.8518207	-1.441617
Dgkq	Dgkq-007	453.77361	0.037136131	0.077489489	0.368171425	-2.716126053	0.994144488	2.700221745	-1.44155
Sirt1	Sirt1-005	1234.895	0.001205141	0.005272877	0.368200781	-2.715909506	3.533101003	9.595582601	-1.441435
Kdm2a	Kdm2a-002	536.53145	0.020678547	0.048749611	0.368249203	-2.715552383	1.812771704	4.922676521	-1.441246
Traf3	Traf3-201	577.72834	0.038583312	0.079833168	0.368270067	-2.715398535	2.524591567	6.855272244	-1.441164
Anapc7	Anapc7-002	1141.6996	0.001473086	0.006171643	0.368308887	-2.715112329	3.674920931	9.977823131	-1.441012
Zfp462	Zfp462-001	2385.9324	0.013251631	0.034389177	0.368443557	-2.71411993	8.400799897	22.80077843	-1.440484
Limd1	Limd1-201	5170	3.85672E-06	8.21471E-05	0.368466669	-2.713949683	19.01524679	51.60642299	-1.440394
AW554918	AW554918-20	651.33713	0.011458365	0.030704568	0.368511797	-2.713617335	1.727017259	4.686463973	-1.440217
Rgl1	Rgl1-001	3898.091	9.10167E-05	0.00074488	0.368602299	-2.712951069	12.23672835	33.19764526	-1.439863
Gm11331	Gm11331-001	367	0.016408678	0.040600869	0.368636649	-2.712698273	1.074884102	2.915836247	-1.439729
Zfp326	Zfp326-002	1409.1566	0.004122241	0.013738129	0.3686378	-2.712689798	4.958029707	13.4495966	-1.439724
Fbxo18	Fbxo18-010	498.73533	0.035393529	0.074534455	0.368643068	-2.712651038	1.508658548	4.092464178	-1.439703
Fbln1	Fbln1-001	960.47991	0.04026508	0.08254498	0.368654263	-2.712568663	3.269020834	8.867443472	-1.43966
Zfc3h1	Zfc3h1-001	27917.466	5.48963E-07	2.4488E-05	0.368741194	-2.711929167	82.37203988	223.3871375	-1.439319
Gm3650	Gm3650-001	275	0.021658578	0.050553619	0.368741476	-2.711927099	0.791322284	2.146008346	-1.439318
Acsl5	Acsl5-201	13396	4.02291E-06	8.45824E-05	0.368749803	-2.711865856	39.08012629	105.9800601	-1.439286
Kdm5c	Kdm5c-001	3869.2923	0.009262677	0.0259523	0.368960803	-2.710315004	11.84676088	32.10845375	-1.438461
Rwdd4a	Rwdd4a-001	1418.6407	0.003568766	0.012290624	0.36896082	-2.710314876	4.578969375	12.41044882	-1.43846
Ankrd50	Ankrd50-001	4118.5988	0.001025833	0.004646844	0.369018927	-2.709888101	12.46610545	33.78175082	-1.438233
Ddx3x	Ddx3x-001	49348.08	3.27426E-07	1.82269E-05	0.369035313	-2.709767781	164.2167088	444.9891467	-1.438169
Tmem63b	Tmem63b-00	1005.8509	0.003728462	0.012716951	0.369045593	-2.709692297	2.771445493	7.509764505	-1.438129
Vegfc	Vegfc-201	1849	0.000514694	0.002732399	0.369079887	-2.709440515	6.519646215	17.66459359	-1.437995
Cdk8	Cdk8-009	340.05981	0.048565992	0.0958834	0.369104358	-2.70926089	1.10154229	2.984365443	-1.437899
Nkap	Nkap-001	4266.4497	5.22138E-06	0.000100298	0.369215136	-2.708448013	12.72219983	34.45741685	-1.437466
Ltv1	Ltv1-201	3234	0.020395731	0.048225899	0.369296064	-2.70785448	12.37213887	33.50195167	-1.43715
Fam3c	Fam3c-001	44558.789	0.001164338	0.00513502	0.369381322	-2.707229471	137.292348	371.6818907	-1.436817
Ick	Ick-003	366.33406	0.025542825	0.057449551	0.369612779	-2.705534161	0.897251205	2.427543786	-1.435913
Tom1l2	Tom1l2-008	1452.7331	0.034208151	0.07260964	0.369669264	-2.705120758	3.566226329	9.647072872	-1.435693
Stk3	Stk3-001	4111.0637	0.013204555	0.034290395	0.369729043	-2.704683383	13.72821049	37.13046279	-1.43546
Cep68	Cep68-001	6383.7657	3.35635E-06	7.55785E-05	0.369781359	-2.704300736	18.26462812	49.39304726	-1.435256
Zhx3	Zhx3-003	21204.24	0.005910306	0.018236754	0.369806726	-2.704115232	53.63668787	145.0397846	-1.435157
Smad1	Smad1-001	6020.0889	0.00053704	0.002826212	0.369842441	-2.703854096	23.57111169	63.7328469	-1.435017
Lrrc16a	Lrrc16a-005	1188.7691	0.001312063	0.005637	0.369843068	-2.703849518	2.430624551	6.572043019	-1.435015
Flt3l	Flt3l-005	986.87044	0.017325776	0.042377193	0.369918897	-2.703295254	3.264298264	8.824362005	-1.434719
BC039771	BC039771-00	548.31993	0.010091719	0.027786011	0.3700248	-2.702521563	1.712430598	4.627880616	-1.434306
Rev3l	Rev3l-002	3963.6145	0.022506024	0.052083198	0.370059111	-2.70227099	8.986205561	24.2831626	-1.434172
Mef2c	Mef2c-004	847.53949	0.00265961	0.00978323	0.370077295	-2.70213821	2.708798112	7.319546883	-1.434101

P23-370G17	23-370G17.4-	4147.9956	0.000202502	0.001346462	0.370103793	-2.701944747	13.95079056	37.69426527	-1.433998
Eif6	Eif6-002	446.7762	0.019409134	0.046394749	0.370133048	-2.701731189	1.412385688	3.815886463	-1.433884
Stat3	Stat3-006	832.58898	0.002890781	0.010453938	0.370195971	-2.70127197	3.050147271	8.239277327	-1.433639
Rcan3	Rcan3-001	5426.3708	3.81638E-05	0.000395854	0.37023415	-2.700993409	16.44979602	44.43079062	-1.43349
P24-235L14	24-235L14.3-	1430	0.000410204	0.002292837	0.370270563	-2.700727794	4.266143346	11.52169191	-1.433348
Brd4	Brd4-002	1271.3981	0.000467806	0.002542022	0.370271488	-2.700721047	4.352006685	11.75355605	-1.433345
AC153941.1	C153941.1-20	1172.4736	0.001581065	0.006512505	0.370397495	-2.699802275	4.941490519	13.34104735	-1.432854
Lig1	Lig1-014	1313.4361	0.010424863	0.028518318	0.370434148	-2.699535144	5.344162882	14.42675552	-1.432711
Dtx2	Dtx2-006	411.98087	0.008722725	0.024740237	0.370551963	-2.698676841	0.985511087	2.659575947	-1.432252
Atrn	Atrn-001	17061.465	1.04429E-05	0.000159248	0.370568896	-2.69855352	47.16242074	127.2703165	-1.432186
Ube2n	Ube2n-201	5686	0.000203983	0.0013516	0.370569974	-2.698545673	17.48167142	47.17508877	-1.432182
930539E08R	0539E08Rik-	578	0.007270579	0.021467012	0.370582586	-2.698453833	1.700869658	4.589718249	-1.432133
Naaa	Naaa-001	1466.2448	0.048044395	0.095072829	0.370619925	-2.69818197	6.887836444	18.5846361	-1.431988
Prkar2a	Prkar2a-001	9089.2016	1.30933E-05	0.000186554	0.370649267	-2.697968376	29.53918959	79.69579936	-1.431873
Nr2c1	Nr2c1-001	1593.8835	0.000226611	0.001465089	0.37068081	-2.697738787	4.640458521	12.51874494	-1.431751
Fgd4	Fgd4-009	34371.261	4.80053E-06	9.51512E-05	0.370741308	-2.697298571	104.8923065	282.9258684	-1.431515
Tmem254c	mem254c-00	894.39333	0.023993393	0.054693757	0.370790929	-2.696937603	2.769789828	7.469950339	-1.431322
Gm38005	Gm38005-001	565	0.003446836	0.011955679	0.370902348	-2.696127444	1.688328395	4.551948519	-1.430889
Gm16938	Gm16938-202	912.5597	0.031394096	0.067749214	0.370913544	-2.69604606	0.78468083	2.115535659	-1.430845
Mprip	Mprip-201	5397.4036	8.56574E-05	0.000712683	0.370985951	-2.695519865	17.25140751	46.50151165	-1.430564
Gm7882	Gm7882-001	417	0.010510876	0.028682773	0.371079786	-2.694838249	1.398048115	3.767513534	-1.430199
Samd5	Samd5-001	586	0.045251543	0.090582428	0.371160422	-2.694252783	1.288002016	3.470203017	-1.429885
Gm37258	Gm37258-001	527	0.017679465	0.043082297	0.371176599	-2.694135358	1.670000256	4.499206737	-1.429822
Ccdc144b	Ccdc144b-002	1217.8022	0.000468826	0.002545546	0.371181214	-2.694101861	3.68127914	9.917740982	-1.429804
Iars2	Iars2-003	1041.5608	0.001046555	0.004716382	0.3712393	-2.693680331	3.362962938	9.058747119	-1.429579
Enpp4	Enpp4-001	8692.2509	0.006683167	0.020059456	0.371239627	-2.693677959	17.73701898	47.77781708	-1.429577
Lyrm9	Lyrm9-001	3569.0796	0.00448071	0.01468277	0.371284239	-2.693354297	9.489354892	25.55819477	-1.429404
Ppp4r1l-ps	Ppp4r1l-ps-00	4698.8952	0.003574538	0.01230679	0.37130903	-2.693174473	12.75985746	34.3645224	-1.429308
Ccnh	Ccnh-002	992.29631	0.032937478	0.070413421	0.371316619	-2.693119425	2.919845685	7.86349313	-1.429278
Ttc21b	Ttc21b-001	1224.1498	0.028697365	0.062989281	0.371316821	-2.693117961	3.867087766	10.41452352	-1.429277
Fam208a	Fam208a-201	19612.886	7.76861E-05	0.000665687	0.371442088	-2.692209721	60.13713899	161.9017901	-1.428791
P23-151G22	23-151G22.1-	1020	0.000548398	0.00287284	0.371507943	-2.691732486	3.603967323	9.700915922	-1.428535
Gm37949	Gm37949-001	953	0.016863998	0.041523504	0.3715167	-2.691669041	3.107072814	8.363211701	-1.428501
Fn1	Fn1-003	21729.047	0.011708812	0.031230185	0.371537651	-2.691517255	78.83174885	212.1770123	-1.42842
Bptf	Bptf-005	912.53682	0.015147519	0.038159491	0.371549977	-2.691427972	2.885949864	7.767326191	-1.428372
Zfp617	Zfp617-001	3381.7173	5.57511E-06	0.000104752	0.371605771	-2.691023874	10.41151854	28.01764494	-1.428155
Cct2	Cct2-201	16875	0.000455993	0.002491266	0.371684727	-2.690452227	62.87047446	169.150008	-1.427849
Cdc73	Cdc73-001	11960.056	1.66622E-05	0.00022128	0.371914132	-2.688792689	38.22340028	102.7747992	-1.426959
Card6	Card6-001	4232.2274	0.000762514	0.003692366	0.371932088	-2.688662883	14.6105284	39.2827854	-1.426889
Abce1	Abce1-201	9277	0.000204763	0.001354935	0.372018778	-2.688036354	34.9942518	94.065821	-1.426553
Kpna3	Kpna3-201	6081	0.000548092	0.002872187	0.37225519	-2.686329198	22.20018153	59.63699584	-1.425636
Grn	Grn-008	2861.8607	0.000607003	0.003105281	0.372274046	-2.686193172	8.520242949	22.88701843	-1.425563
Gm11960	Gm11960-002	7513	2.42005E-06	6.15344E-05	0.372443487	-2.6849711	22.97710745	61.69286946	-1.424907
Etnk1	Etnk1-201	26980	3.82503E-06	8.16753E-05	0.372454216	-2.684893761	88.86535264	238.5940309	-1.424865
Gm10145	Gm10145-201	555	0.009488191	0.026459089	0.372479989	-2.684707983	1.875115869	5.034138543	-1.424765
Hars2	Hars2-001	2167.3427	0.007126845	0.02113006	0.372528524	-2.684358204	6.571667234	17.64070885	-1.424577
Ankrd17	Ankrd17-009	798.79736	0.004963658	0.01591414	0.372550058	-2.684203041	2.283702674	6.129921662	-1.424494
Gm29055	Gm29055-001	24001	4.61826E-06	9.27715E-05	0.372556138	-2.684159239	74.2849172	199.3925468	-1.42447
Gm37529	Gm37529-001	929	0.012157473	0.032129124	0.372588821	-2.68392379	2.179883006	5.850639859	-1.424344
Loxl3	Loxl3-001	1092.3108	0.032667621	0.069955359	0.372599922	-2.683843829	4.986046988	13.38177144	-1.424301
Iws1	Iws1-201	13790	2.35498E-07	1.55411E-05	0.372603519	-2.683817919	45.38328716	121.8004793	-1.424287
Cdkal1	Cdkal1-004	241.32333	0.033679032	0.071699828	0.372636768	-2.68357845	0.595740516	1.598716412	-1.424158
Vps54	Vps54-005	1521.2871	0.000291384	0.001769171	0.372774702	-2.682585472	4.450729663	11.93946273	-1.423624
Glmn	Glmn-001	771.86567	0.005122234	0.01631209	0.372783127	-2.682524846	2.33152268	6.254367517	-1.423592
Gm14052	Gm14052-001	490	0.039677944	0.081546331	0.372792832	-2.682455012	1.667018412	4.471701893	-1.423554
Gm26601	Gm26601-201	1103	0.000492123	0.002641467	0.372824986	-2.682223662	3.634828161	9.749422101	-1.42343
Gm37867	Gm37867-001	530	0.009179524	0.025776725	0.372849445	-2.682047708	1.586767501	4.255786139	-1.423335
L1cam	L1cam-008	17558.069	0.010726484	0.029118023	0.372867532	-2.681917609	30.90311148	82.87959885	-1.423265
Paxip1	Paxip1-001	8125.0688	2.75303E-06	6.65899E-05	0.372881747	-2.681815369	26.39804112	70.79467238	-1.42321
Nrd1	Nrd1-001	7644.0538	7.33542E-06	0.000125368	0.372899898	-2.681684831	19.96743266	53.54636128	-1.42314
P24-230J14	24-230J14.8-	707	0.008254036	0.023668848	0.372901033	-2.681676669	2.353010216	6.310012599	-1.423135
Sos2	Sos2-005	2812.0766	0.000304322	0.00182867	0.372905599	-2.681643836	9.034284791	24.22673412	-1.423118
Dnajc25	Dnajc25-002	1929.6676	0.00219958	0.008432553	0.372911789	-2.681599323	6.608100128	17.72027683	-1.423094
Ywhah	Ywhah-001	25604.785	0.00026071	0.001624464	0.372921423	-2.681530046	70.86841053	190.0357721	-1.423056
Zfp74	Zfp74-001	3643.4674	0.000887523	0.004157279	0.372922548	-2.681521955	10.06579001	26.99163691	-1.423052
Gm21955	Gm21955-001	1608	0.000118446	0.00090695	0.37293044	-2.681465212	5.165899431	13.85217961	-1.423022
Mat2b	Mat2b-001	1941.1134	0.022322955	0.051753956	0.372931092	-2.681460523	6.906942274	18.52069304	-1.423019
Cnih1	Cnih1-001	3189.4819	0.002292332	0.008716671	0.373065297	-2.680495905	9.780429469	26.21640114	-1.4225
Fnbp1l	Fnbp1l-001	26289.484	2.80359E-05	0.000320179	0.373083535	-2.680364869	76.65646666	205.4673002	-1.422429
Polr2g	Polr2g-201	1611	0.000875113	0.004113726	0.373087981	-2.68033293	5.400662487	14.47557351	-1.422412
Aamp	Aamp-002	1027.5381	0.018270305	0.04421205	0.373125301	-2.680064838	4.230624439	11.3383478	-1.422268
Natd1	Natd1-001	1812.3494	0.000182923	0.00124778	0.373129654	-2.680033576	4.988819121	13.37020275	-1.422251
Fdft1	Fdft1-201	2086.2458	0.003122173	0.011081288	0.373165104	-2.679778974	7.092097673	19.00525422	-1.422114
Crbn	Crbn-003	1611.0906	0.002854753	0.010344402	0.37320134	-2.679518784	4.58731178	12.29178792	-1.421974
Gm10110	Gm10110-201	900	0.020229859	0.047906566	0.373298342	-2.678822507	3.558298151	9.532049173	-1.421599
Tle4	Tle4-201	4495.123	0.002565353	0.009510048	0.373325837	-2.678625211	12.32462537	33.01305224	-1.421493
Pdxdc1	Pdxdc1-001	4837.2822	0.000106767	0.00083564	0.373360778	-2.678374538	14.50589518	38.8522203	-1.421358
Midn	Midn-005	934.23214	0.033439462	0.071273979	0.373412099	-2.678006425	2.895037783	7.752929785	-1.421159
Atp6v1d	Atp6v1d-201	4660	2.58136E-06	6.40663E-05	0.373536353	-2.677115604	14.34990849	38.41636394	-1.420679
BC064078	BC064078-00	495.85977	0.040143678	0.082360187	0.37354443	-2.677057722	1.908580643	5.109380549	-1.420648
Ampd2	Ampd2-011	637.4334	0.015418726	0.038694478	0.373619639	-2.676518835	2.301944299	6.161197273	-1.420358
Gm38246	Gm38246-001	1600	0.003821255	0.012955527	0.37369924	-2.67594871	5.026466829	13.45056743	-1.42005
30208D14R	0208D14Rik-	3895	0.001017256	0.004613484	0.373703937	-2.675915079	11.01006145	29.46198946	-1.420032
Gm37084	Gm37084-001	1536	0.00324077	0.011408815	0.373707794	-2.67588746	5.041206425	13.48970105	-1.420017
Naa15	Naa15-002	469.01825	0.007837677	0.022762694	0.373715789	-2.675830218	1.569663072	4.200151879	-1.419987
Arhgef7	Arhgef7-006	520.5595	0.048252543	0.09541306	0.373755347	-2.675547003	1.854244153	4.961117388	-1.419834
Nup153	Nup153-001	11313.545	2.98022E-05	0.000334117	0.373760365	-2.675511082	39.97905987	106.9644177	-1.419815
30579G18R	0579G18Rik-	463	0.03274472	0.070072269	0.373809394	-2.675160163	1.392383328	3.724848411	-1.419625
Map1lc3b	Map1lc3b-002	3749.6865	5.80498E-05	0.000537644	0.373817786	-2.675100111	11.90038153	31.83471196	-1.419593
Gm6286	Gm6286-001	889	0.000915659	0.004256357	0.373828721	-2.67502186	3.354772466	8.974089682	-1.419551
Elmod2	Elmod2-003	9980.783	2.4297E-06	6.16537E-05	0.373836078	-2.674969212	29.96114631	80.14514392	-1.419522
Arrb1	Arrb1-201	27092.81	0.000364389	0.002091906	0.373886256	-2.674610213	73.44756446	196.443606	-1.419329
Prex2	Prex2-002	2314.0523	0.00216049	0.008314254	0.373897056	-2.67453296	8.024387706	21.4614894	-1.419287
Adam10	Adam10-001	43398.964	2.5955E-06	6.42777E-05	0.374037721	-2.673527141	107.8752485	288.4074047	-1.418744
Gm14565	Gm14565-001	4435	5.15504E-06	9.96068E-05	0.374098624	-2.673091895	13.91895315	37.20664084	-1.418509
P24-360B3	24-360B3.1-	3492	6.6642E-05	0.000595368	0.374182991	-2.672489194	9.510050411	25.41550696	-1.418184
Glud1	Glud1-002	424.51195	0.014160012	0.036230421	0.374184859	-2.67247585	1.280037465	3.420869214	-1.418177
Smurf1	Smurf1-004	2124.1665	0.000579683	0.002996853	0.37418631	-2.672465491	7.037771801	18.80820227	-1.418171
Spaca6	Spaca6-010	771.87065	0.031081576	0.067252761	0.374187105	-2.672459809	2.289074792	6.117460381	-1.418168
30118H07R	0118H07Rik-	382.48381	0.047620854	0.094420184	0.374248126	-2.67202407	1.360725161	3.635890384	-1.417933
Pdk3	Pdk3-001	1125.1464	0.030597592	0.066390086	0.374300772	-2.671755313	4.124708719	11.02021243	-1.417788
Tmem206	Tmem206-001	2914	0.012336435	0.032484269	0.374377598	-2.671099993	9.110148469	24.33411751	-1.417434
Clasp2	Clasp2-203	9907.9492	2.55921E-05	0.000300398	0.374418958	-2.670804933	33.8856044	90.50183938	-1.417275
730017L22R	0017L22Rik-	1048.8463	0.007868366	0.022836331	0.374478611	-2.670379483	3.039559164	8.116776431	-1.417045
Creb3l2	Creb3l2-001	48448.943	0.000340572	0.001989527	0.374520857	-2.670078267	180.967051	483.1961899	-1.416882
Itgav	Itgav-001	12161.401	0.002995801	0.010745227	0.374558826	-2.669807598	40.48096128	108.076378	-1.416736
Gm14308	Gm14308-004	754.80099	0.011059251	0.029829843	0.374587438	-2.669603673	1.954416173	5.217516595	-1.416626

Irf6	Irf6-001	33123.665	0.020293623	0.048017727	0.374622455	-2.669354138	106.4000407	284.0193888	-1.416491
Gvin1	Gvin1-002	56635.513	0.000551168	0.002884264	0.374664672	-2.669053352	161.7906203	431.8277975	-1.416328
Slc29a1	Slc29a1-006	475.86779	0.04704386	0.093465526	0.374671392	-2.669005487	0.687949294	1.836140441	-1.416302
Dpagt1	Dpagt1-201	5715	9.90571E-06	0.000153584	0.37470391	-2.66877386	12.51691446	33.4048141	-1.416177
Klf6	Klf6-201	47891	0.000652326	0.003281753	0.374735449	-2.668549247	166.3301204	443.8601177	-1.416056
Ppp2r3a	Ppp2r3a-001	7000.9889	3.52894E-05	0.000374779	0.37475437	-2.66841451	23.68794412	63.20925381	-1.415983
Arnt	Arnt-202	12226.006	0.000122592	0.000930993	0.374790688	-2.668155941	37.24464674	99.37452547	-1.415843
Pvr	Pvr-001	5299.4482	0.000109082	0.000850846	0.374948615	-2.667032123	19.66984136	52.46009876	-1.415235
Selk	Selk-001	4373.0963	1.06436E-05	0.00016166	0.374978403	-2.666820255	14.21008254	37.89573595	-1.415121
Gnptab	Gnptab-004	2449.236	0.018243952	0.044165633	0.375029069	-2.666459973	6.546628055	17.45632167	-1.414926
Dhx9	Dhx9-005	904.72168	0.001836532	0.007321759	0.375097295	-2.665974968	2.870492455	7.65266103	-1.414663
Med16	Med16-002	1429.4013	0.001973689	0.007756609	0.375113831	-2.665857447	4.442995106	11.84439159	-1.4146
Ptpn3	Ptpn3-001	7086.1535	0.026775101	0.059629834	0.375163464	-2.665504765	20.41498034	54.41622737	-1.414409
Ccdc88a	Ccdc88a-004	529.96786	0.036438989	0.076332438	0.37525401	-2.664861598	1.5534509	4.139731649	-1.414061
Anxa2	Anxa2-006	677.6173	0.02243892	0.051966529	0.375294652	-2.664573011	1.820961906	4.852085948	-1.413904
Hmgxb4	Hmgxb4-001	4621.7977	0.000125535	0.000948507	0.375331571	-2.664310911	14.54118256	38.74223135	-1.413762
Cyld	Cyld-202	868.62868	0.049341425	0.09701727	0.375335234	-2.66428491	3.3642102	8.96321447	-1.413748
Abhd17b	Abhd17b-201	1677	8.46327E-05	0.000707657	0.375370487	-2.664034691	4.988011328	13.28823522	-1.413613
Rab31	Rab31-201	2050	0.022541769	0.052141257	0.37553199	-2.662888984	7.486795622	19.93650559	-1.412992
Stab1	Stab1-003	8500.2066	0.007116756	0.021108557	0.375540232	-2.66283054	24.50171923	65.24392624	-1.412961
Abl1	Abl1-002	1998.0733	0.000681795	0.003392632	0.375541614	-2.662820742	5.915677443	15.7523886	-1.412955
Ermard	Ermard-201	1228	0.000237486	0.001515257	0.375611046	-2.662328516	4.017216616	10.69515035	-1.412689
Ccdc34	Ccdc34-007	1197.1491	0.001655883	0.006748452	0.375628196	-2.662206969	4.027550854	10.72217395	-1.412623
Ndufs7	Ndufs7-002	1697.3232	0.000318746	0.001893019	0.375641149	-2.662115164	5.360590836	14.27051015	-1.412573
Pmm2	Pmm2-002	1822.2003	0.000338269	0.001980124	0.375676987	-2.661861215	5.201746951	13.84632846	-1.412435
Acot6	Acot6-001	341	0.016179715	0.040152815	0.375725762	-2.661515664	1.117303303	2.973720243	-1.412248
Git2	Git2-005	4651.8265	0.005977739	0.018391803	0.375783514	-2.661106626	15.41553065	41.02237075	-1.412026
Vps18	Vps18-001	749.39263	0.035841642	0.075279098	0.375889059	-2.660359421	1.933185237	5.142967558	-1.411621
Arhgef5	Arhgef5-006	849.71396	0.00262359	0.009681925	0.376020955	-2.659426258	2.526944814	6.720223391	-1.411115
Gm37215	Gm37215-001	205	0.041279628	0.084251668	0.376062553	-2.659132087	0.695788699	1.850194055	-1.410955
10403A07R	10403A07Rik-	868.39868	0.004984829	0.015971038	0.376067152	-2.659099564	2.546048039	6.770195231	-1.410938
Setd7	Setd7-001	28467.319	1.6462E-05	0.000219301	0.376122631	-2.658707343	90.73961832	241.2500895	-1.410725
Tango2	Tango2-003	814	0.010562768	0.028778522	0.376188498	-2.658241824	2.425166137	6.446678056	-1.410472
Slc9a8	Slc9a8-008	866.60516	0.014893921	0.037692856	0.376225096	-2.657983238	3.096412766	8.23021323	-1.410332
Atic	Atic-001	4447.6069	0.001247836	0.005424339	0.376232312	-2.657932263	18.74659898	49.82719024	-1.410304
Rfc2	Rfc2-002	799.04985	0.045404352	0.09081931	0.376247112	-2.657827709	2.865533074	7.616093207	-1.410248
Plag1	Plag1-001	2068.0671	0.036613894	0.076608901	0.376276166	-2.657622484	6.357942788	16.8970117	-1.410136
Acsl3	Acsl3-003	687.80752	0.029507738	0.064433536	0.376365254	-2.656993412	1.623459594	4.313521446	-1.409795
Tceanc2	Tceanc2-001	2195.8707	0.0001969	0.001317155	0.376404413	-2.656716995	6.474540809	17.20102261	-1.409645
Rnf166	Rnf166-001	3020.8546	2.3755E-05	0.000284181	0.376468085	-2.656267665	11.22593941	29.81909987	-1.409401
Bnip3	Bnip3-001	1549.6418	0.009225132	0.025875094	0.376505135	-2.656006274	5.062320102	13.44555395	-1.409259
Hjurp	Hjurp-001	7735.4018	3.44233E-05	0.000367632	0.376571189	-2.655540382	23.70657738	62.95377355	-1.409005
Szrd1	Szrd1-002	1756.0389	0.012600534	0.033032399	0.376592613	-2.655389316	5.663059383	15.03762738	-1.408923
Chd1	Chd1-001	17184.635	1.49532E-06	4.47681E-05	0.376672603	-2.654825419	56.74769436	150.6552215	-1.408617
Gm10925	Gm10925-001	190852	3.26227E-07	1.82142E-05	0.376757958	-2.654223959	594.768114	1578.647778	-1.40829
Brix1	Brix1-001	3905.1841	0.001892678	0.007495862	0.376858571	-2.653515345	14.29654208	37.9360938	-1.407905
Fdx1l	Fdx1l-201	1143	0.015697043	0.039225428	0.376889504	-2.653297558	4.768376047	12.65192052	-1.407786
Vps13d	Vps13d-007	2048.0535	8.3633E-05	0.000701317	0.37691047	-2.653149965	5.472279587	14.51877839	-1.407706
Zcchc9	Zcchc9-001	1340.7312	0.00670378	0.020108992	0.376958082	-2.652814857	4.526411965	12.00773291	-1.407524
Hacl1	Hacl1-002	1359.9271	0.000514361	0.002731722	0.376997322	-2.652538734	3.876781973	10.28331435	-1.407374
Mgp	Mgp-201	124582	0.015602393	0.039054445	0.377074734	-2.651994179	382.9285103	1015.52418	-1.407078
Gm29514	Gm29514-001	988	0.000853483	0.004028638	0.377166985	-2.65134553	3.121762472	8.276870974	-1.406725
Rasa2	Rasa2-006	992.14077	0.004623614	0.015050254	0.377176059	-2.651281745	2.96424061	7.859037019	-1.40669
Zfp53	Zfp53-201	1467	0.001913778	0.007562812	0.377214924	-2.65100858	5.087820866	13.48785677	-1.406541
Capzb	Capzb-006	491.02387	0.042344131	0.085985577	0.377387037	-2.649799551	1.923725317	5.097486482	-1.405883
Tmem69	Tmem69-201	908.18587	0.001334668	0.005709764	0.377409815	-2.649639621	2.894978803	7.67065054	-1.405796
Bri3bp	Bri3bp-001	1636.76	0.000807557	0.003859873	0.377461012	-2.649280238	6.121578558	16.2177771	-1.4056
Dimt1	Dimt1-201	1493.0906	0.001853064	0.007368166	0.377487016	-2.649097738	5.428132331	14.37965308	-1.405501
Pak2	Pak2-001	23171.685	6.97424E-06	0.000121007	0.377522851	-2.648846281	75.10203398	198.9337434	-1.405364
Cnot4	Cnot4-004	1338.4997	0.008693909	0.024665871	0.377557673	-2.648601976	2.845816054	7.537434024	-1.405231
Ganab	Ganab-201	16903	8.23032E-05	0.000693965	0.377559986	-2.648585756	55.0661063	145.8473048	-1.405222
33420G17R	33420G17Rik-	5644.1028	0.000324257	0.001916775	0.377575363	-2.648477885	19.16002685	50.74490738	-1.405163
Paqr5	Paqr5-003	1854.4755	0.005420308	0.017070856	0.377613384	-2.648211219	3.057977593	8.098170569	-1.405018
Akap5	Akap5-001	520.2629	0.040578874	0.083063974	0.377639775	-2.648026154	1.883445197	4.987412142	-1.404917
Coro2b	Coro2b-001	901.11241	0.01208933	0.03199842	0.377698449	-2.64761479	2.971403904	7.867132924	-1.404693
Ndfip2	Ndfip2-005	1491.4794	0.000743249	0.00361995	0.377735436	-2.647355542	4.227094584	11.19062227	-1.404552
Timp2	Timp2-001	117007.03	0.00999598	0.027572065	0.377741611	-2.647312266	298.7042222	790.7633514	-1.404528
Ube4a	Ube4a-002	782.77825	0.022594471	0.052224363	0.377834923	-2.646658472	2.081939158	5.510181911	-1.404172
Pik3r1	Pik3r1-001	8394.6592	0.001040853	0.004695767	0.37788134	-2.646333368	28.78864034	76.18433956	-1.403995
Uevld	Uevld-001	2515.2542	0.014030696	0.035961267	0.377895441	-2.646234624	8.372428421	22.15540997	-1.403941
Hace1	Hace1-002	3180.4443	5.16666E-05	0.000492919	0.377949105	-2.64585889	9.389372208	24.84295392	-1.403736
Sdccag8	Sdccag8-004	5946.4193	0.000147636	0.001067232	0.378022017	-2.645348563	18.3903538	48.64889599	-1.403458
Fam73a	Fam73a-001	2733.8977	0.002475522	0.009255173	0.378028475	-2.645303376	8.753744413	23.15630965	-1.403433
Nfib	Nfib-202	62084.193	4.74173E-05	0.00046335	0.378097013	-2.644823859	157.6615462	416.987019	-1.403172
Cdk4	Cdk4-001	4195.0034	0.029387523	0.064211972	0.378124781	-2.644629632	14.71699314	38.92099616	-1.403066
Avl9	Avl9-001	6717.1563	0.000141164	0.001035871	0.378188336	-2.644185197	21.29688217	56.31290058	-1.402823
230046K03R	0046K03Rik-	12649	8.14725E-06	0.000134777	0.378208272	-2.644045815	40.86135133	108.039285	-1.402747
Flcn	Flcn-003	1653.2418	0.005430728	0.017099087	0.378212783	-2.644014282	4.751460909	12.56293051	-1.40273
Gtf3c2	Gtf3c2-004	5696.3051	0.000681943	0.003392874	0.37826492	-2.643649853	19.79011467	52.31813372	-1.402531
Rpl10a-ps1	pl10a-ps1-00	8200	0.000575287	0.002979558	0.378270472	-2.643611051	31.15623153	82.36495797	-1.40251
Heg1	Heg1-001	661051.02	0.00083351	0.003952336	0.378279021	-2.643551307	1400.130934	3701.317961	-1.402477
Cstb	Cstb-201	2737	0.000785343	0.003773038	0.378325705	-2.643225099	10.78865602	28.51684637	-1.402299
Stxbp2	Stxbp2-001	4211.8286	4.26189E-05	0.000430335	0.378375123	-2.642879883	13.82825628	36.54642032	-1.402111
Ankrd10	Ankrd10-201	3447.0507	4.66447E-06	9.33712E-05	0.37838195	-2.642832197	10.92721228	28.87878843	-1.402085
Crx	Crx-003	170.37026	0.048354324	0.095559548	0.378446857	-2.642378924	0.551778393	1.458007595	-1.401837
Polg2	Polg2-005	1313.2322	0.000172666	0.00119458	0.378458436	-2.642298083	4.027051589	10.64067069	-1.401793
Gm13157	Gm13157-201	1112.7044	0.006457226	0.019528477	0.378520582	-2.641864265	3.220503905	8.508134181	-1.401556
Ccnc	Ccnc-001	530.00598	0.011834272	0.03147208	0.378663763	-2.640865321	2.163897992	5.714563165	-1.401011
Plekhn1	Plekhn1-201	547	0.036979658	0.077242989	0.378664113	-2.640862882	1.695557124	4.477733871	-1.401009
Gm38046	Gm38046-001	253	0.043122254	0.087201818	0.37877903	-2.640061677	0.930325003	2.456115386	-1.400572
Ube2w	Ube2w-001	1629.6485	0.002733817	0.00999894	0.378844203	-2.639607503	4.970451353	13.12004068	-1.400323
Ccnd3	Ccnd3-001	2119.7799	0.015358293	0.038589214	0.379038041	-2.638257623	5.531110101	14.59249339	-1.399585
Prkar1a	Prkar1a-001	32553.619	8.76606E-06	0.000141677	0.379067754	-2.63805082	101.2191256	267.0211973	-1.399472
Rps25-ps1	Rps25-ps1-00	4946	5.84972E-05	0.000540764	0.379086129	-2.637922952	18.23139274	48.09300937	-1.399402
Mef2d	Mef2d-001	14139.817	7.47997E-06	0.000126889	0.379132912	-2.637597447	44.81964134	118.2161716	-1.399224
Ubr3	Ubr3-201	10720.039	9.43869E-05	0.000765692	0.379213262	-2.637038574	33.07115045	87.20989943	-1.398919
Scyl2	Scyl2-003	1819.1148	0.027078367	0.060215171	0.379224591	-2.636959797	3.827031063	10.09172705	-1.398876
Gm9845	Gm9845-001	1860	0.000477204	0.002581214	0.37924941	-2.636787226	5.329341722	14.05234018	-1.398781
Prpf19	Prpf19-002	918.98819	0.003005139	0.010774209	0.379297145	-2.636455382	3.232859892	8.523290863	-1.3986
Rad9a	Rad9a-201	1617.8381	0.006611634	0.019884831	0.379396587	-2.635764352	5.597904216	14.75475638	-1.398221
Mbnl1	Mbnl1-014	703.79895	0.010982698	0.029665411	0.379429113	-2.635538406	1.758052854	4.633415816	-1.398098
Rpl27-ps3	Rpl27-ps3-201	3634	0.001275058	0.00551478	0.379429765	-2.635533877	13.42035381	35.3697971	-1.398095
Chchd3	Chchd3-002	263.48105	0.031276517	0.067572112	0.379448045	-2.63540691	1.218992502	3.212541262	-1.398026
Rfc2	Rfc2-001	1136.1956	0.000820629	0.0039064	0.379466212	-2.635280738	4.222497512	11.12746636	-1.397957
Gm10576	Gm10576-201	936.89473	0.00135479	0.005777805	0.379537779	-2.634783825	2.549086221	6.716291143	-1.397685
Adgrd1	Adgrd1-001	1806.3683	0.031175788	0.067401259	0.379601345	-2.634342614	5.861668765	15.44164382	-1.397443
Eif3m	Eif3m-001	7174.6459	0.000194771	0.001306229	0.379701883	-2.633645094	26.32870379	69.34046157	-1.397061

Ift52	Ift52-001	2016.4113	0.00077513	0.003737078	0.379713958	-2.633561338	7.292726107	19.20584152	-1.397015
Cse1l	Cse1l-005	379.43857	0.011005059	0.029706361	0.379726908	-2.633471529	1.470923908	3.873636232	-1.396966
Limch1	Limch1-002	2187.7261	0.033577353	0.071532272	0.379766723	-2.633195431	5.323530774	14.01789691	-1.396815
Serinc3	Serinc3-001	229948.2	0.004247388	0.014079854	0.379812276	-2.632879617	658.2644796	1733.131131	-1.396642
Fam213a	Fam213a-001	3303.4156	0.005332126	0.016856684	0.379834502	-2.632725554	11.58135491	30.49052902	-1.396557
Stk38l	Stk38l-001	5176.6016	3.03701E-05	0.000337809	0.37990835	-2.632213796	15.38614669	40.49962758	-1.396277
Gpatch8	Gpatch8-002	19427.076	1.37976E-06	4.27276E-05	0.379916358	-2.632158314	58.43540286	153.8112315	-1.396246
Peak1	Peak1-002	8216.6853	6.99685E-06	0.000121338	0.379965955	-2.631814735	26.06397441	68.5955519	-1.396058
Tmem181a	mem181a-00	4821	1.38352E-05	0.000194204	0.379983462	-2.63169348	15.03954423	39.57947048	-1.395991
AL589742.2	L589742.2-2(	735.1197	0.012218401	0.032252811	0.379984666	-2.631685145	2.148202022	5.653391349	-1.395987
Asap2	Asap2-004	15348.562	0.003239928	0.011407871	0.380019237	-2.631445733	39.1773512	103.0930737	-1.395856
Cd38	Cd38-001	6073.3726	0.009402473	0.02627172	0.380028091	-2.631384424	22.82124598	60.05147122	-1.395822
Neurl1b	Neurl1b-001	1139.7124	0.030054895	0.065432251	0.380061238	-2.631154932	3.558071847	9.361838289	-1.395696
Mrpl40	Mrpl40-001	990.29533	0.009555766	0.026593856	0.380240522	-2.629914337	3.002115523	7.895306654	-1.395016
Proser1	Proser1-001	4758.2879	3.24104E-05	0.000353931	0.380247558	-2.629865672	14.88317522	39.1407516	-1.394989
Atp2b1	Atp2b1-201	20193	2.6975E-06	6.57575E-05	0.380255597	-2.629810074	56.25240218	147.933134	-1.394959
Psme2b	Psme2b-001	5067.4073	0.000762408	0.003692366	0.380387689	-2.62889686	12.70618247	33.40324321	-1.394458
Mapkap1	Mapkap1-008	263.75126	0.044997274	0.090157754	0.380440637	-2.62853098	0.621235829	1.632937621	-1.394257
Tmx4	Tmx4-001	12594.869	5.50449E-05	0.000516518	0.380444472	-2.628504481	37.30119366	98.0463547	-1.394242
Arcn1	Arcn1-001	21708.377	1.2841E-06	4.09614E-05	0.380481691	-2.628247362	68.66998398	180.4817042	-1.394101
Dirc2	Dirc2-001	2907.8616	0.023221408	0.053356584	0.380500707	-2.628116008	8.265930668	21.72382471	-1.394029
Mkln1	Mkln1-012	591.84419	0.023277695	0.053457224	0.38051831	-2.627994435	2.004744879	5.268458386	-1.393962
Spag9	Spag9-007	2456.067	0.005442333	0.017122902	0.380678069	-2.626891545	7.811872275	20.52094123	-1.393357
Rin3	Rin3-004	3583.7165	0.000510634	0.00271445	0.380711569	-2.626660392	10.41459797	27.35561199	-1.39323
Zc3h12c	Zc3h12c-201	7973.8607	0.000202191	0.0013447	0.380734565	-2.626501744	23.67645004	62.18623733	-1.393143
Mfap5	Mfap5-001	10468.619	8.47256E-05	0.000707876	0.380824544	-2.625881175	14.03951215	36.86609067	-1.392802
Trim24	Trim24-005	6423.2587	3.70522E-06	8.05433E-05	0.380906928	-2.625313236	19.6662181	51.62998269	-1.39249
Cntn1	Cntn1-003	2891	0.000192654	0.001295585	0.381052485	-2.624310405	8.414949497	22.08343952	-1.391938
Ss18l1	Ss18l1-001	1303	0.013147351	0.034175529	0.381086371	-2.624077051	4.716915178	12.37754887	-1.39181
Copb1	Copb1-201	12437	2.23758E-07	1.50269E-05	0.381133247	-2.623754308	38.70133694	101.5427995	-1.391633
Bace2	Bace2-201	14615	0.00841945	0.024045979	0.381160983	-2.623563387	27.31344118	71.65854425	-1.391528
Kdm3a	Kdm3a-201	6340.1278	0.004371862	0.01439772	0.381177912	-2.62344687	18.58605219	48.75952045	-1.391464
Hdac1	Hdac1-001	8220.6704	0.000244406	0.001548736	0.381265355	-2.622845182	26.92439652	70.61852369	-1.391133
Eaf2	Eaf2-005	685.9894	0.010621345	0.028911368	0.381324914	-2.622435522	1.848440001	4.84741472	-1.390907
Zfp57	Zfp57-004	4820.4015	0.000115161	0.000887147	0.381390751	-2.621982827	15.71159886	41.1955424	-1.390658
Gm26764	m26764-201	2614	0.000122265	0.000928711	0.381407075	-2.621870611	6.988314595	18.32245666	-1.390596
Gm38228	m38228-001	552	0.044833937	0.089890852	0.381418169	-2.621794346	1.481413228	3.883960825	-1.390555
Rufy2	Rufy2-001	3986.9862	0.000428238	0.002371026	0.381486797	-2.621322697	11.47248496	30.07308521	-1.390295
Ammecr1	mmecr1-001	1356	0.010574383	0.028805584	0.381503878	-2.621205332	4.728485186	12.39433058	-1.39023
Zfp281	Zfp281-002	2208.2654	0.003074411	0.010956412	0.381532233	-2.62101053	7.61837569	19.9678429	-1.390123
Kdm6a	Kdm6a-002	1770.3033	0.015083865	0.038020488	0.381579289	-2.620687309	5.245486091	13.74677883	-1.389945
Atp5e	Atp5e-001	2958.6398	0.004239634	0.014058232	0.38158539	-2.620645405	10.40696754	27.27297165	-1.389922
Nid1	Nid1-201	30498	5.14925E-07	2.3641E-05	0.381761789	-2.6194345	92.74563517	242.9411165	-1.389255
Rsrp1	Rsrp1-001	14113.941	9.9619E-05	0.000795682	0.381769983	-2.619378273	47.94323859	125.5814775	-1.389224
Oxsr1	Oxsr1-001	9017.2092	5.26535E-06	0.000100729	0.381812057	-2.619089634	29.99358062	78.55587609	-1.389065
Gm15342	m15342-001	588.70412	0.013545567	0.034974783	0.381893261	-2.618532723	1.939329843	5.078198655	-1.388759
P23-307I19	23-307I19.1-(	487	0.017431861	0.042603201	0.38196985	-2.618007678	1.508362341	3.948904189	-1.388469
Shoc2	Shoc2-201	13333.331	5.08387E-07	2.34487E-05	0.382017944	-2.617678084	34.21817249	89.57216021	-1.388288
Agl	Agl-201	2904.7576	0.000520373	0.002754679	0.382070899	-2.617315278	8.509000024	22.27073576	-1.388088
Blzf1	Blzf1-004	337.95076	0.019039639	0.045697802	0.3820787	-2.617261834	1.045279935	2.73577128	-1.388058
Lcorl	Lcorl-013	1540.0472	0.003771611	0.012819905	0.382297054	-2.615766951	5.563786164	14.55356797	-1.387234
Gmpr2	Gmpr2-201	2448	0.000422542	0.002348195	0.382352314	-2.615388904	7.947120015	20.7848095	-1.387025
Mrps26	Mrps26-001	1795.9417	2.65829E-05	0.000308006	0.382372194	-2.615252928	5.845170995	15.28660056	-1.38695
Trim3	Trim3-008	478.37274	0.030282673	0.065836076	0.38239142	-2.61512144	1.354275731	3.5415955	-1.386878
Tmem55b	mem55b-00:	1523.7306	0.001297676	0.005592727	0.382394306	-2.615101701	4.965897167	12.98632613	-1.386867
Oma1	Oma1-001	1604.848	0.000309629	0.001851502	0.382453523	-2.614696796	4.604159103	12.03847982	-1.386644
Arhgap21	rhgap21-201	2680.7903	0.010741019	0.029141311	0.382457364	-2.614670536	6.399148152	16.73166413	-1.386629
Mkl2	Mkl2-003	66122.135	0.002624902	0.009685726	0.382484206	-2.61448704	242.2743511	633.4231512	-1.386528
Clca3a1	Clca3a1-002	2067.037	0.000716063	0.003524237	0.382514964	-2.614276808	7.417112159	19.3903843	-1.386412
Pusl1	Pusl1-001	637.73869	0.008289272	0.023759943	0.38256499	-2.613934956	2.449845797	6.403737566	-1.386223
Mlec	Mlec-001	5034.4247	0.001403562	0.005944495	0.382665209	-2.613250373	19.04221873	49.76208521	-1.385845
[truncated: 578,568 more chars]
